# Phospho-proteomic analyses of B-Raf protein complexes reveal new regulatory principles

**DOI:** 10.18632/oncotarget.8427

**Published:** 2016-03-28

**Authors:** Anja E. Eisenhardt, Adrian Sprenger, Michael Röring, Ricarda Herr, Florian Weinberg, Martin Köhler, Sandra Braun, Joachim Orth, Britta Diedrich, Ulrike Lanner, Natalja Tscherwinski, Simon Schuster, Nicolas Dumaz, Enrico Schmidt, Ralf Baumeister, Andreas Schlosser, Jörn Dengjel, Tilman Brummer

**Affiliations:** ^1^ Institute of Molecular Medicine and Cell Research (IMMZ), Faculty of Medicine, Albert-Ludwigs-University (ALU), Freiburg, Germany; ^2^ Institute of Biology III, Faculty of Biology, ALU, Freiburg, Germany; ^3^ Centre for Biological Systems Analysis (ZBSA), Freiburg, Germany; ^4^ Spemann Graduate School of Biology and Medicine (SGBM), ALU, Freiburg, Germany; ^5^ Institute for Experimental and Clinical Pharmacology and Toxicology, ALU, Freiburg, Germany; ^6^ Department of Dermatology, University Medical Centre, ALU, Freiburg, Germany; ^7^ INSERM U976 and Université Paris Diderot, Sorbonne Paris Cité, Paris, France; ^8^ Freiburg Institute for Advanced Studies (FRIAS), ALU, Freiburg, Germany; ^9^ Rudolf Virchow Center for Experimental Biomedicine, University of Würzburg, Würzburg, Germany; ^10^ Centre for Biological Signalling Studies BIOSS, ALU, Freiburg, Germany; ^11^ Department of Biology, University of Fribourg, Fribourg, Switzerland; ^12^ German Cancer Consortium (DKTK), Freiburg, Germany

**Keywords:** BRAF, proteomics, phosphorylation, sorafenib, protein-protein interaction

## Abstract

B-Raf represents a critical physiological regulator of the Ras/RAF/MEK/ERK-pathway and a pharmacological target of growing clinical relevance, in particular in oncology. To understand how B-Raf itself is regulated, we combined mass spectrometry with genetic approaches to map its interactome in MCF-10A cells as well as in B-Raf deficient murine embryonic fibroblasts (MEFs) and B-Raf/Raf-1 double deficient DT40 lymphoma cells complemented with wildtype or mutant B-Raf expression vectors. Using a multi-protease digestion approach, we identified a novel ubiquitination site and provide a detailed B-Raf phospho-map. Importantly, we identify two evolutionary conserved phosphorylation clusters around T401 and S419 in the B-Raf hinge region. SILAC labelling and genetic/biochemical follow-up revealed that these clusters are phosphorylated in the contexts of oncogenic Ras, sorafenib induced Raf dimerization and in the background of the V600E mutation. We further show that the vemurafenib sensitive phosphorylation of the T401 cluster occurs *in trans* within a Raf dimer. Substitution of the Ser/Thr-residues of this cluster by alanine residues enhances the transforming potential of B-Raf, indicating that these phosphorylation sites suppress its signaling output. Moreover, several B-Raf phosphorylation sites, including T401 and S419, are somatically mutated in tumors, further illustrating the importance of phosphorylation for the regulation of this kinase.

## INTRODUCTION

The Ras/Raf/mitogen-activated/extracellular-regulated kinase (MEK)/extracellular signal regulated kinase (ERK) pathway plays a pivotal role in controlling proliferation, survival and differentiation of metazoan cells. The Raf tier represents a particularly important node as these Ser/Thr-kinases are subject to a complex, still ill-defined activation process that integrates various protein-protein and –lipid interactions and positive as well as negative phosphorylation events [1-3]. The Raf family comprises the A-Raf, B-Raf and Raf-1 (aka C-Raf) isoforms in vertebrates as well as D-Raf and LIN-45 in *Drosophila* and *Caenorhabditis*, respectively. Genetic approaches in mice and chicken DT40 B cells demonstrated that Raf-1 and B-Raf have unique, but also overlapping functions [4-7]. B-Raf, the most potent kinase of the family, plays an important role in various developmental processes [8]. This is reflected by the various germ-line *BRAF* mutations found in the neuro-cardio-facio-cutaneous syndromes or RASopathies [9, 10]. Furthermore, B-Raf, as the most frequently mutated kinase in cancer, has become an important target in clinical oncology, in particular in melanoma and hairy cell leukemia, with other diseases following suit [2, 11]. The multi-kinase inhibitor sorafenib, originally developed to block Raf-1 in tumor cells with aberrant Ras signaling [12], also targets B-Raf, although its efficacy in B-Raf driven melanoma has been disappointing [11]. Nevertheless, sorafenib affects B-Raf signaling complexes, in particular Raf dimerization, at concentrations achievable in patients treated with this drug for receptor tyrosine kinase (RTK) driven tumor entities [13, 14]. Thus, we require an in-depth knowledge as to how sorafenib interferes with B-Raf, even if this interaction is not pursued therapeutically. In contrast, more specific B-Raf inhibitors like vemurafenib and dabrafenib yield unprecedented response rates in melanoma [11, 15]. However, the use of existing Raf-inhibitors is restricted to tumor cells with *BRAF*^V600E/K^ mutations as the binding of these compounds to wildtype B-Raf provokes the paradoxical activation of the MEK/ERK pathway. This phenomenon, which causes therapy resistance and side effects including secondary neoplasms, involves the presence of active Ras and hetero-dimerization between a drug-bound and a drug-free Raf protomer [14, 16-19]. Consequently, alternative strategies for the inhibition of Raf-kinases in the context of aberrant Ras signaling are urgently needed and might emerge from studies of basic principles underlying B-Raf regulation.

Our current knowledge of B-Raf regulation is best approached from a structural perspective [2]. B-Raf and the other Raf isoforms share three conserved regions (CR) that display a higher degree of sequence similarity between the Raf family members (Figure 4). The CR1 is placed C-terminal of the B-Raf specific region (BSR) and contains two subdomains, the Ras-binding domain (RBD) and the Cysteine-rich domain (CRD) that are both involved in Ras binding. The CR2 contains several phosphorylation sites of which phosphorylated S365 plays an important role for maintaining B-Raf in an auto-inhibited state by serving as a 14-3-3 binding site. The CR3 encompasses the N-region and the kinase domain and follows the hinge region, a stretch of low inter-paralogue sequence homology and diversity generated by alternative splicing [20]. The kinase domain also contains several residues involved in Raf dimerization with R509 in the dimer interface (DIF) playing a key role in this process [13, 21]. B-Raf is fully activated by conformational changes induced by phosphorylation of the T^599^VKS^602^-motif in its activation loop, a step mimicked by the most common oncogenic *BRAF* mutation, V600E [22-24]. The C-terminal end of the CR3 is marked by a second 14-3-3 binding motif around S729 that is crucial for B-Raf activation [25-28] and contains negative ERK controlled feedback phosphorylation sites in the SPKTP-motif [29, 30].

Although many details are still missing, the following model of the B-Raf activation cycle has emerged from studies conducted on B-Raf and Raf-1 over the last 20 years [31]. In its inactive state, B-Raf is kept in a closed auto-inhibited state in which the N-terminal moiety comprising the BSR, CR1 and CR2 folds over the CR3 and potentially prevents activating phosphorylation and protein-protein interaction events, in particular dimerization. Experiments using B-Raf proteins with mutations in the CRD, e.g. the RASopathy associated Q257R substitution, or in the CR2, e.g. S365A, have revealed the critical role of CR1/CR2 for auto-inhibition [13, 25, 27]. Following its interaction with active Ras-proteins (Ras-GTP), the N-terminal moiety becomes displaced from the CR3 and re-binding of the 14-3-3 dimer, which clamps the N- and C-terminal moieties together, is prevented by de-phosphorylation of S365 [32]. This more open conformation of B-Raf then might trigger a series of post-translational modifications (PTMs), in particular phosphorylation events and its homo- and hetero-dimerization with Raf-1, A-Raf or the related KSR proteins. As hetero-dimers display a distinct MEK phosphorylation potential compared to homo-dimers [30, 33], the control of the composition and stability of B-Raf complexes emerges as important regulatory layer to control the signaling output of the Ras/ERK pathway [3, 34]. Furthermore, dimerization appears to control B-Raf phosphorylation as inhibitors such as sorafenib or L779450 not only promote the formation of heterodimers, but also induce prominent electrophoretic mobility shifts (EMS). Likewise, the kinase-dead B-Raf^D594A^ mutant, which behaves similarly to drug-bound B-Raf in that sense that it provokes paradoxical MEK/ERK phosphorylation by binding and transactivating Raf-1, also undergoes a dramatic EMS in cells with upregulated Ras activity [13, 16]. In both cases, however, the phosphorylation sites involved in these processes are unknown, further illustrating how little we know about the phosphorylation events and protein-protein interaction events guiding B-Raf through its activation cycle.

In this study, we report the detailed analysis of reconstituted and endogenous B-Raf complexes in various cellular model systems by quantitative and qualitative mass spectrometry. We provide a detailed catalog of interaction partners and map phosphorylation events induced by clinically relevant Raf inhibitors. Functional characterization of phosphorylation sites using combinatorial approaches of proteomics, biochemistry and genetics reveals molecular mechanisms underlying B-Raf regulation.

## RESULTS

### Generation of model systems for (phospho)proteomic analyses of B-Raf complexes

**Figure 1:**
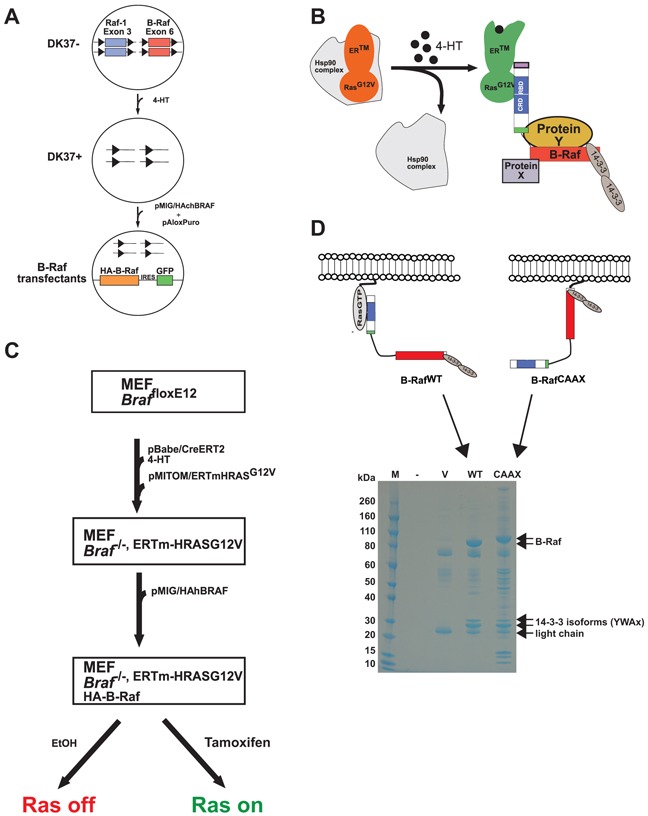
Principle and workflow of the model systems for proteomic studies of B-Raf complexes **A.** Add-back of HA-tagged B-Raf transgenes into conditional B-Raf and Raf-1 double deficient DK37 cells, a clone of DT40 chicken B-lymphocytes expressing the MerCreMer recombinase and containing floxed B-Raf and Raf-1 exons (DT40MCM Raf-1^flE3^/B-Raff^lE6^). Addition of 4-HT leads to Cre-recombinase-mediated inactivation of the *raf* loci and conversion of the DK37- to the DK37+ clone. The resulting B-Raf and Raf-1 deficient cell line was subsequently transfected with bicistronic constructs coding for hemagglutinin (HA)-tagged B-Raf and GFP. The pAloxPuro construct was cotransfected into the DK37+ cells as selection marker. **B.** Scheme of the ERTmH-RAS^G12V^ system. In the absence of 4-HT, the ERTmH-RAS^G12V^ fusion protein is sequestered in large heat shock protein complexes shielding the oncogenic Ras moiety [35]. Binding of 4-HT to the estrogen receptor (ER) moiety of the fusion protein induces a conformational change and exposure of the H-Ras^V12^ moiety, which in turn recruits B-Raf. In this experimental set-up, HA-tagged B-Raf (or mutants thereof) is expressed in *Braf* deficient MEFs and is then purified using anti-HA antibody agarose conjugates. **C.** Workflow of a typical experiment using the MEF complementation system. **D.** Example of a Coomassie stained SDS-PAGE showing size-separated HA-agarose precipitates from MCF-10Atet cells transfected with the empty vector (V), or constructs expressing B-Raf^wt^ (wt) or B-Raf^CAAX^ (CAAX).

To identify core and potentially accessory and cell-type specific components of B-Raf containing protein complexes as well as underlying regulatory mechanisms, we decided to analyze the composition of complexes and phosphorylation/ubiquitination sites on B-Raf itself across various cell lines and under different conditions. Therefore, we established four cell line models, allowing the highly efficient purification of hemagglutinin-(HA)-tagged B-Raf proteins. The first model system represents a genetic complementation system of B-Raf/Raf-1 double deficient DT40 cells [5] that were transfected with a retroviral vector encoding HA-tagged chicken B-Raf (Figure 1A). As chicken lack *Araf*, this system provides the unique opportunity to study the function and protein-protein interactions of Raf mutants in vertebrate cells without the interference by endogenous Raf-proteins.

Secondly, we used *Braf* deficient murine embryonic fibroblasts (MEFs) expressing a 4-hydroxytamoxifen- (4HT) controllable oncogenic H-Ras^G12V^::ER^TM^ fusion protein (Figure 1B/1C). In the absence of 4-HT, the H-Ras^G12V^::ER^TM^ fusion protein is held in an inactive complex organized by Hsp90. Addition of 4-HT induces a conformational change of the ER moiety of the fusion protein, leading to its release and pathway activation [35]. Using this system, we re-introduced and compared B-Raf^WT^ and B-Raf^D594A^ in the absence or presence of oncogenic Ras, the former either singly or in combination with sorafenib. As a third model system, we purified endogenous protein from B-Raf expressing MEFs. In both MEF systems, we applied SILAC-based mass spectrometry (MS) to obtain quantitative insights into protein-protein interactions of B-Raf.

Fourthly, we then used the immortalized human mammary epithelial cell line MCF-10A to compare the sub-proteomes and PTM spectra between B-Raf^WT^ and B-Raf^CAAX^, a constitutively activated Raf protein, which is tethered to the membrane via the polybasic region and CAAX-box of human K-Ras [13]. The pattern of co-purified bands and the interactome are quite distinct between B-Raf^WT^ and B-Raf^CAAX^ complexes (Figure 1D).

The use of cell types from different organisms expressing HA-tagged B-Raf offers two major advantages: Firstly, the two complementation systems (DT40, MEFs) provide the unique opportunity to study the function, phosphorylation status and protein-protein interactions of B-Raf mutants without interference by the endogenous protein. Secondly, the complementation with a B-Raf cDNA allows the use of small epitope-tags such as the hemagglutinin (HA) tag for which highly specific antibody resins of high affinity are available. Since B-Raf is highly phosphorylated and entertains many protein-protein-interactions, antibodies raised against the endogenous protein might enrich or discriminate against certain subpopulations. Indeed, one commercially available antibody displays impaired binding to feedback-phosphorylated B-Raf [29]. Nevertheless, the approach using ectopically expressed epitope-tagged B-Raf bears the risk that detected protein-protein interactions represent overexpression artifacts. Therefore, we aimed to confirm interactions using endogenous B-Raf protein in the aforementioned MEF system by MS (Supplementary Table S1) or, if suitable antibodies were available, by co-immunoprecipitation experiments using either tagged or endogenous B-Raf.

### New insights into the B-Raf interactome and its dynamics

**Figure 2:**
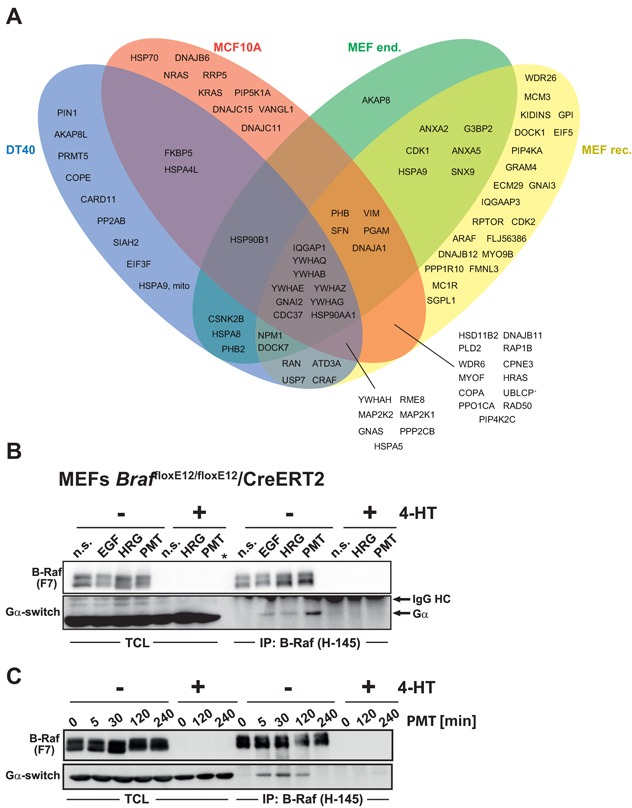
The B-Raf interactome and identification of Gα subunits as novel interaction partners **A.** Venn diagram showing the proteins identified in B-Raf complexes purified from the four different cellular systems (DT40, *Braf* knock-out MEFs complemented with HA-tagged B-Raf (MEF rec.) or MEFs expressing endogenous B-Raf (MEF end.). **B.** and **C.** B-Raf interacts with Gα subunits of hetero-trimeric G-proteins in an inducible manner in MEFs from conditional *Braf* deficient mouse embryos expressing the 4-HT regulated recombinase Cre-ERT2. Prior to the experiment, the *Braf* locus was inactivated by 4-HT exposure or kept intact (- 4HT) and the cells were expanded for 5 days. Subsequently, the cells were stimulated with the indicated reagents (in **B:** EGF: 10 nM epidermal growth factor for 5 min; HRG: 50 ng/ml β-Heregulin for 5 min C: or 10 nM *Pasteurella multocida* (PMT) toxin for 2 h) or left untreated (n.s.). In **C**: same set-up as in B, except that cells were stimulated with 10 nM PMT for the indicated time points. Following lysis, B-Raf was purified from total cellular lysates (TCLs) using anti-B-Raf (H-145) polyclonal antibodies and immunoprecipitates (IPs) were subject to Western blot analysis using an antibody raised against the highly conserved switch region common to all Gα subunits (Gα switch). Note the absence of Gα subunits in immunoprecipitates (IP) from 4-HT treated MEFs.

Using label-free and SILAC-based quantitative mass spectrometry (MS), we aimed to obtain novel insights into the composition of B-Raf signalosomes (Figure 2A and Supplementary Table S1). Therefore, we performed affinity purifications against endogenous or HA-tagged versions of B-Raf and compared protein enrichments to IgG control IPs, or anti-HA IPs using cells infected with the empty vector only, respectively. Interacting proteins had to be consistently enriched in two biological replicates each (p<0.05 in at least one of the two IPs). As expected, we could identify known B-Raf interaction partners such as MEK1, MEK2, the 14-3-3 family and the Hsp90/Cdc37 chaperone complex in all species [36-40]. Chaperones of the Hsp70 family and their regulators, the DnaJ/Hsp40 proteins [41], were also present in B-Raf complexes. Likewise, two components of the PP2A phosphatase complex, which has been implicated in the recycling of feedback-phosphorylated B-Raf [26], were also present. We also co-purified PPP1R10, a regulatory subunit of the PP1 holo-phosphatase complex from MEFs, which is in line with biochemical and genetic data implicating this phosphatase in Raf regulation [32].

In addition to this B-Raf core interactome, we identified several other interaction partners, which have been reported as interaction partners of B-Raf or Raf-1 previously, further validating our approach. We detected peptides derived from the Casein kinase II holoenzyme and the peptidyl-prolyl-cis/trans-isomerase Pin1, which have been implicated in N-region phosphorylation and phosphorylation-dependent isomerization of Raf-kinases, respectively [26, 42]. In addition to Pin1, another peptidyl-prolyl-cis/trans-isomerase, the immunophilin FKBP5, was found in B-Raf complexes from DT40 and MCF-10A cells as it was also recently reported for HEK 293T cells [43]. The identification of FKBP5 in B-Raf complexes is of particular interest since these proteins inhibit the Ser/Thr-Phosphatase Calcineurin that in turn has been shown to regulate B-Raf activity by dephosphorylating T401 [44]. We also confirmed the interaction of B-Raf with the scaffold protein IQGAP1 in all cell line models and identified IQGAP3 as a novel interaction partner in MEFs and MCF-10A cells. The critical role of IQGAP1 as an important scaffold protein organizing the RAF/MEK/ERK pathway and as a potential pharmacological target has been recently demonstrated [45]. Likewise, IQGAP3, an isoform specifically expressed in proliferating cells, plays a critical role in Ras/ERK mediated proliferation [46].

Prohibitin has been implicated in the regulation of Raf-1 signaling [47]. Here we show now that this protein and the related prohibitin-2 bind to B-Raf as well. Likewise, we identified the Raptor subunit of the mTOR complex. This represents an interesting finding, as B-Raf is associated with mTOR (Frap1) and Rictor in murine T cells [48].

Among the novel B-Raf interaction partners we identified several α-subunits of hetero-trimeric G-proteins (Supplementary Table S1). The observation that Gα subunits could be co-purified with B-Raf from distinct cellular model systems belonging to three vertebrate species suggests that they could reflect a novel signaling pathway leading to B-Raf activation. Therefore, we confirmed this interaction by co-immunoprecipitation of endogenous proteins (Figure 2B/2C). To this end, we stimulated B-Raf pro- and deficient MEFs with epidermal growth factor (EGF), β-Heregulin (HRG) and *Pasteurella multocida* toxin (PMT). This highly mitogenic bacterial toxin promotes the deamidation of a glutamine residue essential for GTP hydrolysis in the Gα subunits of heterotrimeric G-proteins, which in turn abolishes their intrinsic GTPase activity [49]. Thus, PMT locks Gα subunits in their GTP-bound state and has a similar effect as oncogenic mutations in Gα subunits or in Ras-proteins. Treatment of *Braf* proficient MEFs with EGF or HRG promoted the interaction between B-Raf and Gα subunits, while stimulation with PMT caused an even stronger interaction between B-Raf and Gα subunits (Figure 2B/2C). Unfortunately, due to the lack of suitable precipitating antibodies, we could not demonstrate the Gα/B-Raf interaction by reciprocal co-immunoprecipitation. Importantly, the Gα subunits were not purified from their *Braf* deficient counterparts, indicating their specific interaction with B-Raf and ruling out an unspecific binding to IgG or protein G sepharose beads. Taken together, these findings confirm Gα subunits as novel components of the B-Raf interactome.

**Figure 3:**
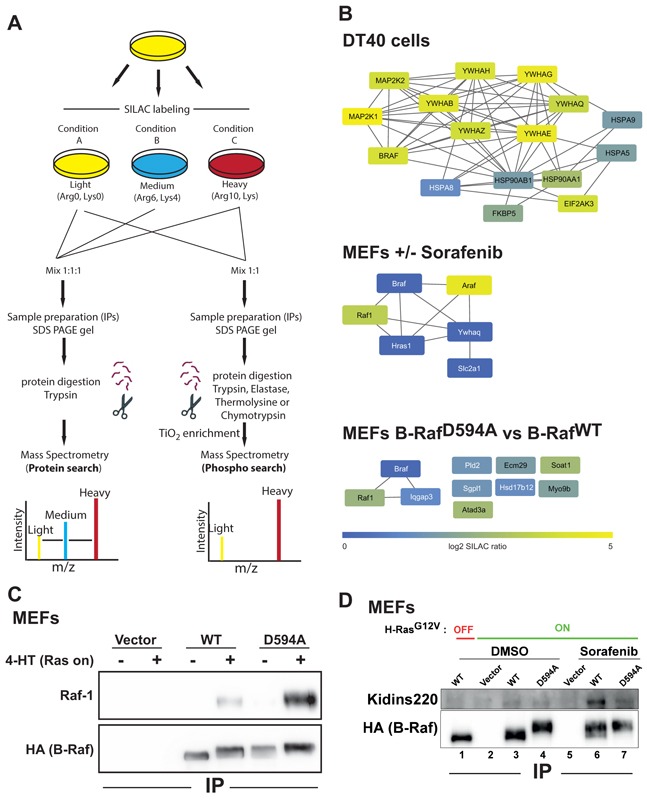
SILAC-based MS reveals inducible B-Raf protein complexes **A.** Flow-chart for SILAC-based MS experiments. **B.** B-Raf network in three experimental systems. Top: B-Raf interacting proteins were identified in DT40 cells by anti-HA-B-Raf IPs compared to vector control cells (n=2; p<0.05). Middle: B-Raf network under sorafenib treatment in MEFs with active H-Ras^V12^ signaling. B-Raf interacting proteins were identified by anti-HA-B-Raf IPs of cells treated with sorafenib compared to vector control cells (n=2; p<0.05). Bottom: B-Raf^D594A^ interacting proteins were identified by anti-HA-B-Raf^D594A^ IPs compared to B-Raf^WT^ IPs (n=2; p<0.05). Proteins were colored according to their log2 SILAC ratios. Networks were generated with STRING DB. **C.** Confirmation of the increase of Raf-1/B-Raf complexes immunoprecipitated with anti-HA antibody 3F10 matrix from MEFs upon H-Ras^G12V^::ER^TM^ release observed by SILAC-based MS by Western blotting. Note that due to its paradoxical behavior B-Raf^D594A^ recruits more Raf-1 than B-Raf^WT^ as it also illustrated in B. **D.** B-Raf interacts with Kidins220/Arms in the presence of sorafenib. *Braf* deficient MEFs were infected with the indicated B-Raf expression constructs or the empty vector control and treated with 10 μM sorafenib or vehicle (DMSO). HA-tagged B-Raf complexes were immunoprecipitated and probed with an anti-Kidins220 antibody.

Compared to label-free MS, SILAC-based MS permits a more precise quantification of known and novel interaction events in the B-Raf signalosome (Figure 3A). For example, SILAC-based MS analysis of B-Raf complexes purified from DT40 cells revealed distinct degrees of enrichment for MEK1 (MAP2K1) versus MEK2 (MAP2K2) and the various 14-3-3 (YWAx) isoforms (Figure 3B and Supplementary Table S2). Furthermore, this approach identified several proteins as regulated interaction partners as their ratio was significantly changed in B-Raf complexes from control vs. the perturbed sample. For example, sorafenib induced the marked increase of B-Raf/Raf-1 and B-Raf/A-Raf heterodimers in MEFs with active Ras-signaling (Figure 3B/3C and Supplementary Table S3), as we had described for this experimental system previously [13]. Likewise, we observed in the protein complex organized by the B-Raf^D594A^ mutant significantly higher levels of Raf-1 derived peptides than in B-Raf^WT^ complexes in the context of H-Ras^G12V^::ER^TM^ release (Figure 3B/3C and Supplementary Table S4). These SILAC ratios are in agreement with our previously published co-immunoprecipitation/Western blot analyses [13] and with the independently reproduced data set in Figure 3C. This validates our approach and provides good confidence into the SILAC ratios for B-Raf interaction partners that could not be confirmed by Western blotting due to the lack of suitable antibodies.

Importantly, our SILAC-based experiments also revealed dynamically regulated novel interaction partners such as the proteasome-associated protein ECM29 homolog, which is enriched in MEFs expressing the B-Raf^D594A^ mutant compared to those complemented with B-Raf^WT^ (Figure 3B). ECM29 represents an adaptor associated with the 26S proteasome and based on the observation that the expression level of B-Raf^D594A^ mutant often appears reduced in cells with oncogenic Ras signaling [13, 16], this finding is of particular interest, also in conjunction with the de-ubiquitinase USP7 found in B-Raf complexes from MEFs and DT40 cells. Interestingly, B-Raf^D594A^ also associated with IQGAP3, Myosin 9b, a unique myosin with Rho-GAP activity, and phospholipid metabolizing enzymes such as phospholipase D2 and sphingosine-1-phosphate lyase in a dynamic fashion (Figure 3B). These interactions might reflect the increased membrane residency of drug-bound or kinase-dead B-Raf that has been described previously [50]. Based on our finding by SILAC-based MS, we also confirmed the marked increase in the interaction of B-Raf with KidinsS220/ARMS by Western blotting in sorafenib-treated MEFs with oncogenic Ras signaling (Figure 3D). This membrane-spanning docking protein is involved in the regulation of ERK signaling in neurons and lymphocytes [48, 51].

### New insights into B-Raf phosphorylation and ubiquitination

**Figure 4:**
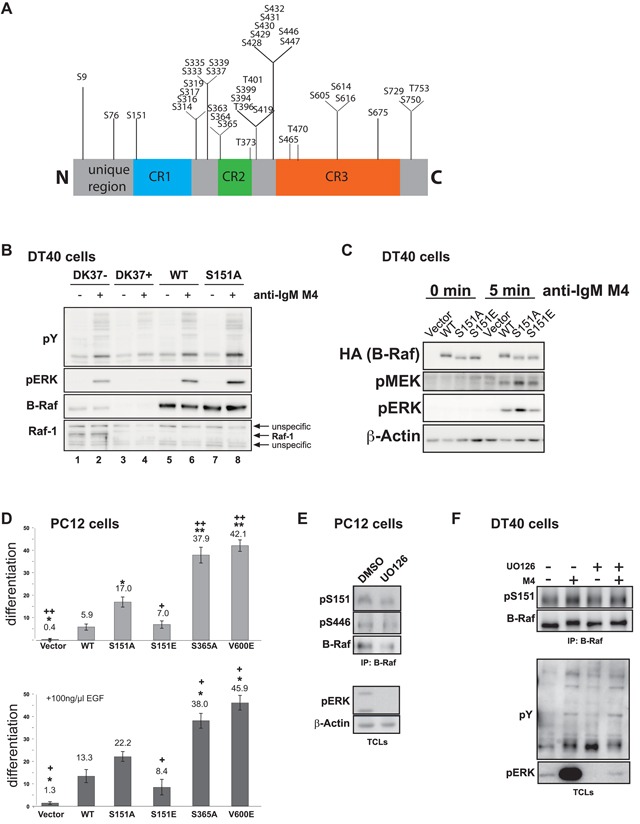
The B-Raf phospho-map and characterization of S151 **A.** The B-Raf phospho-map based on phosphorylation sites identified in this study (see Supplementary Table S6 for additional information). Shown is a representation of the B-Raf primary structure indicating CR1-3. **B.** Rescue of BCR-mediated ERK activation in Raf-1/B-Raf double deficient DT40 cells through add-back of B-Raf^WT^ and B-Raf^S151A^. Parental DK37- cells, Raf-1/B-Raf deficient DK37+ cells and cells stable transfected either with chicken B-Raf^WT^ or B-Raf^S151A^ expression constructs (see Figure 1A) were stimulated with the anti-IgM antibody M4 for 5 min. TCLs were analyzed with the indicated antibodies. Successful stimulation of the cells was verified through detection of tyrosine-phosphorylated proteins (pY). **C.** pMEK/pERK levels are higher in BCR-stimulated DT40 cells re-expressing B-Raf^S151A^ compared to B-Raf^wt^ and B-Raf^S151E^. The inducible system is described in Supplementary Figure S1A/S1B. **D**. B-Raf^S151A^ displays a stronger neuritogenic potential than B-Raf^WT^. PC12 cells transfected with the indicated pMIG/HAhB-Raf plasmids were identified by GFP fluorescence. The graph indicates the proportion of GFP-positive, differentiated cells relative to the total number of GFP-positive cells (n=3-5, S.E.M.). Asterisks or + signs indicate an ANOVA single factor result between the HAhB-Raf^WT^ or the HAhB-Raf^S151A^ expressing cells and the indicated transfectants, respectively (* p < 0.02, ** p < 0.0001, + p < 0.02 and ++ p < 0.005). Upper and lower graph: cells grown in the absence or presence of 100 ng/ml EGF. **E.** and **F**. Phosphorylation of B-Raf at S151 is not affected by UO126. E. Endogenous B-Raf was purified from PC12 cells pre-treated with either DMSO (vehicle) or 20 μM UO126 for 2 h. **F.** B-Raf deprived DT40 cells re-expressing HA-tagged chicken B-Raf were pre-treated with either DMSO (vehicle) or 10 μM UO126 for 30 min and then stimulated with anti-IgM antibody M4. B-Raf was immunoprecipitated using anti-B-Raf H-145 antibodies and probed for phosphorylation at S151. Detection of pERK indicates successful MEK inhibition. Successful BCR stimulation is confirmed by the induction of tyrosine-phosphorylated bands typical for anti-IgM treated DT40 cells.

Next, we used our experimental systems to identify ubiquitination and phosphorylation sites on B-Raf. To this end, we applied a multi-protease approach [52], which has not been applied to B-Raf complexes so far and delivered a very good sequence coverage. In case of phospho-peptide analyses, we used TiO_2_-based enrichment protocols. In addition to K88 as novel ubiquitination site (Supplementary Table S5), we identified in total 36 phosphorylation sites of which 10 have not been listed in the Phosphosite database (http://www.phosphosite.org/proteinAction.action?id=577&showAllSites=true) and only eight have been subject to functional characterization so far (Supplementary Tables S6–S9). Importantly, several phosphorylation sites were observed in the three cell lines from chicken, mouse and man, indicating a conserved function in B-Raf regulation. As noted for other phospho-proteins [53-55], most phosphorylation sites were located outside of the three structured domains, the RBD, CRD and kinase domain, and clustered in the BSR, the HR between CR2 and CR3, and the C-terminus (Figure 4A). This suggests that these areas reside in less-ordered states allowing easy access for kinases and phosphatases. Using bioinformatic tools like KinomeXplorer, Scansite or PhosphoNET, we list which kinases could be implicated in these phosphorylation events (Supplementary Table S6).

### Several B-Raf phosphorylation sites are mutated in cancer

By combining the information obtained from our MS experiments and sequencing of tumor genomes, it is becoming increasingly obvious that somatic mutations identified in human cancers often affect phosphorylation sites [56, 57]. This can occur either directly or indirectly by altering surrounding residues constituting the motif mediating the kinase/substrate interaction or the phosphorylation-dependent recruitment of critical effectors such as 14-3-3 proteins. As shown in Supplementary Table S6, eleven and eight of the phosphorylation sites are directly or indirectly affected by mutations listed in the Catalogue of Somatic Mutation in Cancer (COSMIC; http://cancer.sanger.ac.uk/cosmic/gene/overview?ln=BRAF), respectively. As a proof-of-principle, we noticed that the 14-3-3 binding site S365 and the adjacent S364 are replaced by leucine residues in tumor samples. Furthermore, P367, which represents an integral part of the 14-3-3 binding motif surrounding S365 (RXXpS^365^XP^367^; [58]), is substituted by arginine or serine residues. Loss of 14-3-3 binding to the CR2 due to mutation of S365 has been shown by various groups to increase the signaling potential of B-Raf and could thus contribute to tumorigenesis [13, 20, 25, 27]. At present, it is unclear whether phosphorylation of the conserved S364 is critical for B-Raf regulation or whether its leucine substitution might interfere with phosphorylation of S365 and/or 14-3-3 binding. In any case, the cancer-associated mutations of the 14-3-3 binding motif surrounding S365 represent an interesting parallel to the Noonan-Syndrome associated *RAF*1 mutations, affecting the equivalent motif around S259 in Raf-1 [59], and the cancer-associated mutations in the CR2 of A-Raf [60-62].

We addressed the function of several phosphorylation sites using various genetic and biochemical approaches such as B-Raf mutants in which the residues in question are replaced with either non-phosphorylatable or phospho-mimetic residues. The first residue we re-addressed was S151, a phosphosite that has been recurrently identified in MS experiments (see http://www.phosphosite.org/siteAction.do?id=74357), but whose function is controversially described in the literature. In agreement with two other studies using mammalian cells [26, 63], we show in our chicken DT40 complementation system (Figure 1A) that the S151A substitution does not abrogate B-Raf activity (Figure 4B). This was not necessarily expected as a study in *Xenopus* oocytes suggests that phosphorylation of the S151 equivalent is required for activity of *Xenopus* B-Raf (XeB-Raf; [64]). In fact, S151A, but not S151E, rather confers a slight increase in MEK/ERK phosphorylation upon EGF stimulation in B-Raf deficient DT40 cells in which we inducibly expressed these B-Raf proteins to a similar level as endogenous B-Raf (Figure 4C and Supplementary Figure S1A/S1B). To further assess the biological activity of S151 mutants, we expressed them in PC12 cells, a model system in which B-Raf gain-of-function mutants lower the threshold for spontaneous neuronal differentiation under normal growth conditions or in the presence of otherwise mitogenic growth factors like EGF [22, 27, 30]. Indeed, expression of B-Raf^S151A^ in PC12 cells induced a higher degree of spontaneous neuronal differentiation in the absence and presence of exogenous EGF, while B-Raf^S151E^ behaved similarly to B-Raf^WT^ (Figure 4D). However, the neuritogenic potential of B-Raf^S151A^ did not reach that of the more active B-Raf^S365A^ and B-Raf^V600E^ mutants. Based on pulldown experiments using GST-Ras^V12^ as a bait and B-Raf^WT^ or B-Raf^S151A^ as a prey, S151, which is located at the N-terminal border of the RBD, has been implicated in controlling the Ras/B-Raf interaction [26, 63]. However, by performing alpha screen assays we could not detect statistically significant differences in Ras-binding affinity between the B-Raf^WT^, B-Raf^S151A^ and B-Raf^S151E^, although we noticed a trend for a reduced Ras interaction of the latter mutant that has not been investigated before (Supplementary Figure S1C). Similarly, we did not detect an obvious impact of S151 mutations on B-Raf homo-dimerization (Supplementary Figure S1D).

The presented data and work by the Morrison group [26] indicate that phosphorylation of S151 by a proline-directed kinase, or a negative charge at this position, impairs the signaling potential of B-Raf. This concept is further supported by an entry in the COSMIC database listing a *BRAF*^P152S^ mutation (Supplementary Table S6). This alteration destroys the consensus motif required for S151 phosphorylation by proline-directed kinases of the ERK and CDK subfamilies [65]. These findings raise the question which kinases control S151 phosphorylation. Using metabolic labelling with ^32^P, the Morrison laboratory showed that growth factors induce the phosphorylation of S151 by a process that is sensitive to the MEK inhibitor UO126 [26, 66]. Similarly, UO126 and PD98059, another MEK inhibitor, blocked S151 phosphorylation in melanoma cells [63]. This implies that ERK itself phosphorylates S151. Interestingly, however, our MS analyses show that this site is already phosphorylated in unstimulated PC12 and DT40 cells. Furthermore, we observed in Western blot analyses using an anti-phospho-S151 antibody that UO126, despite strongly suppressing ERK phosphorylation, did not yield a discernible effect on S151 phosphorylation. This was observed for endogenous B-Raf in PC12 and in the B-Raf/Raf-1 proficient DT40 subline DK37- (Figure 4E/4F). Thus, the half-life and mechanisms of S151 phosphorylation might be more versatile and cell-type-specific than previously thought and might even involve other kinases in addition to ERK. As XeB-Raf can be phosphorylated by CDKs [64] and as S151 is embedded in a CDK consensus phosphorylation motif (Supplementary Figure S1E), it might be possible that CDKs contribute to S151 phosphorylation. This remains an area for future studies.

**Figure 5:**
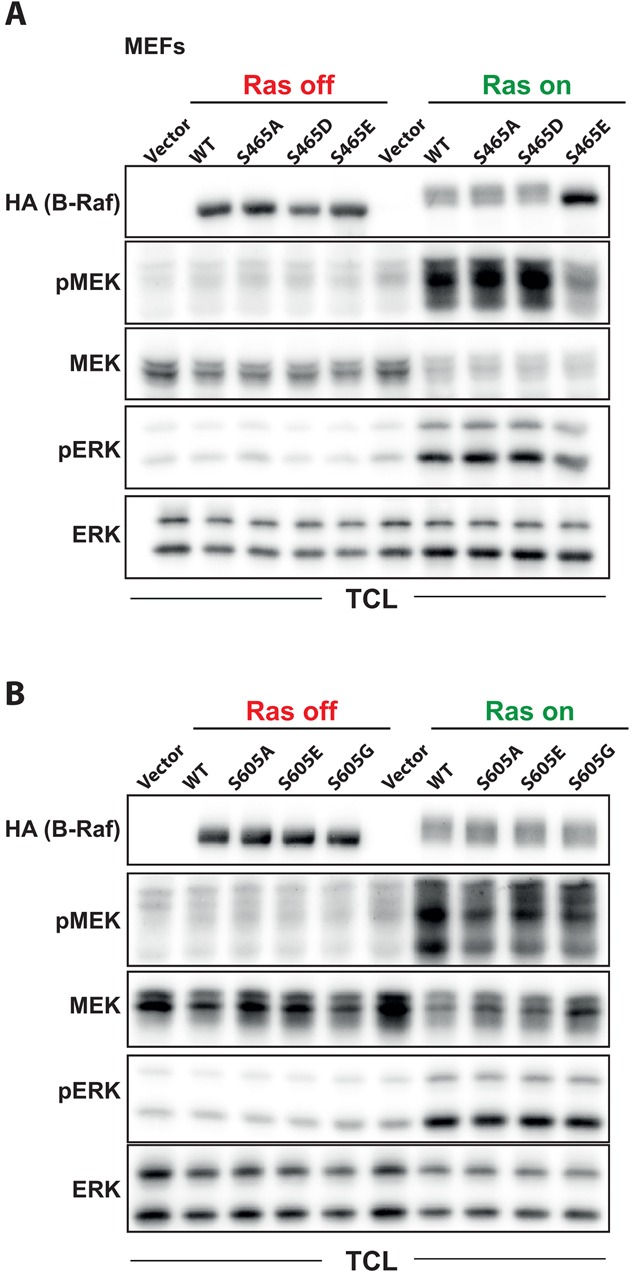
Functional characterization of the phosphorylation sites S465 and S605 in oncogenic Ras signaling *Braf*^−/−^ MEFS expressing the ERTmH-RAS^G12V^ fusion protein were infected and treated with the indicated B-Raf expression vectors as described in Figure 1B/1C. TCLs were analyzed using the indicated antibodies. **A.** Analysis of the P-loop phosphorylation site S465. **B.** Analysis of the activation loop phosphorylation site S605.

Next, we focused on S465, which is located in the glycine-rich or P-loop and has been recently reported as an auto-phosphorylation site [67], although the functional characterization of this residue was here restricted to *in vitro* kinase assays. The P-loop represents the second hotspot for oncogenic mutations, such as G469A [68], or RASopathy mutations such as S467A [69]. Interestingly, a S465F substitution has been also reported for colorectal and lung cancer [70, 71]. It is tempting to speculate that phosphorylation of S465 interferes either with the auto-inhibition imposed by the hydrophobic interaction between P- and activation loop [68], or MEK binding [72]. We now extend the *in vitro* findings by Holderfield et al. [67] by showing that *Braf*^−/−^ MEFs reconstituted with B-Raf^S465A^ display comparable pMEK/pERK levels than B-Raf^WT^, either in the absence or presence of H-Ras^G12V^::ER^TM^ release (Figure 5A). Interestingly, the two phospho-mimetic mutants, B-Raf^S465D^ and B-Raf^S465E^, behaved differently in this system. MEFs reconstituted with B-Raf^S465D^ presented a similar degree of MEK/ERK phosphorylation like B-Raf^WT^ and B-Raf^S465A^ expressing cells. Likewise, all three B-Raf proteins displayed similar electrophoretic mobility shifts (EMS) upon H-Ras^G12V^::ER^TM^ release indicating a similar pattern of PTMs. In contrast, MEFs expressing B-Raf^S465E^ showed lower pMEK/pERK levels compared to B-Raf^WT^ and B-Raf^S465A/D^ expressing cells and only a small and very discrete EMS upon H-Ras^G12V^::ER^TM^ release. This suggests that the glutamate substitution of S465 impairs the signaling potential of B-Raf and its activation dependent PTMs contributing to a maximum EMS. The strong negative charge introduced by the glutamate residue (or phosphate group) could either impair ATP coordination or block recognition of B-Raf by the HSP90 co-chaperone Cdc37, which binds to Raf kinases *via* its consensus motif GS^465^GSFG [73]. Both mechanisms would explain the loss of B-Raf activity.

Another site that was phosphorylated in B-Raf complexes from all three cell lines was S605, located in the activation loop. This evolutionary conserved residue is closely located to the T^599^VKS^602^-motif and is required for maximum B-Raf activity [22, 24], suggesting a potential role in Raf regulation. Furthermore, substitutions of S605 by other residues are listed in the Catalogue of Somatic Mutations in Cancer (COSMIC; http://www.cancer.sanger.ac.uk/cancergenome/projects/cosmic/), albeit at very low frequency compared to other activation segment mutations such as V600E/K. In order to analyze the role of this phosphorylation site and the so far unknown relevance of its mutation for the signaling potential of B-Raf, we replaced S605 by an alanine residue to prevent phosphorylation, by a phospho-mimetic glutamate residue or by a glycine residue, as the latter substitution represents the most frequently observed S605 alteration in the COSMIC database. Surprisingly, however, these substitutions had little to no effect on basal or oncogenic H-Ras stimulated B-Raf activation (Figure 5B). In fact, these S605 substitutions rather slightly reduced the MEK phosphorylation potential of these B-Raf mutants upon H-Ras^G12V^::ER^TM^ release. Furthermore, they did not induce paradoxical MEK/ERK phosphorylation, as it has been described for inhibitor-bound B-Raf or kinase-dead mutants such as B-Raf^D594A^ in this context [13], or show any cooperativity with oncogenic Ras, as we have recently shown for the intermediate activity B-Raf^F595L^ mutant [74]. Thus, phosphorylation of S605, at least by itself, and the cancer-associated S605G mutation are neither playing a pivotal role in B-Raf activity nor as an oncogenic driver, respectively.

### Dynamic phosphorylation events in the hinge region of B-Raf

**Figure 6:**
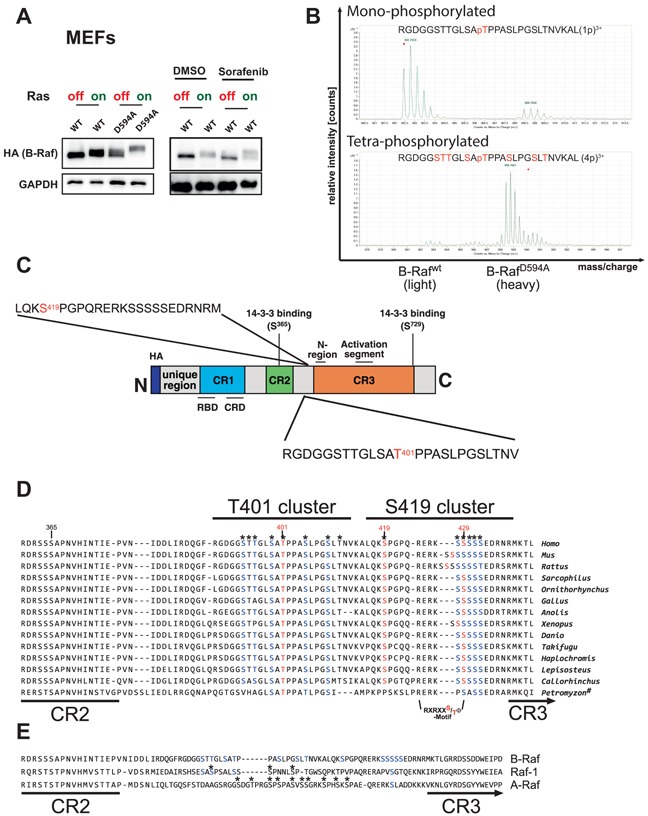
The HR of B-Raf contains two conserved and isoform-specific phosphorylation clusters contributing to the prominent EMS observed under conditions of B-Raf inhibition/inactivity **A.** Oncogenic Ras induces a marked EMS of B-Raf^D594A^ (left) or B-Raf^WT^ (right) in sorafenib-treated MEFs. **B.** Plot showing the relative abundance of the mono-phosphorylated and tetra-phosphorylated peptide encompassing T401 in B-Raf purifications from MEFs expressing B-Raf^WT^ (light medium) and B-Raf^D594A^ (heavy medium). The peptide RGDGGSTTGLSAPTPPASLPGSLTNVKAL (pos. 389-416) was identified as mono- (upper panel), di-, tri- and tetra- (lower panel) phosphorylated. Position 401 was identified as phosphorylated in all versions of the peptide. Additional sites could not be unambiguously localized and are marked in red. Note the abundance of tetra-phosphorylated peptides in peptide preparations from the B-Raf^D594A^ mutant compared to those from B-Raf^WT^. **C.** Position and sequence of the HR peptides differentially phosphorylated upon kinase inhibition or inactivity. The putative initial phosphorylation sites T401 and S419 are indicated by red letters. **D.** Alignment of the hinge region located between the CR2 and the CR3 as defined by [110]. Asterisks indicate confirmed or potential phospho-acceptor sites replaced by alanine residues in the T401 and S419 cluster mutants. **E.** ClustalW2 based alignment of three human Raf-paralogues: A-Raf (NP_001243125), B-Raf (P15056.4) and Raf-1 (P04049.1)). Sites at an equivalent position to the HR phosphorylation sites identified in B-Raf are highlighted in blue. Asterisks indicate phosphorylation sites previously identified in Raf-1 [77] and A-Raf [75].

We also applied SILAC-based MS to obtain quantitative insights into the dynamics of B-Raf phosphorylation under certain conditions. For example, B-Raf, rendered catalytically inactive by either the D594A mutation or by binding to various ATP-competing Raf-inhibitors such as sorafenib, experiences a pronounced EMS in cells expressing oncogenic Ras (Figure 6A) [13, 16]. To identify the phosphorylation events associated with B-Raf inhibition, we complemented *Braf* deficient MEFs with either B-Raf^WT^ or catalytically inactive B-Raf^D594A^ and compared the relative abundances of B-Raf derived phospho-peptides. This analysis revealed a significant increase of phospho-peptides encompassing T401 and S419 in B-Raf^D594A^ preparations compared to those from B-Raf^WT^. Importantly, this analysis demonstrated that, in addition to the previously identified T401, S419 and S429 sites [26], the hinge region of B-Raf (HR) was additionally phosphorylated at multiple residues, thereby representing previously unrecognized phosphorylation clusters (Figure 6B/6C and Supplementary Tables S6 and S9). Likewise, sorafenib treatment induced an increase in the abundance of tetra-phosphorylated peptides encompassing T401 (Supplementary Tables S6 and S9). In both cases, single phosphorylated peptides containing either T401 or S419 could be identified in IPs from control cells, suggesting that these residues are probably “priming” sites that are also phosphorylated under basal conditions. Therefore, we will refer to these areas in the HR as the T401 and S419 cluster in the following. Although it is sometimes difficult to pinpoint the exact phosphorylation site in a multi-phosphorylated peptide, it seems that, in case of the latter peptide, S419 is already phosphorylated in B-Raf^WT^, while the C-terminally located serine cluster becomes completely phosphorylated in B-Raf^D594A^. In case of the peptide encompassing T401, it is more difficult to allocate the sites in addition to phospho-T401. Based on the mass spectra, however, it is obvious that additional residues located N- and C-terminal of T401 become phosphorylated in B-Raf^D594A^ or B-Raf^WT^ purified from sorafenib treated cells. Interestingly, T401 and the N-terminally located phosphorylation sites were also phosphorylated in membrane-tethered and hence activated B-Raf^CAAX^ (Supplementary Tables S6 and S8). A protein sequence alignment revealed that the phosphorylation sites in these residues are highly conserved during vertebrate evolution (Figure 6D). The multi-phosphorylation of the T401 and S419 encompassing peptides is of particular interest in the light of the isoform-specific regulation and function of Raf-kinases. Indeed, as already pointed out previously [75], the three mammalian Raf-isoforms display considerable differences in their amino acid sequences in the HR between CR2 and CR3 and consequently in terms of their potential phosphorylation sites and motifs (Figure 6E). Interestingly, work by the Rapp and Morrison groups, which have mapped and functionally characterized these phosphorylation sites in A-Raf and Raf-1, suggests that they are regulated and contribute to Raf signaling in an isoform-specific manner [75-77]. Indeed, the majority of phosphorylation sites in A-Raf and Raf-1 represent *bona fide* phosphorylation sites for proline-directed kinases such as ERK, as it was also confirmed experimentally [75, 77, 78]. In contrast, all the sites in the hinge region clusters in B-Raf, except for T401 and S419 themselves, do not conform to phosphorylation motifs of proline-directed kinases [79].

### The hinge region phosphorylation events strongly contribute to the EMS of B-Raf

**Figure 7:**
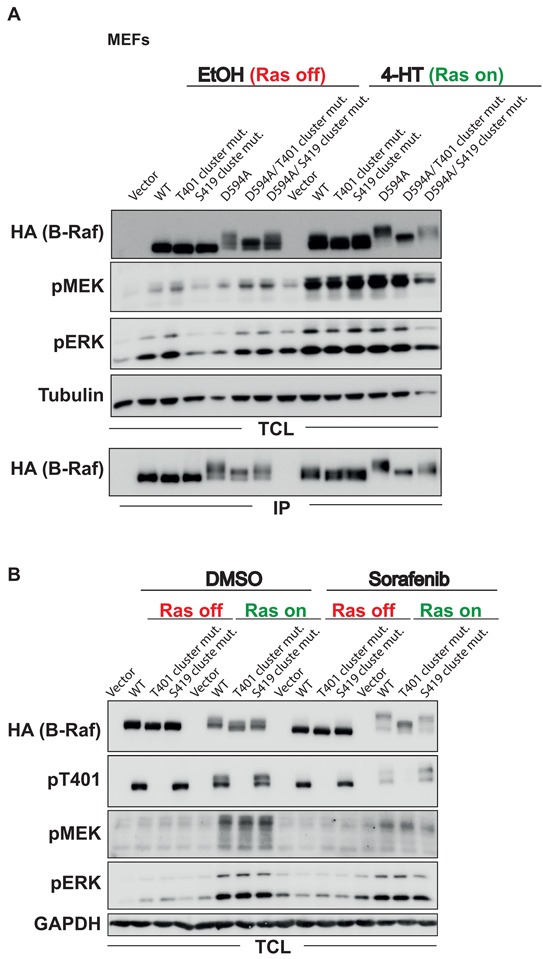
The HR phosphorylation clusters contribute to the EMS associated with B-Raf inhibition or inactivity *Braf*^−/−^ MEFs expressing the ERTmH-RAS^G12V^ fusion protein were infected and treated with the indicated B-Raf expression vectors as described in Figure 1B/1C. TCLs and IPs were analyzed using the indicated antibodies. **A.** Analysis of the HR phosphorylation clusters in the context of the wildtype protein and the kinase-inactivating D594A mutation. **B.** Analysis of the HR phosphorylation clusters in the context of the hyper-phosphorylation displayed by B-Raf^WT^ in the context of sorafenib and oncogenic Ras signaling. Note that the Ras-induced shift of B-Raf^WT^ is further enhanced by the addition of sorafenib.

Next, we asked how these phosphorylation site clusters contribute to the pronounced EMS of B-Raf^D594A^ or B-Raf^WT^ in sorafenib treated cells with active Ras signaling. Therefore, we replaced all phosphorylation sites in the T401 or the S419 cluster (Figure 6D) by alanine residues and expressed these mutants in the *Braf*^−/−^ MEF complementation system (Figure 7A). Alanine substitutions of the phosphorylation sites in the T401 cluster strongly reduced the pronounced EMS of B-Raf^D594A^ in total cellular lysates (TCLs) and immunoprecipitates. In contrast, alanine substitutions of the phosphorylation sites in the S419 cluster affected the EMS of B-Raf^D594A^ to a lesser but discernible extent. Similar findings were made for both clusters in the B-Raf^WT^ background in sorafenib treated MEFs with activated Ras signaling (Figure 7B). These analyses revealed that MEFs complemented with a B-Raf^WT^ protein lacking the phospho-acceptor sites of the T401 cluster displayed slightly elevated pMEK and pERK levels under basal conditions (Figure 7A/7B). Furthermore, using an antibody recognizing phosphorylated T401, we show that this residue was already phosphorylated in MEFs in the absence of 4-HT and that induction of oncogenic Ras signaling did not lead to an increase in T401 phosphorylation. This observation is in agreement with our SILAC-based MS results.

### Phosphorylation of the T401 cluster is vemurafenib sensitive

**Figure 8:**
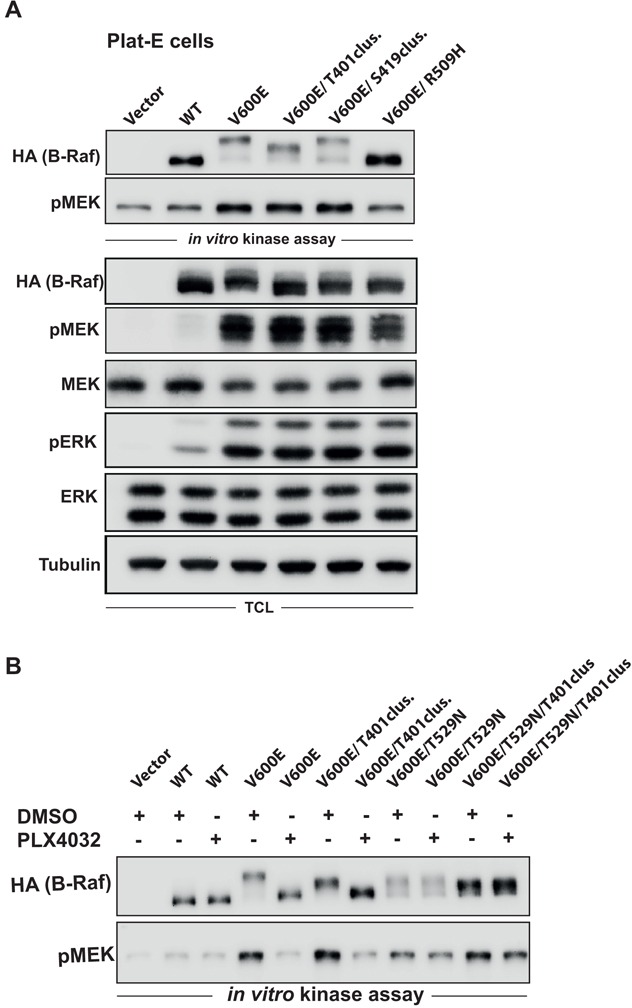
Phosphorylation of the T401 cluster is vemurafenib (PLX4032) sensitive **A.** Plat-E cells were transfected with the indicated B-Raf expression vectors. B-Raf signaling was analyzed by an *in vitro* kinase (IVK) assay and in TCLs. Note that mutation of the T401 cluster reduces the EMS of B-Raf^V600E^ in the IVK but also in the TCL despite not having an effect on its high MEK phosphorylation potential. **B.** IVK assay as performed in (**A**) except that PLX4032 was added to the bead suspension prior to addition of ATP.

Having shown that the HR phosphorylation clusters are strong contributors to the EMS of B-Raf observed in sorafenib treated cells or in the context of the D594A mutation, we next asked which kinase(s) could phosphorylate these clusters. As we previously observed in *in vitro* kinase (IVK) assays involving B-Raf^V600E^ that this hyperactive oncoprotein also undergoes a pronounced EMS, which is reminiscent to that displayed by B-Raf^D594A^ in cells with active Ras signaling, we reasoned that the HR clusters could be targeted by an auto-phosphorylation in *cis* or in *trans* (Ref. 13 and Figure 8A). This assumption was further supported by the observation that this EMS was dependent on the presence of ATP in the IVK reaction and required an intact DIF, suggesting that the associated phosphorylation events are mediated in *trans* in B-Raf^V600E^ homo- or heterodimers [13]. To further test the hypothesis that the HR is subject to such auto-phosphorylation events, we expressed a B-Raf^V600E^ protein with alanine substitutions of HR phosphorylation sites in Plat-E cells (Figure 8A). Plat-E cells, a HEK293 derivative, were chosen as they are highly suitable assays for transient transfection assays as a basis for IVK [13]. Indeed, alanine substitutions of the T401 but not of the S419 cluster significantly affected the EMS of B-Raf^V600E^ in IVK assays and also in TCLs. Importantly, as mutation of neither the T401 nor the S419 cluster had a significant effect on the IVK activity or the MEK/ERK phosphorylation potential of B-Raf^V600E^ in cells (Figure 8A), we can rule out that these alterations block the enzymatic activity of the oncoprotein. To further demonstrate that the maximum EMS of B-Raf^V600E^ is driven by phosphorylation of the T401 cluster, we added vemurafenib (PLX 4032) to the IVK reaction prior to the addition of ATP (Figure 8B). This inhibitor accelerated the electrophoretic mobility of B-Raf^V600E^ but not of B-Raf^V600E/T529N^, a gatekeeper mutant resistant towards various Raf inhibitors [80]. This indicates that the kinase activity of B-Raf^V600E^ is essential for this shift. The migration of B-Raf^V600E/T529N^ as a doublet could be explained by the fact that this gatekeeper mutation also reduces its IVK activity [81]. Consequently, some B-Raf molecules are probably not completely phosphorylated and therefore migrate faster in the gel. Importantly, B-Raf^V600E/T529N/T401cluster^ migrates faster than the B-Raf^V600E/T529N^ mutant, further demonstrating that the phosphorylated T401 cluster contributes to the IVK EMS (Figure 8B). Taken together, these observations confirm that phosphorylation of the T401 cluster can be achieved *in vitro* and that this cluster contributes to the phosphorylation status of B-Raf^V600E^ in cells prior to lysis. As we will further discuss below, the DIF dependency, as demonstrated by the effect of the R509H mutation, and vemurafenib sensitivity of this process suggest an auto-phosphorylation *in trans.*

### Loss of the T401 phosphorylation cluster enhances cellular transformation

**Figure 9:**
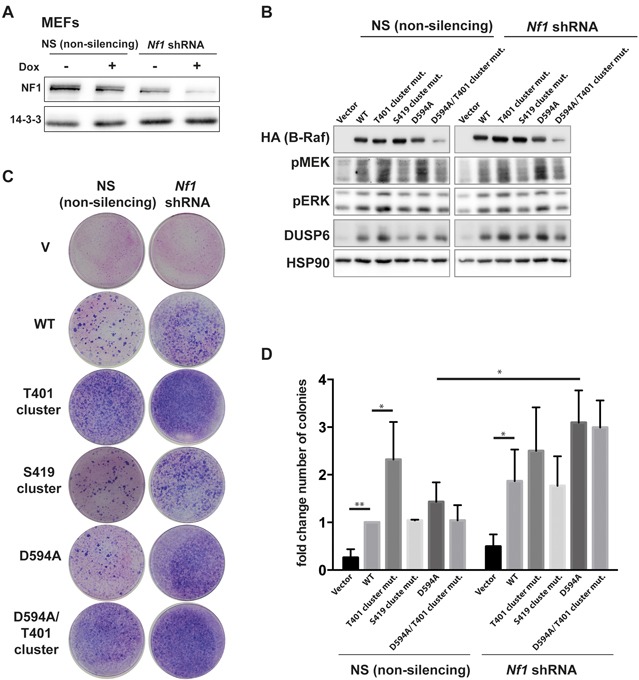
The B-Raf^T401^cluster suppresses the transforming potential of B-Raf^WT^ MEFs were infected with a lentiviral vector allowing the doxycycline (dox) inducible expression of either *Nf*1-specific or non-silencing control shRNA. MEFs were then infected with retroviral vectors encoding the indicated B-Raf proteins and then exposed to dox. **A.** Western Blot demonstrating NF1 depletion after five days of Dox treatment. **B.** Western blot analysis of the MEK/ERK pathway five days after dox addition. Samples were run on the same gel in non-contiguous set-up and intervening lanes were cropped out. **C.** MEFs transduced with dox inducible expression cassettes for either a non-silencing control or *NF*1 specific shRNA were transduced with the indicated B-Raf expression vectors, grown in the presence of dox and stained with Giemsa to reveal foci after 14 days. **D.** Bar graph showing number of colonies from three independent infections. Colony numbers in MEFs infected with the pTRIPZ non-silencing construct and pMIG/HA-BRAF^WT^ were arbitrarily set in each individual infection to 1. Asterisks indicate relevant statistically significant effects (2-way ANOVA; uncorrected Fisher's LSD test); ** p<0.001; *p<0.05. Further statistics are provided in Supplementary Table S10.

As conservation of the T401 cluster during vertebrate evolution suggests an important function, we next addressed its biological relevance. As mutation of both clusters had no discernible impact on the MEK/ERK phosphorylation potential of the highly active B-Raf^V600E^ oncoprotein (Figure 8A), we decided to address their role in the background of B-Raf^WT^, either under basal conditions or in the presence of oncogenic Ras. However, the combination of re-expressing B-Raf, in particular B-Raf^D594A^, with oncogenic Ras strongly reduced cellular adhesion over the time course of two weeks that was necessary for focus formation. Consequently, no stable foci were formed. Therefore, we sought to test the HR mutants in a cellular system with elevated activity of endogenous Ras. To this end, we used MEFs with a doxycycline inducible knockdown of neurofibromatosis 1 (NF1), a potent negative regulator of Ras proteins and tumor suppressor gene product [82, 83] (Figure 9A). Depletion of NF1 results in increased levels of GTP-loaded Ras isoforms and consequently increases downstream signaling [83]. However, the effects of NF1 depletion on pMEK/pERK levels and immediate early gene products such as DUSP6 were rather subtle and could be explained by the dynamic counter-regulation of ERK pathway activity by multiple transcriptional and post-translational feedback loops acting at various levels of this pathway [78, 84-86]. Therefore, as a biological endpoint integrating mitogenic signaling events over a period of 14 days, we studied focus formation of MEFs transduced with the various B-Raf mutants (Figure 9C/9D). In cells transduced with non-silencing control shRNA, expression of B-Raf^WT^ moderately increased the number and diameter of transformed foci compared to MEFs infected with the empty vector control construct, while expression of B-Raf^WT^ and *NF1* knockdown cooperated in a more efficient focus formation. Focus formation was even more pronounced in MEFs transduced with B-Raf^T401cluster^ and, as expected from the well-established cooperation of kinase-inactive B-Raf mutants with active Ras [16], also in cells expressing B-Raf^D594A^ proteins. Commensurate with its lack of an effect on MEK/ERK phosphorylation (compared to B-Raf^WT^ expressing MEFs), alanine substitution of the phosphorylation sites in the S419 cluster had no discernible impact on the transformation potential of B-Raf^WT^. In summary, our data suggest that the hyper-phosphorylation of the T401 cluster represents a negative signaling event limiting the activation of B-Raf^WT^.

## DISCUSSION

Using a proteomic approach, we obtained detailed new insights into the interaction repertoire of B-Raf and the spectrum of its phosphorylation sites. In respect to the former, we could identify a series of novel interaction partners and now also supply information regarding the dynamics of their recruitment. For example, our SILAC experiments have shown that the interaction of B-Raf^D594A^ with Ras-isoforms (or the H-Ras^G12V^::ER^TM^ fusion protein as reflected by the enrichment of estrogen receptor derived peptides) or Raf-1 or A-Raf is strongly enhanced by 4-HT treatment. These findings validate our approach and the identification of novel or less-established dynamic interaction partners such as Kidins220/ARMS (Figure 3D) or ECM29. Unfortunately, we were not able to confirm the latter by Western blot analysis due to the lack of suitable antibodies.

Amongst the novel interaction partners of B-Raf, the a subunits of hetero-trimeric G-proteins are of particular interest for two reasons. Firstly, this interaction could represent a “missing link” in the crosstalk between RTKs and G protein-coupled receptor (GPCR) signaling systems. Indeed, while trans-activation of RTKs by GPCRs is a relatively well-established phenomenon [87, 88], other studies proposed that RTKs could also signal *via* GPCRs or “hijack” their cytoplasmic signal transducers such as hetero-dimeric G-proteins [89]. Secondly, mutated Gα subunits are emerging as potent oncoproteins in human cancer. For example, mutations likely to impair GTP hydrolysis have been identified in the Gα subunits genes *GNAS*, *GNAI2* and *GNAO1* [90, 91]. Furthermore, *GNAQ* and *GNA11* mutations blocking GTP hydrolysis were identified in melanocytic neoplasms such as uveal melanoma and blue nevi [92, 93]. Importantly, uveal melanoma shares with cutaneous melanoma the addiction to high levels of ERK activity, but lack *NRAS* or *BRAF* mutations that are a hallmark of cutaneous melanoma [92]. However, the immediate critical effectors downstream of mutated Gα subunits are still unknown and currently a more indirect activation of the ERK pathway via Gα subunit mediated activation of phospholipase C/Ras and protein kinase C isoforms is discussed in the field [94-96]. Although our data do not rule out an involvement of these enzymes, our discovery that B-Raf signalosomes purified from three distinct cell types contain Gα subunits suggests a more direct link between heterotrimeric G-proteins and the ERK pathway module. This scenario is further supported by our finding that deamidation of Gα subunits by PMT, and thereby preserving their GTP loaded state, increases their interaction with B-Raf.

In addition to the multiple and dynamic protein-protein interactions, the high number of phosphorylation sites as well as the emerging ubiquitination (this study, [97, 98], acetylation (Phosphosite database) and methylation events [99] further demonstrate the role of B-Raf as a signaling hub integrating multiple signaling pathways. In this study, we have focused on establishing a catalog of phosphorylation sites identified on B-Raf proteins purified from various cell types and conditions. In addition to well-established phosphorylation sites linked to B-Raf activation (S446; [100]), 14-3-3 binding (S365/S729; [25, 27, 28]) and feedback regulation (S751/T753; [29, 30]), we have confirmed and further characterized more recently identified sites such as S151 and discovered several novel ones. Indeed, SILAC-based MS analyses enabled us to identify two dynamically regulated phosphorylation clusters located in the HR of B-Raf, which are both mainly responsible for a long-standing phenomenon in B-Raf research, the dramatic and dimerization-dependent EMS of B-Raf^V600E^, of kinase-dead B-Raf^D594A^ or drug-bound B-Raf^WT^ (with the latter two in the context of oncogenic Ras signaling; [13, 16]). This HR promoted “super-shift” is distinct from the typical shift of Raf-kinases that is triggered by growth factor or antigen receptors, or by expression of oncogenic Ras and that is driven by their ERK mediated feedback phosphorylation [26, 29, 48, 78]. Instead, by using B-Raf^V600E^ mutants rendered resistant towards the B-Raf inhibitor vemurafenib, we demonstrate that the T401 cluster in the HR is phosphorylated by a mechanism involving their own kinase activity. At first glance, these findings contradict our observation that the T401 cluster is hyper-phosphorylated in complexes purified from MEFs that either have been complemented with B-Raf^WT^ and treated with sorafenib, or that express kinase-dead B-Raf^D594A^. In both settings, one would expect any auto-phosphorylation to be disabled. However, we have shown previously that the R509H mutation impairs the “super-shift” of B-Raf^V600E^ (*in vitro* and *in vivo*), of B-Raf^D594A^ and even that of B-Raf^WT^ in the presence of oncogenic Ras and sorafenib [13]. This suggests that HR phosphorylation occurs *in trans* and could be even mediated by another Raf-isoform such as Raf-1 or A-Raf that are both highly enriched in B-Raf^D594A^ complexes and should be strongly activated due to the paradoxical action of kinase-dead B-Raf [13, 16]. This model is supported by our recent study showing that a B-Raf^D594A^ mutant (B-Raf^D594A/AVKA^) with alanine substitutions of the activation loop phosphorylation sites T599 and S602 exhibits an impaired EMS in the context of H-Ras^G12V^::ER^TM^ release and recruits significantly less Raf-1 than B-Raf^D594A^ proper [24]. As the differential between the EMS of B-Raf^D594A^ and B-Raf^D594A/AVKA^ is reminiscent of that between B-Raf^D594A^ and B-Raf^D594A/T401cluster^, it is tempting to speculate that the contrasting EMS of B-Raf^D594A^ and B-Raf^D594A/AVKA^ are caused by the less efficacious recruitment and transactivation of Raf-1 (see Ref. [24] for further discussion). Likewise, as the application of 10 μM sorafenib does not completely eradicate MEK/ERK phosphorylation and also strongly stabilizes B-Raf/Raf-1 complexes ([13, 16]; Figure 2B), one might speculate that dimers in which one B-Raf is drug-bound, and hence a potent allosteric activator, mediates hyper-activation of a drug-free protomer that in turn phosphorylates the HR in the activating protomer *in trans*. Furthermore, as we and others have shown that B-Raf^V600E^ exhibits an increased homo-dimerization potential relative to B-Raf^WT^ [13, 23], it is conceivable that the super-shift displayed by this oncoprotein in IVK assays is strongly promoted by trans-phosphorylation of the HR in the precipitated dimer and the presence of phosphatase inhibitors in this setting. The identification of these auto-/transphosphorylation events invites for the identification of Raf consensus phosphorylation sites.

But what is the biological meaning of HR phosphorylation? While the precise role of the S419 cluster, which precedes one of the three established inhibitory AKT phosphorylation site S429 [101], remains to be characterized, we could show that mutation of the T401 cluster enhances the transformation potential of B-Raf^WT^. As the structure of the HR is unknown and is probably highly disordered, we can only speculate about the precise molecular mechanisms by which phosphorylation of the T401 cluster contributes to B-Raf downregulation. For example, it is conceivable that HR phosphorylation counteracts the attachment to the negatively charged inner leaflet of the plasma-membrane by bulk changes in the electrostatic landscape of this B-Raf region [102]. Such a scenario has been shown for the yeast MAPK scaffold Ste5, which harbors a cluster of CDK phosphorylation sites in its basic membrane binding region [103]. Alternatively, HR cluster phosphorylation could contribute to loosen B-Raf homo- or heterodimers in a mechanism already suggested by the Kölch and Morrison laboratories for the phosphorylation of S151 and the C-terminal SPKTP-motif [26, 30, 66]. Interestingly, T401, as a single site and not as a member of a cluster, was also implicated in this process [26, 44, 66].

**Figure 10:**
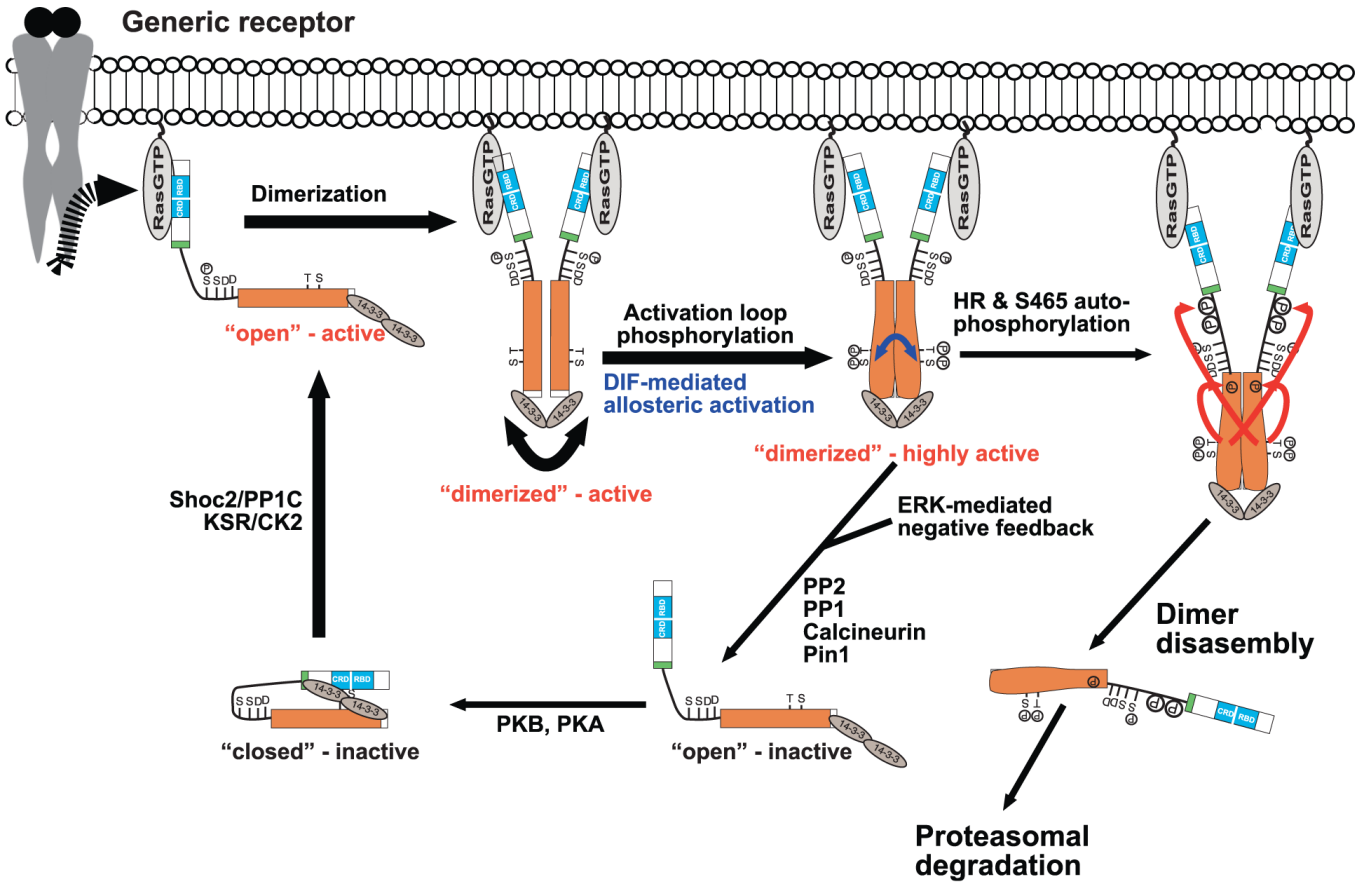
Model summarizing some key findings from the MS analyses of B-Raf complexes Following recruitment by Ras and homo- or heterodimerization mediated transactivation, the Raf dimer becomes fully active following activation loop phosphorylation and DIF mediated allosteric activation (blue bent double-headed arrow). For simplicity, we show only the situation for B-Raf homo-dimers, but we posit a similar mechanism for heterodimers. Under normal activation, the B-Raf homo-or heterodimer is disrupted by feedback phosphorylation and the protomers are recycled to a closed inactive conformation by the action of Pin1 and various phosphatases such as Calcineurin, which dephosphorylates pT401 [44], or the other phosphatases that were identified in B-Raf complexes (Supplementary Table S1). If the activity of the dimer persists, the protomers will auto-phosphorylate themselves either *in cis* or trans at S465 in the kinase domain (orange) or at the HR indicated by the large phosphate group symbols N-terminal of the N-region (SSDD-motif). This hyper-phosphorylation might then lead to dimer disassembly (in concert with the ERK mediated feedback phosphorylations) and/or degradation. See text for further details. Model has been extended from [24].

In summary, our data imply that following Ras-mediated recruitment and dimerization, the HR becomes highly phosphorylated *in trans* thereby contributing to the downregulation of B-Raf activity or expression (Figure 10). In regard to the latter possibility, it should be noted that the T401 equivalent in LIN-45 is part of a phospho-degron promoting its degradation via the Skp1/Cul1/F-box (SCF) complex [104]. As others and we observed previously that B-Raf^D594A^ expression often appears reduced in the presence of oncogenic Ras [13, 16], which is the setting in which T401 cluster phosphorylation occurs, it is tempting to speculate that HR phosphorylation contributes to the control of B-Raf turnover. This concept, which will be addressed in future studies, is further supported by the observation that B-Raf becomes ubiquitinated and interacts with Skp1 as well (this study; [98]). Indeed, while our manuscript was under revision, Hernandez et al. reported that alanine substitution of T401 and S405, a site that was also highlighted by the MASCOT software in our data sets but which was not included in Supplementary Table S6 due to its low score, increased the half-life of B-Raf^V600E^ [105]. Moreover, based on the dynamics of HR phosphorylation revealed by our SILAC approach, we posit that the HR cluster phosphorylation involves a kinetic proofreading mechanism in which only the processive integration of multiple phosphorylation sites can execute a decision such as the disruption of a protein-protein or –lipid interaction or tagging the protein for its degradation [106]. As we found T401 and S419 to be phosphorylated under normal growth conditions, we further postulate that these residues serve as pioneering or seeding sites that might predispose the clusters for maximum phosphorylation. Although this concept needs to be addressed experimentally, the fact that both presumptive pioneering sites are somatically mutated in human cancer (Supplementary Table S6) and the fact that the T401A single mutant already elevates B-Raf signaling output ([44] and our own preliminary data) argue in this direction.

Finally, our study shows that B-Raf is a heavily phosphorylated protein. Although many of the sites identified or confirmed in broader settings by our study await their functional characterization, it is becoming clear that negative regulatory sites such as S151, S365, the T401 cluster, S429, S465 and the SPKTP-motif outnumber the few sites with a clear positive role such as S445, the T^599^VKS^602^-motif and S729. Thus, in line with its role as a potent proto-oncogene product, B-Raf is subject to multiple layers of negative regulation, allowing its fine-tuning in a spatio-temporal manner. Our study further highlights the usefulness to combine phospho-proteomic with sequencing data of tumor genomes, as both data sets can complement each other in the elucidation of phosphorylation site function.

## MATERIALS AND METHODS

### Antibodies and reagents

Raf-B (F7, sc-5284), Raf-B (H145, sc-9002), Raf-1 (C12, sc-133), β-actin (C4, sc-47778), α-Tubulin (B-5-1-2, sc-23948), and NF1 (sc-67)were fromSanta Cruz Biotechnology; HA (3F10) monoclonal rat from Roche Bioscience. Antibodies against (p)ERK, (p)MEK were purchased from Cell Signaling Technologies. The Gα switch antibody and the source of PMT were described recently [96]. DUSP6 was purchased from LsBio. Anti-Kidins220 antibody (ab34790) and GAPDH were purchased from abcam. Anti-phospho-tyrosine 4G10 antibody was obtained from Upstate Biotechnology/ Millipore. Raf-B pT401 was from Epitomics. The anti-pS151 antibody has been described previously [63].

### Plasmids

The pFLU/HAchB-*raf*, pMIG/HAch*B-raf*, pAloxP-puro, pMIG/HAh*BRAF* and pMIG/*BRAF*-His/Myc expression vectors were described previously [13, 29, 107]. The pCDNA3.1loxPuro-HAch*BRAF* vector was a kind gift from Dr. Niklas Engels (Göttingen) and contains the HA-tagged chicken c-Rmil/B-Raf cDNA [29]. Mutations were introduced with standard site-directed mutagenesis. Oligonucleotide sequences and cloning strategies are available on request. To generate the dox-inducible pTRIPZ-NF1 construct, parts of the shRNAmir cassette were isolated from pGIPZ V2LMM_194180 (OpenBiosystems) and subcloned into pTRIPZ (OpenBiosystems). The sequence of the mature shRNA targeting NF1 is 5′-TAAATTTAAGGCTTGTTAC-3′.

### Cell lines

The propagation of DT40, PC12, MCF-10AecoR, Plat-E cells and *Braf*^−/−^ MEFs expressing the H-Ras^G12V^::ER^TM^ fusion protein has been described [5, 13, 29, 108]. For the MS analysis of endogenous B-Raf complexes, MEFs from *Braf*^floxAVKA^ mice were used without previous Cre activation [24]. The generation and culture of immortalized MEFs from conditional *Braf* deficient mouse embryos (MEF#3 *Braf^floxE12/floxE12^*; pMIBerry/CreERT2) were described previously [13]. *The* DT40 subline allowing the 4-hydroxy-tamoxifen (4-HT) inducible deletion of the chicken c-*mil*/*raf*-1 and c-R*mil*/B-*raf* genes, DT40MCM/*raf-1*^flE3^/*B-raf*^flE6^ (DT40^floxRaf^ in short) has been described [5]. DK37^+^ cells were complemented with pMIG/HAchB-*raf* as described previously [5]. For inducible complementation analyses, DT40^floxRaf^ cells (subclone DK37) were first converted to DT40^Raf-less^ cells by 4-HT exposure for 24 h as described previously [5] and subsequently cultured in normal culture medium for additional four days. Subsequently, the cells were electroporated with 20 μg *Ahd*I-linearised pCDNA3.1loxPuro-HAch*BRAF* and selected with 0.5 μg/ml puromycine. PC12 cells were transfected by using Gene Juice (Novagen), kept in differentiation medium and scored for differentiation 6 days later as described previously [27, 29]. Cells were defined as differentiated if their neurites were longer than the size of two cell bodies. In order to generate MEFs with a conditional knockdown of NF1, SV40 Tag immortalized murine embryonic fibroblasts (MEF #3 FloxE12 pMIBerry/CreERT2; [13]) were infected with lentiviral particles containing either the pTRIPZ-non silencing (OpenBiosystems) or pTRIPZ-NF1 construct as described previously [13]. MEFs were selected with 4 μg/ml puromycine (Carl Roth). Retro- and lentiviral infections, incl. focus formation assays, were performed as described previously [13, 24, 74].

### Immunoprecipitation, *in vitro* kinase assays and western blotting

These procedures were conducted as described in detail previously [13]. In brief, cells were lysed in normal lysis buffer (NLB: 50 mM Tris/HCl, pH 7.5; 1% Triton X-100; 137 mM sodium chloride; 1% glycerin; 1 mM sodium orthovanadate; 0.5 mM EDTA; 0.01 μg/μl leupeptin, 0.1 μg/μl aprotinin, 1 mM AEBSF). Blotted proteins were visualized with horseradish peroxidase-conjugated secondary antibodies (Roche) using the SuperSignal West Femto Maximum Sensitivity Substrate (Thermo Scientific) and either a LAS-4000 reader (FujiFilm) or a Fusion Solo chemiluminescence reader. Densitometry was performed using MultiGauge software (FujiFilm) or FusionCapt software (Vilber Lourmat). MS analysis of immunoprecipitated B-Raf complexes is described in detail in the expanded view.

### Ras/Raf interaction assays

HEK293T cells were co-transfected with pMIG/HAh*BRAF* and V5-Ras^G12V^ expression vectors in a 2:1 ratio as described previously [13]. Cells were harvested 24 to 48 h post transfection with cell lysis buffer (50 mM Tris-HCl pH 7.4, 1 % Triton-X100, 137.5 mM NaCl, 1 % glycerol, 1 mM sodium orthovanadate, 0.5 mM EDTA pH 8, protease inhibitor cocktail (Roche)). Immunoprecipitations were performed as described previously [108]. AlphaScreen assays were performed in white 384-well OptiPlates using AlphaScreen Protein A Acceptor and Streptavidin Donor beads provided in a suspension of 5 mg/ml (all PerkinElmer). A detailed description of this assay is available upon request.

### Mass spectrometry

Detailed information on mass spectrometry procedures is provided in the supplement. The mass spectrometry proteomics data have been deposited to the ProteomeXchange Consortium [109] via the PRIDE partner repository with the dataset identifier PXD003256.

## SUPPLEMENTARY METHODS FIGURE AND TABLES






















