# Supplementary material for: Phospho-proteomic analyses of B-Raf protein complexes reveal new regulatory principles
Source: Oncotarget. 2016 Mar 28;7(18):26628–52. doi: 10.18632/oncotarget.8427 (PMC5042004; doi:10.18632/oncotarget.8427)
Supplement: Supplementary file 1 [file oncotarget-07-26628-s001.pdf]

SUPPLEMENTARY METHODS

Large scale purification of B-Raf complexes for MS/MS-based analysis

For labelling cells were grown in media containing L-arginine (Arg0) and L-lysine (Lys0), L- lysine–$^{2}H_{4}$ (Lys4), and L-arginine–U-$^{13}C_{6}$ (Arg6), or L-lysine–U-$^{13}C_{6}$-$^{15}N_{2}$ (Lys8) and L- arginine–U-$^{13}C_{6}$-$^{15}N_{4}$ (Arg10) to generate 'light', 'medium' and 'heavy' labelled cells, respectively. Fully labelled cells were grown to 80-90% confluence, approximately 400-800 million cells per treatment. Cells were split in the SILAC media six times to achieve sufficient labelling. HA-tagged B-Raf proteins were purified using anti-HA 3F10 agarose from cells lysed in NLB.

The co-immunoprecipitation eluate was reduced by DTT (1 mM) (Sigma-Aldrich) for 30 min at 75°C and alkylated using iodoacetamide (5.5 mM) (Sigma-Aldrich) for 30 min at 25°C. The protein mixture was separated by SDS PAGE (4-12% Bis Tris mini gradient gel, NuPAGE (Invitrogen)), gel lanes were cut into 10 equal slices, samples in gel digested using trypsin (Promega, Mannheim, Germany), and resulting peptide mixture was processed on STAGE tips. For phospho-proteomics, B-Raf complexes were subject to a multi-enzyme digest using a previously published protocol (Schlosser et al, 2005) and the following enzymes: Trypsin (modified, sequencing grade), elastase, and recombinant proteinase K (PCR grade) were from Roche (Mannheim, Germany), thermolysin was from Sigma-Aldrich Chemie, GmbH (Taufkirchen, Germany).

TiO_2 based enrichment and elution 1-10 microliters of a 50% slurry of TiO_2 in 30 mg/mL DHB were added to each fraction and flow-through and incubated for 30 min at room temperature. Beads were washed with 10% ACN and 1% TFA followed by of 80% ACN and 1% TFA and finally water. Phosphopeptides were eluted using 25% ammonium hydroxide in 20% and 40% ACN, respectively. Eluted phosphopeptides were dried to less than 5 µL and resuspended in 15 µL of 0.5% acetic acid for analysis. Five microliters of each fraction were used for MS analysis.

Samples for LC-MS/MS were fractionated by nanoscale–HPLC on an Agilent 1200 connected online to a LTQ-Orbitrap XL (Thermo Scientific). Peptides were separated over a linear gradient from 10-30% ACN in 0.5% acetic acid with a flow rate of 250 nl/min. All full-scan acquisition was done in the FT-MS part of the mass spectrometers in the range from m/z 356-2000 with an automatic gain control target value of 10^{6} and at resolution 60,000 at m/z 400. MS acquisition was done in data-dependent mode to sequentially perform MS/MS on the five most intense ions in the full scan in the LTQ using the following parameters target value: 5,000. Ion selection thresholds: 1,000 counts (first replicate) or 100 (second replicate) and a maximum fill time of 100 ms. Wide-band activation was enabled with an activation q = 0.25 applied for 30 ms at a normalized collision energy of 35%. Singly charged and ions with unassigned charge state were excluded from MS/MS. Dynamic exclusion was applied to reject ions from repeated MS/MS selection for 45 s. All recorded LC-MS/MS raw files were processed in MaxQuant with default parameters using Andromeda as search engine and full lengths UniProt databases with added common proteomics contaminants. For databases searching parameters were mass accuracy thresholds of 0.5 (MS/MS) and 6 ppm (precursor), maximum two missed cleavages, carbamidomethylation (C) as fixed modification and deamidation (NQ), oxidation (M), phosphorylation (STY), ubiquitination (K) and protein N-terminal acetylation as variable modifications. MaxQuant was used to filter the identifications for a FDR below 1% for peptides, sites and proteins using forward decoy searching.

Mass spectrometric analysis of B-RAF phosphorylation sites

Proteins in NuPAGE® LDS sample buffer (Life Technologies) were reduced with 50 mM DTT at 70 °C for 10 minutes and alkylated with 120 mM Iodoacetamide at room temperature for 20 minutes and separated on NuPAGE® Novex® 4-12 % Bis-Tris gels (Life Technologies) with MES buffer according to manufacturer's instructions. Gels were washed three times for 5 minutes with water and stained for 45 minutes with Simply Blue™ Safe Stain (Life Technologies). After washing with water for 2 hours corresponding gel bands were excised from the gel.

In-gel digests were performed as described in standard protocols. Briefly, the excised gel bands were destained with 30 % ACN, shrunk with 100 % ACN, and dried in a Vacuum Concentrator (Concentrator 5301, Eppendorf, Hamburg, Germany). Digests with trypsin, elastase, and proteinase K were performed overnight at 37 °C in 0.1 M NH_4HCO_3 (pH 8). About 0.1 µg of protease was used for one gel band. Peptides were extracted from the gel slices with 5 % formic acid and dried in a Vacuum Concentrator.

Phosphorylated peptides were enriched using TiO_2 (Titansphere, 5 µm particle size, GL Sciences, Japan) as described for phosphopeptides (Schlosser et al.,

2005). Briefly, peptides were re-dissolved in 10 µL 50 % acetonitrile, 0.1 % formic acid and loaded on a TiO_2 nano column (0.5 cm length, 100 µm i.d.) at a flow rate of 2 µL/min. After washing with 20 µL 30 % acetonitrile, 2 % formic acid (flow rate: 2 µL/min), phosphorylated peptides were eluted (flow rate: 2 µL/min) with 100 mM NH_4HCO_3 pH 9.

All LC-MS/MS analyses were performed on a Q-TOF mass spectrometer (Agilent 6520, Agilent Technologies) and/or on a 6340 ion trap equipped with an ETD II source (Agilent Technologies) coupled to a 1200 Agilent nanoflow system via a HPLC-Chip cube ESI interface. Peptides were separated on a HPLC-Chip with an analytical column of 75 µm i.d. and 150 mm length and a 40-nL trap column, both packed with Zorbax 300SB C-18 (5 µm particle size). Peptides were elutes with a linear acetonitrile gradient with 1 %/min at a flow rate of 300 nL/min (starting with 3% acetonitrile).

The Q-TOF was operated in the 2 Ghz extended dynamic range mode. MS/MS analyses were performed using data-dependent acquisition mode. After a MS scan (2 spectra/s), a maximum of three peptides were selected for MS/MS (2 spectra/s). Singly charged precursor ions were excluded from selection. Internal calibration was applied using two reference masses.

ETD analyses on the ion trap were performed using data-dependent acquisition mode. After a MS scan (standard enhanced mode), a maximum of three peptides were selected for ETD- MS/MS (standard enhanced mode). The automated gain control (ICC) for MS scans was set to 350000. The maximum accumulation time was set to 300 ms. The following ETD parameters were used. ICC target: 400000, reaction time: 100 ms, cut-off: 140, resonance excitation (Smart Decomp) was used for doubly charged peptides.

Peak lists (mgf files) were generated from raw data with Mascot Distiller 2.3 (MatrixScience, UK) using standard processing options for Agilent Q-TOF and Agilent ion trap. Database searching was performed with Mascot 2.4 against custom databases containing all UniProt entries with taxonomy Gallus gallus (for DT40 cells), Homo sapiens (MCF10A cells) or Mus musculus (MEFs) in addition to the B-RAF sequence with N-terminal HA-tag, S151A mutation, D594A mutation, and the CAAX motif. In addition all three custom databases contained all entries from the contaminants database from Max-Planck-Institute of Biochemistry (Martinsried, Germany).

Identified phosphopeptides from all analyses together with their corresponding features (peptide scores, retention time, mass accuracy, etc.) were extracted from Mascot .dat files using perl scripts. One .html file was generated for MD40 cells, for MCF10A cells and for MEF cells, respectively (see Supplementary information). Peptides were filtered with a fixed score cut-off of 15. For phosphorylation site analysis only rank 1 peptides with a minimum score of 23 were considered. A phosphorylation site was assumed to be reliably localized if the Mascot delta score between two positional isomers was larger than 10 (Savitski et al, 2011).

REFERENCES

1. Savitski MM, Lemeer S, Boesche M, Lang M, Mathieson T, Bantscheff M, Kuster B (2011) Confident phosphorylation site localization using the Mascot Delta Score. Molecular & cellular proteomics : MCP 10: M110 003830.
2. Schlosser A, Vanselow JT, Kramer A (2005) Mapping of phosphorylation sites by a multi-protease approach with specific phosphopeptide enrichment and NanoLC- MS/MS analysis. Analytical chemistry 77: 5243-5250.

SUPPLEMENTARY FIGURE AND TABLES

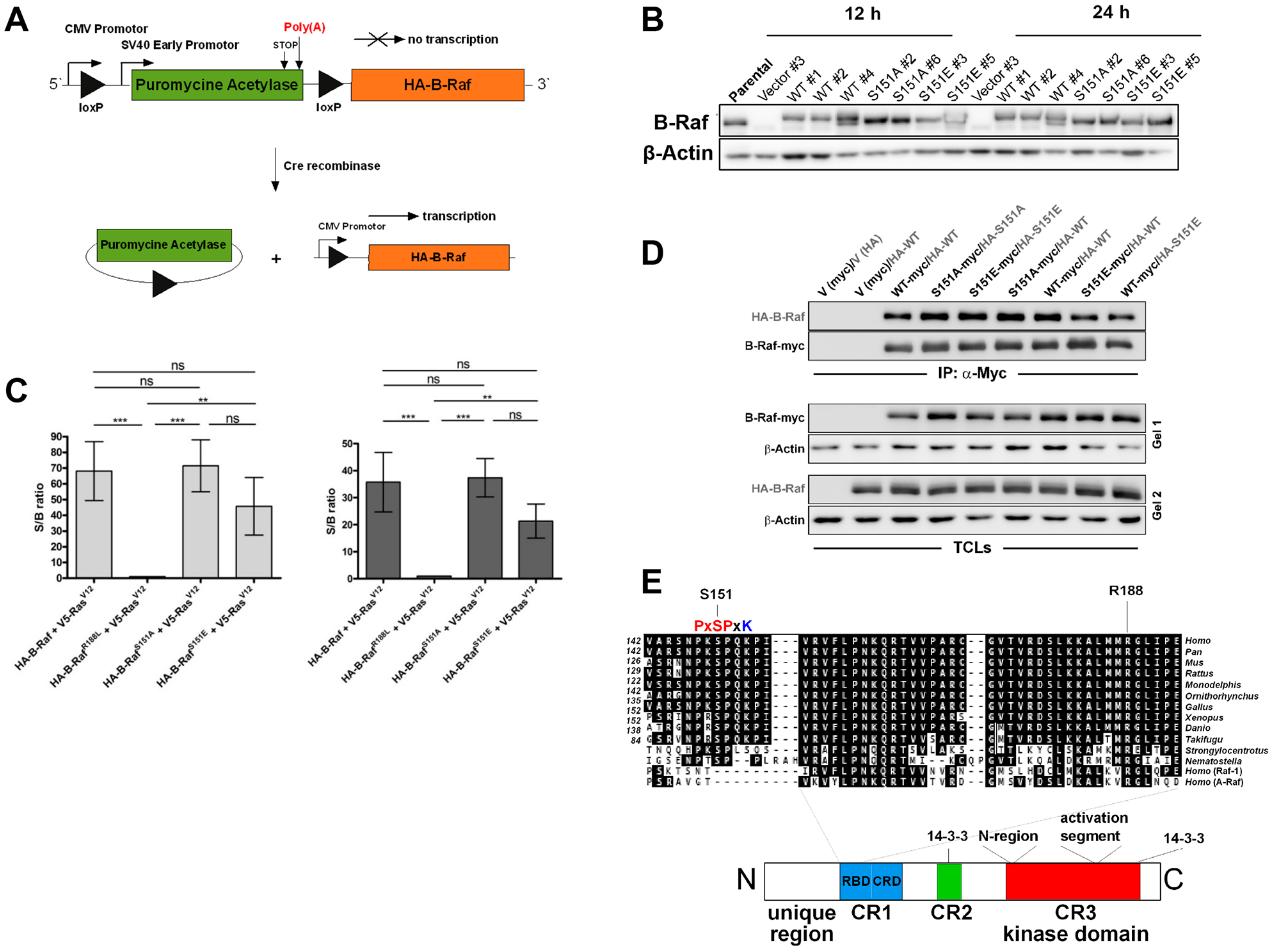

Supplementary Figure S1: (related to Figure 4). A. Strategy used in Figure 4C to re-express B-Raf proteins in B-Raf/Raf-1 double deficient DT40 cells using a Cre-loxP based system and floxed puromycine cassette preventing transcription of the HA-B-Raf cassette. **B.** Time-dependent induction of chicken HA-B-Raf expression after 4-HT induced Cre-mediated recombination in comparison to the physiological expression level of endogenous B-Raf in parental DT40 (DK37-) cells. Different cell clones either transfected with vector control, B-Raf^{WT}, B-Raf^{S151A} or B-Raf^{S151E} cDNA were induced with 200nM 4-HT and analyzed 12 and 24 h post induction. Clones WT#4, S151#5 and S151E#5 were used for the experiment shown in Figure 4C. **C.** AlphaScreen analysis with the two B-Raf mutants S151A and S151E reveals no significant difference in binding affinity to H-Ras^{G12V} compared to B-Raf^{WT}. In comparison to the Ras-binding deficient B-Raf^{R188L} mutant, B-Raf^{WT} and its two mutants with the S151A and S151E substitutions show a significant binding to H-Ras^{G12V} (P at least < 0.001). However, there is no significance in binding affinity to H-Ras^{G12V} for wild type, S151A, and S151E forms of B-Raf. The alpha screen plate was read 1 h (left) after the addition of the streptavidin donor beads as well as after overnight incubation (right). To calculate S/B ratios, AlphaScreen signals of all tested interactions were normalized to the negative control HA-B-Raf-1^{R188L} and V5-tagged H-Ras^{G12V}. The S/B ratios are shown as mean values of four independent assays each performed in triplicate. The error bars represent the SD (standard deviation) of the mean. P values were calculated with One-way ANOVA (*** P < 0.0001, ** P < 0.001, ns – not significant). **D.** Alanine or glutamate substitutions do no significantly modulate the homo-dimerization of B-Raf. The indicated HA- or Myc-tagged B-Raf proteins were co-expressed in Plat-E cells and purified with anti-Myc antibodies as described previously [13]. **E.** Alignment showing the conservation of a proline-directed phosphorylation motif at the N-terminal border of the RBD in Raf kinases from sea anemones to man. Note that only vertebrates carry the PxSPxK-motif typical for phosphorylation by CDKs [79].

Supplementary Table S1: Interaction partners for B-Raf in various cell types. Listed are proteins identified as specific interaction partners (IPs) in B-Raf immunoprecipitates from the indicated cell types. IPs (with Uniprot numbers in the first column) were deemed specific if their peptides were not found in negative control purifications or, in case of SILAC experiments, were enriched compared to control purifications. IPs were classified as novel, if they were not found in http://www.ncbi.nlm.nih.gov/gene/673, or by literature searches. If not stated otherwise, information on protein function was obtained from the "GENE" or "Uniprot" databases. DT40 = IPs from DK37+ cells complemented with B-Raf^{WT}, MEF Rec. = IPs from *Braf*$^{-/-}$ MEFs cells complemented with B-Raf^{WT} or B-Raf^{D594A}, MEF End. = IPs from MEFs expressing endogenous B-Raf; MCF-10A = IPs from MCF-10A cells expressing either B-Raf^{WT} or B-Raf^{CAAX}.

See Supplementary File 1

Supplementary Table S2: BRAF and interacting proteins were identified by SILAC-based MS comparing DT40 cells expressing *HA-BRAF* with vector control cells. Shown are proteins identified as significantly enriched (p<0.05) in two biological replicates. PEP: posterior error probability.

See Supplementary File 2

Supplementary Table S3: BRAF interacting proteins sensitive to sorafenib treatment were identified by SILAC-based MS comparing MEFs expressing HA-BRAF plus/minus sorafenib with vector control cells. Shown are proteins identified as significantly enriched (p<0.05) in two biological replicates in BRAF IPs of sorafenib treated cells compared to vector control cells. The respective figure is colored according to the SILAC ratios of BRAF IPs plus sorafenib vs. BRAF IPs minus sorafenib. PEP: posterior error probability.

See Supplementary File 3

Supplementary Table S4: BRAF^{D594A} interacting proteins were identified by SILAC-based MS comparing MEFs expressing HA-BRAF^{D594A} with HA-BRAF^{WT} cells. Shown are proteins identified as significantly enriched (p<0.05) in two biological replicates and BRAF itself. PEP: posterior error probability.

See Supplementary File 4

Supplementary Table S5: BRAF ubiquitination site was identified by MS in anti-HA IP of MEFs expressing HA-tagged BRAF. Peptide and site FDR were set to ≤ 0.01.

See Supplementary File 5

Supplementary Table S6: List of ubiquitination and phosphorylation sites.

Supplementary File 6

Supplementary Table S7: PepView data (html file) illustrating the phosphopeptides found in B-Raf complexes purified from Raf deficient DT40 cells expressing recombinant HA-tagged chicken B-Raf.

See Supplementary File 7

Supplementary Table S8: PepView data (html file) illustrating the phosphopeptides found in B-Raf complexes purified from MCF-10A cells expressing recombinant human HA-tagged B-Raf-CAAX.

See Supplementary File 8

Supplementary Table S9: PepView data (html file) illustrating the phosphopeptides found in B-Raf complexes purified from MEFs expressing recombinant human HA-tagged B-Raf, either in the presence (Ras plus) or absence of 4-HT (Ras minus).

See Supplementary File 9

Supplementary Table S10: Statistics accompanying Figure 9D.

See Supplementary File 10
