# Supplementary material for: Phospho-proteomic analyses of B-Raf protein complexes reveal new regulatory principles
Source: Oncotarget. 2016 Mar 28;7(18):26628–52. doi: 10.18632/oncotarget.8427 (PMC5042004; doi:10.18632/oncotarget.8427)
Supplement: Supplementary file 2 [file oncotarget-07-26628-s002.docx]

**Supplementary Table 1 Interaction partners for B-Raf in various cell types.** Listed are proteins identified as specific interaction partners (IPs) in B-Raf immunoprecipitates from the indicated cell types. IPs (with Uniprot numbers in the first column) were deemed specific if their peptides were not found in negative control purifications or, in case of SILAC experiments, were enriched compared to control purifications. IPs were classified as novel, if they were not found in <http://www.ncbi.nlm.nih.gov/gene/673>, or by literature searches. If not stated otherwise, information on protein function was obtained from the “GENE” or “Uniprot” databases. DT40 = IPs from DK37+ cells complemented with B-Raf^WT^, MEF Rec. = IPs from *Braf^-/-^* MEFs cells complemented with B-Raf^WT^ or B-Raf^D594A^, MEF End. = IPs from MEFs expressing endogenous B-Raf; MCF-10A = IPs from MCF-10A cells expressing either B-Raf^WT^ or B-Raf^CAAX^.

| **Interaction**  **partner** | **Function** | **DT40** | **MEF**  **Rec.** | **MEF**  **End.** | **MCF-10A** | **Reference/**  **comments** |
| --- | --- | --- | --- | --- | --- | --- |
| **Signalling** |  |  |  |  |  |  |
| 14-3-3 protein beta/alpha (*YWHAB* gene product) P31946 | Adaptor protein, known to play essential roles in Raf-signalling | X | X | X | X | [1-5] |
| 14-3-3 epsilon (*YWHAE* gene product) P62258 | Adaptor protein, known to play essential roles in Raf-signalling | X | X | X | X | [5, 6] |
| 14-3-3 eta (*YWHAH* gene product) Q04917 | Adaptor protein, known to play essential roles in Raf-signalling | X | X |  | X | [5-7] |
| 14-3-3 gamma (*YWHAG* gene product) P61981 | Adaptor protein, known to play essential roles in Raf-signalling | X | X | X | X | [1] |
| 14-3-3 sigma (*YWHAS* gene product; Stratifin) P31947 | Adaptor protein, known to play essential roles in Raf-signalling;  tumour suppressor gene product |  | X | X | X | [8] |
| 14-3-3 theta/tau (YWHAQ gene product) P27348 | Adaptor protein, known to play essential roles in Raf-signalling | X | X | X | X | [1, 5-7] |
| 14-3-3 protein zeta/delta (*YWHAZ* gene product) P63104 | Adaptor protein, known to play essential roles in Raf-signalling | X | X | X | X | [1-3, 5-7] |
| AKAP8L Q9ULX6 | Signalling scaffold protein | X |  |  |  | This study |
| AKAP8 (related to AKAP8L) O43823 |  |  |  | X |  | This study |
| Annexin-2 (ANXA2) P07355 | Calcium-dependent phospholipid-binding protein involved in signal transduction and membrane trafficking pathways |  | X | X |  | This study |
| Annexin 5 (ANX5) P08758 | Calcium-dependent phospholipid-binding protein involved in signal transduction and membrane trafficking pathways |  | X | X |  | This study |
| A-Raf P10398 | Raf-kinase |  | X |  |  | [6, 9] |
| Casein Kinase II, catalytic α subunit P21868 | Ser/Thr-kinase as part of the KSR scaffolding complex contributing to Raf activation | X |  | X |  | [10] |
| Casein Kinase II, catalytic α’ subunit P19784 | Ser/Thr-kinase as part of the KSR scaffolding complex contributing to Raf activation | X | X | X |  | [10] |
| Caspase recruitment domain-containing protein 11 (CARD11) Q9BXL7 | Signalling scaffold downstream of antigen receptors for NF-κB activation | X |  |  |  | This study |
| Copine III (CPNE3) O75131 | Ca^2+^-dependent phospholipid-binding protein; Interacts with ErbB2 and promotes tumourigenesis |  | X |  | X | This study |
| c-Raf (Raf-1) P04049 | Raf-kinase | X | X |  |  | [11] |
| Cyclin dependent kinase 1 (CDK1) P06493 | Ser/Thr-kinase |  | X | X |  | interacts with *Xenopus* B-Raf in M-Phase and phosphorylates the S151 equivalent S144 *in vitro* [12] |
| Dedicator of cytokinesis protein 1 (**DOCK1)** Q14185 | cytoskeletal rearrangements in phagocytosis of apoptotic cells and cell motility.  Guanine nucleotide exchange factor (GEF) for Rac-GTPases |  | X |  |  | This study |
| Dedicator of cytokinesis protein 7 (**DOCK7)**  Q96N67 | Guanine nucleotide exchange factor (GEF) for Rac-GTPases | X | X | X |  | This study |
| Dual specificity mitogen-activated protein kinase kinase 1 (MEK1) Q02750 | Protein kinase, direct substrate of B-Raf | X | X |  | X | [13] |
| Dual specificity mitogen-activated protein kinase kinase 2 (MEK2) P36507 | Protein kinase, direct substrate of B-Raf | X | X |  | X | [13] |
| FKBP5 Q13451 | Peptidyl prolyl cis trans isomerase/  Immunophilin | X |  |  | X | This study; The FKBP10 isoform has been shown to interact exclusively with Raf-1, but not B-Raf [14] |
| Formin-like protein 3 Q8IVF7 | Diaphanous-related formin, regulated by Rho-GTPases, involved in cell migration |  | X |  |  | This study |
| Guanine nucleotide-binding protein G(i) subunit alpha-2 **(GNAI2)** Q96C71 | Heterotrimeric G-protein signalling | X | X | X | X | This study |
| Guanine nucleotide-binding protein G(i) k? subunit alpha-3 **(GNAI3) P08754** | Heterotrimeric G-protein signalling |  | X |  |  | This study |
| Guanine nucleotide-binding protein G(o) subunit alpha GNAO **P09471** | Heterotrimeric G-protein signalling | X |  |  |  | This study |
| Guanine nucleotide-binding protein G(s) subunit alpha (Gαs) GNAS **Q5JWF2** | Heterotrimeric G-protein signalling | X | X |  | X | This study |
| IQGAP1 **P46940** | Signalling scaffold | X | X | X | X | [15] |
| IQGAP3  **Q86VI3** | Signalling scaffold restricted to proliferating cells [16] |  | X |  | X | This study |
| Kidins220/ARMS **Q9ULH0** | Transmembrane scaffolding protein |  | X |  |  | [17] |
| Melanocortin receptor 1 MCIR **Q01726** | G-protein coupled receptor |  | X |  |  | This study |
| Nucleophosmin (NPM) **P06748** | Phosphoprotein regulating diverse cellular functions such as compartimentalisation of K-Ras [18] | X | X | X |  | This study |
| Prohibitin PHB FLJ78511 **P35232** | Prohibitin has been shown to bind to Raf-1, but not to B-Raf [19] |  | X | X | X | This study |
| Prohibitin-2 (PHB-2) **Q99623** | Highly conserved transmembrane protein. Its close relative, prohibitin has been shown to bind to Raf-1, but not to B-Raf [19] | X |  | X |  | This study |
| Peptidyl-prolyl cis-trans isomerase NIMA-interacting 1 (PIN1) **Q13526** | Isomerase, phosphoprotein | X |  |  |  | [20] |
| Phosphatidylinositol 4-kinase alpha (PIP4KA) **P42356** | Lipid signalling |  | X |  |  | This study |
| Phosphatidylinositol-4-phosphate 5-kinase type 1 alpha (PIP5K1A) **Q99755** | Lipid signalling |  |  |  | X | This study |
| **Phosphatidylinositol-5-phosphate 4-kinase type-2 gamma (PIP4K2C) Q8TBX8** | Lipid signalling |  | X |  | X | This study |
| Phospholipase D2 **O14939** | Lipid signalling |  | X |  | X | This study |
| Protein RRP5 homolog/Pcd11 **Q14690** | NF-κB signalling |  |  |  | X | This study |
| PRMT5 O14744 | Protein methylation, methylates and thereby promotes degradation of Raf-1 | X |  |  |  | [21] |
| Ras-GTPase H-Ras **P01112** | Small G-Protein and Raf activator |  | X |  | X | Well-established |
| Ras-GTPase K-Ras **P01116** | Small G-Protein and Raf activator |  |  |  | X | Well-established |
| Ras-GTPase N-Ras **P01111** | Small G-Protein and Raf activator |  |  |  | X | Well-established |
| Ras GTPase-activating protein-binding protein 2 (G3BP-2) **Q9UN86** | Ill-defined scaffold protein, binds to IκBα and promotes its cytoplasmic retention [22] |  | X | X |  | This study |
| Ras-related protein Rap-1b **P61224** | Has been implicated in B-Raf activation by older studies, however indirect binding, e.g. by IQGAP is more likely |  | X |  | X | [23, 24] |
| Regulatory-associated protein of mTOR (Raptor) **Q8N122** | mTOR signalling |  | X |  |  | This study |
| Serine/threonine-protein phosphatase 1 regulatory subunit 10 (**PPP1R10) Q96QC0** | Protein-Phosphatase |  | X |  |  | This study |
| Serine/threonine-protein phosphatase 2A catalytic subunit beta isoform (PPP2CB) **P62714** | Protein-Phosphatase | X |  |  |  | modulation of B-Raf by this phosphatase complex has suggested based on inhibitor action [20] |
| Serine/threonine-protein phosphatase 2A 65 kDa regulatory subunit A alpha isoform (PPP2R1A) P30153 | Protein-Phosphatase | X | X |  | X | modulation of B-Raf by this phosphatase complex has suggested based on inhibitor action [20] |
| Serine/threonine-phosphatase PGAM5 **Q96HS1** | Phospho-glycerate-mutase family member 5,;interacts with Nrf2, Bcl-XL, regulates ASK1 by dephosphorylation |  | X | X | X | This study |
| Serine/threonine-phosphatase PPI alpha catalytic subunit (PPICA) **P62136** | Protein-Phosphatase |  | X |  | X | This study |
| Sphingosine-1-phosphate-lyase SGPL1 O95470 |  |  | X |  |  | This study |
| Ubiquitin like domain containing CTD phosphatase 1 UBLCP1 **Q8WVY7** | Protein-Phosphatase |  | X |  | X | This study |
| Vang-like protein 1; Kitenein VANGLI **Q8TAA9** | Tetraspannin. Mutations in this gene are associated with neural tube defects; contributes to ERK signalling in colorectal cancer cells |  |  |  | X | This study |
| WD repeat containing protein 6 **Q9NNW5** | Interacts with STK11/LKB and IRS4 |  | X |  | X | This study |
| WD repeat-containing protein 26 WDR6 **Q9H7D7** | Gbeta like WD40 protein, suppresses MAPK signalling |  | X |  |  | This study |
| **Chaperones and co-factors** |  |  |  |  |  |  |
| ATPase family AAA domain-containing protein 3A (**ATD3A) Q9NVI7** |  | X | X |  |  | This study |
| DnaJ homolog subfamily A member 1 DNAJA1 **P31689** | DNAJ/Hsp40 chaperones associate and regulate HSP70 family members |  | X | X | X | This study |
| DnaJ homolog subfamily B member 11 DNAJB11 **Q9UBS4** |  |  | X |  | X | This study |
| DnaJ homolog subfamily B member 12 DNAJB12 **Q9NXW2** |  |  | X |  |  | This study |
| DnaJ homolog subfamily B member 6 DNAJB6 O75190 |  |  |  |  | X | This study |
| DnaJ homolog subfamily C member 13 (RME-8) DNAJC13 **O75165** |  | X | X |  | X | This study |
| DnaJ homolog subfamilyC member 11 DNAJC11 **Q9NVH1** |  |  |  |  | X | This study |
| DnaJ homolog subfamilyC member 15 **Q9Y5T4** |  |  |  |  | X | This study |
| Heat shock 70 kDa protein 4L hs74l (HSPA4L) **O95757** | HSP70 family members are abundant in B-Raf purifications [6] | X |  |  | X | [6] |
| HSP 70 kDa protein 8 (HSC70/ HSPA8) **P11142** |  | X |  | X |  | [6] |
| HSP 70 kDa protein 9 HSPA9 **Q8N1C8** |  |  | X | *X* |  | [6] |
| Hsp 70 similar to cDNA FLJ56386 **B4DI54** |  |  | X |  |  | [6] |
| Heat shock cognate 71 kDa protein HSPA8 P11142 |  | X |  |  |  | [6] |
| Stress-70 protein, mitochondrial HSPA9 P38646 |  | X |  |  |  | [6] |
| HSP 70 kDa protein 5 (HSPA5) P11021 |  | X | X |  | X | [6] |
| HSP 70 kDa protein 1A/B (HSP70-1) P08107 |  |  |  |  | X | [6] |
| HSP 90-alpha (HSP90A) P07900 | Important chaperone for several kinases | X | X | X | X | [25-27] |
| HSP 90-beta (HSP90B1) Q96GW1 | Important chaperone for several kinases | X |  | X | X | [6, 25-27] |
| Hsp90 co-chaperone Cdc37 Q16543 | Co-chaperone of Hsp90 required for kinase recruitment | X |  | X | X | [26, 27] |
| **Ubiquitin and proteasomal degradation-pathway** |  |  |  |  |  |  |
| E3 ubiquitin-protein ligase SIAH2 Seven in absentia homolog 2 O43255 | known to interact with Sprouty-2, a negative feedback regulator of B-Raf [28, 29] | X |  |  |  | This study |
| Proteasome-associated protein ECM29 homolog Q5VYK3 | Serves as an adaptor for coupling 26 S proteasomes to specific cellular compartments. |  | X |  |  | This study |
| S-phase kinase-associated protein 1/ Cyclin- A/CDK2 associated protein HSB11B2 P80365 | Essential component of the SCF (SKP1-CUL1-F-box protein) ubiquitin ligase complex, which mediates the ubiquitination of proteins |  | X |  |  | This study |
| Ubiquitin carboxyl-terminal hydrolase 7 (USP7) Q93009 | Hydrolase cleaving ubiquitin from its substrates (Deubiquitinase) | X | X |  |  | This study |
| **Protein synthesis** |  |  |  |  |  |  |
| Eukaryotic translation initiation factor 3 subunit 5 (EIF5) P55010 | Regulates assembly of the 80 S ribosome |  | X |  |  | This study |
| Eukaryotic translation initiation factor 3 subunit F (**EIF3F)** O00303 | As a multisubunit complex, eIF3 contributes to the formation of the pre-initiation complex and prevents the premature association of the 40S to the 60S ribosomal subunits. Negative regulator of ERK signalling [30] | X |  |  |  | This study; However, the eIF3a subunit was recently described as IP for Raf-1 [30] |
| **DNA repair and replication** |  |  |  |  |  |  |
| RAD50 Q92878 | DNA repair protein |  | X |  | X | This study |
| **Cytoskeleton** |  |  |  |  |  |  |
| Vimentin **P08670** | Intermediate filament, mesenchymal marker |  | X | X | X | This study; however, known interaction partner of Raf-1 [31] |
| Myosin IXb Q13459 |  |  | X |  |  | This study |
| **Apoptosis** |  |  |  |  |  |  |
| GRAM domain-containing protein 4 Q6IC98 | Is recruited to mitochondria during apoptosis and interacts with Bcl2 [32] |  | X |  |  | This study |
| **Intracellular transport incl. membrane dynamics** |  |  |  |  |  |  |
| Myoferlin Q9NZM1 | Involved in plasma membrane repair. Believed to be involved in cell migration. Involved in VEGF signalling |  | X |  | X | This study |
| Coatomer subunit alpha **P53621** | Vesicle transport |  | X |  | X | This study |
| Coatomer subunit epsilon (COPE) **O14579** | Golgi protein transport | X |  |  |  | This study |
| GPI inositol-deacylase PGAP1 **Q75T13** | GPI anchor synthesis in the ER |  | X |  |  | This study |
| MCM3-associated protein 80kda **O60318** | Protein import into nucleus |  | X |  |  |  |
| Ran GTPase-activating protein 1 **Q6GYQ0** | Protein import into nucleus | X | X |  |  | This study |
| Sorting nexin-9 **Q9Y5X1** | Interacts with AP-2 (endocytosis) and cooperates with Cdc42-activated kinase in EGFR downregulation |  | X | X |  | This study |
| **Miscellaneous proteins** |  |  |  |  |  |  |
| Corticosteroid 11-beta-dehydrogenase isozyme 2 HSD11B2 hydroxysteroid (11-beta) dehydrogenase 2 **P80365** | Microsomal oxidoreductase; |  | X |  | X | This study |

**References**

1. Jin J, Smith FD, Stark C, Wells CD, Fawcett JP, Kulkarni S, Metalnikov P, O'Donnell P, Taylor P, Taylor L, Zougman A, Woodgett JR, Langeberg LK, Scott JD and Pawson T. Proteomic, functional, and domain-based analysis of in vivo 14-3-3 binding proteins involved in cytoskeletal regulation and cellular organization. Current biology : CB. 2004; 14(16):1436-1450.

2. Ewing RM, Chu P, Elisma F, Li H, Taylor P, Climie S, McBroom-Cerajewski L, Robinson MD, O'Connor L, Li M, Taylor R, Dharsee M, Ho Y, Heilbut A, Moore L, Zhang S, et al. Large-scale mapping of human protein-protein interactions by mass spectrometry. Molecular systems biology. 2007; 3:89.

3. Qiu W, Zhuang S, von Lintig FC, Boss GR and Pilz RB. Cell type-specific regulation of B-Raf kinase by cAMP and 14-3-3 proteins. The Journal of biological chemistry. 2000; 275(41):31921-31929.

4. Yuryev A and Wennogle LP. Novel raf kinase protein-protein interactions found by an exhaustive yeast two-hybrid analysis. Genomics. 2003; 81(2):112-125.

5. Kalmes A, Hagemann C, Weber CK, Wixler L, Schuster T and Rapp UR. Interaction between the protein kinase B-Raf and the alpha-subunit of the 11S proteasome regulator. Cancer research. 1998; 58(14):2986-2990.

6. Gloeckner CJ, Boldt K, Schumacher A, Roepman R and Ueffing M. A novel tandem affinity purification strategy for the efficient isolation and characterisation of native protein complexes. Proteomics. 2007; 7(23):4228-4234.

7. Papin C, Denouel A, Calothy G and Eychene A. Identification of signalling proteins interacting with B-Raf in the yeast two-hybrid system. Oncogene. 1996; 12(10):2213-2221.

8. Benzinger A, Muster N, Koch HB, Yates JR, 3rd and Hermeking H. Targeted proteomic analysis of 14-3-3 sigma, a p53 effector commonly silenced in cancer. Molecular & cellular proteomics : MCP. 2005; 4(6):785-795.

9. Hatzivassiliou G, Song K, Yen I, Brandhuber BJ, Anderson DJ, Alvarado R, Ludlam MJ, Stokoe D, Gloor SL, Vigers G, Morales T, Aliagas I, Liu B, Sideris S, Hoeflich KP, Jaiswal BS, et al. RAF inhibitors prime wild-type RAF to activate the MAPK pathway and enhance growth. Nature. 2010; 464(7287):431-435.

10. Ritt DA, Zhou M, Conrads TP, Veenstra TD, Copeland TD and Morrison DK. CK2 Is a Component of the KSR1 Scaffold Complex that Contributes to Raf Kinase Activation. Current biology : CB. 2007; 17(2):179-184.

11. Weber CK, Slupsky JR, Kalmes HA and Rapp UR. Active Ras induces heterodimerization of cRaf and BRaf. Cancer research. 2001; 61(9):3595-3598.

12. Borysov SI and Guadagno TM. A novel role for Cdk1/cyclin B in regulating B-raf activation at mitosis. Molecular biology of the cell. 2008; 19(7):2907-2915.

13. Catling AD, Reuter CW, Cox ME, Parsons SJ and Weber MJ. Partial purification of a mitogen-activated protein kinase kinase activator from bovine brain. Identification as B-Raf or a B-Raf-associated activity. The Journal of biological chemistry. 1994; 269(47):30014-30021.

14. Coss MC, Stephens RM, Morrison DK, Winterstein D, Smith LM and Simek SL. The immunophilin FKBP65 forms an association with the serine/threonine kinase c-Raf-1. Cell growth & differentiation : the molecular biology journal of the American Association for Cancer Research. 1998; 9(1):41-48.

15. Ren JG, Li Z and Sacks DB. IQGAP1 modulates activation of B-Raf. Proceedings of the National Academy of Sciences of the United States of America. 2007; 104(25):10465-10469.

16. Nojima H, Adachi M, Matsui T, Okawa K, Tsukita S and Tsukita S. IQGAP3 regulates cell proliferation through the Ras/ERK signalling cascade. Nature cell biology. 2008; 10(8):971-978.

17. Deswal S, Meyer A, Fiala GJ, Eisenhardt AE, Schmitt LC, Salek M, Brummer T, Acuto O and Schamel WW. Kidins220/ARMS associates with B-Raf and the TCR, promoting sustained Erk signaling in T cells. J Immunol. 2013; 190(5):1927-1935.

18. Inder KL, Hill MM and Hancock JF. Nucleophosmin and nucleolin regulate K-Ras signaling. Communicative & integrative biology. 2010; 3(2):188-190.

19. Rajalingam K, Wunder C, Brinkmann V, Churin Y, Hekman M, Sievers C, Rapp UR and Rudel T. Prohibitin is required for Ras-induced Raf-MEK-ERK activation and epithelial cell migration. Nature cell biology. 2005; 7(8):837-843.

20. Ritt DA, Monson DM, Specht SI and Morrison DK. Impact of feedback phosphorylation and Raf heterodimerization on normal and mutant B-Raf signaling. Molecular and cellular biology. 2010; 30(3):806-819.

21. Andreu-Perez P, Esteve-Puig R, de Torre-Minguela C, Lopez-Fauqued M, Bech-Serra JJ, Tenbaum S, Garcia-Trevijano ER, Canals F, Merlino G, Avila MA and Recio JA. Protein arginine methyltransferase 5 regulates ERK1/2 signal transduction amplitude and cell fate through CRAF. Science signaling. 2011; 4(190):ra58.

22. Prigent M, Barlat I, Langen H and Dargemont C. IkappaBalpha and IkappaBalpha /NF-kappa B complexes are retained in the cytoplasm through interaction with a novel partner, RasGAP SH3-binding protein 2. The Journal of biological chemistry. 2000; 275(46):36441-36449.

23. Vossler MR, Yao H, York RD, Pan MG, Rim CS and Stork PJ. cAMP activates MAP kinase and Elk-1 through a B-Raf- and Rap1-dependent pathway. Cell. 1997; 89(1):73-82.

24. Okada T, Hu CD, Jin TG, Kariya K, Yamawaki-Kataoka Y and Kataoka T. The strength of interaction at the Raf cysteine-rich domain is a critical determinant of response of Raf to Ras family small GTPases. Molecular and cellular biology. 1999; 19(9):6057-6064.

25. Jaiswal RK, Weissinger E, Kolch W and Landreth GE. Nerve growth factor-mediated activation of the mitogen-activated protein (MAP) kinase cascade involves a signaling complex containing B-Raf and HSP90. The Journal of biological chemistry. 1996; 271(39):23626-23629.

26. da Rocha Dias S, Friedlos F, Light Y, Springer C, Workman P and Marais R. Activated B-RAF is an Hsp90 client protein that is targeted by the anticancer drug 17-allylamino-17-demethoxygeldanamycin. Cancer research. 2005; 65(23):10686-10691.

27. Grbovic OM, Basso AD, Sawai A, Ye Q, Friedlander P, Solit D and Rosen N. V600E B-Raf requires the Hsp90 chaperone for stability and is degraded in response to Hsp90 inhibitors. Proceedings of the National Academy of Sciences of the United States of America. 2006; 103(1):57-62.

28. Tsavachidou D, Coleman ML, Athanasiadis G, Li S, Licht JD, Olson MF and Weber BL. SPRY2 is an inhibitor of the ras/extracellular signal-regulated kinase pathway in melanocytes and melanoma cells with wild-type BRAF but not with the V599E mutant. Cancer research. 2004; 64(16):5556-5559.

29. Brady SC, Coleman ML, Munro J, Feller SM, Morrice NA and Olson MF. Sprouty2 association with B-Raf is regulated by phosphorylation and kinase conformation. Cancer research. 2009; 69(17):6773-6781.

30. Xu TR, Lu RF, Romano D, Pitt A, Houslay MD, Milligan G and Kolch W. Eukaryotic translation initiation factor 3, subunit a, regulates the extracellular signal-regulated kinase pathway. Molecular and cellular biology. 2012; 32(1):88-95.

31. Janosch P, Kieser A, Eulitz M, Lovric J, Sauer G, Reichert M, Gounari F, Buscher D, Baccarini M, Mischak H and Kolch W. The Raf-1 kinase associates with vimentin kinases and regulates the structure of vimentin filaments. FASEB journal : official publication of the Federation of American Societies for Experimental Biology. 2000; 14(13):2008-2021.

32. John K, Alla V, Meier C and Putzer BM. GRAMD4 mimics p53 and mediates the apoptotic function of p73 at mitochondria. Cell death and differentiation. 2011; 18(5):874-886.
