# Supplementary material for: Phospho-proteomic analyses of B-Raf protein complexes reveal new regulatory principles
Source: Oncotarget. 2016 Mar 28;7(18):26628–52. doi: 10.18632/oncotarget.8427 (PMC5042004; doi:10.18632/oncotarget.8427)
Supplement: Supplementary file 7 [file oncotarget-07-26628-s007.pdf]

Supplementary Table 6. List of B-Raf phosphorylation and ubiquitination sites identified by LC-MS/MS in the course of this project. HA-tagged B-Raf was purified from either B-Raf/Raf-1 double deficient chicken DT40 B cells [1], from B-Raf deficient murine embryonic fibroblasts (MEFs; [2]) or from human MCF-10A cells (this study). The numbering for these sites is based on the human B-Raf amino acid sequence. For simplicity, sites identified in B-Raf from unstimulated or stimulated samples or from B-Raf mutants such as B-Raf^{D594A} or B-Raf^{CAAX} were all included. However, sites only identified in these mutants but not in B-Raf^{wt} are marked as such in the comments. A given reference in combination with no entry in the "comments/regulation" column means that the function of this site remains unknown and has been only listed as a MS-identified phosphorylation site, mostly in Supplementary data of the indicated publication. If not supplied with references, kinases involved in the phosphorylation events were predicted using Scansite [3], PhosphoNET (only the top three hits displayed with closely related family members listed as one hit, e.g. ERK1/2; [4] or the KinomeXplorer platform [5] as indicated. Phosphosites (in bold), or adjacent residues being part of a consensus motif for this phosphorylation site, found to be mutated in cancer (COSMIC database) or in RASopathies are listed as well. All phosphopeptides were identified minimally once with MASCOT scores of 23. MASCOT scores for respective peptides are indicated in brackets in blue below respective sites. Our list was also compared with that of the Phosphosite Plus database (5/1/2016).

Site	DT40	MEF	MCF-10A	Comments on regulation, function and mutational status	Localisation in B-Raf	Predicted or implicated Kinase(s)	Mutated in cancer and/or RASopathy	Listed at Phos-phosite Plus	Ref.
S9 (44)	1P				BSR			No	**This study**
S76 (79)			1P		BSR			No	**This study**
K88		X			BSR	N/A		No	**This study**
S151 (77)	1P	1P	1P	Negative regulation. Phosphorylation reduced by Roscovitin, but not U0126 (see text for details)	Junction between BSR and RBD	NetPhorest: ERK1/2<br><br>PhosphoNET:<br>ERK1, JNK1/3, CDK2<br><br>Scansite: CDK5, Cdc2	P152S mutation destroys consensus phosphorylation motif for MAPK and CDKs	Yes	[6-8]
S314 (39)		1P	4P[310-319]					No	**This study**
S316 (25)		1P	4P[310-319]			PhosphoNET:<br>GSK3a/b, p38a/d, JNK1/3	**S316P** (urinary tract)	No	**This study**
S317 (26)		1P	4P[310-319]			PhosphoNET:<br>JNK1-3, p38a, ERK		No	**This study**
S319 (23)			4P[310-319]			NetPhorest:<br>PKCtheta, PKCeta, PKCgamma, CK1delta, PKCalpha, PKCdelta<br><br>PhosphoNET:<br>GSK3a/b, CDK7, ERK1	A320T, A322V, I326V/T	Yes	[9]

S333 (130)	1P[S 333-S337]	1P [S333, S335]				NetPhorest: ERK1/2<br><br>PhosphoNET: JNK1-3, ERK1/2, ERK5<br><br>Scansite: GSK3		Yes	[9]
S335 (181)	1P[S 333-S337]	1P [S333, S335]	1P			NetPhorest: ERK1<br><br>PhosphoNET: CDK2, ERK5, CDK1		Yes	[10]
S337 (45)	1P[S 333-S337]					PhosphoNET: ERK1, JNK1-3, p38d	**S337L** (skin)	No	**This study**
S339 (104)			1P			PhosphoNET: ERK1/2, GSK3a, JNK1/3		No	**This study**
S363 (111)		1P	2P[S 363-S365]		CR2	NetPhorest: PAK2/4<br><br>PhosphoNET: CHK1, ANPa, MAPKAPK2/3		Yes	[11]
S364 (44)			2P[S 363-S365]		CR2	NetPhorest: PAK4<br><br>PhosphoNET: PKG2, PKCd, PKCt	**S364L** (urinary)	Yes	[11]
S365 (179)	1P	1P	2P[S 363-S365]	Negative regulation, 14-3-3 binding site [12]	CR2; 14-3-3 binding motif	NetPhorest: PAK2, PKBa (AKT)<br><br>PhosphoNET: PIM1-3, Raf1, PRKX<br><br>Scansite: AKT (experimentally confirmed)	**S365L** (skin)<br>P367R (lung), P367S (skin)	Yes	[13]
T373 (91)			1P	Autophosphorylation site [14]	CR2	NetPhorest: TTK, PKD1<br><br>PhosphoNET: PRP4, HIPK3, MEK7		Yes	[14]

S394 (59)			1P[S394-S396]		HR	PhosphoNET:<br>PIM1-3, mTOR/FRAP, NEK10		Yes	[15]
T396 (54)			1P[S394-S396]		HR	PhosphoNET:<br>mTOR/FRAP, PEK, Sgk288		Yes	[15]
S399 (87)		1P	1P		HR	NetPhorest:<br>ERK1/2<br><br>PhosphoNET:<br>ERK1/2, mTOR/FRAP, p38a/d		Yes	[16]
T401 (144)	1P	1P	1P	Phosphorylation of this site is not very inducible according to our WB and MS experiments.	HR	NetPhorest:<br>ERK1<br><br>PhosphoNET:<br>JNK1/3, mTOR/FRAP, ERK5	**T401A** (prostate)<br>**T401I** (cervix)	Yes	[7, 17]
S419 (56)	1P	1P	1P	Alanine substitution of this site by itself has no effect on MEK phosphorylation potential of B-Raf. Residue is located adjacent to K418, an acetylation site [18]	HR	NetPhorest:<br>ERK1/2<br><br>PhosphoNET:<br>ERK2, CDKL2, JNK1-3	**S419Y** (endometrium);<br><br>P420S (myelodysplastic syndrome); affects consensus for Pro-directed kinase	Yes	[6, 7]
S428 (37)		1P[428-432]	2P[428-432]	Negative regulation mediated by AGC kinases like PKA and AKT/PKB.	HR	PhosphoNET:<br>PKACa/b, AKT1-3, DAPK2<br><br>Scansite: AKT (experimentally confirmed),	**S428T** (endometrium)	Yes	**[13]**
S429 (53)		1P[428-432]	2P[428-432]		HR	NetPhorest:<br>PAK2<br><br>PhosphoNET:<br>PIM1-3, PRKX, RSK1<br><br>Scansite: AKT PKA, CLK2		Yes	[13, 19]
S430 (38)		1P[428-432]	2P[428-432]		HR	PhosphoNET:<br>P38d, VRK1/3, ANPa/b		Yes	**This study**
S431 (29)		1P[428-432]	2P[428-432]		HR	PhosphoNET:<br>PIM1, PRKX, PKG2<br><br>Scansite PKCα,β,γ		Yes	**This study**
S432 (26)		1P[428-	2P[428-		HR	PhosphoNET:<br>Raf (all 3 isoforms), ANPa/b, PKCe		Yes	**This study**

		432]	432]						
S446 (145)	1P	1P	1P	Well-documented positive role (N-region charge). More or less constitutively phosphorylated.	N-region	NetPhorest:<br>PAK2<br><br>PhosphoNET:<br>PIM1-3, MSK1, CaMK4<br><br>Scansite: PKA, AMPK<br><br>Other:<br>MEK-dependent phosphorylation [20]		Yes	[12, 21, 22]
S447 (85)	1P	1P		Well-documented positive role (N-region charge). More or less constitutively phosphorylated.	N-region	NetPhorest:<br>PKBalpha, RSK1<br><br>PhosphoNET:<br>PIM1-3, MAPKAPK2/3, MSK1		Yes	[12, 21, 22]
S465 (74)	1P	1P	1P	Negative regulation	P-loop;<br>Kinase domain	PhosphoNET:<br>ANPa, RSK2/3, PKACa/b<br><br>Other:<br>Autophosphory-lation [23]	**S465F** (colorectal, lung)<br>S465 belongs to P-loop, the second hotspot for *BRAF* mutations. Consequently several mutations have been mapped to its vicinity in cancer and CFC syndrome (S467A), see COSMIC for details.	Yes	[11, 24-26]
T470 (41)			1P			Scansite:<br>CK1		No	**This study**
S605 (152)	1P	1P	1P	Highly conserved phosphorylation site and mutated to various residues in tumours (COSMIC database). However, our preliminary data suggest that alanine or glutamate substitutions have no discernible effect on MEK phosphorylation of B-Raf or B-Raf^{V600E}.<br>Equivalent to the controversial PKC phosphorylation site S497 in Raf-1. See [27] for discussion.	Activation loop;<br>Kinase domain	NetPhorest:<br>PAK2<br><br>PhosphoNET:<br>PKCa/b, PKG2, p70S6Kb	**S605N** (skin)<br>**S605F** (skin), **S605G** (skin, large intestine), **S605R** (skin),many other mutations in activation loop	Yes	[28]
S614 (38)		1P[S 614, S616]	1P	Serendipous alanine substitution of this residue had no effect on basal or oncogenic Ras-induced B-Raf activity [29]	Activation loop;<br>kinase domain	PhosphoNET:<br>P38d/a, JNK1-3, VRK3<br><br>Scansite: CamK2	**S614P** (skin, large intestine)	Yes	[24]

S616 (34)		1P[S 614, S616]			Activation loop; kinase domain		**S616P** (skin), **S616F** (ovary, pancreas, skin); melanoma [30]	No	**This study**
S675 (42)		1P	1P	Highly conserved site of unknown function. Adjacent to PRMT5 methylation site R671 [31]. Cross talk with arginine methylation ?	Kinase domain	PhosphoNET:<br>JNK1/3, p38d, ERK5	R671Q<br><br>Mutation eliminates methylation site [31]	No	**This study**
S729 (76)	1P	1P	1P	Well established role in 14-3-3 binding and coupling to downstream effectors	14-3-3 binding motif	NetPhorest:<br>PAK2/4, PKBalpha, PKCthta, eta, gamma, RSK1<br><br>PhosphoNET:<br>Raf (all 3 isforms), ANPa, MAPKAPK2/3<br><br>Scansite: PKCδ, AKT<br>Other:<br>AMPK experimentally confirmed [32]	P731S (skin)<br>mutation could prevent phosphorylation and/or 14-3-3 binding	Yes	[12, 33, 34]
S750 (102)	1P	1P	1P	ERK-mediated feedback phosphorylation site controlling Raf dimerisation.		NetPhorest:<br>ERK1/2<br><br>PhosphoNET:<br>JNK1-3, ERK1/2, p38a,d,g<br><br>Scansite:PKCα,β,γ, ERK<br>Other: ERK 8experimentally confirmed [35] [7]		Yes	[7, 35, 36]
T753 (102)	1P	1P	1P	ERK-mediated feedback phosphorylation site controlling Raf dimerisation.		NetPhorest:<br>ERK1<br><br>PhosphoNET:<br>mTOR/FRAPm JNK-13, CDK2/3, ERK2 (as next)<br><br>Scansite CDK5, Cdc2:<br><br>Other: ERK experimentally confirmed [35] [7]		Yes	[7, 35, 36]

References:

1. Brummer T, Shaw PE, Reth M and Misawa Y. Inducible gene deletion reveals different roles for B-Raf and Raf-1 in B-cell antigen receptor signalling. The EMBO journal. 2002; 21(21):5611-5622.
2. Röring M, Herr R, Fiala GJ, Heilmann K, Braun S, Eisenhardt AE, Halbach S, Capper D, von Deimling A, Schamel WW, Saunders DN and Brummer T. Distinct requirement for an intact dimer interface in wild-type, V600E and kinase-dead B-Raf signalling. The EMBO journal. 2012; 31(11):2629-2647.
3. Obenauer JC, Cantley LC and Yaffe MB. Scansite 2.0: Proteome-wide prediction of cell signaling interactions using short sequence motifs. Nucleic acids research. 2003; 31(13):3635-3641.
4. Safaei J, Manuch J, Gupta A, Stacho L and Pelech S. Prediction of 492 human protein kinase substrate specificities. Proteome science. 2011; 9 Suppl 1:S6.
5. Horn H, Schoof EM, Kim J, Robin X, Miller ML, Diella F, Palma A, Cesareni G, Jensen LJ and Linding R. KinomeXplorer: an integrated platform for kinome biology studies. Nature methods. 2014; 11(6):603-604.
6. Wissing J, Jansch L, Nimtz M, Dieterich G, Hornberger R, Keri G, Wehland J and Daub H. Proteomics analysis of protein kinases by target class-selective prefractionation and tandem mass spectrometry. Molecular & cellular proteomics : MCP. 2007; 6(3):537-547.
7. Ritt DA, Monson DM, Specht SI and Morrison DK. Impact of feedback phosphorylation and Raf heterodimerization on normal and mutant B-Raf signaling. Molecular and cellular biology. 2010; 30(3):806-819.
8. Marquette A, Andre J, Bagot M, Bensussan A and Dumaz N. ERK and PDE4 cooperate to induce RAF isoform switching in melanoma. Nature structural & molecular biology. 2011; 18(5):584-591.
9. Dephoure N, Zhou C, Villen J, Beausoleil SA, Bakalarski CE, Elledge SJ and Gygi SP. A quantitative atlas of mitotic phosphorylation. Proceedings of the National Academy of Sciences of the United States of America. 2008; 105(31):10762-10767.
10. Huttlin EL, Jedrychowski MP, Elias JE, Goswami T, Rad R, Beausoleil SA, Villen J, Haas W, Sowa ME and Gygi SP. A tissue-specific atlas of mouse protein phosphorylation and expression. Cell. 2010; 143(7):1174-1189.
11. Moritz A, Li Y, Guo A, Villen J, Wang Y, MacNeill J, Kornhauser J, Sprott K, Zhou J, Possemato A, Ren JM, Hornbeck P, Cantley LC, Gygi SP, Rush J and Comb MJ. Akt-RSK-S6 kinase signaling networks activated by oncogenic receptor tyrosine kinases. Science signaling. 2010; 3(136):ra64.
12. Brummer T, Martin P, Herzog S, Misawa Y, Daly RJ and Reth M. Functional analysis of the regulatory requirements of B-Raf and the B-Raf(V600E) oncoprotein. Oncogene. 2006; 25(47):6262-6276.
13. Guan KL, Figueroa C, Brtva TR, Zhu T, Taylor J, Barber TD and Vojtek AB. Negative regulation of the serine/threonine kinase B-Raf by Akt. The Journal of biological chemistry. 2000; 275(35):27354-27359.
14. Stephens RM, Sithanandam G, Copeland TD, Kaplan DR, Rapp UR and Morrison DK. 95-kilodalton B-Raf serine/threonine kinase: identification of the protein and its major autophosphorylation site. Molecular and cellular biology. 1992; 12(9):3733-3742.
15. Zhou H, Di Palma S, Preisinger C, Peng M, Polat AN, Heck AJ and Mohammed S. Toward a comprehensive characterization of a human cancer cell phosphoproteome. Journal of proteome research. 2013; 12(1):260-271.

16. Bian Y, Song C, Cheng K, Dong M, Wang F, Huang J, Sun D, Wang L, Ye M and Zou H. An enzyme assisted RP-RPLC approach for in-depth analysis of human liver phosphoproteome. Journal of proteomics. 2014; 96:253-262.
17. Beausoleil SA, Jedrychowski M, Schwartz D, Elias JE, Villen J, Li J, Cohn MA, Cantley LC and Gygi SP. Large-scale characterization of HeLa cell nuclear phosphoproteins. Proceedings of the National Academy of Sciences of the United States of America. 2004; 101(33):12130-12135.
18. Choudhary C, Kumar C, Gnad F, Nielsen ML, Rehman M, Walther TC, Olsen JV and Mann M. Lysine acetylation targets protein complexes and co-regulates major cellular functions. Science. 2009; 325(5942):834-840.
19. Konig S, Guibert B, Morice C, Vernier P and Barnier JV. Phosphorylation by PKA of a site unique to B-Raf kinase. Comptes rendus de l'Academie des sciences Serie III, Sciences de la vie. 2001; 324(8):673-681.
20. Hu J, Stites EC, Yu H, Germino EA, Meharena HS, Stork PJ, Kornev AP, Taylor SS and Shaw AS. Allosteric Activation of Functionally Asymmetric RAF Kinase Dimers. Cell. 2013; 154(5):1036-1046.
21. Mason CS, Springer CJ, Cooper RG, Superti-Furga G, Marshall CJ and Marais R. Serine and tyrosine phosphorylations cooperate in Raf-1, but not B-Raf activation. The EMBO journal. 1999; 18(8):2137-2148.
22. Tran NH, Wu X and Frost JA. B-Raf and Raf-1 are regulated by distinct autoregulatory mechanisms. The Journal of biological chemistry. 2005; 280(16):16244-16253.
23. Holderfield M, Merritt H, Chan J, Wallroth M, Tandeske L, Zhai H, Tellew J, Hardy S, Hekmat-Nejad M, Stuart DD, McCormick F and Nagel TE. RAF inhibitors activate the MAPK pathway by relieving inhibitory autophosphorylation. Cancer cell. 2013; 23(5):594-602.
24. Mayya V, Lundgren DH, Hwang SI, Rezaul K, Wu L, Eng JK, Rodionov V and Han DK. Quantitative phosphoproteomic analysis of T cell receptor signaling reveals system-wide modulation of protein-protein interactions. Science signaling. 2009; 2(84):ra46.
25. Bentivegna S, Zheng J, Namsaraev E, Carlton VE, Pavlicek A, Moorhead M, Siddiqui F, Wang Z, Lee L, Ireland JS, Suyenaga K, Willis TD, Faham M and Seymour AB. Rapid identification of somatic mutations in colorectal and breast cancer tissues using mismatch repair detection (MRD). Human mutation. 2008; 29(3):441-450.
26. Tissot C, Couraud S, Tanguy R, Bringuier PP, Girard N and Souquet PJ. Clinical characteristics and outcome of patients with lung cancer harboring BRAF mutations. Lung Cancer. 2016; 91:23-28.
27. Barnard D, Diaz B, Clawson D and Marshall M. Oncogenes, growth factors and phorbol esters regulate Raf-1 through common mechanisms. Oncogene. 1998; 17(12):1539-1547.
28. Zarling AL, Ficarro SB, White FM, Shabanowitz J, Hunt DF and Engelhard VH. Phosphorylated peptides are naturally processed and presented by major histocompatibility complex class I molecules in vivo. The Journal of experimental medicine. 2000; 192(12):1755-1762.
29. Zhang BH and Guan KL. Activation of B-Raf kinase requires phosphorylation of the conserved residues Thr598 and Ser601. The EMBO journal. 2000; 19(20):5429-5439.
30. Fisher KE, Zhang L, Wang J, Smith GH, Newman S, Schneider TM, Pillai RN, Kudchadkar RR, Owonikoko TK, Ramalingam SS, Lawson DH, Delman KA, El-Rayes BF, Wilson MM, Sullivan HC, Morrison AS, et al. Clinical Validation and Implementation of a Targeted Next-Generation Sequencing Assay to Detect Somatic Variants in Non-Small Cell Lung, Melanoma, and Gastrointestinal Malignancies. The Journal of molecular diagnostics : JMD. 2016.

31. Andreu-Perez P, Esteve-Puig R, de Torre-Minguela C, Lopez-Fauqued M, Bech-Serra JJ, Tenbaum S, Garcia-Trevijano ER, Canals F, Merlino G, Avila MA and Recio JA. Protein arginine methyltransferase 5 regulates ERK1/2 signal transduction amplitude and cell fate through CRAF. Science signaling. 2011; 4(190):ra58.
32. Shen CH, Yuan P, Perez-Lorenzo R, Zhang Y, Lee SX, Ou Y, Asara JM, Cantley LC and Zheng B. Phosphorylation of BRAF by AMPK impairs BRAF-KSR1 association and cell proliferation. Molecular cell. 2013; 52(2):161-172.
33. MacNicol MC, Muslin AJ and MacNicol AM. Disruption of the 14-3-3 binding site within the B-Raf kinase domain uncouples catalytic activity from PC12 cell differentiation. The Journal of biological chemistry. 2000; 275(6):3803-3809.
34. Fischer A, Baljuls A, Reinders J, Nekhoroshkova E, Sibilski C, Metz R, Albert S, Rajalingam K, Hekman M and Rapp UR. Regulation of RAF activity by 14-3-3 proteins: RAF kinases associate functionally with both homo- and heterodimeric forms of 14-3-3 proteins. The Journal of biological chemistry. 2009; 284(5):3183-3194.
35. Brummer T, Naegele H, Reth M and Misawa Y. Identification of novel ERK-mediated feedback phosphorylation sites at the C-terminus of B-Raf. Oncogene. 2003; 22(55):8823-8834.
36. Rushworth LK, Hindley AD, O'Neill E and Kolch W. Regulation and role of Raf-1/B-Raf heterodimerization. Molecular and cellular biology. 2006; 26(6):2262-2272.
