# Supplementary material for: Phospho-proteomic analyses of B-Raf protein complexes reveal new regulatory principles
Source: Oncotarget. 2016 Mar 28;7(18):26628–52. doi: 10.18632/oncotarget.8427 (PMC5042004; doi:10.18632/oncotarget.8427)
Supplement: Supplementary file 8 [file oncotarget-07-26628-s008.zip › Supplementary File 7.html]

PepView


| Peptide View (Result) | | | | |
| --- | --- | --- | --- | --- |
| MS File Choice | | Protein Choice | Filter Options | Display Options |
| --- | --- | --- | --- | --- |
| Available MS Files:   tempfile (F006954) BSA Papain Verdau mit Cystein .temp (F011905) tempfile (F003591) tempfile (F003720) tempfile (F003586) tempfile (F003491) Labeled H3 (Trypsine).temp (F008966) tempfile (F003703) tempfile (F003438) tempfile (F003301) HAT3 KO\_Middle\_Thermolysine.temp (F011289) tempfile (F003634) tempfile (F008182) tempfile (F005202) tempfile (F006796) tempfile (F003121) tempfile (F006475) tempfile (F003710) tempfile (F003640) tempfile (F006689) tempfile (F002291) Heavy Acetylated TY1-H2A.temp (F016322) tempfile (F003574) tempfile (F005140) tempfile (F002239) tempfile (F006758) tempfile (F002596)  (F020404) tempfile (F003996) tempfile (F003212) tempfile (F002247) tempfile (F007734) tempfile (F001697) HAT2 KD\_48hr.temp (F011227) tempfile (F006785) tempfile (F003160) tempfile (F003217) tempfile (F016299) No0181\_Trypsin\_ETD.temp (F009585) tempfile (F003993) tempfile (F006961) tempfile (F001728) tempfile (F005307) HAT3 KO\_Lower\_Elastase.temp (F011232) tempfile (F006777) tempfile (F005606) tempfile (F006245) tempfile (F002254) tempfile (F006807) tempfile (F005576) tempfile (F005210) tempfile (F003425) tempfile (F006801) tempfile (F006778) tempfile (F002627) tempfile (F002281)  (F020402) tempfile (F002600) tempfile (F005580) tempfile (F003428) Light Acetylated H3\_Quan.temp (F010891) tempfile (F003709) tempfile (F007298) tempfile (F008561) tempfile (F001560) tempfile (F005669) tempfile (F001620) tempfile (F002236) tempfile (F002632) tempfile (F003628) HAT2 KD\_0hr.temp (F011178) tempfile (F003744)  (F016879) tempfile (F006771) tempfile (F003303) tempfile (F002305) HAT2 KD\_24hr.temp (F011183) tempfile (F006573) tempfile (F005613) tempfile (F008602) tempfile (F003431) tempfile (F002556) tempfile (F007878) tempfile (F001901) tempfile (F003584) tempfile (F001564) tempfile (F005608) tempfile (F006953) HAT2 KD\_24hr.temp (F011182) BSA Papain Verdau ohne Cystein .temp (F011904) tempfile (F003978) tempfile (F003981) tempfile (F003617) tempfile (F002288)  (F016877) tempfile (F003581) tempfile (F005157) tempfile (F002300) tempfile (F005582) tempfile (F001908)  (F020380) tempfile (F007877) tempfile (F009649) tempfile (F006830) tempfile (F003422) HAT2 KD\_48hr.temp (F011219) HAT2 KD\_24h.temp (F011294) tempfile (F003635) tempfile (F003702) tempfile (F006792) tempfile (F003493) HK-2 Histones NOB.temp (F009415) tempfile (F003714) tempfile (F003302) tempfile (F005204) tempfile (F003309) tempfile (F006233) tempfile (F006815) tempfile (F007301) tempfile (F003708) Labeled H3 (Trypsine).temp (F009311) tempfile (F007299) tempfile (F002253) tempfile (F003161) tempfile (F001696) tempfile (F009648) tempfile (F005597) tempfile (F016248) tempfile (F006763) tempfile (F001561) tempfile (F001893) tempfile (F002282) tempfile (F010795) tempfile (F005581) tempfile (F003649) tempfile (F003740) tempfile (F010803) tempfile (F006802) tempfile (F002248) tempfile (F002610) HAT2 KD\_0hr.temp (F011179) tempfile (F006770) tempfile (F002241) tempfile (F002628) tempfile (F006234) Light Acetylated H3\_Quan.temp (F011075) tempfile (F008566) tempfile (F005607) tempfile (F002289) tempfile (F002306) tempfile (F006795) tempfile (F006808) tempfile (F005575) tempfile (F003743) tempfile (F003053) tempfile (F016290) tempfile (F002633) tempfile (F007916) tempfile (F005571) tempfile (F006779) tempfile (F006784) tempfile (F003690) tempfile (F006963) tempfile (F003300) tempfile (F005073) tempfile (F002563) tempfile (F003218) tempfile (F003984) tempfile (F003976) tempfile (F008603) tempfile (F003578) tempfile (F003643) tempfile (F003918) No0181\_Trypsin\_HCD.temp (F009586) tempfile (F003742) tempfile (F005212) tempfile (F006831) tempfile (F003741) tempfile (F003696) tempfile (F009642) tempfile (F002290) tempfile (F001622) tempfile (F005593) tempfile (F010786) tempfile (F002287) Light Acetylated H3\_Thermolysin.temp (F010890) tempfile (F010783) tempfile (F002235) tempfile (F001619) Labeled H3 (Trypsine).temp (F009155) tempfile (F002303) tempfile (F002624) tempfile (F003637) tempfile (F003159) tempfile (F003636) tempfile (F002565) tempfile (F003980) tempfile (F002566) tempfile (F008606) tempfile (F005531) HK-2 Histones Ctrl.temp (F009414) tempfile (F006794) tempfile (F001884) tempfile (F002551) tempfile (F002570) tempfile (F001699) tempfile (F001725) tempfile (F006956) tempfile (F006765) tempfile (F003423) tempfile (F003116) tempfile (F009730) tempfile (F016283)  (F020406) tempfile (F006797) tempfile (F005667) tempfile (F008563) tempfile (F016249) tempfile (F002602) tempfile (F002598) tempfile (F003701) tempfile (F003097) tempfile (F003693) tempfile (F002634) tempfile (F006783) tempfile (F003420) tempfile (F006235) tempfile (F006832) tempfile (F003712) tempfile (F005596) tempfile (F005297) tempfile (F012138) Light Acetylated H3\_Quan.temp (F011074) tempfile (F001551) tempfile (F006962) tempfile (F002564) tempfile (F001695) tempfile (F005572) tempfile (F003589) tempfile (F006762) HAT2 KD\_48hr.temp (F011185) tempfile (F002286) tempfile (F003579) tempfile (F003162) tempfile (F009643) tempfile (F006809) tempfile (F003294) tempfile (F008604) tempfile (F006782) tempfile (F006476) tempfile (F002299) tempfile (F003176) tempfile (F005301) tempfile (F012141) tempfile (F006955)  (F020401) tempfile (F003921) tempfile (F006800) tempfile (F006789) tempfile (F003642) tempfile (F010784) tempfile (F002552) tempfile (F003429) tempfile (F005605) tempfile (F012139) tempfile (F001885) tempfile (F003644) tempfile (F003592) tempfile (F005309) tempfile (F002242) tempfile (F001910) tempfile (F003308) tempfile (F003721) tempfile (F005612) tempfile (F008283) tempfile (F002302) No0181\_Elastase\_ETD.temp (F009583) tempfile (F001698) tempfile (F001553) tempfile (F006244) Labeled H3 50% (Elastase).temp (F008967) tempfile (F003638) tempfile (F008780) tempfile (F008605) tempfile (F005602) tempfile (F002571) tempfile (F002601) HAT2 KD\_0hr.temp (F011177) tempfile (F005598) tempfile (F002597) tempfile (F001892) tempfile (F003715) tempfile (F003430) tempfile (F006236) tempfile (F002250) tempfile (F006764) tempfile (F002298)  (F016878) HAT2 KD\_24hr.temp (F011184) tempfile (F005103) tempfile (F006755) tempfile (F002234) tempfile (F003692) tempfile (F005609) tempfile (F003641) tempfile (F012137) tempfile (F003098) tempfile (F002560) tempfile (F005600) tempfile (F001535) tempfile (F003576) tempfile (F005300)  (F020408) tempfile (F003216) HAT2 KD\_48h.temp (F011317) tempfile (F006966) tempfile (F003931) RE 50pcAc light, 50pcAc heavy.temp (F009309) Light Acetylated H3\_Quan.temp (F011076) tempfile (F003580) tempfile (F003307) Light Acetylated H3\_Quan.temp (F010887) tempfile (F003421) tempfile (F003645) tempfile (F005303) tempfile (F006774) tempfile (F016301) tempfile (F009741) tempfile (F006951) tempfile (F010802) tempfile (F006806) tempfile (F003651) tempfile (F003722) tempfile (F002243) tempfile (F003718) tempfile (F003435) tempfile (F003177) tempfile (F016300) tempfile (F002568) tempfile (F002284) Labeled H3 (Trypsine).temp (F009156) tempfile (F003639) tempfile (F009742)  Heavy Acetylated TY1-H2A.temp (F016324) tempfile (F006246) tempfile (F006834) tempfile (F003306) tempfile (F016302) tempfile (F003650) No0181\_Elastase\_HCD.temp (F009584) tempfile (F001552) Light Acetylated H3\_Quan.temp (F010895) tempfile (F005665) tempfile (F006958) tempfile (F003618) tempfile (F002304) tempfile (F016247)  Heavy Acetylated TY1-H2A.temp (F016323) tempfile (F003440) tempfile (F006964) tempfile (F006241) tempfile (F005573) tempfile (F005579) tempfile (F006767) tempfile (F001886) tempfile (F005585) HAT3 KO\_Upper\_Elastase.temp (F011230) tempfile (F001567) tempfile (F003698) tempfile (F002626) tempfile (F002301) tempfile (F008284) tempfile (F002285) tempfile (F002297) tempfile (F005124) tempfile (F005136) tempfile (F003114) HAT2 KD\_24h.temp (F011311) tempfile (F003695) tempfile (F002567) tempfile (F006766) tempfile (F010785) tempfile (F002611) tempfile (F002244) tempfile (F002629) tempfile (F005614) tempfile (F006754) HAT2 KD\_24h.temp (F011312) tempfile (F002608) tempfile (F003725)  (F020377) tempfile (F006829) tempfile (F003691) tempfile (F003213) tempfile (F006952)  (F020403) tempfile (F006780) tempfile (F003975) tempfile (F016289) tempfile (F005302) tempfile (F002623) tempfile (F002554) tempfile (F016291) tempfile (F006833) tempfile (F003096) tempfile (F003295) tempfile (F006237) tempfile (F003987) tempfile (F006479) tempfile (F002630)  (F020405) Light Acetylated H3\_Elastase.temp (F010886) tempfile (F001730) tempfile (F006571) tempfile (F006757) tempfile (F003697) tempfile (F006788) tempfile (F003723) tempfile (F003624) tempfile (F005532) tempfile (F001887) tempfile (F001854) tempfile (F003646) tempfile (F006791) tempfile (F006965) tempfile (F003113) tempfile (F003713) tempfile (F016284) tempfile (F008562) tempfile (F008285) Light Acetylated H3\_Proteinase K.temp (F010894) tempfile (F009740) tempfile (F003694) tempfile (F005143) tempfile (F002599) tempfile (F006810) tempfile (F006238) tempfile (F006957) tempfile (F003700) tempfile (F005298) tempfile (F006572) tempfile (F006798) tempfile (F003051) tempfile (F016250) tempfile (F010807) tempfile (F005584) tempfile (F006242) tempfile (F009744) tempfile (F005670) tempfile (F001894) tempfile (F008564) tempfile (F012140) tempfile (F002245) tempfile (F001700) HAT3 KO\_Middle\_Elastase.temp (F011231) tempfile (F003484) tempfile (F006761) tempfile (F006803) tempfile (F003989) tempfile (F006787) Labeled H3 50% (Thermolysine).temp (F008968) tempfile (F005304) tempfile (F001722) tempfile (F003588) tempfile (F002603) tempfile (F008600) tempfile (F003724) tempfile (F003926) tempfile (F003178) tempfile (F006773) tempfile (F010805)  (F020397) tempfile (F006959) tempfile (F006472) tempfile (F005142) tempfile (F002252) tempfile (F002249) tempfile (F006814) tempfile (F003997) tempfile (F003492) tempfile (F005139) tempfile (F003625) tempfile (F002555) HAT2 KD\_0h.temp (F011293) tempfile (F007915) tempfile (F002595) tempfile (F001729) tempfile (F003071) tempfile (F002240) tempfile (F002609) tempfile (F007300) tempfile (F001895) tempfile (F016286) tempfile (F006781) tempfile (F003310) tempfile (F001914) tempfile (F002558) tempfile (F003593) tempfile (F006775) tempfile (F001536) tempfile (F001554)  (F020400) HAT2 KD\_48h.temp (F011313)  (F020396) tempfile (F003211) tempfile (F006239) tempfile (F008565) tempfile (F003582) tempfile (F002238) tempfile (F002561) BSA Papain Verdau ohne Cystein .temp (F011903) tempfile (F001855) tempfile (F007882) tempfile (F002251) tempfile (F002625) tempfile (F005299)  (F020407) tempfile (F009743) tempfile (F006799) tempfile (F003719) tempfile (F006243) tempfile (F006811) tempfile (F001621) tempfile (F006804) tempfile (F010806) tempfile (F003590) tempfile (F010808) tempfile (F003707) tempfile (F003699) tempfile (F003711) tempfile (F003998) tempfile (F005601) tempfile (F012136) HAT3 KO\_Upper\_Thermolysine.temp (F011288) tempfile (F005611) tempfile (F006759) HAT2 KD\_0h.temp (F011292) tempfile (F003587) tempfile (F002246) tempfile (F006756) tempfile (F006753) tempfile (F005305) tempfile (F003434) tempfile (F006790) tempfile (F003647) tempfile (F001723) tempfile (F001570) tempfile (F003988) tempfile (F005595) tempfile (F003304) tempfile (F004976) tempfile (F005570) tempfile (F006772) tempfile (F007733) tempfile (F006805) tempfile (F003626) tempfile (F003575) tempfile (F009739) tempfile (F006786) tempfile (F005141) HAT2 KD\_0h.temp (F011291) tempfile (F003054) tempfile (F005306) Rasha.temp (F008810) tempfile (F003648) tempfile (F008762) tempfile (F006769) tempfile (F002237) tempfile (F006960) tempfile (F003439) tempfile (F016292) tempfile (F001557) HAT3 KO\_Lower\_Thermolysine.temp (F011290) tempfile (F007827) tempfile (F006768) tempfile (F001857) tempfile (F008601) tempfile (F006793) tempfile (F002569) tempfile (F003112) tempfile (F008286) tempfile (F005583) tempfile (F009650) tempfile (F001856) tempfile (F002557) tempfile (F006760) tempfile (F010794) tempfile (F006812) tempfile (F005577) tempfile (F006240) HAT2 KD\_48h.temp (F011314) tempfile (F002631) tempfile (F005610) tempfile (F003982) tempfile (F003424) tempfile (F016285) tempfile (F006813) LCMS001711 (F020343) LCMS001834 (F020409) LCMS001835 (F020410) LCMS001836 (F020411) LCMS001841 (F020413) 01\_velos\_2700 (F004434) 01\_velos\_2700 (F004435) 01\_velos\_2701 (F004437) 01\_velos\_2701 (F004436) 01\_velos\_2702 (F004438) 01\_velos\_2703 (F004439) 01\_velos\_2704 (F004440) 01\_velos\_2704 (F004441) 01\_velos\_2705 (F004443) 01\_velos\_2705 (F004442) 01\_velos\_2708 (F004445) 01\_velos\_2708 (F004444) 01\_velos\_2709 (F004447) 01\_velos\_2709 (F004446) 01\_velos\_2710 (F004448) 01\_velos\_2711 (F004449) 01\_velos\_2712 (F004450) 01\_velos\_2712 (F004451) 01\_velos\_2713 (F004452) 01\_velos\_2713 (F004453) 06\_Fusion\_0164 (F017353) 06\_Fusion\_0165 (F017354) 06\_Fusion\_0166 (F017355) 06\_velos\_2737 (F004554) 06\_velos\_2738 (F004556) 06\_velos\_2739 (F004558) 06\_velos\_2740 (F004559) 06\_velos\_2741 (F004560) 06\_velos\_2742 (F004562) 06\_velos\_2745 (F004564) 06\_velos\_2746 (F004566) 06\_velos\_2747 (F004568) 06\_velos\_2748 (F004569) 06\_velos\_2749 (F004570) 06\_velos\_2750 (F004572) 15\_velos\_2834 (F004656) 15\_velos\_2834 (F004657) 15\_velos\_2835 (F004659) 15\_velos\_2835 (F004658) 15\_velos\_2836 (F004660) 15\_velos\_2837 (F004661) 18\_Fusion\_0252 (F017360) 18\_Fusion\_0253 (F017361) 19\_velos\_2535 (F004266) 19\_velos\_2536 (F004267) 19\_velos\_2537 (F004268) 19\_velos\_2538 (F004269) 0021\_QTOF\_0254 (F020414) 0021\_QTOF\_0256 (F020415) 21\_Fusion\_0316 (F017369) 21\_Fusion\_0317 (F017370) 21\_Fusion\_0318 (F017371) 21\_velos\_2594 (F004307) 21\_velos\_2595 (F004308) 21\_velos\_2596 (F004309) 21\_velos\_2597 (F004310) 22\_velos\_2608 (F004431) 22\_velos\_2609 (F004315) 22\_velos\_2610 (F004316) 22\_velos\_2611 (F004432) 22\_velos\_2612 (F004318) 22\_velos\_2613 (F004319) 22\_velos\_2614 (F004433) 22\_velos\_2615 (F004321) 22\_velos\_2616 (F004322) 22\_velos\_2617 (F004392) 22\_velos\_2618 (F004324) 22\_velos\_2619 (F004325) 23\_velos\_1609 (F003507) 23\_velos\_1610 (F003513) 23\_velos\_1611 (F003610) 23\_velos\_1612 (F003512) 23\_velos\_1613 (F003508) 23\_velos\_1614 (F003511) 26\_JV\_0200 (F001498) 26\_JV\_0201 (F001500) 26\_velos\_2672 (F004541) 26\_velos\_2673 (F004542) 26\_velos\_2674 (F004543) 26\_velos\_2675 (F004544) 26\_velos\_2676 (F004545) 26\_velos\_2677 (F004546) 26\_velos\_2685 (F004394) 26\_velos\_2686 (F004396) 26\_velos\_2687 (F004397) 26\_velos\_2688 (F004398) 27\_JV\_0219 (F001534) 29\_velos\_1722 (F003376) 29\_velos\_1723 (F003399) 29\_velos\_1724 (F003407) 29\_velos\_1725 (F003400) 0035\_QTOF\_0374 (F020416) 0035\_QTOF\_0376 (F020418) 1:1 Acetylated H3 (Trypsine).temp (F009289) 06 (F017359) 06 (F017356) 21 (F017990) 21 (F018005) 21 (F018010) 21 (F017996) 21 (F018007) 21 (F017991) 21 (F018002) 21 (F017997) 21 (F018011) 21 (F018006) 21 (F017981) 21 (F017998) 21 (F019355) 21 (F017416) 21 (F017984) 21 (F018000) 21 (F017993) 21 (F018009) 21 (F017982) 21 (F017983) 21 (F017999) 21 (F018008) 21 (F018001) 21 (F017985) 21 (F017417) 21 (F019354) 21 (F017977) 21 (F017995) 21 (F018003) 21 (F017976) 21 (F017992) 21 (F017979) 21 (F017989) 21 (F017418) 21 (F017988) 21 (F019359) 21 (F017987) 21 (F019360) 21 (F017986) 21 (F017980) 21 (F018004) 21 (F017978) 21 (F017994) 50% Acetylated H3\_E 2.temp (F009745) 50% Acetylated H3\_All-in-one method (Trypsin).temp (F009504) 50% Acetylated H3\_Elastase.temp (F010996) 50% Acetylated H3\_Elastase.temp (F010760) 50% Acetylated H3\_Th 2.temp (F009749) 50% Acetylated H3\_Thermolysine.temp (F010761) 50% Acetylated H3\_Elastase.temp (F012673) 50% Heavy Acetylated H3\_Elastase (1st).temp (F012765) 50% Heavy Acetylated H3\_Papain (1st).temp (F012767) 2013-01-15\_velos\_2171\_dPER\_dTIM only (F004129) 2013-01-17\_velos\_2223\_CSF\_Tryp\_ETD\_only\_PLK4\_AGMS (F004077) 2013-01-15\_velos\_2187\_dPER\_dTIM only (F004132) 2013-01-17\_velos\_2231\_Int\_Thermo\_HCD\_only\_PLK4\_AGMS (F004084) 2013-01-17\_velos\_2217\_M\_Thermo\_HCD\_onlyPLK4\_AGMS (F004072) 2013-01-17\_velos\_2229\_Int\_Trypsin\_HCD\_only\_PLK4\_AGMS (F004082) 2013-01-17\_velos\_2230\_Int\_Trypsin\_ETD\_only\_PLK4\_AGMS (F004083) 2013-01-15\_velos\_2188\_dPER\_dTIM only (F004131) 2013-01-17\_velos\_2215\_M\_Tryp\_HCD\_onlyPLK4\_AGMS (F004070) 2013-01-17\_velos\_2216\_M\_Tryp\_ETD\_onlyPLK4\_AGMS (F004071) 2013-01-17\_velos\_2227\_CSF\_Elastase\_ETD\_only\_PLK4\_AGMS (F004081) 2013-01-15\_velos\_2185\_5 \_dPER\_dTIM only (F004127) 2013-01-17\_velos\_2226\_CSF\_Elastase\_HCD\_only\_PLK4\_AGMS (F004079) 2013-01-17\_velos\_2225\_CSF\_Thermo\_ETD\_only\_PLK4\_AGMS (F004080) 2013-01-17\_velos\_2219\_M\_Elastase\_HCD\_onlyPLK4\_AGMS (F004074) 2013-01-15\_velos\_2186-dPER\_dTIM only (F004130) 2013-01-17\_velos\_2234\_Int\_Elastase\_ETD\_only\_PLK4\_AGMS (F004087) 2013-01-17\_velos\_2218\_M\_Thermo\_ETD\_onlyPLK4\_AGMS (F004073) 2013-01-17\_velos\_2222\_CSF\_Tryp\_HCD\_only\_PLK4\_AGMS (F004076) 2013-01-17\_velos\_2233\_Int\_Elastase\_HCD\_only\_PLK4\_AGMS (F004086) 2013-01-17\_velos\_2220\_M\_Elastase\_ETD\_only\_PLK4\_AGMS (F004075) 2013-01-17\_velos\_2232\_Int\_Thermo\_ETD\_only\_PLK4\_AGMS (F004085) 2013-01-17\_velos\_2224\_CSF\_Thermo\_HCD\_only\_PLK4\_AGMS (F004078) 2013-01-15\_velos\_2170\_dPER\_dTIM only (F004128) | |  |  |  | | --- | --- | --- | | MS Files: |  | Color: | |  | | | | (F020377) |  |  | | (F020380) |  |  | | (F020396) |  |  | | (F020397) |  |  | | (F020400) |  |  | | (F020401) |  |  | | (F020402) |  |  | | (F020403) |  |  | | (F020404) |  |  | | (F020405) |  |  | | (F020406) |  |  | | (F020407) |  |  | | (F020408) |  |  | | LCMS001834 (F020409) |  |  | | LCMS001835 (F020410) |  |  | | LCMS001836 (F020411) |  |  | | LCMS001841 (F020413) |  |  | | 0021\_QTOF\_0254 (F020414) |  |  | | 0021\_QTOF\_0256 (F020415) |  |  | | 0035\_QTOF\_0374 (F020416) |  |  | | 0035\_QTOF\_0376 (F020418) |  |  | | |  | | --- | | sp|C04982|C04982\_CHICK sp|Q04982|BRAF\_CHICK tr|F1P1L9|F1P1L9\_CHICK sp|P11501|HS90A\_CHICK tr|F1NVN4|F1NVN4\_CHICK sp|P05625|RAF1\_CHICK tr|V9H0B6|V9H0B6\_CHICK tr|R4GLP6|R4GLP6\_CHICK P04264 tr|Q5ZKZ3|Q5ZKZ3\_CHICK tr|Q5ZMA5|Q5ZMA5\_CHICK tr|Q5ZIC0|Q5ZIC0\_CHICK tr|F1NC33|F1NC33\_CHICK sp|Q04619|HS90B\_CHICK tr|Q2XQE5|Q2XQE5\_CHICK tr|F1NU40|F1NU40\_CHICK sp|P15771|NUCL\_CHICK tr|F1P336|F1P336\_CHICK tr|F1N8V2|F1N8V2\_CHICK P13645 P35527 tr|F1NFS0|F1NFS0\_CHICK sp|Q90705|EF2\_CHICK P02769 tr|R4GK33|R4GK33\_CHICK P00761 tr|A6BM71|A6BM71\_CHICK sp|Q9PU36|PCLO\_CHICK tr|E1BW15|E1BW15\_CHICK tr|E1C037|E1C037\_CHICK tr|F1P4B2|F1P4B2\_CHICK tr|F1NRP8|F1NRP8\_CHICK P02535-1 tr|E1BZI5|E1BZI5\_CHICK tr|E1BT53|E1BT53\_CHICK tr|F1NXZ3|F1NXZ3\_CHICK tr|F1NXZ5|F1NXZ5\_CHICK tr|F1NAC4|F1NAC4\_CHICK tr|E1BR45|E1BR45\_CHICK ENSEMBL:ENSBTAP00000038253 tr|F1NG02|F1NG02\_CHICK tr|F1NGT4|F1NGT4\_CHICK tr|E1BXK4|E1BXK4\_CHICK sp|Q90953|CSPG2\_CHICK tr|F1NZR9|F1NZR9\_CHICK tr|F1NLZ9|F1NLZ9\_CHICK P35908 tr|F1NLP0|F1NLP0\_CHICK tr|F1NMK9|F1NMK9\_CHICK tr|E1C4H7|E1C4H7\_CHICK tr|E1BZM6|E1BZM6\_CHICK tr|E1C245|E1C245\_CHICK tr|F1N865|F1N865\_CHICK tr|F1NG08|F1NG08\_CHICK tr|F1P2G5|F1P2G5\_CHICK tr|F1NFJ6|F1NFJ6\_CHICK tr|H9L0M3|H9L0M3\_CHICK tr|Q197X2|Q197X2\_CHICK tr|F1NV02|F1NV02\_CHICK tr|E1C4T6|E1C4T6\_CHICK tr|E1C186|E1C186\_CHICK Q922U2 tr|F1NET5|F1NET5\_CHICK tr|R4GG37|R4GG37\_CHICK Q5XQN5 tr|Q5ZMF6|Q5ZMF6\_CHICK tr|F1NU73|F1NU73\_CHICK tr|F1NBK4|F1NBK4\_CHICK tr|F1N9R6|F1N9R6\_CHICK tr|F1NWW5|F1NWW5\_CHICK tr|F1P4P1|F1P4P1\_CHICK tr|F1NKL4|F1NKL4\_CHICK tr|F1N9C7|F1N9C7\_CHICK tr|F1NWT2|F1NWT2\_CHICK tr|F1NYR0|F1NYR0\_CHICK tr|F1NU74|F1NU74\_CHICK P50446 tr|F1P3D1|F1P3D1\_CHICK tr|F1NLF6|F1NLF6\_CHICK tr|E1C796|E1C796\_CHICK tr|F1N838|F1N838\_CHICK tr|F1N8N1|F1N8N1\_CHICK Q8BGZ7 tr|Q6KDZ1|Q6KDZ1\_CHICK tr|F1P1U0|F1P1U0\_CHICK tr|F1P2S8|F1P2S8\_CHICK tr|F1NJK1|F1NJK1\_CHICK tr|H9KZA3|H9KZA3\_CHICK tr|Q5ZLW6|Q5ZLW6\_CHICK tr|E1C4X6|E1C4X6\_CHICK P02768-1 tr|F1P394|F1P394\_CHICK tr|E1BW48|E1BW48\_CHICK tr|Q90985|Q90985\_CHICK tr|Q9W6V0|Q9W6V0\_CHICK tr|F1NZF2|F1NZF2\_CHICK tr|E1C6A6|E1C6A6\_CHICK tr|F1NQR9|F1NQR9\_CHICK tr|F1NSR9|F1NSR9\_CHICK P02538 Q6NXH9 tr|F1N8A8|F1N8A8\_CHICK tr|E1C1Q2|E1C1Q2\_CHICK tr|F1NGF6|F1NGF6\_CHICK tr|F1NX10|F1NX10\_CHICK tr|F1NLA9|F1NLA9\_CHICK tr|E1BSS0|E1BSS0\_CHICK tr|F1P3B2|F1P3B2\_CHICK tr|E1BUT8|E1BUT8\_CHICK tr|E1BS70|E1BS70\_CHICK tr|E1C703|E1C703\_CHICK tr|F1NVK1|F1NVK1\_CHICK Q7Z794 tr|Q8UW79|Q8UW79\_CHICK tr|F1NI54|F1NI54\_CHICK tr|F1NGB5|F1NGB5\_CHICK tr|E1BWG0|E1BWG0\_CHICK P48668 sp|Q9YH85|TECTA\_CHICK tr|H9KYS8|H9KYS8\_CHICK tr|F1NWW4|F1NWW4\_CHICK tr|E1C9F6|E1C9F6\_CHICK tr|Q8QFV6|Q8QFV6\_CHICK tr|F1NDW5|F1NDW5\_CHICK tr|F1NNX7|F1NNX7\_CHICK sp|P07898|PGCA\_CHICK tr|F1NJ66|F1NJ66\_CHICK sp|P15989|CO6A3\_CHICK tr|F1NW66|F1NW66\_CHICK tr|E1BYT6|E1BYT6\_CHICK P04259 tr|F1P0U1|F1P0U1\_CHICK sp|P12276|FAS\_CHICK tr|E1BYY8|E1BYY8\_CHICK tr|E1BT10|E1BT10\_CHICK tr|F1NE63|F1NE63\_CHICK sp|P13944|COCA1\_CHICK tr|E1BQS4|E1BQS4\_CHICK tr|E1C566|E1C566\_CHICK tr|E1BUS5|E1BUS5\_CHICK tr|P79787|P79787\_CHICK Q32MB2 tr|Q98918|Q98918\_CHICK tr|F1NX22|F1NX22\_CHICK tr|E1C5C8|E1C5C8\_CHICK tr|F1NAU7|F1NAU7\_CHICK tr|F1NMY8|F1NMY8\_CHICK tr|R4GIB2|R4GIB2\_CHICK tr|F1P2F0|F1P2F0\_CHICK tr|F1NPE9|F1NPE9\_CHICK tr|E1BW33|E1BW33\_CHICK tr|F1NQD1|F1NQD1\_CHICK tr|F1NA05|F1NA05\_CHICK tr|H9L010|H9L010\_CHICK tr|F1N821|F1N821\_CHICK tr|R4GKR8|R4GKR8\_CHICK Q9R0H5 tr|R9PXQ5|R9PXQ5\_CHICK tr|R4GJD2|R4GJD2\_CHICK Q6IFZ6 tr|F1N9G0|F1N9G0\_CHICK tr|G1JT17|G1JT17\_CHICK tr|R4GF68|R4GF68\_CHICK tr|F1NZX1|F1NZX1\_CHICK tr|F1NSF6|F1NSF6\_CHICK sp|Q8QGX4|PRKDC\_CHICK tr|E1BUI5|E1BUI5\_CHICK tr|F1NFI2|F1NFI2\_CHICK tr|F1P2D1|F1P2D1\_CHICK tr|H9KZP2|H9KZP2\_CHICK tr|E1BZS9|E1BZS9\_CHICK tr|E1BZK0|E1BZK0\_CHICK tr|E1C9F0|E1C9F0\_CHICK tr|A6BLM5|A6BLM5\_CHICK tr|E1BWE2|E1BWE2\_CHICK tr|F1NPK0|F1NPK0\_CHICK tr|E1C9G7|E1C9G7\_CHICK tr|F1N812|F1N812\_CHICK tr|E1C3K2|E1C3K2\_CHICK tr|O57580|O57580\_CHICK tr|E1BZ80|E1BZ80\_CHICK tr|D2W6W9|D2W6W9\_CHICK tr|F1NQT0|F1NQT0\_CHICK tr|E1C3T2|E1C3T2\_CHICK tr|F1NHE5|F1NHE5\_CHICK tr|F1NAH8|F1NAH8\_CHICK tr|F1NAK4|F1NAK4\_CHICK tr|E1C6E6|E1C6E6\_CHICK tr|E1C549|E1C549\_CHICK tr|Q6PVZ3|Q6PVZ3\_CHICK tr|H9KZ27|H9KZ27\_CHICK tr|Q6W4W6|Q6W4W6\_CHICK tr|E1BS48|E1BS48\_CHICK tr|E1BXR9|E1BXR9\_CHICK tr|E1BXI6|E1BXI6\_CHICK tr|E1C352|E1C352\_CHICK tr|F1NSG3|F1NSG3\_CHICK sp|Q06A37|CHD7\_CHICK tr|F1NWA0|F1NWA0\_CHICK tr|F1NM47|F1NM47\_CHICK tr|F1P5U7|F1P5U7\_CHICK tr|E1C2U2|E1C2U2\_CHICK sp|Q0ZM14|PCD15\_CHICK tr|F1NBM7|F1NBM7\_CHICK tr|F1NP17|F1NP17\_CHICK tr|F1NEP2|F1NEP2\_CHICK tr|F1NC41|F1NC41\_CHICK tr|F1N8Z9|F1N8Z9\_CHICK sp|Q8AV58|SDK1\_CHICK tr|R4GM79|R4GM79\_CHICK tr|F1NPT1|F1NPT1\_CHICK tr|P79994|P79994\_CHICK tr|F1NV50|F1NV50\_CHICK tr|R4GLV4|R4GLV4\_CHICK tr|E1C224|E1C224\_CHICK tr|F1NWS8|F1NWS8\_CHICK tr|F1NZ85|F1NZ85\_CHICK tr|E1C4G2|E1C4G2\_CHICK tr|H9L0N7|H9L0N7\_CHICK tr|F1P153|F1P153\_CHICK tr|E1BYG4|E1BYG4\_CHICK tr|E1C509|E1C509\_CHICK tr|E1C3K6|E1C3K6\_CHICK tr|F1NSL6|F1NSL6\_CHICK tr|F1NI75|F1NI75\_CHICK tr|F1NBA6|F1NBA6\_CHICK sp|Q9W6V6|TEN1\_CHICK tr|F1NN19|F1NN19\_CHICK Q8VED5 tr|F1P033|F1P033\_CHICK tr|E1C8J1|E1C8J1\_CHICK tr|F1NTR2|F1NTR2\_CHICK sp|O93574|RELN\_CHICK tr|E1BYQ7|E1BYQ7\_CHICK tr|E1BUS3|E1BUS3\_CHICK tr|F1P1A1|F1P1A1\_CHICK tr|F1NV58|F1NV58\_CHICK tr|E1C5B0|E1C5B0\_CHICK tr|E1C756|E1C756\_CHICK sp|P54939|TLN1\_CHICK tr|E1C889|E1C889\_CHICK sp|A8C754|THADA\_CHICK tr|H2EQW7|H2EQW7\_CHICK tr|E1C2S1|E1C2S1\_CHICK sp|P98157|LRP1\_CHICK tr|E1C040|E1C040\_CHICK tr|F1NJS9|F1NJS9\_CHICK Q86YZ3 tr|F1P420|F1P420\_CHICK Q29443 Q0IIK2 tr|F1NKX2|F1NKX2\_CHICK tr|F1NVT4|F1NVT4\_CHICK tr|E1BU04|E1BU04\_CHICK tr|E1C218|E1C218\_CHICK tr|F1NV65|F1NV65\_CHICK tr|E1C0Q6|E1C0Q6\_CHICK tr|R4GJI2|R4GJI2\_CHICK tr|E1C5B4|E1C5B4\_CHICK Q9QWL7 tr|E1C8E4|E1C8E4\_CHICK Q7Z3Y8 tr|E1C712|E1C712\_CHICK tr|E1BTW4|E1BTW4\_CHICK tr|F1NZ92|F1NZ92\_CHICK tr|E1BR51|E1BR51\_CHICK tr|F1NVR7|F1NVR7\_CHICK tr|F1NXC8|F1NXC8\_CHICK tr|F1NAS5|F1NAS5\_CHICK tr|F1NW72|F1NW72\_CHICK tr|F1N8I1|F1N8I1\_CHICK tr|F1NMT0|F1NMT0\_CHICK tr|F1NYY9|F1NYY9\_CHICK sp|Q2PC93|SSPO\_CHICK tr|F1P4F5|F1P4F5\_CHICK Q04695 tr|E1BYU8|E1BYU8\_CHICK sp|O73590|ZFHX4\_CHICK tr|F1ND08|F1ND08\_CHICK Q3TTY5 P20930 tr|E1BQG1|E1BQG1\_CHICK tr|Q5ZJ36|Q5ZJ36\_CHICK tr|F1P1B9|F1P1B9\_CHICK tr|E1BSG1|E1BSG1\_CHICK P02533 tr|F1NSE4|F1NSE4\_CHICK tr|F1NB33|F1NB33\_CHICK tr|E1BQN2|E1BQN2\_CHICK tr|E1BUM1|E1BUM1\_CHICK tr|E1BWI0|E1BWI0\_CHICK tr|E1C835|E1C835\_CHICK tr|E7EL51|E7EL51\_CHICK tr|F9W2Y0|F9W2Y0\_CHICK tr|F1P187|F1P187\_CHICK tr|F1NXK5|F1NXK5\_CHICK P19013 tr|F1NZY1|F1NZY1\_CHICK tr|B4ZAD9|B4ZAD9\_CHICK tr|E1BYQ6|E1BYQ6\_CHICK tr|F1NNK1|F1NNK1\_CHICK tr|E1C9J5|E1C9J5\_CHICK tr|F1NNU6|F1NNU6\_CHICK tr|E1C7E8|E1C7E8\_CHICK tr|E1BST4|E1BST4\_CHICK tr|E1BZT1|E1BZT1\_CHICK tr|F1NQB4|F1NQB4\_CHICK tr|F1NCX7|F1NCX7\_CHICK tr|F1P172|F1P172\_CHICK tr|F1NYT6|F1NYT6\_CHICK tr|F1NJ76|F1NJ76\_CHICK tr|E1BU18|E1BU18\_CHICK tr|F1NGW1|F1NGW1\_CHICK tr|R4GIL8|R4GIL8\_CHICK tr|R4GGY3|R4GGY3\_CHICK tr|F1NLI6|F1NLI6\_CHICK sp|Q9PW38|GEPH\_CHICK sp|E1C231|LTN1\_CHICK Q148H6 tr|F1N855|F1N855\_CHICK tr|E1BVC3|E1BVC3\_CHICK sp|Q9DER5|TEN2\_CHICK tr|F1P479|F1P479\_CHICK tr|H9KZX6|H9KZX6\_CHICK tr|Q6PVZ5|Q6PVZ5\_CHICK tr|F1P236|F1P236\_CHICK P04258 sp|Q8AV57|SDK2\_CHICK tr|F1NUN9|F1NUN9\_CHICK tr|F1N8L6|F1N8L6\_CHICK tr|E1BYP1|E1BYP1\_CHICK tr|E1BS85|E1BS85\_CHICK tr|F1NCT3|F1NCT3\_CHICK tr|F1NJ26|F1NJ26\_CHICK sp|P02717|ACHD\_CHICK Q7Z3Y7 tr|E1BSL3|E1BSL3\_CHICK tr|Q8UUR1|Q8UUR1\_CHICK tr|R4GFE4|R4GFE4\_CHICK tr|R4GI73|R4GI73\_CHICK sp|P11533|DMD\_CHICK tr|F1NS04|F1NS04\_CHICK tr|F1P3M4|F1P3M4\_CHICK tr|F1NMB5|F1NMB5\_CHICK tr|E1BVF4|E1BVF4\_CHICK tr|E1C8P7|E1C8P7\_CHICK tr|F1NLW6|F1NLW6\_CHICK Q7Z3Z0 P13647 tr|Q9IAR9|Q9IAR9\_CHICK tr|E1BSB9|E1BSB9\_CHICK tr|F1NZB4|F1NZB4\_CHICK tr|E1C420|E1C420\_CHICK tr|F1NK98|F1NK98\_CHICK tr|F1NZL2|F1NZL2\_CHICK tr|F1N8D4|F1N8D4\_CHICK tr|F1NLL8|F1NLL8\_CHICK sp|Q90941|PB1\_CHICK tr|E1C8H6|E1C8H6\_CHICK tr|Q76FQ4|Q76FQ4\_CHICK tr|E1C1F2|E1C1F2\_CHICK tr|F1N988|F1N988\_CHICK tr|F1NCU5|F1NCU5\_CHICK tr|F1N972|F1N972\_CHICK tr|E1BSW5|E1BSW5\_CHICK sp|Q5ZI87|TBCD\_CHICK tr|F1NIW7|F1NIW7\_CHICK tr|F1NFE1|F1NFE1\_CHICK tr|Q5F3Q0|Q5F3Q0\_CHICK Q5D862 tr|F1NX51|F1NX51\_CHICK tr|F1NVD2|F1NVD2\_CHICK tr|F1P3B0|F1P3B0\_CHICK tr|F1NR30|F1NR30\_CHICK tr|E1BQT3|E1BQT3\_CHICK tr|R4GGD1|R4GGD1\_CHICK tr|Q90574|Q90574\_CHICK tr|F1NXN7|F1NXN7\_CHICK tr|E1BQY4|E1BQY4\_CHICK tr|F1P1J1|F1P1J1\_CHICK tr|F1NH85|F1NH85\_CHICK tr|E1BWS6|E1BWS6\_CHICK tr|A4D0F7|A4D0F7\_CHICK tr|Q5F3B8|Q5F3B8\_CHICK tr|F1NPN0|F1NPN0\_CHICK tr|F1NYE1|F1NYE1\_CHICK tr|E1BQJ6|E1BQJ6\_CHICK tr|F1NMT3|F1NMT3\_CHICK tr|F1NGZ7|F1NGZ7\_CHICK tr|E1C5Z3|E1C5Z3\_CHICK tr|E1BVU5|E1BVU5\_CHICK tr|F1N8D7|F1N8D7\_CHICK tr|F1NV33|F1NV33\_CHICK tr|F1NMK5|F1NMK5\_CHICK sp|Q5ZJB7|CHMP7\_CHICK tr|E1C368|E1C368\_CHICK tr|E1C0J5|E1C0J5\_CHICK tr|F1NAV5|F1NAV5\_CHICK Q28107 tr|F1N9P6|F1N9P6\_CHICK tr|F1NXK6|F1NXK6\_CHICK tr|F1NS97|F1NS97\_CHICK tr|E1BQT2|E1BQT2\_CHICK tr|E1BZC4|E1BZC4\_CHICK tr|F1NG38|F1NG38\_CHICK tr|L7XFH1|L7XFH1\_CHICK tr|R4GGN4|R4GGN4\_CHICK tr|F1NYP5|F1NYP5\_CHICK tr|Q76CF0|Q76CF0\_CHICK tr|F1NAX8|F1NAX8\_CHICK tr|E1BWU6|E1BWU6\_CHICK tr|E1BW01|E1BW01\_CHICK tr|F1NMU1|F1NMU1\_CHICK sp|Q9IA31|GLI3\_CHICK P08779 tr|Q90681|Q90681\_CHICK tr|E1C780|E1C780\_CHICK tr|H9L033|H9L033\_CHICK tr|F1P4L4|F1P4L4\_CHICK tr|B3TZC1|B3TZC1\_CHICK tr|Q90ZW5|Q90ZW5\_CHICK tr|F1NTL4|F1NTL4\_CHICK tr|G1UCX5|G1UCX5\_CHICK tr|E1C6J4|E1C6J4\_CHICK tr|E1C4Z6|E1C4Z6\_CHICK tr|F5XVB5|F5XVB5\_CHICK ENSEMBL:ENSBTAP00000024146 tr|E1C834|E1C834\_CHICK sp|Q1XI86|IMPG2\_CHICK tr|F1P3R3|F1P3R3\_CHICK tr|F1NNC6|F1NNC6\_CHICK tr|F1NW23|F1NW23\_CHICK tr|E1C576|E1C576\_CHICK tr|E1C1S5|E1C1S5\_CHICK tr|E1BYF7|E1BYF7\_CHICK tr|F6UXS6|F6UXS6\_CHICK tr|E1C6T3|E1C6T3\_CHICK tr|E1BZV7|E1BZV7\_CHICK tr|F1NT50|F1NT50\_CHICK tr|F1P362|F1P362\_CHICK tr|F1NGV2|F1NGV2\_CHICK Q3SY84 sp|Q5ZMV8|RPGP2\_CHICK tr|F1NJZ2|F1NJZ2\_CHICK tr|E1BQZ3|E1BQZ3\_CHICK tr|F1NI79|F1NI79\_CHICK tr|E1BTA1|E1BTA1\_CHICK tr|F1NC54|F1NC54\_CHICK tr|E1BRH3|E1BRH3\_CHICK tr|Q5BMR5|Q5BMR5\_CHICK tr|F1N9S6|F1N9S6\_CHICK tr|E1C293|E1C293\_CHICK sp|P11029|ACAC\_CHICK tr|F1NWT0|F1NWT0\_CHICK tr|R4R041|R4R041\_CHICK tr|R4GH84|R4GH84\_CHICK tr|F1NX21|F1NX21\_CHICK tr|Q3I0Z3|Q3I0Z3\_WNV tr|F1DA41|F1DA41\_WNV tr|F1NJ24|F1NJ24\_CHICK tr|Q9IAU4|Q9IAU4\_CHICK tr|A0M8U5|A0M8U5\_CHICK tr|E1BUF4|E1BUF4\_CHICK tr|F1NVE3|F1NVE3\_CHICK tr|F1NZ05|F1NZ05\_CHICK tr|E1C4Y0|E1C4Y0\_CHICK tr|F1NYZ6|F1NYZ6\_CHICK tr|F1NCI0|F1NCI0\_CHICK tr|F1NG87|F1NG87\_CHICK tr|E1C7T0|E1C7T0\_CHICK tr|F1D836|F1D836\_WNV tr|F1NUR6|F1NUR6\_CHICK tr|Q5F393|Q5F393\_CHICK tr|F1P217|F1P217\_CHICK tr|E1BRV1|E1BRV1\_CHICK tr|F1ND63|F1ND63\_CHICK tr|F1D844|F1D844\_WNV tr|R4GJV2|R4GJV2\_CHICK tr|F1NL47|F1NL47\_CHICK tr|F1NAS1|F1NAS1\_CHICK tr|F1NPG7|F1NPG7\_CHICK tr|F1NRG0|F1NRG0\_CHICK tr|F1NCK1|F1NCK1\_CHICK tr|F1NC46|F1NC46\_CHICK tr|F1NE56|F1NE56\_CHICK tr|F1NVW5|F1NVW5\_CHICK sp|E1BZ85|MARF1\_CHICK tr|F1NDL1|F1NDL1\_CHICK tr|Q5ZKS3|Q5ZKS3\_CHICK tr|F1NJ90|F1NJ90\_CHICK A2A4G1 sp|Q8AV28|PCM1\_CHICK tr|G5CK02|G5CK02\_WNV tr|F1D812|F1D812\_WNV tr|R4GKT6|R4GKT6\_CHICK tr|F1P4W9|F1P4W9\_CHICK tr|E1BSE4|E1BSE4\_CHICK tr|F1NTF4|F1NTF4\_CHICK tr|E1BQY3|E1BQY3\_CHICK tr|A0S0L7|A0S0L7\_CHICK tr|R4GFY0|R4GFY0\_CHICK tr|F1NZI3|F1NZI3\_CHICK tr|E1BVT9|E1BVT9\_CHICK tr|R4GFR3|R4GFR3\_CHICK tr|F1NFD4|F1NFD4\_CHICK sp|P07751|SPTN1\_CHICK tr|F1NEM5|F1NEM5\_CHICK tr|E1C7Y7|E1C7Y7\_CHICK tr|W8VTH6|W8VTH6\_CHICK sp|P02845|VIT2\_CHICK tr|F1NR84|F1NR84\_CHICK tr|F1NWH0|F1NWH0\_CHICK tr|E1BQF9|E1BQF9\_CHICK tr|Q5F3I2|Q5F3I2\_CHICK P08730-1 tr|F1NXA3|F1NXA3\_CHICK tr|R4GJF0|R4GJF0\_CHICK tr|I6WQT8|I6WQT8\_CHICK tr|R4GKZ1|R4GKZ1\_CHICK tr|Q52R01|Q52R01\_CHICK tr|A0A0A0MQ43|A0A0A0MQ43\_CHICK tr|F1NB09|F1NB09\_CHICK tr|F1NT30|F1NT30\_CHICK tr|Q9DF77|Q9DF77\_CHICK tr|Q9DFB7|Q9DFB7\_CHICK tr|F1NJT4|F1NJT4\_CHICK tr|R4QXY1|R4QXY1\_CHICK tr|R4RBA0|R4RBA0\_CHICK tr|E1C8X4|E1C8X4\_CHICK tr|F1NI60|F1NI60\_CHICK tr|F1NPJ1|F1NPJ1\_CHICK sp|Q00546|TENR\_CHICK tr|F1NRQ0|F1NRQ0\_CHICK tr|E1C6U4|E1C6U4\_CHICK tr|F1NIT2|F1NIT2\_CHICK tr|F1NXP9|F1NXP9\_CHICK tr|F1NV90|F1NV90\_CHICK tr|E1C1N7|E1C1N7\_CHICK tr|E1BXT7|E1BXT7\_CHICK tr|E1BTG7|E1BTG7\_CHICK tr|E1BZL0|E1BZL0\_CHICK tr|R4GKS5|R4GKS5\_CHICK sp|P09987|H1\_CHICK tr|F1NP75|F1NP75\_CHICK tr|E1C805|E1C805\_CHICK tr|E1BVC6|E1BVC6\_CHICK tr|F1NEA4|F1NEA4\_CHICK tr|F1NAH4|F1NAH4\_CHICK tr|F1NXV1|F1NXV1\_CHICK tr|F1NW59|F1NW59\_CHICK tr|E1C8H5|E1C8H5\_CHICK tr|F1NGJ3|F1NGJ3\_CHICK tr|F1NT94|F1NT94\_CHICK tr|F1NFL6|F1NFL6\_CHICK tr|F1P2F9|F1P2F9\_CHICK tr|F1NI26|F1NI26\_CHICK sp|Q90600|RB\_CHICK tr|F1NAY3|F1NAY3\_CHICK tr|F1NQ30|F1NQ30\_CHICK sp|Q5ZIT8|TAF2\_CHICK tr|E1BY72|E1BY72\_CHICK Q99456 tr|E1C949|E1C949\_CHICK sp|Q91012|T22D1\_CHICK tr|F1P3C3|F1P3C3\_CHICK tr|F6U7Z0|F6U7Z0\_CHICK sp|Q5ZM33|HP1B3\_CHICK tr|F1N8H6|F1N8H6\_CHICK tr|F1P5C8|F1P5C8\_CHICK tr|E1BUU2|E1BUU2\_CHICK tr|F1NCT5|F1NCT5\_CHICK tr|F1NVH0|F1NVH0\_CHICK tr|F1NJT3|F1NJT3\_CHICK tr|Q90WF0|Q90WF0\_CHICK tr|Q90724|Q90724\_CHICK tr|F1NI57|F1NI57\_CHICK sp|P10039|TENA\_CHICK tr|Q5ZKD2|Q5ZKD2\_CHICK tr|Q9YGM1|Q9YGM1\_CHICK tr|F1NPJ2|F1NPJ2\_CHICK tr|F1NB92|F1NB92\_CHICK tr|E1C0Z0|E1C0Z0\_CHICK tr|F1NZV1|F1NZV1\_CHICK tr|R4GGJ5|R4GGJ5\_CHICK tr|Q90WF1|Q90WF1\_CHICK tr|R4GHI1|R4GHI1\_CHICK sp|O42130|TOP2A\_CHICK tr|F1NAM5|F1NAM5\_CHICK tr|F1NCD0|F1NCD0\_CHICK tr|F1N9H2|F1N9H2\_CHICK tr|F1P243|F1P243\_CHICK tr|F1NAS3|F1NAS3\_CHICK tr|E1C7R9|E1C7R9\_CHICK tr|E1C6Z5|E1C6Z5\_CHICK tr|Q5ZIQ8|Q5ZIQ8\_CHICK tr|E1BY04|E1BY04\_CHICK tr|F1NIL9|F1NIL9\_CHICK tr|E1C6M6|E1C6M6\_CHICK tr|F1NXQ1|F1NXQ1\_CHICK tr|H9KZH9|H9KZH9\_CHICK tr|R4GM42|R4GM42\_CHICK tr|F1NKR5|F1NKR5\_CHICK sp|F1NTD6|ASCC3\_CHICK tr|Q5ZHX4|Q5ZHX4\_CHICK tr|V9GW36|V9GW36\_CHICK tr|F1NE19|F1NE19\_CHICK tr|E1BZQ6|E1BZQ6\_CHICK tr|F1NKB3|F1NKB3\_CHICK tr|F1NJR5|F1NJR5\_CHICK tr|E1C6Q8|E1C6Q8\_CHICK tr|F1P350|F1P350\_CHICK sp|P87498|VIT1\_CHICK tr|F1P413|F1P413\_CHICK tr|F1N9I6|F1N9I6\_CHICK tr|E1C5R5|E1C5R5\_CHICK tr|Q5F3A9|Q5F3A9\_CHICK O95678 tr|Q90995|Q90995\_CHICK tr|E1BV50|E1BV50\_CHICK tr|F1NHI3|F1NHI3\_CHICK tr|E1C5P6|E1C5P6\_CHICK P19001 tr|F1P4X9|F1P4X9\_CHICK tr|E1BVU8|E1BVU8\_CHICK tr|E1C1J1|E1C1J1\_CHICK sp|Q90584|COHA1\_CHICK tr|R4GIS3|R4GIS3\_CHICK tr|F1NE18|F1NE18\_CHICK tr|E1C268|E1C268\_CHICK tr|E1BV37|E1BV37\_CHICK Q2UVX4 tr|E1C9F3|E1C9F3\_CHICK tr|E1C5S0|E1C5S0\_CHICK tr|Q5ZJQ9|Q5ZJQ9\_CHICK tr|E1BWU8|E1BWU8\_CHICK tr|E1BVH9|E1BVH9\_CHICK tr|H9KZS2|H9KZS2\_CHICK tr|R4GL39|R4GL39\_CHICK tr|E1BR07|E1BR07\_CHICK tr|F1NY52|F1NY52\_CHICK tr|F1NH52|F1NH52\_CHICK tr|E1C8G3|E1C8G3\_CHICK tr|R4GIV3|R4GIV3\_CHICK tr|R4GFL7|R4GFL7\_CHICK tr|E1C071|E1C071\_CHICK tr|E1C6F6|E1C6F6\_CHICK tr|E1BVQ3|E1BVQ3\_CHICK tr|F1NE03|F1NE03\_CHICK sp|O93532|K2CO\_CHICK tr|E1BQS3|E1BQS3\_CHICK tr|F1NN86|F1NN86\_CHICK tr|F1NW88|F1NW88\_CHICK tr|E1C6D1|E1C6D1\_CHICK tr|F1NQ15|F1NQ15\_CHICK sp|Q5ZKA3|CWC22\_CHICK tr|R4GIE3|R4GIE3\_CHICK tr|F1NQI1|F1NQI1\_CHICK sp|P02457|CO1A1\_CHICK tr|F1NHZ6|F1NHZ6\_CHICK P19012 tr|F1NCD9|F1NCD9\_CHICK tr|E1BQV1|E1BQV1\_CHICK tr|F1NZJ9|F1NZJ9\_CHICK tr|F1P566|F1P566\_CHICK tr|F1P435|F1P435\_CHICK tr|F1NPQ9|F1NPQ9\_CHICK tr|E1C5L2|E1C5L2\_CHICK tr|Q5ZIE7|Q5ZIE7\_CHICK tr|F1NCV6|F1NCV6\_CHICK tr|F1NIW9|F1NIW9\_CHICK tr|Q90784|Q90784\_CHICK tr|F1N8F4|F1N8F4\_CHICK tr|F1N804|F1N804\_CHICK tr|R4GF59|R4GF59\_CHICK tr|F1N955|F1N955\_CHICK tr|F1NQG8|F1NQG8\_CHICK tr|F1NKQ9|F1NKQ9\_CHICK tr|F1NM82|F1NM82\_CHICK tr|E1C3E2|E1C3E2\_CHICK tr|F1N9Z6|F1N9Z6\_CHICK tr|Q6BCB8|Q6BCB8\_CHICK sp|O73700|CAC1D\_CHICK tr|F1NUU0|F1NUU0\_CHICK tr|E1C7E3|E1C7E3\_CHICK tr|Q91976|Q91976\_CHICK tr|F1NVB1|F1NVB1\_CHICK tr|E1BUU3|E1BUU3\_CHICK tr|F1NGL8|F1NGL8\_CHICK tr|E1BS21|E1BS21\_CHICK tr|F1NNS3|F1NNS3\_CHICK tr|F1NBM6|F1NBM6\_CHICK tr|F1NZZ2|F1NZZ2\_CHICK tr|E1BXD2|E1BXD2\_CHICK tr|F1NQ51|F1NQ51\_CHICK tr|Q59J86|Q59J86\_CHICK tr|E1BWZ2|E1BWZ2\_CHICK P05787 tr|F1NXF1|F1NXF1\_CHICK tr|F1NH79|F1NH79\_CHICK tr|F1NDB1|F1NDB1\_CHICK tr|F1NZ02|F1NZ02\_CHICK tr|Q9DEH4|Q9DEH4\_CHICK tr|E1C7N6|E1C7N6\_CHICK tr|F1NZ00|F1NZ00\_CHICK tr|E1BXA9|E1BXA9\_CHICK tr|F9W2X8|F9W2X8\_CHICK tr|F1N9I0|F1N9I0\_CHICK tr|E1C039|E1C039\_CHICK tr|A7XMT1|A7XMT1\_CHICK tr|E1C4G3|E1C4G3\_CHICK tr|F1P443|F1P443\_CHICK tr|F1NAD8|F1NAD8\_CHICK tr|F1P2P0|F1P2P0\_CHICK tr|Q90YL2|Q90YL2\_CHICK tr|F1NN75|F1NN75\_CHICK tr|H9L0N1|H9L0N1\_CHICK P02662 tr|O57613|O57613\_CHICK tr|F1NRV9|F1NRV9\_CHICK tr|E1BVC5|E1BVC5\_CHICK tr|F1NB34|F1NB34\_CHICK tr|E1BXM7|E1BXM7\_CHICK tr|F1NHT3|F1NHT3\_CHICK tr|F1NMF8|F1NMF8\_CHICK tr|E1C0S4|E1C0S4\_CHICK tr|E1BZY4|E1BZY4\_CHICK tr|R4GGI4|R4GGI4\_CHICK tr|F1NY98|F1NY98\_CHICK tr|H9L3A1|H9L3A1\_CHICK tr|F1NIA7|F1NIA7\_CHICK tr|E1C7Y6|E1C7Y6\_CHICK tr|F1NVV0|F1NVV0\_CHICK tr|H9L1C1|H9L1C1\_CHICK tr|E1BZR4|E1BZR4\_CHICK tr|R4GHT7|R4GHT7\_CHICK sp|D0PRN3|NRX3A\_CHICK tr|E1C012|E1C012\_CHICK tr|F1N8C9|F1N8C9\_CHICK tr|F1NHV0|F1NHV0\_CHICK tr|E1C2P1|E1C2P1\_CHICK tr|F1NH26|F1NH26\_CHICK tr|E1BW21|E1BW21\_CHICK tr|F1NG70|F1NG70\_CHICK tr|F1P3H8|F1P3H8\_CHICK tr|F1NMA8|F1NMA8\_CHICK tr|F1NJX0|F1NJX0\_CHICK tr|E1C8N4|E1C8N4\_CHICK sp|Q25BN1|DICER\_CHICK tr|E1BXQ1|E1BXQ1\_CHICK tr|Q98UC5|Q98UC5\_CHICK tr|E1BSR3|E1BSR3\_CHICK sp|Q5ZKB7|AT134\_CHICK tr|E1C1B8|E1C1B8\_CHICK tr|F1NLL9|F1NLL9\_CHICK tr|E1C5R4|E1C5R4\_CHICK sp|Q5ZKK7|ERCC3\_CHICK tr|F1NZ23|F1NZ23\_CHICK tr|F1NSY7|F1NSY7\_CHICK tr|F1NH78|F1NH78\_CHICK tr|E1BTI8|E1BTI8\_CHICK tr|F1NIK8|F1NIK8\_CHICK tr|Q98UC6|Q98UC6\_CHICK tr|F1NTN0|F1NTN0\_CHICK tr|Q9YGM0|Q9YGM0\_CHICK tr|F1NLZ8|F1NLZ8\_CHICK tr|F1NAR0|F1NAR0\_CHICK tr|F1NMI3|F1NMI3\_CHICK tr|E1C4W3|E1C4W3\_CHICK tr|E1C7N8|E1C7N8\_CHICK tr|G3CAT9|G3CAT9\_CHICK tr|E1BUD4|E1BUD4\_CHICK tr|F1NZV6|F1NZV6\_CHICK tr|F1NXH3|F1NXH3\_CHICK tr|E1BXE7|E1BXE7\_CHICK tr|F1P092|F1P092\_CHICK tr|E1C607|E1C607\_CHICK tr|D3KR65|D3KR65\_CHICK tr|Q08757|Q08757\_CHICK tr|E1C5T2|E1C5T2\_CHICK tr|F1NI11|F1NI11\_CHICK tr|F1NHE7|F1NHE7\_CHICK tr|F1NHJ5|F1NHJ5\_CHICK tr|A5HUM6|A5HUM6\_CHICK tr|F1NIE9|F1NIE9\_CHICK tr|F1N8H3|F1N8H3\_CHICK tr|F1NYX7|F1NYX7\_CHICK tr|E1BXZ2|E1BXZ2\_CHICK tr|G3CAU0|G3CAU0\_CHICK tr|F1NFG3|F1NFG3\_CHICK tr|F1NZ22|F1NZ22\_CHICK tr|B0FLU6|B0FLU6\_CHICK tr|R4GLQ5|R4GLQ5\_CHICK tr|E1BWB8|E1BWB8\_CHICK tr|F1NRC4|F1NRC4\_CHICK tr|F1NPS0|F1NPS0\_CHICK sp|Q8UVD9|FUBP2\_CHICK tr|F1P070|F1P070\_CHICK tr|E1BUH3|E1BUH3\_CHICK tr|F1NIQ3|F1NIQ3\_CHICK tr|E1C3L0|E1C3L0\_CHICK tr|A2PYM3|A2PYM3\_CHICK tr|E1BWB9|E1BWB9\_CHICK tr|F1NSH4|F1NSH4\_CHICK tr|E1C9J1|E1C9J1\_CHICK tr|E1C1H1|E1C1H1\_CHICK sp|P79987|HIRA\_CHICK tr|Q5ZLU9|Q5ZLU9\_CHICK tr|F1NUI2|F1NUI2\_CHICK tr|R4GM21|R4GM21\_CHICK tr|Q98UC4|Q98UC4\_CHICK tr|F1NWG3|F1NWG3\_CHICK tr|F1P4G3|F1P4G3\_CHICK tr|Q5ZLV6|Q5ZLV6\_CHICK tr|E1BUQ4|E1BUQ4\_CHICK tr|F1NJW0|F1NJW0\_CHICK tr|F1P1Z1|F1P1Z1\_CHICK tr|B0I564|B0I564\_CHICK tr|F1NFU5|F1NFU5\_CHICK tr|F1P4F1|F1P4F1\_CHICK tr|V9GWB8|V9GWB8\_CHICK tr|E1BYS5|E1BYS5\_CHICK tr|Q5ZJF8|Q5ZJF8\_CHICK tr|F1NR19|F1NR19\_CHICK tr|F1P0Q9|F1P0Q9\_CHICK tr|E1C3V2|E1C3V2\_CHICK tr|E1BVU7|E1BVU7\_CHICK tr|F1P5W0|F1P5W0\_CHICK tr|E1C4R5|E1C4R5\_CHICK tr|E1BSU3|E1BSU3\_CHICK tr|E1C619|E1C619\_CHICK tr|Q8UVV4|Q8UVV4\_CHICK tr|F1NW82|F1NW82\_CHICK ENSEMBL:ENSBTAP00000024466 tr|Q5ZMD3|Q5ZMD3\_CHICK tr|Q90824|Q90824\_CHICK tr|F1NXL0|F1NXL0\_CHICK tr|F1NM37|F1NM37\_CHICK tr|F1NGI4|F1NGI4\_CHICK tr|E1C618|E1C618\_CHICK tr|Q91008|Q91008\_CHICK tr|F1P5N3|F1P5N3\_CHICK tr|H9KZX4|H9KZX4\_CHICK tr|R4GFY3|R4GFY3\_CHICK tr|E1BZI9|E1BZI9\_CHICK tr|F1NWC8|F1NWC8\_CHICK tr|E1C7I6|E1C7I6\_CHICK tr|E1BSG8|E1BSG8\_CHICK tr|Q90662|Q90662\_CHICK tr|F1NEV3|F1NEV3\_CHICK tr|E1BSG5|E1BSG5\_CHICK tr|E1C1C3|E1C1C3\_CHICK sp|Q2KN97|CYTSA\_CHICK tr|F1NS46|F1NS46\_CHICK tr|E1C4J2|E1C4J2\_CHICK tr|R4GLA8|R4GLA8\_CHICK tr|F1NJL0|F1NJL0\_CHICK sp|Q5ZJP5|FND3A\_CHICK sp|Q9DDD5|NBEA\_CHICK tr|F1NFV8|F1NFV8\_CHICK tr|F1NZF0|F1NZF0\_CHICK tr|F1P1Z6|F1P1Z6\_CHICK tr|F1NPK2|F1NPK2\_CHICK P12035 tr|F1P2R5|F1P2R5\_CHICK tr|F1NSS0|F1NSS0\_CHICK tr|F1NV82|F1NV82\_CHICK tr|Q8QG62|Q8QG62\_CHICK sp|Q5F364|MRP1\_CHICK tr|E1BUT3|E1BUT3\_CHICK tr|E1C5J4|E1C5J4\_CHICK tr|E1BXX9|E1BXX9\_CHICK tr|E1BVM2|E1BVM2\_CHICK tr|E1C5Y5|E1C5Y5\_CHICK tr|Q98940|Q98940\_CHICK tr|E1C314|E1C314\_CHICK tr|E1C683|E1C683\_CHICK tr|F1NQ88|F1NQ88\_CHICK tr|F1NFN9|F1NFN9\_CHICK tr|F1P511|F1P511\_CHICK tr|E1BV83|E1BV83\_CHICK tr|F1P544|F1P544\_CHICK tr|F1N900|F1N900\_CHICK tr|F1P209|F1P209\_CHICK tr|E1C588|E1C588\_CHICK tr|E1C2Q5|E1C2Q5\_CHICK tr|F1P5L2|F1P5L2\_CHICK tr|E1C4T3|E1C4T3\_CHICK Q7RTS7 tr|E1C2S0|E1C2S0\_CHICK sp|P05083|ARLY2\_CHICK tr|E1BU01|E1BU01\_CHICK tr|F1NSN2|F1NSN2\_CHICK tr|D5K9Y5|D5K9Y5\_CHICK tr|H2ESL7|H2ESL7\_CHICK tr|F1NYZ9|F1NYZ9\_CHICK tr|F1P1R2|F1P1R2\_CHICK tr|F1NEV1|F1NEV1\_CHICK tr|F1N850|F1N850\_CHICK tr|F1P0Z8|F1P0Z8\_CHICK tr|F1NU51|F1NU51\_CHICK tr|F1NQ24|F1NQ24\_CHICK tr|F1N968|F1N968\_CHICK P07744 tr|F1P0H9|F1P0H9\_CHICK tr|F1NKP6|F1NKP6\_CHICK tr|F1NHU1|F1NHU1\_CHICK tr|Q90831|Q90831\_CHICK tr|R4GMB2|R4GMB2\_CHICK tr|E1C6A1|E1C6A1\_CHICK tr|F1NJL2|F1NJL2\_CHICK tr|F1NW73|F1NW73\_CHICK tr|E1C7V7|E1C7V7\_CHICK tr|A0A0A0MQ52|A0A0A0MQ52\_CHICK sp|I0IUP4|MCM9\_CHICK tr|E1BQ59|E1BQ59\_CHICK tr|E1C680|E1C680\_CHICK tr|F1NFZ0|F1NFZ0\_CHICK tr|F1NP06|F1NP06\_CHICK tr|F1NGJ0|F1NGJ0\_CHICK tr|E1C326|E1C326\_CHICK tr|E1C9C2|E1C9C2\_CHICK tr|F1NPU4|F1NPU4\_CHICK tr|Q0PVE6|Q0PVE6\_CHICK tr|F1NDJ8|F1NDJ8\_CHICK tr|F1NG49|F1NG49\_CHICK sp|Q91009|NTRK1\_CHICK tr|F1NDF2|F1NDF2\_CHICK tr|Q5F417|Q5F417\_CHICK tr|F1NAT5|F1NAT5\_CHICK sp|Q0WYX8|MDGA1\_CHICK tr|F1NKX6|F1NKX6\_CHICK sp|Q5F3R2|KDM5B\_CHICK tr|E1C6Q9|E1C6Q9\_CHICK tr|Q7LZG7|Q7LZG7\_CHICK tr|F1NJE0|F1NJE0\_CHICK tr|F1NU77|F1NU77\_CHICK tr|Q5F475|Q5F475\_CHICK tr|F1NH88|F1NH88\_CHICK tr|E1BR04|E1BR04\_CHICK tr|A0M8U4|A0M8U4\_CHICK tr|F1P2S6|F1P2S6\_CHICK tr|F1NI02|F1NI02\_CHICK tr|F1NZ83|F1NZ83\_CHICK tr|Q90994|Q90994\_CHICK sp|Q9DDT2|BCAP\_CHICK sp|Q5F3V3|PDS5A\_CHICK tr|F1P168|F1P168\_CHICK tr|F1NK53|F1NK53\_CHICK tr|E1C544|E1C544\_CHICK tr|R9W6V6|R9W6V6\_CHICK tr|F1NWJ7|F1NWJ7\_CHICK tr|Q5F3K3|Q5F3K3\_CHICK tr|E1C8C2|E1C8C2\_CHICK tr|F1N9Y1|F1N9Y1\_CHICK Q1A7A4 tr|F1NDN9|F1NDN9\_CHICK P08727 tr|E1C5W2|E1C5W2\_CHICK tr|F1NNW0|F1NNW0\_CHICK sp|Q02173|MPSF\_CHICK sp|P30985|HTF4\_CHICK tr|F1N891|F1N891\_CHICK tr|E1C5P2|E1C5P2\_CHICK tr|H9KZY5|H9KZY5\_CHICK tr|F1P082|F1P082\_CHICK tr|E1C7C5|E1C7C5\_CHICK tr|F1P406|F1P406\_CHICK tr|F1NJP7|F1NJP7\_CHICK tr|E1C174|E1C174\_CHICK tr|F1NRB4|F1NRB4\_CHICK tr|F1NC67|F1NC67\_CHICK tr|F1NHD9|F1NHD9\_CHICK tr|Q5ZJ55|Q5ZJ55\_CHICK tr|E1C6S5|E1C6S5\_CHICK tr|A0A097QQS6|A0A097QQS6\_CHICK tr|F1NZ32|F1NZ32\_CHICK tr|E1BVZ6|E1BVZ6\_CHICK tr|F1N8E7|F1N8E7\_CHICK tr|F1P306|F1P306\_CHICK tr|F1P591|F1P591\_CHICK tr|F1NHS5|F1NHS5\_CHICK tr|E1BR01|E1BR01\_CHICK tr|Q90ZA0|Q90ZA0\_CHICK Q01546 tr|R4GJP0|R4GJP0\_CHICK tr|P79773|P79773\_CHICK tr|E1BSL8|E1BSL8\_CHICK tr|E1BU34|E1BU34\_CHICK tr|F1N9A5|F1N9A5\_CHICK tr|E1BWF8|E1BWF8\_CHICK tr|Q5F3X5|Q5F3X5\_CHICK tr|F1NPY6|F1NPY6\_CHICK tr|F1P5T1|F1P5T1\_CHICK tr|F1NDF6|F1NDF6\_CHICK tr|F1P3R1|F1P3R1\_CHICK tr|E1C5M1|E1C5M1\_CHICK tr|F1NUZ7|F1NUZ7\_CHICK tr|F1P5L4|F1P5L4\_CHICK tr|F1N993|F1N993\_CHICK tr|F1P1D8|F1P1D8\_CHICK tr|F1NAR6|F1NAR6\_CHICK tr|F1NAB9|F1NAB9\_CHICK tr|E1BZ07|E1BZ07\_CHICK tr|F1NG30|F1NG30\_CHICK tr|F1NG37|F1NG37\_CHICK tr|F1NFQ9|F1NFQ9\_CHICK tr|F1NBB4|F1NBB4\_CHICK tr|F1NGJ4|F1NGJ4\_CHICK tr|F1P3U7|F1P3U7\_CHICK tr|F1NN85|F1NN85\_CHICK tr|R4GHZ1|R4GHZ1\_CHICK tr|E1C5F9|E1C5F9\_CHICK ENSEMBL:ENSBTAP00000007350 sp|P32018|COEA1\_CHICK tr|F1N885|F1N885\_CHICK tr|E1BX02|E1BX02\_CHICK tr|Q5ZLU4|Q5ZLU4\_CHICK tr|F1P4P8|F1P4P8\_CHICK sp|Q8UUW7|KHDR1\_CHICK tr|E1C3A6|E1C3A6\_CHICK tr|E1BRE1|E1BRE1\_CHICK tr|E1BT63|E1BT63\_CHICK tr|E1C716|E1C716\_CHICK tr|F1NUV8|F1NUV8\_CHICK tr|E1BTK2|E1BTK2\_CHICK tr|A8QJ84|A8QJ84\_CHICK tr|F1NQ49|F1NQ49\_CHICK tr|F1NNL4|F1NNL4\_CHICK tr|F1P3X1|F1P3X1\_CHICK tr|W8CMH4|W8CMH4\_CHICK tr|F1N8A2|F1N8A2\_CHICK tr|E1BW99|E1BW99\_CHICK tr|F1NW61|F1NW61\_CHICK tr|F1NK10|F1NK10\_CHICK tr|F1NW38|F1NW38\_CHICK tr|O93419|O93419\_CHICK tr|Q5ZIL7|Q5ZIL7\_CHICK tr|F1N922|F1N922\_CHICK tr|F1P3X4|F1P3X4\_CHICK tr|Q5W9C2|Q5W9C2\_CHICK tr|Q5ZK91|Q5ZK91\_CHICK Q6IME9 tr|F1NE59|F1NE59\_CHICK tr|E1BTX8|E1BTX8\_CHICK tr|F1P1R1|F1P1R1\_CHICK tr|R4GFW8|R4GFW8\_CHICK tr|F1P3E9|F1P3E9\_CHICK sp|Q5ZMT0|1433E\_CHICK tr|E1BT29|E1BT29\_CHICK tr|E1BRA8|E1BRA8\_CHICK sp|P02467|CO1A2\_CHICK tr|F1P1B2|F1P1B2\_CHICK tr|F1NMQ6|F1NMQ6\_CHICK tr|F1NT34|F1NT34\_CHICK tr|Q9PTY2|Q9PTY2\_CHICK sp|Q5ZMD1|1433T\_CHICK tr|F1NCJ5|F1NCJ5\_CHICK tr|F1ND71|F1ND71\_CHICK tr|E1BY27|E1BY27\_CHICK tr|F1NB86|F1NB86\_CHICK tr|Q90ZM5|Q90ZM5\_CHICK tr|R4GME9|R4GME9\_CHICK tr|V9GVG9|V9GVG9\_CHICK tr|E1BWJ5|E1BWJ5\_CHICK tr|R4GGG3|R4GGG3\_CHICK tr|F1NMJ6|F1NMJ6\_CHICK tr|F1NS98|F1NS98\_CHICK tr|F1NKV4|F1NKV4\_CHICK tr|E1BX21|E1BX21\_CHICK tr|Q5F3X7|Q5F3X7\_CHICK tr|A7VMS1|A7VMS1\_CHICK tr|E1C066|E1C066\_CHICK tr|F1NNS4|F1NNS4\_CHICK tr|E1C738|E1C738\_CHICK tr|F1NGZ9|F1NGZ9\_CHICK tr|R4GK59|R4GK59\_CHICK sp|O93256|K1C19\_CHICK tr|F1P3I1|F1P3I1\_CHICK tr|E1C7G9|E1C7G9\_CHICK tr|F1NZV0|F1NZV0\_CHICK tr|F1P3M6|F1P3M6\_CHICK tr|A0A0A0MQ46|A0A0A0MQ46\_CHICK sp|P40618|HMGB3\_CHICK tr|Q5ZM05|Q5ZM05\_CHICK tr|E1C1R6|E1C1R6\_CHICK tr|Q5F396|Q5F396\_CHICK tr|Q9DEH3|Q9DEH3\_CHICK tr|Q6F4E7|Q6F4E7\_CHICK tr|F1NEH9|F1NEH9\_CHICK tr|F1NSR1|F1NSR1\_CHICK tr|F1P2U6|F1P2U6\_CHICK tr|F1P2P1|F1P2P1\_CHICK tr|Q9IBD4|Q9IBD4\_CHICK tr|F1NTV6|F1NTV6\_CHICK tr|F1NNU9|F1NNU9\_CHICK tr|F1NT99|F1NT99\_CHICK tr|F1N9F3|F1N9F3\_CHICK tr|M1ZMR1|M1ZMR1\_CHICK tr|E1BXJ2|E1BXJ2\_CHICK sp|Q5ZIE8|MCAF1\_CHICK tr|E1BQC5|E1BQC5\_CHICK tr|E1BXC4|E1BXC4\_CHICK sp|P13590|NCAM1\_CHICK tr|F1NLB3|F1NLB3\_CHICK tr|F1NT47|F1NT47\_CHICK tr|F1NZB0|F1NZB0\_CHICK tr|R4GH77|R4GH77\_CHICK tr|R4GJV5|R4GJV5\_CHICK tr|R4GFB2|R4GFB2\_CHICK tr|E1C1G0|E1C1G0\_CHICK tr|E1C7X2|E1C7X2\_CHICK tr|H9L107|H9L107\_CHICK tr|Q5F3P5|Q5F3P5\_CHICK tr|F1NLP9|F1NLP9\_CHICK tr|F1NW32|F1NW32\_CHICK tr|E1C8C8|E1C8C8\_CHICK tr|B0BL88|B0BL88\_CHICK tr|Q5ZKZ8|Q5ZKZ8\_CHICK tr|F1NLF2|F1NLF2\_CHICK tr|R4GJR1|R4GJR1\_CHICK tr|F1P3Q2|F1P3Q2\_CHICK tr|F1NDW6|F1NDW6\_CHICK tr|E1C947|E1C947\_CHICK tr|F1NS18|F1NS18\_CHICK tr|E1C5H0|E1C5H0\_CHICK tr|R4GKE7|R4GKE7\_CHICK tr|E1C7M2|E1C7M2\_CHICK tr|E1BXY5|E1BXY5\_CHICK tr|F1NCZ1|F1NCZ1\_CHICK sp|P12105|CO3A1\_CHICK tr|F1P5I4|F1P5I4\_CHICK tr|F1NAE4|F1NAE4\_CHICK tr|F1NWQ6|F1NWQ6\_CHICK tr|F1P204|F1P204\_CHICK tr|E1BQ98|E1BQ98\_CHICK tr|F1NLN5|F1NLN5\_CHICK tr|E1BZ13|E1BZ13\_CHICK tr|R4GLH9|R4GLH9\_CHICK tr|Q5ZMQ1|Q5ZMQ1\_CHICK tr|F1P3J3|F1P3J3\_CHICK tr|Q5ZI70|Q5ZI70\_CHICK sp|Q98UI9|MUC5B\_CHICK tr|F1P0Y0|F1P0Y0\_CHICK tr|E1BQ70|E1BQ70\_CHICK tr|E1C895|E1C895\_CHICK tr|Q98945|Q98945\_CHICK tr|Q90WG3|Q90WG3\_CHICK tr|E1C4S2|E1C4S2\_CHICK tr|E1C2U7|E1C2U7\_CHICK tr|F1P3V7|F1P3V7\_CHICK tr|F1P3U6|F1P3U6\_CHICK tr|F1NRH6|F1NRH6\_CHICK tr|F1NYG9|F1NYG9\_CHICK sp|Q90693|PTC1\_CHICK tr|F1NUX4|F1NUX4\_CHICK tr|Q8QFQ7|Q8QFQ7\_CHICK tr|F1NN90|F1NN90\_CHICK tr|Q8JG72|Q8JG72\_CHICK tr|F1NU79|F1NU79\_CHICK tr|E1BXY6|E1BXY6\_CHICK tr|Q9PWP8|Q9PWP8\_CHICK sp|Q5ZI08|SPT5H\_CHICK tr|H9L0H1|H9L0H1\_CHICK tr|F1P334|F1P334\_CHICK tr|F1P3E3|F1P3E3\_CHICK P01030 tr|Q5F3M1|Q5F3M1\_CHICK tr|R4GLR6|R4GLR6\_CHICK tr|A7XMT9|A7XMT9\_CHICK tr|F1P108|F1P108\_CHICK tr|F1NHJ6|F1NHJ6\_CHICK tr|E1BXB7|E1BXB7\_CHICK tr|F1NUK0|F1NUK0\_CHICK sp|Q6YI48|PTPRU\_CHICK sp|P02565|MYH3\_CHICK tr|F1NH24|F1NH24\_CHICK tr|F1NCE0|F1NCE0\_CHICK tr|E1C355|E1C355\_CHICK tr|F1P2E9|F1P2E9\_CHICK sp|P31696|AGRIN\_CHICK tr|F1NH75|F1NH75\_CHICK tr|F1NUH3|F1NUH3\_CHICK tr|E1BWL4|E1BWL4\_CHICK tr|F1N8F2|F1N8F2\_CHICK tr|F1NC16|F1NC16\_CHICK sp|Q5ZLQ6|1433B\_CHICK tr|Q5ZI17|Q5ZI17\_CHICK tr|F1NUT3|F1NUT3\_CHICK sp|Q5ZMV2|SAS6\_CHICK sp|P11799|MYLK\_CHICK tr|F1NE68|F1NE68\_CHICK tr|F1P199|F1P199\_CHICK tr|W0S6J0|W0S6J0\_CHICK sp|E1C1R4|UBP47\_CHICK tr|R9PXM3|R9PXM3\_CHICK tr|R4GJH4|R4GJH4\_CHICK tr|Q70IK3|Q70IK3\_CHICK tr|E1BW97|E1BW97\_CHICK tr|E1C5N0|E1C5N0\_CHICK tr|E1BVT1|E1BVT1\_CHICK tr|Q6R0H8|Q6R0H8\_CHICK tr|R4GHN9|R4GHN9\_CHICK sp|Q5F3P8|SET1B\_CHICK tr|E1C1K1|E1C1K1\_CHICK sp|Q5ZLC8|ANR52\_CHICK tr|H9L0G9|H9L0G9\_CHICK tr|F1P164|F1P164\_CHICK sp|P35458|DCTN1\_CHICK tr|F1NQ78|F1NQ78\_CHICK tr|Q90YB6|Q90YB6\_CHICK sp|P08941|ROS1\_CHICK tr|F1NQL9|F1NQL9\_CHICK tr|E1BV84|E1BV84\_CHICK tr|F1NBS2|F1NBS2\_CHICK tr|E1C9H0|E1C9H0\_CHICK tr|F1NN09|F1NN09\_CHICK tr|E1BQD9|E1BQD9\_CHICK tr|F1NK61|F1NK61\_CHICK tr|Q90699|Q90699\_CHICK tr|H9KZZ1|H9KZZ1\_CHICK tr|F1P4P9|F1P4P9\_CHICK sp|O42184|CLIP1\_CHICK tr|R9PXL8|R9PXL8\_CHICK sp|Q679P3|PDLI7\_CHICK tr|E1C5V6|E1C5V6\_CHICK tr|F1NBQ4|F1NBQ4\_CHICK tr|E1C0L6|E1C0L6\_CHICK tr|F1NFR0|F1NFR0\_CHICK tr|E1C6G0|E1C6G0\_CHICK tr|B5BV17|B5BV17\_CHICK tr|F1NME8|F1NME8\_CHICK tr|R4GIU7|R4GIU7\_CHICK sp|Q5ZIX8|KDM3A\_CHICK tr|F1NJM8|F1NJM8\_CHICK tr|F1NLS8|F1NLS8\_CHICK tr|F1NXA4|F1NXA4\_CHICK tr|Q800D9|Q800D9\_CHICK tr|E1C3X1|E1C3X1\_CHICK tr|F1NK39|F1NK39\_CHICK tr|R4GKY9|R4GKY9\_CHICK tr|F1P160|F1P160\_CHICK tr|R4GF29|R4GF29\_CHICK tr|F1NYH9|F1NYH9\_CHICK tr|A0SVH2|A0SVH2\_CHICK tr|F1NSK6|F1NSK6\_CHICK tr|F1P1F4|F1P1F4\_CHICK sp|Q5ZI74|DHX30\_CHICK tr|E1BXR8|E1BXR8\_CHICK tr|Q8UVQ9|Q8UVQ9\_CHICK tr|Q8UVR0|Q8UVR0\_CHICK ENSEMBL:ENSBTAP00000024462 tr|F1N8G9|F1N8G9\_CHICK sp|Q5F3W6|1433G\_CHICK tr|F6T4R2|F6T4R2\_CHICK tr|F1NKZ3|F1NKZ3\_CHICK tr|E1BQC3|E1BQC3\_CHICK tr|F1ND22|F1ND22\_CHICK tr|F1NRR2|F1NRR2\_CHICK sp|Q5F478|ANR44\_CHICK sp|Q8QHL3|VGFR1\_CHICK sp|Q5ZIH2|MON1A\_CHICK tr|F1NGD8|F1NGD8\_CHICK sp|P15143|PO2F1\_CHICK tr|F1NQV5|F1NQV5\_CHICK tr|F1NXA1|F1NXA1\_CHICK tr|A7XMV7|A7XMV7\_CHICK sp|Q2VB19|PUM1\_CHICK tr|F1NN22|F1NN22\_CHICK tr|F1NI29|F1NI29\_CHICK tr|Q76EY8|Q76EY8\_CHICK tr|H9L003|H9L003\_CHICK tr|R4GGE3|R4GGE3\_CHICK sp|Q5ZIP4|XRN2\_CHICK tr|R4GKM6|R4GKM6\_CHICK tr|E1C4C8|E1C4C8\_CHICK tr|R4GFS3|R4GFS3\_CHICK tr|F1NWY2|F1NWY2\_CHICK tr|A7XMU9|A7XMU9\_CHICK tr|E1BSA7|E1BSA7\_CHICK tr|Q98TD2|Q98TD2\_CHICK tr|E1C5R8|E1C5R8\_CHICK Q2M2I5 tr|F1NZW1|F1NZW1\_CHICK tr|E1BU81|E1BU81\_CHICK tr|E1C1D2|E1C1D2\_CHICK tr|F1NF99|F1NF99\_CHICK tr|E1C898|E1C898\_CHICK tr|E1C533|E1C533\_CHICK tr|X5D5B1|X5D5B1\_CHICK tr|R4GJ27|R4GJ27\_CHICK tr|A0A0A0MQ45|A0A0A0MQ45\_CHICK sp|E1C2I2|GWL\_CHICK sp|Q04205|TENS\_CHICK tr|F1NTZ8|F1NTZ8\_CHICK tr|A0A0A8J816|A0A0A8J816\_CHICK tr|E1BSN2|E1BSN2\_CHICK tr|R4GIG1|R4GIG1\_CHICK tr|F1P2D8|F1P2D8\_CHICK tr|F1P0H0|F1P0H0\_CHICK sp|Q5ZIB2|FBF1\_CHICK tr|E1C8N3|E1C8N3\_CHICK tr|F1P384|F1P384\_CHICK tr|Q5W9C4|Q5W9C4\_CHICK tr|R4GLV5|R4GLV5\_CHICK tr|F1P1T9|F1P1T9\_CHICK tr|R4GJQ2|R4GJQ2\_CHICK tr|F1N9Z5|F1N9Z5\_CHICK tr|Q5ZLQ1|Q5ZLQ1\_CHICK tr|Q8UWA0|Q8UWA0\_CHICK tr|F1P5P9|F1P5P9\_CHICK tr|U5MXI9|U5MXI9\_CHICK tr|E1BW44|E1BW44\_CHICK tr|F1P491|F1P491\_CHICK tr|F1N9K4|F1N9K4\_CHICK Q9H552 tr|F1P1X4|F1P1X4\_CHICK tr|F1P1Z0|F1P1Z0\_CHICK sp|Q5ZKU4|INT2\_CHICK tr|F1NWT4|F1NWT4\_CHICK tr|B9A0U5|B9A0U5\_CHICK tr|F1NM49|F1NM49\_CHICK tr|F1NIL2|F1NIL2\_CHICK sp|Q04861|NFKB1\_CHICK tr|F1NZY2|F1NZY2\_CHICK tr|F1NFC7|F1NFC7\_CHICK tr|R4MNY4|R4MNY4\_CHICK tr|Q5F3N8|Q5F3N8\_CHICK tr|R4GKC8|R4GKC8\_CHICK tr|F1NVP5|F1NVP5\_CHICK tr|F1P0W2|F1P0W2\_CHICK tr|E1BWE6|E1BWE6\_CHICK tr|F1NNI2|F1NNI2\_CHICK tr|E1BZI6|E1BZI6\_CHICK tr|F1N8N7|F1N8N7\_CHICK sp|P07228|ITB1\_CHICK tr|F1P3Y9|F1P3Y9\_CHICK tr|E1BSP7|E1BSP7\_CHICK tr|R4GLP2|R4GLP2\_CHICK tr|Q3T8Z6|Q3T8Z6\_CHICK tr|E1BSX5|E1BSX5\_CHICK tr|F1NBG3|F1NBG3\_CHICK sp|Q05858|FMN\_CHICK tr|F1P0Y8|F1P0Y8\_CHICK tr|Q5ZL14|Q5ZL14\_CHICK sp|Q5ZJK5|LCORL\_CHICK tr|R4GLI7|R4GLI7\_CHICK tr|E1BZL3|E1BZL3\_CHICK tr|E1ANH5|E1ANH5\_CHICK tr|E1BZB5|E1BZB5\_CHICK tr|E1BYA8|E1BYA8\_CHICK tr|F1ND57|F1ND57\_CHICK O76015 tr|E1C523|E1C523\_CHICK tr|F1NIQ5|F1NIQ5\_CHICK tr|F1P1N0|F1P1N0\_CHICK tr|F1P3B8|F1P3B8\_CHICK sp|Q5F3U9|PDS5B\_CHICK tr|F1NHB4|F1NHB4\_CHICK tr|R9PXQ1|R9PXQ1\_CHICK sp|Q7LZR2|CO8A1\_CHICK tr|F1NIE3|F1NIE3\_CHICK tr|F1N897|F1N897\_CHICK tr|F1NAR1|F1NAR1\_CHICK sp|Q98917|PMEL\_CHICK tr|E1BYP8|E1BYP8\_CHICK tr|F1NB85|F1NB85\_CHICK tr|R4GJH8|R4GJH8\_CHICK tr|F1ND51|F1ND51\_CHICK tr|F1NIJ4|F1NIJ4\_CHICK tr|F1P5K4|F1P5K4\_CHICK tr|F1NEP9|F1NEP9\_CHICK tr|E1C7J4|E1C7J4\_CHICK tr|F1NNR0|F1NNR0\_CHICK tr|F1P1Y1|F1P1Y1\_CHICK tr|Q6F4E8|Q6F4E8\_CHICK tr|F1P5C5|F1P5C5\_CHICK tr|H9L041|H9L041\_CHICK tr|F1NAU5|F1NAU5\_CHICK tr|F1NE80|F1NE80\_CHICK sp|Q5ZJ43|EXOC8\_CHICK tr|F1NQW4|F1NQW4\_CHICK tr|E1C1Z0|E1C1Z0\_CHICK tr|F1P049|F1P049\_CHICK tr|E1C764|E1C764\_CHICK tr|F1NY55|F1NY55\_CHICK tr|F1P4H0|F1P4H0\_CHICK sp|Q9DDD0|NRX1A\_CHICK tr|R4GLY2|R4GLY2\_CHICK tr|R4GJD3|R4GJD3\_CHICK tr|Q38HX7|Q38HX7\_CHICK tr|H9L0P9|H9L0P9\_CHICK tr|F1NVA3|F1NVA3\_CHICK tr|E1C867|E1C867\_CHICK tr|E1C3M6|E1C3M6\_CHICK tr|F1NIP3|F1NIP3\_CHICK tr|Q68Y81|Q68Y81\_CHICK tr|E1BXC5|E1BXC5\_CHICK sp|Q5F371|SBNO1\_CHICK tr|E1C3E9|E1C3E9\_CHICK tr|F1NGF8|F1NGF8\_CHICK tr|F1NNU8|F1NNU8\_CHICK tr|R4GGP7|R4GGP7\_CHICK tr|F1P0I1|F1P0I1\_CHICK tr|H9L063|H9L063\_CHICK sp|Q5F3M0|MED24\_CHICK tr|F1NJY8|F1NJY8\_CHICK tr|F1N9I4|F1N9I4\_CHICK tr|R4GHG1|R4GHG1\_CHICK sp|Q5ZKC9|1433Z\_CHICK tr|F1NHH3|F1NHH3\_CHICK tr|F1NGC9|F1NGC9\_CHICK tr|F1NG55|F1NG55\_CHICK tr|E1BVY1|E1BVY1\_CHICK tr|F1P3T9|F1P3T9\_CHICK tr|F1NLU3|F1NLU3\_CHICK tr|E1BY83|E1BY83\_CHICK sp|Q9I8D1|MYO6\_CHICK tr|F1NWJ9|F1NWJ9\_CHICK tr|Q5ZKY5|Q5ZKY5\_CHICK tr|E1C7L7|E1C7L7\_CHICK tr|D8MIU8|D8MIU8\_CHICK tr|F1P1V3|F1P1V3\_CHICK tr|F1NXA0|F1NXA0\_CHICK tr|F1P2R3|F1P2R3\_CHICK tr|A3FB57|A3FB57\_CHICK tr|F1NZF7|F1NZF7\_CHICK tr|E1C2I6|E1C2I6\_CHICK tr|F1NEY4|F1NEY4\_CHICK tr|E1C4G7|E1C4G7\_CHICK tr|F1NT11|F1NT11\_CHICK tr|Q9W7F9|Q9W7F9\_CHICK tr|F1NA35|F1NA35\_CHICK tr|E1C7M6|E1C7M6\_CHICK tr|Q5F3Q4|Q5F3Q4\_CHICK tr|F1NGZ3|F1NGZ3\_CHICK tr|F1NRI5|F1NRI5\_CHICK sp|Q8QGQ8|PER2\_CHICK tr|F1NGH5|F1NGH5\_CHICK tr|F1NEW8|F1NEW8\_CHICK tr|F1P0V6|F1P0V6\_CHICK tr|E1C5R9|E1C5R9\_CHICK tr|R4GME5|R4GME5\_CHICK tr|E1C8P9|E1C8P9\_CHICK tr|Q9PVI6|Q9PVI6\_CHICK tr|F1P140|F1P140\_CHICK tr|F1NFA8|F1NFA8\_CHICK tr|F1NSQ9|F1NSQ9\_CHICK tr|Q8SPD1|Q8SPD1\_CHICK tr|A0FKN5|A0FKN5\_CHICK tr|B3TZC0|B3TZC0\_CHICK tr|F1P3V5|F1P3V5\_CHICK tr|E1BUY1|E1BUY1\_CHICK sp|Q9W6V5|PTPRJ\_CHICK tr|F1NP22|F1NP22\_CHICK tr|F1N8M2|F1N8M2\_CHICK tr|F1NPH3|F1NPH3\_CHICK tr|E1BW51|E1BW51\_CHICK tr|F1N9S4|F1N9S4\_CHICK tr|E1BZ33|E1BZ33\_CHICK tr|F1NJ23|F1NJ23\_CHICK tr|F1NDQ5|F1NDQ5\_CHICK tr|A1KXK7|A1KXK7\_CHICK tr|Q2QB50|Q2QB50\_CHICK tr|O57484|O57484\_CHICK tr|Q910C5|Q910C5\_CHICK tr|F1NMA5|F1NMA5\_CHICK tr|F1P3V1|F1P3V1\_CHICK tr|F1NEV6|F1NEV6\_CHICK tr|Q9IAI1|Q9IAI1\_CHICK tr|F1NPP8|F1NPP8\_CHICK tr|F1P4F7|F1P4F7\_CHICK tr|E1BX69|E1BX69\_CHICK tr|F1P0J4|F1P0J4\_CHICK tr|F1NNH5|F1NNH5\_CHICK tr|E6N1W0|E6N1W0\_CHICK tr|E1BUY6|E1BUY6\_CHICK tr|Q5F441|Q5F441\_CHICK tr|F1NF33|F1NF33\_CHICK tr|F1NFA4|F1NFA4\_CHICK tr|E1BYV3|E1BYV3\_CHICK tr|E1BYU4|E1BYU4\_CHICK tr|Q8AWB7|Q8AWB7\_CHICK tr|E1C2C3|E1C2C3\_CHICK sp|Q90610|NEO1\_CHICK tr|Q5F454|Q5F454\_CHICK tr|F1P1T5|F1P1T5\_CHICK tr|F1NNV4|F1NNV4\_CHICK tr|F1NPB2|F1NPB2\_CHICK tr|E1C906|E1C906\_CHICK tr|F1P3K3|F1P3K3\_CHICK tr|F1P382|F1P382\_CHICK tr|Q2THW3|Q2THW3\_CHICK tr|F1NNI6|F1NNI6\_CHICK tr|F1NF98|F1NF98\_CHICK tr|E1BQJ4|E1BQJ4\_CHICK tr|A0A0E4AX31|A0A0E4AX31\_CHICK tr|A0A0E4BA89|A0A0E4BA89\_CHICK tr|F1NQB6|F1NQB6\_CHICK sp|Q02440|MYO5A\_CHICK tr|F1NYL7|F1NYL7\_CHICK tr|F1P5V1|F1P5V1\_CHICK tr|F1NLY0|F1NLY0\_CHICK tr|R4GG78|R4GG78\_CHICK tr|F1P2Y7|F1P2Y7\_CHICK tr|Q5F462|Q5F462\_CHICK tr|Q5ZLD0|Q5ZLD0\_CHICK tr|H9KYT0|H9KYT0\_CHICK tr|F1NJM6|F1NJM6\_CHICK tr|F1NDP0|F1NDP0\_CHICK sp|Q6PVZ1|K1C14\_CHICK tr|E1BXA7|E1BXA7\_CHICK tr|F1P543|F1P543\_CHICK tr|F1NZS2|F1NZS2\_CHICK tr|Q5ZK79|Q5ZK79\_CHICK tr|E1C625|E1C625\_CHICK tr|R4GMG2|R4GMG2\_CHICK tr|F1NX72|F1NX72\_CHICK tr|F1NP59|F1NP59\_CHICK tr|R4GLM2|R4GLM2\_CHICK tr|E1BVB2|E1BVB2\_CHICK tr|F1NY33|F1NY33\_CHICK tr|R4GF89|R4GF89\_CHICK tr|E1C0R8|E1C0R8\_CHICK tr|F1N9M1|F1N9M1\_CHICK tr|F1NE84|F1NE84\_CHICK tr|C1KB84|C1KB84\_CHICK tr|F1NSG1|F1NSG1\_CHICK tr|F1NF70|F1NF70\_CHICK tr|F1NKM7|F1NKM7\_CHICK tr|R4GJ09|R4GJ09\_CHICK tr|Q70IK2|Q70IK2\_CHICK tr|F1NTK2|F1NTK2\_CHICK tr|R4GJV1|R4GJV1\_CHICK tr|F1NP23|F1NP23\_CHICK tr|F1NDZ4|F1NDZ4\_CHICK tr|E1BRT3|E1BRT3\_CHICK tr|F1NVB3|F1NVB3\_CHICK tr|E1C8A3|E1C8A3\_CHICK tr|Q6RD80|Q6RD80\_CHICK tr|E1C8B7|E1C8B7\_CHICK tr|R9PXP2|R9PXP2\_CHICK sp|E1C3P4|CBPC1\_CHICK tr|F1NKX8|F1NKX8\_CHICK tr|E1BYB2|E1BYB2\_CHICK tr|E1BQ86|E1BQ86\_CHICK tr|F1NF14|F1NF14\_CHICK tr|E1C264|E1C264\_CHICK tr|Q5ZM53|Q5ZM53\_CHICK tr|F1NVP2|F1NVP2\_CHICK sp|Q98930|SORL\_CHICK tr|E1BX07|E1BX07\_CHICK tr|F1NNZ8|F1NNZ8\_CHICK Q9Z2K1 Q3ZAW8 tr|F1NLI2|F1NLI2\_CHICK tr|F1NB51|F1NB51\_CHICK sp|P36197|ZEB1\_CHICK tr|F1P2R4|F1P2R4\_CHICK P15636 tr|F1P5H8|F1P5H8\_CHICK tr|F1NNQ0|F1NNQ0\_CHICK tr|H9L2B7|H9L2B7\_CHICK tr|H9KZD4|H9KZD4\_CHICK tr|F1NV18|F1NV18\_CHICK tr|D3KR64|D3KR64\_CHICK tr|F1NFT7|F1NFT7\_CHICK tr|F1NRP6|F1NRP6\_CHICK tr|R4GIC8|R4GIC8\_CHICK tr|R4GFF8|R4GFF8\_CHICK sp|Q5F3W3|MK06\_CHICK tr|E1BYN5|E1BYN5\_CHICK tr|Q6F4E5|Q6F4E5\_CHICK tr|F1NSZ5|F1NSZ5\_CHICK tr|R4GH74|R4GH74\_CHICK tr|E1C2F7|E1C2F7\_CHICK tr|E1BZI1|E1BZI1\_CHICK tr|F1NLT1|F1NLT1\_CHICK tr|F1P0Z6|F1P0Z6\_CHICK tr|F1P1D5|F1P1D5\_CHICK tr|Q6PVZ4|Q6PVZ4\_CHICK tr|F1ND06|F1ND06\_CHICK tr|H9KZ26|H9KZ26\_CHICK tr|B4ZBA7|B4ZBA7\_CHICK tr|F1NHC7|F1NHC7\_CHICK tr|E1C0J4|E1C0J4\_CHICK tr|E1BQ78|E1BQ78\_CHICK tr|F1P1W1|F1P1W1\_CHICK tr|F1NSP6|F1NSP6\_CHICK tr|F1NXW4|F1NXW4\_CHICK tr|F1NMV4|F1NMV4\_CHICK sp|Q5ZJT0|SUV3\_CHICK tr|F1NLV6|F1NLV6\_CHICK tr|Q5GR16|Q5GR16\_CHICK tr|Q9PWB9|Q9PWB9\_CHICK tr|Q5F3C2|Q5F3C2\_CHICK tr|F1N8X8|F1N8X8\_CHICK tr|E1C1B9|E1C1B9\_CHICK tr|E1C853|E1C853\_CHICK tr|E1C2W8|E1C2W8\_CHICK tr|E1BXI7|E1BXI7\_CHICK tr|V9GW30|V9GW30\_CHICK tr|A0A0E4B8N5|A0A0E4B8N5\_CHICK tr|A0A0E4B9S2|A0A0E4B9S2\_CHICK tr|E1BWE9|E1BWE9\_CHICK tr|H9L3J8|H9L3J8\_CHICK tr|F1NMI2|F1NMI2\_CHICK tr|F1P2Q3|F1P2Q3\_CHICK tr|Q6R0I5|Q6R0I5\_CHICK tr|E1BUN2|E1BUN2\_CHICK tr|E1BUN1|E1BUN1\_CHICK tr|F1N9U0|F1N9U0\_CHICK tr|U5MY31|U5MY31\_CHICK tr|F1P3W8|F1P3W8\_CHICK tr|R4GHI7|R4GHI7\_CHICK tr|F1NHL2|F1NHL2\_CHICK tr|F1NAB4|F1NAB4\_CHICK tr|R4GJD4|R4GJD4\_CHICK tr|F1NPT2|F1NPT2\_CHICK tr|F1NSC2|F1NSC2\_CHICK tr|E1C343|E1C343\_CHICK tr|E1BUG4|E1BUG4\_CHICK tr|F1NK88|F1NK88\_CHICK tr|F1NBE4|F1NBE4\_CHICK tr|R4GHK5|R4GHK5\_CHICK tr|E1C3M1|E1C3M1\_CHICK tr|R4GJB9|R4GJB9\_CHICK tr|O42094|O42094\_CHICK tr|E1BWR4|E1BWR4\_CHICK tr|F1P1B5|F1P1B5\_CHICK tr|Q9DGM5|Q9DGM5\_CHICK tr|H9KZZ5|H9KZZ5\_CHICK tr|R4GJ14|R4GJ14\_CHICK tr|F9W2X9|F9W2X9\_CHICK tr|F1NVE5|F1NVE5\_CHICK tr|F1P4E4|F1P4E4\_CHICK tr|E1C9J6|E1C9J6\_CHICK tr|Q5ZKF9|Q5ZKF9\_CHICK tr|Q5ZMS0|Q5ZMS0\_CHICK tr|B0Z662|B0Z662\_CHICK tr|F1NQY0|F1NQY0\_CHICK tr|F1NX73|F1NX73\_CHICK tr|F1NVC3|F1NVC3\_CHICK sp|Q6XUX0|DUSTY\_CHICK tr|E1BU62|E1BU62\_CHICK sp|E1BY77|UBP13\_CHICK tr|E1BRZ2|E1BRZ2\_CHICK tr|E1BXZ0|E1BXZ0\_CHICK tr|F1N8X3|F1N8X3\_CHICK tr|E1C8P0|E1C8P0\_CHICK tr|E1BWE0|E1BWE0\_CHICK sp|E1C2Z0|MMS22\_CHICK tr|F1P3W6|F1P3W6\_CHICK tr|F1NYF3|F1NYF3\_CHICK tr|R4GL98|R4GL98\_CHICK sp|Q5ZK92|SPAST\_CHICK tr|E1C2A9|E1C2A9\_CHICK tr|E1C5L6|E1C5L6\_CHICK tr|F1NCE3|F1NCE3\_CHICK tr|E1BUD8|E1BUD8\_CHICK tr|I6LDD9|I6LDD9\_CHICK tr|E1BZ18|E1BZ18\_CHICK tr|F1NPT5|F1NPT5\_CHICK tr|E1C1B5|E1C1B5\_CHICK tr|E1BTB6|E1BTB6\_CHICK tr|F1NEZ8|F1NEZ8\_CHICK tr|F1N8Z0|F1N8Z0\_CHICK tr|R4GFE8|R4GFE8\_CHICK tr|E1C0R2|E1C0R2\_CHICK tr|Q5F3Y7|Q5F3Y7\_CHICK tr|E1BZU6|E1BZU6\_CHICK tr|E1C4T7|E1C4T7\_CHICK tr|Q5W9B8|Q5W9B8\_CHICK sp|Q92072|DNMT1\_CHICK tr|F1NWV6|F1NWV6\_CHICK tr|E1C5I7|E1C5I7\_CHICK tr|Q2PGG8|Q2PGG8\_CHICK tr|F1NQ63|F1NQ63\_CHICK tr|F1P421|F1P421\_CHICK tr|F1NF29|F1NF29\_CHICK tr|F1NV85|F1NV85\_CHICK tr|E1BX05|E1BX05\_CHICK tr|E1C849|E1C849\_CHICK tr|F1NH89|F1NH89\_CHICK tr|F1P2Z2|F1P2Z2\_CHICK tr|E1C4H5|E1C4H5\_CHICK tr|R4GK70|R4GK70\_CHICK tr|F1NUS6|F1NUS6\_CHICK tr|E1BRS7|E1BRS7\_CHICK sp|Q5ZJJ2|RFA1\_CHICK tr|F1NAS4|F1NAS4\_CHICK tr|E1C0J0|E1C0J0\_CHICK tr|F1NZ50|F1NZ50\_CHICK tr|Q800L4|Q800L4\_CHICK tr|E1BZ39|E1BZ39\_CHICK tr|F1P0F0|F1P0F0\_CHICK tr|R4GF50|R4GF50\_CHICK tr|M1XH40|M1XH40\_CHICK tr|Q5ZHM2|Q5ZHM2\_CHICK tr|E1C009|E1C009\_CHICK tr|F1NWS5|F1NWS5\_CHICK tr|Q5F3X9|Q5F3X9\_CHICK tr|R4GIY0|R4GIY0\_CHICK sp|Q5ZIM6|AATF\_CHICK tr|Q98SN6|Q98SN6\_CHICK sp|P28693|EPHB2\_CHICK tr|F1NAF9|F1NAF9\_CHICK tr|M9PNX5|M9PNX5\_CHICK tr|O93437|O93437\_CHICK tr|E1C0I2|E1C0I2\_CHICK tr|F1NDQ2|F1NDQ2\_CHICK tr|F1NBV9|F1NBV9\_CHICK tr|F1NKW3|F1NKW3\_CHICK tr|E1C422|E1C422\_CHICK tr|E1BYQ3|E1BYQ3\_CHICK tr|F1NH99|F1NH99\_CHICK tr|H9L0L9|H9L0L9\_CHICK tr|F1NME9|F1NME9\_CHICK tr|R4GH95|R4GH95\_CHICK tr|Q6R0I8|Q6R0I8\_CHICK tr|Q5F3B3|Q5F3B3\_CHICK tr|E1C0V6|E1C0V6\_CHICK tr|R4GIH5|R4GIH5\_CHICK sp|Q5F418|PSMD1\_CHICK tr|I3VQH4|I3VQH4\_CHICK tr|R4GFP9|R4GFP9\_CHICK tr|F1NYC4|F1NYC4\_CHICK sp|Q5W4S4|FNIP1\_CHICK tr|F1NQH6|F1NQH6\_CHICK tr|F1NGK5|F1NGK5\_CHICK tr|Q5F473|Q5F473\_CHICK tr|Q2EJ72|Q2EJ72\_CHICK tr|I1W6S8|I1W6S8\_CHICK tr|F1NFI4|F1NFI4\_CHICK tr|E1C3D6|E1C3D6\_CHICK tr|E1C311|E1C311\_CHICK tr|Q90868|Q90868\_CHICK tr|F1P1W8|F1P1W8\_CHICK tr|Q5F481|Q5F481\_CHICK tr|C4PCL8|C4PCL8\_CHICK tr|C4PCJ6|C4PCJ6\_CHICK tr|C4PCJ4|C4PCJ4\_GALLA tr|C4PCJ9|C4PCJ9\_CHICK tr|B1NRY4|B1NRY4\_CHICK tr|G1CBQ6|G1CBQ6\_CHICK tr|C4PCK0|C4PCK0\_CHICK tr|C4PCL0|C4PCL0\_CHICK tr|C4PCK3|C4PCK3\_CHICK tr|C4PCK5|C4PCK5\_CHICK tr|R4GF75|R4GF75\_CHICK Q28194 tr|F1NXN8|F1NXN8\_CHICK tr|Q5ZJ57|Q5ZJ57\_CHICK sp|O57604|PODXL\_CHICK tr|F1NXC1|F1NXC1\_CHICK tr|F1NSJ4|F1NSJ4\_CHICK tr|E1BZ32|E1BZ32\_CHICK tr|E1C0M0|E1C0M0\_CHICK tr|F1NDB4|F1NDB4\_CHICK sp|P02521|ARLY1\_CHICK tr|Q5ZI71|Q5ZI71\_CHICK tr|F1NMQ0|F1NMQ0\_CHICK tr|F1NQH4|F1NQH4\_CHICK tr|Q98SN3|Q98SN3\_CHICK tr|F1NFM8|F1NFM8\_CHICK tr|Q5ZKT5|Q5ZKT5\_CHICK tr|E1BQR2|E1BQR2\_CHICK tr|F1P4H7|F1P4H7\_CHICK tr|E1C9D4|E1C9D4\_CHICK tr|R4GJE2|R4GJE2\_CHICK tr|F1NY56|F1NY56\_CHICK tr|F1NX04|F1NX04\_CHICK tr|F1NL90|F1NL90\_CHICK tr|V9GWR3|V9GWR3\_CHICK tr|F1NKC1|F1NKC1\_CHICK tr|E1BZQ5|E1BZQ5\_CHICK tr|E1BV10|E1BV10\_CHICK tr|F1NUU5|F1NUU5\_CHICK tr|R4GL60|R4GL60\_CHICK tr|F1NJF6|F1NJF6\_CHICK tr|A0A0A0MQ35|A0A0A0MQ35\_CHICK sp|E1BZR9|TCPR1\_CHICK sp|O42131|TOP2B\_CHICK tr|Q5ZL85|Q5ZL85\_CHICK tr|E1C9E1|E1C9E1\_CHICK tr|F1P1W6|F1P1W6\_CHICK tr|E1BS84|E1BS84\_CHICK sp|P08284|H101\_CHICK tr|E1C692|E1C692\_CHICK tr|F1NQT3|F1NQT3\_CHICK tr|D3X752|D3X752\_CHICK tr|F1P3I3|F1P3I3\_CHICK tr|Q6R0I9|Q6R0I9\_CHICK tr|Q5ZKU2|Q5ZKU2\_CHICK tr|R9PXM1|R9PXM1\_CHICK tr|R4GIM7|R4GIM7\_CHICK tr|F1NKU9|F1NKU9\_CHICK tr|E1BRB5|E1BRB5\_CHICK tr|Q5F3X6|Q5F3X6\_CHICK tr|F1NWV9|F1NWV9\_CHICK tr|E1BUB5|E1BUB5\_CHICK tr|E1C6X5|E1C6X5\_CHICK sp|Q5ZM71|SP20H\_CHICK tr|E1BRZ7|E1BRZ7\_CHICK tr|R4GLF8|R4GLF8\_CHICK tr|E1C8W6|E1C8W6\_CHICK tr|X2C5I8|X2C5I8\_CHICK tr|F1P480|F1P480\_CHICK tr|R4GFM0|R4GFM0\_CHICK tr|E1C7S5|E1C7S5\_CHICK tr|F1NK34|F1NK34\_CHICK tr|Q6TLV9|Q6TLV9\_CHICK sp|Q8AYD0|CADH5\_CHICK tr|F1NEZ6|F1NEZ6\_CHICK tr|F1NC06|F1NC06\_CHICK tr|F1P1P3|F1P1P3\_CHICK sp|P16053|NFM\_CHICK tr|F1NML1|F1NML1\_CHICK sp|Q589G5|PRTG\_CHICK tr|K7S5D8|K7S5D8\_CHICK tr|E1BVF6|E1BVF6\_CHICK sp|Q90640|KIF4\_CHICK tr|F1P1M8|F1P1M8\_CHICK sp|Q5F363|JARD2\_CHICK tr|F1NKN5|F1NKN5\_CHICK tr|F1P1G6|F1P1G6\_CHICK tr|R4GIP8|R4GIP8\_CHICK tr|Q9YHV2|Q9YHV2\_CHICK tr|E1C6E1|E1C6E1\_CHICK tr|F1NI53|F1NI53\_CHICK tr|Q8AYP8|Q8AYP8\_CHICK tr|F1NL76|F1NL76\_CHICK tr|R4GKD4|R4GKD4\_CHICK tr|F1NLE6|F1NLE6\_CHICK tr|F1N9P5|F1N9P5\_CHICK tr|F1NMH4|F1NMH4\_CHICK tr|F1NN52|F1NN52\_CHICK tr|F1NBA0|F1NBA0\_CHICK tr|E1BU51|E1BU51\_CHICK tr|E1C7M1|E1C7M1\_CHICK tr|E1C0J6|E1C0J6\_CHICK tr|F1NF95|F1NF95\_CHICK tr|F1NG56|F1NG56\_CHICK tr|E1C633|E1C633\_CHICK tr|G1K325|G1K325\_CHICK tr|E1BQQ4|E1BQQ4\_CHICK tr|R4GLW9|R4GLW9\_CHICK tr|F1P586|F1P586\_CHICK tr|S5ZB27|S5ZB27\_CHICK tr|F1NI10|F1NI10\_CHICK sp|Q5ZMS4|NCOA7\_CHICK tr|E1C4E1|E1C4E1\_CHICK tr|H9L073|H9L073\_CHICK tr|E1BRK2|E1BRK2\_CHICK tr|E1C1B6|E1C1B6\_CHICK tr|F1P587|F1P587\_CHICK tr|F1P1I1|F1P1I1\_CHICK sp|Q5ZJ75|SL9A8\_CHICK tr|E1BWZ7|E1BWZ7\_CHICK tr|E1C6N5|E1C6N5\_CHICK tr|E1BYY9|E1BYY9\_CHICK tr|F1P0C5|F1P0C5\_CHICK tr|D3X736|D3X736\_CHICK tr|E1C0R6|E1C0R6\_CHICK tr|R4GJ21|R4GJ21\_CHICK tr|F1NX58|F1NX58\_CHICK tr|F1NU86|F1NU86\_CHICK tr|Q7T2Z7|Q7T2Z7\_CHICK tr|F1NIH6|F1NIH6\_CHICK tr|E1C7P2|E1C7P2\_CHICK tr|F1NY74|F1NY74\_CHICK tr|E1C929|E1C929\_CHICK tr|Q90633|Q90633\_CHICK tr|F1N938|F1N938\_CHICK sp|A6N7Y9|PIWL1\_CHICK tr|E1C582|E1C582\_CHICK tr|E1C5G4|E1C5G4\_CHICK tr|R4GFL8|R4GFL8\_CHICK tr|F1NV36|F1NV36\_CHICK sp|Q5ZJ69|ARI5B\_CHICK tr|E1C473|E1C473\_CHICK tr|Q6R0I0|Q6R0I0\_CHICK tr|E1C784|E1C784\_CHICK tr|H9L251|H9L251\_CHICK tr|F1NLV8|F1NLV8\_CHICK tr|F1NCM3|F1NCM3\_CHICK sp|P13538|MYSS\_CHICK tr|Q5QIE2|Q5QIE2\_CHICK tr|F1P257|F1P257\_CHICK tr|Q677M1|Q677M1\_CHICK tr|F1N8Z5|F1N8Z5\_CHICK tr|Q6JGT6|Q6JGT6\_CHICK tr|E1BUI3|E1BUI3\_CHICK Q95121 tr|F1P1Z2|F1P1Z2\_CHICK Q5XKE5 tr|E1C9K0|E1C9K0\_CHICK tr|Q8QHJ0|Q8QHJ0\_CHICK sp|P11722|FINC\_CHICK tr|E1C8X0|E1C8X0\_CHICK tr|E1C799|E1C799\_CHICK tr|Q71SG2|Q71SG2\_CHICK tr|F1NIU4|F1NIU4\_CHICK tr|F1P0E2|F1P0E2\_CHICK tr|F1N8N8|F1N8N8\_CHICK tr|F1N8W8|F1N8W8\_CHICK tr|R4GFZ3|R4GFZ3\_CHICK tr|F1NVG5|F1NVG5\_CHICK tr|F1NKR1|F1NKR1\_CHICK tr|Q5ZI47|Q5ZI47\_CHICK tr|E1BU88|E1BU88\_CHICK sp|P08286|H110\_CHICK tr|F1NF96|F1NF96\_CHICK tr|F1NJZ7|F1NJZ7\_CHICK sp|P14105|MYH9\_CHICK tr|F1NCD4|F1NCD4\_CHICK tr|F1NUK7|F1NUK7\_CHICK tr|E1BRR1|E1BRR1\_CHICK tr|R4GMB0|R4GMB0\_CHICK tr|F1NEH4|F1NEH4\_CHICK tr|E1BXD9|E1BXD9\_CHICK tr|E1BTY6|E1BTY6\_CHICK tr|F1NWZ0|F1NWZ0\_CHICK tr|F1NNK0|F1NNK0\_CHICK tr|E1C5B8|E1C5B8\_CHICK tr|H9KZP5|H9KZP5\_CHICK tr|F1NIK1|F1NIK1\_CHICK tr|F1NU99|F1NU99\_CHICK tr|E1BQE4|E1BQE4\_CHICK tr|E1C830|E1C830\_CHICK tr|F1NB13|F1NB13\_CHICK tr|F1NNS7|F1NNS7\_CHICK tr|E1BWJ3|E1BWJ3\_CHICK tr|F1NSJ3|F1NSJ3\_CHICK tr|Q90WR9|Q90WR9\_CHICK tr|F1P1V9|F1P1V9\_CHICK tr|R4GMK5|R4GMK5\_CHICK tr|Q5GR02|Q5GR02\_CHICK tr|Q90W37|Q90W37\_CHICK tr|F1NVX6|F1NVX6\_CHICK tr|F1NPR7|F1NPR7\_CHICK tr|Q8AWB8|Q8AWB8\_CHICK tr|E1BRG6|E1BRG6\_CHICK tr|Q5ZIK5|Q5ZIK5\_CHICK tr|F6S174|F6S174\_CHICK tr|E1C4L8|E1C4L8\_CHICK tr|E1BQP5|E1BQP5\_CHICK tr|Q52PF5|Q52PF5\_CHICK tr|F1NWL9|F1NWL9\_CHICK tr|F1NEQ5|F1NEQ5\_CHICK tr|F1P2A5|F1P2A5\_CHICK tr|F1NB60|F1NB60\_CHICK tr|F1NMA4|F1NMA4\_CHICK tr|E1BRP5|E1BRP5\_CHICK tr|F1NY14|F1NY14\_CHICK tr|R4GFD0|R4GFD0\_CHICK tr|Q6PVY9|Q6PVY9\_CHICK tr|Q5ZKJ2|Q5ZKJ2\_CHICK tr|F1NID9|F1NID9\_CHICK tr|F1NE49|F1NE49\_CHICK tr|F1NPG6|F1NPG6\_CHICK tr|H9L0N3|H9L0N3\_CHICK tr|F1NJD5|F1NJD5\_CHICK tr|F1P136|F1P136\_CHICK tr|F1NSW4|F1NSW4\_CHICK tr|M1X8W0|M1X8W0\_CHICK tr|F1NAX7|F1NAX7\_CHICK tr|E1BYH2|E1BYH2\_CHICK tr|V9GWB6|V9GWB6\_CHICK sp|Q91048|PTK7\_CHICK tr|Q6F4E6|Q6F4E6\_CHICK tr|H9KZC9|H9KZC9\_CHICK tr|F1NFH6|F1NFH6\_CHICK tr|F1P0K3|F1P0K3\_CHICK tr|U5TSZ8|U5TSZ8\_CHICK tr|E1BUT2|E1BUT2\_CHICK tr|F1NWI1|F1NWI1\_CHICK tr|F1P326|F1P326\_CHICK tr|Q5Y1E8|Q5Y1E8\_CHICK tr|F1P3F8|F1P3F8\_CHICK tr|H9L044|H9L044\_CHICK tr|F1N8P0|F1N8P0\_CHICK tr|F1NB57|F1NB57\_CHICK tr|F1NQL2|F1NQL2\_CHICK tr|E1C7Y0|E1C7Y0\_CHICK tr|E1BW96|E1BW96\_CHICK tr|E1C287|E1C287\_CHICK sp|Q8QFV0|KCNT1\_CHICK tr|E1BW18|E1BW18\_CHICK tr|F1NY63|F1NY63\_CHICK tr|F1NYR2|F1NYR2\_CHICK tr|F1NDK7|F1NDK7\_CHICK tr|Q5ZI81|Q5ZI81\_CHICK tr|Q5ZIC7|Q5ZIC7\_CHICK tr|E1BXV5|E1BXV5\_CHICK tr|D2XV92|D2XV92\_CHICK tr|E1C2H5|E1C2H5\_CHICK tr|E1BTH6|E1BTH6\_CHICK tr|R4GK67|R4GK67\_CHICK sp|Q8QGQ6|CLOCK\_CHICK tr|F1NKI8|F1NKI8\_CHICK tr|Q5W9C3|Q5W9C3\_CHICK tr|E1BZ87|E1BZ87\_CHICK tr|F1NKB0|F1NKB0\_CHICK tr|E1BR72|E1BR72\_CHICK tr|F1NZJ2|F1NZJ2\_CHICK tr|F1P0N6|F1P0N6\_CHICK tr|E1BZE8|E1BZE8\_CHICK sp|Q9DDD1|GP149\_CHICK tr|F1NN41|F1NN41\_CHICK tr|F1NRL3|F1NRL3\_CHICK tr|G1CBQ1|G1CBQ1\_CHICK tr|F1NWZ8|F1NWZ8\_CHICK tr|Q9I9E2|Q9I9E2\_CHICK tr|F1NLX8|F1NLX8\_CHICK tr|F1P5F2|F1P5F2\_CHICK tr|R4GJ34|R4GJ34\_CHICK tr|E1BQG0|E1BQG0\_CHICK tr|F1NWQ8|F1NWQ8\_CHICK tr|Q5U7A2|Q5U7A2\_CHICK tr|F1NH67|F1NH67\_CHICK tr|E1BSS3|E1BSS3\_CHICK sp|P08106|HSP70\_CHICK tr|F1NCG4|F1NCG4\_CHICK tr|F1NTL3|F1NTL3\_CHICK sp|P06212|ESR1\_CHICK tr|E5L8C9|E5L8C9\_CHICK tr|F1NNT0|F1NNT0\_CHICK tr|Q6R0I3|Q6R0I3\_CHICK tr|F1NBV2|F1NBV2\_CHICK tr|F1NY66|F1NY66\_CHICK tr|F1N8I5|F1N8I5\_CHICK tr|Q90Z51|Q90Z51\_CHICK tr|F1NLS1|F1NLS1\_CHICK sp|Q8JIR8|IMPG1\_CHICK tr|Q5ZKL1|Q5ZKL1\_CHICK tr|D3X739|D3X739\_CHICK tr|Q5F498|Q5F498\_CHICK tr|E1C0Z9|E1C0Z9\_CHICK tr|F1NZV4|F1NZV4\_CHICK tr|F1NPH8|F1NPH8\_CHICK tr|F1P1N2|F1P1N2\_CHICK sp|Q5ZM13|HYCCI\_CHICK tr|F1NB26|F1NB26\_CHICK tr|Q95593|Q95593\_CHICK tr|A0ZXM5|A0ZXM5\_CHICK tr|E1C235|E1C235\_CHICK tr|E1C5B6|E1C5B6\_CHICK tr|E1BSQ0|E1BSQ0\_CHICK tr|F1NIU3|F1NIU3\_CHICK tr|F1NCM6|F1NCM6\_CHICK tr|E1BWM9|E1BWM9\_CHICK tr|E1BZT5|E1BZT5\_CHICK tr|F1NQ87|F1NQ87\_CHICK tr|Q4ZJ82|Q4ZJ82\_CHICK tr|F1ND12|F1ND12\_CHICK tr|F1NR75|F1NR75\_CHICK P05784 tr|F1NLV9|F1NLV9\_CHICK tr|D3X742|D3X742\_CHICK tr|E1C199|E1C199\_CHICK tr|H2D5D8|H2D5D8\_CHICK tr|Q9GIP6|Q9GIP6\_CHICK tr|Q5ZLQ8|Q5ZLQ8\_CHICK tr|A0A024B7I3|A0A024B7I3\_CHICK sp|Q5F3U0|SP130\_CHICK tr|Q08476|Q08476\_CHICK tr|H9KZN9|H9KZN9\_CHICK sp|P18460|FGFR3\_CHICK tr|F1P3S0|F1P3S0\_CHICK tr|F1NC82|F1NC82\_CHICK sp|Q9PUF6|PGFRA\_CHICK tr|Q9PU52|Q9PU52\_CHICK tr|F1NZP1|F1NZP1\_CHICK sp|Q07498|EPHB3\_CHICK tr|F1NIP0|F1NIP0\_CHICK sp|Q5ZLK7|HYOU1\_CHICK tr|E1C8Z2|E1C8Z2\_CHICK tr|Q6R0I7|Q6R0I7\_CHICK tr|F1ND41|F1ND41\_CHICK tr|F6RCM2|F6RCM2\_CHICK sp|F1NSM7|OC116\_CHICK tr|Q6R0I4|Q6R0I4\_CHICK sp|P21265|PUR8\_CHICK tr|E1BR00|E1BR00\_CHICK tr|Q5U7A3|Q5U7A3\_CHICK tr|Q683M9|Q683M9\_CHICK tr|E1BY47|E1BY47\_CHICK tr|E1C9F5|E1C9F5\_CHICK tr|F1NMM7|F1NMM7\_CHICK tr|R4GK99|R4GK99\_CHICK tr|Q5F3H1|Q5F3H1\_CHICK tr|Q5W9C6|Q5W9C6\_CHICK tr|F1NU30|F1NU30\_CHICK tr|E1C1F9|E1C1F9\_CHICK tr|Q5F3V2|Q5F3V2\_CHICK tr|F1P269|F1P269\_CHICK tr|E1C330|E1C330\_CHICK tr|F1NHL1|F1NHL1\_CHICK Q29RQ1 tr|R4GGM7|R4GGM7\_CHICK tr|Q9DFS3|Q9DFS3\_CHICK tr|F1NXT8|F1NXT8\_CHICK sp|Q7T2Z5|UNC5C\_CHICK tr|E1BWN2|E1BWN2\_CHICK Q32PI4 sp|Q5F3X8|SC31A\_CHICK tr|R4GI80|R4GI80\_CHICK tr|F1NGT2|F1NGT2\_CHICK tr|F1NSA6|F1NSA6\_CHICK sp|P10587|MYH11\_CHICK tr|E1C182|E1C182\_CHICK tr|E1C7S2|E1C7S2\_CHICK tr|F1P2X8|F1P2X8\_CHICK tr|F1NPQ6|F1NPQ6\_CHICK tr|F1P1T6|F1P1T6\_CHICK tr|E1C5U0|E1C5U0\_CHICK tr|F1NE81|F1NE81\_CHICK tr|E1BXX4|E1BXX4\_CHICK tr|Q9PUM6|Q9PUM6\_CHICK tr|Q9PW47|Q9PW47\_CHICK tr|F1NMF3|F1NMF3\_CHICK tr|R4GFP0|R4GFP0\_CHICK tr|E1C270|E1C270\_CHICK tr|D3X751|D3X751\_CHICK tr|D3X748|D3X748\_CHICK tr|E1BZ19|E1BZ19\_CHICK tr|F1P0G8|F1P0G8\_CHICK tr|R4GFG5|R4GFG5\_CHICK tr|F1NS82|F1NS82\_CHICK tr|F1NQR2|F1NQR2\_CHICK tr|F1NKU2|F1NKU2\_CHICK tr|F1NI81|F1NI81\_CHICK tr|F1NG39|F1NG39\_CHICK sp|Q5ZLA6|MYO1C\_CHICK tr|H9KZZ2|H9KZZ2\_CHICK tr|F1NIW4|F1NIW4\_CHICK tr|Q5ZK50|Q5ZK50\_CHICK tr|E1C133|E1C133\_CHICK tr|F1NMJ9|F1NMJ9\_CHICK tr|Q7SX63|Q7SX63\_CHICK tr|F1NK09|F1NK09\_CHICK sp|P47990|XDH\_CHICK tr|E1C459|E1C459\_CHICK tr|F1NXD4|F1NXD4\_CHICK tr|E1C4H4|E1C4H4\_CHICK tr|R4GM98|R4GM98\_CHICK tr|R4GGE2|R4GGE2\_CHICK tr|F1NJD8|F1NJD8\_CHICK tr|F1NIC1|F1NIC1\_CHICK Q14CN4-1 tr|F1NQ55|F1NQ55\_CHICK tr|F1NHW2|F1NHW2\_CHICK tr|F1NCP8|F1NCP8\_CHICK tr|E1BVC2|E1BVC2\_CHICK sp|Q5ZIA5|COPB\_CHICK tr|R4GIN0|R4GIN0\_CHICK sp|Q98949|TYRO3\_CHICK tr|E5DEH8|E5DEH8\_CHICK tr|F1NP27|F1NP27\_CHICK tr|F1N8K8|F1N8K8\_CHICK tr|F1NP25|F1NP25\_CHICK tr|F1NHG1|F1NHG1\_CHICK tr|F1NK47|F1NK47\_CHICK tr|E1BXA0|E1BXA0\_CHICK tr|E1BUA1|E1BUA1\_CHICK tr|F1NLV7|F1NLV7\_CHICK tr|E1BVQ9|E1BVQ9\_CHICK tr|F1NAG1|F1NAG1\_CHICK tr|Q5F494|Q5F494\_CHICK tr|E1BY40|E1BY40\_CHICK sp|P20740|OVOS\_CHICK tr|F1NZ57|F1NZ57\_CHICK sp|F1NBT0|STK10\_CHICK tr|Q5ZM75|Q5ZM75\_CHICK tr|F1NMV7|F1NMV7\_CHICK tr|H9KZW4|H9KZW4\_CHICK tr|F1NMK7|F1NMK7\_CHICK tr|F1NY90|F1NY90\_CHICK tr|E1BRJ9|E1BRJ9\_CHICK tr|F1NVR3|F1NVR3\_CHICK tr|F1P1C9|F1P1C9\_CHICK tr|Q5ZM80|Q5ZM80\_CHICK tr|R4GJG7|R4GJG7\_CHICK tr|Q31412|Q31412\_CHICK tr|F1CN11|F1CN11\_CHICK sp|Q2LK54|COL12\_CHICK sp|Q07497|EPHB5\_CHICK tr|E1C7L8|E1C7L8\_CHICK tr|E1C5J7|E1C5J7\_CHICK tr|F1P119|F1P119\_CHICK tr|F1NZC1|F1NZC1\_CHICK sp|B6ZLK2|CHD1\_CHICK tr|Q9DGM4|Q9DGM4\_CHICK tr|E1BVG8|E1BVG8\_CHICK tr|F1NIY2|F1NIY2\_CHICK tr|F1NH05|F1NH05\_CHICK tr|F1NV34|F1NV34\_CHICK tr|Q90947|Q90947\_CHICK tr|O42391|O42391\_CHICK tr|E1C485|E1C485\_CHICK tr|F1NQL7|F1NQL7\_CHICK tr|F1NZH0|F1NZH0\_CHICK tr|F1P589|F1P589\_CHICK tr|R4GJL2|R4GJL2\_CHICK tr|F1P1A9|F1P1A9\_CHICK tr|F1NUJ0|F1NUJ0\_CHICK tr|Q5ZJG6|Q5ZJG6\_CHICK tr|R4GKA6|R4GKA6\_CHICK tr|E1C3R4|E1C3R4\_CHICK tr|E1BR11|E1BR11\_CHICK tr|E1C832|E1C832\_CHICK tr|O13129|O13129\_CHICK tr|R4GLV8|R4GLV8\_CHICK tr|Q5F3M9|Q5F3M9\_CHICK tr|F1ND09|F1ND09\_CHICK tr|F1NLE0|F1NLE0\_CHICK tr|Q7ZT63|Q7ZT63\_CHICK tr|F6RX73|F6RX73\_CHICK tr|F1NKB6|F1NKB6\_CHICK tr|E1C2R8|E1C2R8\_CHICK tr|F1NY18|F1NY18\_CHICK tr|D8UWD9|D8UWD9\_CHICK tr|F1NXB9|F1NXB9\_CHICK tr|Q2HZD7|Q2HZD7\_CHICK tr|E1BVQ5|E1BVQ5\_CHICK tr|R4GG74|R4GG74\_CHICK tr|H9L2H3|H9L2H3\_CHICK tr|E1C807|E1C807\_CHICK tr|H9KZS4|H9KZS4\_CHICK tr|F1NCD2|F1NCD2\_CHICK tr|F1NHR4|F1NHR4\_CHICK tr|E1BUG1|E1BUG1\_CHICK sp|Q03696|NGCA\_CHICK tr|E1BWB2|E1BWB2\_CHICK tr|E1C0V8|E1C0V8\_CHICK tr|E1BW85|E1BW85\_CHICK tr|E1C8R7|E1C8R7\_CHICK tr|F1NUN7|F1NUN7\_CHICK tr|E1C067|E1C067\_CHICK tr|Q5ZM17|Q5ZM17\_CHICK tr|F1NGM0|F1NGM0\_CHICK tr|E1C7W8|E1C7W8\_CHICK tr|F1NHR3|F1NHR3\_CHICK tr|Q5ZL93|Q5ZL93\_CHICK tr|Q5QHR9|Q5QHR9\_CHICK tr|D3X740|D3X740\_CHICK tr|D3X744|D3X744\_CHICK tr|D3X738|D3X738\_CHICK tr|D3X737|D3X737\_CHICK tr|D3X741|D3X741\_CHICK tr|F1N870|F1N870\_CHICK tr|F1NLU5|F1NLU5\_CHICK tr|F1P0S1|F1P0S1\_CHICK tr|F1NH90|F1NH90\_CHICK tr|F1NJ36|F1NJ36\_CHICK tr|F1NES6|F1NES6\_CHICK tr|F6UZC5|F6UZC5\_CHICK tr|Q9PTI7|Q9PTI7\_CHICK P08729 tr|F1ND93|F1ND93\_CHICK tr|Q5ZL97|Q5ZL97\_CHICK tr|E1BUB1|E1BUB1\_CHICK tr|E1BWG9|E1BWG9\_CHICK tr|F1NY34|F1NY34\_CHICK tr|E1C6P8|E1C6P8\_CHICK tr|E5L3Q1|E5L3Q1\_CHICK tr|F1DS93|F1DS93\_CHICK tr|F8VBC0|F8VBC0\_CHICK tr|C4PCL1|C4PCL1\_CHICK tr|E1BY80|E1BY80\_CHICK tr|Q5F3J4|Q5F3J4\_CHICK tr|M1S0Z2|M1S0Z2\_CHICK tr|M1SQS2|M1SQS2\_CHICK tr|F1NES4|F1NES4\_CHICK tr|A0M8T9|A0M8T9\_CHICK tr|F1P040|F1P040\_CHICK tr|F1NDN6|F1NDN6\_CHICK tr|F1NX14|F1NX14\_CHICK tr|F1N9L2|F1N9L2\_CHICK tr|E1C8Z0|E1C8Z0\_CHICK tr|R4GHL7|R4GHL7\_CHICK tr|R4GMK3|R4GMK3\_CHICK tr|F1NYQ8|F1NYQ8\_CHICK tr|F1P4K9|F1P4K9\_CHICK tr|F1P381|F1P381\_CHICK tr|F1NI94|F1NI94\_CHICK tr|Q2HPJ9|Q2HPJ9\_CHICK tr|Q5ZJU1|Q5ZJU1\_CHICK tr|F1NVD6|F1NVD6\_CHICK tr|F1NEL3|F1NEL3\_CHICK tr|E1BQJ5|E1BQJ5\_CHICK tr|F1NPJ6|F1NPJ6\_CHICK tr|E1C6X8|E1C6X8\_CHICK tr|E1BVW9|E1BVW9\_CHICK tr|F1NPY8|F1NPY8\_CHICK tr|Q90755|Q90755\_CHICK tr|R4GH47|R4GH47\_CHICK tr|F1NAV3|F1NAV3\_CHICK tr|F1NV96|F1NV96\_CHICK tr|F1P3V0|F1P3V0\_CHICK tr|Q90777|Q90777\_CHICK tr|F1NCE7|F1NCE7\_CHICK tr|E1C838|E1C838\_CHICK tr|F1NWC5|F1NWC5\_CHICK tr|Q537V4|Q537V4\_CHICK tr|Q5F3S4|Q5F3S4\_CHICK tr|F1P2H3|F1P2H3\_CHICK tr|H9L060|H9L060\_CHICK tr|Q5ZHP1|Q5ZHP1\_CHICK tr|E1C0M5|E1C0M5\_CHICK tr|E1C1V2|E1C1V2\_CHICK tr|Q6EI13|Q6EI13\_CHICK tr|F1NJB8|F1NJB8\_CHICK tr|F1NYY6|F1NYY6\_CHICK tr|F1P5I1|F1P5I1\_CHICK tr|F1NPP1|F1NPP1\_CHICK tr|Q90ZT8|Q90ZT8\_CHICK tr|E1BR42|E1BR42\_CHICK tr|C4PCL5|C4PCL5\_CHICK tr|Q5F3Y2|Q5F3Y2\_CHICK tr|E1C3U0|E1C3U0\_CHICK tr|E1BUW6|E1BUW6\_CHICK tr|E1C3L1|E1C3L1\_CHICK tr|F1NF23|F1NF23\_CHICK tr|E1BSI5|E1BSI5\_CHICK tr|Q5ZID8|Q5ZID8\_CHICK tr|F1P1E3|F1P1E3\_CHICK sp|Q6R748|STXB1\_CHICK tr|F1NS75|F1NS75\_CHICK tr|F1P4D2|F1P4D2\_CHICK sp|Q5ZIV5|PDC10\_CHICK Q7Z3Y9 tr|F1NE82|F1NE82\_CHICK tr|F1P2F7|F1P2F7\_CHICK tr|B3VHV2|B3VHV2\_CHICK tr|F1NQ36|F1NQ36\_CHICK tr|D3X747|D3X747\_CHICK tr|F1NYQ5|F1NYQ5\_CHICK tr|D3X749|D3X749\_CHICK tr|D3X750|D3X750\_CHICK sp|Q8AYS8|KCMA1\_CHICK sp|Q90688|MYPC3\_CHICK tr|E1BV07|E1BV07\_CHICK tr|E1BYQ4|E1BYQ4\_CHICK tr|E1C2N3|E1C2N3\_CHICK tr|F1NQ61|F1NQ61\_CHICK sp|Q5ZL91|INT7\_CHICK tr|F1NPA4|F1NPA4\_CHICK sp|Q1G7G9|TALD3\_CHICK tr|F1NME1|F1NME1\_CHICK sp|P08288|H11R\_CHICK tr|E1C1N6|E1C1N6\_CHICK tr|F1P445|F1P445\_CHICK tr|E1C0W8|E1C0W8\_CHICK tr|Q90XG4|Q90XG4\_CHICK tr|B3VE14|B3VE14\_CHICK tr|F1NVW3|F1NVW3\_CHICK tr|F1N9C3|F1N9C3\_CHICK tr|R4GF99|R4GF99\_CHICK tr|R4GGI3|R4GGI3\_CHICK tr|F1NN89|F1NN89\_CHICK tr|B8ZX71|B8ZX71\_CHICK tr|H9KZI8|H9KZI8\_CHICK tr|R4GMD6|R4GMD6\_CHICK tr|Q8QGU9|Q8QGU9\_CHICK tr|H9L024|H9L024\_CHICK tr|R4GH45|R4GH45\_CHICK sp|Q5ZI43|SPN1\_CHICK tr|R4GJI7|R4GJI7\_CHICK tr|Q90975|Q90975\_CHICK tr|F1NA71|F1NA71\_CHICK sp|Q90623|MYPT1\_CHICK tr|E1C5D5|E1C5D5\_CHICK tr|E1BRH4|E1BRH4\_CHICK tr|R4GGE1|R4GGE1\_CHICK tr|F1ND48|F1ND48\_CHICK tr|A1YKW0|A1YKW0\_CHICK sp|Q98936|PTPRG\_CHICK tr|E1C4E2|E1C4E2\_CHICK tr|F1NH65|F1NH65\_CHICK sp|Q5ZKK5|ODFP2\_CHICK tr|E1BQQ6|E1BQQ6\_CHICK tr|F1NN17|F1NN17\_CHICK tr|E1BQA7|E1BQA7\_CHICK tr|F1NE09|F1NE09\_CHICK tr|Q8JHF6|Q8JHF6\_CHICK tr|R4GLL4|R4GLL4\_CHICK sp|P10288|CADH2\_CHICK tr|R4GL02|R4GL02\_CHICK tr|F1NF88|F1NF88\_CHICK tr|E1BXX0|E1BXX0\_CHICK tr|F1NK32|F1NK32\_CHICK tr|F1P3J9|F1P3J9\_CHICK tr|E1BYZ3|E1BYZ3\_CHICK tr|F1NIV1|F1NIV1\_CHICK tr|F1NS99|F1NS99\_CHICK tr|E1C0G2|E1C0G2\_CHICK tr|R4GG13|R4GG13\_CHICK tr|Q5F3Q2|Q5F3Q2\_CHICK tr|E1C7F3|E1C7F3\_CHICK tr|Q5ZLE8|Q5ZLE8\_CHICK tr|F1NZ69|F1NZ69\_CHICK tr|R4GIS8|R4GIS8\_CHICK sp|Q5ZKG3|DNA2\_CHICK tr|F1NUT6|F1NUT6\_CHICK tr|E1C0M8|E1C0M8\_CHICK tr|B3TZB3|B3TZB3\_CHICK tr|Q6PPB4|Q6PPB4\_CHICK tr|F1NT68|F1NT68\_CHICK tr|F1NW30|F1NW30\_CHICK tr|F1P4I8|F1P4I8\_CHICK sp|Q805F9|DDB1\_CHICK tr|F1NP93|F1NP93\_CHICK tr|R4GLX7|R4GLX7\_CHICK sp|Q90964|FOXG1\_CHICK tr|E1C6S3|E1C6S3\_CHICK tr|F1NWA7|F1NWA7\_CHICK tr|E1BSP2|E1BSP2\_CHICK tr|O57660|O57660\_CHICK sp|Q5ZQU2|NPAS2\_CHICK tr|R4GGZ8|R4GGZ8\_CHICK tr|R4GJS6|R4GJS6\_CHICK tr|H9KZT6|H9KZT6\_CHICK tr|R4GJC5|R4GJC5\_CHICK tr|R4GHM7|R4GHM7\_CHICK tr|E1BRJ4|E1BRJ4\_CHICK tr|E1BXN6|E1BXN6\_CHICK tr|Q5F374|Q5F374\_CHICK sp|P00523|SRC\_CHICK tr|Q9PWM9|Q9PWM9\_CHICK tr|E1BS15|E1BS15\_CHICK tr|F1NWV5|F1NWV5\_CHICK tr|Q4W5Z3|Q4W5Z3\_CHICK tr|F1NYJ6|F1NYJ6\_CHICK tr|H9L0A9|H9L0A9\_CHICK tr|F1NHL4|F1NHL4\_CHICK tr|E1C3Y5|E1C3Y5\_CHICK tr|F1NDF5|F1NDF5\_CHICK tr|F1NAC9|F1NAC9\_CHICK tr|E1BVD2|E1BVD2\_CHICK tr|F1N9A3|F1N9A3\_CHICK tr|E1BYX3|E1BYX3\_CHICK tr|E1BZ23|E1BZ23\_CHICK tr|F1P2L3|F1P2L3\_CHICK tr|E1BYP0|E1BYP0\_CHICK tr|Q9PUM4|Q9PUM4\_CHICK tr|Q9PW45|Q9PW45\_CHICK tr|M9QWS9|M9QWS9\_CHICK tr|A8DC32|A8DC32\_CHICK tr|E1C5J0|E1C5J0\_CHICK tr|Q98TT7|Q98TT7\_CHICK tr|F1NPI8|F1NPI8\_CHICK tr|C7ECT7|C7ECT7\_CHICK tr|F1NIS8|F1NIS8\_CHICK tr|F1NVZ5|F1NVZ5\_CHICK tr|F1NS70|F1NS70\_CHICK tr|E1BQ45|E1BQ45\_CHICK tr|Q5ZJ59|Q5ZJ59\_CHICK tr|E1C5Q1|E1C5Q1\_CHICK tr|E1BUN5|E1BUN5\_CHICK tr|E1C6K8|E1C6K8\_CHICK sp|P35331|NRCAM\_CHICK tr|E1BQR7|E1BQR7\_CHICK tr|F1NMZ1|F1NMZ1\_CHICK tr|F1CN58|F1CN58\_CHICK tr|I6LPK9|I6LPK9\_CHICK tr|E1BQK9|E1BQK9\_CHICK tr|E1BX04|E1BX04\_CHICK tr|B5BSL8|B5BSL8\_CHICK sp|Q5ZL12|PPR21\_CHICK tr|E1C6P5|E1C6P5\_CHICK tr|R4GFK3|R4GFK3\_CHICK tr|C4PC55|C4PC55\_GALLA tr|C4PC75|C4PC75\_GALVA tr|H2D5E0|H2D5E0\_CHICK tr|C4PC57|C4PC57\_CHICK tr|C4PC77|C4PC77\_GALSO sp|Q9DD78|TLR21\_CHICK tr|C4PC78|C4PC78\_GALSO tr|C4PC58|C4PC58\_CHICK tr|C4PC98|C4PC98\_CHICK tr|F1NYS9|F1NYS9\_CHICK tr|F1NPN9|F1NPN9\_CHICK tr|R4GKA5|R4GKA5\_CHICK tr|E1BTU9|E1BTU9\_CHICK tr|F1P4J6|F1P4J6\_CHICK tr|E1C0T6|E1C0T6\_CHICK sp|Q5ZK44|F172A\_CHICK tr|Q5F3G8|Q5F3G8\_CHICK tr|G0ZS69|G0ZS69\_CHICK tr|F1P5X5|F1P5X5\_CHICK tr|E1BQX6|E1BQX6\_CHICK tr|F1NHH2|F1NHH2\_CHICK tr|F1NUT7|F1NUT7\_CHICK tr|F1NII7|F1NII7\_CHICK tr|H9L0H9|H9L0H9\_CHICK tr|G0ZS67|G0ZS67\_CHICK sp|P15988|CO6A2\_CHICK tr|F1P592|F1P592\_CHICK tr|F1N8X1|F1N8X1\_CHICK tr|Q8AYP7|Q8AYP7\_CHICK tr|F1NUG4|F1NUG4\_CHICK tr|F1NGA2|F1NGA2\_CHICK tr|F1NTZ9|F1NTZ9\_CHICK tr|F1NGL9|F1NGL9\_CHICK tr|F1N9E7|F1N9E7\_CHICK tr|E1BVI9|E1BVI9\_CHICK tr|E1BR79|E1BR79\_CHICK tr|F1NVC0|F1NVC0\_CHICK sp|O42414|NFASC\_CHICK tr|E1BZR0|E1BZR0\_CHICK tr|Q9PTF6|Q9PTF6\_CHICK tr|E1C3R3|E1C3R3\_CHICK tr|E1C243|E1C243\_CHICK tr|E1C860|E1C860\_CHICK tr|Q90YK6|Q90YK6\_CHICK tr|R4GL24|R4GL24\_CHICK sp|Q90703|NOS2\_CHICK tr|A9QM68|A9QM68\_CHICK tr|F1NUI7|F1NUI7\_CHICK sp|P08285|H103\_CHICK tr|G0ZS66|G0ZS66\_CHICK tr|F1P130|F1P130\_CHICK tr|F1NYK4|F1NYK4\_CHICK tr|E1BXY1|E1BXY1\_CHICK tr|F1NW21|F1NW21\_CHICK tr|F1NI55|F1NI55\_CHICK tr|Q5ZIW9|Q5ZIW9\_CHICK tr|Q7T2X8|Q7T2X8\_CHICK tr|F1NVC4|F1NVC4\_CHICK tr|E1C6W1|E1C6W1\_CHICK tr|C4PCL3|C4PCL3\_GALLA tr|E1BVH0|E1BVH0\_CHICK tr|F1NJ40|F1NJ40\_CHICK tr|R4GI78|R4GI78\_CHICK tr|F1NY05|F1NY05\_CHICK tr|E1BT21|E1BT21\_CHICK tr|E1BY25|E1BY25\_CHICK tr|F1NB64|F1NB64\_CHICK sp|P08287|H11L\_CHICK tr|Q0PVE5|Q0PVE5\_CHICK tr|F1NDV3|F1NDV3\_CHICK tr|F1NGR4|F1NGR4\_CHICK tr|M1XGN6|M1XGN6\_CHICK tr|E1BY97|E1BY97\_CHICK sp|Q5ZKM0|DTBP1\_CHICK tr|F1NP60|F1NP60\_CHICK tr|F1NMC0|F1NMC0\_CHICK tr|R4GM91|R4GM91\_CHICK tr|F1NKS0|F1NKS0\_CHICK tr|Q5F3A3|Q5F3A3\_CHICK tr|F1NL78|F1NL78\_CHICK tr|F7B5U8|F7B5U8\_CHICK tr|F1P3W0|F1P3W0\_CHICK tr|Q5ZMH7|Q5ZMH7\_CHICK tr|F1NZM2|F1NZM2\_CHICK tr|Q5ZMS7|Q5ZMS7\_CHICK tr|E1C9A9|E1C9A9\_CHICK tr|R4GKR3|R4GKR3\_CHICK tr|F1NIJ3|F1NIJ3\_CHICK tr|R4GGD5|R4GGD5\_CHICK tr|A5HUM3|A5HUM3\_CHICK tr|F1N8T3|F1N8T3\_CHICK tr|F1NFA6|F1NFA6\_CHICK tr|E1BQS8|E1BQS8\_CHICK tr|F1CN36|F1CN36\_CHICK tr|F1CN23|F1CN23\_CHICK tr|E1C113|E1C113\_CHICK tr|E1BT30|E1BT30\_CHICK sp|Q6GVH4|GGNB2\_CHICK tr|C4PC65|C4PC65\_CHICK tr|C4PC53|C4PC53\_GALLA tr|C4PC64|C4PC64\_CHICK tr|E1BTZ6|E1BTZ6\_CHICK tr|E1BS63|E1BS63\_CHICK tr|E1BQM4|E1BQM4\_CHICK tr|F1NRG3|F1NRG3\_CHICK tr|Q5ZKB2|Q5ZKB2\_CHICK tr|E1BUG7|E1BUG7\_CHICK tr|E1BZQ9|E1BZQ9\_CHICK tr|F1P0Y3|F1P0Y3\_CHICK tr|F1P0Z1|F1P0Z1\_CHICK tr|E1BTX0|E1BTX0\_CHICK tr|F1NWU4|F1NWU4\_CHICK tr|F1NU63|F1NU63\_CHICK tr|F1NT62|F1NT62\_CHICK tr|R4GJ07|R4GJ07\_CHICK tr|F1N9A6|F1N9A6\_CHICK sp|Q5ZLS3|BRE1A\_CHICK tr|E1C0S7|E1C0S7\_CHICK tr|F1NCI1|F1NCI1\_CHICK tr|E1BX64|E1BX64\_CHICK tr|E1BW59|E1BW59\_CHICK tr|F1NGE8|F1NGE8\_CHICK tr|F1P2I2|F1P2I2\_CHICK tr|Q5ZL68|Q5ZL68\_CHICK tr|F1NYF1|F1NYF1\_CHICK tr|F1P2U7|F1P2U7\_CHICK Q0V8M9 tr|E1BT38|E1BT38\_CHICK tr|F1NRW0|F1NRW0\_CHICK tr|E1BX53|E1BX53\_CHICK tr|H9L2R4|H9L2R4\_CHICK tr|R4GJA0|R4GJA0\_CHICK tr|F1NHP9|F1NHP9\_CHICK sp|Q5ZK33|LETM1\_CHICK tr|R4GK46|R4GK46\_CHICK tr|F1NKS8|F1NKS8\_CHICK tr|Q5ZJU9|Q5ZJU9\_CHICK tr|E1C9F1|E1C9F1\_CHICK tr|F1NSJ1|F1NSJ1\_CHICK tr|E1BTU4|E1BTU4\_CHICK tr|E1BSC6|E1BSC6\_CHICK tr|F1NMJ8|F1NMJ8\_CHICK tr|E1BV64|E1BV64\_CHICK tr|E1BSU8|E1BSU8\_CHICK tr|E1C746|E1C746\_CHICK tr|Q6R0I6|Q6R0I6\_CHICK tr|F6T168|F6T168\_CHICK tr|E1BYQ5|E1BYQ5\_CHICK tr|Q9DDD3|Q9DDD3\_CHICK tr|C4PCL2|C4PCL2\_GALLA tr|F1NEB4|F1NEB4\_CHICK tr|F1NH40|F1NH40\_CHICK tr|F1NQN1|F1NQN1\_CHICK tr|E1C7C4|E1C7C4\_CHICK tr|F1NUZ3|F1NUZ3\_CHICK tr|E1C6F8|E1C6F8\_CHICK sp|Q5F3K4|WDR48\_CHICK tr|E1C6T5|E1C6T5\_CHICK tr|R4GI61|R4GI61\_CHICK tr|E1BZM8|E1BZM8\_CHICK tr|F1NY17|F1NY17\_CHICK tr|F1NSE0|F1NSE0\_CHICK tr|G0YYQ5|G0YYQ5\_CHICK tr|F1NTZ0|F1NTZ0\_CHICK tr|O42483|O42483\_CHICK tr|E1C7I7|E1C7I7\_CHICK tr|E1C985|E1C985\_CHICK tr|R4GK19|R4GK19\_CHICK tr|E1BTI9|E1BTI9\_CHICK tr|E1BRI4|E1BRI4\_CHICK tr|E1C1S3|E1C1S3\_CHICK tr|E1BTG4|E1BTG4\_CHICK tr|F1NKN2|F1NKN2\_CHICK tr|Q6B0K7|Q6B0K7\_CHICK tr|F6RW97|F6RW97\_CHICK tr|Q98906|Q98906\_CHICK tr|F1NLK6|F1NLK6\_CHICK tr|F1NEX3|F1NEX3\_CHICK tr|E1BY12|E1BY12\_CHICK tr|E1C388|E1C388\_CHICK sp|Q5ZJ58|PI51B\_CHICK tr|E1BWJ1|E1BWJ1\_CHICK tr|F1NAG0|F1NAG0\_CHICK tr|F1NG99|F1NG99\_CHICK Q3SX14 tr|B7UTR6|B7UTR6\_CHICK tr|F1NJW5|F1NJW5\_CHICK tr|F1NNM3|F1NNM3\_CHICK tr|F1N8S7|F1N8S7\_CHICK tr|F1NY27|F1NY27\_CHICK tr|F1NAH3|F1NAH3\_CHICK sp|Q9DE13|BAZ2B\_CHICK tr|E1C427|E1C427\_CHICK tr|E1BSV2|E1BSV2\_CHICK tr|F1NY48|F1NY48\_CHICK tr|E1BWY7|E1BWY7\_CHICK tr|F1NGF1|F1NGF1\_CHICK tr|F1NAE5|F1NAE5\_CHICK tr|F1NJE2|F1NJE2\_CHICK tr|R4GMG6|R4GMG6\_CHICK tr|F1NT81|F1NT81\_CHICK tr|E1BZ78|E1BZ78\_CHICK tr|F1P284|F1P284\_CHICK tr|Q5ZJJ6|Q5ZJJ6\_CHICK tr|E1C200|E1C200\_CHICK tr|E1C5S8|E1C5S8\_CHICK tr|F1N9L8|F1N9L8\_CHICK tr|B5BSG4|B5BSG4\_CHICK tr|E1C7L1|E1C7L1\_CHICK tr|Q5F390|Q5F390\_CHICK tr|D3X734|D3X734\_CHICK tr|F1P0Y4|F1P0Y4\_CHICK tr|Q5F447|Q5F447\_CHICK sp|P28685|CNTN2\_CHICK tr|R4GGW5|R4GGW5\_CHICK tr|Q5ZMT8|Q5ZMT8\_CHICK tr|E1BSY9|E1BSY9\_CHICK tr|R4GIK4|R4GIK4\_CHICK tr|F1N8F9|F1N8F9\_CHICK tr|I0J174|I0J174\_CHICK tr|Q6R0J0|Q6R0J0\_CHICK tr|F1NX46|F1NX46\_CHICK tr|F1NRZ4|F1NRZ4\_CHICK tr|F1N982|F1N982\_CHICK tr|Q90858|Q90858\_CHICK tr|E1C750|E1C750\_CHICK sp|Q800K9|SURF4\_CHICK tr|D3X746|D3X746\_CHICK tr|E1BTL0|E1BTL0\_CHICK tr|F1NZ70|F1NZ70\_CHICK sp|Q5ZJ25|VPS51\_CHICK sp|O13156|GFRA1\_CHICK tr|Q90765|Q90765\_CHICK tr|F1NFB5|F1NFB5\_CHICK tr|E1C477|E1C477\_CHICK tr|E1BR28|E1BR28\_CHICK tr|F1P5T4|F1P5T4\_CHICK tr|F1NSM5|F1NSM5\_CHICK tr|A0ZXM3|A0ZXM3\_CHICK tr|E1C4F9|E1C4F9\_CHICK tr|E1BT27|E1BT27\_CHICK tr|H9L023|H9L023\_CHICK tr|F1NRR5|F1NRR5\_CHICK tr|Q5F496|Q5F496\_CHICK tr|E1BWJ6|E1BWJ6\_CHICK tr|Q5W4T6|Q5W4T6\_CHICK tr|F1NEG1|F1NEG1\_CHICK tr|E1C116|E1C116\_CHICK tr|E1BRV7|E1BRV7\_CHICK tr|E1BWF2|E1BWF2\_CHICK tr|F1NJI9|F1NJI9\_CHICK tr|F1NB47|F1NB47\_CHICK tr|F1CN03|F1CN03\_CHICK tr|Q5ZM67|Q5ZM67\_CHICK tr|E1C814|E1C814\_CHICK tr|Q8JGT3|Q8JGT3\_CHICK tr|E1BVG2|E1BVG2\_CHICK tr|I0J175|I0J175\_CHICK tr|E1C058|E1C058\_CHICK tr|R4GGS2|R4GGS2\_CHICK tr|F1NLT6|F1NLT6\_CHICK tr|Q5F487|Q5F487\_CHICK tr|F1P2D9|F1P2D9\_CHICK tr|Q5F3X3|Q5F3X3\_CHICK tr|E1BSH2|E1BSH2\_CHICK tr|F1N879|F1N879\_CHICK tr|E1BW82|E1BW82\_CHICK tr|E1C1M7|E1C1M7\_CHICK tr|F6RIN4|F6RIN4\_CHICK tr|E1BTQ3|E1BTQ3\_CHICK tr|F1P2A3|F1P2A3\_CHICK tr|I3XHQ5|I3XHQ5\_CHICK tr|I3XHQ4|I3XHQ4\_CHICK tr|F1NL00|F1NL00\_CHICK tr|F1NJ42|F1NJ42\_CHICK tr|E1BTM6|E1BTM6\_CHICK tr|D3X753|D3X753\_CHICK tr|F1NEG5|F1NEG5\_CHICK tr|A1KXK6|A1KXK6\_CHICK tr|F1NKI9|F1NKI9\_CHICK tr|A0A0D5ZCQ3|A0A0D5ZCQ3\_9VIRU tr|E1C900|E1C900\_CHICK tr|Q5F3G2|Q5F3G2\_CHICK sp|Q5ZL79|NOL11\_CHICK tr|Q9PW46|Q9PW46\_CHICK tr|F1NXH0|F1NXH0\_CHICK tr|Q9PUM5|Q9PUM5\_CHICK tr|E1BX38|E1BX38\_CHICK tr|F1NKI2|F1NKI2\_CHICK tr|E1C246|E1C246\_CHICK tr|F1NBD9|F1NBD9\_CHICK tr|F1NG80|F1NG80\_CHICK tr|E1C2F1|E1C2F1\_CHICK sp|O73691|EGR1\_CHICK tr|F1N8Y0|F1N8Y0\_CHICK tr|E1BZA4|E1BZA4\_CHICK tr|F1P0T0|F1P0T0\_CHICK sp|Q5ZIB8|PIRK5\_CHICK tr|F1NK07|F1NK07\_CHICK tr|F1NT76|F1NT76\_CHICK tr|E6N1X5|E6N1X5\_CHICK tr|E1BXW7|E1BXW7\_CHICK tr|F1P277|F1P277\_CHICK tr|E1BXP7|E1BXP7\_CHICK tr|E1C2A8|E1C2A8\_CHICK tr|Q4GWK0|Q4GWK0\_GALLA tr|H2D5E4|H2D5E4\_CHICK tr|C4PC97|C4PC97\_CHICK tr|E1BX79|E1BX79\_CHICK sp|Q90655|AKR\_CHICK tr|E1C205|E1C205\_CHICK tr|Q5ZJ77|Q5ZJ77\_CHICK tr|E1C8Q6|E1C8Q6\_CHICK tr|F1P1P4|F1P1P4\_CHICK tr|E1C0J9|E1C0J9\_CHICK tr|F1NBD8|F1NBD8\_CHICK tr|F1NWM5|F1NWM5\_CHICK tr|U5TU45|U5TU45\_CHICK tr|A0A023VSV8|A0A023VSV8\_CHICK tr|F1NTF2|F1NTF2\_CHICK tr|B9U3F2|B9U3F2\_CHICK tr|F1NVR5|F1NVR5\_CHICK tr|E1C0B2|E1C0B2\_CHICK tr|E1C0K5|E1C0K5\_CHICK tr|E1C1W8|E1C1W8\_CHICK tr|Q5F350|Q5F350\_CHICK tr|F1NMZ5|F1NMZ5\_CHICK tr|B0F2C1|B0F2C1\_CHICK sp|O57579|AMPN\_CHICK tr|E1BTP9|E1BTP9\_CHICK tr|Q5ZLX6|Q5ZLX6\_CHICK tr|E1BQV0|E1BQV0\_CHICK sp|Q5ZMJ7|CIP2A\_CHICK tr|Q789A5|Q789A5\_CHICK tr|Q789A4|Q789A4\_CHICK tr|Q02015|Q02015\_CHICK tr|Q789A6|Q789A6\_CHICK tr|F1P337|F1P337\_CHICK tr|E1BRA3|E1BRA3\_CHICK tr|F1NFV7|F1NFV7\_CHICK tr|F1NVK8|F1NVK8\_CHICK tr|F1P3B6|F1P3B6\_CHICK tr|H9KZJ8|H9KZJ8\_CHICK tr|E1BTJ9|E1BTJ9\_CHICK tr|Q2I2K5|Q2I2K5\_CHICK tr|E1C6L0|E1C6L0\_CHICK sp|Q9IAL8|NCKX1\_CHICK tr|F1NVZ4|F1NVZ4\_CHICK tr|B0F2C4|B0F2C4\_CHICK tr|Q5F466|Q5F466\_CHICK tr|Q5F3G4|Q5F3G4\_CHICK tr|F1NFZ8|F1NFZ8\_CHICK tr|Q5F3S9|Q5F3S9\_CHICK tr|F1NH28|F1NH28\_CHICK sp|Q5ZM41|TEX10\_CHICK tr|E1BVP6|E1BVP6\_CHICK tr|S5TYV1|S5TYV1\_CHICK tr|R9PXQ2|R9PXQ2\_CHICK tr|F1NAD2|F1NAD2\_CHICK tr|E1C0E3|E1C0E3\_CHICK tr|F1NLH2|F1NLH2\_CHICK tr|B1PMA4|B1PMA4\_CHICK sp|Q8UW76|TBX20\_CHICK tr|Q804X3|Q804X3\_CHICK tr|F1NA55|F1NA55\_CHICK sp|Q5ZKC1|EIF2A\_CHICK tr|O42142|O42142\_CHICK tr|F1P563|F1P563\_CHICK tr|F1P5I5|F1P5I5\_CHICK tr|Q3L252|Q3L252\_CHICK tr|E1BWL7|E1BWL7\_CHICK tr|F1P5G7|F1P5G7\_CHICK tr|F1NYN0|F1NYN0\_CHICK tr|F1NIA4|F1NIA4\_CHICK tr|E1BV30|E1BV30\_CHICK tr|H9KZN4|H9KZN4\_CHICK tr|E1C7G4|E1C7G4\_CHICK tr|E1C1L3|E1C1L3\_CHICK tr|F1NG00|F1NG00\_CHICK tr|F1NYI0|F1NYI0\_CHICK tr|R4GKS9|R4GKS9\_CHICK tr|F1NNE7|F1NNE7\_CHICK sp|Q6EE31|TCPQ\_CHICK sp|Q1HGK7|KTNA1\_CHICK tr|A0A0C4ZNU5|A0A0C4ZNU5\_9NEOP tr|F1NXU0|F1NXU0\_CHICK tr|F1NDR3|F1NDR3\_CHICK tr|F1NEP5|F1NEP5\_CHICK tr|E1C3M8|E1C3M8\_CHICK tr|Q76F78|Q76F78\_CHICK tr|Q5ZJS3|Q5ZJS3\_CHICK tr|F1NY37|F1NY37\_CHICK tr|E1BWU3|E1BWU3\_CHICK tr|F1N8I0|F1N8I0\_CHICK tr|A6BLM8|A6BLM8\_CHICK tr|A6BM73|A6BM73\_CHICK tr|F1P3I6|F1P3I6\_CHICK tr|Q5ZLP4|Q5ZLP4\_CHICK tr|F1NBJ2|F1NBJ2\_CHICK Q3KNV1 tr|F1P4R8|F1P4R8\_CHICK tr|E1C757|E1C757\_CHICK tr|E1C8U1|E1C8U1\_CHICK tr|Q9PWB8|Q9PWB8\_CHICK tr|R4GJE6|R4GJE6\_CHICK tr|Q90753|Q90753\_CHICK sp|Q90607|SEM3A\_CHICK tr|F1NC59|F1NC59\_CHICK tr|F1NX89|F1NX89\_CHICK tr|E1BRN8|E1BRN8\_CHICK tr|E1C5K4|E1C5K4\_CHICK tr|Q9W7G2|Q9W7G2\_CHICK tr|E1C0W1|E1C0W1\_CHICK tr|E1BRP8|E1BRP8\_CHICK tr|F1NP51|F1NP51\_CHICK tr|Q5F3F7|Q5F3F7\_CHICK tr|B2MUN7|B2MUN7\_CHICK tr|Q90933|Q90933\_CHICK tr|R4GFE1|R4GFE1\_CHICK tr|E1BXA6|E1BXA6\_CHICK sp|Q91957|XIRP1\_CHICK tr|R4GIJ1|R4GIJ1\_CHICK tr|E1BZK3|E1BZK3\_CHICK sp|Q02092|GHR\_CHICK tr|F1NPB8|F1NPB8\_CHICK tr|Q5ZJI1|Q5ZJI1\_CHICK tr|F1NMC3|F1NMC3\_CHICK tr|Q5ZI76|Q5ZI76\_CHICK tr|F1NL23|F1NL23\_CHICK tr|O42133|O42133\_CHICK tr|E1BWT2|E1BWT2\_CHICK tr|Q76L17|Q76L17\_CHICK tr|F1NGV5|F1NGV5\_CHICK tr|B6EAX2|B6EAX2\_CHICK tr|E1BTK8|E1BTK8\_CHICK tr|F1P3T8|F1P3T8\_CHICK tr|F1NCB0|F1NCB0\_CHICK tr|Q5ZMN9|Q5ZMN9\_CHICK sp|Q5ZKW8|HDX\_CHICK tr|E1BY88|E1BY88\_CHICK tr|F1P5R3|F1P5R3\_CHICK tr|E1BWK0|E1BWK0\_CHICK tr|F1NXX4|F1NXX4\_CHICK tr|F1NVF5|F1NVF5\_CHICK tr|Q4GWL3|Q4GWL3\_GALVA tr|Q90ZI1|Q90ZI1\_CHICK tr|E1BQH1|E1BQH1\_CHICK tr|F1NUN8|F1NUN8\_CHICK tr|Q335Q0|Q335Q0\_CHICK tr|Q335P9|Q335P9\_CHICK tr|F1NHK8|F1NHK8\_CHICK tr|F1NQF9|F1NQF9\_CHICK sp|Q5F485|DDX42\_CHICK tr|E1C5G2|E1C5G2\_CHICK sp|P12003|VINC\_CHICK tr|F1NYY0|F1NYY0\_CHICK tr|E1C9C6|E1C9C6\_CHICK tr|F1P1J5|F1P1J5\_CHICK tr|F1NCI3|F1NCI3\_CHICK tr|F1P3Q5|F1P3Q5\_CHICK tr|F1NV89|F1NV89\_CHICK tr|F1NYN6|F1NYN6\_CHICK tr|H9L0D5|H9L0D5\_CHICK tr|F1NI58|F1NI58\_CHICK tr|E1C3K7|E1C3K7\_CHICK tr|E1BXX6|E1BXX6\_CHICK tr|F1NUS8|F1NUS8\_CHICK tr|F1NDK3|F1NDK3\_CHICK tr|F1P1V0|F1P1V0\_CHICK tr|E1C505|E1C505\_CHICK sp|P79760|CP1A4\_CHICK tr|F1P054|F1P054\_CHICK tr|E1C5W8|E1C5W8\_CHICK tr|R4GGA2|R4GGA2\_CHICK tr|F1NL95|F1NL95\_CHICK tr|E1C972|E1C972\_CHICK tr|F1P5G6|F1P5G6\_CHICK tr|F9W2X7|F9W2X7\_CHICK tr|F1P3G8|F1P3G8\_CHICK tr|E1C9A8|E1C9A8\_CHICK tr|E1C046|E1C046\_CHICK tr|F1P2P9|F1P2P9\_CHICK tr|F1P339|F1P339\_CHICK tr|E1C8Q3|E1C8Q3\_CHICK tr|E1C8X9|E1C8X9\_CHICK tr|F1NMF0|F1NMF0\_CHICK tr|E1BTL1|E1BTL1\_CHICK tr|R4GM55|R4GM55\_CHICK tr|Q6JGT3|Q6JGT3\_CHICK tr|F1NC45|F1NC45\_CHICK tr|Q5ZJD4|Q5ZJD4\_CHICK tr|E1BXP0|E1BXP0\_CHICK tr|F1NB97|F1NB97\_CHICK tr|F1NSV5|F1NSV5\_CHICK tr|F1P5E9|F1P5E9\_CHICK tr|F1NE00|F1NE00\_CHICK tr|F1P2T2|F1P2T2\_CHICK tr|F1NC77|F1NC77\_CHICK tr|F1NCU3|F1NCU3\_CHICK tr|E1BXM2|E1BXM2\_CHICK tr|E1C397|E1C397\_CHICK tr|R4GFC0|R4GFC0\_CHICK tr|R4GHC5|R4GHC5\_CHICK tr|F6V7J1|F6V7J1\_CHICK tr|E1C020|E1C020\_CHICK tr|E1C5E0|E1C5E0\_CHICK tr|F1P4D7|F1P4D7\_CHICK tr|E1BXA5|E1BXA5\_CHICK tr|E1BUT5|E1BUT5\_CHICK tr|F1NV00|F1NV00\_CHICK tr|F1P258|F1P258\_CHICK tr|Q4PLA4|Q4PLA4\_CHICK tr|E1BQK3|E1BQK3\_CHICK sp|P20678|CP2H2\_CHICK sp|Q75R65|JAK2\_CHICK tr|F1NQI2|F1NQI2\_CHICK tr|H9L3L1|H9L3L1\_CHICK tr|F1NRX3|F1NRX3\_CHICK tr|F1NE91|F1NE91\_CHICK sp|Q5XXA9|PSIP1\_CHICK tr|F1NZP8|F1NZP8\_CHICK tr|F1NY54|F1NY54\_CHICK tr|R4GLC3|R4GLC3\_CHICK tr|E1C557|E1C557\_CHICK tr|R4GKB0|R4GKB0\_CHICK tr|Q5ZLE7|Q5ZLE7\_CHICK tr|F1N914|F1N914\_CHICK tr|F1NLJ3|F1NLJ3\_CHICK tr|R4GHM1|R4GHM1\_CHICK tr|L8ECR2|L8ECR2\_CHICK tr|E1C846|E1C846\_CHICK sp|Q5ZJH2|NCLN\_CHICK sp|Q5ZL98|RPC1\_CHICK tr|F1NIK7|F1NIK7\_CHICK tr|F1CN39|F1CN39\_CHICK tr|F1CN24|F1CN24\_CHICK tr|F1NST0|F1NST0\_CHICK tr|R4GM40|R4GM40\_CHICK sp|Q5ZML9|SETD3\_CHICK tr|F1NAX6|F1NAX6\_CHICK tr|F1NC31|F1NC31\_CHICK tr|F1P076|F1P076\_CHICK tr|E1C7U1|E1C7U1\_CHICK sp|Q5ZJY3|GARL3\_CHICK tr|E1BQN1|E1BQN1\_CHICK tr|E1BZT9|E1BZT9\_CHICK tr|F1NQ11|F1NQ11\_CHICK tr|F1NI62|F1NI62\_CHICK tr|E1BQZ7|E1BQZ7\_CHICK tr|F1NKF7|F1NKF7\_CHICK tr|E1C700|E1C700\_CHICK tr|F1NS61|F1NS61\_CHICK tr|F1N932|F1N932\_CHICK tr|A6YJX2|A6YJX2\_CHICK tr|F1NZZ6|F1NZZ6\_CHICK tr|F1NL65|F1NL65\_CHICK tr|F1NCL7|F1NCL7\_CHICK tr|F1NPS4|F1NPS4\_CHICK tr|F1N862|F1N862\_CHICK tr|E1BUU5|E1BUU5\_CHICK tr|E1C3Q8|E1C3Q8\_CHICK tr|F1NV98|F1NV98\_CHICK tr|Q670Z8|Q670Z8\_CHICK tr|E1BQ68|E1BQ68\_CHICK tr|F1NRB6|F1NRB6\_CHICK tr|F1P0G1|F1P0G1\_CHICK tr|R4MXF2|R4MXF2\_CHICK tr|E1BXK1|E1BXK1\_CHICK tr|F1P0T8|F1P0T8\_CHICK tr|F1NUN3|F1NUN3\_CHICK tr|E1BSJ5|E1BSJ5\_CHICK tr|E1BS95|E1BS95\_CHICK tr|F1P4F0|F1P4F0\_CHICK tr|Q5ZIZ8|Q5ZIZ8\_CHICK tr|F1NZU1|F1NZU1\_CHICK tr|F1NZU0|F1NZU0\_CHICK tr|F1P5H0|F1P5H0\_CHICK tr|Q6R0I1|Q6R0I1\_CHICK tr|E1BSH9|E1BSH9\_CHICK tr|F1NC26|F1NC26\_CHICK tr|F1NB56|F1NB56\_CHICK tr|F1P4T6|F1P4T6\_CHICK tr|F1NAZ0|F1NAZ0\_CHICK tr|E1BQL2|E1BQL2\_CHICK tr|F1NRL7|F1NRL7\_CHICK tr|E1C793|E1C793\_CHICK tr|E1BRE5|E1BRE5\_CHICK tr|E1BZ64|E1BZ64\_CHICK tr|F1ND79|F1ND79\_CHICK tr|E1BZ04|E1BZ04\_CHICK tr|F1NKG9|F1NKG9\_CHICK tr|F1NU19|F1NU19\_CHICK tr|E1C8Y7|E1C8Y7\_CHICK tr|E1C0C3|E1C0C3\_CHICK tr|F1NCA5|F1NCA5\_CHICK tr|F1NWR6|F1NWR6\_CHICK tr|E1BUE5|E1BUE5\_CHICK tr|H9L3N8|H9L3N8\_CHICK tr|E1BV52|E1BV52\_CHICK tr|F1NU24|F1NU24\_CHICK tr|F1P4G2|F1P4G2\_CHICK tr|E1C2W2|E1C2W2\_CHICK tr|F1NVG1|F1NVG1\_CHICK tr|E1C5W1|E1C5W1\_CHICK tr|F1NY20|F1NY20\_CHICK tr|E1BXR5|E1BXR5\_CHICK tr|Q98ST1|Q98ST1\_CHICK tr|F1NPH1|F1NPH1\_CHICK tr|H9L0H2|H9L0H2\_CHICK tr|F1NRI7|F1NRI7\_CHICK tr|F1NWX9|F1NWX9\_CHICK tr|O42124|O42124\_CHICK tr|F1NBC5|F1NBC5\_CHICK tr|F1NZW6|F1NZW6\_CHICK tr|F1NA27|F1NA27\_CHICK tr|Q5ZJ88|Q5ZJ88\_CHICK tr|E1C5Y3|E1C5Y3\_CHICK tr|R4GIF6|R4GIF6\_CHICK tr|Q5ZL80|Q5ZL80\_CHICK tr|E1BYA6|E1BYA6\_CHICK tr|H9KYQ9|H9KYQ9\_CHICK tr|E1BSS2|E1BSS2\_CHICK tr|E1BUN6|E1BUN6\_CHICK tr|F1NG59|F1NG59\_CHICK sp|Q5F488|MAGI3\_CHICK tr|Q4F8N2|Q4F8N2\_CHICK tr|F1P0I0|F1P0I0\_CHICK tr|G0ZS65|G0ZS65\_CHICK tr|F1NHB6|F1NHB6\_CHICK tr|Q4GWI7|Q4GWI7\_GALSO tr|F1NXV4|F1NXV4\_CHICK tr|A0A090AVG4|A0A090AVG4\_CHICK tr|H9KZE7|H9KZE7\_CHICK tr|Q5F432|Q5F432\_CHICK tr|R4GG82|R4GG82\_CHICK tr|E1C2J7|E1C2J7\_CHICK tr|F1NQH1|F1NQH1\_CHICK tr|A0A0D5ZD31|A0A0D5ZD31\_9VIRU tr|E1BRQ9|E1BRQ9\_CHICK tr|E1BRG3|E1BRG3\_CHICK tr|F1NA36|F1NA36\_CHICK tr|Q2UXN0|Q2UXN0\_CHICK sp|Q5R1T0|CAF1A\_CHICK tr|F1CN31|F1CN31\_CHICK tr|F1NQU2|F1NQU2\_CHICK tr|F1NB32|F1NB32\_CHICK tr|E1C8G2|E1C8G2\_CHICK tr|F1NA54|F1NA54\_CHICK tr|E1BS67|E1BS67\_CHICK tr|H2D5E3|H2D5E3\_CHICK tr|F1NZZ7|F1NZZ7\_CHICK tr|F5CST5|F5CST5\_CHICK tr|E1BSJ7|E1BSJ7\_CHICK sp|P49140|SKI\_CHICK tr|F1NDM5|F1NDM5\_CHICK tr|Q9I9K3|Q9I9K3\_CHICK sp|F1P065|FARP1\_CHICK tr|R4RAN1|R4RAN1\_CHICK tr|F1NYV9|F1NYV9\_CHICK tr|F1P2W2|F1P2W2\_CHICK sp|Q8UVC3|INVS\_CHICK tr|R4GLF4|R4GLF4\_CHICK tr|F1N8Q9|F1N8Q9\_CHICK sp|P29415|CXA3\_CHICK tr|E1BUV9|E1BUV9\_CHICK tr|F1P404|F1P404\_CHICK tr|E1C1Y1|E1C1Y1\_CHICK sp|P05094|ACTN1\_CHICK tr|R9PXN4|R9PXN4\_CHICK tr|Q335P8|Q335P8\_CHICK tr|E1BTW0|E1BTW0\_CHICK tr|E1C019|E1C019\_CHICK tr|F1P4A6|F1P4A6\_CHICK tr|F1NBK3|F1NBK3\_CHICK tr|F1P0T4|F1P0T4\_CHICK tr|F1N975|F1N975\_CHICK sp|Q04678|SSRP1\_CHICK tr|B6ZLK1|B6ZLK1\_CHICK tr|R4GH56|R4GH56\_CHICK tr|Q5UKZ3|Q5UKZ3\_CHICK tr|E1C8V3|E1C8V3\_CHICK tr|H9KZT7|H9KZT7\_CHICK tr|F1NI67|F1NI67\_CHICK tr|E1C7L9|E1C7L9\_CHICK tr|E1BX84|E1BX84\_CHICK tr|Q5ZL11|Q5ZL11\_CHICK tr|E1C5R3|E1C5R3\_CHICK tr|E1C478|E1C478\_CHICK tr|H9L0K8|H9L0K8\_CHICK tr|Q9DGG7|Q9DGG7\_CHICK tr|A5HDU0|A5HDU0\_CHICK tr|E5DEB3|E5DEB3\_CHICK tr|Q85A52|Q85A52\_CHICK tr|Q4GWN9|Q4GWN9\_CHICK tr|E5DFP4|E5DFP4\_CHICK tr|E5DED9|E5DED9\_CHICK tr|Q4GWM6|Q4GWM6\_CHICK tr|Q4GWR5|Q4GWR5\_GALSO tr|E5DFK5|E5DFK5\_CHICK tr|W8NXV0|W8NXV0\_CHICK tr|Q4GWQ2|Q4GWQ2\_CHICK tr|Q76L08|Q76L08\_CHICK tr|E5DEG5|E5DEG5\_CHICK tr|Q9PW44|Q9PW44\_CHICK tr|Q9PUM3|Q9PUM3\_CHICK tr|F1NY22|F1NY22\_CHICK tr|F1P5V3|F1P5V3\_CHICK tr|E1C2L8|E1C2L8\_CHICK tr|E1BRA6|E1BRA6\_CHICK tr|Q5ZMI2|Q5ZMI2\_CHICK tr|F1P3U1|F1P3U1\_CHICK tr|H9KZD7|H9KZD7\_CHICK tr|H9KYR6|H9KYR6\_CHICK tr|Q90YN1|Q90YN1\_CHICK tr|F1NY99|F1NY99\_CHICK tr|E1C4J3|E1C4J3\_CHICK tr|F1P3K9|F1P3K9\_CHICK tr|F1NCC9|F1NCC9\_CHICK tr|Q5ZLP9|Q5ZLP9\_CHICK tr|E1BY66|E1BY66\_CHICK tr|F1P1C3|F1P1C3\_CHICK sp|P29318|EPHA3\_CHICK tr|F1NJX6|F1NJX6\_CHICK tr|E1BVY0|E1BVY0\_CHICK tr|E1BRM7|E1BRM7\_CHICK tr|F1P1P9|F1P1P9\_CHICK tr|E1C0E2|E1C0E2\_CHICK tr|E1C4F1|E1C4F1\_CHICK tr|A0A0D5ZD45|A0A0D5ZD45\_9VIRU sp|P43691|GATA4\_CHICK tr|Q68PG0|Q68PG0\_CHICK tr|F1NGK9|F1NGK9\_CHICK sp|Q06637|BFSP1\_CHICK tr|F1NI25|F1NI25\_CHICK tr|E1C9C1|E1C9C1\_CHICK tr|O46788|O46788\_CHICK tr|B5BSC2|B5BSC2\_CHICK tr|E1C5C6|E1C5C6\_CHICK tr|E1BZR7|E1BZR7\_CHICK tr|F1NQ83|F1NQ83\_CHICK tr|F1NH69|F1NH69\_CHICK tr|F1NAE3|F1NAE3\_CHICK tr|F1NUG6|F1NUG6\_CHICK tr|F1NW53|F1NW53\_CHICK tr|F1P493|F1P493\_CHICK sp|O42400|AXIN1\_CHICK tr|Q6B3C4|Q6B3C4\_CHICK tr|R4GJA6|R4GJA6\_CHICK tr|E1BWC9|E1BWC9\_CHICK tr|F1N8B5|F1N8B5\_CHICK tr|E1C2F9|E1C2F9\_CHICK tr|E1C642|E1C642\_CHICK tr|F1N9N8|F1N9N8\_CHICK tr|R4GH67|R4GH67\_CHICK tr|F1P4Q0|F1P4Q0\_CHICK tr|F1NCZ7|F1NCZ7\_CHICK tr|F1NAK2|F1NAK2\_CHICK tr|Q9IBA7|Q9IBA7\_CHICK tr|E1C4G6|E1C4G6\_CHICK tr|Q5ZHT2|Q5ZHT2\_CHICK tr|Q6JGU5|Q6JGU5\_CHICK tr|Q9GJA8|Q9GJA8\_CHICK tr|Q6JGU7|Q6JGU7\_CHICK tr|F1NN43|F1NN43\_CHICK tr|E1C514|E1C514\_CHICK tr|F1P1D4|F1P1D4\_CHICK tr|E1C6H0|E1C6H0\_CHICK sp|Q90763|CADH7\_CHICK tr|Q5F3T4|Q5F3T4\_CHICK tr|E1C866|E1C866\_CHICK tr|E1BQP3|E1BQP3\_CHICK tr|F1NBP6|F1NBP6\_CHICK tr|F1P5C3|F1P5C3\_CHICK tr|F1P508|F1P508\_CHICK tr|Q5ZM12|Q5ZM12\_CHICK tr|F1NZZ4|F1NZZ4\_CHICK tr|Q5ZM10|Q5ZM10\_CHICK tr|Q90W08|Q90W08\_CHICK tr|F1NXH4|F1NXH4\_CHICK tr|R4GJK6|R4GJK6\_CHICK tr|E1BX00|E1BX00\_CHICK tr|E1C6E4|E1C6E4\_CHICK tr|F1NZU9|F1NZU9\_CHICK tr|F1NRG5|F1NRG5\_CHICK tr|E1BX44|E1BX44\_CHICK tr|F1NX29|F1NX29\_CHICK tr|F1P0X9|F1P0X9\_CHICK tr|E1C4A3|E1C4A3\_CHICK tr|E1C8F6|E1C8F6\_CHICK tr|F1P369|F1P369\_CHICK tr|E1C230|E1C230\_CHICK tr|E1BQW0|E1BQW0\_CHICK tr|R4GLZ0|R4GLZ0\_CHICK tr|F1P3E8|F1P3E8\_CHICK tr|F1NBZ9|F1NBZ9\_CHICK tr|C4PC61|C4PC61\_CHICK tr|F1NGZ5|F1NGZ5\_CHICK sp|Q5ZKT1|TBCC1\_CHICK tr|Q5ZLW4|Q5ZLW4\_CHICK tr|A2TGX4|A2TGX4\_CHICK tr|F1NGZ2|F1NGZ2\_CHICK tr|F1NLB4|F1NLB4\_CHICK tr|F1NBI1|F1NBI1\_CHICK tr|F1NGV0|F1NGV0\_CHICK tr|F1N9I2|F1N9I2\_CHICK tr|E1BRU7|E1BRU7\_CHICK tr|Q5ZJI4|Q5ZJI4\_CHICK tr|O57658|O57658\_CHICK tr|F1NYW0|F1NYW0\_CHICK tr|E1C1G5|E1C1G5\_CHICK tr|E1BYT9|E1BYT9\_CHICK tr|F1P572|F1P572\_CHICK tr|F1NEP4|F1NEP4\_CHICK tr|F1NX11|F1NX11\_CHICK tr|E1C0W6|E1C0W6\_CHICK tr|E1BQA9|E1BQA9\_CHICK tr|F1NZX7|F1NZX7\_CHICK tr|E1C430|E1C430\_CHICK tr|Q5ZJE9|Q5ZJE9\_CHICK tr|E1C657|E1C657\_CHICK tr|E1C6S7|E1C6S7\_CHICK sp|Q5F3G6|P20L1\_CHICK tr|F1NUJ1|F1NUJ1\_CHICK tr|E1C6B7|E1C6B7\_CHICK tr|F1NXP4|F1NXP4\_CHICK tr|E1BZB0|E1BZB0\_CHICK tr|H9L1V9|H9L1V9\_CHICK tr|F1NV72|F1NV72\_CHICK tr|Q5ZIC1|Q5ZIC1\_CHICK sp|Q5ZMJ9|SRRM1\_CHICK tr|F1NT20|F1NT20\_CHICK sp|Q5F3X4|U5S1\_CHICK sp|Q5ZJF1|CSN3\_CHICK tr|E1BY08|E1BY08\_CHICK tr|A4VAR9|A4VAR9\_CHICK tr|R4GH71|R4GH71\_CHICK tr|F1NY88|F1NY88\_CHICK tr|E1BQK4|E1BQK4\_CHICK tr|F1NYD5|F1NYD5\_CHICK sp|Q5ZJK1|THOC5\_CHICK tr|F1NCM4|F1NCM4\_CHICK Q6IFX2 tr|Q5ZJE0|Q5ZJE0\_CHICK tr|F1NAZ8|F1NAZ8\_CHICK tr|E1C0P4|E1C0P4\_CHICK tr|F1NZB7|F1NZB7\_CHICK tr|Q5ZIK7|Q5ZIK7\_CHICK tr|F1NSN6|F1NSN6\_CHICK tr|F1NDB6|F1NDB6\_CHICK tr|F1NYK0|F1NYK0\_CHICK tr|F1P2G2|F1P2G2\_CHICK tr|F1NP16|F1NP16\_CHICK tr|Q5ZM22|Q5ZM22\_CHICK tr|F1NRW9|F1NRW9\_CHICK tr|F1P023|F1P023\_CHICK tr|F1NVW1|F1NVW1\_CHICK tr|R4GHG9|R4GHG9\_CHICK tr|F1P3J2|F1P3J2\_CHICK sp|Q9DGG6|ADCY9\_CHICK tr|E1C1Q5|E1C1Q5\_CHICK tr|A0A090AQR1|A0A090AQR1\_CHICK tr|E1BZS6|E1BZS6\_CHICK tr|A0A090ATY8|A0A090ATY8\_CHICK tr|E1C1C2|E1C1C2\_CHICK tr|D2D3P4|D2D3P4\_CHICK tr|E1BQM0|E1BQM0\_CHICK sp|Q5F3N6|BAP1\_CHICK tr|F1NTD7|F1NTD7\_CHICK tr|E1BRI2|E1BRI2\_CHICK sp|Q9DGB6|TLR22\_CHICK tr|E1C6L2|E1C6L2\_CHICK tr|E1BS28|E1BS28\_CHICK tr|F1NBY6|F1NBY6\_CHICK tr|F1NGR2|F1NGR2\_CHICK sp|Q7ZT11|ANKR1\_CHICK tr|F1NYV8|F1NYV8\_CHICK tr|Q9DGN6|Q9DGN6\_CHICK tr|F1NN14|F1NN14\_CHICK sp|O57424|HYAS2\_CHICK tr|A0A0D5ZDB7|A0A0D5ZDB7\_9VIRU tr|E6N1W7|E6N1W7\_CHICK tr|F1NBK9|F1NBK9\_CHICK tr|F1NIY9|F1NIY9\_CHICK sp|Q90718|SRF\_CHICK tr|F1NHV2|F1NHV2\_CHICK tr|A0A547|A0A547\_CHICK tr|E1C7P3|E1C7P3\_CHICK tr|R9PXN5|R9PXN5\_CHICK tr|R4GL67|R4GL67\_CHICK tr|F1NW41|F1NW41\_CHICK tr|A0A0A0MQ38|A0A0A0MQ38\_CHICK sp|Q5ZI58|F214A\_CHICK tr|F1NXD7|F1NXD7\_CHICK tr|Q92062|Q92062\_CHICK tr|F1P5Q6|F1P5Q6\_CHICK tr|E1BZV0|E1BZV0\_CHICK tr|E1C4Y7|E1C4Y7\_CHICK tr|E1BUF0|E1BUF0\_CHICK tr|F1N9U8|F1N9U8\_CHICK tr|F1P091|F1P091\_CHICK tr|R4GKD9|R4GKD9\_CHICK tr|Q5F3E9|Q5F3E9\_CHICK tr|E1C1Q1|E1C1Q1\_CHICK tr|F1NZC4|F1NZC4\_CHICK tr|E1C9I7|E1C9I7\_CHICK tr|Q9MPA6|Q9MPA6\_CHICK sp|P18940|NU5M\_CHICK tr|E5DEC6|E5DEC6\_CHICK tr|A0A0B4ZTR9|A0A0B4ZTR9\_CHICK tr|E5DFJ2|E5DFJ2\_CHICK tr|E5DFV9|E5DFV9\_CHICK tr|E1BW90|E1BW90\_CHICK tr|Q5ZLV7|Q5ZLV7\_CHICK tr|F1NIS1|F1NIS1\_CHICK tr|A0A0D5ZDF0|A0A0D5ZDF0\_9VIRU tr|F1NCT9|F1NCT9\_CHICK tr|E1BV44|E1BV44\_CHICK tr|F1NBS7|F1NBS7\_CHICK tr|E1C3Q0|E1C3Q0\_CHICK tr|F1NLF0|F1NLF0\_CHICK tr|Q5ZI26|Q5ZI26\_CHICK tr|F1NIB7|F1NIB7\_CHICK tr|E1C457|E1C457\_CHICK tr|Q076D6|Q076D6\_CHICK tr|Q5F3U4|Q5F3U4\_CHICK tr|Q8AYP9|Q8AYP9\_CHICK sp|A7XYH5|SOBP\_CHICK tr|V5K679|V5K679\_CHICK tr|F1P1T8|F1P1T8\_CHICK tr|F1NLR2|F1NLR2\_CHICK tr|E1C840|E1C840\_CHICK tr|R4GKH4|R4GKH4\_CHICK tr|E1BWT7|E1BWT7\_CHICK tr|F1NB94|F1NB94\_CHICK tr|F1NM84|F1NM84\_CHICK tr|F1N9B9|F1N9B9\_CHICK tr|E1BZT2|E1BZT2\_CHICK tr|F1NEP6|F1NEP6\_CHICK tr|B5BSJ9|B5BSJ9\_CHICK tr|R4GH92|R4GH92\_CHICK tr|R4GLK5|R4GLK5\_CHICK tr|F1NG27|F1NG27\_CHICK tr|C4PCE3|C4PCE3\_CHICK tr|C4PCE0|C4PCE0\_CHICK tr|F1NSY9|F1NSY9\_CHICK tr|E1BU38|E1BU38\_CHICK sp|Q64HY5|SP8\_CHICK tr|F1NCR4|F1NCR4\_CHICK tr|F1NXW9|F1NXW9\_CHICK tr|Q4PLA5|Q4PLA5\_CHICK tr|W8P5E2|W8P5E2\_CHICK tr|R4GG44|R4GG44\_CHICK tr|F1NUE2|F1NUE2\_CHICK tr|F1NBH7|F1NBH7\_CHICK tr|F1NXK3|F1NXK3\_CHICK tr|Q2THW5|Q2THW5\_CHICK tr|Q2THW4|Q2THW4\_CHICK tr|E1C4E0|E1C4E0\_CHICK tr|E1BRZ4|E1BRZ4\_CHICK sp|Q5YCC7|TMC3\_CHICK tr|H9KZE0|H9KZE0\_CHICK tr|Q90ZT4|Q90ZT4\_CHICK tr|F1NWJ4|F1NWJ4\_CHICK tr|R4GH06|R4GH06\_CHICK tr|F1NGP0|F1NGP0\_CHICK tr|H9KZ99|H9KZ99\_CHICK tr|M9NCD4|M9NCD4\_CHICK tr|F1NEN6|F1NEN6\_CHICK tr|Q98TD1|Q98TD1\_CHICK tr|F1CN57|F1CN57\_CHICK tr|F1CN60|F1CN60\_CHICK tr|F1NFW5|F1NFW5\_CHICK tr|E1BS10|E1BS10\_CHICK tr|F1NIN5|F1NIN5\_CHICK tr|H9KZE3|H9KZE3\_CHICK tr|R4GFZ6|R4GFZ6\_CHICK tr|F8UV40|F8UV40\_CHICK sp|Q5ZIJ0|BUD13\_CHICK tr|E1C7R4|E1C7R4\_CHICK tr|F1NHL0|F1NHL0\_CHICK sp|Q66PG3|LARGE\_CHICK tr|E1C4H2|E1C4H2\_CHICK tr|F1N869|F1N869\_CHICK tr|H9L0K6|H9L0K6\_CHICK tr|F1NLL3|F1NLL3\_CHICK tr|R4GFH3|R4GFH3\_CHICK tr|E1C8N6|E1C8N6\_CHICK tr|F1NQW1|F1NQW1\_CHICK sp|P46896|GTR1\_CHICK sp|Q5ZM36|IF4A3\_CHICK tr|F1NA43|F1NA43\_CHICK tr|R4RB95|R4RB95\_CHICK tr|E1C2G3|E1C2G3\_CHICK tr|H9KZN3|H9KZN3\_CHICK tr|F1NF22|F1NF22\_CHICK tr|F1P4W8|F1P4W8\_CHICK tr|H9L081|H9L081\_CHICK tr|E1C1N1|E1C1N1\_CHICK tr|M1XJF4|M1XJF4\_CHICK tr|F1NZA0|F1NZA0\_CHICK tr|E1BVH5|E1BVH5\_CHICK tr|E1C3R6|E1C3R6\_CHICK tr|F1N8G0|F1N8G0\_CHICK tr|D3X735|D3X735\_CHICK tr|R4GKT9|R4GKT9\_CHICK tr|Q90707|Q90707\_CHICK tr|F1NYW2|F1NYW2\_CHICK Q9TRI1 tr|F1NLT5|F1NLT5\_CHICK tr|E1BX75|E1BX75\_CHICK sp|P26009|ITA8\_CHICK tr|F1NSA4|F1NSA4\_CHICK tr|F1N9Z1|F1N9Z1\_CHICK tr|F1NGT5|F1NGT5\_CHICK tr|H9L1C4|H9L1C4\_CHICK tr|F1NK59|F1NK59\_CHICK tr|F1NKW0|F1NKW0\_CHICK tr|W6RT25|W6RT25\_CHICK tr|E1BRM8|E1BRM8\_CHICK tr|E1C3F4|E1C3F4\_CHICK tr|E1C303|E1C303\_CHICK tr|F1NPE0|F1NPE0\_CHICK tr|E1BXS9|E1BXS9\_CHICK sp|Q90YJ9|CLAT\_CHICK tr|E1AWU3|E1AWU3\_CHICK tr|Q5F3W0|Q5F3W0\_CHICK tr|F1CN25|F1CN25\_CHICK tr|F1ND96|F1ND96\_CHICK tr|Q5ZIZ5|Q5ZIZ5\_CHICK sp|Q5ZLF4|ZNT5\_CHICK sp|Q5ZHV2|NAA35\_CHICK tr|E1BYE7|E1BYE7\_CHICK tr|B8XXD6|B8XXD6\_CHICK tr|F1NV94|F1NV94\_CHICK tr|E1BQJ8|E1BQJ8\_CHICK tr|E1BWC3|E1BWC3\_CHICK sp|Q5ZL67|NF2L1\_CHICK tr|H9L378|H9L378\_CHICK tr|A0A089G7L3|A0A089G7L3\_CHICK tr|W0K996|W0K996\_CHICK tr|D2XSJ5|D2XSJ5\_CHICK tr|F1NIF9|F1NIF9\_CHICK tr|R4GG45|R4GG45\_CHICK tr|E1C3A8|E1C3A8\_CHICK tr|E1BQM9|E1BQM9\_CHICK tr|F1NQ91|F1NQ91\_CHICK tr|R4GLW2|R4GLW2\_CHICK tr|F1NQ68|F1NQ68\_CHICK H-INV:HIT000016045 tr|F1P3V8|F1P3V8\_CHICK sp|Q5ZKR7|ACBG2\_CHICK tr|E1BW67|E1BW67\_CHICK tr|E1BYW5|E1BYW5\_CHICK tr|E1BT09|E1BT09\_CHICK tr|E1C6D5|E1C6D5\_CHICK tr|E1BTA5|E1BTA5\_CHICK tr|F1NPJ9|F1NPJ9\_CHICK tr|F1P2H4|F1P2H4\_CHICK tr|E1BUR0|E1BUR0\_CHICK tr|F1NT42|F1NT42\_CHICK tr|E1C735|E1C735\_CHICK ENSEMBL:ENSBTAP00000018574 tr|E1BQH9|E1BQH9\_CHICK tr|F1P2T0|F1P2T0\_CHICK tr|F1P4I4|F1P4I4\_CHICK tr|F1NN31|F1NN31\_CHICK tr|Q5ZJB9|Q5ZJB9\_CHICK tr|E1BVP1|E1BVP1\_CHICK tr|F1NKN7|F1NKN7\_CHICK tr|Q5ZHY3|Q5ZHY3\_CHICK tr|F1NM51|F1NM51\_CHICK tr|E1BS80|E1BS80\_CHICK tr|R4GIK7|R4GIK7\_CHICK tr|F1NFK1|F1NFK1\_CHICK tr|F1P5P6|F1P5P6\_CHICK tr|F1NP02|F1NP02\_CHICK tr|E1BTQ0|E1BTQ0\_CHICK tr|F1NEF2|F1NEF2\_CHICK tr|E1C190|E1C190\_CHICK sp|Q5ZL00|EMC1\_CHICK tr|E1C5A9|E1C5A9\_CHICK sp|Q5ZJF6|DDX10\_CHICK tr|F1P0I3|F1P0I3\_CHICK sp|O93353|HXA3\_CHICK tr|Q2L4W8|Q2L4W8\_CHICK tr|J9QRM9|J9QRM9\_9SAUR tr|F1P320|F1P320\_CHICK tr|F1NH45|F1NH45\_CHICK tr|Q90796|Q90796\_CHICK sp|Q8QGH3|CAD20\_CHICK tr|E1BU31|E1BU31\_CHICK tr|E1BZW2|E1BZW2\_CHICK tr|F1P4S6|F1P4S6\_CHICK tr|R4GID4|R4GID4\_CHICK tr|F1NMN4|F1NMN4\_CHICK sp|P49578|ACM3\_CHICK tr|Q5XNV3|Q5XNV3\_CHICK tr|F1P195|F1P195\_CHICK tr|Q5F3N7|Q5F3N7\_CHICK tr|F1P2S9|F1P2S9\_CHICK sp|Q9DER7|TLL1\_CHICK tr|E1C930|E1C930\_CHICK tr|F1P4E2|F1P4E2\_CHICK tr|E1C282|E1C282\_CHICK tr|E1BU33|E1BU33\_CHICK tr|F1NER7|F1NER7\_CHICK sp|P13648|LMNA\_CHICK tr|F1NFT2|F1NFT2\_CHICK tr|F6R3C4|F6R3C4\_CHICK tr|Q5F3Q7|Q5F3Q7\_CHICK tr|E5DEF2|E5DEF2\_CHICK tr|B1P863|B1P863\_CHICK tr|E1C0J8|E1C0J8\_CHICK tr|F1NWZ3|F1NWZ3\_CHICK sp|Q5ZKN3|PHTF2\_CHICK tr|F1NXV0|F1NXV0\_CHICK tr|E1BXZ4|E1BXZ4\_CHICK tr|R4GGL3|R4GGL3\_CHICK tr|E1BUD7|E1BUD7\_CHICK tr|D2X2H3|D2X2H3\_CHICK tr|F1NH21|F1NH21\_CHICK sp|Q5ZLF0|F10A1\_CHICK tr|D3WGK9|D3WGK9\_CHICK tr|R4GM65|R4GM65\_CHICK O76014 tr|F1NQI5|F1NQI5\_CHICK tr|Q5ZJ86|Q5ZJ86\_CHICK sp|Q58NQ4|FOXP1\_CHICK tr|E1C7Z3|E1C7Z3\_CHICK tr|B6V3I0|B6V3I0\_CHICK tr|Q9I9C6|Q9I9C6\_CHICK tr|E1BR53|E1BR53\_CHICK tr|F1P1W9|F1P1W9\_CHICK tr|Q7SZH6|Q7SZH6\_CHICK tr|E1BVU2|E1BVU2\_CHICK tr|F1NS20|F1NS20\_CHICK tr|E1AVU6|E1AVU6\_CHICK tr|B1AC67|B1AC67\_CHICK tr|Q98SS6|Q98SS6\_CHICK tr|F1NFY3|F1NFY3\_CHICK tr|R4GJ00|R4GJ00\_CHICK tr|F1NFW6|F1NFW6\_CHICK tr|E1C4Y9|E1C4Y9\_CHICK tr|F1NUZ8|F1NUZ8\_CHICK tr|F1NIM2|F1NIM2\_CHICK tr|Q9PTH2|Q9PTH2\_CHICK tr|Q6PTW9|Q6PTW9\_CHICK tr|F1NRZ6|F1NRZ6\_CHICK tr|E1BYQ1|E1BYQ1\_CHICK tr|Q5F3J8|Q5F3J8\_CHICK tr|E1BZE2|E1BZE2\_CHICK tr|E1C890|E1C890\_CHICK tr|Q5ZIS6|Q5ZIS6\_CHICK tr|F1NPA3|F1NPA3\_CHICK tr|Q1G7H0|Q1G7H0\_CHICK tr|H9L1F4|H9L1F4\_CHICK tr|E1C988|E1C988\_CHICK tr|R4GK09|R4GK09\_CHICK tr|F1NCA1|F1NCA1\_CHICK tr|F1NTC6|F1NTC6\_CHICK tr|F1P0D5|F1P0D5\_CHICK tr|E1BQV8|E1BQV8\_CHICK tr|F1NW00|F1NW00\_CHICK tr|N0GS47|N0GS47\_9SAUR tr|F1NHV5|F1NHV5\_CHICK tr|F1NTH4|F1NTH4\_CHICK tr|E1C882|E1C882\_CHICK tr|F1NC32|F1NC32\_CHICK tr|Q5KTI9|Q5KTI9\_CHICK P17697 tr|F1NEN5|F1NEN5\_CHICK tr|E1BY05|E1BY05\_CHICK tr|Q5ZML8|Q5ZML8\_CHICK tr|F1NN37|F1NN37\_CHICK tr|F1NG20|F1NG20\_CHICK tr|F1CN50|F1CN50\_CHICK sp|P01012|OVAL\_CHICK tr|R9PXP5|R9PXP5\_CHICK tr|Q1EGJ8|Q1EGJ8\_CHICK tr|R4GI66|R4GI66\_CHICK tr|F1NS72|F1NS72\_CHICK tr|B2Z9X8|B2Z9X8\_CHICK tr|F1NL74|F1NL74\_CHICK tr|C4PC99|C4PC99\_GALLA tr|Q5ZJ99|Q5ZJ99\_CHICK tr|F1NWD9|F1NWD9\_CHICK tr|E1BQL5|E1BQL5\_CHICK tr|F1NX24|F1NX24\_CHICK sp|Q66PG4|LARG2\_CHICK tr|E1C472|E1C472\_CHICK tr|Q5F455|Q5F455\_CHICK tr|F1NX05|F1NX05\_CHICK tr|R4GKW6|R4GKW6\_CHICK sp|P08250|APOA1\_CHICK tr|Q5F3U1|Q5F3U1\_CHICK tr|F1NID0|F1NID0\_CHICK tr|Q5ZMW2|Q5ZMW2\_CHICK tr|Q5ZIM1|Q5ZIM1\_CHICK tr|F1NW77|F1NW77\_CHICK tr|F1NTF6|F1NTF6\_CHICK tr|F1NF68|F1NF68\_CHICK tr|F1NN99|F1NN99\_CHICK tr|D3WGL0|D3WGL0\_CHICK tr|D3WGL1|D3WGL1\_CHICK tr|F1NCT8|F1NCT8\_CHICK tr|E1BQV7|E1BQV7\_CHICK tr|F1NG03|F1NG03\_CHICK tr|E1BX59|E1BX59\_CHICK tr|Q8AWW1|Q8AWW1\_CHICK tr|R4GFV0|R4GFV0\_CHICK tr|F1NEY2|F1NEY2\_CHICK tr|B3XZF5|B3XZF5\_CHICK tr|B0F2C2|B0F2C2\_CHICK tr|F1P1C6|F1P1C6\_CHICK tr|F1NZN9|F1NZN9\_CHICK tr|F1P067|F1P067\_CHICK sp|Q5F489|TAF3\_CHICK tr|F1NZW0|F1NZW0\_CHICK tr|Q8AWW2|Q8AWW2\_CHICK tr|F1P1D3|F1P1D3\_CHICK tr|R4GG35|R4GG35\_CHICK tr|F1NA14|F1NA14\_CHICK tr|Q2TLY9|Q2TLY9\_CHICK tr|E1C9D2|E1C9D2\_CHICK tr|F6S9J7|F6S9J7\_CHICK tr|Q5ZLJ6|Q5ZLJ6\_CHICK sp|Q07496|EPHA4\_CHICK tr|Q9PW08|Q9PW08\_CHICK tr|E1BRJ3|E1BRJ3\_CHICK tr|Q5ZKL0|Q5ZKL0\_CHICK sp|Q5QJC4|DCR1A\_CHICK tr|F1NPW7|F1NPW7\_CHICK tr|Q5ZJB3|Q5ZJB3\_CHICK tr|F1N9U3|F1N9U3\_CHICK tr|A0ZXL2|A0ZXL2\_CHICK tr|B0F2C3|B0F2C3\_CHICK sp|P21868|CSK21\_CHICK tr|E1C1E9|E1C1E9\_CHICK tr|F1P069|F1P069\_CHICK tr|E1C7L2|E1C7L2\_CHICK tr|F1NGY1|F1NGY1\_CHICK tr|E1C093|E1C093\_CHICK tr|Q6A2A8|Q6A2A8\_CHICK tr|E1C2F5|E1C2F5\_CHICK tr|E1BVF7|E1BVF7\_CHICK sp|O42422|EPHA7\_CHICK tr|F1P110|F1P110\_CHICK tr|E1BT78|E1BT78\_CHICK tr|F1NP44|F1NP44\_CHICK tr|F1NDK2|F1NDK2\_CHICK tr|F1NZS8|F1NZS8\_CHICK tr|Q8AWE3|Q8AWE3\_CHICK tr|E1C8P4|E1C8P4\_CHICK tr|E1BQE1|E1BQE1\_CHICK tr|P79793|P79793\_CHICK sp|Q5ZID5|UBP28\_CHICK tr|F1NUZ5|F1NUZ5\_CHICK tr|F1NLA2|F1NLA2\_CHICK tr|E1C6U7|E1C6U7\_CHICK tr|Q5ZIX2|Q5ZIX2\_CHICK tr|E1C6W9|E1C6W9\_CHICK tr|F1NKH3|F1NKH3\_CHICK tr|E1BQJ0|E1BQJ0\_CHICK tr|E1C6G9|E1C6G9\_CHICK tr|F1P2P4|F1P2P4\_CHICK tr|F1NIF1|F1NIF1\_CHICK tr|E1C470|E1C470\_CHICK tr|F1NPY9|F1NPY9\_CHICK tr|E1BQX9|E1BQX9\_CHICK tr|E1C8U8|E1C8U8\_CHICK tr|F1NTS1|F1NTS1\_CHICK tr|F1NYL2|F1NYL2\_CHICK sp|O42409|GFI1B\_CHICK tr|A2SWM7|A2SWM7\_CHICK tr|F7B5S0|F7B5S0\_CHICK tr|Q5ZJZ0|Q5ZJZ0\_CHICK tr|Q71SG0|Q71SG0\_CHICK tr|F1NQ08|F1NQ08\_CHICK tr|Q5ZI90|Q5ZI90\_CHICK tr|E1BYJ2|E1BYJ2\_CHICK tr|Q5ZL71|Q5ZL71\_CHICK tr|F1NTH5|F1NTH5\_CHICK tr|Q5ZKR8|Q5ZKR8\_CHICK tr|W5XTK2|W5XTK2\_CHICK tr|O42402|O42402\_CHICK tr|E1BSE6|E1BSE6\_CHICK tr|E1BQG5|E1BQG5\_CHICK tr|E1BWH5|E1BWH5\_CHICK tr|F1NY82|F1NY82\_CHICK tr|F1NXH2|F1NXH2\_CHICK tr|F1NJ89|F1NJ89\_CHICK tr|F1N9Q3|F1N9Q3\_CHICK tr|F1NQU4|F1NQU4\_CHICK tr|R4GF49|R4GF49\_CHICK tr|Q2VB18|Q2VB18\_CHICK tr|E1C0S3|E1C0S3\_CHICK tr|F1NQG9|F1NQG9\_CHICK tr|F1NV23|F1NV23\_CHICK tr|F1NSD9|F1NSD9\_CHICK Q3SZ57 Q6ISB0 Q9NSB2 tr|E1BSG7|E1BSG7\_CHICK tr|R4GGC3|R4GGC3\_CHICK sp|P57788|MOT4\_CHICK tr|F1P0V5|F1P0V5\_CHICK tr|F1NJG6|F1NJG6\_CHICK tr|E1C2T1|E1C2T1\_CHICK tr|Q5ZHM8|Q5ZHM8\_CHICK tr|R4GFW3|R4GFW3\_CHICK tr|F1NC49|F1NC49\_CHICK tr|F1NTS4|F1NTS4\_CHICK tr|A0A0C5ANN4|A0A0C5ANN4\_CHICK sp|Q9W6S3|SIN1\_CHICK tr|Q08I97|Q08I97\_CHICK tr|D3X743|D3X743\_CHICK tr|F1N871|F1N871\_CHICK tr|F1P3C2|F1P3C2\_CHICK tr|E1C6G3|E1C6G3\_CHICK tr|Q335P7|Q335P7\_CHICK tr|E1C623|E1C623\_CHICK tr|E1BTG0|E1BTG0\_CHICK tr|R4GHI2|R4GHI2\_CHICK tr|E1BT34|E1BT34\_CHICK tr|F1NBK0|F1NBK0\_CHICK tr|Q5ZHY0|Q5ZHY0\_CHICK sp|Q90762|CADH6\_CHICK tr|E1BQV6|E1BQV6\_CHICK tr|E1C4S8|E1C4S8\_CHICK tr|F1NAQ9|F1NAQ9\_CHICK tr|R4GKK2|R4GKK2\_CHICK tr|E1C3Z8|E1C3Z8\_CHICK sp|Q5ZLE9|N4BP1\_CHICK tr|R4GK29|R4GK29\_CHICK tr|B8XXD3|B8XXD3\_CHICK sp|P42683|LCK\_CHICK tr|F1NI51|F1NI51\_CHICK tr|F1NAK6|F1NAK6\_CHICK sp|O93295|ENTP8\_CHICK tr|F1P266|F1P266\_CHICK tr|Q4AEJ2|Q4AEJ2\_CHICK tr|E1BUH7|E1BUH7\_CHICK tr|R4GI17|R4GI17\_CHICK tr|F1NZE1|F1NZE1\_CHICK tr|F1NLE8|F1NLE8\_CHICK tr|Q5W4S8|Q5W4S8\_CHICK tr|F1NNC7|F1NNC7\_CHICK tr|F1NCK3|F1NCK3\_CHICK tr|Q5ZLL2|Q5ZLL2\_CHICK tr|F1NYT9|F1NYT9\_CHICK tr|F1N9L7|F1N9L7\_CHICK tr|H9L159|H9L159\_CHICK tr|Q6A2A9|Q6A2A9\_CHICK tr|E1C976|E1C976\_CHICK tr|E1BSC1|E1BSC1\_CHICK tr|F1NAL0|F1NAL0\_CHICK tr|E1C2D3|E1C2D3\_CHICK tr|F1NXD8|F1NXD8\_CHICK tr|F1NVN3|F1NVN3\_CHICK tr|E1C4Z7|E1C4Z7\_CHICK tr|R4GH03|R4GH03\_CHICK tr|A5Y5L6|A5Y5L6\_CHICK tr|F1P179|F1P179\_CHICK tr|Q6JGT7|Q6JGT7\_CHICK tr|B5BSC1|B5BSC1\_CHICK tr|B5BSD5|B5BSD5\_CHICK tr|H9L093|H9L093\_CHICK Q0VCM5 tr|Q98UI4|Q98UI4\_CHICK sp|Q5ZMR9|XPO4\_CHICK tr|E1C2B7|E1C2B7\_CHICK tr|F1NP35|F1NP35\_CHICK tr|F1NIP6|F1NIP6\_CHICK tr|R4GH23|R4GH23\_CHICK tr|Q5ZHY2|Q5ZHY2\_CHICK tr|E1C3S5|E1C3S5\_CHICK tr|Q5DUF3|Q5DUF3\_CHICK tr|E1C1H8|E1C1H8\_CHICK sp|Q5ZLV4|NSUN2\_CHICK tr|Q9DEG4|Q9DEG4\_CHICK tr|E1BQV5|E1BQV5\_CHICK tr|E1BT18|E1BT18\_CHICK sp|Q5F389|WWOX\_CHICK sp|Q9IA88|SIK2\_CHICK tr|A8IKD3|A8IKD3\_CHICK tr|F1NQ77|F1NQ77\_CHICK tr|F1NJE8|F1NJE8\_CHICK tr|R4GKH8|R4GKH8\_CHICK tr|F1NWA6|F1NWA6\_CHICK tr|E1BSP4|E1BSP4\_CHICK tr|R4GM14|R4GM14\_CHICK tr|E1BQI8|E1BQI8\_CHICK tr|Q5F3J7|Q5F3J7\_CHICK tr|F1NJ00|F1NJ00\_CHICK tr|E1BZ97|E1BZ97\_CHICK tr|F1NUF8|F1NUF8\_CHICK tr|E1BZP2|E1BZP2\_CHICK tr|Q9W737|Q9W737\_CHICK tr|V9GW33|V9GW33\_CHICK tr|E1BS58|E1BS58\_CHICK tr|Q5ZLR8|Q5ZLR8\_CHICK tr|R4R035|R4R035\_CHICK tr|F1NIQ4|F1NIQ4\_CHICK tr|E1BWL2|E1BWL2\_CHICK tr|E1BSP8|E1BSP8\_CHICK tr|F1NEQ3|F1NEQ3\_CHICK tr|F1NZ38|F1NZ38\_CHICK tr|A4L9I7|A4L9I7\_CHICK tr|F1NUL5|F1NUL5\_CHICK tr|E1C7H7|E1C7H7\_CHICK tr|E1BVS1|E1BVS1\_CHICK tr|F1NQ59|F1NQ59\_CHICK tr|E1BSJ9|E1BSJ9\_CHICK tr|F1N9E6|F1N9E6\_CHICK tr|Q789E4|Q789E4\_CHICK tr|F1NMC6|F1NMC6\_CHICK tr|F1NC75|F1NC75\_CHICK sp|B3TP03|CTR2\_CHICK tr|E1BRU6|E1BRU6\_CHICK tr|F1NYL0|F1NYL0\_CHICK tr|E1C0A5|E1C0A5\_CHICK tr|Q90855|Q90855\_CHICK tr|E1C8I8|E1C8I8\_CHICK tr|F1NYM4|F1NYM4\_CHICK tr|F1NZN8|F1NZN8\_CHICK sp|Q5ZJF3|TAD2A\_CHICK tr|E1BU00|E1BU00\_CHICK tr|E1BT45|E1BT45\_CHICK tr|E1C3F6|E1C3F6\_CHICK tr|Q5ZMP9|Q5ZMP9\_CHICK tr|F1P5V0|F1P5V0\_CHICK tr|F1NAW1|F1NAW1\_CHICK tr|E1C3J8|E1C3J8\_CHICK tr|F1NME7|F1NME7\_CHICK sp|Q90635|DPYL2\_CHICK tr|Q71SG1|Q71SG1\_CHICK tr|E1C229|E1C229\_CHICK tr|R4GGA1|R4GGA1\_CHICK tr|F1NGN0|F1NGN0\_CHICK sp|Q5F3L1|KS6A5\_CHICK tr|G0ZS68|G0ZS68\_CHICK tr|F1NQQ1|F1NQQ1\_CHICK tr|A0A0A7MC91|A0A0A7MC91\_CHICK tr|C4PCA5|C4PCA5\_CHICK tr|C4PCC9|C4PCC9\_CHICK tr|A0A0A7MAD8|A0A0A7MAD8\_CHICK tr|C4PCB5|C4PCB5\_CHICK tr|C4PCE1|C4PCE1\_CHICK tr|E1BQI5|E1BQI5\_CHICK tr|E1C7C0|E1C7C0\_CHICK tr|E1BYI1|E1BYI1\_CHICK tr|E1C903|E1C903\_CHICK sp|Q90738|AFAP1\_CHICK tr|F1NVL2|F1NVL2\_CHICK tr|E1BQB4|E1BQB4\_CHICK tr|F1N8T2|F1N8T2\_CHICK tr|F1NTQ0|F1NTQ0\_CHICK tr|F1NT72|F1NT72\_CHICK tr|B5G557|B5G557\_CHICK tr|F1NVD1|F1NVD1\_CHICK tr|F1NC63|F1NC63\_CHICK tr|F1NT24|F1NT24\_CHICK tr|Q4GWJ6|Q4GWJ6\_GALSO tr|F1P135|F1P135\_CHICK tr|E1C3P6|E1C3P6\_CHICK tr|F1NGX0|F1NGX0\_CHICK tr|F1P020|F1P020\_CHICK tr|Q5ZJS9|Q5ZJS9\_CHICK tr|F1NH62|F1NH62\_CHICK sp|P14732|LMNB2\_CHICK tr|E1BX91|E1BX91\_CHICK tr|Q5XPI1|Q5XPI1\_CHICK tr|F1P5Q0|F1P5Q0\_CHICK tr|Q9YHW0|Q9YHW0\_CHICK tr|E1C4I2|E1C4I2\_CHICK tr|E1C217|E1C217\_CHICK tr|F1P513|F1P513\_CHICK tr|F1NHA0|F1NHA0\_CHICK tr|F1NKW8|F1NKW8\_CHICK tr|A0A088DCY4|A0A088DCY4\_CHICK tr|Q6T722|Q6T722\_CHICK tr|E1BQP2|E1BQP2\_CHICK tr|F1NNW3|F1NNW3\_CHICK tr|F1NC73|F1NC73\_CHICK tr|I6ZIP5|I6ZIP5\_CHICK tr|R4GFG9|R4GFG9\_CHICK tr|E1BX45|E1BX45\_CHICK tr|F1NG81|F1NG81\_CHICK tr|F6T808|F6T808\_CHICK tr|E1C2S9|E1C2S9\_CHICK tr|Q6XD56|Q6XD56\_CHICK tr|F1NYS2|F1NYS2\_CHICK tr|F1NJ43|F1NJ43\_CHICK sp|Q5ZIA2|MAP7\_CHICK tr|F1NRI3|F1NRI3\_CHICK sp|O93277|WDR1\_CHICK tr|Q5F3K6|Q5F3K6\_CHICK tr|R4GGK2|R4GGK2\_CHICK tr|E1BWX4|E1BWX4\_CHICK tr|U3M5R3|U3M5R3\_CHICK tr|U3M6H4|U3M6H4\_CHICK sp|Q9PWA3|GRIN2\_CHICK tr|E1C296|E1C296\_CHICK tr|F1NY02|F1NY02\_CHICK tr|R4GLD8|R4GLD8\_CHICK tr|F1NGR0|F1NGR0\_CHICK tr|A0A0D5ZDC1|A0A0D5ZDC1\_9VIRU tr|E1C6A7|E1C6A7\_CHICK tr|F1NGI5|F1NGI5\_CHICK tr|E1C4M7|E1C4M7\_CHICK tr|E1C608|E1C608\_CHICK tr|F1NBJ7|F1NBJ7\_CHICK tr|F1NHC6|F1NHC6\_CHICK tr|R4GII8|R4GII8\_CHICK sp|Q5F4A1|G2E3\_CHICK H-INV:HIT000292931 tr|F1NDD2|F1NDD2\_CHICK tr|F1NF83|F1NF83\_CHICK tr|R4GJ32|R4GJ32\_CHICK tr|F1NFQ2|F1NFQ2\_CHICK sp|Q5ZJL5|PURA2\_CHICK tr|E1BWW7|E1BWW7\_CHICK tr|F1NDU2|F1NDU2\_CHICK tr|A0A0A0V9I6|A0A0A0V9I6\_CHICK tr|F1NMB7|F1NMB7\_CHICK sp|Q9PTU5|ESR2\_CHICK tr|F1NIB0|F1NIB0\_CHICK tr|E1C7I8|E1C7I8\_CHICK tr|R4GFL1|R4GFL1\_CHICK tr|E1C8J8|E1C8J8\_CHICK tr|E1C8E2|E1C8E2\_CHICK tr|F1NGN6|F1NGN6\_CHICK tr|F1NZI2|F1NZI2\_CHICK sp|P79777|BRAC\_CHICK tr|A0A0D5ZDA3|A0A0D5ZDA3\_9VIRU tr|R4GFN5|R4GFN5\_CHICK sp|Q5ZJ54|TCPZ\_CHICK tr|F1NWH9|F1NWH9\_CHICK tr|F1NB50|F1NB50\_CHICK tr|Q5TMB6|Q5TMB6\_CHICK tr|E1C3P5|E1C3P5\_CHICK tr|E1BQJ2|E1BQJ2\_CHICK tr|D3XFI0|D3XFI0\_CHICK tr|Q5ZL27|Q5ZL27\_CHICK tr|F1P088|F1P088\_CHICK tr|F1NEM2|F1NEM2\_CHICK tr|E1BWH8|E1BWH8\_CHICK tr|F1NMG9|F1NMG9\_CHICK tr|Q9DGH8|Q9DGH8\_CHICK tr|Q5ZK89|Q5ZK89\_CHICK tr|F1NBB2|F1NBB2\_CHICK sp|P24343|HXD12\_CHICK tr|L7NTB9|L7NTB9\_CHICK sp|O57415|RREB1\_CHICK tr|F1NTQ4|F1NTQ4\_CHICK tr|F1NVF6|F1NVF6\_CHICK tr|Q5F3F6|Q5F3F6\_CHICK tr|F1P2G8|F1P2G8\_CHICK tr|Q9I9C5|Q9I9C5\_CHICK tr|F1P5N4|F1P5N4\_CHICK tr|E1C6Z2|E1C6Z2\_CHICK tr|F1NXK4|F1NXK4\_CHICK tr|F1P3B9|F1P3B9\_CHICK tr|H9KZU2|H9KZU2\_CHICK tr|F1P490|F1P490\_CHICK tr|E1C3U5|E1C3U5\_CHICK tr|E1BZD8|E1BZD8\_CHICK tr|E1C6R7|E1C6R7\_CHICK sp|Q5F471|PP6R3\_CHICK tr|Q9I8V6|Q9I8V6\_CHICK tr|F1NDH9|F1NDH9\_CHICK sp|Q90663|SEM3D\_CHICK tr|M1SWX9|M1SWX9\_CHICK tr|F1NJ51|F1NJ51\_CHICK tr|Q5ZFP4|Q5ZFP4\_CHICK tr|B5BSN2|B5BSN2\_CHICK tr|F1NHA5|F1NHA5\_CHICK tr|B5BSA7|B5BSA7\_CHICK tr|B5BST6|B5BST6\_CHICK tr|R4GJE8|R4GJE8\_CHICK tr|F1NN93|F1NN93\_CHICK tr|Q52P71|Q52P71\_CHICK tr|E1BVL6|E1BVL6\_CHICK sp|Q5ZJS0|KC1G1\_CHICK sp|Q5ZM39|BCL6\_CHICK tr|F1NUQ6|F1NUQ6\_CHICK tr|F1NB98|F1NB98\_CHICK tr|E1C8W4|E1C8W4\_CHICK tr|F1NJA1|F1NJA1\_CHICK sp|Q7ZZR3|BRNP1\_CHICK tr|F1NYZ0|F1NYZ0\_CHICK tr|F1NQT4|F1NQT4\_CHICK tr|F1NS89|F1NS89\_CHICK tr|A5HUL5|A5HUL5\_CHICK tr|Q90971|Q90971\_CHICK tr|B5BST0|B5BST0\_CHICK tr|B5BSB5|B5BSB5\_CHICK tr|Q705C2|Q705C2\_CHICK tr|R4GGS7|R4GGS7\_CHICK tr|F1NV17|F1NV17\_CHICK tr|E1BTB8|E1BTB8\_CHICK tr|F1NWK9|F1NWK9\_CHICK tr|K9JTR8|K9JTR8\_CHICK tr|F1NGY5|F1NGY5\_CHICK tr|E1C837|E1C837\_CHICK tr|R4GGZ7|R4GGZ7\_CHICK tr|F1NRB3|F1NRB3\_CHICK tr|Q5ZJ33|Q5ZJ33\_CHICK tr|F1P0P4|F1P0P4\_CHICK tr|F1NPU7|F1NPU7\_CHICK tr|E1BT12|E1BT12\_CHICK tr|F1NE64|F1NE64\_CHICK Q28085 tr|E1BW83|E1BW83\_CHICK tr|R4GJP5|R4GJP5\_CHICK tr|F1NFM2|F1NFM2\_CHICK tr|Q9PTD3|Q9PTD3\_CHICK tr|E1BX29|E1BX29\_CHICK tr|E1C396|E1C396\_CHICK tr|F1NH54|F1NH54\_CHICK tr|E1C7U7|E1C7U7\_CHICK tr|E1BXL8|E1BXL8\_CHICK tr|E1C0H5|E1C0H5\_CHICK tr|F1NAG2|F1NAG2\_CHICK tr|F1N8M7|F1N8M7\_CHICK tr|Q8JG31|Q8JG31\_CHICK tr|Q5ZME3|Q5ZME3\_CHICK tr|R4GJJ9|R4GJJ9\_CHICK tr|R4GHJ7|R4GHJ7\_CHICK tr|F1P459|F1P459\_CHICK tr|E1BYT8|E1BYT8\_CHICK tr|E1BQ83|E1BQ83\_CHICK tr|F1P081|F1P081\_CHICK tr|E1BRT1|E1BRT1\_CHICK tr|V9GVP4|V9GVP4\_CHICK tr|F1NHM0|F1NHM0\_CHICK tr|F1NAM4|F1NAM4\_CHICK tr|F1NGP3|F1NGP3\_CHICK tr|F1NBX5|F1NBX5\_CHICK tr|F1P4I6|F1P4I6\_CHICK tr|E1C0L3|E1C0L3\_CHICK tr|E1C510|E1C510\_CHICK tr|F1NTK9|F1NTK9\_CHICK tr|E1BWH9|E1BWH9\_CHICK tr|F1NLU9|F1NLU9\_CHICK tr|O73665|O73665\_CHICK tr|Q5TLZ3|Q5TLZ3\_CHICK sp|Q6DV79|STAT3\_CHICK sp|Q5ZLR4|ESRP2\_CHICK tr|H9L3N4|H9L3N4\_CHICK tr|E1BTX5|E1BTX5\_CHICK tr|E1C8A0|E1C8A0\_CHICK tr|F1NG90|F1NG90\_CHICK tr|F1P4Q6|F1P4Q6\_CHICK tr|Q8AY28|Q8AY28\_CHICK sp|P02460|CO2A1\_CHICK tr|E1C841|E1C841\_CHICK tr|F1P4Y5|F1P4Y5\_CHICK sp|P22770|ACHA7\_CHICK tr|W0GHX2|W0GHX2\_CHICK tr|F1ND05|F1ND05\_CHICK tr|F1NFS8|F1NFS8\_CHICK tr|F1NVH8|F1NVH8\_CHICK sp|Q8JGM4|QSOX1\_CHICK tr|F1P3C5|F1P3C5\_CHICK sp|Q90734|ACTN4\_CHICK tr|F1N9U1|F1N9U1\_CHICK sp|Q9I9E0|TAOK3\_CHICK tr|R4GMH3|R4GMH3\_CHICK tr|Q4GWM2|Q4GWM2\_GALVA tr|I0J178|I0J178\_CHICK sp|P01014|OVALY\_CHICK tr|A7XMT6|A7XMT6\_CHICK tr|F1NIA0|F1NIA0\_CHICK tr|E1C0I8|E1C0I8\_CHICK tr|F1NNH6|F1NNH6\_CHICK tr|F1NUB2|F1NUB2\_CHICK tr|E1BWT5|E1BWT5\_CHICK tr|E1BVE9|E1BVE9\_CHICK tr|F1NSR6|F1NSR6\_CHICK tr|F1NMY1|F1NMY1\_CHICK tr|E1BQP9|E1BQP9\_CHICK tr|F1NCH8|F1NCH8\_CHICK tr|F1NKN6|F1NKN6\_CHICK tr|F1NXS9|F1NXS9\_CHICK tr|Q9DDZ6|Q9DDZ6\_CHICK tr|F1NNK9|F1NNK9\_CHICK tr|H9KZK0|H9KZK0\_CHICK sp|Q801X6|PHAR1\_CHICK tr|F1NWB1|F1NWB1\_CHICK tr|E1BVI5|E1BVI5\_CHICK tr|F5ANP1|F5ANP1\_CHICK tr|E1BWV9|E1BWV9\_CHICK tr|F1N8B4|F1N8B4\_CHICK tr|F1NHB9|F1NHB9\_CHICK tr|F1NL67|F1NL67\_CHICK tr|F1P575|F1P575\_CHICK tr|F1NPL2|F1NPL2\_CHICK tr|F1NGM5|F1NGM5\_CHICK Q2KJC7 tr|R4GM87|R4GM87\_CHICK tr|F1NG68|F1NG68\_CHICK tr|Q5ZLP0|Q5ZLP0\_CHICK tr|E1C843|E1C843\_CHICK tr|F1N974|F1N974\_CHICK tr|A4ZZ64|A4ZZ64\_CHICK tr|F1NAE2|F1NAE2\_CHICK tr|E1C4W1|E1C4W1\_CHICK tr|F1NF77|F1NF77\_CHICK tr|F1NE37|F1NE37\_CHICK sp|D0PRN4|NRX3B\_CHICK tr|F1NPI6|F1NPI6\_CHICK tr|B5BSG3|B5BSG3\_CHICK tr|E1C3S2|E1C3S2\_CHICK tr|B5BSS2|B5BSS2\_CHICK tr|F5CST3|F5CST3\_CHICK tr|R4GL97|R4GL97\_CHICK tr|F1NG78|F1NG78\_CHICK tr|F1P2V4|F1P2V4\_CHICK tr|E1C3Y4|E1C3Y4\_CHICK tr|Q6XQM9|Q6XQM9\_CHICK tr|E1BZK6|E1BZK6\_CHICK tr|R4GKH3|R4GKH3\_CHICK A3EZ82 tr|F1P0U3|F1P0U3\_CHICK tr|E1BSC0|E1BSC0\_CHICK tr|E1C4E7|E1C4E7\_CHICK tr|B3VKP9|B3VKP9\_CHICK tr|B3GSV6|B3GSV6\_CHICK tr|H9L1B3|H9L1B3\_CHICK sp|Q5ZKL9|PARI\_CHICK tr|R4GKW5|R4GKW5\_CHICK tr|H9L3V7|H9L3V7\_CHICK tr|R4GH57|R4GH57\_CHICK tr|F1NI43|F1NI43\_CHICK tr|R4GFY4|R4GFY4\_CHICK tr|F1NY12|F1NY12\_CHICK tr|F1NA60|F1NA60\_CHICK tr|Q60GM0|Q60GM0\_CHICK tr|F1N8X5|F1N8X5\_CHICK sp|Q9I8T7|BMAL1\_CHICK tr|F1P5G5|F1P5G5\_CHICK tr|E1BQZ4|E1BQZ4\_CHICK tr|F1NE32|F1NE32\_CHICK tr|F1NFB1|F1NFB1\_CHICK tr|F1NX63|F1NX63\_CHICK tr|E1BV75|E1BV75\_CHICK tr|E1C828|E1C828\_CHICK tr|E1C765|E1C765\_CHICK sp|Q9I8D0|VPP1\_CHICK tr|E1C8Q5|E1C8Q5\_CHICK tr|F1NPR3|F1NPR3\_CHICK tr|E1C0Q5|E1C0Q5\_CHICK tr|C4PCB8|C4PCB8\_CHICK tr|F1NPS6|F1NPS6\_CHICK tr|B5BSG2|B5BSG2\_CHICK tr|F1NU18|F1NU18\_CHICK tr|E1C8V4|E1C8V4\_CHICK tr|E1BXH4|E1BXH4\_CHICK sp|Q5ZLG0|AACS\_CHICK tr|E1C3Q6|E1C3Q6\_CHICK tr|E1C4J9|E1C4J9\_CHICK tr|F1N880|F1N880\_CHICK tr|E1C9E4|E1C9E4\_CHICK tr|F5CSS6|F5CSS6\_CHICK tr|B5BSK4|B5BSK4\_CHICK tr|B5BSS1|B5BSS1\_CHICK tr|B5BSD4|B5BSD4\_CHICK tr|F5CST1|F5CST1\_CHICK tr|A5HUM0|A5HUM0\_CHICK tr|Q5ZFP5|Q5ZFP5\_CHICK tr|Q5F3Z8|Q5F3Z8\_CHICK tr|Q5F406|Q5F406\_CHICK tr|R4GGB0|R4GGB0\_CHICK tr|T1P426|T1P426\_CHICK sp|Q5ZKG8|GET4\_CHICK tr|F1P192|F1P192\_CHICK tr|E1BRT7|E1BRT7\_CHICK tr|F1NP62|F1NP62\_CHICK tr|E5DEJ5|E5DEJ5\_CHICK tr|Q6R0I2|Q6R0I2\_CHICK tr|B4XN22|B4XN22\_CHICK tr|H9L092|H9L092\_CHICK tr|Q5F3A5|Q5F3A5\_CHICK tr|E1C501|E1C501\_CHICK tr|Q5QGM3|Q5QGM3\_CHICK tr|F1NB52|F1NB52\_CHICK tr|Q5F402|Q5F402\_CHICK tr|E1BQJ9|E1BQJ9\_CHICK sp|Q5ZIW2|CNO10\_CHICK tr|Q8AV87|Q8AV87\_CHICK sp|Q5F362|CC50A\_CHICK tr|F1NX30|F1NX30\_CHICK tr|R4GJ75|R4GJ75\_CHICK sp|Q5F413|SMAP2\_CHICK tr|E1BRY6|E1BRY6\_CHICK tr|E1BUG0|E1BUG0\_CHICK tr|F1P232|F1P232\_CHICK tr|E1BYR9|E1BYR9\_CHICK tr|E1C8L5|E1C8L5\_CHICK tr|F1NAD5|F1NAD5\_CHICK tr|E1BS72|E1BS72\_CHICK tr|Q90XB9|Q90XB9\_CHICK tr|F1NVX3|F1NVX3\_CHICK sp|Q8AWC7|FUT11\_CHICK tr|F1P0A7|F1P0A7\_CHICK tr|F1NIT1|F1NIT1\_CHICK tr|Q6EE33|Q6EE33\_CHICK tr|F1NIG5|F1NIG5\_CHICK sp|Q5F468|S38A2\_CHICK tr|E1C1S1|E1C1S1\_CHICK tr|Q9PW23|Q9PW23\_CHICK tr|F1NX93|F1NX93\_CHICK sp|Q91044|NTRK3\_CHICK tr|E1BTY2|E1BTY2\_CHICK tr|F1NZY8|F1NZY8\_CHICK tr|E1BXF3|E1BXF3\_CHICK tr|F1NLQ1|F1NLQ1\_CHICK tr|O57534|O57534\_CHICK tr|E1BSX7|E1BSX7\_CHICK tr|F1NQC8|F1NQC8\_CHICK tr|E1BWM7|E1BWM7\_CHICK tr|F1NF58|F1NF58\_CHICK tr|F1NBM4|F1NBM4\_CHICK tr|A0ZY64|A0ZY64\_CHICK tr|A7E3L6|A7E3L6\_CHICK tr|F1NX83|F1NX83\_CHICK tr|E1BXH7|E1BXH7\_CHICK tr|R4GI09|R4GI09\_CHICK tr|E1C2K7|E1C2K7\_CHICK ENSEMBL:ENSBTAP00000018229 tr|E1BTX4|E1BTX4\_CHICK tr|Q335Q1|Q335Q1\_CHICK tr|F1NSS9|F1NSS9\_CHICK tr|Q90ZI2|Q90ZI2\_CHICK tr|H9KZJ4|H9KZJ4\_CHICK tr|Q9PVL6|Q9PVL6\_CHICK tr|F1NZB6|F1NZB6\_CHICK tr|R4GGZ1|R4GGZ1\_CHICK tr|F1NRL8|F1NRL8\_CHICK tr|Q90Z54|Q90Z54\_CHICK tr|F1NTF8|F1NTF8\_CHICK tr|E1BQT7|E1BQT7\_CHICK tr|F1NX74|F1NX74\_CHICK tr|E1C5J5|E1C5J5\_CHICK tr|Q5F4A4|Q5F4A4\_CHICK tr|E1C876|E1C876\_CHICK tr|R4GL09|R4GL09\_CHICK tr|M1XGZ4|M1XGZ4\_CHICK tr|F1NB45|F1NB45\_CHICK tr|F1NN68|F1NN68\_CHICK tr|F1NLW2|F1NLW2\_CHICK tr|H9KZ68|H9KZ68\_CHICK tr|F1NZF3|F1NZF3\_CHICK tr|F1P1U6|F1P1U6\_CHICK tr|Q9PW49|Q9PW49\_CHICK sp|P60706|ACTB\_CHICK sp|P53478|ACT5\_CHICK sp|Q5ZMQ2|ACTG\_CHICK tr|F1NP61|F1NP61\_CHICK tr|F1NT31|F1NT31\_CHICK tr|E1C4W9|E1C4W9\_CHICK tr|F1NCB1|F1NCB1\_CHICK tr|F1P3X6|F1P3X6\_CHICK tr|F1P1M5|F1P1M5\_CHICK tr|F1NSI7|F1NSI7\_CHICK sp|P12106|CO9A1\_CHICK tr|R9PXN8|R9PXN8\_CHICK tr|F1NBK5|F1NBK5\_CHICK tr|U5KCL3|U5KCL3\_CHICK tr|U5KCB1|U5KCB1\_CHICK tr|U5KC18|U5KC18\_CHICK tr|E1C2C5|E1C2C5\_CHICK sp|Q9DE09|HMX1\_CHICK tr|Q6DKR3|Q6DKR3\_CHICK tr|F1NC43|F1NC43\_CHICK tr|E1C2T9|E1C2T9\_CHICK tr|E1C936|E1C936\_CHICK tr|E1BVP3|E1BVP3\_CHICK tr|R4GGS5|R4GGS5\_CHICK tr|E1BYT4|E1BYT4\_CHICK tr|E1BW84|E1BW84\_CHICK tr|F1N824|F1N824\_CHICK tr|F1NIK2|F1NIK2\_CHICK tr|Q5ZKF7|Q5ZKF7\_CHICK tr|E1C6M5|E1C6M5\_CHICK tr|F1P0J7|F1P0J7\_CHICK tr|F1NHU9|F1NHU9\_CHICK tr|F1NC62|F1NC62\_CHICK sp|P21872|PUR2\_CHICK tr|F1NM52|F1NM52\_CHICK sp|P16476|PEPE\_CHICK tr|F1NRQ3|F1NRQ3\_CHICK tr|K4Q4R1|K4Q4R1\_CHICK tr|Q8AV27|Q8AV27\_CHICK tr|R4GHQ5|R4GHQ5\_CHICK tr|F1NAN4|F1NAN4\_CHICK tr|Q9W6F6|Q9W6F6\_CHICK tr|E1C9I0|E1C9I0\_CHICK tr|E1BT43|E1BT43\_CHICK tr|F1NX88|F1NX88\_CHICK tr|F1P4R6|F1P4R6\_CHICK sp|P21804|FGFR1\_CHICK tr|F1NJ92|F1NJ92\_CHICK tr|F1NDU4|F1NDU4\_CHICK tr|Q9PSZ4|Q9PSZ4\_CHICK tr|F1NVW4|F1NVW4\_CHICK sp|P01103|MYB\_CHICK tr|E1BR82|E1BR82\_CHICK tr|F1NQ43|F1NQ43\_CHICK tr|E1BQS0|E1BQS0\_CHICK tr|F1N8E5|F1N8E5\_CHICK tr|F1NLG1|F1NLG1\_CHICK tr|Q6JGV3|Q6JGV3\_CHICK tr|F1NIB8|F1NIB8\_CHICK tr|A0A0C3SFZ3|A0A0C3SFZ3\_CHICK tr|R4GKG4|R4GKG4\_CHICK tr|F1NP57|F1NP57\_CHICK tr|F1NSW9|F1NSW9\_CHICK tr|F1NVL0|F1NVL0\_CHICK sp|Q9W689|ATX3\_CHICK tr|E1BQR4|E1BQR4\_CHICK tr|Q5ZK52|Q5ZK52\_CHICK tr|F1NCU7|F1NCU7\_CHICK tr|E1C155|E1C155\_CHICK tr|Q5ZHM4|Q5ZHM4\_CHICK tr|E1BUH0|E1BUH0\_CHICK tr|R4GGI7|R4GGI7\_CHICK tr|F1NXF6|F1NXF6\_CHICK tr|F1P5K6|F1P5K6\_CHICK tr|E5DFV0|E5DFV0\_CHICK tr|E5DEA4|E5DEA4\_CHICK tr|E5DEP7|E5DEP7\_CHICK tr|E5DF79|E5DF79\_CHICK tr|Q4GWN5|Q4GWN5\_CHICK tr|A0A0B4ZV97|A0A0B4ZV97\_CHICK tr|Q85BF7|Q85BF7\_CHICK tr|Q4GWS4|Q4GWS4\_GALSO tr|E5DFI3|E5DFI3\_CHICK tr|E5DFJ6|E5DFJ6\_CHICK tr|E5DEE3|E5DEE3\_CHICK tr|Q4GWP8|Q4GWP8\_CHICK tr|Q4GWR1|Q4GWR1\_CHICK tr|E5DEW2|E5DEW2\_CHICK tr|E5DEB7|E5DEB7\_CHICK tr|Q5ZKD8|Q5ZKD8\_CHICK tr|F1NE88|F1NE88\_CHICK tr|F1NWL2|F1NWL2\_CHICK tr|F1NU04|F1NU04\_CHICK tr|F1NZH6|F1NZH6\_CHICK tr|R4GL04|R4GL04\_CHICK tr|F1NMX3|F1NMX3\_CHICK tr|E1C3V3|E1C3V3\_CHICK tr|F1NA57|F1NA57\_CHICK tr|E1BTA6|E1BTA6\_CHICK tr|E1C1T3|E1C1T3\_CHICK tr|E1C2A3|E1C2A3\_CHICK tr|F1NQX8|F1NQX8\_CHICK tr|F1NGM2|F1NGM2\_CHICK tr|F1NAU2|F1NAU2\_CHICK tr|Q5F423|Q5F423\_CHICK tr|H9KZY3|H9KZY3\_CHICK tr|F1NRA9|F1NRA9\_CHICK tr|F1NQA6|F1NQA6\_CHICK tr|Q5F3Y6|Q5F3Y6\_CHICK tr|F1P398|F1P398\_CHICK tr|E1BT99|E1BT99\_CHICK tr|F1NS64|F1NS64\_CHICK tr|R4GKW1|R4GKW1\_CHICK tr|R4GHN7|R4GHN7\_CHICK tr|Q5J7V0|Q5J7V0\_CHICK tr|F1NFD7|F1NFD7\_CHICK tr|E1BT90|E1BT90\_CHICK tr|Q5ZIY0|Q5ZIY0\_CHICK tr|F1NAZ3|F1NAZ3\_CHICK tr|F1P548|F1P548\_CHICK tr|F1NSQ4|F1NSQ4\_CHICK tr|F1NYE3|F1NYE3\_CHICK tr|Q6TQF9|Q6TQF9\_CHICK sp|Q8AYS7|CENPI\_CHICK Q7RTT2 tr|G9MBR2|G9MBR2\_CHICK Q8N1N4-2 tr|F1NIL4|F1NIL4\_CHICK tr|E1C283|E1C283\_CHICK tr|B5BSK5|B5BSK5\_CHICK tr|F1ND28|F1ND28\_CHICK tr|E1C5B1|E1C5B1\_CHICK tr|Q5ZKJ8|Q5ZKJ8\_CHICK tr|F1NVZ2|F1NVZ2\_CHICK tr|F1NYV5|F1NYV5\_CHICK sp|P24503|CADH4\_CHICK tr|Q789F1|Q789F1\_CHICK tr|E1C2S3|E1C2S3\_CHICK tr|O42351|O42351\_CHICK tr|Q861N3|Q861N3\_CHICK tr|Q6JGU1|Q6JGU1\_CHICK tr|E1BW20|E1BW20\_CHICK tr|F1NTX7|F1NTX7\_CHICK tr|F1NEN0|F1NEN0\_CHICK tr|F1NKL6|F1NKL6\_CHICK tr|R4GM34|R4GM34\_CHICK tr|F1NZI7|F1NZI7\_CHICK tr|E1C3T8|E1C3T8\_CHICK tr|R4GKU8|R4GKU8\_CHICK tr|F1NQC6|F1NQC6\_CHICK tr|E1C122|E1C122\_CHICK tr|Q9W6E1|Q9W6E1\_CHICK tr|F1NRI8|F1NRI8\_CHICK tr|W0BZF4|W0BZF4\_CHICK tr|R9PXP7|R9PXP7\_CHICK tr|Q4GWK9|Q4GWK9\_GALLA tr|F1NJV1|F1NJV1\_CHICK tr|F1NYK3|F1NYK3\_CHICK tr|E1BSA3|E1BSA3\_CHICK tr|F1NBN7|F1NBN7\_CHICK sp|Q5ZLQ4|IREB2\_CHICK tr|F1NET3|F1NET3\_CHICK tr|F1NX02|F1NX02\_CHICK tr|F1P5L0|F1P5L0\_CHICK tr|E5DFT7|E5DFT7\_CHICK tr|F1NA61|F1NA61\_CHICK tr|F1NZ37|F1NZ37\_CHICK tr|Q03737|Q03737\_CHICK sp|P19098|CP19A\_CHICK tr|F1NX95|F1NX95\_CHICK tr|F1NXQ0|F1NXQ0\_CHICK sp|O73885|HSP7C\_CHICK tr|F1NAA3|F1NAA3\_CHICK tr|F1NXY5|F1NXY5\_CHICK tr|F1ND27|F1ND27\_CHICK tr|E1C259|E1C259\_CHICK tr|F1NL02|F1NL02\_CHICK tr|R4GIJ6|R4GIJ6\_CHICK tr|F1NDX6|F1NDX6\_CHICK tr|E1C6V5|E1C6V5\_CHICK tr|R4GHG8|R4GHG8\_CHICK tr|E1C358|E1C358\_CHICK sp|Q5F450|PAN2\_CHICK sp|P56734|AVR4\_CHICK tr|Q08525|Q08525\_CHICK tr|E1C525|E1C525\_CHICK tr|F1NBD2|F1NBD2\_CHICK sp|Q5ZJ85|PRPF3\_CHICK tr|H9L3J2|H9L3J2\_CHICK tr|F1NZA5|F1NZA5\_CHICK tr|C4PCC5|C4PCC5\_GALSO tr|F1NP30|F1NP30\_CHICK tr|Q5ZHU2|Q5ZHU2\_CHICK tr|Q5ZIA4|Q5ZIA4\_CHICK tr|A5A2G0|A5A2G0\_CHICK tr|Q5F3F8|Q5F3F8\_CHICK tr|F1P2U2|F1P2U2\_CHICK tr|R4GG62|R4GG62\_CHICK tr|H9L022|H9L022\_CHICK tr|F1NNW5|F1NNW5\_CHICK tr|F1NC84|F1NC84\_CHICK tr|Q4LDF5|Q4LDF5\_CHICK tr|Q70XC4|Q70XC4\_CHICK tr|H9L0E0|H9L0E0\_CHICK tr|F1NJT2|F1NJT2\_CHICK tr|F1NDY2|F1NDY2\_CHICK tr|Q5ZJT8|Q5ZJT8\_CHICK tr|F1NYH0|F1NYH0\_CHICK tr|Q5ZL60|Q5ZL60\_CHICK tr|F1NS73|F1NS73\_CHICK tr|Q98942|Q98942\_CHICK tr|F1NF53|F1NF53\_CHICK tr|F1P0L4|F1P0L4\_CHICK tr|F1NQ66|F1NQ66\_CHICK sp|Q5ZLN8|DDX55\_CHICK tr|R4GMD1|R4GMD1\_CHICK tr|E1BXP3|E1BXP3\_CHICK tr|F1NYP8|F1NYP8\_CHICK tr|F1NWM4|F1NWM4\_CHICK tr|G1EIV3|G1EIV3\_9EUCA tr|E1C3P7|E1C3P7\_CHICK tr|F1NVP7|F1NVP7\_CHICK tr|R4GHP9|R4GHP9\_CHICK sp|P28337|GCST\_CHICK tr|F1NL89|F1NL89\_CHICK sp|P02542|DESM\_CHICK tr|E1C0M6|E1C0M6\_CHICK tr|F1NX37|F1NX37\_CHICK tr|F1NPF7|F1NPF7\_CHICK tr|Q90710|Q90710\_CHICK tr|F1P0J1|F1P0J1\_CHICK tr|Q5ZIG6|Q5ZIG6\_CHICK tr|E1BR49|E1BR49\_CHICK tr|E1C442|E1C442\_CHICK tr|I6NC70|I6NC70\_CHICK tr|E1BT47|E1BT47\_CHICK tr|F1NPX3|F1NPX3\_CHICK P02672 tr|E1BTL7|E1BTL7\_CHICK tr|O42389|O42389\_CHICK tr|Q5ZJB0|Q5ZJB0\_CHICK tr|Q6RW63|Q6RW63\_CHICK tr|E1BST6|E1BST6\_CHICK tr|E1BST5|E1BST5\_CHICK tr|F1NQ32|F1NQ32\_CHICK tr|F1P581|F1P581\_CHICK tr|C4PCH2|C4PCH2\_GALSO tr|C4PCH1|C4PCH1\_GALSO tr|O42484|O42484\_CHICK tr|R4GGY2|R4GGY2\_CHICK tr|R4GM60|R4GM60\_CHICK tr|F1NZ58|F1NZ58\_CHICK tr|Q5ZJ09|Q5ZJ09\_CHICK tr|F1NXN1|F1NXN1\_CHICK tr|F1NC11|F1NC11\_CHICK tr|Q5ZK38|Q5ZK38\_CHICK tr|F1NFJ0|F1NFJ0\_CHICK tr|R4GKI6|R4GKI6\_CHICK sp|Q5ZMN2|MCM3\_CHICK tr|Q5F342|Q5F342\_CHICK sp|Q9PU53|TERF2\_CHICK tr|F1NSI5|F1NSI5\_CHICK tr|E1BRV3|E1BRV3\_CHICK tr|R4GH17|R4GH17\_CHICK sp|Q9I920|BLM\_CHICK tr|F1NYL5|F1NYL5\_CHICK tr|Q6J613|Q6J613\_CHICK tr|Q6J614|Q6J614\_CHICK tr|Q5ZJB6|Q5ZJB6\_CHICK tr|Q5ZLU5|Q5ZLU5\_CHICK tr|E1C0Z3|E1C0Z3\_CHICK tr|F1P0N2|F1P0N2\_CHICK tr|E1BZG1|E1BZG1\_CHICK tr|A0A0D9SEG7|A0A0D9SEG7\_CHICK tr|Q9PWA8|Q9PWA8\_CHICK tr|F1P4R2|F1P4R2\_CHICK tr|F1NTL6|F1NTL6\_CHICK tr|Q5ZKE2|Q5ZKE2\_CHICK tr|F1P3G1|F1P3G1\_CHICK tr|E1C7K4|E1C7K4\_CHICK tr|F1NZZ9|F1NZZ9\_CHICK tr|E1BR13|E1BR13\_CHICK tr|E1BWF7|E1BWF7\_CHICK tr|F1NGQ3|F1NGQ3\_CHICK tr|F1P1A8|F1P1A8\_CHICK tr|B2WUP2|B2WUP2\_CHICK tr|Q6IYE8|Q6IYE8\_CHICK tr|F1P0A6|F1P0A6\_CHICK tr|B8Q2V9|B8Q2V9\_CHICK tr|Q702H4|Q702H4\_CHICK tr|Q98SH6|Q98SH6\_CHICK tr|E1BUN9|E1BUN9\_CHICK tr|E1C5K2|E1C5K2\_CHICK tr|F1NX55|F1NX55\_CHICK tr|E1BYJ9|E1BYJ9\_CHICK tr|C4PCF0|C4PCF0\_GALLA tr|F1P318|F1P318\_CHICK tr|F1NLW8|F1NLW8\_CHICK tr|E1C367|E1C367\_CHICK tr|E1BR86|E1BR86\_CHICK sp|P07997|HEM1\_CHICK tr|Q2PBC0|Q2PBC0\_CHICK tr|F1NNR3|F1NNR3\_CHICK tr|F1NER2|F1NER2\_CHICK tr|E1C1V3|E1C1V3\_CHICK tr|A5Y5L7|A5Y5L7\_CHICK tr|F1P0N0|F1P0N0\_CHICK tr|E1C5G3|E1C5G3\_CHICK tr|E1BY22|E1BY22\_CHICK tr|F1NGV4|F1NGV4\_CHICK tr|Q304D3|Q304D3\_CHICK tr|Q6PKI7|Q6PKI7\_CHICK tr|F6RIS7|F6RIS7\_CHICK tr|G8HZH8|G8HZH8\_CHICK tr|G8HZH9|G8HZH9\_CHICK tr|F1NIE6|F1NIE6\_CHICK tr|G8HZI1|G8HZI1\_CHICK tr|G8HZI0|G8HZI0\_CHICK tr|F1NY57|F1NY57\_CHICK tr|H9L006|H9L006\_CHICK tr|Q5ZMK2|Q5ZMK2\_CHICK tr|F1P1V2|F1P1V2\_CHICK tr|F1NUG2|F1NUG2\_CHICK tr|F1NFF3|F1NFF3\_CHICK tr|F1NIC8|F1NIC8\_CHICK tr|F1N8U9|F1N8U9\_CHICK tr|E1BW13|E1BW13\_CHICK tr|E1BZZ7|E1BZZ7\_CHICK tr|Q7T071|Q7T071\_CHICK tr|F1NR89|F1NR89\_CHICK sp|Q5ZKZ4|DJC16\_CHICK tr|F1NEF1|F1NEF1\_CHICK tr|F1NS59|F1NS59\_CHICK tr|E1C414|E1C414\_CHICK tr|C4PCG8|C4PCG8\_GALVA tr|Q5ZM51|Q5ZM51\_CHICK tr|F1NS53|F1NS53\_CHICK tr|O73841|O73841\_CHICK tr|Q9PSQ9|Q9PSQ9\_CHICK tr|E1C392|E1C392\_CHICK tr|D3WGL2|D3WGL2\_CHICK tr|D3WGL3|D3WGL3\_CHICK tr|E9KFA0|E9KFA0\_CHICK tr|F1P438|F1P438\_CHICK tr|E1BU41|E1BU41\_CHICK tr|F1P537|F1P537\_CHICK tr|E1BQ56|E1BQ56\_CHICK tr|H9KZL2|H9KZL2\_CHICK tr|Q67BJ3|Q67BJ3\_CHICK tr|F1NAG9|F1NAG9\_CHICK tr|R4GL74|R4GL74\_CHICK tr|E1BT68|E1BT68\_CHICK tr|F1P0U2|F1P0U2\_CHICK tr|Q6PVZ2|Q6PVZ2\_CHICK tr|E1BWZ5|E1BWZ5\_CHICK tr|E1BYS3|E1BYS3\_CHICK tr|F1NPU9|F1NPU9\_CHICK tr|Q90WR7|Q90WR7\_CHICK tr|F1NXI0|F1NXI0\_CHICK tr|Q9I8H6|Q9I8H6\_CHICK sp|Q5ZL54|K0907\_CHICK tr|Q5ZL28|Q5ZL28\_CHICK tr|F1P4J7|F1P4J7\_CHICK tr|E1C4X5|E1C4X5\_CHICK tr|F1NMT6|F1NMT6\_CHICK tr|F1NKA5|F1NKA5\_CHICK tr|R4GM59|R4GM59\_CHICK tr|F1P571|F1P571\_CHICK tr|F1NNE5|F1NNE5\_CHICK tr|E1C5Z1|E1C5Z1\_CHICK tr|E1C3L4|E1C3L4\_CHICK tr|F1P380|F1P380\_CHICK tr|R4GL86|R4GL86\_CHICK tr|E1C8E0|E1C8E0\_CHICK tr|F1NM35|F1NM35\_CHICK tr|F1NJA0|F1NJA0\_CHICK tr|R4GJ16|R4GJ16\_CHICK tr|O42291|O42291\_CHICK tr|E1C8N2|E1C8N2\_CHICK tr|E1C7P4|E1C7P4\_CHICK sp|Q5ZKV8|KIF2A\_CHICK tr|F1NTF5|F1NTF5\_CHICK tr|E1C6N8|E1C6N8\_CHICK sp|Q5ZHQ6|ACBD5\_CHICK tr|E1BZ01|E1BZ01\_CHICK tr|F1NZ88|F1NZ88\_CHICK tr|Q5XY05|Q5XY05\_CHICK tr|R4GGQ3|R4GGQ3\_CHICK tr|F1NF35|F1NF35\_CHICK tr|E1BWK1|E1BWK1\_CHICK tr|R4GHT4|R4GHT4\_CHICK tr|Q9PS87|Q9PS87\_CHICK tr|E1C7T4|E1C7T4\_CHICK tr|F1NUE3|F1NUE3\_CHICK tr|R4GF37|R4GF37\_CHICK tr|Q9DER4|Q9DER4\_CHICK tr|R4GH98|R4GH98\_CHICK tr|H9L0C5|H9L0C5\_CHICK tr|F1NH72|F1NH72\_CHICK tr|Q8JH32|Q8JH32\_CHICK sp|Q8JJC0|ZIC1\_CHICK tr|F1NVU6|F1NVU6\_CHICK tr|F1NIZ0|F1NIZ0\_CHICK tr|F1P5W3|F1P5W3\_CHICK tr|F1NTX3|F1NTX3\_CHICK tr|E1C207|E1C207\_CHICK tr|F1NIC0|F1NIC0\_CHICK tr|F1P3M3|F1P3M3\_CHICK tr|E1C393|E1C393\_CHICK tr|E1BUF1|E1BUF1\_CHICK sp|Q5ZM88|ASXL2\_CHICK tr|F1NTR5|F1NTR5\_CHICK tr|E1C486|E1C486\_CHICK tr|E1BTW9|E1BTW9\_CHICK tr|E1BZ77|E1BZ77\_CHICK tr|F1P038|F1P038\_CHICK tr|Q5ZLQ0|Q5ZLQ0\_CHICK tr|Q5F439|Q5F439\_CHICK tr|F1NLN4|F1NLN4\_CHICK tr|E1C2Z8|E1C2Z8\_CHICK tr|F1NVD0|F1NVD0\_CHICK tr|F1NMJ1|F1NMJ1\_CHICK tr|Q5ZIL1|Q5ZIL1\_CHICK tr|F1NLI7|F1NLI7\_CHICK tr|Q98950|Q98950\_CHICK tr|D3WGK8|D3WGK8\_CHICK tr|R4GJA2|R4GJA2\_CHICK tr|R4GLX5|R4GLX5\_CHICK tr|R9PXP9|R9PXP9\_CHICK tr|F1P5Y4|F1P5Y4\_CHICK tr|F1P0R9|F1P0R9\_CHICK tr|Q4GWZ4|Q4GWZ4\_CHICK tr|E1C7N7|E1C7N7\_CHICK tr|Q4KXT1|Q4KXT1\_CHICK tr|Q4KXT2|Q4KXT2\_CHICK tr|E1C5G9|E1C5G9\_CHICK sp|Q5ZLW3|DYM\_CHICK tr|F1NLR1|F1NLR1\_CHICK tr|E1C053|E1C053\_CHICK tr|Q5ZMF4|Q5ZMF4\_CHICK tr|Q5ZL53|Q5ZL53\_CHICK tr|F1NPN5|F1NPN5\_CHICK sp|Q8AXV1|SH3G2\_CHICK tr|F1NUE5|F1NUE5\_CHICK tr|R4GJZ5|R4GJZ5\_CHICK tr|Q5DWQ5|Q5DWQ5\_CHICK tr|E1C2B1|E1C2B1\_CHICK tr|F1NH13|F1NH13\_CHICK tr|C4PCG2|C4PCG2\_CHICK tr|D0VX29|D0VX29\_CHICK tr|F1P582|F1P582\_CHICK tr|C4PCD3|C4PCD3\_CHICK Q3T052 tr|F1NDN5|F1NDN5\_CHICK tr|C4PCB3|C4PCB3\_CHICK tr|F1NPG4|F1NPG4\_CHICK tr|R4GKV3|R4GKV3\_CHICK tr|R4GFX6|R4GFX6\_CHICK tr|E1C2I9|E1C2I9\_CHICK tr|F1NK96|F1NK96\_CHICK tr|E1BZL4|E1BZL4\_CHICK tr|H9L2I2|H9L2I2\_CHICK tr|H9KZB2|H9KZB2\_CHICK sp|P47807|MYO1A\_CHICK tr|F1NPB6|F1NPB6\_CHICK sp|P98165|VLDLR\_CHICK tr|F1NLC2|F1NLC2\_CHICK tr|E1C952|E1C952\_CHICK tr|F1P3S9|F1P3S9\_CHICK tr|H9L3V3|H9L3V3\_CHICK tr|E1C5C9|E1C5C9\_CHICK tr|F1N8H5|F1N8H5\_CHICK tr|F1NIY6|F1NIY6\_CHICK tr|F1NT48|F1NT48\_CHICK tr|E1C7K7|E1C7K7\_CHICK tr|A0A0D4WTY6|A0A0D4WTY6\_CHICK sp|O57329|FZD7\_CHICK tr|Q9PW30|Q9PW30\_CHICK tr|A0A0D4WTI4|A0A0D4WTI4\_CHICK tr|F1NW80|F1NW80\_CHICK tr|E1C1N8|E1C1N8\_CHICK tr|F1NTB1|F1NTB1\_CHICK tr|Q6YJI4|Q6YJI4\_CHICK tr|H9KZP6|H9KZP6\_CHICK tr|E1C370|E1C370\_CHICK tr|F1NAC7|F1NAC7\_CHICK tr|R4GFR5|R4GFR5\_CHICK tr|E1C8S8|E1C8S8\_CHICK tr|Q5ZM95|Q5ZM95\_CHICK tr|Q335Q4|Q335Q4\_CHICK tr|H9L3I9|H9L3I9\_CHICK tr|F1NDI5|F1NDI5\_CHICK tr|F1NDK8|F1NDK8\_CHICK tr|Q4GWL8|Q4GWL8\_GALVA tr|F1CLE8|F1CLE8\_CHICK tr|R4GIW0|R4GIW0\_CHICK tr|F1NHJ7|F1NHJ7\_CHICK tr|E1BVN0|E1BVN0\_CHICK tr|F1NHU0|F1NHU0\_CHICK tr|R4GHK7|R4GHK7\_CHICK tr|H9L3N9|H9L3N9\_CHICK tr|Q6IVU9|Q6IVU9\_CHICK sp|Q5ZKQ6|DPOE2\_CHICK tr|F1NGS0|F1NGS0\_CHICK tr|R4GL41|R4GL41\_CHICK tr|E1C7Z1|E1C7Z1\_CHICK tr|F1NXH8|F1NXH8\_CHICK tr|Q4GWJ7|Q4GWJ7\_GALSO tr|F1P1I7|F1P1I7\_CHICK tr|E1BW56|E1BW56\_CHICK tr|E1C0W9|E1C0W9\_CHICK tr|F1NI83|F1NI83\_CHICK tr|F1NCE8|F1NCE8\_CHICK tr|F1P0A3|F1P0A3\_CHICK tr|F1NZV7|F1NZV7\_CHICK tr|F1ND19|F1ND19\_CHICK tr|F1NPL8|F1NPL8\_CHICK sp|Q58NQ5|PLAG1\_CHICK sp|P48434|SOX9\_CHICK tr|F1NYN4|F1NYN4\_CHICK tr|H9KZ90|H9KZ90\_CHICK tr|B5BSL7|B5BSL7\_CHICK tr|F5CSS9|F5CSS9\_CHICK sp|Q75QI0|CFDP1\_CHICK tr|E1BY54|E1BY54\_CHICK tr|E1C529|E1C529\_CHICK tr|Q6Q122|Q6Q122\_CHICK tr|E1C5T4|E1C5T4\_CHICK tr|F1P2E4|F1P2E4\_CHICK sp|P24798|AT1A3\_CHICK tr|R4GKS4|R4GKS4\_CHICK tr|C4PCB1|C4PCB1\_CHICK tr|C4PCA3|C4PCA3\_CHICK tr|C4PCB2|C4PCB2\_CHICK tr|C4PCE2|C4PCE2\_CHICK tr|C4PCD9|C4PCD9\_CHICK tr|F1NHT7|F1NHT7\_CHICK tr|O42486|O42486\_CHICK tr|F1P191|F1P191\_CHICK sp|Q5ZI51|ARHL2\_CHICK tr|H9L3V4|H9L3V4\_CHICK tr|F1NZG4|F1NZG4\_CHICK tr|B5BSN1|B5BSN1\_CHICK sp|Q90632|MOT3\_CHICK tr|F1NSK0|F1NSK0\_CHICK tr|E1BRW0|E1BRW0\_CHICK tr|F1NXF9|F1NXF9\_CHICK tr|Q90880|Q90880\_CHICK tr|Q90989|Q90989\_CHICK tr|F1P0I9|F1P0I9\_CHICK tr|E1C8D6|E1C8D6\_CHICK tr|Q8UW33|Q8UW33\_CHICK tr|E1BQT1|E1BQT1\_CHICK tr|F1NSP5|F1NSP5\_CHICK tr|E1C7M3|E1C7M3\_CHICK tr|E1BZE5|E1BZE5\_CHICK tr|F1P3G7|F1P3G7\_CHICK sp|P27607|PGH2\_CHICK tr|A0A0A0MQ41|A0A0A0MQ41\_CHICK tr|R4GKM1|R4GKM1\_CHICK tr|Q9DEC9|Q9DEC9\_CHICK tr|F1NG58|F1NG58\_CHICK tr|R4GG09|R4GG09\_CHICK tr|F1NIF2|F1NIF2\_CHICK tr|F1NL01|F1NL01\_CHICK tr|Q5F3R5|Q5F3R5\_CHICK tr|F1NRD6|F1NRD6\_CHICK tr|R4GL46|R4GL46\_CHICK tr|E1BQY9|E1BQY9\_CHICK sp|Q5ZKD5|RRP12\_CHICK sp|O93344|AL1A2\_CHICK tr|F1NQQ7|F1NQQ7\_CHICK tr|E1C4J6|E1C4J6\_CHICK tr|A0FKC7|A0FKC7\_CHICK tr|R4GIF2|R4GIF2\_CHICK tr|E1BR74|E1BR74\_CHICK tr|F1P4I5|F1P4I5\_CHICK tr|E1BT70|E1BT70\_CHICK tr|Q5F3T3|Q5F3T3\_CHICK tr|E1BVH4|E1BVH4\_CHICK tr|F1NCS6|F1NCS6\_CHICK tr|Q5ZM62|Q5ZM62\_CHICK tr|F1NN80|F1NN80\_CHICK tr|F1NGL0|F1NGL0\_CHICK tr|Q90800|Q90800\_CHICK tr|O42420|O42420\_CHICK tr|E1C5T1|E1C5T1\_CHICK tr|R4GJ12|R4GJ12\_CHICK sp|Q9DG09|STAR\_CHICK tr|F1NJH3|F1NJH3\_CHICK tr|R4GL06|R4GL06\_CHICK tr|E1C717|E1C717\_CHICK tr|F1NCG8|F1NCG8\_CHICK tr|F1N9K6|F1N9K6\_CHICK tr|F1NCY5|F1NCY5\_CHICK sp|Q5ZMG1|UFL1\_CHICK tr|F1NB44|F1NB44\_CHICK tr|H9L035|H9L035\_CHICK tr|E1BXK7|E1BXK7\_CHICK tr|F1NK97|F1NK97\_CHICK tr|F1NNP7|F1NNP7\_CHICK tr|F1NKU1|F1NKU1\_CHICK tr|E1BT59|E1BT59\_CHICK tr|E1BX74|E1BX74\_CHICK tr|R4GJW7|R4GJW7\_CHICK tr|E1C214|E1C214\_CHICK tr|F1P3Z4|F1P3Z4\_CHICK tr|F1NAV7|F1NAV7\_CHICK tr|F1CN64|F1CN64\_CHICK tr|F1NAQ6|F1NAQ6\_CHICK tr|F1NUW3|F1NUW3\_CHICK sp|P24271|RAG1\_CHICK tr|O57349|O57349\_CHICK tr|Q90806|Q90806\_CHICK tr|Q5ZLC2|Q5ZLC2\_CHICK sp|Q90850|HIC1\_CHICK tr|Q9DEC7|Q9DEC7\_CHICK tr|Q5F3F4|Q5F3F4\_CHICK tr|E1BVB6|E1BVB6\_CHICK tr|F1NKZ2|F1NKZ2\_CHICK tr|E1BX76|E1BX76\_CHICK tr|H9L096|H9L096\_CHICK tr|E1BSI0|E1BSI0\_CHICK tr|R4GM03|R4GM03\_CHICK tr|F1NYF0|F1NYF0\_CHICK tr|F1NQC9|F1NQC9\_CHICK tr|Q5ZJK7|Q5ZJK7\_CHICK tr|E1BQF4|E1BQF4\_CHICK tr|E1BWQ1|E1BWQ1\_CHICK tr|F1P1E7|F1P1E7\_CHICK tr|F1NWD1|F1NWD1\_CHICK tr|E1C8H9|E1C8H9\_CHICK tr|F1P138|F1P138\_CHICK tr|F1P4R5|F1P4R5\_CHICK sp|Q5ZJX7|MB12A\_CHICK tr|K0Q571|K0Q571\_CHICK tr|F1NSH9|F1NSH9\_CHICK tr|E1BQ43|E1BQ43\_CHICK tr|Q9PWA2|Q9PWA2\_CHICK tr|Q9YGX8|Q9YGX8\_CHICK tr|A2TH16|A2TH16\_CHICK tr|E1C822|E1C822\_CHICK sp|Q5ZLD3|KLH13\_CHICK tr|E1BVP7|E1BVP7\_CHICK tr|E1BTC5|E1BTC5\_CHICK sp|Q6IMM1|DJC27\_CHICK tr|F1N8P1|F1N8P1\_CHICK sp|P79795|NRP1\_CHICK tr|F1NCH4|F1NCH4\_CHICK tr|H9KYP8|H9KYP8\_CHICK sp|P14781|CNTN1\_CHICK tr|A0ZY61|A0ZY61\_CHICK tr|Q861P6|Q861P6\_CHICK tr|Q5ZJ29|Q5ZJ29\_CHICK tr|F1NDL9|F1NDL9\_CHICK tr|E1BQU4|E1BQU4\_CHICK tr|E1C753|E1C753\_CHICK tr|F1NDL4|F1NDL4\_CHICK tr|F1NBU7|F1NBU7\_CHICK tr|F1NPX2|F1NPX2\_CHICK tr|E1C8N8|E1C8N8\_CHICK tr|R4GKN5|R4GKN5\_CHICK tr|E1C3B9|E1C3B9\_CHICK sp|Q90854|KCNJ3\_CHICK sp|P79765|NDF1\_CHICK tr|F1NKX5|F1NKX5\_CHICK tr|R4GJC9|R4GJC9\_CHICK sp|Q789F3|MAF\_CHICK tr|E1C1B4|E1C1B4\_CHICK sp|E1C2P3|HSP7E\_CHICK tr|Q2PBB9|Q2PBB9\_CHICK tr|E1BSD9|E1BSD9\_CHICK tr|Q5F3G9|Q5F3G9\_CHICK tr|B5BST5|B5BST5\_CHICK tr|F5CSS5|F5CSS5\_CHICK tr|B5BSC0|B5BSC0\_CHICK tr|B5BSA6|B5BSA6\_CHICK tr|F1NLJ4|F1NLJ4\_CHICK tr|F1NC90|F1NC90\_CHICK tr|F1P157|F1P157\_CHICK tr|Q90711|Q90711\_CHICK tr|P79757|P79757\_CHICK tr|F1N9I3|F1N9I3\_CHICK tr|F1P4M9|F1P4M9\_CHICK tr|Q5F395|Q5F395\_CHICK tr|Q4GWK5|Q4GWK5\_GALLA tr|F1NPU5|F1NPU5\_CHICK tr|E1BZ15|E1BZ15\_CHICK tr|F1NW15|F1NW15\_CHICK sp|Q90WI4|MXRA8\_CHICK tr|F1P409|F1P409\_CHICK tr|Q8AV16|Q8AV16\_CHICK tr|Q5ZLC0|Q5ZLC0\_CHICK tr|F1NEN4|F1NEN4\_CHICK tr|Q5F458|Q5F458\_CHICK tr|F1NEZ9|F1NEZ9\_CHICK tr|E1BXM8|E1BXM8\_CHICK sp|Q90ZK6|ACVR1\_CHICK tr|A6YJX3|A6YJX3\_CHICK tr|E1C7H6|E1C7H6\_CHICK P07224 tr|E1C2W6|E1C2W6\_CHICK tr|E1BR95|E1BR95\_CHICK tr|E1BXM9|E1BXM9\_CHICK tr|E1C4E5|E1C4E5\_CHICK tr|Q5ZIR3|Q5ZIR3\_CHICK tr|E1BYZ4|E1BYZ4\_CHICK tr|F1NJP2|F1NJP2\_CHICK tr|F1NDW0|F1NDW0\_CHICK tr|R4GII3|R4GII3\_CHICK tr|B5AHE5|B5AHE5\_CHICK tr|F1NWK5|F1NWK5\_CHICK tr|F5CST7|F5CST7\_CHICK tr|A5HUM1|A5HUM1\_CHICK tr|B5BSP6|B5BSP6\_CHICK tr|E1BXY3|E1BXY3\_CHICK tr|Q5F427|Q5F427\_CHICK tr|E1C9G0|E1C9G0\_CHICK tr|E1C8W3|E1C8W3\_CHICK tr|E1C0T3|E1C0T3\_CHICK tr|F1NQ70|F1NQ70\_CHICK tr|E1C129|E1C129\_CHICK sp|P33150|CAD13\_CHICK tr|F1P5T8|F1P5T8\_CHICK tr|Q335Q3|Q335Q3\_CHICK tr|Q5ZMU8|Q5ZMU8\_CHICK tr|R4GK13|R4GK13\_CHICK tr|Q6XBN7|Q6XBN7\_CHICK tr|E1BZP9|E1BZP9\_CHICK tr|F1NIF0|F1NIF0\_CHICK tr|E1C221|E1C221\_CHICK tr|F1NBV4|F1NBV4\_CHICK tr|E1BU12|E1BU12\_CHICK tr|F1NWX3|F1NWX3\_CHICK tr|Q5ZIW0|Q5ZIW0\_CHICK tr|F1NK40|F1NK40\_CHICK tr|F1P356|F1P356\_CHICK tr|C6F1H2|C6F1H2\_CHICK tr|J9PBQ3|J9PBQ3\_CHICK sp|Q5ZJR9|TAF1B\_CHICK tr|Q90WD1|Q90WD1\_CHICK tr|Q71R46|Q71R46\_CHICK tr|E1BXL2|E1BXL2\_CHICK sp|Q5ZLM2|DBR1\_CHICK tr|F1NFQ3|F1NFQ3\_CHICK tr|E1BTB4|E1BTB4\_CHICK tr|F1NJN3|F1NJN3\_CHICK tr|F1NGG3|F1NGG3\_CHICK tr|F1NZ93|F1NZ93\_CHICK tr|E1C726|E1C726\_CHICK tr|R4GJL4|R4GJL4\_CHICK tr|R4GGL9|R4GGL9\_CHICK tr|F1NM57|F1NM57\_CHICK tr|Q5ZJT6|Q5ZJT6\_CHICK tr|F1NX39|F1NX39\_CHICK tr|F1N961|F1N961\_CHICK sp|Q07494|EPHB1\_CHICK tr|H9L0J1|H9L0J1\_CHICK tr|F1NBU4|F1NBU4\_CHICK tr|E1BYY7|E1BYY7\_CHICK tr|F1NER6|F1NER6\_CHICK tr|E1BT40|E1BT40\_CHICK tr|F1NXB3|F1NXB3\_CHICK tr|Q90X18|Q90X18\_CHICK tr|F1NLX1|F1NLX1\_CHICK tr|F1NPV9|F1NPV9\_CHICK tr|F1NK33|F1NK33\_CHICK tr|F1NW85|F1NW85\_CHICK tr|R4GIH1|R4GIH1\_CHICK tr|F1NE99|F1NE99\_CHICK tr|E1C8C5|E1C8C5\_CHICK tr|Q5ZLA7|Q5ZLA7\_CHICK tr|F1NL59|F1NL59\_CHICK tr|F1NCR5|F1NCR5\_CHICK tr|F1N8E4|F1N8E4\_CHICK tr|R4GI22|R4GI22\_CHICK tr|F1NYQ3|F1NYQ3\_CHICK tr|Q4GWJ2|Q4GWJ2\_GALSO tr|R4GIJ5|R4GIJ5\_CHICK tr|F1NPI9|F1NPI9\_CHICK tr|E1C577|E1C577\_CHICK tr|F1P0W9|F1P0W9\_CHICK tr|E1BVF3|E1BVF3\_CHICK tr|F1NC35|F1NC35\_CHICK tr|E1BQT5|E1BQT5\_CHICK tr|Q6E236|Q6E236\_CHICK tr|E1BYZ2|E1BYZ2\_CHICK tr|C4PCD7|C4PCD7\_CHICK tr|F1NTA3|F1NTA3\_CHICK tr|F1P2G1|F1P2G1\_CHICK tr|E1C7U9|E1C7U9\_CHICK tr|Q9PWN6|Q9PWN6\_CHICK tr|F1P440|F1P440\_CHICK tr|E1C693|E1C693\_CHICK tr|F1NWM8|F1NWM8\_CHICK tr|S6DG70|S6DG70\_CHICK tr|F1NFR4|F1NFR4\_CHICK sp|Q5ZLT0|XPO7\_CHICK tr|F1NML5|F1NML5\_CHICK tr|F1NH81|F1NH81\_CHICK tr|Q8UWC5|Q8UWC5\_CHICK tr|E1BS98|E1BS98\_CHICK tr|F1P1F1|F1P1F1\_CHICK tr|R4GI76|R4GI76\_CHICK tr|F1NEL9|F1NEL9\_CHICK tr|Q5ZJ45|Q5ZJ45\_CHICK tr|F1P5T2|F1P5T2\_CHICK tr|Q6ZX98|Q6ZX98\_CHICK tr|E1C627|E1C627\_CHICK tr|R4GIU1|R4GIU1\_CHICK tr|F1NMM2|F1NMM2\_CHICK tr|E1C181|E1C181\_CHICK tr|F1P3P6|F1P3P6\_CHICK tr|F1NV53|F1NV53\_CHICK tr|F1NFP4|F1NFP4\_CHICK tr|E1C4H8|E1C4H8\_CHICK tr|Q1H9T7|Q1H9T7\_CHICK P62894 tr|F1NUM7|F1NUM7\_CHICK tr|E1BVG1|E1BVG1\_CHICK tr|E1BTZ4|E1BTZ4\_CHICK tr|Q5ZJA7|Q5ZJA7\_CHICK tr|R4GHK9|R4GHK9\_CHICK tr|F1P0C4|F1P0C4\_CHICK tr|F1NXJ5|F1NXJ5\_CHICK tr|F1NZ51|F1NZ51\_CHICK tr|Q5ZI84|Q5ZI84\_CHICK tr|E1C9I5|E1C9I5\_CHICK tr|F1N9W3|F1N9W3\_CHICK tr|F1NVG7|F1NVG7\_CHICK tr|E1C4L6|E1C4L6\_CHICK tr|R4GKQ7|R4GKQ7\_CHICK tr|F1NEA1|F1NEA1\_CHICK tr|F1NBE5|F1NBE5\_CHICK tr|F1NJT5|F1NJT5\_CHICK tr|E1BUG5|E1BUG5\_CHICK tr|E1C8Y2|E1C8Y2\_CHICK tr|E1C5Y9|E1C5Y9\_CHICK tr|A0A089FJ58|A0A089FJ58\_CHICK tr|F1NSV9|F1NSV9\_CHICK sp|P35915|HMGCL\_CHICK tr|F1NUC4|F1NUC4\_CHICK tr|F1P2W9|F1P2W9\_CHICK tr|Q5F385|Q5F385\_CHICK tr|Q5ZHQ4|Q5ZHQ4\_CHICK tr|F1P2V2|F1P2V2\_CHICK tr|F1NGU3|F1NGU3\_CHICK tr|E1C740|E1C740\_CHICK sp|P09244|TBB7\_CHICK tr|A5A2G4|A5A2G4\_CHICK tr|F1NEC3|F1NEC3\_CHICK tr|M1T4J8|M1T4J8\_CHICK tr|A0A0D3RAM1|A0A0D3RAM1\_CHICK tr|R4GHN6|R4GHN6\_CHICK tr|E1C6P7|E1C6P7\_CHICK tr|F1P064|F1P064\_CHICK tr|Q9PVJ9|Q9PVJ9\_CHICK tr|F1NI59|F1NI59\_CHICK tr|F1NYI8|F1NYI8\_CHICK tr|F1P2U1|F1P2U1\_CHICK tr|E1BS87|E1BS87\_CHICK tr|R4GIR2|R4GIR2\_CHICK tr|E1C9H7|E1C9H7\_CHICK tr|B8XXD7|B8XXD7\_CHICK tr|F1NLJ7|F1NLJ7\_CHICK tr|Q7T195|Q7T195\_CHICK tr|F1NLK0|F1NLK0\_CHICK tr|Q5ZMT3|Q5ZMT3\_CHICK tr|Q9YGW5|Q9YGW5\_CHICK tr|R4GIP3|R4GIP3\_CHICK tr|F1P3Q4|F1P3Q4\_CHICK tr|E1C163|E1C163\_CHICK tr|F1P5I6|F1P5I6\_CHICK tr|F1NZK8|F1NZK8\_CHICK tr|E1C6U2|E1C6U2\_CHICK tr|F1DQG4|F1DQG4\_CHICK tr|F1NQ14|F1NQ14\_CHICK tr|R4GHS2|R4GHS2\_CHICK tr|F1NBG2|F1NBG2\_CHICK sp|Q02391|GSLG1\_CHICK tr|F1N8B2|F1N8B2\_CHICK tr|E1BSK1|E1BSK1\_CHICK tr|O57480|O57480\_CHICK tr|F1NQZ5|F1NQZ5\_CHICK tr|A9Y0G9|A9Y0G9\_CHICK tr|A9Y0H0|A9Y0H0\_CHICK tr|F1NS23|F1NS23\_CHICK tr|R4GG73|R4GG73\_CHICK tr|E1BZE3|E1BZE3\_CHICK sp|P18937|NU2M\_CHICK tr|E1BWY6|E1BWY6\_CHICK tr|Q5ZJM1|Q5ZJM1\_CHICK tr|Q5ZIG4|Q5ZIG4\_CHICK sp|Q5ZHZ4|HAKAI\_CHICK tr|H9KYV9|H9KYV9\_CHICK tr|F1NEU9|F1NEU9\_CHICK tr|E1BWZ3|E1BWZ3\_CHICK tr|A3EYJ5|A3EYJ5\_CHICK tr|E1C8S5|E1C8S5\_CHICK tr|O93382|O93382\_CHICK tr|F1NCZ2|F1NCZ2\_CHICK tr|Q9I8W4|Q9I8W4\_CHICK tr|F1NRA6|F1NRA6\_CHICK tr|F1NJM9|F1NJM9\_CHICK tr|E1C4F3|E1C4F3\_CHICK tr|E1BYI9|E1BYI9\_CHICK tr|C4PCE9|C4PCE9\_GALLA tr|C4PCE7|C4PCE7\_GALLA sp|Q2VRL0|PLCZ1\_CHICK tr|E1C6X2|E1C6X2\_CHICK tr|F1N8N2|F1N8N2\_CHICK sp|Q5ZLL7|WDR91\_CHICK tr|E1C964|E1C964\_CHICK tr|E1C7R8|E1C7R8\_CHICK tr|Q90974|Q90974\_CHICK tr|Q788U7|Q788U7\_CHICK tr|R4GI60|R4GI60\_CHICK tr|Q9YH28|Q9YH28\_CHICK tr|R4GL43|R4GL43\_CHICK tr|R4GFU2|R4GFU2\_CHICK tr|Q5ZHY4|Q5ZHY4\_CHICK tr|F1NHE0|F1NHE0\_CHICK tr|F1NSS5|F1NSS5\_CHICK tr|E1BSV1|E1BSV1\_CHICK tr|Q90689|Q90689\_CHICK sp|Q90935|NEUS\_CHICK tr|F1P230|F1P230\_CHICK tr|Q9YGX6|Q9YGX6\_CHICK tr|E1BVA6|E1BVA6\_CHICK tr|Q1G7H4|Q1G7H4\_CHICK tr|F2YI34|F2YI34\_CHICK tr|R4GGA3|R4GGA3\_CHICK tr|F1CLE7|F1CLE7\_CHICK tr|R4GHW6|R4GHW6\_CHICK tr|E1C9E2|E1C9E2\_CHICK tr|F1CLE9|F1CLE9\_CHICK tr|F1CLF0|F1CLF0\_CHICK tr|E1C0Y8|E1C0Y8\_CHICK tr|F1P5G1|F1P5G1\_CHICK tr|F1NHZ9|F1NHZ9\_CHICK tr|F1NF62|F1NF62\_CHICK tr|R4GK73|R4GK73\_CHICK tr|Q5ZMQ6|Q5ZMQ6\_CHICK tr|E1C456|E1C456\_CHICK tr|F1NUP4|F1NUP4\_CHICK tr|R4GIA0|R4GIA0\_CHICK tr|F1NJV2|F1NJV2\_CHICK tr|Q6YEX8|Q6YEX8\_CHICK tr|R4GLW4|R4GLW4\_CHICK sp|P24797|AT1A2\_CHICK tr|Q5ZMU5|Q5ZMU5\_CHICK tr|F1NY69|F1NY69\_CHICK tr|F1NK56|F1NK56\_CHICK sp|Q5ZKD4|NPL\_CHICK tr|O57393|O57393\_CHICK tr|E1BYG5|E1BYG5\_CHICK tr|E1BVZ2|E1BVZ2\_CHICK sp|O73612|EFNB1\_CHICK tr|F1NZX5|F1NZX5\_CHICK tr|F1P2G7|F1P2G7\_CHICK sp|Q5ZM45|UBP48\_CHICK tr|R4GII5|R4GII5\_CHICK tr|E1C4K6|E1C4K6\_CHICK tr|G1K338|G1K338\_CHICK sp|P32882|TBB2\_CHICK sp|P09203|TBB1\_CHICK tr|F1N9B2|F1N9B2\_CHICK tr|E1BZH8|E1BZH8\_CHICK tr|R4QNW4|R4QNW4\_CHICK tr|E1C341|E1C341\_CHICK tr|Q5ZMQ7|Q5ZMQ7\_CHICK tr|V9GVH4|V9GVH4\_CHICK tr|F1NQC1|F1NQC1\_CHICK sp|P79995|CAD10\_CHICK tr|Q5F3V8|Q5F3V8\_CHICK tr|F1P0Y7|F1P0Y7\_CHICK tr|Q90Z45|Q90Z45\_CHICK tr|F1P4J5|F1P4J5\_CHICK tr|E1BWL1|E1BWL1\_CHICK tr|R4GMC1|R4GMC1\_CHICK tr|F1NRR0|F1NRR0\_CHICK tr|F1NIE7|F1NIE7\_CHICK tr|E1C333|E1C333\_CHICK P41361 tr|Q90815|Q90815\_CHICK tr|F1NFZ9|F1NFZ9\_CHICK tr|F1NEP3|F1NEP3\_CHICK tr|F1NVY3|F1NVY3\_CHICK tr|F1NDB9|F1NDB9\_CHICK sp|P02552|TBA1\_CHICK tr|F1NVX9|F1NVX9\_CHICK tr|F1P106|F1P106\_CHICK tr|E1BUA3|E1BUA3\_CHICK tr|F1NIY8|F1NIY8\_CHICK tr|H9L365|H9L365\_CHICK tr|R4GK35|R4GK35\_CHICK tr|Q4GWL0|Q4GWL0\_GALLA sp|Q98948|SSBP3\_CHICK sp|P02263|H2A4\_CHICK tr|Q92069|Q92069\_CHICK sp|Q5ZJT1|HMCES\_CHICK sp|P35062|H2A3\_CHICK tr|Q4AE89|Q4AE89\_CHICK tr|Q75XU5|Q75XU5\_CHICK tr|H9L0H3|H9L0H3\_CHICK tr|F1NL84|F1NL84\_CHICK tr|R4GIG2|R4GIG2\_CHICK tr|Q5ZLM6|Q5ZLM6\_CHICK tr|F1NGH4|F1NGH4\_CHICK tr|Q7LZ75|Q7LZ75\_CHICK tr|F1NDA3|F1NDA3\_CHICK tr|B9VVJ4|B9VVJ4\_CHICK tr|O93378|O93378\_CHICK tr|F1NIN1|F1NIN1\_CHICK tr|Q8AWB9|Q8AWB9\_CHICK tr|R4GF67|R4GF67\_CHICK tr|F1NMN7|F1NMN7\_CHICK tr|F1NV40|F1NV40\_CHICK tr|Q5U785|Q5U785\_CHICK tr|Q5XKY5|Q5XKY5\_CHICK tr|Q5U784|Q5U784\_CHICK tr|F1P0X4|F1P0X4\_CHICK tr|E5DF48|E5DF48\_CHICK tr|E5DEB2|E5DEB2\_CHICK tr|F1P5L3|F1P5L3\_CHICK tr|Q5ZIM8|Q5ZIM8\_CHICK tr|E1C2T7|E1C2T7\_CHICK tr|E1C3M0|E1C3M0\_CHICK tr|F1NCW1|F1NCW1\_CHICK tr|E1BSZ5|E1BSZ5\_CHICK tr|Q5U783|Q5U783\_CHICK tr|H9KZ37|H9KZ37\_CHICK tr|Q9ML65|Q9ML65\_GALVA tr|E1BX22|E1BX22\_CHICK sp|P70082|H2AJ\_CHICK tr|E1BWC5|E1BWC5\_CHICK tr|F1NT09|F1NT09\_CHICK tr|B8PQ41|B8PQ41\_CHICK tr|F1P4S7|F1P4S7\_CHICK tr|Q5F3B9|Q5F3B9\_CHICK tr|E1BVG7|E1BVG7\_CHICK tr|B3TZB8|B3TZB8\_CHICK tr|O73717|O73717\_CHICK tr|F6MF48|F6MF48\_CHICK tr|F1P3Q8|F1P3Q8\_CHICK tr|E1B2Y2|E1B2Y2\_CHICK tr|E1BXL9|E1BXL9\_CHICK tr|E1C5M4|E1C5M4\_CHICK tr|Q5TM08|Q5TM08\_CHICK Q3SZH5 tr|Q5F328|Q5F328\_CHICK tr|F1P4A7|F1P4A7\_CHICK tr|F1NQU7|F1NQU7\_CHICK tr|E1BRR5|E1BRR5\_CHICK tr|Q6WNG8|Q6WNG8\_CHICK tr|Q5F3I8|Q5F3I8\_CHICK tr|F1NEG6|F1NEG6\_CHICK tr|F1NSR4|F1NSR4\_CHICK tr|E5DEP2|E5DEP2\_CHICK tr|F1NAP7|F1NAP7\_CHICK tr|U6C3W5|U6C3W5\_CHICK tr|F1NI07|F1NI07\_CHICK tr|E1BY73|E1BY73\_CHICK tr|Q71SY9|Q71SY9\_CHICK tr|E1BUF6|E1BUF6\_CHICK tr|F1NF27|F1NF27\_CHICK tr|F1NKW2|F1NKW2\_CHICK tr|Q5ZLB3|Q5ZLB3\_CHICK tr|Q5ZHU1|Q5ZHU1\_CHICK tr|F1NG63|F1NG63\_CHICK tr|F1P1Z5|F1P1Z5\_CHICK sp|P57074|SOX8\_CHICK tr|E5DF54|E5DF54\_CHICK tr|E1C7C2|E1C7C2\_CHICK tr|E1BQ93|E1BQ93\_CHICK tr|E1C260|E1C260\_CHICK tr|F1P1K1|F1P1K1\_CHICK tr|R4GJE5|R4GJE5\_CHICK tr|F1P3V4|F1P3V4\_CHICK tr|E1BS62|E1BS62\_CHICK tr|E1BSN1|E1BSN1\_CHICK tr|R4GM96|R4GM96\_CHICK tr|M9NIK3|M9NIK3\_CHICK tr|F1NKN8|F1NKN8\_CHICK tr|F1NI89|F1NI89\_CHICK sp|Q5ZJW8|DTL\_CHICK tr|R4GKS6|R4GKS6\_CHICK tr|Q4GWS3|Q4GWS3\_GALSO tr|A0A0B4ZVC4|A0A0B4ZVC4\_CHICK tr|Q85AA8|Q85AA8\_CHICK tr|E5DFJ7|E5DFJ7\_CHICK tr|K9JUN2|K9JUN2\_CHICK tr|E1C4B7|E1C4B7\_CHICK tr|F1NJ03|F1NJ03\_CHICK tr|F1NT51|F1NT51\_CHICK sp|P12957|CALD1\_CHICK tr|F1NCC1|F1NCC1\_CHICK tr|Q5ZME7|Q5ZME7\_CHICK tr|Q5F3J9|Q5F3J9\_CHICK tr|E1C0U5|E1C0U5\_CHICK tr|F1NVY4|F1NVY4\_CHICK tr|F1NNP6|F1NNP6\_CHICK tr|E1C826|E1C826\_CHICK tr|Q5IJ76|Q5IJ76\_CHICK tr|F1NLD7|F1NLD7\_CHICK tr|R4GGE5|R4GGE5\_CHICK sp|P79761|CP1A5\_CHICK tr|F1NNW1|F1NNW1\_CHICK tr|F1NVF0|F1NVF0\_CHICK sp|Q1PRL4|LIN41\_CHICK tr|Q5ZL51|Q5ZL51\_CHICK sp|Q5ZI82|P2RY8\_CHICK tr|E1BTI0|E1BTI0\_CHICK tr|F1NZM8|F1NZM8\_CHICK tr|E1BQ73|E1BQ73\_CHICK tr|F1NPN6|F1NPN6\_CHICK tr|F1N902|F1N902\_CHICK tr|E1BVA0|E1BVA0\_CHICK tr|E1BWW5|E1BWW5\_CHICK tr|Q5ZJC6|Q5ZJC6\_CHICK tr|F1NDT3|F1NDT3\_CHICK tr|F1NY10|F1NY10\_CHICK sp|Q5ZID1|MED17\_CHICK tr|Q5ZHL4|Q5ZHL4\_CHICK tr|E1BRI3|E1BRI3\_CHICK tr|F1N979|F1N979\_CHICK tr|F1P1A4|F1P1A4\_CHICK tr|R4GGX4|R4GGX4\_CHICK tr|F1NVV4|F1NVV4\_CHICK tr|F1P5T0|F1P5T0\_CHICK sp|Q90625|ZBT17\_CHICK tr|R4GKE6|R4GKE6\_CHICK tr|F1P4D9|F1P4D9\_CHICK tr|E1C593|E1C593\_CHICK sp|P31335|PUR9\_CHICK tr|Q5F3J0|Q5F3J0\_CHICK tr|Q5ZLM1|Q5ZLM1\_CHICK tr|R4GGP3|R4GGP3\_CHICK tr|D3KR67|D3KR67\_CHICK tr|F1NIK0|F1NIK0\_CHICK tr|Q804Z8|Q804Z8\_CHICK tr|Q804H4|Q804H4\_CHICK tr|E1C761|E1C761\_CHICK tr|F1P0S5|F1P0S5\_CHICK tr|F1P0S6|F1P0S6\_CHICK tr|Q90857|Q90857\_CHICK tr|B2Z9X7|B2Z9X7\_CHICK tr|F7BYG6|F7BYG6\_CHICK tr|Q5F3Y4|Q5F3Y4\_CHICK sp|P35001|NEUM\_CHICK tr|Q0JRI2|Q0JRI2\_CHICK tr|Q9I9D7|Q9I9D7\_CHICK tr|Q9I9D8|Q9I9D8\_CHICK tr|R4GGI2|R4GGI2\_CHICK tr|F1NMB0|F1NMB0\_CHICK tr|Q5ZKN6|Q5ZKN6\_CHICK tr|F1NQ85|F1NQ85\_CHICK tr|F5CSS8|F5CSS8\_CHICK tr|E1C9I4|E1C9I4\_CHICK tr|B2DFX7|B2DFX7\_CHICK tr|F1NY86|F1NY86\_CHICK sp|Q91018|PROX1\_CHICK sp|Q92076|DPOG1\_CHICK tr|F1NV30|F1NV30\_CHICK tr|B5BSR5|B5BSR5\_CHICK tr|F1NLE7|F1NLE7\_CHICK tr|F1NAA2|F1NAA2\_CHICK tr|Q4GWS5|Q4GWS5\_GALSO tr|Q7GTV3|Q7GTV3\_CHICK tr|E5DEK7|E5DEK7\_CHICK tr|Q4GWN6|Q4GWN6\_CHICK tr|Q4GWM3|Q4GWM3\_GALVA sp|P18936|NU1M\_CHICK tr|Q4GWP9|Q4GWP9\_CHICK tr|E5DEA3|E5DEA3\_CHICK tr|Q4GWR2|Q4GWR2\_CHICK tr|E5DFI2|E5DFI2\_CHICK tr|E5DEW1|E5DEW1\_CHICK tr|Q6IVU8|Q6IVU8\_CHICK tr|E1BYM9|E1BYM9\_CHICK tr|E1C2P9|E1C2P9\_CHICK tr|Q5F3A7|Q5F3A7\_CHICK tr|Q5ZKX0|Q5ZKX0\_CHICK tr|E1BT08|E1BT08\_CHICK tr|F1NZ86|F1NZ86\_CHICK tr|F1NXU9|F1NXU9\_CHICK tr|F1NRI1|F1NRI1\_CHICK tr|F1NJE7|F1NJE7\_CHICK tr|Q804X4|Q804X4\_CHICK tr|C4PCE8|C4PCE8\_GALLA tr|I3QHT1|I3QHT1\_CHICK tr|C4PCF3|C4PCF3\_CHICK tr|C4PCH3|C4PCH3\_GALSO tr|C4PCH0|C4PCH0\_GALSO tr|C4PCJ0|C4PCJ0\_CHICK tr|U5U9K2|U5U9K2\_CHICK tr|C4PCI6|C4PCI6\_CHICK tr|C4PCG4|C4PCG4\_CHICK tr|C4PCG7|C4PCG7\_CHICK tr|C4PCF4|C4PCF4\_CHICK tr|I3QHS5|I3QHS5\_CHICK tr|C4PCF6|C4PCF6\_CHICK tr|E1BV56|E1BV56\_CHICK tr|E5DEP6|E5DEP6\_CHICK tr|E1BXX2|E1BXX2\_CHICK tr|R4GG69|R4GG69\_CHICK tr|F1N9K1|F1N9K1\_CHICK sp|Q9IAM7|MRE11\_CHICK tr|F1NF49|F1NF49\_CHICK tr|E1C306|E1C306\_CHICK tr|F5GVB8|F5GVB8\_CHICK tr|R4GJM1|R4GJM1\_CHICK tr|Q5ZK51|Q5ZK51\_CHICK tr|F1NF28|F1NF28\_CHICK tr|Q5ZIC6|Q5ZIC6\_CHICK tr|E1BTA8|E1BTA8\_CHICK tr|O57465|O57465\_CHICK tr|F1N9C1|F1N9C1\_CHICK tr|E1C6H9|E1C6H9\_CHICK tr|E1BR32|E1BR32\_CHICK tr|E1BT89|E1BT89\_CHICK tr|B6UV99|B6UV99\_CHICK tr|F1N8M4|F1N8M4\_CHICK tr|R4GJ94|R4GJ94\_CHICK sp|Q5F499|OPA1\_CHICK tr|F1P0J0|F1P0J0\_CHICK sp|P38529|HSF1\_CHICK tr|Q6X895|Q6X895\_CHICK tr|Q90601|Q90601\_CHICK tr|E1BRF5|E1BRF5\_CHICK tr|R4GIS9|R4GIS9\_CHICK tr|F1NGH3|F1NGH3\_CHICK tr|E1C8P5|E1C8P5\_CHICK tr|Q4GWI8|Q4GWI8\_GALSO tr|H9KZS3|H9KZS3\_CHICK tr|A2NBE2|A2NBE2\_CHICK tr|F1NLK5|F1NLK5\_CHICK tr|E1BVL8|E1BVL8\_CHICK tr|E1BWS0|E1BWS0\_CHICK tr|F1P551|F1P551\_CHICK tr|D2X2H2|D2X2H2\_CHICK tr|F1NGC1|F1NGC1\_CHICK tr|D3WGL5|D3WGL5\_CHICK tr|D3WGL4|D3WGL4\_CHICK tr|E1BYW4|E1BYW4\_CHICK sp|P55878|GLI1\_CHICK tr|F1NZQ1|F1NZQ1\_CHICK tr|F1NEF5|F1NEF5\_CHICK tr|F1NWD0|F1NWD0\_CHICK tr|E1BQC4|E1BQC4\_CHICK tr|F1N8Z7|F1N8Z7\_CHICK tr|F1NG92|F1NG92\_CHICK tr|Q98921|Q98921\_CHICK tr|Q98922|Q98922\_CHICK tr|E1C6L8|E1C6L8\_CHICK tr|Q05951|Q05951\_CHICK sp|Q5ZJV4|MCMBP\_CHICK tr|Q5F3L6|Q5F3L6\_CHICK tr|F1NFM3|F1NFM3\_CHICK tr|R4GH21|R4GH21\_CHICK tr|F1NAY9|F1NAY9\_CHICK tr|E1BWX8|E1BWX8\_CHICK tr|F1NTB2|F1NTB2\_CHICK tr|Q8UWJ6|Q8UWJ6\_CHICK tr|F1P0R4|F1P0R4\_CHICK tr|B3F8C5|B3F8C5\_CHICK tr|R4GIQ1|R4GIQ1\_CHICK tr|F1NAL8|F1NAL8\_CHICK tr|Q5ZK15|Q5ZK15\_CHICK sp|Q5ZJ26|K1671\_CHICK tr|E1C5F3|E1C5F3\_CHICK tr|F1NBH4|F1NBH4\_CHICK tr|R4GJT2|R4GJT2\_CHICK tr|R4GH75|R4GH75\_CHICK tr|F1NZC0|F1NZC0\_CHICK sp|Q90875|ACOC\_CHICK tr|F1NY25|F1NY25\_CHICK tr|F1NYG0|F1NYG0\_CHICK tr|F1NUG0|F1NUG0\_CHICK tr|R4GLI8|R4GLI8\_CHICK tr|E1C4I7|E1C4I7\_CHICK tr|F1NGF7|F1NGF7\_CHICK tr|F1P4V4|F1P4V4\_CHICK tr|F1NS24|F1NS24\_CHICK tr|Q6F4E4|Q6F4E4\_CHICK sp|Q5ZM19|CQ085\_CHICK tr|F1NGX2|F1NGX2\_CHICK tr|Q6RUV9|Q6RUV9\_CHICK tr|E1C2W0|E1C2W0\_CHICK tr|R4GFX1|R4GFX1\_CHICK tr|F1NLX0|F1NLX0\_CHICK tr|R4GFN9|R4GFN9\_CHICK tr|E1C9H8|E1C9H8\_CHICK tr|F1NCF9|F1NCF9\_CHICK tr|E1C6Y0|E1C6Y0\_CHICK tr|F1NL05|F1NL05\_CHICK sp|P26446|PARP1\_CHICK tr|B8XA31|B8XA31\_CHICK sp|Q5ZML1|IMA5\_CHICK tr|F1NEX5|F1NEX5\_CHICK tr|Q5ZI46|Q5ZI46\_CHICK tr|Q805B1|Q805B1\_CHICK tr|Q6WJ02|Q6WJ02\_CHICK tr|E1BXP4|E1BXP4\_CHICK tr|E1C271|E1C271\_CHICK tr|E1BZK2|E1BZK2\_CHICK sp|Q6AW68|SC16B\_CHICK sp|Q5F4B2|SWP70\_CHICK tr|R4GLR0|R4GLR0\_CHICK tr|Q7T1E1|Q7T1E1\_CHICK tr|F1P3E5|F1P3E5\_CHICK sp|Q5ZJN0|ASND1\_CHICK tr|F1P2Z1|F1P2Z1\_CHICK tr|E1C1Q7|E1C1Q7\_CHICK tr|G8YY06|G8YY06\_CHICK tr|E1C1K8|E1C1K8\_CHICK tr|E1C1T9|E1C1T9\_CHICK tr|D8UWE0|D8UWE0\_CHICK tr|Q9PTE0|Q9PTE0\_CHICK tr|F1P1X3|F1P1X3\_CHICK sp|Q5ZJZ4|S35G2\_CHICK tr|F1NYY1|F1NYY1\_CHICK tr|Q5ZK81|Q5ZK81\_CHICK tr|F1NBY3|F1NBY3\_CHICK tr|Q5ZIK1|Q5ZIK1\_CHICK tr|F1NWP0|F1NWP0\_CHICK tr|F1P5H4|F1P5H4\_CHICK sp|Q4KWZ7|REV1\_CHICK tr|A7LAP2|A7LAP2\_CHICK tr|Q5F3W8|Q5F3W8\_CHICK tr|E1C5X1|E1C5X1\_CHICK tr|F1NMG8|F1NMG8\_CHICK tr|Q9W6U9|Q9W6U9\_CHICK tr|E1C584|E1C584\_CHICK tr|F1NRE6|F1NRE6\_CHICK tr|E1BQG4|E1BQG4\_CHICK tr|E1BQF1|E1BQF1\_CHICK ENSEMBL:ENSBTAP00000032840 tr|E1C144|E1C144\_CHICK tr|F4ZWC2|F4ZWC2\_CHICK tr|Q5ZMR8|Q5ZMR8\_CHICK tr|E1BZG5|E1BZG5\_CHICK tr|F1NRJ4|F1NRJ4\_CHICK tr|F1NEH6|F1NEH6\_CHICK sp|Q5ZJX5|TLDC1\_CHICK tr|F1NP63|F1NP63\_CHICK Q9D312 tr|F1NKZ5|F1NKZ5\_CHICK tr|F1NJM1|F1NJM1\_CHICK tr|F1NU57|F1NU57\_CHICK tr|Q5ZMI3|Q5ZMI3\_CHICK tr|F1NWE3|F1NWE3\_CHICK tr|E1C7Y1|E1C7Y1\_CHICK tr|F1NB04|F1NB04\_CHICK tr|E1BZD6|E1BZD6\_CHICK tr|F1NRX7|F1NRX7\_CHICK tr|H9CZQ6|H9CZQ6\_CHICK tr|H9CZR1|H9CZR1\_CHICK tr|H9CZQ5|H9CZQ5\_CHICK tr|H9CZQ0|H9CZQ0\_CHICK tr|H9CZR2|H9CZR2\_CHICK tr|H9CZQ9|H9CZQ9\_CHICK tr|H9CZP9|H9CZP9\_CHICK tr|V9ITD3|V9ITD3\_CHICK tr|H9CZQ4|H9CZQ4\_CHICK tr|F1NHL6|F1NHL6\_CHICK sp|Q08156|KIT\_CHICK tr|E1BZP0|E1BZP0\_CHICK tr|C4PCA2|C4PCA2\_GALLA tr|F1NDN4|F1NDN4\_CHICK tr|E1BSJ1|E1BSJ1\_CHICK tr|T1P3Y7|T1P3Y7\_CHICK tr|Q7ZZR8|Q7ZZR8\_CHICK tr|F1P2B3|F1P2B3\_CHICK tr|E1C7A4|E1C7A4\_CHICK tr|E1C2X4|E1C2X4\_CHICK tr|F1NG86|F1NG86\_CHICK sp|Q9YGL9|AT2A3\_CHICK tr|Q5ZL48|Q5ZL48\_CHICK tr|R4GGY9|R4GGY9\_CHICK A2AB72 tr|E1C1V6|E1C1V6\_CHICK tr|Q5ZHW0|Q5ZHW0\_CHICK sp|Q5ZIF1|APMAP\_CHICK tr|E1C732|E1C732\_CHICK tr|F1N9Q9|F1N9Q9\_CHICK tr|E1C4D4|E1C4D4\_CHICK tr|F1NDQ8|F1NDQ8\_CHICK tr|F1NCD3|F1NCD3\_CHICK tr|Q5ZJ61|Q5ZJ61\_CHICK tr|F1P0B0|F1P0B0\_CHICK sp|P26007|ITA6\_CHICK tr|F1NZ31|F1NZ31\_CHICK tr|E1C2B0|E1C2B0\_CHICK tr|F1P2D7|F1P2D7\_CHICK sp|Q5R1S9|CAF1B\_CHICK tr|R4GH29|R4GH29\_CHICK tr|E1BVI8|E1BVI8\_CHICK tr|R4GFA4|R4GFA4\_CHICK sp|Q90701|TEAD3\_CHICK tr|F1NT61|F1NT61\_CHICK tr|I0J179|I0J179\_CHICK tr|E1BSB3|E1BSB3\_CHICK tr|F1NE67|F1NE67\_CHICK tr|M1ZMM1|M1ZMM1\_CHICK tr|Q2QB49|Q2QB49\_CHICK tr|E1C4N7|E1C4N7\_CHICK sp|P00793|PEPA\_CHICK tr|F1NH08|F1NH08\_CHICK tr|Q98TB5|Q98TB5\_CHICK tr|R4GH26|R4GH26\_CHICK tr|F1P1H5|F1P1H5\_CHICK sp|Q5ZKD7|MOV10\_CHICK tr|Q90783|Q90783\_CHICK tr|E1C028|E1C028\_CHICK tr|F1NZ25|F1NZ25\_CHICK tr|E1BSN8|E1BSN8\_CHICK Q3MHN5 tr|Q5F449|Q5F449\_CHICK tr|D2X2H4|D2X2H4\_CHICK tr|Q8AV70|Q8AV70\_CHICK tr|R4GFC7|R4GFC7\_CHICK tr|Q5ZJC0|Q5ZJC0\_CHICK tr|C4PCD8|C4PCD8\_CHICK tr|Q2PUH1|Q2PUH1\_CHICK tr|E1C4L2|E1C4L2\_CHICK tr|Q5ZJV0|Q5ZJV0\_CHICK tr|F1P3G2|F1P3G2\_CHICK tr|H9KYS4|H9KYS4\_CHICK tr|E1BRY2|E1BRY2\_CHICK tr|F1NFP9|F1NFP9\_CHICK tr|F1ND31|F1ND31\_CHICK sp|P02640|VILI\_CHICK tr|E1C894|E1C894\_CHICK tr|R4GKQ1|R4GKQ1\_CHICK tr|F1P554|F1P554\_CHICK tr|F1NCG7|F1NCG7\_CHICK tr|F1NHT6|F1NHT6\_CHICK sp|P15505|GCSP\_CHICK tr|F1NS45|F1NS45\_CHICK sp|Q90674|LSHR\_CHICK tr|F1NFA7|F1NFA7\_CHICK tr|F1NQ45|F1NQ45\_CHICK tr|Q5ZLV3|Q5ZLV3\_CHICK tr|A5HUK7|A5HUK7\_CHICK tr|E6N1V9|E6N1V9\_CHICK tr|E1BQG2|E1BQG2\_CHICK tr|R4GH08|R4GH08\_CHICK tr|E1BSZ8|E1BSZ8\_CHICK tr|Q5ZHV6|Q5ZHV6\_CHICK tr|R4GI42|R4GI42\_CHICK tr|Q9YH41|Q9YH41\_CHICK tr|R4GL38|R4GL38\_CHICK tr|F1NEY8|F1NEY8\_CHICK tr|Q5F3D2|Q5F3D2\_CHICK tr|F1P5W4|F1P5W4\_CHICK tr|F1P1P6|F1P1P6\_CHICK tr|E1BWJ7|E1BWJ7\_CHICK tr|F1NWM3|F1NWM3\_CHICK tr|R4GGH6|R4GGH6\_CHICK tr|F1NW12|F1NW12\_CHICK tr|E1BSU4|E1BSU4\_CHICK tr|E1C8Y8|E1C8Y8\_CHICK tr|F1NDF1|F1NDF1\_CHICK tr|R4GJR8|R4GJR8\_CHICK tr|F1NAE8|F1NAE8\_CHICK tr|E1BRT8|E1BRT8\_CHICK tr|E1BTB7|E1BTB7\_CHICK tr|E1C8T6|E1C8T6\_CHICK tr|F1NTN4|F1NTN4\_CHICK tr|F1NF75|F1NF75\_CHICK tr|F1NZ84|F1NZ84\_CHICK tr|E1BUS2|E1BUS2\_CHICK tr|F1NN95|F1NN95\_CHICK tr|F1P124|F1P124\_CHICK tr|F1NS34|F1NS34\_CHICK tr|E1C5D4|E1C5D4\_CHICK tr|Q9DGN8|Q9DGN8\_CHICK tr|E1C4Q0|E1C4Q0\_CHICK tr|Q98909|Q98909\_CHICK tr|D0VDV7|D0VDV7\_CHICK tr|F1P0R1|F1P0R1\_CHICK tr|F1NYB1|F1NYB1\_CHICK tr|F1NG22|F1NG22\_CHICK tr|Q5ZM42|Q5ZM42\_CHICK tr|F1NLX2|F1NLX2\_CHICK tr|E1BTF4|E1BTF4\_CHICK tr|E1C7F7|E1C7F7\_CHICK tr|E1BU53|E1BU53\_CHICK tr|Q9DG07|Q9DG07\_CHICK tr|F1NGS1|F1NGS1\_CHICK tr|R4GM77|R4GM77\_CHICK tr|E1BZ96|E1BZ96\_CHICK tr|E1BWR0|E1BWR0\_CHICK tr|R4GIV1|R4GIV1\_CHICK tr|F1N9L9|F1N9L9\_CHICK tr|E1C2N5|E1C2N5\_CHICK tr|F6XS45|F6XS45\_CHICK tr|Q5ZIR4|Q5ZIR4\_CHICK tr|F1N9R5|F1N9R5\_CHICK tr|Q9DER6|Q9DER6\_CHICK tr|E1BWG1|E1BWG1\_CHICK sp|Q5ZJ08|SYYC\_CHICK tr|F1NJU7|F1NJU7\_CHICK tr|F1NUD1|F1NUD1\_CHICK tr|F1NGX1|F1NGX1\_CHICK sp|P26008|ITAV\_CHICK tr|E1BUR7|E1BUR7\_CHICK tr|Q69FK3|Q69FK3\_CHICK tr|E1C758|E1C758\_CHICK tr|F1NRW1|F1NRW1\_CHICK tr|O73871|O73871\_CHICK tr|F1NSB6|F1NSB6\_CHICK tr|F1NG04|F1NG04\_CHICK tr|Q5F4A5|Q5F4A5\_CHICK P02676 tr|E1C5Z2|E1C5Z2\_CHICK sp|P26261|SDC3\_CHICK tr|F1NDZ9|F1NDZ9\_CHICK tr|F1P0N1|F1P0N1\_CHICK tr|F1NM78|F1NM78\_CHICK tr|F1NP45|F1NP45\_CHICK tr|Q5ZIP8|Q5ZIP8\_CHICK tr|F1NMS2|F1NMS2\_CHICK tr|E1C4G9|E1C4G9\_CHICK tr|F1N9U6|F1N9U6\_CHICK tr|F1NZE9|F1NZE9\_CHICK tr|E1C8G8|E1C8G8\_CHICK tr|E1C6Y2|E1C6Y2\_CHICK tr|R4GG76|R4GG76\_CHICK tr|F1NEX7|F1NEX7\_CHICK tr|F1NH49|F1NH49\_CHICK tr|E1C0H4|E1C0H4\_CHICK tr|Q9YI56|Q9YI56\_CHICK tr|F1NPL5|F1NPL5\_CHICK sp|Q5ZKI4|CCD93\_CHICK tr|F1P0K1|F1P0K1\_CHICK tr|F1P2M5|F1P2M5\_CHICK tr|Q3V5M0|Q3V5M0\_CHICK tr|E1BYS4|E1BYS4\_CHICK tr|Q3V5M1|Q3V5M1\_CHICK tr|Q3V5L9|Q3V5L9\_CHICK tr|E1C371|E1C371\_CHICK tr|E1C0P0|E1C0P0\_CHICK tr|Q5NTW9|Q5NTW9\_CHICK tr|F1P1F3|F1P1F3\_CHICK tr|Q5ZM23|Q5ZM23\_CHICK tr|F1NLU1|F1NLU1\_CHICK tr|F1NYG1|F1NYG1\_CHICK A2I7N1 tr|A4F5B3|A4F5B3\_CHICK tr|E1C3C2|E1C3C2\_CHICK tr|B5BSF5|B5BSF5\_CHICK tr|E1BRB1|E1BRB1\_CHICK tr|E1BS59|E1BS59\_CHICK tr|E1C3A7|E1C3A7\_CHICK tr|E1BZT6|E1BZT6\_CHICK tr|F1N802|F1N802\_CHICK tr|F1N9J3|F1N9J3\_CHICK sp|P67881|CYC\_CHICK tr|E1C0H6|E1C0H6\_CHICK tr|F1NMA2|F1NMA2\_CHICK tr|E1BQD7|E1BQD7\_CHICK tr|Q9DFY9|Q9DFY9\_CHICK tr|F1NGW6|F1NGW6\_CHICK tr|F1P3W1|F1P3W1\_CHICK tr|Q5ZLI3|Q5ZLI3\_CHICK tr|Q5ZIZ0|Q5ZIZ0\_CHICK tr|F1NQJ1|F1NQJ1\_CHICK tr|E1C951|E1C951\_CHICK tr|Q9DF31|Q9DF31\_CHICK sp|O42410|IKZF1\_CHICK tr|E1BSQ2|E1BSQ2\_CHICK tr|E1C1X8|E1C1X8\_CHICK tr|F1NUE6|F1NUE6\_CHICK sp|P16047|TGFB3\_CHICK tr|E1C7C1|E1C7C1\_CHICK tr|Q5ZMA8|Q5ZMA8\_CHICK tr|Q5F379|Q5F379\_CHICK tr|E1BXC8|E1BXC8\_CHICK tr|E1C8A4|E1C8A4\_CHICK tr|E1C8Q1|E1C8Q1\_CHICK tr|F1P0J2|F1P0J2\_CHICK tr|F1P2E8|F1P2E8\_CHICK sp|Q5F3Z1|PYRG2\_CHICK tr|E1BQS9|E1BQS9\_CHICK tr|R9PXM5|R9PXM5\_CHICK tr|R4GI12|R4GI12\_CHICK tr|R4GF95|R4GF95\_CHICK tr|E1BY59|E1BY59\_CHICK tr|R4GFD5|R4GFD5\_CHICK tr|E1BST3|E1BST3\_CHICK tr|F1NK93|F1NK93\_CHICK sp|Q5ZKK3|APC5\_CHICK tr|R4GHD6|R4GHD6\_CHICK tr|C4PCC7|C4PCC7\_CHICK tr|F1NJK2|F1NJK2\_CHICK tr|C4PCC1|C4PCC1\_GALVA tr|C4PCA4|C4PCA4\_CHICK tr|C4PCC2|C4PCC2\_GALVA sp|P35440|TSP2\_CHICK tr|E1C3P2|E1C3P2\_CHICK tr|Q68BG0|Q68BG0\_CHICK tr|E1BU09|E1BU09\_CHICK tr|A5A2G2|A5A2G2\_CHICK tr|F1NSA8|F1NSA8\_CHICK sp|Q5ZHX1|RAP1B\_CHICK tr|C4PCC0|C4PCC0\_CHICK tr|C4PCA6|C4PCA6\_CHICK tr|E1BRX7|E1BRX7\_CHICK tr|R4GLE4|R4GLE4\_CHICK tr|E5DEQ7|E5DEQ7\_CHICK tr|F1NLT0|F1NLT0\_CHICK tr|R4GM46|R4GM46\_CHICK tr|O57392|O57392\_CHICK tr|F1NAV2|F1NAV2\_CHICK tr|V5Y0M7|V5Y0M7\_CHICK tr|O46792|O46792\_CHICK tr|R4GJ37|R4GJ37\_CHICK tr|R4GFP1|R4GFP1\_CHICK tr|F1N9K0|F1N9K0\_CHICK sp|Q5ZMC2|MYO1G\_CHICK sp|Q5F3I0|PRRC1\_CHICK tr|F1N9H8|F1N9H8\_CHICK tr|H9KYR2|H9KYR2\_CHICK tr|H2CLW0|H2CLW0\_CHICK tr|E1C3A5|E1C3A5\_CHICK tr|F1NNA0|F1NNA0\_CHICK tr|Q5ZIY9|Q5ZIY9\_CHICK tr|E1BVK0|E1BVK0\_CHICK tr|F1P0M3|F1P0M3\_CHICK tr|R4GKM8|R4GKM8\_CHICK tr|B6E281|B6E281\_CHICK tr|F1NQP0|F1NQP0\_CHICK tr|E1C4M6|E1C4M6\_CHICK tr|F1P3Q9|F1P3Q9\_CHICK tr|E1C7T8|E1C7T8\_CHICK tr|F1NAM8|F1NAM8\_CHICK tr|F1N849|F1N849\_CHICK tr|Q9I878|Q9I878\_CHICK tr|R4GF54|R4GF54\_CHICK tr|F1NN77|F1NN77\_CHICK tr|F1NBR7|F1NBR7\_CHICK tr|Q27JC1|Q27JC1\_GALSO tr|F1NBW2|F1NBW2\_CHICK tr|Q788R4|Q788R4\_CHICK tr|R4GJN7|R4GJN7\_CHICK tr|Q788R3|Q788R3\_CHICK tr|F1P4I7|F1P4I7\_CHICK tr|R4GLA4|R4GLA4\_CHICK tr|Q5ZIL0|Q5ZIL0\_CHICK tr|F1NTX8|F1NTX8\_CHICK tr|Q5ZJ72|Q5ZJ72\_CHICK tr|E1BZJ5|E1BZJ5\_CHICK tr|E1C1U3|E1C1U3\_CHICK tr|F1P4W4|F1P4W4\_CHICK tr|E5DFL7|E5DFL7\_CHICK tr|Q6Q1Q8|Q6Q1Q8\_CHICK tr|F1N9E1|F1N9E1\_CHICK sp|Q5ZJQ2|SYFA\_CHICK tr|F1P528|F1P528\_CHICK tr|F1N8T0|F1N8T0\_CHICK tr|E1C3I4|E1C3I4\_CHICK tr|E1BV14|E1BV14\_CHICK tr|E1C1V9|E1C1V9\_CHICK tr|F1NB03|F1NB03\_CHICK tr|Q5ZME5|Q5ZME5\_CHICK tr|F1NW13|F1NW13\_CHICK tr|F1NFJ5|F1NFJ5\_CHICK tr|A0A0D5ZCQ6|A0A0D5ZCQ6\_9VIRU tr|E1C5S5|E1C5S5\_CHICK tr|H9L340|H9L340\_CHICK sp|Q5ZLC5|ATPB\_CHICK tr|F1NXV6|F1NXV6\_CHICK tr|Q91001|Q91001\_CHICK tr|Q8AYM3|Q8AYM3\_CHICK tr|F1NBW8|F1NBW8\_CHICK tr|Q5ZLG7|Q5ZLG7\_CHICK tr|E1C5B3|E1C5B3\_CHICK P34955 tr|R4GKI9|R4GKI9\_CHICK tr|F1NYN7|F1NYN7\_CHICK sp|Q90W79|CNTN5\_CHICK tr|F1NZI4|F1NZI4\_CHICK tr|F6SU00|F6SU00\_CHICK tr|F1NYD1|F1NYD1\_CHICK tr|R4GJP9|R4GJP9\_CHICK tr|Q92064|Q92064\_CHICK tr|I3QHS7|I3QHS7\_CHICK tr|F1NUU7|F1NUU7\_CHICK sp|Q5ZMW5|RHG26\_CHICK tr|F1N894|F1N894\_CHICK tr|F1NAM7|F1NAM7\_CHICK tr|E1BXY2|E1BXY2\_CHICK tr|Q9YHW6|Q9YHW6\_CHICK tr|F1NM67|F1NM67\_CHICK tr|Q92070|Q92070\_CHICK tr|F1NPW8|F1NPW8\_CHICK tr|F1NF84|F1NF84\_CHICK tr|Q5F3M8|Q5F3M8\_CHICK tr|F1N9Z7|F1N9Z7\_CHICK tr|Q802A0|Q802A0\_CHICK tr|F1P158|F1P158\_CHICK tr|F1NZQ9|F1NZQ9\_CHICK tr|R4GFR6|R4GFR6\_CHICK tr|F1NSN0|F1NSN0\_CHICK tr|E1BR08|E1BR08\_CHICK tr|F1NMQ2|F1NMQ2\_CHICK tr|G9F977|G9F977\_9SAUR tr|F1NI13|F1NI13\_CHICK tr|F1NTI5|F1NTI5\_CHICK sp|Q90WU3|DDX1\_CHICK tr|Q5ZKW3|Q5ZKW3\_CHICK tr|R4GHS7|R4GHS7\_CHICK tr|Q5F3P4|Q5F3P4\_CHICK tr|Q5ZHW6|Q5ZHW6\_CHICK tr|R4GJ71|R4GJ71\_CHICK tr|R4GGL6|R4GGL6\_CHICK tr|H9L0R8|H9L0R8\_CHICK tr|F1NQP3|F1NQP3\_CHICK tr|E1C0G5|E1C0G5\_CHICK tr|Q32TG2|Q32TG2\_CHICK tr|F6U0Q8|F6U0Q8\_CHICK tr|R4GIZ4|R4GIZ4\_CHICK tr|F1NSG6|F1NSG6\_CHICK tr|Q5ZIU6|Q5ZIU6\_CHICK tr|F1N9L3|F1N9L3\_CHICK tr|F1P3E7|F1P3E7\_CHICK tr|F1NAK3|F1NAK3\_CHICK tr|F1NYQ2|F1NYQ2\_CHICK tr|F1NSV8|F1NSV8\_CHICK tr|E1C3F8|E1C3F8\_CHICK tr|F1P0B9|F1P0B9\_CHICK tr|F1NW90|F1NW90\_CHICK tr|Q5F4A0|Q5F4A0\_CHICK tr|E1BSH7|E1BSH7\_CHICK tr|F1NT33|F1NT33\_CHICK tr|F1NMW7|F1NMW7\_CHICK tr|Q5F3U7|Q5F3U7\_CHICK tr|F1P578|F1P578\_CHICK sp|Q5ZM83|MIRO2\_CHICK tr|E1C5T8|E1C5T8\_CHICK tr|F1N8X2|F1N8X2\_CHICK tr|F1NDC7|F1NDC7\_CHICK tr|E1BZ62|E1BZ62\_CHICK tr|F1NZC2|F1NZC2\_CHICK tr|Q5ZJ05|Q5ZJ05\_CHICK sp|Q5ZMP7|AP3M1\_CHICK tr|F1P5A3|F1P5A3\_CHICK sp|Q5ZM73|MIRO1\_CHICK tr|R4GGB8|R4GGB8\_CHICK tr|Q9DDR6|Q9DDR6\_CHICK tr|Q92074|Q92074\_CHICK tr|F1P374|F1P374\_CHICK tr|E1C2K4|E1C2K4\_CHICK tr|E1BV99|E1BV99\_CHICK tr|E1C7Q6|E1C7Q6\_CHICK tr|F1NAP3|F1NAP3\_CHICK sp|Q5ZLT3|N4BP3\_CHICK tr|F1NY49|F1NY49\_CHICK tr|F1P194|F1P194\_CHICK tr|F1NL92|F1NL92\_CHICK tr|R4GLY4|R4GLY4\_CHICK tr|F1NPW9|F1NPW9\_CHICK tr|F1NYI5|F1NYI5\_CHICK tr|E1C4A5|E1C4A5\_CHICK tr|O02870|O02870\_CHICK tr|E1C168|E1C168\_CHICK tr|F1NM76|F1NM76\_CHICK tr|Q8JH94|Q8JH94\_CHICK tr|F1NFT1|F1NFT1\_CHICK tr|E1BQ97|E1BQ97\_CHICK tr|Q5EFZ7|Q5EFZ7\_CHICK tr|F6QP49|F6QP49\_CHICK tr|F1P4U5|F1P4U5\_CHICK tr|F1NKQ3|F1NKQ3\_CHICK tr|Q90749|Q90749\_CHICK tr|E1C8Z9|E1C8Z9\_CHICK tr|F1NKT4|F1NKT4\_CHICK tr|F1NTD0|F1NTD0\_CHICK tr|F1NTD1|F1NTD1\_CHICK tr|B0FLP0|B0FLP0\_CHICK tr|Q8UUQ6|Q8UUQ6\_CHICK tr|Q5F351|Q5F351\_CHICK tr|Q9Y621|Q9Y621\_CHICK tr|A1DYI3|A1DYI3\_CHICK tr|Q9DE64|Q9DE64\_CHICK tr|Q3L256|Q3L256\_CHICK tr|E1BRS3|E1BRS3\_CHICK tr|Q4GWK1|Q4GWK1\_GALLA tr|Q5ZI35|Q5ZI35\_CHICK sp|Q5ZM60|CCPG1\_CHICK tr|F1NBB9|F1NBB9\_CHICK tr|F1NJ73|F1NJ73\_CHICK tr|E5L8B7|E5L8B7\_CHICK tr|F1P451|F1P451\_CHICK tr|E1C6W5|E1C6W5\_CHICK tr|F1NSE8|F1NSE8\_CHICK tr|E1C908|E1C908\_CHICK sp|P07916|ELN\_CHICK tr|F1NGB8|F1NGB8\_CHICK sp|P21566|COF2\_CHICK sp|Q5ZJ20|UIF\_CHICK tr|F1NBH2|F1NBH2\_CHICK sp|Q5ZI20|S39AD\_CHICK tr|F6R1X6|F6R1X6\_CHICK sp|Q90647|VATA\_CHICK tr|F1NWP3|F1NWP3\_CHICK tr|E1BX97|E1BX97\_CHICK tr|H9KYZ6|H9KYZ6\_CHICK tr|F1NR88|F1NR88\_CHICK tr|V9GZR2|V9GZR2\_CHICK tr|Q5ZMN6|Q5ZMN6\_CHICK tr|F1NZS9|F1NZS9\_CHICK tr|F1N9A0|F1N9A0\_CHICK tr|E1BUZ4|E1BUZ4\_CHICK tr|F1NXD3|F1NXD3\_CHICK tr|F1NCZ3|F1NCZ3\_CHICK tr|C4PCB0|C4PCB0\_CHICK tr|A0A0A7MEX3|A0A0A7MEX3\_CHICK tr|A0A0A7M9V5|A0A0A7M9V5\_CHICK tr|A0A0A7MA32|A0A0A7MA32\_CHICK tr|C4PCA7|C4PCA7\_CHICK tr|C4PCE5|C4PCE5\_CHICK tr|Q0PQ88|Q0PQ88\_CHICK tr|A1YV56|A1YV56\_CHICK tr|C4PCB4|C4PCB4\_CHICK tr|F1NYG8|F1NYG8\_CHICK tr|F1P3K6|F1P3K6\_CHICK tr|F1NYM0|F1NYM0\_CHICK tr|F1N8L3|F1N8L3\_CHICK tr|Q7T2X3|Q7T2X3\_CHICK tr|R4GM94|R4GM94\_CHICK tr|E1BWD6|E1BWD6\_CHICK tr|Q789D2|Q789D2\_CHICK tr|F1NXE7|F1NXE7\_CHICK tr|Q5ZHL3|Q5ZHL3\_CHICK tr|Q9I8D5|Q9I8D5\_CHICK tr|A1KXM2|A1KXM2\_CHICK tr|R4GJW2|R4GJW2\_CHICK tr|Q90Z43|Q90Z43\_CHICK tr|F1NTI4|F1NTI4\_CHICK tr|F1NYQ7|F1NYQ7\_CHICK sp|Q5ZIG0|NCDN\_CHICK tr|E1C1T7|E1C1T7\_CHICK tr|F1NF37|F1NF37\_CHICK tr|F1NC25|F1NC25\_CHICK tr|F1NI22|F1NI22\_CHICK tr|E1BZV6|E1BZV6\_CHICK tr|R4GHG7|R4GHG7\_CHICK tr|F1NZ42|F1NZ42\_CHICK tr|Q9DEG5|Q9DEG5\_CHICK sp|Q5F3L4|DEN6A\_CHICK tr|F1NGG5|F1NGG5\_CHICK tr|E1C6X0|E1C6X0\_CHICK tr|F1P5J7|F1P5J7\_CHICK tr|F1NJH7|F1NJH7\_CHICK tr|Q5ZHY9|Q5ZHY9\_CHICK tr|Q5ZM43|Q5ZM43\_CHICK tr|H9KZ12|H9KZ12\_CHICK tr|Q06900|Q06900\_CHICK tr|F1NW70|F1NW70\_CHICK tr|F1NQ71|F1NQ71\_CHICK sp|Q5QJC2|DCR1C\_CHICK sp|P49707|CCNE1\_CHICK tr|E1C2V8|E1C2V8\_CHICK sp|P30372|ACM2\_CHICK tr|R4GJW3|R4GJW3\_CHICK tr|F1NV87|F1NV87\_CHICK tr|F1NAE9|F1NAE9\_CHICK tr|R4GFM3|R4GFM3\_CHICK tr|A4F5A9|A4F5A9\_CHICK tr|B5BSB3|B5BSB3\_CHICK sp|Q5ZKH8|ODR4\_CHICK tr|F1NYX9|F1NYX9\_CHICK tr|P79770|P79770\_CHICK tr|F1NEQ7|F1NEQ7\_CHICK tr|F1NSX4|F1NSX4\_CHICK tr|E1BXU3|E1BXU3\_CHICK tr|E1BUS4|E1BUS4\_CHICK tr|G1K360|G1K360\_CHICK sp|O93602|ATF2\_CHICK tr|E1C285|E1C285\_CHICK tr|R4GIH6|R4GIH6\_CHICK sp|Q5ZKP2|MMAD\_CHICK tr|F1NEE9|F1NEE9\_CHICK tr|E1BTJ1|E1BTJ1\_CHICK tr|E1C695|E1C695\_CHICK tr|R4GJ45|R4GJ45\_CHICK tr|Q5ZIA8|Q5ZIA8\_CHICK tr|F1NPF5|F1NPF5\_CHICK A2I7N0 tr|F1P5I3|F1P5I3\_CHICK tr|F1NYF5|F1NYF5\_CHICK tr|Q4GWM1|Q4GWM1\_GALVA tr|Q4GWK8|Q4GWK8\_GALLA tr|Q4GWR0|Q4GWR0\_CHICK tr|E5DFI4|E5DFI4\_CHICK tr|Q4GWJ5|Q4GWJ5\_GALSO tr|A0A0A0R1G6|A0A0A0R1G6\_CHICK tr|Q195I9|Q195I9\_CHICK tr|A0A0B4ZU13|A0A0B4ZU13\_CHICK tr|E5DEM2|E5DEM2\_CHICK tr|E5DEI3|E5DEI3\_CHICK tr|Q4GWN4|Q4GWN4\_CHICK tr|A0A0B4VJG0|A0A0B4VJG0\_CHICK tr|Q76L16|Q76L16\_CHICK tr|E5DEA5|E5DEA5\_CHICK tr|Q4GWP7|Q4GWP7\_CHICK tr|F1NH64|F1NH64\_CHICK tr|E1BRH0|E1BRH0\_CHICK tr|Q8QGM3|Q8QGM3\_CHICK tr|E1C2M2|E1C2M2\_CHICK tr|F1P1G3|F1P1G3\_CHICK tr|F1NY64|F1NY64\_CHICK tr|F1NBD5|F1NBD5\_CHICK tr|F1NY65|F1NY65\_CHICK tr|Q90856|Q90856\_CHICK tr|F1NKF1|F1NKF1\_CHICK tr|Q5F4A6|Q5F4A6\_CHICK tr|F1P495|F1P495\_CHICK tr|F1NB08|F1NB08\_CHICK tr|R4GJX1|R4GJX1\_CHICK sp|Q5ZK74|ASHWN\_CHICK tr|E1C1F8|E1C1F8\_CHICK tr|R4GGK6|R4GGK6\_CHICK tr|F1N8A7|F1N8A7\_CHICK tr|Q5ZI55|Q5ZI55\_CHICK tr|Q5ZJ34|Q5ZJ34\_CHICK tr|H9BPA3|H9BPA3\_CHICK tr|H9BPA4|H9BPA4\_CHICK tr|H9BPA7|H9BPA7\_CHICK sp|Q90Z72|NFIL3\_CHICK tr|F1NVG0|F1NVG0\_CHICK tr|H9L3B7|H9L3B7\_CHICK sp|Q5ZKV9|SYNDE\_CHICK tr|F1NDR7|F1NDR7\_CHICK tr|Q65YQ3|Q65YQ3\_CHICK tr|Q9PSV6|Q9PSV6\_CHICK tr|E1BXD5|E1BXD5\_CHICK tr|E1C718|E1C718\_CHICK tr|E1BSJ3|E1BSJ3\_CHICK tr|F1P0T6|F1P0T6\_CHICK tr|Q5ZMM8|Q5ZMM8\_CHICK tr|F1N8D3|F1N8D3\_CHICK tr|E1C0M2|E1C0M2\_CHICK tr|F1P1V8|F1P1V8\_CHICK sp|Q90673|PRLD1\_CHICK tr|F1NAR2|F1NAR2\_CHICK tr|D0VYS4|D0VYS4\_CHICK tr|F1NL75|F1NL75\_CHICK tr|F1P1C5|F1P1C5\_CHICK tr|Q5F459|Q5F459\_CHICK tr|F1NFN1|F1NFN1\_CHICK tr|F1NBB3|F1NBB3\_CHICK tr|F1P354|F1P354\_CHICK tr|Q5F424|Q5F424\_CHICK sp|Q5ZLR6|ARHG6\_CHICK tr|F1NS33|F1NS33\_CHICK tr|Q000J5|Q000J5\_CHICK tr|Q2TCL1|Q2TCL1\_CHICK tr|F1NW64|F1NW64\_CHICK ENSEMBL:ENSBTAP00000016046 tr|F1NGE5|F1NGE5\_CHICK tr|F1NUZ2|F1NUZ2\_CHICK tr|F1NU20|F1NU20\_CHICK tr|F1NM31|F1NM31\_CHICK tr|E1BVF2|E1BVF2\_CHICK tr|E1C3U2|E1C3U2\_CHICK tr|K4L8I6|K4L8I6\_CHICK tr|E1C2M1|E1C2M1\_CHICK tr|F1N8I8|F1N8I8\_CHICK tr|R9PXN2|R9PXN2\_CHICK tr|Q9DEF6|Q9DEF6\_CHICK tr|E1BR81|E1BR81\_CHICK tr|F1P102|F1P102\_CHICK tr|Q5ZJP2|Q5ZJP2\_CHICK tr|E1C7J6|E1C7J6\_CHICK tr|F1NIQ1|F1NIQ1\_CHICK tr|A0A0A0MQ62|A0A0A0MQ62\_CHICK tr|F1NMD4|F1NMD4\_CHICK tr|E1C8E1|E1C8E1\_CHICK tr|F1NNK6|F1NNK6\_CHICK tr|F1NK38|F1NK38\_CHICK tr|F1NTR0|F1NTR0\_CHICK tr|F1NI86|F1NI86\_CHICK tr|H9KZ06|H9KZ06\_CHICK tr|F1NJA2|F1NJA2\_CHICK tr|E1C3V0|E1C3V0\_CHICK sp|Q5ZI89|TPC11\_CHICK tr|E1C4H1|E1C4H1\_CHICK tr|E1C0F7|E1C0F7\_CHICK tr|E1BWX9|E1BWX9\_CHICK tr|F1NJU5|F1NJU5\_CHICK tr|F1NME3|F1NME3\_CHICK sp|P05180|CP2H1\_CHICK tr|R4GLD5|R4GLD5\_CHICK sp|Q90988|SMC2\_CHICK tr|E1C612|E1C612\_CHICK sp|O93385|PITX2\_CHICK tr|R4GLB4|R4GLB4\_CHICK tr|E1C4K8|E1C4K8\_CHICK tr|F1NAA5|F1NAA5\_CHICK sp|Q5ZKY2|ATG7\_CHICK tr|Q5ZK45|Q5ZK45\_CHICK tr|F1NXL4|F1NXL4\_CHICK tr|F1NAT2|F1NAT2\_CHICK tr|A5A2G1|A5A2G1\_CHICK tr|A4F5B6|A4F5B6\_CHICK sp|P98152|TF65\_CHICK tr|F1NQ69|F1NQ69\_CHICK tr|Q9DGQ2|Q9DGQ2\_CHICK tr|E1C152|E1C152\_CHICK tr|Q5ZL56|Q5ZL56\_CHICK tr|E1BYS6|E1BYS6\_CHICK tr|E1C1I6|E1C1I6\_CHICK tr|H9L2B1|H9L2B1\_CHICK tr|Q90ZK7|Q90ZK7\_CHICK tr|Q9DEF5|Q9DEF5\_CHICK tr|Q5ZJH3|Q5ZJH3\_CHICK tr|F1NPF4|F1NPF4\_CHICK tr|E1C2L9|E1C2L9\_CHICK tr|R4GG36|R4GG36\_CHICK tr|E1BQI9|E1BQI9\_CHICK tr|F1P0Y2|F1P0Y2\_CHICK tr|F1P3P8|F1P3P8\_CHICK tr|F1NY46|F1NY46\_CHICK tr|F6QYT5|F6QYT5\_CHICK tr|E1BTD4|E1BTD4\_CHICK tr|E1C1E7|E1C1E7\_CHICK tr|F1NT27|F1NT27\_CHICK tr|R4GJS8|R4GJS8\_CHICK tr|F1NPN7|F1NPN7\_CHICK tr|E1C1H4|E1C1H4\_CHICK tr|E1C734|E1C734\_CHICK sp|P56517|HDAC1\_CHICK tr|F1NGG8|F1NGG8\_CHICK tr|D6PVB7|D6PVB7\_CHICK tr|E1C708|E1C708\_CHICK tr|H9D0Z9|H9D0Z9\_CHICK tr|F1P308|F1P308\_CHICK tr|Q3HLR0|Q3HLR0\_CHICK tr|E1C4Q3|E1C4Q3\_CHICK sp|Q90631|KTN1\_CHICK tr|F1NIB3|F1NIB3\_CHICK tr|F1NLX9|F1NLX9\_CHICK tr|E1C530|E1C530\_CHICK tr|F1NR25|F1NR25\_CHICK tr|E1C4D2|E1C4D2\_CHICK tr|R4GJS7|R4GJS7\_CHICK tr|R4GLQ6|R4GLQ6\_CHICK tr|E1C4L4|E1C4L4\_CHICK tr|F1P3H3|F1P3H3\_CHICK tr|F1NKT6|F1NKT6\_CHICK tr|F1P5G8|F1P5G8\_CHICK tr|F1NQ20|F1NQ20\_CHICK tr|F1NLQ7|F1NLQ7\_CHICK tr|F1P520|F1P520\_CHICK sp|Q5ZKL7|BIN2\_CHICK tr|E1BS97|E1BS97\_CHICK tr|E1BX03|E1BX03\_CHICK tr|R4GH35|R4GH35\_CHICK tr|H2D5F8|H2D5F8\_CHICK tr|E1C8K9|E1C8K9\_CHICK tr|Q9YGC8|Q9YGC8\_CHICK tr|F1P5Q1|F1P5Q1\_CHICK tr|A6MCV0|A6MCV0\_CHICK tr|F1NG18|F1NG18\_CHICK tr|F1NIJ7|F1NIJ7\_CHICK tr|F1NBQ7|F1NBQ7\_CHICK sp|P56519|HDAC2\_CHICK tr|F1NM39|F1NM39\_CHICK tr|E5DFJ1|E5DFJ1\_CHICK tr|E5DEK3|E5DEK3\_CHICK tr|E5DEZ6|E5DEZ6\_CHICK tr|Q4GWQ3|Q4GWQ3\_CHICK tr|Q9I8D6|Q9I8D6\_CHICK tr|Q7GTU7|Q7GTU7\_CHICK sp|P18939|NU4M\_CHICK tr|A0A0B5A069|A0A0B5A069\_CHICK tr|K9JTR6|K9JTR6\_CHICK tr|A0A0C5AL54|A0A0C5AL54\_CHICK tr|Q4GWP0|Q4GWP0\_CHICK tr|Q4GWM7|Q4GWM7\_CHICK tr|Q4GWR6|Q4GWR6\_GALSO tr|E5DEJ0|E5DEJ0\_CHICK tr|E5DEV7|E5DEV7\_CHICK tr|F1NKY0|F1NKY0\_CHICK tr|F1NKN4|F1NKN4\_CHICK sp|Q5F384|YIPF3\_CHICK tr|E1BS96|E1BS96\_CHICK tr|G5EI88|G5EI88\_CHICK tr|E1BUW8|E1BUW8\_CHICK tr|H9KYP2|H9KYP2\_CHICK tr|F1NSW2|F1NSW2\_CHICK tr|F1NHD1|F1NHD1\_CHICK tr|F1P1U7|F1P1U7\_CHICK tr|Q5ZLA1|Q5ZLA1\_CHICK tr|E1BYU1|E1BYU1\_CHICK tr|F1NAR4|F1NAR4\_CHICK tr|R4GFD6|R4GFD6\_CHICK tr|F1NTY0|F1NTY0\_CHICK sp|Q5F3Z9|ZC3HE\_CHICK tr|E1BWE7|E1BWE7\_CHICK sp|Q90828|DC1L1\_CHICK tr|E1C2D8|E1C2D8\_CHICK sp|O73718|TBX3\_CHICK tr|H9L3N3|H9L3N3\_CHICK tr|F1NMV2|F1NMV2\_CHICK tr|F1NID3|F1NID3\_CHICK tr|F1NKF8|F1NKF8\_CHICK tr|R4GLE5|R4GLE5\_CHICK tr|Q5ZIF9|Q5ZIF9\_CHICK tr|E1BZT7|E1BZT7\_CHICK tr|F1P327|F1P327\_CHICK tr|F1P5A1|F1P5A1\_CHICK tr|E1BTL6|E1BTL6\_CHICK tr|E1BU98|E1BU98\_CHICK sp|P09644|TBA5\_CHICK tr|F1NXR6|F1NXR6\_CHICK tr|R4GL52|R4GL52\_CHICK tr|R4GG85|R4GG85\_CHICK tr|A5A748|A5A748\_CHICK tr|F1NT49|F1NT49\_CHICK tr|R4GIB7|R4GIB7\_CHICK tr|E1C8I6|E1C8I6\_CHICK tr|F1NZV8|F1NZV8\_CHICK tr|A0A0A0MQ58|A0A0A0MQ58\_CHICK sp|Q5ZHS1|ELP3\_CHICK tr|E1BZ20|E1BZ20\_CHICK sp|O73606|KCNG2\_CHICK tr|Q4W5X0|Q4W5X0\_CHICK tr|F1NYM7|F1NYM7\_CHICK tr|F1NY92|F1NY92\_CHICK tr|E1C032|E1C032\_CHICK tr|R4GIZ6|R4GIZ6\_CHICK tr|H9L3N2|H9L3N2\_CHICK tr|R4GJ11|R4GJ11\_CHICK tr|Q7ZWI1|Q7ZWI1\_CHICK tr|Q5ZKL2|Q5ZKL2\_CHICK tr|R4GGR1|R4GGR1\_CHICK tr|E1C1Z5|E1C1Z5\_CHICK tr|Q6Y2V6|Q6Y2V6\_CHICK tr|F1NZH3|F1NZH3\_CHICK tr|Q8AVB3|Q8AVB3\_CHICK tr|Q5ZM69|Q5ZM69\_CHICK tr|F1NZH1|F1NZH1\_CHICK tr|F1NYV0|F1NYV0\_CHICK tr|E1C6T0|E1C6T0\_CHICK Q9DCV7 tr|H9L0A2|H9L0A2\_CHICK sp|Q5ZIH0|INT11\_CHICK tr|H9L0X6|H9L0X6\_CHICK tr|F1NQW3|F1NQW3\_CHICK tr|E1BUQ8|E1BUQ8\_CHICK tr|F1NEI8|F1NEI8\_CHICK tr|F1NQZ6|F1NQZ6\_CHICK tr|Q7ZTG5|Q7ZTG5\_CHICK tr|C4PCF1|C4PCF1\_CHICK tr|C4PCH4|C4PCH4\_CHICK tr|U5UA05|U5UA05\_CHICK tr|I3QHT0|I3QHT0\_CHICK tr|Q3YI10|Q3YI10\_CHICK tr|F1P2L2|F1P2L2\_CHICK tr|Q5ZI83|Q5ZI83\_CHICK tr|R4GK58|R4GK58\_CHICK tr|F1P2E1|F1P2E1\_CHICK tr|D6R189|D6R189\_CHICK tr|A0A0A0V8Q5|A0A0A0V8Q5\_CHICK tr|B8YL09|B8YL09\_CHICK tr|B8YKE0|B8YKE0\_CHICK tr|H9KYV0|H9KYV0\_CHICK tr|F1NFT3|F1NFT3\_CHICK Q99PS0 ENSEMBL:ENSBTAP00000037665 tr|E1BRF7|E1BRF7\_CHICK tr|A0A089FH01|A0A089FH01\_CHICK tr|Q9PWC6|Q9PWC6\_CHICK tr|B8XXD8|B8XXD8\_CHICK tr|R4GGS4|R4GGS4\_CHICK tr|D2I9F2|D2I9F2\_CHICK tr|Q19NX1|Q19NX1\_CHICK sp|P23682|HXB3\_CHICK tr|E5DFN0|E5DFN0\_CHICK tr|F1NKM5|F1NKM5\_CHICK tr|F1P3I7|F1P3I7\_CHICK tr|E1C8U6|E1C8U6\_CHICK sp|P48801|FGF3\_CHICK tr|Q9YHD2|Q9YHD2\_CHICK tr|R4GHU7|R4GHU7\_CHICK sp|Q5F3L9|FA65B\_CHICK tr|E1C7G8|E1C7G8\_CHICK tr|C8KIN3|C8KIN3\_CHICK tr|Q8UUJ6|Q8UUJ6\_CHICK tr|F1NG46|F1NG46\_CHICK tr|Q8AYT2|Q8AYT2\_CHICK tr|C4PCF2|C4PCF2\_CHICK tr|Q8UVX3|Q8UVX3\_CHICK tr|E1C8S3|E1C8S3\_CHICK sp|Q5ZIV9|ADSV\_CHICK tr|F1NMD8|F1NMD8\_CHICK tr|E1BVN5|E1BVN5\_CHICK tr|Q5ZMK8|Q5ZMK8\_CHICK tr|F1P2Q2|F1P2Q2\_CHICK tr|E1C106|E1C106\_CHICK tr|F1P4U4|F1P4U4\_CHICK sp|Q6U7I1|UBP7\_CHICK tr|F1P0V8|F1P0V8\_CHICK tr|Q5ZMC8|Q5ZMC8\_CHICK tr|E1BQI4|E1BQI4\_CHICK tr|F1NIH1|F1NIH1\_CHICK sp|Q5ZJJ1|ZC11A\_CHICK tr|F1NLA7|F1NLA7\_CHICK sp|P09206|TBB3\_CHICK tr|H9KZ13|H9KZ13\_CHICK tr|F1NPB1|F1NPB1\_CHICK tr|F1NT10|F1NT10\_CHICK tr|Q5ZI24|Q5ZI24\_CHICK tr|F1NQU3|F1NQU3\_CHICK tr|E1C1M6|E1C1M6\_CHICK tr|E1C8H2|E1C8H2\_CHICK tr|B8YLW8|B8YLW8\_CHICK tr|F1NR31|F1NR31\_CHICK tr|F1NPQ5|F1NPQ5\_CHICK tr|F1NG54|F1NG54\_CHICK tr|E1C6M8|E1C6M8\_CHICK tr|E1BZ73|E1BZ73\_CHICK tr|F1CN09|F1CN09\_CHICK tr|E5L948|E5L948\_CHICK tr|H9L3N6|H9L3N6\_CHICK tr|E1C5V5|E1C5V5\_CHICK tr|R4GFN8|R4GFN8\_CHICK tr|E1C6J5|E1C6J5\_CHICK tr|F1NNN7|F1NNN7\_CHICK tr|H9L2I7|H9L2I7\_CHICK tr|Q6X7S8|Q6X7S8\_CHICK tr|F1NA81|F1NA81\_CHICK tr|F1NGS5|F1NGS5\_CHICK tr|E1C3L8|E1C3L8\_CHICK tr|Q4A1V5|Q4A1V5\_CHICK sp|Q90592|GTR2\_CHICK tr|R4GHH3|R4GHH3\_CHICK tr|H9L3K5|H9L3K5\_CHICK tr|E1C7S7|E1C7S7\_CHICK tr|E1C8Z8|E1C8Z8\_CHICK tr|E1C7E5|E1C7E5\_CHICK tr|F1NRM1|F1NRM1\_CHICK tr|E5DEL2|E5DEL2\_CHICK tr|F1NX17|F1NX17\_CHICK tr|Q90ZN3|Q90ZN3\_CHICK sp|Q5ZL77|RIC8A\_CHICK tr|Q90809|Q90809\_CHICK tr|E1C0P8|E1C0P8\_CHICK tr|Q5ZL10|Q5ZL10\_CHICK tr|F1P300|F1P300\_CHICK tr|F1NBS9|F1NBS9\_CHICK tr|E1C3K1|E1C3K1\_CHICK tr|F1N8T5|F1N8T5\_CHICK tr|E1C5E3|E1C5E3\_CHICK tr|F1NWD5|F1NWD5\_CHICK tr|F1NCH3|F1NCH3\_CHICK tr|R4GH41|R4GH41\_CHICK tr|Q5ZK58|Q5ZK58\_CHICK tr|F1P1M1|F1P1M1\_CHICK sp|Q91987|NTRK2\_CHICK tr|R4GFL5|R4GFL5\_CHICK tr|F1NIG2|F1NIG2\_CHICK tr|F1NZT2|F1NZT2\_CHICK tr|G8HT74|G8HT74\_CHICK tr|F1NHC8|F1NHC8\_CHICK tr|F1NYA5|F1NYA5\_CHICK tr|E1BW42|E1BW42\_CHICK tr|E1C476|E1C476\_CHICK tr|E1BQJ7|E1BQJ7\_CHICK tr|E1BZM0|E1BZM0\_CHICK tr|R4GKU2|R4GKU2\_CHICK tr|R4GKN3|R4GKN3\_CHICK sp|Q5ZKK2|INT9\_CHICK tr|F1NP54|F1NP54\_CHICK tr|F1NJ07|F1NJ07\_CHICK tr|E1C265|E1C265\_CHICK tr|F1P494|F1P494\_CHICK tr|F1NEG9|F1NEG9\_CHICK tr|F1NW11|F1NW11\_CHICK tr|B3GS87|B3GS87\_CHICK tr|F1NAM2|F1NAM2\_CHICK tr|B3GS86|B3GS86\_CHICK tr|Q8UUQ8|Q8UUQ8\_CHICK sp|P14731|LMNB1\_CHICK tr|F1NI04|F1NI04\_CHICK sp|P32017|CO9A3\_CHICK tr|F1NZI1|F1NZI1\_CHICK tr|E1C4E9|E1C4E9\_CHICK sp|P30997|CTNA2\_CHICK tr|F1NHD4|F1NHD4\_CHICK tr|F1NJD6|F1NJD6\_CHICK tr|F1P0L2|F1P0L2\_CHICK tr|Q6R7Z7|Q6R7Z7\_CHICK tr|F1N8P3|F1N8P3\_CHICK tr|Q9BD54|Q9BD54\_CHICK tr|E1BWA3|E1BWA3\_CHICK tr|E1BVN3|E1BVN3\_CHICK tr|E2RUH0|E2RUH0\_CHICK tr|E1C569|E1C569\_CHICK tr|F1NDP3|F1NDP3\_CHICK tr|F1NXA6|F1NXA6\_CHICK tr|E1BYZ5|E1BYZ5\_CHICK tr|R4GL12|R4GL12\_CHICK tr|R4GG27|R4GG27\_CHICK tr|F1NMW8|F1NMW8\_CHICK sp|F1NBL0|MUC6\_CHICK sp|Q5ZLK6|KI18B\_CHICK tr|F1CN52|F1CN52\_CHICK tr|Q5ZKZ7|Q5ZKZ7\_CHICK tr|F1NZB3|F1NZB3\_CHICK tr|F1NBG5|F1NBG5\_CHICK tr|H9KZQ2|H9KZQ2\_CHICK tr|F1NJ16|F1NJ16\_CHICK tr|O73840|O73840\_CHICK tr|E1BZG0|E1BZG0\_CHICK tr|F1NVF2|F1NVF2\_CHICK tr|H9L338|H9L338\_CHICK sp|P79778|TBXT\_CHICK tr|E1BZW6|E1BZW6\_CHICK tr|G1JRK4|G1JRK4\_CHICK tr|F1NXV7|F1NXV7\_CHICK tr|Q90YB5|Q90YB5\_CHICK tr|Q9PTX1|Q9PTX1\_CHICK tr|F1ND78|F1ND78\_CHICK tr|F1NQ57|F1NQ57\_CHICK tr|F1NRD5|F1NRD5\_CHICK tr|R4GHU9|R4GHU9\_CHICK tr|R4GJU2|R4GJU2\_CHICK tr|E1BWH3|E1BWH3\_CHICK tr|F1NB25|F1NB25\_CHICK tr|R4GG47|R4GG47\_CHICK tr|F1NTF9|F1NTF9\_CHICK tr|B8YKK9|B8YKK9\_CHICK tr|B8YK86|B8YK86\_CHICK tr|B8YKL8|B8YKL8\_CHICK tr|B8YL39|B8YL39\_CHICK tr|E1C852|E1C852\_CHICK tr|B8YKB6|B8YKB6\_CHICK tr|E1BYJ8|E1BYJ8\_CHICK sp|Q76KB1|HS2ST\_CHICK tr|F1NED2|F1NED2\_CHICK sp|Q50L44|LIGO1\_CHICK tr|Q98TG8|Q98TG8\_CHICK tr|Q8UUR2|Q8UUR2\_CHICK tr|F1NAH6|F1NAH6\_CHICK tr|F1NVD5|F1NVD5\_CHICK tr|A4F5B2|A4F5B2\_CHICK tr|A4F5B4|A4F5B4\_CHICK tr|B5BSM4|B5BSM4\_CHICK sp|O73895|TPSN\_CHICK tr|R4GKL0|R4GKL0\_CHICK tr|F1NPI1|F1NPI1\_CHICK tr|Q31407|Q31407\_CHICK tr|Q31600|Q31600\_CHICK tr|Q31608|Q31608\_CHICK tr|E1BQG6|E1BQG6\_CHICK tr|R4GLA7|R4GLA7\_CHICK tr|E1C372|E1C372\_CHICK tr|Q9DFS5|Q9DFS5\_CHICK tr|F1NHX1|F1NHX1\_CHICK sp|P09572|AT1A1\_CHICK tr|F1NQZ9|F1NQZ9\_CHICK tr|C4PCC3|C4PCC3\_GALSO tr|C4PCA1|C4PCA1\_GALLA tr|C4PCC4|C4PCC4\_GALSO tr|E1BZI8|E1BZI8\_CHICK tr|Q5ZJ70|Q5ZJ70\_CHICK tr|F1NZC5|F1NZC5\_CHICK tr|E1C125|E1C125\_CHICK tr|R4GM36|R4GM36\_CHICK tr|F1NMD2|F1NMD2\_CHICK tr|F1NE04|F1NE04\_CHICK tr|Q5ZIG9|Q5ZIG9\_CHICK sp|Q5ZJL9|SAMH1\_CHICK tr|F1P0T3|F1P0T3\_CHICK tr|H9L0M4|H9L0M4\_CHICK tr|E1BVV2|E1BVV2\_CHICK tr|Q90VW3|Q90VW3\_CHICK tr|Q90VU9|Q90VU9\_CHICK tr|F6UXQ7|F6UXQ7\_CHICK tr|F1N868|F1N868\_CHICK tr|Q90VZ5|Q90VZ5\_CHICK tr|Q90VV7|Q90VV7\_CHICK tr|F1NUR8|F1NUR8\_CHICK sp|P02597|CALMS\_CHICK sp|P62597|POTE1\_CHICK tr|F1NYZ4|F1NYZ4\_CHICK tr|F1NVJ2|F1NVJ2\_CHICK tr|F1P5Y9|F1P5Y9\_CHICK tr|Q90612|Q90612\_CHICK sp|P04210|LV1\_CHICK P02754 tr|F1NDP4|F1NDP4\_CHICK tr|E1BUL4|E1BUL4\_CHICK tr|E1BZ35|E1BZ35\_CHICK tr|G9F979|G9F979\_9SAUR tr|E1BRA5|E1BRA5\_CHICK tr|F1NNK7|F1NNK7\_CHICK tr|R4GGP2|R4GGP2\_CHICK tr|E1BZT3|E1BZT3\_CHICK tr|F1NYJ9|F1NYJ9\_CHICK tr|F1N965|F1N965\_CHICK tr|F1NY41|F1NY41\_CHICK tr|F1NRW5|F1NRW5\_CHICK tr|E1BVP2|E1BVP2\_CHICK tr|F1NWY3|F1NWY3\_CHICK tr|H9L0J4|H9L0J4\_CHICK tr|F1NED1|F1NED1\_CHICK tr|Q5ZJZ9|Q5ZJZ9\_CHICK tr|F1NXX0|F1NXX0\_CHICK tr|R4GLT4|R4GLT4\_CHICK tr|O42394|O42394\_CHICK tr|E1BQ64|E1BQ64\_CHICK tr|E1C4J1|E1C4J1\_CHICK tr|E1BTE9|E1BTE9\_CHICK tr|E1C8H7|E1C8H7\_CHICK tr|H9L2B5|H9L2B5\_CHICK sp|Q9YIB9|HIF1A\_CHICK tr|Q5ZJ79|Q5ZJ79\_CHICK tr|E1BZS3|E1BZS3\_CHICK sp|Q5ZJP9|CCNL1\_CHICK tr|F1NPF6|F1NPF6\_CHICK tr|Q6JGT2|Q6JGT2\_CHICK sp|Q5ZMW0|AGO4\_CHICK sp|Q5ZLG4|AGO3\_CHICK tr|F1P3Z0|F1P3Z0\_CHICK tr|F1NEA9|F1NEA9\_CHICK tr|Q5F3X2|Q5F3X2\_CHICK tr|F1NW79|F1NW79\_CHICK sp|Q0V8S9|CNTP5\_CHICK tr|F1P448|F1P448\_CHICK tr|Q9PRG9|Q9PRG9\_CHICK sp|Q5ZI16|ADAT1\_CHICK tr|E1C1I3|E1C1I3\_CHICK tr|F1P1L0|F1P1L0\_CHICK tr|F1NW24|F1NW24\_CHICK tr|F1NCA2|F1NCA2\_CHICK tr|E1BVA2|E1BVA2\_CHICK tr|F1NSQ3|F1NSQ3\_CHICK tr|Q5ZJH4|Q5ZJH4\_CHICK tr|E1C0A4|E1C0A4\_CHICK tr|F1NS92|F1NS92\_CHICK tr|Q8UVS4|Q8UVS4\_CHICK tr|F1NMF6|F1NMF6\_CHICK sp|P24802|PLOD1\_CHICK tr|E1C2P8|E1C2P8\_CHICK tr|F1NBP4|F1NBP4\_CHICK tr|R4GI57|R4GI57\_CHICK tr|E1C0Q2|E1C0Q2\_CHICK sp|E1C2Q8|AMER2\_CHICK tr|R4GME0|R4GME0\_CHICK tr|E1C6U1|E1C6U1\_CHICK tr|E1C3E3|E1C3E3\_CHICK tr|F1NYK2|F1NYK2\_CHICK tr|F1N9Y3|F1N9Y3\_CHICK tr|Q8JIM6|Q8JIM6\_CHICK tr|F1NKP5|F1NKP5\_CHICK tr|F1P4J1|F1P4J1\_CHICK tr|F1NUX7|F1NUX7\_CHICK tr|O42489|O42489\_CHICK O76013 tr|E1C1Z8|E1C1Z8\_CHICK sp|P23913|LBR\_CHICK tr|F6UZR6|F6UZR6\_CHICK tr|R4GK97|R4GK97\_CHICK tr|R4GHZ7|R4GHZ7\_CHICK tr|E1C8I3|E1C8I3\_CHICK tr|F1NU61|F1NU61\_CHICK tr|E1C0T5|E1C0T5\_CHICK tr|Q5ZLJ5|Q5ZLJ5\_CHICK tr|H9BPA6|H9BPA6\_CHICK tr|F1NYN5|F1NYN5\_CHICK tr|F1P3U4|F1P3U4\_CHICK tr|F1P2J9|F1P2J9\_CHICK tr|Q6R6I2|Q6R6I2\_CHICK tr|F1NKW5|F1NKW5\_CHICK tr|Q5ZLP5|Q5ZLP5\_CHICK tr|E1BUV0|E1BUV0\_CHICK tr|F1P500|F1P500\_CHICK tr|R4GJV0|R4GJV0\_CHICK tr|F1P2B9|F1P2B9\_CHICK sp|P18461|FGFR2\_CHICK tr|E1BQR8|E1BQR8\_CHICK tr|E1BSZ6|E1BSZ6\_CHICK tr|F1NTM6|F1NTM6\_CHICK tr|F1P4S4|F1P4S4\_CHICK tr|Q7LZ57|Q7LZ57\_CHICK tr|R4GG28|R4GG28\_CHICK tr|B6Z9R2|B6Z9R2\_CHICK tr|R4GHW9|R4GHW9\_CHICK tr|E1BYC6|E1BYC6\_CHICK tr|F1NXU8|F1NXU8\_CHICK tr|E1BZQ4|E1BZQ4\_CHICK tr|R4GM64|R4GM64\_CHICK tr|F1NIT4|F1NIT4\_CHICK sp|Q90593|GRP78\_CHICK tr|R4GMC5|R4GMC5\_CHICK tr|E1BYF5|E1BYF5\_CHICK tr|Q5ZKA0|Q5ZKA0\_CHICK tr|C3V8R3|C3V8R3\_CHICK tr|R4GK17|R4GK17\_CHICK tr|F1NYI3|F1NYI3\_CHICK tr|F1NPH5|F1NPH5\_CHICK tr|R4GL59|R4GL59\_CHICK tr|E1C434|E1C434\_CHICK sp|P10741|MOS\_CHICK tr|R4GFU6|R4GFU6\_CHICK tr|E1C2J3|E1C2J3\_CHICK tr|Q98916|Q98916\_CHICK tr|Q90976|Q90976\_CHICK tr|F1NTM0|F1NTM0\_CHICK tr|Q76CE9|Q76CE9\_CHICK tr|F1NU22|F1NU22\_CHICK tr|Q5ILH2|Q5ILH2\_CHICK tr|F1P5G2|F1P5G2\_CHICK tr|F1NDZ7|F1NDZ7\_CHICK tr|E1BW08|E1BW08\_CHICK tr|E1C676|E1C676\_CHICK tr|F1NX32|F1NX32\_CHICK ENSEMBL:ENSBTAP00000001528 tr|F1P555|F1P555\_CHICK tr|Q4ADW0|Q4ADW0\_CHICK tr|E1C2E3|E1C2E3\_CHICK sp|P08125|COAA1\_CHICK tr|E1C406|E1C406\_CHICK sp|Q5ZKI6|GXLT1\_CHICK tr|Q9BCW3|Q9BCW3\_CHICK tr|F1NTY3|F1NTY3\_CHICK tr|R4GL71|R4GL71\_CHICK tr|B8YLT5|B8YLT5\_GALVA tr|H9KZW7|H9KZW7\_CHICK tr|F1NGC2|F1NGC2\_CHICK tr|F1NMS3|F1NMS3\_CHICK tr|K4HZ50|K4HZ50\_CHICK tr|E1C7H3|E1C7H3\_CHICK tr|F1P0J8|F1P0J8\_CHICK sp|P33005|KALM\_CHICK tr|F1NGD7|F1NGD7\_CHICK tr|F1NNJ3|F1NNJ3\_CHICK tr|E1BQS6|E1BQS6\_CHICK tr|R4GG14|R4GG14\_CHICK tr|F1P0W8|F1P0W8\_CHICK tr|F1NY76|F1NY76\_CHICK tr|F1P0W3|F1P0W3\_CHICK tr|F1NMK8|F1NMK8\_CHICK tr|F1NAR9|F1NAR9\_CHICK tr|F1NEZ1|F1NEZ1\_CHICK tr|Q5ZIH6|Q5ZIH6\_CHICK tr|F1P396|F1P396\_CHICK tr|Q8AYS4|Q8AYS4\_CHICK tr|F1NA48|F1NA48\_CHICK tr|Q9PWP7|Q9PWP7\_CHICK sp|P18652|KS6AA\_CHICK tr|R4GG18|R4GG18\_CHICK tr|E1BSI9|E1BSI9\_CHICK tr|F1NM73|F1NM73\_CHICK tr|F1NU68|F1NU68\_CHICK tr|E1C5G5|E1C5G5\_CHICK Q9UE12 Q15323 tr|F1NM59|F1NM59\_CHICK sp|Q00944|FAK1\_CHICK tr|F1NLS6|F1NLS6\_CHICK tr|F1NY30|F1NY30\_CHICK tr|F6V5T5|F6V5T5\_CHICK sp|Q5ZL33|STRAP\_CHICK tr|E1C2F8|E1C2F8\_CHICK tr|E1BTD0|E1BTD0\_CHICK tr|F1P2X2|F1P2X2\_CHICK tr|E1C579|E1C579\_CHICK tr|E1C291|E1C291\_CHICK tr|R4GL15|R4GL15\_CHICK tr|F1NF56|F1NF56\_CHICK sp|Q5F4B1|PGP\_CHICK tr|F1NMH3|F1NMH3\_CHICK tr|R9RVW8|R9RVW8\_CHICK tr|R4GI25|R4GI25\_CHICK tr|E3VLA1|E3VLA1\_CHICK tr|H9L106|H9L106\_CHICK tr|C7ACT2|C7ACT2\_CHICK sp|P36196|ACES\_CHICK tr|F1N9J7|F1N9J7\_CHICK tr|F1NXK0|F1NXK0\_CHICK tr|F1P1X0|F1P1X0\_CHICK tr|F1NSX5|F1NSX5\_CHICK sp|Q5ZHX5|MSD2\_CHICK tr|Q5ZJ04|Q5ZJ04\_CHICK tr|F1NBW6|F1NBW6\_CHICK tr|R4GGU0|R4GGU0\_CHICK tr|F1NWG9|F1NWG9\_CHICK tr|F1NYY2|F1NYY2\_CHICK tr|Q5F3M4|Q5F3M4\_CHICK tr|F1NN82|F1NN82\_CHICK tr|Q9I9L4|Q9I9L4\_CHICK tr|Q5ZKC8|Q5ZKC8\_CHICK tr|Q6ZXD1|Q6ZXD1\_CHICK tr|H2CLV9|H2CLV9\_CHICK tr|E1BR36|E1BR36\_CHICK sp|P51475|OPSP\_CHICK tr|F1NLK3|F1NLK3\_CHICK tr|F1NMF5|F1NMF5\_CHICK tr|F1NZJ6|F1NZJ6\_CHICK sp|Q02977|YRK\_CHICK tr|Q9YH86|Q9YH86\_CHICK tr|F1NXP1|F1NXP1\_CHICK tr|R4GH81|R4GH81\_CHICK tr|Q9DG27|Q9DG27\_CHICK tr|F1NLW3|F1NLW3\_CHICK Q9N2I2 tr|F1NPP2|F1NPP2\_CHICK sp|Q5ZIK6|BSDC1\_CHICK tr|F1NNV0|F1NNV0\_CHICK sp|P24045|GBRB4\_CHICK tr|F1CN17|F1CN17\_CHICK tr|F1P4K3|F1P4K3\_CHICK tr|E1BXF2|E1BXF2\_CHICK tr|F1NPY4|F1NPY4\_CHICK tr|O42108|O42108\_CHICK tr|R4GH76|R4GH76\_CHICK sp|Q5ZJA4|ARP5\_CHICK tr|D3YC78|D3YC78\_CHICK tr|F1NIM6|F1NIM6\_CHICK tr|E1BRI5|E1BRI5\_CHICK tr|F1NPE8|F1NPE8\_CHICK tr|O42403|O42403\_CHICK tr|R4GJP6|R4GJP6\_CHICK tr|Q9I8K5|Q9I8K5\_CHICK tr|E1BVD1|E1BVD1\_CHICK tr|E1BWE1|E1BWE1\_CHICK tr|F1NUL7|F1NUL7\_CHICK sp|Q5ZLG3|DIEXF\_CHICK tr|F1NHV1|F1NHV1\_CHICK tr|E1C8A6|E1C8A6\_CHICK tr|F1NZV5|F1NZV5\_CHICK tr|H9KYX7|H9KYX7\_CHICK tr|R4GFD4|R4GFD4\_CHICK tr|A0A0A7MAE1|A0A0A7MAE1\_CHICK tr|F1P4V5|F1P4V5\_CHICK tr|E1C262|E1C262\_CHICK tr|F1N8W6|F1N8W6\_CHICK tr|F1NDM3|F1NDM3\_CHICK tr|E1BR35|E1BR35\_CHICK sp|Q5ZLR2|IKZF5\_CHICK tr|R4GKH6|R4GKH6\_CHICK tr|R4GHF9|R4GHF9\_CHICK tr|F1NHA6|F1NHA6\_CHICK tr|E1BUZ2|E1BUZ2\_CHICK tr|Q91961|Q91961\_CHICK tr|E1C591|E1C591\_CHICK tr|Q90886|Q90886\_CHICK tr|E1BQZ9|E1BQZ9\_CHICK tr|Q5ZI88|Q5ZI88\_CHICK tr|Q5ZK29|Q5ZK29\_CHICK tr|F1NB95|F1NB95\_CHICK tr|Q5ZLZ3|Q5ZLZ3\_CHICK tr|F1P4W7|F1P4W7\_CHICK tr|F1NWA3|F1NWA3\_CHICK tr|E1C9I6|E1C9I6\_CHICK tr|E1BS61|E1BS61\_CHICK tr|F1NBF1|F1NBF1\_CHICK tr|Q49L21|Q49L21\_CHICK tr|D5LPR1|D5LPR1\_CHICK tr|E1BWF1|E1BWF1\_CHICK tr|F1NNU4|F1NNU4\_CHICK tr|E1C5S3|E1C5S3\_CHICK tr|Q91000|Q91000\_CHICK tr|R9PXL5|R9PXL5\_CHICK tr|A9DA44|A9DA44\_CHICK tr|F1NTQ1|F1NTQ1\_CHICK tr|F1NZV3|F1NZV3\_CHICK tr|Q6R0H9|Q6R0H9\_CHICK tr|F1NPI7|F1NPI7\_CHICK tr|D3KR66|D3KR66\_CHICK tr|Q9DDR4|Q9DDR4\_CHICK tr|Q9DDR5|Q9DDR5\_CHICK tr|R4GHQ8|R4GHQ8\_CHICK tr|F1NVY2|F1NVY2\_CHICK tr|F1NEZ0|F1NEZ0\_CHICK tr|E1BQX5|E1BQX5\_CHICK tr|F1P290|F1P290\_CHICK tr|F1NFS3|F1NFS3\_CHICK sp|Q5ZLS7|GLYR1\_CHICK tr|E1BT98|E1BT98\_CHICK sp|Q08705|CTCF\_CHICK tr|F1NLD6|F1NLD6\_CHICK Q497I4 tr|F1NEF6|F1NEF6\_CHICK tr|F1NV21|F1NV21\_CHICK tr|F1NY08|F1NY08\_CHICK tr|E1BZ54|E1BZ54\_CHICK tr|E1C0H7|E1C0H7\_CHICK sp|E1C213|UBP37\_CHICK tr|R9PXQ8|R9PXQ8\_CHICK tr|Q5EES2|Q5EES2\_CHICK tr|F6V444|F6V444\_CHICK tr|E1BVQ4|E1BVQ4\_CHICK tr|E1BVS8|E1BVS8\_CHICK tr|Q5F3F0|Q5F3F0\_CHICK tr|R4GM71|R4GM71\_CHICK tr|F1N8R0|F1N8R0\_CHICK tr|E1BU15|E1BU15\_CHICK tr|F1ND44|F1ND44\_CHICK sp|P16419|MYPC2\_CHICK Q862S4 tr|E1C493|E1C493\_CHICK tr|F1NTL1|F1NTL1\_CHICK tr|E1BT87|E1BT87\_CHICK tr|F1NDX5|F1NDX5\_CHICK tr|Q90748|Q90748\_CHICK tr|F1NRW2|F1NRW2\_CHICK tr|F1NNT7|F1NNT7\_CHICK tr|Q9YI50|Q9YI50\_CHICK tr|Q5ZIC3|Q5ZIC3\_CHICK tr|F1NE50|F1NE50\_CHICK tr|C4PBN6|C4PBN6\_GALLA sp|Q5ZIQ3|HNRPK\_CHICK tr|F1P1T1|F1P1T1\_CHICK tr|Q5ZMG7|Q5ZMG7\_CHICK tr|R4GI86|R4GI86\_CHICK tr|F1NGT3|F1NGT3\_CHICK tr|F1P3Y8|F1P3Y8\_CHICK tr|Q5ZM87|Q5ZM87\_CHICK tr|M1NJW4|M1NJW4\_CHICK tr|R4GL32|R4GL32\_CHICK tr|E1C0F1|E1C0F1\_CHICK tr|Q6DMS2|Q6DMS2\_CHICK tr|Q5ZKG7|Q5ZKG7\_CHICK sp|Q5F479|S11IP\_CHICK tr|F1NBZ8|F1NBZ8\_CHICK sp|P37069|OLF3\_CHICK tr|E1C957|E1C957\_CHICK tr|F1NNT3|F1NNT3\_CHICK tr|R4GKL1|R4GKL1\_CHICK sp|P49337|WNT4\_CHICK tr|E1BZY9|E1BZY9\_CHICK tr|F1NPA2|F1NPA2\_CHICK sp|Q5F3R7|DCA12\_CHICK tr|Q6XBN6|Q6XBN6\_CHICK tr|O42390|O42390\_CHICK tr|H9L2G9|H9L2G9\_CHICK tr|E1C027|E1C027\_CHICK tr|F1NBR9|F1NBR9\_CHICK tr|F1NYW8|F1NYW8\_CHICK tr|F1NSC4|F1NSC4\_CHICK tr|E1C4E8|E1C4E8\_CHICK sp|Q5ZK47|STRAA\_CHICK tr|F1NZ34|F1NZ34\_CHICK tr|E1BXJ6|E1BXJ6\_CHICK tr|F1P289|F1P289\_CHICK tr|F1N9W1|F1N9W1\_CHICK sp|Q7T199|KCA10\_CHICK tr|R4GI72|R4GI72\_CHICK tr|C0J3M4|C0J3M4\_CHICK tr|E1BXJ4|E1BXJ4\_CHICK sp|P25022|RAG2\_CHICK tr|F1NPP4|F1NPP4\_CHICK tr|A0A077K956|A0A077K956\_CHICK tr|E1C153|E1C153\_CHICK sp|Q5ZLX4|ARHG3\_CHICK tr|F1P5U0|F1P5U0\_CHICK sp|P98150|NFKB2\_CHICK tr|F1NBN0|F1NBN0\_CHICK tr|F1P5U1|F1P5U1\_CHICK tr|F1NTN2|F1NTN2\_CHICK sp|P07812|PRGR\_CHICK tr|Q90W23|Q90W23\_CHICK tr|Q5ZKV5|Q5ZKV5\_CHICK tr|Q8AYP6|Q8AYP6\_CHICK tr|E1BYG6|E1BYG6\_CHICK sp|P18943|COX1\_CHICK tr|E1C222|E1C222\_CHICK tr|E1C1A5|E1C1A5\_CHICK tr|E1BWU9|E1BWU9\_CHICK tr|E1BV19|E1BV19\_CHICK tr|R4GGY5|R4GGY5\_CHICK tr|E1BZR6|E1BZR6\_CHICK tr|F1NPR9|F1NPR9\_CHICK tr|F1NDD5|F1NDD5\_CHICK tr|F1NR62|F1NR62\_CHICK tr|F1NUW4|F1NUW4\_CHICK tr|E1BU97|E1BU97\_CHICK tr|A5HSH7|A5HSH7\_CHICK tr|R4GHD7|R4GHD7\_CHICK ENSEMBL:ENSP00000377550 tr|F1NNQ2|F1NNQ2\_CHICK tr|E1C0H0|E1C0H0\_CHICK tr|Q5XXX1|Q5XXX1\_CHICK sp|Q5ZMK5|JMJD6\_CHICK tr|R4GGN2|R4GGN2\_CHICK P09870 tr|F1NGR8|F1NGR8\_CHICK sp|Q5ZM98|GRP75\_CHICK tr|Q3MUH5|Q3MUH5\_CHICK tr|F1ND90|F1ND90\_CHICK tr|E1BYL9|E1BYL9\_CHICK sp|Q5ZL95|FA46C\_CHICK tr|Q5ZHU7|Q5ZHU7\_CHICK tr|F1P0T9|F1P0T9\_CHICK tr|R4GKK6|R4GKK6\_CHICK tr|E1BQN8|E1BQN8\_CHICK tr|Q5ZJZ1|Q5ZJZ1\_CHICK tr|O02869|O02869\_CHICK sp|Q98919|LSAMP\_CHICK tr|R4GL35|R4GL35\_CHICK tr|E1C7F5|E1C7F5\_CHICK tr|E1C671|E1C671\_CHICK tr|R4GFG7|R4GFG7\_CHICK tr|E1C7W5|E1C7W5\_CHICK tr|F1P502|F1P502\_CHICK tr|Q5RL90|Q5RL90\_CHICK tr|C4PCC6|C4PCC6\_GALSO tr|E1C3I5|E1C3I5\_CHICK tr|Q9IAK2|Q9IAK2\_CHICK sp|Q9YGC1|BLNK\_CHICK tr|R4GF57|R4GF57\_CHICK tr|F1NKU0|F1NKU0\_CHICK tr|F1NR46|F1NR46\_CHICK tr|F1NU46|F1NU46\_CHICK tr|Q1EGJ6|Q1EGJ6\_CHICK tr|F1N9F7|F1N9F7\_CHICK tr|F1P4M2|F1P4M2\_CHICK tr|F1NZL3|F1NZL3\_CHICK tr|F1P3F5|F1P3F5\_CHICK sp|O13154|PACN2\_CHICK tr|E1BQ95|E1BQ95\_CHICK tr|E5DFD1|E5DFD1\_CHICK tr|Q90808|Q90808\_CHICK tr|F1NF40|F1NF40\_CHICK Q8IUT8 Q3KUS7 tr|F1P4Y1|F1P4Y1\_CHICK tr|F1NDH6|F1NDH6\_CHICK sp|P23824|GATA2\_CHICK tr|A6N8N7|A6N8N7\_CHICK tr|R4GKJ7|R4GKJ7\_CHICK tr|H9L029|H9L029\_CHICK tr|F1NYZ1|F1NYZ1\_CHICK tr|E1BY52|E1BY52\_CHICK tr|E1BTM0|E1BTM0\_CHICK tr|B5LEQ3|B5LEQ3\_CHICK tr|E1C135|E1C135\_CHICK tr|E1C687|E1C687\_CHICK tr|H9KZU3|H9KZU3\_CHICK tr|F1NV27|F1NV27\_CHICK tr|Q1PA18|Q1PA18\_CHICK tr|E1C690|E1C690\_CHICK tr|F1N829|F1N829\_CHICK tr|E1BX17|E1BX17\_CHICK tr|F1P0M2|F1P0M2\_CHICK sp|Q5ZLD7|VPS53\_CHICK sp|Q5ZI67|NHLC2\_CHICK sp|Q5ZJ02|ZN326\_CHICK tr|E1C1S6|E1C1S6\_CHICK tr|A7UEB1|A7UEB1\_CHICK tr|E1C8B4|E1C8B4\_CHICK tr|F1NBL5|F1NBL5\_CHICK tr|F1NTA4|F1NTA4\_CHICK tr|Q2PT42|Q2PT42\_CHICK tr|F1NCF1|F1NCF1\_CHICK tr|F1NE21|F1NE21\_CHICK tr|E1BZF9|E1BZF9\_CHICK tr|F1NRN7|F1NRN7\_CHICK tr|F1P4V1|F1P4V1\_CHICK sp|P14448|FIBA\_CHICK tr|F1NNV2|F1NNV2\_CHICK tr|R4GK02|R4GK02\_CHICK tr|F1NDP7|F1NDP7\_CHICK sp|Q5F3N9|CF106\_CHICK tr|R4GJW9|R4GJW9\_CHICK tr|F1NVS7|F1NVS7\_CHICK tr|Q7T198|Q7T198\_CHICK tr|F1NPM2|F1NPM2\_CHICK tr|Q9IAM3|Q9IAM3\_CHICK tr|F1NWZ1|F1NWZ1\_CHICK sp|Q9YGS0|IRX4\_CHICK tr|R4GMH9|R4GMH9\_CHICK tr|E1BR31|E1BR31\_CHICK tr|R4GI23|R4GI23\_CHICK Q92764 tr|O57378|O57378\_CHICK tr|F1NQJ7|F1NQJ7\_CHICK sp|Q91379|NR2E1\_CHICK tr|E1C3D8|E1C3D8\_CHICK sp|Q5ZII9|TFP11\_CHICK tr|E1BR29|E1BR29\_CHICK tr|Q8UUT7|Q8UUT7\_CHICK tr|Q5F3H0|Q5F3H0\_CHICK tr|F1NYH1|F1NYH1\_CHICK tr|F1NTV9|F1NTV9\_CHICK tr|Q49M61|Q49M61\_CHICK sp|P05099|MATN1\_CHICK tr|F1NM06|F1NM06\_CHICK tr|F1NZD2|F1NZD2\_CHICK tr|F1NN92|F1NN92\_CHICK tr|E1BV96|E1BV96\_CHICK tr|Q5ZL86|Q5ZL86\_CHICK tr|E1BZQ3|E1BZQ3\_CHICK sp|P68034|ACTC\_CHICK tr|F1P476|F1P476\_CHICK tr|F1P0Q8|F1P0Q8\_CHICK tr|Q5ZKF2|Q5ZKF2\_CHICK sp|P63270|ACTH\_CHICK sp|Q5ZJH9|DKC1\_CHICK sp|P08023|ACTA\_CHICK tr|R4GIT4|R4GIT4\_CHICK tr|F1NJ74|F1NJ74\_CHICK tr|H9L0E7|H9L0E7\_CHICK tr|Q6WJ00|Q6WJ00\_CHICK tr|E1BUY3|E1BUY3\_CHICK sp|Q5ZKY0|TM175\_CHICK tr|F1NYE2|F1NYE2\_CHICK tr|H9L0L3|H9L0L3\_CHICK tr|E1BUR5|E1BUR5\_CHICK tr|F1NTD5|F1NTD5\_CHICK tr|E1C7T9|E1C7T9\_CHICK tr|E1BQU5|E1BQU5\_CHICK tr|E1BUZ7|E1BUZ7\_CHICK tr|F1NI96|F1NI96\_CHICK tr|F1P4I9|F1P4I9\_CHICK tr|E1C5U6|E1C5U6\_CHICK tr|K4EEL6|K4EEL6\_CHICK tr|Q0GMA8|Q0GMA8\_CHICK tr|E1C4X9|E1C4X9\_CHICK tr|R4GF92|R4GF92\_CHICK tr|R4GFN2|R4GFN2\_CHICK tr|C4PBP8|C4PBP8\_CHICK tr|F1NGQ2|F1NGQ2\_CHICK tr|F1N9R1|F1N9R1\_CHICK tr|F1NJG0|F1NJG0\_CHICK tr|F1NCL8|F1NCL8\_CHICK tr|F1NDR5|F1NDR5\_CHICK tr|R4GJE1|R4GJE1\_CHICK tr|E1BWM0|E1BWM0\_CHICK tr|O73667|O73667\_CHICK tr|Q6E6M6|Q6E6M6\_CHICK tr|E1C5K0|E1C5K0\_CHICK tr|Q6E6M5|Q6E6M5\_CHICK tr|F1NQE8|F1NQE8\_CHICK tr|Q52Z74|Q52Z74\_CHICK tr|F1NJL8|F1NJL8\_CHICK sp|P49024|PAXI\_CHICK tr|F1NSD7|F1NSD7\_CHICK tr|E1C4R0|E1C4R0\_CHICK tr|R4GKX6|R4GKX6\_CHICK tr|E1C5U1|E1C5U1\_CHICK tr|F1NI05|F1NI05\_CHICK tr|E1C4F6|E1C4F6\_CHICK tr|F1NQD4|F1NQD4\_CHICK tr|Q5F4C5|Q5F4C5\_CHICK tr|Q5ZM52|Q5ZM52\_CHICK tr|Q5F3R1|Q5F3R1\_CHICK tr|E1BR02|E1BR02\_CHICK tr|R4GHN5|R4GHN5\_CHICK tr|F1NS13|F1NS13\_CHICK tr|F1NU53|F1NU53\_CHICK tr|F1NG73|F1NG73\_CHICK tr|F1NBP2|F1NBP2\_CHICK tr|F1ND14|F1ND14\_CHICK tr|F1NNV6|F1NNV6\_CHICK tr|R4GGA4|R4GGA4\_CHICK tr|R4GJ31|R4GJ31\_CHICK tr|E1BZ06|E1BZ06\_CHICK tr|Q30D05|Q30D05\_CHICK tr|F1NUW8|F1NUW8\_CHICK tr|F1P150|F1P150\_CHICK sp|Q71R50|DHR11\_CHICK tr|E1BUZ1|E1BUZ1\_CHICK tr|F1NIY4|F1NIY4\_CHICK tr|D3WGL6|D3WGL6\_CHICK tr|F1NV11|F1NV11\_CHICK tr|F1NRB2|F1NRB2\_CHICK tr|E1BXK3|E1BXK3\_CHICK tr|C4PCB6|C4PCB6\_CHICK tr|R4GKJ1|R4GKJ1\_CHICK tr|F1P5G9|F1P5G9\_CHICK tr|T1P465|T1P465\_CHICK tr|T1P482|T1P482\_CHICK tr|E1C9G8|E1C9G8\_CHICK sp|P14154|CXA1\_CHICK tr|F1N9K3|F1N9K3\_CHICK tr|Q90990|Q90990\_CHICK tr|Q5ZM27|Q5ZM27\_CHICK tr|E1C029|E1C029\_CHICK tr|F1NJS4|F1NJS4\_CHICK tr|F1ND18|F1ND18\_CHICK tr|H9L0C4|H9L0C4\_CHICK tr|F1NCS2|F1NCS2\_CHICK tr|F1NDZ8|F1NDZ8\_CHICK tr|E1BSK2|E1BSK2\_CHICK tr|Q5ZMW1|Q5ZMW1\_CHICK tr|Q8AYI3|Q8AYI3\_CHICK tr|R4GGE8|R4GGE8\_CHICK tr|F1NMQ5|F1NMQ5\_CHICK tr|B8XA32|B8XA32\_CHICK tr|E1C4A6|E1C4A6\_CHICK tr|F1NCD6|F1NCD6\_CHICK tr|Q6DRR5|Q6DRR5\_CHICK tr|N0GSP8|N0GSP8\_9SAUR tr|Q8QFN9|Q8QFN9\_CHICK tr|F1NVA1|F1NVA1\_CHICK tr|F1NJH8|F1NJH8\_CHICK tr|F7B5T0|F7B5T0\_CHICK tr|Q5ZLS5|Q5ZLS5\_CHICK tr|F1N996|F1N996\_CHICK tr|F1NDZ6|F1NDZ6\_CHICK tr|F1NRH2|F1NRH2\_CHICK tr|A0A0E3VN65|A0A0E3VN65\_CHICK tr|E1C8E8|E1C8E8\_CHICK tr|O93259|O93259\_CHICK tr|R4GGE4|R4GGE4\_CHICK tr|F1CMZ8|F1CMZ8\_CHICK tr|F1NB16|F1NB16\_CHICK tr|F1P3S5|F1P3S5\_CHICK tr|Q9DER2|Q9DER2\_CHICK tr|E1C7R2|E1C7R2\_CHICK tr|R4GGD0|R4GGD0\_CHICK tr|Q9DG06|Q9DG06\_CHICK tr|Q9DG05|Q9DG05\_CHICK tr|E1BXE6|E1BXE6\_CHICK tr|F1NA37|F1NA37\_CHICK tr|F1NWS6|F1NWS6\_CHICK tr|E1BRT2|E1BRT2\_CHICK tr|E1BQQ5|E1BQQ5\_CHICK sp|Q5ZIN2|SEN2\_CHICK tr|E1C7K2|E1C7K2\_CHICK tr|E1BS75|E1BS75\_CHICK tr|E1C1Z1|E1C1Z1\_CHICK tr|E1BW24|E1BW24\_CHICK tr|R4GH61|R4GH61\_CHICK tr|F1P0R6|F1P0R6\_CHICK tr|R4GGN0|R4GGN0\_CHICK sp|Q5ZLW2|ORAI2\_CHICK tr|R4GFI6|R4GFI6\_CHICK sp|Q10751|ACE\_CHICK tr|Q90998|Q90998\_CHICK tr|E1BZ03|E1BZ03\_CHICK tr|B9VMA9|B9VMA9\_CHICK tr|A0A0C4ZMV7|A0A0C4ZMV7\_9NEOP tr|F1NKZ7|F1NKZ7\_CHICK tr|F1P1M9|F1P1M9\_CHICK tr|E1BUV5|E1BUV5\_CHICK tr|E1C7T5|E1C7T5\_CHICK tr|E1BW66|E1BW66\_CHICK tr|F1NBA8|F1NBA8\_CHICK tr|Q5ZK16|Q5ZK16\_CHICK sp|P00940|TPIS\_CHICK tr|F1NEI2|F1NEI2\_CHICK tr|H9L3L2|H9L3L2\_CHICK tr|Q5F354|Q5F354\_CHICK sp|Q5ZJK8|TCPH\_CHICK tr|E1BWN9|E1BWN9\_CHICK tr|E1C3Z3|E1C3Z3\_CHICK tr|E1C095|E1C095\_CHICK tr|F1N8K5|F1N8K5\_CHICK tr|F1NMN5|F1NMN5\_CHICK tr|F1NUQ4|F1NUQ4\_CHICK tr|F1NT95|F1NT95\_CHICK tr|F1NLF8|F1NLF8\_CHICK sp|Q5ZKB9|DDX6\_CHICK tr|R4GG41|R4GG41\_CHICK sp|Q5F3K0|TTC27\_CHICK tr|F1P046|F1P046\_CHICK sp|Q9W770|SPON1\_CHICK tr|F1NTX9|F1NTX9\_CHICK tr|E1BS74|E1BS74\_CHICK tr|Q5F430|Q5F430\_CHICK sp|Q5ZKY9|MED20\_CHICK tr|E1C458|E1C458\_CHICK tr|E1C031|E1C031\_CHICK tr|F1NKK7|F1NKK7\_CHICK ENSEMBL:ENSBTAP00000025008 tr|E1C1C5|E1C1C5\_CHICK sp|Q8QGQ7|BMAL2\_CHICK tr|F1NBA5|F1NBA5\_CHICK tr|E1C8D3|E1C8D3\_CHICK tr|E1BXX5|E1BXX5\_CHICK Q61726 tr|F1NMH1|F1NMH1\_CHICK tr|E1BQ46|E1BQ46\_CHICK tr|Q5ILG9|Q5ILG9\_CHICK tr|R4GI21|R4GI21\_CHICK tr|O13017|O13017\_CHICK tr|Q5ZJC9|Q5ZJC9\_CHICK tr|E1BZ53|E1BZ53\_CHICK tr|Q90725|Q90725\_CHICK tr|F1NUF6|F1NUF6\_CHICK tr|Q860I0|Q860I0\_CHICK tr|Q31400|Q31400\_CHICK tr|Q5ZI14|Q5ZI14\_CHICK tr|R4GIZ0|R4GIZ0\_CHICK tr|E1BZG4|E1BZG4\_CHICK tr|Q9IAU0|Q9IAU0\_CHICK tr|F1NWL6|F1NWL6\_CHICK sp|F1N8V3|PHAR4\_CHICK tr|E1C550|E1C550\_CHICK tr|F1N8Y5|F1N8Y5\_CHICK tr|E1C7R7|E1C7R7\_CHICK tr|Q9PWK1|Q9PWK1\_CHICK tr|Q9W643|Q9W643\_CHICK tr|Q9IB15|Q9IB15\_CHICK sp|Q5ZMV9|WDR24\_CHICK tr|F1NND9|F1NND9\_CHICK tr|Q98SH5|Q98SH5\_CHICK tr|E1BZR5|E1BZR5\_CHICK tr|F1N8A3|F1N8A3\_CHICK tr|Q5ZM21|Q5ZM21\_CHICK tr|F1P557|F1P557\_CHICK tr|E1BTL2|E1BTL2\_CHICK tr|F1P0F9|F1P0F9\_CHICK tr|E1BW09|E1BW09\_CHICK tr|B6IDG6|B6IDG6\_CHICK sp|Q9DE07|NBN\_CHICK sp|Q5ZJ40|RCOR3\_CHICK tr|E1BXE9|E1BXE9\_CHICK sp|P55879|GLI2\_CHICK tr|F1NZ24|F1NZ24\_CHICK tr|Q5F3F1|Q5F3F1\_CHICK tr|E1BS26|E1BS26\_CHICK sp|Q802T2|ALG6\_CHICK tr|E1BUT6|E1BUT6\_CHICK sp|Q8AXV0|SH3G1\_CHICK tr|R4GFJ1|R4GFJ1\_CHICK tr|E1C220|E1C220\_CHICK tr|F1P3F9|F1P3F9\_CHICK tr|R4GLK4|R4GLK4\_CHICK tr|F1NCZ8|F1NCZ8\_CHICK tr|F1N9I5|F1N9I5\_CHICK tr|Q9PW24|Q9PW24\_CHICK tr|R4GK64|R4GK64\_CHICK tr|E1BZC5|E1BZC5\_CHICK tr|Q68SB0|Q68SB0\_CHICK tr|R4GH20|R4GH20\_CHICK tr|F1NC97|F1NC97\_CHICK tr|E1BWW2|E1BWW2\_CHICK tr|Q90892|Q90892\_CHICK tr|R4GLS7|R4GLS7\_CHICK tr|Q3YI09|Q3YI09\_CHICK tr|F1NEU3|F1NEU3\_CHICK tr|F1P0Z0|F1P0Z0\_CHICK tr|E1C275|E1C275\_CHICK tr|F1N9J9|F1N9J9\_CHICK tr|Q5ZID9|Q5ZID9\_CHICK tr|Q5ZLH5|Q5ZLH5\_CHICK tr|Q5ZJT5|Q5ZJT5\_CHICK tr|R4GGG1|R4GGG1\_CHICK tr|R4GL94|R4GL94\_CHICK tr|F1P144|F1P144\_CHICK tr|F1NML7|F1NML7\_CHICK tr|H9L011|H9L011\_CHICK tr|E1BSL1|E1BSL1\_CHICK tr|F1NJP3|F1NJP3\_CHICK tr|Q2VQV8|Q2VQV8\_CHICK tr|Q5ZJV8|Q5ZJV8\_CHICK tr|E1BTT4|E1BTT4\_CHICK tr|F1NJ18|F1NJ18\_CHICK tr|Q5ZM00|Q5ZM00\_CHICK tr|A5AA28|A5AA28\_CHICK tr|R9PXP1|R9PXP1\_CHICK tr|B4X7N2|B4X7N2\_CHICK tr|F1NJN5|F1NJN5\_CHICK tr|Q5ZKG6|Q5ZKG6\_CHICK sp|P14092|ATP6\_CHICK tr|Q4GWN1|Q4GWN1\_CHICK tr|Q4GWQ7|Q4GWQ7\_CHICK tr|E5DFI7|E5DFI7\_CHICK tr|Q7GTU9|Q7GTU9\_CHICK tr|N0DRQ7|N0DRQ7\_CHICK tr|E5DEA8|E5DEA8\_CHICK tr|Q4GWS0|Q4GWS0\_GALSO tr|E5DF83|E5DF83\_CHICK tr|E5DEN8|E5DEN8\_CHICK tr|Q4GWP4|Q4GWP4\_CHICK tr|F1NBC7|F1NBC7\_CHICK tr|Q5F472|Q5F472\_CHICK tr|F1NX76|F1NX76\_CHICK tr|Q9PVT8|Q9PVT8\_CHICK tr|E0WMW1|E0WMW1\_CHICK tr|Q805A9|Q805A9\_CHICK tr|E1BTI1|E1BTI1\_CHICK tr|F1NHK0|F1NHK0\_CHICK tr|F1NYU6|F1NYU6\_CHICK tr|E1C945|E1C945\_CHICK tr|E1C4E6|E1C4E6\_CHICK tr|R4GFK2|R4GFK2\_CHICK sp|Q5ZIH9|CC174\_CHICK tr|F1P299|F1P299\_CHICK tr|F1NL36|F1NL36\_CHICK tr|R4GH30|R4GH30\_CHICK tr|E1BTC0|E1BTC0\_CHICK tr|Q6Y2V7|Q6Y2V7\_CHICK tr|Q6Y2W1|Q6Y2W1\_CHICK tr|Q5ZMU1|Q5ZMU1\_CHICK tr|A0A023PTY3|A0A023PTY3\_CHICK tr|A0A023PTQ9|A0A023PTQ9\_CHICK tr|E1BYL2|E1BYL2\_CHICK tr|Q5ZLN9|Q5ZLN9\_CHICK tr|F1NBR3|F1NBR3\_CHICK sp|Q98ST7|MOXD1\_CHICK tr|F1NG65|F1NG65\_CHICK tr|R4GGG9|R4GGG9\_CHICK sp|P37068|OLF2\_CHICK tr|F1NM68|F1NM68\_CHICK tr|H9KZE9|H9KZE9\_CHICK tr|F1NAH9|F1NAH9\_CHICK sp|Q6U1I9|SGK1\_CHICK sp|Q5ZKG2|BRD7\_CHICK tr|F1P5S9|F1P5S9\_CHICK tr|F1P1I4|F1P1I4\_CHICK tr|Q9DG15|Q9DG15\_CHICK tr|E1C0N1|E1C0N1\_CHICK tr|E1BRC0|E1BRC0\_CHICK tr|Q90970|Q90970\_CHICK tr|F1NVX7|F1NVX7\_CHICK tr|E1C963|E1C963\_CHICK tr|F1NAX3|F1NAX3\_CHICK tr|E1C055|E1C055\_CHICK tr|F1NU91|F1NU91\_CHICK tr|F1NF63|F1NF63\_CHICK tr|F1NN74|F1NN74\_CHICK tr|F1NS02|F1NS02\_CHICK tr|Q5F3U8|Q5F3U8\_CHICK tr|F1NH09|F1NH09\_CHICK tr|Q5ZLJ2|Q5ZLJ2\_CHICK tr|F1NEF8|F1NEF8\_CHICK tr|F1NEI0|F1NEI0\_CHICK tr|E1C292|E1C292\_CHICK tr|F1P3F2|F1P3F2\_CHICK tr|C4PCA8|C4PCA8\_CHICK tr|C4PCE6|C4PCE6\_CHICK tr|Q5ZJS2|Q5ZJS2\_CHICK tr|F7AY11|F7AY11\_CHICK tr|C4PCA9|C4PCA9\_CHICK tr|E1BXW4|E1BXW4\_CHICK tr|R4GJ47|R4GJ47\_CHICK tr|F1NMM6|F1NMM6\_CHICK tr|Q9I8C8|Q9I8C8\_CHICK tr|E1BVK3|E1BVK3\_CHICK tr|F1NXP0|F1NXP0\_CHICK sp|Q5ZMD6|H2AZ\_CHICK tr|F1NI37|F1NI37\_CHICK tr|F1N9A7|F1N9A7\_CHICK tr|E1BVA1|E1BVA1\_CHICK tr|F1NNA1|F1NNA1\_CHICK tr|Q5ZK76|Q5ZK76\_CHICK tr|F1P0Z2|F1P0Z2\_CHICK tr|F1N886|F1N886\_CHICK tr|Q704X2|Q704X2\_CHICK tr|Q75UT8|Q75UT8\_CHICK tr|Q90579|Q90579\_CHICK tr|F1P1U8|F1P1U8\_CHICK tr|F1NL08|F1NL08\_CHICK sp|Q5ZJH7|CNPD1\_CHICK tr|E1C8V1|E1C8V1\_CHICK tr|F1NB67|F1NB67\_CHICK tr|R4GJE4|R4GJE4\_CHICK tr|Q5J7B6|Q5J7B6\_CHICK tr|F1NMC5|F1NMC5\_CHICK tr|R4GK51|R4GK51\_CHICK tr|E1BST9|E1BST9\_CHICK tr|F1NV49|F1NV49\_CHICK tr|F1P0Y6|F1P0Y6\_CHICK tr|E1C6G6|E1C6G6\_CHICK tr|C4PCF5|C4PCF5\_CHICK tr|F1P399|F1P399\_CHICK tr|E1BRH7|E1BRH7\_CHICK tr|F1NG21|F1NG21\_CHICK tr|E1C1L7|E1C1L7\_CHICK sp|Q90867|HNF1A\_CHICK tr|Q5ZK42|Q5ZK42\_CHICK tr|Q5ZKQ2|Q5ZKQ2\_CHICK tr|R4GHX9|R4GHX9\_CHICK P13646-1 tr|Q5KTT9|Q5KTT9\_CHICK tr|R4GI74|R4GI74\_CHICK sp|Q5ZML4|CHRD1\_CHICK tr|A3EYS4|A3EYS4\_CHICK tr|F1NQ40|F1NQ40\_CHICK tr|E1BQD2|E1BQD2\_CHICK tr|F1NDR6|F1NDR6\_CHICK tr|F1P2V7|F1P2V7\_CHICK tr|F1NTP3|F1NTP3\_CHICK tr|F1NVV1|F1NVV1\_CHICK tr|F1NBY1|F1NBY1\_CHICK sp|Q5ZLN5|TADBP\_CHICK sp|Q7SZI5|RFTN1\_CHICK tr|F1P375|F1P375\_CHICK tr|E1BX13|E1BX13\_CHICK sp|Q90Z16|OPTN\_CHICK tr|R4GIR1|R4GIR1\_CHICK tr|O42349|O42349\_CHICK tr|F1NT00|F1NT00\_CHICK tr|F1NUF4|F1NUF4\_CHICK tr|F1NMC1|F1NMC1\_CHICK tr|E1C3I1|E1C3I1\_CHICK tr|O13256|O13256\_CHICK tr|O13255|O13255\_CHICK tr|R4GJK0|R4GJK0\_CHICK tr|Q90YA3|Q90YA3\_CHICK tr|R4GJ84|R4GJ84\_CHICK tr|F1NLT4|F1NLT4\_CHICK tr|Q5ZIS7|Q5ZIS7\_CHICK tr|E1BVU6|E1BVU6\_CHICK tr|F1NUY0|F1NUY0\_CHICK tr|F1NDR4|F1NDR4\_CHICK sp|Q5ZM55|FEM1B\_CHICK tr|F1NBL6|F1NBL6\_CHICK tr|F1NKK8|F1NKK8\_CHICK sp|Q9W6U8|MEF2A\_CHICK tr|F1N8P7|F1N8P7\_CHICK sp|O93436|STAM2\_CHICK tr|E1C0T2|E1C0T2\_CHICK tr|F1NQG6|F1NQG6\_CHICK tr|F1NDA1|F1NDA1\_CHICK tr|F1NSE3|F1NSE3\_CHICK tr|Q9W610|Q9W610\_CHICK tr|F1NTK0|F1NTK0\_CHICK tr|E1C0W2|E1C0W2\_CHICK sp|P43692|GATA5\_CHICK tr|E1BVP8|E1BVP8\_CHICK tr|F1NUR7|F1NUR7\_CHICK tr|J9R0I1|J9R0I1\_9SAUR tr|F1P4G4|F1P4G4\_CHICK tr|F1NKR8|F1NKR8\_CHICK sp|Q5ZMI4|MVP\_CHICK tr|F1P5D1|F1P5D1\_CHICK tr|Q5ZMH0|Q5ZMH0\_CHICK tr|F1NWB7|F1NWB7\_CHICK sp|P08110|ENPL\_CHICK tr|Q90WA6|Q90WA6\_CHICK tr|F1NTH8|F1NTH8\_CHICK tr|E1BYU3|E1BYU3\_CHICK tr|F1NPC3|F1NPC3\_CHICK tr|F1P556|F1P556\_CHICK tr|E1C1C7|E1C1C7\_CHICK tr|F1NXA7|F1NXA7\_CHICK tr|E1BV98|E1BV98\_CHICK tr|E1C337|E1C337\_CHICK tr|B5BSM0|B5BSM0\_CHICK tr|F1P125|F1P125\_CHICK tr|E1C748|E1C748\_CHICK tr|F1N9Y2|F1N9Y2\_CHICK tr|F1NFM5|F1NFM5\_CHICK tr|F1NL39|F1NL39\_CHICK tr|F6TLI2|F6TLI2\_CHICK tr|F1NVL7|F1NVL7\_CHICK sp|P17200|ACM4\_CHICK tr|F1NUG3|F1NUG3\_CHICK tr|F1NPA5|F1NPA5\_CHICK tr|E1BXQ9|E1BXQ9\_CHICK tr|Q5F341|Q5F341\_CHICK sp|P09654|VIME\_CHICK tr|F1NVQ5|F1NVQ5\_CHICK tr|Q71SG3|Q71SG3\_CHICK tr|Q71SG4|Q71SG4\_CHICK sp|Q03237|MYBB\_CHICK tr|E1BZS0|E1BZS0\_CHICK sp|Q5ZI13|DNJC3\_CHICK tr|B5AAV6|B5AAV6\_CHICK tr|F1N969|F1N969\_CHICK tr|F1P053|F1P053\_CHICK sp|Q5ZLM0|CDC73\_CHICK tr|R9PXM9|R9PXM9\_CHICK sp|Q5ZKI7|CB044\_CHICK sp|P29616|MYSC\_CHICK sp|Q5ZJK0|RGPS1\_CHICK tr|E1BU46|E1BU46\_CHICK tr|O13036|O13036\_CHICK tr|E1C0C2|E1C0C2\_CHICK tr|E1C6I5|E1C6I5\_CHICK sp|Q5ZKN5|FA53A\_CHICK tr|F1P4C6|F1P4C6\_CHICK tr|E1BSA0|E1BSA0\_CHICK tr|F1P1R9|F1P1R9\_CHICK tr|F1N8F1|F1N8F1\_CHICK tr|Q5ZK24|Q5ZK24\_CHICK tr|Q8UWH0|Q8UWH0\_CHICK tr|F1NCE9|F1NCE9\_CHICK tr|Q2YHU5|Q2YHU5\_CHICK tr|E1BW35|E1BW35\_CHICK tr|B0M1D7|B0M1D7\_CHICK tr|H9L0W8|H9L0W8\_CHICK tr|F1NRZ3|F1NRZ3\_CHICK P78386 tr|E1BTG6|E1BTG6\_CHICK tr|E1C6V6|E1C6V6\_CHICK tr|F1NA70|F1NA70\_CHICK tr|Q5ZKE3|Q5ZKE3\_CHICK tr|F1NGI1|F1NGI1\_CHICK sp|Q76KB2|H6ST1\_CHICK sp|P21642|PCKGM\_CHICK tr|E1C1A8|E1C1A8\_CHICK tr|F1NCX2|F1NCX2\_CHICK tr|O57513|O57513\_CHICK tr|F1NMS0|F1NMS0\_CHICK tr|F1NR57|F1NR57\_CHICK sp|P18302|DREB\_CHICK tr|F1P1F8|F1P1F8\_CHICK tr|F1NJ88|F1NJ88\_CHICK tr|F1NC03|F1NC03\_CHICK tr|Q67BJ2|Q67BJ2\_CHICK tr|Q9IAI5|Q9IAI5\_CHICK tr|Q5ZLS0|Q5ZLS0\_CHICK sp|Q8QG60|CRY2\_CHICK tr|F1P3P5|F1P3P5\_CHICK tr|E1BTV0|E1BTV0\_CHICK tr|R4GI46|R4GI46\_CHICK tr|E1C7K6|E1C7K6\_CHICK tr|E1BYD4|E1BYD4\_CHICK tr|E1BRQ3|E1BRQ3\_CHICK tr|Q5F4A2|Q5F4A2\_CHICK sp|O57401|SOX1\_CHICK tr|Q9YHW5|Q9YHW5\_CHICK tr|F1P378|F1P378\_CHICK tr|Q5ZHQ8|Q5ZHQ8\_CHICK tr|F1NP36|F1NP36\_CHICK tr|F1NHD7|F1NHD7\_CHICK tr|E1BZ05|E1BZ05\_CHICK tr|F1NJU1|F1NJU1\_CHICK sp|Q5ZKD9|KLH20\_CHICK tr|F1NDY4|F1NDY4\_CHICK tr|F1NGV7|F1NGV7\_CHICK tr|E1C7M9|E1C7M9\_CHICK tr|R4GF70|R4GF70\_CHICK tr|Q5ZMN5|Q5ZMN5\_CHICK tr|J9QXK8|J9QXK8\_9SAUR tr|F1P388|F1P388\_CHICK tr|F1NNN4|F1NNN4\_CHICK tr|C0IN11|C0IN11\_CHICK tr|E1BT05|E1BT05\_CHICK tr|H9L3V2|H9L3V2\_CHICK tr|R4GL23|R4GL23\_CHICK tr|F1NCB3|F1NCB3\_CHICK sp|Q5ZMV7|WDR82\_CHICK Q6IFU5 tr|F1NRX2|F1NRX2\_CHICK tr|F1NE90|F1NE90\_CHICK tr|E1BTX2|E1BTX2\_CHICK tr|Q5ZHQ9|Q5ZHQ9\_CHICK tr|F1P5E8|F1P5E8\_CHICK tr|Q9I8J4|Q9I8J4\_CHICK tr|F1P4W6|F1P4W6\_CHICK tr|E1C982|E1C982\_CHICK tr|F1NWX6|F1NWX6\_CHICK tr|E1BW50|E1BW50\_CHICK tr|Q5GL29|Q5GL29\_CHICK tr|Q5ZIQ7|Q5ZIQ7\_CHICK tr|Q5ZMV4|Q5ZMV4\_CHICK tr|F1NCB5|F1NCB5\_CHICK tr|F1CN12|F1CN12\_CHICK tr|Q5ZJF9|Q5ZJF9\_CHICK tr|A0A089FKX6|A0A089FKX6\_CHICK tr|A0A089FGZ7|A0A089FGZ7\_CHICK tr|A0A089FJ51|A0A089FJ51\_CHICK tr|E1BQ84|E1BQ84\_CHICK tr|O13265|O13265\_CHICK sp|Q01841|TGM2\_CHICK tr|F1ND60|F1ND60\_CHICK tr|H9KZN0|H9KZN0\_CHICK tr|A0A023J5T3|A0A023J5T3\_CHICK tr|E1C423|E1C423\_CHICK tr|Q9PTF1|Q9PTF1\_CHICK tr|F1NSL1|F1NSL1\_CHICK tr|F1N876|F1N876\_CHICK tr|R4GIW4|R4GIW4\_CHICK sp|P50593|GPC1\_CHICK tr|F1NDV4|F1NDV4\_CHICK sp|P07530|CRBB1\_CHICK tr|E1BQH2|E1BQH2\_CHICK tr|F1NUY1|F1NUY1\_CHICK tr|H9L2E1|H9L2E1\_CHICK tr|E1BT37|E1BT37\_CHICK tr|Q7ZZX8|Q7ZZX8\_CHICK tr|F1NGF2|F1NGF2\_CHICK sp|Q5ZLE6|EIF3H\_CHICK tr|Q2UXM6|Q2UXM6\_CHICK tr|F1NHE2|F1NHE2\_CHICK tr|R4GK85|R4GK85\_CHICK tr|E1C0F2|E1C0F2\_CHICK tr|F1NSI6|F1NSI6\_CHICK tr|H9KZD5|H9KZD5\_CHICK tr|E1BQI1|E1BQI1\_CHICK tr|E1BRF9|E1BRF9\_CHICK tr|F1NZ35|F1NZ35\_CHICK tr|F1NW94|F1NW94\_CHICK tr|Q8JID3|Q8JID3\_CHICK tr|E1C3T6|E1C3T6\_CHICK tr|F1P5B6|F1P5B6\_CHICK sp|Q7T2U9|TFCP2\_CHICK tr|A7VJA9|A7VJA9\_CHICK tr|R4GHQ7|R4GHQ7\_CHICK tr|R4GJH6|R4GJH6\_CHICK tr|E1C488|E1C488\_CHICK sp|Q6JHU7|P3H2\_CHICK tr|F1N912|F1N912\_CHICK tr|E1C5Z6|E1C5Z6\_CHICK tr|F1NJD7|F1NJD7\_CHICK tr|F1NXB0|F1NXB0\_CHICK tr|E1C1L9|E1C1L9\_CHICK tr|E1BV91|E1BV91\_CHICK tr|E1BWF3|E1BWF3\_CHICK tr|F1NTT1|F1NTT1\_CHICK tr|F1NFX7|F1NFX7\_CHICK sp|O12972|RFNG\_CHICK tr|Q5ZLZ0|Q5ZLZ0\_CHICK tr|F1NC23|F1NC23\_CHICK tr|E1BR67|E1BR67\_CHICK sp|P13585|AT2A1\_CHICK tr|F1P332|F1P332\_CHICK tr|E1C4U8|E1C4U8\_CHICK tr|F1NSL7|F1NSL7\_CHICK tr|Q5F3M7|Q5F3M7\_CHICK tr|F1NVC8|F1NVC8\_CHICK tr|F1NPE4|F1NPE4\_CHICK tr|F1NY89|F1NY89\_CHICK tr|F1P2M1|F1P2M1\_CHICK tr|Q5ZKF3|Q5ZKF3\_CHICK tr|F1P5J5|F1P5J5\_CHICK tr|E1C8Z1|E1C8Z1\_CHICK sp|Q3YK19|FANCJ\_CHICK tr|Q5ZIP1|Q5ZIP1\_CHICK tr|F1P2B1|F1P2B1\_CHICK tr|F1N8Y9|F1N8Y9\_CHICK tr|E1BTV2|E1BTV2\_CHICK tr|F1NEK3|F1NEK3\_CHICK tr|Q334G8|Q334G8\_CHICK tr|F1NBZ7|F1NBZ7\_CHICK tr|F1NRE1|F1NRE1\_CHICK tr|F1NK20|F1NK20\_CHICK tr|F1NFJ9|F1NFJ9\_CHICK tr|F1NT97|F1NT97\_CHICK sp|P12108|CO9A2\_CHICK tr|Q5ZIC9|Q5ZIC9\_CHICK tr|F1NYH8|F1NYH8\_CHICK tr|E1C2S8|E1C2S8\_CHICK tr|E1BTD5|E1BTD5\_CHICK tr|E1BVC4|E1BVC4\_CHICK tr|F1NJF8|F1NJF8\_CHICK sp|Q90744|NAGAB\_CHICK tr|Q71SF8|Q71SF8\_CHICK tr|F1NS48|F1NS48\_CHICK tr|R4GJ62|R4GJ62\_CHICK tr|E1C7Z0|E1C7Z0\_CHICK tr|E1C7S3|E1C7S3\_CHICK tr|R4GIA6|R4GIA6\_CHICK tr|F1NTI1|F1NTI1\_CHICK tr|R4GK20|R4GK20\_CHICK tr|E1C0Y0|E1C0Y0\_CHICK tr|E1C1K5|E1C1K5\_CHICK tr|H9L018|H9L018\_CHICK tr|R9PXN6|R9PXN6\_CHICK sp|O42163|COCH\_CHICK tr|R4GI04|R4GI04\_CHICK tr|E1BW54|E1BW54\_CHICK tr|G1EL50|G1EL50\_CHICK tr|F1P0L5|F1P0L5\_CHICK tr|F1NKT0|F1NKT0\_CHICK sp|Q7T2H2|FGRL1\_CHICK tr|Q8AYN2|Q8AYN2\_CHICK tr|Q9DG28|Q9DG28\_CHICK tr|F2Z4M5|F2Z4M5\_CHICK sp|P02272|H2AV\_CHICK tr|F1NCX5|F1NCX5\_CHICK tr|F1NEZ4|F1NEZ4\_CHICK tr|F1NV37|F1NV37\_CHICK tr|K9JV25|K9JV25\_CHICK tr|Q6ZLS4|Q6ZLS4\_CHICK tr|F1P4T7|F1P4T7\_CHICK tr|Q90874|Q90874\_CHICK tr|E5DFH8|E5DFH8\_CHICK tr|F1NAU1|F1NAU1\_CHICK tr|Q91357|Q91357\_CHICK tr|R4GGD2|R4GGD2\_CHICK tr|Q8UVZ3|Q8UVZ3\_CHICK tr|F1NTW7|F1NTW7\_CHICK tr|F1NBN3|F1NBN3\_CHICK tr|H9L0T0|H9L0T0\_CHICK tr|F1N9F2|F1N9F2\_CHICK tr|F1NGZ0|F1NGZ0\_CHICK tr|Q5ZIJ1|Q5ZIJ1\_CHICK tr|F1NEZ7|F1NEZ7\_CHICK tr|Q5ZKI3|Q5ZKI3\_CHICK tr|E1C8R3|E1C8R3\_CHICK sp|Q92179|CHST3\_CHICK tr|B5BSD3|B5BSD3\_CHICK tr|E6N1X1|E6N1X1\_CHICK tr|F1P568|F1P568\_CHICK tr|F1NJ38|F1NJ38\_CHICK tr|F1NSQ2|F1NSQ2\_CHICK tr|Q90589|Q90589\_CHICK tr|E1C8F2|E1C8F2\_CHICK tr|E1BZW9|E1BZW9\_CHICK tr|Q8QH01|Q8QH01\_CHICK tr|F1NZ71|F1NZ71\_CHICK tr|F1NSH3|F1NSH3\_CHICK tr|F1NKD5|F1NKD5\_CHICK sp|Q5ZKH6|HDAC9\_CHICK tr|E5DEI6|E5DEI6\_CHICK tr|F1NGJ7|F1NGJ7\_CHICK tr|Q5ZMM4|Q5ZMM4\_CHICK tr|Q5F366|Q5F366\_CHICK tr|E1BXK5|E1BXK5\_CHICK tr|H9KZL7|H9KZL7\_CHICK tr|E0XAY7|E0XAY7\_CHICK tr|E1BUJ5|E1BUJ5\_CHICK tr|F1NVG3|F1NVG3\_CHICK tr|F1NAG8|F1NAG8\_CHICK sp|P51509|RELB\_CHICK tr|E1C8Q7|E1C8Q7\_CHICK tr|R9PXP0|R9PXP0\_CHICK tr|F1N9T9|F1N9T9\_CHICK tr|F1NEY5|F1NEY5\_CHICK tr|R4GHR6|R4GHR6\_CHICK tr|E1BQK8|E1BQK8\_CHICK tr|F1P1E9|F1P1E9\_CHICK tr|Q8SPC9|Q8SPC9\_CHICK tr|E1BY95|E1BY95\_CHICK tr|F1NMD6|F1NMD6\_CHICK tr|E1BQC0|E1BQC0\_CHICK tr|L7NSX8|L7NSX8\_CHICK tr|F1NAY2|F1NAY2\_CHICK tr|Q1PS52|Q1PS52\_CHICK tr|F1NE60|F1NE60\_CHICK tr|R4GFI2|R4GFI2\_CHICK tr|M4M8W5|M4M8W5\_CHICK sp|P19121|ALBU\_CHICK sp|Q04584|ZYX\_CHICK sp|Q5ZLN0|LRC40\_CHICK tr|F1NAT8|F1NAT8\_CHICK tr|Q5ZL15|Q5ZL15\_CHICK sp|Q9PWR1|KCAB1\_CHICK tr|Q5ZLB5|Q5ZLB5\_CHICK sp|Q5ZJ66|SYEM\_CHICK tr|F1NYM1|F1NYM1\_CHICK tr|Q5F3I4|Q5F3I4\_CHICK Q6NTB9 tr|F1NGU0|F1NGU0\_CHICK tr|Q5ZJ82|Q5ZJ82\_CHICK tr|H9KZV9|H9KZV9\_CHICK tr|F1NSP4|F1NSP4\_CHICK tr|Q6Y2V8|Q6Y2V8\_CHICK tr|C6KHP3|C6KHP3\_CHICK tr|A0A023J5T4|A0A023J5T4\_CHICK tr|Q76KT5|Q76KT5\_CHICK tr|F1NXF2|F1NXF2\_CHICK tr|Q5ZM54|Q5ZM54\_CHICK sp|P53760|LCAT\_CHICK tr|F1NAQ8|F1NAQ8\_CHICK tr|R4GIY4|R4GIY4\_CHICK tr|E1BSY3|E1BSY3\_CHICK tr|F1NLM8|F1NLM8\_CHICK tr|E1C541|E1C541\_CHICK tr|E1C5A8|E1C5A8\_CHICK tr|R4GJV9|R4GJV9\_CHICK tr|F1NAZ1|F1NAZ1\_CHICK tr|F1NW97|F1NW97\_CHICK sp|P53412|LHX3\_CHICK tr|E1C2Q1|E1C2Q1\_CHICK tr|E1BYJ4|E1BYJ4\_CHICK tr|F1NH95|F1NH95\_CHICK tr|E1BZ70|E1BZ70\_CHICK tr|F1NB68|F1NB68\_CHICK tr|E1BWX6|E1BWX6\_CHICK tr|E1BVA4|E1BVA4\_CHICK tr|F1NH73|F1NH73\_CHICK sp|Q5ZMS3|IF2G\_CHICK tr|F1NPF0|F1NPF0\_CHICK tr|I0J171|I0J171\_CHICK tr|R4GFT6|R4GFT6\_CHICK tr|F1NNP0|F1NNP0\_CHICK tr|R4GKU7|R4GKU7\_CHICK tr|F1P5N5|F1P5N5\_CHICK tr|F1NC12|F1NC12\_CHICK sp|P19439|GLRK\_CHICK tr|Q5ZM85|Q5ZM85\_CHICK tr|F1NXB6|F1NXB6\_CHICK tr|F1NTE7|F1NTE7\_CHICK sp|Q5ZIG3|S2546\_CHICK tr|F1NTP6|F1NTP6\_CHICK tr|E1BVP9|E1BVP9\_CHICK tr|Q5ZKZ5|Q5ZKZ5\_CHICK tr|E1C877|E1C877\_CHICK tr|H9KZA0|H9KZA0\_CHICK tr|E1BXA4|E1BXA4\_CHICK tr|E1C4K5|E1C4K5\_CHICK tr|F1P3F1|F1P3F1\_CHICK tr|R9PXM2|R9PXM2\_CHICK tr|E1C0J7|E1C0J7\_CHICK tr|Q5ZLD1|Q5ZLD1\_CHICK sp|O93366|TLX1\_CHICK sp|Q5ZIU8|KTNB1\_CHICK tr|F1P1H2|F1P1H2\_CHICK tr|F1NUH0|F1NUH0\_CHICK tr|E1C452|E1C452\_CHICK tr|Q5F3S5|Q5F3S5\_CHICK tr|F1N8W3|F1N8W3\_CHICK tr|E1BQJ3|E1BQJ3\_CHICK tr|F1P0S4|F1P0S4\_CHICK tr|F1NVQ6|F1NVQ6\_CHICK sp|F1NHE9|KCJ12\_CHICK tr|F1NLZ2|F1NLZ2\_CHICK tr|H9L1L8|H9L1L8\_CHICK tr|Q6Y2W4|Q6Y2W4\_CHICK tr|Q6Y2W0|Q6Y2W0\_CHICK tr|Q6Y2W3|Q6Y2W3\_CHICK sp|P68139|ACTS\_CHICK tr|F1P3U5|F1P3U5\_CHICK sp|Q98SH3|ADRM1\_CHICK tr|Q90WR6|Q90WR6\_CHICK tr|E1BS64|E1BS64\_CHICK tr|F1N9D9|F1N9D9\_CHICK tr|R4GJW4|R4GJW4\_CHICK tr|R4GH27|R4GH27\_CHICK tr|E1BWA8|E1BWA8\_CHICK tr|E1C893|E1C893\_CHICK tr|R4GJ65|R4GJ65\_CHICK tr|R4GL84|R4GL84\_CHICK tr|H9L2J4|H9L2J4\_CHICK tr|F1NJL5|F1NJL5\_CHICK tr|F1NZ39|F1NZ39\_CHICK tr|Q5ZJX6|Q5ZJX6\_CHICK tr|Q2PBA1|Q2PBA1\_CHICK tr|E1BR41|E1BR41\_CHICK sp|Q5ZL18|P20D1\_CHICK tr|F1N9I7|F1N9I7\_CHICK tr|E1BQB2|E1BQB2\_CHICK tr|E1C411|E1C411\_CHICK sp|Q5ZMD2|ANKY2\_CHICK tr|F1NM13|F1NM13\_CHICK tr|F1P5N7|F1P5N7\_CHICK tr|E1C373|E1C373\_CHICK tr|H9KZG0|H9KZG0\_CHICK tr|R4GL16|R4GL16\_CHICK tr|F1NKF3|F1NKF3\_CHICK sp|O93510|GELS\_CHICK tr|F1NV71|F1NV71\_CHICK tr|Q5ZJT9|Q5ZJT9\_CHICK tr|F1NP56|F1NP56\_CHICK tr|F1NL49|F1NL49\_CHICK tr|Q8JFP5|Q8JFP5\_CHICK tr|E1C250|E1C250\_CHICK tr|E1C4U9|E1C4U9\_CHICK tr|Q5ZLX9|Q5ZLX9\_CHICK tr|H9L366|H9L366\_CHICK tr|Q5ZL82|Q5ZL82\_CHICK tr|E1C1X6|E1C1X6\_CHICK tr|F1NZ97|F1NZ97\_CHICK tr|R4GGJ2|R4GGJ2\_CHICK tr|Q5F453|Q5F453\_CHICK tr|F1NPD2|F1NPD2\_CHICK O43790 sp|P54755|EPHA5\_CHICK tr|F1NSE6|F1NSE6\_CHICK tr|F1NKI4|F1NKI4\_CHICK tr|F1NH07|F1NH07\_CHICK sp|Q5ZJA2|CPIN1\_CHICK tr|F1P0W1|F1P0W1\_CHICK tr|F1N918|F1N918\_CHICK tr|Q5ZMI7|Q5ZMI7\_CHICK tr|R4GLK3|R4GLK3\_CHICK tr|Q5ZM89|Q5ZM89\_CHICK tr|A0A0A0MQ65|A0A0A0MQ65\_CHICK sp|Q03669|AT2A2\_CHICK tr|R4GHY9|R4GHY9\_CHICK tr|F1NI76|F1NI76\_CHICK tr|F1P1E8|F1P1E8\_CHICK tr|B2BP17|B2BP17\_CHICK tr|F1N9F4|F1N9F4\_CHICK tr|E1BWG7|E1BWG7\_CHICK tr|E1BV22|E1BV22\_CHICK tr|F1NHL9|F1NHL9\_CHICK tr|F1P3Y7|F1P3Y7\_CHICK tr|Q2XP49|Q2XP49\_CHICK tr|E1BYN7|E1BYN7\_CHICK tr|Q7T1F5|Q7T1F5\_CHICK tr|Q6X3Z0|Q6X3Z0\_CHICK tr|F1NM36|F1NM36\_CHICK tr|F1NIS9|F1NIS9\_CHICK tr|F1NID1|F1NID1\_CHICK tr|F1NRL4|F1NRL4\_CHICK sp|Q5ZJV9|CNOT7\_CHICK tr|R4GHU3|R4GHU3\_CHICK tr|E1C045|E1C045\_CHICK tr|F1NEH8|F1NEH8\_CHICK tr|R4GKI0|R4GKI0\_CHICK tr|E1BWV0|E1BWV0\_CHICK sp|P56733|AVR3\_CHICK tr|F6QGI8|F6QGI8\_CHICK tr|R4GLP8|R4GLP8\_CHICK tr|E1BXS3|E1BXS3\_CHICK tr|R4GG17|R4GG17\_CHICK tr|E1BSG6|E1BSG6\_CHICK tr|E1C6V9|E1C6V9\_CHICK tr|F1P4U8|F1P4U8\_CHICK tr|F1NBT5|F1NBT5\_CHICK tr|Q5F388|Q5F388\_CHICK tr|E1C161|E1C161\_CHICK tr|R4GL49|R4GL49\_CHICK tr|F1NCM5|F1NCM5\_CHICK tr|E1BR22|E1BR22\_CHICK tr|F1NU32|F1NU32\_CHICK tr|F1P0A4|F1P0A4\_CHICK tr|Q5ZLB7|Q5ZLB7\_CHICK tr|Q6XLP9|Q6XLP9\_CHICK tr|E1C6K9|E1C6K9\_CHICK tr|F1NQI6|F1NQI6\_CHICK tr|Q5ZI62|Q5ZI62\_CHICK tr|F1N8I3|F1N8I3\_CHICK tr|E1BX72|E1BX72\_CHICK tr|R9PXL7|R9PXL7\_CHICK tr|R4GJN0|R4GJN0\_CHICK tr|R4GMI6|R4GMI6\_CHICK tr|E1BZF6|E1BZF6\_CHICK tr|Q98SR2|Q98SR2\_CHICK tr|J9QW19|J9QW19\_9SAUR tr|J9QRM7|J9QRM7\_9SAUR tr|J9R0F3|J9R0F3\_9SAUR sp|Q5ZIV7|DESI2\_CHICK tr|F1NG41|F1NG41\_CHICK tr|Q9W7C6|Q9W7C6\_CHICK tr|E1BT61|E1BT61\_CHICK tr|F1P3X5|F1P3X5\_CHICK tr|Q5FY72|Q5FY72\_CHICK tr|H9KYX1|H9KYX1\_CHICK tr|F1P5M3|F1P5M3\_CHICK tr|F1P1F5|F1P1F5\_CHICK sp|Q5ZKX1|CHM1B\_CHICK tr|Q91964|Q91964\_CHICK tr|F1P3V3|F1P3V3\_CHICK tr|F1NDZ0|F1NDZ0\_CHICK sp|Q5ZL57|COPD\_CHICK tr|R9PXM7|R9PXM7\_CHICK tr|Q90930|Q90930\_CHICK tr|F1P2Q5|F1P2Q5\_CHICK tr|F1P1R5|F1P1R5\_CHICK tr|F1P5F4|F1P5F4\_CHICK tr|H9KYP9|H9KYP9\_CHICK tr|Q90978|Q90978\_CHICK tr|J7FPX4|J7FPX4\_CHICK sp|P11602|LIPL\_CHICK tr|F1NYP4|F1NYP4\_CHICK tr|A8TG80|A8TG80\_CHICK tr|F1P1P7|F1P1P7\_CHICK tr|Q5ZM47|Q5ZM47\_CHICK sp|P48463|PP2AA\_CHICK tr|Q5ZMB1|Q5ZMB1\_CHICK tr|Q5ZIC4|Q5ZIC4\_CHICK tr|F1N8Z4|F1N8Z4\_CHICK tr|D8WN45|D8WN45\_CHICK tr|F1NAN0|F1NAN0\_CHICK tr|Q8QH60|Q8QH60\_CHICK tr|R4GG02|R4GG02\_CHICK P06868 tr|F1NWB2|F1NWB2\_CHICK tr|E1BW76|E1BW76\_CHICK sp|Q5ZKV4|NUBP2\_CHICK tr|E1C4P9|E1C4P9\_CHICK tr|F1NQ62|F1NQ62\_CHICK tr|Q7T1A0|Q7T1A0\_CHICK sp|Q9PWH2|FZD10\_CHICK tr|E1C5Z5|E1C5Z5\_CHICK tr|F1NFC8|F1NFC8\_CHICK tr|F1P201|F1P201\_CHICK tr|E1BTK1|E1BTK1\_CHICK tr|F1NG40|F1NG40\_CHICK tr|F1NFH1|F1NFH1\_CHICK tr|E1BVC8|E1BVC8\_CHICK tr|Q5ZKP9|Q5ZKP9\_CHICK tr|E1C8I2|E1C8I2\_CHICK sp|O42273|TENP\_CHICK tr|Q9I881|Q9I881\_CHICK tr|E1C8E6|E1C8E6\_CHICK tr|F1NZ82|F1NZ82\_CHICK tr|F1P3E2|F1P3E2\_CHICK tr|F1P0D9|F1P0D9\_CHICK tr|C6ZL13|C6ZL13\_9EUCA tr|F1NW62|F1NW62\_CHICK tr|F1N899|F1N899\_CHICK tr|E1C8Z7|E1C8Z7\_CHICK tr|F1NVN9|F1NVN9\_CHICK tr|R4GIQ4|R4GIQ4\_CHICK tr|F1NB77|F1NB77\_CHICK tr|F1NR26|F1NR26\_CHICK tr|R4GG40|R4GG40\_CHICK tr|F1P455|F1P455\_CHICK tr|F1NYQ6|F1NYQ6\_CHICK tr|O57389|O57389\_CHICK tr|E1C8I4|E1C8I4\_CHICK tr|Q9W7R0|Q9W7R0\_CHICK tr|E1C3A0|E1C3A0\_CHICK tr|F1P0G3|F1P0G3\_CHICK tr|E1C2J0|E1C2J0\_CHICK tr|E1C8M4|E1C8M4\_CHICK tr|D3X745|D3X745\_CHICK sp|Q8AXP2|CCD80\_CHICK tr|F1NSN5|F1NSN5\_CHICK tr|E1BSR8|E1BSR8\_CHICK tr|F1NYX8|F1NYX8\_CHICK tr|R4GIV9|R4GIV9\_CHICK tr|E1C4Z2|E1C4Z2\_CHICK sp|Q5F3N5|KLH14\_CHICK tr|F1NHX4|F1NHX4\_CHICK tr|E1BWY2|E1BWY2\_CHICK tr|H6SV45|H6SV45\_CHICK tr|F1NPQ0|F1NPQ0\_CHICK tr|Q5ZI37|Q5ZI37\_CHICK tr|F1NLJ1|F1NLJ1\_CHICK tr|F1NDD8|F1NDD8\_CHICK tr|F1NKI1|F1NKI1\_CHICK tr|Q6JHU5|Q6JHU5\_CHICK tr|E1C7V3|E1C7V3\_CHICK tr|F1NXN9|F1NXN9\_CHICK tr|Q5ZJG0|Q5ZJG0\_CHICK tr|R4GI14|R4GI14\_CHICK tr|E7EC82|E7EC82\_CHICK tr|F1P3R2|F1P3R2\_CHICK tr|F1NVG9|F1NVG9\_CHICK tr|E1C3R9|E1C3R9\_CHICK tr|F1NA09|F1NA09\_CHICK tr|Q4ADG4|Q4ADG4\_CHICK tr|Q5ZIY2|Q5ZIY2\_CHICK tr|Q7SYH9|Q7SYH9\_CHICK tr|F1NLK8|F1NLK8\_CHICK tr|F1NGH1|F1NGH1\_CHICK tr|F1NJ21|F1NJ21\_CHICK tr|F1NUY7|F1NUY7\_CHICK sp|P84407|FETA\_CHICK tr|F1P442|F1P442\_CHICK tr|Q91969|Q91969\_CHICK tr|E1BVI4|E1BVI4\_CHICK tr|F1P3M5|F1P3M5\_CHICK sp|Q5ZIE6|ULA1\_CHICK tr|F1NT05|F1NT05\_CHICK sp|P02701|AVID\_CHICK tr|F1NAR8|F1NAR8\_CHICK tr|F1NVB8|F1NVB8\_CHICK tr|R4GF90|R4GF90\_CHICK tr|F1NWI3|F1NWI3\_CHICK tr|Q5ZIF4|Q5ZIF4\_CHICK tr|F1P3D9|F1P3D9\_CHICK ENSEMBL:ENSBTAP00000023402 tr|Q6Q4G2|Q6Q4G2\_CHICK tr|F1NVM9|F1NVM9\_CHICK tr|O42253|O42253\_CHICK tr|F1P3A0|F1P3A0\_CHICK tr|F1NBC1|F1NBC1\_CHICK tr|H9L067|H9L067\_CHICK tr|F1NG01|F1NG01\_CHICK tr|A9UGZ3|A9UGZ3\_CHICK tr|Q5F327|Q5F327\_CHICK tr|E1C7E7|E1C7E7\_CHICK tr|F1NW03|F1NW03\_CHICK sp|P09479|ACHA\_CHICK tr|F1NWG6|F1NWG6\_CHICK tr|F1P579|F1P579\_CHICK tr|Q5ZK39|Q5ZK39\_CHICK tr|F1NLP8|F1NLP8\_CHICK tr|F1NEQ4|F1NEQ4\_CHICK sp|Q5ZMT7|ADCK1\_CHICK tr|F1NNJ1|F1NNJ1\_CHICK tr|G8H1M4|G8H1M4\_CHICK tr|F1NXW5|F1NXW5\_CHICK tr|E1C492|E1C492\_CHICK tr|F1NFL4|F1NFL4\_CHICK tr|F1NEW0|F1NEW0\_CHICK tr|Q5F3E6|Q5F3E6\_CHICK tr|O73685|O73685\_CHICK tr|F1NNB5|F1NNB5\_CHICK tr|E1C6R5|E1C6R5\_CHICK sp|Q07598|NLTP\_CHICK tr|F1P2U8|F1P2U8\_CHICK tr|R4GLQ8|R4GLQ8\_CHICK tr|Q5ZLB0|Q5ZLB0\_CHICK tr|E1C9B8|E1C9B8\_CHICK tr|E1BYN1|E1BYN1\_CHICK tr|F1NI46|F1NI46\_CHICK tr|F1NMM0|F1NMM0\_CHICK tr|F1NTV0|F1NTV0\_CHICK tr|F1NIT0|F1NIT0\_CHICK tr|R4GM57|R4GM57\_CHICK tr|E1BQN6|E1BQN6\_CHICK tr|E1C6T2|E1C6T2\_CHICK O76011 tr|R4GHA9|R4GHA9\_CHICK tr|F1NN69|F1NN69\_CHICK tr|E1C8V5|E1C8V5\_CHICK tr|G3G8J1|G3G8J1\_CHICK tr|E1BQP7|E1BQP7\_CHICK tr|F1NQL6|F1NQL6\_CHICK tr|Q6IEC5|Q6IEC5\_CHICK tr|F1P559|F1P559\_CHICK tr|E1BRJ0|E1BRJ0\_CHICK tr|E1BT46|E1BT46\_CHICK tr|F1NHE4|F1NHE4\_CHICK sp|Q5ZLA5|EIF3E\_CHICK tr|I0J1E3|I0J1E3\_CHICK tr|Q5ZLE5|Q5ZLE5\_CHICK tr|E1BUK1|E1BUK1\_CHICK tr|E1BU96|E1BU96\_CHICK tr|Q7T1E2|Q7T1E2\_CHICK tr|E1BT35|E1BT35\_CHICK tr|F1NNU1|F1NNU1\_CHICK tr|Q2PGY2|Q2PGY2\_CHICK tr|F1NXZ8|F1NXZ8\_CHICK tr|Q5ZK65|Q5ZK65\_CHICK tr|E1BWW3|E1BWW3\_CHICK tr|E1C6T4|E1C6T4\_CHICK tr|E1C299|E1C299\_CHICK tr|Q5ZKE1|Q5ZKE1\_CHICK tr|R4GJZ2|R4GJZ2\_CHICK tr|F1NXX9|F1NXX9\_CHICK tr|F1NC34|F1NC34\_CHICK tr|E1BT81|E1BT81\_CHICK tr|Q5ZMK4|Q5ZMK4\_CHICK tr|H9L1E0|H9L1E0\_CHICK tr|F1NBQ3|F1NBQ3\_CHICK tr|Q5F3E3|Q5F3E3\_CHICK tr|F1NTE0|F1NTE0\_CHICK tr|F1NH87|F1NH87\_CHICK sp|P00356|G3P\_CHICK tr|Q5ZMS5|Q5ZMS5\_CHICK tr|Q9PTS9|Q9PTS9\_CHICK tr|E1C3U8|E1C3U8\_CHICK tr|F1NLU4|F1NLU4\_CHICK tr|F1P2G4|F1P2G4\_CHICK tr|F1N9Z2|F1N9Z2\_CHICK tr|F1P5K9|F1P5K9\_CHICK sp|P79772|FOXD3\_CHICK tr|Q5ZKS9|Q5ZKS9\_CHICK tr|F1NC05|F1NC05\_CHICK tr|E1BW31|E1BW31\_CHICK tr|F1N851|F1N851\_CHICK tr|F1NPK5|F1NPK5\_CHICK sp|Q5ZKD1|CEPT1\_CHICK tr|F1NC85|F1NC85\_CHICK tr|F1NWS9|F1NWS9\_CHICK tr|F1NQJ0|F1NQJ0\_CHICK tr|Q5F446|Q5F446\_CHICK tr|Q5ZJ53|Q5ZJ53\_CHICK tr|Q9IAI6|Q9IAI6\_CHICK tr|F6QFC1|F6QFC1\_CHICK tr|E1BYI3|E1BYI3\_CHICK sp|P79996|MK09\_CHICK tr|F1NVH3|F1NVH3\_CHICK sp|Q9DEB6|CDX1\_CHICK tr|F1NVK5|F1NVK5\_CHICK tr|E1C2G5|E1C2G5\_CHICK tr|Q5TLZ8|Q5TLZ8\_CHICK tr|F1CN55|F1CN55\_CHICK tr|Q5ZKN9|Q5ZKN9\_CHICK sp|P49892|AA1R\_CHICK tr|E1BU39|E1BU39\_CHICK tr|Q5ZKW1|Q5ZKW1\_CHICK tr|E1C881|E1C881\_CHICK tr|F1NVK9|F1NVK9\_CHICK tr|R4GJQ7|R4GJQ7\_CHICK tr|G1K342|G1K342\_CHICK tr|F1NMY9|F1NMY9\_CHICK tr|A4Q912|A4Q912\_CHICK tr|E1C771|E1C771\_CHICK tr|C4N9M9|C4N9M9\_CHICK tr|Q9PU44|Q9PU44\_CHICK tr|F1NT18|F1NT18\_CHICK sp|Q5ZIU9|S39A9\_CHICK tr|R4GHX5|R4GHX5\_CHICK tr|F1N9F6|F1N9F6\_CHICK sp|Q5ZL72|CH60\_CHICK tr|E1C4I1|E1C4I1\_CHICK tr|F1NCN3|F1NCN3\_CHICK tr|R4GIK8|R4GIK8\_CHICK tr|E1C5T0|E1C5T0\_CHICK tr|G4WW42|G4WW42\_CHICK tr|G9F995|G9F995\_9SAUR tr|G9F994|G9F994\_9SAUR tr|O93440|O93440\_CHICK tr|R4GHB5|R4GHB5\_CHICK tr|E1BT36|E1BT36\_CHICK tr|Q76MF7|Q76MF7\_CHICK tr|F1N9R8|F1N9R8\_CHICK tr|F1NGI6|F1NGI6\_CHICK tr|E1BZ00|E1BZ00\_CHICK tr|F1P0P3|F1P0P3\_CHICK sp|P55166|PAX9\_CHICK tr|S4U9V0|S4U9V0\_CHICK tr|H9KZH2|H9KZH2\_CHICK tr|F1NT88|F1NT88\_CHICK tr|F1NEV4|F1NEV4\_CHICK tr|E1C534|E1C534\_CHICK tr|Q58IU6|Q58IU6\_CHICK tr|D3GGX2|D3GGX2\_CHICK tr|F1NGH8|F1NGH8\_CHICK tr|F1NM54|F1NM54\_CHICK tr|H2D5E7|H2D5E7\_CHICK tr|F1P5C0|F1P5C0\_CHICK tr|Q98947|Q98947\_CHICK tr|F1NC68|F1NC68\_CHICK tr|Q5F332|Q5F332\_CHICK tr|Q1EQ66|Q1EQ66\_CHICK tr|F1NHF8|F1NHF8\_CHICK tr|F1P531|F1P531\_CHICK tr|E1C254|E1C254\_CHICK tr|Q07718|Q07718\_CHICK tr|F1NQ29|F1NQ29\_CHICK tr|F1P4Q4|F1P4Q4\_CHICK tr|F1NCB2|F1NCB2\_CHICK tr|F1NM25|F1NM25\_CHICK tr|E1BRY8|E1BRY8\_CHICK tr|F1NC52|F1NC52\_CHICK tr|F1NG47|F1NG47\_CHICK sp|Q9W719|HPRT\_CHICK tr|F1NBZ6|F1NBZ6\_CHICK tr|C4PC51|C4PC51\_CHICK tr|C4PC31|C4PC31\_GALSO tr|C4PC36|C4PC36\_CHICK tr|C4PC13|C4PC13\_CHICK tr|C4PC30|C4PC30\_CHICK tr|C4PC09|C4PC09\_GALLA tr|C4PC32|C4PC32\_GALSO tr|R4GLL8|R4GLL8\_CHICK tr|F1NH70|F1NH70\_CHICK tr|F1NVI8|F1NVI8\_CHICK sp|O42236|SEM3C\_CHICK tr|E1BWX1|E1BWX1\_CHICK tr|M4M663|M4M663\_CHICK sp|Q98938|IHH\_CHICK tr|N0DM22|N0DM22\_CHICK tr|N0DLX8|N0DLX8\_CHICK sp|P0C7A1|ENASE\_CHICK tr|E1BVK1|E1BVK1\_CHICK tr|Q5ZK70|Q5ZK70\_CHICK tr|F1NZ81|F1NZ81\_CHICK tr|R4GFG1|R4GFG1\_CHICK sp|P09652|TBB4\_CHICK sp|Q5ZHX9|DPH1\_CHICK tr|Q9YHY8|Q9YHY8\_CHICK tr|E1C5F0|E1C5F0\_CHICK tr|F1NQI4|F1NQI4\_CHICK tr|R4GHE2|R4GHE2\_CHICK tr|R4GGF6|R4GGF6\_CHICK tr|Q5ZI61|Q5ZI61\_CHICK tr|Q5ZHP2|Q5ZHP2\_CHICK tr|Q9PSD4|Q9PSD4\_CHICK tr|C4PA58|C4PA58\_GALLA tr|C4PA80|C4PA80\_GALVA tr|C4PA82|C4PA82\_GALSO tr|Q5W4U0|Q5W4U0\_CHICK tr|C4PA62|C4PA62\_CHICK tr|Q90723|Q90723\_CHICK tr|N0DM15|N0DM15\_CHICK tr|E1C332|E1C332\_CHICK tr|I3RLD7|I3RLD7\_CHICK tr|E1C7J9|E1C7J9\_CHICK tr|Q5ZJN3|Q5ZJN3\_CHICK tr|Q7T2Z6|Q7T2Z6\_CHICK tr|E1BZE0|E1BZE0\_CHICK tr|Q5ZLW1|Q5ZLW1\_CHICK tr|F1NIB1|F1NIB1\_CHICK tr|R4GFN7|R4GFN7\_CHICK tr|F1P540|F1P540\_CHICK tr|A1EA95|A1EA95\_CHICK sp|Q5ZLS2|EDC3\_CHICK tr|Q8UWJ9|Q8UWJ9\_CHICK tr|Q8UWK0|Q8UWK0\_CHICK tr|F1NLU6|F1NLU6\_CHICK tr|F1P5R7|F1P5R7\_CHICK tr|F1P5R8|F1P5R8\_CHICK tr|F1P4C5|F1P4C5\_CHICK tr|E1BXY4|E1BXY4\_CHICK tr|R4GGJ7|R4GGJ7\_CHICK tr|F1P2K5|F1P2K5\_CHICK sp|Q5ZLG9|WDR59\_CHICK tr|E1C9B1|E1C9B1\_CHICK tr|E1C9I8|E1C9I8\_CHICK tr|F1NJH1|F1NJH1\_CHICK tr|Q5ZK75|Q5ZK75\_CHICK tr|R4GF73|R4GF73\_CHICK tr|E1BY42|E1BY42\_CHICK tr|B3Y9I3|B3Y9I3\_CHICK sp|Q5F4C4|SHOC2\_CHICK tr|F1P0A9|F1P0A9\_CHICK tr|F1NIK3|F1NIK3\_CHICK tr|S5TQ07|S5TQ07\_CHICK tr|F1NZH5|F1NZH5\_CHICK tr|F1NTJ6|F1NTJ6\_CHICK tr|F1NLA8|F1NLA8\_CHICK tr|F5ANJ3|F5ANJ3\_CHICK tr|Q9PTH1|Q9PTH1\_CHICK tr|F1NT56|F1NT56\_CHICK tr|Q9YHC0|Q9YHC0\_CHICK tr|Q8UUX5|Q8UUX5\_CHICK tr|F1NBN1|F1NBN1\_CHICK tr|E1C7D0|E1C7D0\_CHICK tr|Q684L7|Q684L7\_CHICK sp|Q5ZHT1|ACD11\_CHICK tr|R4GMC6|R4GMC6\_CHICK tr|B4ZEM2|B4ZEM2\_CHICK sp|P25433|NTF3\_CHICK tr|D1LWV2|D1LWV2\_CHICK tr|F1NVF4|F1NVF4\_CHICK tr|E1C552|E1C552\_CHICK tr|D1LWV1|D1LWV1\_CHICK tr|Q06BS3|Q06BS3\_CHICK sp|O42220|GDF8\_CHICK tr|F1NLQ0|F1NLQ0\_CHICK sp|Q5ZKZ9|LMF2\_CHICK tr|F1NN25|F1NN25\_CHICK tr|H9KZ53|H9KZ53\_CHICK tr|E1BSM2|E1BSM2\_CHICK tr|E1BXM5|E1BXM5\_CHICK tr|I0J172|I0J172\_CHICK tr|I0J170|I0J170\_CHICK tr|F1NIS0|F1NIS0\_CHICK tr|I0J173|I0J173\_CHICK tr|E1C9F2|E1C9F2\_CHICK tr|Q6PKI8|Q6PKI8\_CHICK tr|R4GL68|R4GL68\_CHICK tr|O42419|O42419\_CHICK tr|Q90XD9|Q90XD9\_CHICK tr|F1NSP1|F1NSP1\_CHICK tr|F1N9W6|F1N9W6\_CHICK tr|F1NZJ7|F1NZJ7\_CHICK sp|Q5ZJL7|DDB2\_CHICK tr|F1NF09|F1NF09\_CHICK sp|Q90678|PAFA\_CHICK tr|R4GKU3|R4GKU3\_CHICK sp|Q90812|CRFR1\_CHICK tr|E1BRV6|E1BRV6\_CHICK tr|F1NRR7|F1NRR7\_CHICK tr|E1BUP6|E1BUP6\_CHICK sp|D3KCC4|CRNS1\_CHICK tr|Q5ZLT6|Q5ZLT6\_CHICK tr|Q90661|Q90661\_CHICK tr|F1P4E7|F1P4E7\_CHICK tr|E1C6A9|E1C6A9\_CHICK tr|R4GJ17|R4GJ17\_CHICK tr|R4GHB8|R4GHB8\_CHICK sp|Q9DG67|RA54B\_CHICK tr|F7B5W6|F7B5W6\_CHICK tr|R4GFZ0|R4GFZ0\_CHICK tr|F1P3D3|F1P3D3\_CHICK tr|F1NRF5|F1NRF5\_CHICK tr|F1P342|F1P342\_CHICK tr|Q5ZJ68|Q5ZJ68\_CHICK tr|Q6XFR0|Q6XFR0\_CHICK tr|F1NSJ5|F1NSJ5\_CHICK tr|Q6XFQ9|Q6XFQ9\_CHICK tr|Q5ZL65|Q5ZL65\_CHICK tr|R4GIF7|R4GIF7\_CHICK tr|Q6XFR1|Q6XFR1\_CHICK tr|E1C4B9|E1C4B9\_CHICK sp|Q9I8D8|CD40L\_CHICK tr|B6RCP9|B6RCP9\_CHICK sp|P42558|RAN\_CHICK tr|R4GGP1|R4GGP1\_CHICK tr|F1NDS1|F1NDS1\_CHICK tr|Q5ZKY7|Q5ZKY7\_CHICK tr|E1C7W3|E1C7W3\_CHICK tr|R4QXY5|R4QXY5\_CHICK tr|Q800W3|Q800W3\_CHICK tr|E1C9I9|E1C9I9\_CHICK Q6KB66-1 tr|F1NJJ7|F1NJJ7\_CHICK tr|A3QW62|A3QW62\_CHICK tr|Q90621|Q90621\_CHICK tr|F1NU16|F1NU16\_CHICK tr|E1BZ29|E1BZ29\_CHICK tr|Q5ZIB4|Q5ZIB4\_CHICK tr|Q9DFH3|Q9DFH3\_CHICK tr|F1NQH0|F1NQH0\_CHICK tr|Q5ZIU7|Q5ZIU7\_CHICK tr|F1NNG6|F1NNG6\_CHICK tr|F1P5R4|F1P5R4\_CHICK tr|F1NTQ9|F1NTQ9\_CHICK tr|R4GJV3|R4GJV3\_CHICK tr|R4GLV0|R4GLV0\_CHICK tr|Q5F3R6|Q5F3R6\_CHICK tr|F1NWH6|F1NWH6\_CHICK tr|A7VMS0|A7VMS0\_CHICK tr|R4GJQ5|R4GJQ5\_CHICK tr|E1BZ22|E1BZ22\_CHICK tr|A9YDV6|A9YDV6\_CHICK tr|Q4JQQ0|Q4JQQ0\_CHICK tr|F1NVM8|F1NVM8\_CHICK tr|E1C0I3|E1C0I3\_CHICK tr|E1C1V7|E1C1V7\_CHICK tr|F1P355|F1P355\_CHICK tr|F1NS60|F1NS60\_CHICK tr|R4GGN1|R4GGN1\_CHICK tr|E1C5R1|E1C5R1\_CHICK tr|F1NJ67|F1NJ67\_CHICK tr|E6Y8U5|E6Y8U5\_CHICK tr|F1NYY7|F1NYY7\_CHICK tr|F1P1Y2|F1P1Y2\_CHICK sp|Q5F3A6|OTU1\_CHICK tr|H9L0B9|H9L0B9\_CHICK tr|F1NYD0|F1NYD0\_CHICK tr|E1C400|E1C400\_CHICK tr|Q8SPC7|Q8SPC7\_CHICK tr|R4GMD8|R4GMD8\_CHICK tr|E1C3Z4|E1C3Z4\_CHICK tr|R4GII0|R4GII0\_CHICK tr|F1NGV6|F1NGV6\_CHICK tr|D5M8S3|D5M8S3\_CHICK tr|R4GHV0|R4GHV0\_CHICK tr|E1C5L3|E1C5L3\_CHICK tr|F1NY42|F1NY42\_CHICK tr|E1BY44|E1BY44\_CHICK tr|Q5ZIF2|Q5ZIF2\_CHICK tr|E1C974|E1C974\_CHICK P35900 sp|P09482|ACHA4\_CHICK tr|E1BQX8|E1BQX8\_CHICK tr|E1BQF2|E1BQF2\_CHICK tr|A3QW67|A3QW67\_CHICK tr|H9L3R9|H9L3R9\_CHICK tr|H9KZP7|H9KZP7\_CHICK tr|A5GZA7|A5GZA7\_CHICK tr|E1BSN3|E1BSN3\_CHICK tr|Q90813|Q90813\_CHICK tr|R4GHV7|R4GHV7\_CHICK tr|F6RII4|F6RII4\_CHICK tr|E1BRP1|E1BRP1\_CHICK tr|Q98T82|Q98T82\_CHICK tr|E1C0K7|E1C0K7\_CHICK tr|F1P4X5|F1P4X5\_CHICK tr|F1NGP6|F1NGP6\_CHICK tr|F1NNL9|F1NNL9\_CHICK tr|Q5ZLQ7|Q5ZLQ7\_CHICK tr|F1P5J8|F1P5J8\_CHICK tr|F1NSY1|F1NSY1\_CHICK tr|Q5ZMU2|Q5ZMU2\_CHICK tr|F1CN00|F1CN00\_CHICK tr|F1CMZ7|F1CMZ7\_CHICK tr|Q5ZMF1|Q5ZMF1\_CHICK tr|E1BZJ3|E1BZJ3\_CHICK tr|F1P4S5|F1P4S5\_CHICK tr|Q5W4T3|Q5W4T3\_CHICK tr|F1P4X7|F1P4X7\_CHICK tr|Q5ZKA8|Q5ZKA8\_CHICK tr|E1C5D8|E1C5D8\_CHICK tr|E1C5N4|E1C5N4\_CHICK ENSEMBL:ENSBTAP00000023055 tr|F1NIB6|F1NIB6\_CHICK tr|E1BWX3|E1BWX3\_CHICK tr|R4GGH1|R4GGH1\_CHICK tr|F1NYB3|F1NYB3\_CHICK tr|E1C9D7|E1C9D7\_CHICK sp|Q5ZJU5|NUSAP\_CHICK tr|F1NWZ7|F1NWZ7\_CHICK tr|R4GFK0|R4GFK0\_CHICK tr|Q9DE15|Q9DE15\_CHICK tr|E1BV28|E1BV28\_CHICK tr|R4GIX6|R4GIX6\_CHICK tr|R4GHF8|R4GHF8\_CHICK tr|Q5ZLD9|Q5ZLD9\_CHICK tr|R4GJZ7|R4GJZ7\_CHICK tr|Q27J91|Q27J91\_CHICK sp|P27043|ANGI\_CHICK tr|R4GHC3|R4GHC3\_CHICK tr|F1NM65|F1NM65\_CHICK sp|P84247|H33\_CHICK tr|R4GGI8|R4GGI8\_CHICK tr|Q92068|Q92068\_CHICK tr|A0A0C4ZMI6|A0A0C4ZMI6\_9NEOP sp|P84229|H32\_CHICK tr|F4YTP7|F4YTP7\_9BIVA tr|K9MGV4|K9MGV4\_9BIVA tr|C6ZL36|C6ZL36\_9EUCA tr|Q06SQ5|Q06SQ5\_9CAEN tr|Q9PW10|Q9PW10\_CHICK tr|R4GII9|R4GII9\_CHICK tr|R4GK83|R4GK83\_CHICK tr|E1C1P8|E1C1P8\_CHICK tr|Q9PU54|Q9PU54\_CHICK tr|Q9DER1|Q9DER1\_CHICK tr|Q9I9V4|Q9I9V4\_CHICK tr|E1C6V2|E1C6V2\_CHICK tr|C4PBR8|C4PBR8\_CHICK tr|F1NR43|F1NR43\_CHICK tr|F1NLK1|F1NLK1\_CHICK tr|E1BZE1|E1BZE1\_CHICK tr|E1C9B4|E1C9B4\_CHICK tr|R4GIY2|R4GIY2\_CHICK tr|F1N9H4|F1N9H4\_CHICK sp|Q5F3B1|MTG16\_CHICK tr|Q5ZHN8|Q5ZHN8\_CHICK tr|F1NGC4|F1NGC4\_CHICK tr|F1NGN4|F1NGN4\_CHICK tr|E1C281|E1C281\_CHICK tr|F1NJT8|F1NJT8\_CHICK tr|F1NHL5|F1NHL5\_CHICK tr|F1NLH0|F1NLH0\_CHICK tr|F1N8P6|F1N8P6\_CHICK tr|Q9PW22|Q9PW22\_CHICK tr|Q5F3M5|Q5F3M5\_CHICK tr|R4GG72|R4GG72\_CHICK tr|R4GLR9|R4GLR9\_CHICK tr|E1C4P2|E1C4P2\_CHICK sp|Q5F3V0|SMYD4\_CHICK tr|F1P137|F1P137\_CHICK tr|R4GFV9|R4GFV9\_CHICK sp|Q5ZJ81|SHLB2\_CHICK tr|B3GS88|B3GS88\_CHICK tr|B3GS85|B3GS85\_CHICK tr|Q9DGI1|Q9DGI1\_CHICK tr|E1C7Q8|E1C7Q8\_CHICK tr|F1NAF4|F1NAF4\_CHICK tr|E1C0E0|E1C0E0\_CHICK tr|F1NQ86|F1NQ86\_CHICK tr|Q8AYE5|Q8AYE5\_CHICK sp|Q5ZI22|NUPL2\_CHICK tr|F1NT60|F1NT60\_CHICK sp|P37072|OLF6\_CHICK tr|F1NI14|F1NI14\_CHICK tr|R4GHF1|R4GHF1\_CHICK tr|R4GFT8|R4GFT8\_CHICK tr|F1P001|F1P001\_CHICK tr|R4GG98|R4GG98\_CHICK tr|R4GLW3|R4GLW3\_CHICK tr|E1C6B2|E1C6B2\_CHICK tr|Q03852|Q03852\_CHICK sp|Q8AXY6|MUSK\_CHICK tr|F1NZW7|F1NZW7\_CHICK tr|R4GLW0|R4GLW0\_CHICK tr|Q45KQ2|Q45KQ2\_CHICK tr|R4GK42|R4GK42\_CHICK tr|Q5ZLU8|Q5ZLU8\_CHICK tr|Q6B842|Q6B842\_CHICK tr|E1C3D9|E1C3D9\_CHICK tr|F1NYZ2|F1NYZ2\_CHICK tr|H9L3I7|H9L3I7\_CHICK tr|Q6QDA0|Q6QDA0\_CHICK tr|E1BTZ2|E1BTZ2\_CHICK tr|E1C3Y1|E1C3Y1\_CHICK tr|Q5ZHS2|Q5ZHS2\_CHICK tr|Q2KNE6|Q2KNE6\_CHICK tr|F1NUP9|F1NUP9\_CHICK tr|Q90ZN2|Q90ZN2\_CHICK tr|F1NPD7|F1NPD7\_CHICK sp|Q5ZK69|PAAF1\_CHICK tr|E1BR23|E1BR23\_CHICK tr|Q5F421|Q5F421\_CHICK tr|F1NW06|F1NW06\_CHICK tr|F1NN71|F1NN71\_CHICK tr|Q9DDC9|Q9DDC9\_CHICK tr|F1NE72|F1NE72\_CHICK tr|R4GM39|R4GM39\_CHICK tr|Q5ZJP4|Q5ZJP4\_CHICK tr|E1BQE9|E1BQE9\_CHICK tr|R4GK94|R4GK94\_CHICK sp|Q5ZJH6|ULK3\_CHICK tr|F1P059|F1P059\_CHICK tr|I3QHS6|I3QHS6\_CHICK tr|A3F965|A3F965\_CHICK tr|F1NES9|F1NES9\_CHICK tr|Q5ZMU4|Q5ZMU4\_CHICK tr|F1NAF6|F1NAF6\_CHICK tr|Q6Q1Q9|Q6Q1Q9\_CHICK tr|F1NL80|F1NL80\_CHICK tr|Q90984|Q90984\_CHICK sp|Q5ZMP6|AP2M1\_CHICK tr|F1NS44|F1NS44\_CHICK tr|E1BSL6|E1BSL6\_CHICK sp|Q5ZMC3|WSDU1\_CHICK tr|A0A0A0MQ50|A0A0A0MQ50\_CHICK tr|E1C3V4|E1C3V4\_CHICK tr|F1NBT2|F1NBT2\_CHICK tr|F1P393|F1P393\_CHICK tr|E1C9A6|E1C9A6\_CHICK tr|F1P360|F1P360\_CHICK tr|E1BWU1|E1BWU1\_CHICK tr|E1BWY8|E1BWY8\_CHICK tr|E1C6K2|E1C6K2\_CHICK tr|R4GKN7|R4GKN7\_CHICK tr|F1NK65|F1NK65\_CHICK tr|F1NFI0|F1NFI0\_CHICK tr|E1BZJ9|E1BZJ9\_CHICK tr|F1NEA7|F1NEA7\_CHICK tr|R4GL75|R4GL75\_CHICK tr|F1NJS5|F1NJS5\_CHICK tr|R4GKP5|R4GKP5\_CHICK tr|F1NMB2|F1NMB2\_CHICK tr|E1C3T1|E1C3T1\_CHICK tr|E1BSS9|E1BSS9\_CHICK tr|F1NUK8|F1NUK8\_CHICK tr|Q5ZKW7|Q5ZKW7\_CHICK tr|E1C266|E1C266\_CHICK tr|A8CWP8|A8CWP8\_CHICK tr|F1P1D7|F1P1D7\_CHICK tr|R4GH01|R4GH01\_CHICK tr|R4GLY3|R4GLY3\_CHICK sp|Q5F4B3|TMLH\_CHICK tr|Q5F353|Q5F353\_CHICK tr|F1P2L9|F1P2L9\_CHICK tr|E1BQ82|E1BQ82\_CHICK sp|P52550|MYBA\_CHICK tr|P87373|P87373\_CHICK sp|P83038|HDAC4\_CHICK tr|F1NP26|F1NP26\_CHICK tr|E1BWR2|E1BWR2\_CHICK tr|F1NG13|F1NG13\_CHICK tr|F1NG10|F1NG10\_CHICK tr|Q5ZID3|Q5ZID3\_CHICK tr|E1C351|E1C351\_CHICK tr|A0A0A7MEX1|A0A0A7MEX1\_CHICK sp|Q65Z91|TSK\_CHICK tr|F1NDH7|F1NDH7\_CHICK tr|Q8QG94|Q8QG94\_CHICK tr|F1NSP8|F1NSP8\_CHICK tr|F1NPK6|F1NPK6\_CHICK tr|E1C5I9|E1C5I9\_CHICK tr|F1NWA5|F1NWA5\_CHICK tr|H2D5E6|H2D5E6\_CHICK tr|F1NB62|F1NB62\_CHICK tr|H2D5E5|H2D5E5\_CHICK tr|H2D5F1|H2D5F1\_CHICK tr|H2D5E8|H2D5E8\_CHICK tr|F1NDJ6|F1NDJ6\_CHICK tr|Q5ISI0|Q5ISI0\_CHICK tr|F1NUH5|F1NUH5\_CHICK tr|B5BSJ3|B5BSJ3\_CHICK tr|B5BSG5|B5BSG5\_CHICK tr|B5BSD7|B5BSD7\_CHICK tr|Q6Y2W2|Q6Y2W2\_CHICK tr|F1NV32|F1NV32\_CHICK tr|F1P1M3|F1P1M3\_CHICK tr|E1C6P0|E1C6P0\_CHICK tr|F1NBF9|F1NBF9\_CHICK tr|H9CZQ7|H9CZQ7\_CHICK tr|H9CZQ3|H9CZQ3\_CHICK tr|F1NZ08|F1NZ08\_CHICK tr|F1P1L5|F1P1L5\_CHICK tr|Q5F3D5|Q5F3D5\_CHICK sp|Q5ZJX0|LAAT1\_CHICK tr|F1N9A8|F1N9A8\_CHICK tr|R4GHR2|R4GHR2\_CHICK tr|E1C7G6|E1C7G6\_CHICK tr|R4GJ79|R4GJ79\_CHICK tr|F1NME2|F1NME2\_CHICK tr|F1N9E5|F1N9E5\_CHICK tr|E1BZV5|E1BZV5\_CHICK tr|F1NW76|F1NW76\_CHICK tr|F1NQN7|F1NQN7\_CHICK tr|R4GKA3|R4GKA3\_CHICK tr|E1BU84|E1BU84\_CHICK tr|E1BQU2|E1BQU2\_CHICK tr|Q5ZIT0|Q5ZIT0\_CHICK tr|Q401R7|Q401R7\_CHICK tr|R4GLV7|R4GLV7\_CHICK tr|F1NQ64|F1NQ64\_CHICK tr|R4GKV2|R4GKV2\_CHICK tr|E1C6D8|E1C6D8\_CHICK tr|Q5ZMC5|Q5ZMC5\_CHICK tr|E1C7P9|E1C7P9\_CHICK tr|R4GL73|R4GL73\_CHICK tr|R4GIK9|R4GIK9\_CHICK tr|F1N8B6|F1N8B6\_CHICK sp|Q5ZIH3|ZNT6\_CHICK sp|Q90615|ITA1\_CHICK tr|F1NK43|F1NK43\_CHICK tr|F1NVB5|F1NVB5\_CHICK tr|F1NGR7|F1NGR7\_CHICK sp|O42101|NR5A2\_CHICK tr|F2VP22|F2VP22\_CHICK tr|D9N195|D9N195\_CHICK tr|Q5ZLW7|Q5ZLW7\_CHICK tr|F1NHN4|F1NHN4\_CHICK tr|F1NEE3|F1NEE3\_CHICK tr|F1NIR8|F1NIR8\_CHICK sp|O42230|GBX2\_CHICK sp|P24899|TAL1\_CHICK tr|Q9I9C9|Q9I9C9\_CHICK tr|E1BTK6|E1BTK6\_CHICK tr|F1NSC0|F1NSC0\_CHICK tr|F1NYC7|F1NYC7\_CHICK tr|F1NQI0|F1NQI0\_CHICK tr|Q5ZJU6|Q5ZJU6\_CHICK tr|P79771|P79771\_CHICK tr|F1NGX5|F1NGX5\_CHICK tr|F1NVZ6|F1NVZ6\_CHICK tr|Q5ZHU4|Q5ZHU4\_CHICK tr|E1BX19|E1BX19\_CHICK tr|F1NQG0|F1NQG0\_CHICK Q2HJF0 tr|Q6EE30|Q6EE30\_CHICK tr|F1NPH6|F1NPH6\_CHICK tr|E1BUZ0|E1BUZ0\_CHICK tr|R4GKL6|R4GKL6\_CHICK tr|E1C833|E1C833\_CHICK tr|E1C348|E1C348\_CHICK tr|D0EL81|D0EL81\_CHICK tr|E1BYV5|E1BYV5\_CHICK tr|F1NT40|F1NT40\_CHICK tr|E1C5E7|E1C5E7\_CHICK tr|F1P419|F1P419\_CHICK tr|E1BYR3|E1BYR3\_CHICK tr|E1BUD0|E1BUD0\_CHICK O76009 tr|E1BWX0|E1BWX0\_CHICK tr|Q5ZM84|Q5ZM84\_CHICK tr|F1P5S0|F1P5S0\_CHICK sp|Q5ZLP2|MTNB\_CHICK tr|F1NNC9|F1NNC9\_CHICK tr|F1NDR9|F1NDR9\_CHICK tr|E1C7Y3|E1C7Y3\_CHICK tr|F1NIZ4|F1NIZ4\_CHICK sp|Q91035|SHH\_CHICK sp|P02713|ACHG\_CHICK sp|P04354|CALB1\_CHICK tr|F1NJ22|F1NJ22\_CHICK tr|B0LY48|B0LY48\_CHICK tr|E1BQY6|E1BQY6\_CHICK tr|F1NDC6|F1NDC6\_CHICK tr|E1C4U5|E1C4U5\_CHICK tr|E1BT96|E1BT96\_CHICK tr|F1P4R4|F1P4R4\_CHICK tr|E1C7A6|E1C7A6\_CHICK tr|R4GLP7|R4GLP7\_CHICK tr|R4RAM7|R4RAM7\_CHICK tr|R4R7H0|R4R7H0\_CHICK tr|Q03853|Q03853\_CHICK tr|Q5CZI6|Q5CZI6\_CHICK tr|F1NPQ8|F1NPQ8\_CHICK tr|F1NGS4|F1NGS4\_CHICK tr|V9H109|V9H109\_CHICK tr|F1NNN3|F1NNN3\_CHICK tr|Q3BKZ5|Q3BKZ5\_CHICK tr|F1NJB7|F1NJB7\_CHICK tr|F1N8Y7|F1N8Y7\_CHICK tr|F1NYI7|F1NYI7\_CHICK tr|F1NCN1|F1NCN1\_CHICK tr|F1NPR8|F1NPR8\_CHICK sp|P79784|ENTP2\_CHICK tr|F1N962|F1N962\_CHICK tr|F1CN18|F1CN18\_CHICK tr|F1CN04|F1CN04\_CHICK tr|F1CN29|F1CN29\_CHICK tr|F1CN16|F1CN16\_CHICK tr|F1CN02|F1CN02\_CHICK tr|R4GIG7|R4GIG7\_CHICK tr|E1BWB0|E1BWB0\_CHICK tr|J9Z1F6|J9Z1F6\_CHICK tr|H9L0G1|H9L0G1\_CHICK tr|H9L017|H9L017\_CHICK sp|P51027|NRAM1\_CHICK tr|F1NUS2|F1NUS2\_CHICK tr|Q7T197|Q7T197\_CHICK tr|E1BV57|E1BV57\_CHICK tr|F1NXL1|F1NXL1\_CHICK tr|F1NPB0|F1NPB0\_CHICK tr|F1NE93|F1NE93\_CHICK tr|F1NAN8|F1NAN8\_CHICK tr|E1C1D7|E1C1D7\_CHICK tr|E1C4I0|E1C4I0\_CHICK tr|F1NCA4|F1NCA4\_CHICK tr|R4GKS0|R4GKS0\_CHICK Q6NT21 tr|F1NB15|F1NB15\_CHICK tr|F1NS14|F1NS14\_CHICK tr|E1C0G0|E1C0G0\_CHICK tr|R4GIT6|R4GIT6\_CHICK tr|F1N9B8|F1N9B8\_CHICK tr|E1C3K9|E1C3K9\_CHICK tr|F1P482|F1P482\_CHICK tr|F1NDP9|F1NDP9\_CHICK tr|Q5F376|Q5F376\_CHICK tr|F1NS42|F1NS42\_CHICK tr|R4GGB1|R4GGB1\_CHICK tr|E1BWP3|E1BWP3\_CHICK tr|E1C6R0|E1C6R0\_CHICK tr|F1NQ93|F1NQ93\_CHICK tr|E1BSM9|E1BSM9\_CHICK tr|F1NX13|F1NX13\_CHICK sp|Q5QJC3|DCR1B\_CHICK tr|E1BQ54|E1BQ54\_CHICK tr|F1NFE6|F1NFE6\_CHICK tr|F1NE22|F1NE22\_CHICK tr|F1NNS8|F1NNS8\_CHICK tr|E1BV41|E1BV41\_CHICK tr|R4GK04|R4GK04\_CHICK tr|F1P4E3|F1P4E3\_CHICK tr|A9XRG9|A9XRG9\_CHICK tr|E1C7X0|E1C7X0\_CHICK tr|H9KZW0|H9KZW0\_CHICK tr|F1NTZ6|F1NTZ6\_CHICK tr|F1NEL4|F1NEL4\_CHICK tr|D5JGF9|D5JGF9\_CHICK tr|Q9PTS7|Q9PTS7\_CHICK tr|R4GIA4|R4GIA4\_CHICK tr|F1NWN8|F1NWN8\_CHICK tr|F1N9C5|F1N9C5\_CHICK tr|Q5ZMA7|Q5ZMA7\_CHICK sp|P50890|RSSA\_CHICK tr|E1BQK0|E1BQK0\_CHICK tr|F1NPE5|F1NPE5\_CHICK tr|E1C164|E1C164\_CHICK tr|E1AP05|E1AP05\_CHICK sp|Q2LMP1|WNT3A\_CHICK tr|E1C508|E1C508\_CHICK tr|F1NVA7|F1NVA7\_CHICK sp|P51641|CNTFR\_CHICK tr|F1NRU5|F1NRU5\_CHICK tr|E1BYL0|E1BYL0\_CHICK A2VCT4 tr|Q5ZIZ6|Q5ZIZ6\_CHICK tr|E1BQI7|E1BQI7\_CHICK tr|H9L008|H9L008\_CHICK tr|E1BQW9|E1BQW9\_CHICK tr|F1NBR1|F1NBR1\_CHICK tr|F1P2T9|F1P2T9\_CHICK tr|Q804X7|Q804X7\_CHICK tr|F1NJJ9|F1NJJ9\_CHICK tr|E1BWZ4|E1BWZ4\_CHICK tr|E1C7B5|E1C7B5\_CHICK sp|P38530|HSF2\_CHICK tr|F1P5T7|F1P5T7\_CHICK tr|E1BS44|E1BS44\_CHICK tr|E1BZM3|E1BZM3\_CHICK tr|R4GM47|R4GM47\_CHICK tr|R4GFB3|R4GFB3\_CHICK tr|E1BY87|E1BY87\_CHICK tr|F1NLG9|F1NLG9\_CHICK tr|F1P450|F1P450\_CHICK tr|F1NIY5|F1NIY5\_CHICK tr|Q6XCE1|Q6XCE1\_CHICK tr|Q9PUA4|Q9PUA4\_CHICK tr|E1C248|E1C248\_CHICK tr|E1BS13|E1BS13\_CHICK tr|F1P5W7|F1P5W7\_CHICK tr|E1BZY8|E1BZY8\_CHICK tr|H9L3S6|H9L3S6\_CHICK tr|Q92071|Q92071\_CHICK Q14532 tr|F1NYV3|F1NYV3\_CHICK sp|Q5ZLL8|GPAT3\_CHICK tr|Q5F3N4|Q5F3N4\_CHICK tr|F6VMG5|F6VMG5\_CHICK tr|E1BRW5|E1BRW5\_CHICK tr|Q8QFX0|Q8QFX0\_CHICK tr|F1P1J6|F1P1J6\_CHICK tr|Q38Q38|Q38Q38\_CHICK sp|P08641|CADH1\_CHICK tr|E1C6M9|E1C6M9\_CHICK tr|R4GIU9|R4GIU9\_CHICK sp|Q9PWE8|TBX5\_CHICK tr|F1NBA3|F1NBA3\_CHICK sp|Q7T2L7|I17RD\_CHICK tr|E1C0L8|E1C0L8\_CHICK sp|P86346|BORE1\_CHICK tr|Q5ZL37|Q5ZL37\_CHICK tr|F1N9V0|F1N9V0\_CHICK tr|E1BSR5|E1BSR5\_CHICK tr|R4GHU1|R4GHU1\_CHICK tr|F1NX61|F1NX61\_CHICK sp|Q5ZHV5|JMJD4\_CHICK tr|R4GJC6|R4GJC6\_CHICK tr|O93439|O93439\_CHICK tr|E1C667|E1C667\_CHICK sp|P46936|YAP1\_CHICK tr|R4GHE4|R4GHE4\_CHICK tr|R4GF51|R4GF51\_CHICK tr|E1BXS5|E1BXS5\_CHICK sp|D0PRN2|NRX1B\_CHICK tr|A4F5B1|A4F5B1\_CHICK tr|B5BSC7|B5BSC7\_CHICK tr|F1NMP3|F1NMP3\_CHICK sp|P08070|TBA2\_CHICK tr|R4GGM8|R4GGM8\_CHICK tr|E1C0P9|E1C0P9\_CHICK tr|Q5ZKH9|Q5ZKH9\_CHICK tr|R4GFK1|R4GFK1\_CHICK tr|R9PXL2|R9PXL2\_CHICK sp|E1C2V1|LMBL1\_CHICK sp|Q9I8K7|CITE3\_CHICK tr|Q5ZI95|Q5ZI95\_CHICK tr|E1BWG5|E1BWG5\_CHICK tr|Q6IFY8|Q6IFY8\_CHICK tr|R4GJU1|R4GJU1\_CHICK tr|F1NXW6|F1NXW6\_CHICK tr|E1BSQ5|E1BSQ5\_CHICK sp|Q5ZMH1|SEPT2\_CHICK sp|P19150|GBRA1\_CHICK tr|E1C484|E1C484\_CHICK tr|A7XMT4|A7XMT4\_CHICK tr|E1BQE0|E1BQE0\_CHICK tr|F1NH66|F1NH66\_CHICK tr|E1BR94|E1BR94\_CHICK tr|R4GIH8|R4GIH8\_CHICK tr|Q5ZLX3|Q5ZLX3\_CHICK tr|R4GMF0|R4GMF0\_CHICK tr|Q5F369|Q5F369\_CHICK tr|A1XGV6|A1XGV6\_CHICK tr|E1C5Y7|E1C5Y7\_CHICK tr|Q5ZI48|Q5ZI48\_CHICK tr|E1BZB2|E1BZB2\_CHICK tr|Q5F3Q8|Q5F3Q8\_CHICK tr|F1NQY7|F1NQY7\_CHICK tr|F1NMT9|F1NMT9\_CHICK tr|A0A023PSR8|A0A023PSR8\_CHICK tr|Q98923|Q98923\_CHICK tr|E1BVV9|E1BVV9\_CHICK tr|E1BY78|E1BY78\_CHICK tr|R4GFH1|R4GFH1\_CHICK tr|E1C128|E1C128\_CHICK tr|Q5QGZ1|Q5QGZ1\_CHICK sp|P27921|JUND\_CHICK tr|F1P4A5|F1P4A5\_CHICK tr|G4XJR9|G4XJR9\_CHICK sp|Q5ZM65|PTSS1\_CHICK tr|Q5F492|Q5F492\_CHICK tr|F1NSM0|F1NSM0\_CHICK Q9C075 tr|E1C5B5|E1C5B5\_CHICK tr|Q5ZJ42|Q5ZJ42\_CHICK tr|F1NWC7|F1NWC7\_CHICK ENSEMBL:ENSBTAP00000006074 tr|F1P3F0|F1P3F0\_CHICK tr|F1NYU5|F1NYU5\_CHICK tr|Q7ZZJ9|Q7ZZJ9\_CHICK tr|Q7ZZJ8|Q7ZZJ8\_CHICK tr|E7FKE5|E7FKE5\_CHICK sp|Q5ZJL4|CLP1\_CHICK tr|E1BS56|E1BS56\_CHICK tr|F1NFB9|F1NFB9\_CHICK tr|R4GG24|R4GG24\_CHICK tr|F1NRD4|F1NRD4\_CHICK tr|Q6QWE7|Q6QWE7\_CHICK sp|P50478|AMPH\_CHICK tr|F1P310|F1P310\_CHICK tr|E1C5X0|E1C5X0\_CHICK tr|F1NJQ4|F1NJQ4\_CHICK tr|E6N1X7|E6N1X7\_CHICK tr|A5HUM5|A5HUM5\_CHICK tr|F1NLC8|F1NLC8\_CHICK tr|E1C4C1|E1C4C1\_CHICK tr|E1BTF7|E1BTF7\_CHICK tr|F1NYN3|F1NYN3\_CHICK tr|Q5TIL8|Q5TIL8\_CHICK tr|R4GHK4|R4GHK4\_CHICK tr|R4GFX3|R4GFX3\_CHICK tr|F1NCJ9|F1NCJ9\_CHICK tr|F1NK31|F1NK31\_CHICK tr|F1NKX9|F1NKX9\_CHICK tr|F1NTA6|F1NTA6\_CHICK tr|Q4FAB3|Q4FAB3\_CHICK tr|E1BW34|E1BW34\_CHICK tr|F1P1A5|F1P1A5\_CHICK tr|Q90846|Q90846\_CHICK tr|R4GG83|R4GG83\_CHICK tr|F1NLN2|F1NLN2\_CHICK tr|F1P5B7|F1P5B7\_CHICK tr|A0A023PS12|A0A023PS12\_CHICK tr|A7UEA8|A7UEA8\_CHICK tr|R4GFW1|R4GFW1\_CHICK tr|Q90WU0|Q90WU0\_CHICK tr|R4GLK8|R4GLK8\_CHICK tr|F1N8T9|F1N8T9\_CHICK tr|Q9IBC9|Q9IBC9\_CHICK tr|R4GKB5|R4GKB5\_CHICK tr|E1BQM2|E1BQM2\_CHICK tr|F1N989|F1N989\_CHICK tr|E1C8E9|E1C8E9\_CHICK tr|F1NYC9|F1NYC9\_CHICK tr|Q8AWC0|Q8AWC0\_CHICK tr|A8TG88|A8TG88\_CHICK tr|Q8UWG7|Q8UWG7\_CHICK sp|P27463|AL1A1\_CHICK tr|E1BX68|E1BX68\_CHICK tr|F1NJC7|F1NJC7\_CHICK tr|Q802F9|Q802F9\_CHICK tr|E9NR85|E9NR85\_CHICK sp|P55167|MSHR\_CHICK tr|M9PNV4|M9PNV4\_CHICK tr|C4PBR2|C4PBR2\_GALSO tr|Q59I39|Q59I39\_CHICK tr|C4PBQ9|C4PBQ9\_GALSO tr|Q802F8|Q802F8\_CHICK tr|F1NWE9|F1NWE9\_CHICK tr|E1C172|E1C172\_CHICK sp|P17924|NFIB\_CHICK tr|F1NN08|F1NN08\_CHICK tr|Q5ZMA0|Q5ZMA0\_CHICK tr|E1BRM0|E1BRM0\_CHICK tr|R4GHU5|R4GHU5\_CHICK tr|E1C8P3|E1C8P3\_CHICK tr|E1C5P5|E1C5P5\_CHICK tr|E1C797|E1C797\_CHICK tr|E1C011|E1C011\_CHICK tr|R4GL51|R4GL51\_CHICK tr|R4GJ35|R4GJ35\_CHICK tr|D3UTA7|D3UTA7\_CHICK tr|F1ND55|F1ND55\_CHICK tr|F1P2U3|F1P2U3\_CHICK tr|F1N970|F1N970\_CHICK tr|R4GJW5|R4GJW5\_CHICK tr|E1BUR8|E1BUR8\_CHICK tr|F1ND89|F1ND89\_CHICK tr|F1N9Y0|F1N9Y0\_CHICK tr|Q2AB82|Q2AB82\_CHICK tr|R4GI03|R4GI03\_CHICK tr|Q860V9|Q860V9\_CHICK tr|A0ZY60|A0ZY60\_CHICK tr|H9L0Y7|H9L0Y7\_CHICK tr|Q5ZIN6|Q5ZIN6\_CHICK tr|F1NKQ2|F1NKQ2\_CHICK sp|Q5ZIK9|COPE\_CHICK ENSEMBL:ENSBTAP00000016285 tr|H9KYY1|H9KYY1\_CHICK sp|Q5ZIW5|VAC14\_CHICK tr|E1BQW7|E1BQW7\_CHICK tr|E1BQW4|E1BQW4\_CHICK tr|Q5ZIA6|Q5ZIA6\_CHICK tr|F1NYN1|F1NYN1\_CHICK tr|Q800D4|Q800D4\_CHICK tr|E1BRR2|E1BRR2\_CHICK tr|E1C0U7|E1C0U7\_CHICK tr|Q5ZHK5|Q5ZHK5\_CHICK tr|F1NF80|F1NF80\_CHICK tr|E1C803|E1C803\_CHICK tr|Q03481|Q03481\_CHICK tr|R4GM33|R4GM33\_CHICK tr|F1P0X1|F1P0X1\_CHICK tr|R4GHL4|R4GHL4\_CHICK tr|F1NT54|F1NT54\_CHICK tr|F1P0X0|F1P0X0\_CHICK tr|N0GTD5|N0GTD5\_9SAUR tr|F1NNL5|F1NNL5\_CHICK tr|Q5ZJ96|Q5ZJ96\_CHICK tr|R4GIX4|R4GIX4\_CHICK tr|Q8UUQ9|Q8UUQ9\_CHICK tr|F1NTB6|F1NTB6\_CHICK tr|A0A088RGT9|A0A088RGT9\_CHICK tr|F1NTJ1|F1NTJ1\_CHICK tr|R4GLQ4|R4GLQ4\_CHICK sp|P09531|TGFB1\_CHICK tr|F7BEK8|F7BEK8\_CHICK tr|F1NCM9|F1NCM9\_CHICK sp|Q5F3F2|MYSM1\_CHICK sp|Q5ZKU8|PK1IP\_CHICK tr|F1NYM3|F1NYM3\_CHICK tr|F1NWB8|F1NWB8\_CHICK tr|I3PL65|I3PL65\_CHICK tr|F1CN45|F1CN45\_CHICK tr|F1NDV9|F1NDV9\_CHICK tr|B5BSR2|B5BSR2\_CHICK tr|E1BWM1|E1BWM1\_CHICK tr|F1P104|F1P104\_CHICK tr|R4GFZ1|R4GFZ1\_CHICK tr|F1NDR0|F1NDR0\_CHICK tr|E1BY10|E1BY10\_CHICK sp|I0IUP3|MCM8\_CHICK tr|F1NDA6|F1NDA6\_CHICK tr|E1BTU5|E1BTU5\_CHICK tr|E1BX23|E1BX23\_CHICK tr|E1BRQ8|E1BRQ8\_CHICK sp|Q90577|SRCA\_CHICK sp|Q9PVY0|RX1\_CHICK tr|F1NC47|F1NC47\_CHICK tr|F1NF86|F1NF86\_CHICK tr|E1C553|E1C553\_CHICK tr|F1NUR1|F1NUR1\_CHICK tr|D2K8P9|D2K8P9\_CHICK tr|F1NI66|F1NI66\_CHICK tr|B2ZUA3|B2ZUA3\_CHICK tr|R4GJJ5|R4GJJ5\_CHICK tr|Q90700|Q90700\_CHICK tr|Q90816|Q90816\_CHICK sp|Q9W6D8|BARX1\_CHICK tr|F1P2L5|F1P2L5\_CHICK sp|Q5ZJD3|LSG1\_CHICK tr|R4GRV9|R4GRV9\_CHICK sp|Q5ZIL4|STRBP\_CHICK tr|R4GJZ4|R4GJZ4\_CHICK tr|R4GJR6|R4GJR6\_CHICK sp|O42237|SEM3E\_CHICK tr|F1P542|F1P542\_CHICK sp|Q5ZIY4|SBDS\_CHICK tr|R9PXL3|R9PXL3\_CHICK tr|A7E3N8|A7E3N8\_CHICK tr|Q5W919|Q5W919\_CHICK tr|Q5W920|Q5W920\_CHICK tr|E1BWX5|E1BWX5\_CHICK sp|P53413|LMX1B\_CHICK tr|F1NDZ5|F1NDZ5\_CHICK tr|F1NVY5|F1NVY5\_CHICK tr|I6L4M8|I6L4M8\_CHICK tr|Q5ZJA5|Q5ZJA5\_CHICK tr|F1NQJ5|F1NQJ5\_CHICK tr|Q7T1F2|Q7T1F2\_CHICK tr|I3RLD8|I3RLD8\_CHICK tr|Q1T768|Q1T768\_CHICK tr|R4GK08|R4GK08\_CHICK tr|H9L039|H9L039\_CHICK tr|E1BYT2|E1BYT2\_CHICK tr|E1C063|E1C063\_CHICK tr|F1NTH9|F1NTH9\_CHICK tr|F1NSJ9|F1NSJ9\_CHICK tr|Q7ZT14|Q7ZT14\_CHICK tr|E1C996|E1C996\_CHICK tr|R4GF80|R4GF80\_CHICK tr|E1C479|E1C479\_CHICK tr|F1NMM8|F1NMM8\_CHICK tr|F1P3J4|F1P3J4\_CHICK tr|Q3L585|Q3L585\_CHICK tr|R4GMH4|R4GMH4\_CHICK tr|F1NPD5|F1NPD5\_CHICK tr|F1P5H2|F1P5H2\_CHICK tr|A0A023H673|A0A023H673\_CHICK tr|Q2LAI0|Q2LAI0\_CHICK sp|Q701R0|SIAT2\_CHICK tr|E1BZW3|E1BZW3\_CHICK tr|F1P4Q1|F1P4Q1\_CHICK tr|F1NXG3|F1NXG3\_CHICK tr|Q5ZMG4|Q5ZMG4\_CHICK tr|E1BRU3|E1BRU3\_CHICK tr|F1N830|F1N830\_CHICK tr|F1NA29|F1NA29\_CHICK tr|E1BZC7|E1BZC7\_CHICK tr|F7BCR4|F7BCR4\_CHICK tr|E1C932|E1C932\_CHICK tr|E1C7Z2|E1C7Z2\_CHICK sp|P68306|THB\_CHICK tr|Q3L250|Q3L250\_CHICK tr|E1BVP0|E1BVP0\_CHICK tr|E1BUD6|E1BUD6\_CHICK tr|F1NPN3|F1NPN3\_CHICK tr|F1NCI5|F1NCI5\_CHICK tr|Q8UWH3|Q8UWH3\_CHICK tr|F1NYS8|F1NYS8\_CHICK tr|Q5ZLI5|Q5ZLI5\_CHICK tr|Q5F358|Q5F358\_CHICK tr|H9L0I0|H9L0I0\_CHICK tr|F1NLE1|F1NLE1\_CHICK tr|F1NRT0|F1NRT0\_CHICK tr|E1BWV4|E1BWV4\_CHICK tr|O57597|O57597\_CHICK tr|F1NKS1|F1NKS1\_CHICK tr|E1C820|E1C820\_CHICK tr|F1P5E3|F1P5E3\_CHICK tr|E1BVT3|E1BVT3\_CHICK tr|E1BZI0|E1BZI0\_CHICK tr|R4GK31|R4GK31\_CHICK tr|F1P210|F1P210\_CHICK sp|Q5ZIV1|MTMR2\_CHICK tr|E1BYY6|E1BYY6\_CHICK sp|P79763|FSHR\_CHICK tr|F1NGD5|F1NGD5\_CHICK tr|S4SCC1|S4SCC1\_CHICK tr|F1NHB7|F1NHB7\_CHICK tr|E1C2T4|E1C2T4\_CHICK tr|E1C2X6|E1C2X6\_CHICK sp|Q5ZHN3|WIPI2\_CHICK tr|F1NW43|F1NW43\_CHICK sp|P00548|KPYM\_CHICK tr|R4GFQ9|R4GFQ9\_CHICK sp|P37067|OLF1\_CHICK tr|R4GL36|R4GL36\_CHICK tr|E1C578|E1C578\_CHICK tr|F1N9V1|F1N9V1\_CHICK sp|Q1T7C1|CENPK\_CHICK tr|E1C305|E1C305\_CHICK tr|O12943|O12943\_CHICK tr|E1BVZ5|E1BVZ5\_CHICK tr|A1IHF9|A1IHF9\_CHICK tr|E1C7B4|E1C7B4\_CHICK tr|Q5ZK28|Q5ZK28\_CHICK tr|F1NRR6|F1NRR6\_CHICK tr|F1NSN1|F1NSN1\_CHICK sp|Q5ZM14|NHRF1\_CHICK tr|F1NPT0|F1NPT0\_CHICK tr|E1BXU1|E1BXU1\_CHICK tr|F1NQ79|F1NQ79\_CHICK tr|F1NL57|F1NL57\_CHICK sp|Q6IVA4|UBA5\_CHICK tr|F1NNM0|F1NNM0\_CHICK tr|E1C6K0|E1C6K0\_CHICK tr|F1NBC9|F1NBC9\_CHICK sp|Q9DEA3|PCNA\_CHICK tr|A7XMV1|A7XMV1\_CHICK tr|D4P5A2|D4P5A2\_CHICK tr|G9BWP6|G9BWP6\_CHICK tr|B8YIV7|B8YIV7\_GALVA tr|E1BTV1|E1BTV1\_CHICK tr|B8LFM3|B8LFM3\_CHICK sp|Q5ZKP4|TPPC2\_CHICK sp|Q90788|NKX25\_CHICK tr|R4GF58|R4GF58\_CHICK sp|O57476|CDC37\_CHICK tr|F1NNJ9|F1NNJ9\_CHICK tr|F1P5Q2|F1P5Q2\_CHICK tr|F1NSX1|F1NSX1\_CHICK tr|F1NDY5|F1NDY5\_CHICK tr|F1NWQ7|F1NWQ7\_CHICK Q05B55 tr|F1NHG4|F1NHG4\_CHICK tr|R4GLK6|R4GLK6\_CHICK tr|E1BZ21|E1BZ21\_CHICK tr|E1BQ63|E1BQ63\_CHICK tr|F1P3M0|F1P3M0\_CHICK tr|F1NMF1|F1NMF1\_CHICK sp|Q76I90|NUF2\_CHICK tr|F1NW34|F1NW34\_CHICK tr|R4GIM6|R4GIM6\_CHICK sp|F1NZP5|TLCD1\_CHICK tr|E1BYF2|E1BYF2\_CHICK tr|F1NWP6|F1NWP6\_CHICK tr|Q8AV07|Q8AV07\_CHICK tr|Q8AUP2|Q8AUP2\_CHICK tr|R4GLS4|R4GLS4\_CHICK tr|F1NT77|F1NT77\_CHICK Q9NSB4 tr|R4GJ19|R4GJ19\_CHICK tr|Q800E0|Q800E0\_CHICK tr|F1N8D6|F1N8D6\_CHICK tr|N0GS71|N0GS71\_9SAUR tr|Q4F8N1|Q4F8N1\_CHICK tr|E1BVI1|E1BVI1\_CHICK tr|E1C597|E1C597\_CHICK sp|Q05623|MYBPH\_CHICK tr|E1BVQ1|E1BVQ1\_CHICK sp|Q5NDE8|PMGT2\_CHICK tr|F1N9B0|F1N9B0\_CHICK tr|F1NI61|F1NI61\_CHICK tr|R4GJF6|R4GJF6\_CHICK tr|F1NC55|F1NC55\_CHICK tr|E1C143|E1C143\_CHICK tr|E1C702|E1C702\_CHICK tr|R9PXP8|R9PXP8\_CHICK sp|P38024|PUR6\_CHICK tr|A7UEA7|A7UEA7\_CHICK tr|F1NIK6|F1NIK6\_CHICK tr|F1NYY3|F1NYY3\_CHICK tr|F1NLR3|F1NLR3\_CHICK tr|R4GFP6|R4GFP6\_CHICK tr|Q5ZKN8|Q5ZKN8\_CHICK tr|E1C6X4|E1C6X4\_CHICK tr|H9L1C2|H9L1C2\_CHICK sp|P49147|PA24A\_CHICK tr|Q5ZJB1|Q5ZJB1\_CHICK tr|B0FMV6|B0FMV6\_CHICK tr|F1P3R7|F1P3R7\_CHICK tr|B8YL57|B8YL57\_CHICK tr|F1NV79|F1NV79\_CHICK tr|F1P120|F1P120\_CHICK tr|F1NIA3|F1NIA3\_CHICK sp|Q800L3|MED22\_CHICK tr|E1BRL4|E1BRL4\_CHICK tr|F1NFN4|F1NFN4\_CHICK tr|R4GI92|R4GI92\_CHICK tr|F1NXP6|F1NXP6\_CHICK tr|Q90WU1|Q90WU1\_CHICK sp|Q9IAK4|NOE1\_CHICK tr|Q5ZKY8|Q5ZKY8\_CHICK tr|H9KZJ3|H9KZJ3\_CHICK tr|Q5ZJ94|Q5ZJ94\_CHICK sp|Q5F3N0|SPX3\_CHICK tr|F1P5Q3|F1P5Q3\_CHICK tr|F1NCW6|F1NCW6\_CHICK tr|A8I1D0|A8I1D0\_CHICK tr|A8I1C6|A8I1C6\_CHICK tr|E1BQU7|E1BQU7\_CHICK tr|Q5ZM32|Q5ZM32\_CHICK tr|E1BTW1|E1BTW1\_CHICK tr|E1BUC0|E1BUC0\_CHICK tr|R4GI10|R4GI10\_CHICK tr|F1NB30|F1NB30\_CHICK tr|E1BW87|E1BW87\_CHICK tr|F1P5K0|F1P5K0\_CHICK tr|E1C573|E1C573\_CHICK tr|F1NIJ9|F1NIJ9\_CHICK tr|F1NL77|F1NL77\_CHICK tr|Q8UUU0|Q8UUU0\_CHICK tr|H9KYW8|H9KYW8\_CHICK tr|F1NX42|F1NX42\_CHICK tr|R4GKV8|R4GKV8\_CHICK sp|Q90837|ERG\_CHICK tr|C4PCP1|C4PCP1\_GALSO tr|C4PCM0|C4PCM0\_GALLA tr|A5JUC4|A5JUC4\_GALSO tr|C0A1C9|C0A1C9\_CHICK tr|Q9PWG2|Q9PWG2\_CHICK tr|E5DGK6|E5DGK6\_CHICK tr|R4GIU8|R4GIU8\_CHICK tr|C0A1D0|C0A1D0\_CHICK tr|C0A1C8|C0A1C8\_CHICK tr|B3F8C6|B3F8C6\_CHICK tr|F1N905|F1N905\_CHICK tr|E1BYX4|E1BYX4\_CHICK tr|F1NBI3|F1NBI3\_CHICK tr|F1NXK7|F1NXK7\_CHICK tr|F1NNE0|F1NNE0\_CHICK tr|A0A0A7MC86|A0A0A7MC86\_CHICK tr|R4GIK3|R4GIK3\_CHICK tr|R4GJ96|R4GJ96\_CHICK tr|R4GGQ8|R4GGQ8\_CHICK tr|F1P5V4|F1P5V4\_CHICK tr|E1C0A1|E1C0A1\_CHICK sp|Q5ZLN7|F122A\_CHICK tr|E1C1J2|E1C1J2\_CHICK tr|F1NJR6|F1NJR6\_CHICK tr|F1P2R1|F1P2R1\_CHICK sp|Q90WR8|SP3\_CHICK tr|F1NRT4|F1NRT4\_CHICK tr|G9IHC2|G9IHC2\_CHICK tr|E1BX56|E1BX56\_CHICK tr|F1NG62|F1NG62\_CHICK tr|Q5F3S1|Q5F3S1\_CHICK tr|E1C2U4|E1C2U4\_CHICK tr|R4GLG2|R4GLG2\_CHICK tr|F1NBD1|F1NBD1\_CHICK tr|Q5ZK08|Q5ZK08\_CHICK tr|Q9PSA3|Q9PSA3\_CHICK tr|B6RCP6|B6RCP6\_CHICK sp|Q5ZMS6|TDRD3\_CHICK tr|F1NAJ8|F1NAJ8\_CHICK tr|E1BXW9|E1BXW9\_CHICK tr|F1NLY3|F1NLY3\_CHICK tr|R4GKK4|R4GKK4\_CHICK tr|R4GLX1|R4GLX1\_CHICK tr|A0A089GLF7|A0A089GLF7\_CHICK tr|F1NSI8|F1NSI8\_CHICK tr|Q9W6S4|Q9W6S4\_CHICK tr|F1NL98|F1NL98\_CHICK tr|F6V5U7|F6V5U7\_CHICK tr|F1NZP0|F1NZP0\_CHICK sp|Q5ZM35|TWF2\_CHICK tr|F1NYQ0|F1NYQ0\_CHICK tr|E1C884|E1C884\_CHICK tr|M5AYC7|M5AYC7\_CHICK tr|F1NFZ1|F1NFZ1\_CHICK tr|F1NMM9|F1NMM9\_CHICK tr|Q91013|Q91013\_CHICK tr|F1NW26|F1NW26\_CHICK tr|F1P377|F1P377\_CHICK tr|H9L168|H9L168\_CHICK tr|Q58HZ9|Q58HZ9\_CHICK tr|Q5ZM99|Q5ZM99\_CHICK tr|F1P5L5|F1P5L5\_CHICK tr|F1NGV9|F1NGV9\_CHICK tr|F1N9R0|F1N9R0\_CHICK tr|Q5F3F3|Q5F3F3\_CHICK tr|F1NL70|F1NL70\_CHICK tr|Q5F456|Q5F456\_CHICK tr|F1CN51|F1CN51\_CHICK tr|R4GHT5|R4GHT5\_CHICK tr|Q98TX6|Q98TX6\_CHICK tr|Q98TX7|Q98TX7\_CHICK tr|E1BWI2|E1BWI2\_CHICK tr|F1P0B6|F1P0B6\_CHICK tr|F1NBV3|F1NBV3\_CHICK tr|E1BW93|E1BW93\_CHICK tr|Q9B620|Q9B620\_GALVA tr|E1BVB7|E1BVB7\_CHICK tr|E1C2Y8|E1C2Y8\_CHICK tr|F1NA00|F1NA00\_CHICK tr|Q5ZHM1|Q5ZHM1\_CHICK tr|F1NI18|F1NI18\_CHICK tr|F1NSZ4|F1NSZ4\_CHICK tr|R4GIP9|R4GIP9\_CHICK tr|R4GHA3|R4GHA3\_CHICK tr|E1BXU0|E1BXU0\_CHICK tr|E1C413|E1C413\_CHICK tr|E1C8A2|E1C8A2\_CHICK sp|Q5ZK88|PSPC1\_CHICK tr|F1NII4|F1NII4\_CHICK sp|Q98931|LRP8\_CHICK tr|Q8UVS5|Q8UVS5\_CHICK tr|F1P3V6|F1P3V6\_CHICK tr|E1C5B7|E1C5B7\_CHICK tr|F1NSS4|F1NSS4\_CHICK tr|F1P4K0|F1P4K0\_CHICK tr|Q5ZIZ9|Q5ZIZ9\_CHICK tr|E1C586|E1C586\_CHICK tr|R4GGV8|R4GGV8\_CHICK tr|H9L0R3|H9L0R3\_CHICK tr|R4GM24|R4GM24\_CHICK tr|E1C660|E1C660\_CHICK tr|F1NBM2|F1NBM2\_CHICK tr|I3XHQ9|I3XHQ9\_CHICK sp|P29332|CCNB2\_CHICK sp|Q05916|HME1\_CHICK tr|F1N8X7|F1N8X7\_CHICK tr|E1C0D8|E1C0D8\_CHICK tr|E1C7K9|E1C7K9\_CHICK tr|R4GGF0|R4GGF0\_CHICK tr|R4GG43|R4GG43\_CHICK tr|E1BZ49|E1BZ49\_CHICK tr|F1P492|F1P492\_CHICK tr|E1BVP4|E1BVP4\_CHICK tr|A0A0B4ZVH0|A0A0B4ZVH0\_CHICK tr|U3NEE3|U3NEE3\_CHICK tr|R4GLJ3|R4GLJ3\_CHICK tr|F1NRW3|F1NRW3\_CHICK sp|Q5ZMW3|API5\_CHICK tr|E1C056|E1C056\_CHICK tr|Q5F442|Q5F442\_CHICK tr|F1NZ21|F1NZ21\_CHICK sp|Q5ZII3|TPRA1\_CHICK tr|Q5ZKS0|Q5ZKS0\_CHICK tr|Q9DGH7|Q9DGH7\_CHICK tr|E1C4G0|E1C4G0\_CHICK tr|R4GGQ7|R4GGQ7\_CHICK tr|F1NA88|F1NA88\_CHICK tr|Q5ZK00|Q5ZK00\_CHICK tr|F1P4D5|F1P4D5\_CHICK tr|H9KZZ3|H9KZZ3\_CHICK tr|F6S9Q3|F6S9Q3\_CHICK tr|Q5F3I1|Q5F3I1\_CHICK tr|R4GM13|R4GM13\_CHICK tr|F1NND0|F1NND0\_CHICK tr|F1NJG7|F1NJG7\_CHICK sp|P23007|CISY\_CHICK tr|Q6T683|Q6T683\_CHICK tr|F1NEV2|F1NEV2\_CHICK tr|Q5ZKC2|Q5ZKC2\_CHICK tr|Q5F372|Q5F372\_CHICK tr|E1BTK0|E1BTK0\_CHICK sp|Q90835|EF1A\_CHICK tr|Q5ZKM2|Q5ZKM2\_CHICK tr|Q9PTF0|Q9PTF0\_CHICK tr|F5ANJ4|F5ANJ4\_CHICK tr|F1NLG4|F1NLG4\_CHICK tr|E1BRT6|E1BRT6\_CHICK tr|E1C7C6|E1C7C6\_CHICK tr|F1NRC2|F1NRC2\_CHICK tr|R4GIJ8|R4GIJ8\_CHICK tr|F1N9M6|F1N9M6\_CHICK tr|F1NSB8|F1NSB8\_CHICK tr|R4GI47|R4GI47\_CHICK sp|P30352|SRSF2\_CHICK tr|Q5F3I7|Q5F3I7\_CHICK tr|Q5ZMH2|Q5ZMH2\_CHICK tr|F1NFE2|F1NFE2\_CHICK tr|F1NH68|F1NH68\_CHICK tr|Q6B344|Q6B344\_CHICK tr|F1NXG6|F1NXG6\_CHICK tr|F1P501|F1P501\_CHICK tr|E1C851|E1C851\_CHICK tr|R4GFW5|R4GFW5\_CHICK tr|F1NVY1|F1NVY1\_CHICK tr|E1BR47|E1BR47\_CHICK tr|Q9YI58|Q9YI58\_CHICK tr|Q5ZKU6|Q5ZKU6\_CHICK tr|F8SZU0|F8SZU0\_9BIVA tr|Q5ZJ95|Q5ZJ95\_CHICK tr|F1NA82|F1NA82\_CHICK tr|E1BT22|E1BT22\_CHICK tr|F1NAF2|F1NAF2\_CHICK tr|F1CN38|F1CN38\_CHICK tr|R4GL17|R4GL17\_CHICK tr|F1P5S5|F1P5S5\_CHICK tr|E1C118|E1C118\_CHICK tr|B8YIR5|B8YIR5\_CHICK tr|R4GIF4|R4GIF4\_CHICK tr|R4GI02|R4GI02\_CHICK tr|E1C8S4|E1C8S4\_CHICK tr|E1C038|E1C038\_CHICK tr|F1NE11|F1NE11\_CHICK tr|F1P2F6|F1P2F6\_CHICK tr|R4GGU5|R4GGU5\_CHICK sp|Q5ZL38|SGF29\_CHICK tr|B8YLI9|B8YLI9\_CHICK tr|B8YKK6|B8YKK6\_CHICK tr|B8YK83|B8YK83\_CHICK tr|Q2YHU4|Q2YHU4\_CHICK tr|Q5F3G1|Q5F3G1\_CHICK tr|F1NTC8|F1NTC8\_CHICK tr|F1NQC5|F1NQC5\_CHICK tr|Q4GWL4|Q4GWL4\_GALVA tr|E1C016|E1C016\_CHICK tr|F1NVF3|F1NVF3\_CHICK sp|Q8AWW5|CRIM1\_CHICK tr|Q2HPN7|Q2HPN7\_CHICK tr|F1NJU6|F1NJU6\_CHICK sp|Q5F3Z5|DNJB6\_CHICK tr|E1C6Y7|E1C6Y7\_CHICK sp|Q5ZKM1|SPB1\_CHICK tr|R4GHW7|R4GHW7\_CHICK tr|Q9W6V7|Q9W6V7\_CHICK tr|Q3HR38|Q3HR38\_CHICK tr|Q6Q4G0|Q6Q4G0\_CHICK tr|F1P2I6|F1P2I6\_CHICK tr|Q6Q4G1|Q6Q4G1\_CHICK tr|F1NTL9|F1NTL9\_CHICK tr|F2Z4K7|F2Z4K7\_CHICK tr|F1NLJ9|F1NLJ9\_CHICK tr|R4GG29|R4GG29\_CHICK tr|E1C2K6|E1C2K6\_CHICK sp|P47191|SYT1\_CHICK tr|R4GJN8|R4GJN8\_CHICK tr|R4GIK6|R4GIK6\_CHICK tr|R4GJM9|R4GJM9\_CHICK tr|F1NQ76|F1NQ76\_CHICK tr|F1NS66|F1NS66\_CHICK tr|E1BTM5|E1BTM5\_CHICK tr|F1NY28|F1NY28\_CHICK tr|E1BX35|E1BX35\_CHICK tr|E1BQM3|E1BQM3\_CHICK tr|E1BSN0|E1BSN0\_CHICK tr|R4GIP0|R4GIP0\_CHICK tr|B3XZF4|B3XZF4\_CHICK tr|R4GHS0|R4GHS0\_CHICK tr|F1NU85|F1NU85\_CHICK tr|O57536|O57536\_CHICK tr|E1BZ28|E1BZ28\_CHICK tr|Q5ZHZ2|Q5ZHZ2\_CHICK tr|Q5YCC9|Q5YCC9\_CHICK tr|Q5ZMB0|Q5ZMB0\_CHICK tr|Q9PSV4|Q9PSV4\_CHICK tr|F1NEQ6|F1NEQ6\_CHICK sp|P43679|ACHB3\_CHICK tr|F1NG24|F1NG24\_CHICK tr|V5Y027|V5Y027\_CHICK tr|F1NU84|F1NU84\_CHICK tr|E1BXS6|E1BXS6\_CHICK tr|Q00G66|Q00G66\_CHICK tr|H9KZ56|H9KZ56\_CHICK tr|F1P4V3|F1P4V3\_CHICK tr|Q31406|Q31406\_CHICK tr|Q8UUX6|Q8UUX6\_CHICK tr|R4GM83|R4GM83\_CHICK tr|E1C9B3|E1C9B3\_CHICK tr|Q1PSV3|Q1PSV3\_CHICK tr|E1C6M2|E1C6M2\_CHICK tr|R4GLF2|R4GLF2\_CHICK tr|F1P3L3|F1P3L3\_CHICK tr|Q31399|Q31399\_CHICK tr|E1BR56|E1BR56\_CHICK tr|E1C4J0|E1C4J0\_CHICK tr|F1NZW5|F1NZW5\_CHICK tr|B5BSS8|B5BSS8\_CHICK tr|E6N1W5|E6N1W5\_CHICK tr|F1NRS3|F1NRS3\_CHICK tr|Q90656|Q90656\_CHICK tr|E1BXV2|E1BXV2\_CHICK tr|F1NK48|F1NK48\_CHICK tr|E1C4S4|E1C4S4\_CHICK tr|I0J1E4|I0J1E4\_CHICK sp|Q5F3G7|C1GLT\_CHICK tr|F1NVL3|F1NVL3\_CHICK tr|F1NF94|F1NF94\_CHICK tr|F1NZC3|F1NZC3\_CHICK tr|Q7SX83|Q7SX83\_CHICK tr|E1C2H4|E1C2H4\_CHICK tr|F1NAR5|F1NAR5\_CHICK tr|R4GFZ2|R4GFZ2\_CHICK tr|Q5ZL39|Q5ZL39\_CHICK tr|Q5ZLB2|Q5ZLB2\_CHICK tr|R4GI70|R4GI70\_CHICK tr|E1BXU6|E1BXU6\_CHICK tr|F1NSP7|F1NSP7\_CHICK tr|E1BUT0|E1BUT0\_CHICK tr|F1P182|F1P182\_CHICK tr|Q90658|Q90658\_CHICK tr|O42417|O42417\_CHICK tr|F1P4F9|F1P4F9\_CHICK tr|F1P3I8|F1P3I8\_CHICK tr|E1BYM6|E1BYM6\_CHICK tr|Q5ZL78|Q5ZL78\_CHICK tr|E1BTF3|E1BTF3\_CHICK tr|F1NVE0|F1NVE0\_CHICK tr|Q58G84|Q58G84\_CHICK tr|Q6Y2V9|Q6Y2V9\_CHICK tr|Q5F469|Q5F469\_CHICK tr|F1NYT3|F1NYT3\_CHICK tr|Q5ZLU2|Q5ZLU2\_CHICK tr|F1NZ04|F1NZ04\_CHICK tr|E1BR61|E1BR61\_CHICK tr|F1NBJ8|F1NBJ8\_CHICK tr|F1NIJ2|F1NIJ2\_CHICK tr|F1NSR5|F1NSR5\_CHICK tr|E1BXJ9|E1BXJ9\_CHICK tr|Q5ZMK9|Q5ZMK9\_CHICK tr|F1NIY0|F1NIY0\_CHICK tr|F1P174|F1P174\_CHICK tr|O42418|O42418\_CHICK sp|P84023|SMAD3\_CHICK tr|H9L2W1|H9L2W1\_CHICK tr|Q5ZLE2|Q5ZLE2\_CHICK tr|F1NH33|F1NH33\_CHICK tr|F1NIE8|F1NIE8\_CHICK tr|Q5ZJD2|Q5ZJD2\_CHICK tr|F1P550|F1P550\_CHICK tr|A0A089FKD3|A0A089FKD3\_CHICK tr|R4GHE7|R4GHE7\_CHICK tr|Q95601|Q95601\_CHICK sp|P15575|B3AT\_CHICK tr|R4GLT5|R4GLT5\_CHICK tr|R4GI68|R4GI68\_CHICK tr|A0A089GLF2|A0A089GLF2\_CHICK tr|R4GLN7|R4GLN7\_CHICK tr|Q91422|Q91422\_CHICK tr|F1NLP7|F1NLP7\_CHICK tr|Q5ZIB1|Q5ZIB1\_CHICK tr|F1NFC4|F1NFC4\_CHICK tr|F1NDT9|F1NDT9\_CHICK tr|E1C5W0|E1C5W0\_CHICK tr|E1BRP4|E1BRP4\_CHICK tr|F1P4E0|F1P4E0\_CHICK tr|H9L0F7|H9L0F7\_CHICK tr|F1NJR8|F1NJR8\_CHICK tr|F1NHX3|F1NHX3\_CHICK tr|R4GFK6|R4GFK6\_CHICK tr|R4GL25|R4GL25\_CHICK tr|D7REI7|D7REI7\_CHICK tr|F1NHZ1|F1NHZ1\_CHICK tr|R4GLD1|R4GLD1\_CHICK tr|E1BV88|E1BV88\_CHICK tr|Q0QJZ3|Q0QJZ3\_CHICK tr|F1P5K3|F1P5K3\_CHICK sp|P62207|PP1B\_CHICK tr|Q9DGN3|Q9DGN3\_CHICK tr|E1C7N9|E1C7N9\_CHICK tr|Q9PWM8|Q9PWM8\_CHICK tr|F1NP39|F1NP39\_CHICK tr|O57400|O57400\_CHICK tr|R4GGB3|R4GGB3\_CHICK tr|E1BUA6|E1BUA6\_CHICK sp|Q90752|BMP4\_CHICK tr|F1NUW6|F1NUW6\_CHICK tr|E1BQH6|E1BQH6\_CHICK tr|F1NHW4|F1NHW4\_CHICK tr|E1C7L0|E1C7L0\_CHICK tr|F1NAI4|F1NAI4\_CHICK tr|Q70ET0|Q70ET0\_CHICK tr|F1P0H8|F1P0H8\_CHICK tr|F1NKE5|F1NKE5\_CHICK tr|Q4ADW1|Q4ADW1\_CHICK tr|F1NPG0|F1NPG0\_CHICK tr|R4GM67|R4GM67\_CHICK sp|Q5F3A4|RELL1\_CHICK tr|E1BTL3|E1BTL3\_CHICK tr|R4GH66|R4GH66\_CHICK tr|Q5ZJI2|Q5ZJI2\_CHICK tr|Q5ZHY7|Q5ZHY7\_CHICK tr|F1N8U0|F1N8U0\_CHICK tr|Q5ZHS8|Q5ZHS8\_CHICK tr|F1NZ94|F1NZ94\_CHICK tr|E1BVD3|E1BVD3\_CHICK tr|F1P180|F1P180\_CHICK sp|P00508|AATM\_CHICK tr|F1P268|F1P268\_CHICK tr|E1C4W2|E1C4W2\_CHICK tr|R4GJ30|R4GJ30\_CHICK tr|E1BZB1|E1BZB1\_CHICK sp|Q5ZI04|FA49A\_CHICK tr|E1C8L4|E1C8L4\_CHICK sp|Q90845|GBRA6\_CHICK tr|E1C453|E1C453\_CHICK tr|F1NBT1|F1NBT1\_CHICK tr|F1NLA4|F1NLA4\_CHICK tr|R4GM15|R4GM15\_CHICK tr|Q5ZKB5|Q5ZKB5\_CHICK sp|Q5YCC5|TMC7\_CHICK tr|F1NIM0|F1NIM0\_CHICK tr|O46789|O46789\_CHICK tr|Q5ZJM7|Q5ZJM7\_CHICK tr|R4GFH6|R4GFH6\_CHICK tr|M9NKB1|M9NKB1\_CHICK sp|Q9YGX2|RPE65\_CHICK sp|O12940|TOM1\_CHICK tr|F1NQ65|F1NQ65\_CHICK tr|E1BVT8|E1BVT8\_CHICK tr|Q5ZIH7|Q5ZIH7\_CHICK sp|Q1T763|CENPR\_CHICK sp|Q5F3T7|CELF1\_CHICK tr|E1C134|E1C134\_CHICK tr|E1BYX9|E1BYX9\_CHICK tr|E1BQZ6|E1BQZ6\_CHICK tr|H2D5F4|H2D5F4\_CHICK tr|F1P231|F1P231\_CHICK tr|Q5ZLS4|Q5ZLS4\_CHICK tr|E1BTH5|E1BTH5\_CHICK tr|Q9DEI0|Q9DEI0\_CHICK tr|Q98SN5|Q98SN5\_CHICK tr|Q0QWA1|Q0QWA1\_CHICK tr|F1NSE7|F1NSE7\_CHICK tr|E1C495|E1C495\_CHICK tr|A1XP60|A1XP60\_CHICK tr|E1C0A7|E1C0A7\_CHICK tr|S0F2F7|S0F2F7\_CHICK tr|G9J1L7|G9J1L7\_CHICK tr|F1NH51|F1NH51\_CHICK tr|E1BZN0|E1BZN0\_CHICK tr|O42415|O42415\_CHICK tr|F1NFB8|F1NFB8\_CHICK tr|F1P3X9|F1P3X9\_CHICK tr|Q90WH8|Q90WH8\_CHICK tr|F1P0K5|F1P0K5\_CHICK tr|R4GG48|R4GG48\_CHICK tr|F1NSL4|F1NSL4\_CHICK tr|F1P1J2|F1P1J2\_CHICK tr|F1NCG5|F1NCG5\_CHICK sp|P09324|YES\_CHICK tr|F1P1R7|F1P1R7\_CHICK tr|E1C3U6|E1C3U6\_CHICK tr|E1BYR7|E1BYR7\_CHICK tr|E1C897|E1C897\_CHICK tr|R4GHT0|R4GHT0\_CHICK tr|R4GL26|R4GL26\_CHICK tr|F1N9S3|F1N9S3\_CHICK tr|F1NMK1|F1NMK1\_CHICK tr|F1NXT9|F1NXT9\_CHICK tr|E1BSC3|E1BSC3\_CHICK tr|R4GIT9|R4GIT9\_CHICK tr|R4GHL5|R4GHL5\_CHICK tr|Q8SPC8|Q8SPC8\_CHICK tr|F1P3Y0|F1P3Y0\_CHICK tr|Q5F367|Q5F367\_CHICK tr|K4LB56|K4LB56\_CHICK tr|Q5F4C2|Q5F4C2\_CHICK tr|F1NV20|F1NV20\_CHICK tr|Q4R1K1|Q4R1K1\_CHICK tr|Q5ZI00|Q5ZI00\_CHICK tr|Q5W9C1|Q5W9C1\_CHICK tr|F1NSQ1|F1NSQ1\_CHICK tr|Q9I9D1|Q9I9D1\_CHICK tr|Q5ZJZ2|Q5ZJZ2\_CHICK tr|Q5F431|Q5F431\_CHICK tr|F1N826|F1N826\_CHICK tr|R4GFU8|R4GFU8\_CHICK tr|H9L198|H9L198\_CHICK tr|R4GKY2|R4GKY2\_CHICK tr|R4GJT0|R4GJT0\_CHICK sp|P42292|CD166\_CHICK tr|Q5ZHK8|Q5ZHK8\_CHICK tr|F6VFK3|F6VFK3\_CHICK tr|E1C327|E1C327\_CHICK tr|B3VKP0|B3VKP0\_CHICK tr|B3GSU6|B3GSU6\_CHICK tr|E1BTD6|E1BTD6\_CHICK tr|E1BV45|E1BV45\_CHICK tr|E1BZE6|E1BZE6\_CHICK tr|E1C455|E1C455\_CHICK tr|Q7T1K3|Q7T1K3\_CHICK sp|P07341|ALDOB\_CHICK tr|E1C804|E1C804\_CHICK tr|E1C8M7|E1C8M7\_CHICK tr|E1BZF4|E1BZF4\_CHICK tr|F1P2J5|F1P2J5\_CHICK tr|F1NLU8|F1NLU8\_CHICK tr|F1NLV4|F1NLV4\_CHICK sp|Q5ZMN3|PHB2\_CHICK tr|O13254|O13254\_CHICK tr|E1BTN3|E1BTN3\_CHICK tr|R4GIJ7|R4GIJ7\_CHICK tr|R4GM32|R4GM32\_CHICK sp|E1C065|CEP41\_CHICK tr|F1NBQ1|F1NBQ1\_CHICK tr|F1NXH9|F1NXH9\_CHICK tr|F1NWJ3|F1NWJ3\_CHICK tr|R4GJ97|R4GJ97\_CHICK sp|O42392|VDR\_CHICK tr|F1NBY5|F1NBY5\_CHICK tr|Q5ZKR3|Q5ZKR3\_CHICK tr|F6SQ85|F6SQ85\_CHICK tr|F1P569|F1P569\_CHICK tr|E1C378|E1C378\_CHICK tr|E1BWP2|E1BWP2\_CHICK tr|F1NPI2|F1NPI2\_CHICK tr|E1BQU8|E1BQU8\_CHICK tr|G4XPB1|G4XPB1\_CHICK tr|G4XPA9|G4XPA9\_CHICK tr|E1C2N7|E1C2N7\_CHICK tr|F1NE95|F1NE95\_CHICK tr|E1C426|E1C426\_CHICK tr|Q5ZI99|Q5ZI99\_CHICK tr|G1EL48|G1EL48\_CHICK tr|C4PBR0|C4PBR0\_GALSO tr|C4PBP1|C4PBP1\_CHICK tr|E5L3Q3|E5L3Q3\_CHICK tr|C4PBP7|C4PBP7\_CHICK tr|C4PBN9|C4PBN9\_CHICK tr|C4PBP2|C4PBP2\_CHICK tr|C4PBR1|C4PBR1\_GALSO tr|C4PBP0|C4PBP0\_CHICK tr|H2D5F2|H2D5F2\_CHICK tr|F6U7W1|F6U7W1\_CHICK tr|C4PBS2|C4PBS2\_CHICK tr|C4PBQ7|C4PBQ7\_GALVA tr|C4PBQ4|C4PBQ4\_CHICK tr|C4PBS4|C4PBS4\_CHICK tr|Q2XQ10|Q2XQ10\_CHICK tr|C4PBQ2|C4PBQ2\_CHICK tr|F1P1B7|F1P1B7\_CHICK tr|R4GM30|R4GM30\_CHICK tr|H9L0L0|H9L0L0\_CHICK tr|R4GKY8|R4GKY8\_CHICK tr|Q2I810|Q2I810\_CHICK tr|F1NCQ3|F1NCQ3\_CHICK tr|H9L3C8|H9L3C8\_CHICK tr|E1BX06|E1BX06\_CHICK tr|F1N8K6|F1N8K6\_CHICK tr|R4GH40|R4GH40\_CHICK tr|F1NKV2|F1NKV2\_CHICK tr|Q9W601|Q9W601\_CHICK tr|F1NUW5|F1NUW5\_CHICK sp|Q5ZKQ8|ARL8A\_CHICK tr|F1NZC7|F1NZC7\_CHICK tr|E1C6G1|E1C6G1\_CHICK tr|E1BWF6|E1BWF6\_CHICK tr|R4GF61|R4GF61\_CHICK tr|F1N8S4|F1N8S4\_CHICK tr|F1NIJ8|F1NIJ8\_CHICK sp|P48430|SOX2\_CHICK sp|P09207|TBB6\_CHICK tr|F1NFZ4|F1NFZ4\_CHICK tr|F1NLA0|F1NLA0\_CHICK tr|E1C2T0|E1C2T0\_CHICK tr|F1P3S2|F1P3S2\_CHICK tr|H9L0J5|H9L0J5\_CHICK tr|E1BV09|E1BV09\_CHICK tr|A0M8U3|A0M8U3\_CHICK tr|R4GKI3|R4GKI3\_CHICK tr|R4GG50|R4GG50\_CHICK tr|F1NIS3|F1NIS3\_CHICK REFSEQ:XP\_986630 tr|Q90787|Q90787\_CHICK tr|Q5ZII4|Q5ZII4\_CHICK tr|R4GKI5|R4GKI5\_CHICK tr|E1BQV4|E1BQV4\_CHICK tr|Q5ZIS4|Q5ZIS4\_CHICK tr|F1P027|F1P027\_CHICK tr|Q9YH59|Q9YH59\_CHICK tr|A0A0C6E619|A0A0C6E619\_CHICK tr|F1NXM1|F1NXM1\_CHICK tr|A0A068Q816|A0A068Q816\_CHICK tr|F1P418|F1P418\_CHICK tr|F1NWA2|F1NWA2\_CHICK tr|Q8JH31|Q8JH31\_CHICK tr|Q5ZIU5|Q5ZIU5\_CHICK tr|E1C5V8|E1C5V8\_CHICK tr|F1NXJ0|F1NXJ0\_CHICK sp|Q1XA76|ASIC1\_CHICK tr|E1BZ86|E1BZ86\_CHICK tr|R9PXM6|R9PXM6\_CHICK sp|P04459|KRSC\_CHICK tr|F1NGQ6|F1NGQ6\_CHICK sp|O13157|GFRA2\_CHICK tr|E1C234|E1C234\_CHICK tr|F1NE16|F1NE16\_CHICK tr|R4GJF8|R4GJF8\_CHICK tr|Q5ZJ18|Q5ZJ18\_CHICK tr|F1NLI8|F1NLI8\_CHICK tr|Q5ZMI6|Q5ZMI6\_CHICK tr|R4GJV7|R4GJV7\_CHICK tr|A4ZZA2|A4ZZA2\_CHICK tr|R4GLI1|R4GLI1\_CHICK sp|Q5ZI11|LRC45\_CHICK tr|F1N9G5|F1N9G5\_CHICK sp|F1NPG5|CENPT\_CHICK sp|P51887|FMOD\_CHICK tr|F1NLZ4|F1NLZ4\_CHICK tr|E1BQI6|E1BQI6\_CHICK tr|E1C6J2|E1C6J2\_CHICK tr|F1NVU4|F1NVU4\_CHICK tr|A1EAT1|A1EAT1\_CHICK tr|F1N864|F1N864\_CHICK sp|P79785|AGTR1\_CHICK tr|J9R0G1|J9R0G1\_9SAUR tr|E1BVR7|E1BVR7\_CHICK tr|R4GGM2|R4GGM2\_CHICK tr|E1BRX4|E1BRX4\_CHICK tr|F1N931|F1N931\_CHICK sp|Q5ZIT9|MFSD1\_CHICK sp|Q90617|LAMP2\_CHICK tr|F1NVD4|F1NVD4\_CHICK tr|R4GMJ7|R4GMJ7\_CHICK tr|E1BXE8|E1BXE8\_CHICK sp|P51901|ANXA6\_CHICK tr|E1BR97|E1BR97\_CHICK tr|E1C815|E1C815\_CHICK tr|E1BWS8|E1BWS8\_CHICK tr|R4GJP3|R4GJP3\_CHICK tr|F1NY39|F1NY39\_CHICK sp|Q98937|FOXD1\_CHICK tr|R4GFC9|R4GFC9\_CHICK sp|Q5ZLK2|UBX2B\_CHICK tr|E1C620|E1C620\_CHICK tr|R4GGT1|R4GGT1\_CHICK tr|F1NSN3|F1NSN3\_CHICK tr|Q8QH06|Q8QH06\_CHICK tr|B0BL87|B0BL87\_CHICK tr|F1P141|F1P141\_CHICK tr|R4GH25|R4GH25\_CHICK sp|F1N9Y5|KSYK\_CHICK tr|A0A0A0MQ53|A0A0A0MQ53\_CHICK tr|E1C6W6|E1C6W6\_CHICK tr|Q90927|Q90927\_CHICK sp|P17923|NFIA\_CHICK tr|F1N9L1|F1N9L1\_CHICK tr|Q90928|Q90928\_CHICK tr|Q90925|Q90925\_CHICK tr|F1NGH6|F1NGH6\_CHICK tr|E1BSE2|E1BSE2\_CHICK tr|E1BUR4|E1BUR4\_CHICK tr|Q5ZIU2|Q5ZIU2\_CHICK tr|E1C1Y9|E1C1Y9\_CHICK tr|E1BUX5|E1BUX5\_CHICK tr|F1NC88|F1NC88\_CHICK tr|Q5ZL73|Q5ZL73\_CHICK tr|F1NU03|F1NU03\_CHICK tr|R4GME2|R4GME2\_CHICK tr|E1C8Y9|E1C8Y9\_CHICK tr|H9KYR1|H9KYR1\_CHICK tr|H9L083|H9L083\_CHICK tr|F1N816|F1N816\_CHICK tr|Q5F429|Q5F429\_CHICK tr|D2CP28|D2CP28\_CHICK sp|Q76I89|NDC80\_CHICK tr|B5AIG4|B5AIG4\_CHICK tr|B8YIG6|B8YIG6\_CHICK tr|B8YIG9|B8YIG9\_CHICK tr|B8YIG5|B8YIG5\_CHICK tr|B8YIR7|B8YIR7\_CHICK tr|B8YIH1|B8YIH1\_CHICK sp|P81021|VIGLN\_CHICK tr|B8YIQ5|B8YIQ5\_CHICK tr|B8YIS3|B8YIS3\_CHICK tr|B8YIT4|B8YIT4\_CHICK tr|F1NKZ8|F1NKZ8\_CHICK tr|E1BSR4|E1BSR4\_CHICK tr|F1NNN6|F1NNN6\_CHICK sp|E1C760|UVSSA\_CHICK tr|A0A0A0MQ54|A0A0A0MQ54\_CHICK tr|R4GHL0|R4GHL0\_CHICK tr|F1NDP8|F1NDP8\_CHICK tr|F1NHV7|F1NHV7\_CHICK tr|E1BV92|E1BV92\_CHICK tr|F1P5D8|F1P5D8\_CHICK tr|E1BWV5|E1BWV5\_CHICK tr|R4GIX2|R4GIX2\_CHICK tr|Q5ZK87|Q5ZK87\_CHICK tr|F1N9Z0|F1N9Z0\_CHICK tr|F1NA01|F1NA01\_CHICK sp|Q90578|VDHAP\_CHICK tr|E1C9C4|E1C9C4\_CHICK tr|H9L141|H9L141\_CHICK tr|Q5ZJB8|Q5ZJB8\_CHICK tr|E1C5C0|E1C5C0\_CHICK tr|F1NHH7|F1NHH7\_CHICK tr|H9KYY3|H9KYY3\_CHICK tr|F1NWR5|F1NWR5\_CHICK tr|F1NTI0|F1NTI0\_CHICK tr|E1C1R3|E1C1R3\_CHICK tr|B5BSP8|B5BSP8\_CHICK tr|E1C4A0|E1C4A0\_CHICK sp|Q9PT84|KCNH2\_CHICK tr|E1BWU5|E1BWU5\_CHICK tr|Q5ZHT5|Q5ZHT5\_CHICK tr|Q5K6J8|Q5K6J8\_CHICK tr|F1NNH8|F1NNH8\_CHICK sp|P60878|SNP25\_CHICK tr|F1P3I9|F1P3I9\_CHICK tr|Q08781|Q08781\_CHICK tr|R4GM68|R4GM68\_CHICK tr|E1C0X4|E1C0X4\_CHICK tr|F1NEM0|F1NEM0\_CHICK tr|R4GG38|R4GG38\_CHICK tr|R4GKJ4|R4GKJ4\_CHICK tr|Q5ZIV2|Q5ZIV2\_CHICK tr|F1P560|F1P560\_CHICK tr|R4GMG7|R4GMG7\_CHICK tr|F1NGS2|F1NGS2\_CHICK tr|Q5F3Y9|Q5F3Y9\_CHICK tr|R4GJN9|R4GJN9\_CHICK tr|A0A0A0MQ42|A0A0A0MQ42\_CHICK tr|F1NGS9|F1NGS9\_CHICK tr|E1BSV4|E1BSV4\_CHICK tr|R4GLJ9|R4GLJ9\_CHICK tr|Q5F3H9|Q5F3H9\_CHICK tr|E1C353|E1C353\_CHICK tr|F1NIX6|F1NIX6\_CHICK tr|E1C228|E1C228\_CHICK tr|Q5EFZ5|Q5EFZ5\_CHICK tr|B5BSK7|B5BSK7\_CHICK tr|F1NMA1|F1NMA1\_CHICK sp|Q8JG30|ST1B1\_CHICK sp|F1NB38|ECHD1\_CHICK tr|H9L0N9|H9L0N9\_CHICK tr|F1NKH2|F1NKH2\_CHICK sp|P38531|HSF3\_CHICK tr|E1BW14|E1BW14\_CHICK sp|A0M8U1|ST7\_CHICK tr|E1BU42|E1BU42\_CHICK tr|Q5ZM40|Q5ZM40\_CHICK tr|F1NIZ7|F1NIZ7\_CHICK tr|Q6JAX8|Q6JAX8\_CHICK tr|O93560|O93560\_CHICK tr|E1BU50|E1BU50\_CHICK tr|E1C2G1|E1C2G1\_CHICK tr|R4GGW6|R4GGW6\_CHICK tr|E1BXF9|E1BXF9\_CHICK tr|R4GI30|R4GI30\_CHICK sp|P13474|ETS1A\_CHICK sp|P28568|GTR3\_CHICK tr|F1NNH9|F1NNH9\_CHICK tr|G9LQW2|G9LQW2\_CHICK tr|Q9W6G0|Q9W6G0\_CHICK tr|Q643S1|Q643S1\_CHICK tr|F1P1Q0|F1P1Q0\_CHICK sp|Q9PWR4|VSIG1\_CHICK tr|E1C3A1|E1C3A1\_CHICK tr|Q5ZIM9|Q5ZIM9\_CHICK tr|E1BTS5|E1BTS5\_CHICK tr|F1NMG5|F1NMG5\_CHICK tr|F1NZ44|F1NZ44\_CHICK tr|Q8UUK1|Q8UUK1\_CHICK tr|J7GKU7|J7GKU7\_CHICK tr|Q5ZJW0|Q5ZJW0\_CHICK tr|H9L1E5|H9L1E5\_CHICK tr|Q90XG2|Q90XG2\_CHICK tr|E1C9D9|E1C9D9\_CHICK tr|R4GFT7|R4GFT7\_CHICK tr|C4PCM1|C4PCM1\_CHICK tr|C4PCP2|C4PCP2\_GALSO tr|C4PCN9|C4PCN9\_GALVA tr|Q5GQ97|Q5GQ97\_CHICK tr|C4PCL9|C4PCL9\_GALLA tr|A0A0A7MC93|A0A0A7MC93\_CHICK tr|F1NBN5|F1NBN5\_CHICK tr|A0A0A7MEX8|A0A0A7MEX8\_CHICK tr|A0A0A7M9V8|A0A0A7M9V8\_CHICK tr|Q5ZJD0|Q5ZJD0\_CHICK tr|A0A0A7MEY1|A0A0A7MEY1\_CHICK tr|Q4VKW2|Q4VKW2\_CHICK tr|R4GGW2|R4GGW2\_CHICK tr|R4GF98|R4GF98\_CHICK tr|B3TZB9|B3TZB9\_CHICK tr|R4GL77|R4GL77\_CHICK tr|R4GG39|R4GG39\_CHICK tr|Q5ZMM7|Q5ZMM7\_CHICK tr|F1NRL1|F1NRL1\_CHICK tr|F1P275|F1P275\_CHICK sp|Q56I99|SMAD5\_CHICK tr|Q9W6N3|Q9W6N3\_CHICK tr|F1P213|F1P213\_CHICK tr|F1NIE0|F1NIE0\_CHICK tr|F1N887|F1N887\_CHICK tr|Q5ZJ14|Q5ZJ14\_CHICK tr|F1P5I8|F1P5I8\_CHICK tr|Q5F382|Q5F382\_CHICK tr|Q8UWG6|Q8UWG6\_CHICK sp|Q9W676|LDB2\_CHICK tr|F1NI93|F1NI93\_CHICK tr|Q804J3|Q804J3\_CHICK sp|P26153|ACHB4\_CHICK tr|F1P4U2|F1P4U2\_CHICK tr|Q7T3Y2|Q7T3Y2\_CHICK tr|E1BTT8|E1BTT8\_CHICK tr|F1NH10|F1NH10\_CHICK sp|P00340|LDHA\_CHICK tr|Q9W6C4|Q9W6C4\_CHICK tr|F1NF82|F1NF82\_CHICK tr|F1N9P7|F1N9P7\_CHICK tr|R4GKC4|R4GKC4\_CHICK tr|F1NV78|F1NV78\_CHICK tr|F1P0P7|F1P0P7\_CHICK tr|F1NG93|F1NG93\_CHICK tr|H9L3K3|H9L3K3\_CHICK tr|F1NAJ4|F1NAJ4\_CHICK tr|R4GJR9|R4GJR9\_CHICK tr|E1BVM4|E1BVM4\_CHICK Q14525 tr|Q5ZL32|Q5ZL32\_CHICK tr|F1NEX9|F1NEX9\_CHICK tr|F1NB65|F1NB65\_CHICK tr|R4GGF9|R4GGF9\_CHICK tr|E1C4Z3|E1C4Z3\_CHICK tr|Q2ACE0|Q2ACE0\_CHICK tr|F1NK66|F1NK66\_CHICK sp|Q91049|OCLN\_CHICK tr|F1P1W3|F1P1W3\_CHICK tr|F1P1S1|F1P1S1\_CHICK tr|Q5ZIY6|Q5ZIY6\_CHICK tr|Q5ZJK3|Q5ZJK3\_CHICK tr|E1BTV9|E1BTV9\_CHICK tr|E1C8W8|E1C8W8\_CHICK tr|F1NJ65|F1NJ65\_CHICK tr|F1NXI9|F1NXI9\_CHICK tr|E1BVX9|E1BVX9\_CHICK sp|Q9PTR5|LIS1\_CHICK tr|F1NM69|F1NM69\_CHICK P17690 tr|F1NAI9|F1NAI9\_CHICK tr|F1P1H4|F1P1H4\_CHICK tr|R4GIZ2|R4GIZ2\_CHICK tr|Q5UMH8|Q5UMH8\_CHICK tr|E1C4A9|E1C4A9\_CHICK tr|F1NXG4|F1NXG4\_CHICK tr|C6EX06|C6EX06\_CHICK tr|Q6XDQ0|Q6XDQ0\_CHICK tr|E1C3M9|E1C3M9\_CHICK tr|B6V3H8|B6V3H8\_CHICK tr|Q8UVE3|Q8UVE3\_CHICK tr|F1P0F6|F1P0F6\_CHICK tr|Q66WK8|Q66WK8\_CHICK tr|Q5ZK46|Q5ZK46\_CHICK tr|E1BWH6|E1BWH6\_CHICK sp|P26152|ACHA5\_CHICK tr|Q5ZLH6|Q5ZLH6\_CHICK tr|F1P101|F1P101\_CHICK tr|R4GKB2|R4GKB2\_CHICK tr|F1NC58|F1NC58\_CHICK REFSEQ:XP\_092267 sp|Q9YH18|QKI\_CHICK tr|F1NWV0|F1NWV0\_CHICK tr|F1N9I8|F1N9I8\_CHICK tr|H9KYW9|H9KYW9\_CHICK sp|P28701|RXRG\_CHICK tr|B8XXD4|B8XXD4\_CHICK tr|F1NZG2|F1NZG2\_CHICK tr|F1P1X9|F1P1X9\_CHICK tr|Q98UJ6|Q98UJ6\_CHICK tr|F1NM08|F1NM08\_CHICK tr|Q9W744|Q9W744\_CHICK Q1RMN8 sp|P33145|CADHK\_CHICK tr|F1NUS5|F1NUS5\_CHICK tr|E3VVM5|E3VVM5\_CHICK tr|F1NCR3|F1NCR3\_CHICK sp|Q5ZIZ4|5NTC\_CHICK tr|F1NBJ5|F1NBJ5\_CHICK tr|E1BXR3|E1BXR3\_CHICK sp|Q90879|UB2V1\_CHICK tr|E1C639|E1C639\_CHICK tr|F1NJK8|F1NJK8\_CHICK tr|R4GH63|R4GH63\_CHICK tr|Q5ZHR2|Q5ZHR2\_CHICK tr|F1NUD4|F1NUD4\_CHICK tr|F1NZ72|F1NZ72\_CHICK tr|Q4H4C7|Q4H4C7\_CHICK sp|Q5ZHP7|EAF2\_CHICK tr|E1BX95|E1BX95\_CHICK tr|R4GFW0|R4GFW0\_CHICK tr|R4GK38|R4GK38\_CHICK tr|F1NF30|F1NF30\_CHICK tr|Q8QGJ2|Q8QGJ2\_CHICK tr|E1BYQ9|E1BYQ9\_CHICK tr|O42102|O42102\_CHICK tr|Q9PWI7|Q9PWI7\_CHICK tr|E1BXV1|E1BXV1\_CHICK tr|F1NHR1|F1NHR1\_CHICK tr|F1NUX8|F1NUX8\_CHICK tr|F1NS51|F1NS51\_CHICK tr|F1NB20|F1NB20\_CHICK tr|E1C769|E1C769\_CHICK tr|F7BWA2|F7BWA2\_CHICK tr|R4GGR8|R4GGR8\_CHICK tr|Q3HLQ9|Q3HLQ9\_CHICK tr|B6RCP1|B6RCP1\_CHICK tr|B5BSR6|B5BSR6\_CHICK tr|F1NNX5|F1NNX5\_CHICK sp|Q1T7C0|CENPL\_CHICK tr|R4GM07|R4GM07\_CHICK tr|F1NYT7|F1NYT7\_CHICK sp|O93505|TYRP2\_CHICK tr|F1CN48|F1CN48\_CHICK tr|F1NJC4|F1NJC4\_CHICK tr|F1P080|F1P080\_CHICK sp|Q5ZJU0|MOT9\_CHICK tr|R4GFQ6|R4GFQ6\_CHICK tr|F1P060|F1P060\_CHICK tr|A0A089FKC7|A0A089FKC7\_CHICK tr|F1NCJ8|F1NCJ8\_CHICK tr|F1NNP8|F1NNP8\_CHICK tr|E1C4L7|E1C4L7\_CHICK tr|R4GFP7|R4GFP7\_CHICK tr|E1BQM1|E1BQM1\_CHICK tr|E1C9A0|E1C9A0\_CHICK tr|Q7SZC3|Q7SZC3\_CHICK tr|F1NVH5|F1NVH5\_CHICK tr|Q8JHJ4|Q8JHJ4\_CHICK sp|P41239|CSK\_CHICK tr|R4GLF7|R4GLF7\_CHICK tr|A7Y7W3|A7Y7W3\_CHICK tr|F1NMH8|F1NMH8\_CHICK sp|P43449|CCNA2\_CHICK tr|F1NFN8|F1NFN8\_CHICK tr|F1NC74|F1NC74\_CHICK tr|Q5ZLX1|Q5ZLX1\_CHICK tr|E1BZA0|E1BZA0\_CHICK tr|E1BVI7|E1BVI7\_CHICK sp|Q5ZI03|CIR1\_CHICK tr|C5HV40|C5HV40\_CHICK tr|R4GJF2|R4GJF2\_CHICK sp|P05300|LAMP1\_CHICK tr|R4GGM4|R4GGM4\_CHICK tr|R4GJF9|R4GJF9\_CHICK tr|Q5ZL96|Q5ZL96\_CHICK tr|Q5ZIG7|Q5ZIG7\_CHICK tr|F1NCQ0|F1NCQ0\_CHICK tr|R4GKQ5|R4GKQ5\_CHICK tr|F1NJC8|F1NJC8\_CHICK tr|E1C2C8|E1C2C8\_CHICK tr|F1NDD6|F1NDD6\_CHICK tr|F1P1K3|F1P1K3\_CHICK tr|F1NND8|F1NND8\_CHICK tr|Q60GF6|Q60GF6\_CHICK tr|R4GKF6|R4GKF6\_CHICK sp|E1C3S7|TDRD7\_CHICK tr|F1NTU1|F1NTU1\_CHICK tr|R4GIT0|R4GIT0\_CHICK tr|E1BX77|E1BX77\_CHICK tr|F1NA92|F1NA92\_CHICK sp|P16075|MYOD1\_CHICK tr|F1NHM3|F1NHM3\_CHICK tr|C5J072|C5J072\_CHICK tr|F1NFG4|F1NFG4\_CHICK tr|E1C173|E1C173\_CHICK tr|E1BTV5|E1BTV5\_CHICK sp|Q3KTM2|GDPD5\_CHICK tr|F1NPA8|F1NPA8\_CHICK tr|Q7T3X8|Q7T3X8\_CHICK tr|Q9YHZ1|Q9YHZ1\_CHICK tr|F1NF24|F1NF24\_CHICK tr|F1P2G6|F1P2G6\_CHICK tr|F1N9P3|F1N9P3\_CHICK tr|Q5F387|Q5F387\_CHICK tr|E1BZ42|E1BZ42\_CHICK tr|F1NWH5|F1NWH5\_CHICK tr|F1NAS0|F1NAS0\_CHICK tr|F1NZZ3|F1NZZ3\_CHICK tr|Q90720|Q90720\_CHICK tr|Q5ZJW9|Q5ZJW9\_CHICK tr|E1BUR1|E1BUR1\_CHICK tr|F1NV60|F1NV60\_CHICK tr|Q5ZIP0|Q5ZIP0\_CHICK tr|Q5F3B6|Q5F3B6\_CHICK tr|Q5ZJP3|Q5ZJP3\_CHICK tr|F1NQ52|F1NQ52\_CHICK tr|Q5ZK11|Q5ZK11\_CHICK tr|F1NWV7|F1NWV7\_CHICK tr|F1NA42|F1NA42\_CHICK tr|Q802E5|Q802E5\_CHICK tr|E1C240|E1C240\_CHICK tr|E1C4U6|E1C4U6\_CHICK sp|P11682|APOB\_CHICK tr|F1NWC2|F1NWC2\_CHICK tr|C3VP78|C3VP78\_CHICK tr|E1BY16|E1BY16\_CHICK tr|Q6EDQ5|Q6EDQ5\_CHICK tr|Q25C35|Q25C35\_CHICK tr|E1BRH1|E1BRH1\_CHICK tr|F1NWK4|F1NWK4\_CHICK tr|E1BZV9|E1BZV9\_CHICK tr|F1NAA9|F1NAA9\_CHICK tr|E1C3D2|E1C3D2\_CHICK tr|E1BQG3|E1BQG3\_CHICK tr|F1NRZ9|F1NRZ9\_CHICK tr|E1C3J3|E1C3J3\_CHICK tr|E1BSF5|E1BSF5\_CHICK tr|F1N8G8|F1N8G8\_CHICK tr|E1C166|E1C166\_CHICK tr|E1C7K5|E1C7K5\_CHICK tr|R4GHJ8|R4GHJ8\_CHICK sp|P48433|SOX3\_CHICK tr|E1BTF5|E1BTF5\_CHICK tr|J9QLI8|J9QLI8\_CHICK tr|N0DN83|N0DN83\_CHICK tr|M1F4T7|M1F4T7\_CHICK tr|I2CD07|I2CD07\_CHICK tr|G9J1L4|G9J1L4\_CHICK tr|F8U4V7|F8U4V7\_CHICK tr|J9QQD5|J9QQD5\_CHICK tr|J9QPP2|J9QPP2\_CHICK tr|M1F3Z8|M1F3Z8\_CHICK tr|D6R469|D6R469\_CHICK tr|J9QPN9|J9QPN9\_CHICK tr|J9QMQ9|J9QMQ9\_CHICK tr|F5C9P2|F5C9P2\_CHICK tr|C0M4X0|C0M4X0\_CHICK tr|V9VL20|V9VL20\_CHICK tr|J7I307|J7I307\_CHICK tr|M1F5E8|M1F5E8\_CHICK tr|R4GGX2|R4GGX2\_CHICK tr|R4GHW3|R4GHW3\_CHICK tr|F5C9P3|F5C9P3\_CHICK tr|E1BSW0|E1BSW0\_CHICK P50448 tr|Q5F4B5|Q5F4B5\_CHICK tr|F1NIY7|F1NIY7\_CHICK tr|F1NQ94|F1NQ94\_CHICK tr|F1NI27|F1NI27\_CHICK sp|Q5ZK36|ING3\_CHICK tr|Q5ZK80|Q5ZK80\_CHICK tr|E1C338|E1C338\_CHICK tr|E1BTV8|E1BTV8\_CHICK tr|Q1XF99|Q1XF99\_CHICK tr|Q1XF81|Q1XF81\_CHICK tr|E1C030|E1C030\_CHICK tr|Q5ZMM9|Q5ZMM9\_CHICK sp|Q5ZMQ0|ARMC1\_CHICK tr|F1NR63|F1NR63\_CHICK sp|Q5F428|EIF3L\_CHICK tr|F1NG96|F1NG96\_CHICK tr|R4GKG7|R4GKG7\_CHICK sp|Q5ZK62|ACAP2\_CHICK tr|F1P304|F1P304\_CHICK tr|E1C3R0|E1C3R0\_CHICK tr|D1MJC6|D1MJC6\_CHICK tr|A6YIJ2|A6YIJ2\_CHICK tr|E1C8M3|E1C8M3\_CHICK tr|E1BWU2|E1BWU2\_CHICK tr|E1BST8|E1BST8\_CHICK tr|R4GIL4|R4GIL4\_CHICK tr|O57403|O57403\_CHICK tr|E1C7A8|E1C7A8\_CHICK tr|F1NC42|F1NC42\_CHICK tr|F1NPE7|F1NPE7\_CHICK tr|E1BY06|E1BY06\_CHICK tr|Q5ZJR4|Q5ZJR4\_CHICK tr|F1P4G9|F1P4G9\_CHICK tr|F1NI87|F1NI87\_CHICK tr|E1C7Q7|E1C7Q7\_CHICK tr|R4GI65|R4GI65\_CHICK tr|F1NHG7|F1NHG7\_CHICK tr|B1B565|B1B565\_CHICK tr|F1P1E4|F1P1E4\_CHICK tr|F1NKQ0|F1NKQ0\_CHICK tr|R4GKT3|R4GKT3\_CHICK tr|R4GH09|R4GH09\_CHICK sp|Q5ZJR3|OSTC\_CHICK tr|F1NIP1|F1NIP1\_CHICK tr|F1CN47|F1CN47\_CHICK tr|Q5ZL44|Q5ZL44\_CHICK tr|E1BZ83|E1BZ83\_CHICK tr|F1P5E2|F1P5E2\_CHICK tr|R4GIB0|R4GIB0\_CHICK tr|B0FSL1|B0FSL1\_CHICK tr|C9DIF5|C9DIF5\_CHICK tr|E1C390|E1C390\_CHICK tr|F1NG89|F1NG89\_CHICK sp|Q98SN7|WNT2B\_CHICK sp|Q5ZJN4|UBP10\_CHICK tr|F1NYR9|F1NYR9\_CHICK tr|Q3HWX0|Q3HWX0\_CHICK sp|P68259|GLUC\_CHICK tr|E1BRI1|E1BRI1\_CHICK tr|A6XKD7|A6XKD7\_CHICK tr|E1BSP1|E1BSP1\_CHICK sp|O13035|SAP\_CHICK tr|F1NAW2|F1NAW2\_CHICK tr|O42347|O42347\_CHICK tr|F1NZ03|F1NZ03\_CHICK tr|F1P134|F1P134\_CHICK tr|F1NUK9|F1NUK9\_CHICK sp|Q5ZLR5|UCRI\_CHICK tr|F1NNJ0|F1NNJ0\_CHICK A2A5Y0 sp|Q5ZHN9|PGPS1\_CHICK tr|F1NYP9|F1NYP9\_CHICK tr|F1N926|F1N926\_CHICK sp|P09653|TBB5\_CHICK tr|Q29ZM6|Q29ZM6\_CHICK tr|F1P1E0|F1P1E0\_CHICK tr|F1NVR6|F1NVR6\_CHICK tr|R4GGM0|R4GGM0\_CHICK tr|E1BXC7|E1BXC7\_CHICK tr|F1P1B8|F1P1B8\_CHICK tr|Q5ZI38|Q5ZI38\_CHICK tr|F1NVZ1|F1NVZ1\_CHICK tr|E1BRQ4|E1BRQ4\_CHICK tr|F1ND58|F1ND58\_CHICK tr|Q1G7H2|Q1G7H2\_CHICK tr|E1BWC6|E1BWC6\_CHICK tr|Q71SY8|Q71SY8\_CHICK tr|F1NSG0|F1NSG0\_CHICK tr|F1P3W2|F1P3W2\_CHICK tr|Q5ZJ52|Q5ZJ52\_CHICK tr|E1C5I8|E1C5I8\_CHICK tr|Q5ZLR0|Q5ZLR0\_CHICK tr|E1C6H2|E1C6H2\_CHICK tr|F1NQ46|F1NQ46\_CHICK tr|F1CN59|F1CN59\_CHICK tr|R9PXQ4|R9PXQ4\_CHICK sp|Q2YHT7|MOR1A\_CHICK tr|F1NSU8|F1NSU8\_CHICK tr|F1NVX4|F1NVX4\_CHICK tr|F1NFS1|F1NFS1\_CHICK tr|Q9YGP0|Q9YGP0\_CHICK tr|F1NMH2|F1NMH2\_CHICK tr|F1NLN6|F1NLN6\_CHICK tr|B3XZF6|B3XZF6\_CHICK tr|F1NAU4|F1NAU4\_CHICK tr|F1NVU2|F1NVU2\_CHICK tr|E1C6R3|E1C6R3\_CHICK tr|F1NML3|F1NML3\_CHICK tr|F1NQS8|F1NQS8\_CHICK tr|R4GIK1|R4GIK1\_CHICK tr|Q5F397|Q5F397\_CHICK tr|F1NGY6|F1NGY6\_CHICK tr|Q804X5|Q804X5\_CHICK tr|F1P0T2|F1P0T2\_CHICK tr|F1NQA7|F1NQA7\_CHICK tr|R4GIP6|R4GIP6\_CHICK tr|A5HMN8|A5HMN8\_CHICK tr|Q5ZKP8|Q5ZKP8\_CHICK tr|F1CN27|F1CN27\_CHICK tr|A0MAR6|A0MAR6\_CHICK tr|F1NGI9|F1NGI9\_CHICK tr|Q6EE57|Q6EE57\_CHICK tr|Q58I02|Q58I02\_CHICK tr|N0DM08|N0DM08\_CHICK tr|K9JTN9|K9JTN9\_CHICK tr|R4GG64|R4GG64\_CHICK tr|Q5ZKU3|Q5ZKU3\_CHICK tr|F1P1Q2|F1P1Q2\_CHICK tr|F6VAJ0|F6VAJ0\_CHICK sp|Q5ZL74|VAMP7\_CHICK tr|Q5F3K8|Q5F3K8\_CHICK tr|E1BR24|E1BR24\_CHICK tr|H9L1W4|H9L1W4\_CHICK tr|E1BSL4|E1BSL4\_CHICK tr|R4GL40|R4GL40\_CHICK tr|Q802S8|Q802S8\_CHICK tr|J9R284|J9R284\_9SAUR tr|Q5ZLP7|Q5ZLP7\_CHICK tr|E1BR52|E1BR52\_CHICK tr|Q5ZKG9|Q5ZKG9\_CHICK tr|E1C800|E1C800\_CHICK tr|E1C9B9|E1C9B9\_CHICK tr|Q5ZI54|Q5ZI54\_CHICK tr|B2X023|B2X023\_CHICK tr|F1P5J6|F1P5J6\_CHICK tr|R4GHN4|R4GHN4\_CHICK tr|R4GKB3|R4GKB3\_CHICK tr|B4ZY91|B4ZY91\_CHICK tr|Q5ZKA1|Q5ZKA1\_CHICK tr|F1P0K9|F1P0K9\_CHICK tr|E1C8W5|E1C8W5\_CHICK tr|E1BS79|E1BS79\_CHICK tr|H9L3I1|H9L3I1\_CHICK tr|E1C0F3|E1C0F3\_CHICK tr|Q5ZHR6|Q5ZHR6\_CHICK tr|E1C997|E1C997\_CHICK sp|Q05199|NRG1\_CHICK tr|F1NIV4|F1NIV4\_CHICK tr|F1NIV5|F1NIV5\_CHICK tr|F1P1K0|F1P1K0\_CHICK tr|E1BTT3|E1BTT3\_CHICK tr|H9L0R7|H9L0R7\_CHICK tr|H9L3C5|H9L3C5\_CHICK tr|F1NHI7|F1NHI7\_CHICK tr|F1P4X8|F1P4X8\_CHICK tr|R4GGR3|R4GGR3\_CHICK tr|J9QW21|J9QW21\_9SAUR sp|Q5ZIA0|MET16\_CHICK tr|F1NEB0|F1NEB0\_CHICK tr|F1NCV8|F1NCV8\_CHICK tr|Q5ZL64|Q5ZL64\_CHICK tr|F1NPK1|F1NPK1\_CHICK tr|E1C6V3|E1C6V3\_CHICK tr|Q5ZL83|Q5ZL83\_CHICK tr|R4GJQ4|R4GJQ4\_CHICK tr|Q5ZI86|Q5ZI86\_CHICK tr|F1P145|F1P145\_CHICK tr|E1BZS4|E1BZS4\_CHICK tr|Q5ZJ11|Q5ZJ11\_CHICK tr|F1NNG3|F1NNG3\_CHICK tr|H9KYY4|H9KYY4\_CHICK tr|F1NTQ2|F1NTQ2\_CHICK tr|E1C6B9|E1C6B9\_CHICK tr|F1NNQ1|F1NNQ1\_CHICK tr|F1NWP1|F1NWP1\_CHICK tr|F1NBB7|F1NBB7\_CHICK tr|Q5ZLX7|Q5ZLX7\_CHICK tr|R4GKL8|R4GKL8\_CHICK tr|F1P015|F1P015\_CHICK sp|Q5ZHX6|S41A2\_CHICK tr|F1P359|F1P359\_CHICK tr|A5HUJ8|A5HUJ8\_CHICK tr|E1C1U1|E1C1U1\_CHICK tr|F1NE79|F1NE79\_CHICK tr|R4GGB5|R4GGB5\_CHICK tr|F1N9W7|F1N9W7\_CHICK tr|F1CN22|F1CN22\_CHICK tr|E1C788|E1C788\_CHICK tr|R4GI85|R4GI85\_CHICK tr|Q5ZKQ9|Q5ZKQ9\_CHICK tr|E1BWN3|E1BWN3\_CHICK sp|P84173|PHB\_CHICK tr|F1NA79|F1NA79\_CHICK tr|F1NTL0|F1NTL0\_CHICK tr|F1NZV2|F1NZV2\_CHICK tr|R4GIY1|R4GIY1\_CHICK tr|Q90XC0|Q90XC0\_CHICK sp|Q9DG25|POPD3\_CHICK tr|Q1XF74|Q1XF74\_CHICK tr|E1BS40|E1BS40\_CHICK tr|Q5ZK48|Q5ZK48\_CHICK tr|F1P0L8|F1P0L8\_CHICK tr|Q0KKP5|Q0KKP5\_CHICK tr|F1N843|F1N843\_CHICK sp|O73770|DGCR6\_CHICK tr|Q5F3I5|Q5F3I5\_CHICK tr|F1NSQ5|F1NSQ5\_CHICK tr|E1BT92|E1BT92\_CHICK tr|Q8JHX2|Q8JHX2\_CHICK tr|E1BWQ8|E1BWQ8\_CHICK tr|F1P0K0|F1P0K0\_CHICK tr|R4GI55|R4GI55\_CHICK tr|E1BS99|E1BS99\_CHICK tr|Q5ZKT7|Q5ZKT7\_CHICK tr|F6UK83|F6UK83\_CHICK tr|Q5ZI31|Q5ZI31\_CHICK tr|F1NFK3|F1NFK3\_CHICK tr|F1NBE0|F1NBE0\_CHICK tr|F1NTJ5|F1NTJ5\_CHICK tr|E1BWW0|E1BWW0\_CHICK tr|E1C7S1|E1C7S1\_CHICK tr|E1BSP9|E1BSP9\_CHICK tr|F1NJ62|F1NJ62\_CHICK tr|E1C7L4|E1C7L4\_CHICK tr|E1BTE0|E1BTE0\_CHICK tr|Q5ZIL3|Q5ZIL3\_CHICK tr|F1NIP7|F1NIP7\_CHICK tr|F1NN83|F1NN83\_CHICK tr|F1P1T3|F1P1T3\_CHICK sp|P05200|NGF\_CHICK tr|B4ZE58|B4ZE58\_CHICK tr|Q5ZJU7|Q5ZJU7\_CHICK tr|E1BU27|E1BU27\_CHICK tr|E1BS25|E1BS25\_CHICK sp|Q5ZLK5|P4HA2\_CHICK tr|F1P3J6|F1P3J6\_CHICK tr|V9H0G0|V9H0G0\_CHICK tr|F1P051|F1P051\_CHICK tr|Q5ZIB7|Q5ZIB7\_CHICK tr|F1NHP5|F1NHP5\_CHICK tr|F1P2N1|F1P2N1\_CHICK tr|F1NF17|F1NF17\_CHICK sp|Q9DDN6|NPY2R\_CHICK tr|F1N9Q1|F1N9Q1\_CHICK tr|F1NEB1|F1NEB1\_CHICK tr|E1C688|E1C688\_CHICK tr|O93568|O93568\_CHICK tr|B1PMA7|B1PMA7\_CHICK tr|Q5ZKL3|Q5ZKL3\_CHICK tr|Q98TY6|Q98TY6\_CHICK tr|Q49B65|Q49B65\_CHICK tr|E1BZU4|E1BZU4\_CHICK tr|E1C461|E1C461\_CHICK tr|Q5F3L2|Q5F3L2\_CHICK tr|Q2YHU2|Q2YHU2\_CHICK tr|R4GFH2|R4GFH2\_CHICK tr|F1NZ61|F1NZ61\_CHICK tr|Q5ZIE5|Q5ZIE5\_CHICK tr|Q5ZLM5|Q5ZLM5\_CHICK tr|R4GJG8|R4GJG8\_CHICK tr|R4GLG5|R4GLG5\_CHICK tr|F1NFK7|F1NFK7\_CHICK tr|R4GIZ8|R4GIZ8\_CHICK tr|F1N9B7|F1N9B7\_CHICK tr|Q5ZMJ5|Q5ZMJ5\_CHICK tr|F6QJK6|F6QJK6\_CHICK tr|R4GMJ3|R4GMJ3\_CHICK tr|B2DFX5|B2DFX5\_CHICK tr|F1NWT1|F1NWT1\_CHICK tr|E1BSM1|E1BSM1\_CHICK tr|F1NY60|F1NY60\_CHICK tr|E1BW27|E1BW27\_CHICK tr|F1NME5|F1NME5\_CHICK tr|Q90996|Q90996\_CHICK sp|Q5ZKJ0|CLP1L\_CHICK tr|V9GVH6|V9GVH6\_CHICK tr|E1C1F4|E1C1F4\_CHICK tr|F1P527|F1P527\_CHICK tr|R4GFQ0|R4GFQ0\_CHICK tr|F1NZD3|F1NZD3\_CHICK tr|F1NHW1|F1NHW1\_CHICK tr|E1BU29|E1BU29\_CHICK sp|P34065|PSB5\_CHICK tr|E1BTT7|E1BTT7\_CHICK tr|E1BS39|E1BS39\_CHICK tr|F1P0R5|F1P0R5\_CHICK tr|F1NEW4|F1NEW4\_CHICK tr|F1NGR3|F1NGR3\_CHICK Q9D646 tr|Q5ZIV0|Q5ZIV0\_CHICK tr|Q5ZHR4|Q5ZHR4\_CHICK sp|Q90944|EPYC\_CHICK tr|R4GHK6|R4GHK6\_CHICK tr|R4GK45|R4GK45\_CHICK tr|Q8QGS3|Q8QGS3\_CHICK tr|Q5ZLZ1|Q5ZLZ1\_CHICK tr|F1NEN7|F1NEN7\_CHICK tr|Q6JAX4|Q6JAX4\_CHICK tr|F5HN09|F5HN09\_CHICK tr|E1C668|E1C668\_CHICK tr|R4GK65|R4GK65\_CHICK tr|F1NT16|F1NT16\_CHICK tr|R4GLZ3|R4GLZ3\_CHICK tr|F1NGI8|F1NGI8\_CHICK tr|F1NDM4|F1NDM4\_CHICK tr|F1NC56|F1NC56\_CHICK tr|F1NU17|F1NU17\_CHICK sp|P51903|PGK\_CHICK tr|E1BR96|E1BR96\_CHICK tr|F1P475|F1P475\_CHICK tr|G9F974|G9F974\_9SAUR tr|N0GS02|N0GS02\_9SAUR tr|A0A060PQF0|A0A060PQF0\_CHICK tr|F1NUA0|F1NUA0\_CHICK tr|F1NSB5|F1NSB5\_CHICK tr|G9F976|G9F976\_9SAUR tr|F1NV83|F1NV83\_CHICK tr|F1NN24|F1NN24\_CHICK tr|E1BY82|E1BY82\_CHICK tr|F1NUM4|F1NUM4\_CHICK tr|A2NR64|A2NR64\_CHICK sp|O13113|DAD1\_CHICK tr|F1NS30|F1NS30\_CHICK tr|B7U497|B7U497\_CHICK tr|Q5ZJZ8|Q5ZJZ8\_CHICK tr|F1NB75|F1NB75\_CHICK tr|F1NLY4|F1NLY4\_CHICK tr|Q2PBB8|Q2PBB8\_CHICK tr|Q5ZJ76|Q5ZJ76\_CHICK tr|F1NP12|F1NP12\_CHICK tr|Q5ZHV9|Q5ZHV9\_CHICK tr|F1ND54|F1ND54\_CHICK sp|P16236|REL\_CHICK tr|F1NQH2|F1NQH2\_CHICK tr|E0A2T5|E0A2T5\_CHICK tr|Q91415|Q91415\_CHICK sp|P14791|HMOX1\_CHICK tr|F1NQF4|F1NQF4\_CHICK tr|F1NTI2|F1NTI2\_CHICK tr|Q8AYG6|Q8AYG6\_CHICK sp|Q804A9|DAZL\_CHICK tr|F1NGJ9|F1NGJ9\_CHICK tr|E1C8Q2|E1C8Q2\_CHICK tr|E1C2Q6|E1C2Q6\_CHICK tr|R4GJ48|R4GJ48\_CHICK tr|E1BRU0|E1BRU0\_CHICK tr|F1NHC3|F1NHC3\_CHICK tr|Q645R0|Q645R0\_CHICK tr|R4GGT9|R4GGT9\_CHICK tr|E1BR58|E1BR58\_CHICK tr|F1NN91|F1NN91\_CHICK tr|R4GKU1|R4GKU1\_CHICK tr|F1P4H6|F1P4H6\_CHICK tr|Q5ZLU1|Q5ZLU1\_CHICK tr|F1NCW0|F1NCW0\_CHICK tr|R4GJB2|R4GJB2\_CHICK tr|F1CN10|F1CN10\_CHICK tr|F1NEG3|F1NEG3\_CHICK sp|Q5F3D1|ZCHC8\_CHICK tr|F1NML4|F1NML4\_CHICK tr|F1NA16|F1NA16\_CHICK tr|E1BVY8|E1BVY8\_CHICK Q3MHH8 tr|F1NUA4|F1NUA4\_CHICK tr|Q5ZM04|Q5ZM04\_CHICK tr|F1NAZ4|F1NAZ4\_CHICK tr|F1NA86|F1NA86\_CHICK tr|H9KZC8|H9KZC8\_CHICK tr|F1NUQ0|F1NUQ0\_CHICK tr|E1C1J4|E1C1J4\_CHICK tr|E1C916|E1C916\_CHICK tr|F1NH19|F1NH19\_CHICK tr|E1C6Q3|E1C6Q3\_CHICK tr|F1NKJ7|F1NKJ7\_CHICK tr|A7DY55|A7DY55\_CHICK tr|Q5ZK07|Q5ZK07\_CHICK tr|F1NXJ8|F1NXJ8\_CHICK tr|Q5ZKA9|Q5ZKA9\_CHICK REFSEQ:XP\_932229 tr|R4GFM1|R4GFM1\_CHICK sp|Q5ZKL5|PPCEL\_CHICK tr|F1NG64|F1NG64\_CHICK tr|Q6F4E1|Q6F4E1\_CHICK tr|B5BSD6|B5BSD6\_CHICK tr|E1BW77|E1BW77\_CHICK tr|Q6F4E0|Q6F4E0\_CHICK tr|F1NUM9|F1NUM9\_CHICK tr|Q90ZA9|Q90ZA9\_CHICK tr|F1NMR0|F1NMR0\_CHICK tr|H9KYW7|H9KYW7\_CHICK tr|F1P197|F1P197\_CHICK tr|A3QW61|A3QW61\_CHICK tr|F1NDH2|F1NDH2\_CHICK tr|F1P0S7|F1P0S7\_CHICK tr|Q5R2J0|Q5R2J0\_CHICK tr|P79792|P79792\_CHICK tr|B8YLV3|B8YLV3\_CHICK tr|Q7T1F1|Q7T1F1\_CHICK sp|Q5ZJY5|SMC5\_CHICK tr|R4GHU0|R4GHU0\_CHICK tr|E1C1A2|E1C1A2\_CHICK tr|F1NFP6|F1NFP6\_CHICK tr|E1C272|E1C272\_CHICK tr|Q5ZL09|Q5ZL09\_CHICK tr|O73664|O73664\_CHICK tr|Q8QGX0|Q8QGX0\_CHICK tr|Q98SU9|Q98SU9\_CHICK tr|Q5ZMB6|Q5ZMB6\_CHICK tr|R4GLU6|R4GLU6\_CHICK tr|Q2HQA3|Q2HQA3\_CHICK tr|E1BUI1|E1BUI1\_CHICK tr|R4GKN8|R4GKN8\_CHICK tr|Q2MCJ7|Q2MCJ7\_CHICK tr|G1EL59|G1EL59\_CHICK tr|H2D5F5|H2D5F5\_CHICK tr|H2D5F7|H2D5F7\_CHICK tr|Q5ZI59|Q5ZI59\_CHICK tr|F1NSP0|F1NSP0\_CHICK tr|F1NVG4|F1NVG4\_CHICK tr|Q860H4|Q860H4\_CHICK tr|E1BZF1|E1BZF1\_CHICK tr|E1BU30|E1BU30\_CHICK tr|E1BS82|E1BS82\_CHICK tr|E1C3U9|E1C3U9\_CHICK tr|F1NAH7|F1NAH7\_CHICK tr|R4GHQ6|R4GHQ6\_CHICK tr|F1N9F9|F1N9F9\_CHICK tr|E1BWT4|E1BWT4\_CHICK tr|R4GJ73|R4GJ73\_CHICK sp|O42479|HEMH\_CHICK tr|F1NBT4|F1NBT4\_CHICK tr|E1C0X2|E1C0X2\_CHICK tr|R4GL55|R4GL55\_CHICK tr|E9LUH8|E9LUH8\_CHICK tr|E9LUH6|E9LUH6\_CHICK tr|F1NGI2|F1NGI2\_CHICK tr|Q5ILH0|Q5ILH0\_CHICK tr|F1NZ15|F1NZ15\_CHICK tr|Q3T1V5|Q3T1V5\_CHICK tr|R4GK11|R4GK11\_CHICK tr|E1BS34|E1BS34\_CHICK tr|E1C127|E1C127\_CHICK tr|E1BSR1|E1BSR1\_CHICK tr|R4GGH4|R4GGH4\_CHICK tr|L7NSZ5|L7NSZ5\_CHICK tr|H9KZK5|H9KZK5\_CHICK sp|Q90X25|HXA13\_CHICK tr|Q9PVJ8|Q9PVJ8\_CHICK tr|Q90853|Q90853\_CHICK tr|F1NMB8|F1NMB8\_CHICK tr|R4GL07|R4GL07\_CHICK tr|F1P323|F1P323\_CHICK tr|F1P2P3|F1P2P3\_CHICK tr|S5DMU6|S5DMU6\_CHICK sp|Q04594|PRLR\_CHICK tr|H9CX01|H9CX01\_CHICK tr|E1BU22|E1BU22\_CHICK sp|Q7T2T1|CELF2\_CHICK tr|F1NZY6|F1NZY6\_CHICK tr|Q90Z44|Q90Z44\_CHICK sp|Q90732|PRS4\_CHICK tr|E1C9C0|E1C9C0\_CHICK tr|E1BSA8|E1BSA8\_CHICK tr|V9LU50|V9LU50\_CHICK tr|F1NPF2|F1NPF2\_CHICK tr|R4GKT8|R4GKT8\_CHICK tr|D1M7A9|D1M7A9\_CHICK tr|A1DPK0|A1DPK0\_CHICK tr|F1P4K4|F1P4K4\_CHICK tr|E1BYU6|E1BYU6\_CHICK tr|Q5ZKE8|Q5ZKE8\_CHICK tr|F1P521|F1P521\_CHICK sp|Q5ZKW2|LZIC\_CHICK tr|E1BXD1|E1BXD1\_CHICK tr|F1NJB4|F1NJB4\_CHICK tr|F1NWE7|F1NWE7\_CHICK tr|E1BZQ1|E1BZQ1\_CHICK tr|F1P0K6|F1P0K6\_CHICK tr|F1NY62|F1NY62\_CHICK tr|E1C7N4|E1C7N4\_CHICK tr|E1C1Q6|E1C1Q6\_CHICK tr|F6R228|F6R228\_CHICK tr|Q5ZKZ2|Q5ZKZ2\_CHICK tr|F1NEP7|F1NEP7\_CHICK tr|Q5ZMN7|Q5ZMN7\_CHICK tr|E1C902|E1C902\_CHICK sp|Q7T0L4|TE2IP\_CHICK tr|F1NKZ1|F1NKZ1\_CHICK tr|F1NPZ3|F1NPZ3\_CHICK tr|R4GKY1|R4GKY1\_CHICK tr|R4GJN1|R4GJN1\_CHICK tr|R4GIA1|R4GIA1\_CHICK tr|Q5ZI63|Q5ZI63\_CHICK tr|F1NHL7|F1NHL7\_CHICK tr|R4GH89|R4GH89\_CHICK tr|Q5ZHT6|Q5ZHT6\_CHICK sp|Q5ZM30|RPR1A\_CHICK tr|F1NQ92|F1NQ92\_CHICK tr|A9QM69|A9QM69\_CHICK tr|F1NA47|F1NA47\_CHICK sp|P46692|HMD1\_CHICK tr|E1C013|E1C013\_CHICK tr|Q5W9B9|Q5W9B9\_CHICK tr|E1BYR0|E1BYR0\_CHICK tr|B4X7M6|B4X7M6\_CHICK tr|A0FK59|A0FK59\_CHICK sp|Q90888|MAFB\_CHICK tr|R4GHZ8|R4GHZ8\_CHICK tr|B7U496|B7U496\_CHICK tr|B5BSS3|B5BSS3\_CHICK tr|F1P4N7|F1P4N7\_CHICK tr|R4GF32|R4GF32\_CHICK tr|F1P090|F1P090\_CHICK tr|E1C2G0|E1C2G0\_CHICK tr|F1P446|F1P446\_CHICK tr|R4GKF3|R4GKF3\_CHICK tr|E1C1G6|E1C1G6\_CHICK tr|F1NNI3|F1NNI3\_CHICK tr|F1N8J2|F1N8J2\_CHICK Q3ZBD7 tr|F1NYG6|F1NYG6\_CHICK tr|F1P5E0|F1P5E0\_CHICK tr|E1C197|E1C197\_CHICK sp|P01013|OVALX\_CHICK tr|H9L2H9|H9L2H9\_CHICK tr|F1NXA5|F1NXA5\_CHICK tr|B2X043|B2X043\_CHICK tr|B2X044|B2X044\_CHICK tr|M4MBM4|M4MBM4\_CHICK tr|B2X026|B2X026\_CHICK tr|B2X024|B2X024\_CHICK tr|F1NM72|F1NM72\_CHICK sp|P00368|DHE3\_CHICK tr|E1C1C9|E1C1C9\_CHICK tr|E1BTM2|E1BTM2\_CHICK tr|E1BRE9|E1BRE9\_CHICK sp|P28675|PGS2\_CHICK tr|E1BT66|E1BT66\_CHICK tr|Q45QT1|Q45QT1\_CHICK sp|Q90XB3|OLIG2\_CHICK tr|F1NXY9|F1NXY9\_CHICK tr|F1P165|F1P165\_CHICK tr|Q3S4W8|Q3S4W8\_CHICK tr|Q5ZK93|Q5ZK93\_CHICK tr|R4GIC0|R4GIC0\_CHICK tr|R4GLE9|R4GLE9\_CHICK tr|F1P4W5|F1P4W5\_CHICK tr|F1P458|F1P458\_CHICK sp|P49152|CRBA4\_CHICK tr|F1NGK4|F1NGK4\_CHICK tr|Q9I9V3|Q9I9V3\_CHICK tr|F1NVX8|F1NVX8\_CHICK tr|E1C2V9|E1C2V9\_CHICK tr|Q98UJ8|Q98UJ8\_CHICK tr|F1NZ06|F1NZ06\_CHICK tr|B2ZAJ9|B2ZAJ9\_CHICK tr|R4GHL3|R4GHL3\_CHICK tr|Q5ZL41|Q5ZL41\_CHICK tr|F1NL29|F1NL29\_CHICK tr|R4GLJ1|R4GLJ1\_CHICK tr|Q9DDW4|Q9DDW4\_CHICK tr|F1NA20|F1NA20\_CHICK tr|F1CN53|F1CN53\_CHICK tr|R4GJI4|R4GJI4\_CHICK tr|E1C1U8|E1C1U8\_CHICK tr|F1NGE6|F1NGE6\_CHICK tr|E1BZ76|E1BZ76\_CHICK sp|Q9YGL6|PALM\_CHICK tr|Q4LES8|Q4LES8\_CHICK tr|E1BRL5|E1BRL5\_CHICK Q2TBQ1 sp|Q1T7B8|CENPO\_CHICK tr|R4GJG9|R4GJG9\_CHICK tr|A5HUJ6|A5HUJ6\_CHICK sp|Q5ZID2|PDD2L\_CHICK tr|A0A0A0MQ56|A0A0A0MQ56\_CHICK tr|F1NFL0|F1NFL0\_CHICK tr|F1NS88|F1NS88\_CHICK tr|F1NB23|F1NB23\_CHICK tr|F1NL99|F1NL99\_CHICK tr|F1NEK9|F1NEK9\_CHICK tr|F1NVB4|F1NVB4\_CHICK sp|Q5ZK90|ARSK\_CHICK sp|P25155|FA10\_CHICK sp|Q9YGV8|TBPL1\_CHICK tr|E1BRU8|E1BRU8\_CHICK tr|F1NB02|F1NB02\_CHICK tr|F1NW50|F1NW50\_CHICK tr|F1NVU9|F1NVU9\_CHICK tr|H9L025|H9L025\_CHICK tr|Q5ZKF1|Q5ZKF1\_CHICK tr|F1P541|F1P541\_CHICK tr|Q5ZHU5|Q5ZHU5\_CHICK tr|R4GGT6|R4GGT6\_CHICK tr|Q5ZM70|Q5ZM70\_CHICK tr|H9L0J2|H9L0J2\_CHICK tr|E1C2R6|E1C2R6\_CHICK sp|P84169|PSD13\_CHICK tr|Q3S2V0|Q3S2V0\_CHICK sp|E1BW58|MIEAP\_CHICK tr|E1C2X1|E1C2X1\_CHICK tr|E1BUT1|E1BUT1\_CHICK tr|Q6EE60|Q6EE60\_CHICK sp|P14315|CAPZB\_CHICK tr|F1NWF7|F1NWF7\_CHICK tr|E1BYF8|E1BYF8\_CHICK tr|F1P045|F1P045\_CHICK tr|F1NSZ7|F1NSZ7\_CHICK tr|R4GGW8|R4GGW8\_CHICK tr|Q5ZKH5|Q5ZKH5\_CHICK tr|F1NV08|F1NV08\_CHICK tr|E1BV25|E1BV25\_CHICK sp|P05153|PCKGC\_CHICK tr|F1NSA7|F1NSA7\_CHICK tr|F1NVY0|F1NVY0\_CHICK tr|Q98SM6|Q98SM6\_CHICK tr|F1NBW0|F1NBW0\_CHICK tr|F1P3B3|F1P3B3\_CHICK tr|Q800J1|Q800J1\_CHICK tr|F1N8E9|F1N8E9\_CHICK tr|F1ND04|F1ND04\_CHICK sp|P30374|RSFR\_CHICK tr|E1C0M9|E1C0M9\_CHICK tr|Q27J90|Q27J90\_CHICK sp|Q5F464|LPP\_CHICK tr|R4GIM9|R4GIM9\_CHICK tr|Q5F3U2|Q5F3U2\_CHICK tr|A0A068FDL3|A0A068FDL3\_CHICK tr|Q2P9U8|Q2P9U8\_CHICK tr|Q5EFE0|Q5EFE0\_CHICK tr|D3K4G3|D3K4G3\_CHICK sp|P20785|CO6A1\_CHICK tr|R4GKM5|R4GKM5\_CHICK tr|F1NFE0|F1NFE0\_CHICK tr|F1NYE6|F1NYE6\_CHICK tr|E1C978|E1C978\_CHICK tr|E1BWR7|E1BWR7\_CHICK tr|F1NSA1|F1NSA1\_CHICK tr|Q5ZI93|Q5ZI93\_CHICK tr|E1BRL2|E1BRL2\_CHICK tr|F1P4I2|F1P4I2\_CHICK tr|F1NBR6|F1NBR6\_CHICK tr|R4GI43|R4GI43\_CHICK sp|P09480|ACHA2\_CHICK tr|F1NHP4|F1NHP4\_CHICK tr|E1BV70|E1BV70\_CHICK tr|R4GGK7|R4GGK7\_CHICK tr|E1C3U3|E1C3U3\_CHICK Q6IFU6 tr|F1NX75|F1NX75\_CHICK tr|F1NRD7|F1NRD7\_CHICK sp|Q90839|DKK3\_CHICK sp|Q5ZLR7|TM251\_CHICK tr|F1NTT2|F1NTT2\_CHICK tr|R4GL44|R4GL44\_CHICK sp|P28173|PUR1\_CHICK tr|F1P014|F1P014\_CHICK tr|F1NFP2|F1NFP2\_CHICK tr|F1NJ32|F1NJ32\_CHICK tr|F1NFQ8|F1NFQ8\_CHICK tr|E1BWP0|E1BWP0\_CHICK tr|E1BQL3|E1BQL3\_CHICK sp|Q5ZLC7|MARE1\_CHICK tr|R4GM52|R4GM52\_CHICK tr|F1P1D0|F1P1D0\_CHICK tr|Q70M89|Q70M89\_CHICK tr|R4GLG4|R4GLG4\_CHICK tr|Q5F3G5|Q5F3G5\_CHICK tr|Q5ZI18|Q5ZI18\_CHICK tr|Q6PWI2|Q6PWI2\_CHICK sp|P18625|FOSL2\_CHICK tr|F1NDF4|F1NDF4\_CHICK tr|Q5ZL88|Q5ZL88\_CHICK tr|E1C6B4|E1C6B4\_CHICK tr|F1P3R0|F1P3R0\_CHICK tr|R4GL27|R4GL27\_CHICK tr|E1BYF1|E1BYF1\_CHICK tr|R4GGY0|R4GGY0\_CHICK tr|Q5F3W4|Q5F3W4\_CHICK sp|Q5F448|GPHR\_CHICK sp|P49285|MTR1A\_CHICK tr|I2E112|I2E112\_CHICK sp|Q5ZML0|BRE\_CHICK tr|F1NPP0|F1NPP0\_CHICK tr|Q6F4E3|Q6F4E3\_CHICK tr|G1K2Z3|G1K2Z3\_CHICK tr|F1NJ97|F1NJ97\_CHICK tr|Q5ZIQ2|Q5ZIQ2\_CHICK tr|R4GFS6|R4GFS6\_CHICK tr|F1NFD9|F1NFD9\_CHICK tr|Q5ZMI9|Q5ZMI9\_CHICK tr|Q5ZI29|Q5ZI29\_CHICK tr|A8WEN5|A8WEN5\_CHICK tr|F1P4H1|F1P4H1\_CHICK tr|A7M7B8|A7M7B8\_CHICK sp|Q5ZMA2|PRP19\_CHICK tr|Q7LZ15|Q7LZ15\_CHICK tr|E1C2F2|E1C2F2\_CHICK tr|F1NSY0|F1NSY0\_CHICK tr|Q9I9F1|Q9I9F1\_CHICK tr|Q90929|Q90929\_CHICK tr|E1BWL0|E1BWL0\_CHICK tr|F1P352|F1P352\_CHICK tr|R4GMA0|R4GMA0\_CHICK tr|Q9DF19|Q9DF19\_CHICK tr|F1NDA9|F1NDA9\_CHICK tr|Q5ZL01|Q5ZL01\_CHICK tr|B4X7M0|B4X7M0\_CHICK tr|E1BT72|E1BT72\_CHICK tr|E1BW49|E1BW49\_CHICK tr|Q5ZIK8|Q5ZIK8\_CHICK tr|R4GIV8|R4GIV8\_CHICK tr|F1NV52|F1NV52\_CHICK tr|R4GL29|R4GL29\_CHICK tr|Q5ZM15|Q5ZM15\_CHICK tr|R4GLC9|R4GLC9\_CHICK tr|R4GLG7|R4GLG7\_CHICK tr|F1NX57|F1NX57\_CHICK tr|E1BVA3|E1BVA3\_CHICK sp|Q5ZKQ3|RPAP3\_CHICK tr|E1C5K8|E1C5K8\_CHICK tr|Q5F4B9|Q5F4B9\_CHICK tr|Q90Y34|Q90Y34\_CHICK tr|F1NUI5|F1NUI5\_CHICK tr|Q9DDJ1|Q9DDJ1\_CHICK sp|Q5ZKE5|CAB45\_CHICK tr|E1BSE7|E1BSE7\_CHICK tr|E0W5Z8|E0W5Z8\_ALEAL tr|F1NJ60|F1NJ60\_CHICK sp|Q5ZJ27|HOOK1\_CHICK tr|H9KZF0|H9KZF0\_CHICK tr|F1NWT3|F1NWT3\_CHICK tr|E1BTF0|E1BTF0\_CHICK tr|F1NRG1|F1NRG1\_CHICK tr|F1NEB5|F1NEB5\_CHICK sp|Q9W734|SMAD6\_CHICK tr|C3U1W5|C3U1W5\_CHICK tr|Q90603|Q90603\_CHICK tr|F1N807|F1N807\_CHICK tr|Q7LZL1|Q7LZL1\_CHICK sp|P13387|EGFR\_CHICK tr|Q6Q247|Q6Q247\_CHICK tr|Q6DKY0|Q6DKY0\_CHICK tr|E1BZC6|E1BZC6\_CHICK tr|E1BZZ5|E1BZZ5\_CHICK sp|Q5F480|ITPK1\_CHICK tr|R9PXQ7|R9PXQ7\_CHICK tr|F1P1L1|F1P1L1\_CHICK tr|F1NSA3|F1NSA3\_CHICK tr|R4GMA9|R4GMA9\_CHICK tr|F1P331|F1P331\_CHICK tr|F1NQD9|F1NQD9\_CHICK tr|R4GJZ9|R4GJZ9\_CHICK sp|P01875|IGHM\_CHICK tr|G3G8J2|G3G8J2\_CHICK tr|Q6W8X3|Q6W8X3\_CHICK tr|Q9I882|Q9I882\_CHICK tr|F1P5K1|F1P5K1\_CHICK tr|F7AVB9|F7AVB9\_CHICK tr|H9L261|H9L261\_CHICK tr|F1NJZ4|F1NJZ4\_CHICK tr|F1NVZ0|F1NVZ0\_CHICK tr|Q5F3R0|Q5F3R0\_CHICK tr|E1BV78|E1BV78\_CHICK tr|Q6X0K9|Q6X0K9\_CHICK tr|F1P439|F1P439\_CHICK tr|R4GJS2|R4GJS2\_CHICK tr|F1NFV6|F1NFV6\_CHICK tr|F1NG67|F1NG67\_CHICK tr|E1C7B2|E1C7B2\_CHICK tr|F1NH77|F1NH77\_CHICK tr|F1NFW7|F1NFW7\_CHICK tr|F1NZ87|F1NZ87\_CHICK tr|R4GJR2|R4GJR2\_CHICK tr|F1N8G4|F1N8G4\_CHICK Q14533 tr|E1C8B6|E1C8B6\_CHICK tr|Q5ZMU9|Q5ZMU9\_CHICK tr|A5HMN7|A5HMN7\_CHICK tr|H9KYT1|H9KYT1\_CHICK sp|Q5ZL23|AB1IP\_CHICK tr|Q3HS34|Q3HS34\_CHICK tr|Q5ZLK0|Q5ZLK0\_CHICK tr|F1NLT2|F1NLT2\_CHICK tr|C5J076|C5J076\_CHICK tr|Q5ZKV1|Q5ZKV1\_CHICK tr|E1C3S1|E1C3S1\_CHICK tr|E1BQR5|E1BQR5\_CHICK tr|F1NS93|F1NS93\_CHICK tr|F1NNN8|F1NNN8\_CHICK tr|Q9I8C9|Q9I8C9\_CHICK tr|R4GKH5|R4GKH5\_CHICK tr|F1NJ08|F1NJ08\_CHICK tr|F1NVL6|F1NVL6\_CHICK tr|E1BWD5|E1BWD5\_CHICK tr|R9PXL9|R9PXL9\_CHICK tr|F1N986|F1N986\_CHICK tr|Q5ZME1|Q5ZME1\_CHICK tr|Q5UHA2|Q5UHA2\_CHICK tr|E1BXF8|E1BXF8\_CHICK tr|A0A0C4ZNN6|A0A0C4ZNN6\_9NEOP tr|E5L8C8|E5L8C8\_CHICK tr|E1BXA3|E1BXA3\_CHICK tr|Q5F460|Q5F460\_CHICK tr|B2ZE94|B2ZE94\_CHICK tr|E1C057|E1C057\_CHICK tr|E1BVL4|E1BVL4\_CHICK tr|H9KZI4|H9KZI4\_CHICK tr|F1N8B8|F1N8B8\_CHICK tr|Q5F4C6|Q5F4C6\_CHICK tr|E1C3M5|E1C3M5\_CHICK tr|R4GHD4|R4GHD4\_CHICK tr|D7RXP0|D7RXP0\_CHICK tr|Q5ZIX6|Q5ZIX6\_CHICK tr|Q9W6F7|Q9W6F7\_CHICK tr|E1C755|E1C755\_CHICK tr|R4GL57|R4GL57\_CHICK tr|B5BSN0|B5BSN0\_CHICK tr|H9L0I5|H9L0I5\_CHICK tr|F1NQC3|F1NQC3\_CHICK tr|F1P372|F1P372\_CHICK tr|R4GFC6|R4GFC6\_CHICK tr|E1C3J0|E1C3J0\_CHICK tr|A3RF19|A3RF19\_CHICK tr|F1NS31|F1NS31\_CHICK tr|E1C1N0|E1C1N0\_CHICK tr|F1P1Y0|F1P1Y0\_CHICK tr|Q5ZIH1|Q5ZIH1\_CHICK sp|O93319|CAD11\_CHICK tr|B5BSF1|B5BSF1\_CHICK tr|E1C9K4|E1C9K4\_CHICK tr|H8Y6B1|H8Y6B1\_CHICK tr|R4GFV1|R4GFV1\_CHICK tr|R4GH04|R4GH04\_CHICK tr|N0DLW6|N0DLW6\_CHICK tr|E1BVN1|E1BVN1\_CHICK tr|R4GLY8|R4GLY8\_CHICK sp|P58270|DPF3\_CHICK tr|Q5ZL61|Q5ZL61\_CHICK tr|Q5XXX2|Q5XXX2\_CHICK tr|F1NNM9|F1NNM9\_CHICK tr|F6UU17|F6UU17\_CHICK tr|Q800I0|Q800I0\_CHICK tr|R4GHV1|R4GHV1\_CHICK tr|R4GJ72|R4GJ72\_CHICK tr|F1NST1|F1NST1\_CHICK tr|E0AEW6|E0AEW6\_CHICK tr|F1P4D6|F1P4D6\_CHICK tr|R4GFB8|R4GFB8\_CHICK tr|F1NI90|F1NI90\_CHICK sp|Q5ZIM5|MAP1\_CHICK tr|F1NV41|F1NV41\_CHICK tr|F1P029|F1P029\_CHICK tr|Q98934|Q98934\_CHICK tr|R4GK16|R4GK16\_CHICK tr|E1C288|E1C288\_CHICK tr|F1P4S3|F1P4S3\_CHICK tr|E1BZ56|E1BZ56\_CHICK tr|F1P0F8|F1P0F8\_CHICK tr|E1BXN4|E1BXN4\_CHICK tr|E1C532|E1C532\_CHICK tr|F1NME0|F1NME0\_CHICK tr|Q5ZKL4|Q5ZKL4\_CHICK tr|F1NVP6|F1NVP6\_CHICK tr|Q5ZI28|Q5ZI28\_CHICK sp|Q05437|PRRX1\_CHICK tr|H9L0U6|H9L0U6\_CHICK tr|F1NK29|F1NK29\_CHICK tr|E1BYG1|E1BYG1\_CHICK tr|E1BSA4|E1BSA4\_CHICK tr|Q4JMA6|Q4JMA6\_CHICK tr|F1P3M9|F1P3M9\_CHICK tr|F1NBI4|F1NBI4\_CHICK tr|F1P5H3|F1P5H3\_CHICK tr|E1BX58|E1BX58\_CHICK tr|R4GGX7|R4GGX7\_CHICK tr|F1P429|F1P429\_CHICK sp|P52731|PDE6C\_CHICK tr|X2J6L4|X2J6L4\_CHICK tr|F7AXQ1|F7AXQ1\_CHICK tr|Q5F370|Q5F370\_CHICK tr|R4GHZ9|R4GHZ9\_CHICK sp|Q98ST6|PI15\_CHICK tr|E1C3M7|E1C3M7\_CHICK tr|Q5ZKU9|Q5ZKU9\_CHICK tr|R4GFL4|R4GFL4\_CHICK sp|P52186|KCNJ2\_CHICK tr|O42229|O42229\_CHICK tr|E1BVT2|E1BVT2\_CHICK tr|F1P4S1|F1P4S1\_CHICK tr|F1NU02|F1NU02\_CHICK tr|F1NSV2|F1NSV2\_CHICK sp|B0BK70|OFCC1\_CHICK tr|F1CN07|F1CN07\_CHICK tr|Q9PTI4|Q9PTI4\_CHICK tr|E1BVG4|E1BVG4\_CHICK tr|E1C2F6|E1C2F6\_CHICK tr|F1NWN5|F1NWN5\_CHICK tr|F1NIX3|F1NIX3\_CHICK tr|F1NVB6|F1NVB6\_CHICK tr|E1C4X0|E1C4X0\_CHICK tr|F1N8V0|F1N8V0\_CHICK tr|R4GF84|R4GF84\_CHICK tr|E1C6P3|E1C6P3\_CHICK tr|F1NK03|F1NK03\_CHICK tr|F1NIZ9|F1NIZ9\_CHICK sp|Q90Y35|ZN622\_CHICK tr|Q5ZHL7|Q5ZHL7\_CHICK tr|F1NIE1|F1NIE1\_CHICK tr|Q90819|Q90819\_CHICK tr|E1BTW5|E1BTW5\_CHICK tr|E1BXH8|E1BXH8\_CHICK tr|R4GFN0|R4GFN0\_CHICK tr|O42399|O42399\_CHICK tr|F1N8U6|F1N8U6\_CHICK sp|Q5ZLS8|INT10\_CHICK tr|R4GJX6|R4GJX6\_CHICK tr|E1C5W4|E1C5W4\_CHICK tr|F1P4N9|F1P4N9\_CHICK tr|F1N9Z4|F1N9Z4\_CHICK tr|Q5ZHR0|Q5ZHR0\_CHICK tr|F1N973|F1N973\_CHICK tr|E1C6B5|E1C6B5\_CHICK tr|F1NJP9|F1NJP9\_CHICK tr|A9X890|A9X890\_CHICK A3EZ79 tr|F1NB88|F1NB88\_CHICK tr|H9L027|H9L027\_CHICK tr|E1C6R4|E1C6R4\_CHICK tr|Q5YCC6|Q5YCC6\_CHICK tr|Q9PSD6|Q9PSD6\_CHICK tr|R4GG25|R4GG25\_CHICK tr|F1ND45|F1ND45\_CHICK tr|E1C6L5|E1C6L5\_CHICK sp|Q90922|NET1\_CHICK tr|K4HVD3|K4HVD3\_CHICK tr|E1C5Q4|E1C5Q4\_CHICK tr|F1NU67|F1NU67\_CHICK tr|R4GHT8|R4GHT8\_CHICK P13717 sp|Q5ZMA6|NADE\_CHICK tr|F1P4D3|F1P4D3\_CHICK tr|Q0R4I5|Q0R4I5\_CHICK tr|R4GHR7|R4GHR7\_CHICK tr|Q9I9K4|Q9I9K4\_CHICK tr|F1N847|F1N847\_CHICK tr|F1NEN3|F1NEN3\_CHICK tr|E1C2A2|E1C2A2\_CHICK tr|C0L7M6|C0L7M6\_CHICK tr|S4V308|S4V308\_GALVA tr|S4V4A6|S4V4A6\_GALSO tr|O73739|O73739\_CHICK tr|F1NY03|F1NY03\_CHICK tr|D5G0G8|D5G0G8\_CHICK tr|S4V3S8|S4V3S8\_GALLA tr|F1NWW1|F1NWW1\_CHICK tr|F1P0C1|F1P0C1\_CHICK tr|E1BTC3|E1BTC3\_CHICK sp|Q5ZK13|KIZ\_CHICK tr|E1BQH0|E1BQH0\_CHICK tr|B5BSA9|B5BSA9\_CHICK tr|R4GF93|R4GF93\_CHICK tr|F1NQ19|F1NQ19\_CHICK tr|E1C3L7|E1C3L7\_CHICK sp|Q9I8C7|ACH10\_CHICK tr|F1NWW3|F1NWW3\_CHICK sp|Q5ZIF3|UFSP2\_CHICK tr|D0QTG3|D0QTG3\_CHICK tr|I3WWD2|I3WWD2\_CHICK tr|D0QTH8|D0QTH8\_CHICK sp|Q90890|LY86\_CHICK tr|F1P4F3|F1P4F3\_CHICK tr|O57596|O57596\_CHICK tr|F1N9Q5|F1N9Q5\_CHICK tr|R4GLP4|R4GLP4\_CHICK tr|F1NGM3|F1NGM3\_CHICK tr|W6CMA7|W6CMA7\_CHICK tr|F1NP76|F1NP76\_CHICK tr|E1C8G6|E1C8G6\_CHICK sp|Q5ZKK1|MARE2\_CHICK tr|E1BX90|E1BX90\_CHICK sp|Q90687|PTN11\_CHICK tr|F1CN08|F1CN08\_CHICK tr|R4GJD7|R4GJD7\_CHICK tr|F1NWS4|F1NWS4\_CHICK tr|F1P062|F1P062\_CHICK tr|E1C6A3|E1C6A3\_CHICK sp|Q90891|MP2K2\_CHICK tr|F1P5B9|F1P5B9\_CHICK tr|Q9PUJ4|Q9PUJ4\_CHICK tr|F1N833|F1N833\_CHICK sp|Q8AYC9|CHK1\_CHICK tr|Q6Y0L7|Q6Y0L7\_CHICK sp|E1C656|HACE1\_CHICK tr|Q6J4Y8|Q6J4Y8\_CHICK tr|E1BXJ0|E1BXJ0\_CHICK tr|F1NNP1|F1NNP1\_CHICK tr|F1NBC4|F1NBC4\_CHICK tr|F1NF93|F1NF93\_CHICK sp|P14840|HXB4\_CHICK tr|F1P3B4|F1P3B4\_CHICK tr|Q6F4F0|Q6F4F0\_CHICK tr|F1NKT3|F1NKT3\_CHICK tr|E1BWY9|E1BWY9\_CHICK tr|E1BUR2|E1BUR2\_CHICK tr|E1BXX7|E1BXX7\_CHICK tr|E1BQU6|E1BQU6\_CHICK sp|Q9I8F4|TPD53\_CHICK tr|E1C6T1|E1C6T1\_CHICK tr|Q5ZII0|Q5ZII0\_CHICK tr|F1NI15|F1NI15\_CHICK tr|Q8UVD4|Q8UVD4\_CHICK tr|F1P2B2|F1P2B2\_CHICK sp|P10157|ETS2\_CHICK sp|P62758|NCALD\_CHICK tr|F1P220|F1P220\_CHICK sp|Q5ZIW1|PEO1\_CHICK tr|E1C878|E1C878\_CHICK tr|U5LXR4|U5LXR4\_CHICK tr|E1C5V2|E1C5V2\_CHICK tr|Q5ZI96|Q5ZI96\_CHICK tr|F1NZJ0|F1NZJ0\_CHICK tr|E1BWV6|E1BWV6\_CHICK tr|F1NB18|F1NB18\_CHICK tr|Q7LZ62|Q7LZ62\_CHICK tr|F1NHC0|F1NHC0\_CHICK tr|R4GHA7|R4GHA7\_CHICK tr|E1C1D1|E1C1D1\_CHICK tr|F1NXS2|F1NXS2\_CHICK tr|F1NNL2|F1NNL2\_CHICK tr|B9W049|B9W049\_CHICK tr|F1NXX1|F1NXX1\_CHICK tr|G4U4M4|G4U4M4\_CHICK tr|F1NH35|F1NH35\_CHICK sp|Q90X07|PSN2\_CHICK sp|P12394|CP17A\_CHICK tr|E1C202|E1C202\_CHICK tr|E1C0V9|E1C0V9\_CHICK tr|F1ND20|F1ND20\_CHICK tr|R4GMB8|R4GMB8\_CHICK tr|Q5ZK83|Q5ZK83\_CHICK tr|Q5ZIB5|Q5ZIB5\_CHICK tr|A6BLM6|A6BLM6\_CHICK tr|H9L2W5|H9L2W5\_CHICK tr|Q9DGG3|Q9DGG3\_CHICK tr|F1NH97|F1NH97\_CHICK tr|Q1XF96|Q1XF96\_CHICK tr|F1NTU7|F1NTU7\_CHICK tr|Q5ZJN5|Q5ZJN5\_CHICK tr|F6R8T3|F6R8T3\_CHICK tr|F1NAT0|F1NAT0\_CHICK tr|R4GM61|R4GM61\_CHICK tr|F1NMU4|F1NMU4\_CHICK tr|E1C4W4|E1C4W4\_CHICK tr|B3TZB4|B3TZB4\_CHICK tr|Q9YHB6|Q9YHB6\_CHICK tr|R4GK91|R4GK91\_CHICK tr|F1NZ67|F1NZ67\_CHICK tr|Q5ZLK9|Q5ZLK9\_CHICK tr|R9TNA6|R9TNA6\_CHICK tr|F1P1F2|F1P1F2\_CHICK tr|E1BXQ7|E1BXQ7\_CHICK tr|F1NT55|F1NT55\_CHICK tr|Q5ZIT1|Q5ZIT1\_CHICK tr|O42134|O42134\_CHICK tr|F1NLU0|F1NLU0\_CHICK tr|F1NB79|F1NB79\_CHICK tr|Q5QJU5|Q5QJU5\_CHICK tr|F1NW22|F1NW22\_CHICK sp|Q90997|TFR1\_CHICK tr|F1NT13|F1NT13\_CHICK tr|C4P7J2|C4P7J2\_CHICK sp|Q5ZMC9|NDE1\_CHICK tr|E1BXT5|E1BXT5\_CHICK tr|E1BY76|E1BY76\_CHICK tr|Q5ZIG8|Q5ZIG8\_CHICK Q2KIU3 tr|F1P596|F1P596\_CHICK tr|Q659W9|Q659W9\_CHICK tr|Q5ZJ49|Q5ZJ49\_CHICK tr|B7U494|B7U494\_CHICK tr|Q5ZL40|Q5ZL40\_CHICK tr|Q5ZHW3|Q5ZHW3\_CHICK tr|E1C9E5|E1C9E5\_CHICK tr|F1NIL0|F1NIL0\_CHICK tr|F1NUA6|F1NUA6\_CHICK tr|H9L315|H9L315\_CHICK tr|Q5ZK59|Q5ZK59\_CHICK tr|E1BRK3|E1BRK3\_CHICK tr|E1C3K5|E1C3K5\_CHICK tr|F1NVI1|F1NVI1\_CHICK tr|F1NW42|F1NW42\_CHICK tr|B8YIW3|B8YIW3\_CHICK tr|F1NMC4|F1NMC4\_CHICK tr|O73909|O73909\_CHICK tr|Q5ZKE6|Q5ZKE6\_CHICK tr|E9LHC8|E9LHC8\_CHICK tr|B0LHV2|B0LHV2\_CHICK tr|F4ZWC1|F4ZWC1\_CHICK tr|R4GGV7|R4GGV7\_CHICK tr|R4GMH5|R4GMH5\_CHICK tr|F1NGL2|F1NGL2\_CHICK tr|Q9YI93|Q9YI93\_CHICK tr|F1P4C1|F1P4C1\_CHICK tr|E1C6W3|E1C6W3\_CHICK tr|F1NI80|F1NI80\_CHICK sp|P23991|ADH1\_CHICK tr|Q5ZK71|Q5ZK71\_CHICK tr|F1P5B8|F1P5B8\_CHICK tr|O93342|O93342\_CHICK tr|Q5ZJY0|Q5ZJY0\_CHICK tr|H9L0H4|H9L0H4\_CHICK tr|F1NEH5|F1NEH5\_CHICK tr|E1BV90|E1BV90\_CHICK tr|Q5ZKQ4|Q5ZKQ4\_CHICK tr|R4QVB0|R4QVB0\_CHICK tr|E1C499|E1C499\_CHICK sp|P28238|MIP\_CHICK tr|R4GHJ0|R4GHJ0\_CHICK tr|F1P3F4|F1P3F4\_CHICK tr|Q9DEG1|Q9DEG1\_CHICK tr|E1C5H3|E1C5H3\_CHICK tr|E1BSX2|E1BSX2\_CHICK tr|E1C4D9|E1C4D9\_CHICK sp|Q5ZHN5|THAP5\_CHICK tr|R4GG11|R4GG11\_CHICK tr|F1NK22|F1NK22\_CHICK tr|E1BZM5|E1BZM5\_CHICK tr|F1NSB1|F1NSB1\_CHICK tr|R4GIL1|R4GIL1\_CHICK sp|Q90733|COT2\_CHICK tr|F1NC78|F1NC78\_CHICK tr|Q6XLQ0|Q6XLQ0\_CHICK tr|B2KSC0|B2KSC0\_CHICK tr|E5G6H7|E5G6H7\_CHICK tr|F1NG83|F1NG83\_CHICK tr|H9KZY8|H9KZY8\_CHICK tr|A0A090HZI4|A0A090HZI4\_CHICK sp|K9JA28|INLR1\_CHICK tr|F1P2Y9|F1P2Y9\_CHICK tr|E1C117|E1C117\_CHICK tr|F1NN05|F1NN05\_CHICK tr|Q5ZK57|Q5ZK57\_CHICK tr|F1P3S7|F1P3S7\_CHICK tr|Q5ZHN7|Q5ZHN7\_CHICK tr|F1NPY2|F1NPY2\_CHICK sp|P16527|MARCS\_CHICK tr|R4GLN8|R4GLN8\_CHICK tr|R4GKP0|R4GKP0\_CHICK tr|F1P552|F1P552\_CHICK tr|F1NIQ9|F1NIQ9\_CHICK tr|H9KYS6|H9KYS6\_CHICK tr|F1NLL5|F1NLL5\_CHICK tr|F1NX52|F1NX52\_CHICK tr|Q5ZIV8|Q5ZIV8\_CHICK tr|F1NJJ2|F1NJJ2\_CHICK tr|Q90WK2|Q90WK2\_CHICK tr|F1NUH7|F1NUH7\_CHICK tr|Q90WK1|Q90WK1\_CHICK tr|Q90WK3|Q90WK3\_CHICK tr|E1BYG3|E1BYG3\_CHICK tr|Q8AW01|Q8AW01\_CHICK tr|F1P1R6|F1P1R6\_CHICK tr|F1NDJ9|F1NDJ9\_CHICK tr|A0A0B5KZ08|A0A0B5KZ08\_CHICK tr|A0A0B5L7F2|A0A0B5L7F2\_CHICK tr|E5RWR0|E5RWR0\_GALLA sp|P14676|PRL\_CHICK tr|Q5ZIN8|Q5ZIN8\_CHICK tr|Q9W718|Q9W718\_CHICK tr|E1BQB0|E1BQB0\_CHICK tr|F1NQZ4|F1NQZ4\_CHICK tr|Q5ZKR6|Q5ZKR6\_CHICK tr|F1NDU5|F1NDU5\_CHICK tr|E1BRE3|E1BRE3\_CHICK tr|Q9W6V3|Q9W6V3\_CHICK tr|F1NGV3|F1NGV3\_CHICK tr|E1BTH4|E1BTH4\_CHICK tr|E1C2L4|E1C2L4\_CHICK tr|B5BS95|B5BS95\_CHICK tr|B5BSS4|B5BSS4\_CHICK tr|Q5F3T2|Q5F3T2\_CHICK tr|Q5ZM92|Q5ZM92\_CHICK tr|F1NTG9|F1NTG9\_CHICK tr|E1BZP1|E1BZP1\_CHICK tr|F1NPV8|F1NPV8\_CHICK tr|Q90807|Q90807\_CHICK tr|E1BTW2|E1BTW2\_CHICK tr|F1NF32|F1NF32\_CHICK tr|E1BUQ6|E1BUQ6\_CHICK sp|Q5ZIN0|ABD12\_CHICK tr|Q7T0L0|Q7T0L0\_CHICK sp|Q5ZL19|STX6\_CHICK tr|A0A0E9K094|A0A0E9K094\_CHICK tr|K4LM98|K4LM98\_CHICK tr|O73684|O73684\_CHICK tr|F1NUL6|F1NUL6\_CHICK tr|E1C2V3|E1C2V3\_CHICK tr|E1BZI7|E1BZI7\_CHICK tr|F1NI24|F1NI24\_CHICK tr|R4GIQ9|R4GIQ9\_CHICK tr|R4GH19|R4GH19\_CHICK tr|F1NZ99|F1NZ99\_CHICK tr|F1NZB2|F1NZB2\_CHICK tr|H9L101|H9L101\_CHICK tr|R4GIW3|R4GIW3\_CHICK tr|F1NFK5|F1NFK5\_CHICK tr|R4GMJ2|R4GMJ2\_CHICK tr|R4GLL2|R4GLL2\_CHICK tr|E1C7W7|E1C7W7\_CHICK tr|R4GKN1|R4GKN1\_CHICK sp|P22328|OPSD\_CHICK tr|F1P3Y2|F1P3Y2\_CHICK tr|F1P3V2|F1P3V2\_CHICK tr|F1NC07|F1NC07\_CHICK tr|I6LL52|I6LL52\_CHICK tr|R4GHA0|R4GHA0\_CHICK tr|G4V263|G4V263\_CHICK tr|Q6JAX5|Q6JAX5\_CHICK tr|F1NSL8|F1NSL8\_CHICK sp|O93603|TRFR\_CHICK tr|F1N8Q2|F1N8Q2\_CHICK tr|F1NW93|F1NW93\_CHICK tr|R4GGS0|R4GGS0\_CHICK tr|F1P0L1|F1P0L1\_CHICK tr|F1N9C4|F1N9C4\_CHICK tr|F1NHT1|F1NHT1\_CHICK tr|F1NP80|F1NP80\_CHICK tr|F1P0Z3|F1P0Z3\_CHICK tr|R4GGA0|R4GGA0\_CHICK tr|F1P5S2|F1P5S2\_CHICK sp|Q8AY65|MB211\_CHICK tr|F1NNK5|F1NNK5\_CHICK tr|E1BUB6|E1BUB6\_CHICK tr|F1NR60|F1NR60\_CHICK tr|F1NN54|F1NN54\_CHICK tr|Q2WBI1|Q2WBI1\_CHICK tr|F1NIF6|F1NIF6\_CHICK tr|F1NZ45|F1NZ45\_CHICK tr|K7VRT9|K7VRT9\_CHICK tr|E1C2K1|E1C2K1\_CHICK tr|F1CN05|F1CN05\_CHICK tr|F1CN30|F1CN30\_CHICK tr|F1NU75|F1NU75\_CHICK tr|F1NKQ8|F1NKQ8\_CHICK tr|A5JU83|A5JU83\_GALSO tr|R9PXP6|R9PXP6\_CHICK sp|E1BTE1|WEE2\_CHICK tr|R4GFJ0|R4GFJ0\_CHICK sp|P02605|MLE3\_CHICK tr|F1NE75|F1NE75\_CHICK tr|F1NKY5|F1NKY5\_CHICK sp|Q5ZMD4|TRI59\_CHICK tr|Q4U127|Q4U127\_CHICK tr|F1NZG0|F1NZG0\_CHICK sp|P02604|MLE1\_CHICK tr|F1P0R2|F1P0R2\_CHICK tr|E1BUJ9|E1BUJ9\_CHICK tr|Q5ZJR7|Q5ZJR7\_CHICK tr|E1BTX1|E1BTX1\_CHICK tr|Q5ZJK2|Q5ZJK2\_CHICK tr|Q5ZKD3|Q5ZKD3\_CHICK tr|F1P1G7|F1P1G7\_CHICK tr|F1NXC0|F1NXC0\_CHICK tr|F1NEK8|F1NEK8\_CHICK tr|A6BLM7|A6BLM7\_CHICK tr|Q5F3T5|Q5F3T5\_CHICK tr|Q6PVZ6|Q6PVZ6\_CHICK tr|B7FBV3|B7FBV3\_CHICK tr|E1BWU7|E1BWU7\_CHICK tr|E1C5S9|E1C5S9\_CHICK tr|F1P3Z3|F1P3Z3\_CHICK tr|E1BZF7|E1BZF7\_CHICK tr|F1C6T7|F1C6T7\_CHICK tr|E1C2A1|E1C2A1\_CHICK tr|F1P510|F1P510\_CHICK tr|R4GFU4|R4GFU4\_CHICK tr|R4GL18|R4GL18\_CHICK tr|F6TAB6|F6TAB6\_CHICK sp|Q90YB1|DNLI4\_CHICK tr|E1BQU9|E1BQU9\_CHICK tr|F1ND21|F1ND21\_CHICK tr|E1C9I2|E1C9I2\_CHICK tr|R4GG96|R4GG96\_CHICK tr|F1NLL6|F1NLL6\_CHICK tr|Q5ZJY2|Q5ZJY2\_CHICK tr|E1C4M9|E1C4M9\_CHICK tr|M9PNP2|M9PNP2\_CHICK tr|E1BVZ3|E1BVZ3\_CHICK tr|R4GGA8|R4GGA8\_CHICK tr|F1NBT7|F1NBT7\_CHICK tr|Q5ZJD5|Q5ZJD5\_CHICK tr|Q5ZIN4|Q5ZIN4\_CHICK tr|R4GIN3|R4GIN3\_CHICK tr|E1BQQ7|E1BQQ7\_CHICK tr|Q2P9U9|Q2P9U9\_CHICK tr|R4GGC0|R4GGC0\_CHICK tr|A0A096X8F8|A0A096X8F8\_CHICK Q3MHN2 tr|Q8JH65|Q8JH65\_CHICK tr|F1NNR1|F1NNR1\_CHICK tr|Q5ZKW4|Q5ZKW4\_CHICK tr|F1NZW3|F1NZW3\_CHICK tr|B3TZB7|B3TZB7\_CHICK tr|E1C6E8|E1C6E8\_CHICK tr|E1C3U1|E1C3U1\_CHICK tr|Q5ZLT2|Q5ZLT2\_CHICK tr|R4GJJ7|R4GJJ7\_CHICK tr|E1BZS1|E1BZS1\_CHICK tr|R4GKD7|R4GKD7\_CHICK sp|P53410|ISL2\_CHICK tr|F1P3S4|F1P3S4\_CHICK tr|F1NQ89|F1NQ89\_CHICK sp|Q9IA95|SFRP3\_CHICK tr|F1N884|F1N884\_CHICK tr|Q5F335|Q5F335\_CHICK tr|F1P4A4|F1P4A4\_CHICK tr|F1NQA3|F1NQA3\_CHICK tr|F1NMW9|F1NMW9\_CHICK tr|Q5ZMF5|Q5ZMF5\_CHICK tr|E1C8M9|E1C8M9\_CHICK tr|F1NYY8|F1NYY8\_CHICK tr|Q5ZKJ1|Q5ZKJ1\_CHICK tr|Q5ZKH7|Q5ZKH7\_CHICK tr|R4GK63|R4GK63\_CHICK tr|F1NSG9|F1NSG9\_CHICK tr|E1C409|E1C409\_CHICK tr|Q4W5Z2|Q4W5Z2\_CHICK sp|Q5ZMU6|PP4R2\_CHICK tr|F1NIW6|F1NIW6\_CHICK tr|F1NSD6|F1NSD6\_CHICK tr|Q5F403|Q5F403\_CHICK tr|Q5F3I3|Q5F3I3\_CHICK tr|R4GGL7|R4GGL7\_CHICK tr|B2X039|B2X039\_CHICK tr|E3VL98|E3VL98\_CHICK tr|B2X017|B2X017\_CHICK tr|B2X018|B2X018\_CHICK tr|B2X032|B2X032\_CHICK sp|Q90597|MX\_CHICK tr|B2X035|B2X035\_CHICK tr|B2X034|B2X034\_CHICK tr|B2X016|B2X016\_CHICK tr|B2X038|B2X038\_CHICK tr|B2X019|B2X019\_CHICK tr|B2X020|B2X020\_CHICK tr|B2X029|B2X029\_CHICK tr|B2X021|B2X021\_CHICK tr|B2X031|B2X031\_CHICK tr|A0A0A0V762|A0A0A0V762\_CHICK tr|E3VL99|E3VL99\_CHICK tr|Q5ZM97|Q5ZM97\_CHICK tr|M4MA50|M4MA50\_CHICK tr|F1NR97|F1NR97\_CHICK tr|R9PXL4|R9PXL4\_CHICK tr|F1P0H2|F1P0H2\_CHICK tr|F1NC27|F1NC27\_CHICK tr|F1NBH1|F1NBH1\_CHICK tr|R4GH22|R4GH22\_CHICK tr|F1NBG7|F1NBG7\_CHICK sp|Q8AXL1|SAT1\_CHICK P78385 tr|Q5UKY7|Q5UKY7\_CHICK tr|E1BVF0|E1BVF0\_CHICK tr|E1BRE6|E1BRE6\_CHICK tr|Q7LZ73|Q7LZ73\_CHICK tr|F1NW19|F1NW19\_CHICK tr|E1C1W0|E1C1W0\_CHICK tr|F1NM85|F1NM85\_CHICK tr|R4GJX8|R4GJX8\_CHICK tr|F1P1W4|F1P1W4\_CHICK tr|H9L2E3|H9L2E3\_CHICK sp|P02259|H5\_CHICK tr|E1C003|E1C003\_CHICK tr|F1NAJ7|F1NAJ7\_CHICK tr|E1C7Y9|E1C7Y9\_CHICK tr|B5BSC3|B5BSC3\_CHICK tr|E6N1W1|E6N1W1\_CHICK tr|E1C8I5|E1C8I5\_CHICK tr|F1NRN5|F1NRN5\_CHICK tr|F1NT07|F1NT07\_CHICK sp|P02789|TRFE\_CHICK tr|D2IE27|D2IE27\_CHICK tr|A0A089PM25|A0A089PM25\_CHICK tr|Q5ZIY8|Q5ZIY8\_CHICK tr|Q5ZMN8|Q5ZMN8\_CHICK tr|F1ND77|F1ND77\_CHICK tr|E1BZA5|E1BZA5\_CHICK tr|F1NNW4|F1NNW4\_CHICK tr|E1C4N0|E1C4N0\_CHICK sp|Q90751|BMP2\_CHICK tr|E1BUX1|E1BUX1\_CHICK tr|F1NT19|F1NT19\_CHICK tr|E1C2V7|E1C2V7\_CHICK tr|F1NSF3|F1NSF3\_CHICK tr|F1P3R8|F1P3R8\_CHICK tr|E1BXR0|E1BXR0\_CHICK tr|B2X047|B2X047\_CHICK tr|B2X030|B2X030\_CHICK tr|Q5W4S7|Q5W4S7\_CHICK tr|Q4PLA6|Q4PLA6\_CHICK tr|F1NH60|F1NH60\_CHICK sp|Q5ZMW6|MDM1\_CHICK tr|F1P1Z7|F1P1Z7\_CHICK sp|O57405|TYRP1\_CHICK tr|F1NFR5|F1NFR5\_CHICK tr|Q7T2S6|Q7T2S6\_CHICK tr|Q5ZI30|Q5ZI30\_CHICK tr|E1C6R1|E1C6R1\_CHICK tr|Q5ZLJ3|Q5ZLJ3\_CHICK tr|E1BSD1|E1BSD1\_CHICK tr|F1P3A5|F1P3A5\_CHICK tr|F1NAF5|F1NAF5\_CHICK tr|F1NIM4|F1NIM4\_CHICK tr|F1NK64|F1NK64\_CHICK tr|E1BYV2|E1BYV2\_CHICK tr|R4GHT6|R4GHT6\_CHICK tr|E1C354|E1C354\_CHICK tr|F1NYE4|F1NYE4\_CHICK tr|Q90979|Q90979\_CHICK tr|B9W050|B9W050\_CHICK tr|Q8JHV4|Q8JHV4\_CHICK tr|R4GGG4|R4GGG4\_CHICK tr|F1NKJ6|F1NKJ6\_CHICK tr|F1NH29|F1NH29\_CHICK tr|D6MLG9|D6MLG9\_CHICK tr|Q1XDY5|Q1XDY5\_CHICK tr|Q8QFM1|Q8QFM1\_CHICK tr|Q53HW8|Q53HW8\_CHICK tr|R4GK61|R4GK61\_CHICK tr|R4GKN4|R4GKN4\_CHICK tr|E1BU56|E1BU56\_CHICK sp|Q5ZIJ9|MIB2\_CHICK tr|F1NGX6|F1NGX6\_CHICK tr|F1NT98|F1NT98\_CHICK tr|E1BUM9|E1BUM9\_CHICK sp|Q5F3F5|LMBD2\_CHICK tr|E1BV17|E1BV17\_CHICK tr|F1NMA7|F1NMA7\_CHICK tr|R4GHV6|R4GHV6\_CHICK tr|F6UGL3|F6UGL3\_CHICK tr|F1NRN9|F1NRN9\_CHICK tr|Q6URH5|Q6URH5\_CHICK tr|E1C2N1|E1C2N1\_CHICK tr|F1NWG7|F1NWG7\_CHICK tr|F1NB99|F1NB99\_CHICK tr|H9L197|H9L197\_CHICK tr|R4GJ25|R4GJ25\_CHICK sp|Q98ST5|CRLD1\_CHICK tr|B2ZAJ3|B2ZAJ3\_CHICK tr|F1NZT1|F1NZT1\_CHICK tr|F1P1N3|F1P1N3\_CHICK tr|Q8SPD0|Q8SPD0\_CHICK tr|Q9PVI5|Q9PVI5\_CHICK tr|E1BQW6|E1BQW6\_CHICK tr|Q5ZIJ6|Q5ZIJ6\_CHICK tr|Q5XX12|Q5XX12\_CHICK tr|R4GH39|R4GH39\_CHICK tr|F1NE71|F1NE71\_CHICK tr|Q9PSA1|Q9PSA1\_CHICK tr|F8SZU3|F8SZU3\_9BIVA tr|D0ELJ6|D0ELJ6\_9BIVA tr|D0ELJ5|D0ELJ5\_9BIVA tr|F4YTJ7|F4YTJ7\_9BIVA tr|E1BVE1|E1BVE1\_CHICK tr|A5YM36|A5YM36\_CHICK sp|Q2YHT5|MOR1B\_CHICK sp|Q5ZJU2|KLH15\_CHICK tr|F1NNV8|F1NNV8\_CHICK tr|Q5ZM77|Q5ZM77\_CHICK tr|E1BVG9|E1BVG9\_CHICK tr|F1P5U5|F1P5U5\_CHICK tr|E1C8R1|E1C8R1\_CHICK tr|F1NGR5|F1NGR5\_CHICK tr|F1NUQ9|F1NUQ9\_CHICK tr|R4GM85|R4GM85\_CHICK tr|A0MPA7|A0MPA7\_CHICK tr|F1P4C7|F1P4C7\_CHICK tr|Q4GWL1|Q4GWL1\_GALVA tr|E5DFN3|E5DFN3\_CHICK tr|Q4GWM4|Q4GWM4\_CHICK tr|E5DEB5|E5DEB5\_CHICK tr|Q7GTU4|Q7GTU4\_CHICK tr|Q4GWR3|Q4GWR3\_GALSO tr|E5DFJ4|E5DFJ4\_CHICK tr|Q4GWJ8|Q4GWJ8\_GALLA tr|Q195H7|Q195H7\_CHICK tr|E5DEX3|E5DEX3\_CHICK sp|P18941|NU6M\_CHICK tr|Q4GWI5|Q4GWI5\_GALSO tr|E5DFU8|E5DFU8\_CHICK tr|E5DEN2|E5DEN2\_CHICK tr|Q4GWN7|Q4GWN7\_CHICK tr|Q4GWQ0|Q4GWQ0\_CHICK tr|E1BZM2|E1BZM2\_CHICK tr|Q90767|Q90767\_CHICK sp|P67869|CSK2B\_CHICK tr|R4GJK5|R4GJK5\_CHICK tr|Q09Y72|Q09Y72\_CHICK tr|Q6TL25|Q6TL25\_CHICK tr|A0ELQ1|A0ELQ1\_CHICK tr|F1N930|F1N930\_CHICK tr|Q8UVX7|Q8UVX7\_CHICK tr|Q9PU50|Q9PU50\_CHICK tr|F1P3G6|F1P3G6\_CHICK tr|E1BS83|E1BS83\_CHICK tr|R4GHK1|R4GHK1\_CHICK tr|Q5ZI98|Q5ZI98\_CHICK tr|Q8AYJ4|Q8AYJ4\_CHICK tr|R4GFD7|R4GFD7\_CHICK sp|Q9PVX0|RX2\_CHICK tr|Q788U5|Q788U5\_CHICK tr|E1BRP2|E1BRP2\_CHICK tr|F1NB19|F1NB19\_CHICK tr|F1P3T6|F1P3T6\_CHICK tr|E1BRP3|E1BRP3\_CHICK tr|R4GLZ1|R4GLZ1\_CHICK tr|R4GGH3|R4GGH3\_CHICK tr|R4GHD5|R4GHD5\_CHICK sp|P16039|NPM\_CHICK tr|F1P3S1|F1P3S1\_CHICK tr|E1BRD6|E1BRD6\_CHICK tr|F1P214|F1P214\_CHICK tr|F1NLZ7|F1NLZ7\_CHICK sp|Q5ZMJ4|GLPK5\_CHICK tr|A0ZPS0|A0ZPS0\_CHICK tr|F1NAN9|F1NAN9\_CHICK tr|R4GFU5|R4GFU5\_CHICK tr|Q5ZMQ4|Q5ZMQ4\_CHICK tr|E1C421|E1C421\_CHICK tr|Q5F3T8|Q5F3T8\_CHICK tr|F1NVE4|F1NVE4\_CHICK tr|E1BZ11|E1BZ11\_CHICK tr|A5HUJ0|A5HUJ0\_CHICK tr|F1N8M3|F1N8M3\_CHICK tr|E1BTL4|E1BTL4\_CHICK tr|R4GIQ3|R4GIQ3\_CHICK tr|Q5ZL76|Q5ZL76\_CHICK tr|B7FBY1|B7FBY1\_CHICK tr|B7FBX9|B7FBX9\_CHICK sp|P49702|ARF5\_CHICK tr|B7FC13|B7FC13\_CHICK tr|F1NWE2|F1NWE2\_CHICK tr|B7FBW7|B7FBW7\_CHICK tr|Q5F3Q3|Q5F3Q3\_CHICK tr|F1NVU8|F1NVU8\_CHICK tr|F1NUT5|F1NUT5\_CHICK tr|F1NAA1|F1NAA1\_CHICK tr|D5JGF8|D5JGF8\_CHICK tr|F1P1Q5|F1P1Q5\_CHICK tr|Q5F3Y8|Q5F3Y8\_CHICK tr|R4GJ54|R4GJ54\_CHICK tr|F1NCC5|F1NCC5\_CHICK sp|P21548|GBRG2\_CHICK sp|Q9PW71|DUS4\_CHICK tr|F1NVG2|F1NVG2\_CHICK tr|Q5F4B4|Q5F4B4\_CHICK sp|Q5ZHV7|SPRY7\_CHICK tr|E1BSU7|E1BSU7\_CHICK tr|F6QXI2|F6QXI2\_CHICK tr|E1C008|E1C008\_CHICK tr|F1NSM4|F1NSM4\_CHICK tr|F1NJ05|F1NJ05\_CHICK tr|O42421|O42421\_CHICK tr|F1NHS6|F1NHS6\_CHICK tr|E1BVK2|E1BVK2\_CHICK sp|Q8QFQ8|IL18\_CHICK tr|F1NM79|F1NM79\_CHICK tr|E1C571|E1C571\_CHICK tr|F1P452|F1P452\_CHICK tr|R4GFA3|R4GFA3\_CHICK tr|Q76EY6|Q76EY6\_CHICK tr|F1NNR2|F1NNR2\_CHICK tr|R4GF45|R4GF45\_CHICK tr|R4GGZ5|R4GGZ5\_CHICK tr|O93263|O93263\_CHICK tr|R4GLJ7|R4GLJ7\_CHICK tr|F1NJ78|F1NJ78\_CHICK tr|R4GIE0|R4GIE0\_CHICK tr|E1BY79|E1BY79\_CHICK tr|Q56IA1|Q56IA1\_CHICK tr|E1BTR2|E1BTR2\_CHICK tr|Q2XP57|Q2XP57\_CHICK tr|F1P3K1|F1P3K1\_CHICK tr|F1NUY3|F1NUY3\_CHICK tr|A9CDT6|A9CDT6\_CHICK tr|E1C2R0|E1C2R0\_CHICK sp|B7FF67|SNTAN\_CHICK tr|E1C0V0|E1C0V0\_CHICK tr|Q5ZJG4|Q5ZJG4\_CHICK sp|P50211|ISL1\_CHICK sp|P0CF65|CRBN\_CHICK tr|E1BU87|E1BU87\_CHICK tr|F1NHU5|F1NHU5\_CHICK tr|E1BXB8|E1BXB8\_CHICK tr|R4GJH3|R4GJH3\_CHICK tr|F1NWL4|F1NWL4\_CHICK tr|Q5F491|Q5F491\_CHICK tr|F1NHN3|F1NHN3\_CHICK tr|F1NIX2|F1NIX2\_CHICK tr|F1NS86|F1NS86\_CHICK tr|H9KZW6|H9KZW6\_CHICK tr|E1BVX5|E1BVX5\_CHICK tr|E1C241|E1C241\_CHICK tr|E1BRA4|E1BRA4\_CHICK tr|R4GM27|R4GM27\_CHICK tr|E1C0C9|E1C0C9\_CHICK tr|R9PXQ0|R9PXQ0\_CHICK tr|Q5ZJ47|Q5ZJ47\_CHICK tr|F1NWA8|F1NWA8\_CHICK tr|F1NVP9|F1NVP9\_CHICK tr|F1N977|F1N977\_CHICK sp|Q5F477|LDAH\_CHICK tr|R4GLX2|R4GLX2\_CHICK tr|E1C7K1|E1C7K1\_CHICK tr|E1C0C4|E1C0C4\_CHICK tr|Q5F3Z4|Q5F3Z4\_CHICK tr|Q5F463|Q5F463\_CHICK tr|E1C6C8|E1C6C8\_CHICK tr|F1NML2|F1NML2\_CHICK tr|R4GJJ2|R4GJJ2\_CHICK tr|Q5ZMG3|Q5ZMG3\_CHICK tr|B5BST7|B5BST7\_CHICK sp|Q5ZI33|KLHL7\_CHICK tr|R4GJB0|R4GJB0\_CHICK tr|Q98SS4|Q98SS4\_CHICK tr|R4GF39|R4GF39\_CHICK tr|E1C2N8|E1C2N8\_CHICK tr|F6T4L5|F6T4L5\_CHICK tr|F6SVX7|F6SVX7\_CHICK tr|F1P215|F1P215\_CHICK tr|F1NUK6|F1NUK6\_CHICK tr|F1P0Q5|F1P0Q5\_CHICK tr|F1N8N9|F1N8N9\_CHICK tr|F1NBD0|F1NBD0\_CHICK tr|F1NLC0|F1NLC0\_CHICK tr|Q07420|Q07420\_CHICK tr|Q5F3N3|Q5F3N3\_CHICK tr|F1N803|F1N803\_CHICK tr|E1BRB2|E1BRB2\_CHICK tr|F1NVI3|F1NVI3\_CHICK tr|E1BU55|E1BU55\_CHICK tr|R4GF43|R4GF43\_CHICK sp|Q5ZJC7|NOC4L\_CHICK tr|F1NF87|F1NF87\_CHICK tr|R4GLE7|R4GLE7\_CHICK tr|E1C0G3|E1C0G3\_CHICK tr|F1NBP0|F1NBP0\_CHICK tr|F1NNU7|F1NNU7\_CHICK sp|P53666|LIMK2\_CHICK tr|F1NGI3|F1NGI3\_CHICK tr|F1NJH4|F1NJH4\_CHICK tr|F1NJ49|F1NJ49\_CHICK tr|E1BW06|E1BW06\_CHICK tr|R4GFG4|R4GFG4\_CHICK tr|H9KZE6|H9KZE6\_CHICK tr|A0FK60|A0FK60\_CHICK tr|R4GGW0|R4GGW0\_CHICK tr|S0F2F3|S0F2F3\_CHICK tr|Q90Z42|Q90Z42\_CHICK tr|E1C7M7|E1C7M7\_CHICK sp|Q5F433|PRAF3\_CHICK tr|F1NBW9|F1NBW9\_CHICK tr|Q5F3E7|Q5F3E7\_CHICK tr|Q5F3T0|Q5F3T0\_CHICK tr|F1NUJ8|F1NUJ8\_CHICK tr|F1N8K3|F1N8K3\_CHICK tr|F1NF39|F1NF39\_CHICK tr|F1NNA3|F1NNA3\_CHICK tr|Q5EEJ9|Q5EEJ9\_CHICK tr|R4GI39|R4GI39\_CHICK tr|E1C2B6|E1C2B6\_CHICK tr|Q197G3|Q197G3\_CHICK tr|F1NBC2|F1NBC2\_CHICK tr|Q5F377|Q5F377\_CHICK tr|F1ND10|F1ND10\_CHICK tr|E1BVC0|E1BVC0\_CHICK tr|Q9I8A1|Q9I8A1\_CHICK tr|Q5ZMT2|Q5ZMT2\_CHICK tr|F1NP64|F1NP64\_CHICK tr|Q9I8A0|Q9I8A0\_CHICK tr|E1BYE0|E1BYE0\_CHICK tr|Q90951|Q90951\_CHICK tr|F1NDR2|F1NDR2\_CHICK sp|O42393|RAPSN\_CHICK tr|E1BWS1|E1BWS1\_CHICK tr|Q5ZL25|Q5ZL25\_CHICK tr|F1NAP1|F1NAP1\_CHICK tr|F1NHZ0|F1NHZ0\_CHICK tr|B7U502|B7U502\_CHICK tr|F1NJT7|F1NJT7\_CHICK tr|E1BVK8|E1BVK8\_CHICK tr|H9KZ23|H9KZ23\_CHICK tr|F1P4Y0|F1P4Y0\_CHICK tr|E1BYK8|E1BYK8\_CHICK tr|F1NJ84|F1NJ84\_CHICK REFSEQ:XP\_001252647 tr|F1NFI7|F1NFI7\_CHICK tr|A3F9M8|A3F9M8\_CHICK tr|I3XHQ7|I3XHQ7\_CHICK tr|I3XHQ6|I3XHQ6\_CHICK tr|E1BV79|E1BV79\_CHICK tr|Q5ZJ16|Q5ZJ16\_CHICK tr|F1P2E5|F1P2E5\_CHICK tr|E1C8X5|E1C8X5\_CHICK sp|Q5ZHV8|RMI1\_CHICK tr|E1C946|E1C946\_CHICK tr|E1C6G2|E1C6G2\_CHICK sp|Q5ZIN1|NUDC\_CHICK tr|F1NE97|F1NE97\_CHICK tr|F1NP38|F1NP38\_CHICK sp|Q5F415|TBC23\_CHICK sp|P37071|OLF5\_CHICK tr|R4GIV7|R4GIV7\_CHICK tr|E1C334|E1C334\_CHICK tr|I3QKS3|I3QKS3\_CHICK tr|E1BR03|E1BR03\_CHICK tr|F1ND59|F1ND59\_CHICK tr|A0A077KY10|A0A077KY10\_CHICK tr|Q5F497|Q5F497\_CHICK sp|Q5F3C1|DEGS1\_CHICK tr|Q98TQ9|Q98TQ9\_CHICK tr|E1C0R9|E1C0R9\_CHICK tr|A3QW65|A3QW65\_CHICK tr|E1BT54|E1BT54\_CHICK sp|P43693|GATA6\_CHICK tr|A0A088BIJ0|A0A088BIJ0\_CHICK tr|F6TYL4|F6TYL4\_CHICK tr|Q5ZM26|Q5ZM26\_CHICK tr|F1NJ93|F1NJ93\_CHICK tr|A3QW68|A3QW68\_CHICK Q58D62 tr|Q2UZR2|Q2UZR2\_CHICK sp|E1C6Q1|PCH2\_CHICK tr|F1NN63|F1NN63\_CHICK tr|F1NXE6|F1NXE6\_CHICK sp|Q98TX3|PDCD4\_CHICK Q3SZV7 tr|R4GGU9|R4GGU9\_CHICK tr|F1P4E1|F1P4E1\_CHICK tr|T2I4A2|T2I4A2\_CHICK tr|Q75XU6|Q75XU6\_CHICK tr|Q6T2D1|Q6T2D1\_CHICK tr|F1NDA0|F1NDA0\_CHICK tr|Q71H64|Q71H64\_CHICK tr|H9L140|H9L140\_CHICK tr|E1C2P7|E1C2P7\_CHICK tr|Q5ZML2|Q5ZML2\_CHICK tr|R4GL22|R4GL22\_CHICK tr|E1BZU5|E1BZU5\_CHICK tr|Q5W9C5|Q5W9C5\_CHICK tr|F1CN44|F1CN44\_CHICK tr|F1CN37|F1CN37\_CHICK tr|Q5W9C7|Q5W9C7\_CHICK tr|O46791|O46791\_CHICK tr|F1NPQ3|F1NPQ3\_CHICK sp|Q5ZJZ6|NCBP1\_CHICK tr|E1C7B6|E1C7B6\_CHICK sp|Q5ZJV7|FA60A\_CHICK tr|R4GJL8|R4GJL8\_CHICK tr|E1BY23|E1BY23\_CHICK tr|E1C4J7|E1C4J7\_CHICK tr|Q1PCF1|Q1PCF1\_CHICK tr|F1NSE5|F1NSE5\_CHICK tr|E1BR88|E1BR88\_CHICK tr|Q5ZLW0|Q5ZLW0\_CHICK tr|E1C5K1|E1C5K1\_CHICK tr|F1NT57|F1NT57\_CHICK tr|A8DU71|A8DU71\_CHICK tr|F1N9P4|F1N9P4\_CHICK tr|F1NZM1|F1NZM1\_CHICK tr|F1NU41|F1NU41\_CHICK tr|R4GHA6|R4GHA6\_CHICK tr|Q702H7|Q702H7\_CHICK tr|F1NPK8|F1NPK8\_CHICK tr|Q5ZKM6|Q5ZKM6\_CHICK tr|E1C603|E1C603\_CHICK sp|Q5ZJ97|K1143\_CHICK tr|R4GFV5|R4GFV5\_CHICK tr|E1C5F7|E1C5F7\_CHICK tr|F1NL22|F1NL22\_CHICK tr|F1NRQ2|F1NRQ2\_CHICK tr|R4GGM1|R4GGM1\_CHICK tr|E1C8U5|E1C8U5\_CHICK tr|E1C5Z0|E1C5Z0\_CHICK tr|A0A077K926|A0A077K926\_CHICK tr|Q9YH84|Q9YH84\_CHICK tr|Q9IAD0|Q9IAD0\_CHICK tr|Q702H3|Q702H3\_CHICK tr|F1NMB1|F1NMB1\_CHICK tr|Q5ZID4|Q5ZID4\_CHICK tr|F1NDX3|F1NDX3\_CHICK tr|E1C6C4|E1C6C4\_CHICK tr|F1NHQ4|F1NHQ4\_CHICK tr|F1NLG6|F1NLG6\_CHICK tr|Q5F4C8|Q5F4C8\_CHICK tr|F1NZP3|F1NZP3\_CHICK tr|R4GFA9|R4GFA9\_CHICK tr|F1NW49|F1NW49\_CHICK tr|F1P0U4|F1P0U4\_CHICK sp|Q6ZXC3|TRH\_CHICK sp|P0CB05|CEP63\_CHICK tr|F1P0Z4|F1P0Z4\_CHICK tr|F1NUN5|F1NUN5\_CHICK tr|M9PNR6|M9PNR6\_CHICK tr|B3F054|B3F054\_CHICK tr|F1NKQ1|F1NKQ1\_CHICK tr|E1C9A1|E1C9A1\_CHICK tr|E1BUF7|E1BUF7\_CHICK sp|Q5ZKS6|BECN1\_CHICK tr|E1C8M8|E1C8M8\_CHICK tr|R4GH69|R4GH69\_CHICK tr|F1NEW5|F1NEW5\_CHICK tr|R4GJ36|R4GJ36\_CHICK tr|F1NM80|F1NM80\_CHICK tr|Q90WI0|Q90WI0\_CHICK sp|Q804X6|FA9\_CHICK tr|U3PVB7|U3PVB7\_CHICK tr|F1NQJ3|F1NQJ3\_CHICK tr|Q5F436|Q5F436\_CHICK tr|F1NYL1|F1NYL1\_CHICK tr|Q5ZMB3|Q5ZMB3\_CHICK sp|Q90628|TRY2\_CHICK sp|Q90627|TRY1\_CHICK sp|Q5ZKH4|NDEL1\_CHICK tr|F1P3Q0|F1P3Q0\_CHICK tr|Q702H5|Q702H5\_CHICK tr|R4GMG4|R4GMG4\_CHICK tr|F1NHP0|F1NHP0\_CHICK tr|F1NVN6|F1NVN6\_CHICK tr|Q5F3B5|Q5F3B5\_CHICK tr|F1P1F7|F1P1F7\_CHICK tr|F1NRA0|F1NRA0\_CHICK tr|R4GFX7|R4GFX7\_CHICK tr|F1NFG8|F1NFG8\_CHICK sp|Q8QGP3|CBPZ\_CHICK tr|R4GII6|R4GII6\_CHICK sp|Q90YK5|HPSE\_CHICK tr|F1NRH7|F1NRH7\_CHICK sp|P36381|CXA8\_CHICK tr|F1NNQ8|F1NNQ8\_CHICK tr|R4GIC1|R4GIC1\_CHICK sp|Q5ZIB9|ANM7\_CHICK tr|F1ND11|F1ND11\_CHICK tr|Q5ZM02|Q5ZM02\_CHICK tr|F1NIQ6|F1NIQ6\_CHICK tr|Q5ZI60|Q5ZI60\_CHICK tr|Q5ZLS1|Q5ZLS1\_CHICK tr|R4GHA4|R4GHA4\_CHICK sp|Q9PSW9|H2B8\_CHICK tr|E1BQH4|E1BQH4\_CHICK tr|F1NK00|F1NK00\_CHICK tr|Q5ZLV0|Q5ZLV0\_CHICK tr|F1NYA3|F1NYA3\_CHICK sp|Q5ZKA2|SYIM\_CHICK tr|F1NNP2|F1NNP2\_CHICK sp|P30371|TGFB2\_CHICK tr|E1BQK1|E1BQK1\_CHICK tr|F1NBG8|F1NBG8\_CHICK tr|E1C480|E1C480\_CHICK tr|Q5F3Z6|Q5F3Z6\_CHICK tr|C6ZL71|C6ZL71\_9EUCA tr|F1NER5|F1NER5\_CHICK tr|F1NN34|F1NN34\_CHICK tr|E1BQM7|E1BQM7\_CHICK tr|Q6JLB1|Q6JLB1\_CHICK sp|Q5F3Z3|UB2V2\_CHICK sp|O13270|TBP\_CHICK tr|B0FLP2|B0FLP2\_CHICK tr|F1NNQ7|F1NNQ7\_CHICK tr|F1NN36|F1NN36\_CHICK tr|B4X7M3|B4X7M3\_CHICK sp|Q98913|OLF8\_CHICK tr|F1NT53|F1NT53\_CHICK sp|Q5ZMB7|PNRC2\_CHICK tr|Q5ZL08|Q5ZL08\_CHICK tr|E1C9F9|E1C9F9\_CHICK tr|R4GM26|R4GM26\_CHICK tr|E1BV01|E1BV01\_CHICK sp|P10286|CALCA\_CHICK tr|F1NB12|F1NB12\_CHICK tr|C5H3Z3|C5H3Z3\_CHICK tr|F1NHY2|F1NHY2\_CHICK tr|E1BUV1|E1BUV1\_CHICK sp|Q5F3D7|UTP15\_CHICK tr|F1NN16|F1NN16\_CHICK sp|P79762|ZP3\_CHICK tr|K9MFB5|K9MFB5\_9BIVA tr|K9MGU4|K9MGU4\_9BIVA tr|F1NHL8|F1NHL8\_CHICK tr|E1C062|E1C062\_CHICK tr|E1C3J5|E1C3J5\_CHICK tr|H9L0G5|H9L0G5\_CHICK tr|F1N842|F1N842\_CHICK tr|F1N8S6|F1N8S6\_CHICK tr|A5HUM9|A5HUM9\_CHICK tr|R4GLA9|R4GLA9\_CHICK tr|E1BY65|E1BY65\_CHICK tr|E1C666|E1C666\_CHICK tr|E1BVV4|E1BVV4\_CHICK tr|Q8QG57|Q8QG57\_CHICK tr|E1BRY3|E1BRY3\_CHICK tr|F1NWE6|F1NWE6\_CHICK tr|Q5ZMH5|Q5ZMH5\_CHICK tr|F6R378|F6R378\_CHICK tr|E1C966|E1C966\_CHICK tr|Q5ZK12|Q5ZK12\_CHICK tr|F1NMN3|F1NMN3\_CHICK tr|F1NN55|F1NN55\_CHICK tr|F1NGG4|F1NGG4\_CHICK tr|Q7ZZZ2|Q7ZZZ2\_CHICK tr|F1NGK6|F1NGK6\_CHICK sp|Q5F3A1|SEC62\_CHICK tr|Q5IJ73|Q5IJ73\_CHICK tr|Q5ZI50|Q5ZI50\_CHICK tr|R4GKI4|R4GKI4\_CHICK tr|R4GFI5|R4GFI5\_CHICK sp|Q5F4A9|AR6P4\_CHICK tr|E1C5F2|E1C5F2\_CHICK tr|R4GLA0|R4GLA0\_CHICK tr|Q6F4F1|Q6F4F1\_CHICK tr|F1NTA5|F1NTA5\_CHICK tr|Q5ZKX5|Q5ZKX5\_CHICK tr|Q98SE8|Q98SE8\_CHICK tr|F1NYU8|F1NYU8\_CHICK tr|F1NLI5|F1NLI5\_CHICK tr|F1NW02|F1NW02\_CHICK tr|Q6JG52|Q6JG52\_CHICK sp|P10042|CRBA1\_CHICK tr|Q5ZJ50|Q5ZJ50\_CHICK tr|E1C4B8|E1C4B8\_CHICK tr|Q5ZKS4|Q5ZKS4\_CHICK sp|Q92010|ZBT14\_CHICK tr|Q5ZLH3|Q5ZLH3\_CHICK tr|E1C2I3|E1C2I3\_CHICK tr|R4GLM0|R4GLM0\_CHICK tr|F1NX19|F1NX19\_CHICK tr|C5HV42|C5HV42\_CHICK tr|F1NE77|F1NE77\_CHICK tr|F1NQU1|F1NQU1\_CHICK tr|F1NDK6|F1NDK6\_CHICK tr|R4GIZ3|R4GIZ3\_CHICK tr|F1ND85|F1ND85\_CHICK tr|B0LVF9|B0LVF9\_CHICK tr|F1NVY7|F1NVY7\_CHICK tr|R4GIT8|R4GIT8\_CHICK tr|Q5ZIK3|Q5ZIK3\_CHICK tr|Q1G1I6|Q1G1I6\_CHICK tr|H9L076|H9L076\_CHICK tr|F1NH25|F1NH25\_CHICK tr|H9KZZ7|H9KZZ7\_CHICK tr|Q5ZHY6|Q5ZHY6\_CHICK sp|Q9W757|SOX10\_CHICK tr|F1NXA8|F1NXA8\_CHICK tr|F1NWT7|F1NWT7\_CHICK tr|F1N8K1|F1N8K1\_CHICK tr|Q8UWB9|Q8UWB9\_CHICK tr|H9L2U9|H9L2U9\_CHICK tr|Q05467|Q05467\_CHICK tr|F1NRX6|F1NRX6\_CHICK tr|R4GGX0|R4GGX0\_CHICK tr|Q9YGM2|Q9YGM2\_CHICK tr|R4GI94|R4GI94\_CHICK tr|F1NUX2|F1NUX2\_CHICK sp|Q5ZJ64|EIF3M\_CHICK sp|Q5ZLK4|GBGT1\_CHICK tr|E1BUG3|E1BUG3\_CHICK tr|Q5ZL89|Q5ZL89\_CHICK tr|F1NIC3|F1NIC3\_CHICK tr|F1NFC3|F1NFC3\_CHICK tr|C1K6P7|C1K6P7\_CHICK tr|E1C0E9|E1C0E9\_CHICK tr|E1C2N6|E1C2N6\_CHICK tr|R4GLE1|R4GLE1\_CHICK tr|F1NVF1|F1NVF1\_CHICK tr|Q5ZHP6|Q5ZHP6\_CHICK tr|F1NNB8|F1NNB8\_CHICK tr|F1NHA4|F1NHA4\_CHICK tr|H9L047|H9L047\_CHICK sp|Q5F4A3|AN32E\_CHICK tr|R4GJX7|R4GJX7\_CHICK sp|O13268|PSA7\_CHICK tr|B2G3F8|B2G3F8\_CHICK tr|A5HUL0|A5HUL0\_CHICK tr|R4GID2|R4GID2\_CHICK sp|Q5ZHX7|NB5R2\_CHICK tr|F1NVT2|F1NVT2\_CHICK tr|F1P1W2|F1P1W2\_CHICK sp|Q05876|FYN\_CHICK tr|E1C112|E1C112\_CHICK tr|F1NIX4|F1NIX4\_CHICK tr|F1NBF3|F1NBF3\_CHICK sp|Q8UVJ7|CDHR1\_CHICK tr|E1BXI2|E1BXI2\_CHICK tr|F1NKC9|F1NKC9\_CHICK tr|Q5ZLV9|Q5ZLV9\_CHICK tr|F1NSS7|F1NSS7\_CHICK tr|F1P4H8|F1P4H8\_CHICK tr|B6RCQ2|B6RCQ2\_CHICK tr|F1NZT4|F1NZT4\_CHICK sp|Q5ZJN1|RTF2\_CHICK tr|F1NU37|F1NU37\_CHICK tr|H9KZH3|H9KZH3\_CHICK tr|Q5ZIN3|Q5ZIN3\_CHICK tr|E1BT75|E1BT75\_CHICK tr|E1BXS1|E1BXS1\_CHICK tr|Q49K83|Q49K83\_CHICK tr|Q5ZKC4|Q5ZKC4\_CHICK tr|F1NJ35|F1NJ35\_CHICK tr|Q5F3J3|Q5F3J3\_CHICK tr|E1C3J7|E1C3J7\_CHICK tr|E1BUH5|E1BUH5\_CHICK tr|E1C3Q4|E1C3Q4\_CHICK tr|E1C4P4|E1C4P4\_CHICK tr|Q6B7Z7|Q6B7Z7\_CHICK tr|F1NS62|F1NS62\_CHICK tr|R4GI84|R4GI84\_CHICK tr|Q9DER0|Q9DER0\_CHICK tr|Q5ZKV3|Q5ZKV3\_CHICK tr|E1BTF9|E1BTF9\_CHICK tr|E1BTU3|E1BTU3\_CHICK tr|R4GJ33|R4GJ33\_CHICK tr|E1BZ17|E1BZ17\_CHICK tr|F1NWV1|F1NWV1\_CHICK tr|F1NM56|F1NM56\_CHICK sp|Q9DED6|BAX1B\_CHICK tr|F1NCL6|F1NCL6\_CHICK tr|F1NR44|F1NR44\_CHICK sp|Q5ZI25|TM129\_CHICK tr|E1BYG8|E1BYG8\_CHICK tr|F1NJI6|F1NJI6\_CHICK tr|Q5ZLK3|Q5ZLK3\_CHICK tr|E1C395|E1C395\_CHICK tr|A0A060PJH4|A0A060PJH4\_CHICK tr|E1BYW1|E1BYW1\_CHICK tr|F1N820|F1N820\_CHICK tr|E1BZ31|E1BZ31\_CHICK tr|F1P2P2|F1P2P2\_CHICK tr|B7FC01|B7FC01\_CHICK sp|Q5ZJ07|CCD61\_CHICK tr|F1NJN7|F1NJN7\_CHICK tr|E1C2K8|E1C2K8\_CHICK tr|F1P3I2|F1P3I2\_CHICK tr|S5ML08|S5ML08\_CHICK tr|F1NYT0|F1NYT0\_CHICK tr|O57317|O57317\_CHICK sp|Q7ZUA6|LMBR1\_CHICK tr|F1P229|F1P229\_CHICK tr|F1NMT1|F1NMT1\_CHICK tr|Q2MV09|Q2MV09\_CHICK tr|Q9PUR3|Q9PUR3\_CHICK tr|A6N9E0|A6N9E0\_CHICK tr|Q5ZHL1|Q5ZHL1\_CHICK tr|H9L0S8|H9L0S8\_CHICK sp|Q5ZJA9|L12R1\_CHICK tr|E1BRN0|E1BRN0\_CHICK tr|R4GK74|R4GK74\_CHICK tr|E1C697|E1C697\_CHICK tr|E1C047|E1C047\_CHICK sp|Q5ZMA3|RBM24\_CHICK tr|F1P5M5|F1P5M5\_CHICK tr|R4GJU7|R4GJU7\_CHICK tr|R4GHV2|R4GHV2\_CHICK tr|Q5CAS7|Q5CAS7\_CHICK tr|F1NJT9|F1NJT9\_CHICK tr|E1BZ82|E1BZ82\_CHICK tr|F1NSZ1|F1NSZ1\_CHICK tr|R4GKK5|R4GKK5\_CHICK tr|R4GHS5|R4GHS5\_CHICK tr|Q5XXX3|Q5XXX3\_CHICK sp|Q92172|TEF\_CHICK tr|E1C9G1|E1C9G1\_CHICK tr|Q9PU40|Q9PU40\_CHICK tr|F6RRW6|F6RRW6\_CHICK tr|H9KZV1|H9KZV1\_CHICK tr|Q5ZHL8|Q5ZHL8\_CHICK tr|F1N863|F1N863\_CHICK tr|F1NUB8|F1NUB8\_CHICK tr|R4GJ70|R4GJ70\_CHICK tr|Q5ZMG9|Q5ZMG9\_CHICK tr|Q5ZHT8|Q5ZHT8\_CHICK sp|Q5ZLY5|PKHF2\_CHICK tr|Q5F3C6|Q5F3C6\_CHICK tr|F1P3N6|F1P3N6\_CHICK tr|Q9IB43|Q9IB43\_CHICK tr|A9YXX2|A9YXX2\_CHICK tr|A9YXX3|A9YXX3\_CHICK tr|F1NXT2|F1NXT2\_CHICK tr|E1BUP3|E1BUP3\_CHICK tr|Q90778|Q90778\_CHICK tr|F1NUT2|F1NUT2\_CHICK tr|E1BUY4|E1BUY4\_CHICK tr|R4GK26|R4GK26\_CHICK tr|Q5F3S3|Q5F3S3\_CHICK tr|F1NHV4|F1NHV4\_CHICK tr|F1NCN0|F1NCN0\_CHICK sp|P18870|JUN\_CHICK tr|R4GL87|R4GL87\_CHICK tr|E1BXI0|E1BXI0\_CHICK tr|E1C8F5|E1C8F5\_CHICK tr|Q5F3P0|Q5F3P0\_CHICK tr|F1NKW9|F1NKW9\_CHICK tr|F1NLN3|F1NLN3\_CHICK tr|F1NYG2|F1NYG2\_CHICK tr|Q5ZIC2|Q5ZIC2\_CHICK tr|F1NAV0|F1NAV0\_CHICK tr|Q5ZLX8|Q5ZLX8\_CHICK sp|Q8JIS3|DER\_CHICK tr|R4GJ93|R4GJ93\_CHICK tr|E1BXW8|E1BXW8\_CHICK tr|R4GFD8|R4GFD8\_CHICK tr|F1NSW5|F1NSW5\_CHICK tr|F1P2I1|F1P2I1\_CHICK sp|Q6XL41|RHCG\_CHICK tr|E1C874|E1C874\_CHICK sp|Q5ZJP7|KCTD7\_CHICK tr|E1BRR4|E1BRR4\_CHICK tr|E1BU49|E1BU49\_CHICK tr|Q5ZL46|Q5ZL46\_CHICK tr|F1NET8|F1NET8\_CHICK tr|E1C0R0|E1C0R0\_CHICK sp|Q8JFQ4|RDM1\_CHICK sp|P27093|INHBB\_CHICK tr|F1NKR6|F1NKR6\_CHICK tr|E1BVH6|E1BVH6\_CHICK tr|R4GM20|R4GM20\_CHICK tr|F1NEW3|F1NEW3\_CHICK tr|R4GHM8|R4GHM8\_CHICK tr|R4GFC1|R4GFC1\_CHICK tr|E1BSL7|E1BSL7\_CHICK sp|P18080|HEM0\_CHICK tr|Q75W80|Q75W80\_CHICK sp|Q8QG61|CRY1\_CHICK tr|F1P5M6|F1P5M6\_CHICK tr|F1NMQ9|F1NMQ9\_CHICK tr|F1NVJ0|F1NVJ0\_CHICK tr|R4GGB7|R4GGB7\_CHICK tr|E1BXT0|E1BXT0\_CHICK tr|F1NUH9|F1NUH9\_CHICK tr|E1BRW8|E1BRW8\_CHICK tr|F1P1M2|F1P1M2\_CHICK sp|Q91025|VIT3\_CHICK tr|Q5ZKR2|Q5ZKR2\_CHICK tr|F1NMZ0|F1NMZ0\_CHICK sp|Q90XD2|SPEB\_CHICK sp|Q91974|IKBA\_CHICK tr|Q5ZIR7|Q5ZIR7\_CHICK sp|Q5ZJA3|CDR2\_CHICK tr|R4GH80|R4GH80\_CHICK tr|E1C109|E1C109\_CHICK tr|R4GMK6|R4GMK6\_CHICK tr|R4GJW6|R4GJW6\_CHICK tr|E1BSW1|E1BSW1\_CHICK sp|E1BYA3|PTSS2\_CHICK tr|Q5ZJD1|Q5ZJD1\_CHICK tr|F1N8Q4|F1N8Q4\_CHICK tr|F1NGI7|F1NGI7\_CHICK tr|E1BWJ9|E1BWJ9\_CHICK tr|F1NJK5|F1NJK5\_CHICK tr|E1BWH1|E1BWH1\_CHICK tr|A0A089FJ48|A0A089FJ48\_CHICK tr|U3PTD3|U3PTD3\_CHICK tr|E1C1C8|E1C1C8\_CHICK tr|A0A089FKB8|A0A089FKB8\_CHICK tr|A0A089FKW9|A0A089FKW9\_CHICK tr|Q7T048|Q7T048\_CHICK tr|F1NNW2|F1NNW2\_CHICK tr|F1NHY8|F1NHY8\_CHICK tr|R4GJB8|R4GJB8\_CHICK tr|H9L0T9|H9L0T9\_CHICK tr|F1NJG4|F1NJG4\_CHICK tr|R4GLZ8|R4GLZ8\_CHICK tr|R4GFF2|R4GFF2\_CHICK sp|Q5ZJY9|T194B\_CHICK tr|H9KZE5|H9KZE5\_CHICK tr|Q5ZL94|Q5ZL94\_CHICK tr|Q6IEC6|Q6IEC6\_CHICK tr|F1NZL5|F1NZL5\_CHICK tr|F1NUP8|F1NUP8\_CHICK tr|E1C0V2|E1C0V2\_CHICK tr|E1BSW6|E1BSW6\_CHICK tr|E1BZE7|E1BZE7\_CHICK sp|P00789|CANX\_CHICK tr|A4ZZ65|A4ZZ65\_CHICK tr|R4GIS4|R4GIS4\_CHICK tr|E1C8J9|E1C8J9\_CHICK sp|Q5ZLP8|IF2B3\_CHICK tr|R4GM88|R4GM88\_CHICK tr|Q5ZLY3|Q5ZLY3\_CHICK tr|Q5ZJ63|Q5ZJ63\_CHICK tr|Q9I9V1|Q9I9V1\_CHICK tr|F1NV16|F1NV16\_CHICK tr|Q6GVH3|Q6GVH3\_CHICK sp|Q5F380|PIGM\_CHICK tr|F1NE39|F1NE39\_CHICK tr|F1N939|F1N939\_CHICK tr|F1NIA5|F1NIA5\_CHICK tr|O12969|O12969\_CHICK tr|R4GFC2|R4GFC2\_CHICK tr|F1P0I8|F1P0I8\_CHICK tr|F1P5Q4|F1P5Q4\_CHICK tr|E1BQX1|E1BQX1\_CHICK tr|F1NHI0|F1NHI0\_CHICK tr|E1BUJ2|E1BUJ2\_CHICK tr|Q60GU0|Q60GU0\_CHICK tr|F1NLG5|F1NLG5\_CHICK tr|F1NG51|F1NG51\_CHICK tr|Z4YJB4|Z4YJB4\_CHICK tr|Q9PTB0|Q9PTB0\_CHICK sp|P33717|IGF2\_CHICK sp|Q90690|HAND2\_CHICK tr|E1BUS8|E1BUS8\_CHICK tr|E1C2Z5|E1C2Z5\_CHICK tr|F1NQ96|F1NQ96\_CHICK tr|F1NYG7|F1NYG7\_CHICK sp|Q9IA06|FZD2\_CHICK sp|Q8QFN3|TM121\_CHICK tr|F1NYC5|F1NYC5\_CHICK tr|F1NPJ4|F1NPJ4\_CHICK sp|Q9YHT1|SDHA\_CHICK sp|Q92178|CAN2\_CHICK tr|E1C7S4|E1C7S4\_CHICK tr|F1N917|F1N917\_CHICK tr|R4GKR9|R4GKR9\_CHICK tr|Q5ZMT5|Q5ZMT5\_CHICK tr|F1NI69|F1NI69\_CHICK tr|F1NIF3|F1NIF3\_CHICK tr|R4GFI3|R4GFI3\_CHICK tr|F1NWY4|F1NWY4\_CHICK tr|Q9I9V6|Q9I9V6\_CHICK tr|F1NPI4|F1NPI4\_CHICK tr|E1BXL5|E1BXL5\_CHICK tr|T1W2V5|T1W2V5\_CHICK tr|F1NSR8|F1NSR8\_CHICK tr|F6SB94|F6SB94\_CHICK tr|F1NJ17|F1NJ17\_CHICK tr|R4GKX8|R4GKX8\_CHICK tr|Q6XVN9|Q6XVN9\_CHICK tr|F1NZ28|F1NZ28\_CHICK tr|F1NG91|F1NG91\_CHICK tr|F1NYZ3|F1NYZ3\_CHICK sp|Q5ZMH6|METL9\_CHICK tr|F1NPA9|F1NPA9\_CHICK tr|Q5ZIZ3|Q5ZIZ3\_CHICK tr|Q90582|Q90582\_CHICK tr|F1NW58|F1NW58\_CHICK tr|F1P2I0|F1P2I0\_CHICK tr|Q6R1Z3|Q6R1Z3\_CHICK tr|F1C6X0|F1C6X0\_CHICK tr|Q8UUR0|Q8UUR0\_CHICK tr|E1BTH8|E1BTH8\_CHICK tr|F1P0S3|F1P0S3\_CHICK tr|F1NEH1|F1NEH1\_CHICK tr|Q5F483|Q5F483\_CHICK tr|E1C551|E1C551\_CHICK tr|E1C1Z9|E1C1Z9\_CHICK tr|H9KZ20|H9KZ20\_CHICK tr|F1P099|F1P099\_CHICK tr|F1NVA0|F1NVA0\_CHICK tr|E1BRR6|E1BRR6\_CHICK tr|H9NDQ3|H9NDQ3\_CHICK tr|E1C7G0|E1C7G0\_CHICK tr|E5DFJ0|E5DFJ0\_CHICK tr|Q5W4S3|Q5W4S3\_CHICK tr|R4GM00|R4GM00\_CHICK tr|Q5ZM58|Q5ZM58\_CHICK tr|Q5F3S6|Q5F3S6\_CHICK tr|F1NQC4|F1NQC4\_CHICK tr|Q7SYI0|Q7SYI0\_CHICK tr|F1NN51|F1NN51\_CHICK tr|Q7SYI1|Q7SYI1\_CHICK sp|P48984|TEAD4\_CHICK tr|F1NIL7|F1NIL7\_CHICK tr|F1NP09|F1NP09\_CHICK tr|F1NG79|F1NG79\_CHICK tr|R4GMA6|R4GMA6\_CHICK tr|R4GKF5|R4GKF5\_CHICK sp|Q8QFX1|RIMB2\_CHICK tr|E1C4Z9|E1C4Z9\_CHICK tr|R4GIR6|R4GIR6\_CHICK tr|Q1EGJ7|Q1EGJ7\_CHICK tr|Q5ZL99|Q5ZL99\_CHICK sp|P35431|CAV1\_CHICK tr|R4GJU4|R4GJU4\_CHICK tr|Q5ZL17|Q5ZL17\_CHICK tr|R4GI01|R4GI01\_CHICK tr|R4GIR3|R4GIR3\_CHICK tr|F1NPJ5|F1NPJ5\_CHICK sp|Q9YH06|HMGB1\_CHICK tr|B7FBV6|B7FBV6\_CHICK tr|B7FBW8|B7FBW8\_CHICK tr|Q08516|Q08516\_CHICK tr|Q90847|Q90847\_CHICK tr|R4GKM9|R4GKM9\_CHICK tr|E1BR27|E1BR27\_CHICK tr|F1NTE9|F1NTE9\_CHICK tr|E1BUB3|E1BUB3\_CHICK tr|E1C722|E1C722\_CHICK sp|E1C1L6|ENTP5\_CHICK tr|E1C6E5|E1C6E5\_CHICK tr|F1NX81|F1NX81\_CHICK tr|Q98TX8|Q98TX8\_CHICK tr|F1NCB9|F1NCB9\_CHICK tr|Q98TX9|Q98TX9\_CHICK tr|Q9PU51|Q9PU51\_CHICK tr|Q98TY1|Q98TY1\_CHICK tr|Q90Z41|Q90Z41\_CHICK tr|Q98TY0|Q98TY0\_CHICK tr|Q98SR1|Q98SR1\_CHICK tr|B4YK14|B4YK14\_CHICK tr|F1NJ45|F1NJ45\_CHICK tr|B8Y8S8|B8Y8S8\_CHICK sp|O93327|H2AY\_CHICK tr|C0L7M5|C0L7M5\_CHICK tr|F1NJM2|F1NJM2\_CHICK tr|E1BSV3|E1BSV3\_CHICK tr|E1C9H1|E1C9H1\_CHICK tr|Q6WV22|Q6WV22\_CHICK sp|Q5ZJ00|EM55\_CHICK tr|F1NR24|F1NR24\_CHICK tr|E1BT93|E1BT93\_CHICK tr|F1NYF7|F1NYF7\_CHICK tr|Q5F4D0|Q5F4D0\_CHICK tr|F1NKH0|F1NKH0\_CHICK tr|F1NAR7|F1NAR7\_CHICK tr|F1NHM8|F1NHM8\_CHICK tr|Q5ZKM4|Q5ZKM4\_CHICK tr|F1NNP4|F1NNP4\_CHICK tr|B4Z8L9|B4Z8L9\_CHICK sp|P25429|BDNF\_CHICK tr|K4MNE5|K4MNE5\_CHICK tr|K4MQC6|K4MQC6\_CHICK tr|E1C3B7|E1C3B7\_CHICK tr|Q8UWH4|Q8UWH4\_CHICK tr|F1NI28|F1NI28\_CHICK tr|F1NUY6|F1NUY6\_CHICK tr|F1NRR4|F1NRR4\_CHICK tr|Q98SS5|Q98SS5\_CHICK tr|F1NJ02|F1NJ02\_CHICK tr|F1NRQ6|F1NRQ6\_CHICK tr|Q1PS65|Q1PS65\_CHICK tr|F1NNG4|F1NNG4\_CHICK tr|Q5ZLZ8|Q5ZLZ8\_CHICK tr|F1N8F0|F1N8F0\_CHICK tr|E1BZK1|E1BZK1\_CHICK tr|F1N9J6|F1N9J6\_CHICK sp|Q90665|SEM4D\_CHICK tr|R9PXN3|R9PXN3\_CHICK P28800 sp|Q90YH9|TES\_CHICK tr|E1BVG6|E1BVG6\_CHICK tr|F1NJH2|F1NJH2\_CHICK tr|R4GGC9|R4GGC9\_CHICK tr|E1C6H7|E1C6H7\_CHICK sp|P19179|PLSI\_CHICK tr|Q98TD0|Q98TD0\_CHICK tr|Q2UXM9|Q2UXM9\_CHICK tr|F1NVR4|F1NVR4\_CHICK tr|F1C6V0|F1C6V0\_CHICK sp|P23825|GATA3\_CHICK tr|F1P4D4|F1P4D4\_CHICK tr|F1NU55|F1NU55\_CHICK sp|Q01406|SRC8\_CHICK tr|F1NPU2|F1NPU2\_CHICK tr|R4GI45|R4GI45\_CHICK tr|E1BZD9|E1BZD9\_CHICK tr|E1C4Y1|E1C4Y1\_CHICK tr|F1NJC1|F1NJC1\_CHICK tr|R4GMI8|R4GMI8\_CHICK sp|E1BXS0|SFR1\_CHICK tr|R9PXN0|R9PXN0\_CHICK tr|F1NWJ0|F1NWJ0\_CHICK tr|B2G3E6|B2G3E6\_CHICK tr|B5BSR3|B5BSR3\_CHICK tr|B5BSK8|B5BSK8\_CHICK tr|Q6ZYP2|Q6ZYP2\_CHICK tr|B5BSF2|B5BSF2\_CHICK tr|B5BSM1|B5BSM1\_CHICK tr|Q9PU48|Q9PU48\_CHICK tr|E6N1W2|E6N1W2\_CHICK tr|B2G3F2|B2G3F2\_CHICK tr|B2G3F4|B2G3F4\_CHICK tr|B2G3E8|B2G3E8\_CHICK tr|B5BSD8|B5BSD8\_CHICK tr|B5BSB0|B5BSB0\_CHICK tr|B5BSS5|B5BSS5\_CHICK tr|B5BS96|B5BS96\_CHICK tr|B5BSJ4|B5BSJ4\_CHICK tr|B5BSC4|B5BSC4\_CHICK tr|E1C3P8|E1C3P8\_CHICK tr|Q5ZI56|Q5ZI56\_CHICK tr|E1C3K3|E1C3K3\_CHICK tr|E1BVN9|E1BVN9\_CHICK tr|E1BX11|E1BX11\_CHICK tr|Q7T1F6|Q7T1F6\_CHICK tr|Q27IP4|Q27IP4\_CHICK tr|R4GJC4|R4GJC4\_CHICK tr|F1NTP8|F1NTP8\_CHICK sp|Q90881|LHX9\_CHICK sp|Q9PTS8|ACHA9\_CHICK tr|F1NMQ3|F1NMQ3\_CHICK tr|Q5ZLV1|Q5ZLV1\_CHICK tr|F1NWQ4|F1NWQ4\_CHICK tr|Q7ZZV5|Q7ZZV5\_CHICK tr|E1BZ47|E1BZ47\_CHICK tr|E1C7C7|E1C7C7\_CHICK tr|Q8UWC8|Q8UWC8\_CHICK tr|R4GH59|R4GH59\_CHICK tr|Q8UVG3|Q8UVG3\_CHICK tr|E1C4A8|E1C4A8\_CHICK tr|Q5ZJ90|Q5ZJ90\_CHICK tr|E1C981|E1C981\_CHICK tr|E1BSI4|E1BSI4\_CHICK tr|Q9PTE9|Q9PTE9\_CHICK tr|F1NRP9|F1NRP9\_CHICK tr|R4GM44|R4GM44\_CHICK tr|R4GK71|R4GK71\_CHICK tr|F1N916|F1N916\_CHICK tr|F1NWP7|F1NWP7\_CHICK tr|F1NXN3|F1NXN3\_CHICK tr|E1BYV6|E1BYV6\_CHICK sp|Q9I9K9|GATM\_CHICK tr|B4X7N0|B4X7N0\_CHICK tr|F1NJ46|F1NJ46\_CHICK tr|E1BUM7|E1BUM7\_CHICK sp|Q5ZMM1|NO66\_CHICK tr|F1NIG0|F1NIG0\_CHICK tr|Q5YCC8|Q5YCC8\_CHICK tr|A4UIL8|A4UIL8\_CHICK tr|Q03171|Q03171\_CHICK sp|O93512|GFRA4\_CHICK tr|R4GLR1|R4GLR1\_CHICK tr|F1P2W1|F1P2W1\_CHICK tr|H9L1M1|H9L1M1\_CHICK tr|E1BTE4|E1BTE4\_CHICK tr|F1P447|F1P447\_CHICK tr|E1C3K0|E1C3K0\_CHICK tr|E1BU03|E1BU03\_CHICK tr|A0A0A0MQ64|A0A0A0MQ64\_CHICK tr|Q5ZIC5|Q5ZIC5\_CHICK tr|Q5ZHT3|Q5ZHT3\_CHICK tr|E1C6L3|E1C6L3\_CHICK tr|R4GF72|R4GF72\_CHICK tr|F1P4I0|F1P4I0\_CHICK tr|F1P3D7|F1P3D7\_CHICK sp|Q9W6H0|MIME\_CHICK tr|F1NF25|F1NF25\_CHICK tr|E1C7U4|E1C7U4\_CHICK tr|Q5ZK64|Q5ZK64\_CHICK tr|F1NQU6|F1NQU6\_CHICK sp|Q5ZJD8|TMM68\_CHICK sp|Q92183|SIA7A\_CHICK tr|Q8JHA6|Q8JHA6\_CHICK sp|P0C1H3|H2B1\_CHICK sp|P0C1H5|H2B7\_CHICK sp|P0C1H4|H2B5\_CHICK tr|R4GK62|R4GK62\_CHICK tr|E1C958|E1C958\_CHICK tr|R4GLZ5|R4GLZ5\_CHICK tr|F1NRM4|F1NRM4\_CHICK tr|Q5F3L8|Q5F3L8\_CHICK tr|F1NFY9|F1NFY9\_CHICK tr|F1NMS1|F1NMS1\_CHICK tr|Q5ZKE9|Q5ZKE9\_CHICK sp|Q5F457|WBP4\_CHICK tr|F1P2K6|F1P2K6\_CHICK tr|Q802G0|Q802G0\_CHICK tr|F1NX31|F1NX31\_CHICK sp|O12944|RAD54\_CHICK tr|Q5MAJ0|Q5MAJ0\_CHICK tr|U5PZE6|U5PZE6\_CHICK tr|Q156C5|Q156C5\_CHICK tr|Q6JGU2|Q6JGU2\_CHICK tr|E1C8K0|E1C8K0\_CHICK tr|F1NZ60|F1NZ60\_CHICK tr|F1NS09|F1NS09\_CHICK tr|E1C8T2|E1C8T2\_CHICK tr|E1BVT0|E1BVT0\_CHICK tr|A1X9G7|A1X9G7\_CHICK tr|Q670N5|Q670N5\_CHICK tr|E1C078|E1C078\_CHICK tr|Q5ZJZ3|Q5ZJZ3\_CHICK tr|F1NS74|F1NS74\_CHICK tr|E1C8P2|E1C8P2\_CHICK tr|Q90803|Q90803\_CHICK tr|R4GJC8|R4GJC8\_CHICK tr|E1C3Q1|E1C3Q1\_CHICK tr|Q4ADJ7|Q4ADJ7\_CHICK tr|Q4ADJ6|Q4ADJ6\_CHICK tr|E1BQC2|E1BQC2\_CHICK tr|E1C3M4|E1C3M4\_CHICK tr|F1NWN4|F1NWN4\_CHICK tr|E1BRP9|E1BRP9\_CHICK tr|Q0P0G6|Q0P0G6\_CHICK Q2KIG3 tr|F1NXG2|F1NXG2\_CHICK tr|R4GLS3|R4GLS3\_CHICK tr|O93559|O93559\_CHICK tr|A3F957|A3F957\_CHICK tr|D1M7B2|D1M7B2\_CHICK tr|A3F962|A3F962\_CHICK sp|Q90873|IFNB\_CHICK tr|A3F963|A3F963\_CHICK tr|Q5MFU9|Q5MFU9\_CHICK tr|A0A0B5L5F3|A0A0B5L5F3\_CHICK tr|Q5F3X1|Q5F3X1\_CHICK tr|F1NKR2|F1NKR2\_CHICK tr|F1NDS2|F1NDS2\_CHICK tr|Q5ZLC4|Q5ZLC4\_CHICK tr|F1NXY3|F1NXY3\_CHICK tr|F1CN28|F1CN28\_CHICK tr|E1BTC4|E1BTC4\_CHICK tr|R4GLC8|R4GLC8\_CHICK tr|R4GKU0|R4GKU0\_CHICK tr|H9CZR0|H9CZR0\_CHICK tr|H9CZQ2|H9CZQ2\_CHICK tr|F1NF48|F1NF48\_CHICK tr|Q5I1Y9|Q5I1Y9\_CHICK tr|M4MA71|M4MA71\_CHICK tr|F1NRN1|F1NRN1\_CHICK tr|R4GJU8|R4GJU8\_CHICK tr|F1NFZ5|F1NFZ5\_CHICK tr|Q5ZJQ1|Q5ZJQ1\_CHICK tr|F6SKR2|F6SKR2\_CHICK tr|F1NNI0|F1NNI0\_CHICK tr|H9L0Q6|H9L0Q6\_CHICK tr|E1C4B0|E1C4B0\_CHICK tr|E1C5C3|E1C5C3\_CHICK sp|P56673|PITX1\_CHICK tr|F1NJZ8|F1NJZ8\_CHICK tr|F1NVJ6|F1NVJ6\_CHICK tr|E1C5F1|E1C5F1\_CHICK tr|E1BZ57|E1BZ57\_CHICK tr|F1N9C2|F1N9C2\_CHICK tr|R4GIS1|R4GIS1\_CHICK sp|Q9W6V2|NEGR1\_CHICK tr|S5RDP9|S5RDP9\_CHICK tr|A0FEQ2|A0FEQ2\_CHICK tr|E1C3N0|E1C3N0\_CHICK tr|R4GG86|R4GG86\_CHICK tr|F1NP95|F1NP95\_CHICK tr|E9NPB4|E9NPB4\_CHICK tr|E1BW11|E1BW11\_CHICK tr|E1BTQ9|E1BTQ9\_CHICK tr|P79884|P79884\_CHICK tr|Q90576|Q90576\_CHICK tr|E1C7K0|E1C7K0\_CHICK tr|E1BQE8|E1BQE8\_CHICK tr|B3TZB5|B3TZB5\_CHICK tr|H9KZM5|H9KZM5\_CHICK tr|E1BUP5|E1BUP5\_CHICK tr|E1C5T7|E1C5T7\_CHICK tr|E1BUJ3|E1BUJ3\_CHICK tr|F1NWF3|F1NWF3\_CHICK tr|Q0KKP4|Q0KKP4\_CHICK tr|A9QUN7|A9QUN7\_CHICK tr|R9PXQ9|R9PXQ9\_CHICK tr|R4GKF7|R4GKF7\_CHICK tr|E1C110|E1C110\_CHICK tr|H9L2K9|H9L2K9\_CHICK sp|Q5ZHN1|CHM2A\_CHICK tr|F1NA97|F1NA97\_CHICK tr|H9KZW5|H9KZW5\_CHICK sp|Q5ZM72|FACR1\_CHICK tr|H9L1E6|H9L1E6\_CHICK tr|R4GM69|R4GM69\_CHICK tr|E1C0D9|E1C0D9\_CHICK tr|F1NRK2|F1NRK2\_CHICK sp|Q5ZKI2|DPH2\_CHICK tr|E1C6X1|E1C6X1\_CHICK tr|Q7T061|Q7T061\_CHICK tr|Q646T7|Q646T7\_CHICK tr|F1NC65|F1NC65\_CHICK tr|F1P4H9|F1P4H9\_CHICK sp|Q32TG3|EFHC2\_CHICK tr|E1BV27|E1BV27\_CHICK tr|Q5F4B6|Q5F4B6\_CHICK tr|F1NMU0|F1NMU0\_CHICK tr|R4GJL6|R4GJL6\_CHICK sp|Q02020|FIBB\_CHICK tr|E1C7E9|E1C7E9\_CHICK tr|F1NUL9|F1NUL9\_CHICK tr|F1P5B4|F1P5B4\_CHICK tr|Q5F440|Q5F440\_CHICK tr|F1NC44|F1NC44\_CHICK tr|E1C7X1|E1C7X1\_CHICK tr|Q5ZLG6|Q5ZLG6\_CHICK tr|F1NLB2|F1NLB2\_CHICK sp|P43347|TCTP\_CHICK tr|F1NHI4|F1NHI4\_CHICK sp|Q5ZLA9|TM263\_CHICK tr|H9L088|H9L088\_CHICK tr|F1NPD4|F1NPD4\_CHICK tr|F1NAC1|F1NAC1\_CHICK tr|I1V3N6|I1V3N6\_9SAUR tr|A5HUK9|A5HUK9\_CHICK tr|F1NW36|F1NW36\_CHICK sp|Q6PZ02|ATG4B\_CHICK tr|F1P3N7|F1P3N7\_CHICK tr|F1NK71|F1NK71\_CHICK tr|Q5ZJ83|Q5ZJ83\_CHICK tr|R4GIV6|R4GIV6\_CHICK tr|E1BQP0|E1BQP0\_CHICK tr|A0A023PRZ6|A0A023PRZ6\_CHICK sp|Q8JFP1|IF4A2\_CHICK tr|R9PXN1|R9PXN1\_CHICK tr|Q5ZKU7|Q5ZKU7\_CHICK tr|C4PBT4|C4PBT4\_CHICK tr|F1NP90|F1NP90\_CHICK tr|R4GIM1|R4GIM1\_CHICK tr|F1NF41|F1NF41\_CHICK tr|F1NZ09|F1NZ09\_CHICK tr|Q8UVD8|Q8UVD8\_CHICK tr|E1C678|E1C678\_CHICK tr|F1NS03|F1NS03\_CHICK sp|Q5ZLM8|F133\_CHICK sp|Q5ZJU3|ASNS\_CHICK tr|F1NWF6|F1NWF6\_CHICK tr|F1NHT9|F1NHT9\_CHICK tr|H9KYR7|H9KYR7\_CHICK tr|H9L0E3|H9L0E3\_CHICK tr|C6KIB2|C6KIB2\_CHICK tr|F1NV68|F1NV68\_CHICK tr|Q2PP41|Q2PP41\_CHICK tr|Q90642|Q90642\_CHICK tr|F1NMN1|F1NMN1\_CHICK tr|R4GG99|R4GG99\_CHICK tr|F1NV57|F1NV57\_CHICK tr|F1NIG4|F1NIG4\_CHICK tr|Q5ZHW1|Q5ZHW1\_CHICK tr|F1P1L4|F1P1L4\_CHICK tr|E1C2T2|E1C2T2\_CHICK tr|B4ZCM0|B4ZCM0\_CHICK tr|F1NLB7|F1NLB7\_CHICK tr|Q8AV15|Q8AV15\_CHICK tr|F1NKR0|F1NKR0\_CHICK tr|E1C7W9|E1C7W9\_CHICK tr|H9KYS1|H9KYS1\_CHICK tr|B4X7M7|B4X7M7\_CHICK tr|F1N946|F1N946\_CHICK tr|R4GKH2|R4GKH2\_CHICK tr|R4GH78|R4GH78\_CHICK tr|Q5ZKB3|Q5ZKB3\_CHICK tr|F1NBV5|F1NBV5\_CHICK tr|F1NT85|F1NT85\_CHICK tr|Q90926|Q90926\_CHICK tr|R4GM19|R4GM19\_CHICK tr|R4GHN8|R4GHN8\_CHICK sp|Q90972|RNF13\_CHICK tr|E1C883|E1C883\_CHICK tr|E1BZN6|E1BZN6\_CHICK tr|F1NJX8|F1NJX8\_CHICK sp|Q92058|PPBT\_CHICK tr|E1C7Y2|E1C7Y2\_CHICK tr|F1NGC8|F1NGC8\_CHICK tr|F1P1V7|F1P1V7\_CHICK tr|F1P3Y1|F1P3Y1\_CHICK tr|Q5ZK31|Q5ZK31\_CHICK tr|E1BUB7|E1BUB7\_CHICK tr|Q5ZLI9|Q5ZLI9\_CHICK tr|R4GLY9|R4GLY9\_CHICK tr|E1C3P9|E1C3P9\_CHICK tr|E1C827|E1C827\_CHICK tr|F1P4Y3|F1P4Y3\_CHICK tr|B2ZAJ6|B2ZAJ6\_CHICK tr|E1BTE2|E1BTE2\_CHICK sp|Q5ZHP8|METL2\_CHICK tr|A0M8T8|A0M8T8\_CHICK sp|Q5ZJK4|STK4\_CHICK tr|Q5ZMF2|Q5ZMF2\_CHICK tr|R4GLV2|R4GLV2\_CHICK tr|E1BQR6|E1BQR6\_CHICK tr|R4GLA5|R4GLA5\_CHICK tr|Q0MRC6|Q0MRC6\_CHICK tr|E1BYK0|E1BYK0\_CHICK tr|E1C2Q9|E1C2Q9\_CHICK tr|J9QXL2|J9QXL2\_9SAUR tr|E1C5C2|E1C5C2\_CHICK tr|E1BWU0|E1BWU0\_CHICK tr|F1P4N8|F1P4N8\_CHICK tr|E1BRK4|E1BRK4\_CHICK tr|E1C809|E1C809\_CHICK tr|Q8QFM2|Q8QFM2\_CHICK tr|F1NRM5|F1NRM5\_CHICK Q3SX28 sp|P53488|ARP2\_CHICK tr|Q5ZJR5|Q5ZJR5\_CHICK tr|F1NM88|F1NM88\_CHICK tr|F1P5V2|F1P5V2\_CHICK tr|F1NBY2|F1NBY2\_CHICK tr|Q5ZMF0|Q5ZMF0\_CHICK tr|E1C2V6|E1C2V6\_CHICK sp|P62846|RS15\_CHICK tr|F2Z4M3|F2Z4M3\_CHICK tr|R4GI83|R4GI83\_CHICK tr|E1BZ71|E1BZ71\_CHICK tr|E1BRG9|E1BRG9\_CHICK tr|E1C0M7|E1C0M7\_CHICK tr|B5BSI9|B5BSI9\_CHICK tr|A0ZXL4|A0ZXL4\_CHICK tr|F1NW54|F1NW54\_CHICK tr|F1NR18|F1NR18\_CHICK tr|F1NQR1|F1NQR1\_CHICK tr|E1C1M8|E1C1M8\_CHICK tr|F1NEX4|F1NEX4\_CHICK tr|R4GHZ6|R4GHZ6\_CHICK tr|F1N9G6|F1N9G6\_CHICK sp|P07090|CALB2\_CHICK sp|Q5F3L3|K1467\_CHICK tr|F1P2K8|F1P2K8\_CHICK tr|F1NV67|F1NV67\_CHICK tr|F1NSG8|F1NSG8\_CHICK tr|F2Z4L6|F2Z4L6\_CHICK tr|R4GK00|R4GK00\_CHICK tr|Q5F434|Q5F434\_CHICK tr|F1NUA8|F1NUA8\_CHICK tr|E1BTH1|E1BTH1\_CHICK sp|Q5ZKI0|KCC2D\_CHICK tr|E1C3I7|E1C3I7\_CHICK tr|F1NCH1|F1NCH1\_CHICK tr|B6IDG5|B6IDG5\_CHICK tr|Q804I5|Q804I5\_CHICK sp|Q5ZLG8|TAPT1\_CHICK tr|Q5ZLV8|Q5ZLV8\_CHICK tr|R4GIA8|R4GIA8\_CHICK tr|F1NWS7|F1NWS7\_CHICK tr|F1NAM6|F1NAM6\_CHICK tr|F1P211|F1P211\_CHICK tr|B3F053|B3F053\_CHICK tr|A6YIJ1|A6YIJ1\_CHICK tr|F1NWW6|F1NWW6\_CHICK tr|Q5ZJM0|Q5ZJM0\_CHICK tr|H9KZ33|H9KZ33\_CHICK sp|P62149|CALM\_CHICK tr|E1BSV8|E1BSV8\_CHICK tr|F2Z4K8|F2Z4K8\_CHICK tr|O93410|O93410\_CHICK sp|P05419|CALN\_CHICK tr|P87381|P87381\_CHICK tr|E1BU64|E1BU64\_CHICK tr|F1NEW6|F1NEW6\_CHICK tr|R4GJH1|R4GJH1\_CHICK tr|E1C101|E1C101\_CHICK sp|Q5ZLN4|FEN1\_CHICK sp|P16924|P4HA1\_CHICK tr|T1W302|T1W302\_CHICK tr|R4GGN5|R4GGN5\_CHICK tr|F1NZ77|F1NZ77\_CHICK tr|F1NUV7|F1NUV7\_CHICK tr|E1BSD0|E1BSD0\_CHICK tr|O93582|O93582\_CHICK tr|Q5ZIV6|Q5ZIV6\_CHICK tr|E1C8J6|E1C8J6\_CHICK tr|E1C167|E1C167\_CHICK tr|L7NSX3|L7NSX3\_CHICK tr|E1C839|E1C839\_CHICK tr|F1NCG3|F1NCG3\_CHICK tr|R4GFZ7|R4GFZ7\_CHICK tr|R4GGJ8|R4GGJ8\_CHICK tr|R4GLY5|R4GLY5\_CHICK tr|R4GI24|R4GI24\_CHICK tr|H9L085|H9L085\_CHICK tr|F1N9Q6|F1N9Q6\_CHICK tr|O73669|O73669\_CHICK tr|Q5F3Y5|Q5F3Y5\_CHICK tr|E1BU14|E1BU14\_CHICK tr|F1NR28|F1NR28\_CHICK tr|F1N9V4|F1N9V4\_CHICK tr|E1C5U8|E1C5U8\_CHICK tr|E1C286|E1C286\_CHICK tr|Q693S0|Q693S0\_CHICK tr|F1P4B6|F1P4B6\_CHICK tr|Q9W645|Q9W645\_CHICK tr|O93415|O93415\_CHICK tr|F1NEL6|F1NEL6\_CHICK tr|Q5F3B4|Q5F3B4\_CHICK tr|E1BXJ1|E1BXJ1\_CHICK tr|F1NEB8|F1NEB8\_CHICK tr|F1NB17|F1NB17\_CHICK sp|Q8AXU9|SH3G3\_CHICK tr|E1C050|E1C050\_CHICK tr|O93601|O93601\_CHICK tr|Q5ZJL2|Q5ZJL2\_CHICK tr|H9KZ88|H9KZ88\_CHICK sp|P50147|GNAI2\_CHICK tr|Q90942|Q90942\_CHICK tr|H9L0H8|H9L0H8\_CHICK tr|E1BS45|E1BS45\_CHICK tr|F1P5I7|F1P5I7\_CHICK tr|Q5F420|Q5F420\_CHICK tr|F1NG69|F1NG69\_CHICK tr|F1P464|F1P464\_CHICK sp|Q8AWB6|S35B1\_CHICK tr|Q2TV23|Q2TV23\_CHICK tr|E1C925|E1C925\_CHICK tr|F1NHZ7|F1NHZ7\_CHICK tr|E1BXH9|E1BXH9\_CHICK tr|E1BXJ3|E1BXJ3\_CHICK tr|Q5ZMW4|Q5ZMW4\_CHICK tr|Q4VVG4|Q4VVG4\_CHICK tr|F1NK12|F1NK12\_CHICK tr|A6NAB8|A6NAB8\_CHICK tr|B1NWL4|B1NWL4\_CHICK sp|Q5ZJV6|RCAN3\_CHICK tr|Q5ZMV0|Q5ZMV0\_CHICK tr|Q5EVY3|Q5EVY3\_CHICK tr|F1NUS3|F1NUS3\_CHICK tr|A5HUK4|A5HUK4\_CHICK tr|F6S3W4|F6S3W4\_CHICK tr|F1NP58|F1NP58\_CHICK tr|F1NN94|F1NN94\_CHICK tr|E1BVB4|E1BVB4\_CHICK tr|F1NXP2|F1NXP2\_CHICK tr|R4GHT2|R4GHT2\_CHICK tr|Q5ZMR6|Q5ZMR6\_CHICK tr|E1C7B7|E1C7B7\_CHICK tr|F1NYU7|F1NYU7\_CHICK tr|F1NVW7|F1NVW7\_CHICK tr|Q5CAQ0|Q5CAQ0\_CHICK tr|F6SXF4|F6SXF4\_CHICK tr|F1P5A5|F1P5A5\_CHICK tr|F1JYB3|F1JYB3\_CHICK tr|F1NNW9|F1NNW9\_CHICK tr|A0A0C4WMI0|A0A0C4WMI0\_CHICK tr|E1BVG0|E1BVG0\_CHICK tr|R4GJJ1|R4GJJ1\_CHICK tr|Q4GWK2|Q4GWK2\_GALLA tr|F1NMR6|F1NMR6\_CHICK tr|F1N9S0|F1N9S0\_CHICK tr|F1NVA9|F1NVA9\_CHICK sp|E1BVR9|HENMT\_CHICK tr|Q5ZHS7|Q5ZHS7\_CHICK tr|F1NPE2|F1NPE2\_CHICK tr|Q5ZLH7|Q5ZLH7\_CHICK tr|F1NZU2|F1NZU2\_CHICK tr|Q5F3C0|Q5F3C0\_CHICK tr|R4GH97|R4GH97\_CHICK sp|P23289|MYPR\_CHICK tr|F1NM63|F1NM63\_CHICK tr|F1NYG4|F1NYG4\_CHICK tr|Q9PSQ5|Q9PSQ5\_CHICK tr|R4GJV8|R4GJV8\_CHICK tr|Q800W4|Q800W4\_CHICK tr|F1NYR6|F1NYR6\_CHICK tr|E6N1V0|E6N1V0\_CHICK sp|O57391|ENOG\_CHICK tr|F1NM87|F1NM87\_CHICK tr|F1NG74|F1NG74\_CHICK tr|R4GJS3|R4GJS3\_CHICK tr|Q8JGM1|Q8JGM1\_CHICK sp|Q5ZI34|F213A\_CHICK tr|F1NV24|F1NV24\_CHICK tr|F1N9T8|F1N9T8\_CHICK tr|Q5F474|Q5F474\_CHICK tr|E1BQL4|E1BQL4\_CHICK tr|F1P474|F1P474\_CHICK tr|H9KYW5|H9KYW5\_CHICK tr|E1C244|E1C244\_CHICK tr|F1P0A1|F1P0A1\_CHICK tr|R4GM56|R4GM56\_CHICK tr|E1C6N7|E1C6N7\_CHICK tr|Q9PT89|Q9PT89\_CHICK tr|B2D2J1|B2D2J1\_CHICK tr|A0ZY65|A0ZY65\_CHICK tr|Q9W7J0|Q9W7J0\_CHICK tr|E1C8Y0|E1C8Y0\_CHICK tr|E5G6H6|E5G6H6\_CHICK tr|F1NMK3|F1NMK3\_CHICK sp|Q5ZKA6|WASH1\_CHICK sp|Q6JHU8|P3H1\_CHICK tr|E1BTG1|E1BTG1\_CHICK tr|E1BSL9|E1BSL9\_CHICK sp|Q5ZJW4|SC22B\_CHICK tr|Q9I9B8|Q9I9B8\_CHICK tr|F1ND91|F1ND91\_CHICK tr|F1NCH7|F1NCH7\_CHICK tr|E1BVA5|E1BVA5\_CHICK sp|O57337|HES1\_CHICK tr|F1NMB4|F1NMB4\_CHICK tr|E1C6P6|E1C6P6\_CHICK tr|E1C6W8|E1C6W8\_CHICK tr|R4GFU1|R4GFU1\_CHICK tr|F1NLH8|F1NLH8\_CHICK tr|E1BRW7|E1BRW7\_CHICK tr|F1NH14|F1NH14\_CHICK tr|D7UT09|D7UT09\_CHICK tr|F1NJJ8|F1NJJ8\_CHICK tr|F1NBV7|F1NBV7\_CHICK tr|Q5ZMD7|Q5ZMD7\_CHICK tr|E1C0H1|E1C0H1\_CHICK H-INV:HIT000015463 tr|E1C977|E1C977\_CHICK tr|Q6L753|Q6L753\_CHICK tr|R4GGH2|R4GGH2\_CHICK tr|F1NPS9|F1NPS9\_CHICK Q1RMK2 tr|Q90746|Q90746\_CHICK tr|F1NRH3|F1NRH3\_CHICK tr|F1NN38|F1NN38\_CHICK tr|R4GF69|R4GF69\_CHICK tr|E1BVR0|E1BVR0\_CHICK tr|Q9YHW7|Q9YHW7\_CHICK sp|Q5F408|SYCC\_CHICK tr|F1P1D9|F1P1D9\_CHICK tr|Q5F4C3|Q5F4C3\_CHICK tr|F1NY43|F1NY43\_CHICK tr|F1N951|F1N951\_CHICK tr|H9L1U6|H9L1U6\_CHICK tr|E1BYZ9|E1BYZ9\_CHICK tr|R4GL03|R4GL03\_CHICK tr|E1C195|E1C195\_CHICK tr|R4GFE7|R4GFE7\_CHICK tr|R4GI52|R4GI52\_CHICK tr|F1NLF1|F1NLF1\_CHICK tr|Q5ZMF7|Q5ZMF7\_CHICK tr|E1C2Y4|E1C2Y4\_CHICK tr|E1C7F4|E1C7F4\_CHICK tr|O93418|O93418\_CHICK tr|F1NMQ4|F1NMQ4\_CHICK tr|Q6F4D9|Q6F4D9\_CHICK tr|F1P0A2|F1P0A2\_CHICK tr|Q5ZL63|Q5ZL63\_CHICK tr|F1NKY1|F1NKY1\_CHICK tr|R4GKG9|R4GKG9\_CHICK sp|Q1T765|CENPN\_CHICK tr|E1C0W7|E1C0W7\_CHICK tr|K9JV29|K9JV29\_CHICK tr|F1P315|F1P315\_CHICK tr|Q90834|Q90834\_CHICK tr|A5HUJ3|A5HUJ3\_CHICK tr|F1NBI5|F1NBI5\_CHICK tr|E1C0U9|E1C0U9\_CHICK tr|Q7LZ14|Q7LZ14\_CHICK tr|F1NSH7|F1NSH7\_CHICK tr|F1NJL7|F1NJL7\_CHICK tr|B2ZAK5|B2ZAK5\_CHICK sp|Q9PTJ6|CTDSL\_CHICK tr|E1BTM4|E1BTM4\_CHICK tr|R4GMF9|R4GMF9\_CHICK tr|Q5W9C0|Q5W9C0\_CHICK tr|E1BQX2|E1BQX2\_CHICK tr|O93242|O93242\_CHICK tr|Q9DGI5|Q9DGI5\_CHICK tr|B4X7K1|B4X7K1\_CHICK tr|E1BYS2|E1BYS2\_CHICK tr|E1C3A9|E1C3A9\_CHICK tr|Q90968|Q90968\_CHICK tr|R4GKL4|R4GKL4\_CHICK tr|F1NCE1|F1NCE1\_CHICK tr|Q9YGW6|Q9YGW6\_CHICK tr|F1NRK3|F1NRK3\_CHICK tr|F1NX69|F1NX69\_CHICK tr|Q5ZKM9|Q5ZKM9\_CHICK tr|Q31414|Q31414\_CHICK sp|Q5ZKN2|PGRC1\_CHICK tr|O42404|O42404\_CHICK tr|Q5ZMH4|Q5ZMH4\_CHICK tr|Q5F3V4|Q5F3V4\_CHICK tr|F1NZ49|F1NZ49\_CHICK tr|F1DPP1|F1DPP1\_CHICK tr|F1P0N5|F1P0N5\_CHICK tr|D7REI9|D7REI9\_CHICK tr|Q9DDT7|Q9DDT7\_CHICK tr|R4GH87|R4GH87\_CHICK tr|Q5ZK78|Q5ZK78\_CHICK tr|Q6DV98|Q6DV98\_CHICK tr|F1NZ56|F1NZ56\_CHICK tr|H9KZ91|H9KZ91\_CHICK tr|E1C999|E1C999\_CHICK tr|A5HUJ9|A5HUJ9\_CHICK tr|E6N1V1|E6N1V1\_CHICK tr|F1C6U5|F1C6U5\_CHICK tr|F1NDA2|F1NDA2\_CHICK tr|F1P366|F1P366\_CHICK tr|E1C8M5|E1C8M5\_CHICK tr|B4X7K9|B4X7K9\_CHICK tr|R4GMG0|R4GMG0\_CHICK tr|E1BYW0|E1BYW0\_CHICK tr|Q9DEG2|Q9DEG2\_CHICK tr|F6QPB3|F6QPB3\_CHICK tr|Q5ZMF8|Q5ZMF8\_CHICK tr|Q309X7|Q309X7\_CHICK tr|F1NXG1|F1NXG1\_CHICK tr|B3GRU6|B3GRU6\_CHICK tr|F1P240|F1P240\_CHICK tr|E1BXN8|E1BXN8\_CHICK tr|F1NP04|F1NP04\_CHICK tr|F1NVJ1|F1NVJ1\_CHICK tr|R4GL95|R4GL95\_CHICK tr|E1BRG0|E1BRG0\_CHICK tr|F1P2J0|F1P2J0\_CHICK tr|Q9W7K9|Q9W7K9\_CHICK tr|Q9GIT6|Q9GIT6\_CHICK tr|Q9GIT4|Q9GIT4\_CHICK tr|Q6JGT5|Q6JGT5\_CHICK tr|Q9GIT8|Q9GIT8\_CHICK tr|E1C589|E1C589\_CHICK tr|B4X7M9|B4X7M9\_CHICK tr|R4GGJ6|R4GGJ6\_CHICK tr|Q5ZIS5|Q5ZIS5\_CHICK tr|R4GIE7|R4GIE7\_CHICK tr|E1BZH5|E1BZH5\_CHICK tr|R4GFC3|R4GFC3\_CHICK tr|D4P6H5|D4P6H5\_CHICK tr|C5HV41|C5HV41\_CHICK tr|Q8QHI6|Q8QHI6\_CHICK sp|O73790|SPB10\_CHICK tr|F1NM20|F1NM20\_CHICK tr|Q90W82|Q90W82\_CHICK tr|E1C7R6|E1C7R6\_CHICK sp|Q9IA05|FZD4\_CHICK tr|Q6X3Y9|Q6X3Y9\_CHICK tr|Q6X3Y8|Q6X3Y8\_CHICK tr|H9KZB0|H9KZB0\_CHICK tr|F1NLN8|F1NLN8\_CHICK tr|Q6LBJ7|Q6LBJ7\_CHICK tr|Q5F3Z0|Q5F3Z0\_CHICK tr|E1C962|E1C962\_CHICK tr|Q90747|Q90747\_CHICK tr|E1BQD1|E1BQD1\_CHICK tr|Q9I883|Q9I883\_CHICK tr|R4GIS6|R4GIS6\_CHICK sp|P23228|HMCS1\_CHICK sp|Q5ZLH9|CDV3\_CHICK tr|R4GKK3|R4GKK3\_CHICK tr|R4GLX4|R4GLX4\_CHICK tr|F1NNZ9|F1NNZ9\_CHICK tr|E1C007|E1C007\_CHICK tr|R4GKY7|R4GKY7\_CHICK tr|F1NHR6|F1NHR6\_CHICK sp|P37070|OLF4\_CHICK tr|Q2XT07|Q2XT07\_CHICK tr|Q2XT04|Q2XT04\_CHICK tr|Q2XT05|Q2XT05\_CHICK tr|Q2XT06|Q2XT06\_CHICK tr|F1NV99|F1NV99\_CHICK tr|F1NDF7|F1NDF7\_CHICK tr|Q90ZK8|Q90ZK8\_CHICK tr|R4GGA6|R4GGA6\_CHICK tr|Q90634|Q90634\_CHICK sp|P86345|BORE2\_CHICK tr|E1BZF2|E1BZF2\_CHICK tr|Q5ZJQ6|Q5ZJQ6\_CHICK tr|Q5ZJQ5|Q5ZJQ5\_CHICK tr|F1NVA2|F1NVA2\_CHICK tr|Q91429|Q91429\_CHICK tr|E1BQI0|E1BQI0\_CHICK tr|Q90X62|Q90X62\_CHICK tr|Q5ZJN6|Q5ZJN6\_CHICK tr|F1NLP1|F1NLP1\_CHICK tr|E1C8M6|E1C8M6\_CHICK tr|Q76F79|Q76F79\_CHICK tr|F1NQJ8|F1NQJ8\_CHICK tr|B2CNR8|B2CNR8\_CHICK tr|Q9I990|Q9I990\_CHICK tr|F1NGP5|F1NGP5\_CHICK tr|F1P4V2|F1P4V2\_CHICK tr|F1P2Y2|F1P2Y2\_CHICK tr|Q8QFM7|Q8QFM7\_CHICK tr|E1C8J4|E1C8J4\_CHICK tr|R4GIU6|R4GIU6\_CHICK tr|Q67ER3|Q67ER3\_CHICK tr|E1BT77|E1BT77\_CHICK tr|F1NWB0|F1NWB0\_CHICK tr|R4GFX2|R4GFX2\_CHICK tr|F1NYS6|F1NYS6\_CHICK tr|E1C9E3|E1C9E3\_CHICK tr|Q5ZIX5|Q5ZIX5\_CHICK tr|F1P216|F1P216\_CHICK tr|Q5ZM29|Q5ZM29\_CHICK tr|Q5ZL69|Q5ZL69\_CHICK tr|F1NW14|F1NW14\_CHICK tr|Q5F355|Q5F355\_CHICK tr|E1C5F4|E1C5F4\_CHICK tr|F1CN32|F1CN32\_CHICK tr|F1CN43|F1CN43\_CHICK tr|O46790|O46790\_CHICK tr|E1C1T4|E1C1T4\_CHICK tr|F1NM43|F1NM43\_CHICK sp|Q5ZLX5|ZRAB2\_CHICK tr|F1N827|F1N827\_CHICK tr|F1NEQ2|F1NEQ2\_CHICK tr|R4GKP1|R4GKP1\_CHICK tr|F1P0C6|F1P0C6\_CHICK tr|H9L1P3|H9L1P3\_CHICK tr|Q68BG1|Q68BG1\_CHICK tr|E1C927|E1C927\_CHICK tr|F1P4W1|F1P4W1\_CHICK tr|Q5ZIZ7|Q5ZIZ7\_CHICK tr|Q5ZJ15|Q5ZJ15\_CHICK tr|F1NAD9|F1NAD9\_CHICK tr|Q7ZZK3|Q7ZZK3\_CHICK sp|Q5ZM91|KAP0\_CHICK tr|F1NZJ3|F1NZJ3\_CHICK tr|F1P0J3|F1P0J3\_CHICK tr|R4GG61|R4GG61\_CHICK tr|E1C0E8|E1C0E8\_CHICK tr|R4GMC2|R4GMC2\_CHICK tr|F1NE86|F1NE86\_CHICK tr|F1NSH6|F1NSH6\_CHICK tr|F1NJY3|F1NJY3\_CHICK tr|E1BVS5|E1BVS5\_CHICK tr|E1BT58|E1BT58\_CHICK tr|R4GII1|R4GII1\_CHICK tr|E1C1Y0|E1C1Y0\_CHICK tr|B0ZDU2|B0ZDU2\_CHICK tr|R4GGE9|R4GGE9\_CHICK tr|E1C0F5|E1C0F5\_CHICK tr|R4GJ68|R4GJ68\_CHICK tr|F1P4B4|F1P4B4\_CHICK tr|F1P1G4|F1P1G4\_CHICK tr|R4GIA2|R4GIA2\_CHICK tr|E1BWT0|E1BWT0\_CHICK tr|R4GKG0|R4GKG0\_CHICK tr|R4GKA9|R4GKA9\_CHICK tr|F1NQY3|F1NQY3\_CHICK tr|F1NYW6|F1NYW6\_CHICK tr|B1NWL3|B1NWL3\_CHICK tr|Q90900|Q90900\_CHICK tr|Q8AYS3|Q8AYS3\_CHICK tr|Q5ZLZ5|Q5ZLZ5\_CHICK tr|F1NYZ7|F1NYZ7\_CHICK tr|E1BWV8|E1BWV8\_CHICK tr|F1NUG7|F1NUG7\_CHICK sp|Q9YHY9|OTC\_CHICK tr|R4GID7|R4GID7\_CHICK tr|H9L0S2|H9L0S2\_CHICK tr|Q90940|Q90940\_CHICK tr|F1NC81|F1NC81\_CHICK tr|E1C592|E1C592\_CHICK tr|E1C650|E1C650\_CHICK tr|P87363|P87363\_CHICK tr|F1NKZ9|F1NKZ9\_CHICK tr|H9KZN5|H9KZN5\_CHICK tr|Q90WE4|Q90WE4\_CHICK tr|A0A088DBQ4|A0A088DBQ4\_CHICK tr|E1BVH8|E1BVH8\_CHICK tr|E1C6H8|E1C6H8\_CHICK tr|R4GGX5|R4GGX5\_CHICK tr|R4GKL9|R4GKL9\_CHICK tr|R4GFQ2|R4GFQ2\_CHICK tr|H9L0P2|H9L0P2\_CHICK tr|B2ZAJ4|B2ZAJ4\_CHICK Q2YDI2 tr|F1NIL3|F1NIL3\_CHICK tr|R4GIF5|R4GIF5\_CHICK tr|F1NPC6|F1NPC6\_CHICK tr|Q58G74|Q58G74\_CHICK tr|Q50L61|Q50L61\_CHICK sp|P81475|CFBL\_CHICK tr|F1NZ14|F1NZ14\_CHICK tr|R4GHR4|R4GHR4\_CHICK sp|Q5F486|BAG5\_CHICK tr|F1NU82|F1NU82\_CHICK tr|F1NKD4|F1NKD4\_CHICK tr|A1XWZ9|A1XWZ9\_CHICK tr|F1P351|F1P351\_CHICK tr|R4GKX4|R4GKX4\_CHICK tr|R4GJW1|R4GJW1\_CHICK tr|F1NNM5|F1NNM5\_CHICK sp|Q3V6R6|CETP\_CHICK tr|F1NTM7|F1NTM7\_CHICK tr|F1P414|F1P414\_CHICK tr|F1NDK1|F1NDK1\_CHICK tr|Q90590|Q90590\_CHICK tr|E1BWM4|E1BWM4\_CHICK tr|R4GJ10|R4GJ10\_CHICK tr|Q5U9T9|Q5U9T9\_CHICK tr|F1NHB8|F1NHB8\_CHICK sp|P59101|SCX\_CHICK tr|F1NIR5|F1NIR5\_CHICK sp|Q2Z1W2|CENPU\_CHICK tr|R4GJY8|R4GJY8\_CHICK tr|F1NAD3|F1NAD3\_CHICK tr|F1NUU9|F1NUU9\_CHICK tr|H9L046|H9L046\_CHICK tr|F1N872|F1N872\_CHICK tr|E1C0G6|E1C0G6\_CHICK sp|Q5F3C8|PKHO1\_CHICK tr|R4GLD7|R4GLD7\_CHICK tr|A7E3K3|A7E3K3\_CHICK tr|Q5ZI36|Q5ZI36\_CHICK tr|Q5ZMU3|Q5ZMU3\_CHICK tr|F1NIJ6|F1NIJ6\_CHICK tr|R4GKP2|R4GKP2\_CHICK tr|M9NJI3|M9NJI3\_CHICK tr|E1C316|E1C316\_CHICK tr|F1P1G5|F1P1G5\_CHICK tr|F1NL97|F1NL97\_CHICK tr|Q5F401|Q5F401\_CHICK tr|F1P2U9|F1P2U9\_CHICK sp|Q5ZIU3|DYRK2\_CHICK tr|Q5ZJL0|Q5ZJL0\_CHICK tr|E1BZK5|E1BZK5\_CHICK tr|E1C3R1|E1C3R1\_CHICK tr|D5LHI3|D5LHI3\_CHICK tr|E1C892|E1C892\_CHICK tr|Q9YHZ0|Q9YHZ0\_CHICK sp|O93533|CXB6\_CHICK tr|H9L329|H9L329\_CHICK tr|R4GHL6|R4GHL6\_CHICK tr|Q71SF9|Q71SF9\_CHICK tr|E1C0P2|E1C0P2\_CHICK tr|H9KZ01|H9KZ01\_CHICK tr|Q9YH83|Q9YH83\_CHICK tr|E1BUU9|E1BUU9\_CHICK sp|P09484|ACHB2\_CHICK tr|H9KZ38|H9KZ38\_CHICK tr|F1NV66|F1NV66\_CHICK tr|E1BQX4|E1BQX4\_CHICK tr|E1BVJ1|E1BVJ1\_CHICK sp|Q9I8G9|RBBP7\_CHICK tr|Q5ZKX4|Q5ZKX4\_CHICK tr|F1NTA9|F1NTA9\_CHICK tr|E1C4F4|E1C4F4\_CHICK tr|F1NRN8|F1NRN8\_CHICK tr|R4GJC7|R4GJC7\_CHICK sp|P54097|TECTB\_CHICK tr|E1C1D8|E1C1D8\_CHICK tr|A7UEB0|A7UEB0\_CHICK tr|E1BVJ5|E1BVJ5\_CHICK sp|P79780|SIAL\_CHICK tr|E1BRH6|E1BRH6\_CHICK tr|F1NPS5|F1NPS5\_CHICK tr|F1NDY9|F1NDY9\_CHICK tr|F1NIP2|F1NIP2\_CHICK tr|Q5ZK20|Q5ZK20\_CHICK tr|Q02612|Q02612\_CHICK tr|E1BSX0|E1BSX0\_CHICK tr|Q98944|Q98944\_CHICK tr|Q5ZJM6|Q5ZJM6\_CHICK tr|F1NIN2|F1NIN2\_CHICK tr|R4GH13|R4GH13\_CHICK tr|F1NKW4|F1NKW4\_CHICK tr|F1P2L8|F1P2L8\_CHICK tr|Q5ZIJ5|Q5ZIJ5\_CHICK tr|E1BTY4|E1BTY4\_CHICK tr|F1NYW7|F1NYW7\_CHICK tr|R4GM28|R4GM28\_CHICK tr|E1BS68|E1BS68\_CHICK tr|E1BXF7|E1BXF7\_CHICK tr|F1NGP7|F1NGP7\_CHICK tr|E1C8T8|E1C8T8\_CHICK tr|Q5ZJS5|Q5ZJS5\_CHICK tr|E1BXD0|E1BXD0\_CHICK tr|R4GJH0|R4GJH0\_CHICK tr|E1BRR7|E1BRR7\_CHICK tr|F1NGS8|F1NGS8\_CHICK tr|R4GGN7|R4GGN7\_CHICK sp|P08938|PURP\_CHICK sp|P07354|HPLN1\_CHICK sp|P37383|RAD51\_CHICK tr|E1C4D8|E1C4D8\_CHICK tr|F1NAQ3|F1NAQ3\_CHICK tr|F1NWC1|F1NWC1\_CHICK tr|F1NLW7|F1NLW7\_CHICK tr|F1NBS6|F1NBS6\_CHICK tr|F1NR71|F1NR71\_CHICK tr|F1NZA1|F1NZA1\_CHICK sp|O93307|SIX6\_CHICK sp|Q5F3L7|T229B\_CHICK tr|E1BY17|E1BY17\_CHICK tr|E1C7H8|E1C7H8\_CHICK tr|F1P2Y0|F1P2Y0\_CHICK tr|R4GIR7|R4GIR7\_CHICK tr|F1NQP9|F1NQP9\_CHICK tr|E1BXL1|E1BXL1\_CHICK sp|Q5ZIF5|TSN12\_CHICK tr|E1C5H2|E1C5H2\_CHICK tr|H9L3M0|H9L3M0\_CHICK tr|F1NHH5|F1NHH5\_CHICK tr|H9KYX6|H9KYX6\_CHICK tr|R4GJK8|R4GJK8\_CHICK tr|Q5ZLT9|Q5ZLT9\_CHICK tr|E1C1F5|E1C1F5\_CHICK sp|Q8UVV7|LIX1\_CHICK tr|E1BU32|E1BU32\_CHICK sp|P11009|KCRS\_CHICK tr|P70069|P70069\_CHICK tr|F1N9E3|F1N9E3\_CHICK tr|Q1L1D2|Q1L1D2\_CHICK tr|B3VMQ8|B3VMQ8\_CHICK sp|Q98910|CD3E\_CHICK tr|F1NNI4|F1NNI4\_CHICK tr|F1NIL6|F1NIL6\_CHICK tr|Q6DMS3|Q6DMS3\_CHICK tr|E1BXL4|E1BXL4\_CHICK tr|E1C049|E1C049\_CHICK tr|E1C256|E1C256\_CHICK tr|R4GFR2|R4GFR2\_CHICK tr|Q49M60|Q49M60\_CHICK tr|E1BU35|E1BU35\_CHICK tr|R4GH90|R4GH90\_CHICK tr|F1NAH2|F1NAH2\_CHICK tr|Q8JHA3|Q8JHA3\_CHICK tr|Q8UVD3|Q8UVD3\_CHICK tr|E1BYE9|E1BYE9\_CHICK sp|Q9DG58|GLL3\_CHICK tr|R4GMH0|R4GMH0\_CHICK tr|G0WKJ7|G0WKJ7\_CHICK sp|P48435|SOX11\_CHICK tr|E1BT84|E1BT84\_CHICK tr|G0T5Q5|G0T5Q5\_CHICK tr|F1NZP4|F1NZP4\_CHICK tr|E1C4A1|E1C4A1\_CHICK tr|F1P4I1|F1P4I1\_CHICK tr|Q5F3N2|Q5F3N2\_CHICK tr|E1C8Q0|E1C8Q0\_CHICK tr|F1NGP4|F1NGP4\_CHICK tr|E1BWI6|E1BWI6\_CHICK tr|F1P5M2|F1P5M2\_CHICK tr|F1NUC5|F1NUC5\_CHICK tr|F1NBU2|F1NBU2\_CHICK tr|Q6SVA6|Q6SVA6\_CHICK tr|E1C7P7|E1C7P7\_CHICK tr|R4GJ64|R4GJ64\_CHICK tr|E1BSI3|E1BSI3\_CHICK tr|F1NSU6|F1NSU6\_CHICK tr|F1NHE8|F1NHE8\_CHICK tr|E1C5I1|E1C5I1\_CHICK tr|E1C848|E1C848\_CHICK tr|E1BRL9|E1BRL9\_CHICK tr|Q5ZMI0|Q5ZMI0\_CHICK tr|F1NU76|F1NU76\_CHICK tr|A1KXM6|A1KXM6\_CHICK tr|Q5ZMP8|Q5ZMP8\_CHICK tr|Q8UUJ7|Q8UUJ7\_CHICK ENSEMBL:ENSBTAP00000031900 tr|E1BS88|E1BS88\_CHICK tr|E1BY09|E1BY09\_CHICK tr|R4GFR1|R4GFR1\_CHICK sp|Q5ZJ17|RBG1L\_CHICK tr|F1P1H7|F1P1H7\_CHICK tr|R4GHT9|R4GHT9\_CHICK tr|Q5ZJH0|Q5ZJH0\_CHICK tr|F1NGM1|F1NGM1\_CHICK tr|E1C1G8|E1C1G8\_CHICK tr|F1NGC7|F1NGC7\_CHICK tr|Q5F411|Q5F411\_CHICK tr|E1C4D5|E1C4D5\_CHICK tr|R4GJC3|R4GJC3\_CHICK tr|F1NU59|F1NU59\_CHICK tr|R4GGH5|R4GGH5\_CHICK tr|F8UV75|F8UV75\_CHICK tr|R4GKE8|R4GKE8\_CHICK tr|Q5ZJF0|Q5ZJF0\_CHICK sp|P23668|LEG6\_CHICK tr|F1NK50|F1NK50\_CHICK tr|F1P225|F1P225\_CHICK tr|Q8UWG9|Q8UWG9\_CHICK tr|F1NLQ2|F1NLQ2\_CHICK tr|F1NWX5|F1NWX5\_CHICK tr|Q9W6F5|Q9W6F5\_CHICK tr|E1C206|E1C206\_CHICK sp|Q5ZJX4|RBM38\_CHICK sp|Q8JHZ8|PALD\_CHICK tr|F1NLE4|F1NLE4\_CHICK sp|Q5ZI23|GLO2\_CHICK tr|H9L0Z3|H9L0Z3\_CHICK tr|G1K303|G1K303\_CHICK tr|F1NIQ8|F1NIQ8\_CHICK tr|F1NEC1|F1NEC1\_CHICK tr|R4GHB0|R4GHB0\_CHICK tr|F1P3D2|F1P3D2\_CHICK tr|F1NQZ7|F1NQZ7\_CHICK tr|Q5F3P2|Q5F3P2\_CHICK tr|E1C8H0|E1C8H0\_CHICK tr|E1C873|E1C873\_CHICK tr|H9KYZ1|H9KYZ1\_CHICK tr|F1P3R9|F1P3R9\_CHICK sp|P15062|ETS1B\_CHICK tr|G9LQW3|G9LQW3\_CHICK tr|F1NLC9|F1NLC9\_CHICK tr|F1NDH4|F1NDH4\_CHICK tr|Q5ZIF6|Q5ZIF6\_CHICK tr|F1NYK9|F1NYK9\_CHICK tr|E1C4X1|E1C4X1\_CHICK tr|F1P0S0|F1P0S0\_CHICK tr|F1P3G3|F1P3G3\_CHICK tr|A3F964|A3F964\_CHICK sp|Q5F336|MBLC2\_CHICK tr|F1N9T0|F1N9T0\_CHICK sp|Q5ZJD7|YIPF4\_CHICK tr|F1P4P3|F1P4P3\_CHICK tr|Q5ZMB9|Q5ZMB9\_CHICK tr|F1ND46|F1ND46\_CHICK tr|R4GFL6|R4GFL6\_CHICK tr|E1C1A4|E1C1A4\_CHICK tr|F1P1J3|F1P1J3\_CHICK tr|E1C7N0|E1C7N0\_CHICK tr|R4GJ56|R4GJ56\_CHICK tr|F1NWY0|F1NWY0\_CHICK sp|P24479|S10AB\_CHICK sp|P09481|ACHA3\_CHICK tr|F1P580|F1P580\_CHICK tr|F1NN84|F1NN84\_CHICK tr|F1NYK8|F1NYK8\_CHICK tr|O93586|O93586\_CHICK tr|E1BVT6|E1BVT6\_CHICK tr|Q5ZK67|Q5ZK67\_CHICK tr|R4GJ24|R4GJ24\_CHICK tr|Q90VW0|Q90VW0\_CHICK tr|Q6ZXD0|Q6ZXD0\_CHICK tr|F1NBV6|F1NBV6\_CHICK tr|E1C0D7|E1C0D7\_CHICK P00978 tr|F1N9H3|F1N9H3\_CHICK tr|Q90836|Q90836\_CHICK sp|P09102|PDIA1\_CHICK tr|H9L279|H9L279\_CHICK tr|H9L2W4|H9L2W4\_CHICK tr|R4GH12|R4GH12\_CHICK tr|F1NDD7|F1NDD7\_CHICK sp|Q5R2I8|APCD1\_CHICK tr|Q6JHX4|Q6JHX4\_CHICK tr|F1NL88|F1NL88\_CHICK tr|F1NEB6|F1NEB6\_CHICK tr|F1NJ98|F1NJ98\_CHICK tr|F1NFM7|F1NFM7\_CHICK tr|Q90811|Q90811\_CHICK tr|S4VGQ1|S4VGQ1\_CHICK tr|Q91030|Q91030\_CHICK tr|F1NSX7|F1NSX7\_CHICK tr|F1NI95|F1NI95\_CHICK tr|E1BSU0|E1BSU0\_CHICK tr|E1BXZ1|E1BXZ1\_CHICK tr|F1N8T1|F1N8T1\_CHICK tr|R4GFX4|R4GFX4\_CHICK tr|B4X7L5|B4X7L5\_CHICK tr|B4X7K6|B4X7K6\_CHICK tr|V9SHW2|V9SHW2\_CHICK tr|B4X7J5|B4X7J5\_CHICK tr|R4GFR8|R4GFR8\_CHICK tr|F1NGX8|F1NGX8\_CHICK tr|E1C119|E1C119\_CHICK tr|Q5ZLF2|Q5ZLF2\_CHICK sp|Q8JH64|BTK\_CHICK tr|R4GF44|R4GF44\_CHICK tr|Q5ZKQ0|Q5ZKQ0\_CHICK tr|F1NJX9|F1NJX9\_CHICK tr|D3Y1H8|D3Y1H8\_CHICK sp|P05081|KAD1\_CHICK tr|R4GK96|R4GK96\_CHICK tr|F1N822|F1N822\_CHICK tr|Q5F333|Q5F333\_CHICK tr|F1NW60|F1NW60\_CHICK tr|R4GI36|R4GI36\_CHICK tr|F1NKP4|F1NKP4\_CHICK tr|F1P2M0|F1P2M0\_CHICK tr|F1P202|F1P202\_CHICK sp|P32760|PTN\_CHICK tr|F1NYN2|F1NYN2\_CHICK tr|K7QRP6|K7QRP6\_CHICK tr|F1N9S1|F1N9S1\_CHICK tr|Q52Z75|Q52Z75\_CHICK tr|E1BX10|E1BX10\_CHICK tr|R4GH60|R4GH60\_CHICK tr|E1C6M3|E1C6M3\_CHICK tr|O42348|O42348\_CHICK tr|F1P383|F1P383\_CHICK sp|Q5F4B8|S46A3\_CHICK tr|F1NFW3|F1NFW3\_CHICK tr|F1NWU6|F1NWU6\_CHICK tr|E1BZG3|E1BZG3\_CHICK tr|Q4ZJF1|Q4ZJF1\_CHICK tr|M4WA95|M4WA95\_CHICK tr|E1C2B5|E1C2B5\_CHICK tr|F1NHJ8|F1NHJ8\_CHICK tr|F1NEX8|F1NEX8\_CHICK tr|Q5ZLJ8|Q5ZLJ8\_CHICK tr|E1BYJ7|E1BYJ7\_CHICK tr|Q5ZK96|Q5ZK96\_CHICK tr|B3GS89|B3GS89\_CHICK tr|B2ZSY6|B2ZSY6\_CHICK tr|E1BYW3|E1BYW3\_CHICK tr|E1C1I7|E1C1I7\_CHICK sp|Q9PTD5|MTFR1\_CHICK tr|E1BUJ6|E1BUJ6\_CHICK tr|R4GJ61|R4GJ61\_CHICK tr|Q66VY4|Q66VY4\_CHICK tr|F1NAC5|F1NAC5\_CHICK tr|E1BQ62|E1BQ62\_CHICK tr|Q90823|Q90823\_CHICK tr|E1BYD2|E1BYD2\_CHICK tr|Q1XF95|Q1XF95\_CHICK tr|A8JYG6|A8JYG6\_CHICK tr|E1C640|E1C640\_CHICK tr|F1ND98|F1ND98\_CHICK tr|E1C1Z2|E1C1Z2\_CHICK tr|Q5ZI19|Q5ZI19\_CHICK tr|Q90910|Q90910\_CHICK tr|E1BV62|E1BV62\_CHICK tr|Q1G7H3|Q1G7H3\_CHICK tr|Q5F3S8|Q5F3S8\_CHICK tr|E1BXI4|E1BXI4\_CHICK tr|K4L906|K4L906\_CHICK tr|E1C3L2|E1C3L2\_CHICK tr|E1C179|E1C179\_CHICK tr|Q3YAU1|Q3YAU1\_CHICK tr|E1BW98|E1BW98\_CHICK tr|F1N8H4|F1N8H4\_CHICK tr|F1P1T2|F1P1T2\_CHICK tr|Q5ZLB4|Q5ZLB4\_CHICK tr|F1NSW3|F1NSW3\_CHICK tr|F1NLC1|F1NLC1\_CHICK tr|F1NHD2|F1NHD2\_CHICK tr|E1BVM8|E1BVM8\_CHICK tr|Q800U5|Q800U5\_CHICK tr|A0A0C6E596|A0A0C6E596\_CHICK tr|H9KZM9|H9KZM9\_CHICK tr|F1N9F0|F1N9F0\_CHICK tr|Q5ZJ21|Q5ZJ21\_CHICK tr|E1C0U8|E1C0U8\_CHICK tr|F1NDG1|F1NDG1\_CHICK tr|F1NNF9|F1NNF9\_CHICK tr|Q5ZKW6|Q5ZKW6\_CHICK tr|F1NC15|F1NC15\_CHICK sp|P17278|HXD4\_CHICK tr|R4GJD0|R4GJD0\_CHICK tr|Q5ZMV1|Q5ZMV1\_CHICK tr|F1NDZ1|F1NDZ1\_CHICK sp|P15979|HA1F\_CHICK tr|E1BY84|E1BY84\_CHICK tr|F1NQI7|F1NQI7\_CHICK tr|E1BV69|E1BV69\_CHICK sp|P61798|APTX\_CHICK tr|E1C9J0|E1C9J0\_CHICK tr|Q9I9G9|Q9I9G9\_CHICK tr|F1NPC4|F1NPC4\_CHICK tr|F1NW56|F1NW56\_CHICK sp|P50146|GNAI1\_CHICK tr|E1BXS2|E1BXS2\_CHICK tr|E1BZ12|E1BZ12\_CHICK tr|R4GJ95|R4GJ95\_CHICK tr|Q90638|Q90638\_CHICK tr|F1NH23|F1NH23\_CHICK tr|F1NIR7|F1NIR7\_CHICK tr|F1ND56|F1ND56\_CHICK tr|F1NSP3|F1NSP3\_CHICK tr|E1BXY7|E1BXY7\_CHICK tr|F1NQP5|F1NQP5\_CHICK tr|F1NLM2|F1NLM2\_CHICK tr|F1P3P2|F1P3P2\_CHICK tr|R4GK28|R4GK28\_CHICK tr|R4GHJ2|R4GHJ2\_CHICK sp|P50223|HMGX7\_CHICK tr|R4GG01|R4GG01\_CHICK tr|F1P2Z4|F1P2Z4\_CHICK tr|Q8JHX3|Q8JHX3\_CHICK sp|Q5ZK40|SNF5\_CHICK tr|F1NB55|F1NB55\_CHICK tr|Q9DEQ5|Q9DEQ5\_CHICK tr|F1NMK6|F1NMK6\_CHICK tr|F1NS39|F1NS39\_CHICK tr|F1NQQ2|F1NQQ2\_CHICK tr|R4GIY3|R4GIY3\_CHICK tr|F1NXL3|F1NXL3\_CHICK tr|Q5ZLM7|Q5ZLM7\_CHICK tr|Q5DWF7|Q5DWF7\_CHICK tr|Q5DWF8|Q5DWF8\_CHICK tr|Q9W6Q3|Q9W6Q3\_CHICK tr|A8QJF2|A8QJF2\_CHICK tr|R4GLT8|R4GLT8\_CHICK tr|E1BVF9|E1BVF9\_CHICK tr|Q5F3G3|Q5F3G3\_CHICK tr|F1NL86|F1NL86\_CHICK tr|Q5F325|Q5F325\_CHICK tr|F1NEC2|F1NEC2\_CHICK tr|Q4GWI6|Q4GWI6\_GALSO tr|F1P3I5|F1P3I5\_CHICK tr|F1P006|F1P006\_CHICK sp|Q5ZK05|TOLIP\_CHICK tr|F1NBJ4|F1NBJ4\_CHICK tr|R4GKE1|R4GKE1\_CHICK tr|E1C5E8|E1C5E8\_CHICK tr|E1BTL9|E1BTL9\_CHICK tr|E1BZR3|E1BZR3\_CHICK tr|Q25QX5|Q25QX5\_CHICK tr|F1NBR2|F1NBR2\_CHICK tr|Q5EVY4|Q5EVY4\_CHICK tr|F1NNA2|F1NNA2\_CHICK tr|E6N1V5|E6N1V5\_CHICK tr|F1NRM8|F1NRM8\_CHICK tr|A5HUK3|A5HUK3\_CHICK tr|R4GK37|R4GK37\_CHICK tr|Q6EE59|Q6EE59\_CHICK tr|Q5ZII1|Q5ZII1\_CHICK tr|F1NWZ4|F1NWZ4\_CHICK tr|Q5F3V6|Q5F3V6\_CHICK tr|F1P114|F1P114\_CHICK tr|E1BRJ6|E1BRJ6\_CHICK sp|P32250|LPAR6\_CHICK tr|F1NRY4|F1NRY4\_CHICK tr|R4GIZ9|R4GIZ9\_CHICK tr|F1NWF2|F1NWF2\_CHICK tr|O93281|O93281\_CHICK tr|F1NJ85|F1NJ85\_CHICK tr|Q9I9U5|Q9I9U5\_GALVA tr|R4GFV6|R4GFV6\_CHICK sp|P17678|GATA1\_CHICK tr|B2CL09|B2CL09\_CHICK sp|P17926|NFIC\_CHICK tr|Q90931|Q90931\_CHICK tr|F1P5E1|F1P5E1\_CHICK tr|R4GKH7|R4GKH7\_CHICK tr|F1NEL2|F1NEL2\_CHICK tr|Q9YIC3|Q9YIC3\_CHICK tr|F1NDC1|F1NDC1\_CHICK tr|F1P3I0|F1P3I0\_CHICK tr|E1BTX9|E1BTX9\_CHICK tr|B4X7L2|B4X7L2\_CHICK tr|E1BXB3|E1BXB3\_CHICK tr|F1NB63|F1NB63\_CHICK tr|Q3C2H5|Q3C2H5\_CHICK tr|E1C531|E1C531\_CHICK tr|E1BWR6|E1BWR6\_CHICK tr|E1BR80|E1BR80\_CHICK tr|Q3HWX1|Q3HWX1\_CHICK tr|F1P3V9|F1P3V9\_CHICK tr|F1NJ68|F1NJ68\_CHICK sp|Q5ZKX9|ERD22\_CHICK tr|F1N840|F1N840\_CHICK tr|R4GK22|R4GK22\_CHICK tr|R4GJG2|R4GJG2\_CHICK tr|R4GIK2|R4GIK2\_CHICK sp|P26932|CNN1\_CHICK tr|F1NTL7|F1NTL7\_CHICK tr|F1N854|F1N854\_CHICK tr|B0FMV4|B0FMV4\_CHICK tr|R4GJU6|R4GJU6\_CHICK sp|P20111|ACTN2\_CHICK tr|E1BR10|E1BR10\_CHICK tr|G4XJR5|G4XJR5\_CHICK tr|C4PBV2|C4PBV2\_CHICK tr|C4PBT9|C4PBT9\_CHICK tr|C4PBV3|C4PBV3\_CHICK tr|G4XJR7|G4XJR7\_CHICK tr|G4XJR3|G4XJR3\_CHICK tr|G4XJR8|G4XJR8\_CHICK tr|C4PBV4|C4PBV4\_CHICK tr|C4PBU5|C4PBU5\_GALSO tr|C4PBS8|C4PBS8\_CHICK tr|C4PBV7|C4PBV7\_CHICK tr|C4PBU7|C4PBU7\_CHICK tr|F6SU23|F6SU23\_CHICK tr|C4PBS5|C4PBS5\_CHICK tr|C4PBU6|C4PBU6\_GALSO tr|C4PBU3|C4PBU3\_GALSO tr|C4PBS9|C4PBS9\_CHICK tr|G4XJR2|G4XJR2\_CHICK tr|G4XJR1|G4XJR1\_CHICK tr|C4PBU0|C4PBU0\_CHICK tr|C4PBW0|C4PBW0\_CHICK tr|C4PBT0|C4PBT0\_CHICK tr|C4PBT8|C4PBT8\_CHICK tr|C4PBU1|C4PBU1\_GALVA tr|Q5ZKH1|Q5ZKH1\_CHICK tr|E1BX28|E1BX28\_CHICK tr|Q9XPE0|Q9XPE0\_GALVA sp|P50141|GCH1\_CHICK tr|E1BWL8|E1BWL8\_CHICK tr|F1NKL7|F1NKL7\_CHICK tr|Q5ZLF3|Q5ZLF3\_CHICK tr|E1BY01|E1BY01\_CHICK tr|I6XMC9|I6XMC9\_CHICK tr|H9KZ45|H9KZ45\_CHICK ENSEMBL:ENSBTAP00000013050 tr|F1NL40|F1NL40\_CHICK tr|E1C350|E1C350\_CHICK tr|Q5F357|Q5F357\_CHICK tr|F1P0U8|F1P0U8\_CHICK tr|F1NBK8|F1NBK8\_CHICK tr|F1NLD1|F1NLD1\_CHICK tr|R4GK88|R4GK88\_CHICK tr|E1C4C6|E1C4C6\_CHICK tr|B2X025|B2X025\_CHICK sp|Q90773|CEPU1\_CHICK tr|E1C431|E1C431\_CHICK tr|A1E345|A1E345\_CHICK tr|E1C1J0|E1C1J0\_CHICK tr|F1N9N1|F1N9N1\_CHICK tr|F1NBM3|F1NBM3\_CHICK tr|F1NN30|F1NN30\_CHICK tr|F1NWU8|F1NWU8\_CHICK tr|Q9I898|Q9I898\_CHICK tr|E1BXC9|E1BXC9\_CHICK tr|F1NDC0|F1NDC0\_CHICK sp|Q90686|CATK\_CHICK tr|Q5ZMJ2|Q5ZMJ2\_CHICK tr|F1P077|F1P077\_CHICK tr|F1P3J5|F1P3J5\_CHICK tr|Q6JAY3|Q6JAY3\_CHICK tr|F1P5E4|F1P5E4\_CHICK tr|R4GK41|R4GK41\_CHICK tr|F1N998|F1N998\_CHICK tr|R4GJX3|R4GJX3\_CHICK tr|R4GIR8|R4GIR8\_CHICK tr|E1C379|E1C379\_CHICK tr|E1BYY2|E1BYY2\_CHICK tr|M1NPU0|M1NPU0\_CHICK tr|F1NSJ2|F1NSJ2\_CHICK tr|A0A090JQG8|A0A090JQG8\_CHICK tr|E1BTY1|E1BTY1\_CHICK tr|Q5ZMI8|Q5ZMI8\_CHICK tr|R4GLM4|R4GLM4\_CHICK tr|E1C4N1|E1C4N1\_CHICK tr|E1BQW2|E1BQW2\_CHICK sp|Q5F3T9|UGDH\_CHICK tr|E1C115|E1C115\_CHICK sp|Q9IAY5|SDOS\_CHICK tr|F1NP28|F1NP28\_CHICK tr|F1NAJ2|F1NAJ2\_CHICK tr|U5LV87|U5LV87\_CHICK tr|F1NKQ5|F1NKQ5\_CHICK tr|I7C3V5|I7C3V5\_CHICK tr|G8YY05|G8YY05\_CHICK tr|C7S302|C7S302\_CHICK tr|R4GLW6|R4GLW6\_CHICK tr|F1NR96|F1NR96\_CHICK tr|F1NK46|F1NK46\_CHICK tr|F1NYK5|F1NYK5\_CHICK tr|E1C100|E1C100\_CHICK sp|Q5ZLK8|CF120\_CHICK tr|Q5ZK94|Q5ZK94\_CHICK tr|F1P3C8|F1P3C8\_CHICK tr|F1N9B4|F1N9B4\_CHICK tr|Q5F3W7|Q5F3W7\_CHICK tr|F1N8L5|F1N8L5\_CHICK tr|O13053|O13053\_CHICK tr|F1NEL5|F1NEL5\_CHICK tr|R4GHB3|R4GHB3\_CHICK tr|F1NDW3|F1NDW3\_CHICK tr|K4Q293|K4Q293\_CHICK tr|R4GKX1|R4GKX1\_CHICK tr|Q5ZHX0|Q5ZHX0\_CHICK tr|Q5F422|Q5F422\_CHICK tr|E1C3C8|E1C3C8\_CHICK tr|A0A088BHA9|A0A088BHA9\_CHICK tr|H9KZG1|H9KZG1\_CHICK tr|B8YIH2|B8YIH2\_CHICK tr|F1N823|F1N823\_CHICK tr|C0MP42|C0MP42\_CHICK tr|F1NY72|F1NY72\_CHICK tr|Q5W4T2|Q5W4T2\_CHICK tr|H9L0P6|H9L0P6\_CHICK tr|G3G8J3|G3G8J3\_CHICK tr|R4GFN4|R4GFN4\_CHICK tr|E1BWZ1|E1BWZ1\_CHICK tr|E1BZX4|E1BZX4\_CHICK tr|F1NXX3|F1NXX3\_CHICK tr|E1BTS9|E1BTS9\_CHICK tr|Q5ZKS2|Q5ZKS2\_CHICK tr|E1C0Q3|E1C0Q3\_CHICK tr|F1P3N5|F1P3N5\_CHICK tr|Q5F426|Q5F426\_CHICK tr|F1NMS8|F1NMS8\_CHICK tr|Q7T1R9|Q7T1R9\_CHICK tr|F1P0Q3|F1P0Q3\_CHICK tr|E1C1L2|E1C1L2\_CHICK tr|F1NR99|F1NR99\_CHICK tr|E1C694|E1C694\_CHICK tr|R4GJE3|R4GJE3\_CHICK tr|F1NIU6|F1NIU6\_CHICK tr|Q5F373|Q5F373\_CHICK tr|F1NF00|F1NF00\_CHICK tr|Q5F451|Q5F451\_CHICK tr|F1NV12|F1NV12\_CHICK tr|R4GI82|R4GI82\_CHICK tr|R4GM89|R4GM89\_CHICK tr|F1P193|F1P193\_CHICK tr|Q69FK2|Q69FK2\_CHICK tr|F1P319|F1P319\_CHICK tr|Q5ZHT0|Q5ZHT0\_CHICK tr|F1NGG2|F1NGG2\_CHICK tr|F1NF07|F1NF07\_CHICK tr|Q5F3C5|Q5F3C5\_CHICK tr|Q5F3S0|Q5F3S0\_CHICK tr|F1NJ94|F1NJ94\_CHICK tr|B5BST4|B5BST4\_CHICK tr|F1NIN3|F1NIN3\_CHICK tr|R4GLS2|R4GLS2\_CHICK tr|R4GLJ8|R4GLJ8\_CHICK tr|F1NTA7|F1NTA7\_CHICK tr|R4GID9|R4GID9\_CHICK tr|E1BT20|E1BT20\_CHICK tr|F1NVE2|F1NVE2\_CHICK tr|F1NIP8|F1NIP8\_CHICK tr|A0A0E4BYL6|A0A0E4BYL6\_CHICK sp|Q9PWA0|RGS17\_CHICK tr|Q5ZHK6|Q5ZHK6\_CHICK sp|Q5ZME8|SMU1\_CHICK tr|R4GMC7|R4GMC7\_CHICK tr|Q5ZL70|Q5ZL70\_CHICK tr|E1BWL9|E1BWL9\_CHICK tr|E1BSG3|E1BSG3\_CHICK tr|F1P0D2|F1P0D2\_CHICK tr|F1NAG3|F1NAG3\_CHICK tr|E1BYA9|E1BYA9\_CHICK tr|R4GF31|R4GF31\_CHICK sp|F1NW29|TYDP2\_CHICK tr|F1NWP9|F1NWP9\_CHICK tr|F1NI78|F1NI78\_CHICK tr|Q9W6A2|Q9W6A2\_CHICK tr|F1NVC1|F1NVC1\_CHICK tr|H9L1F0|H9L1F0\_CHICK tr|R4GIB8|R4GIB8\_CHICK tr|Q5ZJT4|Q5ZJT4\_CHICK sp|Q06066|YBOX1\_CHICK tr|F1NHW3|F1NHW3\_CHICK tr|Q91956|Q91956\_CHICK tr|Q5ZI09|Q5ZI09\_CHICK sp|Q5ZME2|MDHC\_CHICK Q32PJ2 tr|F1NUG8|F1NUG8\_CHICK tr|O12939|O12939\_CHICK tr|F1NGR1|F1NGR1\_CHICK tr|E1C5K7|E1C5K7\_CHICK tr|F1NQI9|F1NQI9\_CHICK tr|E1C5V1|E1C5V1\_CHICK tr|R4GJ06|R4GJ06\_CHICK tr|R4GFG6|R4GFG6\_CHICK tr|R4GLE0|R4GLE0\_CHICK tr|R4GJ80|R4GJ80\_CHICK tr|E1BY75|E1BY75\_CHICK tr|F1NFF7|F1NFF7\_CHICK tr|H9L2N7|H9L2N7\_CHICK tr|Q58S68|Q58S68\_CHICK tr|E1C737|E1C737\_CHICK tr|R4GGX8|R4GGX8\_CHICK tr|F1NZA8|F1NZA8\_CHICK tr|R4GG06|R4GG06\_CHICK tr|F1P116|F1P116\_CHICK tr|F1NTU9|F1NTU9\_CHICK tr|Q90731|Q90731\_CHICK sp|Q5ZIW7|ATG4A\_CHICK tr|E1BTT6|E1BTT6\_CHICK tr|R4GHJ5|R4GHJ5\_CHICK tr|E1BRK6|E1BRK6\_CHICK tr|E1C5T6|E1C5T6\_CHICK tr|F1NRX9|F1NRX9\_CHICK sp|P07031|ACYP2\_CHICK tr|F1P0T5|F1P0T5\_CHICK tr|A1KXM0|A1KXM0\_CHICK tr|O93468|O93468\_CHICK tr|R4GF52|R4GF52\_CHICK tr|Q5ZM50|Q5ZM50\_CHICK tr|F1NRX5|F1NRX5\_CHICK sp|Q5F3I6|TM104\_CHICK sp|Q90980|CNG3\_CHICK tr|E1C391|E1C391\_CHICK sp|Q5ZKH3|EED\_CHICK tr|Q71M47|Q71M47\_CHICK tr|F1NE52|F1NE52\_CHICK tr|Q5F337|Q5F337\_CHICK tr|F1NQW8|F1NQW8\_CHICK tr|E1C3H8|E1C3H8\_CHICK tr|A0A089FKW1|A0A089FKW1\_CHICK tr|E1C4H3|E1C4H3\_CHICK sp|Q9PTW1|FZD6\_CHICK tr|F1NFQ7|F1NFQ7\_CHICK sp|E1C3U7|LOXL2\_CHICK tr|E1C857|E1C857\_CHICK tr|A0A0A0MQ32|A0A0A0MQ32\_CHICK tr|A5HUJ5|A5HUJ5\_CHICK sp|Q5ZMC6|GSKIP\_CHICK tr|R4GKM2|R4GKM2\_CHICK tr|F1NDI1|F1NDI1\_CHICK tr|K7XM59|K7XM59\_CHICK tr|Q2NNA5|Q2NNA5\_CHICK tr|G1E6K3|G1E6K3\_CHICK tr|B5BSF4|B5BSF4\_CHICK tr|F1NF36|F1NF36\_CHICK tr|Q5F4B7|Q5F4B7\_CHICK tr|Q5ZIW3|Q5ZIW3\_CHICK tr|F1NVL5|F1NVL5\_CHICK sp|Q90965|RAB2A\_CHICK tr|R4GLE6|R4GLE6\_CHICK tr|F1NZL6|F1NZL6\_CHICK tr|Q5F435|Q5F435\_CHICK P31096 tr|R4GI29|R4GI29\_CHICK tr|R4GHZ5|R4GHZ5\_CHICK tr|F1NJN2|F1NJN2\_CHICK tr|R4GHJ6|R4GHJ6\_CHICK tr|F1P166|F1P166\_CHICK tr|F1P148|F1P148\_CHICK tr|Q5QHS1|Q5QHS1\_CHICK tr|F1NF21|F1NF21\_CHICK tr|F1NJU2|F1NJU2\_CHICK tr|W8P9B5|W8P9B5\_CHICK tr|F1NCU6|F1NCU6\_CHICK tr|E1C3C1|E1C3C1\_CHICK tr|R4GGL5|R4GGL5\_CHICK tr|Q5ZIQ5|Q5ZIQ5\_CHICK tr|F1NPM6|F1NPM6\_CHICK sp|P63247|GBLP\_CHICK tr|F1NSS8|F1NSS8\_CHICK tr|B5BSK2|B5BSK2\_CHICK tr|A5HUL8|A5HUL8\_CHICK tr|B5BSG0|B5BSG0\_CHICK sp|Q5F368|P33MX\_CHICK tr|E1BZY0|E1BZY0\_CHICK tr|B5BSA4|B5BSA4\_CHICK tr|B5BSI8|B5BSI8\_CHICK tr|F4ZCJ3|F4ZCJ3\_CHICK tr|R4GJY1|R4GJY1\_CHICK tr|R4GL85|R4GL85\_CHICK tr|R4GM10|R4GM10\_CHICK tr|Q9I9P1|Q9I9P1\_CHICK tr|E1C3W3|E1C3W3\_CHICK tr|E1BWI3|E1BWI3\_CHICK tr|H9KZ92|H9KZ92\_CHICK tr|H9L038|H9L038\_CHICK tr|Q9PTN4|Q9PTN4\_CHICK sp|Q5ZHS0|F175A\_CHICK sp|P13914|ARY2\_CHICK tr|F1NMU5|F1NMU5\_CHICK tr|Q5ZJL6|Q5ZJL6\_CHICK tr|F1NFX4|F1NFX4\_CHICK tr|R4GHF2|R4GHF2\_CHICK tr|F1NR90|F1NR90\_CHICK tr|F1NGU9|F1NGU9\_CHICK tr|R4GKW3|R4GKW3\_CHICK tr|F1NUA2|F1NUA2\_CHICK tr|P79783|P79783\_CHICK tr|Q5ZMV5|Q5ZMV5\_CHICK tr|F6TBZ4|F6TBZ4\_CHICK tr|G9BWP7|G9BWP7\_CHICK tr|F1ND53|F1ND53\_CHICK sp|Q09108|SCF\_CHICK tr|Q009U6|Q009U6\_CHICK tr|R4GLB7|R4GLB7\_CHICK tr|Q5ZJF5|Q5ZJF5\_CHICK tr|E1C8X2|E1C8X2\_CHICK Q8N1A0 tr|Q5ZMD0|Q5ZMD0\_CHICK tr|Q5ZLU7|Q5ZLU7\_CHICK tr|H9L0K3|H9L0K3\_CHICK tr|F1NB10|F1NB10\_CHICK tr|A9QM72|A9QM72\_CHICK tr|E1C1M4|E1C1M4\_CHICK tr|A1IHF8|A1IHF8\_CHICK tr|A9QM71|A9QM71\_CHICK tr|Q5F400|Q5F400\_CHICK tr|E1BQN9|E1BQN9\_CHICK tr|J9PBQ2|J9PBQ2\_CHICK sp|Q9PTQ7|DMRT1\_CHICK tr|Q5F443|Q5F443\_CHICK tr|F1P5L6|F1P5L6\_CHICK tr|Q5ZKN4|Q5ZKN4\_CHICK tr|E1C3V8|E1C3V8\_CHICK tr|Q8AY72|Q8AY72\_CHICK tr|A1XF95|A1XF95\_CHICK tr|E1C454|E1C454\_CHICK tr|F1NHW7|F1NHW7\_CHICK tr|Q6LEK3|Q6LEK3\_CHICK tr|Q5W4S2|Q5W4S2\_CHICK tr|E1C6W2|E1C6W2\_CHICK tr|Q8AXU8|Q8AXU8\_CHICK tr|Q5ZLZ6|Q5ZLZ6\_CHICK tr|F1NHW6|F1NHW6\_CHICK tr|R4GHA5|R4GHA5\_CHICK tr|F1NJ12|F1NJ12\_CHICK tr|Q5ZMK3|Q5ZMK3\_CHICK sp|Q5ZKG1|RM51\_CHICK tr|E1C8Y6|E1C8Y6\_CHICK tr|E1BVH3|E1BVH3\_CHICK tr|F1NBZ5|F1NBZ5\_CHICK tr|F1N9R9|F1N9R9\_CHICK tr|E1C7Q3|E1C7Q3\_CHICK sp|O42148|OAZ1\_CHICK tr|R4GFS2|R4GFS2\_CHICK tr|F1NID5|F1NID5\_CHICK sp|Q5ZJI9|UBAC1\_CHICK tr|R4GGS3|R4GGS3\_CHICK tr|F1NY70|F1NY70\_CHICK tr|F1P2N4|F1P2N4\_CHICK tr|E1BSI1|E1BSI1\_CHICK tr|R4GIY6|R4GIY6\_CHICK tr|R4GJY6|R4GJY6\_CHICK tr|E1C6M1|E1C6M1\_CHICK tr|F1NET7|F1NET7\_CHICK tr|Q4AEJ1|Q4AEJ1\_CHICK tr|F1P2M6|F1P2M6\_CHICK tr|Q9W677|Q9W677\_CHICK tr|Q5ZHN2|Q5ZHN2\_CHICK tr|Q5F3D9|Q5F3D9\_CHICK tr|R4GGQ1|R4GGQ1\_CHICK tr|B6V3H7|B6V3H7\_CHICK tr|A0A023PI15|A0A023PI15\_CHICK tr|F1NVE9|F1NVE9\_CHICK tr|F1P5K8|F1P5K8\_CHICK tr|H9L1I6|H9L1I6\_CHICK tr|F1P1K2|F1P1K2\_CHICK sp|Q0GGW5|STK11\_CHICK tr|F1NQU5|F1NQU5\_CHICK tr|C6ZJN3|C6ZJN3\_9EUCA tr|Q5F3Y3|Q5F3Y3\_CHICK tr|F1N8A6|F1N8A6\_CHICK sp|Q90999|TGFR2\_CHICK tr|F1NZX3|F1NZX3\_CHICK tr|F1NHT5|F1NHT5\_CHICK tr|F1NIC7|F1NIC7\_CHICK sp|Q90667|NREP\_CHICK tr|F1NAP9|F1NAP9\_CHICK sp|O57328|FZD1\_CHICK tr|F1NGQ9|F1NGQ9\_CHICK sp|O42398|CAC1S\_CHICK tr|Q5ZHZ9|Q5ZHZ9\_CHICK tr|E1BV94|E1BV94\_CHICK tr|F1NDM7|F1NDM7\_CHICK tr|F1P1F9|F1P1F9\_CHICK sp|Q5ZK63|TCP4\_CHICK tr|E1C211|E1C211\_CHICK tr|F1NPS8|F1NPS8\_CHICK tr|A0A023PTX9|A0A023PTX9\_CHICK tr|E1C8K3|E1C8K3\_CHICK tr|Q9DD46|Q9DD46\_CHICK tr|F1NF51|F1NF51\_CHICK tr|F1NHK2|F1NHK2\_CHICK tr|E1C170|E1C170\_CHICK tr|Q5F3D4|Q5F3D4\_CHICK tr|Q6JGU8|Q6JGU8\_CHICK tr|H9L2U4|H9L2U4\_CHICK tr|Q2MV15|Q2MV15\_CHICK tr|E1C261|E1C261\_CHICK tr|Q5ZI80|Q5ZI80\_CHICK tr|E1C006|E1C006\_CHICK tr|F1NH34|F1NH34\_CHICK tr|R4GJ69|R4GJ69\_CHICK tr|Q75P25|Q75P25\_CHICK tr|E1C8U0|E1C8U0\_CHICK tr|B2ZAK4|B2ZAK4\_CHICK sp|Q5ZJ37|RABEK\_CHICK tr|F1P3T0|F1P3T0\_CHICK tr|E1BYL5|E1BYL5\_CHICK tr|F1NWC9|F1NWC9\_CHICK Streptavidin tr|Q6F4D8|Q6F4D8\_CHICK tr|R4GGL0|R4GGL0\_CHICK tr|F1CN15|F1CN15\_CHICK tr|F1CN13|F1CN13\_CHICK tr|F1CN14|F1CN14\_CHICK tr|A0A089FKW4|A0A089FKW4\_CHICK tr|U3PU81|U3PU81\_CHICK tr|F1CN01|F1CN01\_CHICK tr|F1NBE8|F1NBE8\_CHICK tr|Q5ZMU7|Q5ZMU7\_CHICK tr|E1BY74|E1BY74\_CHICK tr|Q6SVA8|Q6SVA8\_CHICK tr|Q5ZJM5|Q5ZJM5\_CHICK tr|Q6UA24|Q6UA24\_CHICK tr|Q6UA26|Q6UA26\_CHICK tr|Q1XF97|Q1XF97\_CHICK tr|Q8JIY0|Q8JIY0\_CHICK tr|Q8JIX9|Q8JIX9\_CHICK tr|F1P245|F1P245\_CHICK tr|F1NWB4|F1NWB4\_CHICK tr|Q5QHS2|Q5QHS2\_CHICK tr|F1NFT8|F1NFT8\_CHICK sp|Q5ZJH5|WDR61\_CHICK tr|A6XKD6|A6XKD6\_CHICK tr|E1BSZ0|E1BSZ0\_CHICK tr|F1P2M2|F1P2M2\_CHICK tr|Q5F3P9|Q5F3P9\_CHICK tr|R4GF35|R4GF35\_CHICK tr|F1NJD9|F1NJD9\_CHICK tr|E2IPN6|E2IPN6\_CHICK sp|Q9IAL7|NCKX2\_CHICK tr|E1BSV7|E1BSV7\_CHICK tr|Q9I9V8|Q9I9V8\_CHICK tr|Q564K6|Q564K6\_CHICK tr|F1N8V9|F1N8V9\_CHICK tr|F1NLE3|F1NLE3\_CHICK tr|F1NNK8|F1NNK8\_CHICK tr|H9KZF8|H9KZF8\_CHICK tr|F1NB37|F1NB37\_CHICK sp|Q6YJI5|TRM11\_CHICK tr|D5M8S2|D5M8S2\_CHICK tr|F1NZB1|F1NZB1\_CHICK tr|F1NUF5|F1NUF5\_CHICK tr|H9KZS6|H9KZS6\_CHICK sp|P70079|KCRU\_CHICK tr|Q9I8H5|Q9I8H5\_CHICK tr|A0A088T337|A0A088T337\_CHICK sp|Q92075|SCNNA\_CHICK tr|F1NWL3|F1NWL3\_CHICK tr|F1NH06|F1NH06\_CHICK tr|F1NKV0|F1NKV0\_CHICK tr|E1C5Y4|E1C5Y4\_CHICK tr|E1BRX6|E1BRX6\_CHICK tr|Q5F330|Q5F330\_CHICK tr|D2XUT1|D2XUT1\_CHICK tr|F1NLM1|F1NLM1\_CHICK tr|B4Z858|B4Z858\_CHICK tr|A3F958|A3F958\_CHICK tr|E1C2D6|E1C2D6\_CHICK tr|F1NF89|F1NF89\_CHICK sp|P23614|BASP1\_CHICK tr|F1P3E0|F1P3E0\_CHICK tr|Q5F4C1|Q5F4C1\_CHICK tr|B6DTP6|B6DTP6\_CHICK tr|F1NHR7|F1NHR7\_CHICK tr|Q805B0|Q805B0\_CHICK sp|Q8AWB5|FUT10\_CHICK tr|F1N8U3|F1N8U3\_CHICK tr|E1C2U3|E1C2U3\_CHICK tr|F1NSM3|F1NSM3\_CHICK tr|Q49L19|Q49L19\_CHICK tr|F1NNG2|F1NNG2\_CHICK tr|F1NB78|F1NB78\_CHICK tr|Q8MHQ3|Q8MHQ3\_CHICK tr|E1C7D9|E1C7D9\_CHICK tr|F1ND03|F1ND03\_CHICK tr|Q6X3Z1|Q6X3Z1\_CHICK tr|F1NHP3|F1NHP3\_CHICK tr|F1P3R6|F1P3R6\_CHICK tr|Q5ZLB1|Q5ZLB1\_CHICK sp|Q5ZIL9|KBP\_CHICK tr|Q7T066|Q7T066\_CHICK tr|F1NFG6|F1NFG6\_CHICK tr|F1NCC7|F1NCC7\_CHICK tr|Q7T2L9|Q7T2L9\_CHICK sp|Q2LK94|COL11\_CHICK tr|R4GJT3|R4GJT3\_CHICK tr|F1NPM4|F1NPM4\_CHICK tr|E1BQI2|E1BQI2\_CHICK tr|Q9B623|Q9B623\_CHICK tr|D2XQ50|D2XQ50\_CHICK tr|R4GJ05|R4GJ05\_CHICK tr|F1NSE9|F1NSE9\_CHICK tr|F1P0C8|F1P0C8\_CHICK tr|H9KYQ6|H9KYQ6\_CHICK tr|R4GK84|R4GK84\_CHICK tr|E1C652|E1C652\_CHICK tr|F1NZ19|F1NZ19\_CHICK sp|Q05744|CATD\_CHICK tr|R4GI81|R4GI81\_CHICK tr|E1BQN3|E1BQN3\_CHICK tr|Q6PN72|Q6PN72\_CHICK tr|F1NWN6|F1NWN6\_CHICK tr|F1P344|F1P344\_CHICK sp|Q5ZID6|5NT3A\_CHICK tr|H9L1Y8|H9L1Y8\_CHICK tr|R4GHK0|R4GHK0\_CHICK tr|Q5F3M3|Q5F3M3\_CHICK tr|Q5ZIY7|Q5ZIY7\_CHICK tr|F1NLP5|F1NLP5\_CHICK tr|F1NYJ8|F1NYJ8\_CHICK tr|R4GLD0|R4GLD0\_CHICK tr|F1N9I9|F1N9I9\_CHICK tr|E1C0G7|E1C0G7\_CHICK tr|F1NLY5|F1NLY5\_CHICK tr|Q5ZI41|Q5ZI41\_CHICK tr|F1NWI7|F1NWI7\_CHICK tr|F1NVC9|F1NVC9\_CHICK tr|E1C3G0|E1C3G0\_CHICK tr|F1NI92|F1NI92\_CHICK tr|Q5ZM79|Q5ZM79\_CHICK tr|G4XJS7|G4XJS7\_CHICK tr|R4GKR6|R4GKR6\_CHICK tr|F1NUQ7|F1NUQ7\_CHICK tr|Q90818|Q90818\_CHICK tr|F1NCE5|F1NCE5\_CHICK tr|R4GKD5|R4GKD5\_CHICK tr|Q6LC82|Q6LC82\_CHICK tr|F1N8D9|F1N8D9\_CHICK tr|F1NXH6|F1NXH6\_CHICK tr|F1NPV6|F1NPV6\_CHICK tr|F1P3Z2|F1P3Z2\_CHICK sp|Q5ZK17|SETD6\_CHICK sp|Q9PVN2|VAX1\_CHICK tr|F1NWM9|F1NWM9\_CHICK tr|Q6UG48|Q6UG48\_CHICK tr|F1NP55|F1NP55\_CHICK tr|Q7T110|Q7T110\_CHICK tr|O73883|O73883\_CHICK tr|Q5F3R4|Q5F3R4\_CHICK tr|E1BXD6|E1BXD6\_CHICK tr|Q6UG49|Q6UG49\_CHICK tr|O42396|O42396\_CHICK tr|Q5UB38|Q5UB38\_CHICK tr|E1BUY9|E1BUY9\_CHICK tr|F1NG97|F1NG97\_CHICK tr|F1NH16|F1NH16\_CHICK tr|Q7T180|Q7T180\_CHICK tr|Q335Q2|Q335Q2\_CHICK tr|E1BWN6|E1BWN6\_CHICK tr|E1C524|E1C524\_CHICK tr|F1N954|F1N954\_CHICK sp|O42265|PSA1\_CHICK tr|B5BSF0|B5BSF0\_CHICK tr|F1P1U3|F1P1U3\_CHICK tr|F1NIR3|F1NIR3\_CHICK tr|R4GKW0|R4GKW0\_CHICK tr|D4QB12|D4QB12\_CHICK tr|D4QB10|D4QB10\_CHICK tr|D4QB11|D4QB11\_CHICK tr|D4QB13|D4QB13\_CHICK tr|Q9I9V2|Q9I9V2\_CHICK tr|R4GM93|R4GM93\_CHICK tr|E1BYQ2|E1BYQ2\_CHICK sp|Q5F407|MGT4A\_CHICK tr|Q9YHB8|Q9YHB8\_CHICK tr|E1C3T7|E1C3T7\_CHICK sp|P26697|GSTA3\_CHICK tr|F1NQS2|F1NQS2\_CHICK tr|F1NN78|F1NN78\_CHICK tr|Q5ZMR7|Q5ZMR7\_CHICK tr|R4GK79|R4GK79\_CHICK tr|Q3C2H7|Q3C2H7\_CHICK tr|F1NNV7|F1NNV7\_CHICK tr|B0FYW9|B0FYW9\_CHICK tr|E1BZW8|E1BZW8\_CHICK tr|E1C641|E1C641\_CHICK sp|P07660|CALC\_CHICK sp|Q5F383|SPP2B\_CHICK tr|F1NRC1|F1NRC1\_CHICK tr|F1NC01|F1NC01\_CHICK tr|H9KZK2|H9KZK2\_CHICK sp|Q08727|HXA2\_CHICK tr|E1C463|E1C463\_CHICK sp|O42406|SIX3\_CHICK tr|Q5ZLI2|Q5ZLI2\_CHICK tr|F1P0D8|F1P0D8\_CHICK tr|F1P5Y5|F1P5Y5\_CHICK sp|P58268|REQU\_CHICK tr|E1BX61|E1BX61\_CHICK tr|F1NU52|F1NU52\_CHICK sp|Q05438|BMR1B\_CHICK tr|Q957U2|Q957U2\_CHICK tr|Q5ZHJ7|Q5ZHJ7\_CHICK tr|R4GKI7|R4GKI7\_CHICK tr|F1NIW3|F1NIW3\_CHICK tr|V5Y013|V5Y013\_CHICK tr|E1C137|E1C137\_CHICK tr|F1NFQ0|F1NFQ0\_CHICK tr|Q92073|Q92073\_CHICK tr|Q71BV1|Q71BV1\_CHICK tr|F1NGL3|F1NGL3\_CHICK tr|Q6B7Z6|Q6B7Z6\_CHICK tr|Q5ZJI7|Q5ZJI7\_CHICK sp|O57429|UBP2\_CHICK tr|R4GHC8|R4GHC8\_CHICK tr|E1C2E5|E1C2E5\_CHICK tr|R4GK47|R4GK47\_CHICK tr|F1P1G1|F1P1G1\_CHICK tr|F1NCP7|F1NCP7\_CHICK tr|R4GIT7|R4GIT7\_CHICK tr|A0A090HZ95|A0A090HZ95\_CHICK tr|R4GLK7|R4GLK7\_CHICK tr|Q5ZLZ4|Q5ZLZ4\_CHICK tr|C1K6P5|C1K6P5\_CHICK tr|C1K6P6|C1K6P6\_CHICK tr|R4GJ90|R4GJ90\_CHICK tr|F1NG06|F1NG06\_CHICK tr|G1K323|G1K323\_CHICK tr|F1NLD0|F1NLD0\_CHICK sp|Q5F476|CO041\_CHICK sp|Q5ZM96|QTRD1\_CHICK tr|H9L0C0|H9L0C0\_CHICK tr|R4GH36|R4GH36\_CHICK tr|R4GLX3|R4GLX3\_CHICK tr|R4GJZ8|R4GJZ8\_CHICK tr|F1NWK3|F1NWK3\_CHICK tr|F1NL91|F1NL91\_CHICK sp|Q5ZI05|LMBD1\_CHICK tr|R4GGD4|R4GGD4\_CHICK sp|P15720|MBP\_CHICK tr|Q6PPA9|Q6PPA9\_CHICK tr|Q2L7E6|Q2L7E6\_CHICK tr|R4GG79|R4GG79\_CHICK tr|E1BYK4|E1BYK4\_CHICK tr|R4GH85|R4GH85\_CHICK tr|B2ZAI5|B2ZAI5\_CHICK tr|C5J075|C5J075\_CHICK tr|B2ZAL8|B2ZAL8\_CHICK tr|A9QSZ6|A9QSZ6\_CHICK tr|E1C526|E1C526\_CHICK tr|R4GIX0|R4GIX0\_CHICK tr|E1BX14|E1BX14\_CHICK tr|E1BSV5|E1BSV5\_CHICK sp|Q90669|AVR2A\_CHICK tr|F1NZ91|F1NZ91\_CHICK tr|F1N9N7|F1N9N7\_CHICK tr|E1BTV4|E1BTV4\_CHICK tr|H9KZU6|H9KZU6\_CHICK tr|F1NM50|F1NM50\_CHICK tr|E1C1S2|E1C1S2\_CHICK tr|O73911|O73911\_CHICK tr|Q5ZI97|Q5ZI97\_CHICK tr|F1NAY8|F1NAY8\_CHICK tr|R4GK68|R4GK68\_CHICK tr|Q800W1|Q800W1\_CHICK sp|Q9I946|PDGFC\_CHICK tr|Q9PTY8|Q9PTY8\_CHICK sp|Q9YGI5|DNAS1\_CHICK tr|Q6V0N9|Q6V0N9\_CHICK tr|H9L1A2|H9L1A2\_CHICK tr|R4GLI0|R4GLI0\_CHICK tr|Q6UC85|Q6UC85\_CHICK tr|R9PXM4|R9PXM4\_CHICK sp|Q90871|IRF8\_CHICK tr|Q8QFT4|Q8QFT4\_CHICK sp|O42280|WNT9A\_CHICK tr|F1NSC7|F1NSC7\_CHICK tr|Q9DEQ8|Q9DEQ8\_CHICK tr|F1NNA7|F1NNA7\_CHICK tr|F1NKH7|F1NKH7\_CHICK tr|F1NVP3|F1NVP3\_CHICK tr|E1BWH0|E1BWH0\_CHICK tr|Q91411|Q91411\_CHICK tr|E1BYJ0|E1BYJ0\_CHICK sp|Q5F414|PNO1\_CHICK tr|F1NWU0|F1NWU0\_CHICK tr|F1NVQ3|F1NVQ3\_CHICK tr|F1NYB0|F1NYB0\_CHICK tr|F1NC66|F1NC66\_CHICK tr|Q5ZJE6|Q5ZJE6\_CHICK tr|F1NGW4|F1NGW4\_CHICK sp|P00337|LDHB\_CHICK tr|Q5ZHR5|Q5ZHR5\_CHICK tr|R4GIY5|R4GIY5\_CHICK tr|E1BUQ9|E1BUQ9\_CHICK tr|F1P456|F1P456\_CHICK tr|B0LHV3|B0LHV3\_CHICK tr|R4GHA1|R4GHA1\_CHICK tr|R4GKI8|R4GKI8\_CHICK tr|Q9PSA4|Q9PSA4\_CHICK tr|E1BYU0|E1BYU0\_CHICK tr|Q5F3M2|Q5F3M2\_CHICK tr|Q5ZHK3|Q5ZHK3\_CHICK tr|E1BS94|E1BS94\_CHICK tr|H9CX00|H9CX00\_CHICK tr|F1NN44|F1NN44\_CHICK tr|H9L1Y1|H9L1Y1\_CHICK tr|E1C2N4|E1C2N4\_CHICK tr|F1P4C2|F1P4C2\_CHICK tr|R4GIE6|R4GIE6\_CHICK tr|F1P1V1|F1P1V1\_CHICK tr|Q5ZLR3|Q5ZLR3\_CHICK tr|E1C8L2|E1C8L2\_CHICK tr|R4GLI6|R4GLI6\_CHICK tr|E1BZT4|E1BZT4\_CHICK tr|Q8UWJ8|Q8UWJ8\_CHICK tr|E1C9I1|E1C9I1\_CHICK tr|F1NYR3|F1NYR3\_CHICK tr|H9L285|H9L285\_CHICK tr|E1BX32|E1BX32\_CHICK tr|H9A8K8|H9A8K8\_CHICK tr|E1BWG8|E1BWG8\_CHICK tr|F1NKV1|F1NKV1\_CHICK tr|R4GLD6|R4GLD6\_CHICK tr|A5HMN9|A5HMN9\_CHICK tr|F1CN42|F1CN42\_CHICK tr|F1CMZ9|F1CMZ9\_CHICK sp|Q9DGD1|MGT4C\_CHICK tr|F1NKS3|F1NKS3\_CHICK ENSEMBL:ENSBTAP00000031360 sp|Q5ZI27|NAIF1\_CHICK tr|F1P3H4|F1P3H4\_CHICK tr|F1NF76|F1NF76\_CHICK tr|E1BZ50|E1BZ50\_CHICK tr|F1NDW2|F1NDW2\_CHICK tr|F1P0X8|F1P0X8\_CHICK tr|F1P598|F1P598\_CHICK tr|B4X7K8|B4X7K8\_CHICK tr|R4GG90|R4GG90\_CHICK tr|F1NB05|F1NB05\_CHICK tr|H9KZ72|H9KZ72\_CHICK tr|R4GHE9|R4GHE9\_CHICK sp|Q90596|MAFK\_CHICK tr|F1NK95|F1NK95\_CHICK tr|F1NDS5|F1NDS5\_CHICK tr|E1C923|E1C923\_CHICK tr|Q5ZK23|Q5ZK23\_CHICK tr|B8YIQ3|B8YIQ3\_CHICK tr|B8YIH3|B8YIH3\_CHICK tr|B8YIG3|B8YIG3\_CHICK tr|E1C2Q4|E1C2Q4\_CHICK tr|B8YIL1|B8YIL1\_CHICK tr|E1BQA5|E1BQA5\_CHICK tr|F1NLF7|F1NLF7\_CHICK tr|Q5ZK56|Q5ZK56\_CHICK sp|P26990|ARF6\_CHICK tr|R4GLX8|R4GLX8\_CHICK tr|R4GJJ4|R4GJJ4\_CHICK tr|B4X7N1|B4X7N1\_CHICK tr|E1C2M7|E1C2M7\_CHICK tr|F1NF19|F1NF19\_CHICK sp|Q5ZIP6|OTU6B\_CHICK tr|F1NAB6|F1NAB6\_CHICK tr|F1NF74|F1NF74\_CHICK tr|E1C7S0|E1C7S0\_CHICK tr|A0PFW5|A0PFW5\_CHICK tr|E1BZD7|E1BZD7\_CHICK tr|R4GJT5|R4GJT5\_CHICK tr|Q5ZJ62|Q5ZJ62\_CHICK tr|H9KZS5|H9KZS5\_CHICK tr|F1NI12|F1NI12\_CHICK tr|Q6ZYP0|Q6ZYP0\_CHICK tr|F1N8F8|F1N8F8\_CHICK tr|E1BRY5|E1BRY5\_CHICK tr|Q9I8S1|Q9I8S1\_CHICK tr|Q98952|Q98952\_CHICK tr|F1P536|F1P536\_CHICK tr|E1BUZ3|E1BUZ3\_CHICK tr|F1NZE6|F1NZE6\_CHICK tr|A0A0D5ZDF9|A0A0D5ZDF9\_9VIRU tr|Q5ZLE3|Q5ZLE3\_CHICK tr|F1NGK7|F1NGK7\_CHICK tr|Q9I862|Q9I862\_CHICK tr|Q9PSH2|Q9PSH2\_CHICK tr|F1NJ54|F1NJ54\_CHICK tr|Q5ZIW4|Q5ZIW4\_CHICK tr|F1NCV1|F1NCV1\_CHICK tr|Q5ZJN7|Q5ZJN7\_CHICK tr|Q766V2|Q766V2\_CHICK tr|R4GLP5|R4GLP5\_CHICK tr|H9L1T0|H9L1T0\_CHICK tr|Q5F3V1|Q5F3V1\_CHICK sp|P27177|PRIO\_CHICK sp|Q9YGL7|PIT1\_CHICK sp|P48800|FGF2\_CHICK tr|O73933|O73933\_CHICK tr|F1NRJ0|F1NRJ0\_CHICK tr|Q07659|Q07659\_CHICK tr|D6PZR1|D6PZR1\_CHICK tr|F1NRX8|F1NRX8\_CHICK tr|F1NXP8|F1NXP8\_CHICK tr|A0A088BIJ9|A0A088BIJ9\_CHICK tr|Q34575|Q34575\_CHICK tr|E1BUR3|E1BUR3\_CHICK tr|F1N8G6|F1N8G6\_CHICK sp|Q5ZMS1|TAF8\_CHICK tr|O93621|O93621\_CHICK tr|R4GIW1|R4GIW1\_CHICK tr|O73776|O73776\_CHICK tr|Q5ZIA1|Q5ZIA1\_CHICK tr|Q5EVY2|Q5EVY2\_CHICK sp|Q5ZML6|F210A\_CHICK tr|Q9DEB1|Q9DEB1\_CHICK tr|B4X7L1|B4X7L1\_CHICK tr|E1C3C5|E1C3C5\_CHICK tr|Q71U37|Q71U37\_CHICK tr|E1C0G4|E1C0G4\_CHICK tr|E1C6J7|E1C6J7\_CHICK tr|E1C0A3|E1C0A3\_CHICK tr|F1P1W7|F1P1W7\_CHICK tr|F1NJ69|F1NJ69\_CHICK tr|Q5ZMS9|Q5ZMS9\_CHICK tr|F1NCD8|F1NCD8\_CHICK tr|Q5ZME0|Q5ZME0\_CHICK tr|F1NX85|F1NX85\_CHICK tr|Q9I8W3|Q9I8W3\_CHICK tr|F1NHW8|F1NHW8\_CHICK tr|F1N9Y7|F1N9Y7\_CHICK tr|R4GHY8|R4GHY8\_CHICK tr|F1ND62|F1ND62\_CHICK tr|R4GGX1|R4GGX1\_CHICK tr|E1C052|E1C052\_CHICK tr|F1NSZ0|F1NSZ0\_CHICK tr|F1NBT9|F1NBT9\_CHICK sp|P53352|INCE\_CHICK tr|Q9DEG0|Q9DEG0\_CHICK tr|C6KJC5|C6KJC5\_CHICK tr|E1BZ74|E1BZ74\_CHICK tr|E1BU99|E1BU99\_CHICK tr|H9KZD9|H9KZD9\_CHICK sp|Q9DF69|CSPG5\_CHICK tr|F1NGX7|F1NGX7\_CHICK tr|Q588G7|Q588G7\_CHICK tr|Q9I8D9|Q9I8D9\_CHICK tr|F1NYQ1|F1NYQ1\_CHICK tr|R4GIX8|R4GIX8\_CHICK tr|F1NJV0|F1NJV0\_CHICK tr|F1P4I3|F1P4I3\_CHICK tr|Q6JGV2|Q6JGV2\_CHICK tr|R4GK39|R4GK39\_CHICK tr|T1W2T5|T1W2T5\_CHICK tr|Q6JGV0|Q6JGV0\_CHICK tr|T1W2U0|T1W2U0\_CHICK tr|Q6JGV1|Q6JGV1\_CHICK tr|T1W2S9|T1W2S9\_CHICK tr|O98229|O98229\_CHICK tr|E1C8C0|E1C8C0\_CHICK tr|Q5ZKY6|Q5ZKY6\_CHICK tr|H9KYN7|H9KYN7\_CHICK tr|R4GIX5|R4GIX5\_CHICK Q3ZBS7 tr|E1C5L9|E1C5L9\_CHICK tr|Q8JHU1|Q8JHU1\_CHICK tr|H9L147|H9L147\_CHICK tr|R9PXQ6|R9PXQ6\_CHICK tr|F1NI41|F1NI41\_CHICK tr|F1NLS4|F1NLS4\_CHICK tr|E1BR66|E1BR66\_CHICK sp|O42252|LDB1\_CHICK sp|Q90VZ9|HXA7\_CHICK tr|E1BR15|E1BR15\_CHICK tr|E1C0E1|E1C0E1\_CHICK tr|E1C1H6|E1C1H6\_CHICK tr|E1BZ16|E1BZ16\_CHICK tr|F1NC09|F1NC09\_CHICK tr|E1C4Y2|E1C4Y2\_CHICK sp|Q90963|PRRX2\_CHICK tr|Q5ZKH2|Q5ZKH2\_CHICK tr|F1NEI9|F1NEI9\_CHICK tr|Q5F386|Q5F386\_CHICK tr|Q9YH55|Q9YH55\_CHICK tr|Q5ZMD5|Q5ZMD5\_CHICK tr|F1NGN7|F1NGN7\_CHICK sp|P55165|CRBB3\_CHICK tr|R4GF64|R4GF64\_CHICK tr|Q0MS56|Q0MS56\_CHICK tr|E1C0S1|E1C0S1\_CHICK sp|Q5ZL36|ZFY27\_CHICK tr|E1BRU9|E1BRU9\_CHICK sp|Q90955|TEBP\_CHICK tr|Q6SVA5|Q6SVA5\_CHICK tr|F1P3U0|F1P3U0\_CHICK tr|H9L0R6|H9L0R6\_CHICK tr|F1P473|F1P473\_CHICK tr|F1NFU6|F1NFU6\_CHICK tr|Q5ZLV5|Q5ZLV5\_CHICK tr|F1P463|F1P463\_CHICK tr|R4GIG8|R4GIG8\_CHICK tr|R4GKC2|R4GKC2\_CHICK tr|R4GJC2|R4GJC2\_CHICK tr|F1NIA8|F1NIA8\_CHICK tr|F1N8A1|F1N8A1\_CHICK tr|E1C928|E1C928\_CHICK tr|F1NYE5|F1NYE5\_CHICK tr|E1C7B8|E1C7B8\_CHICK tr|Q5ZJM4|Q5ZJM4\_CHICK tr|E1BW57|E1BW57\_CHICK tr|R4GK76|R4GK76\_CHICK sp|Q5F361|TBCK\_CHICK tr|B4X7N3|B4X7N3\_CHICK tr|E1BUH9|E1BUH9\_CHICK tr|B4YK11|B4YK11\_CHICK tr|Q08527|Q08527\_CHICK tr|H9L014|H9L014\_CHICK tr|Q90636|Q90636\_CHICK tr|R4GLQ3|R4GLQ3\_CHICK tr|F1P235|F1P235\_CHICK tr|E1BTR1|E1BTR1\_CHICK tr|I7LRG5|I7LRG5\_CHICK tr|E1C992|E1C992\_CHICK tr|F1P212|F1P212\_CHICK tr|F1P5E6|F1P5E6\_CHICK tr|Q5F437|Q5F437\_CHICK tr|A0A089FKX5|A0A089FKX5\_CHICK tr|E1C6S8|E1C6S8\_CHICK tr|D5JGF7|D5JGF7\_CHICK tr|E1C432|E1C432\_CHICK tr|R4GFS9|R4GFS9\_CHICK tr|Q9YHV9|Q9YHV9\_CHICK tr|R4GM92|R4GM92\_CHICK tr|F1NBX2|F1NBX2\_CHICK tr|F1NKD3|F1NKD3\_CHICK sp|Q5ZJM3|NGLY1\_CHICK tr|G1UHB7|G1UHB7\_CHICK tr|R4GKR1|R4GKR1\_CHICK tr|F1CN62|F1CN62\_CHICK tr|F1NZ12|F1NZ12\_CHICK tr|E1BRR8|E1BRR8\_CHICK tr|H9L258|H9L258\_CHICK tr|F1NIX0|F1NIX0\_CHICK tr|E1C7R3|E1C7R3\_CHICK tr|R4GJ41|R4GJ41\_CHICK tr|R4GJ76|R4GJ76\_CHICK tr|E1C9H9|E1C9H9\_CHICK tr|E3VVZ7|E3VVZ7\_CHICK tr|Q5ZJF2|Q5ZJF2\_CHICK sp|P53545|GSC\_CHICK tr|E1C0B0|E1C0B0\_CHICK tr|H9L143|H9L143\_CHICK tr|F1NDU6|F1NDU6\_CHICK tr|F1NTD3|F1NTD3\_CHICK tr|F1P4D1|F1P4D1\_CHICK sp|Q5MNV6|ZNT7\_CHICK tr|E1BUW1|E1BUW1\_CHICK tr|F1NKP1|F1NKP1\_CHICK tr|D3W668|D3W668\_CHICK tr|Q5ZLZ2|Q5ZLZ2\_CHICK tr|D3W660|D3W660\_CHICK sp|Q01741|CP1A2\_CHICK tr|D3W661|D3W661\_CHICK tr|E1C6P2|E1C6P2\_CHICK tr|F1NFL9|F1NFL9\_CHICK tr|F1NBL4|F1NBL4\_CHICK tr|F1P2F8|F1P2F8\_CHICK tr|A0A023J5T7|A0A023J5T7\_CHICK tr|E1BS42|E1BS42\_CHICK tr|E1BSG9|E1BSG9\_CHICK tr|A0A023J5T6|A0A023J5T6\_CHICK tr|Q6ZXJ6|Q6ZXJ6\_CHICK tr|F1NML9|F1NML9\_CHICK tr|F1NYI9|F1NYI9\_CHICK tr|Q5H7M6|Q5H7M6\_CHICK tr|F1NNE8|F1NNE8\_CHICK tr|Q6SVA9|Q6SVA9\_CHICK sp|Q5ZJZ5|BDH\_CHICK tr|F1NHH9|F1NHH9\_CHICK tr|R4GK34|R4GK34\_CHICK tr|F1NC13|F1NC13\_CHICK tr|F1NF65|F1NF65\_CHICK sp|Q05063|LYOX\_CHICK tr|F1NHJ3|F1NHJ3\_CHICK tr|Q5ZMD9|Q5ZMD9\_CHICK tr|Q9W6N2|Q9W6N2\_CHICK tr|Q6V0P1|Q6V0P1\_CHICK tr|R4GKZ6|R4GKZ6\_CHICK tr|Q56IA0|Q56IA0\_CHICK tr|F1NGJ5|F1NGJ5\_CHICK tr|F1NK58|F1NK58\_CHICK tr|F1NMX0|F1NMX0\_CHICK tr|Q5ZKI8|Q5ZKI8\_CHICK tr|Q5ZJT7|Q5ZJT7\_CHICK tr|Q8UWC1|Q8UWC1\_CHICK tr|F1NLV3|F1NLV3\_CHICK tr|Q9PVK1|Q9PVK1\_CHICK tr|Q5ZJJ4|Q5ZJJ4\_CHICK tr|F1P256|F1P256\_CHICK tr|E1BY69|E1BY69\_CHICK tr|F4ZCJ4|F4ZCJ4\_CHICK tr|R4GF82|R4GF82\_CHICK tr|E1BT03|E1BT03\_CHICK tr|F1NA87|F1NA87\_CHICK tr|R4GIN7|R4GIN7\_CHICK tr|Z4YJC3|Z4YJC3\_CHICK tr|E1C6Q4|E1C6Q4\_CHICK tr|R4GG22|R4GG22\_CHICK tr|Q07784|Q07784\_CHICK tr|E1C5C5|E1C5C5\_CHICK tr|F1NJ28|F1NJ28\_CHICK tr|F1N8U1|F1N8U1\_CHICK sp|Q4W5Z4|DNM3A\_CHICK tr|R4GMA2|R4GMA2\_CHICK tr|H9KYU4|H9KYU4\_CHICK tr|Q5ZHZ7|Q5ZHZ7\_CHICK tr|F1NL52|F1NL52\_CHICK sp|P01109|MYC\_CHICK tr|Q5ZI10|Q5ZI10\_CHICK tr|F1NHS3|F1NHS3\_CHICK tr|E1BRB8|E1BRB8\_CHICK tr|E1C0I5|E1C0I5\_CHICK tr|F1NS00|F1NS00\_CHICK tr|F1C6W5|F1C6W5\_CHICK tr|F1NXE0|F1NXE0\_CHICK tr|F1NI98|F1NI98\_CHICK tr|E1C634|E1C634\_CHICK tr|E1C751|E1C751\_CHICK tr|O42095|O42095\_CHICK sp|P10360|P53\_CHICK sp|Q71SY6|OAF\_CHICK sp|Q5ZJJ5|AKTIP\_CHICK tr|E1C1I5|E1C1I5\_CHICK tr|A7XMV4|A7XMV4\_CHICK tr|E1BW63|E1BW63\_CHICK sp|Q5ZIU0|ORML2\_CHICK tr|F1NXP3|F1NXP3\_CHICK tr|B2ZAM0|B2ZAM0\_CHICK tr|F1NP15|F1NP15\_CHICK sp|Q98943|CASP2\_CHICK tr|F1NLM0|F1NLM0\_CHICK tr|Q5ZKP3|Q5ZKP3\_CHICK tr|A9UGK7|A9UGK7\_CHICK sp|P18519|TNR16\_CHICK tr|E1C312|E1C312\_CHICK tr|R4GKZ5|R4GKZ5\_CHICK tr|F1NLI4|F1NLI4\_CHICK tr|F1NIY3|F1NIY3\_CHICK tr|Q5ZM76|Q5ZM76\_CHICK tr|E1BUL5|E1BUL5\_CHICK sp|P19019|GBRB3\_CHICK tr|Q70M90|Q70M90\_CHICK tr|F1N8N3|F1N8N3\_CHICK tr|E1C516|E1C516\_CHICK tr|F1NDH1|F1NDH1\_CHICK tr|C8CLK2|C8CLK2\_CHICK tr|F1NJ20|F1NJ20\_CHICK tr|E1BSS1|E1BSS1\_CHICK tr|E1BYL6|E1BYL6\_CHICK tr|Q8QG58|Q8QG58\_CHICK tr|F1NPI3|F1NPI3\_CHICK tr|E7EDS8|E7EDS8\_CHICK tr|R4GJE9|R4GJE9\_CHICK tr|H9KZ87|H9KZ87\_CHICK tr|F1P3Q7|F1P3Q7\_CHICK tr|F1NBU3|F1NBU3\_CHICK tr|F1NHQ3|F1NHQ3\_CHICK sp|P20135|GSTT1\_CHICK tr|Q9DF58|Q9DF58\_CHICK tr|F1NP98|F1NP98\_CHICK tr|F1NGG1|F1NGG1\_CHICK tr|F1NNL6|F1NNL6\_CHICK tr|E1BU20|E1BU20\_CHICK tr|F1NZ17|F1NZ17\_CHICK sp|P34996|P2RY1\_CHICK tr|A0A0B4ZVA3|A0A0B4ZVA3\_CHICK tr|E1C0T7|E1C0T7\_CHICK tr|F1NL71|F1NL71\_CHICK Q05443 tr|E1BT91|E1BT91\_CHICK tr|F1NLC3|F1NLC3\_CHICK sp|Q71T09|BHE22\_CHICK tr|Q9DEE1|Q9DEE1\_CHICK tr|Q9DEE5|Q9DEE5\_CHICK tr|E1C2E9|E1C2E9\_CHICK tr|F1ND23|F1ND23\_CHICK tr|R4GL01|R4GL01\_CHICK tr|Q2EJU6|Q2EJU6\_CHICK tr|Q5ZKR1|Q5ZKR1\_CHICK tr|Q5ZK54|Q5ZK54\_CHICK tr|F1NAT4|F1NAT4\_CHICK sp|P24367|PPIB\_CHICK tr|F1NV93|F1NV93\_CHICK tr|F1NJK4|F1NJK4\_CHICK tr|F1NTK1|F1NTK1\_CHICK tr|Q90726|Q90726\_CHICK tr|F1P5H1|F1P5H1\_CHICK tr|F1NYU3|F1NYU3\_CHICK tr|Q802C2|Q802C2\_CHICK tr|Q5F3M6|Q5F3M6\_CHICK tr|F1NEJ5|F1NEJ5\_CHICK tr|F1P1C2|F1P1C2\_CHICK tr|F1NZF1|F1NZF1\_CHICK tr|E1BRL8|E1BRL8\_CHICK tr|Q90865|Q90865\_CHICK tr|E1C8A1|E1C8A1\_CHICK tr|Q8WD65|Q8WD65\_ALEAL tr|E1C2H6|E1C2H6\_CHICK tr|R4GLS9|R4GLS9\_CHICK sp|P08836|FPPS\_CHICK sp|Q5ZK01|PELO\_CHICK tr|E1C7T1|E1C7T1\_CHICK sp|P28682|OPSB\_CHICK tr|F1NX82|F1NX82\_CHICK tr|R4GHZ2|R4GHZ2\_CHICK tr|A0A0A0MQ60|A0A0A0MQ60\_CHICK tr|F1NHZ3|F1NHZ3\_CHICK tr|R4GHI6|R4GHI6\_CHICK tr|Q5ZJC1|Q5ZJC1\_CHICK tr|E1BQF6|E1BQF6\_CHICK tr|F1NXX2|F1NXX2\_CHICK tr|Q5F3R8|Q5F3R8\_CHICK tr|E1BU28|E1BU28\_CHICK tr|B9VV89|B9VV89\_9GALL tr|E1BS43|E1BS43\_CHICK tr|F1NBK1|F1NBK1\_CHICK tr|E1BZT0|E1BZT0\_CHICK tr|Q4ZJF2|Q4ZJF2\_CHICK tr|F1NN62|F1NN62\_CHICK tr|R4GGR6|R4GGR6\_CHICK sp|P11939|FOS\_CHICK tr|F1NAH1|F1NAH1\_CHICK tr|H9L062|H9L062\_CHICK tr|R4GJK9|R4GJK9\_CHICK tr|E1C252|E1C252\_CHICK tr|Q5KRA4|Q5KRA4\_CHICK tr|Q7LZ59|Q7LZ59\_CHICK tr|Q5ZJD6|Q5ZJD6\_CHICK tr|Q4L2A6|Q4L2A6\_CHICK sp|Q7ZZL8|TMHS\_CHICK sp|Q9DG23|POPD1\_CHICK tr|F1NNL3|F1NNL3\_CHICK tr|Q7LZ77|Q7LZ77\_CHICK tr|F1NNG7|F1NNG7\_CHICK tr|Q5ZJY8|Q5ZJY8\_CHICK tr|F1NZ30|F1NZ30\_CHICK sp|P05122|KCRB\_CHICK tr|F1NAI5|F1NAI5\_CHICK tr|F1C6U9|F1C6U9\_CHICK tr|F1P3G5|F1P3G5\_CHICK tr|Q9YGR0|Q9YGR0\_CHICK tr|R4GF40|R4GF40\_CHICK sp|P34822|DSL1\_CHICK tr|F1NWL1|F1NWL1\_CHICK tr|R4GKT0|R4GKT0\_CHICK tr|D2Z1L9|D2Z1L9\_CHICK sp|Q6K1L7|EIF1A\_CHICK tr|F1NJF3|F1NJF3\_CHICK tr|F1P1Z3|F1P1Z3\_CHICK sp|P39963|CCNB3\_CHICK tr|K4Q4R6|K4Q4R6\_CHICK tr|F1NRZ5|F1NRZ5\_CHICK tr|A2MXY3|A2MXY3\_CHICK tr|F1N8G5|F1N8G5\_CHICK tr|E1BZY3|E1BZY3\_CHICK tr|F6RS52|F6RS52\_CHICK tr|F1NNQ9|F1NNQ9\_CHICK tr|E1C487|E1C487\_CHICK tr|B2CL08|B2CL08\_CHICK tr|F1NFK6|F1NFK6\_CHICK tr|Q2UXM7|Q2UXM7\_CHICK tr|B1PMA5|B1PMA5\_CHICK tr|E1C2M3|E1C2M3\_CHICK tr|R4GHX3|R4GHX3\_CHICK tr|C7FGL4|C7FGL4\_CHICK tr|E1C4L3|E1C4L3\_CHICK tr|R4GFL2|R4GFL2\_CHICK sp|Q5ZLL9|BRM1L\_CHICK tr|E1C4N6|E1C4N6\_CHICK tr|R4GKT4|R4GKT4\_CHICK tr|F1NHZ4|F1NHZ4\_CHICK sp|Q7SZC6|RGS4\_CHICK tr|F1NL16|F1NL16\_CHICK tr|R4GIW9|R4GIW9\_CHICK tr|H9L209|H9L209\_CHICK tr|F1NH76|F1NH76\_CHICK tr|F1P0B5|F1P0B5\_CHICK tr|B5G4W1|B5G4W1\_CHICK tr|F1NLP2|F1NLP2\_CHICK tr|E1C010|E1C010\_CHICK tr|A1IIE5|A1IIE5\_CHICK tr|Q9PSS3|Q9PSS3\_CHICK tr|E1BU60|E1BU60\_CHICK tr|Q31620|Q31620\_CHICK tr|F1NYM5|F1NYM5\_CHICK tr|F1NH86|F1NH86\_CHICK tr|F1P533|F1P533\_CHICK tr|R4GMA3|R4GMA3\_CHICK tr|F1P267|F1P267\_CHICK tr|E1BYD3|E1BYD3\_CHICK tr|R4GGR7|R4GGR7\_CHICK tr|Q9PSP7|Q9PSP7\_CHICK tr|Q5ZHT9|Q5ZHT9\_CHICK tr|R4GKB4|R4GKB4\_CHICK tr|E1BV29|E1BV29\_CHICK tr|Q5ZM34|Q5ZM34\_CHICK tr|F1NI85|F1NI85\_CHICK tr|F1N9Y8|F1N9Y8\_CHICK tr|F1NEJ0|F1NEJ0\_CHICK tr|O93288|O93288\_CHICK tr|R4GKS8|R4GKS8\_CHICK tr|F1P1V6|F1P1V6\_CHICK tr|F1NZT9|F1NZT9\_CHICK tr|R4GFI7|R4GFI7\_CHICK tr|C1L370|C1L370\_CHICK tr|F1NY04|F1NY04\_CHICK sp|Q6PVW7|ZPBP2\_CHICK tr|F1NXW8|F1NXW8\_CHICK sp|P80026|PRVM\_CHICK tr|H9L056|H9L056\_CHICK tr|Q5ZHL2|Q5ZHL2\_CHICK tr|R4GFJ5|R4GFJ5\_CHICK tr|Q5F345|Q5F345\_CHICK tr|F1NKF0|F1NKF0\_CHICK tr|Q5ZM56|Q5ZM56\_CHICK tr|F1NV54|F1NV54\_CHICK tr|F1NSJ0|F1NSJ0\_CHICK tr|Q5ZHS5|Q5ZHS5\_CHICK sp|Q05714|CRBB2\_CHICK tr|A2IAR9|A2IAR9\_CHICK tr|E1C4G1|E1C4G1\_CHICK tr|F1NZM5|F1NZM5\_CHICK tr|Q6QIF4|Q6QIF4\_CHICK tr|E1BV34|E1BV34\_CHICK tr|F1NVP4|F1NVP4\_CHICK tr|E1C5L8|E1C5L8\_CHICK tr|R4GJC1|R4GJC1\_CHICK tr|R4GIU5|R4GIU5\_CHICK tr|Q802T0|Q802T0\_CHICK tr|E1C3W8|E1C3W8\_CHICK tr|F1NQ60|F1NQ60\_CHICK tr|E1C9E0|E1C9E0\_CHICK tr|F1NEK7|F1NEK7\_CHICK tr|A3QW72|A3QW72\_CHICK tr|F1NX33|F1NX33\_CHICK tr|Q5ZIN9|Q5ZIN9\_CHICK tr|A1IIE6|A1IIE6\_CHICK tr|E1C517|E1C517\_CHICK tr|Q0PVW8|Q0PVW8\_CHICK tr|Q0PVV1|Q0PVV1\_CHICK tr|R4GIW2|R4GIW2\_CHICK tr|E1BU17|E1BU17\_CHICK tr|W5S118|W5S118\_CHICK tr|R4GMG8|R4GMG8\_CHICK tr|F1NV70|F1NV70\_CHICK sp|Q5ZIP2|LTOR3\_CHICK tr|F1NKF9|F1NKF9\_CHICK tr|E1C770|E1C770\_CHICK tr|F1N8E6|F1N8E6\_CHICK sp|Q91348|F26L\_CHICK tr|F1NUR5|F1NUR5\_CHICK tr|F1NY84|F1NY84\_CHICK tr|F1P2Q9|F1P2Q9\_CHICK tr|F1NVT9|F1NVT9\_CHICK tr|E1BUA5|E1BUA5\_CHICK tr|E1C8J2|E1C8J2\_CHICK tr|F1NX01|F1NX01\_CHICK tr|E1C859|E1C859\_CHICK tr|R4GKS1|R4GKS1\_CHICK tr|Q6Q123|Q6Q123\_CHICK tr|E1C3B4|E1C3B4\_CHICK tr|E1BSN7|E1BSN7\_CHICK tr|Q5ZME4|Q5ZME4\_CHICK tr|Q8AXA8|Q8AXA8\_CHICK tr|E1BXI5|E1BXI5\_CHICK tr|F1NXG9|F1NXG9\_CHICK sp|P53449|ALDOC\_CHICK tr|F6ULT8|F6ULT8\_CHICK tr|A5HUJ7|A5HUJ7\_CHICK tr|R4GJV4|R4GJV4\_CHICK P02668 tr|F1NJI0|F1NJI0\_CHICK sp|P17153|ANXA5\_CHICK tr|F1NCU8|F1NCU8\_CHICK tr|E1C581|E1C581\_CHICK tr|Q6PTW2|Q6PTW2\_9EUCA tr|Q90WF6|Q90WF6\_CHICK tr|Q5MQR1|Q5MQR1\_CHICK tr|E1BZS8|E1BZS8\_CHICK tr|H9KZA7|H9KZA7\_CHICK tr|A3F959|A3F959\_CHICK tr|G8HY00|G8HY00\_CHICK tr|Q98TV2|Q98TV2\_CHICK tr|E1C645|E1C645\_CHICK tr|F1CN06|F1CN06\_CHICK tr|Q5ZKX8|Q5ZKX8\_CHICK tr|F1CN54|F1CN54\_CHICK tr|F1CN61|F1CN61\_CHICK tr|F1CN41|F1CN41\_CHICK tr|A0A089FKC9|A0A089FKC9\_CHICK tr|W5XHT5|W5XHT5\_CHICK tr|R4GLE3|R4GLE3\_CHICK tr|E1BUI0|E1BUI0\_CHICK tr|F1NP13|F1NP13\_CHICK tr|F1NA53|F1NA53\_CHICK sp|P43305|PRVU\_CHICK tr|Q9DEE2|Q9DEE2\_CHICK tr|Q9DEE4|Q9DEE4\_CHICK tr|F1P4S8|F1P4S8\_CHICK tr|Q5F3E0|Q5F3E0\_CHICK tr|Q5ZMP5|Q5ZMP5\_CHICK tr|E1C2B2|E1C2B2\_CHICK tr|R4GMK4|R4GMK4\_CHICK tr|F1NNS0|F1NNS0\_CHICK tr|Q5XXX9|Q5XXX9\_CHICK tr|Q804I4|Q804I4\_CHICK tr|Q5XXX6|Q5XXX6\_CHICK tr|Q5XXX8|Q5XXX8\_CHICK tr|F1NCH0|F1NCH0\_CHICK tr|Q5XXY0|Q5XXY0\_CHICK tr|Q5XXY1|Q5XXY1\_CHICK tr|Q5XXW4|Q5XXW4\_CHICK tr|E1C3V1|E1C3V1\_CHICK tr|Q5XXX7|Q5XXX7\_CHICK tr|F1NDG9|F1NDG9\_CHICK sp|Q5ZKT9|MIER1\_CHICK tr|C7G541|C7G541\_CHICK tr|F1NGW2|F1NGW2\_CHICK tr|E1C4L0|E1C4L0\_CHICK sp|Q5ZLY0|PHAX\_CHICK tr|F1NB74|F1NB74\_CHICK tr|Q5ZIG1|Q5ZIG1\_CHICK tr|E1BWX7|E1BWX7\_CHICK tr|Q7ZZY2|Q7ZZY2\_CHICK tr|F1NW57|F1NW57\_CHICK tr|A0AMH0|A0AMH0\_CHICK tr|R4GJL1|R4GJL1\_CHICK tr|A0A068PD50|A0A068PD50\_CHICK tr|Q2ACD6|Q2ACD6\_CHICK tr|F1NK18|F1NK18\_CHICK tr|Q156C7|Q156C7\_CHICK tr|R9R6L8|R9R6L8\_CHICK tr|F1P4Q2|F1P4Q2\_CHICK tr|E1C0X0|E1C0X0\_CHICK tr|Q7T1P8|Q7T1P8\_CHICK tr|A0A0C4JZ93|A0A0C4JZ93\_CHICK tr|F1NV80|F1NV80\_CHICK tr|F1NPM3|F1NPM3\_CHICK tr|F1NM02|F1NM02\_CHICK tr|F1NKG5|F1NKG5\_CHICK tr|E1C3F7|E1C3F7\_CHICK tr|Q7ZTS9|Q7ZTS9\_CHICK tr|E1BZT8|E1BZT8\_CHICK sp|F1NLH9|ITPA\_CHICK tr|F1NK57|F1NK57\_CHICK tr|E1BUT7|E1BUT7\_CHICK tr|R4GI64|R4GI64\_CHICK tr|Q7LZ74|Q7LZ74\_CHICK tr|H9L1R4|H9L1R4\_CHICK tr|F1NRP0|F1NRP0\_CHICK tr|E1BV18|E1BV18\_CHICK sp|Q5ZIG2|SLU7\_CHICK tr|F1NNB9|F1NNB9\_CHICK sp|P25692|KRCL\_CHICK tr|E1BUP8|E1BUP8\_CHICK tr|F1P011|F1P011\_CHICK tr|F1NR85|F1NR85\_CHICK tr|F1N9L4|F1N9L4\_CHICK tr|E1BZ68|E1BZ68\_CHICK tr|F1NY96|F1NY96\_CHICK tr|R4GGG7|R4GGG7\_CHICK tr|R4GF42|R4GF42\_CHICK tr|F1NLB5|F1NLB5\_CHICK tr|E1C036|E1C036\_CHICK sp|Q5EG71|EPGN\_CHICK tr|F1NBC6|F1NBC6\_CHICK tr|R4GJ85|R4GJ85\_CHICK tr|F1NVF9|F1NVF9\_CHICK tr|R4GI56|R4GI56\_CHICK tr|A5HTT7|A5HTT7\_CHICK tr|R4GG21|R4GG21\_CHICK tr|F1NLI9|F1NLI9\_CHICK tr|E1BUB4|E1BUB4\_CHICK tr|F1NGB1|F1NGB1\_CHICK tr|Q5ZJW3|Q5ZJW3\_CHICK tr|F1NFU4|F1NFU4\_CHICK tr|H9L0D7|H9L0D7\_CHICK tr|F1P3R5|F1P3R5\_CHICK sp|Q91998|BRN3\_CHICK sp|O42254|IF2B1\_CHICK tr|R4GLR8|R4GLR8\_CHICK tr|R4GL99|R4GL99\_CHICK tr|W6JNV0|W6JNV0\_CHICK sp|O42290|MAFA\_CHICK tr|F1NVJ8|F1NVJ8\_CHICK sp|P10184|IOV7\_CHICK tr|D4P8R5|D4P8R5\_CHICK tr|Q90WH5|Q90WH5\_CHICK tr|D7GLD5|D7GLD5\_CHICK tr|R4GKR5|R4GKR5\_CHICK tr|A5A2G3|A5A2G3\_CHICK tr|R4GFD2|R4GFD2\_CHICK tr|E1C111|E1C111\_CHICK tr|E1BSD3|E1BSD3\_CHICK tr|R4GLS0|R4GLS0\_CHICK tr|E1C021|E1C021\_CHICK tr|F1NQP8|F1NQP8\_CHICK tr|E1BT67|E1BT67\_CHICK tr|F1NZC9|F1NZC9\_CHICK tr|F1P4R1|F1P4R1\_CHICK tr|F1P292|F1P292\_CHICK tr|R4GM06|R4GM06\_CHICK tr|G0W2S9|G0W2S9\_CHICK tr|C0LUU5|C0LUU5\_CHICK tr|F1NLL7|F1NLL7\_CHICK tr|B4X7J1|B4X7J1\_CHICK tr|R4GKB8|R4GKB8\_CHICK tr|E1C015|E1C015\_CHICK tr|F1NZR2|F1NZR2\_CHICK tr|Q9DDL1|Q9DDL1\_CHICK tr|F1NDL3|F1NDL3\_CHICK tr|A2TH15|A2TH15\_CHICK tr|R4GF38|R4GF38\_CHICK tr|Q5ZJ31|Q5ZJ31\_CHICK tr|Q6QIU4|Q6QIU4\_CHICK tr|F1C6V3|F1C6V3\_CHICK tr|Q9PVK0|Q9PVK0\_CHICK tr|F1NS90|F1NS90\_CHICK tr|F1NZX2|F1NZX2\_CHICK tr|H9KYX5|H9KYX5\_CHICK tr|Q5ZLJ9|Q5ZLJ9\_CHICK tr|R4GIV5|R4GIV5\_CHICK tr|E1BQ87|E1BQ87\_CHICK tr|E1BS92|E1BS92\_CHICK tr|F1NR48|F1NR48\_CHICK tr|Q9PRW1|Q9PRW1\_CHICK tr|A0A077K840|A0A077K840\_CHICK tr|E1C8H3|E1C8H3\_CHICK sp|Q9PWB0|CER1\_CHICK tr|Q9PUK2|Q9PUK2\_CHICK tr|E1C745|E1C745\_CHICK tr|F1NNA8|F1NNA8\_CHICK tr|E1BSW8|E1BSW8\_CHICK tr|F1NJI2|F1NJI2\_CHICK tr|F1P159|F1P159\_CHICK tr|Q6ZYK4|Q6ZYK4\_CHICK tr|F1NY45|F1NY45\_CHICK tr|E1BZN8|E1BZN8\_CHICK tr|Q5ZLD6|Q5ZLD6\_CHICK tr|Q5ZHV0|Q5ZHV0\_CHICK tr|F1NQA5|F1NQA5\_CHICK tr|F1P282|F1P282\_CHICK sp|P36195|TDT\_CHICK tr|F1P317|F1P317\_CHICK tr|R4GIE9|R4GIE9\_CHICK tr|R4GFQ8|R4GFQ8\_CHICK tr|Q5ZJV5|Q5ZJV5\_CHICK tr|R4GLU4|R4GLU4\_CHICK tr|F1N901|F1N901\_CHICK tr|F1NY83|F1NY83\_CHICK tr|E1BSY4|E1BSY4\_CHICK tr|R4GJF4|R4GJF4\_CHICK tr|Q9W7P7|Q9W7P7\_CHICK tr|F1NSK2|F1NSK2\_CHICK tr|Q5ZJX9|Q5ZJX9\_CHICK tr|F1NSF2|F1NSF2\_CHICK tr|H9KYP7|H9KYP7\_CHICK tr|F1P3T3|F1P3T3\_CHICK tr|E1C821|E1C821\_CHICK tr|F1NLL2|F1NLL2\_CHICK tr|R4GJK2|R4GJK2\_CHICK tr|E1C0X6|E1C0X6\_CHICK tr|R4GKQ4|R4GKQ4\_CHICK tr|F1NG23|F1NG23\_CHICK tr|E1BXK0|E1BXK0\_CHICK tr|B7FC06|B7FC06\_CHICK sp|Q90WD0|ARP3\_CHICK tr|Q4GWI9|Q4GWI9\_GALSO tr|E5DFK3|E5DFK3\_CHICK tr|Q4GWM8|Q4GWM8\_CHICK tr|B9VUB3|B9VUB3\_CHICK sp|Q9IAX2|IOD2\_CHICK tr|Q4GWL5|Q4GWL5\_GALVA tr|Q4GWQ4|Q4GWQ4\_CHICK tr|Q7GTU8|Q7GTU8\_CHICK tr|Q4GWR7|Q4GWR7\_GALSO tr|E5DEB1|E5DEB1\_CHICK tr|Q4GWP1|Q4GWP1\_CHICK sp|P18942|NU4LM\_CHICK tr|F1NIP4|F1NIP4\_CHICK tr|F1N874|F1N874\_CHICK tr|Q5ZII5|Q5ZII5\_CHICK tr|F1NDU1|F1NDU1\_CHICK sp|P62764|VISL1\_CHICK tr|E1C0Q9|E1C0Q9\_CHICK tr|F1P3U3|F1P3U3\_CHICK tr|F1ND25|F1ND25\_CHICK tr|Q6F4E2|Q6F4E2\_CHICK sp|Q98925|IRF2\_CHICK tr|E1C085|E1C085\_CHICK REFSEQ:XP\_585019 tr|W8VSV6|W8VSV6\_CHICK tr|F1NUB7|F1NUB7\_CHICK tr|F1NUB6|F1NUB6\_CHICK tr|F1NZP6|F1NZP6\_CHICK tr|Q683N1|Q683N1\_CHICK sp|P07322|ENOB\_CHICK tr|E1BVZ1|E1BVZ1\_CHICK tr|D5HSX3|D5HSX3\_CHICK tr|E1BYS7|E1BYS7\_CHICK tr|Q5F3H4|Q5F3H4\_CHICK tr|F1NT38|F1NT38\_CHICK tr|Q8QG68|Q8QG68\_CHICK tr|Q8AYA6|Q8AYA6\_CHICK tr|F6VMN5|F6VMN5\_CHICK tr|F1NXQ8|F1NXQ8\_CHICK tr|Q6R746|Q6R746\_CHICK tr|Q6ZXY4|Q6ZXY4\_CHICK tr|F1P3X7|F1P3X7\_CHICK tr|B5BSM5|B5BSM5\_CHICK tr|B5BSB2|B5BSB2\_CHICK tr|F1C6T8|F1C6T8\_CHICK tr|F1C6W4|F1C6W4\_CHICK tr|A5HUL4|A5HUL4\_CHICK tr|F1C6T6|F1C6T6\_CHICK tr|F1C6X1|F1C6X1\_CHICK tr|Q8AXQ0|Q8AXQ0\_CHICK tr|F1C6V7|F1C6V7\_CHICK tr|Q31410|Q31410\_CHICK tr|A5HUL2|A5HUL2\_CHICK tr|B5BSE2|B5BSE2\_CHICK tr|F1C6W3|F1C6W3\_CHICK tr|B5BSL0|B5BSL0\_CHICK tr|E2JF30|E2JF30\_CHICK tr|B6RCN5|B6RCN5\_CHICK tr|F1C6W0|F1C6W0\_CHICK tr|F1C6V9|F1C6V9\_CHICK tr|F1C6U0|F1C6U0\_CHICK tr|E2JF33|E2JF33\_CHICK tr|F1C6U4|F1C6U4\_CHICK tr|F1C6V8|F1C6V8\_CHICK tr|F1C6U8|F1C6U8\_CHICK tr|B5BSB4|B5BSB4\_CHICK tr|F1C6V6|F1C6V6\_CHICK tr|F1C6W8|F1C6W8\_CHICK tr|B5BSF6|B5BSF6\_CHICK tr|B6RCP3|B6RCP3\_CHICK tr|E2JF32|E2JF32\_CHICK tr|B6C862|B6C862\_CHICK tr|E5LFW9|E5LFW9\_CHICK tr|Q4U5Z9|Q4U5Z9\_CHICK tr|B5BS98|B5BS98\_CHICK tr|Q4U600|Q4U600\_CHICK tr|F1C6W9|F1C6W9\_CHICK tr|B5BSJ8|B5BSJ8\_CHICK tr|B5BSJ6|B5BSJ6\_CHICK tr|F1C6W6|F1C6W6\_CHICK tr|F1C6U7|F1C6U7\_CHICK tr|F2Z4M1|F2Z4M1\_CHICK tr|B6RCN7|B6RCN7\_CHICK tr|E2JF31|E2JF31\_CHICK tr|F1C6W7|F1C6W7\_CHICK tr|B6RCP2|B6RCP2\_CHICK tr|F1C6U2|F1C6U2\_CHICK tr|F1C6U3|F1C6U3\_CHICK tr|F1C6V4|F1C6V4\_CHICK tr|F1C6V1|F1C6V1\_CHICK tr|F1C6T9|F1C6T9\_CHICK tr|B6RCN9|B6RCN9\_CHICK tr|E6N1W6|E6N1W6\_CHICK tr|F1NCN4|F1NCN4\_CHICK tr|B6RCP0|B6RCP0\_CHICK tr|B5BSA0|B5BSA0\_CHICK tr|F1C6V2|F1C6V2\_CHICK tr|B6RCN6|B6RCN6\_CHICK tr|B5BSM3|B5BSM3\_CHICK tr|B6RCP5|B6RCP5\_CHICK tr|F1C6U6|F1C6U6\_CHICK tr|Q31413|Q31413\_CHICK tr|F1C6W1|F1C6W1\_CHICK tr|Q31553|Q31553\_CHICK tr|B5BSI2|B5BSI2\_CHICK tr|Q4U5Z8|Q4U5Z8\_CHICK tr|Q5F3P1|Q5F3P1\_CHICK tr|F1P4X3|F1P4X3\_CHICK tr|H9L0F6|H9L0F6\_CHICK tr|H6U650|H6U650\_CHICK tr|H6U668|H6U668\_CHICK tr|F1NUY4|F1NUY4\_CHICK tr|H6U665|H6U665\_CHICK tr|H6U667|H6U667\_CHICK tr|H6U644|H6U644\_CHICK tr|H9L164|H9L164\_CHICK tr|H6U669|H6U669\_CHICK tr|H6U664|H6U664\_CHICK tr|F1NZX8|F1NZX8\_CHICK tr|E1BYB8|E1BYB8\_CHICK tr|Q5ZHU6|Q5ZHU6\_CHICK tr|Q5F4C9|Q5F4C9\_CHICK tr|F1NSS6|F1NSS6\_CHICK tr|F1NH71|F1NH71\_CHICK tr|Q5ZM37|Q5ZM37\_CHICK tr|N0GSY0|N0GSY0\_9SAUR tr|E1C518|E1C518\_CHICK tr|A7M7B7|A7M7B7\_CHICK tr|F1NNC8|F1NNC8\_CHICK tr|E1C257|E1C257\_CHICK tr|R4GL14|R4GL14\_CHICK tr|Q7LZG0|Q7LZG0\_CHICK tr|F1NAZ9|F1NAZ9\_CHICK Q3SX09 tr|E1BSH5|E1BSH5\_CHICK tr|F1P3S8|F1P3S8\_CHICK tr|C4PAA9|C4PAA9\_CHICK tr|C4PAF0|C4PAF0\_CHICK tr|F1C6W2|F1C6W2\_CHICK tr|B6RCP4|B6RCP4\_CHICK tr|F1C6U1|F1C6U1\_CHICK tr|B6C863|B6C863\_CHICK tr|F1C6V5|F1C6V5\_CHICK tr|E1C9E6|E1C9E6\_CHICK sp|Q8QFP8|LIMK1\_CHICK tr|E1BZP8|E1BZP8\_CHICK tr|K4HS80|K4HS80\_CHICK tr|Q5ZKX7|Q5ZKX7\_CHICK tr|F1P189|F1P189\_CHICK tr|E1BT51|E1BT51\_CHICK tr|P79776|P79776\_CHICK tr|F1NNS1|F1NNS1\_CHICK tr|R4GK55|R4GK55\_CHICK tr|Q5ZK68|Q5ZK68\_CHICK sp|Q9I993|BCDO1\_CHICK tr|F1NLJ6|F1NLJ6\_CHICK tr|F1NGA6|F1NGA6\_CHICK tr|A0A088BHB9|A0A088BHB9\_CHICK tr|R4GGA7|R4GGA7\_CHICK tr|F1NAW4|F1NAW4\_CHICK tr|Q5ZIG5|Q5ZIG5\_CHICK tr|Q5F3J1|Q5F3J1\_CHICK tr|D0VX26|D0VX26\_CHICK tr|F1NRV5|F1NRV5\_CHICK tr|B6RCQ0|B6RCQ0\_CHICK tr|F1NB07|F1NB07\_CHICK tr|F1NTP5|F1NTP5\_CHICK tr|A6QR74|A6QR74\_CHICK tr|E1C233|E1C233\_CHICK tr|R9PXL1|R9PXL1\_CHICK sp|P21869|CSK22\_CHICK tr|Q5ZLB9|Q5ZLB9\_CHICK tr|R4GKV1|R4GKV1\_CHICK tr|F1NC20|F1NC20\_CHICK sp|Q5ZJI0|TPST2\_CHICK tr|E1BRH5|E1BRH5\_CHICK tr|F1NGS3|F1NGS3\_CHICK tr|E1BSY1|E1BSY1\_CHICK tr|R4GFX5|R4GFX5\_CHICK sp|Q5ZJS6|PAIP2\_CHICK tr|A5HUJ4|A5HUJ4\_CHICK tr|F1P5U3|F1P5U3\_CHICK sp|P13913|ARY1\_CHICK tr|F1NRS0|F1NRS0\_CHICK tr|F1NYJ0|F1NYJ0\_CHICK tr|B0BL89|B0BL89\_CHICK tr|R4GI53|R4GI53\_CHICK tr|F1NSW0|F1NSW0\_CHICK tr|E1C2D4|E1C2D4\_CHICK tr|F1NMD1|F1NMD1\_CHICK tr|F1NRC6|F1NRC6\_CHICK tr|R4GJJ0|R4GJJ0\_CHICK tr|F1NT08|F1NT08\_CHICK tr|Q5ZHX2|Q5ZHX2\_CHICK tr|Q2XSX4|Q2XSX4\_CHICK tr|Q5ZM59|Q5ZM59\_CHICK tr|Q6TRX0|Q6TRX0\_CHICK tr|R4GFE3|R4GFE3\_CHICK tr|F1NPM0|F1NPM0\_CHICK tr|Q2XSX2|Q2XSX2\_CHICK tr|R4GG20|R4GG20\_CHICK tr|E1C2E7|E1C2E7\_CHICK sp|P34904|GBRG4\_CHICK tr|F1NBM0|F1NBM0\_CHICK tr|E1BX57|E1BX57\_CHICK tr|R4GGA9|R4GGA9\_CHICK tr|E1C7N5|E1C7N5\_CHICK tr|E1BUW2|E1BUW2\_CHICK sp|Q5ZMB2|HEBP1\_CHICK tr|R4GK15|R4GK15\_CHICK tr|R4GG80|R4GG80\_CHICK tr|H9L136|H9L136\_CHICK tr|E1C251|E1C251\_CHICK tr|H9L3K4|H9L3K4\_CHICK tr|F1P4R7|F1P4R7\_CHICK tr|F1NHK1|F1NHK1\_CHICK tr|R4GJS5|R4GJS5\_CHICK tr|H9L2D0|H9L2D0\_CHICK tr|F1P5R6|F1P5R6\_CHICK tr|V5Y129|V5Y129\_CHICK tr|F1NJF9|F1NJF9\_CHICK tr|Q6L754|Q6L754\_CHICK tr|E1C496|E1C496\_CHICK tr|A0A0A0MQ34|A0A0A0MQ34\_CHICK sp|P56732|AVR2\_CHICK A2I7N3 tr|F1NCF4|F1NCF4\_CHICK tr|O42284|O42284\_CHICK tr|F1P0X7|F1P0X7\_CHICK tr|R4GFJ9|R4GFJ9\_CHICK tr|Q4U604|Q4U604\_CHICK tr|F1P2G0|F1P2G0\_CHICK tr|E3VWN1|E3VWN1\_CHICK tr|R4GGL2|R4GGL2\_CHICK tr|Q5XWT6|Q5XWT6\_CHICK sp|Q76LT9|TM258\_CHICK tr|F1P3K5|F1P3K5\_CHICK tr|R4GJA1|R4GJA1\_CHICK sp|Q9DEQ4|SFRP1\_CHICK tr|F1NPV5|F1NPV5\_CHICK tr|E1BWQ3|E1BWQ3\_CHICK tr|E1BR90|E1BR90\_CHICK tr|E1BXX8|E1BXX8\_CHICK tr|Q90637|Q90637\_CHICK tr|R4GLH8|R4GLH8\_CHICK tr|Q9PUF7|Q9PUF7\_CHICK tr|E1C3C4|E1C3C4\_CHICK tr|Q9W6I3|Q9W6I3\_CHICK tr|F6VIY8|F6VIY8\_CHICK tr|H9L0C8|H9L0C8\_CHICK tr|F1NDL6|F1NDL6\_CHICK tr|R4GH58|R4GH58\_CHICK tr|R4GFT4|R4GFT4\_CHICK sp|Q90805|CNG1\_CHICK tr|F1NEW7|F1NEW7\_CHICK tr|F1NE87|F1NE87\_CHICK tr|R4GLZ4|R4GLZ4\_CHICK tr|A5HUK2|A5HUK2\_CHICK tr|F1NXC2|F1NXC2\_CHICK tr|E1C7D6|E1C7D6\_CHICK tr|R4GFS8|R4GFS8\_CHICK tr|R4GGM9|R4GGM9\_CHICK tr|F1NTJ4|F1NTJ4\_CHICK tr|F1NS41|F1NS41\_CHICK sp|Q5F452|MTMR8\_CHICK tr|Q5ZHL5|Q5ZHL5\_CHICK tr|Q9DGG1|Q9DGG1\_CHICK tr|E1C7F9|E1C7F9\_CHICK tr|F1P5M4|F1P5M4\_CHICK sp|Q5ZMQ9|PEX5\_CHICK tr|Q6SVA7|Q6SVA7\_CHICK tr|E1C0N0|E1C0N0\_CHICK tr|B0LVG0|B0LVG0\_CHICK tr|F1NXH7|F1NXH7\_CHICK tr|E1BY34|E1BY34\_CHICK tr|F1P371|F1P371\_CHICK tr|E1C9B0|E1C9B0\_CHICK tr|Q5ZJX8|Q5ZJX8\_CHICK tr|F1NP73|F1NP73\_CHICK sp|P00174|CYB5\_CHICK tr|R4GGC2|R4GGC2\_CHICK tr|E1C6F1|E1C6F1\_CHICK tr|E1BVW0|E1BVW0\_CHICK tr|F6S2M1|F6S2M1\_CHICK tr|F1NV61|F1NV61\_CHICK tr|H9KZA6|H9KZA6\_CHICK tr|R4GM86|R4GM86\_CHICK tr|Q7LZS6|Q7LZS6\_CHICK tr|E1BWS4|E1BWS4\_CHICK tr|Q90650|Q90650\_CHICK tr|E1BQA3|E1BQA3\_CHICK tr|R4GGX3|R4GGX3\_CHICK tr|R4GIR4|R4GIR4\_CHICK tr|F1P0L7|F1P0L7\_CHICK tr|F1P2U5|F1P2U5\_CHICK tr|Q9DDU8|Q9DDU8\_CHICK tr|Q2XSZ6|Q2XSZ6\_CHICK tr|Q2XSZ7|Q2XSZ7\_CHICK tr|B2WUP1|B2WUP1\_CHICK tr|Q2XSZ4|Q2XSZ4\_CHICK tr|Q2XSY8|Q2XSY8\_CHICK tr|Q2XSY9|Q2XSY9\_CHICK tr|Q2XSY7|Q2XSY7\_CHICK tr|E1BVF8|E1BVF8\_CHICK tr|F1P3T4|F1P3T4\_CHICK tr|F1P462|F1P462\_CHICK tr|R4GHC9|R4GHC9\_CHICK tr|Q8QGF8|Q8QGF8\_CHICK tr|E3VVZ8|E3VVZ8\_CHICK tr|F1NQS9|F1NQS9\_CHICK tr|F1NLD4|F1NLD4\_CHICK sp|Q5ZJB4|IKKA\_CHICK tr|E1C6G7|E1C6G7\_CHICK tr|Q4GWN0|Q4GWN0\_CHICK tr|R4GJR0|R4GJR0\_CHICK tr|F1NLH5|F1NLH5\_CHICK tr|Q9PSQ6|Q9PSQ6\_CHICK tr|F1NAE0|F1NAE0\_CHICK tr|F1NLM6|F1NLM6\_CHICK tr|F1P0V3|F1P0V3\_CHICK tr|F1NMF2|F1NMF2\_CHICK tr|F1NC92|F1NC92\_CHICK tr|F1NSV7|F1NSV7\_CHICK sp|Q90643|IRF3\_CHICK tr|Q90ZD4|Q90ZD4\_CHICK tr|F1NAC6|F1NAC6\_CHICK tr|D0VX31|D0VX31\_CHICK sp|P49581|ACHA6\_CHICK tr|R4GF86|R4GF86\_CHICK sp|Q5ZHP5|CHM4B\_CHICK tr|F1P1G2|F1P1G2\_CHICK tr|R4GK36|R4GK36\_CHICK tr|R4GME8|R4GME8\_CHICK sp|Q5ZK35|MET14\_CHICK tr|C4PAQ9|C4PAQ9\_CHICK tr|C4PAP4|C4PAP4\_GALLA tr|C4PAQ5|C4PAQ5\_CHICK tr|C4PAR4|C4PAR4\_GALVA tr|Q2PYN4|Q2PYN4\_CHICK tr|C4PAR6|C4PAR6\_GALSO tr|F1NHZ5|F1NHZ5\_CHICK tr|Q90YK0|Q90YK0\_CHICK tr|Q5ZM18|Q5ZM18\_CHICK tr|F1NTH1|F1NTH1\_CHICK tr|F1P3Q6|F1P3Q6\_CHICK sp|Q9PW72|PDLI4\_CHICK tr|E1C4T5|E1C4T5\_CHICK tr|E1C025|E1C025\_CHICK tr|Q9I871|Q9I871\_CHICK tr|Q3MMY6|Q3MMY6\_CHICK tr|Q78DP6|Q78DP6\_CHICK tr|R4GFV4|R4GFV4\_CHICK tr|F1NTM3|F1NTM3\_CHICK tr|Q7LZ89|Q7LZ89\_CHICK tr|Q5ZMC4|Q5ZMC4\_CHICK sp|Q9IAL1|VSX2\_CHICK tr|F1NL82|F1NL82\_CHICK tr|R4GL28|R4GL28\_CHICK tr|Q5ZKY1|Q5ZKY1\_CHICK tr|Q009U5|Q009U5\_CHICK tr|E1C4S0|E1C4S0\_CHICK tr|B5BSS0|B5BSS0\_CHICK sp|Q5ZKL6|MBOA2\_CHICK tr|Q90X21|Q90X21\_CHICK tr|F1NVK4|F1NVK4\_CHICK tr|Q9YI70|Q9YI70\_CHICK tr|F1N8Y2|F1N8Y2\_CHICK tr|Q5ZKE0|Q5ZKE0\_CHICK tr|F1NJ25|F1NJ25\_CHICK tr|F1N9P9|F1N9P9\_CHICK tr|E1BSB1|E1BSB1\_CHICK tr|R4GL34|R4GL34\_CHICK tr|D5K9Y4|D5K9Y4\_CHICK tr|Q5ZLB6|Q5ZLB6\_CHICK tr|H9L1Y6|H9L1Y6\_CHICK tr|F1P1L8|F1P1L8\_CHICK tr|Q8AYG7|Q8AYG7\_CHICK tr|F1NDE0|F1NDE0\_CHICK sp|Q5ZI69|RM37\_CHICK tr|Q5ZJQ0|Q5ZJQ0\_CHICK tr|F1N9J1|F1N9J1\_CHICK tr|Q5ZIM0|Q5ZIM0\_CHICK tr|O93417|O93417\_CHICK tr|R4GJL3|R4GJL3\_CHICK tr|Q5ZJR6|Q5ZJR6\_CHICK tr|E1BYM2|E1BYM2\_CHICK tr|Q5F3Q9|Q5F3Q9\_CHICK tr|D2SYX9|D2SYX9\_CHICK tr|R4GFP2|R4GFP2\_CHICK tr|Q9PVL2|Q9PVL2\_CHICK tr|B3GSV0|B3GSV0\_CHICK tr|B3VKP4|B3VKP4\_CHICK tr|E1C1U7|E1C1U7\_CHICK tr|F1NYW3|F1NYW3\_CHICK tr|R4GH62|R4GH62\_CHICK tr|Q8QGG9|Q8QGG9\_CHICK tr|R4GM45|R4GM45\_CHICK sp|Q5F3X0|LCLT1\_CHICK tr|F1NWG8|F1NWG8\_CHICK tr|E1BRB7|E1BRB7\_CHICK tr|O93573|O93573\_CHICK sp|Q5ZJJ8|UBCP1\_CHICK tr|Q5ZJG2|Q5ZJG2\_CHICK tr|F6VMJ6|F6VMJ6\_CHICK tr|F1NT28|F1NT28\_CHICK tr|Q8JIG5|Q8JIG5\_CHICK sp|Q5ZLR1|WLS\_CHICK Q9TTE1 tr|F1P154|F1P154\_CHICK tr|F1P376|F1P376\_CHICK tr|R4GK27|R4GK27\_CHICK tr|F1NU90|F1NU90\_CHICK tr|F1NM15|F1NM15\_CHICK tr|B8YLU7|B8YLU7\_GALLA sp|P26652|TIMP3\_CHICK tr|B4X7M8|B4X7M8\_CHICK tr|B8YLU1|B8YLU1\_GALSO tr|B8YLV6|B8YLV6\_CHICK tr|R4GLB3|R4GLB3\_CHICK tr|Q5ZHU3|Q5ZHU3\_CHICK tr|R4GFR4|R4GFR4\_CHICK sp|Q9PU45|RADI\_CHICK tr|F1NKA3|F1NKA3\_CHICK sp|P27118|DCOR\_CHICK tr|E1BV82|E1BV82\_CHICK tr|F1NB91|F1NB91\_CHICK tr|E1BZ65|E1BZ65\_CHICK tr|E1C2R3|E1C2R3\_CHICK tr|Q8QGD9|Q8QGD9\_CHICK tr|Q2WFZ8|Q2WFZ8\_CHICK tr|F1NMY4|F1NMY4\_CHICK tr|Q9PTH0|Q9PTH0\_CHICK tr|Q5ZJG7|Q5ZJG7\_CHICK tr|F1NC39|F1NC39\_CHICK tr|R4GI31|R4GI31\_CHICK tr|E6N1V3|E6N1V3\_CHICK tr|Q0P0G7|Q0P0G7\_CHICK tr|A5HUK1|A5HUK1\_CHICK tr|E1C864|E1C864\_CHICK tr|R4GIN8|R4GIN8\_CHICK sp|Q5ZMT9|INSI1\_CHICK tr|F1NVN1|F1NVN1\_CHICK tr|E1C068|E1C068\_CHICK tr|R4GIA3|R4GIA3\_CHICK tr|E1BYC8|E1BYC8\_CHICK tr|E1BTT1|E1BTT1\_CHICK sp|E1C5V0|SMYD2\_CHICK tr|A0A0A0MQ47|A0A0A0MQ47\_CHICK tr|R4GHI8|R4GHI8\_CHICK tr|F1NF85|F1NF85\_CHICK sp|P09645|TBA8\_CHICK tr|R4GHY3|R4GHY3\_CHICK tr|F1ND74|F1ND74\_CHICK sp|P47838|RS6\_CHICK tr|F7BYQ2|F7BYQ2\_CHICK tr|Q8
[truncated: 386,993 more chars]
