# Supplementary material for: Phospho-proteomic analyses of B-Raf protein complexes reveal new regulatory principles
Source: Oncotarget. 2016 Mar 28;7(18):26628–52. doi: 10.18632/oncotarget.8427 (PMC5042004; doi:10.18632/oncotarget.8427)
Supplement: Supplementary file 9 [file oncotarget-07-26628-s009.zip › Supplementary File 8.html]

PepView


| Peptide View (Result) | | | | |
| --- | --- | --- | --- | --- |
| MS File Choice | | Protein Choice | Filter Options | Display Options |
| --- | --- | --- | --- | --- |
| Available MS Files:   tempfile (F006954) BSA Papain Verdau mit Cystein .temp (F011905) tempfile (F003591) tempfile (F003720) tempfile (F003586) tempfile (F003491) Labeled H3 (Trypsine).temp (F008966) tempfile (F003703) tempfile (F003438) tempfile (F003301) HAT3 KO\_Middle\_Thermolysine.temp (F011289) tempfile (F003634) tempfile (F008182) tempfile (F005202) tempfile (F006796) tempfile (F003121) tempfile (F006475) tempfile (F003710) tempfile (F003640) tempfile (F006689) tempfile (F002291) Heavy Acetylated TY1-H2A.temp (F016322) tempfile (F003574) tempfile (F005140) tempfile (F002239) tempfile (F006758) tempfile (F002596)  (F020404) tempfile (F003996) tempfile (F003212) tempfile (F002247) tempfile (F007734) tempfile (F001697) HAT2 KD\_48hr.temp (F011227) tempfile (F006785) tempfile (F003160) tempfile (F003217) tempfile (F016299) No0181\_Trypsin\_ETD.temp (F009585) tempfile (F003993) tempfile (F006961) tempfile (F001728) tempfile (F005307) HAT3 KO\_Lower\_Elastase.temp (F011232) tempfile (F006777) tempfile (F005606) tempfile (F006245) tempfile (F002254) tempfile (F006807) tempfile (F005576) tempfile (F005210) tempfile (F003425) tempfile (F006801) tempfile (F006778) tempfile (F002627) tempfile (F002281)  (F020491)  (F020402) tempfile (F002600) tempfile (F005580) tempfile (F003428) Light Acetylated H3\_Quan.temp (F010891) tempfile (F003709) tempfile (F007298) tempfile (F008561) tempfile (F001560) tempfile (F005669) tempfile (F001620) tempfile (F002236) tempfile (F002632) tempfile (F003628) HAT2 KD\_0hr.temp (F011178) tempfile (F003744)  (F016879) tempfile (F006771) tempfile (F003303) tempfile (F002305) HAT2 KD\_24hr.temp (F011183) tempfile (F006573) tempfile (F005613) tempfile (F008602) tempfile (F003431) tempfile (F002556) tempfile (F007878) tempfile (F001901) tempfile (F003584) tempfile (F001564) tempfile (F005608) tempfile (F006953) HAT2 KD\_24hr.temp (F011182) BSA Papain Verdau ohne Cystein .temp (F011904) tempfile (F003978) tempfile (F003981) tempfile (F003617) tempfile (F002288)  (F016877) tempfile (F003581) tempfile (F005157) tempfile (F002300) tempfile (F005582) tempfile (F001908)  (F020380) tempfile (F007877) tempfile (F009649) tempfile (F006830) tempfile (F003422) HAT2 KD\_48hr.temp (F011219) HAT2 KD\_24h.temp (F011294) tempfile (F003635) tempfile (F003702) tempfile (F006792) tempfile (F003493) HK-2 Histones NOB.temp (F009415) tempfile (F003714) tempfile (F003302) tempfile (F005204) tempfile (F003309) tempfile (F006233) tempfile (F006815) tempfile (F007301) tempfile (F003708) Labeled H3 (Trypsine).temp (F009311) tempfile (F007299) tempfile (F002253) tempfile (F003161) tempfile (F001696) tempfile (F009648) tempfile (F005597) tempfile (F016248) tempfile (F006763) tempfile (F001561) tempfile (F001893) tempfile (F002282) tempfile (F010795) tempfile (F005581) tempfile (F003649) tempfile (F003740) tempfile (F010803) tempfile (F006802) tempfile (F002248) tempfile (F002610) HAT2 KD\_0hr.temp (F011179) tempfile (F006770) tempfile (F002241) tempfile (F002628) tempfile (F006234) Light Acetylated H3\_Quan.temp (F011075) tempfile (F008566) tempfile (F005607) tempfile (F002289) tempfile (F002306) tempfile (F006795) tempfile (F006808) tempfile (F005575) tempfile (F003743) tempfile (F003053) tempfile (F016290) tempfile (F002633) tempfile (F007916) tempfile (F005571) tempfile (F006779) tempfile (F006784) tempfile (F003690) tempfile (F006963) tempfile (F003300) tempfile (F005073) tempfile (F002563) tempfile (F003218) tempfile (F003984) tempfile (F003976) tempfile (F008603) tempfile (F003578) tempfile (F003643) tempfile (F003918) No0181\_Trypsin\_HCD.temp (F009586) tempfile (F003742) tempfile (F005212) tempfile (F006831) tempfile (F003741) tempfile (F003696) tempfile (F009642) tempfile (F002290) tempfile (F001622) tempfile (F005593) tempfile (F010786) tempfile (F002287) Light Acetylated H3\_Thermolysin.temp (F010890) tempfile (F010783) tempfile (F002235) tempfile (F001619) Labeled H3 (Trypsine).temp (F009155) tempfile (F002303) tempfile (F002624) tempfile (F003637) tempfile (F003159) tempfile (F003636) tempfile (F002565) tempfile (F003980) tempfile (F002566) tempfile (F008606) tempfile (F005531) HK-2 Histones Ctrl.temp (F009414) tempfile (F006794) tempfile (F001884) tempfile (F002551) tempfile (F002570) tempfile (F001699) tempfile (F001725) tempfile (F006956) tempfile (F006765) tempfile (F003423) tempfile (F003116) tempfile (F009730) tempfile (F016283)  (F020406) tempfile (F006797) tempfile (F005667) tempfile (F008563) tempfile (F016249) tempfile (F002602) tempfile (F002598) tempfile (F003701) tempfile (F003097) tempfile (F003693) tempfile (F002634) tempfile (F006783) tempfile (F003420) tempfile (F006235) tempfile (F006832) tempfile (F003712) tempfile (F005596) tempfile (F005297) tempfile (F012138) Light Acetylated H3\_Quan.temp (F011074) tempfile (F001551) tempfile (F006962) tempfile (F002564) tempfile (F001695) tempfile (F005572) tempfile (F003589) tempfile (F006762) HAT2 KD\_48hr.temp (F011185) tempfile (F002286) tempfile (F003579) tempfile (F003162) tempfile (F009643) tempfile (F006809) tempfile (F003294) tempfile (F008604) tempfile (F006782) tempfile (F006476) tempfile (F002299) tempfile (F003176) tempfile (F005301) tempfile (F012141) tempfile (F006955)  (F020401) tempfile (F003921) tempfile (F006800) tempfile (F006789) tempfile (F003642) tempfile (F010784) tempfile (F002552) tempfile (F003429) tempfile (F005605) tempfile (F012139) tempfile (F001885) tempfile (F003644) tempfile (F003592) tempfile (F005309) tempfile (F002242) tempfile (F001910) tempfile (F003308) tempfile (F003721) tempfile (F005612) tempfile (F008283) tempfile (F002302) No0181\_Elastase\_ETD.temp (F009583) tempfile (F001698) tempfile (F001553) tempfile (F006244) Labeled H3 50% (Elastase).temp (F008967) tempfile (F003638) tempfile (F008780) tempfile (F008605) tempfile (F005602) tempfile (F002571) tempfile (F002601) HAT2 KD\_0hr.temp (F011177) tempfile (F005598) tempfile (F002597) tempfile (F001892) tempfile (F003715) tempfile (F003430) tempfile (F006236) tempfile (F002250) tempfile (F006764) tempfile (F002298)  (F016878) HAT2 KD\_24hr.temp (F011184) tempfile (F005103) tempfile (F006755) tempfile (F002234) tempfile (F003692) tempfile (F005609) tempfile (F003641) tempfile (F012137) tempfile (F003098) tempfile (F002560) tempfile (F005600) tempfile (F001535) tempfile (F003576) tempfile (F005300)  (F020408) tempfile (F003216) HAT2 KD\_48h.temp (F011317) tempfile (F006966) tempfile (F003931) RE 50pcAc light, 50pcAc heavy.temp (F009309) Light Acetylated H3\_Quan.temp (F011076) tempfile (F003580) tempfile (F003307) Light Acetylated H3\_Quan.temp (F010887) tempfile (F003421) tempfile (F003645) tempfile (F005303) tempfile (F006774) tempfile (F016301) tempfile (F009741) tempfile (F006951) tempfile (F010802) tempfile (F006806) tempfile (F003651) tempfile (F003722) tempfile (F002243) tempfile (F003718) tempfile (F003435) tempfile (F003177) tempfile (F016300) tempfile (F002568) tempfile (F002284) Labeled H3 (Trypsine).temp (F009156) tempfile (F003639) tempfile (F009742)  Heavy Acetylated TY1-H2A.temp (F016324) tempfile (F006246) tempfile (F006834) tempfile (F003306) tempfile (F016302) tempfile (F003650) No0181\_Elastase\_HCD.temp (F009584) tempfile (F001552) Light Acetylated H3\_Quan.temp (F010895) tempfile (F005665) tempfile (F006958) tempfile (F003618) tempfile (F002304) tempfile (F016247)  Heavy Acetylated TY1-H2A.temp (F016323) tempfile (F003440) tempfile (F006964) tempfile (F006241) tempfile (F005573) tempfile (F005579) tempfile (F006767) tempfile (F001886) tempfile (F005585) HAT3 KO\_Upper\_Elastase.temp (F011230) tempfile (F001567) tempfile (F003698) tempfile (F002626) tempfile (F002301) tempfile (F008284) tempfile (F002285) tempfile (F002297) tempfile (F005124) tempfile (F005136) tempfile (F003114) HAT2 KD\_24h.temp (F011311) tempfile (F003695) tempfile (F002567) tempfile (F006766) tempfile (F010785) tempfile (F002611) tempfile (F002244) tempfile (F002629) tempfile (F005614) tempfile (F006754) HAT2 KD\_24h.temp (F011312) tempfile (F002608) tempfile (F003725)  (F020377) tempfile (F006829) tempfile (F003691) tempfile (F003213) tempfile (F006952)  (F020403) tempfile (F006780) tempfile (F003975) tempfile (F016289) tempfile (F005302) tempfile (F002623) tempfile (F002554) tempfile (F016291) tempfile (F006833) tempfile (F003096) tempfile (F003295) tempfile (F006237) tempfile (F003987) tempfile (F006479) tempfile (F002630)  (F020405) Light Acetylated H3\_Elastase.temp (F010886) tempfile (F001730) tempfile (F006571) tempfile (F006757) tempfile (F003697) tempfile (F006788) tempfile (F003723) tempfile (F003624) tempfile (F005532) tempfile (F001887) tempfile (F001854) tempfile (F003646) tempfile (F006791) tempfile (F006965) tempfile (F003113) tempfile (F003713) tempfile (F016284) tempfile (F008562) tempfile (F008285) Light Acetylated H3\_Proteinase K.temp (F010894) tempfile (F009740) tempfile (F003694) tempfile (F005143) tempfile (F002599) tempfile (F006810) tempfile (F006238) tempfile (F006957) tempfile (F003700) tempfile (F005298) tempfile (F006572) tempfile (F006798) tempfile (F003051) tempfile (F016250) tempfile (F010807) tempfile (F005584) tempfile (F006242) tempfile (F009744) tempfile (F005670) tempfile (F001894) tempfile (F008564) tempfile (F012140) tempfile (F002245) tempfile (F001700) HAT3 KO\_Middle\_Elastase.temp (F011231) tempfile (F003484) tempfile (F006761) tempfile (F006803) tempfile (F003989) tempfile (F006787) Labeled H3 50% (Thermolysine).temp (F008968) tempfile (F005304) tempfile (F001722) tempfile (F003588) tempfile (F002603) tempfile (F008600) tempfile (F003724) tempfile (F003926) tempfile (F003178) tempfile (F006773) tempfile (F010805)  (F020397) tempfile (F006959) tempfile (F006472) tempfile (F005142) tempfile (F002252) tempfile (F002249) tempfile (F006814) tempfile (F003997) tempfile (F003492) tempfile (F005139) tempfile (F003625) tempfile (F002555) HAT2 KD\_0h.temp (F011293) tempfile (F007915) tempfile (F002595) tempfile (F001729) tempfile (F003071) tempfile (F002240) tempfile (F002609) tempfile (F007300) tempfile (F001895) tempfile (F016286) tempfile (F006781) tempfile (F003310) tempfile (F001914) tempfile (F002558) tempfile (F003593) tempfile (F006775) tempfile (F001536) tempfile (F001554)  (F020400) HAT2 KD\_48h.temp (F011313)  (F020396) tempfile (F003211) tempfile (F006239) tempfile (F008565) tempfile (F003582) tempfile (F002238) tempfile (F002561) BSA Papain Verdau ohne Cystein .temp (F011903) tempfile (F001855) tempfile (F007882) tempfile (F002251) tempfile (F002625) tempfile (F005299)  (F020407) tempfile (F009743) tempfile (F006799) tempfile (F003719) tempfile (F006243) tempfile (F006811) tempfile (F001621) tempfile (F006804) tempfile (F010806) tempfile (F003590) tempfile (F010808) tempfile (F003707) tempfile (F003699) tempfile (F003711) tempfile (F003998) tempfile (F005601) tempfile (F012136) HAT3 KO\_Upper\_Thermolysine.temp (F011288) tempfile (F005611) tempfile (F006759) HAT2 KD\_0h.temp (F011292) tempfile (F003587) tempfile (F002246) tempfile (F006756) tempfile (F006753) tempfile (F005305) tempfile (F003434) tempfile (F006790) tempfile (F003647) tempfile (F001723) tempfile (F001570) tempfile (F003988) tempfile (F005595) tempfile (F003304) tempfile (F004976) tempfile (F005570) tempfile (F006772) tempfile (F007733) tempfile (F006805) tempfile (F003626) tempfile (F003575) tempfile (F009739) tempfile (F006786) tempfile (F005141) HAT2 KD\_0h.temp (F011291) tempfile (F003054) tempfile (F005306) Rasha.temp (F008810) tempfile (F003648) tempfile (F008762) tempfile (F006769) tempfile (F002237) tempfile (F006960) tempfile (F003439) tempfile (F016292) tempfile (F001557) HAT3 KO\_Lower\_Thermolysine.temp (F011290) tempfile (F007827) tempfile (F006768) tempfile (F001857) tempfile (F008601) tempfile (F006793) tempfile (F002569) tempfile (F003112) tempfile (F008286) tempfile (F005583) tempfile (F009650) tempfile (F001856) tempfile (F002557) tempfile (F006760) tempfile (F010794) tempfile (F006812) tempfile (F005577) tempfile (F006240) HAT2 KD\_48h.temp (F011314) tempfile (F002631) tempfile (F005610) tempfile (F003982) tempfile (F003424) tempfile (F016285) tempfile (F006813) LCMS001711 (F020343) LCMS001834 (F020409) LCMS001835 (F020410) LCMS001836 (F020411) LCMS001841 (F020413) 01\_velos\_2700 (F004434) 01\_velos\_2700 (F004435) 01\_velos\_2701 (F004437) 01\_velos\_2701 (F004436) 01\_velos\_2702 (F004438) 01\_velos\_2703 (F004439) 01\_velos\_2704 (F004440) 01\_velos\_2704 (F004441) 01\_velos\_2705 (F004443) 01\_velos\_2705 (F004442) 01\_velos\_2708 (F004445) 01\_velos\_2708 (F004444) 01\_velos\_2709 (F004447) 01\_velos\_2709 (F004446) 01\_velos\_2710 (F004448) 01\_velos\_2711 (F004449) 01\_velos\_2712 (F004450) 01\_velos\_2712 (F004451) 01\_velos\_2713 (F004452) 01\_velos\_2713 (F004453) 06\_Fusion\_0164 (F017353) 06\_Fusion\_0165 (F017354) 06\_Fusion\_0166 (F017355) 06\_velos\_2737 (F004554) 06\_velos\_2738 (F004556) 06\_velos\_2739 (F004558) 06\_velos\_2740 (F004559) 06\_velos\_2741 (F004560) 06\_velos\_2742 (F004562) 06\_velos\_2745 (F004564) 06\_velos\_2746 (F004566) 06\_velos\_2747 (F004568) 06\_velos\_2748 (F004569) 06\_velos\_2749 (F004570) 06\_velos\_2750 (F004572) 15\_velos\_2834 (F004656) 15\_velos\_2834 (F004657) 15\_velos\_2835 (F004659) 15\_velos\_2835 (F004658) 15\_velos\_2836 (F004660) 15\_velos\_2837 (F004661) 18\_Fusion\_0252 (F017360) 18\_Fusion\_0253 (F017361) 19\_velos\_2535 (F004266) 19\_velos\_2536 (F004267) 19\_velos\_2537 (F004268) 19\_velos\_2538 (F004269) 0021\_QTOF\_0254 (F020414) 0021\_QTOF\_0256 (F020415) 21\_Fusion\_0316 (F017369) 21\_Fusion\_0317 (F017370) 21\_Fusion\_0318 (F017371) 21\_velos\_2594 (F004307) 21\_velos\_2595 (F004308) 21\_velos\_2596 (F004309) 21\_velos\_2597 (F004310) 22\_velos\_2608 (F004431) 22\_velos\_2609 (F004315) 22\_velos\_2610 (F004316) 22\_velos\_2611 (F004432) 22\_velos\_2612 (F004318) 22\_velos\_2613 (F004319) 22\_velos\_2614 (F004433) 22\_velos\_2615 (F004321) 22\_velos\_2616 (F004322) 22\_velos\_2617 (F004392) 22\_velos\_2618 (F004324) 22\_velos\_2619 (F004325) 23\_velos\_1609 (F003507) 23\_velos\_1610 (F003513) 23\_velos\_1611 (F003610) 23\_velos\_1612 (F003512) 23\_velos\_1613 (F003508) 23\_velos\_1614 (F003511) 26\_JV\_0200 (F001498) 26\_JV\_0201 (F001500) 26\_velos\_2672 (F004541) 26\_velos\_2673 (F004542) 26\_velos\_2674 (F004543) 26\_velos\_2675 (F004544) 26\_velos\_2676 (F004545) 26\_velos\_2677 (F004546) 26\_velos\_2685 (F004394) 26\_velos\_2686 (F004396) 26\_velos\_2687 (F004397) 26\_velos\_2688 (F004398) 27\_JV\_0219 (F001534) 29\_velos\_1722 (F003376) 29\_velos\_1723 (F003399) 29\_velos\_1724 (F003407) 29\_velos\_1725 (F003400) 0035\_QTOF\_0374 (F020416) 0035\_QTOF\_0376 (F020418) 0063\_TRAP\_00801 (F020458) 0063\_TRAP\_00802 (F020459) 0121\_QTOF\_1687 (F020442) 0171\_TRAP\_02202 (F020470) 0195\_TRAP\_02646 (F020472) 0195\_TRAP\_02665 (F020471) 0204\_TRAP\_02780 (F020478) 0204\_TRAP\_02782 (F020479) 0305\_QTOF\_4317 (F020446) 0305\_QTOF\_4318 (F020447) 0348\_QTOF\_5076 (F020482) 0348\_QTOF\_5078 (F020483) 0348\_QTOF\_5096 (F020480) 0348\_QTOF\_5098 (F020481) 1:1 Acetylated H3 (Trypsine).temp (F009289) 06 (F017359) 06 (F017356) 21 (F017990) 21 (F018005) 21 (F018010) 21 (F017996) 21 (F018007) 21 (F017991) 21 (F018002) 21 (F017997) 21 (F018011) 21 (F018006) 21 (F017981) 21 (F017998) 21 (F019355) 21 (F017416) 21 (F017984) 21 (F018000) 21 (F017993) 21 (F018009) 21 (F017982) 21 (F017983) 21 (F017999) 21 (F018008) 21 (F018001) 21 (F017985) 21 (F017417) 21 (F019354) 21 (F017977) 21 (F017995) 21 (F018003) 21 (F017976) 21 (F017992) 21 (F017979) 21 (F017989) 21 (F017418) 21 (F017988) 21 (F019359) 21 (F017987) 21 (F019360) 21 (F017986) 21 (F017980) 21 (F018004) 21 (F017978) 21 (F017994) 50% Acetylated H3\_E 2.temp (F009745) 50% Acetylated H3\_All-in-one method (Trypsin).temp (F009504) 50% Acetylated H3\_Elastase.temp (F010996) 50% Acetylated H3\_Elastase.temp (F010760) 50% Acetylated H3\_Th 2.temp (F009749) 50% Acetylated H3\_Thermolysine.temp (F010761) 50% Acetylated H3\_Elastase.temp (F012673) 50% Heavy Acetylated H3\_Elastase (1st).temp (F012765) 50% Heavy Acetylated H3\_Papain (1st).temp (F012767) 0211\_TRAP\_02862.D (F020473) 0212\_TRAP\_02884.D (F020474) 328\_QTOF\_4752 - F017450 (F020493) 2013-01-15\_velos\_2171\_dPER\_dTIM only (F004129) 2013-01-17\_velos\_2223\_CSF\_Tryp\_ETD\_only\_PLK4\_AGMS (F004077) 2013-01-15\_velos\_2187\_dPER\_dTIM only (F004132) 2013-01-17\_velos\_2231\_Int\_Thermo\_HCD\_only\_PLK4\_AGMS (F004084) 2013-01-17\_velos\_2217\_M\_Thermo\_HCD\_onlyPLK4\_AGMS (F004072) 2013-01-17\_velos\_2229\_Int\_Trypsin\_HCD\_only\_PLK4\_AGMS (F004082) 2013-01-17\_velos\_2230\_Int\_Trypsin\_ETD\_only\_PLK4\_AGMS (F004083) 2013-01-15\_velos\_2188\_dPER\_dTIM only (F004131) 2013-01-17\_velos\_2215\_M\_Tryp\_HCD\_onlyPLK4\_AGMS (F004070) 2013-01-17\_velos\_2216\_M\_Tryp\_ETD\_onlyPLK4\_AGMS (F004071) 2013-01-17\_velos\_2227\_CSF\_Elastase\_ETD\_only\_PLK4\_AGMS (F004081) 2013-01-15\_velos\_2185\_5 \_dPER\_dTIM only (F004127) 2013-01-17\_velos\_2226\_CSF\_Elastase\_HCD\_only\_PLK4\_AGMS (F004079) 2013-01-17\_velos\_2225\_CSF\_Thermo\_ETD\_only\_PLK4\_AGMS (F004080) 2013-01-17\_velos\_2219\_M\_Elastase\_HCD\_onlyPLK4\_AGMS (F004074) 2013-01-15\_velos\_2186-dPER\_dTIM only (F004130) 2013-01-17\_velos\_2234\_Int\_Elastase\_ETD\_only\_PLK4\_AGMS (F004087) 2013-01-17\_velos\_2218\_M\_Thermo\_ETD\_onlyPLK4\_AGMS (F004073) 2013-01-17\_velos\_2222\_CSF\_Tryp\_HCD\_only\_PLK4\_AGMS (F004076) 2013-01-17\_velos\_2233\_Int\_Elastase\_HCD\_only\_PLK4\_AGMS (F004086) 2013-01-17\_velos\_2220\_M\_Elastase\_ETD\_only\_PLK4\_AGMS (F004075) 2013-01-17\_velos\_2232\_Int\_Thermo\_ETD\_only\_PLK4\_AGMS (F004085) 2013-01-17\_velos\_2224\_CSF\_Thermo\_HCD\_only\_PLK4\_AGMS (F004078) 2013-01-15\_velos\_2170\_dPER\_dTIM only (F004128) | |  |  |  | | --- | --- | --- | | MS Files: |  | Color: | |  | | | | 0121\_QTOF\_1687 (F020442) |  |  | | 0305\_QTOF\_4317 (F020446) |  |  | | 0305\_QTOF\_4318 (F020447) |  |  | | 0063\_TRAP\_00801 (F020458) |  |  | | 0063\_TRAP\_00802 (F020459) |  |  | | 0171\_TRAP\_02202 (F020470) |  |  | | 0195\_TRAP\_02665 (F020471) |  |  | | 0211\_TRAP\_02862.D (F020473) |  |  | | 0212\_TRAP\_02884.D (F020474) |  |  | | (F020491) |  |  | | 328\_QTOF\_4752 - F017450 (F020493) |  |  | | |  | | --- | | sp|X004711| sp|P15056|BRAF\_HUMAN tr|H7C560|H7C560\_HUMAN tr|H7C5K3|H7C5K3\_HUMAN tr|H7C4S5|H7C4S5\_HUMAN sp|P04049|RAF1\_HUMAN tr|H7C155|H7C155\_HUMAN sp|P10398|ARAF\_HUMAN tr|Q96II5|Q96II5\_HUMAN sp|Q8WXI7|MUC16\_HUMAN tr|B5ME49|B5ME49\_HUMAN sp|P04264|K2C1\_HUMAN P04264 sp|Q8WZ42|TITIN\_HUMAN P13645 sp|P13645|K1C10\_HUMAN sp|P35908|K22E\_HUMAN P35908 sp|Q685J3|MUC17\_HUMAN sp|Q7Z5P9|MUC19\_HUMAN tr|E7EPM4|E7EPM4\_HUMAN P00761 sp|P08238|HS90B\_HUMAN sp|Q9NR30|DDX21\_HUMAN sp|P35527|K1C9\_HUMAN P35527 sp|Q8NET4|RGAG1\_HUMAN ENSEMBL:ENSBTAP00000038253 P02535-1 tr|A6NP22|A6NP22\_HUMAN tr|A0A087X0N9|A0A087X0N9\_HUMAN sp|P07900|HS90A\_HUMAN sp|P04259|K2C6B\_HUMAN sp|Q58FF7|H90B3\_HUMAN sp|Q86UQ4|ABCAD\_HUMAN P02533 sp|P02533|K1C14\_HUMAN sp|Q9Y6V0|PCLO\_HUMAN P02538 sp|P02538|K2C6A\_HUMAN P13647 sp|P13647|K2C5\_HUMAN P48668 sp|P48668|K2C6C\_HUMAN P04259 sp|Q9NR09|BIRC6\_HUMAN Q922U2 sp|Q8NF91|SYNE1\_HUMAN sp|Q02505|MUC3A\_HUMAN Q3TTY5 tr|E7ENC5|E7ENC5\_HUMAN tr|E9PDY6|E9PDY6\_HUMAN tr|E7EWN1|E7EWN1\_HUMAN tr|E7ENN3|E7ENN3\_HUMAN sp|Q8IZF6|GP112\_HUMAN sp|Q7Z3Y7|K1C28\_HUMAN Q7Z3Y7 Q148H6 sp|Q9HC84|MUC5B\_HUMAN Q6IFZ6 Q5XQN5 sp|Q8WXG9|GPR98\_HUMAN sp|Q58FF8|H90B2\_HUMAN sp|Q5CZC0|FSIP2\_HUMAN tr|J3QTJ6|J3QTJ6\_HUMAN tr|K7EQQ3|K7EQQ3\_HUMAN tr|E7EQG8|E7EQG8\_HUMAN tr|E7EW47|E7EW47\_HUMAN sp|Q9UKN1|MUC12\_HUMAN sp|Q9UPN3|MACF1\_HUMAN tr|E7EQT2|E7EQT2\_HUMAN tr|E7ETT5|E7ETT5\_HUMAN sp|Q8N3K9|CMYA5\_HUMAN tr|H3BQK9|H3BQK9\_HUMAN tr|H3BPE1|H3BPE1\_HUMAN tr|E7ERK0|E7ERK0\_HUMAN tr|E7EUL9|E7EUL9\_HUMAN sp|Q7Z3Y8|K1C27\_HUMAN Q7Z3Y8 P08779 sp|P08779|K1C16\_HUMAN A2A4G1 sp|P04114|APOB\_HUMAN sp|Q6V0I7|FAT4\_HUMAN Q8VED5 Q7Z3Z0 sp|Q7Z3Z0|K1C25\_HUMAN Q9Z2K1 Q3ZAW8 tr|A0A087WTM7|A0A087WTM7\_HUMAN tr|A0A087X1N7|A0A087X1N7\_HUMAN sp|Q63HR2|TENC1\_HUMAN tr|F8VV64|F8VV64\_HUMAN sp|E2RYF6|MUC22\_HUMAN sp|Q9P225|DYH2\_HUMAN sp|Q7Z794|K2C1B\_HUMAN Q7Z794 tr|A6NGQ3|A6NGQ3\_HUMAN tr|H3BPX2|H3BPX2\_HUMAN sp|O75445|USH2A\_HUMAN sp|Q8TCU4|ALMS1\_HUMAN sp|E9PAV3|NACAM\_HUMAN tr|A0A087WZY3|A0A087WZY3\_HUMAN tr|A0A087WTU9|A0A087WTU9\_HUMAN sp|P20929|NEBU\_HUMAN sp|Q15149|PLEC\_HUMAN sp|Q96RW7|HMCN1\_HUMAN tr|H7BY35|H7BY35\_HUMAN sp|Q92736|RYR2\_HUMAN sp|P21817|RYR1\_HUMAN sp|Q9UPA5|BSN\_HUMAN sp|Q9NYQ7|CELR3\_HUMAN Q04695 Q9QWL7 sp|Q04695|K1C17\_HUMAN sp|Q5VST9|OBSCN\_HUMAN tr|A0A096LPK4|A0A096LPK4\_HUMAN sp|Q03164|KMT2A\_HUMAN sp|Q86XX4|FRAS1\_HUMAN P50446 tr|A0A087WV20|A0A087WV20\_HUMAN sp|Q14690|RRP5\_HUMAN tr|Q5T9W8|Q5T9W8\_HUMAN sp|Q99250|SCN2A\_HUMAN Q6NXH9 sp|P53350|PLK1\_HUMAN Q8BGZ7 tr|A0A087WVP1|A0A087WVP1\_HUMAN sp|Q14517|FAT1\_HUMAN sp|Q6ZQQ2|S31D1\_HUMAN tr|A6NEM2|A6NEM2\_HUMAN sp|O15357|SHIP2\_HUMAN sp|Q96L91|EP400\_HUMAN sp|Q4G0P3|HYDIN\_HUMAN sp|O75592|MYCB2\_HUMAN sp|Q8IVF4|DYH10\_HUMAN tr|E7EMW7|E7EMW7\_HUMAN sp|O95071|UBR5\_HUMAN sp|Q8WXH0|SYNE2\_HUMAN sp|O95359|TACC2\_HUMAN sp|Q8IWI9|MGAP\_HUMAN Q9R0H5 sp|Q7Z7G8|VP13B\_HUMAN sp|Q9P2P6|STAR9\_HUMAN sp|P98088|MUC5A\_HUMAN sp|Q8TDX9|PK1L1\_HUMAN sp|Q96T58|MINT\_HUMAN sp|Q5T5U3|RHG21\_HUMAN sp|Q5THJ4|VP13D\_HUMAN tr|F5GWP8|F5GWP8\_HUMAN sp|P51610|HCFC1\_HUMAN sp|Q49A88|CCD14\_HUMAN tr|E9PBC6|E9PBC6\_HUMAN sp|Q9Y6N7|ROBO1\_HUMAN tr|G3V5X4|G3V5X4\_HUMAN sp|Q86WI1|PKHL1\_HUMAN tr|F5GXR6|F5GXR6\_HUMAN tr|A0A075B6F5|A0A075B6F5\_HUMAN sp|Q6ZNJ1|NBEL2\_HUMAN sp|Q7Z6Z7|HUWE1\_HUMAN tr|J3QT39|J3QT39\_HUMAN sp|Q8IVF2|AHNK2\_HUMAN sp|Q01546|K22O\_HUMAN sp|Q92793|CBP\_HUMAN tr|J3KPF0|J3KPF0\_HUMAN tr|F8VWT9|F8VWT9\_HUMAN sp|Q9H4A3|WNK1\_HUMAN sp|Q9BXA9|SALL3\_HUMAN sp|Q14568|HS902\_HUMAN sp|Q8IUG5|MY18B\_HUMAN sp|P18583|SON\_HUMAN tr|E7EMZ9|E7EMZ9\_HUMAN P19013 tr|A0A087WW76|A0A087WW76\_HUMAN sp|Q2LD37|K1109\_HUMAN sp|Q15751|HERC1\_HUMAN sp|Q58FF6|H90B4\_HUMAN sp|A6NMZ7|CO6A6\_HUMAN sp|P08F94|PKHD1\_HUMAN sp|Q96Q15|SMG1\_HUMAN sp|Q8NEZ4|KMT2C\_HUMAN sp|Q9Y4D8|HECD4\_HUMAN sp|P22105|TENX\_HUMAN tr|J3KRA9|J3KRA9\_HUMAN tr|A0A087WTM1|A0A087WTM1\_HUMAN sp|Q9UQ35|SRRM2\_HUMAN sp|Q14204|DYHC1\_HUMAN tr|G3V2J8|G3V2J8\_HUMAN sp|Q6P4R8|NFRKB\_HUMAN tr|H9KVB4|H9KVB4\_HUMAN sp|O15417|TNC18\_HUMAN tr|I6L894|I6L894\_HUMAN sp|Q01484|ANK2\_HUMAN sp|Q12955|ANK3\_HUMAN tr|E9PQ73|E9PQ73\_HUMAN sp|Q8TDW7|FAT3\_HUMAN sp|P20742|PZP\_HUMAN sp|P19013|K2C4\_HUMAN tr|F8W0C6|F8W0C6\_HUMAN tr|F2Z2U4|F2Z2U4\_HUMAN tr|E7EM53|E7EM53\_HUMAN tr|A0A087WZ37|A0A087WZ37\_HUMAN tr|H7BXI1|H7BXI1\_HUMAN tr|H0Y465|H0Y465\_HUMAN sp|A5YKK6|CNOT1\_HUMAN Q28107 sp|Q8IVL1|NAV2\_HUMAN sp|P49815|TSC2\_HUMAN sp|P13611|CSPG2\_HUMAN sp|Q8TE73|DYH5\_HUMAN sp|Q96M86|DNHD1\_HUMAN sp|Q07001|ACHD\_HUMAN sp|P21359|NF1\_HUMAN tr|H0Y4W2|H0Y4W2\_HUMAN sp|P98160|PGBM\_HUMAN tr|A0A087WYM5|A0A087WYM5\_HUMAN sp|Q9BVV6|TALD3\_HUMAN sp|Q12852|M3K12\_HUMAN sp|Q5UIP0|RIF1\_HUMAN sp|Q9Y4A5|TRRAP\_HUMAN sp|Q9UHB7|AFF4\_HUMAN sp|Q02779|M3K10\_HUMAN tr|E7EVA0|E7EVA0\_HUMAN tr|A0A075B6F3|A0A075B6F3\_HUMAN sp|Q96JB1|DYH8\_HUMAN tr|G3V4P9|G3V4P9\_HUMAN sp|P80192|M3K9\_HUMAN tr|J3KPI6|J3KPI6\_HUMAN tr|A2A3E6|A2A3E6\_HUMAN tr|A2A3E7|A2A3E7\_HUMAN tr|A2A3D8|A2A3D8\_HUMAN tr|A0A087X250|A0A087X250\_HUMAN sp|Q96QU1|PCD15\_HUMAN sp|Q9Y485|DMXL1\_HUMAN sp|Q7Z3U7|MON2\_HUMAN sp|Q8NFC6|BD1L1\_HUMAN sp|Q9NR99|MXRA5\_HUMAN sp|P57103|NAC3\_HUMAN tr|A0A087X080|A0A087X080\_HUMAN sp|Q96PE2|ARHGH\_HUMAN tr|A0A087WZ85|A0A087WZ85\_HUMAN sp|P78527|PRKDC\_HUMAN sp|Q9NYQ6|CELR1\_HUMAN tr|F8W1U3|F8W1U3\_HUMAN sp|O43283|M3K13\_HUMAN sp|Q8NG31|CASC5\_HUMAN tr|K7EK91|K7EK91\_HUMAN sp|Q9UFH2|DYH17\_HUMAN sp|Q8IX07|FOG1\_HUMAN tr|A0A087WW06|A0A087WW06\_HUMAN sp|Q96AY4|TTC28\_HUMAN sp|Q9C0G6|DYH6\_HUMAN sp|Q15413|RYR3\_HUMAN sp|Q96Q06|PLIN4\_HUMAN sp|Q9P212|PLCE1\_HUMAN tr|E9PG32|E9PG32\_HUMAN tr|C9JFD3|C9JFD3\_HUMAN sp|Q9Y618|NCOR2\_HUMAN sp|Q14686|NCOA6\_HUMAN sp|Q9H251|CAD23\_HUMAN tr|A0A087X097|A0A087X097\_HUMAN sp|P12259|FA5\_HUMAN tr|A2A3E8|A2A3E8\_HUMAN tr|H0Y7V4|H0Y7V4\_HUMAN tr|F5H269|F5H269\_HUMAN sp|Q8IWZ3|ANKH1\_HUMAN sp|O43663|PRC1\_HUMAN sp|P13612|ITA4\_HUMAN tr|A0A087WX37|A0A087WX37\_HUMAN sp|Q86UK0|ABCAC\_HUMAN sp|P11274|BCR\_HUMAN tr|F8WBS0|F8WBS0\_HUMAN tr|B4DKT6|B4DKT6\_HUMAN tr|F8WB46|F8WB46\_HUMAN sp|Q8IZT6|ASPM\_HUMAN sp|Q5JWR5|DOP1\_HUMAN sp|Q8N2C7|UNC80\_HUMAN tr|Q5TA12|Q5TA12\_HUMAN tr|H3BMQ0|H3BMQ0\_HUMAN tr|A0A087WW79|A0A087WW79\_HUMAN tr|M0QZD8|M0QZD8\_HUMAN sp|Q63HN8|RN213\_HUMAN sp|Q99102|MUC4\_HUMAN tr|C9JE98|C9JE98\_HUMAN tr|C9J0Q5|C9J0Q5\_HUMAN tr|G3V3N7|G3V3N7\_HUMAN sp|Q709C8|VP13C\_HUMAN sp|Q9NU22|MDN1\_HUMAN tr|A0A087WXU3|A0A087WXU3\_HUMAN sp|A0FGR8|ESYT2\_HUMAN sp|P78364|PHC1\_HUMAN tr|A0A096LNH0|A0A096LNH0\_HUMAN sp|Q6V1P9|PCD23\_HUMAN tr|A0A087WZ52|A0A087WZ52\_HUMAN tr|H0Y930|H0Y930\_HUMAN sp|Q6PGP7|TTC37\_HUMAN sp|Q14766|LTBP1\_HUMAN sp|Q12816|TROP\_HUMAN sp|Q9Y520|PRC2C\_HUMAN sp|Q14315|FLNC\_HUMAN sp|Q15772|SPEG\_HUMAN sp|Q5XKE5|K2C79\_HUMAN Q5XKE5 P05787 sp|P05787|K2C8\_HUMAN sp|P35658|NU214\_HUMAN sp|Q7Z3Y9|K1C26\_HUMAN Q7Z3Y9 sp|Q9C0F0|ASXL3\_HUMAN tr|F8VUG4|F8VUG4\_HUMAN tr|F5GWT4|F5GWT4\_HUMAN tr|G3V347|G3V347\_HUMAN sp|Q9NZJ4|SACS\_HUMAN tr|F8VUG2|F8VUG2\_HUMAN sp|Q5T4S7|UBR4\_HUMAN sp|P25391|LAMA1\_HUMAN sp|O60494|CUBN\_HUMAN tr|A0A087X120|A0A087X120\_HUMAN sp|Q7Z442|PK1L2\_HUMAN sp|Q9BY42|RTF2\_HUMAN tr|A2A2L5|A2A2L5\_HUMAN sp|Q5H9F3|BCORL\_HUMAN tr|E7EPB6|E7EPB6\_HUMAN sp|P13569|CFTR\_HUMAN sp|Q8IZL2|MAML2\_HUMAN sp|P98161|PKD1\_HUMAN sp|Q9H799|CE042\_HUMAN sp|Q8NCM8|DYHC2\_HUMAN sp|Q02086|SP2\_HUMAN sp|Q9P2D7|DYH1\_HUMAN tr|H0Y390|H0Y390\_HUMAN sp|P16112|PGCA\_HUMAN tr|E7ESW5|E7ESW5\_HUMAN sp|Q8TAB3|PCD19\_HUMAN sp|Q96CN4|EVI5L\_HUMAN sp|Q9BXW4|MLP3C\_HUMAN sp|Q5T1M5|FKB15\_HUMAN tr|E7EX88|E7EX88\_HUMAN sp|Q9ULG1|INO80\_HUMAN sp|Q9NZR2|LRP1B\_HUMAN tr|A0A087WUR9|A0A087WUR9\_HUMAN sp|Q5T011|SZT2\_HUMAN tr|F5GXV7|F5GXV7\_HUMAN tr|Q5T321|Q5T321\_HUMAN sp|Q8NFP9|NBEA\_HUMAN sp|P23467|PTPRB\_HUMAN tr|A0A087WV15|A0A087WV15\_HUMAN sp|O95714|HERC2\_HUMAN tr|H0Y764|H0Y764\_HUMAN sp|Q96P20|NALP3\_HUMAN sp|O14514|BAI1\_HUMAN sp|O14497|ARI1A\_HUMAN tr|H7C1M2|H7C1M2\_HUMAN sp|P11532|DMD\_HUMAN tr|A0A075B6G3|A0A075B6G3\_HUMAN tr|E9PDN5|E9PDN5\_HUMAN sp|O43182|RHG06\_HUMAN tr|F8W9U9|F8W9U9\_HUMAN sp|Q15746|MYLK\_HUMAN tr|A0A087X0G5|A0A087X0G5\_HUMAN sp|Q99558|M3K14\_HUMAN sp|O15078|CE290\_HUMAN tr|J3KNF5|J3KNF5\_HUMAN sp|Q96L73|NSD1\_HUMAN sp|O15018|PDZD2\_HUMAN sp|A0JNW5|UH1BL\_HUMAN sp|O43298|ZBT43\_HUMAN P05784 sp|Q09666|AHNK\_HUMAN sp|Q9Y2F5|ICE1\_HUMAN sp|Q99996|AKAP9\_HUMAN sp|P01266|THYG\_HUMAN sp|Q8NDA2|HMCN2\_HUMAN sp|O14686|KMT2D\_HUMAN tr|F8W9J4|F8W9J4\_HUMAN sp|Q03001|DYST\_HUMAN sp|Q6ZRS2|SRCAP\_HUMAN sp|Q96S53|TESK2\_HUMAN Q2M2I5 sp|Q2M2I5|K1C24\_HUMAN sp|Q5SZK8|FREM2\_HUMAN sp|Q13029|PRDM2\_HUMAN tr|C9J4F3|C9J4F3\_HUMAN tr|A9Z1W1|A9Z1W1\_HUMAN tr|A0A087X1T6|A0A087X1T6\_HUMAN tr|A0A087WUA8|A0A087WUA8\_HUMAN tr|A0A087WV90|A0A087WV90\_HUMAN tr|A0A087WTU7|A0A087WTU7\_HUMAN tr|M0R1J8|M0R1J8\_HUMAN tr|H7C459|H7C459\_HUMAN sp|Q15154|PCM1\_HUMAN tr|E7ETA6|E7ETA6\_HUMAN tr|E7EPN9|E7EPN9\_HUMAN tr|H3BLS7|H3BLS7\_HUMAN sp|Q9Y4G6|TLN2\_HUMAN tr|M0R0A7|M0R0A7\_HUMAN sp|P00451|FA8\_HUMAN tr|E7EPZ9|E7EPZ9\_HUMAN tr|A0A087X1T7|A0A087X1T7\_HUMAN tr|E7ENV9|E7ENV9\_HUMAN tr|H0YM81|H0YM81\_HUMAN sp|P46821|MAP1B\_HUMAN sp|Q9Y2J0|RP3A\_HUMAN sp|Q6ZR08|DYH12\_HUMAN sp|Q16825|PTN21\_HUMAN sp|Q9Y6D5|BIG2\_HUMAN sp|Q9NYC9|DYH9\_HUMAN sp|Q13233|M3K1\_HUMAN sp|Q2M2H8|MGAL\_HUMAN sp|O60244|MED14\_HUMAN tr|A0A087WZP1|A0A087WZP1\_HUMAN tr|A0A087WWQ0|A0A087WWQ0\_HUMAN sp|Q16787|LAMA3\_HUMAN sp|Q9NR48|ASH1L\_HUMAN tr|E7ERL8|E7ERL8\_HUMAN sp|P15924|DESP\_HUMAN P07744 sp|Q09472|EP300\_HUMAN sp|Q13085|ACACA\_HUMAN sp|A6NDB9|PALM3\_HUMAN sp|Q01974|ROR2\_HUMAN sp|P25054|APC\_HUMAN sp|Q9H5I5|PIEZ2\_HUMAN tr|E7EVM7|E7EVM7\_HUMAN sp|P49750|YLPM1\_HUMAN tr|F5H212|F5H212\_HUMAN sp|P15822|ZEP1\_HUMAN sp|Q9NYQ8|FAT2\_HUMAN sp|Q3YBR2|TBRG1\_HUMAN sp|Q9Y3S1|WNK2\_HUMAN sp|Q08357|S20A2\_HUMAN sp|Q86VV8|RTTN\_HUMAN sp|Q2M3C7|SPKAP\_HUMAN tr|F8VP47|F8VP47\_HUMAN sp|Q14789|GOGB1\_HUMAN sp|O94915|FRYL\_HUMAN tr|A6NNN6|A6NNN6\_HUMAN sp|Q9H1A4|APC1\_HUMAN O95678 sp|Q5HY92|FIGN\_HUMAN tr|J3KQH6|J3KQH6\_HUMAN sp|Q6UB98|ANR12\_HUMAN sp|Q9ULB1|NRX1A\_HUMAN sp|O60287|NPA1P\_HUMAN sp|Q5SYE7|NHSL1\_HUMAN sp|Q14980|NUMA1\_HUMAN sp|Q68DE3|K2018\_HUMAN tr|E7ESW6|E7ESW6\_HUMAN sp|Q6ZQQ6|WDR87\_HUMAN sp|Q86Y46|K2C73\_HUMAN Q32MB2 tr|H0YI76|H0YI76\_HUMAN tr|E7EP17|E7EP17\_HUMAN sp|Q9NZM4|GSCR1\_HUMAN tr|A0A087WWH3|A0A087WWH3\_HUMAN tr|F8W6N5|F8W6N5\_HUMAN sp|Q92629|SGCD\_HUMAN sp|Q9C0D2|K1731\_HUMAN tr|D3DSV6|D3DSV6\_HUMAN sp|Q7Z5L9|I2BP2\_HUMAN sp|A6NKB5|PCX2\_HUMAN sp|O00750|P3C2B\_HUMAN tr|F5GWN5|F5GWN5\_HUMAN sp|Q8WWQ8|STAB2\_HUMAN tr|H0YIQ2|H0YIQ2\_HUMAN sp|O00763|ACACB\_HUMAN tr|A0A087WY61|A0A087WY61\_HUMAN sp|P51805|PLXA3\_HUMAN tr|G3V5X7|G3V5X7\_HUMAN sp|Q99973|TEP1\_HUMAN P19012 sp|P19012|K1C15\_HUMAN sp|Q9H195|MUC3B\_HUMAN tr|A0A087WYA1|A0A087WYA1\_HUMAN sp|Q8IYD8|FANCM\_HUMAN sp|Q7Z407|CSMD3\_HUMAN sp|Q02952|AKA12\_HUMAN sp|P46100|ATRX\_HUMAN sp|Q9H0K1|SIK2\_HUMAN sp|Q12802|AKP13\_HUMAN P08730-1 sp|Q9Y6Q9|NCOA3\_HUMAN tr|J3KQ66|J3KQ66\_HUMAN sp|P78509|RELN\_HUMAN tr|A0A087X127|A0A087X127\_HUMAN sp|Q8NF64|ZMIZ2\_HUMAN tr|E9PPJ1|E9PPJ1\_HUMAN sp|O75362|ZN217\_HUMAN sp|Q15569|TESK1\_HUMAN tr|F8VZV1|F8VZV1\_HUMAN sp|Q7Z4S6|KI21A\_HUMAN sp|O95678|K2C75\_HUMAN sp|Q9Y3R5|DOP2\_HUMAN sp|Q6WKZ4|RFIP1\_HUMAN tr|A0A075B6Q3|A0A075B6Q3\_HUMAN sp|Q14CM0|FRPD4\_HUMAN sp|Q6WRI0|IGS10\_HUMAN sp|A4UGR9|XIRP2\_HUMAN sp|Q14669|TRIPC\_HUMAN sp|Q6W4X9|MUC6\_HUMAN sp|Q8IYW2|CFA46\_HUMAN sp|P42345|MTOR\_HUMAN sp|Q7Z6B0|CCD91\_HUMAN tr|F5GWB1|F5GWB1\_HUMAN sp|Q9Y4H2|IRS2\_HUMAN sp|Q8WYB5|KAT6B\_HUMAN sp|Q5TCQ9|MAGI3\_HUMAN sp|Q5TH69|BIG3\_HUMAN sp|A2RRP1|NBAS\_HUMAN tr|A0A087WX80|A0A087WX80\_HUMAN tr|E9PDP5|E9PDP5\_HUMAN sp|P51587|BRCA2\_HUMAN sp|P42858|HD\_HUMAN tr|H0Y7T5|H0Y7T5\_HUMAN tr|F8W9F9|F8W9F9\_HUMAN tr|A0A096LP30|A0A096LP30\_HUMAN sp|Q0VDD8|DYH14\_HUMAN tr|F5H1U9|F5H1U9\_HUMAN sp|O75970|MPDZ\_HUMAN sp|Q9ULL0|K1210\_HUMAN sp|O43157|PLXB1\_HUMAN sp|P13646|K1C13\_HUMAN tr|J3QT34|J3QT34\_HUMAN sp|O15021|MAST4\_HUMAN sp|Q02817|MUC2\_HUMAN sp|Q05707|COEA1\_HUMAN sp|Q9HCD6|TANC2\_HUMAN sp|Q86XE3|MICU3\_HUMAN tr|F1T0J2|F1T0J2\_HUMAN sp|Q9P2D1|CHD7\_HUMAN tr|A0A087WZW3|A0A087WZW3\_HUMAN tr|A0A087WZN9|A0A087WZN9\_HUMAN tr|A2A3E3|A2A3E3\_HUMAN tr|A0A087WTR6|A0A087WTR6\_HUMAN sp|Q92621|NU205\_HUMAN sp|Q58EX7|PKHG4\_HUMAN sp|O94827|PKHG5\_HUMAN tr|E9PDG8|E9PDG8\_HUMAN tr|E9PEI6|E9PEI6\_HUMAN sp|Q96QU8|XPO6\_HUMAN tr|J3KPQ4|J3KPQ4\_HUMAN tr|R4GN15|R4GN15\_HUMAN sp|Q9BRR9|RHG09\_HUMAN tr|F8VU56|F8VU56\_HUMAN sp|P19338|NUCL\_HUMAN tr|A0A087WYF1|A0A087WYF1\_HUMAN sp|P24043|LAMA2\_HUMAN sp|Q4AC94|C2CD3\_HUMAN sp|Q8NCX0|CC150\_HUMAN sp|Q9H898|ZMAT4\_HUMAN tr|E5RIF5|E5RIF5\_HUMAN sp|Q6ZVL6|K154L\_HUMAN sp|P14625|ENPL\_HUMAN sp|Q96NT5|PCFT\_HUMAN sp|Q86YV5|SG223\_HUMAN sp|A6H8Y1|BDP1\_HUMAN sp|Q9P219|DAPLE\_HUMAN tr|E9PDC2|E9PDC2\_HUMAN sp|Q8WXX0|DYH7\_HUMAN tr|I3L2J0|I3L2J0\_HUMAN tr|Q5T3Q7|Q5T3Q7\_HUMAN sp|Q9H583|HEAT1\_HUMAN tr|H7C4Y7|H7C4Y7\_HUMAN sp|Q9HCJ0|TNR6C\_HUMAN sp|Q9UNY4|TTF2\_HUMAN sp|Q92608|DOCK2\_HUMAN sp|Q12756|KIF1A\_HUMAN sp|Q8IZC6|CORA1\_HUMAN sp|Q9UPU5|UBP24\_HUMAN sp|Q9UIF8|BAZ2B\_HUMAN sp|Q9NR97|TLR8\_HUMAN sp|Q9P267|MBD5\_HUMAN tr|E9PHH0|E9PHH0\_HUMAN sp|Q13635|PTC1\_HUMAN tr|G5E9S8|G5E9S8\_HUMAN tr|G3V3H3|G3V3H3\_HUMAN tr|E7EVH7|E7EVH7\_HUMAN tr|G3V2E7|G3V2E7\_HUMAN sp|Q07866|KLC1\_HUMAN tr|F8W6L3|F8W6L3\_HUMAN tr|G3V5R9|G3V5R9\_HUMAN tr|F8VSD5|F8VSD5\_HUMAN sp|Q8TD26|CHD6\_HUMAN sp|P10071|GLI3\_HUMAN tr|B7Z6D5|B7Z6D5\_HUMAN sp|Q96GQ7|DDX27\_HUMAN tr|E7EMG0|E7EMG0\_HUMAN sp|Q8IVL0|NAV3\_HUMAN tr|H0Y786|H0Y786\_HUMAN tr|D6RIA3|D6RIA3\_HUMAN sp|O75578|ITA10\_HUMAN sp|Q5VT06|CE350\_HUMAN sp|Q9UKN7|MYO15\_HUMAN P19001 sp|O94854|K0754\_HUMAN tr|E9PHV5|E9PHV5\_HUMAN sp|P49792|RBP2\_HUMAN tr|E9PJL5|E9PJL5\_HUMAN sp|P12270|TPR\_HUMAN tr|H0YG16|H0YG16\_HUMAN sp|Q96JG9|ZN469\_HUMAN sp|Q96N67|DOCK7\_HUMAN sp|Q8NEY1|NAV1\_HUMAN sp|O95425|SVIL\_HUMAN sp|O60281|ZN292\_HUMAN tr|J3KNV1|J3KNV1\_HUMAN tr|E7EWQ5|E7EWQ5\_HUMAN sp|Q12923|PTN13\_HUMAN tr|F5H3Z8|F5H3Z8\_HUMAN tr|F5H4N8|F5H4N8\_HUMAN tr|F5GXK6|F5GXK6\_HUMAN tr|F8W1B3|F8W1B3\_HUMAN sp|Q8WUD6|CHPT1\_HUMAN tr|B1AQK6|B1AQK6\_HUMAN tr|B1AQK7|B1AQK7\_HUMAN sp|P24821|TENA\_HUMAN sp|P28290|SSFA2\_HUMAN tr|A0A087WZU1|A0A087WZU1\_HUMAN sp|Q5JSZ5|PRC2B\_HUMAN tr|F8WB18|F8WB18\_HUMAN sp|Q9HCE0|EPG5\_HUMAN sp|Q9UKA4|AKA11\_HUMAN sp|Q6N022|TEN4\_HUMAN sp|O60641|AP180\_HUMAN sp|Q9UGU0|TCF20\_HUMAN sp|Q5VWN6|F208B\_HUMAN sp|P98164|LRP2\_HUMAN tr|H0YMN5|H0YMN5\_HUMAN tr|A0A075B6Z2|A0A075B6Z2\_HUMAN sp|Q14694|UBP10\_HUMAN sp|Q9C0G0|ZN407\_HUMAN Q9D312 tr|X6RKW4|X6RKW4\_HUMAN tr|E7ET55|E7ET55\_HUMAN sp|Q14246|EMR1\_HUMAN sp|Q9UMD9|COHA1\_HUMAN sp|Q9UKJ3|GPTC8\_HUMAN tr|H3BS19|H3BS19\_HUMAN sp|Q8WUY3|PRUN2\_HUMAN tr|A0A088AWP5|A0A088AWP5\_HUMAN tr|Q96GW1|Q96GW1\_HUMAN tr|C9JQE8|C9JQE8\_HUMAN sp|Q5VV67|PPRC1\_HUMAN sp|Q9HD20|AT131\_HUMAN tr|K7EPJ7|K7EPJ7\_HUMAN tr|J3QRF7|J3QRF7\_HUMAN tr|A0A087WYR8|A0A087WYR8\_HUMAN tr|J3KNL6|J3KNL6\_HUMAN sp|Q5D862|FILA2\_HUMAN Q5D862 sp|Q8NFD5|ARI1B\_HUMAN tr|G3V106|G3V106\_HUMAN sp|Q9ULM3|YETS2\_HUMAN tr|H7C4B2|H7C4B2\_HUMAN tr|D3YTG3|D3YTG3\_HUMAN tr|E9PDR3|E9PDR3\_HUMAN tr|A0A087WV66|A0A087WV66\_HUMAN sp|P46013|KI67\_HUMAN tr|H0Y897|H0Y897\_HUMAN sp|Q8IZQ1|WDFY3\_HUMAN sp|Q96HP0|DOCK6\_HUMAN sp|Q00975|CAC1B\_HUMAN tr|F6RH32|F6RH32\_HUMAN sp|Q53TS8|AL2SA\_HUMAN sp|Q8IYW4|ENTD1\_HUMAN sp|Q92616|GCN1L\_HUMAN tr|H0YHT2|H0YHT2\_HUMAN sp|Q96RL7|VP13A\_HUMAN sp|Q13023|AKAP6\_HUMAN tr|H0YJT3|H0YJT3\_HUMAN sp|Q9BXT5|TEX15\_HUMAN sp|Q6P2E9|EDC4\_HUMAN sp|Q99715|COCA1\_HUMAN tr|D6RGG3|D6RGG3\_HUMAN tr|F5GYC7|F5GYC7\_HUMAN tr|A0A087X2H1|A0A087X2H1\_HUMAN sp|Q9ULT8|HECD1\_HUMAN sp|O75165|DJC13\_HUMAN ENSEMBL:ENSP00000377550 tr|K7ERE3|K7ERE3\_HUMAN sp|Q5T5P2|SKT\_HUMAN tr|H0YJU9|H0YJU9\_HUMAN tr|H0YJL0|H0YJL0\_HUMAN tr|F8VNQ3|F8VNQ3\_HUMAN tr|E7EWC2|E7EWC2\_HUMAN tr|F5H7S7|F5H7S7\_HUMAN sp|Q13576|IQGA2\_HUMAN tr|H0YCZ2|H0YCZ2\_HUMAN sp|Q96RT7|GCP6\_HUMAN sp|O15027|SC16A\_HUMAN sp|A6NMS7|L37A1\_HUMAN tr|A8MUI5|A8MUI5\_HUMAN tr|A0A087X0B9|A0A087X0B9\_HUMAN sp|Q9UMZ3|PTPRQ\_HUMAN sp|Q14532|K1H2\_HUMAN sp|Q5VVJ2|MYSM1\_HUMAN tr|H0YMT1|H0YMT1\_HUMAN tr|E9PHW9|E9PHW9\_HUMAN tr|D6RHE1|D6RHE1\_HUMAN tr|F5H7B7|F5H7B7\_HUMAN sp|Q07954|LRP1\_HUMAN sp|Q504Y0|S39AC\_HUMAN sp|Q9P273|TEN3\_HUMAN P08727 tr|C9JM50|C9JM50\_HUMAN sp|P08727|K1C19\_HUMAN tr|K7EKN5|K7EKN5\_HUMAN sp|Q99698|LYST\_HUMAN sp|Q5H8A4|PIGG\_HUMAN tr|E9PEB9|E9PEB9\_HUMAN tr|F6QMI7|F6QMI7\_HUMAN sp|Q13332|PTPRS\_HUMAN tr|G3V2U4|G3V2U4\_HUMAN sp|Q9P2D8|UNC79\_HUMAN sp|P43146|DCC\_HUMAN sp|Q96JE9|MAP6\_HUMAN sp|Q5TC82|RC3H1\_HUMAN tr|J3QS93|J3QS93\_HUMAN sp|P52948|NUP98\_HUMAN tr|E7ERU0|E7ERU0\_HUMAN tr|E9PHM6|E9PHM6\_HUMAN sp|Q5VT52|RPRD2\_HUMAN tr|E7EW49|E7EW49\_HUMAN tr|E7ERI8|E7ERI8\_HUMAN tr|B2RTR1|B2RTR1\_HUMAN tr|A0A087X0K4|A0A087X0K4\_HUMAN sp|Q7Z408|CSMD2\_HUMAN sp|Q70E73|RAPH1\_HUMAN tr|C9K0J5|C9K0J5\_HUMAN tr|E9PC84|E9PC84\_HUMAN sp|Q15911|ZFHX3\_HUMAN sp|Q7Z3F1|GP155\_HUMAN tr|A0A087WXK4|A0A087WXK4\_HUMAN tr|A0A087WWE2|A0A087WWE2\_HUMAN sp|Q5T0W9|FA83B\_HUMAN ENSEMBL:ENSBTAP00000024146 sp|Q9HCM1|K1551\_HUMAN tr|A0A087WWP7|A0A087WWP7\_HUMAN tr|A0A087X1P0|A0A087X1P0\_HUMAN sp|P23470|PTPRG\_HUMAN Q14532 tr|E7EPI0|E7EPI0\_HUMAN sp|O95803|NDST3\_HUMAN tr|E9PQ59|E9PQ59\_HUMAN sp|P35670|ATP7B\_HUMAN sp|Q02224|CENPE\_HUMAN sp|Q96CT2|KLH29\_HUMAN sp|Q8TD84|DSCL1\_HUMAN sp|P58107|EPIPL\_HUMAN tr|H3BRD5|H3BRD5\_HUMAN sp|B2RTY4|MYO9A\_HUMAN tr|F8W026|F8W026\_HUMAN sp|P05997|CO5A2\_HUMAN tr|E9PMV1|E9PMV1\_HUMAN tr|F8W130|F8W130\_HUMAN sp|Q12815|TROAP\_HUMAN tr|H0YIN9|H0YIN9\_HUMAN sp|Q9UMF0|ICAM5\_HUMAN sp|Q9P2D0|IBTK\_HUMAN tr|E9PDR5|E9PDR5\_HUMAN sp|O75153|CLU\_HUMAN tr|Q6ZR19|Q6ZR19\_HUMAN tr|K7EPJ9|K7EPJ9\_HUMAN sp|Q5T1H1|EYS\_HUMAN sp|P10586|PTPRF\_HUMAN sp|P11137|MTAP2\_HUMAN tr|H0Y564|H0Y564\_HUMAN sp|Q8TDJ6|DMXL2\_HUMAN sp|P24928|RPB1\_HUMAN tr|E7ESM9|E7ESM9\_HUMAN sp|Q9Y6Y1|CMTA1\_HUMAN tr|F8W8Y7|F8W8Y7\_HUMAN sp|P28749|RBL1\_HUMAN tr|H0UI80|H0UI80\_HUMAN sp|Q8IXH7|NELFD\_HUMAN tr|X6RLT1|X6RLT1\_HUMAN sp|Q9H6A9|PCX3\_HUMAN tr|A0A087X0E3|A0A087X0E3\_HUMAN tr|A0A096LNH6|A0A096LNH6\_HUMAN sp|Q14185|DOCK1\_HUMAN tr|F5H748|F5H748\_HUMAN sp|Q99466|NOTC4\_HUMAN tr|E7EWM3|E7EWM3\_HUMAN sp|Q8IUS5|EPHX4\_HUMAN sp|Q15714|T22D1\_HUMAN tr|E9PGC8|E9PGC8\_HUMAN sp|P31639|SC5A2\_HUMAN sp|Q8IY33|MILK2\_HUMAN sp|Q9UM73|ALK\_HUMAN sp|O95613|PCNT\_HUMAN tr|H0Y7R3|H0Y7R3\_HUMAN sp|Q9Y283|INVS\_HUMAN sp|Q15652|JHD2C\_HUMAN sp|Q9NT68|TEN2\_HUMAN tr|A0A075B749|A0A075B749\_HUMAN tr|A0A087WX83|A0A087WX83\_HUMAN tr|E7EQM8|E7EQM8\_HUMAN tr|X6R8W7|X6R8W7\_HUMAN tr|R4GNH2|R4GNH2\_HUMAN sp|Q8IWN7|RP1L1\_HUMAN tr|A6NKC6|A6NKC6\_HUMAN sp|P59046|NAL12\_HUMAN sp|Q16594|TAF9\_HUMAN sp|P23471|PTPRZ\_HUMAN sp|A1L390|PKHG3\_HUMAN sp|Q96JK9|MAML3\_HUMAN Q6IFX2 tr|C9JYY6|C9JYY6\_HUMAN sp|Q02388|CO7A1\_HUMAN sp|Q9UHV7|MED13\_HUMAN sp|Q96Q89|KI20B\_HUMAN sp|Q68CQ1|MROH7\_HUMAN REFSEQ:XP\_986630 sp|Q9NS40|KCNH7\_HUMAN tr|A0A087WZQ1|A0A087WZQ1\_HUMAN Q01546 sp|Q5QGS0|K2022\_HUMAN sp|O43149|ZZEF1\_HUMAN tr|A0A087WVU5|A0A087WVU5\_HUMAN sp|O00512|BCL9\_HUMAN sp|P23468|PTPRD\_HUMAN sp|Q9HAZ2|PRD16\_HUMAN sp|A6NHR9|SMHD1\_HUMAN tr|H7BY37|H7BY37\_HUMAN sp|P63128|POK9\_HUMAN tr|J3QT83|J3QT83\_HUMAN tr|H0YMW2|H0YMW2\_HUMAN sp|Q8IWB9|TEX2\_HUMAN tr|A0A087WVA8|A0A087WVA8\_HUMAN sp|O75167|PHAR2\_HUMAN sp|Q2KHR2|RFX7\_HUMAN sp|Q96JQ2|CLMN\_HUMAN tr|E7EUL7|E7EUL7\_HUMAN sp|Q9ULE0|WWC3\_HUMAN sp|O43379|WDR62\_HUMAN sp|P46939|UTRO\_HUMAN sp|Q8IYS4|CP071\_HUMAN sp|Q9Y4D7|PLXD1\_HUMAN tr|A6NMQ1|A6NMQ1\_HUMAN sp|P09884|DPOLA\_HUMAN sp|Q6ZN30|BNC2\_HUMAN sp|Q4LDE5|SVEP1\_HUMAN tr|A0A075B6H2|A0A075B6H2\_HUMAN tr|E7EQL8|E7EQL8\_HUMAN sp|Q8IX01|SUGP2\_HUMAN tr|M0R2Z9|M0R2Z9\_HUMAN sp|P46379|BAG6\_HUMAN tr|F8W8V9|F8W8V9\_HUMAN tr|R4GMW8|R4GMW8\_HUMAN tr|H0YJ59|H0YJ59\_HUMAN sp|Q7Z3B4|NUP54\_HUMAN tr|A0A087X0P0|A0A087X0P0\_HUMAN tr|H7BYZ1|H7BYZ1\_HUMAN tr|F5H2D1|F5H2D1\_HUMAN tr|A2A3F7|A2A3F7\_HUMAN sp|O00327|BMAL1\_HUMAN tr|F5GYM6|F5GYM6\_HUMAN sp|Q9NY84|VNN3\_HUMAN tr|J3KNF3|J3KNF3\_HUMAN sp|O43151|TET3\_HUMAN sp|Q8IX03|KIBRA\_HUMAN sp|Q9Y2K3|MYH15\_HUMAN sp|Q8WZ74|CTTB2\_HUMAN sp|Q562E7|WDR81\_HUMAN sp|O60229|KALRN\_HUMAN tr|J3QSW6|J3QSW6\_HUMAN tr|H7BXZ5|H7BXZ5\_HUMAN tr|K7ESE1|K7ESE1\_HUMAN tr|G3V1B5|G3V1B5\_HUMAN sp|Q8N9S9|SNX31\_HUMAN H-INV:HIT000016045 tr|F8VP67|F8VP67\_HUMAN sp|Q96GZ6|S41A3\_HUMAN sp|Q9P0K8|FOXJ2\_HUMAN sp|Q9HCF6|TRPM3\_HUMAN tr|H3BLZ3|H3BLZ3\_HUMAN tr|J3KSB5|J3KSB5\_HUMAN sp|Q9H568|ACTL8\_HUMAN sp|Q6IE36|OVOS2\_HUMAN tr|A0A087WXL3|A0A087WXL3\_HUMAN sp|Q9Y4C0|NRX3A\_HUMAN tr|H0Y507|H0Y507\_HUMAN sp|Q5TCZ1|SPD2A\_HUMAN tr|H0Y9I8|H0Y9I8\_HUMAN sp|Q96F81|DISP1\_HUMAN sp|Q86UY5|FA83A\_HUMAN tr|G3V2W5|G3V2W5\_HUMAN sp|Q96RN1|S26A8\_HUMAN sp|P01023|A2MG\_HUMAN sp|Q8TE60|ATS18\_HUMAN tr|B8A4K4|B8A4K4\_HUMAN sp|Q01118|SCN7A\_HUMAN sp|O95999|BCL10\_HUMAN tr|A0A087WUV6|A0A087WUV6\_HUMAN sp|Q9ULI3|HEG1\_HUMAN tr|I3L2B0|I3L2B0\_HUMAN tr|J3KR49|J3KR49\_HUMAN tr|E3W994|E3W994\_HUMAN sp|O75122|CLAP2\_HUMAN tr|A0A087WYX8|A0A087WYX8\_HUMAN sp|Q9C0A1|ZFHX2\_HUMAN sp|Q93074|MED12\_HUMAN sp|P0DJD1|RGPD2\_HUMAN tr|F8VYC4|F8VYC4\_HUMAN sp|P0DJD0|RGPD1\_HUMAN sp|Q6UVM3|KCNT2\_HUMAN sp|Q7Z5Y7|KCD20\_HUMAN tr|H0YJS3|H0YJS3\_HUMAN tr|A0A087WVF8|A0A087WVF8\_HUMAN tr|A0A096LNL9|A0A096LNL9\_HUMAN tr|J3KTL8|J3KTL8\_HUMAN tr|E7ERW7|E7ERW7\_HUMAN sp|P53804|TTC3\_HUMAN sp|Q9UD71|PPR1B\_HUMAN tr|J3KSJ8|J3KSJ8\_HUMAN sp|Q5VZL5|ZMYM4\_HUMAN sp|O95229|ZWINT\_HUMAN sp|Q86YA3|ZGRF1\_HUMAN sp|A6NM11|L37A2\_HUMAN sp|Q6KC79|NIPBL\_HUMAN sp|Q96PY5|FMNL2\_HUMAN sp|P00492|HPRT\_HUMAN sp|P52747|ZN143\_HUMAN sp|Q92823|NRCAM\_HUMAN tr|B1APH0|B1APH0\_HUMAN sp|Q6X4U4|SOSD1\_HUMAN tr|F8VU51|F8VU51\_HUMAN sp|Q14677|EPN4\_HUMAN sp|Q14525|KT33B\_HUMAN Q14525 tr|A0A087X2I6|A0A087X2I6\_HUMAN tr|G8JL96|G8JL96\_HUMAN tr|H7BYX7|H7BYX7\_HUMAN sp|Q3L8U1|CHD9\_HUMAN tr|J3KPC5|J3KPC5\_HUMAN sp|P47736|RPGP1\_HUMAN tr|F2Z357|F2Z357\_HUMAN sp|Q96JM2|ZN462\_HUMAN sp|Q9H3P2|NELFA\_HUMAN sp|Q9H792|PEAK1\_HUMAN sp|Q9C091|GRB1L\_HUMAN sp|Q96C45|ULK4\_HUMAN sp|Q14974|IMB1\_HUMAN sp|Q4L235|ACSF4\_HUMAN tr|R4GNB1|R4GNB1\_HUMAN sp|Q96BY7|ATG2B\_HUMAN sp|Q5JV73|FRPD3\_HUMAN tr|F5H7V9|F5H7V9\_HUMAN sp|Q13423|NNTM\_HUMAN tr|E9PCX7|E9PCX7\_HUMAN tr|A6NIW2|A6NIW2\_HUMAN sp|Q5JSL3|DOC11\_HUMAN sp|Q12873|CHD3\_HUMAN tr|C9J7T7|C9J7T7\_HUMAN sp|O75179|ANR17\_HUMAN tr|H0YM23|H0YM23\_HUMAN tr|R4GMS7|R4GMS7\_HUMAN tr|K7ESB7|K7ESB7\_HUMAN tr|H0YN07|H0YN07\_HUMAN sp|O75116|ROCK2\_HUMAN sp|O95665|NTR2\_HUMAN sp|Q8IWV7|UBR1\_HUMAN sp|A8MZ97|CB074\_HUMAN sp|P03891|NU2M\_HUMAN sp|Q86YW9|MD12L\_HUMAN tr|D6RDW0|D6RDW0\_HUMAN tr|D6RFY3|D6RFY3\_HUMAN sp|Q92750|TAF4B\_HUMAN tr|J3KTH2|J3KTH2\_HUMAN tr|H7BYP1|H7BYP1\_HUMAN tr|A2A3F3|A2A3F3\_HUMAN tr|E9PBI7|E9PBI7\_HUMAN tr|G5E9G1|G5E9G1\_HUMAN tr|A2A3F4|A2A3F4\_HUMAN sp|O60721|NCKX1\_HUMAN tr|F5H483|F5H483\_HUMAN tr|F5H127|F5H127\_HUMAN sp|Q9Y5G7|PCDG6\_HUMAN sp|Q9NPG3|UBN1\_HUMAN sp|Q8N2Y8|RUSC2\_HUMAN sp|Q86UU0|BCL9L\_HUMAN tr|A0A087WZX0|A0A087WZX0\_HUMAN sp|P39059|COFA1\_HUMAN tr|A0A087X0K0|A0A087X0K0\_HUMAN sp|Q9UQC9|CLCA2\_HUMAN sp|Q96PX9|PKH4B\_HUMAN sp|Q9HD67|MYO10\_HUMAN sp|P48634|PRC2A\_HUMAN sp|Q70CQ2|UBP34\_HUMAN tr|H0YA13|H0YA13\_HUMAN tr|E7EN86|E7EN86\_HUMAN sp|Q6T4R5|NHS\_HUMAN tr|A0A087WU78|A0A087WU78\_HUMAN tr|A0A087WWC4|A0A087WWC4\_HUMAN tr|G3V470|G3V470\_HUMAN sp|P27708|PYR1\_HUMAN sp|P49454|CENPF\_HUMAN tr|F8VPD4|F8VPD4\_HUMAN sp|Q5VU43|MYOME\_HUMAN tr|F5GX72|F5GX72\_HUMAN tr|H7BYE6|H7BYE6\_HUMAN sp|Q14118|DAG1\_HUMAN tr|H0Y8U6|H0Y8U6\_HUMAN sp|Q12830|BPTF\_HUMAN sp|Q9H7U1|CCSE2\_HUMAN sp|O75962|TRIO\_HUMAN sp|O60346|PHLP1\_HUMAN sp|Q6ZT12|UBR3\_HUMAN sp|Q9UIG0|BAZ1B\_HUMAN tr|E7EP60|E7EP60\_HUMAN tr|A0A087X2B3|A0A087X2B3\_HUMAN sp|P55107|BMP3B\_HUMAN tr|K7EIG1|K7EIG1\_HUMAN sp|Q6ZS81|WDFY4\_HUMAN sp|Q9H422|HIPK3\_HUMAN tr|A0A087WZP7|A0A087WZP7\_HUMAN sp|Q86WG5|MTMRD\_HUMAN sp|Q8NB14|UBP38\_HUMAN sp|Q9UMZ2|SYNRG\_HUMAN sp|Q14008|CKAP5\_HUMAN sp|Q5T1R4|ZEP3\_HUMAN sp|Q96KW2|P12L2\_HUMAN tr|A0A087WYX9|A0A087WYX9\_HUMAN tr|H0YN99|H0YN99\_HUMAN sp|Q9NZM3|ITSN2\_HUMAN tr|A0A087WVF7|A0A087WVF7\_HUMAN tr|H0Y325|H0Y325\_HUMAN sp|Q6ZU35|K1211\_HUMAN tr|F5H1N7|F5H1N7\_HUMAN tr|A0A087WU64|A0A087WU64\_HUMAN tr|H9KV90|H9KV90\_HUMAN sp|Q9Y566|SHAN1\_HUMAN tr|A0A087X1U6|A0A087X1U6\_HUMAN sp|Q6ZQN7|SO4C1\_HUMAN sp|Q9UPY3|DICER\_HUMAN sp|O75051|PLXA2\_HUMAN sp|Q7Z5H3|RHG22\_HUMAN sp|Q495X7|TRI60\_HUMAN sp|Q9BYE9|CDHR2\_HUMAN sp|Q8TD57|DYH3\_HUMAN tr|H0Y7L2|H0Y7L2\_HUMAN sp|Q9Y6X0|SETBP\_HUMAN tr|H7BZH9|H7BZH9\_HUMAN sp|P40145|ADCY8\_HUMAN sp|Q14966|ZN638\_HUMAN tr|H0YIM6|H0YIM6\_HUMAN sp|Q5VYM1|CI131\_HUMAN sp|Q13315|ATM\_HUMAN sp|Q9H7D0|DOCK5\_HUMAN sp|P21580|TNAP3\_HUMAN Q15323 sp|Q15323|K1H1\_HUMAN Q9UE12 tr|F8W8P2|F8W8P2\_HUMAN sp|Q96QE3|ATAD5\_HUMAN tr|D3TTY5|D3TTY5\_HUMAN tr|A0A087WU80|A0A087WU80\_HUMAN tr|H0YLN8|H0YLN8\_HUMAN sp|Q96QT4|TRPM7\_HUMAN sp|Q7Z589|EMSY\_HUMAN sp|A1L4H1|SRCRL\_HUMAN sp|Q15596|NCOA2\_HUMAN sp|Q96NA8|TSNA1\_HUMAN sp|P51826|AFF3\_HUMAN sp|O75417|DPOLQ\_HUMAN sp|Q13535|ATR\_HUMAN sp|Q8N3D4|EH1L1\_HUMAN tr|B7Z574|B7Z574\_HUMAN tr|E7EVY3|E7EVY3\_HUMAN sp|P20810|ICAL\_HUMAN sp|Q8WY21|SORC1\_HUMAN tr|D6RA32|D6RA32\_HUMAN sp|Q96KQ4|ASPP1\_HUMAN sp|Q01970|PLCB3\_HUMAN sp|P63135|POK7\_HUMAN sp|Q9Y261|FOXA2\_HUMAN sp|Q7Z333|SETX\_HUMAN tr|F8VZY9|F8VZY9\_HUMAN sp|P05783|K1C18\_HUMAN sp|Q7RTP6|MICA3\_HUMAN sp|Q494X3|ZN404\_HUMAN tr|A0A087WSV7|A0A087WSV7\_HUMAN sp|Q9NYY3|PLK2\_HUMAN sp|Q9Y2H9|MAST1\_HUMAN sp|O75030|MITF\_HUMAN tr|H7BXX0|H7BXX0\_HUMAN tr|F8W703|F8W703\_HUMAN tr|A0A087WWA3|A0A087WWA3\_HUMAN tr|B1PS43|B1PS43\_HUMAN sp|P49327|FAS\_HUMAN sp|Q9GZP0|PDGFD\_HUMAN tr|H7C1I9|H7C1I9\_HUMAN tr|E5RG83|E5RG83\_HUMAN tr|A0AVG3|A0AVG3\_HUMAN sp|Q9HCK4|ROBO2\_HUMAN sp|Q96BY6|DOC10\_HUMAN sp|Q68CP9|ARID2\_HUMAN sp|Q8TF46|DI3L1\_HUMAN sp|Q9UPS6|SET1B\_HUMAN sp|O95490|LPHN2\_HUMAN tr|E9PCH5|E9PCH5\_HUMAN sp|O15230|LAMA5\_HUMAN sp|O60673|DPOLZ\_HUMAN sp|Q9NZU0|FLRT3\_HUMAN tr|B7ZAV2|B7ZAV2\_HUMAN sp|Q8TBR5|CSAS1\_HUMAN sp|Q86UW6|N4BP2\_HUMAN sp|P08047|SP1\_HUMAN sp|Q92820|GGH\_HUMAN tr|J3KRP9|J3KRP9\_HUMAN sp|Q9P2F8|SI1L2\_HUMAN A2AB72 tr|F8W775|F8W775\_HUMAN sp|P00519|ABL1\_HUMAN sp|O60308|CE104\_HUMAN tr|Q05GC8|Q05GC8\_HUMAN sp|Q96PN7|TREF1\_HUMAN sp|O43150|ASAP2\_HUMAN sp|Q6Q759|SPG17\_HUMAN sp|Q9BY84|DUS16\_HUMAN sp|O43166|SI1L1\_HUMAN tr|H0Y7H8|H0Y7H8\_HUMAN sp|Q9H2X6|HIPK2\_HUMAN Q9H552 sp|P16144|ITB4\_HUMAN sp|Q15648|MED1\_HUMAN tr|H0YJ14|H0YJ14\_HUMAN tr|S4R3N9|S4R3N9\_HUMAN sp|Q9Y613|FHOD1\_HUMAN tr|E7EWN3|E7EWN3\_HUMAN sp|Q9C0A6|SETD5\_HUMAN tr|H0YEQ7|H0YEQ7\_HUMAN tr|F8WE25|F8WE25\_HUMAN sp|Q86VP6|CAND1\_HUMAN tr|F6TRT2|F6TRT2\_HUMAN sp|O76041|NEBL\_HUMAN sp|Q9UK61|F208A\_HUMAN tr|F5H527|F5H527\_HUMAN tr|A6NHM7|A6NHM7\_HUMAN P04258 sp|Q9H0J4|QRIC2\_HUMAN sp|O00468|AGRIN\_HUMAN tr|B4DEX3|B4DEX3\_HUMAN tr|H7C1I7|H7C1I7\_HUMAN sp|Q9H582|ZN644\_HUMAN sp|Q4KWH8|PLCH1\_HUMAN sp|O60292|SI1L3\_HUMAN tr|H0Y5G7|H0Y5G7\_HUMAN sp|Q8IXZ3|SP8\_HUMAN tr|E9PF17|E9PF17\_HUMAN sp|Q6NUN9|ZN746\_HUMAN tr|A0A087WWM5|A0A087WWM5\_HUMAN sp|Q5TBA9|FRY\_HUMAN tr|C9JBI8|C9JBI8\_HUMAN sp|A7KAX9|RHG32\_HUMAN sp|O95180|CAC1H\_HUMAN tr|D6RIE8|D6RIE8\_HUMAN sp|Q13464|ROCK1\_HUMAN sp|P57078|RIPK4\_HUMAN sp|Q9P2D3|HTR5B\_HUMAN tr|E9PG18|E9PG18\_HUMAN sp|Q14524|SCN5A\_HUMAN tr|H9KVD2|H9KVD2\_HUMAN sp|Q96L96|ALPK3\_HUMAN tr|H3BU53|H3BU53\_HUMAN sp|Q92538|GBF1\_HUMAN sp|O43143|DHX15\_HUMAN tr|A0A087WZL3|A0A087WZL3\_HUMAN sp|P78559|MAP1A\_HUMAN sp|O94966|UBP19\_HUMAN tr|G3V1S3|G3V1S3\_HUMAN sp|Q6ZUT9|DEN5B\_HUMAN sp|Q15436|SC23A\_HUMAN sp|O76014|KRT37\_HUMAN tr|E9PMC9|E9PMC9\_HUMAN sp|Q5MNV8|FBX47\_HUMAN tr|H0Y3X6|H0Y3X6\_HUMAN tr|F8W1U0|F8W1U0\_HUMAN sp|Q15788|NCOA1\_HUMAN sp|Q5M775|CYTSB\_HUMAN sp|P13866|SC5A1\_HUMAN sp|O94916|NFAT5\_HUMAN sp|Q04721|NOTC2\_HUMAN tr|F8WC02|F8WC02\_HUMAN tr|G5E9N2|G5E9N2\_HUMAN sp|Q63HK5|TSH3\_HUMAN tr|H0Y5C0|H0Y5C0\_HUMAN tr|B1ALU3|B1ALU3\_HUMAN tr|B7ZLW7|B7ZLW7\_HUMAN sp|Q70Z35|PREX2\_HUMAN sp|Q9NPA5|ZF64A\_HUMAN sp|P14410|SUIS\_HUMAN sp|Q9ULI4|KI26A\_HUMAN tr|E9PEM5|E9PEM5\_HUMAN sp|Q8IY92|SLX4\_HUMAN tr|E9PDE4|E9PDE4\_HUMAN sp|O75643|U520\_HUMAN sp|Q92997|DVL3\_HUMAN sp|Q9P1Z9|CC180\_HUMAN sp|Q9BYP7|WNK3\_HUMAN sp|Q9UKZ4|TEN1\_HUMAN sp|Q96RG2|PASK\_HUMAN sp|Q15735|PI5PA\_HUMAN tr|B4DF95|B4DF95\_HUMAN sp|Q8IZD9|DOCK3\_HUMAN sp|P35251|RFC1\_HUMAN sp|Q8WWI1|LMO7\_HUMAN tr|E9PMT2|E9PMT2\_HUMAN tr|J3KP06|J3KP06\_HUMAN tr|F8WD26|F8WD26\_HUMAN Q1A7A4 sp|Q9NW68|BSDC1\_HUMAN sp|Q15032|R3HD1\_HUMAN tr|H0YLP6|H0YLP6\_HUMAN tr|H0YFS7|H0YFS7\_HUMAN tr|H0YLX2|H0YLX2\_HUMAN sp|Q7Z3J3|RGPD4\_HUMAN tr|R4GN35|R4GN35\_HUMAN sp|Q5VZ66|JKIP3\_HUMAN sp|P51784|UBP11\_HUMAN tr|G5E9A6|G5E9A6\_HUMAN tr|F8VSD0|F8VSD0\_HUMAN Q6IME9 sp|Q15172|2A5A\_HUMAN tr|D6REX3|D6REX3\_HUMAN tr|H7BXG7|H7BXG7\_HUMAN sp|Q7Z745|MRO2B\_HUMAN sp|P56199|ITA1\_HUMAN tr|Q5T1U7|Q5T1U7\_HUMAN sp|P35475|IDUA\_HUMAN sp|P55198|AF17\_HUMAN sp|Q53SF7|COBL1\_HUMAN tr|A0A087WW39|A0A087WW39\_HUMAN tr|F8WCU9|F8WCU9\_HUMAN tr|E9PHB6|E9PHB6\_HUMAN tr|K4DIA1|K4DIA1\_HUMAN sp|Q70EL4|UBP43\_HUMAN O76013 sp|O76013|KRT36\_HUMAN tr|H0YA77|H0YA77\_HUMAN tr|A0A087WUH9|A0A087WUH9\_HUMAN sp|Q14207|NPAT\_HUMAN sp|Q7Z2K8|GRIN1\_HUMAN sp|A6NEL2|SWAHB\_HUMAN sp|A8MQ03|CRTP1\_HUMAN sp|O94967|WDR47\_HUMAN tr|H7C2D6|H7C2D6\_HUMAN tr|Q4R9M9|Q4R9M9\_HUMAN sp|Q9P217|ZSWM5\_HUMAN sp|O60333|KIF1B\_HUMAN sp|Q9WJR5|POK19\_HUMAN sp|O75581|LRP6\_HUMAN tr|F5H7J9|F5H7J9\_HUMAN sp|Q9HCK8|CHD8\_HUMAN sp|Q5T1B0|AXDN1\_HUMAN sp|Q76I76|SSH2\_HUMAN O76015 sp|O76015|KRT38\_HUMAN tr|G8JLP4|G8JLP4\_HUMAN sp|Q9Y4F3|MARF1\_HUMAN sp|O15085|ARHGB\_HUMAN tr|H3BMM1|H3BMM1\_HUMAN sp|Q8WVM8|SCFD1\_HUMAN sp|Q5HYC2|K2026\_HUMAN sp|O60306|AQR\_HUMAN tr|F5GWJ5|F5GWJ5\_HUMAN sp|Q86TB9|PATL1\_HUMAN sp|P27816|MAP4\_HUMAN sp|Q8NGS7|O13C8\_HUMAN tr|G3V3A5|G3V3A5\_HUMAN sp|Q9Y2X0|MED16\_HUMAN sp|A6QL64|AN36A\_HUMAN tr|H0YIC5|H0YIC5\_HUMAN tr|K7EIM5|K7EIM5\_HUMAN sp|Q9BWM7|SFXN3\_HUMAN sp|Q9NZJ5|E2AK3\_HUMAN tr|J3KP92|J3KP92\_HUMAN sp|Q86Z02|HIPK1\_HUMAN sp|Q86WA9|S2611\_HUMAN sp|Q5VU65|P210L\_HUMAN sp|Q9Y2A7|NCKP1\_HUMAN tr|C9JFF0|C9JFF0\_HUMAN sp|P02751|FINC\_HUMAN sp|Q12888|TP53B\_HUMAN sp|Q8IZH2|XRN1\_HUMAN sp|O60309|L37A3\_HUMAN sp|Q5T481|RBM20\_HUMAN sp|Q9NVH2|INT7\_HUMAN sp|Q4VCS5|AMOT\_HUMAN sp|Q7RTY8|TMPS7\_HUMAN sp|Q76N89|HECW1\_HUMAN sp|Q68DK2|ZFY26\_HUMAN tr|A0A087WTW5|A0A087WTW5\_HUMAN sp|Q9UKL3|C8AP2\_HUMAN sp|Q96JQ0|PCD16\_HUMAN sp|P08648|ITA5\_HUMAN tr|D6R9C2|D6R9C2\_HUMAN tr|A0A087X0T3|A0A087X0T3\_HUMAN sp|A7E2Y1|MYH7B\_HUMAN tr|E7EVG6|E7EVG6\_HUMAN tr|H0YGW5|H0YGW5\_HUMAN sp|P63132|PO113\_HUMAN sp|Q08AD1|CAMP2\_HUMAN tr|A0A087X208|A0A087X208\_HUMAN tr|F5H365|F5H365\_HUMAN tr|X6REW1|X6REW1\_HUMAN sp|Q5VT25|MRCKA\_HUMAN tr|F5GZ52|F5GZ52\_HUMAN sp|Q12879|NMDE1\_HUMAN sp|Q53ET0|CRTC2\_HUMAN sp|P11831|SRF\_HUMAN sp|Q99666|RGPD5\_HUMAN sp|P02461|CO3A1\_HUMAN sp|Q8IYE1|CCD13\_HUMAN sp|Q14112|NID2\_HUMAN sp|Q12809|KCNH2\_HUMAN sp|Q4ZG55|GREB1\_HUMAN tr|E9PMS6|E9PMS6\_HUMAN A2A5Y0 sp|P80365|DHI2\_HUMAN sp|Q9Y4K4|M4K5\_HUMAN tr|E7EUW2|E7EUW2\_HUMAN tr|E7ETE3|E7ETE3\_HUMAN tr|A0A087X0Q6|A0A087X0Q6\_HUMAN sp|Q9BX84|TRPM6\_HUMAN tr|E9PLV1|E9PLV1\_HUMAN sp|Q9NQV6|PRD10\_HUMAN sp|Q66K89|E4F1\_HUMAN sp|P51957|NEK4\_HUMAN sp|O95866|G6B\_HUMAN tr|B7ZLJ5|B7ZLJ5\_HUMAN sp|Q6PIF6|MYO7B\_HUMAN tr|C9JH43|C9JH43\_HUMAN sp|Q9H324|ATS10\_HUMAN tr|A0A075B730|A0A075B730\_HUMAN Q1RMK2 sp|Q9UGM3|DMBT1\_HUMAN sp|Q6P1J6|PLB1\_HUMAN sp|P43652|AFAM\_HUMAN tr|H7C207|H7C207\_HUMAN tr|G3V3H7|G3V3H7\_HUMAN sp|Q3T8J9|GON4L\_HUMAN sp|Q9NSI6|BRWD1\_HUMAN tr|A0A087WYK2|A0A087WYK2\_HUMAN sp|O14513|NCKP5\_HUMAN tr|H7C4E1|H7C4E1\_HUMAN tr|B1ALU1|B1ALU1\_HUMAN tr|A0A087X077|A0A087X077\_HUMAN sp|Q14CN4|K2C72\_HUMAN Q14CN4-1 sp|Q9UMN6|KMT2B\_HUMAN sp|Q5T0Z8|CF132\_HUMAN tr|A0A075B6E9|A0A075B6E9\_HUMAN sp|Q96NW7|LRRC7\_HUMAN sp|Q9BX63|FANCJ\_HUMAN tr|E9PQA7|E9PQA7\_HUMAN sp|P31629|ZEP2\_HUMAN tr|H7BXH0|H7BXH0\_HUMAN tr|F8WF75|F8WF75\_HUMAN tr|J3KR12|J3KR12\_HUMAN sp|Q2KJY2|KI26B\_HUMAN tr|B5MEG5|B5MEG5\_HUMAN tr|E7EST9|E7EST9\_HUMAN sp|P42229|STA5A\_HUMAN tr|K7EK35|K7EK35\_HUMAN sp|P20930|FILA\_HUMAN P20930 tr|J3QSU6|J3QSU6\_HUMAN sp|Q6NSJ5|LRC8E\_HUMAN tr|H7BXU7|H7BXU7\_HUMAN tr|M0R3F6|M0R3F6\_HUMAN sp|A6NE01|F186A\_HUMAN tr|F5GYN0|F5GYN0\_HUMAN sp|P35498|SCN1A\_HUMAN sp|P42694|HELZ\_HUMAN tr|J3QS41|J3QS41\_HUMAN sp|A6NI56|CC154\_HUMAN sp|Q9P0N5|TM216\_HUMAN sp|Q92636|FAN\_HUMAN tr|D6RIV9|D6RIV9\_HUMAN sp|P54259|ATN1\_HUMAN sp|Q8IYB8|SUV3\_HUMAN tr|F5GXQ8|F5GXQ8\_HUMAN tr|E7EWD6|E7EWD6\_HUMAN sp|Q76L83|ASXL2\_HUMAN tr|F8W9S7|F8W9S7\_HUMAN sp|Q86UD7|TBC26\_HUMAN tr|Q53S48|Q53S48\_HUMAN sp|Q9BZ95|NSD3\_HUMAN sp|Q9P241|AT10D\_HUMAN sp|Q96PE1|GP124\_HUMAN tr|C9J5B3|C9J5B3\_HUMAN tr|C9JY11|C9JY11\_HUMAN tr|C9J624|C9J624\_HUMAN sp|Q7L775|EPMIP\_HUMAN sp|Q9P266|JCAD\_HUMAN sp|Q2VWP7|PRTG\_HUMAN sp|Q9UKZ1|CNO11\_HUMAN sp|Q9NV88|INT9\_HUMAN sp|Q9HCJ5|ZSWM6\_HUMAN sp|Q2NKX8|ERC6L\_HUMAN tr|B5MDQ0|B5MDQ0\_HUMAN sp|Q86T90|K1328\_HUMAN sp|Q8IUC4|RHPN2\_HUMAN sp|P13535|MYH8\_HUMAN sp|O95789|ZMYM6\_HUMAN sp|Q8IUC6|TCAM1\_HUMAN tr|A0A087WWS1|A0A087WWS1\_HUMAN sp|Q96FV9|THOC1\_HUMAN sp|P55317|FOXA1\_HUMAN sp|Q99707|METH\_HUMAN sp|Q14596|NBR1\_HUMAN sp|Q9UNN5|FAF1\_HUMAN sp|Q38SD2|LRRK1\_HUMAN sp|Q9NVE7|PANK4\_HUMAN tr|E9PHT6|E9PHT6\_HUMAN sp|P51692|STA5B\_HUMAN sp|P55318|FOXA3\_HUMAN sp|O60293|ZC3H1\_HUMAN tr|F8WEF5|F8WEF5\_HUMAN sp|Q9P1Y6|PHRF1\_HUMAN tr|E9PJ24|E9PJ24\_HUMAN sp|O75151|PHF2\_HUMAN sp|Q8NDH2|CC168\_HUMAN sp|Q53EP0|FND3B\_HUMAN sp|Q9UKX2|MYH2\_HUMAN sp|Q93008|USP9X\_HUMAN sp|Q96RN5|MED15\_HUMAN tr|H0Y9H6|H0Y9H6\_HUMAN sp|Q96SN8|CK5P2\_HUMAN sp|Q9Y5H9|PCDA2\_HUMAN tr|D6R9P4|D6R9P4\_HUMAN sp|A8TX70|CO6A5\_HUMAN tr|E9PAL5|E9PAL5\_HUMAN sp|Q12931|TRAP1\_HUMAN tr|H3BQC6|H3BQC6\_HUMAN tr|D6REE7|D6REE7\_HUMAN tr|G3V4Z9|G3V4Z9\_HUMAN sp|A4IF30|S35F4\_HUMAN tr|E9PC90|E9PC90\_HUMAN tr|F2Z341|F2Z341\_HUMAN tr|F8WDG3|F8WDG3\_HUMAN sp|P50851|LRBA\_HUMAN sp|Q9UPV0|CE164\_HUMAN sp|Q00341|VIGLN\_HUMAN sp|Q8NGA2|OR7A2\_HUMAN tr|F1T0B3|F1T0B3\_HUMAN tr|A0A087X2G1|A0A087X2G1\_HUMAN sp|Q92499|DDX1\_HUMAN tr|A0A087WU49|A0A087WU49\_HUMAN sp|Q9Y493|ZAN\_HUMAN tr|F5H0T8|F5H0T8\_HUMAN tr|E7EPJ7|E7EPJ7\_HUMAN sp|P47989|XDH\_HUMAN sp|Q8TD20|GTR12\_HUMAN tr|Q5SQL3|Q5SQL3\_HUMAN sp|Q6ZN18|AEBP2\_HUMAN sp|Q6ZW33|MICLK\_HUMAN sp|P09131|P3\_HUMAN sp|Q6YHU6|THADA\_HUMAN sp|Q01668|CAC1D\_HUMAN sp|Q9Y490|TLN1\_HUMAN tr|Q96K65|Q96K65\_HUMAN sp|Q8NF99|ZN397\_HUMAN tr|K7ERU5|K7ERU5\_HUMAN sp|Q9Y6E7|SIR4\_HUMAN tr|V9GXZ5|V9GXZ5\_HUMAN sp|Q92752|TENR\_HUMAN sp|O95255|MRP6\_HUMAN sp|Q9HAU0|PKHA5\_HUMAN sp|Q2KHR3|QSER1\_HUMAN sp|Q96AV8|E2F7\_HUMAN O76014 tr|H0Y7F0|H0Y7F0\_HUMAN sp|Q9NQC3|RTN4\_HUMAN tr|C9JJN9|C9JJN9\_HUMAN sp|Q86TB3|ALPK2\_HUMAN sp|P30622|CLIP1\_HUMAN sp|Q93100|KPBB\_HUMAN sp|Q9HA65|TBC17\_HUMAN tr|K7EPW6|K7EPW6\_HUMAN sp|P58397|ATS12\_HUMAN tr|A0A087WT50|A0A087WT50\_HUMAN tr|E9PRS0|E9PRS0\_HUMAN tr|F8VP57|F8VP57\_HUMAN tr|B7ZKW7|B7ZKW7\_HUMAN tr|B7ZKJ3|B7ZKJ3\_HUMAN sp|Q14571|ITPR2\_HUMAN sp|Q6ZS30|NBEL1\_HUMAN tr|F2Z2S2|F2Z2S2\_HUMAN tr|E5RG74|E5RG74\_HUMAN sp|O94779|CNTN5\_HUMAN sp|P10266|POK10\_HUMAN sp|P78413|IRX4\_HUMAN tr|E9PFT7|E9PFT7\_HUMAN tr|N0DVX5|N0DVX5\_HUMAN sp|Q9HCK1|ZDBF2\_HUMAN tr|H7C5J8|H7C5J8\_HUMAN sp|Q9Y238|DLEC1\_HUMAN tr|D6R939|D6R939\_HUMAN sp|Q3LXA3|DHAK\_HUMAN tr|A0A087WUL0|A0A087WUL0\_HUMAN sp|O95477|ABCA1\_HUMAN tr|B5MCN7|B5MCN7\_HUMAN tr|G3V394|G3V394\_HUMAN sp|Q5SNT2|TM201\_HUMAN sp|Q9BYW2|SETD2\_HUMAN sp|P41218|MNDA\_HUMAN sp|Q9NS91|RAD18\_HUMAN sp|Q5THK1|PR14L\_HUMAN tr|A0A087WTF3|A0A087WTF3\_HUMAN sp|Q03468|ERCC6\_HUMAN tr|B4DGL8|B4DGL8\_HUMAN sp|O75027|ABCB7\_HUMAN tr|I3L0L0|I3L0L0\_HUMAN sp|Q6IE37|OVOS1\_HUMAN sp|Q92764|KRT35\_HUMAN tr|H7C5U4|H7C5U4\_HUMAN sp|Q9Y6Y8|S23IP\_HUMAN sp|P55145|MANF\_HUMAN sp|O94885|SASH1\_HUMAN tr|H0Y5V1|H0Y5V1\_HUMAN sp|Q9Y2I1|NISCH\_HUMAN sp|P04275|VWF\_HUMAN sp|A8MVM7|YD021\_HUMAN sp|Q96RD6|PANX2\_HUMAN tr|H7C5N8|H7C5N8\_HUMAN sp|Q9Y4K1|AIM1\_HUMAN sp|Q86TY3|CN037\_HUMAN tr|E9PR05|E9PR05\_HUMAN sp|Q5MY95|ENTP8\_HUMAN tr|H0Y853|H0Y853\_HUMAN tr|H7BXF5|H7BXF5\_HUMAN sp|Q32MH5|F214A\_HUMAN sp|P30038|AL4A1\_HUMAN tr|H7C3Y7|H7C3Y7\_HUMAN tr|A0A088AWM3|A0A088AWM3\_HUMAN tr|C9J0Q2|C9J0Q2\_HUMAN sp|Q8IV33|K0825\_HUMAN sp|Q92688|AN32B\_HUMAN tr|A0A087WV69|A0A087WV69\_HUMAN sp|P56645|PER3\_HUMAN tr|A2A3E5|A2A3E5\_HUMAN tr|A2A3E4|A2A3E4\_HUMAN tr|A2A3E1|A2A3E1\_HUMAN tr|J3QRR3|J3QRR3\_HUMAN sp|Q5TZJ5|S31A1\_HUMAN sp|Q92545|TM131\_HUMAN sp|Q14191|WRN\_HUMAN tr|G5E9G6|G5E9G6\_HUMAN sp|Q7L7X3|TAOK1\_HUMAN tr|E7ER02|E7ER02\_HUMAN sp|Q9BQG0|MBB1A\_HUMAN tr|I3L1L3|I3L1L3\_HUMAN tr|Q5JU97|Q5JU97\_HUMAN sp|Q71F56|MD13L\_HUMAN sp|Q7Z2Y8|GVIN1\_HUMAN sp|Q86UR5|RIMS1\_HUMAN sp|Q7Z739|YTHD3\_HUMAN tr|K7EMD9|K7EMD9\_HUMAN tr|E9PDN6|E9PDN6\_HUMAN sp|Q9C0A0|CNTP4\_HUMAN sp|Q58FG0|HS905\_HUMAN sp|Q68DQ2|CRBG3\_HUMAN tr|C9J5N2|C9J5N2\_HUMAN sp|Q8N139|ABCA6\_HUMAN sp|Q9Y2L9|LRCH1\_HUMAN sp|Q9UPS8|ANR26\_HUMAN tr|U3KQN1|U3KQN1\_HUMAN tr|A0A087WW77|A0A087WW77\_HUMAN sp|Q15334|L2GL1\_HUMAN sp|Q8N695|SC5A8\_HUMAN sp|P35749|MYH11\_HUMAN tr|D6RGK3|D6RGK3\_HUMAN tr|D6RHW1|D6RHW1\_HUMAN tr|D6RIY1|D6RIY1\_HUMAN sp|Q9HBM6|TAF9B\_HUMAN tr|H7C5R4|H7C5R4\_HUMAN sp|Q9BXR3|POK6\_HUMAN sp|Q2TAZ0|ATG2A\_HUMAN tr|C9IZ08|C9IZ08\_HUMAN tr|H0Y4E7|H0Y4E7\_HUMAN sp|Q14C86|GAPD1\_HUMAN sp|Q5GFL6|VWA2\_HUMAN sp|P0C221|CC175\_HUMAN tr|H0Y837|H0Y837\_HUMAN sp|Q8N9V7|TOPZ1\_HUMAN sp|P35579|MYH9\_HUMAN sp|Q8IWB6|TEX14\_HUMAN tr|H0Y4R5|H0Y4R5\_HUMAN tr|E7EWP2|E7EWP2\_HUMAN sp|A8MUL3|ADAS1\_HUMAN tr|E7EMG8|E7EMG8\_HUMAN tr|E7EM97|E7EM97\_HUMAN sp|Q9BXT8|RNF17\_HUMAN tr|F5H4Z8|F5H4Z8\_HUMAN tr|F8W9L8|F8W9L8\_HUMAN sp|A8MW92|P20L1\_HUMAN sp|P49746|TSP3\_HUMAN sp|Q96Q42|ALS2\_HUMAN tr|B7WPD9|B7WPD9\_HUMAN Q3T052 sp|Q15434|RBMS2\_HUMAN sp|O60447|EVI5\_HUMAN sp|Q86WJ1|CHD1L\_HUMAN sp|O60563|CCNT1\_HUMAN tr|E9PDE8|E9PDE8\_HUMAN sp|Q13761|RUNX3\_HUMAN sp|Q5W0Q7|USPL1\_HUMAN P01030 sp|Q5XKL5|BTBD8\_HUMAN sp|P57678|GEMI4\_HUMAN tr|I3L2C7|I3L2C7\_HUMAN sp|Q8NFU7|TET1\_HUMAN sp|P54756|EPHA5\_HUMAN tr|Q5HY54|Q5HY54\_HUMAN sp|P21333|FLNA\_HUMAN tr|A0A096LNW1|A0A096LNW1\_HUMAN sp|Q9UI08|EVL\_HUMAN sp|Q9HBL0|TENS1\_HUMAN tr|E9PGF5|E9PGF5\_HUMAN tr|E9PF55|E9PF55\_HUMAN tr|A0A087WWW7|A0A087WWW7\_HUMAN tr|F5H4B5|F5H4B5\_HUMAN tr|F5GZI5|F5GZI5\_HUMAN tr|F5GX59|F5GX59\_HUMAN sp|Q5RGS2|S31A2\_HUMAN sp|Q6Y7W6|PERQ2\_HUMAN sp|Q3SY84|K2C71\_HUMAN tr|E9PEW5|E9PEW5\_HUMAN tr|H0Y7V8|H0Y7V8\_HUMAN sp|Q86XN7|PRSR1\_HUMAN sp|Q86V15|CASZ1\_HUMAN sp|P42336|PK3CA\_HUMAN sp|Q96RK0|CIC\_HUMAN tr|D6R9V6|D6R9V6\_HUMAN tr|A6NJ38|A6NJ38\_HUMAN sp|Q86WT6|TRI69\_HUMAN sp|Q6UB99|ANR11\_HUMAN tr|E7EV93|E7EV93\_HUMAN sp|Q68DN1|CB016\_HUMAN tr|D6REB5|D6REB5\_HUMAN sp|Q96DT5|DYH11\_HUMAN tr|A0A087WYC6|A0A087WYC6\_HUMAN tr|A0A087WX84|A0A087WX84\_HUMAN sp|O94762|RECQ5\_HUMAN tr|H0YDE5|H0YDE5\_HUMAN sp|A8MXV4|NUD19\_HUMAN sp|Q6UVK1|CSPG4\_HUMAN tr|B7Z651|B7Z651\_HUMAN tr|J3KN16|J3KN16\_HUMAN sp|P19634|SL9A1\_HUMAN sp|O60645|EXOC3\_HUMAN sp|P63136|POK25\_HUMAN sp|Q8NB46|ANR52\_HUMAN sp|Q9ULK4|MED23\_HUMAN tr|Q5JWT2|Q5JWT2\_HUMAN tr|Q5T1Z4|Q5T1Z4\_HUMAN tr|Q5T1Z8|Q5T1Z8\_HUMAN sp|Q05469|LIPS\_HUMAN sp|P56159|GFRA1\_HUMAN tr|A0A087X0I0|A0A087X0I0\_HUMAN sp|Q6AWC2|WWC2\_HUMAN sp|P59047|NALP5\_HUMAN sp|Q56P42|PYDC2\_HUMAN sp|Q8IYB4|PEX5R\_HUMAN sp|Q96T37|RBM15\_HUMAN sp|Q8N3P4|VPS8\_HUMAN tr|E7EVL1|E7EVL1\_HUMAN tr|A0A087WTI0|A0A087WTI0\_HUMAN tr|A0A087WTQ9|A0A087WTQ9\_HUMAN tr|A0A075B747|A0A075B747\_HUMAN sp|Q2TB10|ZN800\_HUMAN Q497I4 sp|O15031|PLXB2\_HUMAN sp|Q9UQG0|POK11\_HUMAN sp|Q8TB24|RIN3\_HUMAN sp|Q5T197|DCST1\_HUMAN tr|H0Y659|H0Y659\_HUMAN sp|O60336|MABP1\_HUMAN tr|A0A087WU38|A0A087WU38\_HUMAN tr|A0A087WX15|A0A087WX15\_HUMAN sp|Q9C0E4|GRIP2\_HUMAN sp|Q8N1G1|REXO1\_HUMAN sp|Q9UQD0|SCN8A\_HUMAN sp|Q9ULJ6|ZMIZ1\_HUMAN tr|H0Y6Z7|H0Y6Z7\_HUMAN sp|P48553|TPC10\_HUMAN sp|Q9NPG4|PCD12\_HUMAN tr|A0A087X0I6|A0A087X0I6\_HUMAN sp|Q9P0L2|MARK1\_HUMAN sp|Q86YZ3|HORN\_HUMAN Q86YZ3 tr|H0YGZ2|H0YGZ2\_HUMAN sp|P49281|NRAM2\_HUMAN sp|Q7RTY1|MOT9\_HUMAN Q3SY84 sp|Q86UP3|ZFHX4\_HUMAN tr|H0YKQ8|H0YKQ8\_HUMAN sp|Q9UJ41|RABX5\_HUMAN sp|Q3YBM2|T176B\_HUMAN sp|Q8N423|LIRB2\_HUMAN sp|Q4G0N8|SL9C1\_HUMAN sp|Q9H0X9|OSBL5\_HUMAN tr|A0A087WZ74|A0A087WZ74\_HUMAN sp|Q86UE8|TLK2\_HUMAN sp|O60343|TBCD4\_HUMAN sp|Q5M9N0|CD158\_HUMAN sp|Q96MS0|ROBO3\_HUMAN tr|E7EX52|E7EX52\_HUMAN tr|E7EN28|E7EN28\_HUMAN tr|E7EVD6|E7EVD6\_HUMAN sp|Q9H1B7|I2BPL\_HUMAN sp|Q9HCM3|K1549\_HUMAN tr|R4GNB2|R4GNB2\_HUMAN sp|Q9UMS4|PRP19\_HUMAN sp|O43897|TLL1\_HUMAN tr|E9PD25|E9PD25\_HUMAN sp|Q86UB2|BIVM\_HUMAN sp|Q8TC99|FNDC8\_HUMAN sp|Q58EX2|SDK2\_HUMAN sp|Q99758|ABCA3\_HUMAN sp|Q70Z44|5HT3D\_HUMAN tr|A0A087WZP6|A0A087WZP6\_HUMAN sp|Q8IZ07|AN13A\_HUMAN tr|J3KP11|J3KP11\_HUMAN tr|F8W9Z1|F8W9Z1\_HUMAN sp|Q15878|CAC1E\_HUMAN sp|Q5TCY1|TTBK1\_HUMAN tr|J3KP58|J3KP58\_HUMAN tr|E5RFJ0|E5RFJ0\_HUMAN tr|H0Y9B3|H0Y9B3\_HUMAN tr|Q08E86|Q08E86\_HUMAN tr|K7EQ86|K7EQ86\_HUMAN sp|Q14667|K0100\_HUMAN tr|B4DE15|B4DE15\_HUMAN tr|B5MC89|B5MC89\_HUMAN sp|O00443|P3C2A\_HUMAN sp|Q5S007|LRRK2\_HUMAN sp|Q5JQC9|AKAP4\_HUMAN sp|O00159|MYO1C\_HUMAN tr|F5H6E2|F5H6E2\_HUMAN tr|E9PFK9|E9PFK9\_HUMAN tr|M0R288|M0R288\_HUMAN tr|A0A087WWP4|A0A087WWP4\_HUMAN sp|Q92731|ESR2\_HUMAN tr|C9JJX6|C9JJX6\_HUMAN tr|H0Y7I9|H0Y7I9\_HUMAN sp|P48751|B3A3\_HUMAN sp|Q6ZRI0|OTOG\_HUMAN sp|P46926|GNPI1\_HUMAN tr|D6RFF8|D6RFF8\_HUMAN tr|A0A087WTA1|A0A087WTA1\_HUMAN tr|F5H107|F5H107\_HUMAN tr|H7C1F9|H7C1F9\_HUMAN sp|Q2PPJ7|RGPA2\_HUMAN sp|O94985|CSTN1\_HUMAN sp|Q99959|PKP2\_HUMAN tr|B8QGS9|B8QGS9\_HUMAN tr|A0A087WZ13|A0A087WZ13\_HUMAN tr|E9PAU2|E9PAU2\_HUMAN sp|P12035|K2C3\_HUMAN tr|F8VU39|F8VU39\_HUMAN sp|Q9UIF9|BAZ2A\_HUMAN tr|J3KPG5|J3KPG5\_HUMAN tr|A0A087WTJ9|A0A087WTJ9\_HUMAN tr|A0A087WXW6|A0A087WXW6\_HUMAN sp|Q92766|RREB1\_HUMAN sp|Q2M1P5|KIF7\_HUMAN sp|Q8TEM1|PO210\_HUMAN tr|B7ZBA8|B7ZBA8\_HUMAN sp|Q702N8|XIRP1\_HUMAN sp|P25092|GUC2C\_HUMAN sp|Q9Y4I1|MYO5A\_HUMAN tr|A0A087WY00|A0A087WY00\_HUMAN tr|F8WE88|F8WE88\_HUMAN tr|F8W6H6|F8W6H6\_HUMAN tr|B4DVB0|B4DVB0\_HUMAN sp|P51816|AFF2\_HUMAN sp|Q9NXZ1|SAGE1\_HUMAN sp|Q95460|HMR1\_HUMAN tr|G3V5S2|G3V5S2\_HUMAN tr|E5RHT3|E5RHT3\_HUMAN tr|D6RA40|D6RA40\_HUMAN sp|Q9Y5G9|PCDG4\_HUMAN tr|A0A087WT05|A0A087WT05\_HUMAN tr|A0A087WTI2|A0A087WTI2\_HUMAN sp|Q6ZNE5|BAKOR\_HUMAN tr|C9J5F6|C9J5F6\_HUMAN tr|D2CGD1|D2CGD1\_HUMAN sp|P21802|FGFR2\_HUMAN sp|Q8WXG6|MADD\_HUMAN sp|Q8N6P7|I22R1\_HUMAN tr|F5H5K1|F5H5K1\_HUMAN tr|J3KTP0|J3KTP0\_HUMAN tr|J3QSU1|J3QSU1\_HUMAN sp|Q96QE4|LR37B\_HUMAN tr|A0A087X1N2|A0A087X1N2\_HUMAN sp|Q03701|CEBPZ\_HUMAN tr|D6RB59|D6RB59\_HUMAN tr|H7C2D3|H7C2D3\_HUMAN sp|O00562|PITM1\_HUMAN sp|Q92628|K0232\_HUMAN tr|B1AM31|B1AM31\_HUMAN sp|Q8NCW5|NNRE\_HUMAN sp|Q7Z6E9|RBBP6\_HUMAN tr|A0A087WTT8|A0A087WTT8\_HUMAN sp|Q15029|U5S1\_HUMAN sp|Q6VMQ6|MCAF1\_HUMAN tr|H7C4X9|H7C4X9\_HUMAN sp|Q9ULU4|PKCB1\_HUMAN sp|Q8IW52|SLIK4\_HUMAN sp|Q5VYK3|ECM29\_HUMAN sp|Q6PJG2|EMSA1\_HUMAN sp|Q9P2J2|TUTLA\_HUMAN tr|E9PNZ4|E9PNZ4\_HUMAN tr|C9J185|C9J185\_HUMAN sp|Q8NBJ4|GOLM1\_HUMAN sp|P33981|TTK\_HUMAN sp|Q9UBF2|COPG2\_HUMAN sp|Q96JM3|CHAP1\_HUMAN sp|P20701|ITAL\_HUMAN tr|C9J330|C9J330\_HUMAN sp|Q9UIW2|PLXA1\_HUMAN sp|Q92674|CENPI\_HUMAN P02769 sp|O60337|MARH6\_HUMAN sp|A1X283|SPD2B\_HUMAN sp|Q6IQ23|PKHA7\_HUMAN tr|V9GY68|V9GY68\_HUMAN tr|E9PKC0|E9PKC0\_HUMAN sp|Q5T0F9|C2D1B\_HUMAN sp|Q5VUG0|SMBT2\_HUMAN sp|P47898|5HT5A\_HUMAN tr|G3V2A4|G3V2A4\_HUMAN sp|Q16363|LAMA4\_HUMAN sp|Q6ZS17|FA65A\_HUMAN sp|Q14687|GSE1\_HUMAN tr|B7Z2B6|B7Z2B6\_HUMAN sp|Q9H8Y5|ANKZ1\_HUMAN sp|O15061|SYNEM\_HUMAN tr|D6RCE2|D6RCE2\_HUMAN sp|P05154|IPSP\_HUMAN sp|O15013|ARHGA\_HUMAN Q92764 tr|C4AM86|C4AM86\_HUMAN sp|Q02846|GUC2D\_HUMAN tr|F5GXS0|F5GXS0\_HUMAN sp|P0C0L5|CO4B\_HUMAN sp|Q9Y5Y9|SCNAA\_HUMAN tr|E9PF42|E9PF42\_HUMAN sp|Q70EL1|UBP54\_HUMAN sp|Q9H0E3|SP130\_HUMAN sp|Q5VWQ8|DAB2P\_HUMAN sp|O15050|TRNK1\_HUMAN tr|H0Y7N4|H0Y7N4\_HUMAN sp|Q14114|LRP8\_HUMAN tr|H7C4V8|H7C4V8\_HUMAN sp|Q15811|ITSN1\_HUMAN sp|O43497|CAC1G\_HUMAN tr|A0A087X060|A0A087X060\_HUMAN tr|A0A096LP49|A0A096LP49\_HUMAN tr|G3V2D8|G3V2D8\_HUMAN sp|Q5JR59|MTUS2\_HUMAN tr|J3KQA9|J3KQA9\_HUMAN tr|J3KNE0|J3KNE0\_HUMAN sp|A6NKT7|RGPD3\_HUMAN sp|Q12948|FOXC1\_HUMAN sp|Q9H2D6|TARA\_HUMAN sp|Q9H2Y7|ZN106\_HUMAN sp|Q14146|URB2\_HUMAN sp|O15014|ZN609\_HUMAN sp|Q6ZNA4|RN111\_HUMAN tr|H0YJP0|H0YJP0\_HUMAN sp|Q8WWV6|FCAMR\_HUMAN tr|H0Y2P5|H0Y2P5\_HUMAN tr|Q60FE5|Q60FE5\_HUMAN sp|Q3MII6|TBC25\_HUMAN tr|H7BYG8|H7BYG8\_HUMAN sp|Q8NCR6|SMRP1\_HUMAN tr|F5GWM2|F5GWM2\_HUMAN sp|Q6P0Q8|MAST2\_HUMAN tr|H0Y488|H0Y488\_HUMAN sp|P07686|HEXB\_HUMAN sp|Q8TCW7|ZPLD1\_HUMAN sp|Q8N4Q0|ZADH2\_HUMAN tr|A0A087WTM4|A0A087WTM4\_HUMAN sp|O15164|TIF1A\_HUMAN tr|H7BYL6|H7BYL6\_HUMAN tr|C9JEA7|C9JEA7\_HUMAN tr|U3KQJ8|U3KQJ8\_HUMAN sp|O95373|IPO7\_HUMAN tr|F5GWR7|F5GWR7\_HUMAN sp|P0C091|FREM3\_HUMAN sp|Q86YP4|P66A\_HUMAN sp|Q6UXX5|ITIH6\_HUMAN tr|F8W108|F8W108\_HUMAN sp|P50748|KNTC1\_HUMAN tr|A0A087WYM7|A0A087WYM7\_HUMAN sp|Q8NEV4|MYO3A\_HUMAN sp|Q08378|GOGA3\_HUMAN tr|A0A087WUE6|A0A087WUE6\_HUMAN tr|H3BSL4|H3BSL4\_HUMAN tr|E7EQ12|E7EQ12\_HUMAN tr|J3QS44|J3QS44\_HUMAN tr|H0YGH5|H0YGH5\_HUMAN tr|D6RHI0|D6RHI0\_HUMAN sp|P14635|CCNB1\_HUMAN sp|O15047|SET1A\_HUMAN sp|Q5VWK5|IL23R\_HUMAN sp|Q8NEJ9|NGDN\_HUMAN sp|Q9NP59|S40A1\_HUMAN sp|Q8NGF9|OR4X2\_HUMAN P08729 Q3KNV1 sp|P08729|K2C7\_HUMAN sp|P15498|VAV\_HUMAN tr|Q96D37|Q96D37\_HUMAN tr|F5H5P4|F5H5P4\_HUMAN tr|H7BZU5|H7BZU5\_HUMAN tr|E9PFI2|E9PFI2\_HUMAN sp|Q9Y2K9|STB5L\_HUMAN tr|C9JQS3|C9JQS3\_HUMAN sp|P30968|GNRHR\_HUMAN sp|Q86UL8|MAGI2\_HUMAN tr|Q2HIZ1|Q2HIZ1\_HUMAN tr|H0YCY6|H0YCY6\_HUMAN sp|Q9P1Z0|ZBTB4\_HUMAN sp|O14578|CTRO\_HUMAN sp|Q04671|P\_HUMAN tr|G3V311|G3V311\_HUMAN sp|Q9NRY4|RHG35\_HUMAN tr|H0YDK8|H0YDK8\_HUMAN sp|P57679|EVC\_HUMAN sp|Q12766|HMGX3\_HUMAN tr|J3QQJ5|J3QQJ5\_HUMAN sp|Q9Y2L5|TPPC8\_HUMAN sp|Q96Q27|ASB2\_HUMAN tr|G5E9E7|G5E9E7\_HUMAN sp|Q07157|ZO1\_HUMAN tr|G3V1L9|G3V1L9\_HUMAN tr|A0A087X0K9|A0A087X0K9\_HUMAN sp|Q96P50|ACAP3\_HUMAN tr|B3KV94|B3KV94\_HUMAN sp|Q9UGL1|KDM5B\_HUMAN tr|A0A087WYS3|A0A087WYS3\_HUMAN sp|Q8TCU6|PREX1\_HUMAN sp|Q8TD16|BICD2\_HUMAN sp|Q9NTI2|AT8A2\_HUMAN sp|Q86UT6|NLRX1\_HUMAN sp|O75037|KI21B\_HUMAN sp|Q7Z6G8|ANS1B\_HUMAN sp|Q9H4Z2|ZN335\_HUMAN tr|H9KVB3|H9KVB3\_HUMAN sp|Q96KV7|WDR90\_HUMAN tr|F8VUX9|F8VUX9\_HUMAN sp|Q7Z401|MYCPP\_HUMAN tr|E9PIL3|E9PIL3\_HUMAN tr|H0YJC9|H0YJC9\_HUMAN Q9DCV7 tr|E7ERM3|E7ERM3\_HUMAN tr|D6RAA5|D6RAA5\_HUMAN tr|Q4VXL8|Q4VXL8\_HUMAN sp|Q09428|ABCC8\_HUMAN sp|Q9BQN1|FA83C\_HUMAN sp|Q8IVT2|MISP\_HUMAN tr|D6RJB7|D6RJB7\_HUMAN sp|O76094|SRP72\_HUMAN sp|Q96HH6|TMM19\_HUMAN sp|Q9H1R3|MYLK2\_HUMAN tr|E7ETD6|E7ETD6\_HUMAN tr|H0Y326|H0Y326\_HUMAN sp|Q8IUH2|CREG2\_HUMAN sp|Q5T8A7|PPR26\_HUMAN sp|Q8TEW8|PAR3L\_HUMAN sp|P56975|NRG3\_HUMAN sp|Q7LBC6|KDM3B\_HUMAN sp|Q9P2P1|NYNRI\_HUMAN sp|O75177|CREST\_HUMAN sp|Q9Y5S2|MRCKB\_HUMAN sp|P19367|HXK1\_HUMAN tr|F8WAE6|F8WAE6\_HUMAN sp|Q9UHB6|LIMA1\_HUMAN sp|Q8NG66|NEK11\_HUMAN tr|E9PHI8|E9PHI8\_HUMAN sp|Q92859|NEO1\_HUMAN sp|Q8TEW0|PARD3\_HUMAN Q2UVX4 tr|B1AJS1|B1AJS1\_HUMAN sp|O14525|ASTN1\_HUMAN sp|Q9BQ87|TBL1Y\_HUMAN sp|Q15643|TRIPB\_HUMAN tr|A0A087WXK9|A0A087WXK9\_HUMAN sp|Q5VXH5|PRAM7\_HUMAN sp|A6NMC2|PRA24\_HUMAN sp|Q5VWM4|PRAM8\_HUMAN tr|B7ZMI5|B7ZMI5\_HUMAN tr|A0A087X1I7|A0A087X1I7\_HUMAN sp|Q14563|SEM3A\_HUMAN tr|D6RJ96|D6RJ96\_HUMAN sp|O95757|HS74L\_HUMAN tr|I3L0K7|I3L0K7\_HUMAN sp|P29459|IL12A\_HUMAN sp|O75443|TECTA\_HUMAN tr|E7ERH1|E7ERH1\_HUMAN sp|P42684|ABL2\_HUMAN sp|P29400|CO4A5\_HUMAN sp|Q6ZSZ5|ARHGI\_HUMAN tr|M0R014|M0R014\_HUMAN sp|A4D2P6|GRD2I\_HUMAN sp|Q460N5|PAR14\_HUMAN sp|Q9Y2I9|TBC30\_HUMAN sp|Q7Z5J8|ANKAR\_HUMAN sp|Q7KZ85|SPT6H\_HUMAN sp|Q7Z2T5|TRM1L\_HUMAN sp|Q9NQX3|GEPH\_HUMAN tr|F5H039|F5H039\_HUMAN tr|F8W726|F8W726\_HUMAN sp|Q14157|UBP2L\_HUMAN sp|Q96S15|WDR24\_HUMAN tr|A0A096LNG8|A0A096LNG8\_HUMAN sp|Q9UFN0|NPS3A\_HUMAN sp|O75582|KS6A5\_HUMAN tr|B7Z2Y5|B7Z2Y5\_HUMAN sp|Q15058|KIF14\_HUMAN tr|H7C3P5|H7C3P5\_HUMAN sp|Q6NUJ5|PWP2B\_HUMAN sp|O60271|JIP4\_HUMAN tr|A0A087X2D8|A0A087X2D8\_HUMAN sp|P39880|CUX1\_HUMAN tr|H7C1U3|H7C1U3\_HUMAN sp|Q9Y4F5|C170B\_HUMAN tr|H0Y517|H0Y517\_HUMAN tr|J3KQR7|J3KQR7\_HUMAN tr|B4DJX4|B4DJX4\_HUMAN sp|P54132|BLM\_HUMAN tr|H0YNU5|H0YNU5\_HUMAN sp|Q68DA7|FMN1\_HUMAN tr|H0YM30|H0YM30\_HUMAN sp|Q9P0K7|RAI14\_HUMAN tr|E9PN62|E9PN62\_HUMAN sp|Q96SF7|TBX15\_HUMAN tr|B7Z5R6|B7Z5R6\_HUMAN tr|C9JNS8|C9JNS8\_HUMAN sp|Q8IZF0|NALCN\_HUMAN sp|Q8WUI4|HDAC7\_HUMAN sp|P08572|CO4A2\_HUMAN sp|Q8TC56|FA71B\_HUMAN sp|Q96PV0|SYGP1\_HUMAN sp|O95382|M3K6\_HUMAN tr|I3L4W9|I3L4W9\_HUMAN sp|Q5VTT5|MYOM3\_HUMAN sp|Q14997|PSME4\_HUMAN sp|Q49A26|GLYR1\_HUMAN tr|I3L225|I3L225\_HUMAN sp|P19021|AMD\_HUMAN tr|E7EPU2|E7EPU2\_HUMAN sp|Q9Y2E4|DIP2C\_HUMAN sp|Q14160|SCRIB\_HUMAN tr|B2RNG4|B2RNG4\_HUMAN sp|Q6UXM1|LRIG3\_HUMAN tr|H0YJF0|H0YJF0\_HUMAN sp|Q9HBG6|IF122\_HUMAN tr|H0YBE8|H0YBE8\_HUMAN sp|Q10567|AP1B1\_HUMAN sp|Q8N6G6|ATL1\_HUMAN tr|C9J6U3|C9J6U3\_HUMAN tr|E7EWV1|E7EWV1\_HUMAN tr|B0QYK0|B0QYK0\_HUMAN sp|Q01844|EWS\_HUMAN sp|Q70CQ4|UBP31\_HUMAN sp|Q9P281|BAHC1\_HUMAN tr|F8WBW8|F8WBW8\_HUMAN tr|E7ESJ3|E7ESJ3\_HUMAN sp|P50991|TCPD\_HUMAN tr|A0A087WXK8|A0A087WXK8\_HUMAN sp|Q92905|CSN5\_HUMAN sp|Q15477|SKIV2\_HUMAN tr|B1ANM7|B1ANM7\_HUMAN tr|A0A087WZE4|A0A087WZE4\_HUMAN tr|U5GXS0|U5GXS0\_HUMAN sp|Q5VYJ5|MALR1\_HUMAN sp|Q9P2M7|CING\_HUMAN sp|Q8TEK3|DOT1L\_HUMAN tr|H7C5R8|H7C5R8\_HUMAN sp|Q7Z5N4|SDK1\_HUMAN tr|A0A087WTQ6|A0A087WTQ6\_HUMAN P12035 tr|A0A087WXM3|A0A087WXM3\_HUMAN sp|Q9UHW9|S12A6\_HUMAN sp|Q96NW4|ANR27\_HUMAN tr|E9PKD2|E9PKD2\_HUMAN sp|O43432|IF4G3\_HUMAN tr|G8JLD3|G8JLD3\_HUMAN tr|X6RLX0|X6RLX0\_HUMAN sp|Q8IUD2|RB6I2\_HUMAN sp|O75881|CP7B1\_HUMAN tr|R4GN62|R4GN62\_HUMAN tr|B0QYS7|B0QYS7\_HUMAN tr|B0QYS6|B0QYS6\_HUMAN tr|B1AKB5|B1AKB5\_HUMAN sp|P19484|TFEB\_HUMAN tr|B1AKB4|B1AKB4\_HUMAN sp|Q7Z7M0|MEGF8\_HUMAN sp|Q5VZ89|DEN4C\_HUMAN tr|H0Y3Q9|H0Y3Q9\_HUMAN sp|P41252|SYIC\_HUMAN tr|I3L520|I3L520\_HUMAN sp|Q06033|ITIH3\_HUMAN tr|A0A087WW43|A0A087WW43\_HUMAN sp|Q9Y5F6|PCDGM\_HUMAN sp|Q8N608|DPP10\_HUMAN sp|P49790|NU153\_HUMAN sp|Q2WGN9|GAB4\_HUMAN sp|P42695|CNDD3\_HUMAN sp|O43747|AP1G1\_HUMAN tr|Q3KPI9|Q3KPI9\_HUMAN sp|A0AUZ9|KAL1L\_HUMAN sp|Q63HK3|ZKSC2\_HUMAN sp|Q15637|SF01\_HUMAN tr|H7C2C7|H7C2C7\_HUMAN tr|H3BNT0|H3BNT0\_HUMAN tr|H3BPX6|H3BPX6\_HUMAN tr|H3BUA8|H3BUA8\_HUMAN tr|H3BMW6|H3BMW6\_HUMAN sp|Q10571|MN1\_HUMAN tr|H0YHV1|H0YHV1\_HUMAN tr|E9PJW6|E9PJW6\_HUMAN sp|O43427|FIBP\_HUMAN tr|E9PSD3|E9PSD3\_HUMAN sp|A6NC98|CC88B\_HUMAN sp|P32004|L1CAM\_HUMAN tr|H7C066|H7C066\_HUMAN sp|Q8WU58|F222B\_HUMAN sp|Q8TDW5|SYTL5\_HUMAN sp|Q9H6X2|ANTR1\_HUMAN tr|F5GY56|F5GY56\_HUMAN sp|P12111|CO6A3\_HUMAN tr|E9PIN0|E9PIN0\_HUMAN tr|F6VVE8|F6VVE8\_HUMAN sp|Q9NQX4|MYO5C\_HUMAN tr|E9PR38|E9PR38\_HUMAN sp|Q14671|PUM1\_HUMAN tr|B4DGQ7|B4DGQ7\_HUMAN sp|Q9Y4F4|F179B\_HUMAN tr|G3XAE9|G3XAE9\_HUMAN sp|O95800|GPR75\_HUMAN sp|Q6P2Q9|PRP8\_HUMAN tr|G3XCN8|G3XCN8\_HUMAN sp|Q13099|IFT88\_HUMAN tr|H0YA26|H0YA26\_HUMAN sp|O43795|MYO1B\_HUMAN tr|E9PDF6|E9PDF6\_HUMAN sp|Q8N307|MUC20\_HUMAN tr|C9JJE7|C9JJE7\_HUMAN sp|Q9HAV4|XPO5\_HUMAN sp|Q9UBZ9|REV1\_HUMAN sp|O75143|ATG13\_HUMAN sp|Q08050|FOXM1\_HUMAN tr|H0Y3A3|H0Y3A3\_HUMAN tr|E7ESU0|E7ESU0\_HUMAN sp|Q14683|SMC1A\_HUMAN tr|G8JLG1|G8JLG1\_HUMAN sp|Q6ZU65|UBN2\_HUMAN tr|E9PGV9|E9PGV9\_HUMAN tr|A6NNK5|A6NNK5\_HUMAN sp|P19532|TFE3\_HUMAN tr|C9J8B6|C9J8B6\_HUMAN tr|C9JF43|C9JF43\_HUMAN tr|C9JUR7|C9JUR7\_HUMAN sp|Q8IY67|RAVR1\_HUMAN tr|B9DI81|B9DI81\_HUMAN sp|Q14993|COJA1\_HUMAN tr|F8W1P7|F8W1P7\_HUMAN sp|Q8TF66|LRC15\_HUMAN tr|A0A087WXI2|A0A087WXI2\_HUMAN sp|Q9Y6R7|FCGBP\_HUMAN sp|Q6PIJ6|FBX38\_HUMAN tr|J3QLK5|J3QLK5\_HUMAN tr|H3BM45|H3BM45\_HUMAN tr|H3BUZ5|H3BUZ5\_HUMAN sp|Q9P286|PAK7\_HUMAN tr|H7BYJ3|H7BYJ3\_HUMAN sp|Q01538|MYT1\_HUMAN sp|P49368|TCPG\_HUMAN sp|Q9H967|WDR76\_HUMAN tr|E9PK50|E9PK50\_HUMAN sp|Q6P3W7|SCYL2\_HUMAN tr|H0YDQ8|H0YDQ8\_HUMAN tr|J3QQW9|J3QQW9\_HUMAN sp|Q14689|DIP2A\_HUMAN sp|Q15022|SUZ12\_HUMAN tr|H0Y5J3|H0Y5J3\_HUMAN sp|O15550|KDM6A\_HUMAN tr|F8W8R6|F8W8R6\_HUMAN tr|F5H6S1|F5H6S1\_HUMAN tr|A0A087X0R0|A0A087X0R0\_HUMAN tr|H0Y6I7|H0Y6I7\_HUMAN sp|A7E2V4|ZSWM8\_HUMAN tr|S4R410|S4R410\_HUMAN sp|O94822|LTN1\_HUMAN sp|Q9Y692|GMEB1\_HUMAN sp|O60282|KIF5C\_HUMAN sp|Q8N6C5|IGSF1\_HUMAN tr|H0YIS8|H0YIS8\_HUMAN tr|H0YHY1|H0YHY1\_HUMAN tr|E9PEK0|E9PEK0\_HUMAN tr|X6RI79|X6RI79\_HUMAN sp|O60241|BAI2\_HUMAN tr|A2A3C2|A2A3C2\_HUMAN sp|Q08211|DHX9\_HUMAN sp|Q9NYV4|CDK12\_HUMAN sp|Q96JG6|CC132\_HUMAN tr|X6R7D6|X6R7D6\_HUMAN tr|V9GY86|V9GY86\_HUMAN sp|Q99700|ATX2\_HUMAN sp|Q9UBW7|ZMYM2\_HUMAN sp|P43354|NR4A2\_HUMAN sp|P17948|VGFR1\_HUMAN sp|Q9UPX8|SHAN2\_HUMAN tr|A6NHU9|A6NHU9\_HUMAN sp|O14802|RPC1\_HUMAN tr|A2A2R0|A2A2R0\_HUMAN sp|Q8TDL5|BPIB1\_HUMAN sp|Q68D10|SPT2\_HUMAN sp|P22670|RFX1\_HUMAN sp|Q9NRJ4|TULP4\_HUMAN sp|O95528|GTR10\_HUMAN sp|Q9NQT8|KI13B\_HUMAN sp|Q3T906|GNPTA\_HUMAN sp|P43304|GPDM\_HUMAN sp|Q96PQ1|SIG12\_HUMAN tr|A0A087WW84|A0A087WW84\_HUMAN sp|Q13698|CAC1S\_HUMAN tr|B1ALM3|B1ALM3\_HUMAN sp|Q92835|SHIP1\_HUMAN tr|C9JGE3|C9JGE3\_HUMAN tr|J3KP75|J3KP75\_HUMAN sp|Q9H987|SYP2L\_HUMAN tr|I1E4Y6|I1E4Y6\_HUMAN sp|Q9P2S2|NRX2A\_HUMAN sp|Q6P1X5|TAF2\_HUMAN tr|G5E9G7|G5E9G7\_HUMAN sp|Q86XA9|HTR5A\_HUMAN tr|F5H619|F5H619\_HUMAN tr|D6RA42|D6RA42\_HUMAN tr|Q5SR50|Q5SR50\_HUMAN tr|F8W9P4|F8W9P4\_HUMAN sp|Q86U86|PB1\_HUMAN tr|E7EVG2|E7EVG2\_HUMAN sp|Q9BVN2|RUSC1\_HUMAN sp|P02549|SPTA1\_HUMAN sp|Q5TCS8|KAD9\_HUMAN tr|B1AMJ5|B1AMJ5\_HUMAN tr|E9PGW9|E9PGW9\_HUMAN tr|F5H6I8|F5H6I8\_HUMAN sp|Q4ZHG4|FNDC1\_HUMAN tr|A0A087X248|A0A087X248\_HUMAN tr|H0Y7F7|H0Y7F7\_HUMAN sp|Q96MR6|WDR65\_HUMAN tr|A0A087WVY5|A0A087WVY5\_HUMAN sp|Q8N271|PROM2\_HUMAN sp|P07814|SYEP\_HUMAN sp|Q9UKI8|TLK1\_HUMAN tr|F8W9W0|F8W9W0\_HUMAN tr|Q6P4G0|Q6P4G0\_HUMAN tr|E7ESB6|E7ESB6\_HUMAN tr|X6RAE7|X6RAE7\_HUMAN tr|F5H8B4|F5H8B4\_HUMAN sp|Q8N1F7|NUP93\_HUMAN tr|H3BVG0|H3BVG0\_HUMAN sp|Q9BZV2|S19A3\_HUMAN tr|F5GXJ9|F5GXJ9\_HUMAN sp|Q13740|CD166\_HUMAN tr|K7ELN3|K7ELN3\_HUMAN sp|Q8NEM7|SP20H\_HUMAN tr|E7ESV6|E7ESV6\_HUMAN tr|E7EUP0|E7EUP0\_HUMAN sp|Q9Y666|S12A7\_HUMAN tr|X6RAN8|X6RAN8\_HUMAN sp|Q9NRD8|DUOX2\_HUMAN sp|Q16513|PKN2\_HUMAN sp|P16157|ANK1\_HUMAN sp|Q8WXE9|STON2\_HUMAN sp|O95996|APC2\_HUMAN tr|A0A087X1V3|A0A087X1V3\_HUMAN tr|H0Y9X0|H0Y9X0\_HUMAN sp|P19320|VCAM1\_HUMAN sp|Q8N3V7|SYNPO\_HUMAN sp|Q8WV44|TRI41\_HUMAN tr|E5RJK2|E5RJK2\_HUMAN tr|H3BTF4|H3BTF4\_HUMAN tr|H3BP52|H3BP52\_HUMAN sp|Q9UKX5|ITA11\_HUMAN sp|Q13751|LAMB3\_HUMAN sp|Q8NB91|FANCB\_HUMAN sp|Q6ZMV9|KIF6\_HUMAN sp|O75038|PLCH2\_HUMAN tr|H0Y718|H0Y718\_HUMAN sp|O15054|KDM6B\_HUMAN sp|Q8NGF8|OR4B1\_HUMAN sp|Q8NA82|MARHA\_HUMAN sp|Q8N9B5|JMY\_HUMAN sp|Q8IZP0|ABI1\_HUMAN tr|X6R7S7|X6R7S7\_HUMAN sp|Q6ZUT3|FRMD7\_HUMAN tr|H7C5W5|H7C5W5\_HUMAN sp|Q9UPQ7|PZRN3\_HUMAN sp|Q12805|FBLN3\_HUMAN sp|Q9H0M4|ZCPW1\_HUMAN tr|E7ES00|E7ES00\_HUMAN sp|Q9Y2T1|AXIN2\_HUMAN sp|Q9BXY5|CAYP2\_HUMAN tr|B9A061|B9A061\_HUMAN sp|Q8N398|VW5B2\_HUMAN sp|Q9BPW9|DHRS9\_HUMAN sp|Q9UJX5|APC4\_HUMAN tr|A0A087WY31|A0A087WY31\_HUMAN tr|J3KRK0|J3KRK0\_HUMAN tr|A0A024R7W5|A0A024R7W5\_HUMAN tr|A0A087X0Q1|A0A087X0Q1\_HUMAN sp|Q5FWF5|ESCO1\_HUMAN sp|Q92576|PHF3\_HUMAN sp|Q3BBV0|NBPF1\_HUMAN sp|Q96DN6|MBD6\_HUMAN sp|Q6N043|Z280D\_HUMAN tr|E9PGK7|E9PGK7\_HUMAN tr|H3BNU0|H3BNU0\_HUMAN tr|H7C3U4|H7C3U4\_HUMAN sp|Q14678|KANK1\_HUMAN sp|O60231|DHX16\_HUMAN sp|Q9H2U1|DHX36\_HUMAN tr|C4B7M2|C4B7M2\_HUMAN sp|Q96JI7|SPTCS\_HUMAN sp|P10070|GLI2\_HUMAN sp|Q8N4C6|NIN\_HUMAN tr|C9J066|C9J066\_HUMAN sp|O75952|CABYR\_HUMAN sp|Q96M63|CC114\_HUMAN sp|O43301|HS12A\_HUMAN sp|Q86W24|NAL14\_HUMAN tr|H3BUF6|H3BUF6\_HUMAN sp|Q8WWM7|ATX2L\_HUMAN tr|B1AVV0|B1AVV0\_HUMAN sp|Q8WWZ7|ABCA5\_HUMAN sp|Q13127|REST\_HUMAN sp|Q9UQ26|RIMS2\_HUMAN sp|Q5JRA6|MIA3\_HUMAN tr|F8VU11|F8VU11\_HUMAN tr|H0Y4E8|H0Y4E8\_HUMAN sp|Q9H2X0|CHRD\_HUMAN tr|E7ESX1|E7ESX1\_HUMAN sp|Q5I0G3|MDH1B\_HUMAN tr|H0Y3H2|H0Y3H2\_HUMAN tr|J3QR48|J3QR48\_HUMAN sp|Q9H992|MARH7\_HUMAN tr|A0A075B733|A0A075B733\_HUMAN tr|Q8IWY7|Q8IWY7\_HUMAN sp|Q9H2P9|DPH5\_HUMAN tr|A0A087WXP7|A0A087WXP7\_HUMAN sp|Q8WWQ0|PHIP\_HUMAN sp|Q5GH72|XKR7\_HUMAN sp|Q6ISB3|GRHL2\_HUMAN sp|Q9UN75|PCDAC\_HUMAN tr|S4R3K0|S4R3K0\_HUMAN tr|H7C1X5|H7C1X5\_HUMAN tr|E7EUA2|E7EUA2\_HUMAN tr|K7EMM8|K7EMM8\_HUMAN sp|Q9H9J4|UBP42\_HUMAN sp|Q9HCE3|ZN532\_HUMAN sp|Q9C0D5|TANC1\_HUMAN sp|Q8TB72|PUM2\_HUMAN sp|O15231|ZN185\_HUMAN sp|P10275|ANDR\_HUMAN tr|A0A087X1B6|A0A087X1B6\_HUMAN sp|P53396|ACLY\_HUMAN tr|E9PKF6|E9PKF6\_HUMAN sp|Q5H9R7|PP6R3\_HUMAN tr|H7BXH2|H7BXH2\_HUMAN sp|P54652|HSP72\_HUMAN sp|Q9C0J8|WDR33\_HUMAN tr|V9GZ20|V9GZ20\_HUMAN tr|H0YCJ4|H0YCJ4\_HUMAN tr|F5GYR0|F5GYR0\_HUMAN sp|A6NJI9|LRC72\_HUMAN tr|A0A087WYL6|A0A087WYL6\_HUMAN sp|Q9BU79|TM243\_HUMAN tr|C9JY04|C9JY04\_HUMAN sp|Q9P0X4|CAC1I\_HUMAN tr|E9PDU5|E9PDU5\_HUMAN sp|Q96QC0|PP1RA\_HUMAN sp|Q9NNW5|WDR6\_HUMAN tr|A0A087X295|A0A087X295\_HUMAN tr|H3BU86|H3BU86\_HUMAN tr|E7EVZ1|E7EVZ1\_HUMAN sp|Q07065|CKAP4\_HUMAN tr|B1AP42|B1AP42\_HUMAN sp|O60469|DSCAM\_HUMAN sp|O60312|AT10A\_HUMAN sp|Q562F6|SGOL2\_HUMAN tr|A0A087WZG6|A0A087WZG6\_HUMAN tr|D6RDG4|D6RDG4\_HUMAN sp|Q9P2Q2|FRM4A\_HUMAN sp|Q8NA72|POC5\_HUMAN tr|A6NML8|A6NML8\_HUMAN tr|E9PGR3|E9PGR3\_HUMAN sp|Q16849|PTPRN\_HUMAN tr|D6W5N0|D6W5N0\_HUMAN tr|J3KRQ5|J3KRQ5\_HUMAN sp|Q8IY85|EFC13\_HUMAN sp|P46531|NOTC1\_HUMAN sp|Q6S5L8|SHC4\_HUMAN sp|Q9NZQ8|TRPM5\_HUMAN tr|E9PQF7|E9PQF7\_HUMAN tr|E9PRW0|E9PRW0\_HUMAN tr|A0A087X0Y2|A0A087X0Y2\_HUMAN sp|Q9BVL2|NUPL1\_HUMAN tr|A6NIT2|A6NIT2\_HUMAN tr|A6NMN0|A6NMN0\_HUMAN sp|P37173|TGFR2\_HUMAN sp|B3KS81|SRRM5\_HUMAN sp|Q7Z3K3|POGZ\_HUMAN sp|Q8IWW6|RHG12\_HUMAN sp|O75197|LRP5\_HUMAN sp|P34913|HYES\_HUMAN tr|E9PH32|E9PH32\_HUMAN tr|J3QRF4|J3QRF4\_HUMAN tr|J3QQZ2|J3QQZ2\_HUMAN sp|Q9P0U3|SENP1\_HUMAN tr|E7EQG5|E7EQG5\_HUMAN sp|Q6ZTQ4|CDHR3\_HUMAN sp|Q5JTZ9|SYAM\_HUMAN tr|H0YA93|H0YA93\_HUMAN sp|P42261|GRIA1\_HUMAN tr|C9JXA2|C9JXA2\_HUMAN sp|P29320|EPHA3\_HUMAN tr|E9PHY8|E9PHY8\_HUMAN sp|Q8NDA8|MROH1\_HUMAN tr|E7ENE1|E7ENE1\_HUMAN sp|Q7Z3G6|PRIC2\_HUMAN sp|Q6P0N0|M18BP\_HUMAN sp|O14974|MYPT1\_HUMAN sp|Q8IVK1|GLCM1\_HUMAN sp|Q96BD0|SO4A1\_HUMAN sp|Q6DN14|MCTP1\_HUMAN tr|D3YTJ2|D3YTJ2\_HUMAN sp|A8K8P3|SFI1\_HUMAN sp|Q5VVP1|S31A6\_HUMAN tr|J3KQ19|J3KQ19\_HUMAN sp|Q8N2Q7|NLGN1\_HUMAN sp|Q92896|GSLG1\_HUMAN tr|H0YJ69|H0YJ69\_HUMAN sp|Q6ZMW3|EMAL6\_HUMAN sp|Q5SVQ8|ZBT41\_HUMAN sp|Q5JUK3|KCNT1\_HUMAN tr|C9JZ51|C9JZ51\_HUMAN tr|C9JYL2|C9JYL2\_HUMAN sp|O75112|LDB3\_HUMAN sp|Q96CJ1|EAF2\_HUMAN tr|B4DWJ3|B4DWJ3\_HUMAN sp|P0C0L4|CO4A\_HUMAN sp|Q8N961|ABTB2\_HUMAN Q2KJ62 sp|Q8IZJ3|CPMD8\_HUMAN sp|Q14004|CDK13\_HUMAN sp|Q9C0D6|FHDC1\_HUMAN tr|H0Y7Z9|H0Y7Z9\_HUMAN tr|F5GY88|F5GY88\_HUMAN sp|Q7RTW8|OTOAN\_HUMAN sp|O15440|MRP5\_HUMAN sp|Q8N7Z5|ANR31\_HUMAN tr|E9PDC3|E9PDC3\_HUMAN sp|O00192|ARVC\_HUMAN sp|Q8N5H7|SH2D3\_HUMAN sp|Q6ZMT4|KDM7A\_HUMAN sp|Q9H1B4|NXF5\_HUMAN sp|P53355|DAPK1\_HUMAN tr|A0A087WWY3|A0A087WWY3\_HUMAN tr|B9ZVR7|B9ZVR7\_HUMAN tr|E9PEJ6|E9PEJ6\_HUMAN tr|E7EWI0|E7EWI0\_HUMAN tr|H0YBE7|H0YBE7\_HUMAN sp|P45974|UBP5\_HUMAN sp|Q4VXU2|PAP1L\_HUMAN tr|I3L1L6|I3L1L6\_HUMAN tr|E7EX90|E7EX90\_HUMAN sp|Q14203|DCTN1\_HUMAN tr|H0Y5D1|H0Y5D1\_HUMAN sp|Q8TEU7|RPGF6\_HUMAN tr|E9PCH4|E9PCH4\_HUMAN tr|C9IZN0|C9IZN0\_HUMAN sp|Q684P5|RPGP2\_HUMAN sp|Q9NVN8|GNL3L\_HUMAN sp|Q9H9A6|LRC40\_HUMAN sp|Q8NDZ0|BEND2\_HUMAN sp|Q8N9V6|ANR53\_HUMAN sp|Q3MIW9|DPCR1\_HUMAN sp|P11717|MPRI\_HUMAN tr|J3QSE8|J3QSE8\_HUMAN sp|Q5SY80|CA101\_HUMAN tr|H0Y9Q2|H0Y9Q2\_HUMAN tr|D6RIB5|D6RIB5\_HUMAN tr|C9JUZ5|C9JUZ5\_HUMAN sp|Q05952|STP2\_HUMAN sp|Q9Y226|S22AD\_HUMAN sp|O15381|NVL\_HUMAN sp|O75369|FLNB\_HUMAN sp|O75717|WDHD1\_HUMAN sp|Q659C4|LAR1B\_HUMAN tr|F6S7C4|F6S7C4\_HUMAN sp|Q8TCY9|URGCP\_HUMAN sp|Q6UXN9|WDR82\_HUMAN tr|C9JPC9|C9JPC9\_HUMAN tr|K7EQT8|K7EQT8\_HUMAN sp|Q92698|RAD54\_HUMAN sp|Q8IWJ2|GCC2\_HUMAN tr|E7EVR7|E7EVR7\_HUMAN sp|P10745|RET3\_HUMAN tr|H0YD33|H0YD33\_HUMAN sp|Q8NFY4|SEM6D\_HUMAN sp|Q9ULI1|NWD2\_HUMAN tr|H3BLS9|H3BLS9\_HUMAN tr|A2RU21|A2RU21\_HUMAN tr|F8WA11|F8WA11\_HUMAN sp|Q7Z460|CLAP1\_HUMAN sp|A9Z1Z3|FR1L4\_HUMAN sp|Q92833|JARD2\_HUMAN tr|H7BZ61|H7BZ61\_HUMAN sp|Q12770|SCAP\_HUMAN sp|Q9Y5H2|PCDGB\_HUMAN sp|Q9Y253|POLH\_HUMAN tr|H3BP44|H3BP44\_HUMAN sp|Q9ULL5|PRR12\_HUMAN sp|Q8NFF2|NCKX4\_HUMAN sp|Q9P206|K1522\_HUMAN sp|A3KN83|SBNO1\_HUMAN tr|Q24JQ7|Q24JQ7\_HUMAN tr|H0Y781|H0Y781\_HUMAN sp|Q7Z406|MYH14\_HUMAN tr|F8W8T8|F8W8T8\_HUMAN tr|B7ZCA0|B7ZCA0\_HUMAN sp|P02462|CO4A1\_HUMAN sp|O94856|NFASC\_HUMAN sp|P57071|PRD15\_HUMAN sp|A6NKG5|RTL1\_HUMAN sp|P15586|GNS\_HUMAN sp|Q9NWT1|PK1IP\_HUMAN tr|F5GYF8|F5GYF8\_HUMAN tr|Q76L82|Q76L82\_HUMAN sp|O15265|ATX7\_HUMAN tr|A2A3C1|A2A3C1\_HUMAN sp|Q96ID5|IGS21\_HUMAN tr|A2A3C6|A2A3C6\_HUMAN tr|A2A3C3|A2A3C3\_HUMAN sp|Q96KJ4|MSLNL\_HUMAN sp|O95696|BRD1\_HUMAN tr|A0A075B739|A0A075B739\_HUMAN tr|H0YKT2|H0YKT2\_HUMAN tr|H7C1C4|H7C1C4\_HUMAN sp|Q9BQP9|BPIA3\_HUMAN sp|Q52LW3|RHG29\_HUMAN sp|Q9BZ11|ADA33\_HUMAN tr|A2A2L3|A2A2L3\_HUMAN sp|Q14674|ESPL1\_HUMAN tr|M0QY36|M0QY36\_HUMAN tr|F8VWA3|F8VWA3\_HUMAN tr|F8VVB4|F8VVB4\_HUMAN sp|Q9UHN6|TMEM2\_HUMAN sp|Q9UQ52|CNTN6\_HUMAN sp|Q9NR64|KLHL1\_HUMAN tr|F5H1J3|F5H1J3\_HUMAN sp|Q9H7F0|AT133\_HUMAN sp|Q0VAA2|LR74A\_HUMAN sp|O14715|RGPD8\_HUMAN sp|Q8IY21|DDX60\_HUMAN sp|Q9BY12|SCAPE\_HUMAN sp|Q96S37|S22AC\_HUMAN sp|Q13546|RIPK1\_HUMAN sp|Q9BR39|JPH2\_HUMAN sp|Q9Y6H6|KCNE3\_HUMAN tr|Q2N1I1|Q2N1I1\_HUMAN tr|B9DI82|B9DI82\_HUMAN tr|B7Z3H4|B7Z3H4\_HUMAN sp|Q9Y297|FBW1A\_HUMAN sp|Q8NBP0|TTC13\_HUMAN sp|Q6IPR3|TYW3\_HUMAN sp|P55160|NCKPL\_HUMAN tr|H7C5N3|H7C5N3\_HUMAN sp|Q8NB66|UN13C\_HUMAN tr|A0A087WW63|A0A087WW63\_HUMAN sp|Q9GZU1|MCLN1\_HUMAN tr|H0YJ17|H0YJ17\_HUMAN tr|E7ETZ7|E7ETZ7\_HUMAN sp|Q8IZD2|KMT2E\_HUMAN sp|P08185|CBG\_HUMAN sp|Q9H3S7|PTN23\_HUMAN sp|Q8WWZ4|ABCAA\_HUMAN tr|Q5VWV2|Q5VWV2\_HUMAN sp|Q12965|MYO1E\_HUMAN tr|H0YI20|H0YI20\_HUMAN sp|P00973|OAS1\_HUMAN tr|F8VXY3|F8VXY3\_HUMAN sp|Q7Z4N2|TRPM1\_HUMAN sp|Q8WZ64|ARAP2\_HUMAN sp|Q9HCU4|CELR2\_HUMAN sp|Q5T2Q4|CCYL2\_HUMAN sp|Q674R7|ATG9B\_HUMAN sp|O95677|EYA4\_HUMAN tr|F2Z2Y1|F2Z2Y1\_HUMAN sp|P29558|RBMS1\_HUMAN sp|Q9Y5I2|PCDAA\_HUMAN tr|S4R393|S4R393\_HUMAN sp|Q8N5V2|NGEF\_HUMAN tr|H0Y547|H0Y547\_HUMAN sp|O75400|PR40A\_HUMAN sp|Q8IVE3|PKHH2\_HUMAN sp|Q9Y3R0|GRIP1\_HUMAN sp|Q9NWZ3|IRAK4\_HUMAN sp|Q86SQ7|SDCG8\_HUMAN sp|Q9Y5I4|PCDC2\_HUMAN tr|H0YH27|H0YH27\_HUMAN sp|P49023|PAXI\_HUMAN tr|F5GZ78|F5GZ78\_HUMAN tr|H0Y8K3|H0Y8K3\_HUMAN tr|H0YMQ9|H0YMQ9\_HUMAN tr|F8W6N3|F8W6N3\_HUMAN sp|Q92560|BAP1\_HUMAN sp|P56715|RP1\_HUMAN sp|Q96HA7|TONSL\_HUMAN tr|B1AKY9|B1AKY9\_HUMAN sp|P50993|AT1A2\_HUMAN sp|A4FU69|EFCB5\_HUMAN sp|Q9UK17|KCND3\_HUMAN tr|M0QYC1|M0QYC1\_HUMAN sp|Q5T619|ZN648\_HUMAN sp|Q9Y5G5|PCDG8\_HUMAN sp|O96028|NSD2\_HUMAN tr|F8WC90|F8WC90\_HUMAN tr|A0A087X0F1|A0A087X0F1\_HUMAN tr|H0Y2S9|H0Y2S9\_HUMAN sp|A8MVX0|ARG33\_HUMAN tr|A0A087WV73|A0A087WV73\_HUMAN sp|P46087|NOP2\_HUMAN tr|E7ES10|E7ES10\_HUMAN sp|Q7Z2Q7|LRR70\_HUMAN H-INV:HIT000015463 tr|A0A087X0K8|A0A087X0K8\_HUMAN sp|Q6PRD1|GP179\_HUMAN tr|A0A087WW83|A0A087WW83\_HUMAN sp|P56192|SYMC\_HUMAN sp|Q5FWE3|PRRT3\_HUMAN sp|Q14832|GRM3\_HUMAN ENSEMBL:ENSBTAP00000007350 tr|J3KNQ2|J3KNQ2\_HUMAN tr|H3BN21|H3BN21\_HUMAN sp|Q96PD2|DCBD2\_HUMAN tr|G3V599|G3V599\_HUMAN sp|Q7Z443|PK1L3\_HUMAN sp|Q9UBG0|MRC2\_HUMAN sp|Q5TB80|CE162\_HUMAN tr|A0A087WVP4|A0A087WVP4\_HUMAN sp|Q9HC77|CENPJ\_HUMAN tr|Q5VTI5|Q5VTI5\_HUMAN tr|Q8WY19|Q8WY19\_HUMAN tr|A0A087WUI7|A0A087WUI7\_HUMAN sp|Q5K651|SAMD9\_HUMAN sp|Q9UMS6|SYNP2\_HUMAN sp|Q9NRC6|SPTN5\_HUMAN tr|X6R5I7|X6R5I7\_HUMAN sp|O15303|GRM6\_HUMAN tr|E9PGM9|E9PGM9\_HUMAN tr|F8WCA5|F8WCA5\_HUMAN sp|P78332|RBM6\_HUMAN sp|Q8IWY9|CDAN1\_HUMAN sp|A6NND4|O2AT4\_HUMAN sp|P03886|NU1M\_HUMAN sp|Q99743|NPAS2\_HUMAN sp|Q3SYG4|PTHB1\_HUMAN tr|A0A087WXJ7|A0A087WXJ7\_HUMAN sp|Q99835|SMO\_HUMAN sp|Q6P2S7|GNN\_HUMAN sp|O15034|RIMB2\_HUMAN sp|O75398|DEAF1\_HUMAN tr|H7C5W6|H7C5W6\_HUMAN tr|B4DUR8|B4DUR8\_HUMAN tr|J3KRL7|J3KRL7\_HUMAN sp|O15534|PER1\_HUMAN tr|F8VR36|F8VR36\_HUMAN sp|Q9P2D6|F135A\_HUMAN sp|P48764|SL9A3\_HUMAN tr|H0Y8C6|H0Y8C6\_HUMAN sp|Q8IZS8|CA2D3\_HUMAN sp|O00410|IPO5\_HUMAN sp|Q9UDT6|CLIP2\_HUMAN sp|Q13509|TBB3\_HUMAN sp|Q6ZRK6|CCD73\_HUMAN sp|Q9H0R5|GBP3\_HUMAN sp|Q9Y2U5|M3K2\_HUMAN tr|E9PBG4|E9PBG4\_HUMAN tr|E7EW95|E7EW95\_HUMAN tr|G3V484|G3V484\_HUMAN tr|G3V2Z2|G3V2Z2\_HUMAN sp|Q9P2E7|PCD10\_HUMAN sp|Q12789|TF3C1\_HUMAN tr|F8VQP2|F8VQP2\_HUMAN tr|C9K0P9|C9K0P9\_HUMAN sp|P20020|AT2B1\_HUMAN tr|E9PCN4|E9PCN4\_HUMAN tr|A0A087WW65|A0A087WW65\_HUMAN sp|Q8ND23|LR16B\_HUMAN tr|A0A075B754|A0A075B754\_HUMAN tr|E7EPJ2|E7EPJ2\_HUMAN tr|E7EQA0|E7EQA0\_HUMAN sp|Q5T742|CJ025\_HUMAN tr|Q5TAH7|Q5TAH7\_HUMAN sp|Q9BYN7|ZN341\_HUMAN tr|A6H900|A6H900\_HUMAN sp|Q76LX8|ATS13\_HUMAN sp|P20702|ITAX\_HUMAN tr|H3BN02|H3BN02\_HUMAN tr|M0QZR4|M0QZR4\_HUMAN sp|Q8TF71|MOT10\_HUMAN sp|O00268|TAF4\_HUMAN sp|P51532|SMCA4\_HUMAN sp|Q9NYU2|UGGG1\_HUMAN tr|H3BLT5|H3BLT5\_HUMAN sp|Q9BXW6|OSBL1\_HUMAN tr|A0A087WW20|A0A087WW20\_HUMAN sp|Q9C0B9|ZCHC2\_HUMAN sp|Q9ULD8|KCNH3\_HUMAN tr|K7ESN2|K7ESN2\_HUMAN sp|Q8TEV9|SMCR8\_HUMAN tr|I3L1H1|I3L1H1\_HUMAN tr|I3L124|I3L124\_HUMAN sp|Q15269|PWP2\_HUMAN sp|Q5T848|GP158\_HUMAN sp|Q496J9|SV2C\_HUMAN tr|B3KT41|B3KT41\_HUMAN sp|Q9UQV4|LAMP3\_HUMAN tr|E7ETP9|E7ETP9\_HUMAN sp|Q9Y6X6|MYO16\_HUMAN tr|F8W883|F8W883\_HUMAN sp|Q9P2R6|RERE\_HUMAN tr|B1AKN3|B1AKN3\_HUMAN tr|E9PAX0|E9PAX0\_HUMAN tr|B3KRP1|B3KRP1\_HUMAN sp|P23378|GCSP\_HUMAN sp|Q3BBV2|NBPF8\_HUMAN sp|P48681|NEST\_HUMAN tr|S4R3H3|S4R3H3\_HUMAN sp|Q13873|BMPR2\_HUMAN sp|Q8IX90|SKA3\_HUMAN tr|E7ENL6|E7ENL6\_HUMAN sp|Q8NDX5|PHC3\_HUMAN sp|Q5JTH9|RRP12\_HUMAN sp|Q6IQ32|ADNP2\_HUMAN tr|E7EX82|E7EX82\_HUMAN tr|H7C0T4|H7C0T4\_HUMAN sp|Q92667|AKAP1\_HUMAN tr|F8W6X9|F8W6X9\_HUMAN sp|Q86UU1|PHLB1\_HUMAN sp|Q9HCM2|PLXA4\_HUMAN tr|H0YDJ4|H0YDJ4\_HUMAN tr|H9KV85|H9KV85\_HUMAN tr|H0Y7Z1|H0Y7Z1\_HUMAN tr|F6WR09|F6WR09\_HUMAN tr|F5GWV3|F5GWV3\_HUMAN sp|P49321|NASP\_HUMAN sp|Q86YF9|DZIP1\_HUMAN sp|Q5T3U5|MRP7\_HUMAN tr|H7BXX9|H7BXX9\_HUMAN sp|O95342|ABCBB\_HUMAN tr|F5H1D6|F5H1D6\_HUMAN sp|Q07864|DPOE1\_HUMAN sp|Q9UNW8|GP132\_HUMAN sp|Q9GZY0|NXF2\_HUMAN sp|Q7Z3T8|ZFY16\_HUMAN sp|Q5T7B8|KIF24\_HUMAN sp|A6NNM3|RIM3B\_HUMAN sp|A6NJZ7|RIM3C\_HUMAN tr|J3KP16|J3KP16\_HUMAN sp|P55283|CADH4\_HUMAN sp|Q8WXE0|CSKI2\_HUMAN sp|Q9H8V3|ECT2\_HUMAN sp|Q92508|PIEZ1\_HUMAN sp|Q76M96|CCD80\_HUMAN sp|Q59H18|TNI3K\_HUMAN tr|E9PL24|E9PL24\_HUMAN sp|Q5SW79|CE170\_HUMAN tr|V9GXZ4|V9GXZ4\_HUMAN tr|H0YH15|H0YH15\_HUMAN sp|Q86X10|RLGPB\_HUMAN tr|A2A2F0|A2A2F0\_HUMAN tr|F8VZ81|F8VZ81\_HUMAN sp|P13942|COBA2\_HUMAN sp|Q5VUA4|ZN318\_HUMAN sp|Q6ZRR7|LRRC9\_HUMAN tr|F5GXF9|F5GXF9\_HUMAN tr|H0Y5Q9|H0Y5Q9\_HUMAN sp|Q69YH5|CDCA2\_HUMAN tr|A0A087WVI8|A0A087WVI8\_HUMAN tr|G3V4V5|G3V4V5\_HUMAN sp|Q6ZTY8|CL063\_HUMAN tr|F8VVT9|F8VVT9\_HUMAN tr|H3BUU9|H3BUU9\_HUMAN tr|J3KNM6|J3KNM6\_HUMAN sp|Q99490|AGAP2\_HUMAN sp|P55287|CAD11\_HUMAN sp|Q14CN2|CLCA4\_HUMAN tr|B1ALD9|B1ALD9\_HUMAN sp|Q15063|POSTN\_HUMAN tr|B8ZZI4|B8ZZI4\_HUMAN tr|H0YJV3|H0YJV3\_HUMAN tr|H0Y3R4|H0Y3R4\_HUMAN sp|Q6F5E8|LR16C\_HUMAN tr|H0YBU6|H0YBU6\_HUMAN tr|E5RGM0|E5RGM0\_HUMAN sp|Q9NP73|ALG13\_HUMAN sp|Q9H0H0|INT2\_HUMAN tr|J3KMZ7|J3KMZ7\_HUMAN sp|Q8WUA4|TF3C2\_HUMAN sp|Q9ULL8|SHRM4\_HUMAN tr|B1AJU4|B1AJU4\_HUMAN sp|O14896|IRF6\_HUMAN tr|Q96HN1|Q96HN1\_HUMAN sp|Q9NYL9|TMOD3\_HUMAN sp|P98168|ZXDA\_HUMAN sp|Q8IUR7|ARMC8\_HUMAN sp|Q8N3X1|FNBP4\_HUMAN tr|E9PK61|E9PK61\_HUMAN tr|E7EN95|E7EN95\_HUMAN sp|Q9UHC9|NPCL1\_HUMAN sp|Q15111|PLCL1\_HUMAN sp|Q96BI1|S22AI\_HUMAN tr|E9PRM7|E9PRM7\_HUMAN sp|P18084|ITB5\_HUMAN sp|Q8HWS3|RFX6\_HUMAN sp|Q7Z7M9|GALT5\_HUMAN sp|P11215|ITAM\_HUMAN sp|O75096|LRP4\_HUMAN sp|Q6AI14|SL9A4\_HUMAN sp|O00555|CAC1A\_HUMAN sp|Q7RTS7|K2C74\_HUMAN tr|F8W1S1|F8W1S1\_HUMAN Q7RTS7 sp|Q9P227|RHG23\_HUMAN sp|Q8TET4|GANC\_HUMAN sp|Q69YQ0|CYTSA\_HUMAN sp|Q96KN3|PKNX2\_HUMAN tr|G8JLG2|G8JLG2\_HUMAN sp|Q15517|CDSN\_HUMAN tr|E9PEI0|E9PEI0\_HUMAN sp|Q9BXU7|UBP26\_HUMAN tr|S4R381|S4R381\_HUMAN sp|Q9BQS8|FYCO1\_HUMAN sp|P40692|MLH1\_HUMAN tr|E7EWK3|E7EWK3\_HUMAN tr|A0A075B6Q7|A0A075B6Q7\_HUMAN tr|A0A087WYX6|A0A087WYX6\_HUMAN sp|Q9NRI5|DISC1\_HUMAN tr|Q6AWB1|Q6AWB1\_HUMAN tr|A0A087WW47|A0A087WW47\_HUMAN tr|A0A075B757|A0A075B757\_HUMAN tr|A0A087WVU4|A0A087WVU4\_HUMAN tr|A0A087WZJ2|A0A087WZJ2\_HUMAN tr|A0A075B762|A0A075B762\_HUMAN sp|P78381|S35A2\_HUMAN sp|Q9H2P0|ADNP\_HUMAN sp|O75448|MED24\_HUMAN tr|F5H4P8|F5H4P8\_HUMAN tr|E9PMT0|E9PMT0\_HUMAN tr|F5H2S8|F5H2S8\_HUMAN sp|Q9Y2R2|PTN22\_HUMAN sp|P26640|SYVC\_HUMAN sp|Q01780|EXOSX\_HUMAN sp|Q99489|OXDD\_HUMAN sp|Q13018|PLA2R\_HUMAN sp|P30414|NKTR\_HUMAN tr|G5EA31|G5EA31\_HUMAN sp|P53992|SC24C\_HUMAN sp|Q7Z7A4|PXK\_HUMAN tr|H7BYG4|H7BYG4\_HUMAN sp|Q6P5Z2|PKN3\_HUMAN sp|Q8TBY9|WDR66\_HUMAN sp|Q8N201|INT1\_HUMAN tr|H3BSK8|H3BSK8\_HUMAN sp|H7BZ55|CROL3\_HUMAN tr|R4GN92|R4GN92\_HUMAN tr|B1AQM6|B1AQM6\_HUMAN sp|Q99550|MPP9\_HUMAN sp|Q9Y2X9|ZN281\_HUMAN tr|H0Y6F2|H0Y6F2\_HUMAN tr|E9PDD2|E9PDD2\_HUMAN sp|Q02413|DSG1\_HUMAN sp|Q9NQW1|SC31B\_HUMAN sp|O94911|ABCA8\_HUMAN tr|Q6NX52|Q6NX52\_HUMAN sp|Q9UPM8|AP4E1\_HUMAN tr|K7EP51|K7EP51\_HUMAN sp|Q8TE96|DQX1\_HUMAN sp|Q6XE24|RBMS3\_HUMAN sp|Q9P2F6|RHG20\_HUMAN tr|C9JIJ9|C9JIJ9\_HUMAN tr|F8W6W8|F8W6W8\_HUMAN sp|Q9UMR3|TBX20\_HUMAN tr|H0YL38|H0YL38\_HUMAN sp|O95197|RTN3\_HUMAN sp|Q86UV5|UBP48\_HUMAN sp|Q9NYZ3|GTSE1\_HUMAN sp|Q7RTX1|TS1R1\_HUMAN sp|Q8IXJ9|ASXL1\_HUMAN tr|M0QZW5|M0QZW5\_HUMAN tr|X6RM00|X6RM00\_HUMAN sp|Q96JX3|SRAC1\_HUMAN tr|H3BTX9|H3BTX9\_HUMAN sp|Q68CK6|ACS2B\_HUMAN tr|D3DPC4|D3DPC4\_HUMAN tr|H3BLX4|H3BLX4\_HUMAN tr|H0YAU5|H0YAU5\_HUMAN sp|Q53R41|FAKD1\_HUMAN sp|Q6ZP82|CC141\_HUMAN sp|O14994|SYN3\_HUMAN sp|Q9Y2G3|AT11B\_HUMAN sp|P56282|DPOE2\_HUMAN tr|J3KPH8|J3KPH8\_HUMAN tr|H7C1D9|H7C1D9\_HUMAN sp|Q8NF50|DOCK8\_HUMAN sp|Q5VU97|CAHD1\_HUMAN sp|O75891|AL1L1\_HUMAN tr|F5H5K2|F5H5K2\_HUMAN sp|Q86YN6|PRGC2\_HUMAN tr|A0A096LPD8|A0A096LPD8\_HUMAN sp|O14607|UTY\_HUMAN tr|F5H3N7|F5H3N7\_HUMAN tr|A0A087X2I9|A0A087X2I9\_HUMAN sp|Q96Q04|LMTK3\_HUMAN tr|Q5JTU8|Q5JTU8\_HUMAN sp|Q01581|HMCS1\_HUMAN sp|Q4ADV7|RIC1\_HUMAN tr|H0YFN7|H0YFN7\_HUMAN tr|A0A087X043|A0A087X043\_HUMAN sp|O43424|GRID2\_HUMAN tr|R4GMM8|R4GMM8\_HUMAN tr|C9JD84|C9JD84\_HUMAN tr|E7EV71|E7EV71\_HUMAN tr|E7ENB6|E7ENB6\_HUMAN tr|B7ZAG0|B7ZAG0\_HUMAN sp|Q8NFW1|COMA1\_HUMAN sp|Q96RT1|LAP2\_HUMAN sp|Q9H6R0|DHX33\_HUMAN sp|Q96LZ3|CANB2\_HUMAN tr|F5GZG9|F5GZG9\_HUMAN sp|Q07617|SPAG1\_HUMAN tr|K7EPA3|K7EPA3\_HUMAN sp|Q9NX61|T161A\_HUMAN sp|P38935|SMBP2\_HUMAN sp|O00330|ODPX\_HUMAN sp|P30291|WEE1\_HUMAN sp|Q68DX3|FRPD2\_HUMAN sp|A6NFN3|RFOX3\_HUMAN sp|P04844|RPN2\_HUMAN sp|A3KMH1|VWA8\_HUMAN sp|O15056|SYNJ2\_HUMAN tr|F5H658|F5H658\_HUMAN sp|Q14562|DHX8\_HUMAN tr|E9PB13|E9PB13\_HUMAN sp|Q6VAB6|KSR2\_HUMAN tr|K7ERY3|K7ERY3\_HUMAN tr|J3KT14|J3KT14\_HUMAN sp|Q6RI45|BRWD3\_HUMAN sp|Q9P1A2|PP4RL\_HUMAN tr|F6U0I4|F6U0I4\_HUMAN sp|Q15031|SYLM\_HUMAN sp|O75366|AVIL\_HUMAN sp|Q9C0K7|STRAB\_HUMAN tr|H7BXU9|H7BXU9\_HUMAN sp|Q14566|MCM6\_HUMAN sp|P52732|KIF11\_HUMAN sp|Q8NCM2|KCNH5\_HUMAN sp|Q8WVS4|WDR60\_HUMAN sp|Q6P499|NPAL3\_HUMAN sp|Q9NZL6|RGL1\_HUMAN sp|P16591|FER\_HUMAN tr|A8MX39|A8MX39\_HUMAN tr|A8MZ24|A8MZ24\_HUMAN sp|P11308|ERG\_HUMAN tr|F8WB06|F8WB06\_HUMAN sp|Q05BV3|EMAL5\_HUMAN tr|E7ET52|E7ET52\_HUMAN sp|Q8WW38|FOG2\_HUMAN tr|A0A087WV76|A0A087WV76\_HUMAN sp|Q8TDV2|GP148\_HUMAN sp|Q14676|MDC1\_HUMAN sp|A6NCI4|VWA3A\_HUMAN tr|G3V1Q5|G3V1Q5\_HUMAN tr|H0YJN0|H0YJN0\_HUMAN sp|Q5RHP9|ERIC3\_HUMAN sp|Q9P265|DIP2B\_HUMAN sp|P32418|NAC1\_HUMAN tr|H0YHA8|H0YHA8\_HUMAN sp|Q07092|COGA1\_HUMAN tr|K7EM54|K7EM54\_HUMAN sp|P22897|MRC1\_HUMAN sp|Q9Y4C8|RBM19\_HUMAN tr|F8VWZ8|F8VWZ8\_HUMAN sp|P53634|CATC\_HUMAN tr|H3BMH0|H3BMH0\_HUMAN sp|Q9H1H9|KI13A\_HUMAN sp|Q8N660|NBPFF\_HUMAN sp|Q13426|XRCC4\_HUMAN sp|Q9H252|KCNH6\_HUMAN tr|J9JID4|J9JID4\_HUMAN sp|Q9UQL6|HDAC5\_HUMAN sp|Q8WX93|PALLD\_HUMAN sp|O60602|TLR5\_HUMAN tr|K4DI95|K4DI95\_HUMAN sp|O60879|DIAP2\_HUMAN sp|Q9BXT6|M10L1\_HUMAN sp|Q16647|PTGIS\_HUMAN sp|Q9Y6I3|EPN1\_HUMAN sp|P48200|IREB2\_HUMAN sp|Q5U5Z8|CBPC2\_HUMAN tr|C9JCM7|C9JCM7\_HUMAN tr|H9KV53|H9KV53\_HUMAN tr|B5MCJ9|B5MCJ9\_HUMAN tr|H0YBY1|H0YBY1\_HUMAN sp|Q96N21|AP4AT\_HUMAN sp|Q92908|GATA6\_HUMAN sp|Q9UKX3|MYH13\_HUMAN sp|O94819|KBTBB\_HUMAN tr|B7ZBD4|B7ZBD4\_HUMAN sp|Q9UBK7|RBL2A\_HUMAN tr|E7EWW3|E7EWW3\_HUMAN tr|B7ZBD5|B7ZBD5\_HUMAN sp|Q8IYT1|FA71A\_HUMAN sp|Q9NV12|TM140\_HUMAN tr|B1AM27|B1AM27\_HUMAN tr|A0A087WV83|A0A087WV83\_HUMAN sp|Q8IZK6|MCLN2\_HUMAN sp|Q5VVW2|GARL3\_HUMAN sp|Q9UPQ9|TNR6B\_HUMAN sp|P48552|NRIP1\_HUMAN sp|Q9UHD8|SEPT9\_HUMAN sp|P22307|NLTP\_HUMAN sp|Q13469|NFAC2\_HUMAN tr|B5MCI0|B5MCI0\_HUMAN sp|Q9H7D7|WDR26\_HUMAN sp|Q14134|TRI29\_HUMAN tr|Q5JXR6|Q5JXR6\_HUMAN sp|Q6PI48|SYDM\_HUMAN sp|Q8IY63|AMOL1\_HUMAN sp|Q68CQ4|DIEXF\_HUMAN sp|Q2Y0W8|S4A8\_HUMAN tr|F5GWX5|F5GWX5\_HUMAN sp|Q14839|CHD4\_HUMAN sp|O95340|PAPS2\_HUMAN sp|Q5JPB2|ZN831\_HUMAN tr|Q5VYG5|Q5VYG5\_HUMAN tr|H0Y8V2|H0Y8V2\_HUMAN tr|F6M2K4|F6M2K4\_HUMAN tr|F6M2K2|F6M2K2\_HUMAN tr|H0YNJ6|H0YNJ6\_HUMAN ENSEMBL:ENSBTAP00000001528 sp|Q86TC9|MYPN\_HUMAN sp|Q53GD3|CTL4\_HUMAN tr|J3KN10|J3KN10\_HUMAN tr|J3KNE4|J3KNE4\_HUMAN sp|O14641|DVL2\_HUMAN tr|A0A096LP56|A0A096LP56\_HUMAN sp|Q9BUV0|RSRP1\_HUMAN tr|B7Z637|B7Z637\_HUMAN tr|M0QXN5|M0QXN5\_HUMAN sp|Q3V6T2|GRDN\_HUMAN tr|H0YNH8|H0YNH8\_HUMAN sp|Q9BZF9|UACA\_HUMAN tr|C9JCY1|C9JCY1\_HUMAN tr|C9JPK3|C9JPK3\_HUMAN tr|C9K0E4|C9K0E4\_HUMAN sp|Q7Z7A1|CNTRL\_HUMAN sp|O60500|NPHN\_HUMAN sp|Q9NYI0|PSD3\_HUMAN tr|E5RJ29|E5RJ29\_HUMAN sp|Q93073|SBP2L\_HUMAN sp|Q7Z2Z2|ETUD1\_HUMAN tr|E9PE96|E9PE96\_HUMAN tr|J3QSD7|J3QSD7\_HUMAN tr|J3KQ40|J3KQ40\_HUMAN sp|O95759|TBCD8\_HUMAN sp|P20585|MSH3\_HUMAN sp|Q13283|G3BP1\_HUMAN sp|Q5H8C1|FREM1\_HUMAN sp|Q9H7T0|CTSRB\_HUMAN sp|P37275|ZEB1\_HUMAN P01044-1 P01045-1 tr|F5H1F7|F5H1F7\_HUMAN sp|Q8IZY2|ABCA7\_HUMAN tr|C9IZQ6|C9IZQ6\_HUMAN sp|Q9Y6X8|ZHX2\_HUMAN tr|Q5VVD7|Q5VVD7\_HUMAN sp|Q8IWE5|PKHM2\_HUMAN sp|Q6ZQT7|YJ013\_HUMAN sp|Q76NI1|VKIND\_HUMAN sp|Q8IZ21|PHAR4\_HUMAN tr|H0YGQ3|H0YGQ3\_HUMAN tr|B7ZB24|B7ZB24\_HUMAN sp|Q969Y2|GTPB3\_HUMAN tr|C9IZY8|C9IZY8\_HUMAN sp|Q5FWF4|ZRAB3\_HUMAN sp|Q14397|GCKR\_HUMAN sp|Q86V48|LUZP1\_HUMAN sp|P0CG12|CTF8A\_HUMAN tr|F8WE45|F8WE45\_HUMAN sp|Q92738|US6NL\_HUMAN tr|H0YJL1|H0YJL1\_HUMAN tr|X6RAB3|X6RAB3\_HUMAN tr|A9UF01|A9UF01\_HUMAN sp|P33176|KINH\_HUMAN sp|O60391|NMD3B\_HUMAN sp|Q6ZU69|F205A\_HUMAN sp|Q6XZF7|DNMBP\_HUMAN sp|P29536|LMOD1\_HUMAN tr|F8WE49|F8WE49\_HUMAN sp|Q2M3G0|ABCB5\_HUMAN sp|Q9HBB8|CDHR5\_HUMAN tr|H0YKU7|H0YKU7\_HUMAN sp|O94759|TRPM2\_HUMAN sp|Q08462|ADCY2\_HUMAN sp|Q6NWY9|PR40B\_HUMAN sp|Q99607|ELF4\_HUMAN sp|A8K8V0|ZN785\_HUMAN sp|Q99613|EIF3C\_HUMAN sp|B5ME19|EIFCL\_HUMAN sp|P54296|MYOM2\_HUMAN sp|Q92888|ARHG1\_HUMAN sp|Q08J23|NSUN2\_HUMAN tr|H7C0G8|H7C0G8\_HUMAN tr|H7C582|H7C582\_HUMAN tr|F8VQE1|F8VQE1\_HUMAN tr|D6R9D5|D6R9D5\_HUMAN tr|D6RBD5|D6RBD5\_HUMAN sp|O14841|OPLA\_HUMAN sp|Q8IX18|DHX40\_HUMAN sp|Q6MZW2|FSTL4\_HUMAN tr|F8W6J4|F8W6J4\_HUMAN tr|F6Y5H0|F6Y5H0\_HUMAN tr|E7ETU5|E7ETU5\_HUMAN sp|Q8N655|CJ012\_HUMAN tr|Q5JS74|Q5JS74\_HUMAN sp|O14936|CSKP\_HUMAN tr|H0Y304|H0Y304\_HUMAN tr|E7ESB2|E7ESB2\_HUMAN tr|F8VX32|F8VX32\_HUMAN tr|E7ET48|E7ET48\_HUMAN sp|Q9ULD0|OGDHL\_HUMAN sp|P12955|PEPD\_HUMAN sp|Q96J92|WNK4\_HUMAN sp|Q6UXK5|LRRN1\_HUMAN tr|E7ESC9|E7ESC9\_HUMAN sp|Q96RY7|IF140\_HUMAN sp|Q99456|K1C12\_HUMAN Q99456 sp|Q68D51|DEN2C\_HUMAN tr|E9PEZ3|E9PEZ3\_HUMAN sp|O60610|DIAP1\_HUMAN tr|E9PHQ0|E9PHQ0\_HUMAN tr|E7EMV0|E7EMV0\_HUMAN sp|Q14934|NFAC4\_HUMAN sp|O60678|ANM3\_HUMAN sp|Q9ULD9|ZN608\_HUMAN sp|P46020|KPB1\_HUMAN tr|C9JNZ9|C9JNZ9\_HUMAN sp|Q9UPR5|NAC2\_HUMAN tr|B8ZZS4|B8ZZS4\_HUMAN sp|Q96RR4|KKCC2\_HUMAN tr|F5GZ00|F5GZ00\_HUMAN tr|H0Y9Y3|H0Y9Y3\_HUMAN tr|H7BZN3|H7BZN3\_HUMAN sp|Q9NRM1|ENAM\_HUMAN sp|Q8IY81|SPB1\_HUMAN sp|Q58FG1|HS904\_HUMAN sp|Q6P4Q7|CNNM4\_HUMAN tr|A0A087X256|A0A087X256\_HUMAN sp|Q86VS3|IQCH\_HUMAN sp|Q2M389|WASH7\_HUMAN sp|Q96EV2|RBM33\_HUMAN sp|Q9Y4B6|VPRBP\_HUMAN tr|G3V2R6|G3V2R6\_HUMAN tr|E9PHM2|E9PHM2\_HUMAN sp|O15399|NMDE4\_HUMAN tr|A0A087WX99|A0A087WX99\_HUMAN sp|Q6Q0C0|TRAF7\_HUMAN sp|Q96DT7|ZBT10\_HUMAN tr|A8MUN2|A8MUN2\_HUMAN sp|Q9BX95|SGPP1\_HUMAN sp|O75185|AT2C2\_HUMAN tr|F8W9E7|F8W9E7\_HUMAN sp|P35580|MYH10\_HUMAN sp|Q8NEB9|PK3C3\_HUMAN tr|E9PC85|E9PC85\_HUMAN sp|Q9Y2H2|SAC2\_HUMAN tr|C9JHW1|C9JHW1\_HUMAN sp|Q8TDM6|DLG5\_HUMAN sp|Q7L3V2|BOP\_HUMAN tr|Q5TIG5|Q5TIG5\_HUMAN tr|J3KN01|J3KN01\_HUMAN sp|O60476|MA1A2\_HUMAN tr|A8MQ02|A8MQ02\_HUMAN sp|P55196|AFAD\_HUMAN tr|H0YGQ4|H0YGQ4\_HUMAN sp|Q96CC6|RHDF1\_HUMAN sp|Q8TD31|CCHCR\_HUMAN sp|O60315|ZEB2\_HUMAN sp|Q8TE54|S26A7\_HUMAN sp|O43318|M3K7\_HUMAN sp|Q9H2T7|RBP17\_HUMAN tr|Q5T981|Q5T981\_HUMAN sp|Q5H9U9|DDX6L\_HUMAN tr|M0QZS0|M0QZS0\_HUMAN tr|A0A087WY84|A0A087WY84\_HUMAN sp|Q9NRL2|BAZ1A\_HUMAN tr|X6R9L0|X6R9L0\_HUMAN sp|Q13217|DNJC3\_HUMAN tr|F8VRG4|F8VRG4\_HUMAN tr|H0Y9H5|H0Y9H5\_HUMAN sp|Q9H334|FOXP1\_HUMAN sp|Q5T5Y3|CAMP1\_HUMAN tr|A0A087X299|A0A087X299\_HUMAN sp|Q8N1G0|ZN687\_HUMAN tr|A2A3D6|A2A3D6\_HUMAN tr|E7EVX6|E7EVX6\_HUMAN sp|P01024|CO3\_HUMAN sp|Q9Y289|SC5A6\_HUMAN sp|Q9BYJ4|TRI34\_HUMAN sp|Q9Y597|KCTD3\_HUMAN sp|O00220|TR10A\_HUMAN tr|K7EK72|K7EK72\_HUMAN sp|Q96CS2|HAUS1\_HUMAN tr|K7EKH4|K7EKH4\_HUMAN tr|K7EJA9|K7EJA9\_HUMAN sp|A8MVW0|F1712\_HUMAN tr|H3BLU0|H3BLU0\_HUMAN tr|F8W930|F8W930\_HUMAN sp|Q13164|MK07\_HUMAN sp|Q9Y6M1|IF2B2\_HUMAN tr|Q5T5P1|Q5T5P1\_HUMAN tr|G3V5Q9|G3V5Q9\_HUMAN sp|Q9Y5X1|SNX9\_HUMAN tr|A0A087WYU1|A0A087WYU1\_HUMAN sp|Q9C0D3|ZY11B\_HUMAN tr|F6S8M0|F6S8M0\_HUMAN sp|O14727|APAF\_HUMAN sp|O43823|AKAP8\_HUMAN tr|Q5JTC4|Q5JTC4\_HUMAN sp|Q9Y6N6|LAMC3\_HUMAN sp|Q9UQE7|SMC3\_HUMAN Q6ISB0 sp|Q9NSB2|KRT84\_HUMAN Q9NSB2 sp|P0C874|S31D3\_HUMAN sp|Q6ZUB0|S31D4\_HUMAN sp|A6NNC1|P12LL\_HUMAN sp|Q9Y210|TRPC6\_HUMAN tr|E9PJN4|E9PJN4\_HUMAN sp|Q01064|PDE1B\_HUMAN tr|B4DK72|B4DK72\_HUMAN tr|J3KNV4|J3KNV4\_HUMAN tr|G3V4R5|G3V4R5\_HUMAN tr|G3V411|G3V411\_HUMAN tr|Q5T2J8|Q5T2J8\_HUMAN sp|Q96IC2|REXON\_HUMAN tr|H7C175|H7C175\_HUMAN tr|K7EM17|K7EM17\_HUMAN sp|Q6ZMQ8|LMTK1\_HUMAN sp|Q2WGJ9|FR1L6\_HUMAN sp|Q9Y2H5|PKHA6\_HUMAN sp|Q7Z7B0|FLIP1\_HUMAN sp|Q9HCH0|NCK5L\_HUMAN sp|Q7Z570|Z804A\_HUMAN tr|A0A087X014|A0A087X014\_HUMAN sp|Q96GE4|CEP95\_HUMAN tr|B4DYV8|B4DYV8\_HUMAN sp|Q8WZ75|ROBO4\_HUMAN sp|Q9BRZ2|TRI56\_HUMAN sp|P09619|PGFRB\_HUMAN sp|Q99504|EYA3\_HUMAN tr|H0YEH2|H0YEH2\_HUMAN tr|Q08AM2|Q08AM2\_HUMAN tr|B4DUK8|B4DUK8\_HUMAN tr|E1P5H9|E1P5H9\_HUMAN sp|Q969U6|FBXW5\_HUMAN sp|Q9UG01|IF172\_HUMAN sp|O15084|ANR28\_HUMAN sp|Q99856|ARI3A\_HUMAN sp|Q6UXG2|K1324\_HUMAN tr|X6REB3|X6REB3\_HUMAN sp|O75157|T22D2\_HUMAN sp|P38919|IF4A3\_HUMAN tr|K7EKI8|K7EKI8\_HUMAN tr|K7EQ71|K7EQ71\_HUMAN sp|O60437|PEPL\_HUMAN sp|Q9NRR4|RNC\_HUMAN sp|Q86UP2|KTN1\_HUMAN tr|E5RHP1|E5RHP1\_HUMAN sp|A8MYU2|KCNU1\_HUMAN sp|P40939|ECHA\_HUMAN sp|Q9HC58|NCKX3\_HUMAN tr|K7EIP4|K7EIP4\_HUMAN tr|H0YB14|H0YB14\_HUMAN sp|Q96T49|PP16B\_HUMAN sp|Q8TEP8|CE192\_HUMAN tr|K7ENP4|K7ENP4\_HUMAN tr|A0A075B6G5|A0A075B6G5\_HUMAN sp|Q5TI25|NBPFE\_HUMAN tr|A0A075B761|A0A075B761\_HUMAN tr|H7BY70|H7BY70\_HUMAN sp|B4DH59|NBPFP\_HUMAN tr|A0A087WZE1|A0A087WZE1\_HUMAN tr|A0A087WYQ9|A0A087WYQ9\_HUMAN sp|Q6P3W6|NBPFA\_HUMAN tr|A0A087WZB6|A0A087WZB6\_HUMAN sp|Q5SXJ2|NBPFG\_HUMAN tr|A0A087WUL8|A0A087WUL8\_HUMAN tr|A0A087WWQ1|A0A087WWQ1\_HUMAN tr|A0A075B7F9|A0A075B7F9\_HUMAN tr|A0A087WTW4|A0A087WTW4\_HUMAN tr|A0A087WY62|A0A087WY62\_HUMAN tr|A0A075B7A3|A0A075B7A3\_HUMAN tr|A0A087WY26|A0A087WY26\_HUMAN sp|Q9UHC1|MLH3\_HUMAN tr|E9PCX8|E9PCX8\_HUMAN sp|O94979|SC31A\_HUMAN tr|Q4VXY6|Q4VXY6\_HUMAN tr|H0YIS1|H0YIS1\_HUMAN tr|H0YCE9|H0YCE9\_HUMAN sp|A6NGW2|STRCL\_HUMAN sp|Q6ZS72|CS035\_HUMAN sp|Q92796|DLG3\_HUMAN tr|Q5JUW8|Q5JUW8\_HUMAN sp|P35916|VGFR3\_HUMAN tr|E9PD35|E9PD35\_HUMAN sp|Q66GS9|CP135\_HUMAN sp|Q5SZL2|CE85L\_HUMAN sp|Q13822|ENPP2\_HUMAN tr|E7EUF1|E7EUF1\_HUMAN tr|C9J8W4|C9J8W4\_HUMAN tr|E9PBQ4|E9PBQ4\_HUMAN tr|F8W6I3|F8W6I3\_HUMAN sp|Q9HDB5|NRX3B\_HUMAN sp|Q96JH7|VCIP1\_HUMAN sp|P09104|ENOG\_HUMAN sp|Q9Y5A9|YTHD2\_HUMAN tr|A0A087WXZ3|A0A087WXZ3\_HUMAN tr|A0A087WWY9|A0A087WWY9\_HUMAN sp|Q92614|MY18A\_HUMAN tr|Q5VWU8|Q5VWU8\_HUMAN sp|Q3MIS6|ZN528\_HUMAN tr|K7EJ04|K7EJ04\_HUMAN sp|P50443|S26A2\_HUMAN sp|P39687|AN32A\_HUMAN tr|H0YN26|H0YN26\_HUMAN sp|Q3BBV1|NBPFK\_HUMAN sp|Q9NZP6|NPAP1\_HUMAN tr|J3KR05|J3KR05\_HUMAN sp|Q9UPU3|SORC3\_HUMAN tr|J3QRH7|J3QRH7\_HUMAN tr|J3KTN9|J3KTN9\_HUMAN sp|Q9HC10|OTOF\_HUMAN sp|Q5SSG8|MUC21\_HUMAN tr|H0Y966|H0Y966\_HUMAN tr|J3KN34|J3KN34\_HUMAN sp|O95870|ABHGA\_HUMAN sp|O60299|LZTS3\_HUMAN tr|Q86YQ0|Q86YQ0\_HUMAN tr|X6RGP5|X6RGP5\_HUMAN sp|Q0VF96|CGNL1\_HUMAN sp|Q9H8M5|CNNM2\_HUMAN sp|Q8WYP5|ELYS\_HUMAN sp|Q96R06|SPAG5\_HUMAN tr|H0YAY3|H0YAY3\_HUMAN tr|E9PF32|E9PF32\_HUMAN tr|Q5JPC9|Q5JPC9\_HUMAN sp|P11388|TOP2A\_HUMAN sp|Q9UL03|INT6\_HUMAN sp|Q06190|P2R3A\_HUMAN tr|B4DDR8|B4DDR8\_HUMAN sp|P14651|HXB3\_HUMAN sp|O14917|PCD17\_HUMAN sp|Q05193|DYN1\_HUMAN tr|F6WH68|F6WH68\_HUMAN sp|Q14D04|MELT\_HUMAN sp|P26006|ITA3\_HUMAN sp|Q8WWY6|MB3L1\_HUMAN sp|A6NNM8|TTL13\_HUMAN sp|Q96MI9|CBPC4\_HUMAN tr|J3KQF5|J3KQF5\_HUMAN sp|Q9Y6R4|M3K4\_HUMAN sp|O14594|NCAN\_HUMAN sp|A6NES4|MRO2A\_HUMAN sp|Q66K14|TBC9B\_HUMAN tr|B4DHA8|B4DHA8\_HUMAN sp|O15015|ZN646\_HUMAN tr|H0YF06|H0YF06\_HUMAN sp|Q9GZT6|CC90B\_HUMAN tr|J3KPI3|J3KPI3\_HUMAN tr|H3BQN7|H3BQN7\_HUMAN sp|Q13797|ITA9\_HUMAN tr|A0A087WVC1|A0A087WVC1\_HUMAN sp|Q6Q4G3|AMPQ\_HUMAN sp|P31327|CPSM\_HUMAN sp|Q96PY6|NEK1\_HUMAN tr|M0R150|M0R150\_HUMAN sp|P26045|PTN3\_HUMAN tr|S4R3U7|S4R3U7\_HUMAN sp|Q8ND71|GIMA8\_HUMAN tr|E7ETA5|E7ETA5\_HUMAN sp|O43592|XPOT\_HUMAN sp|P42768|WASP\_HUMAN tr|B7ZM99|B7ZM99\_HUMAN tr|A0A087WVM4|A0A087WVM4\_HUMAN sp|Q13950|RUNX2\_HUMAN tr|A0A075B7F8|A0A075B7F8\_HUMAN sp|Q9Y5L2|HLPDA\_HUMAN tr|A0A087X056|A0A087X056\_HUMAN tr|H7C1G6|H7C1G6\_HUMAN sp|O60503|ADCY9\_HUMAN tr|E9PQP7|E9PQP7\_HUMAN sp|P54792|DVLP1\_HUMAN sp|Q01543|FLI1\_HUMAN sp|O60330|PCDGC\_HUMAN tr|B3KXX3|B3KXX3\_HUMAN sp|Q9Y6J9|TAF6L\_HUMAN sp|Q9HCL0|PCD18\_HUMAN sp|O60733|PLPL9\_HUMAN tr|A0A087X079|A0A087X079\_HUMAN sp|O14863|ZNT4\_HUMAN sp|O75132|ZBED4\_HUMAN sp|Q96PC5|MIA2\_HUMAN tr|F5H5M1|F5H5M1\_HUMAN sp|Q9BZK3|NACP1\_HUMAN tr|H3BT29|H3BT29\_HUMAN sp|O60941|DTNB\_HUMAN tr|A0A087X1H8|A0A087X1H8\_HUMAN tr|E5RGL8|E5RGL8\_HUMAN sp|A2VEC9|SSPO\_HUMAN tr|A0A096LNW2|A0A096LNW2\_HUMAN sp|Q5RGN0|NBPFN\_HUMAN tr|A0A087WTH4|A0A087WTH4\_HUMAN sp|Q86T75|NBPFB\_HUMAN tr|A0A087WVG8|A0A087WVG8\_HUMAN sp|Q9Y623|MYH4\_HUMAN sp|P20591|MX1\_HUMAN sp|P0CJ78|ZN865\_HUMAN sp|Q32M45|ANO4\_HUMAN sp|Q8IVH8|M4K3\_HUMAN tr|F5H5A3|F5H5A3\_HUMAN tr|F5H7S1|F5H7S1\_HUMAN sp|Q2M1K9|ZN423\_HUMAN sp|Q5T6F2|UBAP2\_HUMAN sp|P11055|MYH3\_HUMAN sp|P27815|PDE4A\_HUMAN sp|Q9UF83|YM012\_HUMAN sp|Q9Y3Q4|HCN4\_HUMAN tr|H0Y785|H0Y785\_HUMAN sp|Q92626|PXDN\_HUMAN sp|O95625|ZBT11\_HUMAN tr|E7ESA7|E7ESA7\_HUMAN sp|P29474|NOS3\_HUMAN sp|O15259|NPHP1\_HUMAN sp|Q92523|CPT1B\_HUMAN sp|P41594|GRM5\_HUMAN tr|J3KQG3|J3KQG3\_HUMAN sp|Q5JZY3|EPHAA\_HUMAN tr|E9PE63|E9PE63\_HUMAN sp|Q86UD5|SL9B2\_HUMAN sp|Q13608|PEX6\_HUMAN tr|E9PRF4|E9PRF4\_HUMAN sp|Q7Z403|TMC6\_HUMAN tr|A6NCB9|A6NCB9\_HUMAN sp|Q99502|EYA1\_HUMAN tr|F8WB53|F8WB53\_HUMAN tr|A0A087X1S9|A0A087X1S9\_HUMAN sp|Q9P1W3|CSC1\_HUMAN tr|J3KPC8|J3KPC8\_HUMAN sp|Q9Y2K2|SIK3\_HUMAN tr|B7Z1T1|B7Z1T1\_HUMAN tr|H7BYE5|H7BYE5\_HUMAN sp|O00258|WRB\_HUMAN tr|K7EM46|K7EM46\_HUMAN sp|Q9Y6D9|MD1L1\_HUMAN sp|Q96DI7|SNR40\_HUMAN sp|Q6DT37|MRCKG\_HUMAN sp|O94804|STK10\_HUMAN tr|G5EA42|G5EA42\_HUMAN sp|Q9NZR1|TMOD2\_HUMAN sp|Q13480|GAB1\_HUMAN tr|E9PN67|E9PN67\_HUMAN sp|Q86T65|DAAM2\_HUMAN sp|Q92954|PRG4\_HUMAN tr|U3KQ43|U3KQ43\_HUMAN tr|B9TWZ6|B9TWZ6\_HUMAN tr|K7EKS6|K7EKS6\_HUMAN sp|Q8WWK9|CKAP2\_HUMAN tr|H0YG18|H0YG18\_HUMAN sp|Q9BZA7|PC11X\_HUMAN sp|Q9NQW5|PRDM7\_HUMAN sp|Q96RV3|PCX1\_HUMAN tr|H0YBS0|H0YBS0\_HUMAN tr|E9PQ91|E9PQ91\_HUMAN sp|O75845|SC5D\_HUMAN tr|E9PPW5|E9PPW5\_HUMAN sp|P81133|SIM1\_HUMAN tr|H7C2S2|H7C2S2\_HUMAN sp|Q9UII4|HERC5\_HUMAN sp|O43868|S28A2\_HUMAN sp|Q9NTX7|RN146\_HUMAN sp|Q7Z3S7|CA2D4\_HUMAN tr|K7EJY1|K7EJY1\_HUMAN tr|E9PJC5|E9PJC5\_HUMAN sp|Q6ZXV5|TMTC3\_HUMAN sp|P62195|PRS8\_HUMAN sp|P35712|SOX6\_HUMAN sp|P46940|IQGA1\_HUMAN tr|H7C1P6|H7C1P6\_HUMAN sp|Q9NS15|LTBP3\_HUMAN tr|J3KR24|J3KR24\_HUMAN sp|Q9P2G1|AKIB1\_HUMAN sp|Q9BX69|CARD6\_HUMAN tr|H0Y882|H0Y882\_HUMAN tr|E9PFD3|E9PFD3\_HUMAN tr|X6RE05|X6RE05\_HUMAN tr|F5H0P5|F5H0P5\_HUMAN tr|F5H1Q2|F5H1Q2\_HUMAN sp|Q8NBI2|CYAC3\_HUMAN tr|F5H5L0|F5H5L0\_HUMAN sp|Q9BYB0|SHAN3\_HUMAN sp|Q8WWW8|GAB3\_HUMAN tr|H0YKJ1|H0YKJ1\_HUMAN sp|Q96GQ5|RUS1\_HUMAN sp|Q9BU64|CENPO\_HUMAN tr|H7C4W1|H7C4W1\_HUMAN tr|U3KQC1|U3KQC1\_HUMAN sp|O76011|KRT34\_HUMAN sp|Q96NL6|SCLT1\_HUMAN Q8IUT8 sp|P12882|MYH1\_HUMAN sp|Q8N475|FSTL5\_HUMAN sp|Q8WUX1|S38A5\_HUMAN sp|Q96JP9|CDHR1\_HUMAN sp|Q6IPM2|IQCE\_HUMAN tr|A0A087WX45|A0A087WX45\_HUMAN tr|A0A087WV57|A0A087WV57\_HUMAN sp|P57721|PCBP3\_HUMAN tr|H3BPE7|H3BPE7\_HUMAN sp|P35637|FUS\_HUMAN sp|Q9Y4E6|WDR7\_HUMAN sp|P17066|HSP76\_HUMAN sp|P48741|HSP77\_HUMAN tr|H7C1L9|H7C1L9\_HUMAN tr|A0A087WYI5|A0A087WYI5\_HUMAN tr|A0A087WZN7|A0A087WZN7\_HUMAN sp|O60296|TRAK2\_HUMAN tr|H0Y5B5|H0Y5B5\_HUMAN sp|Q9NYA4|MTMR4\_HUMAN tr|H7BZ42|H7BZ42\_HUMAN tr|J3QR65|J3QR65\_HUMAN tr|A0A087WTV6|A0A087WTV6\_HUMAN sp|Q8NEL9|DDHD1\_HUMAN tr|H7C5E4|H7C5E4\_HUMAN tr|F8W7U8|F8W7U8\_HUMAN tr|B3KTC7|B3KTC7\_HUMAN sp|P49959|MRE11\_HUMAN sp|O43147|SGSM2\_HUMAN sp|Q8IVW6|ARI3B\_HUMAN sp|Q86YJ5|MARH9\_HUMAN sp|O60290|ZN862\_HUMAN tr|H3BUD4|H3BUD4\_HUMAN sp|Q9NRP7|STK36\_HUMAN sp|Q9UBS9|SUCO\_HUMAN sp|Q99570|PI3R4\_HUMAN sp|P01037|CYTN\_HUMAN sp|Q9UM54|MYO6\_HUMAN tr|E7EW20|E7EW20\_HUMAN sp|P51825|AFF1\_HUMAN sp|Q9Y5H3|PCDGA\_HUMAN tr|E9PMR6|E9PMR6\_HUMAN sp|Q8NFQ6|BPIFC\_HUMAN sp|O75385|ULK1\_HUMAN tr|J3QT27|J3QT27\_HUMAN tr|E9PFP8|E9PFP8\_HUMAN sp|Q13224|NMDE2\_HUMAN sp|Q9Y2I7|FYV1\_HUMAN sp|A0AVK6|E2F8\_HUMAN sp|Q9BXF6|RFIP5\_HUMAN sp|Q13129|RLF\_HUMAN sp|O14795|UN13B\_HUMAN tr|H0Y623|H0Y623\_HUMAN sp|Q5SZQ8|CELF3\_HUMAN tr|J3KR69|J3KR69\_HUMAN sp|Q9BWG4|SSBP4\_HUMAN sp|Q9NZ08|ERAP1\_HUMAN sp|Q9P209|CEP72\_HUMAN sp|Q9NT22|EMIL3\_HUMAN sp|Q16478|GRIK5\_HUMAN tr|M0QZI3|M0QZI3\_HUMAN sp|O14526|FCHO1\_HUMAN sp|A8CG34|P121C\_HUMAN sp|Q7Z3C6|ATG9A\_HUMAN sp|Q9ULD4|BRPF3\_HUMAN tr|E9PI60|E9PI60\_HUMAN sp|Q9C0B7|TNG6\_HUMAN sp|Q5T1N1|AKND1\_HUMAN sp|Q6UB35|C1TM\_HUMAN tr|A0A087WVB0|A0A087WVB0\_HUMAN sp|Q9Y5E2|PCDB7\_HUMAN sp|Q4KMP7|TB10B\_HUMAN sp|Q9UPX0|TUTLB\_HUMAN sp|Q9ULT0|TTC7A\_HUMAN tr|G5E9G4|G5E9G4\_HUMAN sp|Q14005|IL16\_HUMAN sp|Q14241|ELOA1\_HUMAN sp|Q86XP1|DGKH\_HUMAN sp|Q569K4|Z385B\_HUMAN sp|P10809|CH60\_HUMAN sp|O95487|SC24B\_HUMAN tr|F8W9B3|F8W9B3\_HUMAN sp|Q504T8|MIDN\_HUMAN sp|Q6IMN6|CAPR2\_HUMAN sp|Q8NDT2|RB15B\_HUMAN sp|O14796|SH21B\_HUMAN sp|O43593|HAIR\_HUMAN tr|G3XAN1|G3XAN1\_HUMAN sp|Q13395|TARB1\_HUMAN sp|Q13111|CAF1A\_HUMAN tr|H3BMW9|H3BMW9\_HUMAN sp|Q15459|SF3A1\_HUMAN tr|H3BRV5|H3BRV5\_HUMAN sp|Q6ZVD8|PHLP2\_HUMAN tr|H0Y493|H0Y493\_HUMAN tr|H3BMS5|H3BMS5\_HUMAN tr|H7C051|H7C051\_HUMAN sp|P45844|ABCG1\_HUMAN sp|Q8TDY8|IGDC4\_HUMAN sp|Q04771|ACVR1\_HUMAN sp|P35914|HMGCL\_HUMAN sp|Q9UP95|S12A4\_HUMAN tr|I3L4N6|I3L4N6\_HUMAN sp|Q8IYA2|C144C\_HUMAN sp|O43699|SIGL6\_HUMAN sp|P13056|NR2C1\_HUMAN sp|P31948|STIP1\_HUMAN tr|G8JLB6|G8JLB6\_HUMAN tr|C9J9Y7|C9J9Y7\_HUMAN tr|F8VS61|F8VS61\_HUMAN sp|O15488|GLYG2\_HUMAN sp|Q92625|ANS1A\_HUMAN tr|C9J9B2|C9J9B2\_HUMAN sp|Q5XX13|FBW10\_HUMAN sp|Q86SQ4|GP126\_HUMAN sp|Q7Z2E3|APTX\_HUMAN sp|P07949|RET\_HUMAN sp|P19174|PLCG1\_HUMAN sp|Q6IWH7|ANO7\_HUMAN sp|Q9UDY8|MALT1\_HUMAN sp|Q8NEV8|EXPH5\_HUMAN sp|Q8NG98|OR7D4\_HUMAN sp|P51812|KS6A3\_HUMAN tr|H0Y818|H0Y818\_HUMAN sp|P29375|KDM5A\_HUMAN sp|O76039|CDKL5\_HUMAN sp|O60840|CAC1F\_HUMAN sp|Q9UFD9|RIM3A\_HUMAN sp|P35556|FBN2\_HUMAN tr|A0A087WYV8|A0A087WYV8\_HUMAN tr|H7BXD0|H7BXD0\_HUMAN sp|O00534|VMA5A\_HUMAN sp|P46977|STT3A\_HUMAN sp|Q8TAP8|PPR35\_HUMAN tr|H7C5R7|H7C5R7\_HUMAN tr|K7ERQ2|K7ERQ2\_HUMAN sp|Q96ND0|F210A\_HUMAN tr|K7EK00|K7EK00\_HUMAN sp|Q9Y2W1|TR150\_HUMAN sp|Q9HCR9|PDE11\_HUMAN tr|H0Y424|H0Y424\_HUMAN sp|Q96NH3|BROMI\_HUMAN sp|Q86UT5|NHRF4\_HUMAN sp|Q5TFE4|NT5D1\_HUMAN tr|Q5TG12|Q5TG12\_HUMAN sp|Q15262|PTPRK\_HUMAN tr|E9PGC5|E9PGC5\_HUMAN tr|U3KQA6|U3KQA6\_HUMAN sp|P25440|BRD2\_HUMAN tr|H0Y6K2|H0Y6K2\_HUMAN sp|Q8WTV0|SCRB1\_HUMAN tr|F5H4R1|F5H4R1\_HUMAN sp|Q8N431|RGF1C\_HUMAN sp|Q15036|SNX17\_HUMAN tr|D3YTF8|D3YTF8\_HUMAN sp|Q9BXX2|AN30B\_HUMAN tr|A8MYT4|A8MYT4\_HUMAN tr|H3BTW5|H3BTW5\_HUMAN tr|H7BY16|H7BY16\_HUMAN sp|A8MWX3|WASH4\_HUMAN sp|Q69YN4|VIR\_HUMAN tr|H0YCY8|H0YCY8\_HUMAN sp|P54687|BCAT1\_HUMAN sp|Q5VW36|FOCAD\_HUMAN sp|P62495|ERF1\_HUMAN tr|B7Z7P8|B7Z7P8\_HUMAN sp|P49754|VPS41\_HUMAN tr|F5H2B9|F5H2B9\_HUMAN sp|Q6P9A2|GLT18\_HUMAN sp|P04629|NTRK1\_HUMAN sp|Q9NYF0|DACT1\_HUMAN tr|A8MTE9|A8MTE9\_HUMAN sp|Q9NPH2|INO1\_HUMAN sp|Q99523|SORT\_HUMAN sp|Q5VTE6|ANGE2\_HUMAN sp|Q8WTS1|ABHD5\_HUMAN sp|P63000|RAC1\_HUMAN tr|A0A087WYB2|A0A087WYB2\_HUMAN tr|A0A087X253|A0A087X253\_HUMAN tr|H0Y6R0|H0Y6R0\_HUMAN sp|O94812|BAIP3\_HUMAN sp|O94941|RNF37\_HUMAN tr|E7ES20|E7ES20\_HUMAN sp|O60341|KDM1A\_HUMAN tr|F5H6W4|F5H6W4\_HUMAN tr|E7ENK1|E7ENK1\_HUMAN sp|Q9UI17|M2GD\_HUMAN tr|H7C3T2|H7C3T2\_HUMAN sp|O95755|RAB36\_HUMAN sp|P16220|CREB1\_HUMAN sp|P02730|B3AT\_HUMAN sp|Q4KMG0|CDON\_HUMAN sp|P98198|AT8B2\_HUMAN sp|Q6GYQ0|RGPA1\_HUMAN sp|Q96B97|SH3K1\_HUMAN tr|Q5JPT2|Q5JPT2\_HUMAN sp|Q9BTE3|MCMBP\_HUMAN sp|Q9UKE5|TNIK\_HUMAN sp|Q9BX66|SRBS1\_HUMAN sp|P53814|SMTN\_HUMAN tr|A0A087X1R1|A0A087X1R1\_HUMAN sp|Q9H6K5|YS027\_HUMAN sp|Q9C099|LRCC1\_HUMAN sp|P08514|ITA2B\_HUMAN sp|Q9BTX1|NDC1\_HUMAN tr|C9JNM7|C9JNM7\_HUMAN sp|Q9UKP4|ATS7\_HUMAN tr|E5RJR1|E5RJR1\_HUMAN sp|Q9NZC9|SMAL1\_HUMAN sp|Q9ULH4|LRFN2\_HUMAN sp|Q2KHT3|CL16A\_HUMAN tr|Q5JT55|Q5JT55\_HUMAN sp|O00592|PODXL\_HUMAN sp|Q9BZA8|PC11Y\_HUMAN sp|Q6PL45|BRID5\_HUMAN sp|Q9Y3D6|FIS1\_HUMAN tr|H0Y3V3|H0Y3V3\_HUMAN sp|P49918|CDN1C\_HUMAN tr|A0A087X2I7|A0A087X2I7\_HUMAN sp|P33076|C2TA\_HUMAN tr|J3QKM3|J3QKM3\_HUMAN tr|F8VXG7|F8VXG7\_HUMAN sp|Q99590|SCAFB\_HUMAN sp|P21399|ACOC\_HUMAN sp|Q58A45|PAN3\_HUMAN sp|Q6UXG8|BTNL9\_HUMAN tr|B6VEX4|B6VEX4\_HUMAN tr|E9PFX0|E9PFX0\_HUMAN sp|Q15047|SETB1\_HUMAN sp|P54840|GYS2\_HUMAN sp|Q7RTU9|STRC\_HUMAN tr|K7ENS1|K7ENS1\_HUMAN sp|O95153|RIMB1\_HUMAN tr|H0YFZ6|H0YFZ6\_HUMAN tr|A2A369|A2A369\_HUMAN tr|A0A087WX95|A0A087WX95\_HUMAN sp|Q99593|TBX5\_HUMAN sp|Q15398|DLGP5\_HUMAN tr|H0YC99|H0YC99\_HUMAN tr|H0YDZ4|H0YDZ4\_HUMAN sp|Q6HA08|ASTL\_HUMAN sp|Q9H091|ZMY15\_HUMAN tr|F5H101|F5H101\_HUMAN sp|Q76FK4|NOL8\_HUMAN sp|Q9NTW7|ZF64B\_HUMAN sp|P47870|GBRB2\_HUMAN sp|Q7Z4G4|TRM11\_HUMAN sp|P35568|IRS1\_HUMAN sp|Q7KZN9|COX15\_HUMAN sp|Q9UNA0|ATS5\_HUMAN sp|Q8N0U7|CA087\_HUMAN tr|E5RGY9|E5RGY9\_HUMAN sp|Q8N3C0|ASCC3\_HUMAN tr|E7EVM0|E7EVM0\_HUMAN tr|E7ERH8|E7ERH8\_HUMAN sp|Q96QD8|S38A2\_HUMAN sp|Q6AI39|GSC1L\_HUMAN sp|Q96JP2|MY15B\_HUMAN tr|H7C070|H7C070\_HUMAN tr|H3BNQ4|H3BNQ4\_HUMAN sp|Q6ZT98|TTLL7\_HUMAN sp|Q13200|PSMD2\_HUMAN tr|F8WCX2|F8WCX2\_HUMAN sp|Q9Y4L1|HYOU1\_HUMAN sp|P78312|F193A\_HUMAN sp|Q8NG27|PJA1\_HUMAN tr|H7C453|H7C453\_HUMAN tr|S4R3B3|S4R3B3\_HUMAN tr|A0A087WXW9|A0A087WXW9\_HUMAN tr|E7ETH1|E7ETH1\_HUMAN sp|Q14003|KCNC3\_HUMAN sp|Q9Y5H8|PCDA3\_HUMAN sp|A2RUS2|DEND3\_HUMAN sp|Q86UP6|CUZD1\_HUMAN tr|F5GZI3|F5GZI3\_HUMAN tr|H0Y2W4|H0Y2W4\_HUMAN tr|H7C121|H7C121\_HUMAN sp|Q86Z14|KLOTB\_HUMAN tr|C9JAX7|C9JAX7\_HUMAN sp|Q92620|PRP16\_HUMAN tr|J3QRK4|J3QRK4\_HUMAN sp|O60732|MAGC1\_HUMAN sp|Q9BQ39|DDX50\_HUMAN sp|Q6P531|GGT6\_HUMAN tr|J3KPJ0|J3KPJ0\_HUMAN sp|Q86SQ0|PHLB2\_HUMAN tr|E9PFQ4|E9PFQ4\_HUMAN tr|H0YH87|H0YH87\_HUMAN tr|A0A087WX19|A0A087WX19\_HUMAN sp|Q14684|RRP1B\_HUMAN sp|O75360|PROP1\_HUMAN sp|P0CG20|PRR35\_HUMAN sp|Q5VZP5|DUS27\_HUMAN tr|F6VUX8|F6VUX8\_HUMAN tr|B5M0C0|B5M0C0\_HUMAN sp|Q9Y570|PPME1\_HUMAN sp|Q8TAD4|ZNT5\_HUMAN sp|P13671|CO6\_HUMAN tr|F8VQW8|F8VQW8\_HUMAN tr|C9K080|C9K080\_HUMAN sp|Q9UQP3|TENN\_HUMAN sp|Q9H330|TM245\_HUMAN sp|P10721|KIT\_HUMAN sp|Q8IUA7|ABCA9\_HUMAN tr|H0Y4U7|H0Y4U7\_HUMAN O76011 tr|H7C4W6|H7C4W6\_HUMAN tr|F5H783|F5H783\_HUMAN tr|F8VV01|F8VV01\_HUMAN tr|H0YKU1|H0YKU1\_HUMAN sp|P42166|LAP2A\_HUMAN tr|H0YNJ8|H0YNJ8\_HUMAN sp|Q8NH94|OR1L1\_HUMAN sp|O60318|GANP\_HUMAN sp|Q9BVQ7|SPA5L\_HUMAN sp|Q8NEP3|DAAF1\_HUMAN sp|Q6NYC8|PPR18\_HUMAN tr|E7ENE0|E7ENE0\_HUMAN tr|F2Z2K5|F2Z2K5\_HUMAN sp|P78368|KC1G2\_HUMAN sp|Q96N16|JKIP1\_HUMAN tr|F8VWM7|F8VWM7\_HUMAN sp|O60486|PLXC1\_HUMAN sp|P48067|SC6A9\_HUMAN tr|E7EVB6|E7EVB6\_HUMAN sp|Q13255|GRM1\_HUMAN sp|P61129|ZC3H6\_HUMAN sp|P08151|GLI1\_HUMAN tr|E9PFB6|E9PFB6\_HUMAN tr|H9KV28|H9KV28\_HUMAN sp|Q9P2G3|KLH14\_HUMAN tr|I3L2N2|I3L2N2\_HUMAN sp|Q8IZQ8|MYCD\_HUMAN sp|P55265|DSRAD\_HUMAN tr|F5GZ06|F5GZ06\_HUMAN sp|P18206|VINC\_HUMAN sp|O15111|IKKA\_HUMAN sp|Q8TEQ8|PIGO\_HUMAN sp|Q5XPI4|RN123\_HUMAN tr|E9PK39|E9PK39\_HUMAN tr|E9PMK9|E9PMK9\_HUMAN tr|A8MYV6|A8MYV6\_HUMAN sp|Q16665|HIF1A\_HUMAN tr|F8W9L0|F8W9L0\_HUMAN sp|Q9BZQ6|EDEM3\_HUMAN sp|Q9BWT7|CAR10\_HUMAN sp|Q96HN2|SAHH3\_HUMAN sp|Q8TDG4|HELQ\_HUMAN sp|Q9UHD9|UBQL2\_HUMAN tr|V5LU97|V5LU97\_HUMAN sp|O00291|HIP1\_HUMAN sp|Q9UKP5|ATS6\_HUMAN sp|Q9UI36|DACH1\_HUMAN tr|A0A087WZP2|A0A087WZP2\_HUMAN tr|Q5SZG2|Q5SZG2\_HUMAN sp|Q6PJT7|ZC3HE\_HUMAN sp|P01133|EGF\_HUMAN sp|Q969Y0|NXPE3\_HUMAN tr|A0A087X211|A0A087X211\_HUMAN sp|Q8TCG1|CIP2A\_HUMAN sp|Q14439|GP176\_HUMAN tr|B4DZ23|B4DZ23\_HUMAN tr|J3KNC6|J3KNC6\_HUMAN tr|H3BNA6|H3BNA6\_HUMAN sp|Q8NFR9|I17RE\_HUMAN tr|J3KQN7|J3KQN7\_HUMAN tr|H0YGE2|H0YGE2\_HUMAN tr|H0Y6I6|H0Y6I6\_HUMAN sp|Q03828|EVX2\_HUMAN sp|O43395|PRPF3\_HUMAN sp|Q07889|SOS1\_HUMAN sp|Q6P995|F171B\_HUMAN sp|Q9BRC7|PLCD4\_HUMAN tr|G5E9C8|G5E9C8\_HUMAN tr|A0JP02|A0JP02\_HUMAN sp|Q15375|EPHA7\_HUMAN tr|H7C2B5|H7C2B5\_HUMAN sp|Q12986|NFX1\_HUMAN sp|Q9Y5I0|PCDAD\_HUMAN tr|J3KSS7|J3KSS7\_HUMAN sp|Q9NZ52|GGA3\_HUMAN sp|O75129|ASTN2\_HUMAN tr|G9CGD6|G9CGD6\_HUMAN tr|A0A087WYY3|A0A087WYY3\_HUMAN sp|Q92585|MAML1\_HUMAN sp|Q9Y5H7|PCDA5\_HUMAN sp|Q99551|MTEF1\_HUMAN tr|B4DPR9|B4DPR9\_HUMAN tr|F5H6A0|F5H6A0\_HUMAN tr|C9J0F0|C9J0F0\_HUMAN sp|Q96GW7|PGCB\_HUMAN tr|E9PCY7|E9PCY7\_HUMAN sp|Q9NQB0|TF7L2\_HUMAN tr|F8VUC8|F8VUC8\_HUMAN sp|Q96K76|UBP47\_HUMAN tr|H0YFY6|H0YFY6\_HUMAN sp|Q01167|FOXK2\_HUMAN sp|Q8NFM7|I17RD\_HUMAN sp|Q9NQA3|WASH6\_HUMAN sp|Q8NH93|OR1L3\_HUMAN tr|C9JYJ0|C9JYJ0\_HUMAN sp|P43405|KSYK\_HUMAN sp|Q63HQ2|EGFLA\_HUMAN sp|Q9H9A5|CNO10\_HUMAN tr|H3BRD9|H3BRD9\_HUMAN sp|Q8TAK5|GABP2\_HUMAN sp|Q9P218|COKA1\_HUMAN tr|E9PB18|E9PB18\_HUMAN sp|P0CG38|POTEI\_HUMAN sp|P52736|ZN133\_HUMAN sp|O60303|K0556\_HUMAN sp|O60931|CTNS\_HUMAN sp|P31513|FMO3\_HUMAN sp|Q7Z628|ARHG8\_HUMAN sp|Q8N3R9|MPP5\_HUMAN tr|A6NCT7|A6NCT7\_HUMAN tr|H0YCG0|H0YCG0\_HUMAN tr|J3KQ37|J3KQ37\_HUMAN sp|P50395|GDIB\_HUMAN sp|Q6BDS2|URFB1\_HUMAN sp|Q9ULU8|CAPS1\_HUMAN tr|F1T0E5|F1T0E5\_HUMAN sp|O75691|UTP20\_HUMAN sp|Q8IXK0|PHC2\_HUMAN sp|Q96MC4|DDC8\_HUMAN sp|Q96CX6|LRC58\_HUMAN sp|Q53HC0|CCD92\_HUMAN sp|Q9H2M9|RBGPR\_HUMAN tr|D6RAR4|D6RAR4\_HUMAN sp|Q9P2J5|SYLC\_HUMAN sp|Q86T82|UBP37\_HUMAN sp|Q14106|TOB2\_HUMAN sp|O00167|EYA2\_HUMAN tr|E7ETN2|E7ETN2\_HUMAN sp|Q8IVF5|TIAM2\_HUMAN P13646-1 sp|P85299|PRR5\_HUMAN sp|Q9NRM6|I17RB\_HUMAN sp|Q13554|KCC2B\_HUMAN sp|Q6UY09|CEA20\_HUMAN tr|A0A087WXE1|A0A087WXE1\_HUMAN sp|O15516|CLOCK\_HUMAN sp|Q9UPZ9|ICK\_HUMAN sp|Q9P202|WHRN\_HUMAN sp|Q969M2|CXA10\_HUMAN tr|H0YB61|H0YB61\_HUMAN sp|Q9ULI0|ATD2B\_HUMAN tr|H0Y767|H0Y767\_HUMAN sp|O00329|PK3CD\_HUMAN sp|Q01804|OTUD4\_HUMAN tr|H0YBB6|H0YBB6\_HUMAN sp|A6NE52|K1875\_HUMAN sp|Q8N7X0|ADGB\_HUMAN sp|Q9BZZ2|SN\_HUMAN sp|Q6MZZ7|CAN13\_HUMAN tr|F8W1F5|F8W1F5\_HUMAN sp|Q8IVF7|FMNL3\_HUMAN sp|Q9BRB3|PIGQ\_HUMAN sp|Q9NUQ8|ABCF3\_HUMAN sp|Q86YV9|HPS6\_HUMAN sp|Q8N6W0|CELF5\_HUMAN sp|Q92794|KAT6A\_HUMAN sp|P06681|CO2\_HUMAN sp|P49917|DNLI4\_HUMAN sp|Q8IWY8|ZSC29\_HUMAN sp|Q9H7R5|ZN665\_HUMAN sp|P15923|TFE2\_HUMAN tr|S4R3Y1|S4R3Y1\_HUMAN tr|H0YN34|H0YN34\_HUMAN sp|Q9P203|BTBD7\_HUMAN sp|Q7Z4P5|GDF7\_HUMAN sp|Q6PIW4|FIGL1\_HUMAN tr|G5EA03|G5EA03\_HUMAN sp|Q96B86|RGMA\_HUMAN sp|Q15717|ELAV1\_HUMAN Q2KIH2 tr|B7ZBT8|B7ZBT8\_HUMAN sp|A4D0V7|CPED1\_HUMAN sp|Q6PJP8|DCR1A\_HUMAN sp|O14964|HGS\_HUMAN sp|Q9C0D4|Z518B\_HUMAN tr|A0A087WX60|A0A087WX60\_HUMAN tr|A0A087WZ79|A0A087WZ79\_HUMAN tr|A0A087X0T2|A0A087X0T2\_HUMAN tr|A0A087WYM6|A0A087WYM6\_HUMAN tr|A0A087X155|A0A087X155\_HUMAN sp|Q96SB8|SMC6\_HUMAN tr|F5H7G2|F5H7G2\_HUMAN sp|Q13207|TBX2\_HUMAN sp|Q8IUH8|SPP2C\_HUMAN sp|Q8IZF2|GP116\_HUMAN tr|H0YAW7|H0YAW7\_HUMAN tr|C9J338|C9J338\_HUMAN tr|J3QSS3|J3QSS3\_HUMAN sp|Q9BZC7|ABCA2\_HUMAN tr|A0A087WXK5|A0A087WXK5\_HUMAN sp|Q96MA6|KAD8\_HUMAN tr|A2A2Y8|A2A2Y8\_HUMAN tr|E9PPH6|E9PPH6\_HUMAN A3EZ82 sp|Q96MW7|TIGD1\_HUMAN sp|Q04724|TLE1\_HUMAN sp|Q6E0U4|DMKN\_HUMAN sp|P01031|CO5\_HUMAN sp|P15391|CD19\_HUMAN sp|Q86W92|LIPB1\_HUMAN tr|H0Y6F6|H0Y6F6\_HUMAN sp|O95628|CNOT4\_HUMAN sp|P31152|MK04\_HUMAN sp|Q8WWZ8|OIT3\_HUMAN sp|O75410|TACC1\_HUMAN sp|Q14151|SAFB2\_HUMAN tr|A0A087WW54|A0A087WW54\_HUMAN sp|Q96QD5|DEPD7\_HUMAN tr|F5H2D4|F5H2D4\_HUMAN sp|Q6ZVM7|TM1L2\_HUMAN sp|Q9NWH9|SLTM\_HUMAN tr|E7ETB3|E7ETB3\_HUMAN sp|Q9ULA0|DNPEP\_HUMAN sp|P08603|CFAH\_HUMAN sp|Q9NWQ8|PHAG1\_HUMAN tr|H0Y995|H0Y995\_HUMAN tr|A0A087WZ65|A0A087WZ65\_HUMAN sp|Q2VPB7|AP5B1\_HUMAN tr|F6X827|F6X827\_HUMAN sp|Q15303|ERBB4\_HUMAN tr|A0A087WW32|A0A087WW32\_HUMAN sp|Q9ULJ7|ANR50\_HUMAN sp|Q8TF72|SHRM3\_HUMAN tr|K7ENL6|K7ENL6\_HUMAN tr|H0YAJ5|H0YAJ5\_HUMAN sp|Q8TDC3|BRSK1\_HUMAN tr|A0A087WV04|A0A087WV04\_HUMAN sp|Q9NY74|ETAA1\_HUMAN sp|Q01831|XPC\_HUMAN sp|Q9Y6L6|SO1B1\_HUMAN tr|F5H6F9|F5H6F9\_HUMAN tr|F5H6F5|F5H6F5\_HUMAN tr|H7C117|H7C117\_HUMAN sp|P35968|VGFR2\_HUMAN sp|Q9Y5H4|PCDG1\_HUMAN tr|B5MDW0|B5MDW0\_HUMAN sp|A6NI28|RHG42\_HUMAN tr|H0YEJ7|H0YEJ7\_HUMAN tr|E9PJK4|E9PJK4\_HUMAN sp|Q9Y3L3|3BP1\_HUMAN sp|P01876|IGHA1\_HUMAN sp|O43683|BUB1\_HUMAN sp|Q7Z6L1|TCPR1\_HUMAN sp|Q9Y5V3|MAGD1\_HUMAN sp|Q99986|VRK1\_HUMAN tr|H0YEX2|H0YEX2\_HUMAN tr|K7EIR0|K7EIR0\_HUMAN sp|Q9BV38|WDR18\_HUMAN tr|A0A087X0S5|A0A087X0S5\_HUMAN sp|P12109|CO6A1\_HUMAN tr|A0A087WV61|A0A087WV61\_HUMAN tr|B4DKY1|B4DKY1\_HUMAN sp|Q8NE09|RGS22\_HUMAN sp|P49589|SYCC\_HUMAN sp|P26232|CTNA2\_HUMAN sp|Q7Z5L2|R3HCL\_HUMAN tr|A0A087X0M0|A0A087X0M0\_HUMAN tr|E7EQS8|E7EQS8\_HUMAN sp|Q6N021|TET2\_HUMAN tr|A0A087WV08|A0A087WV08\_HUMAN sp|P17040|ZSC20\_HUMAN tr|E9PE82|E9PE82\_HUMAN sp|P16219|ACADS\_HUMAN sp|Q9UI33|SCNBA\_HUMAN tr|F8VUY0|F8VUY0\_HUMAN sp|Q15583|TGIF1\_HUMAN tr|F8VVS9|F8VVS9\_HUMAN sp|P32942|ICAM3\_HUMAN tr|K7ERN2|K7ERN2\_HUMAN tr|F8VUA0|F8VUA0\_HUMAN sp|Q8WXU2|DYXC1\_HUMAN sp|Q9BR76|COR1B\_HUMAN tr|A0A087WT58|A0A087WT58\_HUMAN sp|Q6AI08|HEAT6\_HUMAN tr|G3V1A6|G3V1A6\_HUMAN sp|P57764|GSDMD\_HUMAN tr|A0A087WVE3|A0A087WVE3\_HUMAN tr|A0A087WT85|A0A087WT85\_HUMAN tr|A0A087WZS8|A0A087WZS8\_HUMAN tr|A0A087WVG0|A0A087WVG0\_HUMAN sp|Q15389|ANGP1\_HUMAN sp|Q99592|ZBT18\_HUMAN sp|P11498|PYC\_HUMAN sp|Q149N8|SHPRH\_HUMAN sp|P10645|CMGA\_HUMAN sp|P78337|PITX1\_HUMAN sp|Q9H9Y6|RPA2\_HUMAN sp|P48431|SOX2\_HUMAN sp|P78363|ABCA4\_HUMAN sp|Q9H6S0|YTDC2\_HUMAN sp|Q9NZN5|ARHGC\_HUMAN sp|Q8N8S7|ENAH\_HUMAN tr|C9JRL6|C9JRL6\_HUMAN tr|H0Y704|H0Y704\_HUMAN tr|H3BLX7|H3BLX7\_HUMAN sp|Q96M69|LRGUK\_HUMAN sp|Q9ULK0|GRID1\_HUMAN sp|Q8TCV5|WFDC5\_HUMAN sp|P35555|FBN1\_HUMAN sp|Q9NS98|SEM3G\_HUMAN tr|H0YI27|H0YI27\_HUMAN tr|E9PCM4|E9PCM4\_HUMAN sp|Q16650|TBR1\_HUMAN tr|G3V419|G3V419\_HUMAN sp|O15397|IPO8\_HUMAN sp|Q9UFE4|CCD39\_HUMAN sp|A4D1T9|PRS37\_HUMAN sp|O15297|PPM1D\_HUMAN sp|Q96N68|CR015\_HUMAN tr|M9T4I7|M9T4I7\_HUMAN sp|Q5U5Q3|MEX3C\_HUMAN tr|A8MVE2|A8MVE2\_HUMAN tr|D9IDM5|D9IDM5\_HUMAN tr|A0A087WSV6|A0A087WSV6\_HUMAN tr|F6TER3|F6TER3\_HUMAN tr|A0A087WSX8|A0A087WSX8\_HUMAN sp|Q8NHL6|LIRB1\_HUMAN sp|Q92887|MRP2\_HUMAN tr|H7C0P6|H7C0P6\_HUMAN sp|Q96EZ4|MYEOV\_HUMAN tr|C9J430|C9J430\_HUMAN sp|Q17R60|IMPG1\_HUMAN tr|A0A087WYL3|A0A087WYL3\_HUMAN tr|A0A087WYC9|A0A087WYC9\_HUMAN tr|F5H369|F5H369\_HUMAN sp|O75747|P3C2G\_HUMAN tr|H3BNZ4|H3BNZ4\_HUMAN tr|F5GXF5|F5GXF5\_HUMAN tr|G3V164|G3V164\_HUMAN sp|A8K2U0|A2ML1\_HUMAN sp|Q9ULX5|RN112\_HUMAN tr|A0A087WYQ2|A0A087WYQ2\_HUMAN sp|Q5T6C5|AT7L2\_HUMAN tr|Q5T6C4|Q5T6C4\_HUMAN tr|K7EP67|K7EP67\_HUMAN tr|E9PD50|E9PD50\_HUMAN sp|Q96JA1|LRIG1\_HUMAN tr|A4PB68|A4PB68\_HUMAN tr|H0YJ83|H0YJ83\_HUMAN sp|Q9UBU6|FA8A1\_HUMAN sp|Q02446|SP4\_HUMAN sp|A6NJ88|SGE2P\_HUMAN sp|Q14449|GRB14\_HUMAN tr|B4E2D5|B4E2D5\_HUMAN tr|R4GND2|R4GND2\_HUMAN sp|Q8WXI2|CNKR2\_HUMAN tr|C9JG86|C9JG86\_HUMAN sp|Q6ZMC9|SIG15\_HUMAN tr|F8WAZ7|F8WAZ7\_HUMAN tr|F8WFA6|F8WFA6\_HUMAN tr|C9J0Q4|C9J0Q4\_HUMAN sp|Q9Y6C5|PTC2\_HUMAN sp|P24588|AKAP5\_HUMAN sp|Q92574|TSC1\_HUMAN tr|Q8NHS7|Q8NHS7\_HUMAN sp|Q9UNN4|TF2AY\_HUMAN sp|P63010|AP2B1\_HUMAN sp|Q9GZU2|PEG3\_HUMAN sp|Q29RF7|PDS5A\_HUMAN tr|K7EPS3|K7EPS3\_HUMAN sp|Q86VD1|MORC1\_HUMAN tr|J3KSZ8|J3KSZ8\_HUMAN sp|Q68DK7|MSL1\_HUMAN sp|Q12778|FOXO1\_HUMAN tr|H7C2C6|H7C2C6\_HUMAN tr|V9GXZ1|V9GXZ1\_HUMAN tr|E7EX48|E7EX48\_HUMAN sp|O75923|DYSF\_HUMAN tr|J3QLI7|J3QLI7\_HUMAN sp|P47869|GBRA2\_HUMAN sp|Q00610|CLH1\_HUMAN sp|Q9NQU5|PAK6\_HUMAN sp|Q9H1N7|S35B3\_HUMAN tr|E9PBQ7|E9PBQ7\_HUMAN tr|H3BNT4|H3BNT4\_HUMAN tr|H3BU96|H3BU96\_HUMAN sp|Q99547|MPH6\_HUMAN sp|A6NCI8|CB078\_HUMAN tr|F5H2I0|F5H2I0\_HUMAN sp|Q9NY91|SC5A4\_HUMAN sp|Q15386|UBE3C\_HUMAN tr|E7EV07|E7EV07\_HUMAN sp|P11229|ACM1\_HUMAN tr|E7EW59|E7EW59\_HUMAN sp|Q96LT7|CI072\_HUMAN tr|G3V1J5|G3V1J5\_HUMAN sp|O00754|MA2B1\_HUMAN sp|Q9Y2L1|RRP44\_HUMAN sp|Q9UKU6|TRHDE\_HUMAN sp|Q9Y6J0|CABIN\_HUMAN sp|Q2V2M9|FHOD3\_HUMAN tr|H0YA70|H0YA70\_HUMAN sp|Q7Z7H3|CATIP\_HUMAN sp|Q008S8|ECT2L\_HUMAN sp|P35348|ADA1A\_HUMAN sp|P19525|E2AK2\_HUMAN tr|H0Y759|H0Y759\_HUMAN sp|Q96SE7|ZN347\_HUMAN sp|P23109|AMPD1\_HUMAN sp|Q13485|SMAD4\_HUMAN sp|A7MD48|SRRM4\_HUMAN tr|E7EMK3|E7EMK3\_HUMAN sp|Q14254|FLOT2\_HUMAN sp|Q9NQA5|TRPV5\_HUMAN sp|P16499|PDE6A\_HUMAN tr|F1T0K3|F1T0K3\_HUMAN tr|E9PIW2|E9PIW2\_HUMAN sp|O60522|TDRD6\_HUMAN sp|Q9NZV8|KCND2\_HUMAN sp|O95619|YETS4\_HUMAN sp|Q9H4L7|SMRCD\_HUMAN tr|D6RGV6|D6RGV6\_HUMAN tr|D6RJF7|D6RJF7\_HUMAN sp|Q96LB8|PGRP4\_HUMAN sp|Q5XG87|PAPD7\_HUMAN tr|F8WCG5|F8WCG5\_HUMAN tr|H7BXU2|H7BXU2\_HUMAN tr|E7EP41|E7EP41\_HUMAN tr|M0R230|M0R230\_HUMAN sp|Q8TAU3|ZN417\_HUMAN sp|Q96SQ5|ZN587\_HUMAN sp|Q96RY5|CRML\_HUMAN tr|H7BYZ3|H7BYZ3\_HUMAN sp|P29322|EPHA8\_HUMAN tr|A0A075B7G8|A0A075B7G8\_HUMAN sp|O00213|APBB1\_HUMAN tr|J3KPL8|J3KPL8\_HUMAN sp|Q9H3R1|NDST4\_HUMAN tr|H0Y720|H0Y720\_HUMAN sp|Q9Y450|HBS1L\_HUMAN sp|Q06481|APLP2\_HUMAN tr|H0YJR2|H0YJR2\_HUMAN sp|A2VDJ0|T131L\_HUMAN sp|P49641|MA2A2\_HUMAN tr|Q5T1N2|Q5T1N2\_HUMAN tr|F5H1X6|F5H1X6\_HUMAN sp|P20908|CO5A1\_HUMAN sp|Q8IV76|PASD1\_HUMAN tr|F8W7E0|F8W7E0\_HUMAN sp|Q13112|CAF1B\_HUMAN sp|O60320|F1891\_HUMAN tr|E5RG70|E5RG70\_HUMAN sp|Q16799|RTN1\_HUMAN tr|F5H0N7|F5H0N7\_HUMAN sp|P29401|TKT\_HUMAN tr|E9PKB1|E9PKB1\_HUMAN sp|Q0P6D6|CCD15\_HUMAN sp|Q9H598|VIAAT\_HUMAN sp|Q8IVJ1|S41A1\_HUMAN sp|P11169|GTR3\_HUMAN sp|Q6ZQY2|LR74B\_HUMAN tr|C9JK49|C9JK49\_HUMAN sp|O94763|RMP\_HUMAN sp|Q7KZI7|MARK2\_HUMAN tr|A0A088AWN3|A0A088AWN3\_HUMAN sp|Q99569|PKP4\_HUMAN tr|E9PMZ8|E9PMZ8\_HUMAN sp|Q9HCC6|HES4\_HUMAN tr|E9PB28|E9PB28\_HUMAN tr|F8W6G5|F8W6G5\_HUMAN sp|Q96I76|GPTC3\_HUMAN sp|Q03518|TAP1\_HUMAN sp|Q9H0C5|BTBD1\_HUMAN sp|Q86Y91|KI18B\_HUMAN sp|Q96T76|MMS19\_HUMAN tr|C9JA36|C9JA36\_HUMAN tr|K7EKX9|K7EKX9\_HUMAN tr|K7EN83|K7EN83\_HUMAN tr|B4DKV7|B4DKV7\_HUMAN sp|Q8TE68|ES8L1\_HUMAN tr|E7EUU4|E7EUU4\_HUMAN sp|Q8NBP7|PCSK9\_HUMAN sp|Q9H1K0|RBNS5\_HUMAN sp|Q8IUR6|CRERF\_HUMAN tr|A0A087WUG7|A0A087WUG7\_HUMAN tr|G3V112|G3V112\_HUMAN sp|Q8TEC5|SH3R2\_HUMAN tr|H0Y843|H0Y843\_HUMAN sp|Q96M95|CCD42\_HUMAN sp|Q9UNX4|WDR3\_HUMAN tr|J3KTI8|J3KTI8\_HUMAN tr|F5GY05|F5GY05\_HUMAN tr|E9PK91|E9PK91\_HUMAN sp|Q00765|REEP5\_HUMAN sp|Q9NYF8|BCLF1\_HUMAN sp|Q9HBE1|PATZ1\_HUMAN tr|B1AHF5|B1AHF5\_HUMAN sp|Q9HC36|MRM3\_HUMAN tr|Q5T7A9|Q5T7A9\_HUMAN sp|Q86Y26|NUTM1\_HUMAN sp|P98196|AT11A\_HUMAN tr|F6WC43|F6WC43\_HUMAN sp|Q12767|K0195\_HUMAN sp|Q8NB78|KDM1B\_HUMAN tr|C9JL75|C9JL75\_HUMAN tr|H0Y6H0|H0Y6H0\_HUMAN sp|Q9Y467|SALL2\_HUMAN sp|Q9H6R4|NOL6\_HUMAN sp|P19835|CEL\_HUMAN sp|Q9ULK2|AT7L1\_HUMAN tr|X6R868|X6R868\_HUMAN sp|Q9NZL4|HPBP1\_HUMAN tr|R4GNH6|R4GNH6\_HUMAN sp|Q07912|ACK1\_HUMAN tr|E7ER61|E7ER61\_HUMAN tr|H0Y9P1|H0Y9P1\_HUMAN sp|P36888|FLT3\_HUMAN sp|O60907|TBL1X\_HUMAN sp|P19838|NFKB1\_HUMAN sp|Q9Y5I3|PCDA1\_HUMAN tr|H3BQK4|H3BQK4\_HUMAN sp|Q6SZW1|SARM1\_HUMAN tr|C9JFJ0|C9JFJ0\_HUMAN sp|Q96BJ8|ELMO3\_HUMAN tr|H0Y2R3|H0Y2R3\_HUMAN sp|Q9UG63|ABCF2\_HUMAN sp|Q5T4T6|SYC2L\_HUMAN sp|Q8IZX4|TAF1L\_HUMAN tr|K7N7B3|K7N7B3\_HUMAN tr|H7C0W7|H7C0W7\_HUMAN sp|Q6H8Q1|ABLM2\_HUMAN sp|Q93034|CUL5\_HUMAN sp|P49588|SYAC\_HUMAN tr|E7ERW8|E7ERW8\_HUMAN sp|P48547|KCNC1\_HUMAN tr|Q2TB39|Q2TB39\_HUMAN sp|Q8IZU2|WDR17\_HUMAN sp|Q96L34|MARK4\_HUMAN sp|P55197|AF10\_HUMAN sp|Q9UKL2|O52A1\_HUMAN tr|F5H228|F5H228\_HUMAN sp|Q9H2Z4|NKX24\_HUMAN sp|Q15831|STK11\_HUMAN tr|K7EP59|K7EP59\_HUMAN sp|Q8NBR6|FA63B\_HUMAN sp|A2PYH4|HFM1\_HUMAN sp|Q5VT03|NTM2D\_HUMAN sp|Q86VF7|NRAP\_HUMAN tr|A0A087X2C9|A0A087X2C9\_HUMAN tr|F5H7I4|F5H7I4\_HUMAN tr|H0YMZ5|H0YMZ5\_HUMAN sp|Q06547|GABP1\_HUMAN sp|Q8NEN0|ARMC2\_HUMAN tr|V9GYZ6|V9GYZ6\_HUMAN sp|Q06710|PAX8\_HUMAN sp|Q9BXL7|CAR11\_HUMAN sp|Q68EM7|RHG17\_HUMAN sp|P0C617|O5AL1\_HUMAN sp|Q9Y334|VWA7\_HUMAN tr|E9PID8|E9PID8\_HUMAN tr|H7C3K2|H7C3K2\_HUMAN sp|Q14831|GRM7\_HUMAN sp|P33240|CSTF2\_HUMAN tr|E7EWR4|E7EWR4\_HUMAN sp|Q9BY89|K1671\_HUMAN sp|Q16832|DDR2\_HUMAN sp|Q6ZV29|PLPL7\_HUMAN sp|P54219|VMAT1\_HUMAN tr|G5E948|G5E948\_HUMAN sp|O94972|TRI37\_HUMAN sp|Q3B820|F161A\_HUMAN tr|E7EQM5|E7EQM5\_HUMAN tr|C9J6Z6|C9J6Z6\_HUMAN tr|C9JQL4|C9JQL4\_HUMAN tr|C9JY76|C9JY76\_HUMAN tr|C9JEN1|C9JEN1\_HUMAN tr|C9JEH2|C9JEH2\_HUMAN tr|C9J196|C9J196\_HUMAN tr|C9JYS1|C9JYS1\_HUMAN sp|P50454|SERPH\_HUMAN tr|H0Y6F5|H0Y6F5\_HUMAN tr|E9PL33|E9PL33\_HUMAN tr|E9PS58|E9PS58\_HUMAN tr|E9PM05|E9PM05\_HUMAN tr|E9PLB8|E9PLB8\_HUMAN sp|O14681|EI24\_HUMAN sp|Q8N1N0|CLC4F\_HUMAN tr|H7BZT7|H7BZT7\_HUMAN sp|Q16666|IF16\_HUMAN sp|Q6KCM7|SCMC2\_HUMAN tr|H3BSM9|H3BSM9\_HUMAN sp|Q9NSC2|SALL1\_HUMAN tr|F8WCM8|F8WCM8\_HUMAN sp|O15197|EPHB6\_HUMAN sp|Q92539|LPIN2\_HUMAN tr|B5MCG9|B5MCG9\_HUMAN sp|Q9Y2K5|R3HD2\_HUMAN sp|P59510|ATS20\_HUMAN tr|B5MCU0|B5MCU0\_HUMAN tr|J3QKR5|J3QKR5\_HUMAN tr|J3QR29|J3QR29\_HUMAN tr|B7ZVY7|B7ZVY7\_HUMAN tr|J3QR44|J3QR44\_HUMAN sp|P21127|CD11B\_HUMAN sp|Q8IXF0|NPAS3\_HUMAN sp|Q9H254|SPTN4\_HUMAN tr|C9JY79|C9JY79\_HUMAN tr|F8VS42|F8VS42\_HUMAN tr|F8VR32|F8VR32\_HUMAN sp|P11362|FGFR1\_HUMAN tr|C0H5X0|C0H5X0\_HUMAN tr|A0A087X1Y9|A0A087X1Y9\_HUMAN sp|Q9H239|MMP28\_HUMAN tr|B3KV06|B3KV06\_HUMAN tr|E9PFG7|E9PFG7\_HUMAN sp|Q9H892|TTC12\_HUMAN sp|Q9Y2M0|FAN1\_HUMAN tr|C9JZ93|C9JZ93\_HUMAN tr|C9JK83|C9JK83\_HUMAN tr|H0YAW0|H0YAW0\_HUMAN sp|Q13336|UT1\_HUMAN tr|F5H5B9|F5H5B9\_HUMAN sp|O75762|TRPA1\_HUMAN sp|O95630|STABP\_HUMAN tr|C9JEK5|C9JEK5\_HUMAN sp|Q9HCH5|SYTL2\_HUMAN tr|H7C2E4|H7C2E4\_HUMAN tr|Q5T5P0|Q5T5P0\_HUMAN sp|Q9NQH7|XPP3\_HUMAN tr|F8VPI7|F8VPI7\_HUMAN sp|Q9NUL3|STAU2\_HUMAN tr|E7EVJ4|E7EVJ4\_HUMAN tr|E7EPX0|E7EPX0\_HUMAN tr|E9PH62|E9PH62\_HUMAN sp|Q8IWU9|TPH2\_HUMAN sp|Q9UHL9|GT2D1\_HUMAN tr|J9JIF5|J9JIF5\_HUMAN sp|Q6ZWH5|NEK10\_HUMAN sp|Q9Y239|NOD1\_HUMAN tr|F5H1S1|F5H1S1\_HUMAN sp|Q9BQ51|PD1L2\_HUMAN sp|Q92521|PIGB\_HUMAN tr|H0Y713|H0Y713\_HUMAN tr|D6REA0|D6REA0\_HUMAN sp|P35221|CTNA1\_HUMAN sp|Q8IV36|HID1\_HUMAN tr|G3XAM7|G3XAM7\_HUMAN sp|O75879|GATB\_HUMAN sp|Q13733|AT1A4\_HUMAN tr|K7ES32|K7ES32\_HUMAN sp|Q14865|ARI5B\_HUMAN sp|Q9UN72|PCDA7\_HUMAN sp|O75636|FCN3\_HUMAN tr|E7EU81|E7EU81\_HUMAN tr|H7BXS7|H7BXS7\_HUMAN sp|Q86VF2|IGFN1\_HUMAN sp|Q9BXW9|FACD2\_HUMAN tr|J3KNK0|J3KNK0\_HUMAN sp|Q8IX12|CCAR1\_HUMAN sp|Q5T601|GP110\_HUMAN sp|Q8N2R8|FA43A\_HUMAN sp|Q9BZL6|KPCD2\_HUMAN tr|H7C3H4|H7C3H4\_HUMAN sp|Q9HD43|PTPRH\_HUMAN sp|Q6ZMP0|THSD4\_HUMAN sp|Q27J81|INF2\_HUMAN sp|Q99728|BARD1\_HUMAN sp|Q9UQR0|SCML2\_HUMAN sp|Q9BZW2|S13A1\_HUMAN sp|O94991|SLIK5\_HUMAN sp|Q5JU00|TCTE1\_HUMAN sp|Q92900|RENT1\_HUMAN sp|A2RUB6|CCD66\_HUMAN tr|H0YJK7|H0YJK7\_HUMAN sp|Q63ZE4|S22AA\_HUMAN sp|Q6R327|RICTR\_HUMAN sp|Q9UN42|AT1B4\_HUMAN tr|B7ZKW0|B7ZKW0\_HUMAN tr|H0YHD9|H0YHD9\_HUMAN tr|B1AJR9|B1AJR9\_HUMAN sp|Q8WUJ3|CEMIP\_HUMAN sp|Q9NVM9|ASUN\_HUMAN sp|Q86Y56|HEAT2\_HUMAN sp|Q68DC2|ANKS6\_HUMAN tr|G3V1V1|G3V1V1\_HUMAN tr|F8VXY6|F8VXY6\_HUMAN sp|Q8TBF4|ZCRB1\_HUMAN sp|Q16099|GRIK4\_HUMAN sp|A6PVC2|TTLL8\_HUMAN sp|Q8IZP9|GPR64\_HUMAN sp|Q13683|ITA7\_HUMAN sp|Q9NVC6|MED17\_HUMAN sp|Q9ULT6|ZNRF3\_HUMAN tr|M0R3C6|M0R3C6\_HUMAN sp|Q92536|YLAT2\_HUMAN sp|P49902|5NTC\_HUMAN tr|E5RGJ7|E5RGJ7\_HUMAN sp|Q8N9W8|FA71D\_HUMAN sp|P37198|NUP62\_HUMAN tr|J3QTM1|J3QTM1\_HUMAN sp|P14618|KPYM\_HUMAN sp|Q7Z5K2|WAPL\_HUMAN sp|Q96KR1|ZFR\_HUMAN tr|H0YNN7|H0YNN7\_HUMAN sp|P02452|CO1A1\_HUMAN sp|O43313|ATMIN\_HUMAN sp|Q9UPX6|K1024\_HUMAN tr|F8WE42|F8WE42\_HUMAN tr|H0Y3B8|H0Y3B8\_HUMAN tr|H3BUS3|H3BUS3\_HUMAN sp|P52790|HXK3\_HUMAN sp|O15547|P2RX6\_HUMAN tr|C9JC66|C9JC66\_HUMAN tr|A0A087WWM3|A0A087WWM3\_HUMAN sp|P10074|ZBT48\_HUMAN sp|Q14573|ITPR3\_HUMAN sp|Q9HC35|EMAL4\_HUMAN tr|B5MBZ0|B5MBZ0\_HUMAN sp|Q3ZCN5|OTOGL\_HUMAN sp|Q96JN8|NEUL4\_HUMAN tr|H0YCD7|H0YCD7\_HUMAN tr|E9PNH0|E9PNH0\_HUMAN sp|Q9NTX9|F217B\_HUMAN sp|Q13202|DUS8\_HUMAN sp|P54198|HIRA\_HUMAN sp|Q969G2|LHX4\_HUMAN sp|Q5VUB5|F1711\_HUMAN sp|Q4LE39|ARI4B\_HUMAN sp|P04035|HMDH\_HUMAN tr|K7EIX2|K7EIX2\_HUMAN sp|O95279|KCNK5\_HUMAN tr|K7ES98|K7ES98\_HUMAN sp|Q92619|HMHA1\_HUMAN tr|K7EM85|K7EM85\_HUMAN tr|A0A087WVE9|A0A087WVE9\_HUMAN sp|Q9HBZ2|ARNT2\_HUMAN sp|Q8N556|AFAP1\_HUMAN sp|Q96RT8|GCP5\_HUMAN sp|Q9NUN5|LMBD1\_HUMAN tr|A0A087X1Z1|A0A087X1Z1\_HUMAN tr|H0YBK2|H0YBK2\_HUMAN tr|A6NGW1|A6NGW1\_HUMAN tr|A0A087WVM6|A0A087WVM6\_HUMAN tr|H7C1C2|H7C1C2\_HUMAN sp|Q9NXF1|TEX10\_HUMAN tr|A0A087WV07|A0A087WV07\_HUMAN tr|F8U8C0|F8U8C0\_HUMAN sp|Q9BYT8|NEUL\_HUMAN sp|O94988|FA13A\_HUMAN sp|Q9UJX3|APC7\_HUMAN sp|O15042|SR140\_HUMAN sp|Q5T160|SYRM\_HUMAN tr|E7ET15|E7ET15\_HUMAN sp|O94782|UBP1\_HUMAN sp|Q96NR3|PTHD1\_HUMAN sp|P01236|PRL\_HUMAN sp|Q96BF6|NACC2\_HUMAN sp|Q8WYL5|SSH1\_HUMAN tr|F8W705|F8W705\_HUMAN sp|Q9H7P9|PKHG2\_HUMAN sp|Q03721|KCNC4\_HUMAN tr|H7BZ66|H7BZ66\_HUMAN sp|P22681|CBL\_HUMAN tr|A0A087WWT0|A0A087WWT0\_HUMAN sp|Q96JJ6|JPH4\_HUMAN sp|Q8TES7|FBF1\_HUMAN sp|Q86VR2|F134C\_HUMAN sp|Q9ULV0|MYO5B\_HUMAN sp|P78540|ARGI2\_HUMAN sp|P52179|MYOM1\_HUMAN tr|D3YTF9|D3YTF9\_HUMAN tr|F5H5V6|F5H5V6\_HUMAN tr|E9PHA6|E9PHA6\_HUMAN tr|C9K0V9|C9K0V9\_HUMAN sp|Q96RD0|OR8B2\_HUMAN sp|Q6Q8B3|MO2R2\_HUMAN tr|B5MED8|B5MED8\_HUMAN sp|Q96PQ0|SORC2\_HUMAN sp|Q7L4E1|FA73B\_HUMAN tr|H0Y406|H0Y406\_HUMAN sp|P22732|GTR5\_HUMAN sp|Q8TCJ2|STT3B\_HUMAN tr|X6RKN2|X6RKN2\_HUMAN tr|C9JW01|C9JW01\_HUMAN sp|Q16186|ADRM1\_HUMAN tr|A0A075B6N7|A0A075B6N7\_HUMAN sp|P01877|IGHA2\_HUMAN sp|A1A4T8|CN182\_HUMAN tr|K7EMJ5|K7EMJ5\_HUMAN tr|H0Y451|H0Y451\_HUMAN tr|H0YL34|H0YL34\_HUMAN tr|A0A075B7B1|A0A075B7B1\_HUMAN tr|J3KPF3|J3KPF3\_HUMAN tr|H0Y7J9|H0Y7J9\_HUMAN sp|Q9UI26|IPO11\_HUMAN sp|O95886|DLGP3\_HUMAN sp|P0CG39|POTEJ\_HUMAN tr|F8WCY8|F8WCY8\_HUMAN sp|Q5JRX3|PREP\_HUMAN sp|Q6URK8|TEPP\_HUMAN tr|H0Y9Q4|H0Y9Q4\_HUMAN tr|H0Y380|H0Y380\_HUMAN tr|H0Y4H1|H0Y4H1\_HUMAN tr|A0A087WY47|A0A087WY47\_HUMAN sp|O95409|ZIC2\_HUMAN sp|Q7Z6M2|FBX33\_HUMAN sp|Q8TE82|S3TC1\_HUMAN tr|E9PDS3|E9PDS3\_HUMAN tr|C9JRR5|C9JRR5\_HUMAN sp|Q93084|AT2A3\_HUMAN sp|Q8IWC1|MA7D3\_HUMAN sp|Q9Y487|VPP2\_HUMAN sp|P55884|EIF3B\_HUMAN sp|P03951|FA11\_HUMAN sp|Q9Y305|ACOT9\_HUMAN sp|Q8TF01|PNISR\_HUMAN sp|A0PJY2|FEZF1\_HUMAN sp|P54793|ARSF\_HUMAN tr|E9PDH4|E9PDH4\_HUMAN sp|Q6R2W3|SCND3\_HUMAN sp|Q8NEE6|FXL13\_HUMAN sp|O75914|PAK3\_HUMAN sp|Q15270|NKX11\_HUMAN tr|A0A087X294|A0A087X294\_HUMAN tr|A0A087X0M7|A0A087X0M7\_HUMAN sp|O75031|HSF2B\_HUMAN tr|A0A087X216|A0A087X216\_HUMAN sp|Q8NE31|FA13C\_HUMAN tr|B7Z2K3|B7Z2K3\_HUMAN sp|P07197|NFM\_HUMAN tr|E7ESP9|E7ESP9\_HUMAN tr|H3BM14|H3BM14\_HUMAN tr|A0A087X054|A0A087X054\_HUMAN sp|Q7Z2Z1|TICRR\_HUMAN sp|Q92805|GOGA1\_HUMAN tr|A0A087WT28|A0A087WT28\_HUMAN tr|D6RA03|D6RA03\_HUMAN sp|Q14119|VEZF1\_HUMAN tr|J3QSH4|J3QSH4\_HUMAN sp|Q5VZK9|LR16A\_HUMAN tr|H0Y7L6|H0Y7L6\_HUMAN sp|Q86SG6|NEK8\_HUMAN sp|Q02447|SP3\_HUMAN tr|H0Y6K5|H0Y6K5\_HUMAN sp|Q96KN4|FA84A\_HUMAN tr|F2Z2V1|F2Z2V1\_HUMAN sp|Q96C11|FGGY\_HUMAN sp|O43300|LRRT2\_HUMAN tr|H0YJB5|H0YJB5\_HUMAN sp|Q5SR56|HIAL1\_HUMAN sp|Q86XL3|ANKL2\_HUMAN sp|O43303|CP110\_HUMAN sp|Q86YC2|PALB2\_HUMAN sp|O75751|S22A3\_HUMAN sp|O94823|AT10B\_HUMAN tr|Q5T1W7|Q5T1W7\_HUMAN sp|Q9UL54|TAOK2\_HUMAN sp|Q12860|CNTN1\_HUMAN tr|C9JJV8|C9JJV8\_HUMAN sp|P16615|AT2A2\_HUMAN tr|H7C5W9|H7C5W9\_HUMAN sp|P13533|MYH6\_HUMAN sp|O75529|TAF5L\_HUMAN sp|Q9UPW0|FOXJ3\_HUMAN sp|Q53EV4|LRC23\_HUMAN sp|O43490|PROM1\_HUMAN sp|Q8NG99|OR7G2\_HUMAN sp|Q96EB6|SIR1\_HUMAN tr|J3KPH3|J3KPH3\_HUMAN sp|Q9NXL6|SIDT1\_HUMAN tr|Q5VXI4|Q5VXI4\_HUMAN tr|F8WCT9|F8WCT9\_HUMAN tr|H0Y2V6|H0Y2V6\_HUMAN sp|Q7L1I2|SV2B\_HUMAN tr|C9JKF1|C9JKF1\_HUMAN sp|P78357|CNTP1\_HUMAN sp|P14859|PO2F1\_HUMAN sp|Q9NUD7|CT096\_HUMAN tr|F5GZA9|F5GZA9\_HUMAN tr|Q5JYC3|Q5JYC3\_HUMAN tr|H0YGZ3|H0YGZ3\_HUMAN sp|O75694|NU155\_HUMAN tr|E9PF10|E9PF10\_HUMAN tr|C9JFF1|C9JFF1\_HUMAN sp|Q8TBA6|GOGA5\_HUMAN sp|P12956|XRCC6\_HUMAN tr|B1AHC9|B1AHC9\_HUMAN sp|Q9H9A7|RMI1\_HUMAN sp|Q9P1A6|DLGP2\_HUMAN tr|E9PLB2|E9PLB2\_HUMAN tr|H0YBY6|H0YBY6\_HUMAN tr|J3KSK6|J3KSK6\_HUMAN tr|E9PQG4|E9PQG4\_HUMAN sp|P48380|RFX3\_HUMAN sp|Q5W041|ARMC3\_HUMAN sp|Q99808|S29A1\_HUMAN tr|E7ES84|E7ES84\_HUMAN sp|Q14697|GANAB\_HUMAN tr|E9PKU7|E9PKU7\_HUMAN tr|F5H6X6|F5H6X6\_HUMAN sp|Q9UHK0|NUFP1\_HUMAN tr|E9PIQ3|E9PIQ3\_HUMAN sp|O43291|SPIT2\_HUMAN tr|K7EM91|K7EM91\_HUMAN sp|E7EW31|PROB1\_HUMAN tr|A0A087WXT0|A0A087WXT0\_HUMAN tr|A0A087WXC4|A0A087WXC4\_HUMAN sp|P25100|ADA1D\_HUMAN tr|H7C1J4|H7C1J4\_HUMAN sp|Q9Y2D9|ZN652\_HUMAN sp|Q9UBV2|SE1L1\_HUMAN sp|Q14126|DSG2\_HUMAN sp|Q9C0H6|KLHL4\_HUMAN sp|P10253|LYAG\_HUMAN sp|O14924|RGS12\_HUMAN sp|O15228|GNPAT\_HUMAN tr|Q5TBH8|Q5TBH8\_HUMAN sp|Q9NSY1|BMP2K\_HUMAN sp|A6PVS8|LRIQ3\_HUMAN tr|A0A087WWM4|A0A087WWM4\_HUMAN sp|Q9P2T1|GMPR2\_HUMAN tr|F8WAN9|F8WAN9\_HUMAN tr|E7ENG7|E7ENG7\_HUMAN sp|Q6BAA4|FCRLB\_HUMAN sp|Q9UHI6|DDX20\_HUMAN sp|O15120|PLCB\_HUMAN sp|Q01814|AT2B2\_HUMAN sp|Q86V85|GP180\_HUMAN tr|H3BV44|H3BV44\_HUMAN sp|Q16531|DDB1\_HUMAN tr|F5GY55|F5GY55\_HUMAN sp|Q9BQQ3|GORS1\_HUMAN tr|B4E1H8|B4E1H8\_HUMAN sp|P30530|UFO\_HUMAN tr|H3BV49|H3BV49\_HUMAN sp|Q6ZMI0|PPR21\_HUMAN sp|Q96HP8|T176A\_HUMAN sp|C9J798|RAS4B\_HUMAN tr|F5GXT2|F5GXT2\_HUMAN sp|O43374|RASL2\_HUMAN tr|F8W8T1|F8W8T1\_HUMAN sp|Q96PX6|CC85A\_HUMAN tr|I6L9E5|I6L9E5\_HUMAN sp|P28329|CLAT\_HUMAN tr|A0A087WUK4|A0A087WUK4\_HUMAN tr|A0A096LNW3|A0A096LNW3\_HUMAN tr|U3KQB3|U3KQB3\_HUMAN sp|Q8WXF7|ATLA1\_HUMAN tr|M0R152|M0R152\_HUMAN sp|P23490|LORI\_HUMAN sp|Q96SQ9|CP2S1\_HUMAN tr|A0A087WYL5|A0A087WYL5\_HUMAN sp|P30304|MPIP1\_HUMAN sp|Q9NWD8|TM248\_HUMAN tr|Q5TCC4|Q5TCC4\_HUMAN sp|Q9Y6F6|MRVI1\_HUMAN tr|H0YI08|H0YI08\_HUMAN sp|P15918|RAG1\_HUMAN tr|B1ALY0|B1ALY0\_HUMAN sp|Q9H0W8|SMG9\_HUMAN tr|H0Y858|H0Y858\_HUMAN sp|O14640|DVL1\_HUMAN sp|Q9NRF2|SH2B1\_HUMAN sp|P25929|NPY1R\_HUMAN tr|A0A087WSZ7|A0A087WSZ7\_HUMAN sp|Q08499|PDE4D\_HUMAN tr|F5H4N6|F5H4N6\_HUMAN tr|H0YFK8|H0YFK8\_HUMAN sp|Q14938|NFIX\_HUMAN tr|K7EN08|K7EN08\_HUMAN tr|E5RIG2|E5RIG2\_HUMAN sp|Q96PZ7|CSMD1\_HUMAN sp|Q13439|GOGA4\_HUMAN sp|P78310|CXAR\_HUMAN tr|A0A087WTE7|A0A087WTE7\_HUMAN sp|Q96PV6|LENG8\_HUMAN tr|C9JMY0|C9JMY0\_HUMAN tr|A0A087WUE4|A0A087WUE4\_HUMAN tr|H0Y773|H0Y773\_HUMAN sp|Q3YEC7|RABL6\_HUMAN tr|D6RCC7|D6RCC7\_HUMAN tr|A0A087X128|A0A087X128\_HUMAN tr|H0Y6W5|H0Y6W5\_HUMAN tr|J3QKX5|J3QKX5\_HUMAN sp|Q16820|MEP1B\_HUMAN tr|D6R9J9|D6R9J9\_HUMAN tr|E7EM50|E7EM50\_HUMAN tr|D6RD39|D6RD39\_HUMAN sp|P13639|EF2\_HUMAN sp|O15083|ERC2\_HUMAN tr|F5H5C8|F5H5C8\_HUMAN tr|H0YDI0|H0YDI0\_HUMAN tr|A0A075B758|A0A075B758\_HUMAN sp|Q9Y4A8|NF2L3\_HUMAN tr|I3L3K8|I3L3K8\_HUMAN tr|F8VWK5|F8VWK5\_HUMAN tr|I3L266|I3L266\_HUMAN sp|P03372|ESR1\_HUMAN tr|A0A087X207|A0A087X207\_HUMAN sp|Q02930|CREB5\_HUMAN sp|Q9NUE0|ZDH18\_HUMAN tr|A0A087WXN4|A0A087WXN4\_HUMAN sp|Q96CB8|INT12\_HUMAN sp|Q5SSJ5|HP1B3\_HUMAN sp|Q9UKV3|ACINU\_HUMAN tr|G3XAL1|G3XAL1\_HUMAN tr|E7EQT4|E7EQT4\_HUMAN sp|O00411|RPOM\_HUMAN sp|P52756|RBM5\_HUMAN tr|I6L8A6|I6L8A6\_HUMAN tr|E9PNW1|E9PNW1\_HUMAN sp|Q8NC74|RB8NL\_HUMAN sp|Q15075|EEA1\_HUMAN sp|Q9UBF8|PI4KB\_HUMAN tr|E5RGQ4|E5RGQ4\_HUMAN sp|Q6ZVC0|NYAP1\_HUMAN tr|C9JS30|C9JS30\_HUMAN sp|Q9BX46|RBM24\_HUMAN tr|A0A087WVV7|A0A087WVV7\_HUMAN tr|A0A087X2I8|A0A087X2I8\_HUMAN tr|A0A087WTP8|A0A087WTP8\_HUMAN tr|A0A087X0S6|A0A087X0S6\_HUMAN tr|F2Z2Q7|F2Z2Q7\_HUMAN tr|H0YLB9|H0YLB9\_HUMAN sp|Q9BXT4|TDRD1\_HUMAN sp|Q4G0X9|CCD40\_HUMAN sp|O75746|CMC1\_HUMAN tr|Q9UJJ2|Q9UJJ2\_HUMAN sp|Q8ND82|Z280C\_HUMAN sp|P10912|GHR\_HUMAN tr|A0A087X0H5|A0A087X0H5\_HUMAN sp|Q15012|LAP4A\_HUMAN tr|B1AKC3|B1AKC3\_HUMAN sp|O60260|PRKN2\_HUMAN sp|Q13136|LIPA1\_HUMAN tr|J3KP20|J3KP20\_HUMAN tr|F5H578|F5H578\_HUMAN tr|H0YG38|H0YG38\_HUMAN tr|A0A087WYF2|A0A087WYF2\_HUMAN sp|Q8ND61|CC020\_HUMAN sp|Q9UQ84|EXO1\_HUMAN sp|Q9BXB1|LGR4\_HUMAN sp|Q5THR3|EFCB6\_HUMAN sp|Q15019|SEPT2\_HUMAN sp|Q9H2J7|S6A15\_HUMAN tr|K7ERF0|K7ERF0\_HUMAN tr|B4DGI9|B4DGI9\_HUMAN sp|Q99081|HTF4\_HUMAN sp|Q8N715|CC185\_HUMAN sp|Q96DY7|MTBP\_HUMAN tr|J3KNB8|J3KNB8\_HUMAN tr|F5H538|F5H538\_HUMAN tr|F6S6P2|F6S6P2\_HUMAN sp|Q9BZ29|DOCK9\_HUMAN sp|Q7Z7G0|TARSH\_HUMAN sp|Q96NB3|ZN830\_HUMAN sp|Q8NA69|CS045\_HUMAN sp|Q5H9K5|ZMAT1\_HUMAN sp|Q9Y3R4|NEUR2\_HUMAN sp|P32926|DSG3\_HUMAN tr|Q9P1I2|Q9P1I2\_HUMAN sp|Q9UBC0|HNF6\_HUMAN tr|G3V0I8|G3V0I8\_HUMAN tr|A0A087X1T2|A0A087X1T2\_HUMAN sp|Q99708|COM1\_HUMAN tr|F8VZQ2|F8VZQ2\_HUMAN A2VCT4 tr|H0YCX3|H0YCX3\_HUMAN sp|Q9HAQ2|KIF9\_HUMAN tr|F2Z395|F2Z395\_HUMAN sp|Q96LX7|CCD17\_HUMAN tr|H7BYF2|H7BYF2\_HUMAN tr|Q5H928|Q5H928\_HUMAN sp|Q99714|HCD2\_HUMAN sp|Q9UMQ6|CAN11\_HUMAN sp|Q6P4F7|RHGBA\_HUMAN sp|Q01813|PFKAP\_HUMAN tr|F5GWL3|F5GWL3\_HUMAN sp|Q08AH3|ACS2A\_HUMAN sp|Q16827|PTPRO\_HUMAN sp|Q8NG04|S2610\_HUMAN sp|A2A2Z9|AN18B\_HUMAN sp|Q12772|SRBP2\_HUMAN tr|H7BZV0|H7BZV0\_HUMAN tr|A0A087WZF1|A0A087WZF1\_HUMAN sp|Q93052|LPP\_HUMAN tr|C9JUT4|C9JUT4\_HUMAN sp|P26358|DNMT1\_HUMAN tr|U3KPS2|U3KPS2\_HUMAN sp|P24158|PRTN3\_HUMAN sp|Q96JP0|FEM1C\_HUMAN sp|Q6ZMD2|SPNS3\_HUMAN tr|K7ES14|K7ES14\_HUMAN tr|B4DFI3|B4DFI3\_HUMAN sp|P02794|FRIH\_HUMAN tr|C9JJY0|C9JJY0\_HUMAN tr|B4DGD8|B4DGD8\_HUMAN tr|H7C2P2|H7C2P2\_HUMAN sp|Q9BZF3|OSBL6\_HUMAN sp|O15315|RA51B\_HUMAN tr|V9GZ57|V9GZ57\_HUMAN sp|P55285|CADH6\_HUMAN tr|D6RF86|D6RF86\_HUMAN tr|C9JMN1|C9JMN1\_HUMAN tr|E9PC69|E9PC69\_HUMAN tr|E7EME3|E7EME3\_HUMAN sp|Q9HBX8|LGR6\_HUMAN sp|P83436|COG7\_HUMAN sp|Q9UN76|S6A14\_HUMAN tr|I3L3M2|I3L3M2\_HUMAN tr|G5E9K5|G5E9K5\_HUMAN sp|Q7Z2D5|LPPR4\_HUMAN sp|Q9Y251|HPSE\_HUMAN tr|E9PI01|E9PI01\_HUMAN sp|Q9BTC0|DIDO1\_HUMAN tr|F8WEV5|F8WEV5\_HUMAN tr|A0A087X2J1|A0A087X2J1\_HUMAN tr|K7ESE9|K7ESE9\_HUMAN sp|Q9H6U6|BCAS3\_HUMAN tr|H0YKQ2|H0YKQ2\_HUMAN sp|O14543|SOCS3\_HUMAN tr|H0YFX0|H0YFX0\_HUMAN sp|Q8IYM0|F186B\_HUMAN tr|A0A087WV18|A0A087WV18\_HUMAN tr|D6RCC2|D6RCC2\_HUMAN sp|Q9UBU2|DKK2\_HUMAN tr|D6RGF1|D6RGF1\_HUMAN sp|Q6IS24|GLTL3\_HUMAN Q3ZBS7 tr|F8WBQ1|F8WBQ1\_HUMAN sp|Q8NDH6|ICA1L\_HUMAN sp|Q8IWY4|SCUB1\_HUMAN sp|P22033|MUTA\_HUMAN sp|Q9NQW6|ANLN\_HUMAN tr|H0YLI3|H0YLI3\_HUMAN sp|Q8NEY4|VATC2\_HUMAN sp|Q15619|OR1C1\_HUMAN sp|Q9UHD2|TBK1\_HUMAN sp|Q9HD23|MRS2\_HUMAN sp|Q68DD2|PA24F\_HUMAN sp|Q9HAW4|CLSPN\_HUMAN sp|Q15003|CND2\_HUMAN sp|Q7Z6J0|SH3R1\_HUMAN sp|P55291|CAD15\_HUMAN tr|E5RFN6|E5RFN6\_HUMAN sp|P48995|TRPC1\_HUMAN tr|J3KQL8|J3KQL8\_HUMAN sp|Q8N0W3|FUK\_HUMAN sp|Q15276|RABE1\_HUMAN sp|Q9NRW7|VPS45\_HUMAN tr|H7BXQ2|H7BXQ2\_HUMAN sp|Q13416|ORC2\_HUMAN sp|Q6NT76|HMBX1\_HUMAN sp|Q4ZJI4|SL9B1\_HUMAN tr|F8W7U0|F8W7U0\_HUMAN sp|Q5JY77|GASP1\_HUMAN sp|Q12946|FOXF1\_HUMAN tr|C9JNM8|C9JNM8\_HUMAN tr|C9JE25|C9JE25\_HUMAN sp|Q5VV43|K0319\_HUMAN tr|B4DPG6|B4DPG6\_HUMAN sp|Q6IQ26|DEN5A\_HUMAN sp|Q86TD4|SRCA\_HUMAN tr|J3KSG2|J3KSG2\_HUMAN sp|Q9H0R4|HDHD2\_HUMAN tr|K7ER15|K7ER15\_HUMAN sp|Q8NGJ1|OR4D6\_HUMAN tr|F8W8M9|F8W8M9\_HUMAN tr|I6L9J0|I6L9J0\_HUMAN sp|O75054|IGSF3\_HUMAN Q9TRI1 sp|Q04864|REL\_HUMAN tr|F8VXG0|F8VXG0\_HUMAN sp|P78504|JAG1\_HUMAN sp|P53708|ITA8\_HUMAN sp|Q08174|PCDH1\_HUMAN sp|Q9NVE5|UBP40\_HUMAN sp|Q96NY7|CLIC6\_HUMAN sp|Q76KD6|SPERI\_HUMAN tr|J3QQJ7|J3QQJ7\_HUMAN sp|O60725|ICMT\_HUMAN tr|K7ER94|K7ER94\_HUMAN sp|O95248|MTMR5\_HUMAN tr|G5E933|G5E933\_HUMAN tr|E9PB58|E9PB58\_HUMAN sp|Q6UXF1|TM108\_HUMAN sp|Q9BYG0|B3GN5\_HUMAN sp|Q16280|CNGA2\_HUMAN sp|O00716|E2F3\_HUMAN tr|C9J1X3|C9J1X3\_HUMAN tr|Q7Z3A3|Q7Z3A3\_HUMAN sp|O94910|LPHN1\_HUMAN tr|F8WF14|F8WF14\_HUMAN sp|Q9H1X3|DJC25\_HUMAN sp|P06276|CHLE\_HUMAN tr|F8WEX7|F8WEX7\_HUMAN sp|Q14392|LRC32\_HUMAN sp|Q3KNW5|SOAT\_HUMAN tr|G3V482|G3V482\_HUMAN tr|G3V2M1|G3V2M1\_HUMAN tr|G3V264|G3V264\_HUMAN tr|G3V265|G3V265\_HUMAN tr|M0R201|M0R201\_HUMAN sp|O75140|DEPD5\_HUMAN sp|Q96DP5|FMT\_HUMAN tr|J3QQY0|J3QQY0\_HUMAN tr|A0A087WVA0|A0A087WVA0\_HUMAN tr|A0A087WUH2|A0A087WUH2\_HUMAN tr|A0A087WVC7|A0A087WVC7\_HUMAN tr|A0A087X288|A0A087X288\_HUMAN tr|E9PJR8|E9PJR8\_HUMAN sp|Q8WYP3|RIN2\_HUMAN tr|C9JD37|C9JD37\_HUMAN sp|Q9UJX2|CDC23\_HUMAN sp|Q8NCU4|K1407\_HUMAN sp|Q9Y6A5|TACC3\_HUMAN tr|Q5JRM6|Q5JRM6\_HUMAN sp|A0AVI2|FR1L5\_HUMAN tr|A0A096LPB1|A0A096LPB1\_HUMAN sp|Q06587|RING1\_HUMAN tr|H0Y4P6|H0Y4P6\_HUMAN tr|H0Y7Y3|H0Y7Y3\_HUMAN sp|Q99952|PTN18\_HUMAN tr|H0Y472|H0Y472\_HUMAN tr|F5H070|F5H070\_HUMAN sp|Q9Y4C1|KDM3A\_HUMAN sp|Q6NUS8|UD3A1\_HUMAN sp|Q9Y4J8|DTNA\_HUMAN tr|A0A087X1M0|A0A087X1M0\_HUMAN sp|Q9BQI6|ANR32\_HUMAN Q58D62 tr|H7C2F5|H7C2F5\_HUMAN tr|H0Y3K4|H0Y3K4\_HUMAN sp|O14654|IRS4\_HUMAN sp|Q06418|TYRO3\_HUMAN sp|Q5JSJ4|DX26B\_HUMAN tr|H0Y2M0|H0Y2M0\_HUMAN sp|B2CW77|KILIN\_HUMAN sp|Q6P280|ZN529\_HUMAN tr|E7EUL6|E7EUL6\_HUMAN tr|E7EQ57|E7EQ57\_HUMAN sp|Q03112|EVI1\_HUMAN sp|Q68CZ2|TENS3\_HUMAN sp|Q6ZMY3|SPOC1\_HUMAN tr|H0YG46|H0YG46\_HUMAN tr|H0YBZ4|H0YBZ4\_HUMAN sp|Q9HBE5|IL21R\_HUMAN sp|Q6JEL2|KLH10\_HUMAN sp|Q9UQ05|KCNH4\_HUMAN sp|O94933|SLIK3\_HUMAN sp|Q9NRA0|SPHK2\_HUMAN tr|M0R344|M0R344\_HUMAN tr|A4D0Q3|A4D0Q3\_HUMAN sp|Q9Y2W7|CSEN\_HUMAN sp|Q9UKN5|PRDM4\_HUMAN sp|Q96JQ5|M4A4A\_HUMAN sp|Q92556|ELMO1\_HUMAN sp|Q9Y426|CU025\_HUMAN sp|O75635|SPB7\_HUMAN tr|E9PMP7|E9PMP7\_HUMAN sp|Q8IYT8|ULK2\_HUMAN sp|Q96J65|MRP9\_HUMAN tr|A0A087WZL9|A0A087WZL9\_HUMAN sp|Q13183|S13A2\_HUMAN tr|F8WAD8|F8WAD8\_HUMAN sp|Q9P0K1|ADA22\_HUMAN sp|Q96KN7|RPGR1\_HUMAN tr|F5H2F4|F5H2F4\_HUMAN sp|Q14050|CO9A3\_HUMAN sp|Q63HM2|PCX4\_HUMAN sp|P30953|OR1E1\_HUMAN sp|O95427|PIGN\_HUMAN sp|Q12962|TAF10\_HUMAN sp|Q8IVH4|MMAA\_HUMAN tr|Q495G5|Q495G5\_HUMAN tr|Q5W0W1|Q5W0W1\_HUMAN sp|Q9Y6N9|USH1C\_HUMAN sp|P85037|FOXK1\_HUMAN sp|Q8NGB6|OR4M2\_HUMAN tr|B3KR06|B3KR06\_HUMAN tr|E7ENG2|E7ENG2\_HUMAN sp|Q15572|TAF1C\_HUMAN tr|H3BR29|H3BR29\_HUMAN sp|Q9UJA3|MCM8\_HUMAN sp|P07864|LDHC\_HUMAN tr|F5H245|F5H245\_HUMAN tr|A0A087X0E6|A0A087X0E6\_HUMAN sp|Q9Y4X4|KLF12\_HUMAN tr|B5MEG9|B5MEG9\_HUMAN sp|Q2M1V0|ISX\_HUMAN sp|Q9BYI3|HYCCI\_HUMAN sp|O60706|ABCC9\_HUMAN tr|K7EIU8|K7EIU8\_HUMAN tr|Q96GL6|Q96GL6\_HUMAN sp|Q9GZY8|MFF\_HUMAN sp|Q9HBR0|S38AA\_HUMAN tr|A0A087X0B6|A0A087X0B6\_HUMAN sp|Q8N8Q9|NIPA2\_HUMAN tr|F6XU50|F6XU50\_HUMAN sp|Q9HAR2|LPHN3\_HUMAN sp|Q9NRZ9|HELLS\_HUMAN sp|A0AV02|S12A8\_HUMAN sp|O14976|GAK\_HUMAN sp|Q5JU85|IQEC2\_HUMAN sp|Q9NS87|KIF15\_HUMAN tr|H0YC42|H0YC42\_HUMAN tr|F5H0B0|F5H0B0\_HUMAN tr|E9PB90|E9PB90\_HUMAN sp|P52789|HXK2\_HUMAN tr|J3KNP2|J3KNP2\_HUMAN sp|Q58WW2|DCAF6\_HUMAN tr|H0YGZ1|H0YGZ1\_HUMAN sp|Q9Y6X9|MORC2\_HUMAN sp|Q12834|CDC20\_HUMAN sp|Q8N2N9|AN36B\_HUMAN sp|Q9H0X4|ITFG3\_HUMAN tr|B4DGG1|B4DGG1\_HUMAN tr|H0Y2L7|H0Y2L7\_HUMAN sp|Q9UL51|HCN2\_HUMAN sp|P41970|ELK3\_HUMAN sp|Q2T9J0|TYSD1\_HUMAN sp|O15117|FYB\_HUMAN tr|F8WBA3|F8WBA3\_HUMAN sp|Q9H3D4|P63\_HUMAN sp|P16435|NCPR\_HUMAN sp|Q8TD17|ZN398\_HUMAN sp|P13667|PDIA4\_HUMAN sp|Q9Y5F1|PCDBC\_HUMAN sp|Q86YR5|GPSM1\_HUMAN sp|O94913|PCF11\_HUMAN sp|A6NEK1|ARRD5\_HUMAN sp|Q96PN6|ADCYA\_HUMAN sp|Q9UPP2|IQEC3\_HUMAN sp|Q9Y5X2|SNX8\_HUMAN sp|Q8WY07|CTR3\_HUMAN sp|Q5SRE5|NU188\_HUMAN tr|K7EQU2|K7EQU2\_HUMAN sp|O00370|LORF2\_HUMAN tr|Q5JQ13|Q5JQ13\_HUMAN sp|Q9NW82|WDR70\_HUMAN sp|Q8NCA5|FA98A\_HUMAN sp|Q9ULQ1|TPC1\_HUMAN sp|Q8N6Q8|MET25\_HUMAN tr|H3BPF3|H3BPF3\_HUMAN sp|P54707|AT12A\_HUMAN tr|F8W9U4|F8W9U4\_HUMAN tr|H0Y765|H0Y765\_HUMAN sp|Q8TF62|AT8B4\_HUMAN sp|Q5VUJ6|LRCH2\_HUMAN sp|Q7Z3E1|PARPT\_HUMAN tr|G5E9W1|G5E9W1\_HUMAN sp|Q9HC62|SENP2\_HUMAN sp|Q86SP6|GP149\_HUMAN sp|Q9NRR5|UBQL4\_HUMAN tr|H0Y2M6|H0Y2M6\_HUMAN sp|O00409|FOXN3\_HUMAN sp|Q13796|SHRM2\_HUMAN sp|Q9UHR4|BI2L1\_HUMAN tr|M0R0P8|M0R0P8\_HUMAN tr|C9JYE9|C9JYE9\_HUMAN sp|Q13459|MYO9B\_HUMAN tr|H7C4Q5|H7C4Q5\_HUMAN tr|H0YIS3|H0YIS3\_HUMAN tr|H0YCW2|H0YCW2\_HUMAN sp|Q8N0S6|CENPL\_HUMAN sp|O60890|OPHN1\_HUMAN sp|Q8NBP5|MFSD9\_HUMAN tr|I3L354|I3L354\_HUMAN sp|Q6EBC2|IL31\_HUMAN tr|A2A2G4|A2A2G4\_HUMAN tr|A0A087WU36|A0A087WU36\_HUMAN tr|F5GZP6|F5GZP6\_HUMAN tr|C9IYJ2|C9IYJ2\_HUMAN tr|F8WDF7|F8WDF7\_HUMAN sp|Q5VYP0|S31A3\_HUMAN tr|H0YLA6|H0YLA6\_HUMAN sp|Q7Z7L7|ZER1\_HUMAN sp|A6NNA5|DRGX\_HUMAN sp|Q9UK80|UBP21\_HUMAN tr|Q2T9J9|Q2T9J9\_HUMAN tr|U3KQT1|U3KQT1\_HUMAN tr|X6RA14|X6RA14\_HUMAN sp|P10768|ESTD\_HUMAN sp|Q8NCA9|ZN784\_HUMAN sp|Q1HG43|DOXA1\_HUMAN tr|H0Y650|H0Y650\_HUMAN sp|O94898|LRIG2\_HUMAN tr|A0A087WUK6|A0A087WUK6\_HUMAN tr|E9PGY2|E9PGY2\_HUMAN sp|Q96JP5|ZFP91\_HUMAN tr|A0A075B6G6|A0A075B6G6\_HUMAN sp|O75342|LX12B\_HUMAN sp|P21675|TAF1\_HUMAN sp|Q9H2G2|SLK\_HUMAN sp|Q13422|IKZF1\_HUMAN tr|A0A087WU46|A0A087WU46\_HUMAN sp|Q9Y3P9|RBGP1\_HUMAN tr|E7EQ45|E7EQ45\_HUMAN sp|Q9H0R1|AP5M1\_HUMAN sp|Q8TF30|WHAMM\_HUMAN sp|A5PKW4|PSD1\_HUMAN sp|Q5TZ20|OR2G6\_HUMAN tr|A0A087WWM1|A0A087WWM1\_HUMAN sp|Q7LBR1|CHM1B\_HUMAN sp|Q9H165|BC11A\_HUMAN sp|P08107|HSP71\_HUMAN tr|E7ERX9|E7ERX9\_HUMAN sp|Q9ULH0|KDIS\_HUMAN tr|H0Y494|H0Y494\_HUMAN tr|M0QZB5|M0QZB5\_HUMAN sp|Q15173|2A5B\_HUMAN tr|H0YEX4|H0YEX4\_HUMAN sp|Q8NFT6|DBF4B\_HUMAN sp|P38398|BRCA1\_HUMAN tr|E9PFZ0|E9PFZ0\_HUMAN tr|H7BY58|H7BY58\_HUMAN tr|E7EMR3|E7EMR3\_HUMAN tr|F6S8N6|F6S8N6\_HUMAN tr|J3KN18|J3KN18\_HUMAN tr|Q8IVM9|Q8IVM9\_HUMAN tr|H0YBM6|H0YBM6\_HUMAN sp|Q14587|ZN268\_HUMAN sp|Q92540|SMG7\_HUMAN tr|J3KQL4|J3KQL4\_HUMAN sp|Q5T6X5|GPC6A\_HUMAN tr|X6R7H2|X6R7H2\_HUMAN tr|G8JLI8|G8JLI8\_HUMAN tr|K7EQI4|K7EQI4\_HUMAN sp|Q13061|TRDN\_HUMAN tr|Q5SWK9|Q5SWK9\_HUMAN sp|Q96QP1|ALPK1\_HUMAN sp|Q9NY46|SCN3A\_HUMAN tr|A0A087WWA5|A0A087WWA5\_HUMAN sp|O15067|PUR4\_HUMAN sp|Q12788|TBL3\_HUMAN tr|A0A087WYP7|A0A087WYP7\_HUMAN sp|O14827|RGRF2\_HUMAN sp|Q7Z692|CEA19\_HUMAN sp|Q9UIU6|SIX4\_HUMAN sp|Q96I51|WBS16\_HUMAN sp|Q2M3T9|HYAL4\_HUMAN tr|A0A087X241|A0A087X241\_HUMAN sp|Q2NKQ1|SGSM1\_HUMAN sp|O75182|SIN3B\_HUMAN sp|Q08999|RBL2\_HUMAN sp|Q96HA1|P121A\_HUMAN sp|Q8NEC5|CTSR1\_HUMAN tr|C9JDI8|C9JDI8\_HUMAN tr|C9JYP5|C9JYP5\_HUMAN sp|O75916|RGS9\_HUMAN tr|E9PD91|E9PD91\_HUMAN tr|A0A087X059|A0A087X059\_HUMAN sp|Q9BYV9|BACH2\_HUMAN tr|Q5YLB2|Q5YLB2\_HUMAN tr|E9PNM3|E9PNM3\_HUMAN tr|A0A087WU44|A0A087WU44\_HUMAN sp|Q13045|FLII\_HUMAN sp|P98169|ZXDB\_HUMAN sp|A6NJY1|SL9P1\_HUMAN sp|Q15329|E2F5\_HUMAN sp|P28358|HXD10\_HUMAN tr|H3BSM7|H3BSM7\_HUMAN sp|Q86XM0|CTSRD\_HUMAN sp|Q86UK5|LBN\_HUMAN sp|Q96I34|PP16A\_HUMAN sp|Q5VT97|SYDE2\_HUMAN sp|O94901|SUN1\_HUMAN sp|Q9P2E3|ZNFX1\_HUMAN sp|Q8NFW9|MYRIP\_HUMAN tr|E9PHI4|E9PHI4\_HUMAN sp|P15884|ITF2\_HUMAN tr|H3BPJ7|H3BPJ7\_HUMAN tr|H3BTP3|H3BTP3\_HUMAN tr|E9PH57|E9PH57\_HUMAN sp|Q86YJ7|AN13B\_HUMAN tr|H0YLB8|H0YLB8\_HUMAN sp|Q969H4|CNKR1\_HUMAN sp|Q04917|1433F\_HUMAN sp|Q13495|MAMD1\_HUMAN sp|O95470|SGPL1\_HUMAN tr|H0Y5I7|H0Y5I7\_HUMAN sp|Q8NC42|RN149\_HUMAN sp|Q9UHC3|ASIC3\_HUMAN sp|O43156|TTI1\_HUMAN tr|F8W1T6|F8W1T6\_HUMAN sp|P00395|COX1\_HUMAN tr|F8W8C2|F8W8C2\_HUMAN sp|Q8NH48|OR5B3\_HUMAN sp|Q17R89|RHG44\_HUMAN sp|Q96MC2|DRC1\_HUMAN sp|O75533|SF3B1\_HUMAN sp|Q15147|PLCB4\_HUMAN tr|B4DEW2|B4DEW2\_HUMAN sp|Q8NGQ2|OR6Q1\_HUMAN sp|Q9BXM0|PRAX\_HUMAN tr|H0YNB1|H0YNB1\_HUMAN sp|Q96F05|CK024\_HUMAN sp|Q6ULP2|AFTIN\_HUMAN sp|O75330|HMMR\_HUMAN sp|Q8ND83|SLAI1\_HUMAN sp|Q9Y3M2|CBY1\_HUMAN tr|E9PRI4|E9PRI4\_HUMAN tr|E9PGT3|E9PGT3\_HUMAN tr|A0A087WUW0|A0A087WUW0\_HUMAN sp|Q15418|KS6A1\_HUMAN sp|O15438|MRP3\_HUMAN tr|E7EUT5|E7EUT5\_HUMAN sp|P04406|G3P\_HUMAN sp|O14662|STX16\_HUMAN tr|Q96NX8|Q96NX8\_HUMAN tr|F8W9Z6|F8W9Z6\_HUMAN sp|P78562|PHEX\_HUMAN sp|Q9BV73|CP250\_HUMAN sp|Q8NI27|THOC2\_HUMAN sp|Q6XPR3|RPTN\_HUMAN sp|Q9NSI5|IGSF5\_HUMAN tr|A0A087X1W0|A0A087X1W0\_HUMAN sp|O60524|NEMF\_HUMAN tr|G3V5V3|G3V5V3\_HUMAN sp|P03905|NU4M\_HUMAN sp|P00558|PGK1\_HUMAN tr|E9PEZ1|E9PEZ1\_HUMAN sp|Q8IWT3|CUL9\_HUMAN sp|P16234|PGFRA\_HUMAN tr|E7ESZ3|E7ESZ3\_HUMAN sp|Q9UQR1|ZN148\_HUMAN sp|Q96RQ3|MCCA\_HUMAN tr|G5E965|G5E965\_HUMAN sp|Q6UWL2|SUSD1\_HUMAN tr|A0A087X0I1|A0A087X0I1\_HUMAN sp|O76064|RNF8\_HUMAN tr|A0A087WTZ0|A0A087WTZ0\_HUMAN tr|H7C3P0|H7C3P0\_HUMAN sp|Q71RC2|LARP4\_HUMAN sp|Q04759|KPCT\_HUMAN tr|A0A087X0I9|A0A087X0I9\_HUMAN sp|Q5SXM2|SNPC4\_HUMAN sp|Q8TAP6|CEP76\_HUMAN tr|H3BRM4|H3BRM4\_HUMAN sp|P51168|SCNNB\_HUMAN sp|Q86Y38|XYLT1\_HUMAN tr|H0YEP9|H0YEP9\_HUMAN sp|Q75QN2|INT8\_HUMAN sp|Q86SJ6|DSG4\_HUMAN sp|Q9NPE6|SPAG4\_HUMAN sp|Q6ZRI6|CO039\_HUMAN sp|Q92543|SNX19\_HUMAN tr|E9PBG7|E9PBG7\_HUMAN sp|Q13557|KCC2D\_HUMAN tr|E9PF82|E9PF82\_HUMAN sp|C4AMC7|WASH3\_HUMAN tr|E7EQL2|E7EQL2\_HUMAN sp|Q9H720|PG2IP\_HUMAN tr|F5H0W4|F5H0W4\_HUMAN sp|Q9UBW5|BIN2\_HUMAN tr|A0A087X188|A0A087X188\_HUMAN tr|H0Y7S3|H0Y7S3\_HUMAN sp|Q8NBJ5|GT251\_HUMAN sp|Q9Y6M4|KC1G3\_HUMAN tr|C9IYI1|C9IYI1\_HUMAN sp|A7MBM2|DISP2\_HUMAN tr|F5GYK7|F5GYK7\_HUMAN sp|Q5SYB0|FRPD1\_HUMAN sp|Q6ZRV2|FA83H\_HUMAN sp|Q9Y5H6|PCDA8\_HUMAN sp|Q8NGQ3|OR1S2\_HUMAN tr|A0A087X2C0|A0A087X2C0\_HUMAN sp|Q9UL68|MYT1L\_HUMAN tr|A0A087WYJ9|A0A087WYJ9\_HUMAN sp|A2A3N6|PIPSL\_HUMAN sp|Q8WYK1|CNTP5\_HUMAN tr|K7EP52|K7EP52\_HUMAN sp|Q9BY15|EMR3\_HUMAN tr|H0Y599|H0Y599\_HUMAN tr|E9PDG3|E9PDG3\_HUMAN sp|P23246|SFPQ\_HUMAN sp|Q14973|NTCP\_HUMAN tr|H0YBG7|H0YBG7\_HUMAN tr|E7ERL6|E7ERL6\_HUMAN tr|I3L443|I3L443\_HUMAN tr|B9EGE7|B9EGE7\_HUMAN sp|Q8IV53|DEN1C\_HUMAN sp|Q8TCG5|CPT1C\_HUMAN sp|Q8TCB6|O51E1\_HUMAN tr|F2Z2J7|F2Z2J7\_HUMAN tr|H7BXT4|H7BXT4\_HUMAN sp|Q8NGG3|OR5T3\_HUMAN tr|K7EPM3|K7EPM3\_HUMAN sp|O15016|TRI66\_HUMAN sp|Q9UEF7|KLOT\_HUMAN tr|A0A087WU39|A0A087WU39\_HUMAN tr|B7WPL9|B7WPL9\_HUMAN tr|H0YNH0|H0YNH0\_HUMAN tr|F8W9A8|F8W9A8\_HUMAN sp|Q6PFW1|VIP1\_HUMAN sp|O60502|OGA\_HUMAN sp|O75071|EFC14\_HUMAN tr|A0A087WWF5|A0A087WWF5\_HUMAN sp|O60759|CYTIP\_HUMAN sp|P23508|CRCM\_HUMAN sp|Q9Y615|ACL7A\_HUMAN sp|Q9Y263|PLAP\_HUMAN sp|Q9Y2N7|HIF3A\_HUMAN sp|Q7KZF4|SND1\_HUMAN sp|Q9UK41|VPS28\_HUMAN sp|Q92562|FIG4\_HUMAN sp|Q14999|CUL7\_HUMAN sp|Q9Y4B5|MTCL1\_HUMAN tr|H7BZ52|H7BZ52\_HUMAN tr|C9JDV3|C9JDV3\_HUMAN tr|C9IZN2|C9IZN2\_HUMAN sp|Q9UGH3|S23A2\_HUMAN sp|Q15468|STIL\_HUMAN sp|Q9Y3T9|NOC2L\_HUMAN tr|E9PSF2|E9PSF2\_HUMAN sp|O43719|HTSF1\_HUMAN sp|Q53EL9|SEZ6\_HUMAN sp|Q19T08|ECSCR\_HUMAN Q2KIG3 sp|Q9UPN6|SCAF8\_HUMAN sp|P26639|SYTC\_HUMAN tr|C9JZG1|C9JZG1\_HUMAN sp|Q5T699|CF183\_HUMAN tr|D6RAS9|D6RAS9\_HUMAN tr|F5GZ18|F5GZ18\_HUMAN sp|Q12791|KCMA1\_HUMAN tr|Q5SVJ8|Q5SVJ8\_HUMAN sp|P08173|ACM4\_HUMAN tr|Q5SVJ7|Q5SVJ7\_HUMAN sp|Q8N1T3|MYO1H\_HUMAN sp|A8MUU9|YV023\_HUMAN tr|F8VRN8|F8VRN8\_HUMAN sp|P43026|GDF5\_HUMAN sp|P55795|HNRH2\_HUMAN tr|F8VZA8|F8VZA8\_HUMAN sp|Q8IVT5|KSR1\_HUMAN sp|Q8IYU4|UBQLN\_HUMAN sp|O75665|OFD1\_HUMAN tr|E7ERS3|E7ERS3\_HUMAN sp|O94818|NOL4\_HUMAN sp|Q9UM01|YLAT1\_HUMAN sp|O75473|LGR5\_HUMAN sp|O14981|BTAF1\_HUMAN tr|H7C3P6|H7C3P6\_HUMAN tr|D3YTK1|D3YTK1\_HUMAN sp|Q9C0K0|BC11B\_HUMAN sp|P15291|B4GT1\_HUMAN sp|Q9C0E2|XPO4\_HUMAN tr|F2Z2X4|F2Z2X4\_HUMAN sp|P29374|ARI4A\_HUMAN tr|M0QY43|M0QY43\_HUMAN tr|I3L399|I3L399\_HUMAN sp|P05543|THBG\_HUMAN sp|Q8IZU8|DSEL\_HUMAN tr|F5H562|F5H562\_HUMAN sp|Q9UF56|FXL17\_HUMAN sp|Q9HAP2|MLXIP\_HUMAN sp|Q8N7J2|AMER2\_HUMAN tr|I3L104|I3L104\_HUMAN sp|Q53FA7|QORX\_HUMAN sp|Q7Z6V5|ADAT2\_HUMAN tr|C9JVJ0|C9JVJ0\_HUMAN tr|I3NI51|I3NI51\_HUMAN tr|I3L2V7|I3L2V7\_HUMAN sp|Q8N7C7|RN148\_HUMAN tr|H7BZ78|H7BZ78\_HUMAN tr|H7BZH6|H7BZH6\_HUMAN tr|I3L400|I3L400\_HUMAN tr|A0A087X1I6|A0A087X1I6\_HUMAN tr|B1APN9|B1APN9\_HUMAN sp|Q9UHB9|SRP68\_HUMAN tr|C9JER5|C9JER5\_HUMAN tr|E9PS95|E9PS95\_HUMAN sp|Q9H936|GHC1\_HUMAN sp|Q9UPT8|ZC3H4\_HUMAN tr|E9PJH7|E9PJH7\_HUMAN tr|F6SPX6|F6SPX6\_HUMAN tr|E9PNB6|E9PNB6\_HUMAN tr|A0A087WWW8|A0A087WWW8\_HUMAN sp|Q9P107|GMIP\_HUMAN sp|Q13813|SPTN1\_HUMAN sp|P54289|CA2D1\_HUMAN sp|P63244|GBLP\_HUMAN tr|D6RHH4|D6RHH4\_HUMAN tr|D6RBD0|D6RBD0\_HUMAN tr|D6REE5|D6REE5\_HUMAN tr|D6RAC2|D6RAC2\_HUMAN tr|H7C3N6|H7C3N6\_HUMAN sp|Q9NW15|ANO10\_HUMAN tr|G0XQ39|G0XQ39\_HUMAN tr|H3BLT0|H3BLT0\_HUMAN sp|Q9BZ72|PITM2\_HUMAN sp|Q99650|OSMR\_HUMAN sp|Q9Y5H1|PCDG2\_HUMAN tr|F8WDV0|F8WDV0\_HUMAN sp|Q8IWK6|GP125\_HUMAN tr|A0A096LPK7|A0A096LPK7\_HUMAN sp|Q9NNW7|TRXR2\_HUMAN sp|Q9P2G4|MAP10\_HUMAN sp|Q96NG3|TTC25\_HUMAN sp|Q8IYY4|DZI1L\_HUMAN tr|A0A087WYN4|A0A087WYN4\_HUMAN sp|P0DKV0|S31C1\_HUMAN tr|F5H586|F5H586\_HUMAN sp|Q8TBP0|TBC16\_HUMAN tr|F8WE85|F8WE85\_HUMAN sp|Q8IWB7|WDFY1\_HUMAN tr|H0YHS3|H0YHS3\_HUMAN sp|Q5TB30|DEP1A\_HUMAN tr|E7ESS2|E7ESS2\_HUMAN sp|P04440|DPB1\_HUMAN tr|E7EN19|E7EN19\_HUMAN sp|Q7Z4N8|P4HA3\_HUMAN sp|P43004|EAA2\_HUMAN sp|Q9NTG1|PKDRE\_HUMAN tr|F8W7L6|F8W7L6\_HUMAN sp|P42262|GRIA2\_HUMAN sp|Q9H158|PCDC1\_HUMAN sp|Q9ULL1|PKHG1\_HUMAN sp|Q92544|TM9S4\_HUMAN sp|A6NMB9|FIGL2\_HUMAN sp|Q6UX68|XKR5\_HUMAN tr|H0YMU7|H0YMU7\_HUMAN sp|Q15139|KPCD1\_HUMAN sp|Q9Y5R5|DMRT2\_HUMAN tr|A0A087X0K6|A0A087X0K6\_HUMAN sp|P49366|DHYS\_HUMAN tr|Q5J8M5|Q5J8M5\_HUMAN tr|A0A087WZK0|A0A087WZK0\_HUMAN sp|Q10570|CPSF1\_HUMAN sp|Q8NEE8|TTC16\_HUMAN sp|Q99436|PSB7\_HUMAN tr|D3YTD1|D3YTD1\_HUMAN sp|Q96QS1|TSN32\_HUMAN sp|P23921|RIR1\_HUMAN tr|X6R6S3|X6R6S3\_HUMAN sp|Q8WX94|NALP7\_HUMAN tr|K7ERG0|K7ERG0\_HUMAN sp|Q9BWL3|CA043\_HUMAN tr|A0A087WTD7|A0A087WTD7\_HUMAN tr|A8MYJ1|A8MYJ1\_HUMAN sp|P04843|RPN1\_HUMAN tr|H0YCK3|H0YCK3\_HUMAN tr|B0QZ65|B0QZ65\_HUMAN sp|Q96JE7|SC16B\_HUMAN sp|Q9HD34|LYRM4\_HUMAN tr|C9JY28|C9JY28\_HUMAN tr|F5H189|F5H189\_HUMAN tr|C9JRX8|C9JRX8\_HUMAN sp|P46934|NEDD4\_HUMAN sp|Q9BZC1|CELF4\_HUMAN tr|M0QY66|M0QY66\_HUMAN tr|X6R5W5|X6R5W5\_HUMAN tr|S4R3V5|S4R3V5\_HUMAN tr|A0A087WUH0|A0A087WUH0\_HUMAN sp|P28715|ERCC5\_HUMAN tr|G3XAI2|G3XAI2\_HUMAN sp|P07942|LAMB1\_HUMAN sp|Q9NP60|IRPL2\_HUMAN sp|Q8TF05|PP4R1\_HUMAN sp|O60784|TOM1\_HUMAN sp|Q969M3|YIPF5\_HUMAN tr|I3L3Y9|I3L3Y9\_HUMAN tr|A0A087WU15|A0A087WU15\_HUMAN tr|H0YD97|H0YD97\_HUMAN sp|Q9NQW8|CNGB3\_HUMAN sp|Q8IVJ8|APRG1\_HUMAN tr|Q9BY48|Q9BY48\_HUMAN tr|F8WCD0|F8WCD0\_HUMAN sp|Q9NYK1|TLR7\_HUMAN sp|Q495M3|S36A2\_HUMAN tr|E5RJJ5|E5RJJ5\_HUMAN tr|W0Z7M9|W0Z7M9\_HUMAN sp|Q96Q91|B3A4\_HUMAN sp|O75094|SLIT3\_HUMAN sp|Q9UJ98|STAG3\_HUMAN tr|D6W5U7|D6W5U7\_HUMAN tr|Q68CX2|Q68CX2\_HUMAN sp|P48643|TCPE\_HUMAN sp|Q9UHX1|PUF60\_HUMAN sp|Q5TIE3|VW5B1\_HUMAN tr|F2Z2J1|F2Z2J1\_HUMAN tr|F8VZN8|F8VZN8\_HUMAN sp|P30260|CDC27\_HUMAN sp|A5PL33|KRBA1\_HUMAN sp|Q2VWA4|SKOR2\_HUMAN tr|K7EPU6|K7EPU6\_HUMAN sp|P21439|MDR3\_HUMAN tr|F5GZY3|F5GZY3\_HUMAN tr|E7EQS5|E7EQS5\_HUMAN tr|E7EQR9|E7EQR9\_HUMAN tr|H7C222|H7C222\_HUMAN sp|Q5T8D3|ACBD5\_HUMAN sp|Q9Y252|RNF6\_HUMAN tr|A0A087WUB9|A0A087WUB9\_HUMAN tr|A0A087WUY3|A0A087WUY3\_HUMAN sp|P51798|CLCN7\_HUMAN tr|H7C566|H7C566\_HUMAN sp|Q9Y6V7|DDX49\_HUMAN sp|P55060|XPO2\_HUMAN sp|Q9BYJ9|YTHD1\_HUMAN sp|Q5U623|MCAF2\_HUMAN sp|Q8TDY2|RBCC1\_HUMAN sp|Q9Y5G6|PCDG7\_HUMAN tr|I3L4J3|I3L4J3\_HUMAN sp|Q7Z3B3|KANL1\_HUMAN sp|Q8WTR4|GDPD5\_HUMAN sp|Q9Y620|RA54B\_HUMAN tr|A0A087X0H2|A0A087X0H2\_HUMAN tr|B0QYJ6|B0QYJ6\_HUMAN sp|O95782|AP2A1\_HUMAN tr|E9PD53|E9PD53\_HUMAN sp|Q9NTJ3|SMC4\_HUMAN sp|Q9UNW9|NOVA2\_HUMAN sp|Q2M3M2|SC5A9\_HUMAN sp|Q6ZUB1|S31E1\_HUMAN tr|G3V1W5|G3V1W5\_HUMAN sp|Q92673|SORL\_HUMAN tr|A0A087WY75|A0A087WY75\_HUMAN sp|Q8NG75|OR5T1\_HUMAN tr|A0A087WU65|A0A087WU65\_HUMAN sp|Q8WXH6|RB40A\_HUMAN tr|J3QSY2|J3QSY2\_HUMAN tr|H0Y7E2|H0Y7E2\_HUMAN sp|Q9NWK9|BCD1\_HUMAN sp|O15350|P73\_HUMAN sp|Q9NPR2|SEM4B\_HUMAN tr|J3KNP4|J3KNP4\_HUMAN tr|Q5JXL1|Q5JXL1\_HUMAN sp|Q9BVI0|PHF20\_HUMAN tr|Q5JWZ0|Q5JWZ0\_HUMAN sp|Q04912|RON\_HUMAN tr|H7BYR8|H7BYR8\_HUMAN sp|Q8IWA5|CTL2\_HUMAN sp|Q9UHI7|S23A1\_HUMAN tr|D6RHC4|D6RHC4\_HUMAN sp|Q9H694|BICC1\_HUMAN tr|E5RJ67|E5RJ67\_HUMAN tr|F8WAQ1|F8WAQ1\_HUMAN tr|H3BLV4|H3BLV4\_HUMAN tr|A8MXH5|A8MXH5\_HUMAN tr|F5H851|F5H851\_HUMAN sp|Q14031|CO4A6\_HUMAN tr|C9JVP0|C9JVP0\_HUMAN sp|Q9UQQ2|SH2B3\_HUMAN sp|Q96CG8|CTHR1\_HUMAN tr|M0QYL4|M0QYL4\_HUMAN tr|Q8IVZ9|Q8IVZ9\_HUMAN sp|Q9Y5X4|NR2E3\_HUMAN sp|Q9UQB3|CTND2\_HUMAN Q9TTE1 sp|Q99598|TSNAX\_HUMAN tr|H0YD40|H0YD40\_HUMAN sp|Q9Y2Y6|TMM98\_HUMAN tr|C9J6Q8|C9J6Q8\_HUMAN tr|H7C3B5|H7C3B5\_HUMAN sp|O60239|3BP5\_HUMAN sp|Q9NZ09|UBAP1\_HUMAN sp|Q92878|RAD50\_HUMAN sp|Q8WWA1|TMM40\_HUMAN sp|Q6P3S6|FBX42\_HUMAN sp|O15156|ZBT7B\_HUMAN sp|Q96S90|LYSM1\_HUMAN sp|Q8WVV5|BT2A2\_HUMAN tr|I3L0V0|I3L0V0\_HUMAN sp|Q53H80|AKIR2\_HUMAN sp|Q9H205|O2AG1\_HUMAN tr|H0Y9Y6|H0Y9Y6\_HUMAN sp|Q56P03|EAPP\_HUMAN tr|E9PGM1|E9PGM1\_HUMAN sp|Q04637|IF4G1\_HUMAN tr|H7C1T2|H7C1T2\_HUMAN sp|Q8TEH3|DEN1A\_HUMAN sp|P54253|ATX1\_HUMAN tr|E7EXB4|E7EXB4\_HUMAN tr|F5H6B2|F5H6B2\_HUMAN tr|E7ESH4|E7ESH4\_HUMAN tr|C9JI46|C9JI46\_HUMAN sp|Q8N3X6|LCORL\_HUMAN tr|H0YBI2|H0YBI2\_HUMAN sp|Q8WXA8|5HT3C\_HUMAN tr|H0YDA5|H0YDA5\_HUMAN sp|Q96SI9|STRBP\_HUMAN tr|Q5T6R1|Q5T6R1\_HUMAN tr|C9J6P4|C9J6P4\_HUMAN sp|Q7Z2W4|ZCCHV\_HUMAN tr|A0A087WY71|A0A087WY71\_HUMAN sp|Q8NEA9|GMCLL\_HUMAN sp|Q9H765|ASB8\_HUMAN tr|C9JZH4|C9JZH4\_HUMAN sp|Q9Y5F9|PCDGI\_HUMAN sp|P51617|IRAK1\_HUMAN tr|D3YTB5|D3YTB5\_HUMAN sp|Q9UPN4|CP131\_HUMAN sp|Q96EG1|ARSG\_HUMAN tr|I3L2J8|I3L2J8\_HUMAN tr|C9J155|C9J155\_HUMAN tr|H7C1S1|H7C1S1\_HUMAN tr|J9JIC7|J9JIC7\_HUMAN sp|Q6ZRS4|CC129\_HUMAN tr|A0A087WUB1|A0A087WUB1\_HUMAN sp|Q9BUJ2|HNRL1\_HUMAN tr|B7Z4B8|B7Z4B8\_HUMAN tr|M0QZ28|M0QZ28\_HUMAN sp|Q8TC41|RN217\_HUMAN tr|A0A087WWG8|A0A087WWG8\_HUMAN sp|Q12840|KIF5A\_HUMAN sp|Q92570|NR4A3\_HUMAN tr|H0YCV8|H0YCV8\_HUMAN tr|I3L0M7|I3L0M7\_HUMAN tr|F8W6L0|F8W6L0\_HUMAN tr|H0YMA2|H0YMA2\_HUMAN tr|I3L2W9|I3L2W9\_HUMAN tr|E7ERK1|E7ERK1\_HUMAN sp|Q7Z2H8|S36A1\_HUMAN sp|Q96JW4|S41A2\_HUMAN sp|Q9UDR5|AASS\_HUMAN tr|E7EMV2|E7EMV2\_HUMAN sp|Q69YN2|C19L1\_HUMAN sp|P31943|HNRH1\_HUMAN sp|Q75VX8|GAREL\_HUMAN tr|J3KNG9|J3KNG9\_HUMAN sp|Q12959|DLG1\_HUMAN tr|A8MX12|A8MX12\_HUMAN sp|Q9H8W5|TRI45\_HUMAN sp|Q70EL2|UBP45\_HUMAN sp|P21860|ERBB3\_HUMAN tr|E7EPK0|E7EPK0\_HUMAN tr|H0Y8P3|H0Y8P3\_HUMAN sp|Q9UPQ0|LIMC1\_HUMAN sp|Q9HBM0|VEZA\_HUMAN tr|E9PDJ9|E9PDJ9\_HUMAN sp|Q9BX59|TPSNR\_HUMAN tr|H0YNK6|H0YNK6\_HUMAN tr|Q68DL3|Q68DL3\_HUMAN sp|Q13905|RPGF1\_HUMAN sp|Q6PCT2|FXL19\_HUMAN tr|E7EV46|E7EV46\_HUMAN tr|H3BM42|H3BM42\_HUMAN tr|F5H5G7|F5H5G7\_HUMAN sp|Q15034|HERC3\_HUMAN sp|Q6TFL3|CC171\_HUMAN sp|Q8WZA1|PMGT1\_HUMAN tr|B7Z524|B7Z524\_HUMAN tr|J3QT46|J3QT46\_HUMAN tr|H0YDX7|H0YDX7\_HUMAN tr|E5RIF6|E5RIF6\_HUMAN sp|Q1MSJ5|CSPP1\_HUMAN sp|Q9H347|UBQL3\_HUMAN sp|A0PJK1|SC5AA\_HUMAN sp|Q8N157|AHI1\_HUMAN sp|Q17RY0|CPEB4\_HUMAN tr|E5RJM0|E5RJM0\_HUMAN tr|B7ZLQ8|B7ZLQ8\_HUMAN sp|P58401|NRX2B\_HUMAN sp|Q9P0U4|CXXC1\_HUMAN tr|B5MCL8|B5MCL8\_HUMAN tr|F5H3M9|F5H3M9\_HUMAN sp|Q8WZA9|IRGQ\_HUMAN tr|H7BY55|H7BY55\_HUMAN tr|M0R300|M0R300\_HUMAN sp|P46059|S15A1\_HUMAN sp|P51160|PDE6C\_HUMAN tr|Q5VZA6|Q5VZA6\_HUMAN sp|Q96L93|KI16B\_HUMAN sp|Q96S44|PRPK\_HUMAN sp|Q8NGT0|O13C9\_HUMAN tr|E7ERP4|E7ERP4\_HUMAN sp|Q9NZQ3|SPN90\_HUMAN tr|H0Y300|H0Y300\_HUMAN tr|C1KBH7|C1KBH7\_HUMAN sp|P61981|1433G\_HUMAN sp|Q17RG1|KCD19\_HUMAN sp|P48378|RFX2\_HUMAN sp|O14772|FPGT\_HUMAN sp|P48357|LEPR\_HUMAN sp|Q8N1K5|THMS1\_HUMAN sp|Q8WXW3|PIBF1\_HUMAN tr|A0A087WUI6|A0A087WUI6\_HUMAN tr|H0YAC6|H0YAC6\_HUMAN tr|A0A087WUF1|A0A087WUF1\_HUMAN sp|P11387|TOP1\_HUMAN tr|A0A087WY24|A0A087WY24\_HUMAN sp|Q92609|TBCD5\_HUMAN tr|A0A075B764|A0A075B764\_HUMAN sp|P22061|PIMT\_HUMAN tr|A0A087WUW2|A0A087WUW2\_HUMAN tr|G3V3Y1|G3V3Y1\_HUMAN sp|P08581|MET\_HUMAN sp|Q5VUJ9|EFCB2\_HUMAN tr|B7ZBM2|B7ZBM2\_HUMAN sp|Q14186|TFDP1\_HUMAN tr|E7EW71|E7EW71\_HUMAN tr|F6VGP8|F6VGP8\_HUMAN sp|Q8WUT9|S2543\_HUMAN sp|Q9P1Y5|CAMP3\_HUMAN sp|Q6NUQ1|RINT1\_HUMAN tr|C9JXV0|C9JXV0\_HUMAN tr|E5RFG9|E5RFG9\_HUMAN sp|Q9HCC0|MCCB\_HUMAN sp|P00387|NB5R3\_HUMAN tr|F8WAN4|F8WAN4\_HUMAN sp|Q9HCP6|HHATL\_HUMAN sp|P78426|NKX61\_HUMAN tr|C9JZL6|C9JZL6\_HUMAN sp|Q13428|TCOF\_HUMAN tr|E7ETY2|E7ETY2\_HUMAN sp|Q8TCN5|ZN507\_HUMAN sp|Q96PL2|TECTB\_HUMAN sp|Q13478|IL18R\_HUMAN sp|Q8N2E2|VWDE\_HUMAN sp|Q9H6T0|ESRP2\_HUMAN sp|Q5JPF3|AN36C\_HUMAN tr|F5H3S6|F5H3S6\_HUMAN sp|Q8NFN8|GP156\_HUMAN tr|B7Z2U2|B7Z2U2\_HUMAN sp|Q9NY15|STAB1\_HUMAN tr|I3L239|I3L239\_HUMAN sp|P43627|KI2L2\_HUMAN sp|O15020|SPTN2\_HUMAN sp|Q07343|PDE4B\_HUMAN tr|F5H1R4|F5H1R4\_HUMAN tr|K7ES92|K7ES92\_HUMAN sp|Q8N957|ANKF1\_HUMAN tr|D6R968|D6R968\_HUMAN sp|Q92802|N42L2\_HUMAN tr|H0YBS1|H0YBS1\_HUMAN sp|Q16348|S15A2\_HUMAN sp|Q8IVF6|AN18A\_HUMAN tr|E7EW16|E7EW16\_HUMAN tr|B4E3L3|B4E3L3\_HUMAN sp|Q96RU2|UBP28\_HUMAN tr|B7Z3Z9|B7Z3Z9\_HUMAN tr|X6RGK9|X6RGK9\_HUMAN sp|Q9Y2J8|PADI2\_HUMAN tr|E7EPM6|E7EPM6\_HUMAN sp|Q96F45|ZN503\_HUMAN sp|P33121|ACSL1\_HUMAN tr|F5GXD8|F5GXD8\_HUMAN sp|Q9P2J9|PDP2\_HUMAN sp|P43694|GATA4\_HUMAN tr|C4P092|C4P092\_HUMAN tr|C4P0A0|C4P0A0\_HUMAN tr|C4P0B0|C4P0B0\_HUMAN tr|C4P093|C4P093\_HUMAN sp|P00749|UROK\_HUMAN sp|Q96N64|PWP2A\_HUMAN tr|K7ES96|K7ES96\_HUMAN sp|Q9BW04|SARG\_HUMAN sp|P07333|CSF1R\_HUMAN sp|Q9UN71|PCDGG\_HUMAN tr|F5H0F9|F5H0F9\_HUMAN sp|Q9UJX4|APC5\_HUMAN sp|Q9UNA1|RHG26\_HUMAN sp|Q9Y232|CDYL1\_HUMAN sp|Q96R27|OR2M4\_HUMAN tr|H7BYD9|H7BYD9\_HUMAN sp|Q9Y4B4|ARIP4\_HUMAN tr|A0JD36|A0JD36\_HUMAN tr|Q8WUD0|Q8WUD0\_HUMAN sp|B1AJZ9|FHAD1\_HUMAN sp|Q8N100|ATOH7\_HUMAN tr|H0YLB0|H0YLB0\_HUMAN sp|P40424|PBX1\_HUMAN sp|O95819|M4K4\_HUMAN tr|E7ETY4|E7ETY4\_HUMAN tr|F8WAN1|F8WAN1\_HUMAN sp|Q8TB52|FBX30\_HUMAN sp|P98175|RBM10\_HUMAN sp|P19022|CADH2\_HUMAN sp|P78325|ADAM8\_HUMAN sp|Q9P2H0|K1377\_HUMAN sp|P57075|UBS3A\_HUMAN tr|F8VYH7|F8VYH7\_HUMAN tr|F8VXC8|F8VXC8\_HUMAN sp|Q8TAQ2|SMRC2\_HUMAN sp|Q9NZW4|DSPP\_HUMAN sp|A6NHR8|FA47D\_HUMAN sp|Q8N9U0|TAC2N\_HUMAN tr|J3KPA5|J3KPA5\_HUMAN sp|Q8NA31|TERB1\_HUMAN sp|Q7Z7L1|SLN11\_HUMAN tr|H3BNU7|H3BNU7\_HUMAN sp|Q9H9K5|MER34\_HUMAN sp|Q6UY01|LRC31\_HUMAN tr|F8VV52|F8VV52\_HUMAN sp|Q9NZN8|CNOT2\_HUMAN tr|Q96E41|Q96E41\_HUMAN sp|Q9Y4P3|TBL2\_HUMAN tr|H7C3W7|H7C3W7\_HUMAN sp|P31994|FCG2B\_HUMAN sp|A6NNL0|NTM2B\_HUMAN sp|Q8IVF1|NTM2A\_HUMAN sp|B1AL46|NTM2E\_HUMAN sp|Q8N766|EMC1\_HUMAN sp|Q9HCJ3|RAVR2\_HUMAN tr|F5H4Q0|F5H4Q0\_HUMAN sp|Q5T447|HECD3\_HUMAN tr|J3KNA1|J3KNA1\_HUMAN sp|Q9Y3Q0|NALD2\_HUMAN tr|J3KNJ3|J3KNJ3\_HUMAN tr|C9JA99|C9JA99\_HUMAN tr|G3V3X5|G3V3X5\_HUMAN sp|Q14767|LTBP2\_HUMAN tr|G3V511|G3V511\_HUMAN tr|G3V1P5|G3V1P5\_HUMAN sp|Q96RT6|CTGE2\_HUMAN sp|Q6ZW49|PAXI1\_HUMAN sp|Q8TCS8|PNPT1\_HUMAN tr|B4E0G6|B4E0G6\_HUMAN sp|Q6UXC1|AEGP\_HUMAN sp|Q5TEA3|CT194\_HUMAN tr|A0A087WU18|A0A087WU18\_HUMAN sp|Q9UIA9|XPO7\_HUMAN tr|E7ESC6|E7ESC6\_HUMAN sp|O75335|LIPA4\_HUMAN tr|B8ZZW5|B8ZZW5\_HUMAN sp|Q8NBF6|AVL9\_HUMAN tr|D6RCT7|D6RCT7\_HUMAN tr|F5H2J1|F5H2J1\_HUMAN sp|Q8WXR4|MYO3B\_HUMAN sp|Q9H2Y9|SO5A1\_HUMAN tr|E5KLJ5|E5KLJ5\_HUMAN sp|O60313|OPA1\_HUMAN tr|E5KLK1|E5KLK1\_HUMAN tr|E5KLJ9|E5KLJ9\_HUMAN tr|E5KLJ6|E5KLJ6\_HUMAN tr|H7BZB8|H7BZB8\_HUMAN tr|B3KQH5|B3KQH5\_HUMAN sp|Q7Z5Q1|CPEB2\_HUMAN tr|H7C152|H7C152\_HUMAN sp|O60423|AT8B3\_HUMAN sp|P14672|GTR4\_HUMAN sp|P0C7W6|CC172\_HUMAN tr|J3KQS7|J3KQS7\_HUMAN sp|Q9BW19|KIFC1\_HUMAN sp|Q8NGP4|OR5M3\_HUMAN sp|Q9C093|SPEF2\_HUMAN tr|C9JSX6|C9JSX6\_HUMAN tr|R4GMU1|R4GMU1\_HUMAN tr|A2RRC6|A2RRC6\_HUMAN sp|O95479|G6PE\_HUMAN tr|Q96RY8|Q96RY8\_HUMAN sp|Q6P996|PDXD1\_HUMAN tr|H3BND4|H3BND4\_HUMAN sp|Q15223|PVRL1\_HUMAN sp|Q9BS86|ZPBP1\_HUMAN sp|Q6VEQ5|WASH2\_HUMAN tr|A0A096LP75|A0A096LP75\_HUMAN sp|P04150|GCR\_HUMAN tr|A0A096LNU2|A0A096LNU2\_HUMAN sp|Q9NZ56|FMN2\_HUMAN sp|Q6H9L7|ISM2\_HUMAN sp|Q8TER0|SNED1\_HUMAN sp|O43175|SERA\_HUMAN tr|A0A087WZZ4|A0A087WZZ4\_HUMAN tr|Q5SZU1|Q5SZU1\_HUMAN tr|H0Y8S7|H0Y8S7\_HUMAN tr|G5E9Z6|G5E9Z6\_HUMAN sp|P41225|SOX3\_HUMAN sp|A0AVT1|UBA6\_HUMAN tr|A0A087WYH6|A0A087WYH6\_HUMAN tr|A0A087WZX4|A0A087WZX4\_HUMAN sp|Q8IWU2|LMTK2\_HUMAN sp|Q7Z4Q2|HEAT3\_HUMAN sp|Q9GZU7|CTDS1\_HUMAN sp|Q86V25|VASH2\_HUMAN tr|H7BXL8|H7BXL8\_HUMAN sp|Q9NTZ6|RBM12\_HUMAN sp|A1A5C7|S22AN\_HUMAN tr|C9J4Z0|C9J4Z0\_HUMAN sp|O14647|CHD2\_HUMAN sp|Q9GZN7|ROGDI\_HUMAN tr|K7EPN1|K7EPN1\_HUMAN tr|F8VZV4|F8VZV4\_HUMAN sp|Q9NZ71|RTEL1\_HUMAN sp|Q14940|SL9A5\_HUMAN tr|W0HKK7|W0HKK7\_HUMAN sp|P17858|PFKAL\_HUMAN sp|Q9Y250|LZTS1\_HUMAN tr|K7EPD6|K7EPD6\_HUMAN sp|Q8NFW5|DMBX1\_HUMAN sp|Q2YD98|UVSSA\_HUMAN tr|H0Y5T2|H0Y5T2\_HUMAN sp|O43865|SAHH2\_HUMAN sp|P49279|NRAM1\_HUMAN sp|Q15061|WDR43\_HUMAN tr|A0A087WUE1|A0A087WUE1\_HUMAN tr|E7EX53|E7EX53\_HUMAN tr|E7EQV9|E7EQV9\_HUMAN sp|P61313|RL15\_HUMAN sp|Q9UPW6|SATB2\_HUMAN sp|Q8IY22|CMIP\_HUMAN sp|P14543|NID1\_HUMAN tr|A0A087WU05|A0A087WU05\_HUMAN tr|A0A087WY48|A0A087WY48\_HUMAN tr|A0A087X0E8|A0A087X0E8\_HUMAN tr|F5GY28|F5GY28\_HUMAN sp|P35125|UBP6\_HUMAN tr|H3BLW0|H3BLW0\_HUMAN sp|Q99575|POP1\_HUMAN tr|D6RBB0|D6RBB0\_HUMAN sp|O60683|PEX10\_HUMAN sp|Q8NAP3|ZBT38\_HUMAN tr|Q9H2M1|Q9H2M1\_HUMAN sp|Q14147|DHX34\_HUMAN tr|K7ER00|K7ER00\_HUMAN tr|M0QXE1|M0QXE1\_HUMAN sp|Q13572|ITPK1\_HUMAN sp|P82279|CRUM1\_HUMAN tr|F5H0L2|F5H0L2\_HUMAN sp|Q8NHY2|RFWD2\_HUMAN sp|P18846|ATF1\_HUMAN sp|Q9BQT9|CSTN3\_HUMAN sp|Q9HAC8|UBTD1\_HUMAN sp|Q9Y5B0|CTDP1\_HUMAN tr|K7EJD2|K7EJD2\_HUMAN tr|H0YCU6|H0YCU6\_HUMAN tr|E7EMB8|E7EMB8\_HUMAN sp|P43235|CATK\_HUMAN sp|P42704|LPPRC\_HUMAN tr|E9PGM7|E9PGM7\_HUMAN tr|H0Y9K5|H0Y9K5\_HUMAN sp|Q8WU10|PYRD1\_HUMAN tr|B7WPN9|B7WPN9\_HUMAN sp|Q4VNC1|AT134\_HUMAN tr|H0YKB7|H0YKB7\_HUMAN tr|M0R3F1|M0R3F1\_HUMAN sp|Q00587|BORG5\_HUMAN sp|Q13219|PAPP1\_HUMAN tr|A0A087WXN9|A0A087WXN9\_HUMAN sp|Q765P7|MTSSL\_HUMAN tr|E5RJ14|E5RJ14\_HUMAN tr|G3V2K0|G3V2K0\_HUMAN tr|E5RH16|E5RH16\_HUMAN sp|Q9H156|SLIK2\_HUMAN sp|Q3BBW0|NBPF9\_HUMAN sp|O43451|MGA\_HUMAN tr|E7ER45|E7ER45\_HUMAN tr|B3KTY4|B3KTY4\_HUMAN sp|Q5TIA1|MEI1\_HUMAN tr|G5EA50|G5EA50\_HUMAN sp|Q6DKI7|PVRIG\_HUMAN tr|J3KNR0|J3KNR0\_HUMAN sp|P27448|MARK3\_HUMAN sp|Q86UD0|SAPC2\_HUMAN sp|Q14164|IKKE\_HUMAN tr|F5GX87|F5GX87\_HUMAN tr|F6UGA0|F6UGA0\_HUMAN tr|E7EWH9|E7EWH9\_HUMAN sp|O43312|MTSS1\_HUMAN tr|E7EWW5|E7EWW5\_HUMAN tr|K7EL81|K7EL81\_HUMAN sp|P19793|RXRA\_HUMAN sp|Q8N5U6|RNF10\_HUMAN tr|H0Y9C8|H0Y9C8\_HUMAN tr|H7C183|H7C183\_HUMAN tr|J3KPH0|J3KPH0\_HUMAN sp|Q96RD3|O52E6\_HUMAN sp|Q6ZRH7|CTSRG\_HUMAN sp|Q9NTN9|SEM4G\_HUMAN sp|Q7Z6B7|SRGP1\_HUMAN sp|P00480|OTC\_HUMAN sp|Q9UK96|FBX10\_HUMAN tr|H0YBF1|H0YBF1\_HUMAN sp|Q96N46|TTC14\_HUMAN tr|H7C3T1|H7C3T1\_HUMAN tr|H7C2C4|H7C2C4\_HUMAN tr|H3BNR4|H3BNR4\_HUMAN sp|O60240|PLIN1\_HUMAN sp|Q07687|DLX2\_HUMAN sp|Q8IUI8|CRLF3\_HUMAN tr|M0QZ22|M0QZ22\_HUMAN sp|Q5PRF9|SMAG2\_HUMAN tr|E9PFE2|E9PFE2\_HUMAN tr|E9PE95|E9PE95\_HUMAN sp|Q6NUI2|GPAT2\_HUMAN sp|O60741|HCN1\_HUMAN tr|J3KNL7|J3KNL7\_HUMAN tr|M0R2A6|M0R2A6\_HUMAN tr|M0R178|M0R178\_HUMAN tr|S4R2X8|S4R2X8\_HUMAN sp|Q6UX71|PXDC2\_HUMAN tr|M0R082|M0R082\_HUMAN tr|M0R0M6|M0R0M6\_HUMAN tr|H0YJA2|H0YJA2\_HUMAN tr|J3QL70|J3QL70\_HUMAN sp|P55289|CAD12\_HUMAN tr|H7C4P5|H7C4P5\_HUMAN sp|Q6N069|NAA16\_HUMAN sp|Q8NHQ8|RASF8\_HUMAN sp|Q9UK53|ING1\_HUMAN tr|Q08AT0|Q08AT0\_HUMAN sp|Q16720|AT2B3\_HUMAN tr|B4DTS2|B4DTS2\_HUMAN tr|A6NGW4|A6NGW4\_HUMAN sp|O75128|COBL\_HUMAN tr|H7C1N2|H7C1N2\_HUMAN tr|E7ESL9|E7ESL9\_HUMAN sp|Q6ICL3|TNG2\_HUMAN tr|F5H0R1|F5H0R1\_HUMAN sp|Q9H6E5|STPAP\_HUMAN tr|A0A087WU08|A0A087WU08\_HUMAN tr|H0Y9V3|H0Y9V3\_HUMAN tr|J3QLC9|J3QLC9\_HUMAN sp|P00738|HPT\_HUMAN tr|J3QR68|J3QR68\_HUMAN sp|P53675|CLH2\_HUMAN sp|P53794|SC5A3\_HUMAN sp|P04350|TBB4A\_HUMAN tr|A0A087WTE4|A0A087WTE4\_HUMAN tr|H7BYX6|H7BYX6\_HUMAN sp|P05165|PCCA\_HUMAN sp|Q8NHU2|CT026\_HUMAN sp|Q02246|CNTN2\_HUMAN sp|Q03933|HSF2\_HUMAN sp|Q8TDM0|BCAS4\_HUMAN sp|Q12904|AIMP1\_HUMAN sp|O14718|OPSX\_HUMAN tr|Q86XA6|Q86XA6\_HUMAN tr|A0A087WXZ7|A0A087WXZ7\_HUMAN sp|P86791|CCZ1\_HUMAN tr|H0YIN8|H0YIN8\_HUMAN sp|P86790|CCZ1B\_HUMAN tr|F8WDM8|F8WDM8\_HUMAN tr|H7C410|H7C410\_HUMAN sp|Q969F9|HPS3\_HUMAN tr|G5E9Y1|G5E9Y1\_HUMAN sp|Q92870|APBB2\_HUMAN sp|Q9HC29|NOD2\_HUMAN tr|E3W980|E3W980\_HUMAN sp|P32519|ELF1\_HUMAN tr|M0R0W6|M0R0W6\_HUMAN tr|E7EUN2|E7EUN2\_HUMAN tr|H0YGG5|H0YGG5\_HUMAN sp|Q92858|ATOH1\_HUMAN sp|Q13887|KLF5\_HUMAN sp|Q13606|OR5I1\_HUMAN sp|Q15011|HERP1\_HUMAN sp|Q8TCW9|PKR1\_HUMAN tr|K7ES28|K7ES28\_HUMAN sp|Q9Y2G9|SBNO2\_HUMAN sp|P56817|BACE1\_HUMAN sp|Q9GZV9|FGF23\_HUMAN sp|A6NMU1|O52A4\_HUMAN tr|E9PJG7|E9PJG7\_HUMAN tr|J3KTQ2|J3KTQ2\_HUMAN sp|Q9HB14|KCNKD\_HUMAN tr|F5GXC5|F5GXC5\_HUMAN sp|Q6ZNF0|PAPL\_HUMAN sp|P20290|BTF3\_HUMAN tr|E7EV41|E7EV41\_HUMAN sp|Q02880|TOP2B\_HUMAN tr|M0QY29|M0QY29\_HUMAN tr|F6Q6H0|F6Q6H0\_HUMAN tr|H0Y448|H0Y448\_HUMAN tr|X6RDD7|X6RDD7\_HUMAN tr|Q5T8E0|Q5T8E0\_HUMAN tr|K9J956|K9J956\_HUMAN sp|Q13002|GRIK2\_HUMAN tr|A0A087WUR1|A0A087WUR1\_HUMAN sp|P08195|4F2\_HUMAN sp|Q8WWH4|ASZ1\_HUMAN sp|Q9UL62|TRPC5\_HUMAN sp|Q13472|TOP3A\_HUMAN Q3MHN5 ENSEMBL:ENSBTAP00000018229 sp|Q9Y468|LMBL1\_HUMAN tr|E7EMB6|E7EMB6\_HUMAN sp|Q9H3R2|MUC13\_HUMAN tr|J3KTE8|J3KTE8\_HUMAN sp|Q8N653|LZTR1\_HUMAN sp|Q5JS13|RGPS1\_HUMAN tr|E9PHA2|E9PHA2\_HUMAN sp|Q8N743|KI3L3\_HUMAN sp|Q53H82|LACB2\_HUMAN sp|Q8TBB6|S7A14\_HUMAN sp|Q9UGF6|OR5V1\_HUMAN sp|Q13555|KCC2G\_HUMAN tr|E7EMS7|E7EMS7\_HUMAN tr|C9J2Y9|C9J2Y9\_HUMAN tr|C9J4M6|C9J4M6\_HUMAN sp|P30876|RPB2\_HUMAN tr|H7BYI2|H7BYI2\_HUMAN tr|I3L187|I3L187\_HUMAN sp|Q9NQ89|CL004\_HUMAN tr|E7ER40|E7ER40\_HUMAN sp|Q9Y678|COPG1\_HUMAN sp|Q92824|PCSK5\_HUMAN sp|O75145|LIPA3\_HUMAN sp|Q15858|SCN9A\_HUMAN sp|Q06124|PTN11\_HUMAN sp|O95936|EOMES\_HUMAN sp|Q9BYX4|IFIH1\_HUMAN tr|M0QZR9|M0QZR9\_HUMAN tr|Q5VYL4|Q5VYL4\_HUMAN tr|B7Z3I5|B7Z3I5\_HUMAN sp|Q9NV39|PRR34\_HUMAN sp|P23515|OMGP\_HUMAN tr|H7BZL6|H7BZL6\_HUMAN sp|P0C5W0|PNM6B\_HUMAN sp|P0CZ20|PNM6D\_HUMAN sp|O94880|PHF14\_HUMAN sp|Q9Y2X3|NOP58\_HUMAN sp|P0CW26|PNM6C\_HUMAN sp|P0CW24|PNM6A\_HUMAN sp|Q6ZT07|TBCD9\_HUMAN sp|A2RUB1|CQ104\_HUMAN sp|P12757|SKIL\_HUMAN tr|K7EPA2|K7EPA2\_HUMAN sp|Q9NZB8|MOCS1\_HUMAN sp|P37088|SCNNA\_HUMAN sp|P04220|MUCB\_HUMAN sp|Q6ZSY5|PPR3F\_HUMAN tr|H0YF95|H0YF95\_HUMAN tr|A0A096LNN3|A0A096LNN3\_HUMAN tr|E9PBT5|E9PBT5\_HUMAN tr|F5GZP4|F5GZP4\_HUMAN sp|Q9NPB8|GPCP1\_HUMAN sp|P57682|KLF3\_HUMAN sp|Q4U2R8|S22A6\_HUMAN sp|Q9Y3T6|R3HC1\_HUMAN sp|Q9UM07|PADI4\_HUMAN tr|E9PK09|E9PK09\_HUMAN tr|E9PKI6|E9PKI6\_HUMAN tr|E9PQN2|E9PQN2\_HUMAN tr|G3V256|G3V256\_HUMAN tr|H0YFP5|H0YFP5\_HUMAN sp|P50995|ANX11\_HUMAN sp|Q8N531|FBXL6\_HUMAN sp|Q15361|TTF1\_HUMAN sp|Q5VU57|CBPC6\_HUMAN sp|Q9Y5H0|PCDG3\_HUMAN sp|Q92854|SEM4D\_HUMAN sp|Q9BZ76|CNTP3\_HUMAN tr|H7C5N1|H7C5N1\_HUMAN sp|Q13972|RGRF1\_HUMAN tr|H3BM74|H3BM74\_HUMAN sp|Q9Y5A7|NUB1\_HUMAN tr|G3V154|G3V154\_HUMAN tr|F5GWS4|F5GWS4\_HUMAN tr|K7EJ74|K7EJ74\_HUMAN tr|K7EMY7|K7EMY7\_HUMAN tr|H0YBH8|H0YBH8\_HUMAN tr|H0YBA1|H0YBA1\_HUMAN tr|F8VWY3|F8VWY3\_HUMAN sp|O43633|CHM2A\_HUMAN tr|H0Y4R2|H0Y4R2\_HUMAN sp|Q8N8N7|PTGR2\_HUMAN tr|G3V2R9|G3V2R9\_HUMAN sp|Q66PJ3|AR6P4\_HUMAN tr|A0A087WZA7|A0A087WZA7\_HUMAN tr|A0A087WY66|A0A087WY66\_HUMAN sp|Q0VDF9|HSP7E\_HUMAN sp|Q2TAC2|CCD57\_HUMAN tr|E9PCN5|E9PCN5\_HUMAN tr|A0A087WVS7|A0A087WVS7\_HUMAN tr|H7C3P4|H7C3P4\_HUMAN tr|M0R0K6|M0R0K6\_HUMAN sp|Q9ULH7|MKL2\_HUMAN sp|Q99962|SH3G2\_HUMAN sp|O14867|BACH1\_HUMAN sp|Q92973|TNPO1\_HUMAN sp|Q96DM1|PGBD4\_HUMAN sp|Q9UJT2|TSKS\_HUMAN tr|A0A087WZT0|A0A087WZT0\_HUMAN sp|Q7RTM1|OTOP1\_HUMAN tr|H7C394|H7C394\_HUMAN sp|Q6W2J9|BCOR\_HUMAN tr|J3QQW3|J3QQW3\_HUMAN sp|P43243|MATR3\_HUMAN tr|M0R277|M0R277\_HUMAN sp|O95081|AGFG2\_HUMAN sp|O43164|PJA2\_HUMAN sp|O14617|AP3D1\_HUMAN sp|Q9NVH0|EXD2\_HUMAN sp|Q5HYA8|MKS3\_HUMAN sp|O15090|ZN536\_HUMAN sp|Q86UU5|GGN\_HUMAN sp|Q99712|KCJ15\_HUMAN Q9TT36 sp|O43361|ZN749\_HUMAN tr|B5MCX3|B5MCX3\_HUMAN sp|Q96MT8|CEP63\_HUMAN tr|D6RB16|D6RB16\_HUMAN tr|G3V350|G3V350\_HUMAN tr|A0A096LPG5|A0A096LPG5\_HUMAN sp|P0C7Q5|S35G4\_HUMAN tr|B1AKZ5|B1AKZ5\_HUMAN tr|H0YC60|H0YC60\_HUMAN sp|Q15121|PEA15\_HUMAN tr|D9ZHQ8|D9ZHQ8\_HUMAN sp|Q05682|CALD1\_HUMAN sp|Q5JRV8|T255A\_HUMAN sp|P78333|GPC5\_HUMAN sp|Q8IY17|PLPL6\_HUMAN sp|Q9Y2G1|MRF\_HUMAN sp|O95486|SC24A\_HUMAN sp|Q658Y4|F91A1\_HUMAN tr|E7ER68|E7ER68\_HUMAN tr|M0QY97|M0QY97\_HUMAN sp|Q15365|PCBP1\_HUMAN sp|Q6IN84|MRM1\_HUMAN sp|O95163|ELP1\_HUMAN sp|Q15397|K0020\_HUMAN sp|Q9BQS7|HEPH\_HUMAN tr|A2A3P3|A2A3P3\_HUMAN sp|Q15835|RK\_HUMAN tr|K7ESF7|K7ESF7\_HUMAN sp|P34932|HSP74\_HUMAN tr|M0QZ32|M0QZ32\_HUMAN sp|Q8IZA0|K319L\_HUMAN sp|Q5VSY0|GKAP1\_HUMAN sp|Q9NP80|PLPL8\_HUMAN tr|E7EN73|E7EN73\_HUMAN tr|H9KVC9|H9KVC9\_HUMAN tr|H9KVC7|H9KVC7\_HUMAN sp|Q86WB0|NIPA\_HUMAN sp|Q13490|BIRC2\_HUMAN tr|A0A087X2B2|A0A087X2B2\_HUMAN tr|E9PEK4|E9PEK4\_HUMAN sp|P29475|NOS1\_HUMAN tr|A0A087WZC4|A0A087WZC4\_HUMAN sp|P21554|CNR1\_HUMAN sp|Q9UBS5|GABR1\_HUMAN tr|C9JJ25|C9JJ25\_HUMAN sp|P59045|NAL11\_HUMAN sp|P49247|RPIA\_HUMAN sp|Q9NXL2|ARH38\_HUMAN sp|Q6UXI7|VITRN\_HUMAN tr|B5MD45|B5MD45\_HUMAN tr|X6R6V8|X6R6V8\_HUMAN sp|Q9Y2P7|ZN256\_HUMAN tr|F8W8C1|F8W8C1\_HUMAN sp|Q6ZTA4|TRI67\_HUMAN tr|H7BZ93|H7BZ93\_HUMAN tr|A0A087WXH1|A0A087WXH1\_HUMAN tr|H0YHI8|H0YHI8\_HUMAN tr|Q5JT35|Q5JT35\_HUMAN tr|H0YBC7|H0YBC7\_HUMAN sp|O60238|BNI3L\_HUMAN sp|Q96SD1|DCR1C\_HUMAN tr|A0A087WVL5|A0A087WVL5\_HUMAN sp|A1A5D9|BICR2\_HUMAN sp|Q8NH10|OR8U1\_HUMAN sp|Q9NW13|RBM28\_HUMAN sp|Q9BVJ6|UT14A\_HUMAN tr|J3KPJ6|J3KPJ6\_HUMAN sp|Q96RR1|PEO1\_HUMAN tr|J3QLM1|J3QLM1\_HUMAN tr|J3KS37|J3KS37\_HUMAN sp|Q9UKG4|S13A4\_HUMAN sp|Q2WEN9|CEA16\_HUMAN sp|P04198|MYCN\_HUMAN tr|F8VSE7|F8VSE7\_HUMAN tr|E9PDI6|E9PDI6\_HUMAN tr|A0A087WZV3|A0A087WZV3\_HUMAN sp|Q13936|CAC1C\_HUMAN tr|F5H522|F5H522\_HUMAN tr|A0A087WXD3|A0A087WXD3\_HUMAN tr|A0A087X293|A0A087X293\_HUMAN tr|A0A087WUZ0|A0A087WUZ0\_HUMAN tr|A0A087WWK5|A0A087WWK5\_HUMAN tr|B1AN15|B1AN15\_HUMAN P15636 tr|C9JDW2|C9JDW2\_HUMAN tr|H7C4L5|H7C4L5\_HUMAN sp|Q7Z3K6|MIER3\_HUMAN sp|O00625|PIR\_HUMAN sp|P23497|SP100\_HUMAN sp|Q9H8H0|NOL11\_HUMAN sp|Q9H2X8|I27L2\_HUMAN tr|K7EN89|K7EN89\_HUMAN sp|Q9UBF9|MYOTI\_HUMAN sp|B7ZC32|KIF28\_HUMAN sp|Q13231|CHIT1\_HUMAN tr|D6REY1|D6REY1\_HUMAN tr|K7END3|K7END3\_HUMAN sp|P11047|LAMC1\_HUMAN sp|Q969K4|ABTB1\_HUMAN sp|P20248|CCNA2\_HUMAN tr|R4GMU7|R4GMU7\_HUMAN sp|Q6DKI1|RL7L\_HUMAN sp|Q96KR4|LMLN\_HUMAN tr|H0Y314|H0Y314\_HUMAN sp|Q96I13|ABHD8\_HUMAN sp|Q5T7P8|SYT6\_HUMAN tr|M0QXZ3|M0QXZ3\_HUMAN sp|Q6NV75|GP153\_HUMAN sp|Q6UXE8|BTNL3\_HUMAN tr|B4DIP2|B4DIP2\_HUMAN tr|A0A087WSW7|A0A087WSW7\_HUMAN tr|B0QZ35|B0QZ35\_HUMAN tr|E9PC49|E9PC49\_HUMAN tr|Q5VUV5|Q5VUV5\_HUMAN sp|Q9BZP6|CHIA\_HUMAN tr|I3L2W2|I3L2W2\_HUMAN sp|Q8TAQ5|ZN420\_HUMAN tr|H7C4C5|H7C4C5\_HUMAN sp|Q9C0I3|CCSE1\_HUMAN tr|J3KQ96|J3KQ96\_HUMAN tr|H7BZA8|H7BZA8\_HUMAN sp|Q9BR11|ZSWM1\_HUMAN tr|G3V515|G3V515\_HUMAN sp|Q9HCX4|TRPC7\_HUMAN tr|Q70T25|Q70T25\_HUMAN tr|E9PGF6|E9PGF6\_HUMAN tr|H0YCI1|H0YCI1\_HUMAN sp|Q6PKC3|TXD11\_HUMAN tr|E9PG22|E9PG22\_HUMAN sp|Q8IW35|CEP97\_HUMAN tr|A0A087WWZ6|A0A087WWZ6\_HUMAN tr|A0A087WTR5|A0A087WTR5\_HUMAN tr|B9TX31|B9TX31\_HUMAN sp|Q71SY5|MED25\_HUMAN sp|Q96MW5|COG8\_HUMAN sp|Q05586|NMDZ1\_HUMAN tr|Q5VSF9|Q5VSF9\_HUMAN tr|E7EX73|E7EX73\_HUMAN tr|Q5SR54|Q5SR54\_HUMAN sp|Q8TDB8|GTR14\_HUMAN tr|A0A087WSV4|A0A087WSV4\_HUMAN tr|A0A087X0Z1|A0A087X0Z1\_HUMAN sp|Q5VUJ5|AGAP7\_HUMAN sp|P41180|CASR\_HUMAN sp|Q5VW22|AGAP6\_HUMAN sp|A6NIR3|AGAP5\_HUMAN sp|Q96P64|AGAP4\_HUMAN sp|Q5SRD3|AGAP8\_HUMAN sp|Q6U841|S4A10\_HUMAN tr|C9J240|C9J240\_HUMAN tr|E7EW28|E7EW28\_HUMAN tr|E9PHT4|E9PHT4\_HUMAN sp|O75676|KS6A4\_HUMAN tr|E9PJN1|E9PJN1\_HUMAN tr|F8W9C3|F8W9C3\_HUMAN sp|Q9NYL2|MLTK\_HUMAN tr|E7ESG2|E7ESG2\_HUMAN sp|P19827|ITIH1\_HUMAN sp|Q96DT6|ATG4C\_HUMAN sp|Q9UHJ6|SHPK\_HUMAN sp|Q9P2S6|ANKY1\_HUMAN sp|Q9C004|SPY4\_HUMAN tr|J3KQ21|J3KQ21\_HUMAN tr|E7EPC8|E7EPC8\_HUMAN Q0VCM5 sp|Q9H172|ABCG4\_HUMAN tr|M0R211|M0R211\_HUMAN sp|P00846|ATP6\_HUMAN sp|O14646|CHD1\_HUMAN sp|Q9UN73|PCDA6\_HUMAN sp|P20823|HNF1A\_HUMAN tr|E9PND6|E9PND6\_HUMAN tr|H0YEW0|H0YEW0\_HUMAN tr|E9PLV7|E9PLV7\_HUMAN tr|B7ZBM4|B7ZBM4\_HUMAN tr|B7ZBM5|B7ZBM5\_HUMAN sp|P18146|EGR1\_HUMAN tr|C9K0W8|C9K0W8\_HUMAN sp|P10124|SRGN\_HUMAN tr|H0YA49|H0YA49\_HUMAN sp|O14948|TFEC\_HUMAN sp|Q16134|ETFD\_HUMAN tr|H1UBN3|H1UBN3\_HUMAN sp|O00459|P85B\_HUMAN sp|Q3ZLR7|SP201\_HUMAN sp|P52294|IMA5\_HUMAN tr|G3V186|G3V186\_HUMAN sp|Q7L5D6|GET4\_HUMAN sp|O75339|CILP1\_HUMAN tr|H3BMZ2|H3BMZ2\_HUMAN sp|Q9BZD4|NUF2\_HUMAN sp|Q9NP71|MLXPL\_HUMAN sp|Q7Z4T9|MAAT1\_HUMAN sp|Q8N1I0|DOCK4\_HUMAN tr|B1AKD8|B1AKD8\_HUMAN sp|Q5TZA2|CROCC\_HUMAN tr|E9PHF7|E9PHF7\_HUMAN tr|F5GYT8|F5GYT8\_HUMAN tr|H3BM58|H3BM58\_HUMAN sp|Q9Y2B5|VP9D1\_HUMAN sp|Q9UKY7|CDV3\_HUMAN sp|Q96ME1|FXL18\_HUMAN sp|O95985|TOP3B\_HUMAN sp|Q9HBA9|FOH1B\_HUMAN sp|Q8TAL5|CI043\_HUMAN sp|Q13574|DGKZ\_HUMAN sp|O60583|CCNT2\_HUMAN sp|O14522|PTPRT\_HUMAN tr|B1AJS0|B1AJS0\_HUMAN tr|A0A075B6H0|A0A075B6H0\_HUMAN tr|B1AJR6|B1AJR6\_HUMAN tr|B1AJR8|B1AJR8\_HUMAN tr|H3BV54|H3BV54\_HUMAN sp|Q8WWN8|ARAP3\_HUMAN tr|G5E9Y3|G5E9Y3\_HUMAN sp|Q9P278|FNIP2\_HUMAN sp|Q9UBK8|MTRR\_HUMAN tr|D3DX46|D3DX46\_HUMAN sp|Q13368|MPP3\_HUMAN sp|Q8TDI0|CHD5\_HUMAN sp|P01229|LSHB\_HUMAN tr|H7C5T8|H7C5T8\_HUMAN sp|Q96QZ7|MAGI1\_HUMAN tr|H7C535|H7C535\_HUMAN tr|H7C4U7|H7C4U7\_HUMAN tr|H0Y8W8|H0Y8W8\_HUMAN sp|Q13634|CAD18\_HUMAN sp|Q8TCU5|NMD3A\_HUMAN sp|O43854|EDIL3\_HUMAN tr|E7ETR9|E7ETR9\_HUMAN sp|Q9HCP0|KC1G1\_HUMAN sp|P27348|1433T\_HUMAN tr|F5H0U9|F5H0U9\_HUMAN tr|H7C0U5|H7C0U5\_HUMAN tr|J3KQU5|J3KQU5\_HUMAN sp|Q96LU7|MRFL\_HUMAN tr|F8VVR8|F8VVR8\_HUMAN tr|B4DP72|B4DP72\_HUMAN sp|Q5HYK3|COQ5\_HUMAN tr|E9PCV4|E9PCV4\_HUMAN tr|A0A087WU30|A0A087WU30\_HUMAN tr|F8VNV8|F8VNV8\_HUMAN sp|P54284|CACB3\_HUMAN sp|Q5R3I4|TTC38\_HUMAN sp|A6NHC0|CAN8\_HUMAN sp|Q6AHZ1|Z518A\_HUMAN tr|J3QL79|J3QL79\_HUMAN tr|F8WD03|F8WD03\_HUMAN tr|H0Y696|H0Y696\_HUMAN sp|Q8NDW8|TT21A\_HUMAN tr|G5EA48|G5EA48\_HUMAN tr|B7Z3T3|B7Z3T3\_HUMAN sp|O00170|AIP\_HUMAN tr|J3KMZ9|J3KMZ9\_HUMAN tr|H7BXR3|H7BXR3\_HUMAN tr|C9JEB6|C9JEB6\_HUMAN tr|K7EPY5|K7EPY5\_HUMAN sp|Q68DL7|CR063\_HUMAN tr|H0YIY6|H0YIY6\_HUMAN tr|G3V3I3|G3V3I3\_HUMAN tr|G3V278|G3V278\_HUMAN sp|Q9Y2V7|COG6\_HUMAN tr|G3V1N5|G3V1N5\_HUMAN sp|Q9Y4G2|PKHM1\_HUMAN tr|A0A087X112|A0A087X112\_HUMAN tr|H0YB24|H0YB24\_HUMAN sp|Q8N163|CCAR2\_HUMAN tr|H3BR17|H3BR17\_HUMAN sp|Q8TBC5|ZSC18\_HUMAN sp|Q8N543|OGFD1\_HUMAN tr|H0Y7R9|H0Y7R9\_HUMAN tr|J3QT09|J3QT09\_HUMAN tr|E9PNV5|E9PNV5\_HUMAN sp|P04083|ANXA1\_HUMAN tr|Q5T3N1|Q5T3N1\_HUMAN tr|A0A087WTQ1|A0A087WTQ1\_HUMAN sp|O75061|AUXI\_HUMAN sp|Q9H4D0|CSTN2\_HUMAN sp|P21397|AOFA\_HUMAN sp|Q96JB6|LOXL4\_HUMAN sp|P61570|ENK25\_HUMAN tr|A0A087WZ09|A0A087WZ09\_HUMAN tr|E9PEY4|E9PEY4\_HUMAN sp|Q96GN5|CDA7L\_HUMAN tr|H0Y6S3|H0Y6S3\_HUMAN sp|A1L1A6|IGS23\_HUMAN sp|O43909|EXTL3\_HUMAN sp|Q9UBB5|MBD2\_HUMAN sp|A4D1P6|WDR91\_HUMAN tr|C9J1X0|C9J1X0\_HUMAN tr|J3KQ07|J3KQ07\_HUMAN tr|Q6GPI0|Q6GPI0\_HUMAN sp|P01042|KNG1\_HUMAN tr|A0A087X1F1|A0A087X1F1\_HUMAN tr|J3KPY5|J3KPY5\_HUMAN tr|G3V1A1|G3V1A1\_HUMAN tr|E9PKZ0|E9PKZ0\_HUMAN sp|P62917|RL8\_HUMAN sp|Q96ME7|ZN512\_HUMAN tr|G3XAG1|G3XAG1\_HUMAN sp|Q9P2N4|ATS9\_HUMAN tr|A0A087X165|A0A087X165\_HUMAN sp|Q96RS0|TGS1\_HUMAN tr|C9J896|C9J896\_HUMAN tr|H7C1R5|H7C1R5\_HUMAN tr|Q59FP8|Q59FP8\_HUMAN tr|Q5QPR4|Q5QPR4\_HUMAN tr|Q5QPR3|Q5QPR3\_HUMAN sp|Q9UQ88|CD11A\_HUMAN sp|Q9BXL5|HEMGN\_HUMAN tr|H0YG85|H0YG85\_HUMAN sp|Q9BVR0|HRC23\_HUMAN sp|A6NKX4|S22AV\_HUMAN sp|Q9P2B2|FPRP\_HUMAN sp|Q9BZI7|REN3B\_HUMAN sp|Q5VWK0|NBPF6\_HUMAN sp|Q96M43|NBPF4\_HUMAN sp|A6NC57|ANR62\_HUMAN tr|Q5HY78|Q5HY78\_HUMAN sp|O60749|SNX2\_HUMAN sp|Q9BXN2|CLC7A\_HUMAN tr|E7ETB9|E7ETB9\_HUMAN sp|Q7Z3T1|OR2W3\_HUMAN sp|P15907|SIAT1\_HUMAN sp|P08697|A2AP\_HUMAN sp|Q9NP84|TNR12\_HUMAN sp|Q92481|AP2B\_HUMAN sp|Q9HBD1|RC3H2\_HUMAN sp|Q2NL82|TSR1\_HUMAN tr|V9GZ37|V9GZ37\_HUMAN tr|B8ZZJ1|B8ZZJ1\_HUMAN tr|H0YFV4|H0YFV4\_HUMAN sp|Q96RA2|OR7D2\_HUMAN tr|H0Y343|H0Y343\_HUMAN tr|H0Y9L4|H0Y9L4\_HUMAN sp|Q6ZSS7|MFSD6\_HUMAN sp|P48723|HSP13\_HUMAN tr|I3L2G5|I3L2G5\_HUMAN sp|Q9C000|NALP1\_HUMAN tr|A0A087WX40|A0A087WX40\_HUMAN tr|H7BXC7|H7BXC7\_HUMAN sp|Q92783|STAM1\_HUMAN sp|Q96MN2|NALP4\_HUMAN sp|Q9Y5G4|PCDG9\_HUMAN tr|E9PMG1|E9PMG1\_HUMAN tr|Q59FY4|Q59FY4\_HUMAN tr|E7EQ78|E7EQ78\_HUMAN sp|Q8NER1|TRPV1\_HUMAN tr|I3L1R6|I3L1R6\_HUMAN sp|Q8IVU1|IGDC3\_HUMAN tr|H0Y917|H0Y917\_HUMAN tr|A0A087WX12|A0A087WX12\_HUMAN tr|G3V582|G3V582\_HUMAN sp|Q6ZRP7|QSOX2\_HUMAN tr|A0A087X176|A0A087X176\_HUMAN sp|P17544|ATF7\_HUMAN sp|P29074|PTN4\_HUMAN sp|P50570|DYN2\_HUMAN sp|Q13009|TIAM1\_HUMAN sp|A5YM72|CRNS1\_HUMAN sp|Q8IVI9|NOSTN\_HUMAN sp|Q8TD55|PKHO2\_HUMAN tr|G3V236|G3V236\_HUMAN tr|G3V3F7|G3V3F7\_HUMAN sp|Q9P2R7|SUCB1\_HUMAN tr|H0Y7Y5|H0Y7Y5\_HUMAN sp|Q13107|UBP4\_HUMAN tr|H3BNX5|H3BNX5\_HUMAN tr|A0A087WZ03|A0A087WZ03\_HUMAN sp|Q9UJ14|GGT7\_HUMAN tr|J3KQJ9|J3KQJ9\_HUMAN sp|Q9C0H9|SRCN1\_HUMAN sp|P43080|GUC1A\_HUMAN tr|A6NP16|A6NP16\_HUMAN sp|Q9BTN0|LRFN3\_HUMAN sp|Q96JB2|COG3\_HUMAN sp|Q08AI6|S38AB\_HUMAN tr|B8ZZ86|B8ZZ86\_HUMAN sp|Q96IG2|FXL20\_HUMAN tr|A9Z1X7|A9Z1X7\_HUMAN tr|J3KTA1|J3KTA1\_HUMAN sp|Q8IYB3|SRRM1\_HUMAN sp|B4DYI2|S31C2\_HUMAN tr|B7ZKJ8|B7ZKJ8\_HUMAN tr|Q9UG54|Q9UG54\_HUMAN sp|P43681|ACHA4\_HUMAN sp|Q8IX21|F178A\_HUMAN tr|A0A024R0K5|A0A024R0K5\_HUMAN sp|P06731|CEAM5\_HUMAN sp|P54753|EPHB3\_HUMAN sp|P0C7V9|ME15P\_HUMAN sp|A6NJ78|MET15\_HUMAN tr|H3BMX3|H3BMX3\_HUMAN tr|H3BTJ0|H3BTJ0\_HUMAN tr|H3BT33|H3BT33\_HUMAN tr|H3BVB7|H3BVB7\_HUMAN sp|Q15390|MTFR1\_HUMAN sp|Q8NGX6|O10R2\_HUMAN tr|E5RJS5|E5RJS5\_HUMAN sp|O95239|KIF4A\_HUMAN sp|P24385|CCND1\_HUMAN sp|Q96HV5|TM41A\_HUMAN sp|A0PJE2|DHR12\_HUMAN tr|B4DEW4|B4DEW4\_HUMAN tr|Q86SW4|Q86SW4\_HUMAN sp|P36957|ODO2\_HUMAN sp|P31995|FCG2C\_HUMAN tr|H0YGT0|H0YGT0\_HUMAN tr|A0A087WX59|A0A087WX59\_HUMAN tr|A0A087WXE5|A0A087WXE5\_HUMAN tr|A0A096LNV2|A0A096LNV2\_HUMAN sp|Q4KMQ2|ANO6\_HUMAN tr|A0A096LPA8|A0A096LPA8\_HUMAN sp|Q09470|KCNA1\_HUMAN tr|H0Y8M9|H0Y8M9\_HUMAN sp|O95007|OR6B1\_HUMAN tr|H7C298|H7C298\_HUMAN sp|Q8IV48|ERI1\_HUMAN tr|H7C0D9|H7C0D9\_HUMAN sp|O00308|WWP2\_HUMAN tr|H7BYA1|H7BYA1\_HUMAN tr|F6VRR5|F6VRR5\_HUMAN sp|Q9BY77|PDIP3\_HUMAN tr|H0YNG1|H0YNG1\_HUMAN sp|Q8NH76|O56B4\_HUMAN sp|O00571|DDX3X\_HUMAN sp|Q9H6Z4|RANB3\_HUMAN sp|P59923|ZN445\_HUMAN tr|F2Z2T0|F2Z2T0\_HUMAN sp|Q96QB1|RHG07\_HUMAN tr|H7C3Z2|H7C3Z2\_HUMAN tr|Q15624|Q15624\_HUMAN sp|Q9H3W5|LRRN3\_HUMAN sp|Q14643|ITPR1\_HUMAN tr|B8ZZI7|B8ZZI7\_HUMAN tr|J9JID5|J9JID5\_HUMAN tr|E9PBG5|E9PBG5\_HUMAN tr|H0YJT0|H0YJT0\_HUMAN sp|Q9UL36|ZN236\_HUMAN sp|Q8NGN8|OR4A4\_HUMAN tr|S4R3H4|S4R3H4\_HUMAN tr|I3L0G6|I3L0G6\_HUMAN tr|K7EL21|K7EL21\_HUMAN sp|Q5QJ38|TCHL1\_HUMAN tr|H0Y8H0|H0Y8H0\_HUMAN sp|Q9H2K2|TNKS2\_HUMAN sp|Q08ER8|ZN543\_HUMAN tr|Q6P0N6|Q6P0N6\_HUMAN tr|E7ER26|E7ER26\_HUMAN tr|E7EWE5|E7EWE5\_HUMAN sp|P51659|DHB4\_HUMAN sp|Q5T4J0|GCNT6\_HUMAN sp|Q7Z392|TPC11\_HUMAN tr|A8DPD7|A8DPD7\_HUMAN tr|A6NC89|A6NC89\_HUMAN sp|Q15800|MSMO1\_HUMAN sp|O15069|NACAD\_HUMAN sp|O95294|RASL1\_HUMAN sp|Q9Y6Q2|STON1\_HUMAN sp|P48960|CD97\_HUMAN sp|Q9H6X5|CS044\_HUMAN tr|H0YBJ5|H0YBJ5\_HUMAN tr|H7C2J6|H7C2J6\_HUMAN sp|P07951|TPM2\_HUMAN Q3SX28 tr|H0YKX5|H0YKX5\_HUMAN tr|H0YKP3|H0YKP3\_HUMAN tr|Q6ZN40|Q6ZN40\_HUMAN sp|P09493|TPM1\_HUMAN tr|Q5TCU8|Q5TCU8\_HUMAN tr|Q5TCU3|Q5TCU3\_HUMAN tr|H0YL52|H0YL52\_HUMAN sp|O95271|TNKS1\_HUMAN sp|Q9UPP5|K1107\_HUMAN sp|P15814|IGLL1\_HUMAN tr|E9PGN7|E9PGN7\_HUMAN tr|H9KV48|H9KV48\_HUMAN sp|P05155|IC1\_HUMAN tr|F6VZ39|F6VZ39\_HUMAN sp|Q69YW2|STUM\_HUMAN tr|Q5I0G2|Q5I0G2\_HUMAN sp|Q96JK2|DCAF5\_HUMAN sp|O75795|UDB17\_HUMAN sp|Q6ZUT6|CO052\_HUMAN sp|Q9HBT8|Z286A\_HUMAN tr|J3QQM0|J3QQM0\_HUMAN tr|F5H2E6|F5H2E6\_HUMAN sp|Q01826|SATB1\_HUMAN sp|P07205|PGK2\_HUMAN tr|H0YJ91|H0YJ91\_HUMAN tr|B7ZAR1|B7ZAR1\_HUMAN tr|E9PCA1|E9PCA1\_HUMAN sp|Q99608|NECD\_HUMAN sp|P35523|CLCN1\_HUMAN tr|B7Z4Y8|B7Z4Y8\_HUMAN sp|Q96JB3|HIC2\_HUMAN sp|Q9H344|O51I2\_HUMAN tr|D6RGX7|D6RGX7\_HUMAN sp|Q9Y4F9|FA65B\_HUMAN tr|F5GX51|F5GX51\_HUMAN sp|Q8NE18|NSUN7\_HUMAN tr|A0A096LNR9|A0A096LNR9\_HUMAN tr|K7EID0|K7EID0\_HUMAN tr|K7EPU5|K7EPU5\_HUMAN sp|Q9UH92|MLX\_HUMAN sp|Q6ZU64|CC108\_HUMAN sp|Q8TDN6|BRX1\_HUMAN tr|C9JFM4|C9JFM4\_HUMAN tr|C9IYS8|C9IYS8\_HUMAN sp|C9JH25|PRRT4\_HUMAN tr|C9JI90|C9JI90\_HUMAN tr|C9JPG6|C9JPG6\_HUMAN tr|C9JR47|C9JR47\_HUMAN tr|C9J6X5|C9J6X5\_HUMAN tr|C9JTR0|C9JTR0\_HUMAN tr|C9JKG0|C9JKG0\_HUMAN tr|C9K0R8|C9K0R8\_HUMAN sp|O14529|CUX2\_HUMAN sp|Q9Y2E6|DTX4\_HUMAN sp|Q9NW08|RPC2\_HUMAN tr|A0A087WYC1|A0A087WYC1\_HUMAN sp|Q14995|NR1D2\_HUMAN sp|Q6ZMI3|GLDN\_HUMAN sp|Q8IYA8|CCD36\_HUMAN tr|H0YKN8|H0YKN8\_HUMAN tr|H0YNT2|H0YNT2\_HUMAN sp|Q04726|TLE3\_HUMAN sp|Q9BRR8|GPTC1\_HUMAN tr|H0YL70|H0YL70\_HUMAN tr|H7C1W1|H7C1W1\_HUMAN sp|Q11201|SIA4A\_HUMAN sp|Q6IEZ7|OR2T5\_HUMAN sp|Q8NH02|O2T29\_HUMAN sp|Q5T655|CC147\_HUMAN tr|V9GYI8|V9GYI8\_HUMAN tr|A0A087WU25|A0A087WU25\_HUMAN sp|O75064|DEN4B\_HUMAN sp|Q9NY28|GALT8\_HUMAN sp|P11217|PYGM\_HUMAN sp|Q9H4B4|PLK3\_HUMAN sp|Q9BUF5|TBB6\_HUMAN sp|Q9BVP2|GNL3\_HUMAN tr|K7ESM5|K7ESM5\_HUMAN sp|Q8NFJ6|PKR2\_HUMAN tr|G5EA18|G5EA18\_HUMAN tr|E5RJN7|E5RJN7\_HUMAN tr|A0A087WWD8|A0A087WWD8\_HUMAN tr|A0A075B795|A0A075B795\_HUMAN tr|J3KPM9|J3KPM9\_HUMAN sp|P42224|STAT1\_HUMAN sp|O00273|DFFA\_HUMAN tr|E5RHH5|E5RHH5\_HUMAN tr|E2QRF0|E2QRF0\_HUMAN sp|Q9HCE6|ARGAL\_HUMAN tr|H7C163|H7C163\_HUMAN tr|B1AKN8|B1AKN8\_HUMAN tr|B1AKN5|B1AKN5\_HUMAN sp|Q12857|NFIA\_HUMAN sp|Q14896|MYPC3\_HUMAN sp|O43347|MSI1H\_HUMAN sp|Q8NC26|ZN114\_HUMAN sp|P25705|ATPA\_HUMAN sp|P52306|GDS1\_HUMAN tr|D6RHJ0|D6RHJ0\_HUMAN tr|A0A087WY08|A0A087WY08\_HUMAN sp|Q9UPZ6|THS7A\_HUMAN tr|H9KV63|H9KV63\_HUMAN tr|H9KV62|H9KV62\_HUMAN sp|Q86VM9|ZCH18\_HUMAN tr|H0YLX0|H0YLX0\_HUMAN sp|Q96JG8|MAGD4\_HUMAN tr|H7C1L2|H7C1L2\_HUMAN tr|H3BS25|H3BS25\_HUMAN tr|F8VYN3|F8VYN3\_HUMAN tr|H3BPM0|H3BPM0\_HUMAN tr|D6REC3|D6REC3\_HUMAN sp|P27352|IF\_HUMAN tr|C9IZX4|C9IZX4\_HUMAN tr|C9JN62|C9JN62\_HUMAN sp|Q0VD86|INCA1\_HUMAN tr|X6R5A3|X6R5A3\_HUMAN sp|Q6P9H4|CNKR3\_HUMAN sp|Q5KSL6|DGKK\_HUMAN tr|B9TX11|B9TX11\_HUMAN tr|B9TX03|B9TX03\_HUMAN tr|H0Y382|H0Y382\_HUMAN sp|Q9NRD9|DUOX1\_HUMAN sp|Q9H8L6|MMRN2\_HUMAN sp|Q9HCM7|FBSL\_HUMAN sp|Q9NZ01|TECR\_HUMAN sp|P53621|COPA\_HUMAN sp|Q9P246|STIM2\_HUMAN tr|H0Y860|H0Y860\_HUMAN tr|E7EWB6|E7EWB6\_HUMAN sp|Q71RC9|SMIM5\_HUMAN tr|A0A087WZD8|A0A087WZD8\_HUMAN tr|A0A087WW93|A0A087WW93\_HUMAN tr|H7BZX4|H7BZX4\_HUMAN tr|K7EQ21|K7EQ21\_HUMAN tr|K7ENH8|K7ENH8\_HUMAN tr|J3KQ45|J3KQ45\_HUMAN tr|F8W8W7|F8W8W7\_HUMAN tr|B9TX33|B9TX33\_HUMAN sp|Q8IW45|NNRD\_HUMAN tr|A0A087WYW9|A0A087WYW9\_HUMAN sp|O43493|TGON2\_HUMAN sp|O15055|PER2\_HUMAN sp|Q96RD7|PANX1\_HUMAN tr|E9PIP6|E9PIP6\_HUMAN sp|Q7Z3Z3|PIWL3\_HUMAN tr|H0Y799|H0Y799\_HUMAN tr|C9JB25|C9JB25\_HUMAN tr|C9IZU3|C9IZU3\_HUMAN tr|C9JQJ4|C9JQJ4\_HUMAN sp|Q96T21|SEBP2\_HUMAN tr|H3BLW6|H3BLW6\_HUMAN tr|C9J938|C9J938\_HUMAN tr|A0A087X1X6|A0A087X1X6\_HUMAN tr|E9PFH7|E9PFH7\_HUMAN tr|R4GNC1|R4GNC1\_HUMAN tr|H0YKF0|H0YKF0\_HUMAN tr|Q5JXV9|Q5JXV9\_HUMAN sp|Q96SZ4|ZSC10\_HUMAN tr|A0A087WY96|A0A087WY96\_HUMAN sp|Q9NQ66|PLCB1\_HUMAN tr|A0A087WT80|A0A087WT80\_HUMAN sp|Q86V21|AACS\_HUMAN tr|Q9BRW5|Q9BRW5\_HUMAN sp|Q06730|ZN33A\_HUMAN sp|P18433|PTPRA\_HUMAN sp|Q9H015|S22A4\_HUMAN sp|Q9H9P5|UNKL\_HUMAN tr|B7Z673|B7Z673\_HUMAN tr|E9PDK2|E9PDK2\_HUMAN tr|H0YAN8|H0YAN8\_HUMAN sp|A8MYZ6|FOXO6\_HUMAN sp|Q8IYW5|RN168\_HUMAN sp|Q6ZN04|MEX3B\_HUMAN tr|D4PHA4|D4PHA4\_HUMAN sp|Q86WP2|GPBP1\_HUMAN sp|Q8TBM8|DJB14\_HUMAN tr|C9JKD9|C9JKD9\_HUMAN tr|H0Y746|H0Y746\_HUMAN sp|Q9UJY4|GGA2\_HUMAN tr|H7C177|H7C177\_HUMAN sp|Q9Y6D6|BIG1\_HUMAN tr|K7EPT2|K7EPT2\_HUMAN sp|Q5T1A1|DCST2\_HUMAN sp|P10636|TAU\_HUMAN tr|E9PNN8|E9PNN8\_HUMAN tr|A0A075B710|A0A075B710\_HUMAN tr|G3V4V7|G3V4V7\_HUMAN sp|Q08493|PDE4C\_HUMAN sp|Q9H3V2|MS4A5\_HUMAN sp|Q8NH08|O10AC\_HUMAN sp|P13637|AT1A3\_HUMAN sp|Q13586|STIM1\_HUMAN tr|M0R116|M0R116\_HUMAN sp|Q8N0Z3|SPICE\_HUMAN tr|F5GXG5|F5GXG5\_HUMAN sp|Q96KM6|Z512B\_HUMAN tr|E7EPA6|E7EPA6\_HUMAN tr|C9IYM5|C9IYM5\_HUMAN sp|Q8IYN0|ZN100\_HUMAN tr|A0A075B7G6|A0A075B7G6\_HUMAN sp|Q96LI9|CX058\_HUMAN sp|Q3B7T1|EDRF1\_HUMAN tr|A0A087WVZ9|A0A087WVZ9\_HUMAN sp|Q3KR16|PKHG6\_HUMAN sp|Q8N2M8|CLASR\_HUMAN tr|F5H0Q6|F5H0Q6\_HUMAN sp|Q9Y3I1|FBX7\_HUMAN tr|D6RDZ8|D6RDZ8\_HUMAN tr|B5MDN3|B5MDN3\_HUMAN tr|A0A087WVC6|A0A087WVC6\_HUMAN tr|A0A087WTK0|A0A087WTK0\_HUMAN sp|Q12913|PTPRJ\_HUMAN tr|A0A088AWL3|A0A088AWL3\_HUMAN sp|O14733|MP2K7\_HUMAN sp|O75376|NCOR1\_HUMAN sp|Q8NGQ1|OR9G4\_HUMAN tr|B1APK8|B1APK8\_HUMAN tr|D6RBW0|D6RBW0\_HUMAN sp|O75925|PIAS1\_HUMAN sp|Q5T0L3|CA111\_HUMAN sp|A6NFN9|ANKUB\_HUMAN sp|Q9NZI5|GRHL1\_HUMAN sp|Q5VIR6|VPS53\_HUMAN tr|E7EVT8|E7EVT8\_HUMAN sp|Q9H4G0|E41L1\_HUMAN tr|Q9NTY2|Q9NTY2\_HUMAN sp|O94761|RECQ4\_HUMAN tr|K7EKI0|K7EKI0\_HUMAN tr|K7EJ61|K7EJ61\_HUMAN sp|Q92817|EVPL\_HUMAN tr|K7EP66|K7EP66\_HUMAN tr|I3NI25|I3NI25\_HUMAN sp|P06576|ATPB\_HUMAN sp|Q96NB1|FOPNL\_HUMAN sp|A6NN14|ZN729\_HUMAN tr|A0A087X1H7|A0A087X1H7\_HUMAN tr|C9JXK9|C9JXK9\_HUMAN sp|Q8N1H7|S6OS1\_HUMAN tr|Q5SY18|Q5SY18\_HUMAN sp|Q5TYX0|PRAM5\_HUMAN tr|A0A087WVD2|A0A087WVD2\_HUMAN sp|Q6IFG1|O52E8\_HUMAN sp|Q9GZX5|ZN350\_HUMAN tr|Q6N065|Q6N065\_HUMAN sp|Q5VXH4|PRAM6\_HUMAN tr|E9PL17|E9PL17\_HUMAN sp|Q92989|CLP1\_HUMAN tr|F5H0I3|F5H0I3\_HUMAN sp|P35711|SOX5\_HUMAN tr|M0R0J4|M0R0J4\_HUMAN tr|F5H4B6|F5H4B6\_HUMAN sp|Q8IZ83|A16A1\_HUMAN tr|F5GZS6|F5GZS6\_HUMAN sp|Q6UVY6|MOXD1\_HUMAN tr|J9JIC8|J9JIC8\_HUMAN sp|Q6UUV7|CRTC3\_HUMAN sp|O14763|TR10B\_HUMAN tr|C9J3Y0|C9J3Y0\_HUMAN tr|I3L4A4|I3L4A4\_HUMAN tr|J3QS57|J3QS57\_HUMAN tr|I3L4M4|I3L4M4\_HUMAN tr|J3QLG7|J3QLG7\_HUMAN tr|E9PCY5|E9PCY5\_HUMAN sp|Q86SG7|LYG2\_HUMAN sp|Q8N309|LRC43\_HUMAN tr|D6RIS5|D6RIS5\_HUMAN tr|C9JBA4|C9JBA4\_HUMAN tr|C9J4J0|C9J4J0\_HUMAN sp|Q9H3C7|GGNB2\_HUMAN tr|H0YLV5|H0YLV5\_HUMAN tr|V9GYM8|V9GYM8\_HUMAN sp|P01861|IGHG4\_HUMAN sp|O15063|K0355\_HUMAN sp|Q8IYF1|ELOA2\_HUMAN sp|Q6UXD5|SE6L2\_HUMAN sp|Q9BYK8|HELZ2\_HUMAN sp|Q9Y6B7|AP4B1\_HUMAN tr|B1ALD0|B1ALD0\_HUMAN tr|A0A087WXA3|A0A087WXA3\_HUMAN tr|H0Y608|H0Y608\_HUMAN sp|A8MY62|BLML\_HUMAN sp|P17927|CR1\_HUMAN tr|Q5SR44|Q5SR44\_HUMAN tr|E9PDY4|E9PDY4\_HUMAN sp|Q6ZVF9|GRIN3\_HUMAN tr|A0A087WU22|A0A087WU22\_HUMAN tr|A0A087WUW3|A0A087WUW3\_HUMAN sp|A6NGN4|PRA25\_HUMAN sp|H0Y7S4|PRA26\_HUMAN sp|Q9HCU0|CD248\_HUMAN sp|Q8NEC7|GSTCD\_HUMAN tr|H0YB53|H0YB53\_HUMAN sp|O75843|AP1G2\_HUMAN sp|Q5C9Z4|NOM1\_HUMAN sp|Q9UKT9|IKZF3\_HUMAN sp|Q96AY2|EME1\_HUMAN sp|Q12874|SF3A3\_HUMAN tr|H0Y6V3|H0Y6V3\_HUMAN tr|F8WEE6|F8WEE6\_HUMAN tr|H0Y5R1|H0Y5R1\_HUMAN sp|Q00889|PSG6\_HUMAN sp|Q64LD2|WDR25\_HUMAN tr|Q6PEW3|Q6PEW3\_HUMAN tr|J3KRN9|J3KRN9\_HUMAN sp|P17643|TYRP1\_HUMAN tr|K7EKQ0|K7EKQ0\_HUMAN sp|Q9P2Y5|UVRAG\_HUMAN tr|E9PR71|E9PR71\_HUMAN sp|P35680|HNF1B\_HUMAN tr|K7EJS4|K7EJS4\_HUMAN tr|K7EKC8|K7EKC8\_HUMAN tr|D6RHV6|D6RHV6\_HUMAN tr|A8CTZ0|A8CTZ0\_HUMAN sp|Q5T2P9|AGA10\_HUMAN sp|Q3SXY7|LRIT3\_HUMAN sp|Q8WTW3|COG1\_HUMAN tr|A0A087WVI0|A0A087WVI0\_HUMAN sp|Q9NP55|BPIA1\_HUMAN sp|Q8NHS2|AATC2\_HUMAN tr|B7Z8K7|B7Z8K7\_HUMAN sp|O75208|COQ9\_HUMAN sp|Q08AH1|ACSM1\_HUMAN sp|P35913|PDE6B\_HUMAN tr|H3BNT2|H3BNT2\_HUMAN tr|H0Y498|H0Y498\_HUMAN sp|Q9Y608|LRRF2\_HUMAN sp|Q8IV63|VRK3\_HUMAN sp|P50895|BCAM\_HUMAN tr|E5RHJ4|E5RHJ4\_HUMAN tr|A0A087WX26|A0A087WX26\_HUMAN tr|F8WAR6|F8WAR6\_HUMAN tr|F8WER6|F8WER6\_HUMAN sp|O14782|KIF3C\_HUMAN sp|Q8WXD9|CSKI1\_HUMAN tr|H0YD03|H0YD03\_HUMAN sp|P35573|GDE\_HUMAN tr|B9EH20|B9EH20\_HUMAN sp|Q8NGF4|O5AP2\_HUMAN sp|Q8N4S9|MALD2\_HUMAN sp|O71037|ENK19\_HUMAN tr|D6RA09|D6RA09\_HUMAN tr|E7EX44|E7EX44\_HUMAN tr|C9J813|C9J813\_HUMAN tr|E9PGZ1|E9PGZ1\_HUMAN tr|H0Y3A8|H0Y3A8\_HUMAN sp|Q9BT81|SOX7\_HUMAN sp|Q13433|S39A6\_HUMAN tr|A6NN97|A6NN97\_HUMAN tr|B7Z589|B7Z589\_HUMAN sp|P40189|IL6RB\_HUMAN sp|Q3B8N5|PROX2\_HUMAN tr|G3V3G0|G3V3G0\_HUMAN sp|P49765|VEGFB\_HUMAN sp|P56730|NETR\_HUMAN tr|V9GYD3|V9GYD3\_HUMAN sp|Q0VD83|APOBR\_HUMAN sp|Q7L0X0|TRIL\_HUMAN sp|Q9Y4X1|UD2A1\_HUMAN tr|D6RFW5|D6RFW5\_HUMAN sp|Q8IVV2|LOXH1\_HUMAN tr|H7BZ41|H7BZ41\_HUMAN sp|Q9BXJ9|NAA15\_HUMAN sp|O75113|N4BP1\_HUMAN sp|O75023|LIRB5\_HUMAN sp|Q8IV04|TB10C\_HUMAN sp|P12318|FCG2A\_HUMAN sp|Q6UWM7|LCTL\_HUMAN tr|Q8N7N9|Q8N7N9\_HUMAN sp|Q8TD19|NEK9\_HUMAN tr|H0YCB6|H0YCB6\_HUMAN tr|H7C1T9|H7C1T9\_HUMAN tr|V9GY83|V9GY83\_HUMAN sp|P58505|CU058\_HUMAN tr|Q4VXL4|Q4VXL4\_HUMAN sp|Q9UFB7|ZBT47\_HUMAN tr|H0Y3X7|H0Y3X7\_HUMAN sp|P17301|ITA2\_HUMAN sp|Q8N4T4|ARG39\_HUMAN tr|C9JMJ0|C9JMJ0\_HUMAN sp|Q8TC12|RDH11\_HUMAN tr|H7C3I0|H7C3I0\_HUMAN sp|Q96R72|OR4K3\_HUMAN sp|O60427|FADS1\_HUMAN sp|O43734|CIKS\_HUMAN tr|B9A063|B9A063\_HUMAN tr|H7C543|H7C543\_HUMAN sp|Q5T200|ZC3HD\_HUMAN tr|E7ES21|E7ES21\_HUMAN sp|O60810|PRAM4\_HUMAN sp|Q5VWM5|PRAM9\_HUMAN tr|A0A096LNW4|A0A096LNW4\_HUMAN sp|O60813|PRA11\_HUMAN tr|A0A087X198|A0A087X198\_HUMAN tr|A0A087X1J8|A0A087X1J8\_HUMAN tr|A0A087WW85|A0A087WW85\_HUMAN tr|A3QJZ7|A3QJZ7\_HUMAN sp|Q9Y2B4|T53G5\_HUMAN tr|A6PVV2|A6PVV2\_HUMAN sp|A2RUR9|C144A\_HUMAN tr|C9JT67|C9JT67\_HUMAN sp|Q9UBC2|EP15R\_HUMAN tr|H3BQP1|H3BQP1\_HUMAN sp|Q9UEE9|CFDP1\_HUMAN tr|A0A087WZ30|A0A087WZ30\_HUMAN sp|Q8IWV8|UBR2\_HUMAN sp|O14920|IKKB\_HUMAN tr|H0Y4G9|H0Y4G9\_HUMAN sp|O00339|MATN2\_HUMAN tr|H3BPR7|H3BPR7\_HUMAN sp|O75603|GCM2\_HUMAN sp|Q96PR1|KCNC2\_HUMAN tr|E7EM83|E7EM83\_HUMAN tr|H0Y583|H0Y583\_HUMAN sp|Q3SY69|AL1L2\_HUMAN sp|Q9H1V8|S6A17\_HUMAN sp|Q92932|PTPR2\_HUMAN sp|Q8NGP2|OR8J1\_HUMAN sp|P40763|STAT3\_HUMAN tr|C9JHF5|C9JHF5\_HUMAN tr|E9PC74|E9PC74\_HUMAN sp|Q13144|EI2BE\_HUMAN tr|E7ERF0|E7ERF0\_HUMAN tr|E9PJB1|E9PJB1\_HUMAN tr|E9PIT2|E9PIT2\_HUMAN tr|C9JHR8|C9JHR8\_HUMAN tr|E9PMM0|E9PMM0\_HUMAN sp|Q9NXG0|CNTLN\_HUMAN tr|B4DY92|B4DY92\_HUMAN sp|Q17RW2|COOA1\_HUMAN tr|F5H8D7|F5H8D7\_HUMAN tr|F6U341|F6U341\_HUMAN tr|F6VEM6|F6VEM6\_HUMAN tr|F6X9W3|F6X9W3\_HUMAN tr|F6UR09|F6UR09\_HUMAN sp|P56945|BCAR1\_HUMAN sp|Q9H4I2|ZHX3\_HUMAN sp|Q7Z6I6|RHG30\_HUMAN tr|F5H326|F5H326\_HUMAN tr|C9J2C7|C9J2C7\_HUMAN tr|F5H155|F5H155\_HUMAN tr|G3XAP5|G3XAP5\_HUMAN tr|F5GZ11|F5GZ11\_HUMAN tr|F5GWS6|F5GWS6\_HUMAN sp|P04201|MAS\_HUMAN sp|Q96CN9|GCC1\_HUMAN tr|C9JMH9|C9JMH9\_HUMAN sp|Q9Y6F7|CDY2\_HUMAN tr|A0A087X239|A0A087X239\_HUMAN sp|Q8TF50|ZN526\_HUMAN sp|Q9NWW7|CB042\_HUMAN tr|C9J102|C9J102\_HUMAN sp|Q9BZ23|PANK2\_HUMAN sp|Q8WUY9|DEP1B\_HUMAN tr|C9JNI4|C9JNI4\_HUMAN tr|E9PDF2|E9PDF2\_HUMAN sp|O95834|EMAL2\_HUMAN sp|Q02218|ODO1\_HUMAN tr|K7EIK7|K7EIK7\_HUMAN tr|E9PCR7|E9PCR7\_HUMAN sp|Q8WY41|NANO1\_HUMAN tr|J3QLD7|J3QLD7\_HUMAN tr|J3QRH1|J3QRH1\_HUMAN tr|J3QRY0|J3QRY0\_HUMAN sp|O14830|PPE2\_HUMAN sp|P56524|HDAC4\_HUMAN tr|E5RI36|E5RI36\_HUMAN sp|Q8IY50|S35F3\_HUMAN sp|Q96AP7|ESAM\_HUMAN sp|Q8NI77|KI18A\_HUMAN sp|Q14863|PO6F1\_HUMAN sp|Q8TF17|S3TC2\_HUMAN tr|E9PDF1|E9PDF1\_HUMAN sp|P31512|FMO4\_HUMAN tr|B7Z3B9|B7Z3B9\_HUMAN tr|H0YAG1|H0YAG1\_HUMAN sp|Q96JC1|VPS39\_HUMAN tr|F5H1T5|F5H1T5\_HUMAN sp|Q8N129|CNPY4\_HUMAN tr|H3BMJ2|H3BMJ2\_HUMAN sp|Q9UM47|NOTC3\_HUMAN tr|A0A087X0U0|A0A087X0U0\_HUMAN sp|Q99611|SPS2\_HUMAN sp|Q6P2H3|CEP85\_HUMAN tr|H7BZW2|H7BZW2\_HUMAN tr|E5RII0|E5RII0\_HUMAN sp|Q13625|ASPP2\_HUMAN sp|Q96FN5|KIF12\_HUMAN tr|A0A096LPH6|A0A096LPH6\_HUMAN tr|A0A087WX62|A0A087WX62\_HUMAN sp|Q8IYJ1|CPNE9\_HUMAN tr|E7EXA6|E7EXA6\_HUMAN sp|Q8WVB6|CTF18\_HUMAN tr|A0A087WU43|A0A087WU43\_HUMAN sp|P12830|CADH1\_HUMAN tr|A0A087WXI5|A0A087WXI5\_HUMAN tr|A0A087WX17|A0A087WX17\_HUMAN sp|Q6L8Q7|PDE12\_HUMAN tr|S4R3Q8|S4R3Q8\_HUMAN tr|A0A044PY82|A0A044PY82\_HUMAN tr|E7EW39|E7EW39\_HUMAN sp|Q9UKH3|ENK9\_HUMAN tr|I3L448|I3L448\_HUMAN sp|Q5TC12|ATPF1\_HUMAN sp|Q969P0|IGSF8\_HUMAN sp|Q9UKU7|ACAD8\_HUMAN sp|Q14145|KEAP1\_HUMAN sp|A5A3E0|POTEF\_HUMAN sp|A6NKL6|T200C\_HUMAN tr|Q5VT82|Q5VT82\_HUMAN sp|Q9HC56|PCDH9\_HUMAN sp|Q9H7M6|ZSWM4\_HUMAN sp|Q5QJ74|TBCEL\_HUMAN sp|Q96EP1|CHFR\_HUMAN sp|Q9BR26|OCSTP\_HUMAN sp|Q16204|CCDC6\_HUMAN sp|Q8IYM2|SLN12\_HUMAN sp|P35663|CYLC1\_HUMAN sp|Q9NRX5|SERC1\_HUMAN tr|J3QL40|J3QL40\_HUMAN sp|O15375|MOT6\_HUMAN sp|Q13769|THOC5\_HUMAN tr|K7EKN3|K7EKN3\_HUMAN tr|K7EJL6|K7EJL6\_HUMAN tr|E7EVX8|E7EVX8\_HUMAN tr|E7EN72|E7EN72\_HUMAN tr|E7ESA8|E7ESA8\_HUMAN sp|Q8WWY3|PRP31\_HUMAN tr|E7EU94|E7EU94\_HUMAN tr|E7ESX0|E7ESX0\_HUMAN tr|J3KR58|J3KR58\_HUMAN sp|Q7Z736|PKHH3\_HUMAN tr|X6R3U4|X6R3U4\_HUMAN tr|C9JY47|C9JY47\_HUMAN sp|O43520|AT8B1\_HUMAN sp|Q14137|BOP1\_HUMAN tr|E9PJX3|E9PJX3\_HUMAN sp|Q9H361|PABP3\_HUMAN sp|O95671|ASML\_HUMAN tr|H7C360|H7C360\_HUMAN tr|F8W714|F8W714\_HUMAN tr|K7ENB0|K7ENB0\_HUMAN tr|D6RIT8|D6RIT8\_HUMAN sp|O00270|GPR31\_HUMAN tr|H7BZ95|H7BZ95\_HUMAN tr|A6XMH3|A6XMH3\_HUMAN sp|Q9BSJ8|ESYT1\_HUMAN tr|H7C384|H7C384\_HUMAN sp|Q9NZI8|IF2B1\_HUMAN tr|H3BM72|H3BM72\_HUMAN sp|Q9P2K9|PTHD2\_HUMAN tr|Q5JVM6|Q5JVM6\_HUMAN sp|Q5T7N3|KANK4\_HUMAN sp|Q7Z2Y5|NRK\_HUMAN sp|P30203|CD6\_HUMAN sp|P42226|STAT6\_HUMAN tr|E9NSU1|E9NSU1\_HUMAN sp|Q9NXD2|MTMRA\_HUMAN sp|P56270|MAZ\_HUMAN sp|P60201|MYPR\_HUMAN tr|G8JLD5|G8JLD5\_HUMAN tr|Q5JXD3|Q5JXD3\_HUMAN sp|O00429|DNM1L\_HUMAN sp|Q4KMQ1|TPRN\_HUMAN tr|C9J6S2|C9J6S2\_HUMAN tr|E2QRD4|E2QRD4\_HUMAN sp|O00444|PLK4\_HUMAN tr|J3KR82|J3KR82\_HUMAN sp|O95935|TBX18\_HUMAN sp|Q6YP21|KAT3\_HUMAN sp|P34949|MPI\_HUMAN tr|F5GX71|F5GX71\_HUMAN tr|H3BPB8|H3BPB8\_HUMAN tr|A0A087WZV0|A0A087WZV0\_HUMAN sp|P0C671|CF222\_HUMAN tr|K7EPK8|K7EPK8\_HUMAN tr|H0YAK4|H0YAK4\_HUMAN tr|E9PCB6|E9PCB6\_HUMAN sp|P17787|ACHB2\_HUMAN sp|A5D8V7|CC151\_HUMAN sp|Q9UPT6|JIP3\_HUMAN tr|A0A087WYG2|A0A087WYG2\_HUMAN sp|Q8NGA0|OR7G1\_HUMAN tr|J3KRZ8|J3KRZ8\_HUMAN tr|E7EVW1|E7EVW1\_HUMAN sp|Q02040|AK17A\_HUMAN sp|Q6PI73|LIRA6\_HUMAN tr|C9J8U1|C9J8U1\_HUMAN tr|C9JEE0|C9JEE0\_HUMAN sp|Q16842|SIA4B\_HUMAN sp|P08912|ACM5\_HUMAN sp|Q9P0J7|KCMF1\_HUMAN sp|O43324|MCA3\_HUMAN tr|H3BLU5|H3BLU5\_HUMAN sp|Q8IWU6|SULF1\_HUMAN sp|Q9UPW8|UN13A\_HUMAN tr|F8W059|F8W059\_HUMAN tr|A0A087WWR8|A0A087WWR8\_HUMAN tr|H7BXE5|H7BXE5\_HUMAN sp|Q15287|RNPS1\_HUMAN sp|Q8N8Z6|DCBD1\_HUMAN sp|Q8NGG8|OR8B3\_HUMAN tr|B0QYJ7|B0QYJ7\_HUMAN tr|K7ELE3|K7ELE3\_HUMAN sp|Q9P2N6|KANL3\_HUMAN sp|O95466|FMNL\_HUMAN tr|Q5JX62|Q5JX62\_HUMAN sp|Q96T23|RSF1\_HUMAN sp|Q92187|SIA8D\_HUMAN sp|Q9UJ55|MAGL2\_HUMAN tr|H0YDT0|H0YDT0\_HUMAN tr|H0YM82|H0YM82\_HUMAN sp|P35590|TIE1\_HUMAN tr|H0YD48|H0YD48\_HUMAN sp|Q16850|CP51A\_HUMAN sp|P43003|EAA1\_HUMAN tr|A0A087WT87|A0A087WT87\_HUMAN sp|Q9NRM7|LATS2\_HUMAN sp|Q6ZN66|GBP6\_HUMAN tr|E9PF19|E9PF19\_HUMAN tr|H7BYY2|H7BYY2\_HUMAN sp|P0CW27|CC166\_HUMAN tr|K7ELR8|K7ELR8\_HUMAN sp|Q2WGJ6|KLH38\_HUMAN tr|M0QZQ3|M0QZQ3\_HUMAN tr|G3V1B2|G3V1B2\_HUMAN sp|Q8TE76|MORC4\_HUMAN sp|Q96H72|S39AD\_HUMAN tr|E9PNN7|E9PNN7\_HUMAN tr|E9PMH5|E9PMH5\_HUMAN sp|O14511|NRG2\_HUMAN sp|Q96N66|MBOA7\_HUMAN sp|P54829|PTN5\_HUMAN tr|F5GZS7|F5GZS7\_HUMAN tr|H0YFT1|H0YFT1\_HUMAN sp|Q9BX82|ZN471\_HUMAN sp|P41235|HNF4A\_HUMAN sp|Q5DID0|UROL1\_HUMAN tr|A0A087WXV4|A0A087WXV4\_HUMAN tr|D6W573|D6W573\_HUMAN sp|A8MX76|CAN14\_HUMAN sp|A6BM72|MEG11\_HUMAN sp|Q155Q3|DIXC1\_HUMAN tr|D6RIW8|D6RIW8\_HUMAN sp|Q9UNH7|SNX6\_HUMAN sp|Q8TDI7|TMC2\_HUMAN sp|Q8TDN4|CABL1\_HUMAN tr|A0A087WW74|A0A087WW74\_HUMAN sp|Q969V4|TEKT1\_HUMAN tr|B7Z212|B7Z212\_HUMAN sp|Q9UPI3|FLVC2\_HUMAN sp|Q8TB92|HMGC2\_HUMAN tr|H0YDE2|H0YDE2\_HUMAN sp|Q9NQG7|HPS4\_HUMAN tr|A0A087X0F3|A0A087X0F3\_HUMAN tr|G5EA36|G5EA36\_HUMAN tr|A0A096LP26|A0A096LP26\_HUMAN sp|Q8N6Y0|USBP1\_HUMAN sp|Q8WUM0|NU133\_HUMAN tr|Q5QPD1|Q5QPD1\_HUMAN sp|P40306|PSB10\_HUMAN tr|C9J8C4|C9J8C4\_HUMAN tr|H7C1G2|H7C1G2\_HUMAN tr|H0YIA0|H0YIA0\_HUMAN sp|Q15349|KS6A2\_HUMAN tr|B7Z3B5|B7Z3B5\_HUMAN tr|E9PPZ1|E9PPZ1\_HUMAN sp|Q96EU7|C1GLC\_HUMAN sp|Q9Y2U8|MAN1\_HUMAN sp|Q902F9|EN113\_HUMAN sp|Q9H8Y1|VRTN\_HUMAN sp|O43310|CTIF\_HUMAN sp|O15439|MRP4\_HUMAN P28800 tr|C9J2S3|C9J2S3\_HUMAN sp|Q9NZN1|IRPL1\_HUMAN sp|O94983|CMTA2\_HUMAN tr|I3L3W6|I3L3W6\_HUMAN tr|J3KQ61|J3KQ61\_HUMAN sp|Q9H0B3|K1683\_HUMAN tr|E9PFI7|E9PFI7\_HUMAN sp|Q8N3A8|PARP8\_HUMAN sp|O95155|UBE4B\_HUMAN sp|Q5VTE0|EF1A3\_HUMAN tr|A0A087X251|A0A087X251\_HUMAN sp|Q2M2Z5|KIZ\_HUMAN tr|A0A087X0T6|A0A087X0T6\_HUMAN sp|P42356|PI4KA\_HUMAN tr|G3V192|G3V192\_HUMAN tr|G3V1D1|G3V1D1\_HUMAN sp|Q2M3R5|S35G1\_HUMAN sp|Q13177|PAK2\_HUMAN sp|A4D263|CG072\_HUMAN sp|A8K0R7|ZN839\_HUMAN sp|Q6ZTR5|CX022\_HUMAN sp|Q9ULG6|CCPG1\_HUMAN tr|A0A087X010|A0A087X010\_HUMAN tr|F8VVB8|F8VVB8\_HUMAN sp|Q7RTT9|S29A4\_HUMAN sp|Q969G3|SMCE1\_HUMAN sp|P47887|OR1E2\_HUMAN sp|O60911|CATL2\_HUMAN sp|A6NN90|CB081\_HUMAN tr|G3XAA6|G3XAA6\_HUMAN tr|A0A087WXZ4|A0A087WXZ4\_HUMAN tr|E5RJQ4|E5RJQ4\_HUMAN tr|H0Y8M6|H0Y8M6\_HUMAN sp|Q13237|KGP2\_HUMAN tr|H0YJ30|H0YJ30\_HUMAN tr|H0YCB0|H0YCB0\_HUMAN tr|E9PLD1|E9PLD1\_HUMAN tr|H0YKT5|H0YKT5\_HUMAN tr|F8VP97|F8VP97\_HUMAN tr|F5H7D6|F5H7D6\_HUMAN sp|Q96MG2|JSPR1\_HUMAN sp|P23229|ITA6\_HUMAN sp|P17174|AATC\_HUMAN tr|S4R3T2|S4R3T2\_HUMAN sp|Q96SM3|CPXM1\_HUMAN tr|Q8TCC6|Q8TCC6\_HUMAN sp|Q92819|HYAS2\_HUMAN tr|B4E1Z4|B4E1Z4\_HUMAN sp|Q2VIQ3|KIF4B\_HUMAN sp|Q9NS56|TOPRS\_HUMAN tr|D6RBP0|D6RBP0\_HUMAN sp|A6NIV6|LRIQ4\_HUMAN tr|H3BU57|H3BU57\_HUMAN sp|Q8WYA1|BMAL2\_HUMAN sp|Q9NZN4|EHD2\_HUMAN sp|Q96ST3|SIN3A\_HUMAN tr|U3KQS2|U3KQS2\_HUMAN tr|U3KPU7|U3KPU7\_HUMAN sp|O43826|G6PT1\_HUMAN tr|H7C174|H7C174\_HUMAN tr|A0A096LNU0|A0A096LNU0\_HUMAN sp|Q9Y6H5|SNCAP\_HUMAN tr|D6R9G8|D6R9G8\_HUMAN tr|A0A096LNN4|A0A096LNN4\_HUMAN tr|E7ENA2|E7ENA2\_HUMAN sp|Q9HCC9|LST2\_HUMAN sp|Q07075|AMPE\_HUMAN sp|Q05940|VMAT2\_HUMAN tr|H7C0E9|H7C0E9\_HUMAN sp|Q8NH70|O4A16\_HUMAN tr|Q6P4E2|Q6P4E2\_HUMAN sp|Q6UWV6|ENPP7\_HUMAN sp|Q9Y2M5|KLH20\_HUMAN tr|A0A096LNP0|A0A096LNP0\_HUMAN sp|A8MT82|CTLFB\_HUMAN sp|Q8N944|AMER3\_HUMAN tr|H0Y8B3|H0Y8B3\_HUMAN sp|O75508|CLD11\_HUMAN sp|Q9P1Z2|CACO1\_HUMAN tr|E9PGB3|E9PGB3\_HUMAN sp|Q9NYA1|SPHK1\_HUMAN sp|P13497|BMP1\_HUMAN sp|Q8NDV3|SMC1B\_HUMAN sp|Q9UBN7|HDAC6\_HUMAN tr|H7C5B1|H7C5B1\_HUMAN tr|H7C1Z7|H7C1Z7\_HUMAN sp|Q6IA17|SIGIR\_HUMAN tr|C9JFX4|C9JFX4\_HUMAN sp|O43246|CTR4\_HUMAN tr|S4R400|S4R400\_HUMAN tr|H7C274|H7C274\_HUMAN tr|A0A087WVQ6|A0A087WVQ6\_HUMAN tr|C9JK69|C9JK69\_HUMAN sp|Q9NQX7|ITM2C\_HUMAN tr|C9JG41|C9JG41\_HUMAN sp|A6NJJ6|CS067\_HUMAN sp|O43189|PHF1\_HUMAN tr|J3KP97|J3KP97\_HUMAN sp|Q5T9S5|CCD18\_HUMAN tr|E9PFB9|E9PFB9\_HUMAN sp|Q8IZJ1|UNC5B\_HUMAN tr|A0A087WWW4|A0A087WWW4\_HUMAN tr|B3KPY8|B3KPY8\_HUMAN tr|D9ZHQ7|D9ZHQ7\_HUMAN sp|Q8IV77|CNGA4\_HUMAN sp|Q5T890|ER6L2\_HUMAN tr|D9ZHQ6|D9ZHQ6\_HUMAN sp|P49335|PO3F4\_HUMAN sp|Q6P474|PDXD2\_HUMAN tr|F8W719|F8W719\_HUMAN sp|Q96D71|REPS1\_HUMAN tr|K7EJZ9|K7EJZ9\_HUMAN tr|C9JF80|C9JF80\_HUMAN tr|C9JAH2|C9JAH2\_HUMAN tr|H7BXK9|H7BXK9\_HUMAN sp|Q9NP58|ABCB6\_HUMAN sp|Q9HAV0|GBB4\_HUMAN tr|H7C0H2|H7C0H2\_HUMAN tr|B8ZZV3|B8ZZV3\_HUMAN sp|A4FU28|CTGE9\_HUMAN sp|Q8TDY4|ASAP3\_HUMAN sp|Q8NFR7|CC148\_HUMAN tr|Q5SVK3|Q5SVK3\_HUMAN tr|Q5SVJ9|Q5SVJ9\_HUMAN sp|A5D8W1|CG063\_HUMAN sp|Q969J3|L12R1\_HUMAN tr|H3BVI7|H3BVI7\_HUMAN tr|D6RDK8|D6RDK8\_HUMAN sp|Q9UJP4|KLH21\_HUMAN tr|A0A087WT67|A0A087WT67\_HUMAN tr|E9PDT6|E9PDT6\_HUMAN tr|D6RGC6|D6RGC6\_HUMAN sp|O14628|ZN195\_HUMAN sp|Q8N960|CE120\_HUMAN tr|D6REX9|D6REX9\_HUMAN tr|H3BQA7|H3BQA7\_HUMAN tr|H0Y7Q2|H0Y7Q2\_HUMAN sp|Q9H0D2|ZN541\_HUMAN tr|A0A075B6F0|A0A075B6F0\_HUMAN sp|O94813|SLIT2\_HUMAN tr|E9PQT8|E9PQT8\_HUMAN tr|G3V3P8|G3V3P8\_HUMAN tr|X6R3P0|X6R3P0\_HUMAN tr|A0A087WYV5|A0A087WYV5\_HUMAN tr|G3V4B2|G3V4B2\_HUMAN tr|A0A087WY22|A0A087WY22\_HUMAN tr|A0A087X1X2|A0A087X1X2\_HUMAN tr|H0Y5I3|H0Y5I3\_HUMAN tr|M0R2J8|M0R2J8\_HUMAN sp|O95150|TNF15\_HUMAN sp|Q9BSK4|FEM1A\_HUMAN sp|Q6IF82|O4A47\_HUMAN tr|H0YAB5|H0YAB5\_HUMAN tr|H7C0M8|H7C0M8\_HUMAN sp|Q9Y662|HS3SB\_HUMAN sp|P30531|SC6A1\_HUMAN tr|C9J8K5|C9J8K5\_HUMAN tr|D6W648|D6W648\_HUMAN tr|A0A096LNH2|A0A096LNH2\_HUMAN tr|D6RBD3|D6RBD3\_HUMAN tr|K7EJC3|K7EJC3\_HUMAN tr|E9PLC4|E9PLC4\_HUMAN sp|Q92995|UBP13\_HUMAN tr|F8W148|F8W148\_HUMAN sp|P23280|CAH6\_HUMAN tr|A0A087X136|A0A087X136\_HUMAN sp|O75899|GABR2\_HUMAN sp|O43759|SNG1\_HUMAN sp|Q3MIP1|IPIL2\_HUMAN sp|O94842|TOX4\_HUMAN sp|Q09MP3|R51A2\_HUMAN sp|Q9UQN3|CHM2B\_HUMAN tr|D6R938|D6R938\_HUMAN sp|Q9UGP4|LIMD1\_HUMAN sp|P42331|RHG25\_HUMAN tr|C9JRJ5|C9JRJ5\_HUMAN sp|Q86WV6|STING\_HUMAN sp|Q9Y2I6|NINL\_HUMAN sp|Q8IXQ5|KLHL7\_HUMAN sp|Q08629|TICN1\_HUMAN tr|F5H4Q7|F5H4Q7\_HUMAN tr|H7C2K6|H7C2K6\_HUMAN sp|Q03154|ACY1\_HUMAN sp|A1XBS5|F92A1\_HUMAN tr|C9JMV9|C9JMV9\_HUMAN sp|P20062|TCO2\_HUMAN tr|B5MBX2|B5MBX2\_HUMAN tr|E9PRA5|E9PRA5\_HUMAN tr|K4DIB3|K4DIB3\_HUMAN sp|Q9HC44|GPBL1\_HUMAN tr|K7EQV0|K7EQV0\_HUMAN sp|Q9H5Z1|DHX35\_HUMAN sp|Q8NCQ2|CNAS1\_HUMAN tr|Q5THR1|Q5THR1\_HUMAN tr|K7EN40|K7EN40\_HUMAN tr|C9J695|C9J695\_HUMAN tr|C9JDT9|C9JDT9\_HUMAN sp|O00267|SPT5H\_HUMAN sp|C9JLR9|CK095\_HUMAN tr|G3V2I3|G3V2I3\_HUMAN sp|A6NED2|RCCD1\_HUMAN sp|Q8NBN3|TM87A\_HUMAN tr|F5GXP4|F5GXP4\_HUMAN sp|Q9H9T3|ELP3\_HUMAN sp|Q15041|AR6P1\_HUMAN sp|Q68CJ6|SLIP\_HUMAN sp|Q9BYF1|ACE2\_HUMAN tr|B1AKN7|B1AKN7\_HUMAN tr|H0YJD1|H0YJD1\_HUMAN sp|Q86WK6|AMGO1\_HUMAN sp|O43295|SRGP3\_HUMAN sp|Q9BXU0|TEX12\_HUMAN sp|Q9P1P4|TAAR3\_HUMAN tr|H3BT97|H3BT97\_HUMAN sp|A6NDH6|O5H15\_HUMAN sp|Q587J7|TDR12\_HUMAN tr|M0QZM0|M0QZM0\_HUMAN sp|Q96RI1|NR1H4\_HUMAN tr|A0A087WTS8|A0A087WTS8\_HUMAN sp|Q70EK9|UBP51\_HUMAN sp|P38646|GRP75\_HUMAN sp|Q9H8N7|ZN395\_HUMAN sp|A6NIM6|S15A5\_HUMAN tr|V9GZ46|V9GZ46\_HUMAN tr|G3V234|G3V234\_HUMAN sp|Q7Z5Q5|DPOLN\_HUMAN sp|O94986|CE152\_HUMAN sp|Q9UKV0|HDAC9\_HUMAN sp|Q86WN1|FCSD1\_HUMAN tr|E5RHU4|E5RHU4\_HUMAN sp|Q9NQY0|BIN3\_HUMAN sp|Q13402|MYO7A\_HUMAN sp|Q14CB8|RHG19\_HUMAN tr|H7BYV6|H7BYV6\_HUMAN sp|Q8WWL7|CCNB3\_HUMAN tr|H0YEN1|H0YEN1\_HUMAN Q29RQ1 tr|E9PBL8|E9PBL8\_HUMAN sp|Q9H4H8|FA83D\_HUMAN sp|P49915|GUAA\_HUMAN sp|Q9BZK7|TBL1R\_HUMAN tr|J9JIG6|J9JIG6\_HUMAN sp|Q8TAD8|SNIP1\_HUMAN tr|H7C3E5|H7C3E5\_HUMAN tr|H7C189|H7C189\_HUMAN sp|P35900|K1C20\_HUMAN tr|G3V578|G3V578\_HUMAN P35900 sp|Q9UN36|NDRG2\_HUMAN sp|O75420|PERQ1\_HUMAN tr|S4R418|S4R418\_HUMAN sp|Q96JH8|RADIL\_HUMAN sp|Q6UVJ0|SAS6\_HUMAN sp|Q01973|ROR1\_HUMAN tr|H0Y7I6|H0Y7I6\_HUMAN tr|B4E2I7|B4E2I7\_HUMAN sp|P23786|CPT2\_HUMAN sp|P50607|TUB\_HUMAN tr|Q5JP53|Q5JP53\_HUMAN sp|P07437|TBB5\_HUMAN sp|Q8IVM0|CCD50\_HUMAN tr|Q5ST81|Q5ST81\_HUMAN sp|Q9UBG7|RBPJL\_HUMAN tr|E9PQR4|E9PQR4\_HUMAN sp|Q9HBT6|CAD20\_HUMAN tr|A0A087WYN9|A0A087WYN9\_HUMAN sp|Q7Z478|DHX29\_HUMAN sp|O00634|NET3\_HUMAN tr|V9GY05|V9GY05\_HUMAN tr|D6RFH5|D6RFH5\_HUMAN tr|H0Y9K3|H0Y9K3\_HUMAN tr|E9PK47|E9PK47\_HUMAN sp|P06737|PYGL\_HUMAN tr|E7EPJ1|E7EPJ1\_HUMAN tr|E9PLY5|E9PLY5\_HUMAN sp|Q86XE5|HOGA1\_HUMAN tr|A0A075B6R3|A0A075B6R3\_HUMAN sp|Q9NR96|TLR9\_HUMAN sp|Q9P2I0|CPSF2\_HUMAN tr|A0A087WZR0|A0A087WZR0\_HUMAN tr|A0A087WY68|A0A087WY68\_HUMAN tr|H0YJF4|H0YJF4\_HUMAN sp|Q8NGF6|O10W1\_HUMAN tr|H0YM31|H0YM31\_HUMAN sp|O15260|SURF4\_HUMAN tr|C9JPP7|C9JPP7\_HUMAN sp|Q96KN1|FA84B\_HUMAN sp|Q2M1Z3|RHG31\_HUMAN tr|I3L228|I3L228\_HUMAN sp|Q9Y230|RUVB2\_HUMAN tr|F8WE53|F8WE53\_HUMAN tr|F8WAH1|F8WAH1\_HUMAN sp|Q8NDG6|TDRD9\_HUMAN sp|Q9UKT8|FBXW2\_HUMAN sp|Q96A26|F162A\_HUMAN tr|E9PH05|E9PH05\_HUMAN sp|Q7Z410|TMPS9\_HUMAN sp|Q9UBM4|OPT\_HUMAN tr|E9PBK6|E9PBK6\_HUMAN tr|H3BRB1|H3BRB1\_HUMAN sp|P98095|FBLN2\_HUMAN sp|Q9UN70|PCDGK\_HUMAN sp|A5PLK6|RGSL\_HUMAN sp|Q9UGN4|CLM8\_HUMAN tr|C9JZ40|C9JZ40\_HUMAN tr|A8MX17|A8MX17\_HUMAN tr|E9PM90|E9PM90\_HUMAN tr|E9PR04|E9PR04\_HUMAN tr|E9PQR7|E9PQR7\_HUMAN sp|Q9Y2H0|DLGP4\_HUMAN sp|Q8ND30|LIPB2\_HUMAN tr|H0YDV2|H0YDV2\_HUMAN sp|Q9H4A9|DPEP2\_HUMAN sp|Q8IWD5|MFS6L\_HUMAN sp|Q9Y6M7|S4A7\_HUMAN tr|H0Y6V5|H0Y6V5\_HUMAN tr|E5RGR3|E5RGR3\_HUMAN tr|E9PFN4|E9PFN4\_HUMAN sp|O75365|TP4A3\_HUMAN tr|H0Y6I0|H0Y6I0\_HUMAN tr|A0A075B6N2|A0A075B6N2\_HUMAN sp|P43116|PE2R2\_HUMAN tr|G5E9X1|G5E9X1\_HUMAN sp|Q6UUV9|CRTC1\_HUMAN tr|H0Y6N5|H0Y6N5\_HUMAN tr|G3V2A3|G3V2A3\_HUMAN sp|Q9Y4W6|AFG32\_HUMAN sp|P11216|PYGB\_HUMAN Q9NSB4 sp|Q8N813|CC056\_HUMAN sp|Q9NSB4|KRT82\_HUMAN sp|O15553|MEFV\_HUMAN sp|P59827|BPIB4\_HUMAN tr|K7ELX0|K7ELX0\_HUMAN tr|Q5T980|Q5T980\_HUMAN tr|E7EQN5|E7EQN5\_HUMAN tr|I3L1D5|I3L1D5\_HUMAN sp|O15296|LX15B\_HUMAN tr|C9J119|C9J119\_HUMAN sp|Q8WTR7|ZN473\_HUMAN sp|Q6ZWE6|PKHM3\_HUMAN tr|F8WEC7|F8WEC7\_HUMAN sp|Q9UHG3|PCYOX\_HUMAN tr|H7C5F9|H7C5F9\_HUMAN tr|Q59G71|Q59G71\_HUMAN sp|Q9P2C4|TM181\_HUMAN tr|E9PFF2|E9PFF2\_HUMAN tr|A0A087WU31|A0A087WU31\_HUMAN tr|E9PE15|E9PE15\_HUMAN sp|P51790|CLCN3\_HUMAN tr|F8W888|F8W888\_HUMAN tr|J3KR88|J3KR88\_HUMAN sp|Q53FP2|TMM35\_HUMAN tr|J3KR90|J3KR90\_HUMAN sp|Q9Y5Q5|CORIN\_HUMAN tr|A0A087X1D5|A0A087X1D5\_HUMAN sp|A6NMV5|PRA23\_HUMAN tr|A0A087WWD0|A0A087WWD0\_HUMAN tr|E7ER60|E7ER60\_HUMAN sp|Q8NI08|NCOA7\_HUMAN sp|Q96MT1|RN145\_HUMAN tr|J3QSG6|J3QSG6\_HUMAN tr|E9PJK9|E9PJK9\_HUMAN sp|O95905|SGT1\_HUMAN tr|E9PM43|E9PM43\_HUMAN tr|E9PJC7|E9PJC7\_HUMAN sp|P27701|CD82\_HUMAN tr|E9PJ45|E9PJ45\_HUMAN tr|E9PJB7|E9PJB7\_HUMAN tr|H0Y867|H0Y867\_HUMAN sp|Q96T51|RUFY1\_HUMAN sp|Q5VV42|CDKAL\_HUMAN tr|H0Y8E4|H0Y8E4\_HUMAN tr|E9PH70|E9PH70\_HUMAN tr|E9PKU4|E9PKU4\_HUMAN tr|H0Y871|H0Y871\_HUMAN tr|F5GXT3|F5GXT3\_HUMAN sp|Q8NCE2|MTMRE\_HUMAN sp|Q96LA5|FCRL2\_HUMAN tr|H0YHK3|H0YHK3\_HUMAN sp|O75334|LIPA2\_HUMAN sp|O95405|ZFYV9\_HUMAN tr|F8W0P6|F8W0P6\_HUMAN tr|F8VZH8|F8VZH8\_HUMAN sp|Q6IEU7|OR5MA\_HUMAN sp|Q8NGP8|OR5M1\_HUMAN sp|P10828|THB\_HUMAN tr|H0Y4R3|H0Y4R3\_HUMAN tr|J3QL36|J3QL36\_HUMAN sp|O76021|RL1D1\_HUMAN tr|A0A087X0R7|A0A087X0R7\_HUMAN sp|Q969I6|S38A4\_HUMAN sp|Q9H4L4|SENP3\_HUMAN tr|J3QSV6|J3QSV6\_HUMAN tr|J3QLE6|J3QLE6\_HUMAN tr|J3KRI5|J3KRI5\_HUMAN tr|X6R3Y6|X6R3Y6\_HUMAN sp|P55286|CADH8\_HUMAN tr|E9PRV7|E9PRV7\_HUMAN tr|M9MMK8|M9MMK8\_HUMAN sp|P18887|XRCC1\_HUMAN sp|Q9C0C7|AMRA1\_HUMAN tr|H3BTN5|H3BTN5\_HUMAN sp|Q8NGH5|O56A1\_HUMAN sp|Q8NGH8|O56A4\_HUMAN sp|Q9NP91|S6A20\_HUMAN tr|H0Y742|H0Y742\_HUMAN tr|A0A087WZC1|A0A087WZC1\_HUMAN tr|H8Y6P7|H8Y6P7\_HUMAN sp|Q9BVM2|DPCD\_HUMAN tr|Q5JQQ4|Q5JQQ4\_HUMAN sp|A2A288|ZC12D\_HUMAN sp|Q86UP0|CAD24\_HUMAN tr|B7Z1G8|B7Z1G8\_HUMAN sp|Q69384|ENK6\_HUMAN tr|Q5SX87|Q5SX87\_HUMAN sp|Q6IE81|JADE1\_HUMAN tr|H0Y9S8|H0Y9S8\_HUMAN sp|Q96CW9|NTNG2\_HUMAN tr|A6NF31|A6NF31\_HUMAN tr|K7EQG2|K7EQG2\_HUMAN sp|Q9UPN7|PP6R1\_HUMAN sp|Q96JT2|S45A3\_HUMAN sp|Q13415|ORC1\_HUMAN tr|E9PIR1|E9PIR1\_HUMAN tr|E9PPG8|E9PPG8\_HUMAN sp|Q6ZUM4|RHG27\_HUMAN sp|Q9UL63|MKLN1\_HUMAN sp|Q9N2K0|ENH1\_HUMAN sp|Q8WWW0|RASF5\_HUMAN tr|Q4VXT4|Q4VXT4\_HUMAN tr|Q4VXT5|Q4VXT5\_HUMAN tr|Q4VXT6|Q4VXT6\_HUMAN tr|H0Y386|H0Y386\_HUMAN sp|P40197|GPV\_HUMAN tr|B1AN89|B1AN89\_HUMAN sp|A4D126|ISPD\_HUMAN sp|Q8NDI1|EHBP1\_HUMAN tr|A0A096LPI3|A0A096LPI3\_HUMAN sp|Q96EW2|HBAP1\_HUMAN tr|F5H442|F5H442\_HUMAN tr|H0Y394|H0Y394\_HUMAN sp|Q9Y215|COLQ\_HUMAN sp|Q99816|TS101\_HUMAN sp|Q9NV70|EXOC1\_HUMAN sp|Q8TAM1|BBS10\_HUMAN sp|P28356|HXD9\_HUMAN tr|H7C2K3|H7C2K3\_HUMAN sp|O75173|ATS4\_HUMAN sp|Q9NVW2|RNF12\_HUMAN sp|P30456|1A43\_HUMAN sp|P30450|1A26\_HUMAN tr|G3V119|G3V119\_HUMAN sp|P78539|SRPX\_HUMAN tr|F8WDH9|F8WDH9\_HUMAN sp|Q9UN88|GBRT\_HUMAN tr|E7EN65|E7EN65\_HUMAN tr|E7ENM0|E7ENM0\_HUMAN sp|Q9P0V3|SH3B4\_HUMAN tr|E9PH82|E9PH82\_HUMAN tr|H0Y626|H0Y626\_HUMAN sp|P58400|NRX1B\_HUMAN tr|I3L4D6|I3L4D6\_HUMAN sp|Q9HAV5|TNR27\_HUMAN sp|P13762|DRB4\_HUMAN tr|H7BZH1|H7BZH1\_HUMAN sp|Q13562|NDF1\_HUMAN sp|Q02094|RHAG\_HUMAN sp|A8MTY0|ZN724\_HUMAN tr|M0R287|M0R287\_HUMAN P00978 tr|H7C413|H7C413\_HUMAN sp|Q99519|NEUR1\_HUMAN tr|H0YA27|H0YA27\_HUMAN tr|F2Z2X2|F2Z2X2\_HUMAN sp|Q8NDM7|CFA43\_HUMAN tr|F8W145|F8W145\_HUMAN tr|H3BN66|H3BN66\_HUMAN sp|Q8N4N8|KIF2B\_HUMAN sp|Q16821|PPR3A\_HUMAN sp|Q8NHP8|PLBL2\_HUMAN sp|Q92994|TF3B\_HUMAN tr|U3KQA5|U3KQA5\_HUMAN sp|A6NH11|GLTD2\_HUMAN tr|H7C3L1|H7C3L1\_HUMAN tr|H7BXL6|H7BXL6\_HUMAN sp|Q13049|TRI32\_HUMAN tr|C9JVQ0|C9JVQ0\_HUMAN sp|Q9BX26|SYCP2\_HUMAN tr|B1AMG5|B1AMG5\_HUMAN tr|G3V198|G3V198\_HUMAN tr|E9PR16|E9PR16\_HUMAN sp|Q12769|NU160\_HUMAN tr|F5H581|F5H581\_HUMAN sp|Q9UKK3|PARP4\_HUMAN tr|E9PKH2|E9PKH2\_HUMAN tr|A0A087WUF0|A0A087WUF0\_HUMAN sp|P40967|PMEL\_HUMAN sp|P0C604|OR4A8\_HUMAN sp|Q15399|TLR1\_HUMAN sp|Q8WYA6|CTBL1\_HUMAN sp|Q86V42|F124A\_HUMAN sp|Q9HCG8|CWC22\_HUMAN tr|B7WP74|B7WP74\_HUMAN sp|Q08AM6|VAC14\_HUMAN sp|Q9NVM4|ANM7\_HUMAN sp|O14977|AZIN1\_HUMAN sp|Q8N8W4|PLPL1\_HUMAN tr|H0Y8V7|H0Y8V7\_HUMAN sp|Q8NGT2|O13J1\_HUMAN tr|F5GZ90|F5GZ90\_HUMAN sp|Q9NZJ0|DTL\_HUMAN tr|B1AL16|B1AL16\_HUMAN tr|F8VRH0|F8VRH0\_HUMAN tr|F5H7X4|F5H7X4\_HUMAN sp|Q14651|PLSI\_HUMAN tr|H0Y2V1|H0Y2V1\_HUMAN sp|P25208|NFYB\_HUMAN tr|A0A087WW73|A0A087WW73\_HUMAN tr|Q5T241|Q5T241\_HUMAN tr|D6REM6|D6REM6\_HUMAN tr|F6U1T9|F6U1T9\_HUMAN tr|D3YTA9|D3YTA9\_HUMAN sp|P63098|CANB1\_HUMAN sp|Q9NPH9|IL26\_HUMAN sp|Q902F8|ENK8\_HUMAN tr|B7Z4W5|B7Z4W5\_HUMAN sp|O60763|USO1\_HUMAN sp|Q16773|KAT1\_HUMAN sp|P24903|CP2F1\_HUMAN tr|W4VSQ3|W4VSQ3\_HUMAN tr|A0A087X146|A0A087X146\_HUMAN tr|F5H534|F5H534\_HUMAN sp|Q8N7X4|MAGB6\_HUMAN sp|Q96H15|TIMD4\_HUMAN tr|A6NKZ2|A6NKZ2\_HUMAN sp|P51606|RENBP\_HUMAN sp|Q99985|SEM3C\_HUMAN sp|P02671|FIBA\_HUMAN sp|Q02297|NRG1\_HUMAN tr|H0Y6S1|H0Y6S1\_HUMAN sp|Q7L0J3|SV2A\_HUMAN tr|H7BZJ7|H7BZJ7\_HUMAN sp|Q9NS62|THSD1\_HUMAN sp|P09327|VILI\_HUMAN sp|Q6ZMZ0|RN19B\_HUMAN tr|H0Y413|H0Y413\_HUMAN sp|O43900|PRIC3\_HUMAN tr|H0YE75|H0YE75\_HUMAN sp|Q8NGZ5|OR2G2\_HUMAN tr|A0A087X2B7|A0A087X2B7\_HUMAN Q0V8M9 sp|Q96QT6|PHF12\_HUMAN sp|Q8IX30|SCUB3\_HUMAN sp|Q9H7S9|ZN703\_HUMAN tr|E9PFW3|E9PFW3\_HUMAN sp|Q96CW1|AP2M1\_HUMAN tr|C9JN07|C9JN07\_HUMAN tr|A2A306|A2A306\_HUMAN tr|E7EQT3|E7EQT3\_HUMAN sp|Q12774|ARHG5\_HUMAN sp|Q9H340|O51B6\_HUMAN sp|Q96MT7|CFA44\_HUMAN tr|H7BY83|H7BY83\_HUMAN tr|E9PDL8|E9PDL8\_HUMAN sp|Q9HB55|CP343\_HUMAN sp|P11586|C1TC\_HUMAN sp|Q16676|FOXD1\_HUMAN sp|O95154|ARK73\_HUMAN tr|A8MXZ9|A8MXZ9\_HUMAN sp|Q86WS4|CL040\_HUMAN sp|Q9NRL3|STRN4\_HUMAN sp|P26374|RAE2\_HUMAN sp|Q6NUS6|TECT3\_HUMAN sp|Q9H808|TLE6\_HUMAN sp|Q56UN5|M3K19\_HUMAN sp|Q7Z2W7|TRPM8\_HUMAN tr|F5H3Q5|F5H3Q5\_HUMAN tr|A0A087WZY5|A0A087WZY5\_HUMAN tr|A0A075B7B4|A0A075B7B4\_HUMAN tr|J3KNA0|J3KNA0\_HUMAN sp|Q1EHB4|SC5AC\_HUMAN sp|P47972|NPTX2\_HUMAN tr|E9PCH6|E9PCH6\_HUMAN tr|C9JN33|C9JN33\_HUMAN tr|F8VVQ4|F8VVQ4\_HUMAN tr|Q5TCC6|Q5TCC6\_HUMAN sp|Q96FS4|SIPA1\_HUMAN sp|O75822|EIF3J\_HUMAN tr|F6RY50|F6RY50\_HUMAN sp|Q13438|OS9\_HUMAN tr|C9JJ63|C9JJ63\_HUMAN tr|H0YDB9|H0YDB9\_HUMAN tr|A0A087WZL8|A0A087WZL8\_HUMAN sp|P11172|UMPS\_HUMAN tr|B8A595|B8A595\_HUMAN tr|A8K0G1|A8K0G1\_HUMAN tr|B8A597|B8A597\_HUMAN sp|P56706|WNT7B\_HUMAN tr|G5EA25|G5EA25\_HUMAN tr|Q6ZSU2|Q6ZSU2\_HUMAN tr|H7C5M8|H7C5M8\_HUMAN sp|Q9UNK9|ANGE1\_HUMAN sp|Q8IW93|ARHGJ\_HUMAN sp|Q96S66|CLCC1\_HUMAN sp|O00548|DLL1\_HUMAN sp|P08922|ROS1\_HUMAN tr|Q5H8Y1|Q5H8Y1\_HUMAN sp|O95977|S1PR4\_HUMAN tr|F5H081|F5H081\_HUMAN tr|I3L2R4|I3L2R4\_HUMAN sp|P01011|AACT\_HUMAN tr|G3V595|G3V595\_HUMAN tr|H0YBR0|H0YBR0\_HUMAN sp|Q9Y680|FKBP7\_HUMAN tr|F8WDJ0|F8WDJ0\_HUMAN sp|O60481|ZIC3\_HUMAN tr|A9UJP8|A9UJP8\_HUMAN tr|H7C544|H7C544\_HUMAN sp|P78362|SRPK2\_HUMAN sp|P15941|MUC1\_HUMAN sp|O95297|MPZL1\_HUMAN sp|P51570|GALK1\_HUMAN sp|Q2NL68|PRSR3\_HUMAN sp|Q8NB59|SYT14\_HUMAN sp|P34982|OR1D2\_HUMAN tr|A0A087WXC9|A0A087WXC9\_HUMAN sp|Q9Y3A6|TMED5\_HUMAN sp|Q13477|MADCA\_HUMAN tr|A0A087X1M5|A0A087X1M5\_HUMAN tr|H0YH02|H0YH02\_HUMAN tr|A0A087X0U8|A0A087X0U8\_HUMAN tr|A0A087WWC0|A0A087WWC0\_HUMAN sp|Q969H0|FBXW7\_HUMAN tr|B7Z2C8|B7Z2C8\_HUMAN sp|A2A3K4|PTPC1\_HUMAN tr|A0A087WTF0|A0A087WTF0\_HUMAN sp|Q9BPZ2|SPI2B\_HUMAN sp|O43776|SYNC\_HUMAN tr|Q5JZB8|Q5JZB8\_HUMAN tr|F5H0K0|F5H0K0\_HUMAN tr|H0Y6T5|H0Y6T5\_HUMAN sp|P30154|2AAB\_HUMAN tr|H0Y4F5|H0Y4F5\_HUMAN tr|J3KTF1|J3KTF1\_HUMAN tr|K7EQK2|K7EQK2\_HUMAN tr|J3QL06|J3QL06\_HUMAN tr|A0A087WWI4|A0A087WWI4\_HUMAN tr|E9PJ21|E9PJ21\_HUMAN sp|Q9H4B6|SAV1\_HUMAN sp|Q96J94|PIWL1\_HUMAN tr|H3BPS8|H3BPS8\_HUMAN sp|Q6PIV7|S2534\_HUMAN tr|F5H4U6|F5H4U6\_HUMAN sp|Q96GR2|ACBG1\_HUMAN sp|Q15406|NR6A1\_HUMAN sp|P82987|ATL3\_HUMAN sp|O15118|NPC1\_HUMAN sp|Q8N1B4|VPS52\_HUMAN tr|B4DGM3|B4DGM3\_HUMAN sp|P49961|ENTP1\_HUMAN tr|H3BLU4|H3BLU4\_HUMAN sp|Q99687|MEIS3\_HUMAN sp|P05111|INHA\_HUMAN tr|B7WPL0|B7WPL0\_HUMAN sp|Q9NVN3|RIC8B\_HUMAN sp|Q6ZSI9|CAN12\_HUMAN tr|A8MXP9|A8MXP9\_HUMAN tr|M0R3D7|M0R3D7\_HUMAN sp|Q6ZR85|CQ107\_HUMAN sp|Q5VTQ0|TT39B\_HUMAN sp|P0CB48|UBFL6\_HUMAN tr|A0A087WXR8|A0A087WXR8\_HUMAN tr|K7EQI9|K7EQI9\_HUMAN sp|O95455|TGDS\_HUMAN sp|Q9NRS6|SNX15\_HUMAN tr|A0A087WWT6|A0A087WWT6\_HUMAN tr|X6R9N0|X6R9N0\_HUMAN sp|O43272|PROD\_HUMAN sp|Q12794|HYAL1\_HUMAN tr|F5H0Y3|F5H0Y3\_HUMAN sp|P32242|OTX1\_HUMAN sp|Q8NGC4|O10G3\_HUMAN sp|P15502|ELN\_HUMAN tr|H0YI98|H0YI98\_HUMAN sp|Q9UI10|EI2BD\_HUMAN sp|Q13561|DCTN2\_HUMAN tr|A0A087WTA5|A0A087WTA5\_HUMAN tr|E7ERK9|E7ERK9\_HUMAN sp|Q9Y2B1|TMEM5\_HUMAN tr|F5H0U2|F5H0U2\_HUMAN tr|A0A087WU63|A0A087WU63\_HUMAN tr|C9JYB8|C9JYB8\_HUMAN tr|A0A087WWB0|A0A087WWB0\_HUMAN sp|Q8WTU2|SRB4D\_HUMAN tr|G3V3T2|G3V3T2\_HUMAN sp|Q6ZWJ1|STXB4\_HUMAN tr|E7EPP7|E7EPP7\_HUMAN sp|A0MZ66|SHOT1\_HUMAN tr|G3XAG6|G3XAG6\_HUMAN sp|P03915|NU5M\_HUMAN tr|E9PK67|E9PK67\_HUMAN sp|P04920|B3A2\_HUMAN tr|E9PPE7|E9PPE7\_HUMAN tr|E9PNI7|E9PNI7\_HUMAN sp|Q53GL7|PAR10\_HUMAN tr|K7EQ17|K7EQ17\_HUMAN sp|Q86Y13|DZIP3\_HUMAN sp|Q9NZI2|KCIP1\_HUMAN sp|Q6NT32|EST5A\_HUMAN tr|E9PS82|E9PS82\_HUMAN tr|H7BZD9|H7BZD9\_HUMAN tr|B4DXN4|B4DXN4\_HUMAN sp|Q92830|KAT2A\_HUMAN sp|O95376|ARI2\_HUMAN sp|P22459|KCNA4\_HUMAN sp|O95777|LSM8\_HUMAN tr|F2Z2Y6|F2Z2Y6\_HUMAN sp|P12524|MYCL\_HUMAN tr|C9JNV3|C9JNV3\_HUMAN tr|K7ESP4|K7ESP4\_HUMAN sp|Q8WVC6|DCAKD\_HUMAN tr|E9PMP8|E9PMP8\_HUMAN tr|A6NF51|A6NF51\_HUMAN sp|Q6UWZ7|F175A\_HUMAN sp|O95861|BPNT1\_HUMAN tr|D6REL5|D6REL5\_HUMAN tr|E7EUZ8|E7EUZ8\_HUMAN tr|S4R3K2|S4R3K2\_HUMAN tr|S4R2X0|S4R2X0\_HUMAN sp|Q15059|BRD3\_HUMAN tr|C9J164|C9J164\_HUMAN tr|I3L0I9|I3L0I9\_HUMAN sp|P33991|MCM4\_HUMAN sp|Q9NYY1|IL20\_HUMAN tr|L7N484|L7N484\_HUMAN sp|P23634|AT2B4\_HUMAN tr|F5H6P9|F5H6P9\_HUMAN tr|Q5STZ8|Q5STZ8\_HUMAN sp|Q8NE71|ABCF1\_HUMAN tr|E9PJI0|E9PJI0\_HUMAN sp|Q01968|OCRL\_HUMAN sp|Q9UBT6|POLK\_HUMAN sp|Q9UJT0|TBE\_HUMAN sp|E9PGG2|ANHX\_HUMAN tr|B4DYN2|B4DYN2\_HUMAN sp|Q8IVB4|SL9A9\_HUMAN tr|G3XAL8|G3XAL8\_HUMAN tr|E9PD10|E9PD10\_HUMAN tr|J3QLD9|J3QLD9\_HUMAN sp|Q86U90|YRDC\_HUMAN sp|A0PJZ0|A20A5\_HUMAN tr|B7Z7F3|B7Z7F3\_HUMAN sp|P54577|SYYC\_HUMAN tr|B4DZK5|B4DZK5\_HUMAN tr|J3KMY8|J3KMY8\_HUMAN ENSEMBL:ENSBTAP00000016285 sp|Q9UNG2|TNF18\_HUMAN tr|C9J2Z9|C9J2Z9\_HUMAN sp|Q96FA3|PELI1\_HUMAN sp|Q9HBW1|LRRC4\_HUMAN tr|F8WAH6|F8WAH6\_HUMAN sp|Q13492|PICAL\_HUMAN tr|X6R7M0|X6R7M0\_HUMAN tr|E9PK18|E9PK18\_HUMAN sp|Q5TAH2|SL9C2\_HUMAN sp|P21589|5NTD\_HUMAN tr|Q96B60|Q96B60\_HUMAN sp|Q96PD5|PGRP2\_HUMAN sp|P59541|T2R30\_HUMAN tr|A0A087WVW2|A0A087WVW2\_HUMAN tr|C9JUF9|C9JUF9\_HUMAN sp|P15085|CBPA1\_HUMAN sp|P08473|NEP\_HUMAN sp|Q9UNQ0|ABCG2\_HUMAN sp|Q8NCE0|SEN2\_HUMAN tr|H7C3J9|H7C3J9\_HUMAN sp|Q9BVX2|T106C\_HUMAN tr|A0A087WTK1|A0A087WTK1\_HUMAN tr|C9JUY7|C9JUY7\_HUMAN sp|Q9UBN6|TR10D\_HUMAN tr|B1APP6|B1APP6\_HUMAN tr|V9GY25|V9GY25\_HUMAN sp|Q9UKM9|RALY\_HUMAN sp|Q9Y266|NUDC\_HUMAN sp|Q8NHC6|O14L1\_HUMAN tr|C9J5X9|C9J5X9\_HUMAN tr|E7EWK1|E7EWK1\_HUMAN tr|A0A087WT21|A0A087WT21\_HUMAN sp|O94993|SOX30\_HUMAN sp|P46089|GPR3\_HUMAN tr|E7ENQ1|E7ENQ1\_HUMAN tr|G3XAA2|G3XAA2\_HUMAN sp|O76081|RGS20\_HUMAN tr|H0YI14|H0YI14\_HUMAN tr|H0YBK1|H0YBK1\_HUMAN sp|Q02161|RHD\_HUMAN tr|E7ENZ3|E7ENZ3\_HUMAN tr|I3L252|I3L252\_HUMAN sp|Q9NWF9|RN216\_HUMAN sp|Q8N5C8|TAB3\_HUMAN sp|O43306|ADCY6\_HUMAN sp|Q8TB96|TIP\_HUMAN sp|Q8N5R6|CCD33\_HUMAN sp|Q9H8M2|BRD9\_HUMAN tr|A0A087X0N3|A0A087X0N3\_HUMAN tr|A0A096LP39|A0A096LP39\_HUMAN sp|Q9HCM9|TRI39\_HUMAN sp|Q0D2K0|NIPA4\_HUMAN sp|Q8IYI6|EXOC8\_HUMAN tr|A0A087X143|A0A087X143\_HUMAN sp|Q96SZ6|CK5P1\_HUMAN tr|B0QY83|B0QY83\_HUMAN tr|A0A087WU73|A0A087WU73\_HUMAN sp|Q86UE6|LRRT1\_HUMAN tr|E7ER32|E7ER32\_HUMAN tr|A0A087X287|A0A087X287\_HUMAN sp|Q969V6|MKL1\_HUMAN sp|Q9BXD5|NPL\_HUMAN tr|A0A087X1L3|A0A087X1L3\_HUMAN tr|A0A087WT73|A0A087WT73\_HUMAN sp|Q9BQ08|RETNB\_HUMAN tr|U3KQD0|U3KQD0\_HUMAN sp|Q6ZWJ8|KCP\_HUMAN sp|O43526|KCNQ2\_HUMAN tr|Q4VXP6|Q4VXP6\_HUMAN tr|I3L0U4|I3L0U4\_HUMAN sp|P22059|OSBP1\_HUMAN tr|H0YLE8|H0YLE8\_HUMAN sp|Q9NYZ4|SIGL8\_HUMAN tr|I3L269|I3L269\_HUMAN tr|C9JFB2|C9JFB2\_HUMAN sp|Q9Y548|YIPF1\_HUMAN tr|B9ZVN9|B9ZVN9\_HUMAN sp|O95602|RPA1\_HUMAN sp|P0C7N1|OR8U8\_HUMAN sp|Q92902|HPS1\_HUMAN sp|P35354|PGH2\_HUMAN tr|R4GMY1|R4GMY1\_HUMAN tr|A2TJX0|A2TJX0\_HUMAN sp|Q9Y473|ZN175\_HUMAN sp|Q8NCD3|HJURP\_HUMAN sp|P57740|NU107\_HUMAN tr|C9JKA8|C9JKA8\_HUMAN tr|M0R259|M0R259\_HUMAN sp|Q8IXQ4|GPAM1\_HUMAN sp|P11597|CETP\_HUMAN sp|Q9Y2Q0|AT8A1\_HUMAN sp|Q9Y2J2|E41L3\_HUMAN tr|H0YDD5|H0YDD5\_HUMAN tr|K7EMN8|K7EMN8\_HUMAN tr|F8WEZ8|F8WEZ8\_HUMAN sp|P57058|HUNK\_HUMAN sp|Q7Z7M1|GP144\_HUMAN tr|E5RFM9|E5RFM9\_HUMAN tr|R4GMT7|R4GMT7\_HUMAN sp|Q68CL5|TPGS2\_HUMAN sp|Q9UIQ6|LCAP\_HUMAN tr|E7EMA9|E7EMA9\_HUMAN sp|P82914|RT15\_HUMAN sp|Q9ULJ8|NEB1\_HUMAN sp|Q9NY37|ASIC5\_HUMAN tr|F5H2U4|F5H2U4\_HUMAN sp|Q9BT22|ALG1\_HUMAN tr|C9J266|C9J266\_HUMAN tr|H0Y328|H0Y328\_HUMAN sp|O14966|RAB7L\_HUMAN sp|P01773|HV312\_HUMAN tr|H0Y7G5|H0Y7G5\_HUMAN sp|Q8N9L9|ACOT4\_HUMAN tr|D6REB4|D6REB4\_HUMAN tr|D6RD46|D6RD46\_HUMAN tr|J3KS13|J3KS13\_HUMAN sp|Q96JA4|M4A14\_HUMAN sp|O00755|WNT7A\_HUMAN tr|H7C1V4|H7C1V4\_HUMAN sp|Q86U10|LPP60\_HUMAN tr|M0R055|M0R055\_HUMAN tr|C9JU34|C9JU34\_HUMAN sp|Q63HM9|PLCX3\_HUMAN tr|D6RJH2|D6RJH2\_HUMAN sp|P52739|ZN131\_HUMAN sp|P49753|ACOT2\_HUMAN tr|A0A087X0W7|A0A087X0W7\_HUMAN tr|D6R9I2|D6R9I2\_HUMAN tr|G8JLH9|G8JLH9\_HUMAN tr|A0A087WT95|A0A087WT95\_HUMAN sp|Q3SX64|OD3L2\_HUMAN tr|K7EM76|K7EM76\_HUMAN sp|Q14012|KCC1A\_HUMAN sp|Q15208|STK38\_HUMAN tr|A0A087X0H7|A0A087X0H7\_HUMAN sp|Q9H5V8|CDCP1\_HUMAN sp|P23469|PTPRE\_HUMAN tr|H0YIA8|H0YIA8\_HUMAN tr|C9JST7|C9JST7\_HUMAN sp|O95070|YIF1A\_HUMAN tr|E9PIZ0|E9PIZ0\_HUMAN sp|O14910|LIN7A\_HUMAN sp|Q9BXX3|AN30A\_HUMAN sp|Q8NB12|SMYD1\_HUMAN tr|E9PHG3|E9PHG3\_HUMAN tr|R4GNA2|R4GNA2\_HUMAN tr|Q5W026|Q5W026\_HUMAN tr|H0YI00|H0YI00\_HUMAN tr|F8W0V3|F8W0V3\_HUMAN tr|Q5T6P1|Q5T6P1\_HUMAN tr|C9JUW9|C9JUW9\_HUMAN tr|C9JP03|C9JP03\_HUMAN tr|X6RDJ2|X6RDJ2\_HUMAN tr|C9JD09|C9JD09\_HUMAN tr|H0YM22|H0YM22\_HUMAN tr|C9K0M5|C9K0M5\_HUMAN sp|P29728|OAS2\_HUMAN tr|A0A087X0V5|A0A087X0V5\_HUMAN sp|Q70EK8|UBP53\_HUMAN tr|F5GYF0|F5GYF0\_HUMAN sp|P18827|SDC1\_HUMAN tr|F5H6A7|F5H6A7\_HUMAN tr|F8VS92|F8VS92\_HUMAN sp|P14735|IDE\_HUMAN tr|F5GZZ3|F5GZZ3\_HUMAN tr|H0Y5U2|H0Y5U2\_HUMAN tr|F5H5S9|F5H5S9\_HUMAN tr|F5H772|F5H772\_HUMAN tr|F5H7B4|F5H7B4\_HUMAN tr|A0A087WXZ2|A0A087WXZ2\_HUMAN sp|Q9H1R2|DUS15\_HUMAN tr|H3BQ24|H3BQ24\_HUMAN sp|Q9BZD2|S29A3\_HUMAN tr|G3V5Y5|G3V5Y5\_HUMAN sp|P57789|KCNKA\_HUMAN sp|Q6UX01|LMBRL\_HUMAN tr|A0A087WXH3|A0A087WXH3\_HUMAN tr|I3L2A0|I3L2A0\_HUMAN tr|Q2L696|Q2L696\_HUMAN tr|A0A087WSV8|A0A087WSV8\_HUMAN tr|E9PLE9|E9PLE9\_HUMAN tr|E9PKG6|E9PKG6\_HUMAN sp|P80303|NUCB2\_HUMAN tr|V9HW75|V9HW75\_HUMAN tr|E9PLR0|E9PLR0\_HUMAN sp|P20151|KLK2\_HUMAN tr|H3BSZ6|H3BSZ6\_HUMAN tr|F8WDQ8|F8WDQ8\_HUMAN sp|Q01344|IL5RA\_HUMAN tr|B1APR7|B1APR7\_HUMAN sp|Q9P275|UBP36\_HUMAN tr|A0A075B784|A0A075B784\_HUMAN sp|P31641|SC6A6\_HUMAN sp|Q86U17|SPA11\_HUMAN sp|Q92575|UBXN4\_HUMAN sp|Q9NUV7|SPTC3\_HUMAN tr|F8W8G5|F8W8G5\_HUMAN tr|A0A087WTI6|A0A087WTI6\_HUMAN sp|Q6IEE8|SN12L\_HUMAN sp|Q10981|FUT2\_HUMAN sp|P40199|CEAM6\_HUMAN tr|B1B0G9|B1B0G9\_HUMAN tr|H0Y9I4|H0Y9I4\_HUMAN sp|Q9HCN3|TMM8A\_HUMAN tr|K4DI83|K4DI83\_HUMAN tr|B7Z7Y1|B7Z7Y1\_HUMAN tr|H0YE06|H0YE06\_HUMAN tr|E7EPG3|E7EPG3\_HUMAN tr|E7EWV2|E7EWV2\_HUMAN tr|A8MVK1|A8MVK1\_HUMAN sp|Q8TBE0|BAHD1\_HUMAN sp|Q92797|SYMPK\_HUMAN tr|C9JRP1|C9JRP1\_HUMAN sp|A6NIJ5|F90AK\_HUMAN tr|B8ZZI9|B8ZZI9\_HUMAN tr|B7ZKM0|B7ZKM0\_HUMAN sp|Q15020|SART3\_HUMAN tr|C9J7D0|C9J7D0\_HUMAN sp|Q9UM44|HHLA2\_HUMAN tr|A0A087X0E2|A0A087X0E2\_HUMAN tr|E9PLW3|E9PLW3\_HUMAN tr|Q5JY88|Q5JY88\_HUMAN sp|O95258|UCP5\_HUMAN tr|F5H142|F5H142\_HUMAN tr|H0YBK3|H0YBK3\_HUMAN tr|E7ET17|E7ET17\_HUMAN sp|Q13591|SEM5A\_HUMAN tr|F5H0T9|F5H0T9\_HUMAN sp|P0C7N5|OR8U9\_HUMAN tr|A0A075B6N9|A0A075B6N9\_HUMAN sp|P01871|IGHM\_HUMAN tr|B7Z2Y2|B7Z2Y2\_HUMAN sp|Q14746|COG2\_HUMAN sp|Q9NP85|PODO\_HUMAN sp|Q9BRR6|ADPGK\_HUMAN sp|Q86TZ1|TTC6\_HUMAN sp|Q9P270|SLAI2\_HUMAN sp|O15119|TBX3\_HUMAN sp|Q96EP9|NTCP4\_HUMAN sp|P40123|CAP2\_HUMAN tr|A0A087X0J3|A0A087X0J3\_HUMAN tr|B7Z385|B7Z385\_HUMAN tr|A0A087WZ15|A0A087WZ15\_HUMAN tr|J3KP74|J3KP74\_HUMAN tr|A0A087WWM6|A0A087WWM6\_HUMAN sp|Q76N32|CEP68\_HUMAN sp|Q5SV97|PERM1\_HUMAN tr|X6R647|X6R647\_HUMAN sp|Q6S8J3|POTEE\_HUMAN tr|H0YIW3|H0YIW3\_HUMAN sp|Q9H159|CAD19\_HUMAN tr|X6R456|X6R456\_HUMAN tr|H7C4X8|H7C4X8\_HUMAN tr|H0Y9Z7|H0Y9Z7\_HUMAN sp|Q8IW75|SPA12\_HUMAN sp|O95807|TM50A\_HUMAN sp|Q9BXB5|OSB10\_HUMAN tr|A0A087WZT9|A0A087WZT9\_HUMAN sp|P54802|ANAG\_HUMAN tr|H0Y5T1|H0Y5T1\_HUMAN sp|P23327|SRCH\_HUMAN tr|M0QZ43|M0QZ43\_HUMAN tr|Q5VSN0|Q5VSN0\_HUMAN tr|F5H234|F5H234\_HUMAN sp|P10588|NR2F6\_HUMAN tr|H3BN71|H3BN71\_HUMAN tr|F6WYE2|F6WYE2\_HUMAN tr|H3BUN9|H3BUN9\_HUMAN tr|B4DGE1|B4DGE1\_HUMAN tr|H3BRM7|H3BRM7\_HUMAN tr|H3BR36|H3BR36\_HUMAN sp|A6NNH2|F90AR\_HUMAN tr|H7C0E3|H7C0E3\_HUMAN P34955 tr|B1AMW3|B1AMW3\_HUMAN sp|Q14833|GRM4\_HUMAN sp|P51608|MECP2\_HUMAN tr|A0A087WXF0|A0A087WXF0\_HUMAN sp|A1L020|MEX3A\_HUMAN sp|Q96PF2|TSSK2\_HUMAN tr|M0R2B3|M0R2B3\_HUMAN tr|B1AM15|B1AM15\_HUMAN sp|Q6ZUX3|F179A\_HUMAN sp|Q9H503|BAFL\_HUMAN sp|P51648|AL3A2\_HUMAN sp|Q9HCE9|ANO8\_HUMAN tr|C9J0F2|C9J0F2\_HUMAN tr|C9J0I9|C9J0I9\_HUMAN tr|F6V2D4|F6V2D4\_HUMAN sp|Q8N4F4|S22AO\_HUMAN sp|P06756|ITAV\_HUMAN tr|F2Z2M5|F2Z2M5\_HUMAN tr|E9PQR6|E9PQR6\_HUMAN tr|E9PPS9|E9PPS9\_HUMAN tr|E9PN75|E9PN75\_HUMAN tr|E9PQX0|E9PQX0\_HUMAN sp|Q9NZC4|EHF\_HUMAN sp|Q9UJC3|HOOK1\_HUMAN sp|O95069|KCNK2\_HUMAN sp|Q5T5N4|CF118\_HUMAN sp|O75807|PR15A\_HUMAN tr|A0A087X2A4|A0A087X2A4\_HUMAN tr|E5RFM2|E5RFM2\_HUMAN tr|E5RJF8|E5RJF8\_HUMAN tr|K7EL16|K7EL16\_HUMAN tr|K7EKM6|K7EKM6\_HUMAN tr|K7ERT9|K7ERT9\_HUMAN sp|Q8NA03|FSIP1\_HUMAN tr|K7EMM7|K7EMM7\_HUMAN sp|P98082|DAB2\_HUMAN sp|O15182|CETN3\_HUMAN sp|A2RRH5|WDR27\_HUMAN sp|P36639|8ODP\_HUMAN sp|Q9Y5I1|PCDAB\_HUMAN tr|A0A087WWT8|A0A087WWT8\_HUMAN sp|Q9Y5H5|PCDA9\_HUMAN sp|P0CK96|S352B\_HUMAN sp|O00624|NPT3\_HUMAN sp|Q68D06|SLN13\_HUMAN sp|Q8IYU2|HACE1\_HUMAN tr|K7ESK5|K7ESK5\_HUMAN sp|Q9Y5E9|PCDBE\_HUMAN sp|Q7Z7E8|UB2Q1\_HUMAN tr|U3KQ46|U3KQ46\_HUMAN sp|O14983|AT2A1\_HUMAN sp|Q8NGF7|OR5BH\_HUMAN tr|E9PI31|E9PI31\_HUMAN tr|E9PJ94|E9PJ94\_HUMAN tr|E9PLI4|E9PLI4\_HUMAN tr|E9PJD4|E9PJD4\_HUMAN tr|H3BPY0|H3BPY0\_HUMAN sp|O14815|CAN9\_HUMAN sp|Q6ZMN7|PZRN4\_HUMAN sp|P43629|KI3L1\_HUMAN tr|W5QJC1|W5QJC1\_HUMAN tr|E7ESS6|E7ESS6\_HUMAN tr|W4VSQ6|W4VSQ6\_HUMAN sp|P49418|AMPH\_HUMAN sp|Q9H4L5|OSBL3\_HUMAN sp|A6NKP2|D42E2\_HUMAN sp|Q6PL18|ATAD2\_HUMAN tr|A0A087X1K2|A0A087X1K2\_HUMAN sp|P43246|MSH2\_HUMAN sp|Q9H9P8|L2HDH\_HUMAN tr|G3V5S1|G3V5S1\_HUMAN sp|Q9Y5M8|SRPRB\_HUMAN tr|B4DKB2|B4DKB2\_HUMAN tr|C9JVN9|C9JVN9\_HUMAN sp|P42892|ECE1\_HUMAN tr|G3V272|G3V272\_HUMAN tr|A0A087WYG4|A0A087WYG4\_HUMAN sp|Q6ZUA9|MROH5\_HUMAN sp|Q9H981|ARP8\_HUMAN tr|M0QWZ7|M0QWZ7\_HUMAN sp|Q9NP81|SYSM\_HUMAN tr|M0R2C6|M0R2C6\_HUMAN sp|Q8NE35|CPEB3\_HUMAN tr|C9JR83|C9JR83\_HUMAN sp|Q9H6R6|ZDHC6\_HUMAN sp|A6NGH8|ANR61\_HUMAN tr|H7C4C9|H7C4C9\_HUMAN sp|Q99665|I12R2\_HUMAN sp|Q8IZC4|RTKN2\_HUMAN tr|H7C2Q7|H7C2Q7\_HUMAN sp|A4GXA9|EME2\_HUMAN tr|H3BV62|H3BV62\_HUMAN tr|K7ESB6|K7ESB6\_HUMAN sp|Q494W8|CRFM7\_HUMAN tr|H0Y8R7|H0Y8R7\_HUMAN tr|D6RFZ4|D6RFZ4\_HUMAN tr|E7EUR8|E7EUR8\_HUMAN sp|Q9H2S1|KCNN2\_HUMAN sp|Q8N3G9|TM130\_HUMAN tr|G3V0E7|G3V0E7\_HUMAN sp|P06127|CD5\_HUMAN tr|C9JQ42|C9JQ42\_HUMAN sp|P46976|GLYG\_HUMAN sp|Q9BXP5|SRRT\_HUMAN tr|A6NGQ4|A6NGQ4\_HUMAN tr|C9JQR7|C9JQR7\_HUMAN tr|A0A087WTA0|A0A087WTA0\_HUMAN sp|Q96SN7|ORAI2\_HUMAN tr|A0A087WYD9|A0A087WYD9\_HUMAN tr|A0A087WTC1|A0A087WTC1\_HUMAN sp|O15551|CLD3\_HUMAN tr|M0R1E3|M0R1E3\_HUMAN tr|C9J2H9|C9J2H9\_HUMAN tr|E7EV50|E7EV50\_HUMAN tr|B7Z279|B7Z279\_HUMAN sp|Q8NAC3|I17RC\_HUMAN tr|B4DT68|B4DT68\_HUMAN sp|Q9NPF0|CD320\_HUMAN sp|O14975|S27A2\_HUMAN tr|J3QTB0|J3QTB0\_HUMAN tr|F5H2B5|F5H2B5\_HUMAN sp|Q96BZ4|PLD4\_HUMAN sp|O75364|PITX3\_HUMAN sp|Q9Y5P0|O51B4\_HUMAN tr|B3KT28|B3KT28\_HUMAN tr|A0A087WXM0|A0A087WXM0\_HUMAN sp|Q8WTP8|AEN\_HUMAN sp|Q9P2K8|E2AK4\_HUMAN sp|Q9BT49|THAP7\_HUMAN sp|Q6PJI9|WDR59\_HUMAN sp|Q96AP0|ACD\_HUMAN sp|Q8NHA4|O2AE1\_HUMAN sp|P84996|ALEX\_HUMAN sp|P23193|TCEA1\_HUMAN tr|H3BT10|H3BT10\_HUMAN sp|Q9C0K1|S39A8\_HUMAN sp|P54762|EPHB1\_HUMAN tr|A0A087WVA5|A0A087WVA5\_HUMAN tr|H3BS45|H3BS45\_HUMAN tr|H0YIL7|H0YIL7\_HUMAN tr|B5MEF5|B5MEF5\_HUMAN sp|Q5TEA6|SE1L2\_HUMAN sp|Q9BWW8|APOL6\_HUMAN tr|A0A087X0U3|A0A087X0U3\_HUMAN sp|Q96RP8|KCNA7\_HUMAN sp|Q12972|PP1R8\_HUMAN sp|P15172|MYOD1\_HUMAN sp|Q6J9G0|STYK1\_HUMAN sp|Q9Y2L8|ZKSC5\_HUMAN tr|H3BQJ4|H3BQJ4\_HUMAN sp|Q9ULP0|NDRG4\_HUMAN tr|J3KTF9|J3KTF9\_HUMAN tr|B7Z9X4|B7Z9X4\_HUMAN sp|Q2VYF4|LETM2\_HUMAN tr|H0YHE8|H0YHE8\_HUMAN tr|J3KPE3|J3KPE3\_HUMAN sp|Q6P158|DHX57\_HUMAN sp|P36871|PGM1\_HUMAN sp|Q96MP5|ZSWM3\_HUMAN sp|Q9UDV6|ZN212\_HUMAN tr|A0A087X032|A0A087X032\_HUMAN sp|Q86WI3|NLRC5\_HUMAN tr|A0A087WTH3|A0A087WTH3\_HUMAN sp|Q6WRX3|ZY11A\_HUMAN sp|P05976|MYL1\_HUMAN tr|D6RIG4|D6RIG4\_HUMAN sp|O95639|CPSF4\_HUMAN tr|C9JEV9|C9JEV9\_HUMAN tr|B4DPY1|B4DPY1\_HUMAN sp|Q99680|GPR22\_HUMAN sp|A6NM62|LRC53\_HUMAN tr|B1APY4|B1APY4\_HUMAN tr|F8W6Z2|F8W6Z2\_HUMAN sp|P19440|GGT1\_HUMAN tr|E7ET76|E7ET76\_HUMAN sp|Q13435|SF3B2\_HUMAN tr|E9PPJ0|E9PPJ0\_HUMAN tr|A0A087WZZ5|A0A087WZZ5\_HUMAN sp|Q9UGU5|HMGX4\_HUMAN sp|O95944|NCTR2\_HUMAN sp|Q9Y6F8|CDY1\_HUMAN sp|Q9H1K6|MESD1\_HUMAN tr|H0Y911|H0Y911\_HUMAN tr|G3V0E6|G3V0E6\_HUMAN sp|Q6NZI2|PTRF\_HUMAN tr|H0Y920|H0Y920\_HUMAN sp|P17181|INAR1\_HUMAN sp|Q8TEQ6|GEMI5\_HUMAN sp|Q8IWE2|NXP20\_HUMAN tr|K7EP73|K7EP73\_HUMAN sp|Q8TDZ2|MICA1\_HUMAN sp|Q16854|DGUOK\_HUMAN sp|P54709|AT1B3\_HUMAN sp|Q13309|SKP2\_HUMAN sp|Q7Z7L8|CK096\_HUMAN sp|Q8WXH2|JPH3\_HUMAN sp|Q5H9I0|TFDP3\_HUMAN sp|Q8NBI5|S43A3\_HUMAN sp|Q9NX05|F120C\_HUMAN sp|Q6ZVN8|RGMC\_HUMAN tr|F8W881|F8W881\_HUMAN tr|Q5JSB5|Q5JSB5\_HUMAN sp|Q5TGP6|MROH9\_HUMAN tr|E7EQD7|E7EQD7\_HUMAN tr|H7C3U0|H7C3U0\_HUMAN tr|J3QLV6|J3QLV6\_HUMAN sp|P21917|DRD4\_HUMAN tr|J3KSS5|J3KSS5\_HUMAN sp|Q6QEF8|CORO6\_HUMAN tr|E2QRH1|E2QRH1\_HUMAN sp|A1L443|NTM2F\_HUMAN sp|Q4G0J3|LARP7\_HUMAN tr|E7EX83|E7EX83\_HUMAN tr|C9J840|C9J840\_HUMAN sp|A1L170|CA226\_HUMAN sp|P53420|CO4A4\_HUMAN sp|Q12866|MERTK\_HUMAN sp|Q96KT7|S35G5\_HUMAN sp|Q14139|UBE4A\_HUMAN sp|O60841|IF2P\_HUMAN tr|H7BZZ8|H7BZZ8\_HUMAN tr|A0A087WYK8|A0A087WYK8\_HUMAN tr|B7ZKW8|B7ZKW8\_HUMAN sp|P48637|GSHB\_HUMAN sp|Q6JBY9|CPZIP\_HUMAN tr|C9JK28|C9JK28\_HUMAN sp|Q9H013|ADA19\_HUMAN tr|E7EWS1|E7EWS1\_HUMAN sp|Q9H2E6|SEM6A\_HUMAN tr|B3KU01|B3KU01\_HUMAN tr|H0Y8V9|H0Y8V9\_HUMAN tr|E9PDV9|E9PDV9\_HUMAN sp|Q14978|NOLC1\_HUMAN tr|F5H1H2|F5H1H2\_HUMAN sp|P20848|A1ATR\_HUMAN sp|Q6YBV0|S36A4\_HUMAN tr|C9JLV4|C9JLV4\_HUMAN sp|Q05925|HME1\_HUMAN tr|C9JAA9|C9JAA9\_HUMAN tr|H7C5K5|H7C5K5\_HUMAN sp|Q92838|EDA\_HUMAN tr|X6R8H3|X6R8H3\_HUMAN tr|E7EU09|E7EU09\_HUMAN sp|Q8NDB2|BANK1\_HUMAN sp|Q9Y2L6|FRM4B\_HUMAN tr|E7EQ29|E7EQ29\_HUMAN tr|V9GZ67|V9GZ67\_HUMAN tr|V9GY40|V9GY40\_HUMAN tr|F8WD61|F8WD61\_HUMAN tr|C9JLY8|C9JLY8\_HUMAN sp|Q9NU02|ANKE1\_HUMAN sp|Q9H2X9|S12A5\_HUMAN sp|P54886|P5CS\_HUMAN sp|Q6P179|ERAP2\_HUMAN sp|A8MQ27|NEU1B\_HUMAN sp|Q8ND04|SMG8\_HUMAN sp|P54760|EPHB4\_HUMAN tr|Q96L35|Q96L35\_HUMAN sp|Q96CW5|GCP3\_HUMAN tr|J3KTE1|J3KTE1\_HUMAN tr|H3BTZ0|H3BTZ0\_HUMAN sp|Q8WU67|ABHD3\_HUMAN sp|O14613|BORG1\_HUMAN tr|Q5JU01|Q5JU01\_HUMAN sp|Q86TM3|DDX53\_HUMAN sp|Q9Y573|IPP\_HUMAN tr|A0A075B781|A0A075B781\_HUMAN tr|I3L0A5|I3L0A5\_HUMAN sp|O00206|TLR4\_HUMAN sp|Q15014|MO4L2\_HUMAN sp|Q08830|FGL1\_HUMAN sp|P32322|P5CR1\_HUMAN sp|Q01432|AMPD3\_HUMAN tr|C9JSR1|C9JSR1\_HUMAN tr|E9PKC5|E9PKC5\_HUMAN tr|E9PLK6|E9PLK6\_HUMAN tr|E9PIR5|E9PIR5\_HUMAN sp|Q9NQ88|TIGAR\_HUMAN sp|A6NGR9|MROH6\_HUMAN tr|H7C4V5|H7C4V5\_HUMAN sp|Q86X24|HORM1\_HUMAN tr|D6RBF7|D6RBF7\_HUMAN sp|Q9UBC5|MYO1A\_HUMAN tr|E7EX35|E7EX35\_HUMAN tr|C9JEF1|C9JEF1\_HUMAN tr|C9JHL0|C9JHL0\_HUMAN tr|C9JL39|C9JL39\_HUMAN tr|C9JKQ1|C9JKQ1\_HUMAN sp|Q08188|TGM3\_HUMAN tr|H0YIS7|H0YIS7\_HUMAN tr|F8W1H0|F8W1H0\_HUMAN sp|Q8IXM2|BAP18\_HUMAN tr|A0A024R214|A0A024R214\_HUMAN sp|Q9BZB8|CPEB1\_HUMAN tr|D6RAL0|D6RAL0\_HUMAN tr|H0YEN2|H0YEN2\_HUMAN sp|Q9P2E9|RRBP1\_HUMAN sp|Q2NKJ3|CTC1\_HUMAN sp|Q13286|CLN3\_HUMAN tr|B4DXL3|B4DXL3\_HUMAN tr|A0A087WYH7|A0A087WYH7\_HUMAN sp|Q13011|ECH1\_HUMAN tr|C9J5T4|C9J5T4\_HUMAN sp|O15204|ADEC1\_HUMAN sp|Q06455|MTG8\_HUMAN tr|E7ET40|E7ET40\_HUMAN sp|Q9BWV3|CDAC1\_HUMAN tr|H0Y307|H0Y307\_HUMAN sp|Q8NH72|OR4C6\_HUMAN sp|Q9Y316|MEMO1\_HUMAN sp|Q504Q3|PAN2\_HUMAN sp|O95428|PPN\_HUMAN tr|B5MDP7|B5MDP7\_HUMAN sp|O15530|PDPK1\_HUMAN sp|Q86Y34|GPR97\_HUMAN tr|E9PER6|E9PER6\_HUMAN tr|F5H285|F5H285\_HUMAN tr|F8W8F7|F8W8F7\_HUMAN tr|F5H7P8|F5H7P8\_HUMAN sp|Q99941|ATF6B\_HUMAN sp|O75311|GLRA3\_HUMAN tr|H7C0G1|H7C0G1\_HUMAN sp|P22079|PERL\_HUMAN tr|H0YDF7|H0YDF7\_HUMAN sp|Q15773|MLF2\_HUMAN sp|Q8IWT6|LRC8A\_HUMAN sp|Q9H7Z3|NRDE2\_HUMAN sp|O76082|S22A5\_HUMAN sp|Q5FVE4|ACBG2\_HUMAN sp|Q8NBJ9|SIDT2\_HUMAN tr|F5H8L4|F5H8L4\_HUMAN tr|B4DEM9|B4DEM9\_HUMAN sp|Q52LR7|EPC2\_HUMAN sp|Q9Y2S7|PDIP2\_HUMAN sp|Q7LDG7|GRP2\_HUMAN tr|D6RGC4|D6RGC4\_HUMAN sp|P32314|FOXN2\_HUMAN sp|Q4KMZ1|IQCC\_HUMAN tr|H0Y4K4|H0Y4K4\_HUMAN tr|A0A087WWA7|A0A087WWA7\_HUMAN sp|Q9H900|ZWILC\_HUMAN tr|Q5TAA8|Q5TAA8\_HUMAN tr|A2IDB2|A2IDB2\_HUMAN tr|D6RA70|D6RA70\_HUMAN sp|P27487|DPP4\_HUMAN sp|Q9NPJ1|MKKS\_HUMAN sp|P61201|CSN2\_HUMAN tr|G3V4M9|G3V4M9\_HUMAN tr|B4DIH5|B4DIH5\_HUMAN tr|H0Y9M1|H0Y9M1\_HUMAN tr|K7EQ23|K7EQ23\_HUMAN sp|Q92529|SHC3\_HUMAN sp|Q8N149|LIRA2\_HUMAN tr|K7EN31|K7EN31\_HUMAN tr|F6XBR9|F6XBR9\_HUMAN sp|Q5SSQ6|SAPC1\_HUMAN sp|P78371|TCPB\_HUMAN sp|P40879|S26A3\_HUMAN tr|F5GWF6|F5GWF6\_HUMAN sp|Q9UMX0|UBQL1\_HUMAN tr|F8W7Q4|F8W7Q4\_HUMAN tr|H7C4W0|H7C4W0\_HUMAN sp|Q96PK6|RBM14\_HUMAN tr|H7C4V4|H7C4V4\_HUMAN tr|H0YER1|H0YER1\_HUMAN sp|P20273|CD22\_HUMAN sp|P06213|INSR\_HUMAN tr|H0Y3Y7|H0Y3Y7\_HUMAN sp|P31939|PUR9\_HUMAN sp|Q9BXS5|AP1M1\_HUMAN tr|K7EJL1|K7EJL1\_HUMAN tr|J3QKZ7|J3QKZ7\_HUMAN sp|P09237|MMP7\_HUMAN sp|Q7L5Y1|ENOF1\_HUMAN sp|O15360|FANCA\_HUMAN sp|Q14209|E2F2\_HUMAN sp|Q9H6R7|CB044\_HUMAN sp|O43524|FOXO3\_HUMAN sp|Q8WWT9|S13A3\_HUMAN tr|F1T0D7|F1T0D7\_HUMAN sp|Q9H7E2|TDRD3\_HUMAN sp|Q9UBR4|LHX3\_HUMAN sp|Q02643|GHRHR\_HUMAN sp|Q04756|HGFA\_HUMAN sp|P35368|ADA1B\_HUMAN tr|R4GN84|R4GN84\_HUMAN tr|A0A087WZG2|A0A087WZG2\_HUMAN REFSEQ:XP\_001252647 tr|A0A087WUS3|A0A087WUS3\_HUMAN sp|Q15052|ARHG6\_HUMAN sp|P35270|SPRE\_HUMAN sp|Q9BZE9|ASPC1\_HUMAN tr|B6ZDN3|B6ZDN3\_HUMAN sp|Q969X6|CIR1A\_HUMAN sp|Q8WW52|F151A\_HUMAN tr|H3BSH7|H3BSH7\_HUMAN sp|Q9BX51|GGTL1\_HUMAN sp|Q8N1W2|ZN710\_HUMAN sp|Q6ZSZ6|TSH1\_HUMAN sp|Q9Y657|SPIN1\_HUMAN sp|Q96J66|ABCCB\_HUMAN tr|A0A087WX23|A0A087WX23\_HUMAN sp|P58180|OR4D2\_HUMAN sp|O14508|SOCS2\_HUMAN tr|S4R3W1|S4R3W1\_HUMAN tr|F8VRV3|F8VRV3\_HUMAN sp|Q58F21|BRDT\_HUMAN sp|O43280|TREA\_HUMAN tr|H0YJL6|H0YJL6\_HUMAN tr|H7C548|H7C548\_HUMAN sp|Q9P2K1|C2D2A\_HUMAN sp|P52630|STAT2\_HUMAN tr|A0A087WT38|A0A087WT38\_HUMAN sp|Q6UXZ3|CLM4\_HUMAN sp|Q8NE79|POPD1\_HUMAN tr|A0A087WYC5|A0A087WYC5\_HUMAN tr|B4DSF2|B4DSF2\_HUMAN tr|F8W7X0|F8W7X0\_HUMAN tr|J3QTJ5|J3QTJ5\_HUMAN sp|Q96P44|COLA1\_HUMAN tr|F5GZK2|F5GZK2\_HUMAN tr|A0A087WXD5|A0A087WXD5\_HUMAN tr|H0YGM4|H0YGM4\_HUMAN tr|H0YDB0|H0YDB0\_HUMAN sp|P54725|RD23A\_HUMAN tr|H7BZB4|H7BZB4\_HUMAN sp|Q6ZN54|DEFI8\_HUMAN tr|M0QZX2|M0QZX2\_HUMAN sp|O95400|CD2B2\_HUMAN sp|Q6NUT3|MFS12\_HUMAN sp|A8K0Z3|WASH1\_HUMAN sp|Q5VXU9|CI084\_HUMAN tr|A6PVK7|A6PVK7\_HUMAN sp|Q9NUR3|TM74B\_HUMAN tr|C9JGF5|C9JGF5\_HUMAN tr|Q86YH7|Q86YH7\_HUMAN tr|C9JZ79|C9JZ79\_HUMAN tr|C9JBC2|C9JBC2\_HUMAN tr|C9JVZ1|C9JVZ1\_HUMAN sp|O15091|MRRP3\_HUMAN sp|O94850|DEND\_HUMAN sp|P60852|ZP1\_HUMAN sp|Q3KP44|ANR55\_HUMAN sp|Q9UKJ1|PILRA\_HUMAN sp|P49748|ACADV\_HUMAN sp|Q9H0F6|SHRPN\_HUMAN tr|E7ET07|E7ET07\_HUMAN sp|Q9NXR8|ING3\_HUMAN sp|Q8N9C0|IGS22\_HUMAN tr|Q86XE2|Q86XE2\_HUMAN sp|H3BV60|TGR3L\_HUMAN tr|H0Y894|H0Y894\_HUMAN tr|K7ERU8|K7ERU8\_HUMAN tr|M0QYZ3|M0QYZ3\_HUMAN tr|H7C265|H7C265\_HUMAN tr|H7C5R6|H7C5R6\_HUMAN sp|P03928|ATP8\_HUMAN sp|Q9UKA2|FBXL4\_HUMAN sp|Q9NQ90|ANO2\_HUMAN tr|Q5JUQ1|Q5JUQ1\_HUMAN sp|P06340|DOA\_HUMAN tr|Q5JUQ2|Q5JUQ2\_HUMAN sp|Q2TAC6|KIF19\_HUMAN sp|P49221|TGM4\_HUMAN sp|Q13523|PRP4B\_HUMAN sp|Q9H329|E41LB\_HUMAN sp|Q14242|SELPL\_HUMAN sp|Q9HCG1|ZN160\_HUMAN tr|F5H126|F5H126\_HUMAN tr|H3BUQ1|H3BUQ1\_HUMAN tr|F5H1N2|F5H1N2\_HUMAN tr|M0QZI7|M0QZI7\_HUMAN sp|Q7Z624|CMKMT\_HUMAN tr|B5MC16|B5MC16\_HUMAN tr|B5MC79|B5MC79\_HUMAN tr|F5H2T0|F5H2T0\_HUMAN sp|Q8N6S5|AR6P6\_HUMAN sp|Q96DR7|ARHGQ\_HUMAN tr|H0YJ97|H0YJ97\_HUMAN sp|P07359|GP1BA\_HUMAN sp|O15211|RGL2\_HUMAN tr|Q9BR54|Q9BR54\_HUMAN tr|B7Z676|B7Z676\_HUMAN sp|P55327|TPD52\_HUMAN tr|F5H5D3|F5H5D3\_HUMAN sp|Q9BQE3|TBA1C\_HUMAN sp|Q8NDV7|TNR6A\_HUMAN tr|F8VYK4|F8VYK4\_HUMAN sp|Q96D31|CRCM1\_HUMAN tr|Q5SQY0|Q5SQY0\_HUMAN tr|D6RAX3|D6RAX3\_HUMAN tr|D6R9R7|D6R9R7\_HUMAN tr|C9J8E7|C9J8E7\_HUMAN tr|Q5T0C7|Q5T0C7\_HUMAN tr|A0A087WVS3|A0A087WVS3\_HUMAN sp|A6NF01|P121B\_HUMAN tr|A0A087WVW4|A0A087WVW4\_HUMAN sp|O43548|TGM5\_HUMAN sp|Q2M385|MPEG1\_HUMAN tr|I3L3Q8|I3L3Q8\_HUMAN sp|Q99965|ADAM2\_HUMAN sp|Q96KT0|FAAS1\_HUMAN tr|Q6P2G0|Q6P2G0\_HUMAN sp|Q8N1G2|CMTR1\_HUMAN sp|Q9NVH1|DJC11\_HUMAN tr|E9PBP6|E9PBP6\_HUMAN sp|P55157|MTP\_HUMAN sp|Q8N5C1|FA26E\_HUMAN sp|Q9NQW7|XPP1\_HUMAN tr|E9PEZ5|E9PEZ5\_HUMAN tr|M0R261|M0R261\_HUMAN sp|O95336|6PGL\_HUMAN sp|Q9UIK5|TEFF2\_HUMAN sp|Q6ZNL6|FGD5\_HUMAN tr|B7ZM68|B7ZM68\_HUMAN tr|H0YHL6|H0YHL6\_HUMAN tr|H0YI94|H0YI94\_HUMAN sp|Q96II8|LRCH3\_HUMAN sp|P36544|ACHA7\_HUMAN tr|H3BV90|H3BV90\_HUMAN sp|Q7Z3J2|CP062\_HUMAN tr|F5H7K1|F5H7K1\_HUMAN tr|E7EWW0|E7EWW0\_HUMAN tr|H3BQZ7|H3BQZ7\_HUMAN sp|Q1KMD3|HNRL2\_HUMAN tr|H3BSV5|H3BSV5\_HUMAN tr|E9PAK5|E9PAK5\_HUMAN tr|H3BMG9|H3BMG9\_HUMAN sp|Q5VV41|ARHGG\_HUMAN sp|Q8NGI2|O52N4\_HUMAN tr|A0A087WUH1|A0A087WUH1\_HUMAN sp|Q7Z4K8|TRI46\_HUMAN tr|F5GYK0|F5GYK0\_HUMAN sp|P49675|STAR\_HUMAN sp|O14773|TPP1\_HUMAN tr|A0A087WVC5|A0A087WVC5\_HUMAN sp|Q9NSG2|CA112\_HUMAN tr|H7C3C4|H7C3C4\_HUMAN sp|Q9H2K8|TAOK3\_HUMAN sp|P09848|LPH\_HUMAN sp|Q6H3X3|RET1G\_HUMAN tr|B1AKP8|B1AKP8\_HUMAN sp|Q9Y2P5|S27A5\_HUMAN sp|Q9H0H5|RGAP1\_HUMAN sp|Q02078|MEF2A\_HUMAN sp|Q9UEG4|ZN629\_HUMAN sp|Q9BQF6|SENP7\_HUMAN sp|Q8WWB7|NCUG1\_HUMAN tr|A0A087X0W3|A0A087X0W3\_HUMAN sp|Q15904|VAS1\_HUMAN tr|E5RFG2|E5RFG2\_HUMAN sp|Q99836|MYD88\_HUMAN tr|H0YKH9|H0YKH9\_HUMAN tr|H0YLU3|H0YLU3\_HUMAN sp|P49759|CLK1\_HUMAN sp|Q9Y672|ALG6\_HUMAN sp|Q14943|KI3S1\_HUMAN tr|H0YHI9|H0YHI9\_HUMAN tr|H0Y5X5|H0Y5X5\_HUMAN sp|Q07890|SOS2\_HUMAN tr|A0A087WVT3|A0A087WVT3\_HUMAN sp|Q96H55|MYO19\_HUMAN sp|B7Z1M9|C2D4D\_HUMAN tr|F5H563|F5H563\_HUMAN sp|Q9Y5N1|HRH3\_HUMAN tr|H0YCP8|H0YCP8\_HUMAN sp|P16452|EPB42\_HUMAN sp|Q96FC9|DDX11\_HUMAN tr|G5EA30|G5EA30\_HUMAN sp|Q9UNH5|CC14A\_HUMAN sp|Q14511|CASL\_HUMAN sp|Q8NBI6|XXLT1\_HUMAN sp|Q9NT99|LRC4B\_HUMAN tr|H7C5A5|H7C5A5\_HUMAN sp|Q8IYR0|CF165\_HUMAN sp|Q86VY4|TSYL5\_HUMAN tr|H7C2S7|H7C2S7\_HUMAN tr|B7ZL65|B7ZL65\_HUMAN sp|O95208|EPN2\_HUMAN tr|I3L2B2|I3L2B2\_HUMAN tr|H0YA37|H0YA37\_HUMAN sp|Q13148|TADBP\_HUMAN sp|P51689|ARSD\_HUMAN tr|H0Y3R8|H0Y3R8\_HUMAN sp|Q92837|FRAT1\_HUMAN sp|Q5TAX3|TUT4\_HUMAN tr|C9IY40|C9IY40\_HUMAN tr|I3L207|I3L207\_HUMAN sp|Q5T653|RM02\_HUMAN tr|H0Y952|H0Y952\_HUMAN sp|Q8NGN3|O10G4\_HUMAN sp|Q5JUX0|SPIN3\_HUMAN tr|H0Y3P5|H0Y3P5\_HUMAN sp|Q5QP82|DCA10\_HUMAN sp|Q8TC05|MDM1\_HUMAN sp|Q9NYB5|SO1C1\_HUMAN tr|H0YFS3|H0YFS3\_HUMAN sp|Q8WWF3|SSMM1\_HUMAN sp|Q8NEA6|GLIS3\_HUMAN sp|Q9BUB5|MKNK1\_HUMAN tr|H0Y9Y0|H0Y9Y0\_HUMAN sp|Q9UKA1|FBXL5\_HUMAN sp|Q5T6F0|DCA12\_HUMAN tr|A0A087WTJ8|A0A087WTJ8\_HUMAN tr|X6R344|X6R344\_HUMAN tr|F5H7Q8|F5H7Q8\_HUMAN sp|P28324|ELK4\_HUMAN sp|Q9NR63|CP26B\_HUMAN tr|H0YJA8|H0YJA8\_HUMAN tr|A0A087WZG4|A0A087WZG4\_HUMAN tr|E7EPF1|E7EPF1\_HUMAN tr|B4DZ31|B4DZ31\_HUMAN tr|A0A087WYI6|A0A087WYI6\_HUMAN tr|K7EP39|K7EP39\_HUMAN tr|A0A087WUB0|A0A087WUB0\_HUMAN tr|F8VS07|F8VS07\_HUMAN sp|Q86W56|PARG\_HUMAN tr|F6UV76|F6UV76\_HUMAN tr|F6XM74|F6XM74\_HUMAN sp|Q5R3F8|PPR29\_HUMAN sp|Q53GS7|GLE1\_HUMAN tr|V9GYF5|V9GYF5\_HUMAN sp|P50542|PEX5\_HUMAN tr|B4E0T2|B4E0T2\_HUMAN tr|G3V3G9|G3V3G9\_HUMAN tr|U3KQN9|U3KQN9\_HUMAN tr|H3BQV3|H3BQV3\_HUMAN tr|H3BUN2|H3BUN2\_HUMAN sp|Q8N9M1|CS047\_HUMAN tr|F8VXY1|F8VXY1\_HUMAN tr|M0QYM7|M0QYM7\_HUMAN sp|A6NL88|SHSA7\_HUMAN sp|Q8IWZ6|BBS7\_HUMAN sp|O75486|SUPT3\_HUMAN tr|H0YLF2|H0YLF2\_HUMAN tr|E7ETU9|E7ETU9\_HUMAN tr|Q8WU40|Q8WU40\_HUMAN tr|E5RGZ2|E5RGZ2\_HUMAN sp|P57059|SIK1\_HUMAN tr|Q580Q6|Q580Q6\_HUMAN sp|Q5SGD2|PPM1L\_HUMAN sp|Q8ND56|LS14A\_HUMAN sp|P08571|CD14\_HUMAN sp|Q8NGG2|OR5T2\_HUMAN tr|C9J1I1|C9J1I1\_HUMAN tr|C9JGN2|C9JGN2\_HUMAN sp|Q9UN74|PCDA4\_HUMAN tr|C9JZV5|C9JZV5\_HUMAN sp|O15457|MSH4\_HUMAN sp|P35626|ARBK2\_HUMAN tr|B5ME97|B5ME97\_HUMAN sp|P80108|PHLD\_HUMAN sp|Q96EZ8|MCRS1\_HUMAN sp|O43529|CHSTA\_HUMAN tr|A0A087WV31|A0A087WV31\_HUMAN sp|Q13615|MTMR3\_HUMAN tr|H7C4T4|H7C4T4\_HUMAN sp|Q96NZ8|WFKN1\_HUMAN sp|P28068|DMB\_HUMAN sp|Q8NI35|INADL\_HUMAN sp|P39060|COIA1\_HUMAN tr|B7ZKQ9|B7ZKQ9\_HUMAN sp|Q96M02|CJ090\_HUMAN tr|Q5T025|Q5T025\_HUMAN tr|F5H4X0|F5H4X0\_HUMAN sp|Q68E01|INT3\_HUMAN sp|Q8WV15|T255B\_HUMAN tr|I3L3R3|I3L3R3\_HUMAN tr|E7ETV6|E7ETV6\_HUMAN sp|Q8NEK8|FA46D\_HUMAN tr|H7BZ29|H7BZ29\_HUMAN sp|Q96K78|GP128\_HUMAN sp|Q76MJ5|ERN2\_HUMAN tr|E7ETG2|E7ETG2\_HUMAN sp|P31947|1433S\_HUMAN sp|Q96G04|FA86A\_HUMAN sp|Q8WYQ9|ZCH14\_HUMAN tr|H3BS18|H3BS18\_HUMAN tr|D6RGZ6|D6RGZ6\_HUMAN sp|Q8IXH8|CAD26\_HUMAN tr|G3V4L8|G3V4L8\_HUMAN sp|Q6ZRT6|PR23B\_HUMAN A2I7N1 tr|C9J0G0|C9J0G0\_HUMAN sp|Q99424|ACOX2\_HUMAN sp|P58004|SESN2\_HUMAN tr|J3KN23|J3KN23\_HUMAN sp|Q9HDC5|JPH1\_HUMAN sp|Q9H9S5|FKRP\_HUMAN tr|E7ERV5|E7ERV5\_HUMAN tr|C9IYB7|C9IYB7\_HUMAN sp|O14498|ISLR\_HUMAN sp|P26885|FKBP2\_HUMAN sp|Q8N461|FXL16\_HUMAN sp|Q9H3H5|GPT\_HUMAN sp|Q96IZ5|RBM41\_HUMAN tr|H0Y470|H0Y470\_HUMAN tr|G5E9W5|G5E9W5\_HUMAN tr|E5RIA2|E5RIA2\_HUMAN tr|F5H4Z2|F5H4Z2\_HUMAN tr|E9PKY7|E9PKY7\_HUMAN tr|E9PRK8|E9PRK8\_HUMAN tr|F8WB65|F8WB65\_HUMAN tr|C9JZI2|C9JZI2\_HUMAN tr|C9JFT1|C9JFT1\_HUMAN sp|Q5GH77|XKR3\_HUMAN tr|M0R1W3|M0R1W3\_HUMAN sp|Q9NPG1|FZD3\_HUMAN sp|Q8IV38|ANKY2\_HUMAN sp|Q8NHC5|O14AG\_HUMAN sp|Q9H910|HN1L\_HUMAN sp|O94973|AP2A2\_HUMAN sp|Q7Z6J4|FGD2\_HUMAN tr|B5MCE1|B5MCE1\_HUMAN sp|Q9UPU9|SMAG1\_HUMAN sp|P16519|NEC2\_HUMAN sp|A6NMK8|F196B\_HUMAN tr|F5H008|F5H008\_HUMAN sp|Q9H267|VP33B\_HUMAN sp|O75509|TNR21\_HUMAN sp|Q9BZJ6|GPR63\_HUMAN sp|A6NCV1|O6C74\_HUMAN sp|Q8WY54|PPM1E\_HUMAN tr|H3BQ95|H3BQ95\_HUMAN tr|F5GYK2|F5GYK2\_HUMAN tr|A0A087WY01|A0A087WY01\_HUMAN sp|P29323|EPHB2\_HUMAN tr|B8ZZ31|B8ZZ31\_HUMAN tr|G3V5P6|G3V5P6\_HUMAN tr|J3QTH6|J3QTH6\_HUMAN sp|P52209|6PGD\_HUMAN tr|B8ZZC7|B8ZZC7\_HUMAN tr|B1AKC9|B1AKC9\_HUMAN sp|D6RCP7|U17LJ\_HUMAN sp|C9JVI0|U17LB\_HUMAN sp|D6R9N7|U17LI\_HUMAN sp|P48065|S6A12\_HUMAN sp|Q17RH7|TPRXL\_HUMAN sp|Q5T0N1|TTC18\_HUMAN sp|Q6ZRF8|RN207\_HUMAN tr|E9PIF2|E9PIF2\_HUMAN sp|Q13206|DDX10\_HUMAN sp|Q5VTB9|RN220\_HUMAN sp|Q14956|GPNMB\_HUMAN sp|Q7LGC8|CHST3\_HUMAN sp|P08582|TRFM\_HUMAN sp|Q53FD0|ZC21C\_HUMAN tr|M0R264|M0R264\_HUMAN sp|Q08043|ACTN3\_HUMAN sp|O94886|CSCL1\_HUMAN sp|Q9H161|ALX4\_HUMAN tr|C9J3M8|C9J3M8\_HUMAN sp|O95235|KI20A\_HUMAN sp|Q6ZVT6|CC067\_HUMAN tr|M0QZD4|M0QZD4\_HUMAN tr|H0YDU8|H0YDU8\_HUMAN sp|P53041|PPP5\_HUMAN sp|Q569K6|CC157\_HUMAN tr|Q59GD8|Q59GD8\_HUMAN sp|Q15256|PTPRR\_HUMAN tr|H7C0X4|H7C0X4\_HUMAN tr|C9J7S5|C9J7S5\_HUMAN sp|Q92685|ALG3\_HUMAN tr|A0A075B785|A0A075B785\_HUMAN sp|Q9P260|K1468\_HUMAN sp|Q6ZW61|BBS12\_HUMAN tr|A0A087X1S2|A0A087X1S2\_HUMAN sp|Q96RQ9|OXLA\_HUMAN tr|Q9HB44|Q9HB44\_HUMAN tr|B5MDG6|B5MDG6\_HUMAN sp|Q8IUW3|SPA2L\_HUMAN sp|Q8IXT1|DDIAS\_HUMAN sp|O95747|OXSR1\_HUMAN sp|Q9Y345|SC6A5\_HUMAN tr|C9JIG9|C9JIG9\_HUMAN sp|Q8NHS3|MFSD8\_HUMAN tr|E7ERQ4|E7ERQ4\_HUMAN tr|M0R1Z4|M0R1Z4\_HUMAN sp|Q9UGT4|SUSD2\_HUMAN sp|O95622|ADCY5\_HUMAN sp|Q8N5T2|TBC19\_HUMAN sp|Q7L311|ARMX2\_HUMAN sp|P49761|CLK3\_HUMAN tr|J3KQ26|J3KQ26\_HUMAN sp|Q8TE58|ATS15\_HUMAN sp|Q6XR72|ZNT10\_HUMAN sp|Q86SE5|RALYL\_HUMAN tr|C6GKH1|C6GKH1\_HUMAN sp|A6NHX0|GATL2\_HUMAN sp|Q16555|DPYL2\_HUMAN tr|G3V4S9|G3V4S9\_HUMAN tr|G3V5H8|G3V5H8\_HUMAN tr|G3V5S0|G3V5S0\_HUMAN tr|G3V5L7|G3V5L7\_HUMAN tr|G3V392|G3V392\_HUMAN tr|E0YMJ8|E0YMJ8\_HUMAN tr|A0A087WZC2|A0A087WZC2\_HUMAN tr|G3V5G0|G3V5G0\_HUMAN tr|G3V2A6|G3V2A6\_HUMAN tr|G3V2I9|G3V2I9\_HUMAN tr|X6R8I9|X6R8I9\_HUMAN tr|H0Y6L9|H0Y6L9\_HUMAN sp|Q5XKR4|OTP\_HUMAN sp|Q8N0X7|SPG20\_HUMAN sp|Q8N0V3|RBFA\_HUMAN sp|Q96NM4|TOX2\_HUMAN sp|P01009|A1AT\_HUMAN sp|Q8N0Z9|VSI10\_HUMAN sp|P10515|ODP2\_HUMAN tr|H0Y8C9|H0Y8C9\_HUMAN sp|Q9P2K3|RCOR3\_HUMAN sp|P58170|OR1D5\_HUMAN sp|Q5JYT7|K1755\_HUMAN sp|Q9BYT3|STK33\_HUMAN tr|F6TH74|F6TH74\_HUMAN tr|B4DDH2|B4DDH2\_HUMAN sp|Q5TDP6|LGSN\_HUMAN sp|P51797|CLCN6\_HUMAN sp|Q8TF44|C2C4C\_HUMAN sp|Q9UF33|EPHA6\_HUMAN tr|C9J7L4|C9J7L4\_HUMAN tr|B4DXG3|B4DXG3\_HUMAN sp|O60383|GDF9\_HUMAN sp|Q6IQ20|NAPEP\_HUMAN sp|P21266|GSTM3\_HUMAN sp|Q9UEW3|MARCO\_HUMAN sp|Q8N4U5|T11L2\_HUMAN sp|Q13443|ADAM9\_HUMAN tr|F8WC54|F8WC54\_HUMAN tr|A0AVL1|A0AVL1\_HUMAN tr|K7EME4|K7EME4\_HUMAN sp|Q8WY98|TM234\_HUMAN sp|Q99624|S38A3\_HUMAN sp|Q8IWG1|WDR63\_HUMAN tr|H7C3N3|H7C3N3\_HUMAN sp|Q96N23|CL055\_HUMAN tr|J3KRJ0|J3KRJ0\_HUMAN tr|J3KNY7|J3KNY7\_HUMAN sp|Q9NXS3|KLH28\_HUMAN sp|B3EWF7|EP2A2\_HUMAN sp|Q9UBQ6|EXTL2\_HUMAN tr|H3BMW8|H3BMW8\_HUMAN sp|Q96EE4|CC126\_HUMAN tr|H0YLY0|H0YLY0\_HUMAN tr|J3QQM3|J3QQM3\_HUMAN sp|O76027|ANXA9\_HUMAN tr|F5H4J1|F5H4J1\_HUMAN tr|Q5JUV4|Q5JUV4\_HUMAN sp|O60658|PDE8A\_HUMAN tr|E9PKB0|E9PKB0\_HUMAN sp|Q9UKD2|MRT4\_HUMAN tr|Q6N017|Q6N017\_HUMAN tr|A6NHG8|A6NHG8\_HUMAN sp|Q13114|TRAF3\_HUMAN tr|I3L0J9|I3L0J9\_HUMAN Q2KJC7 tr|H0YLK9|H0YLK9\_HUMAN tr|J3KQ32|J3KQ32\_HUMAN tr|C9IY94|C9IY94\_HUMAN tr|C9J2Q4|C9J2Q4\_HUMAN tr|A6NHN7|A6NHN7\_HUMAN tr|F8W6M1|F8W6M1\_HUMAN sp|Q99500|S1PR3\_HUMAN sp|Q92759|TF2H4\_HUMAN Q7RTT2 sp|Q8N1N4|K2C78\_HUMAN Q8N1N4-2 sp|Q4G112|HSF5\_HUMAN tr|A0A075B6P9|A0A075B6P9\_HUMAN tr|H0YED1|H0YED1\_HUMAN sp|Q9UBY0|SL9A2\_HUMAN tr|F8VS03|F8VS03\_HUMAN tr|H7BXJ2|H7BXJ2\_HUMAN tr|C9J1I0|C9J1I0\_HUMAN tr|C9J2B6|C9J2B6\_HUMAN sp|Q9P244|LRFN1\_HUMAN sp|Q6UXZ4|UNC5D\_HUMAN sp|P06748|NPM\_HUMAN tr|E9PDS8|E9PDS8\_HUMAN sp|P36956|SRBP1\_HUMAN sp|Q99467|CD180\_HUMAN tr|A0A096LP03|A0A096LP03\_HUMAN tr|K7EK08|K7EK08\_HUMAN tr|E9PQ01|E9PQ01\_HUMAN tr|A0A087WXD7|A0A087WXD7\_HUMAN tr|E9PKN0|E9PKN0\_HUMAN tr|Q5HYY5|Q5HYY5\_HUMAN tr|H0YI72|H0YI72\_HUMAN sp|Q9UM63|PLAL1\_HUMAN tr|R4GN16|R4GN16\_HUMAN sp|O60603|TLR2\_HUMAN sp|Q96PB7|NOE3\_HUMAN sp|P78347|GTF2I\_HUMAN tr|E9PQ57|E9PQ57\_HUMAN sp|P30512|1A29\_HUMAN tr|H7C5K7|H7C5K7\_HUMAN sp|Q9NRU3|CNNM1\_HUMAN sp|Q9NXF8|ZDHC7\_HUMAN sp|Q9Y278|HS3S2\_HUMAN tr|B1AUU8|B1AUU8\_HUMAN tr|E9PIE4|E9PIE4\_HUMAN tr|H0Y7U8|H0Y7U8\_HUMAN sp|Q8IX15|HOMEZ\_HUMAN sp|Q13474|DRP2\_HUMAN tr|Q15635|Q15635\_HUMAN tr|Q86W68|Q86W68\_HUMAN tr|E7EPF2|E7EPF2\_HUMAN sp|Q9UP79|ATS8\_HUMAN sp|Q9NUM4|T106B\_HUMAN sp|Q86UX2|ITIH5\_HUMAN tr|C9J2H1|C9J2H1\_HUMAN tr|E9PKW1|E9PKW1\_HUMAN sp|Q9NUB1|ACS2L\_HUMAN sp|Q8N568|DCLK2\_HUMAN sp|O75152|ZC11A\_HUMAN tr|M0QXD5|M0QXD5\_HUMAN tr|M0R1K2|M0R1K2\_HUMAN tr|H3BLU1|H3BLU1\_HUMAN sp|Q9H257|CARD9\_HUMAN sp|Q14957|NMDE3\_HUMAN tr|H0Y2V8|H0Y2V8\_HUMAN tr|H0YJD4|H0YJD4\_HUMAN tr|H7C517|H7C517\_HUMAN tr|F6SS63|F6SS63\_HUMAN tr|H0Y6M8|H0Y6M8\_HUMAN sp|Q9HCS4|TF7L1\_HUMAN tr|H0YMD3|H0YMD3\_HUMAN sp|O43281|EFS\_HUMAN sp|Q6ZMH5|S39A5\_HUMAN tr|H0YND9|H0YND9\_HUMAN tr|H0Y9U7|H0Y9U7\_HUMAN tr|E9PFD9|E9PFD9\_HUMAN sp|P26367|PAX6\_HUMAN sp|A0AVF1|TTC26\_HUMAN tr|E9PPR7|E9PPR7\_HUMAN tr|C9JU19|C9JU19\_HUMAN tr|M0R0U3|M0R0U3\_HUMAN tr|E7ERK8|E7ERK8\_HUMAN tr|E9PK14|E9PK14\_HUMAN tr|A0A087WW82|A0A087WW82\_HUMAN tr|A0A087WW78|A0A087WW78\_HUMAN tr|E7EQE7|E7EQE7\_HUMAN tr|H0Y5J1|H0Y5J1\_HUMAN sp|Q8IU85|KCC1D\_HUMAN sp|Q86VL8|S47A2\_HUMAN tr|A0A087WUR6|A0A087WUR6\_HUMAN tr|H3BV69|H3BV69\_HUMAN tr|A0A087X092|A0A087X092\_HUMAN tr|E7EW60|E7EW60\_HUMAN tr|C9JMM4|C9JMM4\_HUMAN tr|B8ZZF3|B8ZZF3\_HUMAN tr|I3L4D8|I3L4D8\_HUMAN tr|C9JTP4|C9JTP4\_HUMAN sp|O95402|MED26\_HUMAN tr|F6VX93|F6VX93\_HUMAN tr|F8W1J9|F8W1J9\_HUMAN sp|Q6NSW7|NANP8\_HUMAN sp|Q9H9S0|NANOG\_HUMAN tr|F8VYL4|F8VYL4\_HUMAN tr|F8VZH2|F8VZH2\_HUMAN tr|J3KS32|J3KS32\_HUMAN tr|F8VW34|F8VW34\_HUMAN tr|F8VXG3|F8VXG3\_HUMAN tr|F8VX54|F8VX54\_HUMAN sp|P54136|SYRC\_HUMAN sp|A6NKD2|TSPY2\_HUMAN tr|H7C296|H7C296\_HUMAN sp|P0CV98|TSPY3\_HUMAN tr|R4GMP3|R4GMP3\_HUMAN tr|A6NGT6|A6NGT6\_HUMAN tr|R4GNE9|R4GNE9\_HUMAN sp|Q01534|TSPY1\_HUMAN tr|F2Z2I4|F2Z2I4\_HUMAN tr|Q8N433|Q8N433\_HUMAN sp|P0CV99|TSPY4\_HUMAN sp|P52701|MSH6\_HUMAN sp|P0CW01|TSPYA\_HUMAN tr|A6NEC3|A6NEC3\_HUMAN sp|Q96NB2|SFXN2\_HUMAN tr|K7EJ41|K7EJ41\_HUMAN sp|Q5BKT4|AG10A\_HUMAN sp|Q9Y2A4|ZN443\_HUMAN tr|F6TF88|F6TF88\_HUMAN sp|P08133|ANXA6\_HUMAN sp|Q86UW9|DTX2\_HUMAN tr|H3BNC6|H3BNC6\_HUMAN tr|K7EQY9|K7EQY9\_HUMAN tr|Q5T0V0|Q5T0V0\_HUMAN sp|A8MPX8|PP2D1\_HUMAN sp|O75093|SLIT1\_HUMAN tr|S4R3W2|S4R3W2\_HUMAN sp|Q6ZRQ5|MMS22\_HUMAN tr|S4R3Q9|S4R3Q9\_HUMAN tr|H0YCG5|H0YCG5\_HUMAN sp|Q15070|OXA1L\_HUMAN sp|Q9Y6A1|POMT1\_HUMAN tr|H0YEL2|H0YEL2\_HUMAN tr|H3BUJ7|H3BUJ7\_HUMAN tr|A0A087X0L5|A0A087X0L5\_HUMAN sp|P58304|VSX2\_HUMAN sp|P14210|HGF\_HUMAN sp|P15407|FOSL1\_HUMAN tr|K7EQZ1|K7EQZ1\_HUMAN tr|E9PPX2|E9PPX2\_HUMAN tr|K7EJR0|K7EJR0\_HUMAN sp|P07099|HYEP\_HUMAN sp|Q9BSA9|TM175\_HUMAN tr|B1AHF3|B1AHF3\_HUMAN sp|Q8IZ96|CKLF1\_HUMAN sp|P62508|ERR3\_HUMAN tr|H0YE48|H0YE48\_HUMAN tr|H0YDG1|H0YDG1\_HUMAN tr|B3KP41|B3KP41\_HUMAN tr|D6R9X2|D6R9X2\_HUMAN sp|Q96F46|I17RA\_HUMAN sp|P14678|RSMB\_HUMAN tr|A8MT02|A8MT02\_HUMAN sp|P46777|RL5\_HUMAN tr|H0YDN0|H0YDN0\_HUMAN sp|Q86X59|CQ082\_HUMAN sp|Q6GMV2|SMYD5\_HUMAN tr|E9PCC8|E9PCC8\_HUMAN tr|Q5JRH0|Q5JRH0\_HUMAN tr|G5E9U2|G5E9U2\_HUMAN tr|A0A087X070|A0A087X070\_HUMAN sp|A2A2Y4|FRMD3\_HUMAN sp|O00341|EAA5\_HUMAN tr|H7C0L5|H7C0L5\_HUMAN tr|A0A087WUF9|A0A087WUF9\_HUMAN sp|Q9HD33|RM47\_HUMAN tr|A0A087X1U4|A0A087X1U4\_HUMAN sp|P30740|ILEU\_HUMAN sp|Q96D09|GASP2\_HUMAN sp|Q8TED4|SPX2\_HUMAN tr|A0A087WWP5|A0A087WWP5\_HUMAN sp|Q9Y575|ASB3\_HUMAN tr|D6RIH8|D6RIH8\_HUMAN tr|H7C2Q6|H7C2Q6\_HUMAN tr|F6R0L0|F6R0L0\_HUMAN tr|H0Y760|H0Y760\_HUMAN sp|P58335|ANTR2\_HUMAN tr|K7EQY5|K7EQY5\_HUMAN sp|P42679|MATK\_HUMAN sp|Q86VN1|VPS36\_HUMAN sp|P56696|KCNQ4\_HUMAN sp|Q6ZU80|CE128\_HUMAN sp|Q6NXT4|ZNT6\_HUMAN sp|Q9UBI4|STML1\_HUMAN tr|B5MCR8|B5MCR8\_HUMAN sp|Q8N2H9|PELI3\_HUMAN sp|P62879|GBB2\_HUMAN tr|C9JXA5|C9JXA5\_HUMAN tr|E9PI91|E9PI91\_HUMAN tr|C9JIS1|C9JIS1\_HUMAN sp|Q8NCS7|CTL5\_HUMAN tr|F8W9U0|F8W9U0\_HUMAN tr|C9JW43|C9JW43\_HUMAN tr|K7EMW4|K7EMW4\_HUMAN sp|Q5SRN2|CF010\_HUMAN sp|Q969V3|NCLN\_HUMAN tr|H0YJR5|H0YJR5\_HUMAN tr|B1AKJ5|B1AKJ5\_HUMAN sp|Q9NZM1|MYOF\_HUMAN tr|F8VPL3|F8VPL3\_HUMAN sp|P41181|AQP2\_HUMAN tr|F8W0S2|F8W0S2\_HUMAN sp|Q01415|GALK2\_HUMAN tr|D6RAY6|D6RAY6\_HUMAN sp|Q8IWA6|CCD60\_HUMAN sp|Q14390|GGTL2\_HUMAN sp|Q86WA8|LONP2\_HUMAN sp|P0C866|F91A2\_HUMAN sp|P28335|5HT2C\_HUMAN tr|E9PFT2|E9PFT2\_HUMAN sp|B1ANS9|WDR64\_HUMAN tr|H7C319|H7C319\_HUMAN tr|R4GN27|R4GN27\_HUMAN tr|M0R1L7|M0R1L7\_HUMAN tr|B1ALC3|B1ALC3\_HUMAN tr|H0YG32|H0YG32\_HUMAN tr|H0YGM1|H0YGM1\_HUMAN tr|C9J470|C9J470\_HUMAN tr|H3BV68|H3BV68\_HUMAN tr|C9J7I2|C9J7I2\_HUMAN sp|Q9UH90|FBX40\_HUMAN sp|P47884|OR1D4\_HUMAN sp|P11801|KPSH1\_HUMAN sp|Q6ZN28|MACC1\_HUMAN tr|J3KRG6|J3KRG6\_HUMAN tr|F5H2Z8|F5H2Z8\_HUMAN sp|Q6XPS3|TPTE2\_HUMAN tr|A0A087WYY6|A0A087WYY6\_HUMAN tr|E9PPB3|E9PPB3\_HUMAN tr|F8W7A7|F8W7A7\_HUMAN tr|E9PS32|E9PS32\_HUMAN tr|E9PP43|E9PP43\_HUMAN sp|Q5JUK2|SOLH1\_HUMAN sp|Q5BJE1|CC178\_HUMAN sp|Q13835|PKP1\_HUMAN tr|M0R1G9|M0R1G9\_HUMAN tr|A0A087WW66|A0A087WW66\_HUMAN sp|Q99460|PSMD1\_HUMAN sp|Q8N697|S15A4\_HUMAN tr|V9GYN8|V9GYN8\_HUMAN sp|Q8N3Z6|ZCHC7\_HUMAN tr|D6RAA0|D6RAA0\_HUMAN sp|Q5VX71|SUSD4\_HUMAN sp|Q86TX2|ACOT1\_HUMAN sp|Q8TF40|FNIP1\_HUMAN tr|J3KNG8|J3KNG8\_HUMAN tr|A0A087WTG3|A0A087WTG3\_HUMAN tr|Q5VVQ1|Q5VVQ1\_HUMAN tr|C4P0D8|C4P0D8\_HUMAN tr|J3KNV5|J3KNV5\_HUMAN sp|Q9Y6W3|CAN7\_HUMAN tr|C4P0D4|C4P0D4\_HUMAN tr|C4P0D6|C4P0D6\_HUMAN sp|Q9H211|CDT1\_HUMAN sp|Q86UF1|TSN33\_HUMAN tr|E9PQM8|E9PQM8\_HUMAN tr|F8W038|F8W038\_HUMAN sp|Q7Z4L5|TT21B\_HUMAN tr|B1ALP6|B1ALP6\_HUMAN sp|Q16706|MA2A1\_HUMAN tr|D6RA50|D6RA50\_HUMAN sp|P48775|T23O\_HUMAN tr|D6RB68|D6RB68\_HUMAN sp|Q13938|CAYP1\_HUMAN tr|I3L2R2|I3L2R2\_HUMAN sp|P19113|DCHS\_HUMAN tr|S4R3Y2|S4R3Y2\_HUMAN sp|Q9Y4X0|AMMR1\_HUMAN tr|E9PIF4|E9PIF4\_HUMAN tr|H0Y735|H0Y735\_HUMAN sp|O43423|AN32C\_HUMAN tr|V9GYN0|V9GYN0\_HUMAN tr|E7ENM8|E7ENM8\_HUMAN sp|Q9H0V1|TM168\_HUMAN tr|Q32MM7|Q32MM7\_HUMAN sp|Q8IUC8|GLT13\_HUMAN tr|B1AK20|B1AK20\_HUMAN tr|H0YJT2|H0YJT2\_HUMAN sp|Q8N148|OR6V1\_HUMAN tr|U3KQL4|U3KQL4\_HUMAN sp|Q96C12|ARMC5\_HUMAN sp|A6NHN0|OTOL1\_HUMAN tr|A0A087WZI2|A0A087WZI2\_HUMAN tr|D6RFN0|D6RFN0\_HUMAN sp|P52741|ZN134\_HUMAN sp|Q96EK7|F120B\_HUMAN sp|Q9BXC0|HCAR1\_HUMAN tr|A0A087WSW2|A0A087WSW2\_HUMAN sp|P17038|ZNF43\_HUMAN tr|E9PN83|E9PN83\_HUMAN sp|Q8TC21|ZN596\_HUMAN tr|E5RHT8|E5RHT8\_HUMAN sp|Q9UH99|SUN2\_HUMAN tr|B4DKH3|B4DKH3\_HUMAN sp|Q8TB22|SPT20\_HUMAN sp|O43314|VIP2\_HUMAN sp|Q8TAG6|CH046\_HUMAN tr|E5RH60|E5RH60\_HUMAN sp|Q9UKF7|PITC1\_HUMAN sp|Q8IX94|CTGE4\_HUMAN sp|P0CG41|CTGE8\_HUMAN tr|K9N0C7|K9N0C7\_HUMAN tr|K9N2Q6|K9N2Q6\_HUMAN tr|K9N163|K9N163\_HUMAN tr|F5GYY5|F5GYY5\_HUMAN sp|P51690|ARSE\_HUMAN sp|Q96N76|HUTU\_HUMAN sp|Q96J87|CELF6\_HUMAN sp|Q96EY5|MB12A\_HUMAN tr|E9PQA6|E9PQA6\_HUMAN sp|Q05215|EGR4\_HUMAN sp|P26440|IVD\_HUMAN sp|Q8N5J2|FA63A\_HUMAN tr|A0A087WU72|A0A087WU72\_HUMAN tr|H7C3S5|H7C3S5\_HUMAN sp|Q15782|CH3L2\_HUMAN tr|Q5T1B5|Q5T1B5\_HUMAN tr|B4DFI2|B4DFI2\_HUMAN sp|Q14642|I5P1\_HUMAN tr|M0R0A5|M0R0A5\_HUMAN sp|Q13507|TRPC3\_HUMAN Q28085 sp|Q8N1W1|ARG28\_HUMAN tr|F5H515|F5H515\_HUMAN sp|Q53GG5|PDLI3\_HUMAN sp|Q9BQG1|SYT3\_HUMAN tr|C9JD05|C9JD05\_HUMAN tr|D6RG09|D6RG09\_HUMAN sp|Q9BXM9|FSD1L\_HUMAN tr|F8W946|F8W946\_HUMAN sp|Q9BY64|UDB28\_HUMAN tr|H0YE38|H0YE38\_HUMAN tr|A0A087WYG9|A0A087WYG9\_HUMAN tr|F5H386|F5H386\_HUMAN tr|H9KV87|H9KV87\_HUMAN tr|H0Y8D6|H0Y8D6\_HUMAN tr|E9PIR7|E9PIR7\_HUMAN sp|P26436|ASPX\_HUMAN tr|F8W809|F8W809\_HUMAN tr|A0A087WSY9|A0A087WSY9\_HUMAN tr|E9PNQ6|E9PNQ6\_HUMAN tr|A0A087WSW9|A0A087WSW9\_HUMAN sp|Q9H1K4|GHC2\_HUMAN sp|Q16881|TRXR1\_HUMAN tr|E2QRB9|E2QRB9\_HUMAN sp|Q96PV7|F193B\_HUMAN sp|Q8N9I9|DTX3\_HUMAN tr|H0YHF9|H0YHF9\_HUMAN sp|Q9H3M9|ATX3L\_HUMAN sp|P10276|RARA\_HUMAN tr|H3BPZ0|H3BPZ0\_HUMAN tr|B0QZ55|B0QZ55\_HUMAN tr|C9JSC3|C9JSC3\_HUMAN sp|Q9NUK0|MBNL3\_HUMAN tr|F5H6L7|F5H6L7\_HUMAN tr|B1AKI4|B1AKI4\_HUMAN sp|P18510|IL1RA\_HUMAN tr|A0A087WW88|A0A087WW88\_HUMAN tr|B3KWG5|B3KWG5\_HUMAN sp|Q9H3P7|GCP60\_HUMAN tr|Q5T3P9|Q5T3P9\_HUMAN sp|Q8NAA4|A16L2\_HUMAN tr|H0YG67|H0YG67\_HUMAN tr|H0YF72|H0YF72\_HUMAN sp|P25106|ACKR3\_HUMAN tr|H0YG45|H0YG45\_HUMAN tr|H9KVD6|H9KVD6\_HUMAN tr|H3BPQ9|H3BPQ9\_HUMAN sp|Q16610|ECM1\_HUMAN tr|Q9NW36|Q9NW36\_HUMAN tr|E7ESJ2|E7ESJ2\_HUMAN Q61782 sp|Q14624|ITIH4\_HUMAN sp|P28799|GRN\_HUMAN tr|Q0PRL4|Q0PRL4\_HUMAN sp|Q6ECI4|ZN470\_HUMAN tr|K7EKL3|K7EKL3\_HUMAN sp|O75460|ERN1\_HUMAN sp|Q96BR9|ZBT8A\_HUMAN sp|Q99733|NP1L4\_HUMAN tr|H7BZZ3|H7BZZ3\_HUMAN tr|E9PLJ2|E9PLJ2\_HUMAN sp|Q9Y2S2|CRYL1\_HUMAN sp|Q96MZ0|GD1L1\_HUMAN tr|E9PS74|E9PS74\_HUMAN tr|A0A087X057|A0A087X057\_HUMAN sp|O60259|KLK8\_HUMAN tr|G3V2W9|G3V2W9\_HUMAN sp|Q495T6|MMEL1\_HUMAN tr|U3KQV2|U3KQV2\_HUMAN tr|H0YBX9|H0YBX9\_HUMAN sp|Q14781|CBX2\_HUMAN sp|P07384|CAN1\_HUMAN tr|A0A087WW95|A0A087WW95\_HUMAN tr|A0A087X0B4|A0A087X0B4\_HUMAN sp|Q6ZW31|SYDE1\_HUMAN tr|H3BV80|H3BV80\_HUMAN tr|G3XAN5|G3XAN5\_HUMAN sp|O43586|PPIP1\_HUMAN tr|J3KPG6|J3KPG6\_HUMAN tr|H3BTT7|H3BTT7\_HUMAN tr|H3BP08|H3BP08\_HUMAN tr|A0A087WZQ5|A0A087WZQ5\_HUMAN tr|A0A087WYE1|A0A087WYE1\_HUMAN sp|O94851|MICA2\_HUMAN sp|Q6YHK3|CD109\_HUMAN sp|O76050|NEUL1\_HUMAN sp|Q9NV58|RN19A\_HUMAN sp|O95222|OR6A2\_HUMAN tr|H7C2S5|H7C2S5\_HUMAN tr|M0R1T5|M0R1T5\_HUMAN sp|P01597|KV105\_HUMAN tr|F8WA32|F8WA32\_HUMAN sp|Q05639|EF1A2\_HUMAN sp|Q9BZW7|TSG10\_HUMAN tr|B8ZZZ9|B8ZZZ9\_HUMAN tr|H3BLU3|H3BLU3\_HUMAN sp|Q96P65|QRFPR\_HUMAN tr|J3KNR3|J3KNR3\_HUMAN sp|Q5SW24|DACT2\_HUMAN tr|F2Z3L3|F2Z3L3\_HUMAN sp|Q5T440|CAF17\_HUMAN tr|B4DW73|B4DW73\_HUMAN tr|H0YML5|H0YML5\_HUMAN sp|Q16822|PCKGM\_HUMAN sp|Q8NH01|O2T11\_HUMAN tr|E7EP65|E7EP65\_HUMAN tr|H0YLW6|H0YLW6\_HUMAN tr|H0YLM1|H0YLM1\_HUMAN sp|Q494U1|PKHN1\_HUMAN sp|Q9NYB9|ABI2\_HUMAN tr|F8WAL6|F8WAL6\_HUMAN tr|H0YLD8|H0YLD8\_HUMAN sp|Q9BPX3|CND3\_HUMAN tr|K7ELX2|K7ELX2\_HUMAN sp|Q9Y2H6|FND3A\_HUMAN tr|G3V1X8|G3V1X8\_HUMAN tr|K7EQ19|K7EQ19\_HUMAN sp|Q9Y5B9|SP16H\_HUMAN tr|B8ZZY2|B8ZZY2\_HUMAN sp|P52594|AGFG1\_HUMAN tr|E9PFZ1|E9PFZ1\_HUMAN sp|Q5JPI3|CC038\_HUMAN tr|M0QYD1|M0QYD1\_HUMAN sp|Q86XN8|MEX3D\_HUMAN tr|M0R189|M0R189\_HUMAN sp|Q96A19|C102A\_HUMAN tr|S4R446|S4R446\_HUMAN sp|O76036|NCTR1\_HUMAN sp|P49768|PSN1\_HUMAN tr|E7ES96|E7ES96\_HUMAN sp|Q8N594|MPND\_HUMAN sp|O60264|SMCA5\_HUMAN sp|P50548|ERF\_HUMAN sp|Q96LD1|SGCZ\_HUMAN tr|I3L100|I3L100\_HUMAN tr|H3BMM9|H3BMM9\_HUMAN tr|H3BTC0|H3BTC0\_HUMAN sp|Q6ZVD7|STOX1\_HUMAN sp|P35557|HXK4\_HUMAN tr|B1AKT3|B1AKT3\_HUMAN sp|P14923|PLAK\_HUMAN tr|H0YLW9|H0YLW9\_HUMAN tr|E7ETN3|E7ETN3\_HUMAN sp|O15379|HDAC3\_HUMAN sp|Q86XK3|SFR1\_HUMAN tr|B1AR60|B1AR60\_HUMAN sp|Q8NGI0|O52N2\_HUMAN sp|Q14574|DSC3\_HUMAN tr|K7EM07|K7EM07\_HUMAN sp|Q96IK0|TM101\_HUMAN sp|Q15057|ACAP2\_HUMAN tr|A0A087X1H5|A0A087X1H5\_HUMAN tr|H0YGW3|H0YGW3\_HUMAN tr|E9PLY0|E9PLY0\_HUMAN sp|P40425|PBX2\_HUMAN sp|O75306|NDUS2\_HUMAN tr|F5GYX2|F5GYX2\_HUMAN sp|Q8IWB4|S31A7\_HUMAN sp|Q4VX67|S31A4\_HUMAN tr|H7C5K9|H7C5K9\_HUMAN tr|Q9BV00|Q9BV00\_HUMAN tr|A2AVK2|A2AVK2\_HUMAN sp|Q10713|MPPA\_HUMAN sp|Q9P0L1|ZKSC7\_HUMAN sp|Q7RTS9|DYM\_HUMAN sp|Q13033|STRN3\_HUMAN sp|O75840|KLF7\_HUMAN tr|C9JX20|C9JX20\_HUMAN tr|G3V318|G3V318\_HUMAN tr|H3BS99|H3BS99\_HUMAN sp|Q58G82|SY14L\_HUMAN sp|O60242|BAI3\_HUMAN sp|Q96M20|CNBD2\_HUMAN sp|O75787|RENR\_HUMAN tr|I3L3C6|I3L3C6\_HUMAN sp|Q04743|EMX2\_HUMAN tr|H0YLM8|H0YLM8\_HUMAN tr|H7C1L0|H7C1L0\_HUMAN tr|V9GYQ3|V9GYQ3\_HUMAN tr|H0YED4|H0YED4\_HUMAN tr|H0YM90|H0YM90\_HUMAN sp|Q16288|NTRK3\_HUMAN tr|A8MY87|A8MY87\_HUMAN sp|O15049|N4BP3\_HUMAN sp|Q02108|GCYA3\_HUMAN sp|A8MWA6|F90AM\_HUMAN sp|Q8N9H8|MUT7\_HUMAN sp|Q9BZV3|IMPG2\_HUMAN sp|O00151|PDLI1\_HUMAN sp|Q5JSP0|FGD3\_HUMAN sp|Q8WTU0|DDI1\_HUMAN sp|Q9H201|EPN3\_HUMAN sp|Q8NG97|OR2Z1\_HUMAN sp|Q9NQE7|TSSP\_HUMAN sp|Q5T4F7|SFRP5\_HUMAN sp|Q9H222|ABCG5\_HUMAN sp|Q08828|ADCY1\_HUMAN sp|P43631|KI2S2\_HUMAN sp|Q9ULE4|F184B\_HUMAN tr|J3KRF6|J3KRF6\_HUMAN sp|Q9UBZ4|APEX2\_HUMAN sp|Q8IZ41|RASEF\_HUMAN tr|H3BV15|H3BV15\_HUMAN sp|Q96R45|OR2A7\_HUMAN sp|O95047|OR2A4\_HUMAN sp|Q6B0I6|KDM4D\_HUMAN sp|Q96FE7|P3IP1\_HUMAN tr|C9JWC4|C9JWC4\_HUMAN tr|E7EUS2|E7EUS2\_HUMAN tr|J3KRD7|J3KRD7\_HUMAN tr|J3QQZ3|J3QQZ3\_HUMAN sp|Q13003|GRIK3\_HUMAN sp|Q8TBZ0|CC110\_HUMAN sp|O43934|MFS11\_HUMAN sp|Q96DB2|HDA11\_HUMAN tr|B5MCQ6|B5MCQ6\_HUMAN tr|E7ETT9|E7ETT9\_HUMAN tr|H0YJW4|H0YJW4\_HUMAN tr|C9J4A7|C9J4A7\_HUMAN tr|G3V4C4|G3V4C4\_HUMAN sp|O43426|SYNJ1\_HUMAN tr|J3KQV8|J3KQV8\_HUMAN sp|Q6MZP7|LIN54\_HUMAN sp|O43405|COCH\_HUMAN sp|Q92974|ARHG2\_HUMAN sp|A6NKC9|SH2D7\_HUMAN sp|P78536|ADA17\_HUMAN tr|J3KNS1|J3KNS1\_HUMAN tr|C9IZ03|C9IZ03\_HUMAN tr|C9J5S2|C9J5S2\_HUMAN sp|Q9HCI7|MSL2\_HUMAN sp|Q9UQ13|SHOC2\_HUMAN sp|Q96PP9|GBP4\_HUMAN sp|Q8TDG2|ACTT1\_HUMAN tr|C9IZL7|C9IZL7\_HUMAN tr|C9JYS8|C9JYS8\_HUMAN sp|Q15233|NONO\_HUMAN sp|Q2M329|CCD96\_HUMAN sp|Q96A23|CPNE4\_HUMAN tr|D6RAH0|D6RAH0\_HUMAN tr|G3V261|G3V261\_HUMAN tr|H3BRB0|H3BRB0\_HUMAN tr|Q5JUB8|Q5JUB8\_HUMAN tr|B7Z3Q7|B7Z3Q7\_HUMAN tr|H7C1Q6|H7C1Q6\_HUMAN sp|Q10586|DBP\_HUMAN tr|M0QXP1|M0QXP1\_HUMAN tr|F8WAI0|F8WAI0\_HUMAN tr|H0Y5P2|H0Y5P2\_HUMAN tr|H7C1X4|H7C1X4\_HUMAN tr|H7BZU1|H7BZU1\_HUMAN tr|H0Y7G7|H0Y7G7\_HUMAN sp|Q7Z429|LFG1\_HUMAN sp|Q8NHU6|TDRD7\_HUMAN sp|Q07666|KHDR1\_HUMAN sp|P41229|KDM5C\_HUMAN tr|R4GMZ0|R4GMZ0\_HUMAN sp|Q8NBT3|TM145\_HUMAN sp|Q9Y446|PKP3\_HUMAN tr|E7ERG8|E7ERG8\_HUMAN tr|A6NC58|A6NC58\_HUMAN sp|P22460|KCNA5\_HUMAN tr|F8WAS8|F8WAS8\_HUMAN tr|A0A087WYM3|A0A087WYM3\_HUMAN sp|O95171|SCEL\_HUMAN sp|P20849|CO9A1\_HUMAN sp|Q6UXG3|CLM9\_HUMAN sp|Q12864|CAD17\_HUMAN tr|J3KQU2|J3KQU2\_HUMAN sp|Q05C16|LRC63\_HUMAN tr|Q5T6N4|Q5T6N4\_HUMAN sp|O14639|ABLM1\_HUMAN sp|P49863|GRAK\_HUMAN tr|H7C3B0|H7C3B0\_HUMAN sp|Q9BXN1|ASPN\_HUMAN tr|H7C2T0|H7C2T0\_HUMAN tr|H7C2N4|H7C2N4\_HUMAN tr|H3BQ98|H3BQ98\_HUMAN tr|E9PS81|E9PS81\_HUMAN sp|Q86UN2|R4RL1\_HUMAN tr|Q5T6H7|Q5T6H7\_HUMAN sp|Q9UJ99|CAD22\_HUMAN tr|U3NG26|U3NG26\_HUMAN sp|Q06187|BTK\_HUMAN sp|P23141|EST1\_HUMAN sp|Q9UPY6|WASF3\_HUMAN sp|Q9ULC8|ZDHC8\_HUMAN tr|F8WCY4|F8WCY4\_HUMAN tr|H0Y936|H0Y936\_HUMAN tr|D6REB6|D6REB6\_HUMAN sp|O14770|MEIS2\_HUMAN Q0VBK2 sp|Q5T3F8|CSCL2\_HUMAN sp|Q9BRL6|SRSF8\_HUMAN sp|P68371|TBB4B\_HUMAN tr|E9PP16|E9PP16\_HUMAN tr|E9PIH6|E9PIH6\_HUMAN tr|H0Y4N1|H0Y4N1\_HUMAN sp|P51003|PAPOA\_HUMAN tr|G3XAH6|G3XAH6\_HUMAN tr|H0Y7H7|H0Y7H7\_HUMAN tr|H0Y4Y8|H0Y4Y8\_HUMAN tr|H0YDP6|H0YDP6\_HUMAN tr|A0A087WTU8|A0A087WTU8\_HUMAN sp|O95397|PKHA9\_HUMAN sp|P12532|KCRU\_HUMAN sp|P14136|GFAP\_HUMAN tr|K7EKH9|K7EKH9\_HUMAN tr|K7ELP4|K7ELP4\_HUMAN tr|B4DIR1|B4DIR1\_HUMAN tr|K7EJU1|K7EJU1\_HUMAN sp|A8MPY1|GBRR3\_HUMAN sp|A6NK44|GLOD5\_HUMAN sp|Q7Z434|MAVS\_HUMAN tr|H0YIW7|H0YIW7\_HUMAN sp|Q66K74|MAP1S\_HUMAN tr|K7EKF7|K7EKF7\_HUMAN Q32PI4 sp|Q6DD88|ATLA3\_HUMAN tr|F5H6I7|F5H6I7\_HUMAN sp|P0C854|CECR9\_HUMAN sp|Q96P70|IPO9\_HUMAN sp|Q9P2U8|VGLU2\_HUMAN tr|F8W079|F8W079\_HUMAN tr|Q6NSM0|Q6NSM0\_HUMAN sp|P0CF75|EBLN1\_HUMAN sp|P17017|ZNF14\_HUMAN sp|P48029|SC6A8\_HUMAN tr|A0A087WZM2|A0A087WZM2\_HUMAN tr|J3QQX0|J3QQX0\_HUMAN sp|P01778|HV317\_HUMAN sp|Q13106|ZN154\_HUMAN tr|H0Y7U4|H0Y7U4\_HUMAN sp|Q9Y6J8|STYL1\_HUMAN sp|Q99814|EPAS1\_HUMAN tr|C9JBE1|C9JBE1\_HUMAN sp|Q8IWW8|HOT\_HUMAN tr|B9ZVU2|B9ZVU2\_HUMAN tr|F8WAN0|F8WAN0\_HUMAN sp|P12107|COBA1\_HUMAN sp|P13688|CEAM1\_HUMAN tr|H0Y379|H0Y379\_HUMAN tr|G3V3A7|G3V3A7\_HUMAN sp|Q8NH85|OR5R1\_HUMAN sp|Q8NG68|TTL\_HUMAN tr|B7ZC39|B7ZC39\_HUMAN tr|B7ZC38|B7ZC38\_HUMAN tr|G5E9V4|G5E9V4\_HUMAN sp|Q9NR46|SHLB2\_HUMAN sp|P07204|TRBM\_HUMAN sp|P82251|BAT1\_HUMAN sp|Q6QNK2|GP133\_HUMAN sp|Q5RKV6|EXOS6\_HUMAN tr|K7ERH0|K7ERH0\_HUMAN tr|H0YAM7|H0YAM7\_HUMAN sp|P20333|TNR1B\_HUMAN sp|P16118|F261\_HUMAN sp|Q5JTC6|AMER1\_HUMAN tr|Q5SWX3|Q5SWX3\_HUMAN sp|Q99685|MGLL\_HUMAN tr|H0YNG5|H0YNG5\_HUMAN sp|Q6UW60|PCSK4\_HUMAN sp|Q9BV10|ALG12\_HUMAN sp|Q92879|CELF1\_HUMAN sp|Q96PH1|NOX5\_HUMAN sp|Q9Y5T5|UBP16\_HUMAN sp|Q13613|MTMR1\_HUMAN tr|F8WA39|F8WA39\_HUMAN sp|Q5VTR2|BRE1A\_HUMAN tr|F8W126|F8W126\_HUMAN sp|Q9P2N5|RBM27\_HUMAN sp|O43511|S26A4\_HUMAN sp|Q9H777|RNZ1\_HUMAN sp|P48436|SOX9\_HUMAN tr|E9PS66|E9PS66\_HUMAN tr|F8WEP8|F8WEP8\_HUMAN sp|Q9NZI7|UBIP1\_HUMAN tr|B5MC34|B5MC34\_HUMAN tr|B5MC36|B5MC36\_HUMAN sp|Q8N4W9|ZN808\_HUMAN sp|P41220|RGS2\_HUMAN tr|F6TT59|F6TT59\_HUMAN sp|P26012|ITB8\_HUMAN tr|H7C260|H7C260\_HUMAN tr|Q5VTW1|Q5VTW1\_HUMAN sp|P05546|HEP2\_HUMAN tr|Q96HV7|Q96HV7\_HUMAN tr|E9PFW2|E9PFW2\_HUMAN sp|Q02252|MMSA\_HUMAN sp|O00462|MANBA\_HUMAN sp|Q9C098|DCLK3\_HUMAN sp|O75911|DHRS3\_HUMAN tr|E7ETK5|E7ETK5\_HUMAN sp|P22695|QCR2\_HUMAN tr|M0R0K5|M0R0K5\_HUMAN sp|Q3C1V9|YK041\_HUMAN tr|E9PLC8|E9PLC8\_HUMAN sp|Q3SY56|SP6\_HUMAN sp|Q9Y624|JAM1\_HUMAN tr|A0A087WY82|A0A087WY82\_HUMAN tr|B4DFJ8|B4DFJ8\_HUMAN tr|A0A087WUW7|A0A087WUW7\_HUMAN tr|A0A087WWE9|A0A087WWE9\_HUMAN tr|A0A087WUD4|A0A087WUD4\_HUMAN tr|A0A087WTF5|A0A087WTF5\_HUMAN sp|Q9Y458|TBX22\_HUMAN tr|H3BS75|H3BS75\_HUMAN tr|A0A087X1Q3|A0A087X1Q3\_HUMAN tr|H7C5A9|H7C5A9\_HUMAN tr|Q8NCK8|Q8NCK8\_HUMAN tr|C9JT30|C9JT30\_HUMAN sp|Q8TDR2|STK35\_HUMAN tr|A0A087WYW3|A0A087WYW3\_HUMAN tr|J3KNK7|J3KNK7\_HUMAN sp|Q9Y264|ANGP4\_HUMAN sp|P05388|RLA0\_HUMAN sp|Q8TCU3|S7A13\_HUMAN sp|Q07837|SLC31\_HUMAN tr|B8ZZK1|B8ZZK1\_HUMAN tr|F8VWK7|F8VWK7\_HUMAN sp|Q14296|FASTK\_HUMAN tr|A0A087X0R9|A0A087X0R9\_HUMAN tr|G3V1R6|G3V1R6\_HUMAN tr|F8WF53|F8WF53\_HUMAN tr|G3V174|G3V174\_HUMAN tr|K7EN59|K7EN59\_HUMAN sp|Q8N8A2|ANR44\_HUMAN sp|Q9P2F5|STOX2\_HUMAN tr|H0YG43|H0YG43\_HUMAN sp|Q6XYQ8|SYT10\_HUMAN sp|P0C7Q6|S35G6\_HUMAN tr|H0YJ73|H0YJ73\_HUMAN sp|O95833|CLIC3\_HUMAN tr|C9JCD5|C9JCD5\_HUMAN tr|E7ERY9|E7ERY9\_HUMAN tr|C9J6C4|C9J6C4\_HUMAN tr|E7ERY4|E7ERY4\_HUMAN tr|E9PIB2|E9PIB2\_HUMAN sp|Q9NY47|CA2D2\_HUMAN sp|Q96DA2|RB39B\_HUMAN tr|C9JVC9|C9JVC9\_HUMAN tr|C9JE82|C9JE82\_HUMAN sp|O43847|NRDC\_HUMAN sp|Q9HBT7|ZN287\_HUMAN tr|H0Y5X4|H0Y5X4\_HUMAN tr|E5RIM3|E5RIM3\_HUMAN sp|Q8IUE1|TF2LX\_HUMAN sp|P59534|T2R39\_HUMAN sp|Q96KV6|BT2A3\_HUMAN sp|Q6P0A1|F180B\_HUMAN sp|Q96R08|OR5BC\_HUMAN tr|C9J6I7|C9J6I7\_HUMAN tr|H7C4E0|H7C4E0\_HUMAN sp|Q8WUU4|ZN296\_HUMAN tr|Q8IX33|Q8IX33\_HUMAN tr|F8VRS1|F8VRS1\_HUMAN tr|M0QZP6|M0QZP6\_HUMAN sp|Q16537|2A5E\_HUMAN sp|O43895|XPP2\_HUMAN sp|P15863|PAX1\_HUMAN tr|A0A087WXV5|A0A087WXV5\_HUMAN sp|P55771|PAX9\_HUMAN tr|H3BT24|H3BT24\_HUMAN tr|A0A075B723|A0A075B723\_HUMAN tr|E9PEX9|E9PEX9\_HUMAN tr|B4DFZ5|B4DFZ5\_HUMAN sp|O95198|KLHL2\_HUMAN sp|Q03014|HHEX\_HUMAN tr|F8WBW2|F8WBW2\_HUMAN sp|Q9C0B0|UNK\_HUMAN sp|O95067|CCNB2\_HUMAN sp|Q8IVS2|FABD\_HUMAN tr|F8W681|F8W681\_HUMAN tr|F8WE55|F8WE55\_HUMAN tr|C9JL25|C9JL25\_HUMAN sp|O75882|ATRN\_HUMAN tr|H0YHQ7|H0YHQ7\_HUMAN sp|Q8NI17|IL31R\_HUMAN tr|M0QZK6|M0QZK6\_HUMAN sp|Q00577|PURA\_HUMAN tr|K7EN36|K7EN36\_HUMAN tr|C9JT60|C9JT60\_HUMAN tr|C9J068|C9J068\_HUMAN sp|Q9UPW5|CBPC1\_HUMAN sp|O75928|PIAS2\_HUMAN sp|Q8ND07|BBOF1\_HUMAN sp|Q9H6R3|ACSS3\_HUMAN sp|Q8WWC4|CB047\_HUMAN tr|H7C0V0|H7C0V0\_HUMAN sp|A6NKF9|GPHRC\_HUMAN sp|B7ZAQ6|GPHRA\_HUMAN sp|P0CG08|GPHRB\_HUMAN sp|Q96P31|FCRL3\_HUMAN tr|J3KRH4|J3KRH4\_HUMAN tr|E9PJF1|E9PJF1\_HUMAN tr|E9PQQ2|E9PQQ2\_HUMAN tr|J3KSQ3|J3KSQ3\_HUMAN tr|B4DGB8|B4DGB8\_HUMAN tr|A0A087WVJ7|A0A087WVJ7\_HUMAN sp|O95838|GLP2R\_HUMAN tr|I3L0P5|I3L0P5\_HUMAN sp|Q96SA4|SERC2\_HUMAN sp|P47804|RGR\_HUMAN sp|O14874|BCKD\_HUMAN tr|K7EJD7|K7EJD7\_HUMAN tr|Q5VVC8|Q5VVC8\_HUMAN sp|P62913|RL11\_HUMAN tr|A0A087WW40|A0A087WW40\_HUMAN sp|Q9UQ16|DYN3\_HUMAN sp|Q96KN9|CXD4\_HUMAN sp|Q7L576|CYFP1\_HUMAN tr|A0A087X199|A0A087X199\_HUMAN sp|Q9C019|TRI15\_HUMAN tr|A0A087WVK4|A0A087WVK4\_HUMAN sp|Q5VXJ0|LIPK\_HUMAN tr|X6R8A1|X6R8A1\_HUMAN tr|F5H1X3|F5H1X3\_HUMAN sp|P41091|IF2G\_HUMAN tr|X6R5C5|X6R5C5\_HUMAN sp|Q9H0E7|UBP44\_HUMAN sp|Q2VIR3|IF2GL\_HUMAN tr|A0A087WU35|A0A087WU35\_HUMAN sp|P10619|PPGB\_HUMAN sp|Q96N38|ZN714\_HUMAN tr|F8W810|F8W810\_HUMAN tr|E7EX20|E7EX20\_HUMAN sp|Q14679|TTLL4\_HUMAN sp|Q8IYX3|CC116\_HUMAN tr|H0Y7C1|H0Y7C1\_HUMAN sp|Q711Q0|CJ071\_HUMAN sp|O00295|TULP2\_HUMAN sp|Q8NH00|OR2T4\_HUMAN sp|Q6PCB5|RSBNL\_HUMAN tr|X6R7U9|X6R7U9\_HUMAN sp|Q5JWF2|GNAS1\_HUMAN sp|Q86U38|NOP9\_HUMAN tr|H0Y5J9|H0Y5J9\_HUMAN sp|O95347|SMC2\_HUMAN tr|B7ZLW4|B7ZLW4\_HUMAN tr|A0A087X152|A0A087X152\_HUMAN sp|O95185|UNC5C\_HUMAN tr|H0YCP9|H0YCP9\_HUMAN tr|E7EV89|E7EV89\_HUMAN sp|A6NDL7|MT21E\_HUMAN tr|D6RHE0|D6RHE0\_HUMAN sp|P30453|1A34\_HUMAN sp|Q9Y639|NPTN\_HUMAN sp|P04279|SEMG1\_HUMAN tr|K7ENA6|K7ENA6\_HUMAN sp|P32121|ARRB2\_HUMAN sp|O15294|OGT1\_HUMAN tr|A0A087X1M9|A0A087X1M9\_HUMAN sp|Q8NH64|O51A7\_HUMAN tr|D6REY2|D6REY2\_HUMAN sp|Q9NZS2|KLRF1\_HUMAN tr|A0A096LP08|A0A096LP08\_HUMAN tr|B7Z2X5|B7Z2X5\_HUMAN tr|H0YDC6|H0YDC6\_HUMAN tr|A0A087WYA8|A0A087WYA8\_HUMAN tr|H0YBY0|H0YBY0\_HUMAN sp|P20916|MAG\_HUMAN sp|Q96S55|WRIP1\_HUMAN sp|Q9P0W8|SPAT7\_HUMAN sp|P33241|LSP1\_HUMAN tr|D6RHG9|D6RHG9\_HUMAN tr|D6RHC6|D6RHC6\_HUMAN sp|Q7LFL8|CXXC5\_HUMAN tr|D6RBE0|D6RBE0\_HUMAN tr|D6R966|D6R966\_HUMAN tr|E7EV55|E7EV55\_HUMAN tr|D6RIR8|D6RIR8\_HUMAN tr|D6R9V1|D6R9V1\_HUMAN tr|D6RDY2|D6RDY2\_HUMAN tr|E7EVI8|E7EVI8\_HUMAN tr|D6RCN9|D6RCN9\_HUMAN tr|J3QLE9|J3QLE9\_HUMAN sp|O43889|CREB3\_HUMAN tr|B7ZAX5|B7ZAX5\_HUMAN tr|J3QQH7|J3QQH7\_HUMAN tr|A0A087WW13|A0A087WW13\_HUMAN tr|Q9BST8|Q9BST8\_HUMAN sp|Q969V5|MUL1\_HUMAN sp|Q8TDS5|OXER1\_HUMAN tr|Q6NZX9|Q6NZX9\_HUMAN sp|Q4ZIN3|MBRL\_HUMAN sp|Q96C03|MID49\_HUMAN sp|Q8IYD1|ERF3B\_HUMAN sp|Q9Y5W7|SNX14\_HUMAN tr|K7EKM2|K7EKM2\_HUMAN sp|Q8NI38|IKBD\_HUMAN sp|P47211|GALR1\_HUMAN sp|Q5SQN1|SNP47\_HUMAN tr|U3KPT7|U3KPT7\_HUMAN tr|A0A087X0B7|A0A087X0B7\_HUMAN tr|H7C3C7|H7C3C7\_HUMAN sp|P55809|SCOT1\_HUMAN sp|Q02487|DSC2\_HUMAN tr|H7C1C8|H7C1C8\_HUMAN tr|H0YCL3|H0YCL3\_HUMAN sp|Q7RTN6|STRAA\_HUMAN sp|Q96R47|O2A14\_HUMAN sp|Q8N3Y1|FBXW8\_HUMAN sp|Q9Y2I2|NTNG1\_HUMAN sp|Q96QF0|RAB3I\_HUMAN tr|D6RDY0|D6RDY0\_HUMAN tr|F8WC35|F8WC35\_HUMAN sp|O15205|UBD\_HUMAN sp|Q9NVI1|FANCI\_HUMAN tr|C9J3Z9|C9J3Z9\_HUMAN tr|R4GNI4|R4GNI4\_HUMAN tr|R4GMS4|R4GMS4\_HUMAN tr|H0YKM7|H0YKM7\_HUMAN sp|Q86XI2|CNDG2\_HUMAN tr|H0YG99|H0YG99\_HUMAN tr|F5H3X8|F5H3X8\_HUMAN sp|Q8IZE3|PACE1\_HUMAN tr|H3BQN8|H3BQN8\_HUMAN tr|H3BSG4|H3BSG4\_HUMAN sp|Q9UJJ7|RUSD1\_HUMAN tr|C9J8D4|C9J8D4\_HUMAN tr|A0A087X222|A0A087X222\_HUMAN sp|Q13724|MOGS\_HUMAN tr|H3BMZ6|H3BMZ6\_HUMAN sp|P52597|HNRPF\_HUMAN sp|Q96P26|5NT1B\_HUMAN sp|Q01955|CO4A3\_HUMAN tr|F6T1Q0|F6T1Q0\_HUMAN tr|F8VWP4|F8VWP4\_HUMAN sp|A1L453|PRS38\_HUMAN tr|A0A087WZV9|A0A087WZV9\_HUMAN sp|Q5SRR4|LY65C\_HUMAN tr|K7N7D6|K7N7D6\_HUMAN tr|H7C140|H7C140\_HUMAN tr|H7BYB1|H7BYB1\_HUMAN tr|M0R2H6|M0R2H6\_HUMAN tr|E9PD61|E9PD61\_HUMAN tr|E7ENK3|E7ENK3\_HUMAN tr|F5H1W5|F5H1W5\_HUMAN tr|E7EPY9|E7EPY9\_HUMAN sp|P23759|PAX7\_HUMAN tr|D6R9F0|D6R9F0\_HUMAN sp|Q92610|ZN592\_HUMAN sp|Q9NRR6|INP5E\_HUMAN sp|O94830|DDHD2\_HUMAN sp|Q8NAU1|FNDC5\_HUMAN sp|Q53HC5|KLH26\_HUMAN tr|F5H3R9|F5H3R9\_HUMAN sp|Q9Y5Z4|HEBP2\_HUMAN tr|A0A087X1P2|A0A087X1P2\_HUMAN sp|Q9GIP4|LAT1L\_HUMAN sp|Q9HD90|NDF4\_HUMAN sp|P11166|GTR1\_HUMAN sp|Q9P2H3|IFT80\_HUMAN tr|H3BRJ5|H3BRJ5\_HUMAN sp|P29371|NK3R\_HUMAN sp|Q8NCU7|C2C4A\_HUMAN sp|P23458|JAK1\_HUMAN tr|A0A087WSX5|A0A087WSX5\_HUMAN sp|Q8N434|SVOPL\_HUMAN sp|Q5EBL2|ZN628\_HUMAN tr|B4DV59|B4DV59\_HUMAN tr|H0Y5M9|H0Y5M9\_HUMAN sp|Q08379|GOGA2\_HUMAN tr|A0A087WVK0|A0A087WVK0\_HUMAN sp|Q6ZMB5|T184A\_HUMAN tr|B8ZZR0|B8ZZR0\_HUMAN sp|P51398|RT29\_HUMAN tr|G3V2W1|G3V2W1\_HUMAN sp|P01019|ANGT\_HUMAN tr|E7EN44|E7EN44\_HUMAN tr|G3V3F5|G3V3F5\_HUMAN sp|Q9NXT0|ZN586\_HUMAN tr|G3V4B4|G3V4B4\_HUMAN sp|O14972|DSCR3\_HUMAN tr|A0A087WYH3|A0A087WYH3\_HUMAN tr|E9PCD1|E9PCD1\_HUMAN tr|H0Y4P9|H0Y4P9\_HUMAN sp|Q13042|CDC16\_HUMAN tr|Q5T8C6|Q5T8C6\_HUMAN tr|Q7Z651|Q7Z651\_HUMAN sp|P42681|TXK\_HUMAN tr|I3L2Z9|I3L2Z9\_HUMAN sp|Q8NGJ8|O51S1\_HUMAN sp|P49005|DPOD2\_HUMAN sp|Q8NBW4|S38A9\_HUMAN tr|G3V4Y7|G3V4Y7\_HUMAN tr|F8W8R3|F8W8R3\_HUMAN tr|A0A087WXY2|A0A087WXY2\_HUMAN sp|Q8IU99|CAHM1\_HUMAN sp|Q9HCJ1|ANKH\_HUMAN sp|Q96P56|CTSR2\_HUMAN sp|O76024|WFS1\_HUMAN sp|P15260|INGR1\_HUMAN sp|Q5SRH9|TT39A\_HUMAN sp|Q15622|OR7A5\_HUMAN tr|G3V3D1|G3V3D1\_HUMAN sp|Q8N9R8|SCAI\_HUMAN sp|O75346|ZN253\_HUMAN sp|O95059|RPP14\_HUMAN sp|Q7Z3V5|ZN571\_HUMAN sp|Q969R5|LMBL2\_HUMAN tr|E9PL69|E9PL69\_HUMAN sp|O95674|CDS2\_HUMAN tr|B7Z757|B7Z757\_HUMAN tr|K4DIA9|K4DIA9\_HUMAN tr|E9PNE7|E9PNE7\_HUMAN tr|E9PRH4|E9PRH4\_HUMAN sp|Q9NSU2|TREX1\_HUMAN tr|E9PMI2|E9PMI2\_HUMAN sp|P30939|5HT1F\_HUMAN sp|Q6BCY4|NB5R2\_HUMAN sp|A1L4K1|FSD2\_HUMAN sp|Q9H999|PANK3\_HUMAN sp|O42043|ENK18\_HUMAN sp|Q96LW2|SG494\_HUMAN tr|A4D1K4|A4D1K4\_HUMAN tr|Q5T2X4|Q5T2X4\_HUMAN sp|C9JQI7|TM232\_HUMAN tr|A0A087WTP5|A0A087WTP5\_HUMAN tr|A0A087WXB2|A0A087WXB2\_HUMAN tr|D3DPQ1|D3DPQ1\_HUMAN sp|Q8NGS9|O13C2\_HUMAN tr|Q5SVK0|Q5SVK0\_HUMAN tr|S4R2X4|S4R2X4\_HUMAN tr|H0Y5Z6|H0Y5Z6\_HUMAN tr|Q5SVK5|Q5SVK5\_HUMAN tr|J3KQ16|J3KQ16\_HUMAN sp|Q9Y651|SOX21\_HUMAN tr|Q5T097|Q5T097\_HUMAN tr|G3V4Y8|G3V4Y8\_HUMAN sp|P54750|PDE1A\_HUMAN tr|H0Y9H4|H0Y9H4\_HUMAN tr|C9J0J0|C9J0J0\_HUMAN sp|Q8N4C8|MINK1\_HUMAN tr|Q5QNY5|Q5QNY5\_HUMAN tr|F8WBH4|F8WBH4\_HUMAN tr|C9JZT2|C9JZT2\_HUMAN sp|O76002|OR2J2\_HUMAN tr|D6RH24|D6RH24\_HUMAN sp|P40855|PEX19\_HUMAN sp|Q6ZU67|BEND4\_HUMAN tr|I3L247|I3L247\_HUMAN sp|Q2MJR0|SPRE3\_HUMAN tr|I3L208|I3L208\_HUMAN sp|P34910|EVI2B\_HUMAN tr|Q86WV7|Q86WV7\_HUMAN sp|Q96MW1|CCD43\_HUMAN tr|K7EKL5|K7EKL5\_HUMAN tr|K7ENM0|K7ENM0\_HUMAN tr|H7C1N9|H7C1N9\_HUMAN tr|K7EMN9|K7EMN9\_HUMAN tr|K7ESE0|K7ESE0\_HUMAN tr|K7EJD8|K7EJD8\_HUMAN tr|M0QXC1|M0QXC1\_HUMAN tr|M0R045|M0R045\_HUMAN tr|E9PLN6|E9PLN6\_HUMAN sp|Q9H0H3|KLH25\_HUMAN tr|K7EJ49|K7EJ49\_HUMAN sp|Q0D2I5|IFFO1\_HUMAN tr|H0YM74|H0YM74\_HUMAN tr|D6R9X6|D6R9X6\_HUMAN sp|P12931|SRC\_HUMAN sp|P26599|PTBP1\_HUMAN sp|Q5JRM2|CX066\_HUMAN tr|B7Z7H5|B7Z7H5\_HUMAN tr|C9JST2|C9JST2\_HUMAN sp|P32297|ACHA3\_HUMAN sp|Q8NHJ6|LIRB4\_HUMAN tr|Q5T4Y8|Q5T4Y8\_HUMAN tr|H3BQ53|H3BQ53\_HUMAN sp|Q9UI46|DNAI1\_HUMAN sp|P47883|OR3A4\_HUMAN sp|Q6P988|NOTUM\_HUMAN tr|C9JJ54|C9JJ54\_HUMAN tr|A0A087WWV9|A0A087WWV9\_HUMAN tr|E9PE19|E9PE19\_HUMAN sp|O14544|SOCS6\_HUMAN sp|Q8N3U4|STAG2\_HUMAN sp|Q16696|CP2AD\_HUMAN tr|C9K0F9|C9K0F9\_HUMAN sp|Q13889|TF2H3\_HUMAN tr|H0YFF7|H0YFF7\_HUMAN tr|F5GWD3|F5GWD3\_HUMAN tr|E5RHF2|E5RHF2\_HUMAN sp|Q6ZS62|COLC1\_HUMAN tr|A0A087WY55|A0A087WY55\_HUMAN tr|H7C229|H7C229\_HUMAN tr|E7EVJ5|E7EVJ5\_HUMAN sp|Q9NP79|VTA1\_HUMAN sp|Q96F07|CYFP2\_HUMAN sp|Q8N9H6|CH031\_HUMAN sp|Q14765|STAT4\_HUMAN tr|E7EW33|E7EW33\_HUMAN sp|Q9UQ07|MOK\_HUMAN sp|P28066|PSA5\_HUMAN tr|H7BZ47|H7BZ47\_HUMAN tr|H7C2P5|H7C2P5\_HUMAN tr|H0Y3A4|H0Y3A4\_HUMAN sp|Q9NZJ7|MTCH1\_HUMAN sp|Q7Z417|NUFP2\_HUMAN sp|O75081|MTG16\_HUMAN tr|F8VRU3|F8VRU3\_HUMAN sp|Q8NDZ2|SIMC1\_HUMAN sp|Q14520|HABP2\_HUMAN tr|H3BM22|H3BM22\_HUMAN tr|H0Y452|H0Y452\_HUMAN tr|A0A087WWK8|A0A087WWK8\_HUMAN sp|Q6DN90|IQEC1\_HUMAN tr|E5RJG6|E5RJG6\_HUMAN sp|Q13585|MTR1L\_HUMAN tr|H7C0Q8|H7C0Q8\_HUMAN tr|A8MWG7|A8MWG7\_HUMAN Q28065 tr|F8VRN5|F8VRN5\_HUMAN tr|E7EQI7|E7EQI7\_HUMAN sp|P31260|HXA10\_HUMAN sp|Q12768|STRUM\_HUMAN tr|E5RJ49|E5RJ49\_HUMAN tr|E7EMF1|E7EMF1\_HUMAN tr|E7ESP4|E7ESP4\_HUMAN tr|H7C4V1|H7C4V1\_HUMAN sp|Q96M34|CC030\_HUMAN tr|S4R404|S4R404\_HUMAN P02676 sp|Q9Y5Q0|FADS3\_HUMAN tr|A0A087WVJ8|A0A087WVJ8\_HUMAN tr|H3BMF4|H3BMF4\_HUMAN tr|I3L0W2|I3L0W2\_HUMAN sp|O75019|LIRA1\_HUMAN sp|Q9H2V7|SPNS1\_HUMAN sp|Q15465|SHH\_HUMAN sp|O95104|SFR15\_HUMAN tr|B0QY89|B0QY89\_HUMAN tr|H7BYZ4|H7BYZ4\_HUMAN sp|Q9Y262|EIF3L\_HUMAN sp|P20936|RASA1\_HUMAN tr|J3QT52|J3QT52\_HUMAN sp|Q86UW1|OSTA\_HUMAN tr|Q7Z7A5|Q7Z7A5\_HUMAN tr|H0Y430|H0Y430\_HUMAN sp|P05113|IL5\_HUMAN tr|C9JQP9|C9JQP9\_HUMAN sp|P40238|TPOR\_HUMAN sp|Q9H0B6|KLC2\_HUMAN sp|Q60I27|AL2CL\_HUMAN sp|Q5U5X8|F222A\_HUMAN sp|P40222|TXLNA\_HUMAN tr|A0A087WTU1|A0A087WTU1\_HUMAN tr|J3QLC5|J3QLC5\_HUMAN sp|O75616|ERAL1\_HUMAN sp|Q6ZVW7|I17EL\_HUMAN tr|E9PNW5|E9PNW5\_HUMAN sp|Q9P2E2|KIF17\_HUMAN tr|X6RC15|X6RC15\_HUMAN tr|D6RBC1|D6RBC1\_HUMAN sp|Q96LB0|MRGX3\_HUMAN tr|A0A087WUT6|A0A087WUT6\_HUMAN tr|E9PPY5|E9PPY5\_HUMAN sp|P19388|RPAB1\_HUMAN sp|Q6EMB2|TTLL5\_HUMAN tr|J3KSK2|J3KSK2\_HUMAN tr|G3V2J9|G3V2J9\_HUMAN sp|O95206|PCDH8\_HUMAN tr|B6ZDE5|B6ZDE5\_HUMAN sp|P10523|ARRS\_HUMAN sp|P56746|CLD15\_HUMAN sp|P40938|RFC3\_HUMAN tr|E9PFH2|E9PFH2\_HUMAN sp|Q9BY66|KDM5D\_HUMAN tr|B4DWY7|B4DWY7\_HUMAN sp|Q9HBY8|SGK2\_HUMAN tr|A0A075B6R8|A0A075B6R8\_HUMAN tr|A6NM42|A6NM42\_HUMAN tr|H0YKW5|H0YKW5\_HUMAN sp|Q9HBA0|TRPV4\_HUMAN sp|Q08477|CP4F3\_HUMAN sp|Q494V2|CCD37\_HUMAN sp|P01893|HLAH\_HUMAN tr|A0A087X0S7|A0A087X0S7\_HUMAN tr|H7C5T3|H7C5T3\_HUMAN tr|B4DYM4|B4DYM4\_HUMAN tr|C9JU82|C9JU82\_HUMAN sp|P25098|ARBK1\_HUMAN tr|A6NG92|A6NG92\_HUMAN sp|Q9NZH5|PTTG2\_HUMAN sp|Q8N1V2|CFA52\_HUMAN sp|Q9BSH4|TACO1\_HUMAN sp|Q6UWU4|CF089\_HUMAN sp|O75380|NDUS6\_HUMAN sp|Q8TEL6|TP4AP\_HUMAN tr|D6RBT3|D6RBT3\_HUMAN sp|P35606|COPB2\_HUMAN sp|P35227|PCGF2\_HUMAN sp|Q9NRE2|TSH2\_HUMAN tr|B9A041|B9A041\_HUMAN tr|B8ZZ51|B8ZZ51\_HUMAN sp|P40925|MDHC\_HUMAN sp|Q05209|PTN12\_HUMAN sp|Q9UJU5|FOXD3\_HUMAN sp|Q9ULV3|CIZ1\_HUMAN sp|Q99675|CGRF1\_HUMAN tr|F5H2X7|F5H2X7\_HUMAN sp|Q9Y5P4|C43BP\_HUMAN sp|O95573|ACSL3\_HUMAN tr|H0YC16|H0YC16\_HUMAN sp|Q5JPH6|SYEM\_HUMAN tr|D6RJI3|D6RJI3\_HUMAN tr|H3BTB7|H3BTB7\_HUMAN tr|H3BRF5|H3BRF5\_HUMAN tr|G3V4Y0|G3V4Y0\_HUMAN tr|M0QXU9|M0QXU9\_HUMAN tr|H3BQ90|H3BQ90\_HUMAN tr|H3BPM8|H3BPM8\_HUMAN tr|M0R0T1|M0R0T1\_HUMAN sp|Q6NXR0|IIGP5\_HUMAN tr|J3KPL7|J3KPL7\_HUMAN sp|Q8IV32|CCD71\_HUMAN sp|Q5T5X7|BEND3\_HUMAN sp|A4D2H0|CTGEF\_HUMAN sp|Q8N7X1|RMXL3\_HUMAN sp|Q86UF2|CTGE6\_HUMAN tr|E7ET84|E7ET84\_HUMAN tr|H0YBY7|H0YBY7\_HUMAN sp|Q9NZB2|F120A\_HUMAN sp|P0CB46|CASPG\_HUMAN tr|A0A087WUX9|A0A087WUX9\_HUMAN tr|A0A075B6T8|A0A075B6T8\_HUMAN sp|Q9Y5Y2|NUBP2\_HUMAN tr|H7C2Q0|H7C2Q0\_HUMAN tr|H0YJH0|H0YJH0\_HUMAN sp|I3L273|GFY\_HUMAN tr|A0A075B6S9|A0A075B6S9\_HUMAN sp|Q5TGY3|AHDC1\_HUMAN sp|Q86YS7|C2CD5\_HUMAN sp|P00352|AL1A1\_HUMAN sp|Q01113|IL9R\_HUMAN tr|H7C071|H7C071\_HUMAN tr|H7C2M8|H7C2M8\_HUMAN tr|F8WF98|F8WF98\_HUMAN sp|Q8N6G5|CGAT2\_HUMAN tr|A0A087WX41|A0A087WX41\_HUMAN tr|G3V2H1|G3V2H1\_HUMAN sp|Q9Y4P8|WIPI2\_HUMAN tr|M0QZZ1|M0QZZ1\_HUMAN sp|Q9H1J7|WNT5B\_HUMAN tr|K7ENF6|K7ENF6\_HUMAN tr|H0YAK1|H0YAK1\_HUMAN sp|O75069|TMCC2\_HUMAN sp|Q8WYA0|IFT81\_HUMAN tr|H3BS42|H3BS42\_HUMAN tr|B3KPE6|B3KPE6\_HUMAN sp|Q9H5H4|ZN768\_HUMAN tr|J3KMY0|J3KMY0\_HUMAN tr|H0YHE2|H0YHE2\_HUMAN sp|Q52LC2|VAS1L\_HUMAN sp|Q8WTR8|NET5\_HUMAN tr|B1AMA2|B1AMA2\_HUMAN tr|A0A087WUH3|A0A087WUH3\_HUMAN sp|O95551|TYDP2\_HUMAN sp|Q8TF65|GIPC2\_HUMAN tr|B9A012|B9A012\_HUMAN tr|A0A087WV21|A0A087WV21\_HUMAN tr|A0A087WT71|A0A087WT71\_HUMAN sp|Q9NP70|AMBN\_HUMAN sp|P20023|CR2\_HUMAN sp|Q92782|DPF1\_HUMAN tr|E9PEZ7|E9PEZ7\_HUMAN sp|Q14108|SCRB2\_HUMAN tr|A0A087X0X3|A0A087X0X3\_HUMAN sp|Q03188|CENPC\_HUMAN sp|Q9ULH1|ASAP1\_HUMAN tr|H0Y8J2|H0Y8J2\_HUMAN sp|Q9H1U9|S2551\_HUMAN tr|V9H019|V9H019\_HUMAN tr|C9J872|C9J872\_HUMAN sp|Q9NSC7|SIA7A\_HUMAN tr|H7C4Q8|H7C4Q8\_HUMAN sp|Q14188|TFDP2\_HUMAN tr|C9J461|C9J461\_HUMAN sp|Q9NQC1|JADE2\_HUMAN tr|G3XAA4|G3XAA4\_HUMAN tr|H7C0K6|H7C0K6\_HUMAN sp|Q9Y5P3|RAI2\_HUMAN sp|Q8NBN7|RDH13\_HUMAN tr|I3NI36|I3NI36\_HUMAN sp|Q8N4M7|CJ126\_HUMAN tr|B0ZBF6|B0ZBF6\_HUMAN tr|B0ZBF8|B0ZBF8\_HUMAN sp|Q8NGK6|O52I1\_HUMAN sp|Q7L8C5|SYT13\_HUMAN sp|Q96FK6|WDR89\_HUMAN tr|J3KQU7|J3KQU7\_HUMAN sp|Q9H190|SDCB2\_HUMAN sp|P22223|CADH3\_HUMAN sp|P08236|BGLR\_HUMAN sp|Q8WUM4|PDC6I\_HUMAN tr|H0Y5P4|H0Y5P4\_HUMAN sp|Q8NGL9|OR4CG\_HUMAN sp|Q9BV94|EDEM2\_HUMAN ENSEMBL:ENSBTAP00000025008 sp|Q96CM8|ACSF2\_HUMAN tr|E9PF16|E9PF16\_HUMAN sp|Q5VW00|DC122\_HUMAN sp|P55916|UCP3\_HUMAN tr|F2Z305|F2Z305\_HUMAN sp|Q86YJ6|THNS2\_HUMAN sp|Q9NVV4|PAPD1\_HUMAN sp|O75161|NPHP4\_HUMAN sp|P28698|MZF1\_HUMAN sp|Q96QF7|ACRC\_HUMAN tr|E9PI62|E9PI62\_HUMAN sp|P48443|RXRG\_HUMAN tr|J3KR84|J3KR84\_HUMAN sp|A6NHZ5|LR14B\_HUMAN tr|F8WCX5|F8WCX5\_HUMAN tr|Q8WUT1|Q8WUT1\_HUMAN sp|Q96JN2|CC136\_HUMAN tr|H0YI31|H0YI31\_HUMAN sp|Q8N8L6|ARL10\_HUMAN sp|Q6PCB7|S27A1\_HUMAN sp|Q6P3R8|NEK5\_HUMAN tr|A0A087WUP7|A0A087WUP7\_HUMAN sp|Q9NQQ7|S35C2\_HUMAN sp|P49019|HCAR3\_HUMAN sp|Q8N131|PORIM\_HUMAN sp|Q16864|VATF\_HUMAN tr|B7ZBK4|B7ZBK4\_HUMAN sp|Q6PD74|AAGAB\_HUMAN tr|H7C4J7|H7C4J7\_HUMAN tr|H0YKH0|H0YKH0\_HUMAN sp|P01859|IGHG2\_HUMAN tr|Q6ZSL4|Q6ZSL4\_HUMAN tr|R9R4D9|R9R4D9\_HUMAN sp|Q5UAW9|GP157\_HUMAN tr|Q658J9|Q658J9\_HUMAN tr|H0YCD6|H0YCD6\_HUMAN sp|Q8TAT5|NEIL3\_HUMAN tr|K7ERQ0|K7ERQ0\_HUMAN sp|Q5BJH7|YIF1B\_HUMAN sp|P59773|K102L\_HUMAN sp|Q9GZR7|DDX24\_HUMAN tr|D6RF22|D6RF22\_HUMAN tr|G3V529|G3V529\_HUMAN tr|K7EIT1|K7EIT1\_HUMAN sp|Q96E11|RRFM\_HUMAN tr|H0Y2X5|H0Y2X5\_HUMAN sp|P08123|CO1A2\_HUMAN tr|A0A087WTA8|A0A087WTA8\_HUMAN sp|P47895|AL1A3\_HUMAN tr|D6RFF0|D6RFF0\_HUMAN sp|Q9Y2P0|ZN835\_HUMAN sp|Q9BYD6|RM01\_HUMAN sp|Q9C0J1|B3GN4\_HUMAN sp|P11802|CDK4\_HUMAN tr|F8VWX7|F8VWX7\_HUMAN sp|Q8WU49|CG033\_HUMAN tr|A2AAZ6|A2AAZ6\_HUMAN tr|B3KM87|B3KM87\_HUMAN sp|Q16620|NTRK2\_HUMAN tr|A0A087WV86|A0A087WV86\_HUMAN sp|Q86WC4|OSTM1\_HUMAN sp|Q6PGQ7|BORA\_HUMAN sp|Q96LQ0|PPR36\_HUMAN tr|A0A096LPJ5|A0A096LPJ5\_HUMAN sp|Q8N442|GUF1\_HUMAN sp|Q96GS6|AB17A\_HUMAN tr|Q5T3I4|Q5T3I4\_HUMAN sp|Q8TDB4|HUMMR\_HUMAN tr|F8WCE5|F8WCE5\_HUMAN tr|H7C3G8|H7C3G8\_HUMAN sp|Q9BX40|LS14B\_HUMAN sp|Q8IZF5|GP113\_HUMAN sp|P37268|FDFT\_HUMAN sp|Q9BT92|TCHP\_HUMAN tr|B4DRK2|B4DRK2\_HUMAN sp|Q9NVE4|CCD87\_HUMAN tr|C9JWJ8|C9JWJ8\_HUMAN sp|Q9Y6I7|WSB1\_HUMAN sp|Q15776|ZKSC8\_HUMAN sp|Q9BXP2|S12A9\_HUMAN tr|J3KSL8|J3KSL8\_HUMAN tr|J3QSA1|J3QSA1\_HUMAN tr|I3L1T9|I3L1T9\_HUMAN sp|Q86VB7|C163A\_HUMAN tr|B0YIW2|B0YIW2\_HUMAN tr|H7BXH9|H7BXH9\_HUMAN tr|H7BY57|H7BY57\_HUMAN tr|E9PQG0|E9PQG0\_HUMAN tr|H7BY53|H7BY53\_HUMAN sp|Q9Y4U1|MMAC\_HUMAN tr|H7C3A1|H7C3A1\_HUMAN sp|Q9NRK6|ABCBA\_HUMAN sp|A6NIK2|LR10B\_HUMAN tr|C9JAJ6|C9JAJ6\_HUMAN tr|C9IY66|C9IY66\_HUMAN sp|P59901|LIRA4\_HUMAN tr|Q5QPD4|Q5QPD4\_HUMAN tr|Q5QPD2|Q5QPD2\_HUMAN tr|A3KFJ0|A3KFJ0\_HUMAN tr|A3KFJ1|A3KFJ1\_HUMAN sp|Q8IYB5|SMAP1\_HUMAN tr|E5RG56|E5RG56\_HUMAN sp|Q12797|ASPH\_HUMAN sp|Q8NC44|F134A\_HUMAN tr|A0A087X1X9|A0A087X1X9\_HUMAN sp|Q8N7U6|EFHB\_HUMAN tr|H9KV82|H9KV82\_HUMAN sp|Q96KA5|CLP1L\_HUMAN tr|L7MUG5|L7MUG5\_HUMAN tr|J3KRR8|J3KRR8\_HUMAN sp|Q495M9|USH1G\_HUMAN tr|J3QRV4|J3QRV4\_HUMAN tr|K7ERF4|K7ERF4\_HUMAN tr|K7EMM4|K7EMM4\_HUMAN tr|Q9UEL6|Q9UEL6\_HUMAN sp|Q96SF2|TCPQM\_HUMAN sp|Q8NGY6|OR6N2\_HUMAN sp|Q8NH55|O52E5\_HUMAN sp|Q96SE0|ABHD1\_HUMAN tr|F8WD46|F8WD46\_HUMAN sp|O60938|KERA\_HUMAN sp|P21506|ZNF10\_HUMAN sp|Q6P2C0|WDR93\_HUMAN sp|Q14156|EFR3A\_HUMAN sp|O15123|ANGP2\_HUMAN tr|E7EVQ3|E7EVQ3\_HUMAN tr|H0YH64|H0YH64\_HUMAN tr|M0QX29|M0QX29\_HUMAN sp|Q9H553|ALG2\_HUMAN sp|P36959|GMPR1\_HUMAN tr|H0YLL1|H0YLL1\_HUMAN tr|H3BT15|H3BT15\_HUMAN tr|D6RBV2|D6RBV2\_HUMAN tr|C9K0I9|C9K0I9\_HUMAN tr|H3BTX6|H3BTX6\_HUMAN tr|E7EQD9|E7EQD9\_HUMAN tr|C9JYW1|C9JYW1\_HUMAN tr|C9JUP5|C9JUP5\_HUMAN sp|O95140|MFN2\_HUMAN sp|Q9H6I2|SOX17\_HUMAN sp|Q8NEM2|SHCBP\_HUMAN sp|P46019|KPB2\_HUMAN sp|Q9H5Y7|SLIK6\_HUMAN tr|G3V5S4|G3V5S4\_HUMAN sp|Q02962|PAX2\_HUMAN sp|P11168|GTR2\_HUMAN tr|Q5SZP1|Q5SZP1\_HUMAN tr|G3V200|G3V200\_HUMAN tr|H7C1P1|H7C1P1\_HUMAN tr|E7ENX8|E7ENX8\_HUMAN tr|A0A5E8|A0A5E8\_HUMAN tr|F5H8D1|F5H8D1\_HUMAN sp|Q99501|GA2L1\_HUMAN sp|Q9C0F1|CEP44\_HUMAN sp|Q14872|MTF1\_HUMAN sp|P0C025|NUD17\_HUMAN sp|O15466|SIA8E\_HUMAN tr|D6RC25|D6RC25\_HUMAN tr|D6RBX1|D6RBX1\_HUMAN tr|D6RGX6|D6RGX6\_HUMAN sp|O94906|PRP6\_HUMAN tr|G3V3J9|G3V3J9\_HUMAN sp|Q9NS71|GKN1\_HUMAN sp|Q4L180|FIL1L\_HUMAN tr|C9JYJ6|C9JYJ6\_HUMAN sp|Q9UP38|FZD1\_HUMAN sp|Q13367|AP3B2\_HUMAN sp|Q9Y6Y0|NS1BP\_HUMAN sp|Q9BY49|PECR\_HUMAN sp|P51795|CLCN5\_HUMAN sp|Q96AM1|MRGRF\_HUMAN tr|A0A096LNX8|A0A096LNX8\_HUMAN tr|G8JLM4|G8JLM4\_HUMAN sp|Q9Y6R9|CCD61\_HUMAN tr|H0Y5A1|H0Y5A1\_HUMAN tr|C9JPI1|C9JPI1\_HUMAN tr|C9JG07|C9JG07\_HUMAN tr|C9JIA0|C9JIA0\_HUMAN sp|Q9BZI1|IRX2\_HUMAN sp|Q96LB1|MRGX2\_HUMAN tr|C9JP71|C9JP71\_HUMAN tr|H7C309|H7C309\_HUMAN sp|P55085|PAR2\_HUMAN tr|C9J8J5|C9J8J5\_HUMAN sp|Q8IZ08|GP135\_HUMAN sp|O95500|CLD14\_HUMAN tr|H0YD79|H0YD79\_HUMAN sp|Q5VTT2|CI135\_HUMAN sp|P78563|RED1\_HUMAN tr|H7C1E9|H7C1E9\_HUMAN sp|Q92618|ZN516\_HUMAN tr|K7EKZ1|K7EKZ1\_HUMAN sp|Q8NFA0|UBP32\_HUMAN tr|E5RIW1|E5RIW1\_HUMAN tr|J3KT00|J3KT00\_HUMAN sp|Q9BPW8|NIPS1\_HUMAN sp|Q08945|SSRP1\_HUMAN sp|Q6NW40|RGMB\_HUMAN tr|J3KNF6|J3KNF6\_HUMAN sp|Q9ULP9|TBC24\_HUMAN sp|Q9H0I9|TKTL2\_HUMAN tr|G3V357|G3V357\_HUMAN tr|I3L3Y3|I3L3Y3\_HUMAN tr|A0A087WYU4|A0A087WYU4\_HUMAN sp|P59544|T2R50\_HUMAN sp|A6NH52|TV23A\_HUMAN sp|Q96GD3|SCMH1\_HUMAN tr|F5GXW1|F5GXW1\_HUMAN sp|Q9NSV4|DIAP3\_HUMAN tr|A0A087WTF6|A0A087WTF6\_HUMAN tr|A0A087WV75|A0A087WV75\_HUMAN tr|E7ET89|E7ET89\_HUMAN tr|A0A087WX77|A0A087WX77\_HUMAN tr|J3QL92|J3QL92\_HUMAN sp|Q86UQ8|NFE4\_HUMAN sp|O43815|STRN\_HUMAN tr|Q5SVM7|Q5SVM7\_HUMAN sp|P13591|NCAM1\_HUMAN tr|A0A087WWD4|A0A087WWD4\_HUMAN tr|H9KV31|H9KV31\_HUMAN sp|O15394|NCAM2\_HUMAN tr|M0R1P1|M0R1P1\_HUMAN tr|Q5QPV1|Q5QPV1\_HUMAN tr|C9JLT6|C9JLT6\_HUMAN sp|O75150|BRE1B\_HUMAN tr|H3BP71|H3BP71\_HUMAN sp|Q5VZ18|SHE\_HUMAN sp|Q9H6L2|TM231\_HUMAN sp|Q9BT25|HAUS8\_HUMAN tr|H0YHU2|H0YHU2\_HUMAN tr|A0A087WYD4|A0A087WYD4\_HUMAN tr|H7C3V8|H7C3V8\_HUMAN tr|F8W001|F8W001\_HUMAN tr|A0A087WUW4|A0A087WUW4\_HUMAN tr|C9JAA8|C9JAA8\_HUMAN tr|H7C4J6|H7C4J6\_HUMAN P02754 sp|Q9NSA2|KCND1\_HUMAN sp|Q8NDF8|PAPD5\_HUMAN tr|A6NNT0|A6NNT0\_HUMAN tr|H3BQM0|H3BQM0\_HUMAN sp|Q6IC98|GRAM4\_HUMAN sp|P32456|GBP2\_HUMAN tr|F8VV04|F8VV04\_HUMAN tr|F6WMF4|F6WMF4\_HUMAN sp|P41219|PERI\_HUMAN sp|Q6T4P5|LPPR3\_HUMAN tr|A0A087WXQ2|A0A087WXQ2\_HUMAN tr|A0A087WTL2|A0A087WTL2\_HUMAN sp|Q9P291|ARMX1\_HUMAN sp|Q9UKT4|FBX5\_HUMAN sp|Q5QJE6|TDIF2\_HUMAN tr|H7C3H1|H7C3H1\_HUMAN tr|C9JJK9|C9JJK9\_HUMAN tr|C9JVY3|C9JVY3\_HUMAN tr|C9JMI3|C9JMI3\_HUMAN tr|C9JGN4|C9JGN4\_HUMAN tr|V9GY85|V9GY85\_HUMAN sp|O60568|PLOD3\_HUMAN tr|C9JHD7|C9JHD7\_HUMAN tr|I3L2F9|I3L2F9\_HUMAN sp|P57772|SELB\_HUMAN tr|C9J8T0|C9J8T0\_HUMAN sp|Q92466|DDB2\_HUMAN sp|D6RJB6|U17LK\_HUMAN sp|Q0WX57|U17LO\_HUMAN sp|Q6QN14|U17L6\_HUMAN sp|A8MUK1|U17L5\_HUMAN sp|D6R901|U17LL\_HUMAN sp|C9JPN9|UL17C\_HUMAN sp|D6RA61|U17LM\_HUMAN tr|A2AB20|A2AB20\_HUMAN sp|C9J2P7|U17LF\_HUMAN sp|O14958|CASQ2\_HUMAN sp|Q9UKN8|TF3C4\_HUMAN tr|A0A087WZK6|A0A087WZK6\_HUMAN tr|I3L1G2|I3L1G2\_HUMAN ENSEMBL:ENSBTAP00000031900 sp|Q7RTZ2|U17L1\_HUMAN tr|D6RD23|D6RD23\_HUMAN tr|H0YLU7|H0YLU7\_HUMAN tr|D6RIH0|D6RIH0\_HUMAN tr|B7Z8X0|B7Z8X0\_HUMAN sp|P13804|ETFA\_HUMAN sp|A6NCW7|U17L4\_HUMAN tr|E9PBL0|E9PBL0\_HUMAN tr|A0A096LNL8|A0A096LNL8\_HUMAN tr|D6RB02|D6RB02\_HUMAN sp|A6NCW0|U17L3\_HUMAN sp|Q9NWS1|PARI\_HUMAN tr|E2QRB3|E2QRB3\_HUMAN tr|M0QXT4|M0QXT4\_HUMAN sp|Q8WTS6|SETD7\_HUMAN sp|Q7L4I2|RSRC2\_HUMAN tr|D6RJA0|D6RJA0\_HUMAN sp|A8MTJ6|FOXI3\_HUMAN tr|M0QY95|M0QY95\_HUMAN sp|P52746|ZN142\_HUMAN sp|Q96PV4|PNMA5\_HUMAN sp|P02656|APOC3\_HUMAN tr|C9J2Q0|C9J2Q0\_HUMAN sp|Q6EEV6|SUMO4\_HUMAN tr|E9PC47|E9PC47\_HUMAN sp|Q9NP90|RAB9B\_HUMAN sp|O95644|NFAC1\_HUMAN sp|Q03167|TGBR3\_HUMAN sp|Q14240|IF4A2\_HUMAN sp|Q5BJF6|ODFP2\_HUMAN sp|Q502W6|VWA3B\_HUMAN tr|F5GXL4|F5GXL4\_HUMAN tr|H7BZY7|H7BZY7\_HUMAN sp|O76090|BEST1\_HUMAN sp|P19447|ERCC3\_HUMAN tr|K7EM67|K7EM67\_HUMAN sp|P28472|GBRB3\_HUMAN tr|F5H7N0|F5H7N0\_HUMAN sp|Q9H0M0|WWP1\_HUMAN sp|Q8WXE1|ATRIP\_HUMAN tr|F5H626|F5H626\_HUMAN sp|Q8NHV4|NEDD1\_HUMAN sp|Q0VF49|K2012\_HUMAN sp|Q9GZS0|DNAI2\_HUMAN sp|Q8NDQ6|ZN540\_HUMAN tr|J3QRG2|J3QRG2\_HUMAN sp|O14786|NRP1\_HUMAN tr|H7C097|H7C097\_HUMAN tr|E9PEP6|E9PEP6\_HUMAN sp|Q8IY47|KBTB2\_HUMAN sp|P43007|SATT\_HUMAN tr|H0YAF7|H0YAF7\_HUMAN sp|Q14332|FZD2\_HUMAN tr|H3BM21|H3BM21\_HUMAN sp|P05106|ITB3\_HUMAN tr|A0A087X0A8|A0A087X0A8\_HUMAN tr|M0R276|M0R276\_HUMAN sp|Q9UBN1|CCG4\_HUMAN tr|Q5T8U5|Q5T8U5\_HUMAN sp|O00221|IKBE\_HUMAN tr|A0A087WVX9|A0A087WVX9\_HUMAN tr|D6RFK0|D6RFK0\_HUMAN tr|D6RGE7|D6RGE7\_HUMAN sp|P15169|CBPN\_HUMAN tr|K7EQ35|K7EQ35\_HUMAN tr|D6RBI3|D6RBI3\_HUMAN sp|P10826|RARB\_HUMAN tr|E9PJR3|E9PJR3\_HUMAN sp|B0I1T2|MYO1G\_HUMAN tr|D6RAD5|D6RAD5\_HUMAN tr|F8W0U9|F8W0U9\_HUMAN sp|Q149M9|NWD1\_HUMAN tr|C9JBL4|C9JBL4\_HUMAN sp|P55268|LAMB2\_HUMAN sp|Q9HD40|SPCS\_HUMAN tr|A0A087WV47|A0A087WV47\_HUMAN tr|A0A087X1C7|A0A087X1C7\_HUMAN sp|P01857|IGHG1\_HUMAN tr|E7ENS2|E7ENS2\_HUMAN tr|E7EPR4|E7EPR4\_HUMAN tr|E7EN32|E7EN32\_HUMAN tr|E7ET29|E7ET29\_HUMAN sp|Q86VZ6|JAZF1\_HUMAN sp|Q96AQ7|CIDEC\_HUMAN tr|H0Y403|H0Y403\_HUMAN tr|C9JWY1|C9JWY1\_HUMAN sp|Q6P9B6|TLDC1\_HUMAN sp|Q9BWW9|APOL5\_HUMAN tr|A8MYR7|A8MYR7\_HUMAN tr|A0A087X050|A0A087X050\_HUMAN sp|Q9UPV9|TRAK1\_HUMAN tr|E7END7|E7END7\_HUMAN sp|P62820|RAB1A\_HUMAN sp|Q9H0U4|RAB1B\_HUMAN tr|C9JC32|C9JC32\_HUMAN sp|Q93099|HGD\_HUMAN tr|G5E9U9|G5E9U9\_HUMAN sp|Q9H0J9|PAR12\_HUMAN tr|B8ZZC8|B8ZZC8\_HUMAN sp|Q8N0V5|GNT2A\_HUMAN tr|C9JD14|C9JD14\_HUMAN sp|Q8NFM4|ADCY4\_HUMAN sp|Q6UW78|CK083\_HUMAN tr|F8WAT5|F8WAT5\_HUMAN sp|Q6TFL4|KLH24\_HUMAN sp|Q7Z602|GP141\_HUMAN sp|Q9BWT3|PAPOG\_HUMAN tr|Q5TCQ3|Q5TCQ3\_HUMAN sp|Q9UMS5|PHTF1\_HUMAN tr|Q5TCQ5|Q5TCQ5\_HUMAN tr|Q5TI73|Q5TI73\_HUMAN tr|Q5TI75|Q5TI75\_HUMAN tr|A6NGY7|A6NGY7\_HUMAN sp|Q6ZQN5|FOXI2\_HUMAN sp|P16581|LYAM2\_HUMAN tr|Q3ZCV0|Q3ZCV0\_HUMAN sp|P30411|BKRB2\_HUMAN sp|O95932|TGM3L\_HUMAN tr|F8VR14|F8VR14\_HUMAN tr|H0Y6P5|H0Y6P5\_HUMAN tr|D6R9B1|D6R9B1\_HUMAN tr|H0YCJ2|H0YCJ2\_HUMAN tr|D6RHI1|D6RHI1\_HUMAN tr|H0Y6V4|H0Y6V4\_HUMAN tr|E5RK47|E5RK47\_HUMAN sp|Q9UBU8|MO4L1\_HUMAN tr|H0YMJ0|H0YMJ0\_HUMAN tr|H0YLJ3|H0YLJ3\_HUMAN tr|H0YBQ3|H0YBQ3\_HUMAN tr|E5RJ88|E5RJ88\_HUMAN tr|B3KTM8|B3KTM8\_HUMAN tr|H0YBJ4|H0YBJ4\_HUMAN sp|P29083|T2EA\_HUMAN tr|C9IYL4|C9IYL4\_HUMAN sp|Q9Y2K6|UBP20\_HUMAN tr|J3KNX4|J3KNX4\_HUMAN sp|Q2TAK8|MUM1\_HUMAN tr|D6RI07|D6RI07\_HUMAN tr|D6RF40|D6RF40\_HUMAN tr|D6RAG5|D6RAG5\_HUMAN tr|Q5T376|Q5T376\_HUMAN tr|A0A087WT20|A0A087WT20\_HUMAN sp|Q96D42|HAVR1\_HUMAN sp|Q14849|STAR3\_HUMAN tr|H7C3D4|H7C3D4\_HUMAN sp|Q92552|RT27\_HUMAN sp|Q9UKB1|FBW1B\_HUMAN sp|Q5HYK7|SH319\_HUMAN tr|A0A075B6I7|A0A075B6I7\_HUMAN tr|A0A087WTP0|A0A087WTP0\_HUMAN tr|B1AHB1|B1AHB1\_HUMAN sp|Q8NGD3|OR4K5\_HUMAN sp|P33765|AA3R\_HUMAN tr|I3L182|I3L182\_HUMAN tr|E9PRF2|E9PRF2\_HUMAN tr|H0UIB3|H0UIB3\_HUMAN sp|Q14680|MELK\_HUMAN tr|H0Y845|H0Y845\_HUMAN tr|Q5SQT6|Q5SQT6\_HUMAN sp|Q15181|IPYR\_HUMAN sp|Q15678|PTN14\_HUMAN sp|Q9Y375|CIA30\_HUMAN tr|H7C524|H7C524\_HUMAN sp|P26378|ELAV4\_HUMAN tr|B1APY8|B1APY8\_HUMAN tr|B1APY9|B1APY9\_HUMAN sp|Q9H501|ESF1\_HUMAN sp|Q8TDT2|GP152\_HUMAN tr|D6RHU4|D6RHU4\_HUMAN tr|A0A087X1F6|A0A087X1F6\_HUMAN sp|Q53FE4|CD017\_HUMAN tr|B4DIW9|B4DIW9\_HUMAN sp|O95992|CH25H\_HUMAN tr|H7C3F4|H7C3F4\_HUMAN sp|Q9H019|MFR1L\_HUMAN sp|Q96HR8|NAF1\_HUMAN tr|H0YBT2|H0YBT2\_HUMAN tr|E9PMS7|E9PMS7\_HUMAN sp|Q15025|TNIP1\_HUMAN tr|A8MT23|A8MT23\_HUMAN tr|E5RGT6|E5RGT6\_HUMAN tr|F6THM6|F6THM6\_HUMAN tr|A9Z1Z2|A9Z1Z2\_HUMAN sp|Q8NEZ3|WDR19\_HUMAN tr|G3V180|G3V180\_HUMAN sp|Q9NY33|DPP3\_HUMAN sp|Q8N5B7|CERS5\_HUMAN tr|G3V1D3|G3V1D3\_HUMAN tr|C9JA41|C9JA41\_HUMAN sp|A8MWY0|K132L\_HUMAN tr|F8WCY0|F8WCY0\_HUMAN sp|O94806|KPCD3\_HUMAN tr|H7C2N5|H7C2N5\_HUMAN sp|Q86YI8|PHF13\_HUMAN tr|R4GN58|R4GN58\_HUMAN tr|Q5HYA6|Q5HYA6\_HUMAN sp|Q86WV1|SKAP1\_HUMAN sp|O75844|FACE1\_HUMAN sp|O60909|B4GT2\_HUMAN sp|Q9BVC3|DCC1\_HUMAN sp|Q9NQ40|S52A3\_HUMAN tr|F8VQ14|F8VQ14\_HUMAN sp|P09496|CLCA\_HUMAN tr|C9J8P9|C9J8P9\_HUMAN sp|Q8N3F8|MILK1\_HUMAN sp|Q9UPZ3|HPS5\_HUMAN sp|P51801|CLCKB\_HUMAN sp|Q7L8L6|FAKD5\_HUMAN tr|C9JVN1|C9JVN1\_HUMAN sp|O75164|KDM4A\_HUMAN tr|Q5T5N3|Q5T5N3\_HUMAN sp|Q6P1N0|C2D1A\_HUMAN sp|Q86W28|NALP8\_HUMAN sp|Q9C0B6|BRNP2\_HUMAN tr|H0Y4Q6|H0Y4Q6\_HUMAN tr|Q5JWM4|Q5JWM4\_HUMAN tr|C9JRQ8|C9JRQ8\_HUMAN tr|E9PG15|E9PG15\_HUMAN sp|Q53RD9|FBLN7\_HUMAN tr|B8ZZC1|B8ZZC1\_HUMAN tr|B4DP81|B4DP81\_HUMAN tr|H7C0I1|H7C0I1\_HUMAN tr|C9J9N5|C9J9N5\_HUMAN sp|Q14749|GNMT\_HUMAN tr|Q5RJ85|Q5RJ85\_HUMAN sp|P17693|HLAG\_HUMAN sp|Q7Z3E5|ARMC9\_HUMAN tr|K7EQX3|K7EQX3\_HUMAN tr|A0A087X1I8|A0A087X1I8\_HUMAN tr|H3BQ65|H3BQ65\_HUMAN sp|Q3V5L5|MGT5B\_HUMAN sp|P60891|PRPS1\_HUMAN tr|B1ALA9|B1ALA9\_HUMAN tr|F8VS29|F8VS29\_HUMAN tr|H3BSW6|H3BSW6\_HUMAN sp|Q2VPK5|CTU2\_HUMAN tr|H0YNM6|H0YNM6\_HUMAN sp|Q6DCA0|AMERL\_HUMAN tr|B4DY40|B4DY40\_HUMAN sp|Q14C87|T132D\_HUMAN tr|A0A087WUP9|A0A087WUP9\_HUMAN sp|P22492|H1T\_HUMAN tr|J3QRV1|J3QRV1\_HUMAN tr|J3KSE2|J3KSE2\_HUMAN sp|Q13103|SPP24\_HUMAN sp|Q9UQ03|COR2B\_HUMAN sp|Q96B33|CLD23\_HUMAN tr|Q5SWC8|Q5SWC8\_HUMAN sp|O43296|ZN264\_HUMAN tr|X6RGJ2|X6RGJ2\_HUMAN Q9N2I2 sp|Q8IW70|T151B\_HUMAN tr|F8W6G6|F8W6G6\_HUMAN sp|Q9NPD5|SO1B3\_HUMAN tr|B1AHL2|B1AHL2\_HUMAN sp|Q86WB7|UN93A\_HUMAN tr|D6RFH7|D6RFH7\_HUMAN sp|Q8NFZ4|NLGN2\_HUMAN tr|F8W8U2|F8W8U2\_HUMAN sp|Q7Z6P3|RAB44\_HUMAN sp|E7EU14|PP5D1\_HUMAN tr|D6R976|D6R976\_HUMAN sp|Q9ULS5|TMCC3\_HUMAN sp|Q13287|NMI\_HUMAN tr|G3V207|G3V207\_HUMAN sp|A8MQ11|PM2P5\_HUMAN sp|Q6P9G4|TM154\_HUMAN sp|O00515|LAD1\_HUMAN sp|Q9P2K5|MYEF2\_HUMAN tr|A0A087WUT0|A0A087WUT0\_HUMAN sp|Q13227|GPS2\_HUMAN tr|I3L4X7|I3L4X7\_HUMAN tr|F2Z2B9|F2Z2B9\_HUMAN sp|Q9BSJ2|GCP2\_HUMAN tr|F6TR96|F6TR96\_HUMAN sp|Q6ZT89|S2548\_HUMAN tr|J3KQI1|J3KQI1\_HUMAN tr|M0QYE7|M0QYE7\_HUMAN sp|O00748|EST2\_HUMAN sp|Q6TCH7|PAQR3\_HUMAN tr|F8W784|F8W784\_HUMAN tr|C9JB85|C9JB85\_HUMAN sp|O00469|PLOD2\_HUMAN sp|Q9H4I8|SEHL2\_HUMAN tr|F2Z3M3|F2Z3M3\_HUMAN tr|M0QZE8|M0QZE8\_HUMAN sp|Q9BRH9|ZN251\_HUMAN tr|E7EWM1|E7EWM1\_HUMAN sp|Q6UWM9|UD2A3\_HUMAN tr|D6RBL8|D6RBL8\_HUMAN tr|F5GZZ9|F5GZZ9\_HUMAN tr|H7C270|H7C270\_HUMAN sp|Q96PM9|Z385A\_HUMAN tr|K7EQ95|K7EQ95\_HUMAN sp|Q13530|SERC3\_HUMAN tr|H0YCC9|H0YCC9\_HUMAN tr|G3V325|G3V325\_HUMAN tr|K7ENG9|K7ENG9\_HUMAN sp|O75127|PTCD1\_HUMAN tr|J3QS83|J3QS83\_HUMAN sp|P23416|GLRA2\_HUMAN sp|P0C628|O5AC1\_HUMAN sp|O15409|FOXP2\_HUMAN tr|A8MUV4|A8MUV4\_HUMAN tr|B7ZBM3|B7ZBM3\_HUMAN tr|X6R6F3|X6R6F3\_HUMAN tr|F8W8A5|F8W8A5\_HUMAN tr|C9J4K5|C9J4K5\_HUMAN tr|C9JSP0|C9JSP0\_HUMAN sp|Q9NYP3|DONS\_HUMAN tr|H7C1C1|H7C1C1\_HUMAN tr|D6RCE4|D6RCE4\_HUMAN tr|H0YHT7|H0YHT7\_HUMAN sp|Q8IWA0|WDR75\_HUMAN tr|A0A087WU89|A0A087WU89\_HUMAN sp|P05556|ITB1\_HUMAN sp|Q8NGB9|OR4F6\_HUMAN sp|P42338|PK3CB\_HUMAN tr|H0YAH5|H0YAH5\_HUMAN sp|Q92613|JADE3\_HUMAN tr|H0Y544|H0Y544\_HUMAN tr|D6RDU9|D6RDU9\_HUMAN tr|B4E0P2|B4E0P2\_HUMAN tr|H0Y9F2|H0Y9F2\_HUMAN sp|P15144|AMPN\_HUMAN sp|Q5VZ46|K1614\_HUMAN tr|C9K0I0|C9K0I0\_HUMAN sp|P35612|ADDB\_HUMAN sp|Q9NX01|TXN4B\_HUMAN tr|Q96LM7|Q96LM7\_HUMAN sp|P29317|EPHA2\_HUMAN tr|C9J1V9|C9J1V9\_HUMAN tr|D6RBD7|D6RBD7\_HUMAN tr|B5MCS2|B5MCS2\_HUMAN tr|F8W0I5|F8W0I5\_HUMAN sp|A6NJU9|NPIL5\_HUMAN sp|Q9ULJ3|ZBT21\_HUMAN sp|P27658|CO8A1\_HUMAN sp|Q0VAK6|LMOD3\_HUMAN tr|F5H3U5|F5H3U5\_HUMAN sp|Q96GM5|SMRD1\_HUMAN sp|Q9H209|O10A4\_HUMAN tr|J3KSR4|J3KSR4\_HUMAN tr|J3KPC0|J3KPC0\_HUMAN sp|Q4V9L6|TM119\_HUMAN sp|P56880|CLD20\_HUMAN sp|Q86XD8|ZFAN4\_HUMAN tr|K7ES27|K7ES27\_HUMAN sp|Q96MF4|CC140\_HUMAN tr|A0A5B5|A0A5B5\_HUMAN tr|A0A087WZ08|A0A087WZ08\_HUMAN sp|Q86UW7|CAPS2\_HUMAN tr|A6ZIE3|A6ZIE3\_HUMAN tr|H7BYR4|H7BYR4\_HUMAN tr|A0A087X1P3|A0A087X1P3\_HUMAN tr|H7C4L8|H7C4L8\_HUMAN sp|Q8IZF4|GP114\_HUMAN tr|B7Z7E1|B7Z7E1\_HUMAN tr|F8W8P5|F8W8P5\_HUMAN tr|E9PEJ4|E9PEJ4\_HUMAN tr|C9IYE1|C9IYE1\_HUMAN sp|P51911|CNN1\_HUMAN sp|Q7Z5J4|RAI1\_HUMAN sp|Q9UBD0|HSFX1\_HUMAN sp|Q96MC6|HIAT1\_HUMAN sp|Q92771|DDX12\_HUMAN tr|E7ETZ8|E7ETZ8\_HUMAN sp|Q49MG5|MAP9\_HUMAN sp|O75626|PRDM1\_HUMAN tr|A2VCS9|A2VCS9\_HUMAN tr|A0A087WU16|A0A087WU16\_HUMAN sp|P31314|TLX1\_HUMAN sp|P61371|ISL1\_HUMAN sp|O75909|CCNK\_HUMAN sp|Q9NX40|OCAD1\_HUMAN tr|D6RG39|D6RG39\_HUMAN tr|D6RBN5|D6RBN5\_HUMAN tr|D6R9T5|D6R9T5\_HUMAN tr|D6RIT9|D6RIT9\_HUMAN sp|Q9ULC3|RAB23\_HUMAN tr|F5GWH4|F5GWH4\_HUMAN sp|Q01959|SC6A3\_HUMAN sp|Q9NYB0|TE2IP\_HUMAN sp|Q9NRN7|ADPPT\_HUMAN tr|G3V229|G3V229\_HUMAN tr|G3V412|G3V412\_HUMAN tr|G3V2K9|G3V2K9\_HUMAN sp|P01716|LV402\_HUMAN tr|C9JIG0|C9JIG0\_HUMAN tr|K7EL17|K7EL17\_HUMAN tr|Q68DZ5|Q68DZ5\_HUMAN tr|B1ANA3|B1ANA3\_HUMAN tr|B2REB0|B2REB0\_HUMAN Q2KJ83 tr|Q5T6P2|Q5T6P2\_HUMAN tr|F8VWP7|F8VWP7\_HUMAN tr|E9PC86|E9PC86\_HUMAN sp|Q9BQI5|SGIP1\_HUMAN sp|P13232|IL7\_HUMAN tr|Q5FBX5|Q5FBX5\_HUMAN tr|D6RGY4|D6RGY4\_HUMAN sp|O15455|TLR3\_HUMAN sp|Q8NBF2|NHLC2\_HUMAN sp|Q6P9B9|INT5\_HUMAN sp|Q5JSH3|WDR44\_HUMAN tr|H0YAX8|H0YAX8\_HUMAN sp|P54727|RD23B\_HUMAN tr|K7ER91|K7ER91\_HUMAN sp|P12110|CO6A2\_HUMAN tr|K7ENX3|K7ENX3\_HUMAN sp|Q9H4B7|TBB1\_HUMAN ENSEMBL:ENSBTAP00000006074 sp|Q8NH57|O52P1\_HUMAN tr|C9K0D8|C9K0D8\_HUMAN tr|C9JA89|C9JA89\_HUMAN tr|B7Z739|B7Z739\_HUMAN tr|E7EU49|E7EU49\_HUMAN sp|Q9NR81|ARHG3\_HUMAN tr|E9PG37|E9PG37\_HUMAN Q3MHN2 tr|H0Y6W2|H0Y6W2\_HUMAN tr|J3QSC0|J3QSC0\_HUMAN tr|A2A3C4|A2A3C4\_HUMAN tr|B4DQ67|B4DQ67\_HUMAN tr|S4R3V3|S4R3V3\_HUMAN sp|Q9NQN1|OR2S1\_HUMAN tr|E5RFK3|E5RFK3\_HUMAN sp|Q6NXE6|ARMC6\_HUMAN sp|Q53GQ0|DHB12\_HUMAN sp|Q6ZMS7|ZN783\_HUMAN sp|Q1MX18|INSC\_HUMAN sp|O60885|BRD4\_HUMAN sp|Q8NE28|STKL1\_HUMAN sp|P51530|DNA2\_HUMAN sp|Q13057|COASY\_HUMAN tr|H3BNQ9|H3BNQ9\_HUMAN tr|F5H7Q6|F5H7Q6\_HUMAN tr|H7C3K1|H7C3K1\_HUMAN tr|H0YI84|H0YI84\_HUMAN sp|O75506|HSBP1\_HUMAN sp|A9YTQ3|AHRR\_HUMAN sp|Q8IZ16|CG061\_HUMAN tr|H3BP78|H3BP78\_HUMAN tr|F5H364|F5H364\_HUMAN tr|I3L0N3|I3L0N3\_HUMAN sp|P46459|NSF\_HUMAN sp|Q7Z388|D19L4\_HUMAN tr|B5MCT9|B5MCT9\_HUMAN sp|Q8NBH2|KY\_HUMAN sp|Q86VZ1|P2RY8\_HUMAN sp|Q96NR8|RDH12\_HUMAN tr|K7ENJ9|K7ENJ9\_HUMAN tr|H0YND0|H0YND0\_HUMAN sp|O75298|RTN2\_HUMAN sp|O14792|HS3S1\_HUMAN tr|Q5JYR7|Q5JYR7\_HUMAN tr|J3KP65|J3KP65\_HUMAN sp|P55011|S12A2\_HUMAN tr|H0YM91|H0YM91\_HUMAN sp|O15178|BRAC\_HUMAN sp|P58181|O10A3\_HUMAN sp|Q96RE7|NACC1\_HUMAN tr|M0QY46|M0QY46\_HUMAN sp|Q16602|CALRL\_HUMAN sp|Q8TDU6|GPBAR\_HUMAN tr|A0A096LPD9|A0A096LPD9\_HUMAN sp|Q8WU79|SMAP2\_HUMAN tr|H0YH25|H0YH25\_HUMAN sp|Q9UJ78|ZMYM5\_HUMAN sp|A8MU10|YQ047\_HUMAN tr|H0Y6W4|H0Y6W4\_HUMAN tr|A0A087WV97|A0A087WV97\_HUMAN tr|E7EU71|E7EU71\_HUMAN sp|Q02338|BDH\_HUMAN tr|A0A087WZ32|A0A087WZ32\_HUMAN sp|Q6WCQ1|MPRIP\_HUMAN tr|A8K727|A8K727\_HUMAN sp|Q9HB19|PKHA2\_HUMAN tr|Q5JVD1|Q5JVD1\_HUMAN sp|P22314|UBA1\_HUMAN tr|K7EKC0|K7EKC0\_HUMAN sp|Q8IWE4|DCNL3\_HUMAN sp|Q14153|FA53B\_HUMAN sp|Q5FYB1|ARSI\_HUMAN sp|Q9Y5E3|PCDB6\_HUMAN tr|B4DGA7|B4DGA7\_HUMAN sp|Q5JQS5|OR2BB\_HUMAN sp|Q8NAT2|TDRD5\_HUMAN sp|P11182|ODB2\_HUMAN tr|C9K073|C9K073\_HUMAN tr|C9J2Z7|C9J2Z7\_HUMAN tr|C9JF13|C9JF13\_HUMAN sp|Q8NGU9|GP150\_HUMAN sp|P51170|SCNNG\_HUMAN sp|Q9NY64|GTR8\_HUMAN tr|H3BTS7|H3BTS7\_HUMAN tr|A0A087WW14|A0A087WW14\_HUMAN sp|A6H8Z2|F221B\_HUMAN tr|F8W8N9|F8W8N9\_HUMAN sp|O75363|BCAS1\_HUMAN tr|G3XAF7|G3XAF7\_HUMAN tr|H0Y542|H0Y542\_HUMAN tr|J3KR23|J3KR23\_HUMAN sp|O14638|ENPP3\_HUMAN tr|H3BQJ5|H3BQJ5\_HUMAN tr|H7C2Z3|H7C2Z3\_HUMAN tr|E7ERY8|E7ERY8\_HUMAN sp|Q16891|MIC60\_HUMAN tr|H0YDX3|H0YDX3\_HUMAN sp|Q9BUL9|RPP25\_HUMAN tr|H7C4G6|H7C4G6\_HUMAN sp|Q8N1L4|CP4Z2\_HUMAN tr|J3KSJ1|J3KSJ1\_HUMAN sp|Q9UBL6|CPNE7\_HUMAN sp|O60279|SUSD5\_HUMAN tr|E9PQZ5|E9PQZ5\_HUMAN tr|C9JR56|C9JR56\_HUMAN sp|Q6SJ93|F111B\_HUMAN tr|H7C2G0|H7C2G0\_HUMAN sp|Q16082|HSPB2\_HUMAN sp|Q2KHT4|GSG1\_HUMAN tr|A6PW57|A6PW57\_HUMAN sp|Q8N5S1|S2541\_HUMAN sp|Q15366|PCBP2\_HUMAN sp|Q9NX45|SOLH2\_HUMAN tr|J3QSZ5|J3QSZ5\_HUMAN tr|H7BXE3|H7BXE3\_HUMAN tr|G3V4F2|G3V4F2\_HUMAN tr|A0A087WTU6|A0A087WTU6\_HUMAN tr|A0A087WWN4|A0A087WWN4\_HUMAN tr|E7ESG9|E7ESG9\_HUMAN tr|G3V124|G3V124\_HUMAN sp|Q9NRS4|TMPS4\_HUMAN tr|E9PK08|E9PK08\_HUMAN sp|Q8IXW5|RPAP2\_HUMAN tr|H0YB83|H0YB83\_HUMAN sp|Q9BZM5|N2DL2\_HUMAN sp|Q96DB5|RMD1\_HUMAN tr|E5RH53|E5RH53\_HUMAN tr|M0R0Y3|M0R0Y3\_HUMAN tr|H7C165|H7C165\_HUMAN sp|Q5T9C9|PI5L1\_HUMAN tr|F2Z393|F2Z393\_HUMAN sp|P37837|TALDO\_HUMAN sp|P0C7U0|ELFN1\_HUMAN sp|P02458|CO2A1\_HUMAN sp|O00237|RN103\_HUMAN sp|P16109|LYAM3\_HUMAN tr|A0A087WZ67|A0A087WZ67\_HUMAN tr|Q5VVY6|Q5VVY6\_HUMAN sp|O60811|PRAM2\_HUMAN sp|O43688|LPP2\_HUMAN sp|P11712|CP2C9\_HUMAN sp|Q8N122|RPTOR\_HUMAN tr|E9PD68|E9PD68\_HUMAN sp|Q14194|DPYL1\_HUMAN sp|P54317|LIPR2\_HUMAN tr|H0YA91|H0YA91\_HUMAN sp|P22004|BMP6\_HUMAN tr|A0A087WX88|A0A087WX88\_HUMAN sp|P61550|ENVT1\_HUMAN sp|P34998|CRFR1\_HUMAN tr|J9JIC6|J9JIC6\_HUMAN sp|Q7Z5H5|VN1R4\_HUMAN sp|O75475|PSIP1\_HUMAN sp|Q63ZY3|KANK2\_HUMAN tr|H7BXM6|H7BXM6\_HUMAN sp|Q13410|BT1A1\_HUMAN sp|P48546|GIPR\_HUMAN tr|K7EPD9|K7EPD9\_HUMAN tr|H3BTS6|H3BTS6\_HUMAN sp|Q9Y5Y3|GPR45\_HUMAN tr|H0Y3S5|H0Y3S5\_HUMAN sp|Q14247|SRC8\_HUMAN sp|Q6P4D5|F222C\_HUMAN sp|Q9NP61|ARFG3\_HUMAN tr|K7EKZ0|K7EKZ0\_HUMAN tr|E5RIF2|E5RIF2\_HUMAN tr|Q5SUY4|Q5SUY4\_HUMAN sp|Q96TA0|PCDBI\_HUMAN tr|E9PPV6|E9PPV6\_HUMAN sp|Q9H773|DCTP1\_HUMAN tr|J3KS36|J3KS36\_HUMAN sp|Q9H2G9|GO45\_HUMAN tr|J3QSE2|J3QSE2\_HUMAN tr|E9PR70|E9PR70\_HUMAN tr|C9J3F6|C9J3F6\_HUMAN tr|Q5W042|Q5W042\_HUMAN sp|Q9NTK5|OLA1\_HUMAN sp|Q08345|DDR1\_HUMAN sp|Q9HAK2|COE2\_HUMAN tr|B7Z934|B7Z934\_HUMAN sp|O94829|IPO13\_HUMAN sp|P45985|MP2K4\_HUMAN tr|M0R167|M0R167\_HUMAN tr|A8K0U1|A8K0U1\_HUMAN tr|M0R1Q9|M0R1Q9\_HUMAN tr|K7ESP7|K7ESP7\_HUMAN sp|Q8N584|TT39C\_HUMAN tr|Q8WX70|Q8WX70\_HUMAN tr|K7ENL9|K7ENL9\_HUMAN tr|A0A087WZD4|A0A087WZD4\_HUMAN sp|Q5R372|RBG1L\_HUMAN sp|Q96DM3|MIC1\_HUMAN sp|Q86WC6|PPR27\_HUMAN tr|V9GYG1|V9GYG1\_HUMAN tr|Q5JVD2|Q5JVD2\_HUMAN sp|Q9UNF0|PACN2\_HUMAN tr|B5MCZ8|B5MCZ8\_HUMAN sp|Q86YT6|MIB1\_HUMAN sp|P55822|SH3BG\_HUMAN tr|F8WJN3|F8WJN3\_HUMAN sp|Q16630|CPSF6\_HUMAN tr|Q504U8|Q504U8\_HUMAN sp|Q96M27|PRRC1\_HUMAN sp|Q6IN85|P4R3A\_HUMAN tr|J3QSE7|J3QSE7\_HUMAN tr|C9JZ61|C9JZ61\_HUMAN tr|H0YA88|H0YA88\_HUMAN tr|F8VQ19|F8VQ19\_HUMAN sp|P0CW00|TSPY8\_HUMAN tr|F8WDE6|F8WDE6\_HUMAN sp|P07332|FES\_HUMAN tr|B4DGU6|B4DGU6\_HUMAN tr|A6NGL4|A6NGL4\_HUMAN tr|Q6ZW95|Q6ZW95\_HUMAN sp|Q9UBB6|NCDN\_HUMAN tr|A6NDJ3|A6NDJ3\_HUMAN sp|Q8NAM6|ZSCA4\_HUMAN sp|Q8TDV5|GP119\_HUMAN tr|H7C450|H7C450\_HUMAN sp|Q17RM4|CC142\_HUMAN sp|Q96PU5|NED4L\_HUMAN tr|A0A087X119|A0A087X119\_HUMAN sp|Q5VTH9|WDR78\_HUMAN sp|P52888|THOP1\_HUMAN sp|O00214|LEG8\_HUMAN sp|Q9NVR2|INT10\_HUMAN tr|H7BXD8|H7BXD8\_HUMAN tr|E5RGZ9|E5RGZ9\_HUMAN sp|Q96RI9|TAAR9\_HUMAN sp|Q9H3M0|KCNF1\_HUMAN tr|H7C2T8|H7C2T8\_HUMAN tr|Q5VZW6|Q5VZW6\_HUMAN tr|A0A087WUD2|A0A087WUD2\_HUMAN sp|O14798|TR10C\_HUMAN tr|H0YDI4|H0YDI4\_HUMAN sp|Q9H8T0|AKTIP\_HUMAN tr|J3QRS6|J3QRS6\_HUMAN tr|E9PPE6|E9PPE6\_HUMAN sp|Q9P2X3|IMPCT\_HUMAN sp|Q9H1Z4|WDR13\_HUMAN tr|I3NI32|I3NI32\_HUMAN tr|D6RB01|D6RB01\_HUMAN sp|Q9Y6X4|F169A\_HUMAN tr|B1B1G6|B1B1G6\_HUMAN tr|A0A087WWP9|A0A087WWP9\_HUMAN tr|H7C0C3|H7C0C3\_HUMAN tr|E7EPK1|E7EPK1\_HUMAN tr|E7EVK1|E7EVK1\_HUMAN tr|B1B1G3|B1B1G3\_HUMAN tr|B1B1G4|B1B1G4\_HUMAN tr|H7C2U8|H7C2U8\_HUMAN tr|F8W1T8|F8W1T8\_HUMAN sp|Q6ZVK8|NUD18\_HUMAN tr|A2A274|A2A274\_HUMAN tr|H7C5L6|H7C5L6\_HUMAN tr|H7C521|H7C521\_HUMAN sp|Q8ND24|RN214\_HUMAN tr|J3QL24|J3QL24\_HUMAN tr|A0A087WTE6|A0A087WTE6\_HUMAN tr|J3QL32|J3QL32\_HUMAN tr|J3KQ22|J3KQ22\_HUMAN tr|J3QKT4|J3QKT4\_HUMAN sp|Q9UJX0|OSGI1\_HUMAN tr|H7C3I2|H7C3I2\_HUMAN tr|Q5W0F9|Q5W0F9\_HUMAN sp|Q9Y5Q9|TF3C3\_HUMAN sp|P47974|TISD\_HUMAN sp|P15428|PGDH\_HUMAN sp|P59535|T2R40\_HUMAN tr|A0A096LP59|A0A096LP59\_HUMAN tr|B7Z2T4|B7Z2T4\_HUMAN sp|Q15700|DLG2\_HUMAN tr|B7Z264|B7Z264\_HUMAN tr|A0A087WYZ5|A0A087WYZ5\_HUMAN sp|Q99679|GPR21\_HUMAN tr|Q5T670|Q5T670\_HUMAN tr|H0YJ04|H0YJ04\_HUMAN tr|C9JWT6|C9JWT6\_HUMAN sp|Q7L590|MCM10\_HUMAN tr|H0Y524|H0Y524\_HUMAN tr|H0YHT0|H0YHT0\_HUMAN sp|Q16526|CRY1\_HUMAN tr|Q68DL0|Q68DL0\_HUMAN tr|H3BTV0|H3BTV0\_HUMAN tr|C9J8Q3|C9J8Q3\_HUMAN tr|Q5T3N0|Q5T3N0\_HUMAN tr|E7EWX8|E7EWX8\_HUMAN tr|H3BTY0|H3BTY0\_HUMAN tr|H3BTR6|H3BTR6\_HUMAN tr|H3BNI3|H3BNI3\_HUMAN tr|H7C599|H7C599\_HUMAN tr|H3BUL0|H3BUL0\_HUMAN tr|H3BUG0|H3BUG0\_HUMAN tr|H3BP82|H3BP82\_HUMAN sp|Q9NZ94|NLGN3\_HUMAN sp|A4D0S4|LAMB4\_HUMAN sp|Q8IY18|SMC5\_HUMAN sp|Q9BXQ6|CECR6\_HUMAN tr|E9PFZ2|E9PFZ2\_HUMAN tr|B5ME60|B5ME60\_HUMAN sp|P61764|STXB1\_HUMAN sp|P00450|CERU\_HUMAN sp|P49069|CAMLG\_HUMAN tr|H0Y8J8|H0Y8J8\_HUMAN sp|Q86YV6|MYLK4\_HUMAN sp|A8K0S8|ME3L2\_HUMAN sp|Q96EK9|KTI12\_HUMAN tr|J3QQZ1|J3QQZ1\_HUMAN tr|E5RFH7|E5RFH7\_HUMAN sp|P35499|SCN4A\_HUMAN sp|Q9UGJ1|GCP4\_HUMAN tr|A0A087WTS3|A0A087WTS3\_HUMAN tr|E5RGP4|E5RGP4\_HUMAN sp|P55199|ELL\_HUMAN tr|A0A087WVB5|A0A087WVB5\_HUMAN sp|Q8NDN9|RCBT1\_HUMAN sp|Q9NQ11|AT132\_HUMAN sp|Q9H8K7|CJ088\_HUMAN sp|Q8N349|OR2LD\_HUMAN tr|M0R1J6|M0R1J6\_HUMAN sp|Q7RTU3|OLIG3\_HUMAN tr|H0YMP8|H0YMP8\_HUMAN sp|Q70SY1|CR3L2\_HUMAN tr|C9JMN2|C9JMN2\_HUMAN sp|O14556|G3PT\_HUMAN sp|Q99612|KLF6\_HUMAN sp|Q5TEJ8|THMS2\_HUMAN sp|Q9GZN2|TGIF2\_HUMAN tr|H0YKN5|H0YKN5\_HUMAN sp|Q8NBV4|PPAC3\_HUMAN sp|Q8TBZ9|CG062\_HUMAN tr|C9J6F5|C9J6F5\_HUMAN sp|Q8NGT7|O2A12\_HUMAN sp|Q9Y6C9|MTCH2\_HUMAN tr|A0A087WZ54|A0A087WZ54\_HUMAN sp|A6NHM9|MOXD2\_HUMAN tr|H0YH31|H0YH31\_HUMAN sp|P43005|EAA3\_HUMAN tr|H0YB78|H0YB78\_HUMAN tr|F8WDW9|F8WDW9\_HUMAN sp|Q9Y237|PIN4\_HUMAN tr|B7Z3A9|B7Z3A9\_HUMAN tr|K9N2S2|K9N2S2\_HUMAN tr|G3V3N5|G3V3N5\_HUMAN tr|J3KR22|J3KR22\_HUMAN tr|F8W050|F8W050\_HUMAN sp|Q8IUN9|CLC10\_HUMAN tr|Q5TB19|Q5TB19\_HUMAN sp|Q9NX31|OSER1\_HUMAN sp|Q6ZV73|FGD6\_HUMAN sp|Q9BTT0|AN32E\_HUMAN tr|K7EMS6|K7EMS6\_HUMAN tr|G3XAG9|G3XAG9\_HUMAN tr|R4GMN4|R4GMN4\_HUMAN sp|Q92901|RL3L\_HUMAN tr|V9GY36|V9GY36\_HUMAN sp|Q6UX65|DRAM2\_HUMAN sp|P43363|MAGAA\_HUMAN sp|Q6MZQ0|PRR5L\_HUMAN tr|E7ES19|E7ES19\_HUMAN tr|E9PIZ1|E9PIZ1\_HUMAN sp|P35443|TSP4\_HUMAN sp|A8MU76|YP034\_HUMAN sp|A8MUA0|YB057\_HUMAN tr|F8VTR6|F8VTR6\_HUMAN tr|H3BNG1|H3BNG1\_HUMAN tr|H0YKG5|H0YKG5\_HUMAN sp|Q6VY07|PACS1\_HUMAN sp|Q8NEP7|KLDC9\_HUMAN tr|H7C005|H7C005\_HUMAN tr|A0A087WYJ8|A0A087WYJ8\_HUMAN sp|Q96M89|CC138\_HUMAN sp|Q96KQ7|EHMT2\_HUMAN tr|A2ABF9|A2ABF9\_HUMAN tr|A2ABF8|A2ABF8\_HUMAN sp|Q9Y646|CBPQ\_HUMAN sp|Q9UBR1|BUP1\_HUMAN tr|H7C2J4|H7C2J4\_HUMAN sp|Q9NQL9|DMRT3\_HUMAN tr|H7C4J3|H7C4J3\_HUMAN sp|O43196|MSH5\_HUMAN tr|J3KTB3|J3KTB3\_HUMAN tr|H0YED8|H0YED8\_HUMAN sp|P20618|PSB1\_HUMAN tr|Q6P6D5|Q6P6D5\_HUMAN sp|Q8NGG7|OR8A1\_HUMAN sp|Q16625|OCLN\_HUMAN sp|P12268|IMDH2\_HUMAN tr|B4E321|B4E321\_HUMAN tr|F8VZI7|F8VZI7\_HUMAN sp|A6NDP7|MADL2\_HUMAN tr|F8W1V2|F8W1V2\_HUMAN tr|F8VVY1|F8VVY1\_HUMAN tr|F8W0R2|F8W0R2\_HUMAN tr|F8W1N0|F8W1N0\_HUMAN tr|F8VUB4|F8VUB4\_HUMAN sp|Q05901|ACHB3\_HUMAN tr|F8VX63|F8VX63\_HUMAN sp|Q14739|LBR\_HUMAN tr|H0YCY7|H0YCY7\_HUMAN sp|P43364|MAGAB\_HUMAN sp|P61574|RE113\_HUMAN tr|E9PP77|E9PP77\_HUMAN sp|Q92484|ASM3A\_HUMAN tr|Q05BZ3|Q05BZ3\_HUMAN sp|Q5EBM0|CMPK2\_HUMAN sp|O95600|KLF8\_HUMAN sp|Q9H175|CSRN2\_HUMAN sp|Q8IYT2|CMTR2\_HUMAN sp|Q8NGR4|OR5C1\_HUMAN tr|H0Y8X7|H0Y8X7\_HUMAN tr|F5H7Y7|F5H7Y7\_HUMAN tr|F8VNW4|F8VNW4\_HUMAN tr|F8W0W4|F8W0W4\_HUMAN tr|F8VZ58|F8VZ58\_HUMAN sp|Q13765|NACA\_HUMAN tr|F5GZR4|F5GZR4\_HUMAN tr|G3V414|G3V414\_HUMAN tr|J3QRX2|J3QRX2\_HUMAN sp|P09919|CSF3\_HUMAN sp|P42566|EPS15\_HUMAN sp|Q9BWX5|GATA5\_HUMAN tr|A0A087WYU2|A0A087WYU2\_HUMAN sp|Q96S19|CP013\_HUMAN tr|F8W0P7|F8W0P7\_HUMAN sp|Q9P242|NYAP2\_HUMAN sp|P27930|IL1R2\_HUMAN tr|I3L3H2|I3L3H2\_HUMAN sp|Q5MIZ7|P4R3B\_HUMAN tr|G5E953|G5E953\_HUMAN sp|P27338|AOFB\_HUMAN tr|G3V5P5|G3V5P5\_HUMAN tr|H0Y9J2|H0Y9J2\_HUMAN sp|P20853|CP2A7\_HUMAN tr|E7EMH4|E7EMH4\_HUMAN tr|E7ER04|E7ER04\_HUMAN tr|B1AHC4|B1AHC4\_HUMAN tr|F5GXP8|F5GXP8\_HUMAN sp|Q8NER5|ACV1C\_HUMAN tr|F5GXJ6|F5GXJ6\_HUMAN tr|Q5SQH5|Q5SQH5\_HUMAN tr|E9PHT3|E9PHT3\_HUMAN sp|O43639|NCK2\_HUMAN sp|P16050|LOX15\_HUMAN sp|P16150|LEUK\_HUMAN sp|Q9NWL6|ASND1\_HUMAN tr|C9IYZ1|C9IYZ1\_HUMAN sp|Q04727|TLE4\_HUMAN sp|Q8IZD6|S22AF\_HUMAN sp|P00390|GSHR\_HUMAN tr|C9JUK7|C9JUK7\_HUMAN tr|E7EMD0|E7EMD0\_HUMAN sp|Q8IVM8|S22A9\_HUMAN sp|Q5VYV0|FOXB2\_HUMAN tr|I3L3W4|I3L3W4\_HUMAN sp|Q8IXT2|DMRTD\_HUMAN sp|P36955|PEDF\_HUMAN tr|H7C5E8|H7C5E8\_HUMAN sp|O43825|B3GT2\_HUMAN sp|Q8NH79|OR6X1\_HUMAN sp|Q96GC6|ZN274\_HUMAN tr|B4DX56|B4DX56\_HUMAN sp|P18859|ATP5J\_HUMAN tr|A8MUH2|A8MUH2\_HUMAN sp|Q8WUM9|S20A1\_HUMAN tr|A2A3Q2|A2A3Q2\_HUMAN sp|O95848|NUD14\_HUMAN sp|Q9HAC7|SUCHY\_HUMAN sp|P18507|GBRG2\_HUMAN tr|B1AQ20|B1AQ20\_HUMAN sp|Q8N448|LNX2\_HUMAN tr|K7EJZ1|K7EJZ1\_HUMAN sp|Q15021|CND1\_HUMAN sp|Q9BZG2|PPAT\_HUMAN sp|Q13618|CUL3\_HUMAN tr|F5GWS2|F5GWS2\_HUMAN tr|A0A087WYI2|A0A087WYI2\_HUMAN tr|A0A087WXG7|A0A087WXG7\_HUMAN tr|E9PPH5|E9PPH5\_HUMAN sp|Q9H2F5|EPC1\_HUMAN tr|H0YLV8|H0YLV8\_HUMAN tr|B3KXJ3|B3KXJ3\_HUMAN tr|J3QRM6|J3QRM6\_HUMAN sp|Q8NGK0|O51G2\_HUMAN tr|J3KT56|J3KT56\_HUMAN tr|J3KQ33|J3KQ33\_HUMAN tr|B4DKA4|B4DKA4\_HUMAN sp|Q9BQR3|PRS27\_HUMAN sp|Q13496|MTM1\_HUMAN sp|O15353|FOXN1\_HUMAN tr|H3BNI0|H3BNI0\_HUMAN sp|P17023|ZNF19\_HUMAN sp|P50213|IDH3A\_HUMAN tr|A2A341|A2A341\_HUMAN sp|O43315|AQP9\_HUMAN tr|H0YL72|H0YL72\_HUMAN sp|A6NFZ4|FA24A\_HUMAN sp|Q96JY6|PDLI2\_HUMAN sp|Q9Y5J1|UTP18\_HUMAN sp|Q86U44|MTA70\_HUMAN tr|J3QRG0|J3QRG0\_HUMAN tr|J3QLD6|J3QLD6\_HUMAN tr|B4DTN4|B4DTN4\_HUMAN sp|P63162|RSMN\_HUMAN tr|J3QLE5|J3QLE5\_HUMAN tr|F5H6D8|F5H6D8\_HUMAN sp|Q9ULW2|FZD10\_HUMAN sp|P13521|SCG2\_HUMAN tr|X6RDF7|X6RDF7\_HUMAN sp|Q9Y694|S22A7\_HUMAN tr|J3QKS7|J3QKS7\_HUMAN tr|V9GZ14|V9GZ14\_HUMAN sp|Q96NI8|ZN570\_HUMAN tr|V9GYG5|V9GYG5\_HUMAN sp|P16190|1A33\_HUMAN tr|F8VTV8|F8VTV8\_HUMAN sp|P16189|1A31\_HUMAN tr|F8VYH9|F8VYH9\_HUMAN tr|E5RHH4|E5RHH4\_HUMAN sp|Q96MU8|KREM1\_HUMAN sp|O14813|PHX2A\_HUMAN sp|P05164|PERM\_HUMAN tr|B8ZZY4|B8ZZY4\_HUMAN sp|Q969R8|ITFG2\_HUMAN sp|Q16658|FSCN1\_HUMAN sp|Q14DG7|T132B\_HUMAN tr|S4R458|S4R458\_HUMAN sp|P82094|TMF1\_HUMAN tr|H7C3Q8|H7C3Q8\_HUMAN tr|F5H0N1|F5H0N1\_HUMAN tr|H0Y6N7|H0Y6N7\_HUMAN sp|P54257|HAP1\_HUMAN sp|Q5T8I3|F102B\_HUMAN sp|Q8IYS0|GRM1C\_HUMAN tr|F5H5B4|F5H5B4\_HUMAN sp|Q01740|FMO1\_HUMAN tr|E7EM78|E7EM78\_HUMAN sp|P34903|GBRA3\_HUMAN tr|B5MDU9|B5MDU9\_HUMAN sp|P05181|CP2E1\_HUMAN sp|Q9UBL0|ARP21\_HUMAN tr|A0A087WWL5|A0A087WWL5\_HUMAN tr|Q5R341|Q5R341\_HUMAN sp|P07093|GDN\_HUMAN tr|Q5R345|Q5R345\_HUMAN sp|Q5T7W7|TSTD2\_HUMAN tr|Q5T7F5|Q5T7F5\_HUMAN tr|H7BY82|H7BY82\_HUMAN tr|C9JAG2|C9JAG2\_HUMAN sp|P15036|ETS2\_HUMAN sp|Q9UEU0|VTI1B\_HUMAN sp|Q7L4S7|ARMX6\_HUMAN tr|E7EUW0|E7EUW0\_HUMAN tr|Q8N6L6|Q8N6L6\_HUMAN sp|Q8NGP6|OR5M8\_HUMAN sp|Q6ZU52|K0408\_HUMAN tr|H0YK69|H0YK69\_HUMAN sp|Q7Z5M5|TMC3\_HUMAN sp|Q9BST9|RTKN\_HUMAN sp|Q96HB5|CC120\_HUMAN sp|Q5IJ48|CRUM2\_HUMAN tr|J3KSP6|J3KSP6\_HUMAN sp|O75170|PP6R2\_HUMAN sp|P12525|MYCP1\_HUMAN tr|T1ECW5|T1ECW5\_HUMAN tr|G3V4R8|G3V4R8\_HUMAN sp|P16473|TSHR\_HUMAN sp|P50148|GNAQ\_HUMAN tr|F8VRI7|F8VRI7\_HUMAN sp|Q6UY18|LIGO4\_HUMAN sp|Q8N5N4|CC022\_HUMAN sp|Q6ZSB9|ZBT49\_HUMAN tr|J3KQ47|J3KQ47\_HUMAN tr|J3KSD7|J3KSD7\_HUMAN tr|C9JJ12|C9JJ12\_HUMAN tr|C9JCQ3|C9JCQ3\_HUMAN sp|Q9HCS5|E41LA\_HUMAN tr|E9PPP2|E9PPP2\_HUMAN sp|Q9HCE5|MET14\_HUMAN tr|A0A087X162|A0A087X162\_HUMAN sp|P08908|5HT1A\_HUMAN tr|H0YIT6|H0YIT6\_HUMAN sp|Q86XJ1|GA2L3\_HUMAN tr|G3V1N3|G3V1N3\_HUMAN tr|E9PM87|E9PM87\_HUMAN tr|F8WJN6|F8WJN6\_HUMAN tr|H0Y8E5|H0Y8E5\_HUMAN sp|Q9H9H5|MA6D1\_HUMAN tr|H0YBH9|H0YBH9\_HUMAN tr|E9PK01|E9PK01\_HUMAN sp|Q8NBS9|TXND5\_HUMAN tr|H0YEH1|H0YEH1\_HUMAN tr|H0YCP6|H0YCP6\_HUMAN tr|G3V1Y8|G3V1Y8\_HUMAN tr|A0A075B6I4|A0A075B6I4\_HUMAN sp|Q8TDD5|MCLN3\_HUMAN tr|H7BYF1|H7BYF1\_HUMAN tr|H7C5A2|H7C5A2\_HUMAN sp|Q9C0C2|TB182\_HUMAN sp|Q8TE56|ATS17\_HUMAN sp|Q96EF0|MTMR8\_HUMAN sp|P0C7V8|DC8L2\_HUMAN sp|P35548|MSX2\_HUMAN sp|Q9H3Y8|PPDPF\_HUMAN tr|H7C555|H7C555\_HUMAN tr|F5H375|F5H375\_HUMAN sp|Q6T423|S22AP\_HUMAN sp|Q8WXD0|RXFP2\_HUMAN tr|V9GYB4|V9GYB4\_HUMAN sp|O96019|ACL6A\_HUMAN sp|Q8N5I2|ARRD1\_HUMAN tr|D6R9Q4|D6R9Q4\_HUMAN sp|Q9UER7|DAXX\_HUMAN tr|F5ANJ6|F5ANJ6\_HUMAN tr|H7C316|H7C316\_HUMAN sp|P08243|ASNS\_HUMAN sp|Q8TDC0|MYOZ3\_HUMAN sp|P47755|CAZA2\_HUMAN sp|Q5GLZ8|HERC4\_HUMAN sp|Q8IXQ6|PARP9\_HUMAN tr|Q5W0W2|Q5W0W2\_HUMAN tr|F6U1F2|F6U1F2\_HUMAN tr|H0YGK4|H0YGK4\_HUMAN tr|F6TC96|F6TC96\_HUMAN sp|Q13261|I15RA\_HUMAN sp|A6NNY8|UBP27\_HUMAN tr|F6RG75|F6RG75\_HUMAN tr|F6WML8|F6WML8\_HUMAN tr|F6XTU0|F6XTU0\_HUMAN sp|Q8IXZ2|ZC3H3\_HUMAN sp|P28070|PSB4\_HUMAN tr|F5GXA4|F5GXA4\_HUMAN tr|Q9BTX6|Q9BTX6\_HUMAN sp|P04053|TDT\_HUMAN tr|G5E9V3|G5E9V3\_HUMAN tr|M4W6S4|M4W6S4\_HUMAN sp|Q8NDC0|MISSL\_HUMAN tr|H7C5Q2|H7C5Q2\_HUMAN sp|Q9BRY0|S39A3\_HUMAN tr|A0A087WSZ2|A0A087WSZ2\_HUMAN tr|F5H385|F5H385\_HUMAN sp|Q96GP6|SREC2\_HUMAN tr|A0A087WVE7|A0A087WVE7\_HUMAN sp|Q9BZS1|FOXP3\_HUMAN sp|Q96T92|INSM2\_HUMAN sp|O15243|OBRG\_HUMAN tr|A0A087X0N2|A0A087X0N2\_HUMAN sp|P47897|SYQ\_HUMAN sp|Q86Y22|CONA1\_HUMAN sp|P30550|GRPR\_HUMAN tr|M0QYS8|M0QYS8\_HUMAN tr|K7EQM7|K7EQM7\_HUMAN sp|Q9UNP4|SIAT9\_HUMAN tr|F8VZI9|F8VZI9\_HUMAN tr|E9PLV3|E9PLV3\_HUMAN tr|H0Y935|H0Y935\_HUMAN tr|D6RCP5|D6RCP5\_HUMAN sp|Q8N264|RHG24\_HUMAN sp|Q8WW24|TEKT4\_HUMAN sp|Q9Y2K7|KDM2A\_HUMAN tr|H3BU64|H3BU64\_HUMAN tr|F5GXE4|F5GXE4\_HUMAN tr|B4DFB6|B4DFB6\_HUMAN tr|H7C3W3|H7C3W3\_HUMAN sp|Q3SXZ3|ZN718\_HUMAN sp|Q9NPD7|NRN1\_HUMAN sp|O60716|CTND1\_HUMAN tr|H0YAZ4|H0YAZ4\_HUMAN sp|Q9UJM8|HAOX1\_HUMAN sp|Q969F2|NKD2\_HUMAN sp|Q2M243|CCD27\_HUMAN sp|Q9BXC9|BBS2\_HUMAN tr|H7C010|H7C010\_HUMAN tr|H0YCJ8|H0YCJ8\_HUMAN tr|F5H6T4|F5H6T4\_HUMAN tr|C9JIF9|C9JIF9\_HUMAN sp|P26010|ITB7\_HUMAN tr|H7BZS1|H7BZS1\_HUMAN sp|P13798|ACPH\_HUMAN sp|O75900|MMP23\_HUMAN tr|D6R918|D6R918\_HUMAN tr|H0YAA9|H0YAA9\_HUMAN tr|D6RDI5|D6RDI5\_HUMAN tr|D6RA54|D6RA54\_HUMAN tr|D6RC55|D6RC55\_HUMAN tr|D6RBC5|D6RBC5\_HUMAN tr|Q4VXB0|Q4VXB0\_HUMAN sp|Q99755|PI51A\_HUMAN sp|Q9ULL4|PLXB3\_HUMAN tr|A0A096LPC9|A0A096LPC9\_HUMAN sp|P56178|DLX5\_HUMAN sp|Q86XG9|NBPF5\_HUMAN tr|A0A087WZJ0|A0A087WZJ0\_HUMAN tr|E5RGG2|E5RGG2\_HUMAN sp|Q8N4E4|PDCL2\_HUMAN sp|Q5HYM0|ZC12B\_HUMAN tr|E9PEG3|E9PEG3\_HUMAN tr|A0A087WZ66|A0A087WZ66\_HUMAN sp|Q14894|CRYM\_HUMAN tr|F8W9X7|F8W9X7\_HUMAN sp|Q4VC44|FWCH1\_HUMAN tr|A0A087X072|A0A087X072\_HUMAN tr|F8W7D1|F8W7D1\_HUMAN tr|I3L325|I3L325\_HUMAN tr|E9PB77|E9PB77\_HUMAN sp|Q567U6|CCD93\_HUMAN tr|A2AAS7|A2AAS7\_HUMAN sp|Q8NE00|TM104\_HUMAN sp|Q14249|NUCG\_HUMAN sp|P49593|PPM1F\_HUMAN sp|Q5VWG9|TAF3\_HUMAN tr|R4GNJ5|R4GNJ5\_HUMAN tr|E5RK82|E5RK82\_HUMAN tr|H0YBL5|H0YBL5\_HUMAN tr|E5RH67|E5RH67\_HUMAN sp|A6NFQ2|F115C\_HUMAN tr|H0YC74|H0YC74\_HUMAN tr|E9PR03|E9PR03\_HUMAN tr|H0YNA8|H0YNA8\_HUMAN sp|Q86UY8|NT5D3\_HUMAN tr|F5GXE6|F5GXE6\_HUMAN sp|P21781|FGF7\_HUMAN tr|H7BYB7|H7BYB7\_HUMAN tr|A0A087X1V8|A0A087X1V8\_HUMAN tr|E9PMA4|E9PMA4\_HUMAN tr|G3V286|G3V286\_HUMAN sp|P25963|IKBA\_HUMAN tr|G3V3I4|G3V3I4\_HUMAN sp|Q9NPI6|DCP1A\_HUMAN tr|H0Y922|H0Y922\_HUMAN sp|O75888|TNF13\_HUMAN tr|C9JFN2|C9JFN2\_HUMAN tr|C9JF68|C9JF68\_HUMAN sp|Q2KHM9|K0753\_HUMAN tr|A0A087WUP4|A0A087WUP4\_HUMAN tr|E7EQL6|E7EQL6\_HUMAN sp|Q8IXS8|F126B\_HUMAN tr|B9ZVT1|B9ZVT1\_HUMAN sp|Q8WVM7|STAG1\_HUMAN sp|Q8IXT5|RB12B\_HUMAN sp|Q99453|PHX2B\_HUMAN sp|P47712|PA24A\_HUMAN sp|Q9BY07|S4A5\_HUMAN sp|O95835|LATS1\_HUMAN sp|Q96NU0|CNT3B\_HUMAN tr|H0YA02|H0YA02\_HUMAN sp|O15066|KIF3B\_HUMAN sp|P61573|REC9\_HUMAN sp|P61579|ERK25\_HUMAN tr|H0Y9U4|H0Y9U4\_HUMAN sp|P10632|CP2C8\_HUMAN sp|P61572|REC19\_HUMAN tr|B7Z1F5|B7Z1F5\_HUMAN sp|Q14774|HLX\_HUMAN sp|Q9H6L5|F134B\_HUMAN sp|Q12872|SFSWA\_HUMAN sp|Q69383|REC6\_HUMAN tr|B8ZZD3|B8ZZD3\_HUMAN tr|D6RDB0|D6RDB0\_HUMAN sp|P02708|ACHA\_HUMAN tr|D6RHE3|D6RHE3\_HUMAN tr|Q5JV20|Q5JV20\_HUMAN sp|Q14929|ZN169\_HUMAN sp|Q52WX2|SBK1\_HUMAN tr|C9J1W9|C9J1W9\_HUMAN sp|O15427|MOT4\_HUMAN tr|C9JXM5|C9JXM5\_HUMAN tr|C9JF75|C9JF75\_HUMAN tr|E5RK45|E5RK45\_HUMAN sp|Q13639|5HT4R\_HUMAN tr|F8W0A3|F8W0A3\_HUMAN sp|Q9HCE1|MOV10\_HUMAN tr|Q5JR04|Q5JR04\_HUMAN sp|Q6STE5|SMRD3\_HUMAN sp|Q6ZV50|RFX8\_HUMAN tr|C9JRK1|C9JRK1\_HUMAN sp|Q9NZI6|TF2L1\_HUMAN tr|H7C164|H7C164\_HUMAN sp|Q3ZCM7|TBB8\_HUMAN tr|A0A075B736|A0A075B736\_HUMAN sp|A6NNZ2|TBB8L\_HUMAN sp|Q5PR19|YI024\_HUMAN tr|Q3SY61|Q3SY61\_HUMAN tr|E9PM17|E9PM17\_HUMAN tr|H7C1L3|H7C1L3\_HUMAN tr|H0Y6A7|H0Y6A7\_HUMAN sp|Q9C035|TRIM5\_HUMAN tr|Q5TH61|Q5TH61\_HUMAN tr|A0A087X1E2|A0A087X1E2\_HUMAN sp|P30837|AL1B1\_HUMAN sp|P17213|BPI\_HUMAN sp|Q9H063|MAF1\_HUMAN tr|E9PR76|E9PR76\_HUMAN tr|E9PSH4|E9PSH4\_HUMAN sp|Q0JRZ9|FCHO2\_HUMAN sp|Q9BVG9|PTSS2\_HUMAN sp|Q7L2J0|MEPCE\_HUMAN sp|Q8TAM2|TTC8\_HUMAN sp|P08686|CP21A\_HUMAN sp|Q5T124|UBX11\_HUMAN sp|O43414|ERI3\_HUMAN sp|P59817|Z280A\_HUMAN tr|B5MBX1|B5MBX1\_HUMAN tr|F8VW56|F8VW56\_HUMAN sp|Q9P2P5|HECW2\_HUMAN tr|H7C4W8|H7C4W8\_HUMAN sp|Q8NGK9|OR5DG\_HUMAN sp|Q5W188|CST9P\_HUMAN sp|Q32P51|RA1L2\_HUMAN sp|Q66LE6|2ABD\_HUMAN sp|Q92729|PTPRU\_HUMAN sp|Q9Y4E5|ZN451\_HUMAN tr|E9PH99|E9PH99\_HUMAN sp|Q8NGS3|OR1J1\_HUMAN tr|H3BQL0|H3BQL0\_HUMAN tr|H3BV82|H3BV82\_HUMAN tr|H3BP99|H3BP99\_HUMAN sp|Q8TBB5|KLDC4\_HUMAN tr|F8WES3|F8WES3\_HUMAN tr|J3KNW1|J3KNW1\_HUMAN tr|J3KN78|J3KN78\_HUMAN sp|Q7Z444|RASE\_HUMAN tr|C9JVJ9|C9JVJ9\_HUMAN tr|C9JVX5|C9JVX5\_HUMAN tr|F8WBR2|F8WBR2\_HUMAN tr|F8W837|F8W837\_HUMAN sp|P19823|ITIH2\_HUMAN sp|Q9UHV2|SRTD1\_HUMAN tr|A0A087WTE1|A0A087WTE1\_HUMAN tr|H0YCZ8|H0YCZ8\_HUMAN sp|P0C264|SBK3\_HUMAN tr|C9JQD1|C9JQD1\_HUMAN tr|E9PIR9|E9PIR9\_HUMAN sp|Q9NXB9|ELOV2\_HUMAN tr|H0YD64|H0YD64\_HUMAN tr|Q5H9P2|Q5H9P2\_HUMAN tr|H0YD47|H0YD47\_HUMAN sp|Q15915|ZIC1\_HUMAN sp|Q6ZS92|YD022\_HUMAN sp|Q6P5X7|TMM71\_HUMAN tr|D3YTC4|D3YTC4\_HUMAN sp|P30613|KPYR\_HUMAN sp|O14672|ADA10\_HUMAN tr|E7EQF3|E7EQF3\_HUMAN tr|E9PH25|E9PH25\_HUMAN sp|P06028|GLPB\_HUMAN sp|Q8NH04|O2T27\_HUMAN tr|E7ERJ5|E7ERJ5\_HUMAN sp|P0C7T2|OR2T7\_HUMAN sp|Q9UDV7|ZN282\_HUMAN tr|H7C0E4|H7C0E4\_HUMAN sp|Q96S42|NODAL\_HUMAN tr|D6RB32|D6RB32\_HUMAN sp|Q6P4A8|PLBL1\_HUMAN tr|K7EJA3|K7EJA3\_HUMAN tr|K7EIM4|K7EIM4\_HUMAN sp|Q96MH2|HEXI2\_HUMAN tr|K7ERG7|K7ERG7\_HUMAN tr|K7ESM2|K7ESM2\_HUMAN tr|K7ELS4|K7ELS4\_HUMAN tr|C9JZI7|C9JZI7\_HUMAN sp|Q96HY7|DHTK1\_HUMAN tr|E7EQN8|E7EQN8\_HUMAN tr|A0A087WY36|A0A087WY36\_HUMAN sp|Q14542|S29A2\_HUMAN tr|C9JWH2|C9JWH2\_HUMAN sp|Q9H1I8|ASCC2\_HUMAN P15497 tr|F8WC65|F8WC65\_HUMAN tr|H7C021|H7C021\_HUMAN sp|Q96M60|F227B\_HUMAN sp|Q9UPT5|EXOC7\_HUMAN sp|O00629|IMA3\_HUMAN tr|B4DJ07|B4DJ07\_HUMAN tr|B7WPR2|B7WPR2\_HUMAN tr|F8W6G1|F8W6G1\_HUMAN sp|Q9UHY1|NRBP\_HUMAN sp|P57727|TMPS3\_HUMAN sp|A6NK02|TRI75\_HUMAN tr|H7C2U6|H7C2U6\_HUMAN sp|Q96BD5|PF21A\_HUMAN tr|A0A096LNL2|A0A096LNL2\_HUMAN sp|O60307|MAST3\_HUMAN sp|Q13349|ITAD\_HUMAN sp|Q8IWV2|CNTN4\_HUMAN sp|Q9NR23|GDF3\_HUMAN sp|P25116|PAR1\_HUMAN sp|Q96QI5|HS3S6\_HUMAN tr|C9JYY8|C9JYY8\_HUMAN sp|Q04725|TLE2\_HUMAN tr|K7EMK7|K7EMK7\_HUMAN tr|J3KR40|J3KR40\_HUMAN sp|Q8IUM7|NPAS4\_HUMAN sp|Q8IWU4|ZNT8\_HUMAN sp|O95447|LCA5L\_HUMAN sp|Q9BZJ8|GPR61\_HUMAN tr|G3V220|G3V220\_HUMAN tr|Q5JZ08|Q5JZ08\_HUMAN sp|O14776|TCRG1\_HUMAN Q3MHH8 sp|Q2TBE0|C19L2\_HUMAN tr|H7C3G7|H7C3G7\_HUMAN tr|B7Z4D4|B7Z4D4\_HUMAN sp|Q9HBW0|LPAR2\_HUMAN tr|O76104|O76104\_HUMAN sp|O95793|STAU1\_HUMAN sp|Q8WZA6|OR1E3\_HUMAN tr|A0A087X1A5|A0A087X1A5\_HUMAN tr|Q5JW30|Q5JW30\_HUMAN tr|H3BNW8|H3BNW8\_HUMAN sp|Q7Z402|TMC7\_HUMAN tr|B5MCV5|B5MCV5\_HUMAN sp|O75688|PPM1B\_HUMAN sp|O14512|SOCS7\_HUMAN tr|A0A087X1Q5|A0A087X1Q5\_HUMAN sp|Q9Y5F3|PCDB1\_HUMAN tr|A0A087WZ14|A0A087WZ14\_HUMAN tr|A0A075B6T1|A0A075B6T1\_HUMAN tr|A1E5M1|A1E5M1\_HUMAN tr|E9PF63|E9PF63\_HUMAN tr|M0R002|M0R002\_HUMAN tr|H7C523|H7C523\_HUMAN tr|M0QX15|M0QX15\_HUMAN sp|Q8NAG6|ANKL1\_HUMAN tr|M0R1H8|M0R1H8\_HUMAN sp|Q8TF32|ZN431\_HUMAN tr|H3BUU5|H3BUU5\_HUMAN tr|K7EP20|K7EP20\_HUMAN tr|K7EP91|K7EP91\_HUMAN tr|E9PLZ3|E9PLZ3\_HUMAN tr|E9PIM9|E9PIM9\_HUMAN tr|E9PMJ3|E9PMJ3\_HUMAN sp|Q96GK7|FAH2A\_HUMAN tr|H0YJX4|H0YJX4\_HUMAN sp|Q6P2I3|FAH2B\_HUMAN tr|E9PIK5|E9PIK5\_HUMAN sp|Q9BUY7|EFC11\_HUMAN sp|P13489|RINI\_HUMAN tr|F5H5Z1|F5H5Z1\_HUMAN tr|F5H510|F5H510\_HUMAN tr|H0Y941|H0Y941\_HUMAN sp|Q8N394|TMTC2\_HUMAN tr|F8VSH2|F8VSH2\_HUMAN sp|O00482|NR5A2\_HUMAN tr|F5H2E5|F5H2E5\_HUMAN tr|U3KQM1|U3KQM1\_HUMAN tr|F5H595|F5H595\_HUMAN sp|Q9Y6K5|OAS3\_HUMAN sp|Q9C040|TRIM2\_HUMAN tr|I3L168|I3L168\_HUMAN tr|I3L3Y6|I3L3Y6\_HUMAN tr|I3L204|I3L204\_HUMAN tr|I3L4D4|I3L4D4\_HUMAN sp|Q99832|TCPH\_HUMAN tr|I3L501|I3L501\_HUMAN sp|Q6ZR37|PKHG7\_HUMAN tr|I3L3F5|I3L3F5\_HUMAN sp|Q99643|C560\_HUMAN sp|Q5XXA6|ANO1\_HUMAN sp|Q9UQM7|KCC2A\_HUMAN sp|Q5K4L6|S27A3\_HUMAN tr|A0A087X115|A0A087X115\_HUMAN tr|X6R3N0|X6R3N0\_HUMAN sp|Q49A17|GLTL6\_HUMAN tr|H7BZH4|H7BZH4\_HUMAN tr|E7EPJ9|E7EPJ9\_HUMAN sp|P05120|PAI2\_HUMAN tr|E7ERB5|E7ERB5\_HUMAN sp|Q6ISU1|PTCRA\_HUMAN tr|H7C4E6|H7C4E6\_HUMAN tr|A0A087WTE9|A0A087WTE9\_HUMAN sp|Q2T9L4|CO059\_HUMAN sp|Q9P1Q0|VPS54\_HUMAN tr|F8VVU2|F8VVU2\_HUMAN sp|Q9C0I1|MTMRC\_HUMAN tr|C9J9T8|C9J9T8\_HUMAN sp|Q9UBV8|PEF1\_HUMAN sp|Q7L5N7|PCAT2\_HUMAN sp|O75110|ATP9A\_HUMAN tr|D6RF93|D6RF93\_HUMAN sp|Q9HCS7|SYF1\_HUMAN tr|A0A087WV26|A0A087WV26\_HUMAN tr|H9KVD4|H9KVD4\_HUMAN sp|P49642|PRI1\_HUMAN tr|E7EQ61|E7EQ61\_HUMAN sp|Q9GZZ9|UBA5\_HUMAN tr|E7EWE1|E7EWE1\_HUMAN sp|Q9BSK2|S2533\_HUMAN tr|H3BT25|H3BT25\_HUMAN tr|H3BQ34|H3BQ34\_HUMAN sp|Q8IWF9|CCD83\_HUMAN sp|Q9UBH6|XPR1\_HUMAN tr|H3BUW1|H3BUW1\_HUMAN tr|H0YAZ5|H0YAZ5\_HUMAN tr|H3BTJ2|H3BTJ2\_HUMAN tr|H0YNC7|H0YNC7\_HUMAN A2I7N0 tr|A0A087WV02|A0A087WV02\_HUMAN tr|A0A087WVP9|A0A087WVP9\_HUMAN sp|Q8TAF7|ZN461\_HUMAN tr|B4DRP8|B4DRP8\_HUMAN tr|E7EUE0|E7EUE0\_HUMAN sp|Q9Y5W8|SNX13\_HUMAN tr|A0A087WUZ7|A0A087WUZ7\_HUMAN sp|P12272|PTHR\_HUMAN tr|F5GZD9|F5GZD9\_HUMAN sp|Q96NR2|C2AS1\_HUMAN sp|Q9NZV1|CRIM1\_HUMAN tr|A0A075B6J7|A0A075B6J7\_HUMAN sp|Q7Z3Z4|PIWL4\_HUMAN tr|H0YET3|H0YET3\_HUMAN sp|Q9NYJ8|TAB2\_HUMAN sp|Q9C0C6|CIPC\_HUMAN sp|Q9NQS7|INCE\_HUMAN tr|H0YHX9|H0YHX9\_HUMAN sp|Q92551|IP6K1\_HUMAN sp|B1AK53|ESPN\_HUMAN tr|C9J453|C9J453\_HUMAN sp|O15344|TRI18\_HUMAN tr|A0A087X255|A0A087X255\_HUMAN tr|E7EPW7|E7EPW7\_HUMAN sp|Q9HB31|SEBOX\_HUMAN tr|A0A087X214|A0A087X214\_HUMAN tr|F8W9A4|F8W9A4\_HUMAN tr|F6T8T6|F6T8T6\_HUMAN sp|Q9UL49|TCFL5\_HUMAN sp|Q8N6M6|AMPO\_HUMAN tr|H7C012|H7C012\_HUMAN tr|H7C463|H7C463\_HUMAN tr|G3V4G1|G3V4G1\_HUMAN sp|Q9Y5R6|DMRT1\_HUMAN tr|H7BZC6|H7BZC6\_HUMAN sp|Q13939|CALI\_HUMAN tr|K7EP56|K7EP56\_HUMAN tr|D6RCF8|D6RCF8\_HUMAN tr|K7ELR0|K7ELR0\_HUMAN sp|Q9BRS2|RIOK1\_HUMAN sp|Q96NL8|CH037\_HUMAN tr|A0A088AWQ4|A0A088AWQ4\_HUMAN tr|Q5T9Q5|Q5T9Q5\_HUMAN tr|B1AKE7|B1AKE7\_HUMAN tr|K7EKM0|K7EKM0\_HUMAN tr|B1AKE6|B1AKE6\_HUMAN tr|D6RBY8|D6RBY8\_HUMAN tr|K7ESA1|K7ESA1\_HUMAN tr|I6L893|I6L893\_HUMAN sp|Q8TDN2|KCNV2\_HUMAN sp|Q9H339|O51B5\_HUMAN sp|Q9UL15|BAG5\_HUMAN sp|P41226|UBA7\_HUMAN sp|O15370|SOX12\_HUMAN tr|G3V2A0|G3V2A0\_HUMAN sp|P51843|NR0B1\_HUMAN tr|H7C835|H7C835\_HUMAN sp|O60832|DKC1\_HUMAN tr|Q5W0W3|Q5W0W3\_HUMAN tr|F8VXU8|F8VXU8\_HUMAN tr|H0YKR2|H0YKR2\_HUMAN tr|F8VTQ9|F8VTQ9\_HUMAN sp|Q7Z4M0|RE114\_HUMAN tr|H0YA21|H0YA21\_HUMAN tr|H0YCG3|H0YCG3\_HUMAN tr|D6RBV3|D6RBV3\_HUMAN sp|Q9H343|O51I1\_HUMAN tr|G3V1X1|G3V1X1\_HUMAN tr|D6RC01|D6RC01\_HUMAN sp|P13727|PRG2\_HUMAN tr|H3BQD9|H3BQD9\_HUMAN sp|Q5VZF2|MBNL2\_HUMAN sp|A6NMN3|F170B\_HUMAN tr|O95205|O95205\_HUMAN tr|K7ESM7|K7ESM7\_HUMAN tr|F8WFC4|F8WFC4\_HUMAN tr|A0A087WVT8|A0A087WVT8\_HUMAN sp|Q8N3L3|TXLNB\_HUMAN sp|O75698|HUG1\_HUMAN sp|Q8NFJ8|BHE22\_HUMAN tr|F8VU90|F8VU90\_HUMAN sp|P21583|SCF\_HUMAN sp|P06400|RB\_HUMAN tr|Q5JWQ6|Q5JWQ6\_HUMAN sp|P16885|PLCG2\_HUMAN sp|Q8NGL3|OR5DE\_HUMAN tr|F6PQP6|F6PQP6\_HUMAN tr|E9PBC1|E9PBC1\_HUMAN sp|Q8N987|NECA1\_HUMAN tr|G3V150|G3V150\_HUMAN sp|O94766|B3GA3\_HUMAN sp|P01781|HV320\_HUMAN tr|E9PNA1|E9PNA1\_HUMAN sp|Q9N2J8|ENH3\_HUMAN sp|Q96PZ0|PUS7\_HUMAN tr|E7EUH7|E7EUH7\_HUMAN tr|H0YEX1|H0YEX1\_HUMAN tr|H0Y980|H0Y980\_HUMAN sp|P38432|COIL\_HUMAN tr|H7C2V2|H7C2V2\_HUMAN tr|H7C2Z5|H7C2Z5\_HUMAN sp|O14746|TERT\_HUMAN tr|E7ES33|E7ES33\_HUMAN tr|H0Y6R4|H0Y6R4\_HUMAN tr|H0Y3Y4|H0Y3Y4\_HUMAN sp|Q16181|SEPT7\_HUMAN sp|Q92935|EXTL1\_HUMAN tr|Q05BE5|Q05BE5\_HUMAN tr|H3BNC0|H3BNC0\_HUMAN sp|Q7Z7F0|K0907\_HUMAN tr|V9GZ64|V9GZ64\_HUMAN sp|Q9BX10|GTPB2\_HUMAN tr|H0YNF4|H0YNF4\_HUMAN tr|H0YK56|H0YK56\_HUMAN tr|J3KSM7|J3KSM7\_HUMAN sp|P10643|CO7\_HUMAN sp|O14490|DLGP1\_HUMAN sp|P13164|IFM1\_HUMAN tr|K7ENJ1|K7ENJ1\_HUMAN tr|H0YHN9|H0YHN9\_HUMAN sp|P35052|GPC1\_HUMAN tr|Q5STU3|Q5STU3\_HUMAN tr|K7EQS6|K7EQS6\_HUMAN tr|H7BZL4|H7BZL4\_HUMAN sp|Q6TDP4|KLH17\_HUMAN sp|Q6ZSJ9|SHSA6\_HUMAN sp|P63133|POK8\_HUMAN tr|H0Y570|H0Y570\_HUMAN tr|Q9HB45|Q9HB45\_HUMAN sp|Q5TAG4|NBPFC\_HUMAN tr|B5MCH3|B5MCH3\_HUMAN sp|Q8N3C7|CLIP4\_HUMAN tr|H0YB35|H0YB35\_HUMAN tr|C9K0C0|C9K0C0\_HUMAN tr|K7EMZ9|K7EMZ9\_HUMAN sp|Q6IBW4|CNDH2\_HUMAN sp|Q6ZUK4|TMM26\_HUMAN tr|H3BMV3|H3BMV3\_HUMAN tr|F8WDQ2|F8WDQ2\_HUMAN sp|Q13621|S12A1\_HUMAN tr|J3QL99|J3QL99\_HUMAN tr|H0YLJ2|H0YLJ2\_HUMAN tr|J3QSB6|J3QSB6\_HUMAN tr|M0QYC5|M0QYC5\_HUMAN tr|D6RBL5|D6RBL5\_HUMAN tr|D6RBE9|D6RBE9\_HUMAN sp|P08758|ANXA5\_HUMAN sp|Q15928|ZN141\_HUMAN sp|O14678|ABCD4\_HUMAN tr|A2T115|A2T115\_HUMAN tr|H0Y8G7|H0Y8G7\_HUMAN tr|A0A087WWJ9|A0A087WWJ9\_HUMAN tr|A0A087WZ78|A0A087WZ78\_HUMAN tr|G3V2M4|G3V2M4\_HUMAN tr|G3V2R2|G3V2R2\_HUMAN sp|Q9P055|JKAMP\_HUMAN tr|H3BQI2|H3BQI2\_HUMAN tr|F5H858|F5H858\_HUMAN tr|C9JFZ1|C9JFZ1\_HUMAN tr|H0YFB5|H0YFB5\_HUMAN sp|P16260|GDC\_HUMAN tr|E9PLK2|E9PLK2\_HUMAN sp|Q96QZ0|PANX3\_HUMAN sp|Q9NYK6|EURL\_HUMAN tr|E7ETB0|E7ETB0\_HUMAN tr|H7BXN7|H7BXN7\_HUMAN tr|H0Y2Y1|H0Y2Y1\_HUMAN tr|H0Y4Y6|H0Y4Y6\_HUMAN sp|P51522|ZNF83\_HUMAN sp|Q8TB69|ZN519\_HUMAN tr|R4GN32|R4GN32\_HUMAN sp|Q8IUL8|CILP2\_HUMAN tr|K7EPJ4|K7EPJ4\_HUMAN sp|Q8WUP2|FBLI1\_HUMAN tr|C9J5P6|C9J5P6\_HUMAN tr|B5MD47|B5MD47\_HUMAN sp|Q8IXL6|DMP4\_HUMAN tr|D3DWI5|D3DWI5\_HUMAN tr|F8WBR3|F8WBR3\_HUMAN sp|Q96HE7|ERO1A\_HUMAN sp|Q96NI6|LRFN5\_HUMAN sp|P02786|TFR1\_HUMAN tr|Q5T0G7|Q5T0G7\_HUMAN sp|P0CZ25|D10OS\_HUMAN tr|G3V4N1|G3V4N1\_HUMAN tr|B1AN91|B1AN91\_HUMAN tr|G3V364|G3V364\_HUMAN tr|Q5T0G9|Q5T0G9\_HUMAN sp|Q6P4E1|CASC4\_HUMAN tr|H3BRL3|H3BRL3\_HUMAN sp|O14562|UBFD1\_HUMAN tr|H0YMR0|H0YMR0\_HUMAN sp|Q9UI14|PRAF1\_HUMAN tr|M0R1H9|M0R1H9\_HUMAN sp|Q5T2W1|NHRF3\_HUMAN sp|P31644|GBRA5\_HUMAN tr|M0R3D4|M0R3D4\_HUMAN sp|P42568|AF9\_HUMAN tr|I3L3U7|I3L3U7\_HUMAN tr|A0A096LNI7|A0A096LNI7\_HUMAN tr|C9JD20|C9JD20\_HUMAN sp|Q504Y2|PKDCC\_HUMAN sp|P63104|1433Z\_HUMAN tr|H7C1K5|H7C1K5\_HUMAN tr|E7EX29|E7EX29\_HUMAN tr|E7ESK7|E7ESK7\_HUMAN tr|H0YDJ8|H0YDJ8\_HUMAN tr|B4DLN1|B4DLN1\_HUMAN tr|F8WCP5|F8WCP5\_HUMAN sp|A2RU49|HYKK\_HUMAN tr|F5H7W1|F5H7W1\_HUMAN sp|Q5H9S7|DCA17\_HUMAN tr|J3KQB2|J3KQB2\_HUMAN sp|Q9Y4R7|TTLL3\_HUMAN tr|H3BQX7|H3BQX7\_HUMAN tr|M0QYZ9|M0QYZ9\_HUMAN tr|E9PM06|E9PM06\_HUMAN sp|P35790|CHKA\_HUMAN tr|F5H422|F5H422\_HUMAN sp|P08519|APOA\_HUMAN sp|O15062|ZBTB5\_HUMAN tr|F8WD86|F8WD86\_HUMAN tr|E7EW73|E7EW73\_HUMAN sp|Q6GV28|TM225\_HUMAN sp|P26022|PTX3\_HUMAN tr|E9PLT9|E9PLT9\_HUMAN sp|Q8TD90|MAGE2\_HUMAN sp|O75387|LAT3\_HUMAN tr|A8MTF7|A8MTF7\_HUMAN sp|Q9UQF0|SYCY1\_HUMAN tr|B4DTL1|B4DTL1\_HUMAN tr|K7ERY2|K7ERY2\_HUMAN tr|K7EPQ7|K7EPQ7\_HUMAN tr|H0YF29|H0YF29\_HUMAN tr|K7EJQ6|K7EJQ6\_HUMAN sp|Q5SNV9|CA167\_HUMAN tr|E9PBF9|E9PBF9\_HUMAN tr|C9JCI8|C9JCI8\_HUMAN tr|G3V4B8|G3V4B8\_HUMAN tr|E9PEK8|E9PEK8\_HUMAN sp|P15336|ATF2\_HUMAN sp|Q9NVA1|UQCC1\_HUMAN tr|M0QZM1|M0QZM1\_HUMAN sp|P52272|HNRPM\_HUMAN tr|B1AKV3|B1AKV3\_HUMAN sp|Q15645|PCH2\_HUMAN sp|Q2TAM9|TUSC1\_HUMAN tr|A0A075B763|A0A075B763\_HUMAN sp|Q86T20|CF001\_HUMAN tr|H0YMB3|H0YMB3\_HUMAN tr|A1L2Z2|A1L2Z2\_HUMAN sp|Q8IZN3|ZDH14\_HUMAN tr|X6RFN3|X6RFN3\_HUMAN sp|Q99496|RING2\_HUMAN sp|Q17RD7|SYT16\_HUMAN sp|Q8N6Q3|CD177\_HUMAN tr|H0YJT7|H0YJT7\_HUMAN tr|C9JHU5|C9JHU5\_HUMAN tr|A0A087WVM2|A0A087WVM2\_HUMAN sp|Q9NQC7|CYLD\_HUMAN sp|Q5T7N2|LITD1\_HUMAN sp|Q96PX1|RN157\_HUMAN tr|J3KRR7|J3KRR7\_HUMAN tr|H0YM94|H0YM94\_HUMAN sp|Q674X7|KAZRN\_HUMAN tr|K7ELV3|K7ELV3\_HUMAN tr|B4DSP5|B4DSP5\_HUMAN sp|Q00973|B4GN1\_HUMAN sp|P10244|MYBB\_HUMAN tr|F8VU35|F8VU35\_HUMAN sp|O43869|OR2T1\_HUMAN sp|O15287|FANCG\_HUMAN tr|H7C4E4|H7C4E4\_HUMAN tr|E9PH58|E9PH58\_HUMAN sp|P00156|CYB\_HUMAN tr|J3KPG0|J3KPG0\_HUMAN tr|J3KT50|J3KT50\_HUMAN tr|J3KT61|J3KT61\_HUMAN tr|C9JUK9|C9JUK9\_HUMAN tr|S4R3I1|S4R3I1\_HUMAN sp|O43316|PAX4\_HUMAN sp|Q8N5D6|GBGT1\_HUMAN sp|Q9H0Z9|RBM38\_HUMAN tr|E9PRI8|E9PRI8\_HUMAN tr|H0YMZ3|H0YMZ3\_HUMAN tr|F8W8D4|F8W8D4\_HUMAN sp|P11117|PPAL\_HUMAN sp|Q9GZK4|OR2H1\_HUMAN tr|A6NHH0|A6NHH0\_HUMAN sp|Q8TBC4|UBA3\_HUMAN sp|Q9UNH6|SNX7\_HUMAN tr|B4DJV5|B4DJV5\_HUMAN sp|Q13610|PWP1\_HUMAN sp|P22102|PUR2\_HUMAN tr|H7C4S7|H7C4S7\_HUMAN sp|P29122|PCSK6\_HUMAN tr|H3BNV4|H3BNV4\_HUMAN sp|Q9HDB9|GAK5\_HUMAN tr|C9J3R8|C9J3R8\_HUMAN sp|Q8NGE8|OR4D9\_HUMAN sp|Q9H4I0|RD21L\_HUMAN sp|O60513|B4GT4\_HUMAN tr|F8WBF5|F8WBF5\_HUMAN tr|Q5QP40|Q5QP40\_HUMAN sp|Q96JN0|LCOR\_HUMAN sp|Q8TCT7|SPP2B\_HUMAN tr|A0A096LNZ5|A0A096LNZ5\_HUMAN tr|D6RDN4|D6RDN4\_HUMAN tr|A0A096LNP5|A0A096LNP5\_HUMAN tr|C8C3P2|C8C3P2\_HUMAN sp|P0CG32|ZCC18\_HUMAN tr|E9PDV3|E9PDV3\_HUMAN sp|Q8NGW1|OR6B3\_HUMAN tr|H0YC98|H0YC98\_HUMAN sp|Q9UIF3|TEKT2\_HUMAN sp|Q5VYS8|TUT7\_HUMAN sp|P35754|GLRX1\_HUMAN sp|Q9NS39|RED2\_HUMAN sp|Q8NFH8|REPS2\_HUMAN tr|C9JZ52|C9JZ52\_HUMAN sp|Q8N3E9|PLCD3\_HUMAN tr|A0A087WZQ8|A0A087WZQ8\_HUMAN tr|E9PCP0|E9PCP0\_HUMAN tr|F8WBF8|F8WBF8\_HUMAN tr|F8VUJ3|F8VUJ3\_HUMAN sp|P16520|GBB3\_HUMAN tr|B4DRN8|B4DRN8\_HUMAN tr|J3KQD3|J3KQD3\_HUMAN sp|Q5W0Z9|ZDH20\_HUMAN sp|Q8NGC0|O5AU1\_HUMAN sp|P29965|CD40L\_HUMAN tr|Q3L8U2|Q3L8U2\_HUMAN sp|Q8IZ73|RUSD2\_HUMAN sp|P28039|AOAH\_HUMAN sp|P27987|IP3KB\_HUMAN sp|Q9NRA1|PDGFC\_HUMAN tr|H0YES3|H0YES3\_HUMAN sp|Q6ZVT0|TTL10\_HUMAN tr|H0Y6H9|H0Y6H9\_HUMAN sp|Q9H208|O10A2\_HUMAN sp|Q9BYE2|TMPSD\_HUMAN tr|J3KQC6|J3KQC6\_HUMAN tr|E9PRA0|E9PRA0\_HUMAN sp|O00757|F16P2\_HUMAN tr|F5GY10|F5GY10\_HUMAN sp|Q5TGI4|SAMD5\_HUMAN sp|Q8IYB7|DI3L2\_HUMAN sp|Q9Y5R2|MMP24\_HUMAN sp|P25445|TNR6\_HUMAN tr|A0A087X1Z7|A0A087X1Z7\_HUMAN tr|H0YDS4|H0YDS4\_HUMAN sp|Q9UHI8|ATS1\_HUMAN sp|Q8WXG8|S100Z\_HUMAN sp|Q68G74|LHX8\_HUMAN sp|Q9GZW8|MS4A7\_HUMAN sp|Q9P2K2|TXD16\_HUMAN tr|A0A087WZX2|A0A087WZX2\_HUMAN tr|A0A096LNT6|A0A096LNT6\_HUMAN tr|C9J9N2|C9J9N2\_HUMAN tr|H7BXS4|H7BXS4\_HUMAN sp|O95139|NDUB6\_HUMAN tr|E9PN40|E9PN40\_HUMAN tr|E9PIV6|E9PIV6\_HUMAN sp|Q9BZL4|PP12C\_HUMAN tr|H7C132|H7C132\_HUMAN sp|A0PJW6|TM223\_HUMAN tr|E9PRU3|E9PRU3\_HUMAN sp|P54278|PMS2\_HUMAN tr|C9J167|C9J167\_HUMAN tr|F8WE24|F8WE24\_HUMAN sp|Q9BX74|TM2D1\_HUMAN tr|J3KPA2|J3KPA2\_HUMAN tr|E9PHV6|E9PHV6\_HUMAN tr|C9JDQ1|C9JDQ1\_HUMAN Q3ZBD7 sp|Q9ULV5|HSF4\_HUMAN tr|F8WDH4|F8WDH4\_HUMAN tr|E5RK26|E5RK26\_HUMAN sp|P27540|ARNT\_HUMAN sp|Q6ZS86|GLPK5\_HUMAN tr|X6RAS2|X6RAS2\_HUMAN tr|A0A087WV49|A0A087WV49\_HUMAN sp|Q8NAP1|GATS\_HUMAN sp|A6NNH0|GATL1\_HUMAN sp|Q86YN1|DOPP1\_HUMAN sp|Q8IU68|TMC8\_HUMAN tr|C9JBX0|C9JBX0\_HUMAN sp|P60508|SYCY2\_HUMAN sp|Q9P0J1|PDP1\_HUMAN tr|E5RGJ1|E5RGJ1\_HUMAN tr|H3BPT3|H3BPT3\_HUMAN sp|O60682|MUSC\_HUMAN tr|B5MBZ3|B5MBZ3\_HUMAN tr|B7Z7V6|B7Z7V6\_HUMAN tr|E5RIT1|E5RIT1\_HUMAN sp|A6NF89|OR6C6\_HUMAN sp|Q629K1|TRIQK\_HUMAN tr|E5RG44|E5RG44\_HUMAN sp|Q96K58|ZN668\_HUMAN tr|E7EV03|E7EV03\_HUMAN tr|H0YCA0|H0YCA0\_HUMAN tr|I3L4H9|I3L4H9\_HUMAN tr|D6RBU5|D6RBU5\_HUMAN tr|H0Y7H6|H0Y7H6\_HUMAN tr|H0Y4Z8|H0Y4Z8\_HUMAN sp|Q8N1P7|AIM1L\_HUMAN sp|Q8NGG6|OR8BC\_HUMAN tr|A8MSG4|A8MSG4\_HUMAN sp|Q8IXI2|MIRO1\_HUMAN tr|J3KSR5|J3KSR5\_HUMAN tr|E9PLG6|E9PLG6\_HUMAN tr|H0Y7L7|H0Y7L7\_HUMAN sp|Q7L3B6|CD37L\_HUMAN tr|H7C5U0|H7C5U0\_HUMAN tr|B1AL69|B1AL69\_HUMAN tr|A0A075B7E8|A0A075B7E8\_HUMAN tr|H3BPU4|H3BPU4\_HUMAN tr|F5GWV6|F5GWV6\_HUMAN sp|Q92611|EDEM1\_HUMAN sp|P43699|NKX21\_HUMAN sp|P09960|LKHA4\_HUMAN tr|H3BRW8|H3BRW8\_HUMAN tr|B4DQ77|B4DQ77\_HUMAN tr|H3BSV1|H3BSV1\_HUMAN tr|A0A087WU99|A0A087WU99\_HUMAN tr|M0R047|M0R047\_HUMAN tr|H0Y4V2|H0Y4V2\_HUMAN tr|Q6ZN50|Q6ZN50\_HUMAN sp|Q969R2|OSBP2\_HUMAN sp|Q9HC24|LFG4\_HUMAN tr|E7EWY5|E7EWY5\_HUMAN tr|F6UZH7|F6UZH7\_HUMAN sp|Q8NG94|O11H1\_HUMAN tr|A0A087WWP8|A0A087WWP8\_HUMAN sp|Q5VWQ0|RSBN1\_HUMAN sp|O15131|IMA6\_HUMAN sp|Q5T0N5|FBP1L\_HUMAN tr|H7C399|H7C399\_HUMAN sp|O96020|CCNE2\_HUMAN tr|A0A087X052|A0A087X052\_HUMAN tr|Q8WUE3|Q8WUE3\_HUMAN sp|Q5SVZ6|ZMYM1\_HUMAN tr|S4R347|S4R347\_HUMAN tr|Q5SXQ3|Q5SXQ3\_HUMAN sp|Q8NB16|MLKL\_HUMAN tr|Q86YC8|Q86YC8\_HUMAN sp|Q8NHP1|ARK74\_HUMAN tr|G3V2Y8|G3V2Y8\_HUMAN sp|Q8IYK4|GT252\_HUMAN sp|Q6ZNA5|FRRS1\_HUMAN tr|A0A075B6Q2|A0A075B6Q2\_HUMAN sp|Q08AF3|SLFN5\_HUMAN sp|P48066|S6A11\_HUMAN tr|A0A087WXV2|A0A087WXV2\_HUMAN tr|Q5JZB7|Q5JZB7\_HUMAN sp|Q96L42|KCNH8\_HUMAN tr|C9JEI7|C9JEI7\_HUMAN tr|A0A087X0W9|A0A087X0W9\_HUMAN sp|A2VCL2|CC162\_HUMAN tr|H7BYK4|H7BYK4\_HUMAN sp|P41182|BCL6\_HUMAN sp|Q9UHG0|DCDC2\_HUMAN tr|A0A087WVQ4|A0A087WVQ4\_HUMAN tr|F5H625|F5H625\_HUMAN sp|Q2QGD7|ZXDC\_HUMAN tr|J3KRG1|J3KRG1\_HUMAN sp|P23946|CMA1\_HUMAN tr|B0QY90|B0QY90\_HUMAN tr|K7ES84|K7ES84\_HUMAN tr|J3KTQ8|J3KTQ8\_HUMAN sp|Q9HB15|KCNKC\_HUMAN tr|H7C0S8|H7C0S8\_HUMAN sp|O14649|KCNK3\_HUMAN tr|H3BU58|H3BU58\_HUMAN tr|H0Y426|H0Y426\_HUMAN sp|Q8N807|PDILT\_HUMAN sp|Q96RK4|BBS4\_HUMAN tr|Q5JRS0|Q5JRS0\_HUMAN tr|Q5JRR9|Q5JRR9\_HUMAN sp|Q96AG3|S2546\_HUMAN tr|H3BLU7|H3BLU7\_HUMAN sp|O43488|ARK72\_HUMAN sp|O00533|NCHL1\_HUMAN tr|A0A087X0M8|A0A087X0M8\_HUMAN sp|Q9Y5F7|PCDGL\_HUMAN sp|Q86YT5|S13A5\_HUMAN sp|Q8IXB1|DJC10\_HUMAN tr|C9J5N1|C9J5N1\_HUMAN sp|Q96J84|KIRR1\_HUMAN tr|H0Y994|H0Y994\_HUMAN sp|Q9NVG8|TBC13\_HUMAN tr|H7C368|H7C368\_HUMAN tr|H7C2E1|H7C2E1\_HUMAN tr|F8W7L3|F8W7L3\_HUMAN sp|Q8N7R7|CCYL1\_HUMAN tr|B7Z2T3|B7Z2T3\_HUMAN tr|B7Z6R6|B7Z6R6\_HUMAN sp|Q08AN1|ZN616\_HUMAN sp|O95302|FKBP9\_HUMAN tr|H7C1A4|H7C1A4\_HUMAN tr|H3BTE0|H3BTE0\_HUMAN sp|Q63HQ0|AP1AR\_HUMAN sp|Q93070|NAR4\_HUMAN tr|H7C2G2|H7C2G2\_HUMAN sp|P14784|IL2RB\_HUMAN sp|Q14123|PDE1C\_HUMAN sp|Q9HCN6|GPVI\_HUMAN sp|Q8NGS4|O13F1\_HUMAN tr|E9PG71|E9PG71\_HUMAN sp|P54764|EPHA4\_HUMAN sp|Q9HAI6|CX021\_HUMAN sp|Q8N8Q3|ENDOV\_HUMAN sp|Q9H7P6|MB12B\_HUMAN sp|Q9BWV1|BOC\_HUMAN tr|H3BPT7|H3BPT7\_HUMAN sp|P56856|CLD18\_HUMAN tr|H0YEX9|H0YEX9\_HUMAN sp|Q8N554|ZN276\_HUMAN sp|P57087|JAM2\_HUMAN tr|H3BTT0|H3BTT0\_HUMAN sp|Q9H6P5|TASP1\_HUMAN tr|A0A096LNK9|A0A096LNK9\_HUMAN sp|Q9HCI5|MAGE1\_HUMAN tr|F8VVY9|F8VVY9\_HUMAN tr|J3QR03|J3QR03\_HUMAN tr|F8W1G4|F8W1G4\_HUMAN tr|F8W0P9|F8W0P9\_HUMAN tr|C9JCE0|C9JCE0\_HUMAN sp|Q9P0K9|FRS1L\_HUMAN tr|H7BYP8|H7BYP8\_HUMAN tr|D6RGD1|D6RGD1\_HUMAN sp|Q9NP99|TREM1\_HUMAN sp|O95948|ONEC2\_HUMAN sp|E5RHQ5|NPB11\_HUMAN sp|Q9NRQ2|PLS4\_HUMAN sp|P98171|RHG04\_HUMAN tr|E9PCM6|E9PCM6\_HUMAN tr|E9PQ61|E9PQ61\_HUMAN sp|O60384|ZN861\_HUMAN tr|E9PRX9|E9PRX9\_HUMAN tr|A0A088AWN7|A0A088AWN7\_HUMAN sp|Q8IZU9|KIRR3\_HUMAN sp|O15389|SIGL5\_HUMAN sp|Q6PD62|CTR9\_HUMAN sp|Q9H1D9|RPC6\_HUMAN tr|A0A087WZA0|A0A087WZA0\_HUMAN sp|Q9NRJ5|PAPOB\_HUMAN sp|Q6IMI4|ST6B1\_HUMAN tr|G3V1B4|G3V1B4\_HUMAN sp|Q15813|TBCE\_HUMAN sp|Q8N4T0|CBPA6\_HUMAN sp|P12036|NFH\_HUMAN sp|P35558|PCKGC\_HUMAN tr|Q5VSJ8|Q5VSJ8\_HUMAN sp|A6NGB7|TM221\_HUMAN sp|P18577|RHCE\_HUMAN tr|Q5VSJ9|Q5VSJ9\_HUMAN tr|Q5VSJ7|Q5VSJ7\_HUMAN tr|E7EQ47|E7EQ47\_HUMAN tr|F6XSS0|F6XSS0\_HUMAN tr|E7EU00|E7EU00\_HUMAN tr|E7EMF2|E7EMF2\_HUMAN sp|Q9HBJ7|UBP29\_HUMAN sp|Q6ZW76|ANKS3\_HUMAN sp|Q9NWS0|PIHD1\_HUMAN tr|M0R0J2|M0R0J2\_HUMAN sp|P48058|GRIA4\_HUMAN tr|H0YJX0|H0YJX0\_HUMAN sp|Q8WVB3|HEXDC\_HUMAN sp|Q86US8|EST1A\_HUMAN sp|P49336|CDK8\_HUMAN sp|O95263|PDE8B\_HUMAN sp|A0PK00|T120B\_HUMAN sp|Q15119|PDK2\_HUMAN sp|A9QM74|IMA8\_HUMAN tr|H7BZN6|H7BZN6\_HUMAN tr|H0YFA3|H0YFA3\_HUMAN tr|G3V391|G3V391\_HUMAN tr|G3V458|G3V458\_HUMAN sp|Q96EX3|WDR34\_HUMAN tr|H7BZA0|H7BZA0\_HUMAN tr|C9JUZ4|C9JUZ4\_HUMAN sp|P17813|EGLN\_HUMAN sp|Q96EC8|YIPF6\_HUMAN tr|C9JVC3|C9JVC3\_HUMAN tr|H0YM03|H0YM03\_HUMAN tr|C9IZM8|C9IZM8\_HUMAN tr|H0YKU5|H0YKU5\_HUMAN tr|H7C288|H7C288\_HUMAN tr|H0YJT9|H0YJT9\_HUMAN tr|C9JC88|C9JC88\_HUMAN tr|C9J5G7|C9J5G7\_HUMAN sp|P0C7P4|UCRIL\_HUMAN sp|Q9Y4R8|TELO2\_HUMAN sp|Q9P104|DOK5\_HUMAN sp|P22087|FBRL\_HUMAN sp|Q8NHS4|CLHC1\_HUMAN sp|C9JJ37|BTBDJ\_HUMAN sp|P08183|MDR1\_HUMAN sp|Q9Y574|ASB4\_HUMAN sp|Q2M3V2|SWAHA\_HUMAN sp|Q00536|CDK16\_HUMAN tr|A0A087WZU2|A0A087WZU2\_HUMAN tr|E5RGN0|E5RGN0\_HUMAN tr|G3V314|G3V314\_HUMAN REFSEQ:XP\_585019 sp|Q9NYW0|T2R10\_HUMAN tr|M0QYS3|M0QYS3\_HUMAN tr|H7C3E4|H7C3E4\_HUMAN sp|Q05DH4|F16A1\_HUMAN sp|Q9UBX7|KLK11\_HUMAN tr|M0QZI8|M0QZI8\_HUMAN tr|H3BNQ5|H3BNQ5\_HUMAN sp|O60404|O10H3\_HUMAN sp|Q7L0X2|ERIP6\_HUMAN sp|Q9UKG1|DP13A\_HUMAN sp|Q9Y6X5|ENPP4\_HUMAN sp|Q9GZK6|OR2J1\_HUMAN sp|Q9Y463|DYR1B\_HUMAN tr|H0YF61|H0YF61\_HUMAN sp|P35414|APJ\_HUMAN tr|H0YD06|H0YD06\_HUMAN tr|F5GWN9|F5GWN9\_HUMAN tr|H0YEU8|H0YEU8\_HUMAN sp|Q92583|CCL17\_HUMAN tr|H7C3F5|H7C3F5\_HUMAN sp|Q9Y2D4|EXC6B\_HUMAN sp|P01599|KV107\_HUMAN tr|A0A087WX20|A0A087WX20\_HUMAN tr|F8W8M4|F8W8M4\_HUMAN sp|P50453|SPB9\_HUMAN sp|Q49AJ0|F135B\_HUMAN sp|Q9UPY5|XCT\_HUMAN sp|Q99705|MCHR1\_HUMAN sp|Q9NRJ1|MOST1\_HUMAN tr|H0Y997|H0Y997\_HUMAN sp|P22413|ENPP1\_HUMAN sp|Q9BWD1|THIC\_HUMAN sp|Q17RB8|LONF1\_HUMAN tr|H0Y3G5|H0Y3G5\_HUMAN sp|P78411|IRX5\_HUMAN tr|Q6J334|Q6J334\_HUMAN sp|Q9Y2D5|AKAP2\_HUMAN sp|Q5JTZ5|CI152\_HUMAN tr|C9JVY5|C9JVY5\_HUMAN tr|A0A087WV39|A0A087WV39\_HUMAN tr|E5RK69|E5RK69\_HUMAN tr|E7EMC6|E7EMC6\_HUMAN tr|A0A087WTG8|A0A087WTG8\_HUMAN tr|Q5T3E1|Q5T3E1\_HUMAN sp|Q70CQ1|UBP49\_HUMAN sp|Q9NQS5|GPR84\_HUMAN tr|A0A087WVH0|A0A087WVH0\_HUMAN sp|Q9Y5S1|TRPV2\_HUMAN tr|C9JXQ0|C9JXQ0\_HUMAN tr|C9J0V6|C9J0V6\_HUMAN tr|H0YFC4|H0YFC4\_HUMAN tr|C9JHT0|C9JHT0\_HUMAN tr|C9JW88|C9JW88\_HUMAN tr|C9JRZ2|C9JRZ2\_HUMAN sp|Q33E94|RFX4\_HUMAN tr|C9JPV7|C9JPV7\_HUMAN tr|F8WCD5|F8WCD5\_HUMAN tr|H0Y3F1|H0Y3F1\_HUMAN sp|P61565|ENK21\_HUMAN sp|P20807|CAN3\_HUMAN sp|P53667|LIMK1\_HUMAN tr|M0QZ09|M0QZ09\_HUMAN tr|A8MXQ8|A8MXQ8\_HUMAN sp|Q8N6T7|SIR6\_HUMAN sp|Q9H9F9|ARP5\_HUMAN tr|C9JPZ9|C9JPZ9\_HUMAN tr|H7BZV2|H7BZV2\_HUMAN tr|D6RFL4|D6RFL4\_HUMAN tr|A0A087WWF6|A0A087WWF6\_HUMAN sp|P08235|MCR\_HUMAN sp|Q9NXL9|MCM9\_HUMAN tr|H0YBB0|H0YBB0\_HUMAN sp|A6NLW8|DUXA\_HUMAN tr|A0A087WU33|A0A087WU33\_HUMAN tr|M0QYD2|M0QYD2\_HUMAN tr|C9JJ38|C9JJ38\_HUMAN tr|M0QYX4|M0QYX4\_HUMAN sp|Q8WX92|NELFB\_HUMAN sp|Q9UKY1|ZHX1\_HUMAN tr|D6RC54|D6RC54\_HUMAN sp|A8MT19|RHN2P\_HUMAN sp|Q9UHC7|MKRN1\_HUMAN sp|A8MPS7|YDJC\_HUMAN tr|H0YL95|H0YL95\_HUMAN tr|H7C300|H7C300\_HUMAN tr|B8ZZW2|B8ZZW2\_HUMAN tr|Q5SZC6|Q5SZC6\_HUMAN tr|Q5QPL9|Q5QPL9\_HUMAN sp|P0CAP1|MYZAP\_HUMAN tr|Q4VXH1|Q4VXH1\_HUMAN tr|Q08AM3|Q08AM3\_HUMAN tr|H7C516|H7C516\_HUMAN tr|D6RGK8|D6RGK8\_HUMAN tr|D6RAU2|D6RAU2\_HUMAN tr|H3BP33|H3BP33\_HUMAN tr|J3QQK4|J3QQK4\_HUMAN tr|E9PD14|E9PD14\_HUMAN tr|H0YA72|H0YA72\_HUMAN tr|D6R909|D6R909\_HUMAN sp|Q86YL7|PDPN\_HUMAN sp|Q0VG99|MESP2\_HUMAN sp|Q5VX52|SPAT1\_HUMAN tr|H0Y642|H0Y642\_HUMAN sp|Q14CS0|UBX2B\_HUMAN tr|D6R9W6|D6R9W6\_HUMAN sp|P05093|CP17A\_HUMAN tr|F8WB05|F8WB05\_HUMAN tr|J3KS74|J3KS74\_HUMAN sp|Q9Y496|KIF3A\_HUMAN tr|H7C022|H7C022\_HUMAN sp|Q6NZY4|ZCHC8\_HUMAN tr|F5H4P3|F5H4P3\_HUMAN tr|F5GWV0|F5GWV0\_HUMAN sp|Q9Y2C9|TLR6\_HUMAN sp|P48594|SPB4\_HUMAN tr|J3KPF9|J3KPF9\_HUMAN tr|E9PES4|E9PES4\_HUMAN tr|A0A087X011|A0A087X011\_HUMAN tr|B4E3M2|B4E3M2\_HUMAN tr|E9PQ63|E9PQ63\_HUMAN sp|Q9H3S5|PIGM\_HUMAN tr|M0QX43|M0QX43\_HUMAN sp|Q6P597|KLC3\_HUMAN tr|K7EKX2|K7EKX2\_HUMAN tr|C9IYQ4|C9IYQ4\_HUMAN tr|K7EKX6|K7EKX6\_HUMAN tr|M0R253|M0R253\_HUMAN sp|Q5GAN3|RNS13\_HUMAN tr|D6RF73|D6RF73\_HUMAN tr|C9JTB2|C9JTB2\_HUMAN tr|D6RE68|D6RE68\_HUMAN tr|C9JHJ4|C9JHJ4\_HUMAN tr|E7ESY0|E7ESY0\_HUMAN tr|C9JKI6|C9JKI6\_HUMAN sp|Q9P215|POGK\_HUMAN sp|Q8NEX5|WFDC9\_HUMAN sp|P32019|I5P2\_HUMAN tr|M0QXZ8|M0QXZ8\_HUMAN sp|Q9BRX9|WDR83\_HUMAN tr|J3KN52|J3KN52\_HUMAN tr|K7EJI0|K7EJI0\_HUMAN sp|Q16515|ASIC2\_HUMAN tr|K7ERJ6|K7ERJ6\_HUMAN sp|P78406|RAE1L\_HUMAN tr|E9PRI5|E9PRI5\_HUMAN tr|A0A087WWN8|A0A087WWN8\_HUMAN sp|Q13325|IFIT5\_HUMAN tr|A0A087WXI0|A0A087WXI0\_HUMAN tr|A0A087WVG2|A0A087WVG2\_HUMAN sp|Q5U651|RAIN\_HUMAN tr|H0Y334|H0Y334\_HUMAN sp|P11161|EGR2\_HUMAN tr|H0YBE5|H0YBE5\_HUMAN sp|Q96MR9|ZN560\_HUMAN tr|H0Y849|H0Y849\_HUMAN sp|Q96PB1|CASD1\_HUMAN tr|Q8WZ77|Q8WZ77\_HUMAN tr|E9PQV6|E9PQV6\_HUMAN sp|Q8N878|FRMD1\_HUMAN sp|Q92581|SL9A6\_HUMAN tr|A0A075B6I1|A0A075B6I1\_HUMAN tr|A0A075B6J6|A0A075B6J6\_HUMAN tr|H0YMG7|H0YMG7\_HUMAN sp|O94788|AL1A2\_HUMAN sp|Q86W33|TPRA1\_HUMAN sp|Q8WVT3|TPC12\_HUMAN sp|A6NL26|OR5BL\_HUMAN tr|J3KMX3|J3KMX3\_HUMAN sp|Q6E213|AWAT2\_HUMAN tr|A0A087WYV2|A0A087WYV2\_HUMAN sp|A6NCM1|IQCAL\_HUMAN sp|O43896|KIF1C\_HUMAN sp|P02679|FIBG\_HUMAN sp|P02771|FETA\_HUMAN tr|C9JC84|C9JC84\_HUMAN sp|Q15744|CEBPE\_HUMAN tr|C9JEU5|C9JEU5\_HUMAN sp|Q8NET8|TRPV3\_HUMAN tr|F8WD55|F8WD55\_HUMAN tr|A0A087X170|A0A087X170\_HUMAN tr|A0A087WXD8|A0A087WXD8\_HUMAN sp|Q58HT5|AWAT1\_HUMAN sp|Q6P1L5|F117B\_HUMAN sp|Q9H171|ZBP1\_HUMAN sp|O43543|XRCC2\_HUMAN tr|J3KR97|J3KR97\_HUMAN sp|Q6PCB0|VWA1\_HUMAN sp|P87889|GAK10\_HUMAN sp|Q9UQ90|SPG7\_HUMAN sp|Q15431|SYCP1\_HUMAN sp|P30457|1A66\_HUMAN sp|P25089|FPR3\_HUMAN tr|Q5VXJ5|Q5VXJ5\_HUMAN tr|A0A087WZC3|A0A087WZC3\_HUMAN sp|P18462|1A25\_HUMAN P02672 sp|Q9BU19|ZN692\_HUMAN sp|O95758|PTBP3\_HUMAN sp|Q5TKA1|LIN9\_HUMAN tr|F6VUY7|F6VUY7\_HUMAN sp|Q9Y2Z2|MTO1\_HUMAN tr|H7C4T7|H7C4T7\_HUMAN tr|H7C4J5|H7C4J5\_HUMAN tr|H0Y322|H0Y322\_HUMAN tr|C9J5J4|C9J5J4\_HUMAN sp|O43614|OX2R\_HUMAN tr|Q5VVR7|Q5VVR7\_HUMAN tr|S4R3J8|S4R3J8\_HUMAN tr|Q5VVR8|Q5VVR8\_HUMAN sp|Q96NN9|AIFM3\_HUMAN sp|Q96MK2|FA65C\_HUMAN tr|E9PHB5|E9PHB5\_HUMAN tr|E2PU09|E2PU09\_HUMAN sp|A4D1B5|GSAP\_HUMAN tr|B4DLR2|B4DLR2\_HUMAN tr|E9PN76|E9PN76\_HUMAN sp|P41146|OPRX\_HUMAN tr|C9K082|C9K082\_HUMAN tr|B8ZZB3|B8ZZB3\_HUMAN sp|Q9Y223|GLCNE\_HUMAN sp|Q12884|SEPR\_HUMAN sp|P24468|COT2\_HUMAN sp|P62873|GBB1\_HUMAN tr|F6UT28|F6UT28\_HUMAN tr|F6X3N5|F6X3N5\_HUMAN sp|Q9Y388|RBMX2\_HUMAN sp|Q6P3X3|TTC27\_HUMAN tr|C9JHQ1|C9JHQ1\_HUMAN tr|E5RGB1|E5RGB1\_HUMAN sp|Q9Y3X0|CCDC9\_HUMAN tr|Q5JQ33|Q5JQ33\_HUMAN tr|F2Z3E0|F2Z3E0\_HUMAN tr|F8WEC0|F8WEC0\_HUMAN sp|Q9H3L0|MMAD\_HUMAN tr|C9JTB0|C9JTB0\_HUMAN sp|Q9NTI5|PDS5B\_HUMAN sp|Q86XD5|F131B\_HUMAN tr|E7ESV9|E7ESV9\_HUMAN sp|Q8NC01|CLC1A\_HUMAN sp|Q8TAA1|RNS11\_HUMAN tr|E9PFB4|E9PFB4\_HUMAN tr|A0A087WZN4|A0A087WZN4\_HUMAN tr|A0A087WX70|A0A087WX70\_HUMAN tr|K7EIN2|K7EIN2\_HUMAN tr|E7ERD7|E7ERD7\_HUMAN sp|Q9BRJ7|SDOS\_HUMAN sp|Q8N4F7|RN175\_HUMAN tr|G3V5I6|G3V5I6\_HUMAN tr|B8ZZU9|B8ZZU9\_HUMAN sp|P01213|PDYN\_HUMAN sp|P13995|MTDC\_HUMAN tr|K7ENP7|K7ENP7\_HUMAN tr|X6RAJ1|X6RAJ1\_HUMAN sp|Q8TC92|ENOX1\_HUMAN sp|Q13316|DMP1\_HUMAN sp|Q8TBB1|LNX1\_HUMAN tr|Q5SZR1|Q5SZR1\_HUMAN tr|H7C3Z3|H7C3Z3\_HUMAN sp|Q9BYD2|RM09\_HUMAN tr|J9JIE5|J9JIE5\_HUMAN sp|Q9NWW5|CLN6\_HUMAN sp|Q14494|NF2L1\_HUMAN sp|Q9H221|ABCG8\_HUMAN sp|Q13087|PDIA2\_HUMAN tr|F6RGN5|F6RGN5\_HUMAN tr|Q5T277|Q5T277\_HUMAN tr|A0A087WWY2|A0A087WWY2\_HUMAN sp|O14965|AURKA\_HUMAN tr|H0Y5P0|H0Y5P0\_HUMAN sp|Q5HYJ3|FA76B\_HUMAN sp|Q96PJ5|FCRL4\_HUMAN tr|F5GX09|F5GX09\_HUMAN tr|K7EP46|K7EP46\_HUMAN tr|E7EQX7|E7EQX7\_HUMAN sp|Q9H0A0|NAT10\_HUMAN tr|M0R296|M0R296\_HUMAN sp|Q16880|CGT\_HUMAN sp|Q9NZQ0|DJC27\_HUMAN tr|E9PIW1|E9PIW1\_HUMAN tr|Q5SR47|Q5SR47\_HUMAN tr|E7ENF1|E7ENF1\_HUMAN tr|V9GY37|V9GY37\_HUMAN sp|Q9UDY2|ZO2\_HUMAN tr|H0YNJ5|H0YNJ5\_HUMAN sp|O14683|P5I11\_HUMAN tr|E0CX15|E0CX15\_HUMAN sp|Q9P0L9|PK2L1\_HUMAN tr|E7EQW0|E7EQW0\_HUMAN sp|Q13075|BIRC1\_HUMAN tr|F5H8B7|F5H8B7\_HUMAN sp|Q96C57|CL043\_HUMAN tr|F5H7W8|F5H7W8\_HUMAN tr|C9J2Q8|C9J2Q8\_HUMAN tr|G5E971|G5E971\_HUMAN tr|C9JPL0|C9JPL0\_HUMAN sp|Q9UK58|CCNL1\_HUMAN sp|Q9UKW6|ELF5\_HUMAN tr|K7EKU0|K7EKU0\_HUMAN tr|J3KP02|J3KP02\_HUMAN sp|Q8N328|PGBD3\_HUMAN sp|Q9Y483|MTF2\_HUMAN tr|H0YE03|H0YE03\_HUMAN sp|Q15054|DPOD3\_HUMAN sp|Q9UHF7|TRPS1\_HUMAN sp|Q5T7P6|TMM78\_HUMAN tr|H0Y338|H0Y338\_HUMAN tr|C9JAE6|C9JAE6\_HUMAN tr|K7ELQ4|K7ELQ4\_HUMAN sp|Q8IZC7|ZN101\_HUMAN tr|E7EWY6|E7EWY6\_HUMAN sp|P55259|GP2\_HUMAN tr|V9GY54|V9GY54\_HUMAN sp|P41238|ABEC1\_HUMAN sp|P46098|5HT3A\_HUMAN sp|Q5TAQ9|DCAF8\_HUMAN sp|Q8NI36|WDR36\_HUMAN sp|Q9ULC0|MUCEN\_HUMAN tr|C9J2L2|C9J2L2\_HUMAN tr|A0A087WZD3|A0A087WZD3\_HUMAN sp|O75326|SEM7A\_HUMAN sp|P22415|USF1\_HUMAN sp|Q8WZ84|OR8D1\_HUMAN tr|A6PVS1|A6PVS1\_HUMAN sp|Q9BWE0|REPI1\_HUMAN sp|P22001|KCNA3\_HUMAN tr|H0Y3C5|H0Y3C5\_HUMAN sp|P08631|HCK\_HUMAN tr|D6RB95|D6RB95\_HUMAN tr|D6R9F4|D6R9F4\_HUMAN tr|J3KPD6|J3KPD6\_HUMAN tr|M0QXM3|M0QXM3\_HUMAN tr|H7C073|H7C073\_HUMAN sp|Q96P69|GPR78\_HUMAN sp|O60266|ADCY3\_HUMAN sp|Q8NHH1|TTL11\_HUMAN tr|H0YAZ2|H0YAZ2\_HUMAN tr|M0R165|M0R165\_HUMAN sp|P61296|HAND2\_HUMAN sp|Q9BR01|ST4A1\_HUMAN tr|I3L2H1|I3L2H1\_HUMAN tr|E9PB85|E9PB85\_HUMAN tr|B1AMU3|B1AMU3\_HUMAN sp|Q9BSU3|NAA11\_HUMAN tr|B1AKL4|B1AKL4\_HUMAN tr|F5GZC2|F5GZC2\_HUMAN tr|C9JC46|C9JC46\_HUMAN tr|F5H6C1|F5H6C1\_HUMAN sp|A6NM03|O2AG2\_HUMAN sp|P43268|ETV4\_HUMAN tr|E7ENV7|E7ENV7\_HUMAN tr|R4GNA6|R4GNA6\_HUMAN sp|Q15906|VPS72\_HUMAN tr|B7Z5F4|B7Z5F4\_HUMAN sp|Q9UGR2|Z3H7B\_HUMAN sp|Q7Z6J8|UBE3D\_HUMAN tr|A0A087WT09|A0A087WT09\_HUMAN sp|Q9NXE8|CWC25\_HUMAN tr|A0A087WSZ1|A0A087WSZ1\_HUMAN tr|J3KPI1|J3KPI1\_HUMAN sp|Q13046|PSG7\_HUMAN tr|Q5JSG7|Q5JSG7\_HUMAN sp|Q7Z3Q1|S46A3\_HUMAN sp|Q99767|APBA2\_HUMAN tr|D6RGV9|D6RGV9\_HUMAN sp|Q9UHF4|I20RA\_HUMAN tr|D6R967|D6R967\_HUMAN sp|Q9H2U2|IPYR2\_HUMAN tr|H0YJZ6|H0YJZ6\_HUMAN tr|E9PCE7|E9PCE7\_HUMAN tr|H0Y9D8|H0Y9D8\_HUMAN sp|Q9BY41|HDAC8\_HUMAN sp|Q7L273|KCTD9\_HUMAN tr|E7EVA8|E7EVA8\_HUMAN tr|A6NGJ7|A6NGJ7\_HUMAN tr|K7ER14|K7ER14\_HUMAN tr|G3V3T8|G3V3T8\_HUMAN sp|Q9Y5K8|VATD\_HUMAN tr|G3V559|G3V559\_HUMAN tr|X6RHX1|X6RHX1\_HUMAN tr|E7EWF5|E7EWF5\_HUMAN sp|Q14135|VGLL4\_HUMAN tr|J3KNJ7|J3KNJ7\_HUMAN sp|Q8N3J2|METL4\_HUMAN tr|A0A075B6E4|A0A075B6E4\_HUMAN tr|E7EQU6|E7EQU6\_HUMAN tr|E7EUJ2|E7EUJ2\_HUMAN tr|F8W708|F8W708\_HUMAN sp|Q7Z419|R144B\_HUMAN sp|P02724|GLPA\_HUMAN tr|J3KRJ9|J3KRJ9\_HUMAN sp|Q9HAU8|RNPL1\_HUMAN tr|E9PBD5|E9PBD5\_HUMAN tr|H3BQX2|H3BQX2\_HUMAN tr|E7EPG1|E7EPG1\_HUMAN tr|E9PNI5|E9PNI5\_HUMAN sp|Q13201|MMRN1\_HUMAN tr|K7ES25|K7ES25\_HUMAN tr|K7EKF6|K7EKF6\_HUMAN tr|H0YM06|H0YM06\_HUMAN tr|E9PN27|E9PN27\_HUMAN sp|Q6ZWT7|MBOA2\_HUMAN tr|E9PR09|E9PR09\_HUMAN tr|E9PRZ7|E9PRZ7\_HUMAN sp|Q14244|MAP7\_HUMAN sp|A6NDS4|TBC3B\_HUMAN tr|A0A087X1Y6|A0A087X1Y6\_HUMAN sp|Q8NI99|ANGL6\_HUMAN tr|E9PIQ8|E9PIQ8\_HUMAN sp|Q6IPX1|TBC3C\_HUMAN tr|A0A087X2G3|A0A087X2G3\_HUMAN sp|P32929|CGL\_HUMAN tr|E5RHH9|E5RHH9\_HUMAN sp|Q96C92|SDCG3\_HUMAN tr|H7C1E7|H7C1E7\_HUMAN tr|B5A958|B5A958\_HUMAN tr|E9PKV7|E9PKV7\_HUMAN tr|E9PKF2|E9PKF2\_HUMAN tr|F5GXI8|F5GXI8\_HUMAN sp|Q96N19|G137A\_HUMAN sp|Q8NG92|O13H1\_HUMAN sp|Q5QJU3|ACER2\_HUMAN tr|C9K020|C9K020\_HUMAN tr|F5H0Q1|F5H0Q1\_HUMAN tr|H7C348|H7C348\_HUMAN tr|Q5XG96|Q5XG96\_HUMAN tr|G3V171|G3V171\_HUMAN sp|P49326|FMO5\_HUMAN tr|Q9UBD8|Q9UBD8\_HUMAN tr|A0A087WTI7|A0A087WTI7\_HUMAN sp|P11509|CP2A6\_HUMAN sp|Q6ZWL3|CP4V2\_HUMAN sp|Q9BWQ8|LFG2\_HUMAN sp|O00476|NPT4\_HUMAN sp|P10155|RO60\_HUMAN sp|Q05823|RN5A\_HUMAN tr|H7BYM2|H7BYM2\_HUMAN sp|P17405|ASM\_HUMAN sp|P25686|DNJB2\_HUMAN sp|Q8WWN9|ICEF1\_HUMAN tr|E5RGK5|E5RGK5\_HUMAN tr|G3V132|G3V132\_HUMAN sp|Q14593|ZN273\_HUMAN tr|F6X344|F6X344\_HUMAN tr|E9PLZ8|E9PLZ8\_HUMAN tr|F5GYP1|F5GYP1\_HUMAN tr|F5H837|F5H837\_HUMAN sp|Q8NFT8|DNER\_HUMAN sp|Q86WZ6|ZN227\_HUMAN sp|Q16769|QPCT\_HUMAN sp|P06733|ENOA\_HUMAN sp|Q9NS86|LANC2\_HUMAN tr|K7EM90|K7EM90\_HUMAN sp|Q9P232|CNTN3\_HUMAN sp|Q9BXP8|PAPP2\_HUMAN tr|F2Z3F1|F2Z3F1\_HUMAN tr|A0A087WXD6|A0A087WXD6\_HUMAN sp|P43166|CAH7\_HUMAN sp|Q9NUU7|DD19A\_HUMAN tr|I3L0H8|I3L0H8\_HUMAN sp|Q8NB42|ZN527\_HUMAN sp|P54855|UDB15\_HUMAN sp|Q96HD9|ACY3\_HUMAN sp|Q9BXA6|TSSK6\_HUMAN P07224 tr|C9J9C5|C9J9C5\_HUMAN tr|H3BN26|H3BN26\_HUMAN tr|A0A087WXP3|A0A087WXP3\_HUMAN sp|P18564|ITB6\_HUMAN tr|E9PEE8|E9PEE8\_HUMAN tr|H0YMD1|H0YMD1\_HUMAN sp|Q9H7F4|T185B\_HUMAN sp|P01130|LDLR\_HUMAN tr|C9IZK7|C9IZK7\_HUMAN sp|Q8N816|TMM99\_HUMAN sp|Q9Y225|RNF24\_HUMAN tr|V9GZ28|V9GZ28\_HUMAN sp|O00445|SYT5\_HUMAN tr|F8WAI8|F8WAI8\_HUMAN tr|H7C3V2|H7C3V2\_HUMAN tr|E9PNM1|E9PNM1\_HUMAN tr|V9GYN1|V9GYN1\_HUMAN sp|Q9GZP1|NRSN2\_HUMAN tr|C9J6B6|C9J6B6\_HUMAN sp|Q9NUA8|ZBT40\_HUMAN tr|E5RFU2|E5RFU2\_HUMAN sp|O00160|MYO1F\_HUMAN sp|Q8N130|NPT2C\_HUMAN tr|Q86YP9|Q86YP9\_HUMAN tr|H0YG54|H0YG54\_HUMAN sp|Q9Y3B8|ORN\_HUMAN tr|E5RFH6|E5RFH6\_HUMAN tr|F8WDT6|F8WDT6\_HUMAN tr|Q5VT94|Q5VT94\_HUMAN sp|Q9H3K2|GHITM\_HUMAN tr|Q9Y6G2|Q9Y6G2\_HUMAN sp|Q9BXF3|CECR2\_HUMAN tr|B7WPH3|B7WPH3\_HUMAN sp|O15072|ATS3\_HUMAN tr|D6RJB0|D6RJB0\_HUMAN tr|H0YBT8|H0YBT8\_HUMAN sp|Q8NFK1|CXG3\_HUMAN tr|E7ET87|E7ET87\_HUMAN tr|F8W6M3|F8W6M3\_HUMAN tr|H0Y8K6|H0Y8K6\_HUMAN tr|Q5T370|Q5T370\_HUMAN tr|B4DHS6|B4DHS6\_HUMAN sp|Q9NYG2|ZDHC3\_HUMAN tr|H3BMY2|H3BMY2\_HUMAN sp|Q15393|SF3B3\_HUMAN sp|Q96G42|KLD7B\_HUMAN tr|H0Y3H6|H0Y3H6\_HUMAN tr|A2ABF4|A2ABF4\_HUMAN sp|Q8IVS8|GLCTK\_HUMAN sp|A8MPP1|D11L8\_HUMAN tr|B4DTQ9|B4DTQ9\_HUMAN tr|E9PSG1|E9PSG1\_HUMAN sp|Q9H094|NBPF3\_HUMAN tr|E7ERK4|E7ERK4\_HUMAN sp|Q9UJQ4|SALL4\_HUMAN tr|E7EQR1|E7EQR1\_HUMAN tr|Q6NVH2|Q6NVH2\_HUMAN tr|A0A087WU68|A0A087WU68\_HUMAN sp|P01770|HV309\_HUMAN tr|H0YAR3|H0YAR3\_HUMAN sp|Q86UD3|MARH3\_HUMAN sp|Q8IVG5|SAM9L\_HUMAN tr|K7ESJ1|K7ESJ1\_HUMAN sp|P48169|GBRA4\_HUMAN sp|Q96DU7|IP3KC\_HUMAN sp|Q8N228|SCML4\_HUMAN sp|P55290|CAD13\_HUMAN tr|A0A087X0X6|A0A087X0X6\_HUMAN sp|O14576|DC1I1\_HUMAN sp|O94875|SRBS2\_HUMAN tr|M0R216|M0R216\_HUMAN tr|F8W7H5|F8W7H5\_HUMAN sp|P02545|LMNA\_HUMAN tr|K7EIV9|K7EIV9\_HUMAN sp|Q96R09|OR5B2\_HUMAN sp|Q9NYT0|PLEK2\_HUMAN sp|Q12929|EPS8\_HUMAN sp|Q6AI12|ANR40\_HUMAN tr|K7EQQ5|K7EQQ5\_HUMAN tr|Q6IPE9|Q6IPE9\_HUMAN sp|Q8NBL1|PGLT1\_HUMAN sp|Q9Y606|TRUA\_HUMAN sp|Q8NG50|RDM1\_HUMAN tr|K7ES09|K7ES09\_HUMAN sp|O94776|MTA2\_HUMAN tr|B8ZZV6|B8ZZV6\_HUMAN sp|Q5T5J6|SWT1\_HUMAN sp|O43709|WBS22\_HUMAN tr|H7BZK2|H7BZK2\_HUMAN tr|A0A087WVX6|A0A087WVX6\_HUMAN tr|B7ZBF8|B7ZBF8\_HUMAN sp|P35609|ACTN2\_HUMAN sp|P08476|INHBA\_HUMAN tr|Q9UI23|Q9UI23\_HUMAN sp|O75382|TRIM3\_HUMAN tr|A0A087WXS2|A0A087WXS2\_HUMAN tr|M0R0H3|M0R0H3\_HUMAN sp|Q96Q05|TPPC9\_HUMAN sp|Q9H7N4|SFR19\_HUMAN sp|Q8TF68|ZN384\_HUMAN tr|B7Z3R2|B7Z3R2\_HUMAN sp|Q9BV86|NTM1A\_HUMAN tr|G5E9L9|G5E9L9\_HUMAN sp|Q5F1R6|DJC21\_HUMAN tr|A0A087WWQ5|A0A087WWQ5\_HUMAN sp|Q8NAN2|FA73A\_HUMAN tr|F8W7S1|F8W7S1\_HUMAN sp|P36575|ARRC\_HUMAN sp|Q15842|KCNJ8\_HUMAN sp|P05023|AT1A1\_HUMAN tr|C9JE09|C9JE09\_HUMAN tr|C9JPJ6|C9JPJ6\_HUMAN tr|X6R7I6|X6R7I6\_HUMAN tr|H0Y3C6|H0Y3C6\_HUMAN tr|U3KQR8|U3KQR8\_HUMAN tr|F8W9H2|F8W9H2\_HUMAN tr|D6RGJ3|D6RGJ3\_HUMAN sp|Q8TEA7|TBCK\_HUMAN tr|D6RCL2|D6RCL2\_HUMAN tr|J3KNN5|J3KNN5\_HUMAN tr|C9J7E8|C9J7E8\_HUMAN sp|Q9UJV9|DDX41\_HUMAN sp|Q96P88|GNRR2\_HUMAN sp|Q6UXD7|MFSD7\_HUMAN sp|P52737|ZN136\_HUMAN sp|Q6P1K8|T2H2L\_HUMAN sp|Q13895|BYST\_HUMAN tr|K7EKU5|K7EKU5\_HUMAN sp|Q8WYR1|PI3R5\_HUMAN tr|Q5T795|Q5T795\_HUMAN tr|U3KQB5|U3KQB5\_HUMAN sp|Q2TB90|HKDC1\_HUMAN sp|Q02221|CX6A2\_HUMAN tr|J3KSW1|J3KSW1\_HUMAN tr|H3BSP3|H3BSP3\_HUMAN sp|Q86SQ3|EMR4\_HUMAN sp|Q02241|KIF23\_HUMAN sp|Q9HC78|ZBT20\_HUMAN sp|Q9NR28|DBLOH\_HUMAN sp|Q5T442|CXG2\_HUMAN tr|H3BRX6|H3BRX6\_HUMAN sp|Q5T5C0|STXB5\_HUMAN tr|H3BP53|H3BP53\_HUMAN sp|Q96DX8|RTP4\_HUMAN tr|H7BXI3|H7BXI3\_HUMAN tr|M0QZT4|M0QZT4\_HUMAN sp|Q8NGS5|O13C4\_HUMAN tr|F5GX50|F5GX50\_HUMAN tr|F5H796|F5H796\_HUMAN sp|Q9UL12|SARDH\_HUMAN sp|Q6NUP7|PP4R4\_HUMAN sp|Q96LW7|BINCA\_HUMAN tr|F5GXT8|F5GXT8\_HUMAN tr|F5GYH3|F5GYH3\_HUMAN sp|Q8NFY9|KBTB8\_HUMAN tr|J3QL64|J3QL64\_HUMAN sp|Q8IZ63|PRR22\_HUMAN sp|P02686|MBP\_HUMAN tr|H0YGJ6|H0YGJ6\_HUMAN tr|A8MZH3|A8MZH3\_HUMAN sp|Q9UM82|SPAT2\_HUMAN tr|E9PIK1|E9PIK1\_HUMAN tr|C9J6H1|C9J6H1\_HUMAN tr|E9PJ72|E9PJ72\_HUMAN sp|Q8IV45|UN5CL\_HUMAN tr|H0YBR2|H0YBR2\_HUMAN tr|E9PKK8|E9PKK8\_HUMAN tr|E9PPK9|E9PPK9\_HUMAN tr|H3BTG6|H3BTG6\_HUMAN sp|Q14722|KCAB1\_HUMAN sp|Q16587|ZNF74\_HUMAN sp|P62324|BTG1\_HUMAN tr|B1ANM0|B1ANM0\_HUMAN sp|Q96A46|MFRN2\_HUMAN sp|Q86VW0|SESD1\_HUMAN tr|E9PJ77|E9PJ77\_HUMAN tr|E9PRN3|E9PRN3\_HUMAN tr|X6RFA8|X6RFA8\_HUMAN tr|C9JPF8|C9JPF8\_HUMAN tr|H7BZQ6|H7BZQ6\_HUMAN sp|Q50LG9|LRC24\_HUMAN sp|Q9BU89|DOHH\_HUMAN sp|O00241|SIRB1\_HUMAN tr|H0Y827|H0Y827\_HUMAN tr|G3V1D8|G3V1D8\_HUMAN tr|K7EIV2|K7EIV2\_HUMAN tr|H0Y9G3|H0Y9G3\_HUMAN tr|Q2L6I5|Q2L6I5\_HUMAN sp|Q6ZN44|UNC5A\_HUMAN sp|Q6PJG9|LRFN4\_HUMAN sp|Q8TDX6|CGAT1\_HUMAN tr|F5H415|F5H415\_HUMAN sp|Q6DN12|MCTP2\_HUMAN sp|Q6ZN03|CU136\_HUMAN sp|P04217|A1BG\_HUMAN sp|Q9H467|CUED2\_HUMAN tr|D6RAP5|D6RAP5\_HUMAN tr|K7EJZ0|K7EJZ0\_HUMAN tr|K7EQI3|K7EQI3\_HUMAN tr|H7C317|H7C317\_HUMAN tr|H3BUQ3|H3BUQ3\_HUMAN tr|H3BP59|H3BP59\_HUMAN tr|H3BME8|H3BME8\_HUMAN tr|H3BTC3|H3BTC3\_HUMAN tr|H3BMC8|H3BMC8\_HUMAN sp|Q9Y4E8|UBP15\_HUMAN tr|F8VZW0|F8VZW0\_HUMAN tr|G0LNT4|G0LNT4\_HUMAN sp|P78356|PI42B\_HUMAN sp|A6NML5|TM212\_HUMAN sp|Q9HC07|TM165\_HUMAN sp|Q6NSI4|CX057\_HUMAN tr|H7C390|H7C390\_HUMAN sp|Q8TE57|ATS16\_HUMAN tr|Q68D65|Q68D65\_HUMAN tr|K7ELW4|K7ELW4\_HUMAN sp|Q16762|THTR\_HUMAN sp|Q56UQ5|TPT1L\_HUMAN tr|B1AH48|B1AH48\_HUMAN sp|Q8NH89|O5AK3\_HUMAN sp|Q969N2|PIGT\_HUMAN sp|P58743|S26A5\_HUMAN tr|Q7Z7F4|Q7Z7F4\_HUMAN tr|A0A087X2J6|A0A087X2J6\_HUMAN sp|Q8IWF2|FXRD2\_HUMAN sp|Q5H9B9|BM2KL\_HUMAN sp|Q7Z6J2|GRASP\_HUMAN sp|P53990|IST1\_HUMAN tr|K7EQ72|K7EQ72\_HUMAN tr|H3BQ97|H3BQ97\_HUMAN tr|H7C0T7|H7C0T7\_HUMAN sp|O43390|HNRPR\_HUMAN tr|K7ENM7|K7ENM7\_HUMAN sp|Q7L513|FCRLA\_HUMAN sp|Q12882|DPYD\_HUMAN sp|P38606|VATA\_HUMAN tr|E9PML3|E9PML3\_HUMAN sp|Q9UPR6|ZFR2\_HUMAN sp|O60393|NOBOX\_HUMAN tr|H0Y8D9|H0Y8D9\_HUMAN tr|A0A087X1Z8|A0A087X1Z8\_HUMAN sp|Q86VP1|TAXB1\_HUMAN tr|B8ZZD4|B8ZZD4\_HUMAN tr|H7C3L5|H7C3L5\_HUMAN tr|Q6PID9|Q6PID9\_HUMAN sp|Q9P0I2|EMC3\_HUMAN tr|D6RF85|D6RF85\_HUMAN tr|C9JLM9|C9JLM9\_HUMAN tr|B0QZK4|B0QZK4\_HUMAN sp|O43819|SCO2\_HUMAN tr|J3KQ69|J3KQ69\_HUMAN tr|E9PRA6|E9PRA6\_HUMAN tr|F5H6N4|F5H6N4\_HUMAN tr|D6RJI4|D6RJI4\_HUMAN tr|F6SFZ6|F6SFZ6\_HUMAN sp|Q99571|P2RX4\_HUMAN sp|Q15238|PSG5\_HUMAN tr|E7EQY3|E7EQY3\_HUMAN tr|H7C1T0|H7C1T0\_HUMAN sp|Q9BT40|INP5K\_HUMAN sp|Q9NRF8|PYRG2\_HUMAN sp|O14836|TR13B\_HUMAN tr|D6RAX7|D6RAX7\_HUMAN sp|P52824|DGKQ\_HUMAN sp|Q9BY31|ZN717\_HUMAN tr|D6RDF7|D6RDF7\_HUMAN tr|D6R9R1|D6R9R1\_HUMAN sp|Q9H6W3|NO66\_HUMAN sp|Q13620|CUL4B\_HUMAN sp|P13760|2B14\_HUMAN tr|I3L492|I3L492\_HUMAN sp|O60806|TBX19\_HUMAN sp|Q9Y2V3|RX\_HUMAN sp|P60842|IF4A1\_HUMAN sp|Q9HCD5|NCOA5\_HUMAN tr|Q8NEC6|Q8NEC6\_HUMAN sp|P17538|CTRB1\_HUMAN sp|Q6GPI1|CTRB2\_HUMAN tr|H7C0M6|H7C0M6\_HUMAN tr|F5H7P7|F5H7P7\_HUMAN sp|Q96BP3|PPWD1\_HUMAN sp|O00499|BIN1\_HUMAN tr|H0YCM5|H0YCM5\_HUMAN tr|H0Y4R1|H0Y4R1\_HUMAN tr|Q5JRG1|Q5JRG1\_HUMAN sp|Q9BYE7|PCGF6\_HUMAN sp|P07711|CATL1\_HUMAN tr|I7HJS6|I7HJS6\_HUMAN sp|Q7Z3V4|UBE3B\_HUMAN tr|E9PQ78|E9PQ78\_HUMAN sp|Q9UBB9|TFP11\_HUMAN tr|A4D137|A4D137\_HUMAN tr|F5H5T5|F5H5T5\_HUMAN tr|H3BMY4|H3BMY4\_HUMAN tr|F8W816|F8W816\_HUMAN sp|Q8TAC9|SCAM5\_HUMAN sp|Q93098|WNT8B\_HUMAN sp|Q9H8S5|CNTD2\_HUMAN tr|F5GZF8|F5GZF8\_HUMAN sp|Q9BRG2|SH23A\_HUMAN tr|M0R127|M0R127\_HUMAN tr|F8W8F5|F8W8F5\_HUMAN tr|H0YIZ6|H0YIZ6\_HUMAN tr|G3V4T3|G3V4T3\_HUMAN tr|E9PSB7|E9PSB7\_HUMAN tr|A0A087WVV2|A0A087WVV2\_HUMAN sp|O94919|ENDD1\_HUMAN tr|H7BZX7|H7BZX7\_HUMAN tr|F2Z3L8|F2Z3L8\_HUMAN tr|C9JGV7|C9JGV7\_HUMAN tr|H0YN01|H0YN01\_HUMAN sp|Q8WUH2|TGFA1\_HUMAN tr|H0YEM4|H0YEM4\_HUMAN tr|H0YGS1|H0YGS1\_HUMAN tr|H7C4H2|H7C4H2\_HUMAN tr|D6RED9|D6RED9\_HUMAN tr|H0Y301|H0Y301\_HUMAN sp|Q9Y3M8|STA13\_HUMAN tr|F8WAB6|F8WAB6\_HUMAN tr|H7C1F0|H7C1F0\_HUMAN sp|Q8NEQ5|CA162\_HUMAN tr|H0YDE6|H0YDE6\_HUMAN tr|H0YA04|H0YA04\_HUMAN tr|E9PL79|E9PL79\_HUMAN sp|Q96AN5|TM143\_HUMAN tr|F6SDX0|F6SDX0\_HUMAN tr|B4DMT0|B4DMT0\_HUMAN sp|A8MRT5|NPIB5\_HUMAN sp|C9JG80|NPIB4\_HUMAN sp|Q92617|NPIB3\_HUMAN tr|G3V3J8|G3V3J8\_HUMAN tr|E9PKP1|E9PKP1\_HUMAN tr|F8W1E3|F8W1E3\_HUMAN sp|Q86Y37|CACL1\_HUMAN sp|Q5U4P2|ASPH1\_HUMAN tr|A0A088AWP2|A0A088AWP2\_HUMAN tr|H0YMB8|H0YMB8\_HUMAN tr|E9PGQ0|E9PGQ0\_HUMAN sp|Q96H78|S2544\_HUMAN sp|P78412|IRX6\_HUMAN sp|Q5SQS7|SH24B\_HUMAN sp|Q9BZR9|TRIM8\_HUMAN tr|Q5JSM3|Q5JSM3\_HUMAN sp|P28827|PTPRM\_HUMAN sp|Q5VXU1|NKAI2\_HUMAN sp|Q9P253|VPS18\_HUMAN sp|Q96G01|BICD1\_HUMAN tr|A8MVZ6|A8MVZ6\_HUMAN sp|Q6NXP0|EFC12\_HUMAN sp|P14316|IRF2\_HUMAN tr|F5GWJ4|F5GWJ4\_HUMAN tr|K7ERP1|K7ERP1\_HUMAN tr|K7EL91|K7EL91\_HUMAN sp|Q13627|DYR1A\_HUMAN sp|Q00722|PLCB2\_HUMAN sp|Q9QC07|POK18\_HUMAN sp|O00321|ETV2\_HUMAN tr|Q4VAN1|Q4VAN1\_HUMAN sp|Q9BRK4|LZTS2\_HUMAN tr|B1AL13|B1AL13\_HUMAN tr|E5RIQ7|E5RIQ7\_HUMAN tr|D6RFX5|D6RFX5\_HUMAN tr|F8WB69|F8WB69\_HUMAN tr|M0R0W2|M0R0W2\_HUMAN tr|C9JHN8|C9JHN8\_HUMAN tr|C9JPA8|C9JPA8\_HUMAN tr|F5H8H3|F5H8H3\_HUMAN sp|Q5T7W0|ZN618\_HUMAN tr|A0A087WXR2|A0A087WXR2\_HUMAN sp|Q9Y365|PCTL\_HUMAN sp|O60687|SRPX2\_HUMAN tr|H7C3Z6|H7C3Z6\_HUMAN tr|Q86WV8|Q86WV8\_HUMAN sp|P0C881|R10B1\_HUMAN tr|H0Y738|H0Y738\_HUMAN sp|P00505|AATM\_HUMAN sp|B2RC85|R10B2\_HUMAN tr|J3QSS1|J3QSS1\_HUMAN sp|Q8WTT2|NOC3L\_HUMAN sp|P55017|S12A3\_HUMAN sp|Q9Y619|ORNT1\_HUMAN tr|B8ZZA2|B8ZZA2\_HUMAN sp|Q8NHA6|OR2W6\_HUMAN sp|O95292|VAPB\_HUMAN tr|H7C255|H7C255\_HUMAN sp|Q04656|ATP7A\_HUMAN sp|Q9NSD5|S6A13\_HUMAN sp|Q9Y5P6|GMPPB\_HUMAN sp|Q86YS3|RFIP4\_HUMAN sp|Q8WZA2|RPGF4\_HUMAN sp|Q9H1D0|TRPV6\_HUMAN sp|Q9Y2M2|SSUH2\_HUMAN sp|Q8WWX8|SC5AB\_HUMAN tr|C9JAQ0|C9JAQ0\_HUMAN tr|E9PB94|E9PB94\_HUMAN tr|Q5T987|Q5T987\_HUMAN sp|Q8NFU3|TSTD1\_HUMAN tr|H3BPH2|H3BPH2\_HUMAN tr|E7ES64|E7ES64\_HUMAN tr|A0A087WYY1|A0A087WYY1\_HUMAN tr|E9PE76|E9PE76\_HUMAN tr|D6RAS8|D6RAS8\_HUMAN tr|Q5JY65|Q5JY65\_HUMAN sp|Q9BZJ0|CRNL1\_HUMAN sp|Q8WZ92|OR5P2\_HUMAN tr|A0A087WVK5|A0A087WVK5\_HUMAN sp|A4D1S5|RAB19\_HUMAN sp|O60936|NOL3\_HUMAN tr|D6RAU0|D6RAU0\_HUMAN tr|H3BUN4|H3BUN4\_HUMAN tr|J3QKR7|J3QKR7\_HUMAN tr|H3BUP2|H3BUP2\_HUMAN sp|O60518|RNBP6\_HUMAN tr|A0A087WY06|A0A087WY06\_HUMAN sp|Q13268|DHRS2\_HUMAN sp|P20783|NTF3\_HUMAN tr|C9JZP6|C9JZP6\_HUMAN tr|H3BM67|H3BM67\_HUMAN sp|Q14703|MBTP1\_HUMAN tr|H0Y9S4|H0Y9S4\_HUMAN tr|F5GZV9|F5GZV9\_HUMAN tr|J3KSK5|J3KSK5\_HUMAN tr|A0A087WW96|A0A087WW96\_HUMAN sp|Q96LV5|IN4L1\_HUMAN sp|Q2T9F4|IN4L2\_HUMAN tr|Q8WXQ4|Q8WXQ4\_HUMAN sp|O00238|BMR1B\_HUMAN tr|E9PHZ9|E9PHZ9\_HUMAN tr|C9JSC0|C9JSC0\_HUMAN sp|P0DM48|SMCR9\_HUMAN tr|H0YNE5|H0YNE5\_HUMAN sp|A1A4F0|CC055\_HUMAN tr|A0A087X201|A0A087X201\_HUMAN tr|Q5VZ42|Q5VZ42\_HUMAN tr|H7C1E0|H7C1E0\_HUMAN tr|E9PDR1|E9PDR1\_HUMAN sp|Q9BYG4|PAR6G\_HUMAN sp|Q92537|K0247\_HUMAN tr|H7C150|H7C150\_HUMAN tr|Q96FX9|Q96FX9\_HUMAN tr|G3XAL9|G3XAL9\_HUMAN tr|H0Y931|H0Y931\_HUMAN tr|J3KNZ9|J3KNZ9\_HUMAN tr|H0YJL4|H0YJL4\_HUMAN P41361 tr|H0Y6B5|H0Y6B5\_HUMAN sp|Q9Y5G8|PCDG5\_HUMAN sp|A8MXT2|MAGBH\_HUMAN sp|P58340|MLF1\_HUMAN sp|Q92526|TCPW\_HUMAN sp|Q9BS26|ERP44\_HUMAN tr|C9JB28|C9JB28\_HUMAN sp|Q719I0|AHSA2\_HUMAN tr|H0Y5G9|H0Y5G9\_HUMAN tr|C9JPS5|C9JPS5\_HUMAN tr|J3KQC0|J3KQC0\_HUMAN sp|Q03060|CREM\_HUMAN sp|Q9UJS0|CMC2\_HUMAN tr|H0Y561|H0Y561\_HUMAN tr|G5E9U8|G5E9U8\_HUMAN tr|I3L4U4|I3L4U4\_HUMAN sp|Q9Y4A0|JERKL\_HUMAN sp|Q8TBZ2|MYBPP\_HUMAN sp|Q53T59|H1BP3\_HUMAN tr|H0Y7R1|H0Y7R1\_HUMAN tr|I3L4T6|I3L4T6\_HUMAN tr|I3L0V9|I3L0V9\_HUMAN sp|A6NCL7|AN33B\_HUMAN sp|P60604|UB2G2\_HUMAN sp|O00472|ELL2\_HUMAN tr|A0A075B7H0|A0A075B7H0\_HUMAN sp|Q12967|GNDS\_HUMAN sp|Q16539|MK14\_HUMAN tr|B5TY33|B5TY33\_HUMAN tr|A0A096LNS6|A0A096LNS6\_HUMAN tr|H0Y6F9|H0Y6F9\_HUMAN tr|Q6PIR0|Q6PIR0\_HUMAN tr|Q5T8M0|Q5T8M0\_HUMAN tr|K4DIA4|K4DIA4\_HUMAN sp|P30518|V2R\_HUMAN sp|Q6PJW8|CNST\_HUMAN sp|Q9BW62|KATL1\_HUMAN tr|H7C0X0|H7C0X0\_HUMAN sp|P49757|NUMB\_HUMAN tr|B4DLH4|B4DLH4\_HUMAN tr|H7BY36|H7BY36\_HUMAN tr|H9KVB1|H9KVB1\_HUMAN sp|Q01196|RUNX1\_HUMAN tr|H3BU16|H3BU16\_HUMAN tr|G3V3Z8|G3V3Z8\_HUMAN tr|A6NGP5|A6NGP5\_HUMAN tr|H0YH12|H0YH12\_HUMAN sp|Q8IU80|TMPS6\_HUMAN tr|H7C4Y8|H7C4Y8\_HUMAN tr|H0YCX2|H0YCX2\_HUMAN tr|A0A087WYD5|A0A087WYD5\_HUMAN tr|K7EIG3|K7EIG3\_HUMAN tr|C9JGQ4|C9JGQ4\_HUMAN tr|Q5VV23|Q5VV23\_HUMAN sp|P20592|MX2\_HUMAN tr|H0Y9G1|H0Y9G1\_HUMAN tr|H7C257|H7C257\_HUMAN sp|Q8TC29|ENKUR\_HUMAN tr|E9PRS8|E9PRS8\_HUMAN tr|S4R385|S4R385\_HUMAN tr|H9KV56|H9KV56\_HUMAN sp|P01233|CGHB\_HUMAN tr|C9JYG8|C9JYG8\_HUMAN sp|A6NKQ9|CGB1\_HUMAN sp|Q6NT52|CGB2\_HUMAN sp|Q6P3S1|DEN1B\_HUMAN tr|J3KR83|J3KR83\_HUMAN sp|Q96G79|S35A4\_HUMAN tr|E9PL56|E9PL56\_HUMAN sp|Q6UWF7|NXPE4\_HUMAN tr|H0YMT6|H0YMT6\_HUMAN sp|Q6ZNI0|GCNT7\_HUMAN tr|H0YLL2|H0YLL2\_HUMAN tr|V9GY63|V9GY63\_HUMAN tr|A0A087WZE6|A0A087WZE6\_HUMAN sp|P22557|HEM0\_HUMAN tr|Q68DH1|Q68DH1\_HUMAN sp|Q6DKK2|TTC19\_HUMAN tr|E7EW00|E7EW00\_HUMAN sp|P0C7V6|SP202\_HUMAN tr|H0YCG4|H0YCG4\_HUMAN tr|E9PQ40|E9PQ40\_HUMAN sp|Q16563|SYPL1\_HUMAN tr|H0YBL4|H0YBL4\_HUMAN tr|C9J1L5|C9J1L5\_HUMAN tr|E7EMV7|E7EMV7\_HUMAN sp|Q6U949|IG2AS\_HUMAN tr|B5ME96|B5ME96\_HUMAN tr|H3BMU4|H3BMU4\_HUMAN tr|C9K0J8|C9K0J8\_HUMAN sp|Q9NPH0|PPA6\_HUMAN tr|X5D289|X5D289\_HUMAN tr|D3YTH1|D3YTH1\_HUMAN sp|Q8WW22|DNJA4\_HUMAN tr|C9J598|C9J598\_HUMAN sp|Q96ES6|MFSD3\_HUMAN tr|E9PLR3|E9PLR3\_HUMAN sp|O43187|IRAK2\_HUMAN tr|E7EVU1|E7EVU1\_HUMAN sp|Q16853|AOC3\_HUMAN tr|M0R333|M0R333\_HUMAN tr|E9PPG2|E9PPG2\_HUMAN tr|A0A087X0Q9|A0A087X0Q9\_HUMAN sp|Q9BVL4|SELO\_HUMAN tr|J3KPY9|J3KPY9\_HUMAN tr|Q49A12|Q49A12\_HUMAN tr|H0Y895|H0Y895\_HUMAN tr|H0Y8J5|H0Y8J5\_HUMAN sp|Q03923|ZNF85\_HUMAN tr|K7ENV2|K7ENV2\_HUMAN sp|O14793|GDF8\_HUMAN tr|H3BLT2|H3BLT2\_HUMAN sp|Q8NCN5|PDPR\_HUMAN tr|H7C1F2|H7C1F2\_HUMAN tr|E9PKY2|E9PKY2\_HUMAN tr|F5H067|F5H067\_HUMAN sp|Q8TB40|ABHD4\_HUMAN sp|Q9H6S3|ES8L2\_HUMAN sp|P0CW18|PRS56\_HUMAN tr|J3KQW1|J3KQW1\_HUMAN tr|G3V4G2|G3V4G2\_HUMAN tr|M0QZW4|M0QZW4\_HUMAN sp|Q9NP86|CABP5\_HUMAN tr|Q5JUE5|Q5JUE5\_HUMAN tr|H0YAE8|H0YAE8\_HUMAN sp|Q8IWA4|MFN1\_HUMAN tr|E9PM75|E9PM75\_HUMAN sp|Q92928|RAB1C\_HUMAN tr|E9PLD0|E9PLD0\_HUMAN sp|Q9NSI8|SAMN1\_HUMAN sp|Q9Y2Z0|SUGT1\_HUMAN tr|C9JLB7|C9JLB7\_HUMAN tr|U3KQU8|U3KQU8\_HUMAN sp|Q96HP4|OXND1\_HUMAN tr|A2SY06|A2SY06\_HUMAN sp|Q9Y581|INSL6\_HUMAN tr|K7ES11|K7ES11\_HUMAN tr|B5ME80|B5ME80\_HUMAN tr|C9JKR3|C9JKR3\_HUMAN sp|Q9P283|SEM5B\_HUMAN tr|H7BZA3|H7BZA3\_HUMAN tr|H0Y4J5|H0Y4J5\_HUMAN Q05443 sp|Q9HCM4|E41L5\_HUMAN tr|F8WD21|F8WD21\_HUMAN sp|Q9BWF2|TRAIP\_HUMAN sp|Q9Y2W2|WBP11\_HUMAN tr|E7EN91|E7EN91\_HUMAN tr|X6RE50|X6RE50\_HUMAN sp|Q8NEZ5|FBX22\_HUMAN tr|H3BUC1|H3BUC1\_HUMAN tr|C9JGT0|C9JGT0\_HUMAN tr|E7EM75|E7EM75\_HUMAN tr|H3BVA4|H3BVA4\_HUMAN tr|G3V1A9|G3V1A9\_HUMAN sp|Q86VW1|S22AG\_HUMAN sp|Q9C0I4|THS7B\_HUMAN sp|Q8WY91|THAP4\_HUMAN tr|A0A087X2I1|A0A087X2I1\_HUMAN sp|P62333|PRS10\_HUMAN sp|Q03692|COAA1\_HUMAN tr|E9PLN4|E9PLN4\_HUMAN tr|E9PPB0|E9PPB0\_HUMAN tr|E9PJF2|E9PJF2\_HUMAN tr|C9JRS3|C9JRS3\_HUMAN tr|C9JTC5|C9JTC5\_HUMAN tr|H0YI78|H0YI78\_HUMAN sp|Q6UWI2|PARM1\_HUMAN tr|H0YDB5|H0YDB5\_HUMAN tr|E7EW77|E7EW77\_HUMAN tr|X6REY2|X6REY2\_HUMAN sp|Q9NX38|F206A\_HUMAN tr|Q5T3I3|Q5T3I3\_HUMAN sp|O95859|TSN12\_HUMAN tr|H0YIC9|H0YIC9\_HUMAN tr|E7ET86|E7ET86\_HUMAN tr|B4DX41|B4DX41\_HUMAN sp|Q8IVW4|CDKL3\_HUMAN tr|F8VZX1|F8VZX1\_HUMAN tr|Q5T6W8|Q5T6W8\_HUMAN tr|E9PIJ7|E9PIJ7\_HUMAN sp|O95626|AN32D\_HUMAN tr|H7BYE3|H7BYE3\_HUMAN sp|P49916|DNLI3\_HUMAN tr|E7EVI1|E7EVI1\_HUMAN sp|Q96M11|HYLS1\_HUMAN sp|Q7L014|DDX46\_HUMAN sp|Q86TM6|SYVN1\_HUMAN sp|C9JJH3|U17LA\_HUMAN tr|I3L0E4|I3L0E4\_HUMAN tr|A0A087X1H9|A0A087X1H9\_HUMAN tr|H0Y339|H0Y339\_HUMAN tr|G3V5B7|G3V5B7\_HUMAN tr|G3V4S2|G3V4S2\_HUMAN tr|A0A087WWD1|A0A087WWD1\_HUMAN sp|Q8N966|ZDH22\_HUMAN tr|A8MX75|A8MX75\_HUMAN sp|P18074|ERCC2\_HUMAN tr|G3V358|G3V358\_HUMAN tr|H0YMG1|H0YMG1\_HUMAN tr|E7EVE9|E7EVE9\_HUMAN sp|B7Z6K7|ZN814\_HUMAN tr|G3V383|G3V383\_HUMAN tr|G3V285|G3V285\_HUMAN tr|D6RJG9|D6RJG9\_HUMAN tr|H0YJ56|H0YJ56\_HUMAN tr|G3V271|G3V271\_HUMAN sp|Q06828|FMOD\_HUMAN sp|Q99439|CNN2\_HUMAN tr|H0Y850|H0Y850\_HUMAN sp|A8K7I4|CLCA1\_HUMAN sp|Q8TEY7|UBP33\_HUMAN sp|Q8NA54|IQUB\_HUMAN sp|A6NFF2|NP1L6\_HUMAN sp|O60235|TM11D\_HUMAN tr|B1ANV5|B1ANV5\_HUMAN tr|C9J7L8|C9J7L8\_HUMAN tr|M0QX46|M0QX46\_HUMAN sp|P13796|PLSL\_HUMAN sp|Q8NCR9|CLRN3\_HUMAN tr|G3V471|G3V471\_HUMAN sp|P56179|DLX6\_HUMAN sp|Q13153|PAK1\_HUMAN sp|Q8NGI4|OR4DB\_HUMAN tr|B3KNX7|B3KNX7\_HUMAN tr|I3L3V6|I3L3V6\_HUMAN tr|A8MV53|A8MV53\_HUMAN tr|C9J3W3|C9J3W3\_HUMAN sp|Q9NQ55|SSF1\_HUMAN sp|Q9P2A4|ABI3\_HUMAN tr|M0R0S9|M0R0S9\_HUMAN tr|E9PKL2|E9PKL2\_HUMAN tr|M0R1X0|M0R1X0\_HUMAN tr|E9PRB6|E9PRB6\_HUMAN tr|G5EA44|G5EA44\_HUMAN tr|C9IZW5|C9IZW5\_HUMAN sp|Q9Y5E6|PCDB3\_HUMAN sp|O75159|SOCS5\_HUMAN tr|H0Y7Z4|H0Y7Z4\_HUMAN tr|H0Y790|H0Y790\_HUMAN tr|H7BZ98|H7BZ98\_HUMAN sp|Q5K4E3|POLS2\_HUMAN tr|J3QR12|J3QR12\_HUMAN tr|J3QL04|J3QL04\_HUMAN sp|P24394|IL4RA\_HUMAN sp|Q13630|FCL\_HUMAN tr|A0A087X171|A0A087X171\_HUMAN tr|H3BNC9|H3BNC9\_HUMAN tr|A0A087WVR7|A0A087WVR7\_HUMAN tr|E9PKL9|E9PKL9\_HUMAN tr|A0A087WT23|A0A087WT23\_HUMAN sp|C9JL84|HHLA1\_HUMAN sp|Q9H5J0|ZBTB3\_HUMAN sp|P51800|CLCKA\_HUMAN tr|A0A087WZW2|A0A087WZW2\_HUMAN tr|M0R104|M0R104\_HUMAN tr|J3KSU3|J3KSU3\_HUMAN tr|C9JYZ0|C9JYZ0\_HUMAN sp|Q96DW6|S2538\_HUMAN sp|Q9UJJ9|GNPTG\_HUMAN sp|Q6IV72|ZN425\_HUMAN tr|D6RB07|D6RB07\_HUMAN tr|A4QMS1|A4QMS1\_HUMAN tr|D6RIZ6|D6RIZ6\_HUMAN tr|H7BYJ1|H7BYJ1\_HUMAN sp|P41440|S19A1\_HUMAN tr|D3DSM6|D3DSM6\_HUMAN sp|Q6J4K2|NCKX6\_HUMAN tr|F8VR99|F8VR99\_HUMAN tr|F8W134|F8W134\_HUMAN tr|F8VWW9|F8VWW9\_HUMAN tr|F8VTU4|F8VTU4\_HUMAN tr|H7C420|H7C420\_HUMAN tr|H0Y8E8|H0Y8E8\_HUMAN sp|O75604|UBP2\_HUMAN sp|Q8IWU5|SULF2\_HUMAN tr|I3L0E5|I3L0E5\_HUMAN tr|B1AMP9|B1AMP9\_HUMAN tr|H0YLH9|H0YLH9\_HUMAN tr|A0A096P6K9|A0A096P6K9\_HUMAN tr|F6RVM3|F6RVM3\_HUMAN sp|Q9UH73|COE1\_HUMAN tr|Q68D78|Q68D78\_HUMAN tr|H0YFV6|H0YFV6\_HUMAN sp|P32745|SSR3\_HUMAN tr|H0YNU7|H0YNU7\_HUMAN tr|A0A087WTE5|A0A087WTE5\_HUMAN tr|D6RE77|D6RE77\_HUMAN sp|Q9UPN9|TRI33\_HUMAN tr|H0Y612|H0Y612\_HUMAN tr|E7EN20|E7EN20\_HUMAN tr|F2Z3L2|F2Z3L2\_HUMAN sp|O76070|SYUG\_HUMAN tr|A0A087X1U1|A0A087X1U1\_HUMAN tr|F5GXM9|F5GXM9\_HUMAN sp|Q9UPQ3|AGAP1\_HUMAN sp|Q96JK4|HIPL1\_HUMAN sp|Q96C00|ZBTB9\_HUMAN sp|C9J069|CI172\_HUMAN sp|Q96E16|SMI19\_HUMAN tr|Q5JUE6|Q5JUE6\_HUMAN sp|Q58DX5|NADL2\_HUMAN tr|A0A087WW67|A0A087WW67\_HUMAN tr|A0A087WT42|A0A087WT42\_HUMAN tr|Q5VVW5|Q5VVW5\_HUMAN sp|Q96M94|KLH15\_HUMAN tr|Q5VVV3|Q5VVV3\_HUMAN tr|Q5VVV4|Q5VVV4\_HUMAN tr|C9J5Z5|C9J5Z5\_HUMAN tr|E9PKH0|E9PKH0\_HUMAN tr|E9PRH3|E9PRH3\_HUMAN tr|A0A087WTW6|A0A087WTW6\_HUMAN tr|M3ZCP1|M3ZCP1\_HUMAN tr|K7ELT9|K7ELT9\_HUMAN sp|O75310|UDB11\_HUMAN sp|Q96BQ1|FAM3D\_HUMAN sp|Q32NB8|PGPS1\_HUMAN tr|H7C3F8|H7C3F8\_HUMAN tr|H3BRG5|H3BRG5\_HUMAN sp|Q9P2N2|RHG28\_HUMAN tr|Q5SW02|Q5SW02\_HUMAN sp|Q4G176|ACSF3\_HUMAN tr|F8W6J7|F8W6J7\_HUMAN tr|A8MT40|A8MT40\_HUMAN sp|Q8N118|CP4X1\_HUMAN sp|Q9Y2W3|S45A1\_HUMAN sp|Q86X29|LSR\_HUMAN tr|J3QR07|J3QR07\_HUMAN sp|Q96MU7|YTDC1\_HUMAN sp|O43167|ZBT24\_HUMAN sp|Q8TE59|ATS19\_HUMAN sp|P61578|REC16\_HUMAN tr|J3QQQ8|J3QQQ8\_HUMAN sp|O76003|GLRX3\_HUMAN tr|B4DDF4|B4DDF4\_HUMAN tr|H0Y4Y1|H0Y4Y1\_HUMAN tr|C9JK03|C9JK03\_HUMAN tr|B4DUT8|B4DUT8\_HUMAN sp|Q16322|KCA10\_HUMAN tr|G3V5F7|G3V5F7\_HUMAN tr|H0YB60|H0YB60\_HUMAN tr|H0YBS2|H0YBS2\_HUMAN tr|E9PNL2|E9PNL2\_HUMAN tr|J3KSW8|J3KSW8\_HUMAN sp|Q8N137|CNTRB\_HUMAN tr|Q5T276|Q5T276\_HUMAN tr|A0A087WTM8|A0A087WTM8\_HUMAN tr|E9PDH6|E9PDH6\_HUMAN tr|M0QZC7|M0QZC7\_HUMAN tr|K4DI94|K4DI94\_HUMAN tr|M0R2N0|M0R2N0\_HUMAN tr|Q5T278|Q5T278\_HUMAN tr|H0YF26|H0YF26\_HUMAN tr|M0QYR7|M0QYR7\_HUMAN sp|Q8N8B7|TEANC\_HUMAN tr|M0QYT1|M0QYT1\_HUMAN tr|H7C008|H7C008\_HUMAN tr|A0A087WWC3|A0A087WWC3\_HUMAN tr|K7EMY3|K7EMY3\_HUMAN tr|K7EPY1|K7EPY1\_HUMAN tr|K7ENL0|K7ENL0\_HUMAN tr|K7EJV0|K7EJV0\_HUMAN tr|G3V4E5|G3V4E5\_HUMAN tr|E9PEW0|E9PEW0\_HUMAN tr|J3KNN1|J3KNN1\_HUMAN sp|Q9HCQ5|GALT9\_HUMAN sp|A8MQ14|ZN850\_HUMAN tr|C9J826|C9J826\_HUMAN tr|A0A087X0M6|A0A087X0M6\_HUMAN sp|P23284|PPIB\_HUMAN sp|Q14432|PDE3A\_HUMAN tr|C9JTX4|C9JTX4\_HUMAN tr|C9JKY1|C9JKY1\_HUMAN tr|C9JK18|C9JK18\_HUMAN tr|Q8N4W3|Q8N4W3\_HUMAN tr|J3KTH8|J3KTH8\_HUMAN sp|Q86UD4|ZN329\_HUMAN tr|K7EIY4|K7EIY4\_HUMAN tr|M0QZ19|M0QZ19\_HUMAN sp|P36542|ATPG\_HUMAN tr|M0R271|M0R271\_HUMAN tr|F5H7L7|F5H7L7\_HUMAN sp|P22735|TGM1\_HUMAN sp|Q4VXA5|KHDC1\_HUMAN sp|Q15363|TMED2\_HUMAN tr|E7EQ72|E7EQ72\_HUMAN tr|Q29704|Q29704\_HUMAN sp|Q6P4F2|ADXL\_HUMAN tr|K7EMB0|K7EMB0\_HUMAN sp|A6NFC5|TM235\_HUMAN sp|Q6ZRR5|TM136\_HUMAN sp|Q15542|TAF5\_HUMAN sp|Q8IV13|CCNJL\_HUMAN tr|A0A087WYU6|A0A087WYU6\_HUMAN tr|F6RF56|F6RF56\_HUMAN tr|H0YLC3|H0YLC3\_HUMAN tr|A0A067XG54|A0A067XG54\_HUMAN sp|Q6PID8|KLD10\_HUMAN tr|K7EMR1|K7EMR1\_HUMAN tr|M0R1X7|M0R1X7\_HUMAN sp|Q96N77|ZN641\_HUMAN sp|Q9HC96|CAN10\_HUMAN tr|B7WPF5|B7WPF5\_HUMAN tr|E9PIM7|E9PIM7\_HUMAN tr|B7Z369|B7Z369\_HUMAN tr|V9GZ49|V9GZ49\_HUMAN tr|K7ERK4|K7ERK4\_HUMAN tr|H7C199|H7C199\_HUMAN tr|K7EL44|K7EL44\_HUMAN sp|Q8N6C8|LIRA3\_HUMAN tr|J3KNU6|J3KNU6\_HUMAN sp|Q9BPU9|B9D2\_HUMAN sp|P40933|IL15\_HUMAN sp|A6NFX1|MFS2B\_HUMAN tr|A0A087X0N0|A0A087X0N0\_HUMAN tr|H3BP92|H3BP92\_HUMAN tr|C9J1B5|C9J1B5\_HUMAN tr|A0A075B768|A0A075B768\_HUMAN sp|A4FU49|SH321\_HUMAN tr|K7EKN6|K7EKN6\_HUMAN tr|K7ELI3|K7ELI3\_HUMAN sp|Q9ULM0|PKHH1\_HUMAN sp|Q6UWY2|PRS57\_HUMAN tr|Q96JS4|Q96JS4\_HUMAN sp|Q6NY19|KANK3\_HUMAN sp|O94844|RHBT1\_HUMAN sp|Q96GX1|TECT2\_HUMAN tr|J3QL54|J3QL54\_HUMAN tr|H0Y6E8|H0Y6E8\_HUMAN sp|Q8IVV8|NKAI4\_HUMAN tr|H0Y702|H0Y702\_HUMAN tr|J3KT10|J3KT10\_HUMAN sp|Q9BW27|NUP85\_HUMAN tr|S4R3V7|S4R3V7\_HUMAN sp|Q8NGJ6|O51A4\_HUMAN sp|Q8NGJ7|O51A2\_HUMAN tr|F5H0I5|F5H0I5\_HUMAN sp|P14550|AK1A1\_HUMAN sp|P08240|SRPR\_HUMAN sp|Q99865|SPI2A\_HUMAN sp|O43508|TNF12\_HUMAN tr|J3KTM5|J3KTM5\_HUMAN sp|Q96RP7|G3ST4\_HUMAN sp|P20366|TKN1\_HUMAN tr|H7BZ77|H7BZ77\_HUMAN tr|J3KT01|J3KT01\_HUMAN tr|H7C0I5|H7C0I5\_HUMAN tr|C9JLW9|C9JLW9\_HUMAN tr|H7C4P0|H7C4P0\_HUMAN tr|M0R3C8|M0R3C8\_HUMAN tr|M0QYC6|M0QYC6\_HUMAN tr|Q5SQ17|Q5SQ17\_HUMAN sp|A6NGU5|GGT3\_HUMAN tr|B5MCK8|B5MCK8\_HUMAN sp|P59540|T2R46\_HUMAN sp|P36268|GGT2\_HUMAN tr|F5H0W7|F5H0W7\_HUMAN tr|E9PRY8|E9PRY8\_HUMAN tr|J3QLS9|J3QLS9\_HUMAN sp|Q9Y6K8|KAD5\_HUMAN tr|J3QLH0|J3QLH0\_HUMAN tr|E9PS41|E9PS41\_HUMAN tr|E9PD56|E9PD56\_HUMAN sp|Q9P1V8|SAM15\_HUMAN sp|Q6RFH5|WDR74\_HUMAN tr|H0YFL1|H0YFL1\_HUMAN tr|E9PLD2|E9PLD2\_HUMAN sp|Q7L2R6|ZN765\_HUMAN sp|Q13508|NAR3\_HUMAN tr|E7ESB3|E7ESB3\_HUMAN tr|E7EMT0|E7EMT0\_HUMAN tr|C9JA91|C9JA91\_HUMAN sp|Q86UZ6|ZBT46\_HUMAN tr|H7C441|H7C441\_HUMAN tr|Q86XJ5|Q86XJ5\_HUMAN tr|H7BZJ5|H7BZJ5\_HUMAN sp|A6NJW9|CD8BL\_HUMAN sp|Q2TAA5|ALG11\_HUMAN tr|Q31611|Q31611\_HUMAN tr|H0Y5A9|H0Y5A9\_HUMAN tr|D6RBB6|D6RBB6\_HUMAN sp|Q9UBF1|MAGC2\_HUMAN tr|Q29897|Q29897\_HUMAN sp|Q9HCL3|ZFP14\_HUMAN sp|Q9UBE8|NLK\_HUMAN tr|A0A087WSY3|A0A087WSY3\_HUMAN tr|H0YD75|H0YD75\_HUMAN tr|A6NJB5|A6NJB5\_HUMAN tr|E7EQN9|E7EQN9\_HUMAN sp|Q5HYJ1|TECRL\_HUMAN tr|I3L3R7|I3L3R7\_HUMAN sp|Q8IZM8|ZN654\_HUMAN tr|E9PD39|E9PD39\_HUMAN tr|K7ESF6|K7ESF6\_HUMAN tr|F5H0A9|F5H0A9\_HUMAN sp|Q0D2J5|ZN763\_HUMAN tr|J3QRW1|J3QRW1\_HUMAN tr|J3QSA9|J3QSA9\_HUMAN tr|J3QQM1|J3QQM1\_HUMAN tr|V9GXZ6|V9GXZ6\_HUMAN sp|O60575|ISK4\_HUMAN tr|J3QLH6|J3QLH6\_HUMAN tr|J3KRP2|J3KRP2\_HUMAN tr|J3QSE0|J3QSE0\_HUMAN tr|E9PCY0|E9PCY0\_HUMAN tr|G3XAB9|G3XAB9\_HUMAN sp|O75830|SPI2\_HUMAN sp|P46721|SO1A2\_HUMAN sp|P40337|VHL\_HUMAN tr|A0A087X018|A0A087X018\_HUMAN tr|B4DIZ1|B4DIZ1\_HUMAN tr|A2A2M0|A2A2M0\_HUMAN sp|Q7RTY7|OVCH1\_HUMAN tr|A0A087WXD2|A0A087WXD2\_HUMAN tr|H0Y3K0|H0Y3K0\_HUMAN sp|O60684|IMA7\_HUMAN sp|Q13330|MTA1\_HUMAN tr|E7ESY4|E7ESY4\_HUMAN tr|B4DXC8|B4DXC8\_HUMAN sp|Q7L7V1|DHX32\_HUMAN tr|X6R717|X6R717\_HUMAN tr|H0Y8X6|H0Y8X6\_HUMAN sp|Q330K2|NDUF6\_HUMAN sp|P15408|FOSL2\_HUMAN tr|A0A087WZ46|A0A087WZ46\_HUMAN sp|P18505|GBRB1\_HUMAN tr|S4R313|S4R313\_HUMAN tr|J3QQW2|J3QQW2\_HUMAN sp|Q9NP74|PALMD\_HUMAN sp|Q8N2F6|ARM10\_HUMAN sp|Q8N612|F16A2\_HUMAN tr|C9JBG5|C9JBG5\_HUMAN tr|J3KS54|J3KS54\_HUMAN sp|Q5VZM2|RRAGB\_HUMAN tr|H0YDP8|H0YDP8\_HUMAN tr|E9PJK5|E9PJK5\_HUMAN tr|B3KT61|B3KT61\_HUMAN tr|E9PKQ9|E9PKQ9\_HUMAN tr|H0Y8R5|H0Y8R5\_HUMAN tr|G5E977|G5E977\_HUMAN tr|K7ELT4|K7ELT4\_HUMAN tr|D6R9L0|D6R9L0\_HUMAN tr|H0Y8W2|H0Y8W2\_HUMAN tr|D6R9Z1|D6R9Z1\_HUMAN sp|Q6XQN6|PNCB\_HUMAN tr|C9J8U2|C9J8U2\_HUMAN tr|D6RFX4|D6RFX4\_HUMAN sp|Q9H9L3|I20L2\_HUMAN sp|O00391|QSOX1\_HUMAN tr|Q5TH30|Q5TH30\_HUMAN tr|U3KQ97|U3KQ97\_HUMAN sp|Q9H0A3|T191A\_HUMAN sp|P0C7N4|T191B\_HUMAN tr|X6RHM1|X6RHM1\_HUMAN tr|A8MUE1|A8MUE1\_HUMAN tr|H3BM18|H3BM18\_HUMAN sp|Q6P3X8|PGBD2\_HUMAN sp|Q96FC7|PHIPL\_HUMAN sp|Q8NH61|O51F2\_HUMAN sp|Q12907|LMAN2\_HUMAN tr|C9JLV3|C9JLV3\_HUMAN tr|D6RDX1|D6RDX1\_HUMAN tr|H0YJG9|H0YJG9\_HUMAN tr|F5H6C2|F5H6C2\_HUMAN tr|V9GYJ9|V9GYJ9\_HUMAN sp|Q5T1V6|DDX59\_HUMAN tr|V9GYL9|V9GYL9\_HUMAN tr|A2A343|A2A343\_HUMAN tr|C9JW75|C9JW75\_HUMAN tr|H3BSS6|H3BSS6\_HUMAN sp|O94832|MYO1D\_HUMAN tr|J3QRN6|J3QRN6\_HUMAN tr|G3V201|G3V201\_HUMAN sp|Q92637|FCGRB\_HUMAN tr|A0A087WXZ6|A0A087WXZ6\_HUMAN tr|H0Y9T5|H0Y9T5\_HUMAN sp|O95025|SEM3D\_HUMAN sp|A6NKC4|FCGRC\_HUMAN sp|Q9Y4D1|DAAM1\_HUMAN sp|P80370|DLK1\_HUMAN sp|Q9H427|KCNKF\_HUMAN tr|H3BTK7|H3BTK7\_HUMAN sp|O15503|INSI1\_HUMAN tr|D6RAR6|D6RAR6\_HUMAN sp|Q14CZ7|FAKD3\_HUMAN sp|Q9UJV3|TRIM1\_HUMAN sp|Q8NGZ9|O2T10\_HUMAN sp|P23769|GATA2\_HUMAN sp|Q9GZZ6|ACH10\_HUMAN sp|Q13607|OR2F1\_HUMAN tr|F5H0U6|F5H0U6\_HUMAN sp|Q32MK0|MYLK3\_HUMAN tr|A0A087WXM8|A0A087WXM8\_HUMAN sp|P06133|UD2B4\_HUMAN tr|I1Z9G3|I1Z9G3\_HUMAN tr|H0YD21|H0YD21\_HUMAN tr|C9J8U9|C9J8U9\_HUMAN tr|H0YEB7|H0YEB7\_HUMAN tr|H7C3R2|H7C3R2\_HUMAN sp|Q96IK1|BOD1\_HUMAN tr|I3L2X7|I3L2X7\_HUMAN tr|Q5JTF2|Q5JTF2\_HUMAN sp|P35241|RADI\_HUMAN tr|H0YET8|H0YET8\_HUMAN sp|P04040|CATA\_HUMAN tr|C9JRT9|C9JRT9\_HUMAN tr|K7EQH5|K7EQH5\_HUMAN sp|Q08117|AES\_HUMAN sp|Q8IZW8|TENS4\_HUMAN sp|B2RNN3|C1T9B\_HUMAN tr|X6R8F3|X6R8F3\_HUMAN sp|P0C862|C1T9A\_HUMAN sp|Q9NPB3|CABP2\_HUMAN sp|P80188|NGAL\_HUMAN tr|F5H458|F5H458\_HUMAN sp|Q5XUX1|FBXW9\_HUMAN tr|C9J2U0|C9J2U0\_HUMAN sp|A6ND48|O14I1\_HUMAN tr|I3L413|I3L413\_HUMAN sp|O15209|ZBT22\_HUMAN tr|I3L4R7|I3L4R7\_HUMAN sp|P24864|CCNE1\_HUMAN tr|F5H6X0|F5H6X0\_HUMAN tr|F5GX35|F5GX35\_HUMAN sp|Q96DD7|SHSA4\_HUMAN sp|Q8NB49|AT11C\_HUMAN tr|B7ZA25|B7ZA25\_HUMAN tr|B1AQP1|B1AQP1\_HUMAN sp|Q16695|H31T\_HUMAN tr|H0YDJ0|H0YDJ0\_HUMAN sp|Q9NRW3|ABC3C\_HUMAN tr|J3KTM2|J3KTM2\_HUMAN tr|H7C1J6|H7C1J6\_HUMAN sp|Q01082|SPTB2\_HUMAN tr|A0A087WUZ3|A0A087WUZ3\_HUMAN tr|A5PKY6|A5PKY6\_HUMAN sp|P29144|TPP2\_HUMAN tr|Q5VZU9|Q5VZU9\_HUMAN sp|Q8N4J0|CI041\_HUMAN tr|E9PC52|E9PC52\_HUMAN sp|Q16576|RBBP7\_HUMAN tr|V9GZ22|V9GZ22\_HUMAN sp|O00139|KIF2A\_HUMAN tr|H7C4N3|H7C4N3\_HUMAN tr|H7C570|H7C570\_HUMAN tr|K7EIE6|K7EIE6\_HUMAN P50448 sp|Q5TEZ5|CF163\_HUMAN tr|M0R1T2|M0R1T2\_HUMAN tr|H0Y8F4|H0Y8F4\_HUMAN sp|O00337|S28A1\_HUMAN sp|O75886|STAM2\_HUMAN sp|Q13291|SLAF1\_HUMAN sp|Q96A58|RERG\_HUMAN tr|H7C4Q3|H7C4Q3\_HUMAN tr|F8W0G4|F8W0G4\_HUMAN tr|B4DXP5|B4DXP5\_HUMAN tr|F8VZX2|F8VZX2\_HUMAN sp|P16471|PRLR\_HUMAN tr|J3QRW8|J3QRW8\_HUMAN sp|Q8WW62|TMED6\_HUMAN tr|J3KT08|J3KT08\_HUMAN tr|M0R1M2|M0R1M2\_HUMAN tr|K7EM25|K7EM25\_HUMAN sp|Q5SVS4|KMCP1\_HUMAN sp|Q9H6F5|CCD86\_HUMAN tr|J3KSP1|J3KSP1\_HUMAN tr|F8WDC7|F8WDC7\_HUMAN tr|H7C405|H7C405\_HUMAN tr|E7EMB1|E7EMB1\_HUMAN tr|A0A087WUA5|A0A087WUA5\_HUMAN sp|Q9UH65|SWP70\_HUMAN sp|Q9H223|EHD4\_HUMAN tr|C9J8Z4|C9J8Z4\_HUMAN tr|J3QKX9|J3QKX9\_HUMAN sp|P48167|GLRB\_HUMAN sp|Q8WWF8|CAPSL\_HUMAN sp|Q8N6N3|CA052\_HUMAN tr|J3QT25|J3QT25\_HUMAN tr|A0A087WVI4|A0A087WVI4\_HUMAN tr|A0A087WT65|A0A087WT65\_HUMAN tr|H0YNS9|H0YNS9\_HUMAN sp|Q5SWH9|TMM69\_HUMAN tr|B4DJK6|B4DJK6\_HUMAN tr|F8WAV5|F8WAV5\_HUMAN sp|Q9UK55|ZPI\_HUMAN sp|O60809|PRA10\_HUMAN sp|Q15545|TAF7\_HUMAN tr|H0Y5H7|H0Y5H7\_HUMAN sp|Q92547|TOPB1\_HUMAN sp|Q8NGN6|O10G7\_HUMAN sp|Q9H8Q6|HEAS1\_HUMAN sp|Q9C073|F117A\_HUMAN tr|D6RHG4|D6RHG4\_HUMAN sp|P31150|GDIA\_HUMAN tr|K4DI93|K4DI93\_HUMAN sp|P12883|MYH7\_HUMAN tr|B4DHB2|B4DHB2\_HUMAN tr|K7ERP6|K7ERP6\_HUMAN sp|Q13425|SNTB2\_HUMAN tr|B5MCM7|B5MCM7\_HUMAN tr|F8WB79|F8WB79\_HUMAN tr|H7C1H1|H7C1H1\_HUMAN sp|Q9BYW1|GTR11\_HUMAN tr|A0A087WTP3|A0A087WTP3\_HUMAN sp|Q92945|FUBP2\_HUMAN tr|M0R0I5|M0R0I5\_HUMAN sp|Q99594|TEAD3\_HUMAN tr|B5MCM0|B5MCM0\_HUMAN sp|Q14165|MLEC\_HUMAN tr|C9JB06|C9JB06\_HUMAN tr|E5RFX7|E5RFX7\_HUMAN tr|F5GX14|F5GX14\_HUMAN tr|C9JRH1|C9JRH1\_HUMAN sp|Q8N5I4|DHRSX\_HUMAN sp|O94903|PROSC\_HUMAN tr|Q5T454|Q5T454\_HUMAN tr|K7EJ43|K7EJ43\_HUMAN sp|Q9NZL9|MAT2B\_HUMAN tr|E9PKL6|E9PKL6\_HUMAN sp|Q9NV72|ZN701\_HUMAN tr|C9JM20|C9JM20\_HUMAN sp|Q9Y4C4|MFHA1\_HUMAN tr|M0R085|M0R085\_HUMAN tr|H0Y577|H0Y577\_HUMAN sp|P21246|PTN\_HUMAN tr|D1MPS5|D1MPS5\_HUMAN sp|Q12799|TCP10\_HUMAN tr|M0QZ18|M0QZ18\_HUMAN tr|A0A087X213|A0A087X213\_HUMAN tr|A0A087WTQ2|A0A087WTQ2\_HUMAN sp|Q8TF21|ANR24\_HUMAN sp|Q6ZUF6|NC336\_HUMAN sp|Q9C0H5|RHG39\_HUMAN tr|G5E9G9|G5E9G9\_HUMAN sp|Q9GZS1|RPA49\_HUMAN tr|E7ENE5|E7ENE5\_HUMAN tr|E7EX70|E7EX70\_HUMAN tr|F8WBG9|F8WBG9\_HUMAN tr|E5RJL1|E5RJL1\_HUMAN tr|K7EL87|K7EL87\_HUMAN sp|Q99884|SC6A7\_HUMAN tr|F5H6L3|F5H6L3\_HUMAN tr|E5RGZ4|E5RGZ4\_HUMAN tr|K7EKN2|K7EKN2\_HUMAN tr|D6RBE5|D6RBE5\_HUMAN sp|P28370|SMCA1\_HUMAN tr|K7EPM1|K7EPM1\_HUMAN sp|Q9H489|TSY26\_HUMAN tr|Q495P1|Q495P1\_HUMAN tr|D3DVN5|D3DVN5\_HUMAN sp|P13929|ENOB\_HUMAN tr|E7ETE6|E7ETE6\_HUMAN tr|F5H836|F5H836\_HUMAN sp|Q8NHQ1|CEP70\_HUMAN tr|A8MZ31|A8MZ31\_HUMAN tr|C9J3F5|C9J3F5\_HUMAN sp|Q8NGD2|OR4K2\_HUMAN tr|C9JJ39|C9JJ39\_HUMAN sp|Q9UMY4|SNX12\_HUMAN sp|O95772|MENTO\_HUMAN tr|A0A087X0R6|A0A087X0R6\_HUMAN tr|C9JPX5|C9JPX5\_HUMAN tr|C9JKL2|C9JKL2\_HUMAN tr|U3KQS6|U3KQS6\_HUMAN tr|K7ERQ9|K7ERQ9\_HUMAN sp|O00458|IFRD1\_HUMAN tr|H0YH91|H0YH91\_HUMAN tr|A0A087WSX0|A0A087WSX0\_HUMAN tr|H0YGF0|H0YGF0\_HUMAN tr|H7C3R1|H7C3R1\_HUMAN sp|P49116|NR2C2\_HUMAN sp|Q8IYF3|TEX11\_HUMAN tr|H0YFE6|H0YFE6\_HUMAN sp|Q6IQ55|TTBK2\_HUMAN sp|Q71F23|CENPU\_HUMAN tr|C9J381|C9J381\_HUMAN sp|Q9P2H5|UBP35\_HUMAN sp|P20839|IMDH1\_HUMAN sp|Q12837|PO4F2\_HUMAN sp|P55773|CCL23\_HUMAN tr|F8WF55|F8WF55\_HUMAN tr|F8WD82|F8WD82\_HUMAN sp|P11766|ADHX\_HUMAN tr|H7C2Z8|H7C2Z8\_HUMAN tr|H0Y5M1|H0Y5M1\_HUMAN tr|H0Y5P9|H0Y5P9\_HUMAN tr|H7C3K8|H7C3K8\_HUMAN sp|O43638|FOXS1\_HUMAN sp|Q9HAB3|S52A2\_HUMAN tr|F8VWT8|F8VWT8\_HUMAN tr|E7EQY4|E7EQY4\_HUMAN tr|K7ELR4|K7ELR4\_HUMAN tr|F8VS45|F8VS45\_HUMAN tr|E9PDM0|E9PDM0\_HUMAN tr|M0R0Z5|M0R0Z5\_HUMAN sp|P31153|METK2\_HUMAN sp|Q96MN9|ZN488\_HUMAN tr|J3QLW1|J3QLW1\_HUMAN sp|O95754|SEM4F\_HUMAN tr|A0A087WYZ7|A0A087WYZ7\_HUMAN tr|M0R028|M0R028\_HUMAN tr|B7ZKR5|B7ZKR5\_HUMAN tr|H0YJ47|H0YJ47\_HUMAN tr|H7BXG1|H7BXG1\_HUMAN tr|G3V341|G3V341\_HUMAN tr|M0QZM3|M0QZM3\_HUMAN tr|Q3MIM8|Q3MIM8\_HUMAN tr|H7C4R2|H7C4R2\_HUMAN sp|O75503|CLN5\_HUMAN sp|Q9UGV2|NDRG3\_HUMAN sp|Q9UNI6|DUS12\_HUMAN tr|F8WBF9|F8WBF9\_HUMAN tr|H0YHB5|H0YHB5\_HUMAN tr|H3BNL2|H3BNL2\_HUMAN tr|H0YHN6|H0YHN6\_HUMAN tr|H0YHM3|H0YHM3\_HUMAN tr|E9PDI2|E9PDI2\_HUMAN tr|I3L188|I3L188\_HUMAN sp|Q9UKG9|OCTC\_HUMAN sp|O60359|CCG3\_HUMAN sp|P61968|LMO4\_HUMAN tr|E7ER08|E7ER08\_HUMAN sp|Q15742|NAB2\_HUMAN tr|H0Y4Z2|H0Y4Z2\_HUMAN tr|D6RF49|D6RF49\_HUMAN tr|D6RIF8|D6RIF8\_HUMAN tr|D6RIS0|D6RIS0\_HUMAN tr|D6RAF3|D6RAF3\_HUMAN sp|Q03519|TAP2\_HUMAN tr|H0YC50|H0YC50\_HUMAN sp|Q96G91|P2Y11\_HUMAN tr|A0A087WYD6|A0A087WYD6\_HUMAN tr|H0Y835|H0Y835\_HUMAN tr|B5MC74|B5MC74\_HUMAN sp|P15514|AREG\_HUMAN tr|D6RFH2|D6RFH2\_HUMAN tr|B3KSW4|B3KSW4\_HUMAN sp|O00522|KRIT1\_HUMAN tr|H7C1J1|H7C1J1\_HUMAN sp|P58658|EVA1C\_HUMAN tr|D6RAV0|D6RAV0\_HUMAN tr|H0YCN3|H0YCN3\_HUMAN tr|H7C3P8|H7C3P8\_HUMAN tr|D6R9V8|D6R9V8\_HUMAN sp|Q6ZMY9|ZN517\_HUMAN sp|Q8WYQ5|DGCR8\_HUMAN tr|H0Y549|H0Y549\_HUMAN tr|H7C1X0|H7C1X0\_HUMAN tr|E9PII3|E9PII3\_HUMAN sp|O43491|E41L2\_HUMAN tr|E9PK52|E9PK52\_HUMAN tr|E9PHY5|E9PHY5\_HUMAN tr|Q5TI72|Q5TI72\_HUMAN tr|I3L0I5|I3L0I5\_HUMAN tr|Q5TI74|Q5TI74\_HUMAN sp|F8W1W9|NPIB9\_HUMAN sp|P49796|RGS3\_HUMAN sp|Q6ZS10|CL17A\_HUMAN tr|Q5T508|Q5T508\_HUMAN sp|Q16619|CTF1\_HUMAN sp|Q8N573|OXR1\_HUMAN tr|U3KPV2|U3KPV2\_HUMAN tr|E9PKY3|E9PKY3\_HUMAN sp|P49619|DGKG\_HUMAN sp|Q8NBF1|GLIS1\_HUMAN sp|Q96E39|RMXL1\_HUMAN tr|H0YDG6|H0YDG6\_HUMAN tr|H0YCY0|H0YCY0\_HUMAN sp|Q8WXK1|ASB15\_HUMAN tr|C9K086|C9K086\_HUMAN tr|B4DKD5|B4DKD5\_HUMAN tr|A0A087WXT7|A0A087WXT7\_HUMAN tr|C9JTA2|C9JTA2\_HUMAN tr|E9PL37|E9PL37\_HUMAN tr|C9JIR0|C9JIR0\_HUMAN sp|Q7Z6M4|MTEF4\_HUMAN tr|H0Y6T6|H0Y6T6\_HUMAN tr|H7C072|H7C072\_HUMAN tr|B1AKI2|B1AKI2\_HUMAN sp|Q8IXM6|NRM\_HUMAN sp|A8MTL0|IQCF5\_HUMAN tr|E7ER27|E7ER27\_HUMAN tr|F8WCT4|F8WCT4\_HUMAN sp|Q9Y3Y4|PYGO1\_HUMAN tr|C9JRD2|C9JRD2\_HUMAN tr|F5H038|F5H038\_HUMAN tr|E9PMT9|E9PMT9\_HUMAN sp|Q8NBA8|DTWD2\_HUMAN sp|Q6ZT83|YR005\_HUMAN sp|Q29980|MICB\_HUMAN tr|M0R3C9|M0R3C9\_HUMAN sp|P50440|GATM\_HUMAN tr|H3BRU6|H3BRU6\_HUMAN tr|G3V3Y4|G3V3Y4\_HUMAN tr|H7C042|H7C042\_HUMAN tr|F8W922|F8W922\_HUMAN sp|Q9NQX0|PRDM6\_HUMAN sp|Q9UBJ2|ABCD2\_HUMAN sp|Q13670|PM2PB\_HUMAN sp|Q8N5J4|SPIC\_HUMAN sp|Q6ZV65|FA47E\_HUMAN tr|A0A087X0I8|A0A087X0I8\_HUMAN tr|J3QQU7|J3QQU7\_HUMAN tr|I3NI53|I3NI53\_HUMAN tr|H0YFH9|H0YFH9\_HUMAN tr|H0Y593|H0Y593\_HUMAN tr|I3L2W5|I3L2W5\_HUMAN sp|Q9BYV1|AGT2\_HUMAN tr|H0Y7H4|H0Y7H4\_HUMAN tr|E9PQC8|E9PQC8\_HUMAN tr|E9PR23|E9PR23\_HUMAN tr|A0A088AWP6|A0A088AWP6\_HUMAN tr|Q5QPP3|Q5QPP3\_HUMAN tr|Q6ZWB5|Q6ZWB5\_HUMAN sp|Q14376|GALE\_HUMAN tr|Q5QPP1|Q5QPP1\_HUMAN tr|Q5QPP4|Q5QPP4\_HUMAN tr|E9PNU3|E9PNU3\_HUMAN sp|Q71H61|ILDR2\_HUMAN tr|Q5JVA3|Q5JVA3\_HUMAN tr|E9PRA4|E9PRA4\_HUMAN tr|F5H5G4|F5H5G4\_HUMAN tr|A8MRA7|A8MRA7\_HUMAN tr|H7C4D0|H7C4D0\_HUMAN sp|Q9BXU1|STK31\_HUMAN tr|B4DDG0|B4DDG0\_HUMAN tr|B4E171|B4E171\_HUMAN tr|C9J6L6|C9J6L6\_HUMAN sp|P41732|TSN7\_HUMAN sp|Q6ZMV5|SMEK3\_HUMAN sp|O43617|TPPC3\_HUMAN tr|D6RBN1|D6RBN1\_HUMAN tr|E5RHR0|E5RHR0\_HUMAN sp|Q8N5W9|F101B\_HUMAN tr|E5RH05|E5RH05\_HUMAN tr|A0A087WWM0|A0A087WWM0\_HUMAN sp|P20073|ANXA7\_HUMAN sp|P06401|PRGR\_HUMAN sp|Q70EL3|UBP50\_HUMAN tr|E7ETI1|E7ETI1\_HUMAN tr|H7BZD7|H7BZD7\_HUMAN tr|E9PP54|E9PP54\_HUMAN sp|Q9HD15|SRA1\_HUMAN tr|H7C228|H7C228\_HUMAN sp|Q01650|LAT1\_HUMAN tr|H3BU62|H3BU62\_HUMAN sp|P49760|CLK2\_HUMAN sp|Q8NGH9|O52E4\_HUMAN sp|Q9HAZ1|CLK4\_HUMAN tr|B1AVT0|B1AVT0\_HUMAN sp|Q9Y4G8|RPGF2\_HUMAN sp|Q9H2J1|CI037\_HUMAN sp|O75564|JERKY\_HUMAN sp|Q96B01|R51A1\_HUMAN tr|C9JMQ4|C9JMQ4\_HUMAN sp|Q9UGI9|AAKG3\_HUMAN sp|Q92664|TF3A\_HUMAN sp|Q9HAW8|UD110\_HUMAN tr|A8MUT6|A8MUT6\_HUMAN tr|C9JKK0|C9JKK0\_HUMAN sp|P78344|IF4G2\_HUMAN tr|H3BLY2|H3BLY2\_HUMAN tr|G5E943|G5E943\_HUMAN sp|Q9Y3F1|TA6P\_HUMAN sp|Q9UKF2|ADA30\_HUMAN tr|D6RCM1|D6RCM1\_HUMAN tr|A0A087WU91|A0A087WU91\_HUMAN tr|D6RA45|D6RA45\_HUMAN sp|Q06330|SUH\_HUMAN tr|D6R927|D6R927\_HUMAN sp|P11277|SPTB1\_HUMAN sp|P58512|CU067\_HUMAN sp|P40126|TYRP2\_HUMAN sp|Q9UJW2|TINAG\_HUMAN tr|D6RD20|D6RD20\_HUMAN sp|Q07002|CDK18\_HUMAN tr|H7C437|H7C437\_HUMAN tr|D6RA44|D6RA44\_HUMAN tr|H0YJG7|H0YJG7\_HUMAN tr|D6RJC2|D6RJC2\_HUMAN sp|P30307|MPIP3\_HUMAN tr|C9J3Y6|C9J3Y6\_HUMAN tr|H7C3L6|H7C3L6\_HUMAN sp|Q8NH40|OR6S1\_HUMAN sp|Q9BXG8|SPZ1\_HUMAN tr|C9K0C5|C9K0C5\_HUMAN tr|H0YHZ8|H0YHZ8\_HUMAN tr|E9PPQ4|E9PPQ4\_HUMAN tr|C9IYB9|C9IYB9\_HUMAN tr|C9JP72|C9JP72\_HUMAN sp|Q05996|ZP2\_HUMAN tr|M0QZY3|M0QZY3\_HUMAN tr|M0R2E2|M0R2E2\_HUMAN sp|O95841|ANGL1\_HUMAN tr|E9PJR2|E9PJR2\_HUMAN sp|Q8IYE0|CC146\_HUMAN sp|Q9UL33|TPC2L\_HUMAN tr|H3BP13|H3BP13\_HUMAN tr|C9JYG3|C9JYG3\_HUMAN sp|O43581|SYT7\_HUMAN tr|I3L3P4|I3L3P4\_HUMAN sp|P01825|HV207\_HUMAN sp|Q6UXN8|CLC9A\_HUMAN sp|O00519|FAAH1\_HUMAN tr|I3L539|I3L539\_HUMAN tr|A0A087WY04|A0A087WY04\_HUMAN tr|Q5VZW3|Q5VZW3\_HUMAN sp|Q16281|CNGA3\_HUMAN tr|I3L0S4|I3L0S4\_HUMAN sp|Q8IWP9|CC28A\_HUMAN sp|Q9UQ72|PSG11\_HUMAN tr|E9PCK9|E9PCK9\_HUMAN sp|O75154|RFIP3\_HUMAN sp|Q9HCQ7|NPVF\_HUMAN tr|H7C0N9|H7C0N9\_HUMAN tr|F8WEA4|F8WEA4\_HUMAN sp|Q9NR82|KCNQ5\_HUMAN sp|Q9UKU0|ACSL6\_HUMAN sp|P28347|TEAD1\_HUMAN tr|B5MC47|B5MC47\_HUMAN tr|E9PKB7|E9PKB7\_HUMAN tr|H0YE88|H0YE88\_HUMAN tr|H0YEJ9|H0YEJ9\_HUMAN tr|J3KP52|J3KP52\_HUMAN tr|A0A087X0U5|A0A087X0U5\_HUMAN tr|F8WAD5|F8WAD5\_HUMAN sp|Q8N769|CN178\_HUMAN sp|Q4VC12|MSS51\_HUMAN sp|Q9Y6R1|S4A4\_HUMAN sp|P48448|AL3B2\_HUMAN tr|M0R148|M0R148\_HUMAN tr|K7EJM7|K7EJM7\_HUMAN sp|Q8TE23|TS1R2\_HUMAN sp|Q9NTQ9|CXB4\_HUMAN sp|P0CG40|SP9\_HUMAN sp|Q9H6D3|XKR8\_HUMAN tr|R4GNH7|R4GNH7\_HUMAN sp|P50452|SPB8\_HUMAN tr|C9JVA8|C9JVA8\_HUMAN tr|H7C2K2|H7C2K2\_HUMAN tr|K7ERP5|K7ERP5\_HUMAN tr|C9JTJ8|C9JTJ8\_HUMAN tr|K7EMH5|K7EMH5\_HUMAN sp|O94887|FARP2\_HUMAN tr|H7BZT5|H7BZT5\_HUMAN tr|E5RFR0|E5RFR0\_HUMAN P01966 sp|Q53G59|KLH12\_HUMAN tr|A0A087WVR3|A0A087WVR3\_HUMAN sp|Q96T88|UHRF1\_HUMAN tr|A0A087WTW0|A0A087WTW0\_HUMAN sp|Q8N9M5|TM102\_HUMAN sp|Q6NUN7|CK063\_HUMAN tr|Q6ZV33|Q6ZV33\_HUMAN tr|A0A087WUL4|A0A087WUL4\_HUMAN tr|H0YBG6|H0YBG6\_HUMAN tr|H0YL60|H0YL60\_HUMAN sp|A6NGC4|TLCD2\_HUMAN sp|P49770|EI2BB\_HUMAN sp|P0C7M8|CLC2L\_HUMAN tr|H0YJJ8|H0YJJ8\_HUMAN tr|H0YK53|H0YK53\_HUMAN tr|G3V5E5|G3V5E5\_HUMAN sp|Q6ZQX7|CQ097\_HUMAN sp|Q9H3T3|SEM6B\_HUMAN tr|Q5SYW2|Q5SYW2\_HUMAN sp|Q96JM4|LRIQ1\_HUMAN sp|Q9H706|GAREM\_HUMAN sp|Q19AV6|ZSWM7\_HUMAN tr|M0QXW0|M0QXW0\_HUMAN tr|K7ER25|K7ER25\_HUMAN sp|Q9H5L6|THAP9\_HUMAN tr|J3QQW8|J3QQW8\_HUMAN sp|P23677|IP3KA\_HUMAN sp|Q8TBH0|ARRD2\_HUMAN sp|Q9H3T2|SEM6C\_HUMAN tr|J3KPA4|J3KPA4\_HUMAN tr|H0YCE3|H0YCE3\_HUMAN tr|F8VY40|F8VY40\_HUMAN tr|F8W1Z5|F8W1Z5\_HUMAN tr|C9JUC3|C9JUC3\_HUMAN tr|C9J7N0|C9J7N0\_HUMAN tr|C9JHH5|C9JHH5\_HUMAN tr|C9JXQ7|C9JXQ7\_HUMAN sp|Q9Y6I9|TX264\_HUMAN sp|Q6UY14|ATL4\_HUMAN tr|J3QKT3|J3QKT3\_HUMAN tr|J3KTA8|J3KTA8\_HUMAN sp|Q8NGZ3|O13G1\_HUMAN tr|J3QLK9|J3QLK9\_HUMAN tr|J3QL23|J3QL23\_HUMAN tr|J3KSA9|J3KSA9\_HUMAN sp|Q9H9D4|ZN408\_HUMAN tr|J3QRZ0|J3QRZ0\_HUMAN tr|H3BP51|H3BP51\_HUMAN sp|P53671|LIMK2\_HUMAN tr|C9JWE6|C9JWE6\_HUMAN tr|H7C576|H7C576\_HUMAN sp|Q96EY1|DNJA3\_HUMAN tr|H0YK32|H0YK32\_HUMAN sp|Q658P3|STEA3\_HUMAN tr|B8ZZX6|B8ZZX6\_HUMAN tr|H0YFD4|H0YFD4\_HUMAN tr|J3QRJ8|J3QRJ8\_HUMAN tr|J3KSM5|J3KSM5\_HUMAN tr|H7C4I5|H7C4I5\_HUMAN sp|Q9UN79|SOX13\_HUMAN sp|Q9BRT3|MIEN1\_HUMAN tr|J3KTI2|J3KTI2\_HUMAN sp|Q5H9T9|FSCB\_HUMAN sp|P08237|PFKAM\_HUMAN tr|C9JWI2|C9JWI2\_HUMAN tr|G3V544|G3V544\_HUMAN tr|F5H3K2|F5H3K2\_HUMAN sp|Q8TDB6|DTX3L\_HUMAN tr|G3V5R8|G3V5R8\_HUMAN sp|P0C7T8|TM253\_HUMAN tr|E7ET33|E7ET33\_HUMAN tr|G3V387|G3V387\_HUMAN tr|A0A087WU42|A0A087WU42\_HUMAN tr|A0A087X2A1|A0A087X2A1\_HUMAN tr|H7BZH7|H7BZH7\_HUMAN sp|P31151|S10A7\_HUMAN sp|Q86SG5|S1A7A\_HUMAN tr|H7BZ51|H7BZ51\_HUMAN sp|O43292|GPAA1\_HUMAN tr|M0QZT3|M0QZT3\_HUMAN tr|H7C326|H7C326\_HUMAN sp|Q9Y2Q9|RT28\_HUMAN Q8N1A0 sp|Q8N1A0|KT222\_HUMAN sp|Q8NEV1|CSK23\_HUMAN sp|Q92804|RBP56\_HUMAN sp|P02753|RET4\_HUMAN tr|F5GZN3|F5GZN3\_HUMAN sp|Q8IZ26|ZNF34\_HUMAN tr|E2QRG9|E2QRG9\_HUMAN sp|P61567|ENK7\_HUMAN sp|Q9NQ69|LHX9\_HUMAN tr|E7EVW7|E7EVW7\_HUMAN tr|H7C4A1|H7C4A1\_HUMAN sp|P14317|HCLS1\_HUMAN tr|M0QZ53|M0QZ53\_HUMAN sp|Q9UI47|CTNA3\_HUMAN sp|Q86W10|CP4Z1\_HUMAN sp|Q9Y5I7|CLD16\_HUMAN tr|Q49AQ3|Q49AQ3\_HUMAN sp|Q99683|M3K5\_HUMAN tr|X6RD87|X6RD87\_HUMAN sp|Q01718|ACTHR\_HUMAN sp|Q86XK2|FBX11\_HUMAN tr|I3L412|I3L412\_HUMAN tr|H3BNH2|H3BNH2\_HUMAN sp|Q14641|INSL4\_HUMAN tr|F8W9W2|F8W9W2\_HUMAN tr|E9PJB8|E9PJB8\_HUMAN tr|E9PN30|E9PN30\_HUMAN sp|Q96C34|RUND1\_HUMAN sp|P43250|GRK6\_HUMAN tr|D6RHX8|D6RHX8\_HUMAN sp|Q9BQE4|SELS\_HUMAN sp|Q8N4X5|AF1L2\_HUMAN tr|H7C440|H7C440\_HUMAN sp|Q92889|XPF\_HUMAN tr|E9PFL1|E9PFL1\_HUMAN sp|Q9NXB0|MKS1\_HUMAN tr|F5GZG7|F5GZG7\_HUMAN sp|Q96S65|CSRN1\_HUMAN tr|B0QYY4|B0QYY4\_HUMAN sp|Q9UK32|KS6A6\_HUMAN tr|U3KQP2|U3KQP2\_HUMAN sp|Q8IUR5|TMTC1\_HUMAN sp|Q07283|TRHY\_HUMAN sp|Q8N413|S2545\_HUMAN tr|H0YE05|H0YE05\_HUMAN tr|E9PK53|E9PK53\_HUMAN sp|Q4J6C6|PPCEL\_HUMAN tr|K7ER98|K7ER98\_HUMAN sp|Q8IVU3|HERC6\_HUMAN sp|P46937|YAP1\_HUMAN sp|P86452|ZBED6\_HUMAN tr|F5GYQ7|F5GYQ7\_HUMAN tr|F8WC44|F8WC44\_HUMAN tr|J3QKQ4|J3QKQ4\_HUMAN sp|P0C7I0|U17L8\_HUMAN tr|H0Y5N2|H0Y5N2\_HUMAN tr|J3KRY8|J3KRY8\_HUMAN sp|O43805|SSNA1\_HUMAN sp|P17010|ZFX\_HUMAN tr|G5E9R9|G5E9R9\_HUMAN tr|H0YF57|H0YF57\_HUMAN tr|K7EPK2|K7EPK2\_HUMAN tr|H0YMF3|H0YMF3\_HUMAN tr|F5H6W6|F5H6W6\_HUMAN sp|Q9NPC4|A4GAT\_HUMAN tr|H0Y3W8|H0Y3W8\_HUMAN sp|O94805|ACL6B\_HUMAN tr|H0Y5M8|H0Y5M8\_HUMAN sp|Q9UHX3|EMR2\_HUMAN sp|P16278|BGAL\_HUMAN sp|Q13510|ASAH1\_HUMAN sp|Q9NXK8|FXL12\_HUMAN tr|K7ELS1|K7ELS1\_HUMAN sp|Q96HA8|NTAQ1\_HUMAN tr|F8VR77|F8VR77\_HUMAN tr|A0A087WYX0|A0A087WYX0\_HUMAN sp|Q92903|CDS1\_HUMAN sp|Q5H9L2|TCAL5\_HUMAN sp|Q9H611|PIF1\_HUMAN tr|H3BUV4|H3BUV4\_HUMAN sp|Q9UQ80|PA2G4\_HUMAN sp|Q7L5Y6|DET1\_HUMAN sp|Q9BTV4|TMM43\_HUMAN sp|P20340|RAB6A\_HUMAN tr|C9JDI9|C9JDI9\_HUMAN sp|Q00534|CDK6\_HUMAN tr|D6RG30|D6RG30\_HUMAN sp|Q2TB18|ASTE1\_HUMAN sp|O75525|KHDR3\_HUMAN sp|Q8WXX7|AUTS2\_HUMAN tr|Q75MD7|Q75MD7\_HUMAN sp|A8K979|ERI2\_HUMAN sp|O95461|LARGE\_HUMAN tr|H0YLP3|H0YLP3\_HUMAN sp|P01704|LV201\_HUMAN tr|Q5SSR2|Q5SSR2\_HUMAN sp|Q3SXY8|AR13B\_HUMAN tr|H7C5D6|H7C5D6\_HUMAN sp|Q9H9B1|EHMT1\_HUMAN tr|A0A087WTL9|A0A087WTL9\_HUMAN tr|Q8TBL5|Q8TBL5\_HUMAN tr|U3KQT6|U3KQT6\_HUMAN tr|K7EQ34|K7EQ34\_HUMAN tr|Q96J85|Q96J85\_HUMAN tr|H0YD14|H0YD14\_HUMAN sp|Q15722|LT4R1\_HUMAN sp|Q8N392|RHG18\_HUMAN tr|C9J6W9|C9J6W9\_HUMAN tr|H7BZ54|H7BZ54\_HUMAN sp|Q6P1A2|MBOA5\_HUMAN tr|F5H0M4|F5H0M4\_HUMAN tr|H0YKP9|H0YKP9\_HUMAN sp|Q08AE8|SPIR1\_HUMAN sp|Q8TAA9|VANG1\_HUMAN tr|C9J8R9|C9J8R9\_HUMAN sp|Q8NA42|ZN383\_HUMAN sp|P49795|RGS19\_HUMAN tr|A0A087WTM0|A0A087WTM0\_HUMAN tr|C9JIE2|C9JIE2\_HUMAN sp|Q562R1|ACTBL\_HUMAN tr|H7BXN9|H7BXN9\_HUMAN sp|Q86TI0|TBCD1\_HUMAN sp|Q8TCC7|S22A8\_HUMAN tr|K7EJ96|K7EJ96\_HUMAN tr|H0YEM7|H0YEM7\_HUMAN tr|E5RG33|E5RG33\_HUMAN tr|H0YCL5|H0YCL5\_HUMAN sp|P18850|ATF6A\_HUMAN sp|Q5TYW1|ZN658\_HUMAN tr|G5E9C5|G5E9C5\_HUMAN sp|P52333|JAK3\_HUMAN tr|H7C1V7|H7C1V7\_HUMAN tr|A0A087WVA2|A0A087WVA2\_HUMAN sp|O76074|PDE5A\_HUMAN tr|A0A087WUS4|A0A087WUS4\_HUMAN sp|Q6ZUJ8|BCAP\_HUMAN sp|Q9HAA7|YG046\_HUMAN tr|Q5SZK0|Q5SZK0\_HUMAN tr|A0A087WVG5|A0A087WVG5\_HUMAN tr|A0A096LP10|A0A096LP10\_HUMAN tr|A0A096LNJ4|A0A096LNJ4\_HUMAN sp|Q8N4C9|CQ078\_HUMAN tr|Q5T0V4|Q5T0V4\_HUMAN tr|D6RE84|D6RE84\_HUMAN tr|A0A087WX01|A0A087WX01\_HUMAN tr|A0A087WY28|A0A087WY28\_HUMAN tr|A0A087WX43|A0A087WX43\_HUMAN tr|E9PFD2|E9PFD2\_HUMAN sp|Q9C0C9|UBE2O\_HUMAN tr|X6RDV4|X6RDV4\_HUMAN sp|P20648|ATP4A\_HUMAN tr|H0YGM0|H0YGM0\_HUMAN tr|K7EQH0|K7EQH0\_HUMAN sp|Q06546|GABPA\_HUMAN tr|A0A087X076|A0A087X076\_HUMAN sp|P24534|EF1B\_HUMAN tr|Q5SR26|Q5SR26\_HUMAN tr|Q5JVH2|Q5JVH2\_HUMAN sp|P53618|COPB\_HUMAN tr|Q5TE61|Q5TE61\_HUMAN tr|H0YHM6|H0YHM6\_HUMAN sp|Q7Z404|TMC4\_HUMAN sp|O15482|TEX28\_HUMAN sp|Q9H4W6|COE3\_HUMAN sp|P26992|CNTFR\_HUMAN sp|Q9Y5Z7|HCFC2\_HUMAN sp|Q8NFZ0|FBX18\_HUMAN tr|F6UZG9|F6UZG9\_HUMAN tr|K7EQ65|K7EQ65\_HUMAN sp|Q9NQ36|SCUB2\_HUMAN tr|K7EIS6|K7EIS6\_HUMAN tr|K7EKN1|K7EKN1\_HUMAN tr|A6XMV5|A6XMV5\_HUMAN sp|A1L0T0|ILVBL\_HUMAN sp|Q96CP6|GRM1A\_HUMAN tr|M0QZ12|M0QZ12\_HUMAN tr|D6RFL8|D6RFL8\_HUMAN tr|G3V2Q2|G3V2Q2\_HUMAN sp|Q6PKG0|LARP1\_HUMAN sp|Q2MV58|TECT1\_HUMAN sp|P78524|ST5\_HUMAN tr|B4DDL8|B4DDL8\_HUMAN tr|I3L537|I3L537\_HUMAN sp|Q9NQT6|FSCN3\_HUMAN tr|H7C0H6|H7C0H6\_HUMAN sp|P0CG33|GOG6D\_HUMAN sp|A6NDK9|GOG6C\_HUMAN tr|A0A087WXI9|A0A087WXI9\_HUMAN tr|U3KPX2|U3KPX2\_HUMAN tr|K7EIV7|K7EIV7\_HUMAN sp|Q8NGA5|O10H4\_HUMAN sp|Q14410|GLPK2\_HUMAN sp|Q96MK3|FA20A\_HUMAN sp|Q96BT7|ALKB8\_HUMAN sp|Q96SI1|KCD15\_HUMAN tr|E7ET14|E7ET14\_HUMAN tr|H0YAB3|H0YAB3\_HUMAN tr|A0A087WW48|A0A087WW48\_HUMAN tr|D6RHZ5|D6RHZ5\_HUMAN tr|F6QPS0|F6QPS0\_HUMAN sp|P10109|ADX\_HUMAN tr|B3KSJ7|B3KSJ7\_HUMAN tr|A2BF26|A2BF26\_HUMAN sp|Q9Y696|CLIC4\_HUMAN sp|Q14526|HIC1\_HUMAN tr|B5MCM1|B5MCM1\_HUMAN tr|H0Y4M5|H0Y4M5\_HUMAN tr|F8WCV3|F8WCV3\_HUMAN sp|O43559|FRS3\_HUMAN sp|P23276|KELL\_HUMAN tr|F8WFA8|F8WFA8\_HUMAN sp|Q9Y3A0|COQ4\_HUMAN sp|P48735|IDHP\_HUMAN tr|A0A087WZ07|A0A087WZ07\_HUMAN tr|H0YJ72|H0YJ72\_HUMAN tr|K7ESC4|K7ESC4\_HUMAN sp|Q6UWP8|SBSN\_HUMAN sp|Q8NGL4|OR5DD\_HUMAN sp|Q9UPR3|SMG5\_HUMAN tr|K7EJ21|K7EJ21\_HUMAN sp|Q9YNA8|GAK19\_HUMAN tr|B5MCI6|B5MCI6\_HUMAN tr|E9PDL4|E9PDL4\_HUMAN sp|Q05084|ICA69\_HUMAN tr|A0A087WUU9|A0A087WUU9\_HUMAN tr|B1AMU4|B1AMU4\_HUMAN sp|Q6PF05|TT23L\_HUMAN sp|Q9Y3B2|EXOS1\_HUMAN tr|H0YC89|H0YC89\_HUMAN sp|Q9NYW1|TA2R9\_HUMAN tr|E9PB61|E9PB61\_HUMAN tr|H0Y864|H0Y864\_HUMAN tr|H3BSM2|H3BSM2\_HUMAN tr|Q5T0G3|Q5T0G3\_HUMAN sp|Q9BTT6|LRRC1\_HUMAN sp|Q8NDL9|CBPC5\_HUMAN sp|Q9H0D6|XRN2\_HUMAN sp|O00566|MPP10\_HUMAN sp|Q92844|TANK\_HUMAN tr|E7EQA9|E7EQA9\_HUMAN tr|C9J887|C9J887\_HUMAN tr|E9PPF2|E9PPF2\_HUMAN tr|H7C3L4|H7C3L4\_HUMAN sp|Q99928|GBRG3\_HUMAN tr|E9PJX4|E9PJX4\_HUMAN sp|Q9H1U4|MEGF9\_HUMAN sp|Q8WUA7|TB22A\_HUMAN tr|F8VWL3|F8VWL3\_HUMAN tr|B7Z493|B7Z493\_HUMAN tr|Q68DW7|Q68DW7\_HUMAN tr|D6RID8|D6RID8\_HUMAN sp|Q8N8P7|CK044\_HUMAN tr|B0QYI1|B0QYI1\_HUMAN sp|P49903|SPS1\_HUMAN sp|P30825|CTR1\_HUMAN tr|D6RA20|D6RA20\_HUMAN tr|H7C1R8|H7C1R8\_HUMAN sp|P51888|PRELP\_HUMAN sp|Q9HAT8|PELI2\_HUMAN sp|P10911|MCF2\_HUMAN tr|D6RAF9|D6RAF9\_HUMAN tr|H0YAV1|H0YAV1\_HUMAN sp|Q15612|OR1Q1\_HUMAN sp|Q7LG56|RIR2B\_HUMAN sp|Q6P9F0|CCD62\_HUMAN tr|H7C5D9|H7C5D9\_HUMAN tr|C9JS80|C9JS80\_HUMAN tr|H0YEY1|H0YEY1\_HUMAN tr|K7ERK2|K7ERK2\_HUMAN tr|K7ES91|K7ES91\_HUMAN tr|K7EJU0|K7EJU0\_HUMAN tr|E9PLZ4|E9PLZ4\_HUMAN tr|K7EM03|K7EM03\_HUMAN sp|Q53F39|MPPE1\_HUMAN sp|O14559|RHG33\_HUMAN tr|K7EQV4|K7EQV4\_HUMAN tr|C9WSJ3|C9WSJ3\_HUMAN tr|H0YEA4|H0YEA4\_HUMAN tr|C9J8C2|C9J8C2\_HUMAN tr|K7EQ70|K7EQ70\_HUMAN tr|C9JDP4|C9JDP4\_HUMAN sp|Q9GZK7|O11A1\_HUMAN tr|J3KN81|J3KN81\_HUMAN tr|C9JZ65|C9JZ65\_HUMAN tr|H0Y9J8|H0Y9J8\_HUMAN sp|Q09328|MGT5A\_HUMAN sp|Q8N2G6|ZCH24\_HUMAN tr|G3V499|G3V499\_HUMAN sp|Q96AP4|ZUFSP\_HUMAN sp|P46199|IF2M\_HUMAN tr|B0QZE6|B0QZE6\_HUMAN tr|E9PS59|E9PS59\_HUMAN tr|X6R732|X6R732\_HUMAN sp|Q9NVV0|TM38B\_HUMAN tr|X6RHV1|X6RHV1\_HUMAN tr|A2A370|A2A370\_HUMAN tr|H7BZW4|H7BZW4\_HUMAN sp|P51582|P2RY4\_HUMAN tr|H0YHF4|H0YHF4\_HUMAN sp|Q9UJA9|ENPP5\_HUMAN sp|P49286|MTR1B\_HUMAN sp|O60609|GFRA3\_HUMAN sp|Q8NFU1|BEST2\_HUMAN tr|A0A087X1C3|A0A087X1C3\_HUMAN tr|H7C4H7|H7C4H7\_HUMAN sp|Q16690|DUS5\_HUMAN tr|K7EPL3|K7EPL3\_HUMAN tr|F5H6R8|F5H6R8\_HUMAN tr|J3KSA8|J3KSA8\_HUMAN sp|Q92604|LGAT1\_HUMAN tr|C9JTF8|C9JTF8\_HUMAN tr|H0YFJ7|H0YFJ7\_HUMAN sp|Q9NVU0|RPC5\_HUMAN tr|H7C1U4|H7C1U4\_HUMAN tr|I3L4N7|I3L4N7\_HUMAN tr|F8VVI9|F8VVI9\_HUMAN tr|H3BPZ3|H3BPZ3\_HUMAN tr|J3KTP3|J3KTP3\_HUMAN tr|I3L4F9|I3L4F9\_HUMAN tr|I3L107|I3L107\_HUMAN sp|Q9NZH8|IL36G\_HUMAN tr|B4DVT3|B4DVT3\_HUMAN sp|Q9H4D5|NXF3\_HUMAN sp|Q96SY0|VWA9\_HUMAN tr|B4DJL6|B4DJL6\_HUMAN sp|Q9NZ38|IDAS1\_HUMAN sp|Q7L099|RUFY3\_HUMAN tr|E5RIA9|E5RIA9\_HUMAN sp|P07196|NFL\_HUMAN sp|Q8N2C9|UMAS1\_HUMAN tr|C9J9E4|C9J9E4\_HUMAN tr|C9JJ64|C9JJ64\_HUMAN tr|C9JWF0|C9JWF0\_HUMAN sp|P61575|RECK8\_HUMAN tr|C9IYK2|C9IYK2\_HUMAN tr|E7EM56|E7EM56\_HUMAN sp|Q8NH60|O52J3\_HUMAN tr|Q4G141|Q4G141\_HUMAN sp|Q8NH56|O52N5\_HUMAN sp|Q99541|PLIN2\_HUMAN tr|A0A087X0Y1|A0A087X0Y1\_HUMAN sp|Q5H9J9|T11X2\_HUMAN sp|P56537|IF6\_HUMAN sp|Q86SF2|GALT7\_HUMAN sp|Q7Z3E2|CC186\_HUMAN tr|H7C1D6|H7C1D6\_HUMAN sp|Q9HAE3|EFCB1\_HUMAN tr|H0Y3K7|H0Y3K7\_HUMAN tr|H0YC53|H0YC53\_HUMAN sp|O95136|S1PR2\_HUMAN sp|Q8TEQ0|SNX29\_HUMAN sp|Q12979|ABR\_HUMAN sp|O14531|DPYL4\_HUMAN sp|C9JR72|KBTBD\_HUMAN sp|Q16663|CCL15\_HUMAN sp|Q7M4L6|SHF\_HUMAN tr|F5H3P6|F5H3P6\_HUMAN sp|O75553|DAB1\_HUMAN tr|I3L1M7|I3L1M7\_HUMAN tr|G3V2G7|G3V2G7\_HUMAN sp|Q70UQ0|IKIP\_HUMAN sp|Q07699|SCN1B\_HUMAN tr|B4DI92|B4DI92\_HUMAN sp|Q9ULF5|S39AA\_HUMAN tr|A0A075B6H3|A0A075B6H3\_HUMAN sp|O75078|ADA11\_HUMAN tr|G5E9D8|G5E9D8\_HUMAN sp|Q5TGY1|TMCO4\_HUMAN tr|B4DKD2|B4DKD2\_HUMAN sp|P54868|HMCS2\_HUMAN tr|D6RA51|D6RA51\_HUMAN tr|F2Z2L2|F2Z2L2\_HUMAN tr|E9PD12|E9PD12\_HUMAN tr|H0YAM8|H0YAM8\_HUMAN tr|F5H013|F5H013\_HUMAN tr|D6RAZ0|D6RAZ0\_HUMAN tr|Q49AN9|Q49AN9\_HUMAN sp|Q96AX9|MIB2\_HUMAN sp|P62308|RUXG\_HUMAN sp|Q6ZRP0|PR23C\_HUMAN sp|Q8NGA4|G32P1\_HUMAN tr|B1B0G8|B1B0G8\_HUMAN sp|O15143|ARC1B\_HUMAN tr|C9JWU6|C9JWU6\_HUMAN sp|Q86UA1|PRP39\_HUMAN sp|Q96P66|GP101\_HUMAN sp|O60344|ECE2\_HUMAN tr|E9PPC7|E9PPC7\_HUMAN tr|E9PPM1|E9PPM1\_HUMAN tr|H0Y5G8|H0Y5G8\_HUMAN sp|O43155|FLRT2\_HUMAN tr|J3QR23|J3QR23\_HUMAN tr|E9PMG3|E9PMG3\_HUMAN sp|Q9UHM6|OPN4\_HUMAN tr|E9PMD0|E9PMD0\_HUMAN sp|Q96HW7|INT4\_HUMAN sp|Q96A47|ISL2\_HUMAN tr|Q5JYW1|Q5JYW1\_HUMAN sp|Q86VI3|IQGA3\_HUMAN tr|C9JHC7|C9JHC7\_HUMAN tr|Q2NKQ5|Q2NKQ5\_HUMAN tr|G3V4J5|G3V4J5\_HUMAN sp|Q92567|F168A\_HUMAN sp|Q9Y5E7|PCDB2\_HUMAN tr|F2Z2E2|F2Z2E2\_HUMAN tr|B5MBY2|B5MBY2\_HUMAN tr|B5MCD5|B5MCD5\_HUMAN sp|Q9Y6T7|DGKB\_HUMAN tr|K7EP30|K7EP30\_HUMAN sp|Q9UP65|PA24C\_HUMAN sp|P51636|CAV2\_HUMAN tr|G5E9N6|G5E9N6\_HUMAN sp|Q99470|SDF2\_HUMAN tr|Q5ST79|Q5ST79\_HUMAN tr|E9PKE3|E9PKE3\_HUMAN tr|M0R0K9|M0R0K9\_HUMAN tr|U3KQB4|U3KQB4\_HUMAN tr|E9PGA6|E9PGA6\_HUMAN sp|P24046|GBRR1\_HUMAN sp|Q13263|TIF1B\_HUMAN tr|C9JKA9|C9JKA9\_HUMAN tr|H0YCU5|H0YCU5\_HUMAN sp|Q9Y2R4|DDX52\_HUMAN sp|Q8NGU2|OR9A4\_HUMAN sp|Q96MT3|PRIC1\_HUMAN sp|P17600|SYN1\_HUMAN sp|P98073|ENTK\_HUMAN tr|A0A087WUK1|A0A087WUK1\_HUMAN sp|P62683|GAK21\_HUMAN sp|Q8IUX1|T126B\_HUMAN tr|H7C0J5|H7C0J5\_HUMAN tr|H0Y8A4|H0Y8A4\_HUMAN sp|Q9NXW2|DJB12\_HUMAN tr|J3KPS0|J3KPS0\_HUMAN sp|Q9UP52|TFR2\_HUMAN sp|Q9H8H2|DDX31\_HUMAN sp|Q9ULB5|CADH7\_HUMAN sp|Q96A54|ADR1\_HUMAN tr|C9JNM5|C9JNM5\_HUMAN sp|P78352|DLG4\_HUMAN sp|Q9UBP9|GULP1\_HUMAN sp|Q6ZN19|ZN841\_HUMAN tr|F8W782|F8W782\_HUMAN tr|C9J0W7|C9J0W7\_HUMAN tr|J3KS31|J3KS31\_HUMAN sp|Q13888|TF2H2\_HUMAN tr|A0A087WW33|A0A087WW33\_HUMAN tr|A0A087WU87|A0A087WU87\_HUMAN sp|P30084|ECHM\_HUMAN tr|B6EC88|B6EC88\_HUMAN tr|B3KUF4|B3KUF4\_HUMAN sp|P38435|VKGC\_HUMAN tr|F8WF17|F8WF17\_HUMAN tr|H0Y455|H0Y455\_HUMAN sp|Q96FW1|OTUB1\_HUMAN sp|Q9BSU1|CP070\_HUMAN tr|J3KR44|J3KR44\_HUMAN tr|H3BTW3|H3BTW3\_HUMAN tr|F5GYN4|F5GYN4\_HUMAN tr|F5H6Q1|F5H6Q1\_HUMAN tr|F5GYJ8|F5GYJ8\_HUMAN sp|P30566|PUR8\_HUMAN tr|B1APF6|B1APF6\_HUMAN tr|B1APG3|B1APG3\_HUMAN tr|A0A087WVV9|A0A087WVV9\_HUMAN tr|B1APF9|B1APF9\_HUMAN tr|B1APG0|B1APG0\_HUMAN sp|P22694|KAPCB\_HUMAN tr|A0A096LNY6|A0A096LNY6\_HUMAN tr|B1APG1|B1APG1\_HUMAN sp|Q14916|NPT1\_HUMAN tr|B1APF8|B1APF8\_HUMAN tr|A0A087WVC4|A0A087WVC4\_HUMAN sp|Q8N1Q8|ACO15\_HUMAN tr|B1APG2|B1APG2\_HUMAN tr|B1APF7|B1APF7\_HUMAN tr|H0YJT4|H0YJT4\_HUMAN tr|H7C124|H7C124\_HUMAN tr|D6REW5|D6REW5\_HUMAN tr|Q4VXD4|Q4VXD4\_HUMAN tr|Q504Y1|Q504Y1\_HUMAN sp|O43681|ASNA\_HUMAN tr|A0A087WXS7|A0A087WXS7\_HUMAN tr|F8WEJ5|F8WEJ5\_HUMAN tr|M0R2W8|M0R2W8\_HUMAN tr|M0QYW3|M0QYW3\_HUMAN sp|P00966|ASSY\_HUMAN sp|P52848|NDST1\_HUMAN sp|P61073|CXCR4\_HUMAN tr|E7EVJ3|E7EVJ3\_HUMAN sp|P31269|HXA9\_HUMAN sp|O95096|NKX22\_HUMAN tr|E7EUW9|E7EUW9\_HUMAN sp|O60245|PCDH7\_HUMAN sp|Q9BRR0|ZKSC3\_HUMAN tr|B1ANW7|B1ANW7\_HUMAN sp|Q8N8E3|CE112\_HUMAN tr|F5GYE8|F5GYE8\_HUMAN sp|Q14416|GRM2\_HUMAN tr|H3BTG8|H3BTG8\_HUMAN tr|H7BYW1|H7BYW1\_HUMAN sp|Q8N5S3|CB073\_HUMAN tr|C9JGI3|C9JGI3\_HUMAN tr|F8WBH9|F8WBH9\_HUMAN sp|Q75N90|FBN3\_HUMAN sp|P53602|MVD1\_HUMAN sp|P19971|TYPH\_HUMAN sp|Q9H0C8|ILKAP\_HUMAN tr|B0QYT4|B0QYT4\_HUMAN tr|F8SNU7|F8SNU7\_HUMAN tr|G5E9N5|G5E9N5\_HUMAN tr|H0Y4Q3|H0Y4Q3\_HUMAN tr|E5RJ97|E5RJ97\_HUMAN sp|P46060|RAGP1\_HUMAN sp|Q8N1C3|GBRG1\_HUMAN tr|H7C2I8|H7C2I8\_HUMAN tr|M0QZZ9|M0QZZ9\_HUMAN tr|F6V803|F6V803\_HUMAN sp|Q6ZMR3|LDH6A\_HUMAN tr|H0YE34|H0YE34\_HUMAN tr|D6RIF6|D6RIF6\_HUMAN sp|Q9H3U5|MFSD1\_HUMAN sp|Q9BXI2|ORNT2\_HUMAN sp|Q16445|GBRA6\_HUMAN tr|E7EV53|E7EV53\_HUMAN sp|Q96C24|SYTL4\_HUMAN tr|H0YL25|H0YL25\_HUMAN tr|E9PS71|E9PS71\_HUMAN tr|B7Z8B3|B7Z8B3\_HUMAN tr|E9PRI6|E9PRI6\_HUMAN tr|E9PLU0|E9PLU0\_HUMAN tr|H0YD05|H0YD05\_HUMAN sp|P52655|TF2AA\_HUMAN sp|Q499Z3|SLNL1\_HUMAN sp|Q8WVV9|HNRLL\_HUMAN tr|H0Y360|H0Y360\_HUMAN tr|D6W592|D6W592\_HUMAN tr|B7WPG3|B7WPG3\_HUMAN tr|F5H134|F5H134\_HUMAN tr|F5GYH0|F5GYH0\_HUMAN tr|F5H0V9|F5H0V9\_HUMAN sp|Q5VZI3|CI091\_HUMAN tr|Q5TA01|Q5TA01\_HUMAN tr|H0YEY8|H0YEY8\_HUMAN tr|E5RK99|E5RK99\_HUMAN sp|Q8IVB5|LIX1L\_HUMAN tr|G3V1M7|G3V1M7\_HUMAN tr|E9PI19|E9PI19\_HUMAN tr|D6REA1|D6REA1\_HUMAN sp|Q9H173|SIL1\_HUMAN tr|E5RJ15|E5RJ15\_HUMAN tr|D6RFI6|D6RFI6\_HUMAN tr|D6RAT8|D6RAT8\_HUMAN tr|D6RE06|D6RE06\_HUMAN sp|C9J442|CV046\_HUMAN sp|Q8N5M9|JAGN1\_HUMAN sp|Q9HA64|KT3K\_HUMAN tr|D6RBK1|D6RBK1\_HUMAN sp|C9JN71|ZN878\_HUMAN sp|Q969S8|HDA10\_HUMAN sp|A8MVS1|Z705F\_HUMAN tr|J3KTD2|J3KTD2\_HUMAN tr|J3QSZ3|J3QSZ3\_HUMAN sp|O14929|HAT1\_HUMAN tr|E9PP10|E9PP10\_HUMAN sp|Q9BUG6|ZSA5A\_HUMAN sp|O75446|SAP30\_HUMAN tr|D6RIE9|D6RIE9\_HUMAN sp|Q02410|APBA1\_HUMAN sp|P56705|WNT4\_HUMAN sp|P18615|NELFE\_HUMAN tr|E9PD43|E9PD43\_HUMAN tr|M0QXL1|M0QXL1\_HUMAN sp|P83105|HTRA4\_HUMAN tr|E5RI41|E5RI41\_HUMAN tr|E5RHW3|E5RHW3\_HUMAN tr|H0YH81|H0YH81\_HUMAN sp|P34810|CD68\_HUMAN sp|Q6EKJ0|GTD2B\_HUMAN sp|Q86UP8|GTD2A\_HUMAN sp|Q06278|AOXA\_HUMAN tr|F5GZB4|F5GZB4\_HUMAN sp|B1ATL7|PRR32\_HUMAN tr|H0YJ46|H0YJ46\_HUMAN ENSEMBL:ENSBTAP00000034412 tr|B7Z8Q8|B7Z8Q8\_HUMAN sp|Q7Z6L0|PRRT2\_HUMAN sp|Q96DT0|LEG12\_HUMAN tr|G8JLA1|G8JLA1\_HUMAN tr|H3BU37|H3BU37\_HUMAN tr|A0A087WWJ1|A0A087WWJ1\_HUMAN tr|H3BMU3|H3BMU3\_HUMAN tr|A0A087WZ63|A0A087WZ63\_HUMAN tr|C9JNE5|C9JNE5\_HUMAN sp|Q9UF12|PROD2\_HUMAN tr|A0A087WXI4|A0A087WXI4\_HUMAN tr|C9K0D3|C9K0D3\_HUMAN sp|Q14152|EIF3A\_HUMAN tr|C9K0D4|C9K0D4\_HUMAN sp|O60268|K0513\_HUMAN tr|C9J3M3|C9J3M3\_HUMAN sp|Q5JNZ3|ZN311\_HUMAN tr|J3QKK8|J3QKK8\_HUMAN tr|A8MT18|A8MT18\_HUMAN sp|P04003|C4BPA\_HUMAN tr|F8WCL7|F8WCL7\_HUMAN tr|D6RBB3|D6RBB3\_HUMAN sp|Q9C086|IN80B\_HUMAN tr|I3L356|I3L356\_HUMAN sp|P49788|TIG1\_HUMAN sp|Q9HAU5|RENT2\_HUMAN tr|D6RC05|D6RC05\_HUMAN tr|B8ZZH7|B8ZZH7\_HUMAN tr|J3KQ70|J3KQ70\_HUMAN tr|H7BYW9|H7BYW9\_HUMAN tr|D6RDH9|D6RDH9\_HUMAN sp|Q1ZYL8|IZUM4\_HUMAN tr|E9PLV8|E9PLV8\_HUMAN sp|P43355|MAGA1\_HUMAN tr|E9PP94|E9PP94\_HUMAN tr|E9PK90|E9PK90\_HUMAN sp|Q6DHV7|ADAL\_HUMAN tr|F5GY04|F5GY04\_HUMAN sp|Q01954|BNC1\_HUMAN sp|P29033|CXB2\_HUMAN tr|A0A087WUP5|A0A087WUP5\_HUMAN tr|E9PFV2|E9PFV2\_HUMAN sp|Q4G0S4|C27C1\_HUMAN tr|H7BXI0|H7BXI0\_HUMAN sp|O94953|KDM4B\_HUMAN sp|P37231|PPARG\_HUMAN tr|F5GX28|F5GX28\_HUMAN tr|Q5T985|Q5T985\_HUMAN tr|E9PND1|E9PND1\_HUMAN tr|E5RHD2|E5RHD2\_HUMAN sp|Q9UFV3|YO007\_HUMAN tr|Q6ZYK7|Q6ZYK7\_HUMAN sp|Q6P1J9|CDC73\_HUMAN sp|O75056|SDC3\_HUMAN sp|P35453|HXD13\_HUMAN tr|H7BZQ5|H7BZQ5\_HUMAN tr|E9PGA7|E9PGA7\_HUMAN sp|Q15582|BGH3\_HUMAN sp|Q8WV83|S35F5\_HUMAN sp|Q8WVV4|POF1B\_HUMAN tr|Q5TBP9|Q5TBP9\_HUMAN sp|Q13258|PD2R\_HUMAN tr|A0A087WWR3|A0A087WWR3\_HUMAN tr|F5GYK3|F5GYK3\_HUMAN tr|J3KTC8|J3KTC8\_HUMAN sp|Q7Z5J1|DHI1L\_HUMAN tr|G3V2V3|G3V2V3\_HUMAN tr|G3V2M9|G3V2M9\_HUMAN tr|C9J524|C9J524\_HUMAN tr|G3V4L2|G3V4L2\_HUMAN sp|O60291|MGRN1\_HUMAN tr|K7EPJ5|K7EPJ5\_HUMAN sp|Q9UIJ5|ZDHC2\_HUMAN sp|Q8NGX8|OR6Y1\_HUMAN tr|G5E9W8|G5E9W8\_HUMAN tr|H0Y3T7|H0Y3T7\_HUMAN sp|P08588|ADRB1\_HUMAN tr|M0R308|M0R308\_HUMAN tr|H7BXF6|H7BXF6\_HUMAN tr|X6RGF2|X6RGF2\_HUMAN tr|X6RHN7|X6RHN7\_HUMAN tr|X6RH50|X6RH50\_HUMAN tr|H3BRJ9|H3BRJ9\_HUMAN tr|H0YF49|H0YF49\_HUMAN tr|E9PJG8|E9PJG8\_HUMAN tr|K7EJ37|K7EJ37\_HUMAN sp|Q6ZS02|DMP46\_HUMAN tr|H0Y2Z1|H0Y2Z1\_HUMAN tr|E9PJ34|E9PJ34\_HUMAN tr|E9PSH6|E9PSH6\_HUMAN sp|P06746|DPOLB\_HUMAN tr|A8MXR3|A8MXR3\_HUMAN tr|B1AKQ8|B1AKQ8\_HUMAN tr|D6RD67|D6RD67\_HUMAN tr|C9JZN1|C9JZN1\_HUMAN tr|E7EQ52|E7EQ52\_HUMAN tr|J3QKZ5|J3QKZ5\_HUMAN sp|Q7Z591|AKNA\_HUMAN tr|J3QS16|J3QS16\_HUMAN tr|K7END7|K7END7\_HUMAN tr|M0R1U8|M0R1U8\_HUMAN sp|Q9UKK6|NXT1\_HUMAN tr|M0QZ34|M0QZ34\_HUMAN sp|Q9NZM5|GSCR2\_HUMAN sp|Q8NGB8|O4F15\_HUMAN tr|M0QZU5|M0QZU5\_HUMAN tr|H0YCY1|H0YCY1\_HUMAN tr|K7ER53|K7ER53\_HUMAN tr|J3KN13|J3KN13\_HUMAN tr|H0YCP4|H0YCP4\_HUMAN tr|G3XAD9|G3XAD9\_HUMAN sp|Q15669|RHOH\_HUMAN sp|Q9H845|ACAD9\_HUMAN tr|H0YAJ8|H0YAJ8\_HUMAN sp|Q9NR45|SIAS\_HUMAN sp|P54578|UBP14\_HUMAN sp|Q96PE3|INP4A\_HUMAN tr|E7EPS8|E7EPS8\_HUMAN tr|J3KMY6|J3KMY6\_HUMAN sp|Q8IV31|TM139\_HUMAN sp|A6NCF5|KLH33\_HUMAN tr|C9JLM1|C9JLM1\_HUMAN sp|Q8NGI7|O10V1\_HUMAN sp|Q6AWC8|YK026\_HUMAN sp|A5PLL1|AN34B\_HUMAN sp|Q15532|SSXT\_HUMAN tr|B4DLD3|B4DLD3\_HUMAN sp|O15040|TCPR2\_HUMAN sp|Q9BWU0|NADAP\_HUMAN sp|Q8N1G4|LRC47\_HUMAN tr|A0A087WXC3|A0A087WXC3\_HUMAN sp|P30542|AA1R\_HUMAN tr|E9PJX2|E9PJX2\_HUMAN tr|A0A087WTU5|A0A087WTU5\_HUMAN tr|A0A087WX49|A0A087WX49\_HUMAN sp|Q96MY1|NOL4L\_HUMAN tr|A0A087WWT5|A0A087WWT5\_HUMAN tr|A0A087WYN7|A0A087WYN7\_HUMAN tr|A0A087WWC9|A0A087WWC9\_HUMAN tr|A0A087WWU7|A0A087WWU7\_HUMAN tr|C9JUN4|C9JUN4\_HUMAN sp|Q6UX06|OLFM4\_HUMAN tr|R4GMW4|R4GMW4\_HUMAN tr|B4DP31|B4DP31\_HUMAN tr|C9J168|C9J168\_HUMAN sp|Q05329|DCE2\_HUMAN tr|F8W6L8|F8W6L8\_HUMAN tr|E9PC35|E9PC35\_HUMAN sp|Q14558|KPRA\_HUMAN tr|H0YCQ7|H0YCQ7\_HUMAN sp|Q6IFH4|OR6B2\_HUMAN sp|P49682|CXCR3\_HUMAN sp|Q7Z4F1|LRP10\_HUMAN sp|Q99676|ZN184\_HUMAN tr|G3V167|G3V167\_HUMAN sp|Q9UGN5|PARP2\_HUMAN tr|C9J977|C9J977\_HUMAN tr|E9PK21|E9PK21\_HUMAN tr|B5MC96|B5MC96\_HUMAN tr|Q5T8M8|Q5T8M8\_HUMAN tr|Q5T8M7|Q5T8M7\_HUMAN sp|P68133|ACTS\_HUMAN tr|A6NL76|A6NL76\_HUMAN tr|B0AZS6|B0AZS6\_HUMAN tr|X6RBZ7|X6RBZ7\_HUMAN tr|E5RK87|E5RK87\_HUMAN sp|P29376|LTK\_HUMAN sp|P31946|1433B\_HUMAN sp|Q96S94|CCNL2\_HUMAN tr|H0YB80|H0YB80\_HUMAN sp|Q9H2U9|ADAM7\_HUMAN tr|K7EMP1|K7EMP1\_HUMAN sp|A8MU46|SMTL1\_HUMAN sp|P22680|CP7A1\_HUMAN tr|H7C240|H7C240\_HUMAN tr|E9PPJ3|E9PPJ3\_HUMAN tr|B3KQ23|B3KQ23\_HUMAN sp|Q9BSD7|NTPCR\_HUMAN tr|Q5TDF0|Q5TDF0\_HUMAN sp|Q9Y5X5|NPFF2\_HUMAN tr|H0YJ31|H0YJ31\_HUMAN tr|J3KNC7|J3KNC7\_HUMAN sp|P00167|CYB5\_HUMAN sp|Q8TBZ5|ZN502\_HUMAN tr|E9PNU6|E9PNU6\_HUMAN sp|Q5JVG8|ZN506\_HUMAN sp|P17019|ZN708\_HUMAN sp|Q96NG5|ZN558\_HUMAN sp|Q86W26|NAL10\_HUMAN tr|E9PMS0|E9PMS0\_HUMAN tr|E9PSD1|E9PSD1\_HUMAN sp|Q5SXM1|ZN678\_HUMAN tr|E9PMZ6|E9PMZ6\_HUMAN tr|D6RAM3|D6RAM3\_HUMAN tr|Q5TE25|Q5TE25\_HUMAN tr|D6RC22|D6RC22\_HUMAN tr|D6R977|D6R977\_HUMAN sp|Q7L591|DOK3\_HUMAN sp|P28845|DHI1\_HUMAN sp|Q9C026|TRIM9\_HUMAN tr|A0A087WU76|A0A087WU76\_HUMAN tr|M0QYR3|M0QYR3\_HUMAN tr|D6RH03|D6RH03\_HUMAN tr|A2RRE0|A2RRE0\_HUMAN tr|H0Y8Z8|H0Y8Z8\_HUMAN sp|Q8NG76|O2T33\_HUMAN tr|H3BLT9|H3BLT9\_HUMAN sp|Q7RTR2|NLRC3\_HUMAN tr|Q3SAH0|Q3SAH0\_HUMAN tr|Q5T2H4|Q5T2H4\_HUMAN sp|Q8N567|ZCHC9\_HUMAN tr|H7C5B3|H7C5B3\_HUMAN tr|E7EUG2|E7EUG2\_HUMAN sp|Q9NVV5|AIG1\_HUMAN tr|H0YFH6|H0YFH6\_HUMAN sp|Q8WTT0|CLC4C\_HUMAN tr|C9JNG9|C9JNG9\_HUMAN sp|Q12905|ILF2\_HUMAN tr|B4DY09|B4DY09\_HUMAN tr|H0YGB2|H0YGB2\_HUMAN tr|H0YJ78|H0YJ78\_HUMAN tr|H7C3R4|H7C3R4\_HUMAN ENSEMBL:ENSBTAP00000024462 sp|P68104|EF1A1\_HUMAN sp|Q6DWJ6|GP139\_HUMAN tr|A0A087WVQ9|A0A087WVQ9\_HUMAN tr|C9JLF4|C9JLF4\_HUMAN tr|E5RIE0|E5RIE0\_HUMAN sp|Q495N2|S36A3\_HUMAN sp|A8MU93|CQ100\_HUMAN tr|D3DTM5|D3DTM5\_HUMAN tr|E5RHV7|E5RHV7\_HUMAN tr|H3BMF3|H3BMF3\_HUMAN tr|E5RG03|E5RG03\_HUMAN tr|E5RGY6|E5RGY6\_HUMAN tr|E5RJL0|E5RJL0\_HUMAN tr|E5RFM3|E5RFM3\_HUMAN tr|E5RIB1|E5RIB1\_HUMAN sp|Q86UC2|RSPH3\_HUMAN sp|Q15345|LRC41\_HUMAN tr|G3V2C6|G3V2C6\_HUMAN sp|Q9H939|PPIP2\_HUMAN tr|B1AKV4|B1AKV4\_HUMAN tr|H0YM92|H0YM92\_HUMAN sp|P07948|LYN\_HUMAN tr|H3BM00|H3BM00\_HUMAN tr|B8ZZ60|B8ZZ60\_HUMAN tr|H0Y7T7|H0Y7T7\_HUMAN tr|K7EMR9|K7EMR9\_HUMAN tr|H0YJE6|H0YJE6\_HUMAN sp|Q32M88|ATHL1\_HUMAN tr|A0A087X2E7|A0A087X2E7\_HUMAN sp|Q92952|KCNN1\_HUMAN sp|Q96C74|ROP1L\_HUMAN sp|P18089|ADA2B\_HUMAN tr|A2RUS0|A2RUS0\_HUMAN sp|Q9BUM1|G6PC3\_HUMAN sp|Q9H3E2|SNX25\_HUMAN tr|M0QZQ2|M0QZQ2\_HUMAN tr|G3V0G6|G3V0G6\_HUMAN tr|G5E950|G5E950\_HUMAN tr|H0Y859|H0Y859\_HUMAN tr|B3KRT8|B3KRT8\_HUMAN tr|M0R025|M0R025\_HUMAN tr|M0QYG0|M0QYG0\_HUMAN tr|M0QXD7|M0QXD7\_HUMAN sp|Q7Z7H8|RM10\_HUMAN tr|M0QYA8|M0QYA8\_HUMAN tr|M0QZ79|M0QZ79\_HUMAN tr|M0QXV1|M0QXV1\_HUMAN tr|A0A087WWE7|A0A087WWE7\_HUMAN tr|A0A087WWG0|A0A087WWG0\_HUMAN sp|Q8N0T1|CH059\_HUMAN sp|Q15424|SAFB1\_HUMAN tr|A6NCE0|A6NCE0\_HUMAN sp|Q8N2W9|PIAS4\_HUMAN sp|Q8NH87|OR9G1\_HUMAN sp|Q9Y5Y5|PEX16\_HUMAN sp|O00203|AP3B1\_HUMAN sp|P49190|PTH2R\_HUMAN sp|Q9NYS0|KBRS1\_HUMAN tr|H7C4D8|H7C4D8\_HUMAN tr|G5E9P3|G5E9P3\_HUMAN tr|E9PP98|E9PP98\_HUMAN tr|E9PNL3|E9PNL3\_HUMAN sp|Q9HAD4|WDR41\_HUMAN tr|H0YAA3|H0YAA3\_HUMAN sp|Q99437|VATO\_HUMAN sp|Q6KB66|K2C80\_HUMAN tr|E9PKM2|E9PKM2\_HUMAN tr|H0Y5C6|H0Y5C6\_HUMAN sp|O94900|TOX\_HUMAN sp|Q8N6T0|CK080\_HUMAN tr|H0YE21|H0YE21\_HUMAN tr|D3DTX6|D3DTX6\_HUMAN sp|Q96SB3|NEB2\_HUMAN tr|B4DXL1|B4DXL1\_HUMAN Q6KB66-1 tr|H0YG77|H0YG77\_HUMAN tr|Q14DU5|Q14DU5\_HUMAN tr|Q5JSK7|Q5JSK7\_HUMAN tr|Q5JSK8|Q5JSK8\_HUMAN sp|Q496M5|PLK5\_HUMAN sp|P82970|HMGN5\_HUMAN tr|Q5JSL0|Q5JSL0\_HUMAN sp|Q9NPY3|C1QR1\_HUMAN tr|Q5JSK6|Q5JSK6\_HUMAN sp|Q8IZT8|HS3S5\_HUMAN sp|Q8WXS5|CCG8\_HUMAN tr|A0A087X0M4|A0A087X0M4\_HUMAN sp|Q6UX41|BTNL8\_HUMAN tr|H0YBZ0|H0YBZ0\_HUMAN sp|P15692|VEGFA\_HUMAN sp|Q9Y2C4|EXOG\_HUMAN tr|K7EQ93|K7EQ93\_HUMAN sp|Q8NH06|OR1P1\_HUMAN tr|J3QT35|J3QT35\_HUMAN sp|Q8TCP9|F200A\_HUMAN sp|Q6A1A2|PDPK2\_HUMAN tr|H7BZK1|H7BZK1\_HUMAN sp|Q9NX00|TM160\_HUMAN tr|H0YJ41|H0YJ41\_HUMAN tr|H7C109|H7C109\_HUMAN sp|Q9BV44|THUM3\_HUMAN tr|M0R1X1|M0R1X1\_HUMAN sp|O60542|PSPN\_HUMAN sp|Q68DI1|ZN776\_HUMAN sp|P27694|RFA1\_HUMAN tr|Q8WYZ0|Q8WYZ0\_HUMAN tr|H0Y926|H0Y926\_HUMAN sp|Q9ULQ0|STRP2\_HUMAN tr|B4E2J4|B4E2J4\_HUMAN tr|H0YLX4|H0YLX4\_HUMAN sp|Q9UKZ9|PCOC2\_HUMAN sp|Q3MJ13|WDR72\_HUMAN tr|H0YKE0|H0YKE0\_HUMAN sp|Q9BXJ3|C1QT4\_HUMAN tr|H0YEI1|H0YEI1\_HUMAN sp|Q15120|PDK3\_HUMAN sp|Q53HL2|BOREA\_HUMAN tr|Q5JT03|Q5JT03\_HUMAN tr|A0A075B719|A0A075B719\_HUMAN tr|D6REX0|D6REX0\_HUMAN tr|H0YJV8|H0YJV8\_HUMAN tr|H7C3A3|H7C3A3\_HUMAN tr|C9JQY5|C9JQY5\_HUMAN tr|G3V148|G3V148\_HUMAN sp|Q9H920|RN121\_HUMAN sp|Q99798|ACON\_HUMAN tr|H3BUQ8|H3BUQ8\_HUMAN sp|Q6ZNG9|KRBA2\_HUMAN tr|H3BN61|H3BN61\_HUMAN tr|F5H211|F5H211\_HUMAN sp|O95256|I18RA\_HUMAN sp|Q8NG77|O2T12\_HUMAN sp|Q7L523|RRAGA\_HUMAN sp|Q8IZA3|H1FOO\_HUMAN sp|Q9UQ74|PSG8\_HUMAN sp|Q96KX2|CAZA3\_HUMAN tr|C9J7G0|C9J7G0\_HUMAN tr|D6RIS1|D6RIS1\_HUMAN tr|F5H4N4|F5H4N4\_HUMAN tr|Q6ZRQ1|Q6ZRQ1\_HUMAN tr|D6R9V2|D6R9V2\_HUMAN sp|E7ETH6|Z587B\_HUMAN tr|M0QY62|M0QY62\_HUMAN tr|H3BPG8|H3BPG8\_HUMAN tr|F5H8D6|F5H8D6\_HUMAN sp|Q9H707|ZN552\_HUMAN sp|P30039|PBLD\_HUMAN tr|B4E0P5|B4E0P5\_HUMAN tr|F8WEM1|F8WEM1\_HUMAN tr|H0YDA7|H0YDA7\_HUMAN sp|Q96EN8|MOCOS\_HUMAN sp|A6NNE9|MARHB\_HUMAN tr|M0R1N2|M0R1N2\_HUMAN sp|Q5VV63|ATRN1\_HUMAN tr|B1AKR1|B1AKR1\_HUMAN sp|Q9UGP8|SEC63\_HUMAN sp|Q8TD86|CALL6\_HUMAN sp|Q86VE3|SATL1\_HUMAN sp|Q8N402|YV020\_HUMAN tr|J3KPD3|J3KPD3\_HUMAN sp|Q8TB73|NDNF\_HUMAN sp|Q8WXI4|ACO11\_HUMAN tr|H0YMJ6|H0YMJ6\_HUMAN tr|H0YLG2|H0YLG2\_HUMAN tr|D6RAL9|D6RAL9\_HUMAN tr|A0A096LP62|A0A096LP62\_HUMAN tr|F8WEY8|F8WEY8\_HUMAN sp|Q86T24|KAISO\_HUMAN tr|D6RA89|D6RA89\_HUMAN sp|A8MVJ9|YI028\_HUMAN tr|E9PJZ7|E9PJZ7\_HUMAN tr|F8WBJ4|F8WBJ4\_HUMAN sp|Q8NGV5|O13D1\_HUMAN tr|E9PJX8|E9PJX8\_HUMAN tr|M0QYY8|M0QYY8\_HUMAN tr|E9PME6|E9PME6\_HUMAN tr|F5H5P2|F5H5P2\_HUMAN tr|C9JC74|C9JC74\_HUMAN tr|H7C2U3|H7C2U3\_HUMAN tr|F5H890|F5H890\_HUMAN tr|E7EMZ0|E7EMZ0\_HUMAN tr|H0YFB4|H0YFB4\_HUMAN tr|F2Z2X8|F2Z2X8\_HUMAN sp|Q6NSI8|K1841\_HUMAN tr|A0A087X1S3|A0A087X1S3\_HUMAN tr|H0YEW9|H0YEW9\_HUMAN tr|E9PQ18|E9PQ18\_HUMAN sp|Q5ST30|SYVM\_HUMAN sp|Q6ZNB5|TRC2L\_HUMAN sp|Q9P2L0|WDR35\_HUMAN tr|E7EN12|E7EN12\_HUMAN tr|E9PP57|E9PP57\_HUMAN tr|C9JWL8|C9JWL8\_HUMAN tr|E9PLZ0|E9PLZ0\_HUMAN tr|E9PJ82|E9PJ82\_HUMAN sp|Q6ZV89|SH2D5\_HUMAN tr|F8WD89|F8WD89\_HUMAN tr|K7ELI0|K7ELI0\_HUMAN tr|M0R268|M0R268\_HUMAN sp|P09012|SNRPA\_HUMAN tr|K7ESH2|K7ESH2\_HUMAN tr|M0QZG7|M0QZG7\_HUMAN tr|G3V3E1|G3V3E1\_HUMAN sp|Q96KN2|CNDP1\_HUMAN tr|G3V5H7|G3V5H7\_HUMAN tr|J3KRP0|J3KRP0\_HUMAN tr|C9JDK1|C9JDK1\_HUMAN tr|C9JXY2|C9JXY2\_HUMAN tr|H0YDV3|H0YDV3\_HUMAN sp|P84550|SKOR1\_HUMAN tr|B1AMX5|B1AMX5\_HUMAN tr|C9JSR2|C9JSR2\_HUMAN tr|C9J0X3|C9J0X3\_HUMAN sp|P52951|GBX2\_HUMAN tr|B4DFF3|B4DFF3\_HUMAN tr|C9JS55|C9JS55\_HUMAN tr|C9K0F0|C9K0F0\_HUMAN tr|Q2TA70|Q2TA70\_HUMAN tr|C9J3D7|C9J3D7\_HUMAN tr|H0Y768|H0Y768\_HUMAN tr|F8W696|F8W696\_HUMAN tr|Q6P2Q0|Q6P2Q0\_HUMAN sp|Q9NP78|ABCB9\_HUMAN Q3KUS7 sp|P02647|APOA1\_HUMAN tr|A0A075B7A0|A0A075B7A0\_HUMAN tr|H7C2H0|H7C2H0\_HUMAN tr|K7EIE7|K7EIE7\_HUMAN tr|V9GY11|V9GY11\_HUMAN sp|Q06136|KDSR\_HUMAN tr|B4DLA0|B4DLA0\_HUMAN tr|A0A087WX09|A0A087WX09\_HUMAN tr|H7C490|H7C490\_HUMAN sp|Q9NX47|MARH5\_HUMAN tr|C9JFW8|C9JFW8\_HUMAN tr|B1ALD5|B1ALD5\_HUMAN sp|Q7Z4V0|ZN438\_HUMAN tr|C9JX16|C9JX16\_HUMAN sp|Q9UQQ1|NALDL\_HUMAN tr|F8WBU8|F8WBU8\_HUMAN sp|O43929|ORC4\_HUMAN tr|H0YDJ2|H0YDJ2\_HUMAN tr|H0YEF7|H0YEF7\_HUMAN sp|P22303|ACES\_HUMAN sp|P01619|KV301\_HUMAN tr|F8WD68|F8WD68\_HUMAN tr|V9GYJ7|V9GYJ7\_HUMAN tr|F8WAR7|F8WAR7\_HUMAN tr|Q5SX90|Q5SX90\_HUMAN tr|H0Y6M6|H0Y6M6\_HUMAN sp|O43670|ZN207\_HUMAN tr|A0A096LP01|A0A096LP01\_HUMAN sp|Q3SYA9|P12L1\_HUMAN tr|K7EMK9|K7EMK9\_HUMAN tr|B4E021|B4E021\_HUMAN tr|W4VSQ9|W4VSQ9\_HUMAN tr|Q5JZG9|Q5JZG9\_HUMAN sp|P08254|MMP3\_HUMAN sp|Q9BT78|CSN4\_HUMAN tr|A6NFI1|A6NFI1\_HUMAN tr|E9PMA1|E9PMA1\_HUMAN tr|A6NKM8|A6NKM8\_HUMAN sp|Q6NUT2|D19L2\_HUMAN sp|Q86V40|TIKI1\_HUMAN tr|B0QZK6|B0QZK6\_HUMAN tr|B0QZK8|B0QZK8\_HUMAN sp|Q9H867|MT21D\_HUMAN tr|V9GYH5|V9GYH5\_HUMAN tr|V9GY71|V9GY71\_HUMAN tr|B0QXZ9|B0QXZ9\_HUMAN tr|B0QXZ8|B0QXZ8\_HUMAN tr|E9PKK4|E9PKK4\_HUMAN sp|O60294|TYW4\_HUMAN tr|C9JWP9|C9JWP9\_HUMAN sp|Q9BUH8|BEGIN\_HUMAN tr|I3L1J9|I3L1J9\_HUMAN tr|H7BZX1|H7BZX1\_HUMAN sp|Q7L5A8|FA2H\_HUMAN sp|Q92990|GLMN\_HUMAN tr|D6RBQ8|D6RBQ8\_HUMAN tr|G3V3R5|G3V3R5\_HUMAN tr|K7EKT4|K7EKT4\_HUMAN tr|F8VWS0|F8VWS0\_HUMAN sp|P31350|RIR2\_HUMAN sp|Q8ND25|ZNRF1\_HUMAN tr|E7ETB4|E7ETB4\_HUMAN tr|H3BRB6|H3BRB6\_HUMAN sp|Q9P2F9|ZN319\_HUMAN sp|Q9H869|YYAP1\_HUMAN tr|B5MC98|B5MC98\_HUMAN tr|J3KR59|J3KR59\_HUMAN tr|J3KR68|J3KR68\_HUMAN sp|Q9UID6|ZN639\_HUMAN sp|Q9HCU5|PREB\_HUMAN sp|Q9Y6A4|CFA20\_HUMAN tr|F8WCN6|F8WCN6\_HUMAN tr|F5H3E1|F5H3E1\_HUMAN tr|M0R042|M0R042\_HUMAN sp|Q4G0T1|SRCRM\_HUMAN tr|A0A087WZY6|A0A087WZY6\_HUMAN tr|J3KNX1|J3KNX1\_HUMAN sp|A6NNW6|ENO4\_HUMAN tr|E9PR06|E9PR06\_HUMAN tr|A6NI74|A6NI74\_HUMAN sp|Q5SXH7|PKHS1\_HUMAN sp|Q9NQC8|IFT46\_HUMAN sp|Q9UBP5|HEY2\_HUMAN sp|Q9Y519|T184B\_HUMAN sp|Q5T2S8|ARMC4\_HUMAN tr|M0QY06|M0QY06\_HUMAN tr|A0A087WW81|A0A087WW81\_HUMAN sp|O75391|SPAG7\_HUMAN tr|D6RGE2|D6RGE2\_HUMAN sp|Q86W25|NAL13\_HUMAN sp|Q9H0Q0|FA49A\_HUMAN sp|Q8IV01|SYT12\_HUMAN sp|Q96CN7|ISOC1\_HUMAN tr|C9IYV6|C9IYV6\_HUMAN tr|C9JPE5|C9JPE5\_HUMAN tr|M4PM71|M4PM71\_HUMAN tr|M4PNB5|M4PNB5\_HUMAN tr|F8W8D3|F8W8D3\_HUMAN sp|O14581|OR7AH\_HUMAN tr|H7C331|H7C331\_HUMAN tr|M0R1N0|M0R1N0\_HUMAN sp|Q9HBV2|SACA1\_HUMAN tr|B7Z6U3|B7Z6U3\_HUMAN sp|P10314|1A32\_HUMAN sp|P30459|1A74\_HUMAN tr|M0R1Y2|M0R1Y2\_HUMAN tr|E7EVC2|E7EVC2\_HUMAN tr|A8MZ87|A
[truncated: 987,105 more chars]
