# Supplementary material for: Phospho-proteomic analyses of B-Raf protein complexes reveal new regulatory principles
Source: Oncotarget. 2016 Mar 28;7(18):26628–52. doi: 10.18632/oncotarget.8427 (PMC5042004; doi:10.18632/oncotarget.8427)
Supplement: Supplementary file 10 [file oncotarget-07-26628-s010.zip › Supplementary File 9.html]

PepView


| Peptide View (Result) | | | | |
| --- | --- | --- | --- | --- |
| MS File Choice | | Protein Choice | Filter Options | Display Options |
| --- | --- | --- | --- | --- |
| Available MS Files:    (F020656) tempfile (F006954)  (F020658) BSA Papain Verdau mit Cystein .temp (F011905) tempfile (F003591) tempfile (F003720) tempfile (F003586) tempfile (F003491) Labeled H3 (Trypsine).temp (F008966) tempfile (F003703) tempfile (F003438) tempfile (F003301) HAT3 KO\_Middle\_Thermolysine.temp (F011289) tempfile (F003634) tempfile (F008182) tempfile (F005202) tempfile (F006796) tempfile (F003121) tempfile (F006475) tempfile (F003710) tempfile (F003640) tempfile (F006689) tempfile (F002291) Heavy Acetylated TY1-H2A.temp (F016322) tempfile (F003574) tempfile (F005140) tempfile (F002239) tempfile (F006758) tempfile (F002596)  (F020404) tempfile (F003996) tempfile (F003212) tempfile (F002247) tempfile (F007734) tempfile (F001697) HAT2 KD\_48hr.temp (F011227) tempfile (F006785) tempfile (F003160) tempfile (F003217) tempfile (F016299) No0181\_Trypsin\_ETD.temp (F009585) tempfile (F003993) tempfile (F006961) tempfile (F001728) tempfile (F005307) HAT3 KO\_Lower\_Elastase.temp (F011232) tempfile (F006777) tempfile (F005606) tempfile (F006245) tempfile (F002254) tempfile (F006807) tempfile (F005576) tempfile (F005210) tempfile (F003425) tempfile (F006801) tempfile (F006778) tempfile (F002627) tempfile (F002281)  (F020491)  (F020402) tempfile (F002600) tempfile (F005580) tempfile (F003428) Light Acetylated H3\_Quan.temp (F010891) tempfile (F003709) tempfile (F007298) tempfile (F008561) tempfile (F001560) tempfile (F005669) tempfile (F001620) tempfile (F002236) tempfile (F002632) tempfile (F003628) HAT2 KD\_0hr.temp (F011178) tempfile (F003744)  (F016879) tempfile (F006771) tempfile (F003303) tempfile (F002305) HAT2 KD\_24hr.temp (F011183) tempfile (F006573) tempfile (F005613) tempfile (F008602) tempfile (F003431) tempfile (F002556) tempfile (F007878) tempfile (F001901) tempfile (F003584) tempfile (F001564) tempfile (F005608) tempfile (F006953) HAT2 KD\_24hr.temp (F011182) BSA Papain Verdau ohne Cystein .temp (F011904) tempfile (F003978) tempfile (F003981) tempfile (F003617) tempfile (F002288)  (F016877) tempfile (F003581) tempfile (F005157) tempfile (F002300) tempfile (F005582) tempfile (F001908)  (F020380) tempfile (F007877) tempfile (F009649) tempfile (F006830) tempfile (F003422) HAT2 KD\_48hr.temp (F011219) HAT2 KD\_24h.temp (F011294) tempfile (F003635) tempfile (F003702) tempfile (F006792) tempfile (F003493)  (F020655) HK-2 Histones NOB.temp (F009415) tempfile (F003714) tempfile (F003302) tempfile (F005204) tempfile (F003309) tempfile (F006233) tempfile (F006815) tempfile (F007301) tempfile (F003708) Labeled H3 (Trypsine).temp (F009311) tempfile (F007299) tempfile (F002253) tempfile (F003161) tempfile (F001696) tempfile (F009648) tempfile (F005597) tempfile (F016248) tempfile (F006763) tempfile (F001561) tempfile (F001893) tempfile (F002282) tempfile (F010795) tempfile (F005581) tempfile (F003649) tempfile (F003740) tempfile (F010803) tempfile (F006802) tempfile (F002248) tempfile (F002610) HAT2 KD\_0hr.temp (F011179) tempfile (F006770) tempfile (F002241) tempfile (F002628) tempfile (F006234) Light Acetylated H3\_Quan.temp (F011075) tempfile (F008566) tempfile (F005607) tempfile (F002289) tempfile (F002306) tempfile (F006795) tempfile (F006808) tempfile (F005575) tempfile (F003743) tempfile (F003053) tempfile (F016290) tempfile (F002633) tempfile (F007916) tempfile (F005571) tempfile (F006779) tempfile (F006784) tempfile (F003690) tempfile (F006963) tempfile (F003300) tempfile (F005073) tempfile (F002563) tempfile (F003218) tempfile (F003984) tempfile (F003976) tempfile (F008603) tempfile (F003578) tempfile (F003643) tempfile (F003918) No0181\_Trypsin\_HCD.temp (F009586) tempfile (F003742) tempfile (F005212) tempfile (F006831) tempfile (F003741) tempfile (F003696) tempfile (F009642) tempfile (F002290) tempfile (F001622) tempfile (F005593) tempfile (F010786) tempfile (F002287)  (F020654) Light Acetylated H3\_Thermolysin.temp (F010890) tempfile (F010783) tempfile (F002235) tempfile (F001619) Labeled H3 (Trypsine).temp (F009155) tempfile (F002303) tempfile (F002624) tempfile (F003637) tempfile (F003159) tempfile (F003636) tempfile (F002565) tempfile (F003980) tempfile (F002566) tempfile (F008606) tempfile (F005531) HK-2 Histones Ctrl.temp (F009414) tempfile (F006794) tempfile (F001884) tempfile (F002551) tempfile (F002570) tempfile (F001699) tempfile (F001725) tempfile (F006956) tempfile (F006765) tempfile (F003423) tempfile (F003116) tempfile (F009730) tempfile (F016283)  (F020406) tempfile (F006797) tempfile (F005667) tempfile (F008563) tempfile (F016249) tempfile (F002602) tempfile (F002598) tempfile (F003701) tempfile (F003097) tempfile (F003693) tempfile (F002634) tempfile (F006783) tempfile (F003420) tempfile (F006235) tempfile (F006832) tempfile (F003712) tempfile (F005596) tempfile (F005297) tempfile (F012138) Light Acetylated H3\_Quan.temp (F011074) tempfile (F001551) tempfile (F006962) tempfile (F002564) tempfile (F001695) tempfile (F005572) tempfile (F003589) tempfile (F006762) HAT2 KD\_48hr.temp (F011185) tempfile (F002286) tempfile (F003579) tempfile (F003162) tempfile (F009643) tempfile (F006809) tempfile (F003294) tempfile (F008604) tempfile (F006782) tempfile (F006476) tempfile (F002299) tempfile (F003176) tempfile (F005301) tempfile (F012141) tempfile (F006955)  (F020401) tempfile (F003921) tempfile (F006800) tempfile (F006789) tempfile (F003642) tempfile (F010784) tempfile (F002552) tempfile (F003429) tempfile (F005605) tempfile (F012139) tempfile (F001885) tempfile (F003644) tempfile (F003592) tempfile (F005309) tempfile (F002242) tempfile (F001910) tempfile (F003308) tempfile (F003721) tempfile (F005612) tempfile (F008283) tempfile (F002302) No0181\_Elastase\_ETD.temp (F009583) tempfile (F001698) tempfile (F001553) tempfile (F006244) Labeled H3 50% (Elastase).temp (F008967) tempfile (F003638) tempfile (F008780) tempfile (F008605) tempfile (F005602) tempfile (F002571) tempfile (F002601) HAT2 KD\_0hr.temp (F011177) tempfile (F005598) tempfile (F002597) tempfile (F001892) tempfile (F003715) tempfile (F003430) tempfile (F006236) tempfile (F002250) tempfile (F006764) tempfile (F002298)  (F016878) HAT2 KD\_24hr.temp (F011184) tempfile (F005103) tempfile (F006755) tempfile (F002234) tempfile (F003692) tempfile (F005609) tempfile (F003641) tempfile (F012137) tempfile (F003098) tempfile (F002560) tempfile (F005600) tempfile (F001535) tempfile (F003576) tempfile (F005300)  (F020408) tempfile (F003216) HAT2 KD\_48h.temp (F011317) tempfile (F006966) tempfile (F003931) RE 50pcAc light, 50pcAc heavy.temp (F009309) Light Acetylated H3\_Quan.temp (F011076) tempfile (F003580) tempfile (F003307) Light Acetylated H3\_Quan.temp (F010887) tempfile (F003421) tempfile (F003645) tempfile (F005303) tempfile (F006774) tempfile (F016301) tempfile (F009741) tempfile (F006951) tempfile (F010802) tempfile (F006806) tempfile (F003651) tempfile (F003722) tempfile (F002243) tempfile (F003718) tempfile (F003435) tempfile (F003177) tempfile (F016300) tempfile (F002568) tempfile (F002284) Labeled H3 (Trypsine).temp (F009156) tempfile (F003639) tempfile (F009742)  Heavy Acetylated TY1-H2A.temp (F016324) tempfile (F006246) tempfile (F006834) tempfile (F003306)  (F020659) tempfile (F016302) tempfile (F003650) No0181\_Elastase\_HCD.temp (F009584) tempfile (F001552) Light Acetylated H3\_Quan.temp (F010895) tempfile (F005665) tempfile (F006958) tempfile (F003618) tempfile (F002304) tempfile (F016247)  Heavy Acetylated TY1-H2A.temp (F016323) tempfile (F003440) tempfile (F006964) tempfile (F006241) tempfile (F005573) tempfile (F005579) tempfile (F006767) tempfile (F001886) tempfile (F005585) HAT3 KO\_Upper\_Elastase.temp (F011230) tempfile (F001567) tempfile (F003698) tempfile (F002626) tempfile (F002301) tempfile (F008284) tempfile (F002285) tempfile (F002297) tempfile (F005124) tempfile (F005136) tempfile (F003114) HAT2 KD\_24h.temp (F011311) tempfile (F003695) tempfile (F002567) tempfile (F006766) tempfile (F010785) tempfile (F002611) tempfile (F002244) tempfile (F002629) tempfile (F005614) tempfile (F006754) HAT2 KD\_24h.temp (F011312) tempfile (F002608) tempfile (F003725)  (F020377) tempfile (F006829) tempfile (F003691) tempfile (F003213) tempfile (F006952)  (F020403) tempfile (F006780) tempfile (F003975) tempfile (F016289) tempfile (F005302) tempfile (F002623) tempfile (F002554) tempfile (F016291) tempfile (F006833) tempfile (F003096) tempfile (F003295) tempfile (F006237) tempfile (F003987) tempfile (F006479) tempfile (F002630)  (F020405) Light Acetylated H3\_Elastase.temp (F010886) tempfile (F001730) tempfile (F006571) tempfile (F006757) tempfile (F003697) tempfile (F006788) tempfile (F003723) tempfile (F003624) tempfile (F005532) tempfile (F001887) tempfile (F001854) tempfile (F003646) tempfile (F006791) tempfile (F006965) tempfile (F003113) tempfile (F003713) tempfile (F016284) tempfile (F008562) tempfile (F008285) Light Acetylated H3\_Proteinase K.temp (F010894) tempfile (F009740) tempfile (F003694) tempfile (F005143) tempfile (F002599) tempfile (F006810) tempfile (F006238) tempfile (F006957) tempfile (F003700) tempfile (F005298) tempfile (F006572) tempfile (F006798) tempfile (F003051) tempfile (F016250) tempfile (F010807) tempfile (F005584) tempfile (F006242) tempfile (F009744) tempfile (F005670) tempfile (F001894) tempfile (F008564) tempfile (F012140) tempfile (F002245) tempfile (F001700) HAT3 KO\_Middle\_Elastase.temp (F011231) tempfile (F003484) tempfile (F006761) tempfile (F006803) tempfile (F003989) tempfile (F006787) Labeled H3 50% (Thermolysine).temp (F008968) tempfile (F005304) tempfile (F001722) tempfile (F003588) tempfile (F002603) tempfile (F008600) tempfile (F003724) tempfile (F003926) tempfile (F003178) tempfile (F006773) tempfile (F010805)  (F020397) tempfile (F006959) tempfile (F006472) tempfile (F005142) tempfile (F002252) tempfile (F002249) tempfile (F006814) tempfile (F003997) tempfile (F003492) tempfile (F005139) tempfile (F003625) tempfile (F002555) HAT2 KD\_0h.temp (F011293) tempfile (F007915) tempfile (F002595) tempfile (F001729) tempfile (F003071) tempfile (F002240) tempfile (F002609) tempfile (F007300) tempfile (F001895) tempfile (F016286) tempfile (F006781) tempfile (F003310) tempfile (F001914) tempfile (F002558) tempfile (F003593) tempfile (F006775) tempfile (F001536)  (F020657) tempfile (F001554)  (F020400) HAT2 KD\_48h.temp (F011313)  (F020396) tempfile (F003211) tempfile (F006239) tempfile (F008565) tempfile (F003582) tempfile (F002238) tempfile (F002561) BSA Papain Verdau ohne Cystein .temp (F011903) tempfile (F001855) tempfile (F007882) tempfile (F002251) tempfile (F002625) tempfile (F005299)  (F020407) tempfile (F009743) tempfile (F006799) tempfile (F003719) tempfile (F006243) tempfile (F006811) tempfile (F001621) tempfile (F006804) tempfile (F010806) tempfile (F003590) tempfile (F010808) tempfile (F003707) tempfile (F003699) tempfile (F003711) tempfile (F003998) tempfile (F005601) tempfile (F012136) HAT3 KO\_Upper\_Thermolysine.temp (F011288) tempfile (F005611) tempfile (F006759) HAT2 KD\_0h.temp (F011292) tempfile (F003587) tempfile (F002246) tempfile (F006756) tempfile (F006753) tempfile (F005305) tempfile (F003434) tempfile (F006790) tempfile (F003647) tempfile (F001723) tempfile (F001570) tempfile (F003988) tempfile (F005595) tempfile (F003304) tempfile (F004976) tempfile (F005570) tempfile (F006772) tempfile (F007733) tempfile (F006805) tempfile (F003626) tempfile (F003575) tempfile (F009739) tempfile (F006786) tempfile (F005141) HAT2 KD\_0h.temp (F011291) tempfile (F003054) tempfile (F005306) Rasha.temp (F008810) tempfile (F003648) tempfile (F008762) tempfile (F006769) tempfile (F002237) tempfile (F006960) tempfile (F003439) tempfile (F016292) tempfile (F001557) HAT3 KO\_Lower\_Thermolysine.temp (F011290) tempfile (F007827) tempfile (F006768) tempfile (F001857) tempfile (F008601) tempfile (F006793) tempfile (F002569) tempfile (F003112) tempfile (F008286) tempfile (F005583) tempfile (F009650) tempfile (F001856) tempfile (F002557) tempfile (F006760) tempfile (F010794) tempfile (F006812) tempfile (F005577) tempfile (F006240) HAT2 KD\_48h.temp (F011314) tempfile (F002631) tempfile (F005610) tempfile (F003982) tempfile (F003424) tempfile (F016285) tempfile (F006813) LCMS001711 (F020343) LCMS001834 (F020409) LCMS001835 (F020410) LCMS001836 (F020411) LCMS001841 (F020413) 01\_velos\_2700 (F004434) 01\_velos\_2700 (F004435) 01\_velos\_2701 (F004437) 01\_velos\_2701 (F004436) 01\_velos\_2702 (F004438) 01\_velos\_2703 (F004439) 01\_velos\_2704 (F004440) 01\_velos\_2704 (F004441) 01\_velos\_2705 (F004443) 01\_velos\_2705 (F004442) 01\_velos\_2708 (F004445) 01\_velos\_2708 (F004444) 01\_velos\_2709 (F004447) 01\_velos\_2709 (F004446) 01\_velos\_2710 (F004448) 01\_velos\_2711 (F004449) 01\_velos\_2712 (F004450) 01\_velos\_2712 (F004451) 01\_velos\_2713 (F004452) 01\_velos\_2713 (F004453) 06\_Fusion\_0164 (F017353) 06\_Fusion\_0165 (F017354) 06\_Fusion\_0166 (F017355) 06\_velos\_2737 (F004554) 06\_velos\_2738 (F004556) 06\_velos\_2739 (F004558) 06\_velos\_2740 (F004559) 06\_velos\_2741 (F004560) 06\_velos\_2742 (F004562) 06\_velos\_2745 (F004564) 06\_velos\_2746 (F004566) 06\_velos\_2747 (F004568) 06\_velos\_2748 (F004569) 06\_velos\_2749 (F004570) 06\_velos\_2750 (F004572) 15\_velos\_2834 (F004656) 15\_velos\_2834 (F004657) 15\_velos\_2835 (F004659) 15\_velos\_2835 (F004658) 15\_velos\_2836 (F004660) 15\_velos\_2837 (F004661) 18\_Fusion\_0252 (F017360) 18\_Fusion\_0253 (F017361) 19\_velos\_2535 (F004266) 19\_velos\_2536 (F004267) 19\_velos\_2537 (F004268) 19\_velos\_2538 (F004269) 0021\_QTOF\_0254 (F020414) 0021\_QTOF\_0256 (F020415) 21\_Fusion\_0316 (F017369) 21\_Fusion\_0317 (F017370) 21\_Fusion\_0318 (F017371) 21\_velos\_2594 (F004307) 21\_velos\_2595 (F004308) 21\_velos\_2596 (F004309) 21\_velos\_2597 (F004310) 22\_velos\_2608 (F004431) 22\_velos\_2609 (F004315) 22\_velos\_2610 (F004316) 22\_velos\_2611 (F004432) 22\_velos\_2612 (F004318) 22\_velos\_2613 (F004319) 22\_velos\_2614 (F004433) 22\_velos\_2615 (F004321) 22\_velos\_2616 (F004322) 22\_velos\_2617 (F004392) 22\_velos\_2618 (F004324) 22\_velos\_2619 (F004325) 23\_velos\_1609 (F003507) 23\_velos\_1610 (F003513) 23\_velos\_1611 (F003610) 23\_velos\_1612 (F003512) 23\_velos\_1613 (F003508) 23\_velos\_1614 (F003511) 26\_JV\_0200 (F001498) 26\_JV\_0201 (F001500) 26\_velos\_2672 (F004541) 26\_velos\_2673 (F004542) 26\_velos\_2674 (F004543) 26\_velos\_2675 (F004544) 26\_velos\_2676 (F004545) 26\_velos\_2677 (F004546) 26\_velos\_2685 (F004394) 26\_velos\_2686 (F004396) 26\_velos\_2687 (F004397) 26\_velos\_2688 (F004398) 27\_JV\_0219 (F001534) 29\_velos\_1722 (F003376) 29\_velos\_1723 (F003399) 29\_velos\_1724 (F003407) 29\_velos\_1725 (F003400) 0035\_QTOF\_0374 (F020416) 0035\_QTOF\_0376 (F020418) 0063\_TRAP\_00801 (F020458) 0063\_TRAP\_00802 (F020459) 0121\_QTOF\_1687 (F020442) 0171\_TRAP\_02202 (F020470) 0195\_TRAP\_02646 (F020472) 0195\_TRAP\_02665 (F020471) 0204\_TRAP\_02780 (F020478) 0204\_TRAP\_02782 (F020479) 0305\_QTOF\_4317 (F020446) 0305\_QTOF\_4318 (F020447) 0348\_QTOF\_5076 (F020482) 0348\_QTOF\_5078 (F020483) 0348\_QTOF\_5096 (F020480) 0348\_QTOF\_5098 (F020481) 1:1 Acetylated H3 (Trypsine).temp (F009289) 06 (F017359) 06 (F017356) 21 (F017990) 21 (F018005) 21 (F018010) 21 (F017996) 21 (F018007) 21 (F017991) 21 (F018002) 21 (F017997) 21 (F018011) 21 (F018006) 21 (F017981) 21 (F017998) 21 (F019355) 21 (F017416) 21 (F017984) 21 (F018000) 21 (F017993) 21 (F018009) 21 (F017982) 21 (F017983) 21 (F017999) 21 (F018008) 21 (F018001) 21 (F017985) 21 (F017417) 21 (F019354) 21 (F017977) 21 (F017995) 21 (F018003) 21 (F017976) 21 (F017992) 21 (F017979) 21 (F017989) 21 (F017418) 21 (F017988) 21 (F019359) 21 (F017987) 21 (F019360) 21 (F017986) 21 (F017980) 21 (F018004) 21 (F017978) 21 (F017994) 50% Acetylated H3\_E 2.temp (F009745) 50% Acetylated H3\_All-in-one method (Trypsin).temp (F009504) 50% Acetylated H3\_Elastase.temp (F010996) 50% Acetylated H3\_Elastase.temp (F010760) 50% Acetylated H3\_Th 2.temp (F009749) 50% Acetylated H3\_Thermolysine.temp (F010761) 50% Acetylated H3\_Elastase.temp (F012673) 50% Heavy Acetylated H3\_Elastase (1st).temp (F012765) 50% Heavy Acetylated H3\_Papain (1st).temp (F012767) 0211\_TRAP\_02862.D (F020473) 0212\_TRAP\_02884.D (F020474) 328\_QTOF\_4752 - F017450 (F020493) 2013-01-15\_velos\_2171\_dPER\_dTIM only (F004129) 2013-01-17\_velos\_2223\_CSF\_Tryp\_ETD\_only\_PLK4\_AGMS (F004077) 2013-01-15\_velos\_2187\_dPER\_dTIM only (F004132) 2013-01-17\_velos\_2231\_Int\_Thermo\_HCD\_only\_PLK4\_AGMS (F004084) 2013-01-17\_velos\_2217\_M\_Thermo\_HCD\_onlyPLK4\_AGMS (F004072) 2013-01-17\_velos\_2229\_Int\_Trypsin\_HCD\_only\_PLK4\_AGMS (F004082) 2013-01-17\_velos\_2230\_Int\_Trypsin\_ETD\_only\_PLK4\_AGMS (F004083) 2013-01-15\_velos\_2188\_dPER\_dTIM only (F004131) 2013-01-17\_velos\_2215\_M\_Tryp\_HCD\_onlyPLK4\_AGMS (F004070) 2013-01-17\_velos\_2216\_M\_Tryp\_ETD\_onlyPLK4\_AGMS (F004071) 2013-01-17\_velos\_2227\_CSF\_Elastase\_ETD\_only\_PLK4\_AGMS (F004081) 2013-01-15\_velos\_2185\_5 \_dPER\_dTIM only (F004127) 2013-01-17\_velos\_2226\_CSF\_Elastase\_HCD\_only\_PLK4\_AGMS (F004079) 2013-01-17\_velos\_2225\_CSF\_Thermo\_ETD\_only\_PLK4\_AGMS (F004080) 2013-01-17\_velos\_2219\_M\_Elastase\_HCD\_onlyPLK4\_AGMS (F004074) 2013-01-15\_velos\_2186-dPER\_dTIM only (F004130) 2013-01-17\_velos\_2234\_Int\_Elastase\_ETD\_only\_PLK4\_AGMS (F004087) 2013-01-17\_velos\_2218\_M\_Thermo\_ETD\_onlyPLK4\_AGMS (F004073) 2013-01-17\_velos\_2222\_CSF\_Tryp\_HCD\_only\_PLK4\_AGMS (F004076) 2013-01-17\_velos\_2233\_Int\_Elastase\_HCD\_only\_PLK4\_AGMS (F004086) 2013-01-17\_velos\_2220\_M\_Elastase\_ETD\_only\_PLK4\_AGMS (F004075) 2013-01-17\_velos\_2232\_Int\_Thermo\_ETD\_only\_PLK4\_AGMS (F004085) 2013-01-17\_velos\_2224\_CSF\_Thermo\_HCD\_only\_PLK4\_AGMS (F004078) 2013-01-15\_velos\_2170\_dPER\_dTIM only (F004128) | |  |  |  | | --- | --- | --- | | MS Files: |  | Color: | |  | | | | (F020654) |  |  | | (F020655) |  |  | | (F020656) |  |  | | (F020657) |  |  | | (F020658) |  |  | | (F020659) |  |  | | |  | | --- | | sp|X004716| sp|X004715| sp|X004711| tr|F6SZ47|F6SZ47\_MOUSE sp|P28028|BRAF\_MOUSE sp|P28028-2|BRAF\_MOUSE sp|P11499|HS90B\_MOUSE sp|P07901|HS90A\_MOUSE tr|B7ZC50|B7ZC50\_MOUSE sp|B2RSH2|GNAI1\_MOUSE P04264 sp|Q99N57|RAF1\_MOUSE sp|Q99N57-2|RAF1\_MOUSE tr|E9Q3D6|E9Q3D6\_MOUSE tr|B7ZC49|B7ZC49\_MOUSE sp|Q8VIJ6|SFPQ\_MOUSE P35527 P13645 sp|P04627|ARAF\_MOUSE tr|A2A6A2|A2A6A2\_MOUSE tr|E9PX27|E9PX27\_MOUSE tr|B1AUP0|B1AUP0\_MOUSE sp|P13020|GELS\_MOUSE sp|P13020-2|GELS\_MOUSE P35908 tr|D3Z1R1|D3Z1R1\_MOUSE sp|Q61033|LAP2A\_MOUSE tr|E9Q0C3|E9Q0C3\_MOUSE sp|Q60865|CAPR1\_MOUSE sp|A2ASS6|TITIN\_MOUSE sp|Q8VDF2|UHRF1\_MOUSE tr|E9Q8N1|E9Q8N1\_MOUSE tr|E9Q8K5|E9Q8K5\_MOUSE sp|P58252|EF2\_MOUSE sp|Q8VDF2-2|UHRF1\_MOUSE Q3SX14 sp|P04104|K2C1\_MOUSE ENSEMBL:ENSBTAP00000038253 sp|Q9DC51|GNAI3\_MOUSE tr|F6TUC4|F6TUC4\_MOUSE P00761 sp|O35286|DHX15\_MOUSE sp|P02535|K1C10\_MOUSE tr|A2A513|A2A513\_MOUSE sp|P02535-2|K1C10\_MOUSE P02535-1 sp|P02535-3|K1C10\_MOUSE sp|A2ASS6-2|TITIN\_MOUSE sp|Q8BQ30|PPR18\_MOUSE sp|Q8BQ30-2|PPR18\_MOUSE Q3TTY5 sp|Q3TTY5|K22E\_MOUSE sp|Q922U2|K2C5\_MOUSE Q922U2 Q5XQN5 sp|Q3TZZ7|ESYT2\_MOUSE P02538 sp|Q8CAQ8-5|MIC60\_MOUSE sp|Q8CAQ8-2|MIC60\_MOUSE sp|Q8CAQ8|MIC60\_MOUSE P02533 P04259 P48668 sp|Q6IFZ6|K2C1B\_MOUSE Q6IFZ6 sp|P11679|K2C8\_MOUSE P13647 tr|B2KF34|B2KF34\_MOUSE sp|P47811-2|MK14\_MOUSE sp|P47811-3|MK14\_MOUSE sp|P47811|MK14\_MOUSE tr|B2KF35|B2KF35\_MOUSE Q8VED5 sp|Q8VED5|K2C79\_MOUSE sp|Q921N6|DDX27\_MOUSE sp|Q8CAQ8-3|MIC60\_MOUSE tr|E9Q800|E9Q800\_MOUSE P08779 sp|P70168|IMB1\_MOUSE sp|P08113|ENPL\_MOUSE sp|Q61414|K1C15\_MOUSE tr|B1AQ77|B1AQ77\_MOUSE A2A4G1 sp|Q6IFZ9|K2C74\_MOUSE Q04695 Q9QWL7 sp|Q9QWL7|K1C17\_MOUSE sp|Q8BGZ7|K2C75\_MOUSE Q8BGZ7 sp|P50446|K2C6A\_MOUSE P50446 tr|A2AS05|A2AS05\_MOUSE tr|Q3UV11|Q3UV11\_MOUSE sp|Q9CQN1|TRAP1\_MOUSE sp|Q8CGC6|RBM28\_MOUSE sp|Q9Z331|K2C6B\_MOUSE Q7Z3Y8 Q6NXH9 sp|Q6NXH9|K2C73\_MOUSE tr|A2AS03|A2AS03\_MOUSE sp|E9QAM5|HELZ2\_MOUSE sp|P08752|GNAI2\_MOUSE sp|Q61029|LAP2B\_MOUSE sp|Q61029-3|LAP2B\_MOUSE sp|Q61033-2|LAP2A\_MOUSE sp|Q61029-4|LAP2B\_MOUSE sp|Q61029-2|LAP2B\_MOUSE tr|E9QAY6|E9QAY6\_MOUSE Q9R0H5 sp|Q9R0H5|K2C71\_MOUSE sp|Q61781|K1C14\_MOUSE Q7Z794 tr|A6PWS5|A6PWS5\_MOUSE tr|E9Q0F0|E9Q0F0\_MOUSE sp|A2AAE1-3|K1109\_MOUSE sp|A2AAE1-6|K1109\_MOUSE sp|A2AAE1-2|K1109\_MOUSE sp|A2AAE1|K1109\_MOUSE sp|A2AAE1-4|K1109\_MOUSE tr|F7CR78|F7CR78\_MOUSE sp|O54943|PER2\_MOUSE Q7Z3Y7 Q148H6 Q9Z2K1 Q3ZAW8 sp|Q9Z2K1|K1C16\_MOUSE sp|Q91VR5|DDX1\_MOUSE tr|H3BLJ6|H3BLJ6\_MOUSE sp|Q921N6-2|DDX27\_MOUSE P19013 P05787 P19012 sp|Q6ZWR6|SYNE1\_MOUSE sp|Q6ZWR6-4|SYNE1\_MOUSE sp|E9Q555|RN213\_MOUSE tr|F7A6H4|F7A6H4\_MOUSE P08730-1 sp|P08730|K1C13\_MOUSE tr|E9Q5Y5|E9Q5Y5\_MOUSE sp|P08730-2|K1C13\_MOUSE sp|P05784|K1C18\_MOUSE P05784 Q5XKE5 tr|E9Q7P0|E9Q7P0\_MOUSE sp|Q69Z23-2|DYH17\_MOUSE sp|Q69Z23|DYH17\_MOUSE tr|K3W4R0|K3W4R0\_MOUSE tr|S4R1P5|S4R1P5\_MOUSE sp|Q91ZU6-3|DYST\_MOUSE tr|E9Q9X1|E9Q9X1\_MOUSE sp|Q91ZU6|DYST\_MOUSE sp|Q91ZU6-4|DYST\_MOUSE tr|D3YXG0|D3YXG0\_MOUSE tr|D3Z2Q7|D3Z2Q7\_MOUSE P19001 sp|P19001|K1C19\_MOUSE P08727 tr|A2A520|A2A520\_MOUSE tr|B1ART2|B1ART2\_MOUSE tr|B1ART1|B1ART1\_MOUSE O95678 sp|Q8K440|ABC8B\_MOUSE sp|Q8K440-2|ABC8B\_MOUSE tr|Q6DIC6|Q6DIC6\_MOUSE sp|B2RWS6|EP300\_MOUSE tr|B7ZCJ0|B7ZCJ0\_MOUSE tr|B7ZCJ1|B7ZCJ1\_MOUSE sp|Q6DFV3|RHG21\_MOUSE sp|Q9Z320|K1C27\_MOUSE tr|A9C473|A9C473\_MOUSE tr|A9C474|A9C474\_MOUSE tr|E9PZ45|E9PZ45\_MOUSE tr|Q6P5B5|Q6P5B5\_MOUSE tr|E9QA63|E9QA63\_MOUSE tr|B1ARU4|B1ARU4\_MOUSE sp|Q9QXZ0|MACF1\_MOUSE tr|E9PVY8|E9PVY8\_MOUSE sp|Q3V0Q1|DYH12\_MOUSE tr|S4R1L5|S4R1L5\_MOUSE tr|E9Q0P8|E9Q0P8\_MOUSE tr|E9Q1W3|E9Q1W3\_MOUSE tr|A2AQA9|A2AQA9\_MOUSE sp|O88738-3|BIRC6\_MOUSE tr|S4R2P8|S4R2P8\_MOUSE sp|O88738|BIRC6\_MOUSE sp|O88738-2|BIRC6\_MOUSE Q7Z3Z0 sp|Q8C4J7|TBL3\_MOUSE sp|Q9WVR4|FXR2\_MOUSE tr|E9Q5I3|E9Q5I3\_MOUSE Q32MB2 sp|Q80TF6|STAR9\_MOUSE tr|A2AKH9|A2AKH9\_MOUSE sp|P55200|KMT2A\_MOUSE sp|P55200-2|KMT2A\_MOUSE sp|Q8BSQ9-2|PB1\_MOUSE tr|E9Q2E4|E9Q2E4\_MOUSE sp|A2ASS6-3|TITIN\_MOUSE sp|Q3UV17|K22O\_MOUSE sp|P0CG50|UBC\_MOUSE tr|E9PX61|E9PX61\_MOUSE tr|E9Q4Y5|E9Q4Y5\_MOUSE tr|E9Q7L3|E9Q7L3\_MOUSE tr|D3Z1N4|D3Z1N4\_MOUSE sp|Q8BSQ9|PB1\_MOUSE tr|D3YYF2|D3YYF2\_MOUSE tr|E9Q7L2|E9Q7L2\_MOUSE tr|F8VQD1|F8VQD1\_MOUSE sp|Q3TZZ7-2|ESYT2\_MOUSE sp|P14873|MAP1B\_MOUSE tr|J3QQ22|J3QQ22\_MOUSE sp|Q8BKX6|SMG1\_MOUSE sp|O08852|PKD1\_MOUSE sp|Q80W93|HYDIN\_MOUSE H-INV:HIT000016045 sp|Q01853|TERA\_MOUSE tr|F6YLI0|F6YLI0\_MOUSE sp|Q80TA9|EPG5\_MOUSE sp|Q8VHG0|FMO4\_MOUSE sp|Q2PZL6|FAT4\_MOUSE tr|V9GX23|V9GX23\_MOUSE tr|A2ANY6|A2ANY6\_MOUSE tr|J3QMC5|J3QMC5\_MOUSE tr|B1ARU1|B1ARU1\_MOUSE tr|E9PVX6|E9PVX6\_MOUSE sp|Q4U4S6|XIRP2\_MOUSE tr|Q9CZ86|Q9CZ86\_MOUSE tr|D3Z667|D3Z667\_MOUSE sp|P0C6F1|DYH2\_MOUSE sp|Q921Q7|RIN1\_MOUSE sp|Q03172|ZEP1\_MOUSE tr|F8VPM9|F8VPM9\_MOUSE sp|Q6ZQA0|NBEL2\_MOUSE tr|E9QAE4|E9QAE4\_MOUSE sp|Q8C170-2|MYO9A\_MOUSE tr|D3Z3A8|D3Z3A8\_MOUSE sp|Q8C170|MYO9A\_MOUSE tr|Q3TA68|Q3TA68\_MOUSE tr|Q3TAQ9|Q3TAQ9\_MOUSE sp|Q4U4S6-2|XIRP2\_MOUSE sp|P49025-4|CTRO\_MOUSE sp|Q91ZU6-2|DYST\_MOUSE sp|O88491|NSD1\_MOUSE sp|O08788-2|DCTN1\_MOUSE sp|Q9QXZ0-2|MACF1\_MOUSE sp|Q9QXZ0-3|MACF1\_MOUSE tr|G3X9N4|G3X9N4\_MOUSE sp|Q9DAI4|ZBT43\_MOUSE sp|O35219-2|KCNH2\_MOUSE sp|O35219|KCNH2\_MOUSE tr|Q53Z09|Q53Z09\_MOUSE sp|A2AN08|UBR4\_MOUSE sp|A2AN08-3|UBR4\_MOUSE sp|A2AN08-5|UBR4\_MOUSE sp|P19785|ESR1\_MOUSE tr|E9PUM4|E9PUM4\_MOUSE sp|Q9D479|UVSSA\_MOUSE tr|E9PZL9|E9PZL9\_MOUSE sp|Q2EG98|PK1L3\_MOUSE sp|Q2EG98-4|PK1L3\_MOUSE sp|Q2EG98-3|PK1L3\_MOUSE tr|F8VPR5|F8VPR5\_MOUSE sp|P45481|CBP\_MOUSE tr|E9PZP8|E9PZP8\_MOUSE tr|S4R2N8|S4R2N8\_MOUSE tr|F6SBR5|F6SBR5\_MOUSE tr|F7ABZ6|F7ABZ6\_MOUSE tr|E9Q886|E9Q886\_MOUSE sp|Q3UEI1-2|PDE4C\_MOUSE sp|Q3UEI1|PDE4C\_MOUSE tr|E9Q3P4|E9Q3P4\_MOUSE sp|E9Q414|APOB\_MOUSE tr|E9Q1Y3|E9Q1Y3\_MOUSE sp|Q3KNY0|IGFN1\_MOUSE sp|Q71LX4|TLN2\_MOUSE tr|E9PZ16|E9PZ16\_MOUSE tr|B1B0C7|B1B0C7\_MOUSE tr|E9Q586|E9Q586\_MOUSE tr|E9Q3M3|E9Q3M3\_MOUSE sp|O08788|DCTN1\_MOUSE tr|D3YX34|D3YX34\_MOUSE tr|E9PVZ8|E9PVZ8\_MOUSE sp|Q8BTI8-3|SRRM2\_MOUSE sp|Q8BTI8|SRRM2\_MOUSE tr|H3BJQ8|H3BJQ8\_MOUSE tr|D3Z1W6|D3Z1W6\_MOUSE sp|Q8BW94|DYH3\_MOUSE sp|P49025-3|CTRO\_MOUSE tr|D3YU89|D3YU89\_MOUSE sp|P49025-5|CTRO\_MOUSE tr|D3Z1U0|D3Z1U0\_MOUSE tr|E9QL53|E9QL53\_MOUSE sp|P49025|CTRO\_MOUSE sp|Q8VHE6|DYH5\_MOUSE tr|E9Q098|E9Q098\_MOUSE sp|Q9QXS1-11|PLEC\_MOUSE tr|E9Q160|E9Q160\_MOUSE tr|A2AFN3|A2AFN3\_MOUSE sp|Q9QXS1-12|PLEC\_MOUSE tr|F7AYW2|F7AYW2\_MOUSE sp|Q8C3J5|DOCK2\_MOUSE tr|G3X9F2|G3X9F2\_MOUSE sp|P79457|UTY\_MOUSE sp|Q2EG98-8|PK1L3\_MOUSE sp|Q2EG98-2|PK1L3\_MOUSE tr|B1AR51|B1AR51\_MOUSE sp|Q3V3I2|GNAT3\_MOUSE sp|P70670|NACAM\_MOUSE tr|Q3V1N2|Q3V1N2\_MOUSE tr|E9Q4E2|E9Q4E2\_MOUSE sp|Q99ME3|SNCAP\_MOUSE tr|G5E848|G5E848\_MOUSE tr|J3QNK1|J3QNK1\_MOUSE sp|Q924A2|CIC\_MOUSE sp|Q05793|PGBM\_MOUSE sp|Q704Y3|TRPV1\_MOUSE tr|Q543M9|Q543M9\_MOUSE sp|Q704Y3-2|TRPV1\_MOUSE sp|Q99PW4|PRPK\_MOUSE sp|O70546|KDM6A\_MOUSE sp|O70546-2|KDM6A\_MOUSE tr|E9PWW8|E9PWW8\_MOUSE tr|Q7TS90|Q7TS90\_MOUSE tr|E9PX50|E9PX50\_MOUSE tr|S4R2P9|S4R2P9\_MOUSE tr|D3YYQ8|D3YYQ8\_MOUSE sp|Q4U2R1|HERC2\_MOUSE tr|D3Z3R4|D3Z3R4\_MOUSE sp|Q4U2R1-2|HERC2\_MOUSE sp|Q6ZQA0-2|NBEL2\_MOUSE tr|E9Q9L6|E9Q9L6\_MOUSE tr|D3Z4I0|D3Z4I0\_MOUSE tr|D3Z3Y1|D3Z3Y1\_MOUSE sp|Q8VHY0|CSPG4\_MOUSE tr|E9QAH1|E9QAH1\_MOUSE tr|H3BKH3|H3BKH3\_MOUSE sp|Q8VI24|SATB2\_MOUSE sp|Q8VI24-2|SATB2\_MOUSE tr|B8JJE0|B8JJE0\_MOUSE sp|Q8VHN7|GPR98\_MOUSE sp|Q8CAL5|GPC5\_MOUSE sp|Q91XQ0|DYH8\_MOUSE sp|Q99MY8|ASH1L\_MOUSE tr|E9QKK1|E9QKK1\_MOUSE sp|Q6RT24|CENPE\_MOUSE sp|Q8VHY0-3|CSPG4\_MOUSE sp|Q9JI18|LRP1B\_MOUSE tr|A2API5|A2API5\_MOUSE tr|E9Q6E9|E9Q6E9\_MOUSE sp|Q8R0W0|EPIPL\_MOUSE tr|E9Q474|E9Q474\_MOUSE tr|E9Q394|E9Q394\_MOUSE sp|P70336|ROCK2\_MOUSE tr|F8VPK5|F8VPK5\_MOUSE sp|P70336-2|ROCK2\_MOUSE sp|A2A870|FBF1\_MOUSE tr|Q5U452|Q5U452\_MOUSE sp|P83741-2|WNK1\_MOUSE sp|P83741-3|WNK1\_MOUSE sp|A2CG49|KALRN\_MOUSE sp|A2CG49-7|KALRN\_MOUSE tr|E9QK16|E9QK16\_MOUSE sp|Q8BNA6|FAT3\_MOUSE sp|Q9Z0E0-2|NCDN\_MOUSE sp|Q9Z0E0|NCDN\_MOUSE tr|D6RI94|D6RI94\_MOUSE tr|F6THL5|F6THL5\_MOUSE tr|D6RIL0|D6RIL0\_MOUSE tr|A0A087WSP0|A0A087WSP0\_MOUSE sp|O35219-3|KCNH2\_MOUSE Q99456 sp|Q9QXZ0-4|MACF1\_MOUSE tr|F6Q750|F6Q750\_MOUSE tr|E9QNP1|E9QNP1\_MOUSE tr|F7ACR9|F7ACR9\_MOUSE tr|D3YTQ6|D3YTQ6\_MOUSE tr|D3Z2G1|D3Z2G1\_MOUSE sp|Q8BTI8-2|SRRM2\_MOUSE sp|Q6P5D8|SMHD1\_MOUSE tr|G3UWM5|G3UWM5\_MOUSE sp|E9Q8I9-2|FRY\_MOUSE sp|E9Q8I9|FRY\_MOUSE sp|Q30D77|COOA1\_MOUSE tr|Q8VD73|Q8VD73\_MOUSE sp|Q6URW6-3|MYH14\_MOUSE sp|Q80TN7|NAV3\_MOUSE tr|E9QMF5|E9QMF5\_MOUSE tr|E9QK20|E9QK20\_MOUSE tr|A0A087WS83|A0A087WS83\_MOUSE sp|Q61290|CAC1E\_MOUSE sp|E9PVD3|PCD16\_MOUSE sp|Q8CJ27|ASPM\_MOUSE Q01546 sp|Q9WUF3|C8AP2\_MOUSE tr|A2AT70|A2AT70\_MOUSE sp|Q80X19|COEA1\_MOUSE sp|Q80X19-2|COEA1\_MOUSE tr|B7ZNH7|B7ZNH7\_MOUSE tr|K3W4R4|K3W4R4\_MOUSE sp|A2ARZ3|FSIP2\_MOUSE sp|Q4KWH5|PLCH1\_MOUSE tr|I1E4X4|I1E4X4\_MOUSE tr|H3BKK4|H3BKK4\_MOUSE sp|Q4KWH5-2|PLCH1\_MOUSE tr|I1E4X6|I1E4X6\_MOUSE sp|Q9QXS1-8|PLEC\_MOUSE sp|Q9QXS1-5|PLEC\_MOUSE sp|Q9QXS1-13|PLEC\_MOUSE sp|Q9QXS1-15|PLEC\_MOUSE sp|Q9QXS1-16|PLEC\_MOUSE sp|Q9QXS1-6|PLEC\_MOUSE sp|Q9QXS1-7|PLEC\_MOUSE sp|Q9QXS1-3|PLEC\_MOUSE sp|Q9QXS1-9|PLEC\_MOUSE sp|Q9QXS1-2|PLEC\_MOUSE tr|E9Q3W4|E9Q3W4\_MOUSE sp|Q9QXS1-10|PLEC\_MOUSE sp|Q9QXS1-14|PLEC\_MOUSE sp|Q9QXS1|PLEC\_MOUSE sp|Q9QXS1-4|PLEC\_MOUSE sp|Q91ZU6-5|DYST\_MOUSE tr|E9PYL4|E9PYL4\_MOUSE sp|O70496|CLCN7\_MOUSE tr|E9PX70|E9PX70\_MOUSE sp|Q60847-2|COCA1\_MOUSE sp|Q60847-5|COCA1\_MOUSE sp|Q60847|COCA1\_MOUSE sp|O54754|AOXA\_MOUSE tr|G3X8P9|G3X8P9\_MOUSE tr|E9PYK8|E9PYK8\_MOUSE tr|E9QAQ7|E9QAQ7\_MOUSE sp|A2BH40|ARI1A\_MOUSE sp|Q5DTU0-2|AF1L2\_MOUSE sp|Q5DTU0|AF1L2\_MOUSE sp|Q9JKZ2|SC5A3\_MOUSE tr|A2AN33|A2AN33\_MOUSE tr|F8WHT3|F8WHT3\_MOUSE sp|Q9Z329-3|ITPR2\_MOUSE sp|Q9Z329|ITPR2\_MOUSE sp|Q8C3Y4|KNTC1\_MOUSE sp|A2AJ76|HMCN2\_MOUSE sp|Q4G0F8|UBN1\_MOUSE sp|Q4G0F8-2|UBN1\_MOUSE sp|Q4G0F8-3|UBN1\_MOUSE tr|E9PX48|E9PX48\_MOUSE tr|A2A9M5|A2A9M5\_MOUSE sp|Q8R1A4|DOCK7\_MOUSE sp|Q8R1A4-2|DOCK7\_MOUSE sp|Q8BW94-2|DYH3\_MOUSE sp|A6H5Z3|EXC6B\_MOUSE sp|A6H5Z3-2|EXC6B\_MOUSE sp|Q9Z2G1|FM1AA\_MOUSE sp|Q8BG30|NELFA\_MOUSE tr|H9KV00|H9KV00\_MOUSE sp|Q6ZPV2|INO80\_MOUSE sp|Q6ZPV2-2|INO80\_MOUSE sp|Q60603|KCNH1\_MOUSE tr|Q3UHC9|Q3UHC9\_MOUSE sp|Q80T79|CSMD3\_MOUSE sp|Q80T79-1|CSMD3\_MOUSE sp|Q3UHF7|ZEP2\_MOUSE sp|Q811L6|MAST4\_MOUSE tr|E9QPR4|E9QPR4\_MOUSE tr|E9PWX8|E9PWX8\_MOUSE tr|Q6DFZ1|Q6DFZ1\_MOUSE tr|Q6A099|Q6A099\_MOUSE sp|Q6IME9|K2C72\_MOUSE Q6IME9 sp|Q8BMA5|NPAT\_MOUSE tr|E9QAF2|E9QAF2\_MOUSE tr|E9PWJ7|E9PWJ7\_MOUSE sp|Q9D5S7|LRGUK\_MOUSE tr|E9QPW4|E9QPW4\_MOUSE sp|Q8BQ48-2|K1731\_MOUSE sp|Q8BQ48|K1731\_MOUSE sp|Q8BQ48-5|K1731\_MOUSE sp|Q8BQ48-3|K1731\_MOUSE sp|Q8BQ48-6|K1731\_MOUSE sp|Q5DTH5|TSH1\_MOUSE sp|Q9JLC8-3|SACS\_MOUSE tr|E9QNY8|E9QNY8\_MOUSE sp|Q9JLC8|SACS\_MOUSE sp|A2ARV4|LRP2\_MOUSE sp|Q8BRH4|KMT2C\_MOUSE tr|F8WI37|F8WI37\_MOUSE sp|Q9CTY5|MICU3\_MOUSE tr|Q8K4L2|Q8K4L2\_MOUSE sp|Q8JZM8|MUC4\_MOUSE tr|E9Q5M6|E9Q5M6\_MOUSE sp|Q8K0T7|UN13C\_MOUSE Q92764 tr|Q8R266|Q8R266\_MOUSE sp|Q9D168|INT12\_MOUSE tr|F6TP58|F6TP58\_MOUSE sp|P57016|LAD1\_MOUSE tr|F8VPN2|F8VPN2\_MOUSE sp|Q91XQ0-2|DYH8\_MOUSE sp|Q9QYC1|PCX1\_MOUSE sp|Q9QYC1-2|PCX1\_MOUSE tr|E9Q8T7|E9Q8T7\_MOUSE tr|E9PZ56|E9PZ56\_MOUSE tr|F6SUM2|F6SUM2\_MOUSE sp|Q9CR37|PPDPF\_MOUSE sp|Q9QYX7|PCLO\_MOUSE sp|Q8BX70-3|VP13C\_MOUSE sp|Q8BX70|VP13C\_MOUSE sp|Q8BX70-2|VP13C\_MOUSE tr|E9Q9J0|E9Q9J0\_MOUSE tr|E9QNP0|E9QNP0\_MOUSE tr|J3QK04|J3QK04\_MOUSE tr|E9Q4P0|E9Q4P0\_MOUSE sp|P62984|RL40\_MOUSE tr|E9Q5F6|E9Q5F6\_MOUSE tr|Q5SX22|Q5SX22\_MOUSE sp|P0CG49|UBB\_MOUSE sp|P62983|RS27A\_MOUSE sp|Q61315-3|APC\_MOUSE tr|B2RUG9|B2RUG9\_MOUSE sp|Q61315|APC\_MOUSE sp|P70211-2|DCC\_MOUSE sp|P70211|DCC\_MOUSE sp|A2BH40-3|ARI1A\_MOUSE tr|F7DC70|F7DC70\_MOUSE tr|F6QYT9|F6QYT9\_MOUSE sp|A2CG49-2|KALRN\_MOUSE tr|E9PVB7|E9PVB7\_MOUSE sp|P07744|K2C4\_MOUSE P07744 tr|F6XC25|F6XC25\_MOUSE sp|Q8BRN9|C2D1B\_MOUSE sp|Q6NZP1|ZRAB3\_MOUSE tr|E9Q202|E9Q202\_MOUSE sp|Q91Z69|SRGP1\_MOUSE tr|D3YZW1|D3YZW1\_MOUSE sp|Q5HZG4|TAF3\_MOUSE sp|P00520|ABL1\_MOUSE sp|P00520-2|ABL1\_MOUSE sp|P00520-4|ABL1\_MOUSE sp|P00520-3|ABL1\_MOUSE tr|Q5RIM6|Q5RIM6\_MOUSE sp|A1L317|K1C24\_MOUSE tr|D3Z2X2|D3Z2X2\_MOUSE sp|P70335-2|ROCK1\_MOUSE sp|P70335|ROCK1\_MOUSE tr|E9PY14|E9PY14\_MOUSE tr|E9PUV7|E9PUV7\_MOUSE tr|F2Z4A3|F2Z4A3\_MOUSE sp|Q9ES97|RTN3\_MOUSE sp|Q9ES97-2|RTN3\_MOUSE tr|Q640N4|Q640N4\_MOUSE sp|Q8K5B8|HXC12\_MOUSE O76014 tr|E9PYH0|E9PYH0\_MOUSE tr|E9Q7N9|E9Q7N9\_MOUSE tr|Z4YJD4|Z4YJD4\_MOUSE sp|Q6EDY6|LR16A\_MOUSE tr|D3Z030|D3Z030\_MOUSE sp|Q6EDY6-3|LR16A\_MOUSE tr|K3W4R2|K3W4R2\_MOUSE sp|Q6URW6-2|MYH14\_MOUSE sp|Q6URW6|MYH14\_MOUSE tr|E9QAN8|E9QAN8\_MOUSE sp|E9PZJ8|ASCC3\_MOUSE sp|F7A4A7|OTOGL\_MOUSE sp|F7A4A7-2|OTOGL\_MOUSE sp|Q7TPM1|PRC2B\_MOUSE tr|F7AAP4|F7AAP4\_MOUSE tr|E9Q828|E9Q828\_MOUSE sp|Q62168|K1H2\_MOUSE tr|B1ATJ5|B1ATJ5\_MOUSE A2AB72 Q7Z3Y9 sp|Q8VCW2|K1C25\_MOUSE sp|Q62059|CSPG2\_MOUSE sp|Q7TSJ2|MAP6\_MOUSE tr|Z4YK74|Z4YK74\_MOUSE sp|Q8C0T1|FM1AB\_MOUSE sp|Q60867|NDF1\_MOUSE O76015 sp|Q91VS8|FARP2\_MOUSE sp|Q32KG4-2|RGAG1\_MOUSE sp|Q32KG4|RGAG1\_MOUSE sp|Q99NE5|RIMS1\_MOUSE tr|F6VBR4|F6VBR4\_MOUSE sp|A2AAJ9|OBSCN\_MOUSE tr|H7BX05|H7BX05\_MOUSE tr|E0CYG3|E0CYG3\_MOUSE sp|P97929|BRCA2\_MOUSE tr|G8JL85|G8JL85\_MOUSE tr|A2AIS0|A2AIS0\_MOUSE tr|A2AIR7|A2AIR7\_MOUSE sp|O55017|CAC1B\_MOUSE sp|O55017-2|CAC1B\_MOUSE tr|A2AIR9|A2AIR9\_MOUSE sp|Q60611|SATB1\_MOUSE tr|E9Q1N0|E9Q1N0\_MOUSE tr|F8VQK0|F8VQK0\_MOUSE tr|A2AUY4|A2AUY4\_MOUSE sp|P26040|EZRI\_MOUSE sp|Q80WC3|TNC18\_MOUSE sp|Q9D2Z8|KIF12\_MOUSE sp|P97313|PRKDC\_MOUSE sp|Q5SSE9|ABCAD\_MOUSE sp|Q5DU05|CE164\_MOUSE sp|O89001|CBPD\_MOUSE sp|Q3TDD9|PPR21\_MOUSE tr|E9PWJ0|E9PWJ0\_MOUSE sp|Q9JLC8-2|SACS\_MOUSE tr|E9Q411|E9Q411\_MOUSE sp|Q497I4|KRT35\_MOUSE Q497I4 sp|Q3KNY0-2|IGFN1\_MOUSE tr|E9Q4F7|E9Q4F7\_MOUSE tr|E9Q4F8|E9Q4F8\_MOUSE tr|D3YVU3|D3YVU3\_MOUSE sp|Q5DU05-2|CE164\_MOUSE tr|E9Q1M1|E9Q1M1\_MOUSE sp|Q7TMY8|HUWE1\_MOUSE sp|Q7TMY8-3|HUWE1\_MOUSE tr|A2AFQ0|A2AFQ0\_MOUSE Q9H552 tr|E9Q4D4|E9Q4D4\_MOUSE sp|Q7TN88|PK1L2\_MOUSE tr|E9QPG2|E9QPG2\_MOUSE tr|E9Q2B2|E9Q2B2\_MOUSE sp|G3X982|AOXC\_MOUSE tr|B1ARM8|B1ARM8\_MOUSE sp|Q5NBX1-2|COBL\_MOUSE sp|Q5NBX1|COBL\_MOUSE tr|G3UWY3|G3UWY3\_MOUSE tr|G5E8P4|G5E8P4\_MOUSE sp|Q5NBX1-3|COBL\_MOUSE tr|E9PWQ3|E9PWQ3\_MOUSE sp|Q3V1M1|IGS10\_MOUSE tr|G3X8T7|G3X8T7\_MOUSE sp|Q9ER60|SCN4A\_MOUSE Q3SY84 tr|Q6Q477|Q6Q477\_MOUSE tr|Q32ME1|Q32ME1\_MOUSE sp|P97382|KCAB3\_MOUSE tr|K3W4Q5|K3W4Q5\_MOUSE sp|Q3V1N1|MFHA1\_MOUSE tr|B9EKJ3|B9EKJ3\_MOUSE sp|Q80TL7-2|MON2\_MOUSE sp|Q80TL7|MON2\_MOUSE sp|Q8BWR4-2|UBP40\_MOUSE sp|Q5SX39|MYH4\_MOUSE tr|E9PZ36|E9PZ36\_MOUSE sp|Q9JJV9-2|SCN5A\_MOUSE sp|Q9JJV9|SCN5A\_MOUSE tr|K3W4N7|K3W4N7\_MOUSE sp|Q6ZPE2|MTMR5\_MOUSE sp|A2ALK8|PTN3\_MOUSE sp|B1AQ75|KRT36\_MOUSE sp|B9EKR1|PRPTZ\_MOUSE tr|I7HPW8|I7HPW8\_MOUSE sp|Q8K284|TF3C1\_MOUSE tr|Q8C5J0|Q8C5J0\_MOUSE sp|A2APV2-2|FMNL2\_MOUSE tr|E9PXE6|E9PXE6\_MOUSE sp|Q8CH77-2|NAV1\_MOUSE tr|Q6IFZ8|Q6IFZ8\_MOUSE tr|H7BWZ9|H7BWZ9\_MOUSE sp|P18872-2|GNAO\_MOUSE Q2M2I5 tr|Q543V3|Q543V3\_MOUSE sp|P35569|IRS1\_MOUSE sp|Q7SIG6|ASAP2\_MOUSE tr|E9PX52|E9PX52\_MOUSE tr|D3YX85|D3YX85\_MOUSE sp|Q61315-2|APC\_MOUSE tr|E9QLQ9|E9QLQ9\_MOUSE sp|Q61315-4|APC\_MOUSE sp|Q9D504|ANKR7\_MOUSE sp|Q5SYL3|K0100\_MOUSE sp|Q80U22|RUSC2\_MOUSE sp|Q76HP3|T132D\_MOUSE tr|E9QNI1|E9QNI1\_MOUSE sp|A2AUM9|CE152\_MOUSE tr|E9Q504|E9Q504\_MOUSE sp|Q9D2H6|SP2\_MOUSE tr|Q8BNQ4|Q8BNQ4\_MOUSE sp|Q9D2H6-2|SP2\_MOUSE tr|E9Q8F8|E9Q8F8\_MOUSE sp|Q3UHA3|SPTCS\_MOUSE ENSEMBL:ENSP00000377550 tr|G8JL63|G8JL63\_MOUSE sp|Q0KK59|UNC79\_MOUSE tr|J3QNP0|J3QNP0\_MOUSE tr|E5CYJ9|E5CYJ9\_MOUSE tr|Q3V1Z0|Q3V1Z0\_MOUSE sp|Q8BGE5|FANCM\_MOUSE sp|Q6PR54-3|RIF1\_MOUSE sp|Q6PR54|RIF1\_MOUSE sp|Q6PR54-2|RIF1\_MOUSE sp|Q06194|FA8\_MOUSE tr|B2RRC9|B2RRC9\_MOUSE sp|Q9Z2B5|E2AK3\_MOUSE tr|E9QQ30|E9QQ30\_MOUSE tr|D3Z6V3|D3Z6V3\_MOUSE tr|E9Q309|E9Q309\_MOUSE sp|Q99NI3|GT2D2\_MOUSE sp|P70211-3|DCC\_MOUSE sp|Q03173|ENAH\_MOUSE tr|E9QKR1|E9QKR1\_MOUSE sp|Q03173-4|ENAH\_MOUSE sp|Q03173-5|ENAH\_MOUSE tr|J3QNM3|J3QNM3\_MOUSE tr|E9QLZ9|E9QLZ9\_MOUSE tr|E9QKQ9|E9QKQ9\_MOUSE sp|Q6P9L6|KIF15\_MOUSE sp|Q99NE5-3|RIMS1\_MOUSE tr|F6VBT9|F6VBT9\_MOUSE tr|F6VBV0|F6VBV0\_MOUSE sp|Q99NE5-5|RIMS1\_MOUSE tr|F6Y0S3|F6Y0S3\_MOUSE sp|Q99NE5-4|RIMS1\_MOUSE sp|Q60575-3|KIF1B\_MOUSE tr|A2AH75|A2AH75\_MOUSE Q14525 Q9UE12 Q15323 tr|A2A9H6|A2A9H6\_MOUSE tr|G3X904|G3X904\_MOUSE sp|Q9JIL5|TULP4\_MOUSE sp|Q8C9J3|SPEF2\_MOUSE sp|Q5SW19|CLU\_MOUSE sp|Q80U22-2|RUSC2\_MOUSE tr|Q3TZK4|Q3TZK4\_MOUSE sp|Q6PDK2|KMT2D\_MOUSE sp|Q640L5|CCD18\_MOUSE O76013 tr|F6VTL9|F6VTL9\_MOUSE sp|A2AJ88|PLPL7\_MOUSE tr|F8VPR2|F8VPR2\_MOUSE sp|A2APV2-3|FMNL2\_MOUSE sp|A2APV2|FMNL2\_MOUSE sp|E9Q4Z2|ACACB\_MOUSE sp|O88379|BAZ1A\_MOUSE tr|G3UWZ0|G3UWZ0\_MOUSE sp|A2AVR2|MROH7\_MOUSE tr|O35452|O35452\_MOUSE tr|Q3UQE8|Q3UQE8\_MOUSE Q14532 sp|Q8K5C0|GRHL2\_MOUSE tr|Q9DCN4|Q9DCN4\_MOUSE tr|H3BJZ2|H3BJZ2\_MOUSE sp|Q921C3-2|BRWD1\_MOUSE tr|E9Q2N1|E9Q2N1\_MOUSE sp|Q921C3|BRWD1\_MOUSE sp|Q8BWR4-3|UBP40\_MOUSE sp|Q8BWR4|UBP40\_MOUSE sp|Q6ZPE2-2|MTMR5\_MOUSE sp|Q5XG71|UTP20\_MOUSE tr|E9QK83|E9QK83\_MOUSE sp|Q505K2|F16A1\_MOUSE sp|Q6P8Y7|SNX31\_MOUSE sp|Q6P8Y7-2|SNX31\_MOUSE Q7RTS7 sp|P60330|ESPL1\_MOUSE tr|S4R294|S4R294\_MOUSE tr|B0V2M3|B0V2M3\_MOUSE sp|Q99PP2|ZN318\_MOUSE sp|A2AGH6-2|MED12\_MOUSE sp|A2AGH6|MED12\_MOUSE tr|A2AGH8|A2AGH8\_MOUSE tr|A2AGH9|A2AGH9\_MOUSE tr|F6SMY7|F6SMY7\_MOUSE sp|Q6QI06|RICTR\_MOUSE tr|L0N7N1|L0N7N1\_MOUSE tr|E9Q3T3|E9Q3T3\_MOUSE sp|Q99M73|KRT84\_MOUSE sp|Q8C9J3-2|SPEF2\_MOUSE sp|Q8BRH4-2|KMT2C\_MOUSE sp|Q5DU05-3|CE164\_MOUSE tr|F8VQK3|F8VQK3\_MOUSE tr|E9Q1W7|E9Q1W7\_MOUSE tr|E9Q9C6|E9Q9C6\_MOUSE tr|E9Q0B5|E9Q0B5\_MOUSE Q6ISB0 Q9NSB2 sp|Q3UHK6-2|TEN4\_MOUSE tr|H3BLM8|H3BLM8\_MOUSE tr|Q8CGZ7|Q8CGZ7\_MOUSE sp|Q925F2|ESAM\_MOUSE tr|F8VPP8|F8VPP8\_MOUSE tr|A2ALK9|A2ALK9\_MOUSE sp|Q62205|SCN9A\_MOUSE tr|B1AYL0|B1AYL0\_MOUSE tr|F8VPQ7|F8VPQ7\_MOUSE tr|E9PW82|E9PW82\_MOUSE sp|Q7TMY8-4|HUWE1\_MOUSE tr|E9Q2T3|E9Q2T3\_MOUSE tr|J3QQ16|J3QQ16\_MOUSE tr|A0A087WS16|A0A087WS16\_MOUSE tr|F6SSP6|F6SSP6\_MOUSE sp|Q8BL99|DOP1\_MOUSE sp|Q8BL99-5|DOP1\_MOUSE sp|Q8BL99-6|DOP1\_MOUSE tr|E9QNW4|E9QNW4\_MOUSE sp|Q8CD54|PIEZ2\_MOUSE sp|Q60575-2|KIF1B\_MOUSE P12035 sp|Q61193|RGL2\_MOUSE tr|Q8BML3|Q8BML3\_MOUSE tr|E9Q6Q8|E9Q6Q8\_MOUSE sp|Q8BYJ6|TBCD4\_MOUSE tr|F6SUV7|F6SUV7\_MOUSE tr|E0CX53|E0CX53\_MOUSE sp|P08032|SPTA1\_MOUSE sp|P06684|CO5\_MOUSE sp|A2AIV2|VIR\_MOUSE tr|E9PZY8|E9PZY8\_MOUSE sp|Q4ACU6|SHAN3\_MOUSE sp|Q8BFX3|KCTD3\_MOUSE sp|P97379-2|G3BP2\_MOUSE sp|P97379|G3BP2\_MOUSE sp|Q8CH77|NAV1\_MOUSE sp|Q8CJH3|PLXB1\_MOUSE sp|P33174|KIF4\_MOUSE tr|E9Q7Q0|E9Q7Q0\_MOUSE sp|Q5SWU9-2|ACACA\_MOUSE sp|Q5SWU9|ACACA\_MOUSE sp|P68134|ACTS\_MOUSE sp|Q6KAR6|EXOC3\_MOUSE sp|Q61483|DLL1\_MOUSE sp|Q9QUG2-2|POLK\_MOUSE tr|Q5Q9H7|Q5Q9H7\_MOUSE sp|Q9QUG2|POLK\_MOUSE tr|Q91Y11|Q91Y11\_MOUSE sp|Q8K411-2|PREP\_MOUSE sp|Q8K411|PREP\_MOUSE tr|E9PX95|E9PX95\_MOUSE sp|Q9JHU4|DYHC1\_MOUSE sp|Q60575|KIF1B\_MOUSE sp|A2AQH4|BCORL\_MOUSE sp|Q0QWG9-2|GRD2I\_MOUSE sp|Q0QWG9|GRD2I\_MOUSE sp|Q0QWG9-3|GRD2I\_MOUSE tr|E9PUJ6|E9PUJ6\_MOUSE sp|Q62414|NDF2\_MOUSE tr|D3Z4R2|D3Z4R2\_MOUSE sp|P52479|UBP10\_MOUSE sp|P52479-2|UBP10\_MOUSE tr|F8VQH0|F8VQH0\_MOUSE sp|Q8BL48|UNK\_MOUSE sp|Q8BL48-2|UNK\_MOUSE tr|E9PV26|E9PV26\_MOUSE sp|Q6ZQJ5|DNA2\_MOUSE sp|Q80WJ6|MRP9\_MOUSE sp|Q6QI06-2|RICTR\_MOUSE sp|Q5SYD0|MYO1D\_MOUSE sp|Q5SYD0-2|MYO1D\_MOUSE sp|Q9QX47|SON\_MOUSE sp|Q9QX47-3|SON\_MOUSE sp|Q70FJ1-2|AKAP9\_MOUSE sp|Q70FJ1|AKAP9\_MOUSE sp|Q70FJ1-3|AKAP9\_MOUSE sp|Q9QYX7-2|PCLO\_MOUSE sp|Q8BJ34-4|MARF1\_MOUSE sp|Q8BJ34-6|MARF1\_MOUSE sp|Q8BJ34|MARF1\_MOUSE sp|Q8BJ34-3|MARF1\_MOUSE sp|A3KFM7|CHD6\_MOUSE sp|A3KFM7-2|CHD6\_MOUSE sp|O35600|ABCA4\_MOUSE sp|P70429|EVL\_MOUSE tr|F8WJB9|F8WJB9\_MOUSE sp|P70429-2|EVL\_MOUSE tr|E9PVP4|E9PVP4\_MOUSE sp|Q8K019-2|BCLF1\_MOUSE sp|Q8K019|BCLF1\_MOUSE tr|A0A087WQ48|A0A087WQ48\_MOUSE tr|H7BX79|H7BX79\_MOUSE sp|Q0VG85|CC162\_MOUSE sp|Q5SW19-2|CLU\_MOUSE sp|P83741-4|WNK1\_MOUSE sp|P83741|WNK1\_MOUSE Q7RTT2 Q8N1N4-2 tr|A2APX8|A2APX8\_MOUSE tr|A2APX6|A2APX6\_MOUSE tr|A2APX7|A2APX7\_MOUSE sp|Q2MKA5-2|ACHA5\_MOUSE sp|Q2MKA5|ACHA5\_MOUSE tr|G3UVV3|G3UVV3\_MOUSE sp|Q8VDD9|PHIP\_MOUSE tr|A2AI20|A2AI20\_MOUSE tr|A2AI21|A2AI21\_MOUSE tr|A2AI16|A2AI16\_MOUSE tr|A2AI14|A2AI14\_MOUSE sp|Q8BR76|MKS3\_MOUSE tr|A2AJP5|A2AJP5\_MOUSE sp|Q9ERU9|RBP2\_MOUSE tr|E9PWT1|E9PWT1\_MOUSE tr|E9PZA7|E9PZA7\_MOUSE tr|E9QLK7|E9QLK7\_MOUSE tr|F7CGG2|F7CGG2\_MOUSE sp|Q60974|NCOR1\_MOUSE tr|A2ASY0|A2ASY0\_MOUSE tr|F6U7V1|F6U7V1\_MOUSE sp|E9Q401|RYR2\_MOUSE sp|Q8BW94-3|DYH3\_MOUSE tr|Q8VGW2|Q8VGW2\_MOUSE sp|Q91ZX7|LRP1\_MOUSE sp|Q8BM55|TM214\_MOUSE tr|D3Z6S1|D3Z6S1\_MOUSE tr|A3KGJ7|A3KGJ7\_MOUSE tr|E9Q5A8|E9Q5A8\_MOUSE sp|Q60952|CP250\_MOUSE sp|Q61169|GATA6\_MOUSE sp|Q8CDM4|CCD73\_MOUSE sp|Q8C115-3|PKHH2\_MOUSE sp|Q8C115|PKHH2\_MOUSE Q14CN4-1 tr|B9EHJ3|B9EHJ3\_MOUSE sp|P39447|ZO1\_MOUSE tr|E9QMK2|E9QMK2\_MOUSE sp|P70365-3|NCOA1\_MOUSE sp|P70365|NCOA1\_MOUSE sp|P70365-4|NCOA1\_MOUSE sp|P70365-2|NCOA1\_MOUSE sp|Q9DCV7|K2C7\_MOUSE Q9DCV7 sp|P02463|CO4A1\_MOUSE sp|Q8K411-3|PREP\_MOUSE sp|Q8BQ48-4|K1731\_MOUSE sp|Q80XJ3|TTC28\_MOUSE sp|Q9ESC8|AFF4\_MOUSE tr|E9PXX8|E9PXX8\_MOUSE sp|Q8C0R9|LRRD1\_MOUSE sp|Q80SX8|PIF1\_MOUSE tr|E9Q718|E9Q718\_MOUSE tr|E9Q390|E9Q390\_MOUSE sp|Q69ZN7|MYOF\_MOUSE tr|E9PV66|E9PV66\_MOUSE tr|J3QMK1|J3QMK1\_MOUSE sp|Q02566|MYH6\_MOUSE tr|B2RQ57|B2RQ57\_MOUSE sp|Q62059-3|CSPG2\_MOUSE tr|Z4YJE4|Z4YJE4\_MOUSE sp|A2AAJ9-2|OBSCN\_MOUSE tr|E9QQ96|E9QQ96\_MOUSE tr|E9Q6L0|E9Q6L0\_MOUSE tr|A0A087WNW8|A0A087WNW8\_MOUSE tr|A0A087WQF1|A0A087WQF1\_MOUSE sp|Q6NS46|RRP5\_MOUSE sp|Q9Z160|COG1\_MOUSE sp|A2RT91|ANKAR\_MOUSE sp|P27546-2|MAP4\_MOUSE sp|P27546-3|MAP4\_MOUSE sp|P27546|MAP4\_MOUSE sp|Q80VW5-11|WHRN\_MOUSE tr|F6XR20|F6XR20\_MOUSE sp|Q9QX47-4|SON\_MOUSE tr|H9KV15|H9KV15\_MOUSE sp|Q9QX47-2|SON\_MOUSE sp|Q8C4V1|RHG24\_MOUSE tr|H9KV01|H9KV01\_MOUSE sp|Q8CB77|ELOA1\_MOUSE sp|Q8C9B9|DIDO1\_MOUSE sp|E9Q286|ICE1\_MOUSE tr|E9QLJ0|E9QLJ0\_MOUSE sp|Q569L8|CENPJ\_MOUSE sp|O35161|CELR1\_MOUSE sp|P40201|CHD1\_MOUSE tr|G3X937|G3X937\_MOUSE sp|Q921R8|S41A3\_MOUSE sp|Q921R8-2|S41A3\_MOUSE sp|P11881-4|ITPR1\_MOUSE sp|P11881-3|ITPR1\_MOUSE sp|P11881|ITPR1\_MOUSE sp|P11881-7|ITPR1\_MOUSE sp|P11881-5|ITPR1\_MOUSE sp|P11881-8|ITPR1\_MOUSE sp|P11881-2|ITPR1\_MOUSE sp|P11881-6|ITPR1\_MOUSE sp|Q3UHK6|TEN4\_MOUSE sp|Q3UHK6-4|TEN4\_MOUSE sp|Q3UHK6-3|TEN4\_MOUSE sp|Q8C4Y3|NELFB\_MOUSE sp|A2AJ88-2|PLPL7\_MOUSE tr|Q5F4T0|Q5F4T0\_MOUSE tr|Q5F4S9|Q5F4S9\_MOUSE tr|Q5F4S8|Q5F4S8\_MOUSE sp|Q69ZS7|HBS1L\_MOUSE tr|A0A087WNM1|A0A087WNM1\_MOUSE tr|A0A087WS59|A0A087WS59\_MOUSE sp|Q91Z67|SRGP2\_MOUSE tr|F8WJL9|F8WJL9\_MOUSE tr|B9EJ54|B9EJ54\_MOUSE sp|Q8C2A2-2|SEN54\_MOUSE sp|Q8C2A2|SEN54\_MOUSE sp|A2RSJ4|UH1BL\_MOUSE sp|Q9CWN7|CNO11\_MOUSE tr|Q3UH45|Q3UH45\_MOUSE sp|Q8R5K2|UBP33\_MOUSE sp|Q8R5K2-2|UBP33\_MOUSE tr|S4R209|S4R209\_MOUSE sp|O88447|KLC1\_MOUSE tr|E9PYJ7|E9PYJ7\_MOUSE sp|Q6ZPQ6-2|PITM2\_MOUSE sp|O35379|MRP1\_MOUSE sp|Q9Z1T5-2|DEAF1\_MOUSE sp|Q9Z1T5|DEAF1\_MOUSE tr|A2ASI5|A2ASI5\_MOUSE sp|Q60829-2|PPR1B\_MOUSE sp|Q60829|PPR1B\_MOUSE tr|Q8BR97|Q8BR97\_MOUSE sp|Q8K1Y2|KPCD3\_MOUSE sp|P48986|NDF6\_MOUSE sp|Q8BWQ4|CMTR2\_MOUSE tr|D3Z6I0|D3Z6I0\_MOUSE tr|Q5FWX6|Q5FWX6\_MOUSE sp|P83741-5|WNK1\_MOUSE sp|Q8BYJ6-5|TBCD4\_MOUSE sp|Q7M6Y6|MRO2B\_MOUSE sp|Q9R0M0-2|CELR2\_MOUSE sp|Q9R0M0|CELR2\_MOUSE tr|F7BWD7|F7BWD7\_MOUSE sp|Q64G17|AN32C\_MOUSE sp|B9EJA2|CTTB2\_MOUSE sp|B9EJA2-5|CTTB2\_MOUSE tr|G3UWD9|G3UWD9\_MOUSE tr|E9QP46|E9QP46\_MOUSE sp|Q6ZWQ0|SYNE2\_MOUSE sp|Q8CG46|SMC5\_MOUSE sp|Q8CG46-2|SMC5\_MOUSE tr|A2AQH3|A2AQH3\_MOUSE sp|Q9ERH7|HIPK3\_MOUSE sp|Q60974-2|NCOR1\_MOUSE tr|G3X956|G3X956\_MOUSE tr|A0A087WNU5|A0A087WNU5\_MOUSE sp|Q920B9|SP16H\_MOUSE tr|F6RS26|F6RS26\_MOUSE sp|O35945|AL1A7\_MOUSE sp|Q9EST5|AN32B\_MOUSE sp|Q9EST5-2|AN32B\_MOUSE sp|P49586|PCY1A\_MOUSE tr|F7CLE5|F7CLE5\_MOUSE tr|A0A087WP83|A0A087WP83\_MOUSE sp|Q8VDJ3|VIGLN\_MOUSE tr|E9PVA6|E9PVA6\_MOUSE sp|Q9JLQ2|GIT2\_MOUSE tr|A2AEE7|A2AEE7\_MOUSE sp|O88737-2|BSN\_MOUSE sp|O88737|BSN\_MOUSE sp|O35927-2|CTND2\_MOUSE tr|E9QKH8|E9QKH8\_MOUSE sp|O35927|CTND2\_MOUSE sp|O88466|ZN106\_MOUSE tr|D6RGQ2|D6RGQ2\_MOUSE tr|E9Q6R7|E9Q6R7\_MOUSE sp|Q8BFX3-2|KCTD3\_MOUSE tr|A2AP58|A2AP58\_MOUSE sp|Q6KCD5|NIPBL\_MOUSE tr|Q6NZL1|Q6NZL1\_MOUSE sp|Q6P4T0|ATG2A\_MOUSE sp|A2A9C3-2|SZT2\_MOUSE sp|A2A9C3|SZT2\_MOUSE sp|Q8CIR4|TRPM6\_MOUSE sp|Q91ZR4|NEK8\_MOUSE sp|Q3TLH4|PRC2C\_MOUSE tr|A2AI19|A2AI19\_MOUSE sp|P35438-2|NMDZ1\_MOUSE tr|A2AI17|A2AI17\_MOUSE sp|P35438|NMDZ1\_MOUSE sp|P51125-2|ICAL\_MOUSE sp|P51125-3|ICAL\_MOUSE tr|F8WJ40|F8WJ40\_MOUSE sp|Q61897|KT33B\_MOUSE sp|A6BLY7|K1C28\_MOUSE sp|Q6IFX2|K1C42\_MOUSE A2A5Y0 sp|Q6IFX3|K1C40\_MOUSE sp|Q61765|K1H1\_MOUSE REFSEQ:XP\_986630 Q6IFX2 H-INV:HIT000015463 tr|G3X8X6|G3X8X6\_MOUSE sp|Q62230|SN\_MOUSE sp|Q03146|DDR1\_MOUSE sp|Q8CGV9|TSH3\_MOUSE tr|E9QPN7|E9QPN7\_MOUSE tr|F6RNB2|F6RNB2\_MOUSE sp|P97393|RHG05\_MOUSE sp|Q8BWZ3|NAA25\_MOUSE sp|Q8BWZ3-2|NAA25\_MOUSE sp|Q80YR6|COM1\_MOUSE tr|E9QJU6|E9QJU6\_MOUSE sp|E5FYH1|TOPZ1\_MOUSE sp|Q8CH77-3|NAV1\_MOUSE tr|J3QP08|J3QP08\_MOUSE tr|A0A087WP11|A0A087WP11\_MOUSE tr|Q9DAD9|Q9DAD9\_MOUSE tr|D3Z4C0|D3Z4C0\_MOUSE tr|Q5M8Q2|Q5M8Q2\_MOUSE tr|Q810S6|Q810S6\_MOUSE sp|Q8CJF7|ELYS\_MOUSE sp|Q8BYH8|CHD9\_MOUSE sp|Q8BYH8-2|CHD9\_MOUSE sp|Q8CJ40-3|CROCC\_MOUSE sp|Q8CJ40|CROCC\_MOUSE sp|A2BH40-2|ARI1A\_MOUSE sp|A2BH40-4|ARI1A\_MOUSE sp|Q6ZPR6|IBTK\_MOUSE sp|Q6ZPR6-2|IBTK\_MOUSE sp|Q9D8C3-2|ALG13\_MOUSE sp|Q4QRL3-2|CC88B\_MOUSE sp|Q4QRL3|CC88B\_MOUSE sp|Q61329|ZFHX3\_MOUSE sp|Q32M02|FSCN2\_MOUSE sp|Q6PAR5-4|GAPD1\_MOUSE tr|E9QQ10|E9QQ10\_MOUSE sp|Q6PAR5-2|GAPD1\_MOUSE sp|Q6PAR5-6|GAPD1\_MOUSE sp|Q6PAR5|GAPD1\_MOUSE sp|Q6PAR5-5|GAPD1\_MOUSE tr|E9PYP2|E9PYP2\_MOUSE tr|F6QA95|F6QA95\_MOUSE sp|P20357|MTAP2\_MOUSE tr|H3BLL3|H3BLL3\_MOUSE tr|H3BJU9|H3BJU9\_MOUSE sp|Q921G6|LRCH4\_MOUSE tr|H3BLB4|H3BLB4\_MOUSE sp|Q5SWW4|MED13\_MOUSE sp|Q8R1W8-2|IMPG1\_MOUSE sp|Q8R1W8|IMPG1\_MOUSE sp|Q8R1W8-3|IMPG1\_MOUSE tr|E9Q7S1|E9Q7S1\_MOUSE tr|R4GML0|R4GML0\_MOUSE tr|E9Q5W2|E9Q5W2\_MOUSE tr|B1AY26|B1AY26\_MOUSE tr|H3BKE1|H3BKE1\_MOUSE sp|Q4KWH5-4|PLCH1\_MOUSE sp|Q4KWH5-3|PLCH1\_MOUSE tr|Q80ZX2|Q80ZX2\_MOUSE sp|P86792|CC21B\_MOUSE sp|P86793|CC21C\_MOUSE sp|Q6R0H7-2|GNAS1\_MOUSE tr|A2AT37|A2AT37\_MOUSE tr|E9PZ50|E9PZ50\_MOUSE sp|P61407|TDRD6\_MOUSE tr|F2Z429|F2Z429\_MOUSE tr|A0A087WRK0|A0A087WRK0\_MOUSE tr|J3QP81|J3QP81\_MOUSE tr|A0A087WS18|A0A087WS18\_MOUSE sp|P53564-4|CUX1\_MOUSE tr|H3BKF6|H3BKF6\_MOUSE sp|P53564|CUX1\_MOUSE tr|H3BJQ9|H3BJQ9\_MOUSE sp|P51125|ICAL\_MOUSE sp|Q8C8R3-2|ANK2\_MOUSE tr|S4R291|S4R291\_MOUSE tr|S4R285|S4R285\_MOUSE tr|S4R249|S4R249\_MOUSE tr|S4R1F9|S4R1F9\_MOUSE sp|Q8C8R3|ANK2\_MOUSE sp|Q8C8R3-4|ANK2\_MOUSE sp|Q2HXL6|EDEM3\_MOUSE tr|A0A087WQK5|A0A087WQK5\_MOUSE tr|B7ZNP0|B7ZNP0\_MOUSE tr|A0A087WR24|A0A087WR24\_MOUSE tr|V9GXT3|V9GXT3\_MOUSE tr|E9Q842|E9Q842\_MOUSE tr|V9GX91|V9GX91\_MOUSE sp|Q80VW5|WHRN\_MOUSE sp|Q9R0W9|ACHA6\_MOUSE sp|Q5SSZ5|TENS3\_MOUSE sp|Q8BH52|CR3L2\_MOUSE sp|Q8K2Z4-2|CND1\_MOUSE sp|Q8K2Z4|CND1\_MOUSE tr|E9PYT0|E9PYT0\_MOUSE tr|F6X8R3|F6X8R3\_MOUSE sp|E9PZQ0|RYR1\_MOUSE tr|K3W4M2|K3W4M2\_MOUSE tr|A0A087WQS9|A0A087WQS9\_MOUSE tr|G3UW82|G3UW82\_MOUSE sp|Q5SX40|MYH1\_MOUSE tr|E9QAJ6|E9QAJ6\_MOUSE sp|Q6PCP7|GP156\_MOUSE sp|Q0VGY8|TANC1\_MOUSE tr|E9QAF9|E9QAF9\_MOUSE sp|Q8VDR9|DOCK6\_MOUSE sp|Q3TLH4-5|PRC2C\_MOUSE tr|S4R2J9|S4R2J9\_MOUSE tr|E9Q6R4|E9Q6R4\_MOUSE tr|E9Q4N7|E9Q4N7\_MOUSE tr|B1B1D3|B1B1D3\_MOUSE sp|B9EJA2-4|CTTB2\_MOUSE tr|E9Q6M5|E9Q6M5\_MOUSE tr|Q91WJ2|Q91WJ2\_MOUSE sp|Q4JIM5|ABL2\_MOUSE sp|O08648|M3K4\_MOUSE sp|O08648-2|M3K4\_MOUSE sp|Q5SW19-3|CLU\_MOUSE tr|Z4YLI8|Z4YLI8\_MOUSE sp|Q9R053|SCNBA\_MOUSE sp|Q99N50-2|SYTL2\_MOUSE tr|Q8CBC8|Q8CBC8\_MOUSE tr|E9Q4K3|E9Q4K3\_MOUSE sp|P24288|BCAT1\_MOUSE tr|G5E893|G5E893\_MOUSE tr|A2AQ53|A2AQ53\_MOUSE tr|E9Q1Z0|E9Q1Z0\_MOUSE sp|Q7TMI3|UHRF2\_MOUSE sp|P62737|ACTA\_MOUSE sp|P68033|ACTC\_MOUSE sp|P63268|ACTH\_MOUSE tr|F8VQ05|F8VQ05\_MOUSE sp|P19246|NFH\_MOUSE sp|Q9DB41|GHC2\_MOUSE sp|Q9DB41-2|GHC2\_MOUSE sp|P10810|CD14\_MOUSE sp|Q80TY5|VP13B\_MOUSE tr|E9QKX5|E9QKX5\_MOUSE sp|Q2MV57|TECT2\_MOUSE sp|O08550|KMT2B\_MOUSE sp|Q2MV57-2|TECT2\_MOUSE P04258 sp|Q62230-3|SN\_MOUSE tr|H9KUZ3|H9KUZ3\_MOUSE tr|E9QAS4|E9QAS4\_MOUSE tr|E9QAS5|E9QAS5\_MOUSE sp|Q6PDQ2|CHD4\_MOUSE tr|Q3UGX2|Q3UGX2\_MOUSE sp|P15508|SPTB1\_MOUSE tr|E9Q397|E9Q397\_MOUSE tr|E9PVS5|E9PVS5\_MOUSE sp|Q9CWU0-2|TDR12\_MOUSE sp|P08775|RPB1\_MOUSE tr|J3QM81|J3QM81\_MOUSE sp|B1AY13|UBP24\_MOUSE tr|E9PV45|E9PV45\_MOUSE sp|A8C756|THADA\_MOUSE sp|Q7TSJ2-2|MAP6\_MOUSE tr|G3X9N1|G3X9N1\_MOUSE sp|Q64697|PTCA\_MOUSE P08729 Q3KNV1 tr|D3YZ21|D3YZ21\_MOUSE sp|Q5DTT3|F208B\_MOUSE tr|H3BLS0|H3BLS0\_MOUSE sp|P53564-2|CUX1\_MOUSE tr|E9QKE9|E9QKE9\_MOUSE sp|Q5DU41|LRC8B\_MOUSE tr|E9PWG2|E9PWG2\_MOUSE sp|Q6KCD5-2|NIPBL\_MOUSE sp|Q8CDI6|CD158\_MOUSE sp|Q3UFS0|ZY11B\_MOUSE tr|L7N274|L7N274\_MOUSE sp|Q99KK7|DPP3\_MOUSE sp|Q921M3-2|SF3B3\_MOUSE sp|Q8CD54-2|PIEZ2\_MOUSE tr|B1AWN6|B1AWN6\_MOUSE tr|J3QP58|J3QP58\_MOUSE sp|Q9EQC5|NTKL\_MOUSE tr|J3QPL7|J3QPL7\_MOUSE sp|Q7TPH6|MYCB2\_MOUSE sp|Q7TPH6-2|MYCB2\_MOUSE tr|E9PW34|E9PW34\_MOUSE sp|A2AGL3-2|RYR3\_MOUSE sp|A2AGL3|RYR3\_MOUSE sp|Q91VW5|GOGA4\_MOUSE tr|B1AUN8|B1AUN8\_MOUSE tr|Q8CAD1|Q8CAD1\_MOUSE sp|Q8VIC6|V1R51\_MOUSE sp|O88799|ZAN\_MOUSE tr|E9PWQ7|E9PWQ7\_MOUSE sp|Q6ZPZ3|ZC3H4\_MOUSE sp|Q6ZPZ3-2|ZC3H4\_MOUSE tr|Q7M739|Q7M739\_MOUSE sp|F6ZDS4|TPR\_MOUSE sp|Q6PIJ4|NFRKB\_MOUSE sp|Q80YV3|TRRAP\_MOUSE sp|Q921X6|RPC6\_MOUSE sp|Q7TSZ8|NACC1\_MOUSE sp|P02469|LAMB1\_MOUSE tr|E9QN70|E9QN70\_MOUSE sp|Q9Z2J0|S23A1\_MOUSE sp|Q9WTI7-3|MYO1C\_MOUSE sp|Q9WTI7-4|MYO1C\_MOUSE sp|Q9WTI7|MYO1C\_MOUSE tr|Q6W4W7|Q6W4W7\_MOUSE tr|E9Q4U7|E9Q4U7\_MOUSE tr|E9PUU8|E9PUU8\_MOUSE sp|Q8BPN8-2|DMXL2\_MOUSE tr|B0V2P5|B0V2P5\_MOUSE sp|Q8BPN8|DMXL2\_MOUSE tr|A2AQB2|A2AQB2\_MOUSE sp|Q8BJ63|AP5M1\_MOUSE sp|O08901|BUB1\_MOUSE sp|Q8CHI8|EP400\_MOUSE sp|Q8CHI8-4|EP400\_MOUSE sp|Q8CHI8-2|EP400\_MOUSE sp|Q8CHI8-3|EP400\_MOUSE sp|Q69ZF7|CNNM4\_MOUSE sp|Q80T14|FRAS1\_MOUSE sp|Q61983|NPT1\_MOUSE sp|Q61037-4|TSC2\_MOUSE sp|Q8BHK9|ERC6L\_MOUSE sp|Q78PY7|SND1\_MOUSE sp|Q810C1|SLIK1\_MOUSE tr|G3UZM1|G3UZM1\_MOUSE sp|Q69ZK6|JHD2C\_MOUSE sp|Q91ZI0|CELR3\_MOUSE tr|A2A9M4|A2A9M4\_MOUSE tr|E9QPE7|E9QPE7\_MOUSE sp|Q9D7J9|ECHD3\_MOUSE sp|Q9JKK7|TMOD2\_MOUSE sp|Q8C0N1|KIF2B\_MOUSE sp|Q8K013|GTPBA\_MOUSE tr|D6RFC6|D6RFC6\_MOUSE sp|Q99MV7|RNF17\_MOUSE sp|Q52KR3|PRUN2\_MOUSE sp|Q52KR3-6|PRUN2\_MOUSE sp|Q9ES07|S15A2\_MOUSE tr|E9QMN8|E9QMN8\_MOUSE sp|Q68FE9|TSH2\_MOUSE tr|E9QNG6|E9QNG6\_MOUSE sp|Q6PHS6|SNX13\_MOUSE tr|E9Q7T4|E9Q7T4\_MOUSE tr|Q8CHB6|Q8CHB6\_MOUSE tr|A2AG36|A2AG36\_MOUSE sp|Q9JJI6|PIGO\_MOUSE tr|Q562G0|Q562G0\_MOUSE sp|P97872|FMO5\_MOUSE tr|Z4YJC9|Z4YJC9\_MOUSE sp|O70445|BARD1\_MOUSE sp|Q8CJ40-2|CROCC\_MOUSE sp|Q56A10|ZN608\_MOUSE sp|Q56A10-2|ZN608\_MOUSE sp|Q8BV79|TRNK1\_MOUSE tr|B9EJX2|B9EJX2\_MOUSE tr|B1AV60|B1AV60\_MOUSE sp|P11531|DMD\_MOUSE sp|Q9ER65-2|CSTN2\_MOUSE sp|Q9ER65|CSTN2\_MOUSE sp|Q3USY0-3|S38AB\_MOUSE sp|Q3USY0|S38AB\_MOUSE tr|F6X0X3|F6X0X3\_MOUSE sp|Q3USY0-2|S38AB\_MOUSE tr|F6WJL8|F6WJL8\_MOUSE sp|Q8C0L8|COG5\_MOUSE sp|P81122|IRS2\_MOUSE sp|Q80UU1|ANKZ1\_MOUSE tr|D3Z7D3|D3Z7D3\_MOUSE tr|F8WI56|F8WI56\_MOUSE sp|Q8BWT5|DIP2A\_MOUSE tr|Q3UH13|Q3UH13\_MOUSE sp|Q5NCE8|MRS2\_MOUSE sp|P24527|LKHA4\_MOUSE sp|O35206|COFA1\_MOUSE tr|E9Q8Z5|E9Q8Z5\_MOUSE sp|P30999-2|CTND1\_MOUSE sp|P30999-3|CTND1\_MOUSE tr|E9Q907|E9Q907\_MOUSE tr|E9Q986|E9Q986\_MOUSE sp|P30999|CTND1\_MOUSE tr|G3X9V2|G3X9V2\_MOUSE tr|E9Q903|E9Q903\_MOUSE tr|E9Q8Z4|E9Q8Z4\_MOUSE tr|E9Q904|E9Q904\_MOUSE tr|E9Q906|E9Q906\_MOUSE tr|E9Q8Z9|E9Q8Z9\_MOUSE tr|D3Z7H6|D3Z7H6\_MOUSE tr|D3Z2H2|D3Z2H2\_MOUSE tr|E9Q8Z8|E9Q8Z8\_MOUSE tr|E9Q905|E9Q905\_MOUSE tr|E9Q8Z6|E9Q8Z6\_MOUSE tr|E9Q901|E9Q901\_MOUSE sp|Q8K3K7|PLCB\_MOUSE sp|Q8C078|KKCC2\_MOUSE sp|Q8C078-5|KKCC2\_MOUSE sp|Q8C078-2|KKCC2\_MOUSE sp|Q8C078-3|KKCC2\_MOUSE sp|Q6NXI6|RPRD2\_MOUSE tr|Q8CGZ8|Q8CGZ8\_MOUSE sp|P18121|PR3D1\_MOUSE tr|Q5SZY7|Q5SZY7\_MOUSE tr|Q5SY42|Q5SY42\_MOUSE tr|Q8CE80|Q8CE80\_MOUSE tr|D3Z7C8|D3Z7C8\_MOUSE sp|O35855|BCAT2\_MOUSE sp|P54265-5|DMPK\_MOUSE sp|P54265-6|DMPK\_MOUSE sp|Q7TSF1|DSG1B\_MOUSE sp|P01029|CO4B\_MOUSE sp|Q99MX7|CECR6\_MOUSE sp|Q6IMP4-2|PANX2\_MOUSE sp|Q6IMP4|PANX2\_MOUSE sp|P97300|NPTN\_MOUSE sp|O70570|PIGR\_MOUSE tr|D3Z3U6|D3Z3U6\_MOUSE tr|G3X9X7|G3X9X7\_MOUSE tr|F8WIA8|F8WIA8\_MOUSE sp|O54967-3|ACK1\_MOUSE sp|O54967-2|ACK1\_MOUSE sp|O54967|ACK1\_MOUSE tr|D3Z3U5|D3Z3U5\_MOUSE tr|E9Q6G4|E9Q6G4\_MOUSE sp|Q91V24|ABCA7\_MOUSE sp|Q5SV85|SYNRG\_MOUSE tr|V9GX40|V9GX40\_MOUSE sp|Q7TT50|MRCKB\_MOUSE sp|P28825|MEP1A\_MOUSE sp|Q8C0E3|TRI47\_MOUSE sp|Q8C0E3-2|TRI47\_MOUSE sp|Q922D4-2|PP6R3\_MOUSE tr|D3Z534|D3Z534\_MOUSE tr|D3Z532|D3Z532\_MOUSE sp|Q922D4|PP6R3\_MOUSE tr|G5E8R4|G5E8R4\_MOUSE sp|A2CG49-4|KALRN\_MOUSE tr|H3BLK8|H3BLK8\_MOUSE sp|Q70KF4|CMYA5\_MOUSE tr|E9Q9J4|E9Q9J4\_MOUSE tr|E9Q043|E9Q043\_MOUSE sp|Q8CGF1|RHG29\_MOUSE tr|E9QJQ9|E9QJQ9\_MOUSE sp|Q3UH66|WNK2\_MOUSE sp|Q3UH66-2|WNK2\_MOUSE tr|E0CZE3|E0CZE3\_MOUSE tr|E0CYT1|E0CYT1\_MOUSE tr|E9QMI8|E9QMI8\_MOUSE sp|Q3UH66-3|WNK2\_MOUSE sp|Q3UH66-4|WNK2\_MOUSE tr|E9Q6Q2|E9Q6Q2\_MOUSE sp|Q3UH66-6|WNK2\_MOUSE sp|Q8BV57|SRCRL\_MOUSE tr|A0A087WR13|A0A087WR13\_MOUSE sp|P54265-3|DMPK\_MOUSE tr|E9Q6J9|E9Q6J9\_MOUSE sp|P54265|DMPK\_MOUSE sp|P54265-7|DMPK\_MOUSE sp|P54265-2|DMPK\_MOUSE tr|D6RI32|D6RI32\_MOUSE sp|P54265-10|DMPK\_MOUSE sp|P54265-8|DMPK\_MOUSE sp|P54265-9|DMPK\_MOUSE sp|Q5DTK1|CHSS3\_MOUSE tr|E9PVD7|E9PVD7\_MOUSE sp|P26361|CFTR\_MOUSE sp|Q24JP3-3|F178B\_MOUSE sp|Q24JP3|F178B\_MOUSE sp|Q0KL02-4|TRIO\_MOUSE tr|Q5SXR6|Q5SXR6\_MOUSE sp|Q68FD5|CLH1\_MOUSE sp|Q80YX1-3|TENA\_MOUSE sp|Q80YX1-2|TENA\_MOUSE sp|Q80YX1|TENA\_MOUSE tr|E9Q3H9|E9Q3H9\_MOUSE tr|E9Q7G0|E9Q7G0\_MOUSE sp|P59672-2|ANS1A\_MOUSE sp|P59672|ANS1A\_MOUSE tr|Q3UHP6|Q3UHP6\_MOUSE tr|E9Q1I5|E9Q1I5\_MOUSE sp|P60710|ACTB\_MOUSE tr|F6ST92|F6ST92\_MOUSE tr|D3YXL0|D3YXL0\_MOUSE sp|Q69ZZ9|K0754\_MOUSE sp|Q9CWU0|TDR12\_MOUSE tr|L7MU04|L7MU04\_MOUSE sp|Q9CQ70|H2AB1\_MOUSE tr|Q497L1|Q497L1\_MOUSE tr|E9Q264|E9Q264\_MOUSE tr|E9Q8K8|E9Q8K8\_MOUSE sp|Q03146-2|DDR1\_MOUSE sp|Q5SSZ5-2|TENS3\_MOUSE sp|Q8K0E1|KCD15\_MOUSE sp|Q8VHK1|CSKI2\_MOUSE tr|Q8VGD8|Q8VGD8\_MOUSE sp|Q8VIM6|STRC\_MOUSE sp|Q69ZT1|FAN1\_MOUSE sp|Q05909|PTPRG\_MOUSE tr|F8VQD7|F8VQD7\_MOUSE tr|J3QNR8|J3QNR8\_MOUSE sp|P51125-6|ICAL\_MOUSE sp|Q9EPQ8-2|TCF20\_MOUSE sp|Q9EPQ8|TCF20\_MOUSE sp|Q91WC9|DGLB\_MOUSE sp|P63260|ACTG\_MOUSE sp|Q80TN5|ZDH17\_MOUSE sp|Q80TN5-2|ZDH17\_MOUSE sp|Q7TSH3|ZN516\_MOUSE sp|Q9D952|EVPL\_MOUSE sp|Q61824|ADA12\_MOUSE sp|Q8BQ33|TICRR\_MOUSE sp|Q7M732|RTL1\_MOUSE sp|O08696|FOXM1\_MOUSE tr|Q6P1H7|Q6P1H7\_MOUSE tr|E9Q7Y0|E9Q7Y0\_MOUSE sp|Q5SFM8-3|RBM27\_MOUSE sp|Q5SFM8|RBM27\_MOUSE sp|Q5SFM8-2|RBM27\_MOUSE sp|Q80VK6|ARH38\_MOUSE sp|Q61687|ATRX\_MOUSE sp|Q66X05|NAL4F\_MOUSE tr|E9Q4N6|E9Q4N6\_MOUSE sp|Q6P1Y8|INP4B\_MOUSE tr|E9PVM1|E9PVM1\_MOUSE tr|E9PVM3|E9PVM3\_MOUSE tr|E9Q784|E9Q784\_MOUSE sp|Q9ES52-3|SHIP1\_MOUSE sp|Q9ES52-4|SHIP1\_MOUSE sp|Q9ES52|SHIP1\_MOUSE sp|Q9ES52-2|SHIP1\_MOUSE tr|B8XCJ6|B8XCJ6\_MOUSE sp|Q8CE13|CCD17\_MOUSE sp|Q9WUG6|INSL5\_MOUSE tr|A2AIY3|A2AIY3\_MOUSE tr|D3Z151|D3Z151\_MOUSE sp|Q99LC8|EI2BA\_MOUSE tr|D3YZC9|D3YZC9\_MOUSE tr|F7ADS7|F7ADS7\_MOUSE sp|P97868-2|RBBP6\_MOUSE sp|O08638|MYH11\_MOUSE sp|O08638-2|MYH11\_MOUSE tr|F6ZS70|F6ZS70\_MOUSE sp|Q05512|MARK2\_MOUSE sp|Q05512-3|MARK2\_MOUSE tr|E9QMP6|E9QMP6\_MOUSE tr|Q3T9A3|Q3T9A3\_MOUSE sp|Q05512-4|MARK2\_MOUSE tr|Z4YL66|Z4YL66\_MOUSE sp|E9Q349|WDR25\_MOUSE tr|Q91Y18|Q91Y18\_MOUSE sp|Q9Z1N9|UN13B\_MOUSE tr|E9Q263|E9Q263\_MOUSE sp|Q9Z1N9-2|UN13B\_MOUSE sp|Q9Z1N9-3|UN13B\_MOUSE sp|Q8R0G9|NU133\_MOUSE tr|F6X9P4|F6X9P4\_MOUSE sp|Q8R2K1-5|FUCM\_MOUSE sp|P19137|LAMA1\_MOUSE tr|F8VQ40|F8VQ40\_MOUSE sp|Q6T3U4|NPCL1\_MOUSE sp|Q3UYI5|RGL3\_MOUSE sp|Q3UYI5-2|RGL3\_MOUSE sp|Q3LAC4|PREX2\_MOUSE sp|Q8CIP5|DISP2\_MOUSE sp|Q61037-2|TSC2\_MOUSE sp|Q61037-7|TSC2\_MOUSE tr|Q7TT21|Q7TT21\_MOUSE sp|Q61037|TSC2\_MOUSE sp|Q61037-6|TSC2\_MOUSE sp|Q61037-3|TSC2\_MOUSE sp|Q61037-5|TSC2\_MOUSE tr|D3Z741|D3Z741\_MOUSE tr|J3QK07|J3QK07\_MOUSE tr|Q80XR8|Q80XR8\_MOUSE tr|D3Z1I2|D3Z1I2\_MOUSE sp|Q8BH06|I17RE\_MOUSE sp|Q0KL02|TRIO\_MOUSE sp|Q8R526|PK1L1\_MOUSE sp|Q8CGY6|UN45B\_MOUSE tr|A2A654|A2A654\_MOUSE tr|E9Q6A7|E9Q6A7\_MOUSE tr|A2A655|A2A655\_MOUSE sp|Q8CGY6-2|UN45B\_MOUSE tr|D3YVM4|D3YVM4\_MOUSE tr|D3Z2D3|D3Z2D3\_MOUSE tr|F8VQN6|F8VQN6\_MOUSE sp|O54824|IL16\_MOUSE Q2UVX4 tr|Q5SVF9|Q5SVF9\_MOUSE sp|Q8BKT7|THOC5\_MOUSE tr|D3YWB9|D3YWB9\_MOUSE sp|Q99P47|CNTP4\_MOUSE sp|Q8BKN5|GCP5\_MOUSE sp|Q8BKN5-2|GCP5\_MOUSE sp|Q8BKN5-3|GCP5\_MOUSE sp|Q4V9Z5-3|SE6L2\_MOUSE sp|Q4V9Z5-2|SE6L2\_MOUSE sp|Q61464|ZN638\_MOUSE sp|Q8C4V1-3|RHG24\_MOUSE tr|D3Z5T4|D3Z5T4\_MOUSE sp|Q8C4V1-2|RHG24\_MOUSE tr|Q5SZ92|Q5SZ92\_MOUSE tr|G5E894|G5E894\_MOUSE tr|E9PYX3|E9PYX3\_MOUSE tr|E9Q9N6|E9Q9N6\_MOUSE sp|Q04690-2|NF1\_MOUSE sp|Q04690|NF1\_MOUSE tr|A0A087WR59|A0A087WR59\_MOUSE sp|Q64213-2|SF01\_MOUSE tr|F6VVY4|F6VVY4\_MOUSE tr|S4R2S8|S4R2S8\_MOUSE tr|D3YXH6|D3YXH6\_MOUSE sp|Q2QI47-3|USH2A\_MOUSE sp|Q2QI47|USH2A\_MOUSE sp|Q9ET54|PALLD\_MOUSE sp|Q9ET54-6|PALLD\_MOUSE sp|Q8CGF7-2|TCRG1\_MOUSE sp|Q8CGF7|TCRG1\_MOUSE sp|Q8CGF7-3|TCRG1\_MOUSE tr|E9Q9E1|E9Q9E1\_MOUSE sp|Q6NZJ6-2|IF4G1\_MOUSE tr|E9PVC6|E9PVC6\_MOUSE tr|E9PVC5|E9PVC5\_MOUSE sp|Q6NZJ6|IF4G1\_MOUSE tr|F6ZBU4|F6ZBU4\_MOUSE sp|Q01098|NMDE3\_MOUSE tr|F6XI52|F6XI52\_MOUSE sp|Q80YE4-3|LMTK1\_MOUSE tr|B1AZF9|B1AZF9\_MOUSE tr|B1AZF3|B1AZF3\_MOUSE sp|Q80YE4|LMTK1\_MOUSE sp|Q80YE4-2|LMTK1\_MOUSE sp|Q8BG22|CLCA2\_MOUSE sp|Q8K0E3|SC5AB\_MOUSE sp|Q8BG22-2|CLCA2\_MOUSE tr|F8WIF5|F8WIF5\_MOUSE tr|E9Q7M6|E9Q7M6\_MOUSE sp|Q99PF4|CAD23\_MOUSE sp|Q99PF4-2|CAD23\_MOUSE tr|K4DI74|K4DI74\_MOUSE tr|D3YYE1|D3YYE1\_MOUSE tr|D3Z7M9|D3Z7M9\_MOUSE tr|F6UFG6|F6UFG6\_MOUSE sp|O35381|AN32A\_MOUSE sp|O35684|NEUS\_MOUSE sp|Q99JP7|GGT7\_MOUSE sp|Q80XS7|FA83E\_MOUSE tr|Q80X98|Q80X98\_MOUSE tr|E9QP49|E9QP49\_MOUSE sp|Q8K1H7|T11L2\_MOUSE tr|E9Q7C9|E9Q7C9\_MOUSE tr|Q5UE59|Q5UE59\_MOUSE tr|Q8CD76|Q8CD76\_MOUSE tr|Q7TNF4|Q7TNF4\_MOUSE tr|F6UYN4|F6UYN4\_MOUSE tr|V9GX34|V9GX34\_MOUSE tr|F8WIE5|F8WIE5\_MOUSE sp|Q69ZR2|HECD1\_MOUSE sp|Q9R0B9|PLOD2\_MOUSE sp|Q8K448|ABCA5\_MOUSE sp|Q99MR6-4|SRRT\_MOUSE sp|Q99MR6-3|SRRT\_MOUSE sp|Q99MR6|SRRT\_MOUSE sp|Q99MR6-2|SRRT\_MOUSE sp|Q6PGL7|FAM21\_MOUSE tr|G3XA35|G3XA35\_MOUSE sp|Q62059-2|CSPG2\_MOUSE tr|F6ZBD9|F6ZBD9\_MOUSE tr|A2AJQ0|A2AJQ0\_MOUSE sp|Q3UV70|PDP1\_MOUSE tr|A8Y5Q0|A8Y5Q0\_MOUSE tr|A2AJP9|A2AJP9\_MOUSE tr|D3YY75|D3YY75\_MOUSE sp|Q91ZD4|VANG2\_MOUSE tr|O88374|O88374\_MOUSE sp|A6PWD2|FHAD1\_MOUSE tr|A2AQA7|A2AQA7\_MOUSE tr|V9GXD9|V9GXD9\_MOUSE sp|Q5SV85-2|SYNRG\_MOUSE tr|E9Q7R9|E9Q7R9\_MOUSE tr|F7CGP0|F7CGP0\_MOUSE sp|Q80WQ8|M18BP\_MOUSE sp|Q8BTH6|SCRT2\_MOUSE sp|Q80U40|RIMB2\_MOUSE sp|Q80U40-2|RIMB2\_MOUSE tr|D3YXR8|D3YXR8\_MOUSE sp|Q99MV1|TDRD1\_MOUSE tr|E9Q0B6|E9Q0B6\_MOUSE tr|E9Q3H7|E9Q3H7\_MOUSE tr|E9QMC2|E9QMC2\_MOUSE sp|Q3UVX5|GRM5\_MOUSE sp|Q80U35|ARHGH\_MOUSE tr|A2A7U2|A2A7U2\_MOUSE tr|Q8BS54|Q8BS54\_MOUSE sp|Q6ZQ08-2|CNOT1\_MOUSE sp|Q6ZQ08-4|CNOT1\_MOUSE sp|Q6ZQ08|CNOT1\_MOUSE tr|B7ZWL1|B7ZWL1\_MOUSE sp|Q3UQ44|IQGA2\_MOUSE tr|E9Q706|E9Q706\_MOUSE tr|S4R245|S4R245\_MOUSE sp|Q8C8R3-5|ANK2\_MOUSE tr|S4R2D0|S4R2D0\_MOUSE sp|P51830|ADCY9\_MOUSE sp|A2ALS5-3|RPGP1\_MOUSE sp|A2ALS5-2|RPGP1\_MOUSE sp|Q2PZL6-2|FAT4\_MOUSE sp|A2ALS5|RPGP1\_MOUSE tr|Q3UH59|Q3UH59\_MOUSE sp|Q61879|MYH10\_MOUSE tr|Q5SV64|Q5SV64\_MOUSE sp|O35640|ANXA8\_MOUSE tr|Q921D0|Q921D0\_MOUSE sp|Q8K0V4|CNOT3\_MOUSE sp|Q8BNE1|F115A\_MOUSE sp|Q60520|SIN3A\_MOUSE sp|Q8BNE1-2|F115A\_MOUSE sp|Q60520-1|SIN3A\_MOUSE sp|Q6ZPF4-2|FMNL3\_MOUSE tr|D3Z7A7|D3Z7A7\_MOUSE sp|Q6ZPF4|FMNL3\_MOUSE tr|E9PZ12|E9PZ12\_MOUSE tr|D3Z2Z1|D3Z2Z1\_MOUSE sp|Q922J3|CLIP1\_MOUSE tr|F8WIA1|F8WIA1\_MOUSE tr|A0A087WPR7|A0A087WPR7\_MOUSE sp|Q5NC05|TTF2\_MOUSE sp|Q80WC3-2|TNC18\_MOUSE sp|Q6P7W0|SENP6\_MOUSE tr|F6Z9A1|F6Z9A1\_MOUSE sp|P0C5E4|PTPRQ\_MOUSE sp|Q9QWK5|BIR1A\_MOUSE sp|B2RU80|PTPRB\_MOUSE sp|Q6A068|CDC5L\_MOUSE sp|Q60875-4|ARHG2\_MOUSE tr|H3BKH9|H3BKH9\_MOUSE sp|Q60875-5|ARHG2\_MOUSE sp|Q60875|ARHG2\_MOUSE tr|H3BJU7|H3BJU7\_MOUSE tr|H3BJ45|H3BJ45\_MOUSE tr|H3BJ40|H3BJ40\_MOUSE tr|H3BJX8|H3BJX8\_MOUSE tr|G3X939|G3X939\_MOUSE tr|G3X9G2|G3X9G2\_MOUSE tr|Q5SXG1|Q5SXG1\_MOUSE sp|Q9JM52-2|MINK1\_MOUSE sp|Q9JM52|MINK1\_MOUSE sp|Q9JM52-3|MINK1\_MOUSE tr|F7AMS7|F7AMS7\_MOUSE tr|Q5SXG3|Q5SXG3\_MOUSE sp|Q6A058|ARMX2\_MOUSE sp|Q91WG0|EST2C\_MOUSE tr|A2A4B4|A2A4B4\_MOUSE sp|P59900|EMIL3\_MOUSE tr|F6YPG3|F6YPG3\_MOUSE sp|Q8VHE0|SEC63\_MOUSE tr|E9Q0S6|E9Q0S6\_MOUSE tr|A0A087WQ94|A0A087WQ94\_MOUSE sp|Q9JKK7-3|TMOD2\_MOUSE sp|O08553|DPYL2\_MOUSE sp|O35382|EXOC4\_MOUSE tr|G3UYW1|G3UYW1\_MOUSE sp|Q6VNB8|WDFY3\_MOUSE tr|Q91ZZ2|Q91ZZ2\_MOUSE tr|Q3UHC1|Q3UHC1\_MOUSE sp|Q8CHI8-5|EP400\_MOUSE tr|D3YZZ3|D3YZZ3\_MOUSE sp|Q60662-2|AKAP4\_MOUSE sp|Q60662|AKAP4\_MOUSE sp|Q03173-2|ENAH\_MOUSE tr|B2RUS0|B2RUS0\_MOUSE tr|D3YV17|D3YV17\_MOUSE sp|Q6ZQ93|UBP34\_MOUSE tr|F6WJB7|F6WJB7\_MOUSE sp|P25118|TNR1A\_MOUSE sp|Q6NXH3|GPBP1\_MOUSE sp|Q6NXH3-2|GPBP1\_MOUSE tr|F8VPZ9|F8VPZ9\_MOUSE sp|Q6NXH3-3|GPBP1\_MOUSE sp|Q69ZM6-2|STK36\_MOUSE sp|Q69ZM6-3|STK36\_MOUSE sp|Q69ZM6|STK36\_MOUSE sp|P62340|TBPL1\_MOUSE tr|Q8R056|Q8R056\_MOUSE sp|O88783|FA5\_MOUSE sp|Q6ZQ93-3|UBP34\_MOUSE sp|Q6ZQ93-2|UBP34\_MOUSE sp|Q9D236-1|HTRA3\_MOUSE sp|Q9D236-4|HTRA3\_MOUSE sp|Q9D236|HTRA3\_MOUSE tr|D3YYM4|D3YYM4\_MOUSE sp|A2AJ15|MA1B1\_MOUSE sp|Q6P5E4|UGGG1\_MOUSE sp|Q8BR76-2|MKS3\_MOUSE tr|D3Z3M7|D3Z3M7\_MOUSE tr|J3KMJ8|J3KMJ8\_MOUSE tr|G3UVW7|G3UVW7\_MOUSE sp|A2AD83|FRMD7\_MOUSE sp|Q3V3R4|ITA1\_MOUSE sp|Q3UQ28|PXDN\_MOUSE tr|F6YZH6|F6YZH6\_MOUSE sp|Q8CDU4|FXL13\_MOUSE sp|Q6R3M4-2|POLI\_MOUSE sp|Q6R3M4|POLI\_MOUSE tr|A2A4N9|A2A4N9\_MOUSE sp|A2A4P0|DHX8\_MOUSE sp|Q921L8|GLT11\_MOUSE tr|E9PYP1|E9PYP1\_MOUSE sp|Q05BQ1|TUTLA\_MOUSE sp|Q03173-6|ENAH\_MOUSE sp|Q03173-3|ENAH\_MOUSE sp|Q61584|FXR1\_MOUSE sp|Q61584-7|FXR1\_MOUSE sp|Q61584-6|FXR1\_MOUSE sp|P51125-7|ICAL\_MOUSE sp|Q6P1G0|HEAT6\_MOUSE sp|Q4ACU6-8|SHAN3\_MOUSE sp|Q9QZR5-4|HIPK2\_MOUSE sp|Q9QZR5-2|HIPK2\_MOUSE sp|Q9QZR5|HIPK2\_MOUSE sp|P53564-3|CUX1\_MOUSE tr|F7C950|F7C950\_MOUSE tr|H3BK24|H3BK24\_MOUSE sp|Q99NE5-6|RIMS1\_MOUSE tr|F6TZK4|F6TZK4\_MOUSE sp|Q0VGT4|ZGRF1\_MOUSE tr|H3BJH9|H3BJH9\_MOUSE sp|Q60625|ICAM5\_MOUSE sp|Q9WV30-2|NFAT5\_MOUSE sp|Q9WV30|NFAT5\_MOUSE tr|E9QP01|E9QP01\_MOUSE sp|Q924A2-2|CIC\_MOUSE tr|F6Z7L1|F6Z7L1\_MOUSE tr|M0QWU9|M0QWU9\_MOUSE sp|Q8BUR4|DOCK1\_MOUSE tr|E9Q981|E9Q981\_MOUSE tr|E9Q6S4|E9Q6S4\_MOUSE tr|F6QN44|F6QN44\_MOUSE tr|A0A087WSF4|A0A087WSF4\_MOUSE tr|Q6NVD6|Q6NVD6\_MOUSE tr|L7N2E7|L7N2E7\_MOUSE tr|F6ZZH7|F6ZZH7\_MOUSE sp|Q60847-3|COCA1\_MOUSE sp|Q60847-4|COCA1\_MOUSE sp|Q9JJN2|ZFHX4\_MOUSE Q0VCM5 sp|Q5SWY7|FA83G\_MOUSE sp|Q9D486|CMIP\_MOUSE sp|Q9D486-2|CMIP\_MOUSE sp|Q9EP89|LACTB\_MOUSE tr|Q8BVR5|Q8BVR5\_MOUSE sp|Q5NCY0|KDM6B\_MOUSE tr|D3Z649|D3Z649\_MOUSE sp|O35177|CASL\_MOUSE tr|D3YXJ8|D3YXJ8\_MOUSE tr|A2AJY2|A2AJY2\_MOUSE sp|A3KGS3|RGPA2\_MOUSE sp|Q80U44|ZFY16\_MOUSE tr|A2AKP6|A2AKP6\_MOUSE tr|D6RE33|D6RE33\_MOUSE tr|G5E896|G5E896\_MOUSE sp|Q80SX8-2|PIF1\_MOUSE tr|D3YZ87|D3YZ87\_MOUSE tr|Q5SUT0|Q5SUT0\_MOUSE tr|Q5SUS9|Q5SUS9\_MOUSE sp|Q61545|EWS\_MOUSE tr|F6YB52|F6YB52\_MOUSE tr|Q3TJ56|Q3TJ56\_MOUSE tr|D3YTR0|D3YTR0\_MOUSE tr|G5E832|G5E832\_MOUSE sp|Q9Z1K7|APC2\_MOUSE sp|Q8BGQ7|SYAC\_MOUSE sp|P56716|RP1\_MOUSE sp|Q8BG26-3|RUSC1\_MOUSE sp|Q8BG26|RUSC1\_MOUSE sp|Q7TNC6|KI26B\_MOUSE tr|E9PX64|E9PX64\_MOUSE sp|Q4ACU6-10|SHAN3\_MOUSE sp|Q4ACU6-9|SHAN3\_MOUSE sp|Q9Z2E2-3|MBD1\_MOUSE sp|Q9Z2E2-5|MBD1\_MOUSE sp|Q8VE73|CUL7\_MOUSE sp|Q8VE73-3|CUL7\_MOUSE tr|A2AB97|A2AB97\_MOUSE tr|F6RPM4|F6RPM4\_MOUSE tr|F6YAT5|F6YAT5\_MOUSE sp|Q61554|FBN1\_MOUSE sp|Q571C7-2|BDP1\_MOUSE sp|Q571C7|BDP1\_MOUSE sp|Q8CH77-4|NAV1\_MOUSE tr|F8VPS6|F8VPS6\_MOUSE tr|E9Q7C3|E9Q7C3\_MOUSE tr|R4GML6|R4GML6\_MOUSE sp|A2AL36|CNTRL\_MOUSE tr|B1AR69|B1AR69\_MOUSE sp|Q9DC11|PXDC2\_MOUSE tr|B1AY86|B1AY86\_MOUSE tr|B1AY85|B1AY85\_MOUSE tr|Q5SRI3|Q5SRI3\_MOUSE sp|Q3TMV7|PYRD1\_MOUSE tr|F7C134|F7C134\_MOUSE tr|E9Q9Y2|E9Q9Y2\_MOUSE tr|D3YZD0|D3YZD0\_MOUSE sp|Q64213|SF01\_MOUSE tr|E9PYX5|E9PYX5\_MOUSE tr|E9Q4Q2|E9Q4Q2\_MOUSE sp|Q64213-3|SF01\_MOUSE tr|D3YTU6|D3YTU6\_MOUSE tr|Q3URI3|Q3URI3\_MOUSE sp|Q63850|NUP62\_MOUSE tr|E9Q6R1|E9Q6R1\_MOUSE sp|Q80VW5-2|WHRN\_MOUSE sp|Q80VW5-10|WHRN\_MOUSE sp|Q80VW5-3|WHRN\_MOUSE sp|Q80VW5-12|WHRN\_MOUSE sp|O88466-2|ZN106\_MOUSE sp|Q80VW5-4|WHRN\_MOUSE tr|E9PXZ3|E9PXZ3\_MOUSE sp|Q9CX86|ROA0\_MOUSE tr|G3X975|G3X975\_MOUSE sp|Q8BYM8|SYCM\_MOUSE sp|Q8C033|ARHGA\_MOUSE tr|Q8K119|Q8K119\_MOUSE sp|Q63811|CANB2\_MOUSE sp|Q8CI96|CLIP4\_MOUSE sp|Q80W03|TOX3\_MOUSE sp|A2RSY1|KANL3\_MOUSE sp|A2RSY1-2|KANL3\_MOUSE sp|O88904-3|HIPK1\_MOUSE sp|O88904-2|HIPK1\_MOUSE sp|O88904|HIPK1\_MOUSE tr|E9QM73|E9QM73\_MOUSE sp|Q3UH66-5|WNK2\_MOUSE tr|E9QMI9|E9QMI9\_MOUSE sp|Q3UH66-7|WNK2\_MOUSE sp|Q80ZA4|PKHL1\_MOUSE tr|F8WH29|F8WH29\_MOUSE sp|O09053|WRN\_MOUSE sp|Q80WQ8-3|M18BP\_MOUSE sp|Q80TG1|KANL1\_MOUSE tr|E9Q6A6|E9Q6A6\_MOUSE sp|Q8C6K9|CO6A6\_MOUSE sp|Q80WQ8-2|M18BP\_MOUSE tr|E9PVP1|E9PVP1\_MOUSE tr|E9Q6M6|E9Q6M6\_MOUSE tr|Q91XZ5|Q91XZ5\_MOUSE sp|Q9WTS4|TEN1\_MOUSE tr|A2ANL9|A2ANL9\_MOUSE tr|F6QNG5|F6QNG5\_MOUSE tr|F8VQ72|F8VQ72\_MOUSE tr|F6QUA9|F6QUA9\_MOUSE sp|Q60949|TBCD1\_MOUSE sp|Q60949-2|TBCD1\_MOUSE tr|E9Q6L4|E9Q6L4\_MOUSE sp|Q4VA61|DSCL1\_MOUSE tr|E9QPR7|E9QPR7\_MOUSE tr|A2APR8|A2APR8\_MOUSE sp|Q5NCI0-2|URGCP\_MOUSE sp|Q5NCI0|URGCP\_MOUSE tr|F6XMP1|F6XMP1\_MOUSE tr|L7N248|L7N248\_MOUSE tr|H3BL89|H3BL89\_MOUSE tr|H3BJC5|H3BJC5\_MOUSE sp|Q8VBX6-2|MPDZ\_MOUSE sp|Q8VBX6-4|MPDZ\_MOUSE sp|Q8VBX6-3|MPDZ\_MOUSE sp|Q8VBX6|MPDZ\_MOUSE tr|D3YUX2|D3YUX2\_MOUSE tr|B2RT41|B2RT41\_MOUSE sp|Q6PCM2-2|INT6\_MOUSE sp|Q6PCM2|INT6\_MOUSE tr|Q3UHL6|Q3UHL6\_MOUSE tr|B7ZNJ1|B7ZNJ1\_MOUSE tr|A0A087WR50|A0A087WR50\_MOUSE tr|A0A087WS56|A0A087WS56\_MOUSE tr|A0A087WSN6|A0A087WSN6\_MOUSE tr|B9EHT6|B9EHT6\_MOUSE sp|P11276|FINC\_MOUSE sp|E9Q7X6-2|HEG1\_MOUSE sp|E9Q7X6|HEG1\_MOUSE sp|Q3TRM4-2|PLPL6\_MOUSE sp|Q3TRM4-3|PLPL6\_MOUSE sp|Q3TRM4|PLPL6\_MOUSE sp|Q7TQB0|TR134\_MOUSE sp|Q6IQX7|CHSS2\_MOUSE sp|Q8C9S4|CC186\_MOUSE sp|Q3V1H3|HPHL1\_MOUSE tr|E9QK36|E9QK36\_MOUSE sp|Q3U3T8|WDR62\_MOUSE sp|A2CG49-6|KALRN\_MOUSE sp|Q62407|SPEG\_MOUSE tr|A0A087WSE3|A0A087WSE3\_MOUSE sp|Q62407-4|SPEG\_MOUSE tr|E9QQ25|E9QQ25\_MOUSE sp|Q62407-3|SPEG\_MOUSE tr|S4R255|S4R255\_MOUSE sp|Q9Z0J4-3|NOS1\_MOUSE sp|Q9Z0J4-4|NOS1\_MOUSE sp|Q9Z0J4|NOS1\_MOUSE tr|F8WGF2|F8WGF2\_MOUSE sp|Q9Z0J4-5|NOS1\_MOUSE sp|A2ABV5|MED14\_MOUSE sp|Q80WJ6-4|MRP9\_MOUSE tr|D6RCU5|D6RCU5\_MOUSE sp|Q80WJ6-2|MRP9\_MOUSE tr|D6RGU5|D6RGU5\_MOUSE sp|Q80WJ6-3|MRP9\_MOUSE sp|Q8CDI7|CC150\_MOUSE tr|Q91UZ8|Q91UZ8\_MOUSE sp|Q9EPN1-4|NBEA\_MOUSE tr|E9Q6T8|E9Q6T8\_MOUSE sp|O88466-3|ZN106\_MOUSE sp|O70566|DIAP2\_MOUSE sp|E1U8D0|SOGA1\_MOUSE tr|A2ACV6|A2ACV6\_MOUSE tr|G5E8K2|G5E8K2\_MOUSE sp|G5E8K5-3|ANK3\_MOUSE sp|G5E8K5|ANK3\_MOUSE tr|G5E8K3|G5E8K3\_MOUSE tr|H7BX02|H7BX02\_MOUSE sp|Q4VGL6|RC3H1\_MOUSE tr|H3BK32|H3BK32\_MOUSE sp|Q6P7W0-2|SENP6\_MOUSE sp|Q80VC9|CAMP3\_MOUSE tr|E9Q5B0|E9Q5B0\_MOUSE sp|Q80VC9-2|CAMP3\_MOUSE sp|Q6PAJ3|GAREL\_MOUSE tr|G5E866|G5E866\_MOUSE sp|Q99NB9|SF3B1\_MOUSE sp|Q06335|APLP2\_MOUSE tr|Q60709|Q60709\_MOUSE sp|Q06335-2|APLP2\_MOUSE Q28107 sp|Q8BRT1|CLAP2\_MOUSE tr|E9Q8N5|E9Q8N5\_MOUSE tr|Q08EB6|Q08EB6\_MOUSE tr|F7DCH5|F7DCH5\_MOUSE sp|Q3TZ89|SC31B\_MOUSE sp|Q8BH06-4|I17RE\_MOUSE tr|Q9Z202|Q9Z202\_MOUSE tr|A2A5Y4|A2A5Y4\_MOUSE tr|F6RHI8|F6RHI8\_MOUSE sp|Q9CS84-4|NRX1A\_MOUSE sp|Q9CS84-2|NRX1A\_MOUSE tr|E0CY11|E0CY11\_MOUSE sp|Q9CS84|NRX1A\_MOUSE tr|E0CZA5|E0CZA5\_MOUSE sp|Q9CS84-3|NRX1A\_MOUSE tr|F8VQ93|F8VQ93\_MOUSE sp|Q3UH60|DIP2B\_MOUSE sp|Q9EPE9|AT131\_MOUSE sp|Q8R4H2|ARHGC\_MOUSE tr|E9Q9Q0|E9Q9Q0\_MOUSE sp|Q66JY2|IN80D\_MOUSE tr|B1AT34|B1AT34\_MOUSE tr|B1AT32|B1AT32\_MOUSE tr|B1AT33|B1AT33\_MOUSE sp|Q66JY2-2|IN80D\_MOUSE sp|Q9D3E6|STAG1\_MOUSE sp|P48725|PCNT\_MOUSE tr|F8VPV0|F8VPV0\_MOUSE sp|P48725-3|PCNT\_MOUSE sp|Q923L3|CSMD1\_MOUSE sp|Q923L3-2|CSMD1\_MOUSE sp|Q8K4Q0|RPTOR\_MOUSE tr|A2ACM0|A2ACM0\_MOUSE sp|Q61006|MUSK\_MOUSE sp|Q61006-3|MUSK\_MOUSE tr|Q497X0|Q497X0\_MOUSE sp|Q61006-2|MUSK\_MOUSE sp|Q61006-4|MUSK\_MOUSE sp|Q8BGY3|LUZP2\_MOUSE sp|Q9Z329-2|ITPR2\_MOUSE tr|F6WF36|F6WF36\_MOUSE tr|Q8CEQ9|Q8CEQ9\_MOUSE sp|Q62315-2|JARD2\_MOUSE sp|Q62315|JARD2\_MOUSE tr|G3UZT8|G3UZT8\_MOUSE tr|Q8BGT1|Q8BGT1\_MOUSE sp|Q76KF0|SEM6D\_MOUSE sp|Q76KF0-4|SEM6D\_MOUSE tr|A2AW73|A2AW73\_MOUSE sp|Q76KF0-6|SEM6D\_MOUSE sp|Q2KN98|CYTSA\_MOUSE sp|Q76KF0-3|SEM6D\_MOUSE sp|Q4ACU6-7|SHAN3\_MOUSE sp|Q4ACU6-11|SHAN3\_MOUSE sp|Q4ACU6-3|SHAN3\_MOUSE sp|Q4ACU6-12|SHAN3\_MOUSE tr|E9Q5G1|E9Q5G1\_MOUSE sp|F8VQB6|MYO10\_MOUSE sp|F8VQB6-2|MYO10\_MOUSE tr|Q9JLQ4|Q9JLQ4\_MOUSE tr|A2ADM8|A2ADM8\_MOUSE sp|A2AM05|CNTLN\_MOUSE tr|E9QP44|E9QP44\_MOUSE sp|Q8BZ05|ARAP2\_MOUSE tr|E9Q7S8|E9Q7S8\_MOUSE sp|Q9D0C4-2|TRM5\_MOUSE tr|E9PUL8|E9PUL8\_MOUSE tr|D3YZ05|D3YZ05\_MOUSE sp|Q6ZQB6|VIP2\_MOUSE tr|L7N2B1|L7N2B1\_MOUSE sp|Q6ZQB6-2|VIP2\_MOUSE tr|V9GXD5|V9GXD5\_MOUSE tr|B0QZT2|B0QZT2\_MOUSE tr|D3YUI0|D3YUI0\_MOUSE sp|A2ARJ3|TM236\_MOUSE sp|Q8VHS6|ASB15\_MOUSE sp|Q3U2I3-2|F16A2\_MOUSE tr|E9PXB2|E9PXB2\_MOUSE tr|K7N6Y7|K7N6Y7\_MOUSE sp|Q6PGF7|EXOC8\_MOUSE tr|F8WHJ1|F8WHJ1\_MOUSE sp|Q8R307|VPS18\_MOUSE tr|E9PXI5|E9PXI5\_MOUSE sp|Q8VCZ9|PROD2\_MOUSE sp|Q3U2I3|F16A2\_MOUSE sp|Q9D4R2|MGT4D\_MOUSE sp|Q6IQX7-3|CHSS2\_MOUSE sp|Q6IQX7-2|CHSS2\_MOUSE tr|D3YXJ0|D3YXJ0\_MOUSE sp|Q5SS80|DHR13\_MOUSE sp|Q99P91|GPNMB\_MOUSE sp|Q9D0C4|TRM5\_MOUSE sp|Q3U2I3-3|F16A2\_MOUSE sp|Q6ZQB6-3|VIP2\_MOUSE sp|Q5SS80-2|DHR13\_MOUSE tr|J3QP99|J3QP99\_MOUSE sp|A2A6A1|GPTC8\_MOUSE tr|G3XA23|G3XA23\_MOUSE sp|Q8VDP2|CX056\_MOUSE tr|Q80YS7|Q80YS7\_MOUSE tr|H3BLH0|H3BLH0\_MOUSE tr|H3BJK2|H3BJK2\_MOUSE tr|F2Z4A9|F2Z4A9\_MOUSE tr|E9Q341|E9Q341\_MOUSE sp|Q61670|HLX\_MOUSE tr|E9QKK4|E9QKK4\_MOUSE sp|Q8K3I9|GLCI1\_MOUSE sp|Q9R0L6-2|PCM1\_MOUSE sp|Q9R0L6|PCM1\_MOUSE tr|B2RPZ5|B2RPZ5\_MOUSE sp|Q8CDU6|HECD2\_MOUSE sp|Q5DU37|ZFY26\_MOUSE sp|Q5DU37-2|ZFY26\_MOUSE tr|E9QLR8|E9QLR8\_MOUSE sp|Q924W6|TRI66\_MOUSE sp|Q924W6-2|TRI66\_MOUSE tr|F6TIN5|F6TIN5\_MOUSE tr|F6SWS9|F6SWS9\_MOUSE sp|Q69Z98|BRSK2\_MOUSE sp|Q6PDH4|RNB3L\_MOUSE tr|E9PWD9|E9PWD9\_MOUSE sp|Q8BKF1|RPOM\_MOUSE tr|B2RQL2|B2RQL2\_MOUSE tr|G5E8R3|G5E8R3\_MOUSE sp|Q05920|PYC\_MOUSE tr|E9QPD7|E9QPD7\_MOUSE sp|P22682|CBL\_MOUSE sp|Q8BHY8|SNX14\_MOUSE sp|P52431|DPOD1\_MOUSE tr|G3UX33|G3UX33\_MOUSE tr|G3UWA3|G3UWA3\_MOUSE tr|G3UXB6|G3UXB6\_MOUSE sp|Q3UKK2|CEAM5\_MOUSE sp|Q9R0Q4|MO4L2\_MOUSE tr|E9QMD3|E9QMD3\_MOUSE sp|Q61086|FZD3\_MOUSE sp|Q60936|ADCK3\_MOUSE tr|D3Z535|D3Z535\_MOUSE sp|Q9ET54-2|PALLD\_MOUSE sp|Q8C547|HTR5B\_MOUSE tr|F6W623|F6W623\_MOUSE sp|Q9JHG7|PK3CG\_MOUSE sp|Q5S006|LRRK2\_MOUSE sp|P11247|PERM\_MOUSE sp|Q8CAS9|PARP9\_MOUSE sp|Q3UGF1|WDR19\_MOUSE sp|Q3UGF1-2|WDR19\_MOUSE sp|Q3UGF1-3|WDR19\_MOUSE sp|P97814|PPIP1\_MOUSE tr|F7AI27|F7AI27\_MOUSE sp|Q9CSH3|RRP44\_MOUSE sp|Q80TR8-2|VPRBP\_MOUSE sp|P09405|NUCL\_MOUSE sp|Q8VDN2|AT1A1\_MOUSE sp|Q9Z2I4|ROBO3\_MOUSE sp|Q5PRF0|HTR5A\_MOUSE tr|E9QJS7|E9QJS7\_MOUSE tr|F6V3Y9|F6V3Y9\_MOUSE sp|Q5SZA1-2|NPT3\_MOUSE sp|Q5SZA1|NPT3\_MOUSE tr|L7N1W9|L7N1W9\_MOUSE sp|Q6ZPQ6|PITM2\_MOUSE tr|L7N1Z0|L7N1Z0\_MOUSE sp|Q8BUR4-2|DOCK1\_MOUSE tr|B2RXE2|B2RXE2\_MOUSE sp|Q3SYK4|VRTN\_MOUSE sp|Q66K08|CILP1\_MOUSE tr|E9Q3Z5|E9Q3Z5\_MOUSE sp|Q8K4L3|SVIL\_MOUSE tr|F5H8M8|F5H8M8\_MOUSE sp|P80205|OTX1\_MOUSE tr|A2ALS4|A2ALS4\_MOUSE sp|A2ALS5-4|RPGP1\_MOUSE sp|Q9D312|K1C20\_MOUSE Q9D312 sp|Q69ZF8|MSL2\_MOUSE sp|Q9ERZ4|ACM2\_MOUSE sp|Q8BZ52|FSD2\_MOUSE sp|P69744|TRPV5\_MOUSE tr|A0A087WSS1|A0A087WSS1\_MOUSE sp|Q148V7-2|K1468\_MOUSE tr|G3X9J4|G3X9J4\_MOUSE sp|Q148V7-3|K1468\_MOUSE tr|E9QM90|E9QM90\_MOUSE sp|Q148V7|K1468\_MOUSE tr|F6ZGR6|F6ZGR6\_MOUSE sp|A6H5Y3|METH\_MOUSE sp|P30658|CBX2\_MOUSE tr|L7N1Y0|L7N1Y0\_MOUSE tr|Q5SVT2|Q5SVT2\_MOUSE tr|Q5SVT1|Q5SVT1\_MOUSE sp|P35969|VGFR1\_MOUSE sp|A2A690-2|TANC2\_MOUSE sp|Q61191|HCFC1\_MOUSE tr|B1AUX2|B1AUX2\_MOUSE sp|A2A690|TANC2\_MOUSE sp|P35419|PERT\_MOUSE tr|A0A067XG53|A0A067XG53\_MOUSE sp|O70589-3|CSKP\_MOUSE tr|E9Q8A5|E9Q8A5\_MOUSE tr|J3QMF5|J3QMF5\_MOUSE tr|Q9DC42|Q9DC42\_MOUSE sp|P53564-5|CUX1\_MOUSE sp|Q80VM7|ANR24\_MOUSE sp|Q8BQC3|IGDC3\_MOUSE sp|P47873|IL11\_MOUSE sp|Q5DU41-2|LRC8B\_MOUSE sp|P14483|HB2A\_MOUSE sp|P06345|HB2S\_MOUSE sp|P06344|HB2U\_MOUSE sp|P06343|HB2K\_MOUSE tr|H3BLJ4|H3BLJ4\_MOUSE tr|F6UU42|F6UU42\_MOUSE sp|P01132|EGF\_MOUSE tr|G3UZM9|G3UZM9\_MOUSE sp|Q8BWW9|PKN2\_MOUSE sp|Q8BWW9-2|PKN2\_MOUSE tr|G3UXH4|G3UXH4\_MOUSE sp|Q5F226|FAT2\_MOUSE sp|Q8BIZ1|ANS1B\_MOUSE tr|S4R286|S4R286\_MOUSE tr|K4DI73|K4DI73\_MOUSE sp|Q6IFT4|RHG20\_MOUSE tr|A2AP82|A2AP82\_MOUSE sp|Q6NZA9|TAF9B\_MOUSE sp|Q91XT4|SC16B\_MOUSE tr|A0A087WQ57|A0A087WQ57\_MOUSE sp|Q80Y84-2|KDM5B\_MOUSE sp|Q80Y84|KDM5B\_MOUSE sp|Q8CFQ3|AQR\_MOUSE sp|C3VPR6|NLRC5\_MOUSE tr|H3BIX7|H3BIX7\_MOUSE sp|Q80XR2|AT2C1\_MOUSE tr|H3BLI6|H3BLI6\_MOUSE tr|Q3UZR5|Q3UZR5\_MOUSE tr|H3BL44|H3BL44\_MOUSE tr|A0A087WSJ2|A0A087WSJ2\_MOUSE tr|A0A087WRS4|A0A087WRS4\_MOUSE tr|A0A087WQN5|A0A087WQN5\_MOUSE tr|A0A087WQ41|A0A087WQ41\_MOUSE sp|Q9WTI7-2|MYO1C\_MOUSE tr|A0A087WP00|A0A087WP00\_MOUSE tr|E9Q0B1|E9Q0B1\_MOUSE sp|Q9D067-4|MDM1\_MOUSE tr|E9PW14|E9PW14\_MOUSE tr|B2RPU6|B2RPU6\_MOUSE sp|Q9D067-2|MDM1\_MOUSE sp|Q9D067|MDM1\_MOUSE tr|B1ASP2|B1ASP2\_MOUSE sp|P52332|JAK1\_MOUSE sp|O89084|PDE4A\_MOUSE sp|Q8HW98|IGLO5\_MOUSE sp|Q8CJ00|NOXA1\_MOUSE sp|Q8CJ00-2|NOXA1\_MOUSE sp|Q6P5D3|DHX57\_MOUSE tr|Q3U569|Q3U569\_MOUSE sp|E9Q6X9|RHG40\_MOUSE tr|G5E8Q0|G5E8Q0\_MOUSE sp|P53347|ONCM\_MOUSE sp|P97412|LYST\_MOUSE tr|D3Z2E7|D3Z2E7\_MOUSE tr|E9Q3L4|E9Q3L4\_MOUSE sp|P56960|EXOSX\_MOUSE tr|Q8K366|Q8K366\_MOUSE sp|Q3U381|ZN692\_MOUSE sp|O88572|LRP6\_MOUSE tr|E9PZD2|E9PZD2\_MOUSE sp|Q3TN34|MILK2\_MOUSE tr|A0A087WQS0|A0A087WQS0\_MOUSE tr|E9PWP9|E9PWP9\_MOUSE tr|A0A087WRU0|A0A087WRU0\_MOUSE tr|A0A087WQM0|A0A087WQM0\_MOUSE sp|Q99KY4-2|GAK\_MOUSE sp|Q99KY4|GAK\_MOUSE sp|P70324-2|TBX3\_MOUSE sp|P70289|PTPRV\_MOUSE sp|P70324|TBX3\_MOUSE tr|J3QMF0|J3QMF0\_MOUSE sp|Q8VIG0-3|ZCH14\_MOUSE tr|A2AEQ5|A2AEQ5\_MOUSE sp|P70303|PYRG2\_MOUSE sp|Q9QXB9|DRG2\_MOUSE sp|Q9WTS6-2|TEN3\_MOUSE sp|Q91ZP3-2|LPIN1\_MOUSE tr|E9QKQ5|E9QKQ5\_MOUSE sp|Q91ZP3|LPIN1\_MOUSE sp|P15975|UBP53\_MOUSE tr|Q8VD69|Q8VD69\_MOUSE sp|Q8K4S1|PLCE1\_MOUSE tr|S4R1Q8|S4R1Q8\_MOUSE sp|Q9D4H9-2|PHF14\_MOUSE tr|J3QMZ5|J3QMZ5\_MOUSE sp|Q9D4H9-3|PHF14\_MOUSE tr|G5E8S0|G5E8S0\_MOUSE sp|Q9D4H9|PHF14\_MOUSE tr|E9PYA9|E9PYA9\_MOUSE sp|Q8BRK9|MA2A2\_MOUSE sp|Q02789|CAC1S\_MOUSE tr|E9Q0T8|E9Q0T8\_MOUSE sp|P29699|FETUA\_MOUSE sp|O35400-2|ST2B1\_MOUSE sp|O35400|ST2B1\_MOUSE tr|E9QKC1|E9QKC1\_MOUSE sp|Q9QYR6-2|MAP1A\_MOUSE sp|Q9QYR6|MAP1A\_MOUSE tr|A2ARP8|A2ARP8\_MOUSE sp|Q6DIC0|SMCA2\_MOUSE tr|G3X9B1|G3X9B1\_MOUSE tr|S4R1Y1|S4R1Y1\_MOUSE tr|E9QP09|E9QP09\_MOUSE tr|E9PVG8|E9PVG8\_MOUSE sp|O35595|PTC2\_MOUSE sp|Q8VHJ5|MARK1\_MOUSE sp|P57748|MMP20\_MOUSE sp|Q99J21-2|MCLN1\_MOUSE sp|Q99J21|MCLN1\_MOUSE tr|G3X9C3|G3X9C3\_MOUSE sp|Q8BZL4|GPR22\_MOUSE sp|Q9QXY1|ZO3\_MOUSE sp|Q91XL9|OSBL1\_MOUSE sp|Q7TT41|MOXD2\_MOUSE sp|A2AHC3|CAMP1\_MOUSE tr|A2A4B1|A2A4B1\_MOUSE tr|A2A4B3|A2A4B3\_MOUSE sp|Q99PI4|LPIN3\_MOUSE tr|E9PVJ7|E9PVJ7\_MOUSE tr|K7N727|K7N727\_MOUSE sp|Q62396|ZFP92\_MOUSE tr|Q91V89|Q91V89\_MOUSE tr|F8VPJ6|F8VPJ6\_MOUSE sp|Q8BZ32|ASXL2\_MOUSE tr|D3Z4M6|D3Z4M6\_MOUSE tr|B2RQG2|B2RQG2\_MOUSE sp|Q6I6G8|HECW2\_MOUSE sp|Q80VA0-2|GALT7\_MOUSE sp|Q80VA0|GALT7\_MOUSE sp|Q149L7-2|CRTAM\_MOUSE sp|P00493|HPRT\_MOUSE sp|Q99NE5-7|RIMS1\_MOUSE tr|E9PWP6|E9PWP6\_MOUSE sp|Q8CCB4|VPS53\_MOUSE tr|E9PXG9|E9PXG9\_MOUSE sp|Q6ZPY5|ZN507\_MOUSE sp|O88554|PARP2\_MOUSE sp|B2RWW0|TAGAP\_MOUSE tr|F6X819|F6X819\_MOUSE sp|Q02248|CTNB1\_MOUSE tr|E9Q6A9|E9Q6A9\_MOUSE sp|Q5DTT1|K2022\_MOUSE sp|Q5DTT1-2|K2022\_MOUSE tr|Q8BGR1|Q8BGR1\_MOUSE sp|P35235-2|PTN11\_MOUSE sp|P35235|PTN11\_MOUSE sp|E9Q7X6-3|HEG1\_MOUSE tr|B1AXI9|B1AXI9\_MOUSE tr|J3QMP9|J3QMP9\_MOUSE sp|Q62036|CP131\_MOUSE tr|Q8BU47|Q8BU47\_MOUSE sp|Q8BQQ1-2|ZDH14\_MOUSE tr|F8VPJ7|F8VPJ7\_MOUSE sp|Q3UPP8|CEP63\_MOUSE sp|Q3UPP8-3|CEP63\_MOUSE sp|Q8VBT1|TXLNB\_MOUSE sp|Q80VK6-2|ARH38\_MOUSE sp|Q80VK6-1|ARH38\_MOUSE tr|E0CXB4|E0CXB4\_MOUSE sp|Q8BJI1|S6A17\_MOUSE sp|Q69ZN7-4|MYOF\_MOUSE sp|Q6ZQK5-2|ACAP2\_MOUSE sp|Q6ZQK5|ACAP2\_MOUSE tr|Q0VDM6|Q0VDM6\_MOUSE sp|O35409|FOLH1\_MOUSE sp|Q8VD75|HIP1\_MOUSE sp|Q64737|PUR2\_MOUSE sp|Q62187|TTF1\_MOUSE tr|J3QM90|J3QM90\_MOUSE sp|Q5GIG6|TNI3K\_MOUSE sp|Q5GIG6-2|TNI3K\_MOUSE sp|Q6P9J5|KANK4\_MOUSE sp|Q8C1W1|VASH1\_MOUSE sp|Q61464-4|ZN638\_MOUSE tr|E9QML5|E9QML5\_MOUSE sp|Q80VA0-3|GALT7\_MOUSE tr|E9Q5E2|E9Q5E2\_MOUSE sp|P16627|HS71L\_MOUSE tr|D6REV1|D6REV1\_MOUSE tr|G3UZC8|G3UZC8\_MOUSE tr|F6WV21|F6WV21\_MOUSE tr|L7N2E8|L7N2E8\_MOUSE tr|L7N480|L7N480\_MOUSE sp|Q0GGX2|ZN541\_MOUSE tr|D3YXL4|D3YXL4\_MOUSE sp|Q14BI7-2|TDRD9\_MOUSE sp|Q14BI7|TDRD9\_MOUSE sp|Q63810|CANB1\_MOUSE sp|Q63810-2|CANB1\_MOUSE sp|P13542|MYH8\_MOUSE sp|Q5FWH7|S39AC\_MOUSE tr|S4R2S0|S4R2S0\_MOUSE tr|S4R1C9|S4R1C9\_MOUSE sp|Q9CS84-5|NRX1A\_MOUSE sp|Q6NWW5-2|KIF24\_MOUSE sp|Q6NWW5|KIF24\_MOUSE sp|P80313|TCPH\_MOUSE sp|P08121|CO3A1\_MOUSE sp|P97868|RBBP6\_MOUSE sp|Q9JLI7|SPAG6\_MOUSE tr|Q8C391|Q8C391\_MOUSE tr|Q9CXE1|Q9CXE1\_MOUSE sp|Q9WV30-3|NFAT5\_MOUSE tr|Q80TR0|Q80TR0\_MOUSE sp|P59862|CAD26\_MOUSE tr|Q3V1P3|Q3V1P3\_MOUSE sp|Q80WC1-2|UBN2\_MOUSE tr|F6TB64|F6TB64\_MOUSE sp|Q80WC1-4|UBN2\_MOUSE sp|Q80WC1|UBN2\_MOUSE sp|Q80WC1-3|UBN2\_MOUSE sp|Q9EPN1-3|NBEA\_MOUSE tr|E9PYH6|E9PYH6\_MOUSE sp|Q9EPN1|NBEA\_MOUSE tr|Q8HWE5|Q8HWE5\_MOUSE tr|Q8HWE6|Q8HWE6\_MOUSE tr|A2ALV5|A2ALV5\_MOUSE sp|O70589-5|CSKP\_MOUSE sp|O70589|CSKP\_MOUSE sp|O70589-4|CSKP\_MOUSE sp|O70589-2|CSKP\_MOUSE sp|Q921M3|SF3B3\_MOUSE sp|Q3TBD2|HMHA1\_MOUSE tr|G3X9Q3|G3X9Q3\_MOUSE sp|Q3TBD2-2|HMHA1\_MOUSE tr|D3Z7R7|D3Z7R7\_MOUSE tr|M0QWH1|M0QWH1\_MOUSE sp|Q8BGE5-4|FANCM\_MOUSE tr|F6U6G6|F6U6G6\_MOUSE sp|Q3UH60-2|DIP2B\_MOUSE tr|B2RQC7|B2RQC7\_MOUSE tr|D3Z5G8|D3Z5G8\_MOUSE sp|Q7TQ07|DPOLN\_MOUSE sp|Q7TQ07-2|DPOLN\_MOUSE sp|O89084-3|PDE4A\_MOUSE tr|G5E8C4|G5E8C4\_MOUSE sp|Q8BRH0|TMTC3\_MOUSE sp|Q9WTR1|TRPV2\_MOUSE sp|Q45VK7|DYHC2\_MOUSE sp|Q45VK7-2|DYHC2\_MOUSE sp|Q91WG2|RABE2\_MOUSE sp|P42859|HD\_MOUSE tr|G3X9H5|G3X9H5\_MOUSE sp|Q9JLB4|CUBN\_MOUSE sp|Q9CXV0|ISL2\_MOUSE tr|E9Q8T1|E9Q8T1\_MOUSE sp|Q9CRD0|OCAD1\_MOUSE sp|Q80X90|FLNB\_MOUSE sp|Q8K2F8|LS14A\_MOUSE tr|E9PUM9|E9PUM9\_MOUSE tr|E9Q7X7|E9Q7X7\_MOUSE sp|Q8CDN1|CC020\_MOUSE tr|E9Q839|E9Q839\_MOUSE sp|Q6P9P0|F178A\_MOUSE sp|Q69ZT1-2|FAN1\_MOUSE tr|Q4VAD4|Q4VAD4\_MOUSE sp|Q8R3N1|NOP14\_MOUSE tr|D6RI87|D6RI87\_MOUSE tr|A2AR07|A2AR07\_MOUSE sp|P32067|LA\_MOUSE tr|Q9CV82|Q9CV82\_MOUSE sp|P51557|STAR\_MOUSE sp|Q8VI33|TAF9\_MOUSE sp|Q8BNE1-3|F115A\_MOUSE tr|E9Q7P2|E9Q7P2\_MOUSE sp|Q3V079|BBOF1\_MOUSE tr|E9PYI7|E9PYI7\_MOUSE tr|E9PYF4|E9PYF4\_MOUSE tr|F6VG99|F6VG99\_MOUSE sp|Q68FE6|FA65A\_MOUSE tr|Q6PCS8|Q6PCS8\_MOUSE sp|A2AH22-2|AMRA1\_MOUSE tr|F6XQW1|F6XQW1\_MOUSE sp|A2AH22-6|AMRA1\_MOUSE sp|A2AH22-7|AMRA1\_MOUSE sp|A2AH22|AMRA1\_MOUSE sp|Q0ZCJ7|MASTR\_MOUSE sp|Q0ZCJ7-2|MASTR\_MOUSE sp|Q6RHR9-3|MAGI1\_MOUSE sp|Q6RHR9-2|MAGI1\_MOUSE sp|Q6RHR9|MAGI1\_MOUSE tr|E9QLN4|E9QLN4\_MOUSE tr|G3UWI0|G3UWI0\_MOUSE tr|G3X8T0|G3X8T0\_MOUSE tr|D3YWG8|D3YWG8\_MOUSE tr|A0A087WNQ1|A0A087WNQ1\_MOUSE tr|Q9JLI2|Q9JLI2\_MOUSE sp|Q99MV5|M10L1\_MOUSE tr|E9PWI3|E9PWI3\_MOUSE sp|Q8QZW2|FEV\_MOUSE sp|Q8C0L9-3|GPCP1\_MOUSE tr|A2AJW5|A2AJW5\_MOUSE tr|L7N2E1|L7N2E1\_MOUSE tr|L7N1Z4|L7N1Z4\_MOUSE tr|A8Y5P4|A8Y5P4\_MOUSE sp|A2AJI0-2|MA7D1\_MOUSE sp|A2AJI0|MA7D1\_MOUSE tr|A2AJI1|A2AJI1\_MOUSE sp|Q69ZT1-4|FAN1\_MOUSE sp|Q69ZT1-3|FAN1\_MOUSE tr|E9Q8Q6|E9Q8Q6\_MOUSE tr|A2AST1|A2AST1\_MOUSE sp|B7ZCC9|GP112\_MOUSE sp|Q08460-3|KCMA1\_MOUSE sp|Q08460|KCMA1\_MOUSE sp|Q08460-4|KCMA1\_MOUSE tr|E9QAU4|E9QAU4\_MOUSE sp|B2RRF6|Z518A\_MOUSE sp|Q8K0U4|HS12A\_MOUSE sp|Q9DBY8|NVL\_MOUSE sp|Q6JHY2|SMGC\_MOUSE sp|Q6JHY2-2|SMGC\_MOUSE sp|P17433|SPI1\_MOUSE tr|E9Q0V6|E9Q0V6\_MOUSE sp|B1AXH1|NHSL2\_MOUSE sp|Q3TZM9|ALG11\_MOUSE tr|A0A087WPL6|A0A087WPL6\_MOUSE tr|M0QWH9|M0QWH9\_MOUSE sp|O54929|WSB2\_MOUSE tr|E9Q8J9|E9Q8J9\_MOUSE sp|Q62388|ATM\_MOUSE sp|Q9JMB1|THEG\_MOUSE sp|Q9JMB1-2|THEG\_MOUSE tr|Q8R2Q3|Q8R2Q3\_MOUSE sp|Q91WR3-2|ASCC2\_MOUSE sp|P97310|MCM2\_MOUSE sp|Q91WR3-3|ASCC2\_MOUSE sp|Q91WR3|ASCC2\_MOUSE tr|Q5SV54|Q5SV54\_MOUSE tr|G3X8U8|G3X8U8\_MOUSE sp|Q6PCN3|TTBK1\_MOUSE tr|E9Q088|E9Q088\_MOUSE sp|O88622-2|PARG\_MOUSE tr|E9Q7T9|E9Q7T9\_MOUSE sp|O88622|PARG\_MOUSE sp|Q05512-2|MARK2\_MOUSE tr|E9QL65|E9QL65\_MOUSE tr|G3UWS8|G3UWS8\_MOUSE tr|O19443|O19443\_MOUSE tr|G5E8G3|G5E8G3\_MOUSE tr|Q6DFY2|Q6DFY2\_MOUSE sp|Q8CAQ8-4|MIC60\_MOUSE sp|Q99MV7-4|RNF17\_MOUSE sp|Q99MV7-2|RNF17\_MOUSE tr|S4R2K9|S4R2K9\_MOUSE sp|A6H690|IQCAL\_MOUSE sp|Q8K595|MCLN2\_MOUSE sp|Q8K595-2|MCLN2\_MOUSE sp|Q3UQU0|BRD9\_MOUSE sp|Q9ESN2|TRI39\_MOUSE sp|Q9ESN2-2|TRI39\_MOUSE sp|Q8CHE4|PHLP1\_MOUSE sp|B9EJA2-3|CTTB2\_MOUSE tr|Q3UV08|Q3UV08\_MOUSE sp|A6H6E9|TT23L\_MOUSE sp|Q6NWV3|IF122\_MOUSE tr|A0A087WNM2|A0A087WNM2\_MOUSE sp|Q7TNR6|IGS21\_MOUSE tr|A0A087WR08|A0A087WR08\_MOUSE tr|E9Q9G8|E9Q9G8\_MOUSE sp|Q99KU1|DHDDS\_MOUSE tr|A3KGL0|A3KGL0\_MOUSE tr|A3KGL2|A3KGL2\_MOUSE tr|A3KGL3|A3KGL3\_MOUSE sp|Q99KU1-2|DHDDS\_MOUSE sp|Q6NWV3-2|IF122\_MOUSE sp|Q3U1N2-2|SRBP2\_MOUSE sp|Q3U1N2|SRBP2\_MOUSE sp|Q8BGA5|KRR1\_MOUSE tr|G3UZA0|G3UZA0\_MOUSE sp|Q9Z2E2-4|MBD1\_MOUSE sp|Q9Z2E2-2|MBD1\_MOUSE sp|Q9Z2E2|MBD1\_MOUSE sp|Q9JHX6|ALLC\_MOUSE sp|Q8CGB6-3|TENC1\_MOUSE sp|Q8CGB6|TENC1\_MOUSE sp|Q8CGB6-2|TENC1\_MOUSE sp|Q8CGB6-4|TENC1\_MOUSE sp|O88685|PRS6A\_MOUSE tr|A2AGN7|A2AGN7\_MOUSE sp|Q3UJB9|EDC4\_MOUSE sp|Q3UJB9-2|EDC4\_MOUSE tr|E9QAN2|E9QAN2\_MOUSE sp|A2AM05-4|CNTLN\_MOUSE tr|E9Q7E1|E9Q7E1\_MOUSE sp|Q6P5G0|MK04\_MOUSE tr|E9Q1Q8|E9Q1Q8\_MOUSE tr|D3Z0A2|D3Z0A2\_MOUSE tr|E9PV90|E9PV90\_MOUSE sp|Q03311|CHLE\_MOUSE sp|Q9ESN2-3|TRI39\_MOUSE tr|E9PY45|E9PY45\_MOUSE sp|Q9D6M3|GHC1\_MOUSE sp|Q9EST3|4ET\_MOUSE sp|Q9WTS5|TEN2\_MOUSE tr|Q8BGF0|Q8BGF0\_MOUSE sp|Q9D8N1|CK024\_MOUSE sp|Q8R0H9|GGA1\_MOUSE tr|E9PVY0|E9PVY0\_MOUSE tr|D3YYN8|D3YYN8\_MOUSE sp|Q3UU96|MRCKA\_MOUSE tr|Q3TZR9|Q3TZR9\_MOUSE sp|Q61474|MSI1H\_MOUSE sp|G5E829|AT2B1\_MOUSE sp|P97793|ALK\_MOUSE sp|Q60674|NR1D2\_MOUSE tr|D3Z117|D3Z117\_MOUSE tr|D3YUK3|D3YUK3\_MOUSE sp|Q9ESJ4|SPN90\_MOUSE tr|E9Q6Y8|E9Q6Y8\_MOUSE sp|Q0KL02-3|TRIO\_MOUSE tr|D3YU08|D3YU08\_MOUSE sp|Q8BWB1|SYP2L\_MOUSE tr|B2RQK7|B2RQK7\_MOUSE tr|F8VQE0|F8VQE0\_MOUSE tr|E9Q7B5|E9Q7B5\_MOUSE tr|F6ZZB1|F6ZZB1\_MOUSE sp|P52480|KPYM\_MOUSE sp|P52480-2|KPYM\_MOUSE tr|E9Q8L9|E9Q8L9\_MOUSE sp|Q3UVY1|GP149\_MOUSE tr|F6VQ96|F6VQ96\_MOUSE tr|E9PXP7|E9PXP7\_MOUSE sp|Q8BZ60|STON2\_MOUSE sp|Q7TSU7|KIRR2\_MOUSE sp|Q7TSU7-2|KIRR2\_MOUSE tr|Z4YJP0|Z4YJP0\_MOUSE sp|Q8CGK3|LONM\_MOUSE sp|Q6NXI6-2|RPRD2\_MOUSE sp|Q02789-2|CAC1S\_MOUSE tr|E9PWD1|E9PWD1\_MOUSE sp|O35904|PK3CD\_MOUSE tr|Q3TBW3|Q3TBW3\_MOUSE tr|Q3T9Y0|Q3T9Y0\_MOUSE tr|Q3UDT3|Q3UDT3\_MOUSE tr|Q8CI98|Q8CI98\_MOUSE tr|E0CYI9|E0CYI9\_MOUSE sp|Q8JZV7|NAGA\_MOUSE sp|Q8BH06-5|I17RE\_MOUSE sp|Q14DN9|AKD1B\_MOUSE sp|Q14DN9-2|AKD1B\_MOUSE tr|E9Q5Z7|E9Q5Z7\_MOUSE sp|Q8R5H6|WASF1\_MOUSE sp|Q99K67|AASS\_MOUSE sp|Q6ZQ93-4|UBP34\_MOUSE sp|Q69ZS7-2|HBS1L\_MOUSE sp|A6H584|CO6A5\_MOUSE tr|A0A087WNL6|A0A087WNL6\_MOUSE tr|F6RDB7|F6RDB7\_MOUSE tr|G1K381|G1K381\_MOUSE tr|Q9D2F1|Q9D2F1\_MOUSE tr|Q5SZ93|Q5SZ93\_MOUSE tr|E9PYR6|E9PYR6\_MOUSE sp|Q9ESF1|OTOF\_MOUSE sp|Q9ESF1-3|OTOF\_MOUSE tr|G3X9M9|G3X9M9\_MOUSE tr|D3YXV0|D3YXV0\_MOUSE sp|Q9ESF1-2|OTOF\_MOUSE tr|G3X901|G3X901\_MOUSE sp|Q80U93|NU214\_MOUSE tr|G3UYY4|G3UYY4\_MOUSE O43790 sp|Q5QNQ9|CORA1\_MOUSE tr|F7BIK4|F7BIK4\_MOUSE sp|O08599-2|STXB1\_MOUSE sp|O08599|STXB1\_MOUSE sp|P59016|VP33B\_MOUSE sp|B1AUH1-2|PTPRU\_MOUSE sp|B1AUH1|PTPRU\_MOUSE sp|Q8R4D5|TRPM8\_MOUSE tr|B7ZCD1|B7ZCD1\_MOUSE sp|B7ZCC9-2|GP112\_MOUSE sp|Q7TNB8|SBNO2\_MOUSE sp|Q7TNB8-2|SBNO2\_MOUSE tr|Q91XY7|Q91XY7\_MOUSE sp|Q9Z1M8|RED\_MOUSE tr|Q8K0M7|Q8K0M7\_MOUSE sp|D3YZV8|CCDC8\_MOUSE sp|Q80XE1-1|RIC8B\_MOUSE sp|P51436|DRD4\_MOUSE sp|Q5SVL6|RPGP2\_MOUSE sp|Q5SVL6-2|RPGP2\_MOUSE tr|E9Q4X2|E9Q4X2\_MOUSE tr|Q80Z68|Q80Z68\_MOUSE tr|B1AUY2|B1AUY2\_MOUSE tr|E9PYS6|E9PYS6\_MOUSE sp|Q52KB6|C2CD3\_MOUSE tr|E9Q526|E9Q526\_MOUSE sp|Q52KB6-2|C2CD3\_MOUSE sp|Q52KB6-3|C2CD3\_MOUSE sp|Q80YA7|DPP8\_MOUSE tr|E9PUI7|E9PUI7\_MOUSE sp|Q3U3T8-2|WDR62\_MOUSE sp|Q9QZZ4-2|MYO15\_MOUSE sp|Q9QZZ4|MYO15\_MOUSE sp|Q9QZM0|UBQL2\_MOUSE sp|P70699|LYAG\_MOUSE tr|D3Z592|D3Z592\_MOUSE tr|E9PY46|E9PY46\_MOUSE sp|Q0VBV7|K1377\_MOUSE sp|Q8BGZ3|DCA12\_MOUSE tr|E9PYQ9|E9PYQ9\_MOUSE sp|Q9WU62|INCE\_MOUSE sp|Q9WU62-2|INCE\_MOUSE sp|Q80YE4-4|LMTK1\_MOUSE sp|P56844|TCLB4\_MOUSE sp|O55201-2|SPT5H\_MOUSE sp|Q0KL02-2|TRIO\_MOUSE sp|Q9CQV3|SPB11\_MOUSE sp|Q5SNZ0-2|GRDN\_MOUSE sp|Q5SNZ0-3|GRDN\_MOUSE sp|Q5SNZ0|GRDN\_MOUSE tr|E9Q4D1|E9Q4D1\_MOUSE tr|F6RTQ0|F6RTQ0\_MOUSE sp|Q6WKZ8-2|UBR2\_MOUSE sp|Q8CI04|COG3\_MOUSE sp|Q6WKZ8|UBR2\_MOUSE tr|D3YX90|D3YX90\_MOUSE sp|Q19LI2|A1BG\_MOUSE tr|D3Z2W7|D3Z2W7\_MOUSE tr|E9PV60|E9PV60\_MOUSE tr|E9Q2M9|E9Q2M9\_MOUSE tr|A0A087WRI9|A0A087WRI9\_MOUSE tr|D3YY07|D3YY07\_MOUSE sp|Q99M80|PTPRT\_MOUSE sp|Q99M80-3|PTPRT\_MOUSE tr|B1AQN2|B1AQN2\_MOUSE sp|Q99M80-4|PTPRT\_MOUSE sp|Q99M80-5|PTPRT\_MOUSE sp|Q8BG26-2|RUSC1\_MOUSE tr|G3X907|G3X907\_MOUSE sp|Q9WTS6|TEN3\_MOUSE tr|F6RJC1|F6RJC1\_MOUSE tr|B2RQ80|B2RQ80\_MOUSE tr|E9PUL9|E9PUL9\_MOUSE tr|E0CZD7|E0CZD7\_MOUSE tr|E0CY98|E0CY98\_MOUSE sp|P83510|TNIK\_MOUSE tr|E0CXD6|E0CXD6\_MOUSE tr|B9EKN8|B9EKN8\_MOUSE tr|E0CZF8|E0CZF8\_MOUSE sp|P83510-2|TNIK\_MOUSE sp|Q3UFQ8|LR16B\_MOUSE sp|Q9Z206|ARHG8\_MOUSE tr|E9PWM3|E9PWM3\_MOUSE sp|Q62398|CNGA2\_MOUSE tr|E9Q5A7|E9Q5A7\_MOUSE tr|G5E8G5|G5E8G5\_MOUSE sp|O54990-4|PROM1\_MOUSE tr|Q8CDK8|Q8CDK8\_MOUSE sp|O54990-3|PROM1\_MOUSE tr|G3X9J8|G3X9J8\_MOUSE sp|O54990-6|PROM1\_MOUSE sp|O54990|PROM1\_MOUSE sp|O54990-5|PROM1\_MOUSE sp|O54990-2|PROM1\_MOUSE tr|F6ZSN2|F6ZSN2\_MOUSE sp|Q3TZ89-2|SC31B\_MOUSE tr|J3QN27|J3QN27\_MOUSE tr|F7BHL0|F7BHL0\_MOUSE tr|F6WSZ3|F6WSZ3\_MOUSE sp|Q08460-5|KCMA1\_MOUSE sp|Q08460-2|KCMA1\_MOUSE tr|F6XW53|F6XW53\_MOUSE tr|F6V0P5|F6V0P5\_MOUSE tr|J3QMT8|J3QMT8\_MOUSE tr|J3QP84|J3QP84\_MOUSE tr|F7C0E4|F7C0E4\_MOUSE tr|Z4YKJ7|Z4YKJ7\_MOUSE sp|Q8JZR4|EAA5\_MOUSE sp|Q8C6S9|CL055\_MOUSE sp|Q3TKT4|SMCA4\_MOUSE sp|Q3TKT4-2|SMCA4\_MOUSE tr|G5E8L9|G5E8L9\_MOUSE tr|D3YY08|D3YY08\_MOUSE sp|Q80UG8|TTLL4\_MOUSE tr|Q62458|Q62458\_MOUSE tr|J3QK44|J3QK44\_MOUSE tr|A0A087WSM5|A0A087WSM5\_MOUSE tr|J3QK67|J3QK67\_MOUSE tr|Q62460|Q62460\_MOUSE tr|Q5FWB5|Q5FWB5\_MOUSE tr|Q5FWD4|Q5FWD4\_MOUSE tr|G3UW40|G3UW40\_MOUSE tr|Q5NCB3|Q5NCB3\_MOUSE tr|F6TZV3|F6TZV3\_MOUSE sp|Q9Z0M6|CD97\_MOUSE tr|E9QMJ5|E9QMJ5\_MOUSE sp|Q9Z0M6-3|CD97\_MOUSE sp|Q9Z0M6-2|CD97\_MOUSE tr|Q8K205|Q8K205\_MOUSE tr|Q9D4G5|Q9D4G5\_MOUSE sp|Q9Z0H8|CLIP2\_MOUSE sp|Q6ZPF3-2|TIAM2\_MOUSE sp|Q6ZPF3|TIAM2\_MOUSE sp|Q9Z0H8-2|CLIP2\_MOUSE sp|Q9JME5|AP3B2\_MOUSE sp|Q91ZJ9|HYAL1\_MOUSE sp|Q91ZJ9-2|HYAL1\_MOUSE tr|D6RFV7|D6RFV7\_MOUSE tr|F6PYJ6|F6PYJ6\_MOUSE sp|Q3UPP8-2|CEP63\_MOUSE tr|F6UV46|F6UV46\_MOUSE sp|Q3U4H6-2|HEXDC\_MOUSE tr|F6RSJ3|F6RSJ3\_MOUSE sp|P27671|RGRF1\_MOUSE tr|A6PW47|A6PW47\_MOUSE sp|Q99K74-2|MED24\_MOUSE sp|Q99K74|MED24\_MOUSE sp|Q7TT36|GP125\_MOUSE tr|D3Z6W1|D3Z6W1\_MOUSE sp|O70491|STRA6\_MOUSE sp|D3Z750|MRO2A\_MOUSE tr|F6TFN2|F6TFN2\_MOUSE tr|H3BL86|H3BL86\_MOUSE sp|Q923I7|SC5A2\_MOUSE tr|F7CK47|F7CK47\_MOUSE tr|A0A087WNX7|A0A087WNX7\_MOUSE tr|A0A087WQ44|A0A087WQ44\_MOUSE tr|A0A087WP63|A0A087WP63\_MOUSE tr|A0A087WNL7|A0A087WNL7\_MOUSE sp|Q8K298|ANLN\_MOUSE tr|Q5F2A3|Q5F2A3\_MOUSE tr|Q8BXS2|Q8BXS2\_MOUSE sp|Q8BWW9-3|PKN2\_MOUSE tr|Q5F2A4|Q5F2A4\_MOUSE sp|Q9D777|TNF13\_MOUSE tr|Q5F2A1|Q5F2A1\_MOUSE sp|Q8VDC1|FYCO1\_MOUSE sp|Q922J3-2|CLIP1\_MOUSE sp|Q8BQC3-2|IGDC3\_MOUSE sp|Q9WVL3|S12A7\_MOUSE sp|Q9WVL3-2|S12A7\_MOUSE sp|Q9DCS2|CP013\_MOUSE tr|B7ZCR6|B7ZCR6\_MOUSE tr|Q6PHP4|Q6PHP4\_MOUSE sp|Q99K48-2|NONO\_MOUSE sp|Q99K48|NONO\_MOUSE tr|Q3TCD4|Q3TCD4\_MOUSE tr|E9PUY9|E9PUY9\_MOUSE sp|Q9WUR2-2|ECI2\_MOUSE tr|E9PVM6|E9PVM6\_MOUSE tr|E9Q858|E9Q858\_MOUSE tr|E9PYC6|E9PYC6\_MOUSE sp|Q9WUR2|ECI2\_MOUSE tr|E9QKQ3|E9QKQ3\_MOUSE tr|G3X9J0|G3X9J0\_MOUSE sp|Q60841-3|RELN\_MOUSE sp|Q60841|RELN\_MOUSE sp|Q60841-2|RELN\_MOUSE sp|Q60991|CP7B1\_MOUSE sp|Q9DCA5|BRX1\_MOUSE tr|F6WEP7|F6WEP7\_MOUSE sp|Q8VDG6|M3KL4\_MOUSE tr|B7FAU9|B7FAU9\_MOUSE sp|Q8BTM8|FLNA\_MOUSE sp|Q9R1Y5|HIC1\_MOUSE tr|A0A087WNN3|A0A087WNN3\_MOUSE sp|Q7TPM1-2|PRC2B\_MOUSE tr|E9PUN0|E9PUN0\_MOUSE tr|E9Q4T3|E9Q4T3\_MOUSE sp|A7XUY5-2|SKIT5\_MOUSE tr|E9Q072|E9Q072\_MOUSE sp|A7XUY5|SKIT5\_MOUSE sp|Q8C159|TTC22\_MOUSE sp|Q9WV54|ASAH1\_MOUSE tr|L7N451|L7N451\_MOUSE sp|A2AMM0|MURC\_MOUSE sp|Q8BR90-2|CE051\_MOUSE sp|Q8BR90|CE051\_MOUSE tr|E9QKA6|E9QKA6\_MOUSE tr|F6T2B3|F6T2B3\_MOUSE tr|E9PUE3|E9PUE3\_MOUSE tr|L7N2B8|L7N2B8\_MOUSE tr|F6TLB0|F6TLB0\_MOUSE sp|A2RSY1-3|KANL3\_MOUSE sp|Q9QXD8|LIMD1\_MOUSE sp|Q80Y17|L2GL1\_MOUSE sp|Q9WVP6|PAPOB\_MOUSE tr|A1L349|A1L349\_MOUSE tr|D3YWE6|D3YWE6\_MOUSE sp|Q80XP9|WNK3\_MOUSE tr|V9GWU7|V9GWU7\_MOUSE sp|Q80XP9-2|WNK3\_MOUSE sp|Q91YR1|TWF1\_MOUSE sp|Q14B70|HDX\_MOUSE sp|Q14B70-2|HDX\_MOUSE tr|D3YWD1|D3YWD1\_MOUSE sp|P26048|GBRA2\_MOUSE sp|O54851|CXD2\_MOUSE tr|B7ZDF7|B7ZDF7\_MOUSE tr|Q8BMS7|Q8BMS7\_MOUSE Q9NSB4 tr|D3Z640|D3Z640\_MOUSE tr|E9PZU5|E9PZU5\_MOUSE sp|Q5NC83|CG072\_MOUSE sp|P12265|BGLR\_MOUSE sp|Q9R1C7|PR40A\_MOUSE sp|Q80TM9-4|NISCH\_MOUSE tr|E9Q8V6|E9Q8V6\_MOUSE sp|Q8R0I0|ACE2\_MOUSE tr|F6X479|F6X479\_MOUSE sp|P11369|LORF2\_MOUSE tr|A2A6Z8|A2A6Z8\_MOUSE sp|Q03142|FGFR4\_MOUSE sp|Q03142-2|FGFR4\_MOUSE sp|Q9EPQ2|RPGR1\_MOUSE tr|E7CHD7|E7CHD7\_MOUSE tr|F8VPU6|F8VPU6\_MOUSE tr|Q3UHH4|Q3UHH4\_MOUSE tr|E9QKC6|E9QKC6\_MOUSE sp|P53349|M3K1\_MOUSE sp|P00416|COX3\_MOUSE tr|Q7JCX7|Q7JCX7\_MOUSE sp|O55201|SPT5H\_MOUSE tr|E9PXF0|E9PXF0\_MOUSE sp|Q9Z277-2|BAZ1B\_MOUSE sp|Q9Z277|BAZ1B\_MOUSE sp|Q00547|HMMR\_MOUSE sp|Q00547-2|HMMR\_MOUSE sp|Q9WV38|GTR5\_MOUSE tr|H3BIX4|H3BIX4\_MOUSE sp|Q8R317|UBQL1\_MOUSE sp|E9Q349-2|WDR25\_MOUSE tr|Q91Y19|Q91Y19\_MOUSE tr|B7ZDF5|B7ZDF5\_MOUSE tr|Q3UJF1|Q3UJF1\_MOUSE sp|Q3UHK8|TNR6A\_MOUSE tr|A2AWS5|A2AWS5\_MOUSE tr|F6ZMJ4|F6ZMJ4\_MOUSE sp|Q9Z2V6|HDAC5\_MOUSE sp|Q9EP53-2|TSC1\_MOUSE sp|Q9EP53-3|TSC1\_MOUSE sp|Q9EP53|TSC1\_MOUSE sp|Q5SW75|SSH2\_MOUSE sp|Q5SW75-4|SSH2\_MOUSE sp|Q5SW75-2|SSH2\_MOUSE tr|Q8VFI0|Q8VFI0\_MOUSE tr|S4R2L9|S4R2L9\_MOUSE sp|Q3UHI0-2|CCSE2\_MOUSE sp|Q99ML9-2|RN111\_MOUSE sp|Q99ML9|RN111\_MOUSE sp|Q8C5U9|UBQL3\_MOUSE sp|Q61584-3|FXR1\_MOUSE sp|Q61584-5|FXR1\_MOUSE sp|Q61584-4|FXR1\_MOUSE tr|Q8VCT8|Q8VCT8\_MOUSE tr|G3UZ41|G3UZ41\_MOUSE sp|Q80YF9|RHG33\_MOUSE tr|F8VQ70|F8VQ70\_MOUSE sp|Q9D9B0|CCD70\_MOUSE sp|Q62190|RON\_MOUSE tr|E9Q5A3|E9Q5A3\_MOUSE sp|Q9D832|DNJB4\_MOUSE sp|Q99N50-3|SYTL2\_MOUSE sp|Q99N50|SYTL2\_MOUSE sp|Q99N50-4|SYTL2\_MOUSE sp|Q99N50-6|SYTL2\_MOUSE sp|Q569Z5|DDX46\_MOUSE sp|Q569Z5-2|DDX46\_MOUSE tr|F8WHR6|F8WHR6\_MOUSE sp|Q99LE6|ABCF2\_MOUSE sp|B2KFW1|ZSC20\_MOUSE sp|B2KFW1-2|ZSC20\_MOUSE sp|Q9WV70|NOC2L\_MOUSE tr|E9PYK3|E9PYK3\_MOUSE tr|F6WWY8|F6WWY8\_MOUSE sp|A2AUC9|KLH41\_MOUSE sp|Q80YX1-5|TENA\_MOUSE sp|Q80YX1-4|TENA\_MOUSE sp|Q99PU8|DHX30\_MOUSE tr|D3Z2J3|D3Z2J3\_MOUSE sp|Q99PU8-3|DHX30\_MOUSE sp|Q99PU8-2|DHX30\_MOUSE sp|A4Q9E8-2|TTLL6\_MOUSE sp|A4Q9E8|TTLL6\_MOUSE tr|F6QF13|F6QF13\_MOUSE tr|E9Q2Y1|E9Q2Y1\_MOUSE tr|F6Z3M4|F6Z3M4\_MOUSE tr|D3Z633|D3Z633\_MOUSE sp|Q8BXL9-5|IFFO1\_MOUSE tr|A2AD93|A2AD93\_MOUSE tr|F6YK91|F6YK91\_MOUSE sp|Q03141-3|MARK3\_MOUSE sp|Q03141|MARK3\_MOUSE sp|Q03141-2|MARK3\_MOUSE sp|Q99PP9-3|TRI16\_MOUSE sp|Q99PP9|TRI16\_MOUSE sp|Q99PP9-2|TRI16\_MOUSE sp|A2A7S8-2|K1522\_MOUSE sp|A2A7S8-4|K1522\_MOUSE sp|A2A7S8|K1522\_MOUSE sp|A2A7S8-3|K1522\_MOUSE tr|B0V2P6|B0V2P6\_MOUSE sp|Q9Z0J1|RECK\_MOUSE tr|E9Q1L0|E9Q1L0\_MOUSE tr|E9Q983|E9Q983\_MOUSE sp|Q8C9B9-3|DIDO1\_MOUSE sp|Q03391|NMDE4\_MOUSE sp|A2ADZ8|IQCC\_MOUSE sp|Q07563-2|COHA1\_MOUSE sp|P24822|PPBI\_MOUSE tr|J3QK46|J3QK46\_MOUSE sp|Q8BXF8|ACTT3\_MOUSE sp|Q3UVX5-2|GRM5\_MOUSE sp|Q6GQT1|A2MP\_MOUSE sp|P48193-2|41\_MOUSE tr|A2A841|A2A841\_MOUSE sp|P48193|41\_MOUSE tr|A2A842|A2A842\_MOUSE sp|Q8CHY3|DYM\_MOUSE tr|E9QPX8|E9QPX8\_MOUSE tr|F7CNJ1|F7CNJ1\_MOUSE tr|Q91Y09|Q91Y09\_MOUSE tr|A0A087WS73|A0A087WS73\_MOUSE tr|G3UZF1|G3UZF1\_MOUSE tr|Q8VGG6|Q8VGG6\_MOUSE sp|Q80VW7-4|AKNA\_MOUSE sp|Q80VW7-3|AKNA\_MOUSE sp|Q8BLG0-2|PHF20\_MOUSE sp|Q80VW7|AKNA\_MOUSE tr|A0A087WS38|A0A087WS38\_MOUSE tr|E9Q4F2|E9Q4F2\_MOUSE sp|Q9JHJ0|TMOD3\_MOUSE sp|Q8BKG4|FZD10\_MOUSE tr|E9Q8K1|E9Q8K1\_MOUSE tr|D3Z201|D3Z201\_MOUSE tr|E0CZA7|E0CZA7\_MOUSE sp|Q8CJI4|H1FNT\_MOUSE tr|F6W8R9|F6W8R9\_MOUSE tr|Q6DFZ2|Q6DFZ2\_MOUSE sp|Q6ZWQ0-3|SYNE2\_MOUSE sp|Q6P9J9-2|ANO6\_MOUSE sp|Q8R2Z3|S26A7\_MOUSE sp|Q922C1|CS044\_MOUSE tr|E9Q7C0|E9Q7C0\_MOUSE tr|E9QJT8|E9QJT8\_MOUSE sp|Q3UZY0|SFI1\_MOUSE tr|E9Q8V3|E9Q8V3\_MOUSE tr|E9Q799|E9Q799\_MOUSE sp|Q3UZY0-2|SFI1\_MOUSE tr|E9Q7G4|E9Q7G4\_MOUSE sp|Q3UHR0|BAHC1\_MOUSE sp|Q6ZPY2|SMG5\_MOUSE sp|Q6ZQJ5-2|DNA2\_MOUSE sp|O09039|SH2B3\_MOUSE tr|D3Z3Y5|D3Z3Y5\_MOUSE sp|Q9WTR2|M3K6\_MOUSE sp|Q91YK2|RRP1B\_MOUSE tr|D3YUH8|D3YUH8\_MOUSE sp|Q499E4|DZI1L\_MOUSE sp|P50636|RN19A\_MOUSE sp|Q8C006|TRI35\_MOUSE tr|Q921G9|Q921G9\_MOUSE tr|Q9D5W6|Q9D5W6\_MOUSE sp|O70340|NPTX2\_MOUSE sp|O88329|MYO1A\_MOUSE tr|B8JJH5|B8JJH5\_MOUSE sp|Q9QXE2|DPOLL\_MOUSE tr|F6Q1W8|F6Q1W8\_MOUSE tr|E9Q2C0|E9Q2C0\_MOUSE tr|A2BFF5|A2BFF5\_MOUSE sp|O88487|DC1I2\_MOUSE tr|A2BFF8|A2BFF8\_MOUSE tr|A2BFF9|A2BFF9\_MOUSE tr|Q3TPJ8|Q3TPJ8\_MOUSE sp|Q14AT2|TEX11\_MOUSE sp|Q5SZV5|K0319\_MOUSE sp|Q8CI96-2|CLIP4\_MOUSE tr|Q8C9M8|Q8C9M8\_MOUSE tr|G3X9U2|G3X9U2\_MOUSE tr|Q2MHN3|Q2MHN3\_MOUSE sp|Q9Z1T1|AP3B1\_MOUSE tr|B7ZCB9|B7ZCB9\_MOUSE tr|B7ZCB7|B7ZCB7\_MOUSE tr|B7ZCB8|B7ZCB8\_MOUSE tr|F6X4N5|F6X4N5\_MOUSE sp|Q8BVI5|STX16\_MOUSE tr|F7CJY7|F7CJY7\_MOUSE Q86YZ3 sp|Q3TRM4-4|PLPL6\_MOUSE sp|Q9QZZ4-3|MYO15\_MOUSE sp|Q6P5D3-2|DHX57\_MOUSE tr|D3YYA0|D3YYA0\_MOUSE tr|E9PU93|E9PU93\_MOUSE sp|Q91X20|ASH2L\_MOUSE sp|Q68EF8|RPGFL\_MOUSE tr|A2AQ52|A2AQ52\_MOUSE sp|P55014-3|S12A1\_MOUSE sp|Q5PSV9|MDC1\_MOUSE tr|E9QK89|E9QK89\_MOUSE sp|Q80TV8|CLAP1\_MOUSE tr|E9QKH0|E9QKH0\_MOUSE tr|A0A087WQ31|A0A087WQ31\_MOUSE tr|F6WNG1|F6WNG1\_MOUSE tr|S4R1Y6|S4R1Y6\_MOUSE tr|J3QMN6|J3QMN6\_MOUSE sp|A6PWD2-4|FHAD1\_MOUSE sp|Q61137-2|ASTN1\_MOUSE sp|Q61137|ASTN1\_MOUSE sp|Q3UH68-3|LIMC1\_MOUSE tr|G3UXB4|G3UXB4\_MOUSE sp|Q3U308|CTU2\_MOUSE tr|F6XNR8|F6XNR8\_MOUSE tr|E9Q0C6|E9Q0C6\_MOUSE sp|Q61502|E2F5\_MOUSE tr|E9Q011|E9Q011\_MOUSE tr|Q3TLQ0|Q3TLQ0\_MOUSE tr|Q80ZL4|Q80ZL4\_MOUSE tr|Q80X35|Q80X35\_MOUSE sp|Q9JKR6|HYOU1\_MOUSE sp|Q6DI86-3|FAKD1\_MOUSE sp|Q6DI86-2|FAKD1\_MOUSE sp|Q6DI86|FAKD1\_MOUSE sp|P70333|HNRH2\_MOUSE tr|J3QPH6|J3QPH6\_MOUSE tr|Z4YJG1|Z4YJG1\_MOUSE tr|Q5FW96|Q5FW96\_MOUSE sp|O35627-2|NR1I3\_MOUSE sp|O35627|NR1I3\_MOUSE sp|Q769J6|ATS13\_MOUSE tr|F6Y6G7|F6Y6G7\_MOUSE tr|F6SRT8|F6SRT8\_MOUSE tr|E9QAN9|E9QAN9\_MOUSE tr|A2AU91|A2AU91\_MOUSE sp|P70399-3|TP53B\_MOUSE sp|P70399|TP53B\_MOUSE tr|A2AU89|A2AU89\_MOUSE sp|Q920I9-2|WDR7\_MOUSE sp|Q920I9|WDR7\_MOUSE sp|Q80TR8-4|VPRBP\_MOUSE sp|Q80TR8|VPRBP\_MOUSE sp|Q9D9R9|F186A\_MOUSE tr|F8WGM8|F8WGM8\_MOUSE sp|Q9WTN6|S22AL\_MOUSE sp|Q9DCC7|ISC2B\_MOUSE tr|J3QML2|J3QML2\_MOUSE sp|B2RX12|MRP3\_MOUSE sp|B2RX12-3|MRP3\_MOUSE sp|B2RX12-2|MRP3\_MOUSE sp|Q3UY96-3|K1751\_MOUSE sp|Q3UY96-2|K1751\_MOUSE sp|Q3UY96|K1751\_MOUSE sp|Q7TMY8-2|HUWE1\_MOUSE sp|Q8R1G6|PDLI2\_MOUSE sp|Q6P3Y5-3|Z280C\_MOUSE sp|Q6P3Y5|Z280C\_MOUSE tr|G3X8Q1|G3X8Q1\_MOUSE sp|Q69ZW3-2|EHBP1\_MOUSE sp|Q69ZW3|EHBP1\_MOUSE sp|Q6NZG4|DDIAS\_MOUSE sp|Q3UIR3-2|DTX3L\_MOUSE sp|Q3UIR3|DTX3L\_MOUSE sp|Q91YT8|CSCL1\_MOUSE sp|Q6ZQ08-3|CNOT1\_MOUSE sp|G3X9K3|BIG1\_MOUSE sp|Q5K6N0|TM232\_MOUSE sp|Q8C5W0|CLMN\_MOUSE sp|Q5K6N0-3|TM232\_MOUSE sp|Q8C5W0-3|CLMN\_MOUSE tr|G5E8G4|G5E8G4\_MOUSE sp|Q8C5W0-4|CLMN\_MOUSE sp|Q8C5W0-2|CLMN\_MOUSE sp|Q5K6N0-2|TM232\_MOUSE sp|O08710|THYG\_MOUSE sp|Q99NB8|UBQL4\_MOUSE tr|A3KG81|A3KG81\_MOUSE tr|A3KG84|A3KG84\_MOUSE sp|Q684R7|FREM1\_MOUSE sp|Q684R7-2|FREM1\_MOUSE tr|E9QPS7|E9QPS7\_MOUSE sp|Q149L7|CRTAM\_MOUSE sp|Q8BWG4|K1755\_MOUSE sp|Q922U1|PRPF3\_MOUSE sp|Q8BNA6-2|FAT3\_MOUSE sp|Q5SYL1|SG494\_MOUSE tr|F6YAR3|F6YAR3\_MOUSE sp|Q05D44|IF2P\_MOUSE sp|Q63918|SDPR\_MOUSE tr|H7BWY5|H7BWY5\_MOUSE sp|Q8CAS9-2|PARP9\_MOUSE sp|Q8BKR5|PPR37\_MOUSE tr|Q3V3Y6|Q3V3Y6\_MOUSE tr|Q8C0X6|Q8C0X6\_MOUSE tr|Q14BL9|Q14BL9\_MOUSE sp|Q9WV04|KIF9\_MOUSE tr|Q8BUJ6|Q8BUJ6\_MOUSE sp|Q64343|ABCG1\_MOUSE sp|D3Z0R2|PPR36\_MOUSE sp|Q9JIM5|YIPF7\_MOUSE tr|A2AWT7|A2AWT7\_MOUSE sp|Q9CWQ0|DPH5\_MOUSE tr|Q8BG20|Q8BG20\_MOUSE tr|V9GXP1|V9GXP1\_MOUSE sp|Q9Z0Z4-2|HEPH\_MOUSE tr|Q7M766|Q7M766\_MOUSE sp|Q9Z0Z4|HEPH\_MOUSE sp|Q9DCS3|MECR\_MOUSE sp|Q61838|A2M\_MOUSE tr|D3YW52|D3YW52\_MOUSE tr|F6SB18|F6SB18\_MOUSE sp|Q08775-5|RUNX2\_MOUSE sp|Q08775-2|RUNX2\_MOUSE tr|E9PUK7|E9PUK7\_MOUSE sp|Q08775-3|RUNX2\_MOUSE tr|F8WHN7|F8WHN7\_MOUSE tr|E0CY45|E0CY45\_MOUSE sp|Q08775-6|RUNX2\_MOUSE sp|Q08775|RUNX2\_MOUSE tr|E9Q896|E9Q896\_MOUSE tr|E9Q1S5|E9Q1S5\_MOUSE tr|B2RQR8|B2RQR8\_MOUSE sp|Q07409|CNTN3\_MOUSE tr|G5E8P0|G5E8P0\_MOUSE tr|E9PWW9|E9PWW9\_MOUSE tr|E9Q4Y2|E9Q4Y2\_MOUSE tr|E9Q6P4|E9Q6P4\_MOUSE tr|Q3UQW2|Q3UQW2\_MOUSE sp|Q8CC88|VWA8\_MOUSE sp|Q8K449|ABCA9\_MOUSE sp|Q923P0-2|COKA1\_MOUSE sp|Q923P0|COKA1\_MOUSE tr|F6UFI2|F6UFI2\_MOUSE tr|H7BX50|H7BX50\_MOUSE sp|A2AWL7|MGAP\_MOUSE tr|E9QLG3|E9QLG3\_MOUSE tr|A2AL85|A2AL85\_MOUSE sp|Q8BSY0|ASPH\_MOUSE tr|Q8CBM2|Q8CBM2\_MOUSE sp|Q8CGM2|RP1L1\_MOUSE sp|Q3TAA7|S11IP\_MOUSE tr|Q0VGU8|Q0VGU8\_MOUSE tr|D3Z499|D3Z499\_MOUSE sp|Q0PMG2|MDGA1\_MOUSE tr|E9PY95|E9PY95\_MOUSE sp|P59598|ASXL1\_MOUSE tr|K4DI62|K4DI62\_MOUSE sp|P13541|MYH3\_MOUSE sp|Q8C5W4-2|MOR2B\_MOUSE sp|Q8C5W4|MOR2B\_MOUSE sp|Q62059-4|CSPG2\_MOUSE tr|Q8BS97|Q8BS97\_MOUSE tr|E9QMK3|E9QMK3\_MOUSE sp|Q9CSB4|PAR3L\_MOUSE sp|Q9CSB4-2|PAR3L\_MOUSE tr|Q5SV55|Q5SV55\_MOUSE sp|A2AJK6-3|CHD7\_MOUSE sp|A2AJK6|CHD7\_MOUSE tr|H3BJ46|H3BJ46\_MOUSE sp|Q8CAF4|NHSL1\_MOUSE sp|Q8R420|ABCA3\_MOUSE sp|Q8CAF4-3|NHSL1\_MOUSE Q6NT21 P78386 Q14533 sp|Q4FZC9|SYNE3\_MOUSE sp|Q4FZC9-2|SYNE3\_MOUSE sp|Q9JLM8|DCLK1\_MOUSE tr|H7BX36|H7BX36\_MOUSE tr|E9QPU2|E9QPU2\_MOUSE tr|F8WJA5|F8WJA5\_MOUSE tr|F7CB97|F7CB97\_MOUSE sp|P13705|MSH3\_MOUSE sp|Q9Z1R2|BAG6\_MOUSE tr|Q3UF95|Q3UF95\_MOUSE tr|G5E8X1|G5E8X1\_MOUSE tr|E9Q7K5|E9Q7K5\_MOUSE tr|Z4YLD0|Z4YLD0\_MOUSE sp|Q8VDP2-2|CX056\_MOUSE tr|E9PUN5|E9PUN5\_MOUSE sp|Q9Z138|ROR2\_MOUSE sp|Q91YY5|SO1A5\_MOUSE tr|E9Q9M0|E9Q9M0\_MOUSE sp|Q6A078|CE290\_MOUSE tr|A2AEP5|A2AEP5\_MOUSE sp|Q6ZPU9|KBP\_MOUSE sp|Q6ZPU9-3|KBP\_MOUSE tr|F6VUT6|F6VUT6\_MOUSE sp|Q5I1X5|IASPP\_MOUSE tr|Q91XY8|Q91XY8\_MOUSE tr|E9QA57|E9QA57\_MOUSE sp|Q8CI95|OSB11\_MOUSE tr|G5E8A0|G5E8A0\_MOUSE tr|E9Q7E2|E9Q7E2\_MOUSE tr|Q14C10|Q14C10\_MOUSE sp|Q8K3A9|MEPCE\_MOUSE tr|E9Q6K7|E9Q6K7\_MOUSE sp|Q6PHQ8|NAA35\_MOUSE tr|A2CG33|A2CG33\_MOUSE sp|Q9Z0E2|CHRD\_MOUSE sp|Q61696|HS71A\_MOUSE tr|E9Q9G6|E9Q9G6\_MOUSE tr|G3UW47|G3UW47\_MOUSE sp|P17879|HS71B\_MOUSE sp|P33173|KIF1A\_MOUSE tr|E9QAN4|E9QAN4\_MOUSE sp|Q8CGF1-2|RHG29\_MOUSE tr|E9QQ17|E9QQ17\_MOUSE sp|Q8C561|LMBD2\_MOUSE sp|Q921U8|SMTN\_MOUSE tr|D3Z3Q3|D3Z3Q3\_MOUSE sp|Q921U8-2|SMTN\_MOUSE tr|A0A087WSA4|A0A087WSA4\_MOUSE tr|F7DC05|F7DC05\_MOUSE sp|Q99M80-2|PTPRT\_MOUSE sp|Q6A065|CE170\_MOUSE sp|Q8BYF6|SC5A8\_MOUSE sp|Q9EPL2-2|CSTN1\_MOUSE tr|Q4V784|Q4V784\_MOUSE sp|Q9EPL2|CSTN1\_MOUSE sp|P54754|EPHB3\_MOUSE sp|Q5XJY4|PARL\_MOUSE tr|D3Z5R2|D3Z5R2\_MOUSE sp|P33267|CP2F2\_MOUSE tr|F6UCX4|F6UCX4\_MOUSE tr|D3YUT2|D3YUT2\_MOUSE sp|Q6A0A2-2|LAR4B\_MOUSE sp|Q6A0A2|LAR4B\_MOUSE sp|Q924C5|ALPK3\_MOUSE sp|Q3TMW1|C102A\_MOUSE sp|O89026|ROBO1\_MOUSE tr|G5E843|G5E843\_MOUSE sp|P19467|MUC13\_MOUSE sp|Q8K284-2|TF3C1\_MOUSE sp|Q6PGL7-2|FAM21\_MOUSE sp|Q8BXL9-3|IFFO1\_MOUSE sp|Q924C1|XPO5\_MOUSE sp|Q9ESN6|TRIM2\_MOUSE tr|E9Q6J5|E9Q6J5\_MOUSE sp|P70399-2|TP53B\_MOUSE tr|F6R059|F6R059\_MOUSE tr|J3QN01|J3QN01\_MOUSE sp|Q9D7Q1-2|CHIT1\_MOUSE sp|Q9D7Q1|CHIT1\_MOUSE tr|D3YWE3|D3YWE3\_MOUSE sp|A2AVA0|SVEP1\_MOUSE tr|A0A087WQC4|A0A087WQC4\_MOUSE tr|H3BJR2|H3BJR2\_MOUSE sp|Q8R2K4-2|TAF6L\_MOUSE tr|L7N209|L7N209\_MOUSE tr|J3KMK0|J3KMK0\_MOUSE sp|Q61982|NOTC3\_MOUSE sp|P30416|FKBP4\_MOUSE sp|Q8BQH6|CDCP2\_MOUSE sp|P50296|ARY3\_MOUSE tr|Q5SVP0|Q5SVP0\_MOUSE sp|Q7TQF2|FBX10\_MOUSE tr|Q14DL2|Q14DL2\_MOUSE tr|E0CXJ0|E0CXJ0\_MOUSE sp|Q8VDD5|MYH9\_MOUSE tr|Q91XY5|Q91XY5\_MOUSE tr|A0A087WRN1|A0A087WRN1\_MOUSE tr|F8WI22|F8WI22\_MOUSE tr|B1AYL1|B1AYL1\_MOUSE sp|Q76KF0-5|SEM6D\_MOUSE sp|Q76KF0-2|SEM6D\_MOUSE sp|Q5DW34-2|EHMT1\_MOUSE sp|Q5DW34|EHMT1\_MOUSE tr|E9Q6G0|E9Q6G0\_MOUSE sp|Q5DW34-3|EHMT1\_MOUSE tr|Z4YJZ7|Z4YJZ7\_MOUSE tr|E9Q1F2|E9Q1F2\_MOUSE tr|D3YVH4|D3YVH4\_MOUSE sp|Q9QXP7|C1QT1\_MOUSE tr|B7ZCC2|B7ZCC2\_MOUSE tr|A2A845|A2A845\_MOUSE sp|Q9DA44|TSACC\_MOUSE sp|Q8R570|SNP47\_MOUSE sp|Q9CUU3|SYCP2\_MOUSE sp|Q924Z6-2|XPO6\_MOUSE sp|Q924Z6|XPO6\_MOUSE tr|Q8VFJ5|Q8VFJ5\_MOUSE sp|Q7JJ13|BRD2\_MOUSE sp|Q7JJ13-2|BRD2\_MOUSE tr|E9Q413|E9Q413\_MOUSE tr|E9Q6S8|E9Q6S8\_MOUSE tr|Q5SVF8|Q5SVF8\_MOUSE sp|Q9QXD6|F16P1\_MOUSE sp|P61979|HNRPK\_MOUSE tr|H3BLL4|H3BLL4\_MOUSE tr|H3BLP7|H3BLP7\_MOUSE tr|A2ARK0|A2ARK0\_MOUSE sp|P61979-3|HNRPK\_MOUSE tr|H3BK96|H3BK96\_MOUSE sp|P61979-2|HNRPK\_MOUSE tr|D3Z5X4|D3Z5X4\_MOUSE tr|D3YWG1|D3YWG1\_MOUSE tr|H3BKD0|H3BKD0\_MOUSE tr|B2M1R6|B2M1R6\_MOUSE tr|E9QK39|E9QK39\_MOUSE tr|A2CEE7|A2CEE7\_MOUSE tr|A2CEE6|A2CEE6\_MOUSE tr|E9PUP0|E9PUP0\_MOUSE sp|Q8BKC8-2|PI4KB\_MOUSE tr|D3YWC9|D3YWC9\_MOUSE sp|Q5ND52|MRM3\_MOUSE sp|Q6P9S0|MTSSL\_MOUSE sp|Q5DTW7-3|K1551\_MOUSE sp|Q5DTW7|K1551\_MOUSE tr|A0A087WP74|A0A087WP74\_MOUSE sp|Q8C4X1-4|CENPX\_MOUSE sp|Q9D8C3|ALG13\_MOUSE tr|E9PX10|E9PX10\_MOUSE sp|Q8C196|CPSM\_MOUSE tr|Q3U1U6|Q3U1U6\_MOUSE sp|O54714-3|PIAS3\_MOUSE sp|O54714|PIAS3\_MOUSE sp|Q9CR68|UCRI\_MOUSE tr|Q8K2D8|Q8K2D8\_MOUSE sp|Q9JI19|FIBP\_MOUSE sp|Q9D4H4|AMOL1\_MOUSE sp|Q9D4H4-2|AMOL1\_MOUSE tr|B1ATY1|B1ATY1\_MOUSE sp|Q8K202|RPA49\_MOUSE tr|E9Q793|E9Q793\_MOUSE sp|Q80VA5|S3TC2\_MOUSE tr|F6XLD9|F6XLD9\_MOUSE sp|Q9JK30-2|ORC3\_MOUSE sp|Q9JK30|ORC3\_MOUSE tr|S4R1S2|S4R1S2\_MOUSE tr|D4AFX7|D4AFX7\_MOUSE tr|G3X922|G3X922\_MOUSE sp|Q68FE8|Z280D\_MOUSE tr|V9GWW1|V9GWW1\_MOUSE sp|P30276|CCNB2\_MOUSE sp|Q8BR92|PALM2\_MOUSE sp|Q60707|TBX2\_MOUSE sp|Q8BLG0|PHF20\_MOUSE sp|Q8K124|PKHO2\_MOUSE tr|E9QA61|E9QA61\_MOUSE tr|E9Q5W5|E9Q5W5\_MOUSE tr|G3UX13|G3UX13\_MOUSE sp|P49194|RET3\_MOUSE sp|Q9D4F8|GCP4\_MOUSE tr|Q8BYN2|Q8BYN2\_MOUSE sp|Q9WV30-4|NFAT5\_MOUSE sp|Q7TSC1|PRC2A\_MOUSE tr|G3UX48|G3UX48\_MOUSE tr|D3YXM8|D3YXM8\_MOUSE sp|Q66L42|M3K10\_MOUSE sp|P28740-2|KIF2A\_MOUSE tr|E0CZ72|E0CZ72\_MOUSE sp|Q9QXC1|FETUB\_MOUSE sp|P28740|KIF2A\_MOUSE tr|F8VQ42|F8VQ42\_MOUSE sp|P28740-1|KIF2A\_MOUSE tr|S4R2R6|S4R2R6\_MOUSE sp|Q8CD54-3|PIEZ2\_MOUSE sp|Q8CCJ9-1|P20L1\_MOUSE sp|Q8K4Q0-2|RPTOR\_MOUSE sp|Q3UTJ2-2|SRBS2\_MOUSE tr|Z4YJR7|Z4YJR7\_MOUSE sp|Q7TNU6|ZN250\_MOUSE sp|Q8BW86|ARG33\_MOUSE sp|Q9ET77|JPH3\_MOUSE tr|E9QK62|E9QK62\_MOUSE sp|Q8CHT1|NGEF\_MOUSE sp|Q8R317-2|UBQL1\_MOUSE tr|E9Q2V5|E9Q2V5\_MOUSE sp|Q3UTZ3|CG043\_MOUSE sp|P42227|STAT3\_MOUSE sp|P42227-3|STAT3\_MOUSE tr|K3W4P2|K3W4P2\_MOUSE tr|B7ZC18|B7ZC18\_MOUSE sp|Q8R0S4|CACB4\_MOUSE sp|Q8R0S4-1|CACB4\_MOUSE tr|J3QK20|J3QK20\_MOUSE sp|Q8R0S4-2|CACB4\_MOUSE sp|Q7TNB8-3|SBNO2\_MOUSE tr|A3KG83|A3KG83\_MOUSE tr|F8VPT3|F8VPT3\_MOUSE sp|Q9R1C7-2|PR40A\_MOUSE sp|P17426|AP2A1\_MOUSE sp|Q8BL99-2|DOP1\_MOUSE tr|L7N265|L7N265\_MOUSE tr|D3Z452|D3Z452\_MOUSE tr|Q80W76|Q80W76\_MOUSE sp|P35123|UBP4\_MOUSE tr|E9PYJ0|E9PYJ0\_MOUSE tr|E9Q2X0|E9Q2X0\_MOUSE sp|O88492|PLIN4\_MOUSE tr|B1AR09|B1AR09\_MOUSE tr|B1AR10|B1AR10\_MOUSE sp|Q9D478-3|ODF2L\_MOUSE sp|Q9D478|ODF2L\_MOUSE sp|Q9D478-2|ODF2L\_MOUSE tr|E9Q4X5|E9Q4X5\_MOUSE sp|Q91XL9-2|OSBL1\_MOUSE tr|Q3V156|Q3V156\_MOUSE sp|Q91XL9-3|OSBL1\_MOUSE tr|E9PUK3|E9PUK3\_MOUSE sp|Q8K012|FBP1L\_MOUSE tr|E9PUI5|E9PUI5\_MOUSE sp|Q8K012-2|FBP1L\_MOUSE sp|Q61749-2|EI2BD\_MOUSE sp|Q9ET78|JPH2\_MOUSE tr|G3UZN4|G3UZN4\_MOUSE sp|Q69Z98-3|BRSK2\_MOUSE sp|Q8K4J6|MKL1\_MOUSE tr|A2BE32|A2BE32\_MOUSE tr|D3YUI2|D3YUI2\_MOUSE sp|Q8K4J6-2|MKL1\_MOUSE tr|D3Z3M3|D3Z3M3\_MOUSE tr|D3YUG5|D3YUG5\_MOUSE sp|Q80TT8|CUL9\_MOUSE tr|D3YWX8|D3YWX8\_MOUSE sp|Q9R0X4|ACOT9\_MOUSE tr|E9PUG7|E9PUG7\_MOUSE sp|Q61211-2|EIF2D\_MOUSE sp|Q7TNG5-2|EMAL2\_MOUSE sp|Q7TNG5|EMAL2\_MOUSE sp|Q61211|EIF2D\_MOUSE tr|E9QK48|E9QK48\_MOUSE tr|F7AL76|F7AL76\_MOUSE sp|Q69ZT9|TBC30\_MOUSE sp|Q9JJY4|DDX20\_MOUSE sp|Q9Z0J4-2|NOS1\_MOUSE sp|Q91WG2-2|RABE2\_MOUSE tr|A2AS98|A2AS98\_MOUSE sp|P28660-2|NCKP1\_MOUSE sp|P28660|NCKP1\_MOUSE sp|Q921L5|COG2\_MOUSE sp|P35922-8|FMR1\_MOUSE sp|Q9CTG6|AT132\_MOUSE tr|E9Q2A4|E9Q2A4\_MOUSE sp|P35922-2|FMR1\_MOUSE sp|P70207|PLXA2\_MOUSE sp|Q8BII1|PROX2\_MOUSE tr|E9Q4Y4|E9Q4Y4\_MOUSE tr|F6U329|F6U329\_MOUSE sp|Q9WTU3-3|SCN8A\_MOUSE sp|Q9WTU3-4|SCN8A\_MOUSE sp|Q9WTU3-5|SCN8A\_MOUSE tr|E9Q3Y4|E9Q3Y4\_MOUSE sp|Q9ESE1|LRBA\_MOUSE sp|Q9ESE1-3|LRBA\_MOUSE sp|Q9ESE1-2|LRBA\_MOUSE sp|Q9WTU3|SCN8A\_MOUSE tr|F7D6H8|F7D6H8\_MOUSE tr|F7D6J5|F7D6J5\_MOUSE sp|Q8C0G2|T3JAM\_MOUSE tr|G3X949|G3X949\_MOUSE tr|E9Q0A7|E9Q0A7\_MOUSE sp|B1AVY7|KI16B\_MOUSE sp|B1AUC7|RHG36\_MOUSE tr|Z4YJK0|Z4YJK0\_MOUSE sp|B1AUC7-2|RHG36\_MOUSE sp|B1AUC7-3|RHG36\_MOUSE sp|Q0V8T9|CTP5A\_MOUSE tr|M0QWC0|M0QWC0\_MOUSE tr|E9QQ97|E9QQ97\_MOUSE sp|Q9EPQ2-2|RPGR1\_MOUSE tr|F6QXA3|F6QXA3\_MOUSE tr|F7A4H5|F7A4H5\_MOUSE sp|P25206|MCM3\_MOUSE tr|E9Q1C1|E9Q1C1\_MOUSE sp|Q62210|BIRC2\_MOUSE sp|Q07563|COHA1\_MOUSE tr|S4R165|S4R165\_MOUSE tr|S4R2J6|S4R2J6\_MOUSE tr|D3Z4U2|D3Z4U2\_MOUSE tr|A2AC60|A2AC60\_MOUSE tr|Q91VE5|Q91VE5\_MOUSE sp|Q8CCB4-2|VPS53\_MOUSE sp|Q3UGY8|BIG3\_MOUSE tr|A0A087WNV3|A0A087WNV3\_MOUSE tr|A2CEE5|A2CEE5\_MOUSE sp|B2RXS4|PLXB2\_MOUSE sp|B2RS91|RRN3\_MOUSE sp|Q62504-3|MINT\_MOUSE sp|Q62504-2|MINT\_MOUSE tr|A2ADB0|A2ADB0\_MOUSE sp|Q62504|MINT\_MOUSE tr|A2ADB1|A2ADB1\_MOUSE sp|Q9EP53-4|TSC1\_MOUSE sp|Q6R0H7-3|GNAS1\_MOUSE sp|Q6R0H7-4|GNAS1\_MOUSE sp|Q6R0H7|GNAS1\_MOUSE tr|G3UW56|G3UW56\_MOUSE tr|Q80XB7|Q80XB7\_MOUSE sp|O88199|CHST3\_MOUSE tr|E9QPK1|E9QPK1\_MOUSE sp|Q8R1U2|CGRE1\_MOUSE tr|G5E8X5|G5E8X5\_MOUSE sp|Q62217|SEM5A\_MOUSE sp|Q8R4U0|STAB2\_MOUSE sp|Q9ESD7-2|DYSF\_MOUSE sp|Q9ESD7|DYSF\_MOUSE tr|E9QL12|E9QL12\_MOUSE sp|Q9ESD7-3|DYSF\_MOUSE sp|O88307|SORL\_MOUSE tr|E9Q423|E9Q423\_MOUSE tr|E9PXU9|E9PXU9\_MOUSE sp|Q9ET54-4|PALLD\_MOUSE sp|Q3UPH1|PRRC1\_MOUSE sp|Q80XQ2|TBCD5\_MOUSE tr|S4R2I9|S4R2I9\_MOUSE tr|F6UKN5|F6UKN5\_MOUSE sp|A6H619|PHRF1\_MOUSE sp|A6H619-2|PHRF1\_MOUSE tr|E9Q3V6|E9Q3V6\_MOUSE sp|Q9CQS6|GKN2\_MOUSE sp|Q3U133|ZN746\_MOUSE sp|Q8BKI2|TNR6B\_MOUSE tr|F6RDL4|F6RDL4\_MOUSE sp|Q0VBN2|DSEL\_MOUSE tr|E9Q2L7|E9Q2L7\_MOUSE tr|B2KG08|B2KG08\_MOUSE sp|A2A7Q9|RN19B\_MOUSE sp|Q8C4A5|ASXL3\_MOUSE sp|Q8C4A5-2|ASXL3\_MOUSE sp|Q9EQN3|T22D4\_MOUSE sp|A2A5K6|ZN335\_MOUSE sp|Q61941|NNTM\_MOUSE sp|Q5H8C4-2|VP13A\_MOUSE sp|Q5H8C4|VP13A\_MOUSE tr|B1AWM0|B1AWM0\_MOUSE sp|Q9QZ05|E2AK4\_MOUSE tr|E9Q6J7|E9Q6J7\_MOUSE tr|Q810J1|Q810J1\_MOUSE sp|O35955|PSB10\_MOUSE tr|F6YNY8|F6YNY8\_MOUSE tr|E9PWH2|E9PWH2\_MOUSE tr|A2A468|A2A468\_MOUSE tr|Q05BA5|Q05BA5\_MOUSE sp|O09000|NCOA3\_MOUSE P01030 ENSEMBL:ENSBTAP00000007350 tr|E9Q512|E9Q512\_MOUSE tr|H3BL88|H3BL88\_MOUSE tr|H3BKP8|H3BKP8\_MOUSE sp|Q8C033-2|ARHGA\_MOUSE sp|Q3U3T8-3|WDR62\_MOUSE tr|D3Z7R9|D3Z7R9\_MOUSE sp|Q640L3|CCPG1\_MOUSE sp|Q640L3-2|CCPG1\_MOUSE sp|Q640L3-3|CCPG1\_MOUSE sp|Q9D2F7|P210L\_MOUSE tr|E9PWF0|E9PWF0\_MOUSE sp|Q05895|TSP3\_MOUSE sp|Q99JH7|CSTN3\_MOUSE sp|Q9Z1X4-2|ILF3\_MOUSE sp|Q9Z1X4|ILF3\_MOUSE tr|Q45VK5|Q45VK5\_MOUSE sp|Q9Z1X4-3|ILF3\_MOUSE sp|A2A884|ZEP3\_MOUSE tr|Q3U1C4|Q3U1C4\_MOUSE sp|E2JF22|PIEZ1\_MOUSE tr|E9PUQ9|E9PUQ9\_MOUSE sp|O08784|TCOF\_MOUSE sp|P25976-2|UBF1\_MOUSE sp|P25976|UBF1\_MOUSE tr|H3BL37|H3BL37\_MOUSE tr|A2AWT6|A2AWT6\_MOUSE tr|A2AWT5|A2AWT5\_MOUSE tr|H3BKL1|H3BKL1\_MOUSE tr|H3BLC8|H3BLC8\_MOUSE tr|H3BJH1|H3BJH1\_MOUSE sp|Q99JY1|TIRAP\_MOUSE sp|Q3UJC8-2|CE034\_MOUSE tr|S4R1K6|S4R1K6\_MOUSE sp|Q3UJC8|CE034\_MOUSE sp|Q07105|GDF9\_MOUSE sp|Q60610|TIAM1\_MOUSE tr|G3UWG2|G3UWG2\_MOUSE sp|Q61493|DPOLZ\_MOUSE sp|A2CG49-5|KALRN\_MOUSE sp|Q9CU65|ZMYM2\_MOUSE sp|A2CG49-9|KALRN\_MOUSE tr|D3Z560|D3Z560\_MOUSE tr|D3Z559|D3Z559\_MOUSE tr|B1B1A7|B1B1A7\_MOUSE sp|A2CG49-8|KALRN\_MOUSE sp|Q9QX29|TRPC5\_MOUSE sp|C6KI89|CTSG2\_MOUSE tr|D3Z2H0|D3Z2H0\_MOUSE tr|A2AW15|A2AW15\_MOUSE sp|Q3UVR3|TTBK2\_MOUSE tr|A2ATY4|A2ATY4\_MOUSE tr|B1ATC3|B1ATC3\_MOUSE tr|Z4YLB7|Z4YLB7\_MOUSE sp|P97300-1|NPTN\_MOUSE sp|P97300-3|NPTN\_MOUSE sp|Q8K4I3|ARHG6\_MOUSE tr|F6WMJ3|F6WMJ3\_MOUSE tr|A2AFJ8|A2AFJ8\_MOUSE sp|Q69ZA1|CDK13\_MOUSE tr|B9EHK9|B9EHK9\_MOUSE tr|A2AQ81|A2AQ81\_MOUSE sp|Q9QY80|HACD1\_MOUSE tr|E0CXV8|E0CXV8\_MOUSE sp|Q64028-3|PHC1\_MOUSE tr|Q3V116|Q3V116\_MOUSE sp|Q64028|PHC1\_MOUSE tr|Q7TT35|Q7TT35\_MOUSE sp|Q64028-2|PHC1\_MOUSE tr|F2Z3U3|F2Z3U3\_MOUSE tr|F6ZZL0|F6ZZL0\_MOUSE sp|Q91ZB0|ALPK2\_MOUSE sp|A6PWV5|ARI3C\_MOUSE tr|B7ZP08|B7ZP08\_MOUSE sp|O88207|CO5A1\_MOUSE sp|O35492|CLK3\_MOUSE tr|Q3TTL3|Q3TTL3\_MOUSE sp|Q80VP2|SPAT7\_MOUSE sp|Q67FY2|BCL9L\_MOUSE sp|Q67FY2-2|BCL9L\_MOUSE sp|Q9DC04-1|RGS3\_MOUSE sp|Q9DC04|RGS3\_MOUSE sp|Q9DC04-4|RGS3\_MOUSE sp|P97477-2|AURKA\_MOUSE sp|P97477|AURKA\_MOUSE tr|E9PYB0|E9PYB0\_MOUSE sp|Q80U35-2|ARHGH\_MOUSE sp|Q8R4K8|PAPP1\_MOUSE sp|Q8BKC8|PI4KB\_MOUSE tr|E9Q8A3|E9Q8A3\_MOUSE sp|Q8R4K8-2|PAPP1\_MOUSE sp|Q80TK0|K1107\_MOUSE tr|E9QLA8|E9QLA8\_MOUSE sp|Q8R2Q4-3|RRF2M\_MOUSE sp|Q8R2Q4|RRF2M\_MOUSE sp|Q8R2Q4-2|RRF2M\_MOUSE tr|E9Q7N5|E9Q7N5\_MOUSE sp|Q04690-3|NF1\_MOUSE sp|Q04690-4|NF1\_MOUSE sp|Q3U095|NXPE2\_MOUSE sp|Q9ER47|KCNH7\_MOUSE sp|P56203|CATW\_MOUSE sp|Q6U7R4|TIMD4\_MOUSE sp|Q3UZY0-3|SFI1\_MOUSE tr|Q5XJV5|Q5XJV5\_MOUSE sp|P56546-2|CTBP2\_MOUSE sp|A2A863-3|ITB4\_MOUSE tr|A2A864|A2A864\_MOUSE sp|Q5XPT3-2|LARG2\_MOUSE sp|Q5XPT3|LARG2\_MOUSE tr|Q14AT0|Q14AT0\_MOUSE tr|A2AHG6|A2AHG6\_MOUSE sp|Q8CHC4|SYNJ1\_MOUSE tr|D3Z656|D3Z656\_MOUSE sp|Q7SIG6-2|ASAP2\_MOUSE sp|Q68FH0-2|PKP4\_MOUSE tr|A2AS45|A2AS45\_MOUSE tr|Q9D3V8|Q9D3V8\_MOUSE tr|A0A087WRJ7|A0A087WRJ7\_MOUSE sp|Q8BPY9|FIGL1\_MOUSE tr|A8Y5K6|A8Y5K6\_MOUSE tr|A2AIT6|A2AIT6\_MOUSE sp|Q8BWP5|TTPA\_MOUSE tr|A8Y5N9|A8Y5N9\_MOUSE sp|P51656|DHB1\_MOUSE sp|Q3UHB8|CC177\_MOUSE tr|F6RXM1|F6RXM1\_MOUSE tr|Q6GQX8|Q6GQX8\_MOUSE sp|O55070|DNSL3\_MOUSE tr|Q68FG1|Q68FG1\_MOUSE tr|F8WGM5|F8WGM5\_MOUSE sp|Q64324|STXB2\_MOUSE tr|E9Q0J5|E9Q0J5\_MOUSE sp|Q9QXL2-4|KI21A\_MOUSE tr|F8WGN6|F8WGN6\_MOUSE sp|Q9QXL2-2|KI21A\_MOUSE sp|Q9QXL2|KI21A\_MOUSE sp|Q4ACU6-2|SHAN3\_MOUSE sp|Q9QXL2-3|KI21A\_MOUSE sp|Q6A070-2|F179B\_MOUSE sp|Q6A070|F179B\_MOUSE sp|Q6NSW3-4|SPKAP\_MOUSE sp|Q6NSW3-3|SPKAP\_MOUSE sp|Q6NSW3|SPKAP\_MOUSE sp|Q8BSS9|LIPA2\_MOUSE sp|O89084-2|PDE4A\_MOUSE tr|B8QI34|B8QI34\_MOUSE tr|D3Z2H7|D3Z2H7\_MOUSE sp|P26039|TLN1\_MOUSE sp|Q9D067-3|MDM1\_MOUSE tr|F7BAB2|F7BAB2\_MOUSE tr|E9Q161|E9Q161\_MOUSE sp|Q8K2I1|FNTB\_MOUSE sp|Q3TYG6|F179A\_MOUSE tr|F6YU21|F6YU21\_MOUSE tr|E9PV86|E9PV86\_MOUSE tr|D3Z2D1|D3Z2D1\_MOUSE tr|D3YZP3|D3YZP3\_MOUSE sp|P0C192|LRC4B\_MOUSE sp|Q9DBP0|NPT2B\_MOUSE tr|Q3TDL4|Q3TDL4\_MOUSE tr|G3UZT6|G3UZT6\_MOUSE tr|G3UYQ2|G3UYQ2\_MOUSE tr|Q3UDC3|Q3UDC3\_MOUSE sp|O88746|TOM1\_MOUSE tr|E9Q2X6|E9Q2X6\_MOUSE sp|Q9WVH4|FOXO3\_MOUSE sp|Q9QYC0|ADDA\_MOUSE sp|Q3TTY0|PLB1\_MOUSE sp|Q8C0R0-2|UBP37\_MOUSE sp|Q8C963-2|CC159\_MOUSE sp|Q8C963|CC159\_MOUSE sp|Q8C0R0|UBP37\_MOUSE sp|Q5F204|MDH1B\_MOUSE sp|Q62009-4|POSTN\_MOUSE sp|Q62009-2|POSTN\_MOUSE sp|Q62009-5|POSTN\_MOUSE sp|Q62009|POSTN\_MOUSE sp|Q62009-3|POSTN\_MOUSE sp|Q9QXA6|BAT1\_MOUSE sp|Q8BLQ7|CTR4\_MOUSE sp|P63239|NEC1\_MOUSE sp|A2ACJ2|FP100\_MOUSE sp|P97305-2|NFAC3\_MOUSE tr|E9Q4K7|E9Q4K7\_MOUSE sp|Q60675|LAMA2\_MOUSE tr|E9Q5B2|E9Q5B2\_MOUSE sp|Q9JL25|RGS1\_MOUSE sp|Q9JL25-2|RGS1\_MOUSE tr|E9Q682|E9Q682\_MOUSE tr|E9PYD3|E9PYD3\_MOUSE sp|Q811U4|MFN1\_MOUSE sp|C3VPR6-2|NLRC5\_MOUSE tr|Q6AXE3|Q6AXE3\_MOUSE sp|Q5DQR4-4|STB5L\_MOUSE sp|Q5DQR4|STB5L\_MOUSE sp|Q5DQR4-5|STB5L\_MOUSE sp|Q5DQR4-2|STB5L\_MOUSE sp|Q5DQR4-3|STB5L\_MOUSE sp|Q61602|GLI3\_MOUSE sp|O35594|IFT81\_MOUSE sp|Q99MQ3|PINK1\_MOUSE tr|E9Q7P9|E9Q7P9\_MOUSE tr|A2AM78|A2AM78\_MOUSE sp|Q80TM6|R3HD2\_MOUSE tr|Q148X5|Q148X5\_MOUSE sp|Q8CGK5|INLR1\_MOUSE sp|Q8BH05|ZN750\_MOUSE sp|A2AGT5-3|CKAP5\_MOUSE sp|A2AGT5|CKAP5\_MOUSE tr|K3W4R5|K3W4R5\_MOUSE tr|H3BKE5|H3BKE5\_MOUSE sp|Q99J72-2|ABEC3\_MOUSE sp|Q8K114|INT9\_MOUSE tr|H3BJ44|H3BJ44\_MOUSE sp|Q99J72-4|ABEC3\_MOUSE tr|H3BJL0|H3BJL0\_MOUSE sp|F8VPU2|FARP1\_MOUSE tr|H3BJQ3|H3BJQ3\_MOUSE sp|Q99J72|ABEC3\_MOUSE tr|E9QMH1|E9QMH1\_MOUSE sp|Q8K3H5|MYO3A\_MOUSE tr|Q3TI69|Q3TI69\_MOUSE sp|Q99J72-3|ABEC3\_MOUSE Q1A7A4 tr|F6UUZ3|F6UUZ3\_MOUSE tr|E9Q7N4|E9Q7N4\_MOUSE sp|P59240|NPHP4\_MOUSE sp|O88653|LTOR3\_MOUSE tr|Q810R8|Q810R8\_MOUSE sp|O55125|NIPS1\_MOUSE sp|Q9ES18|FOXJ2\_MOUSE sp|O08644|EPHB6\_MOUSE tr|B9EKP8|B9EKP8\_MOUSE sp|Q91W50|CSDE1\_MOUSE tr|E9Q0B0|E9Q0B0\_MOUSE sp|Q3UTJ2|SRBS2\_MOUSE tr|B7ZWM6|B7ZWM6\_MOUSE tr|Q5NC84|Q5NC84\_MOUSE sp|Q62522|ZPBP1\_MOUSE sp|Q8C854-3|MYEF2\_MOUSE tr|G8JL68|G8JL68\_MOUSE tr|D6RI15|D6RI15\_MOUSE tr|Q8VEU4|Q8VEU4\_MOUSE sp|Q9JLN9|MTOR\_MOUSE tr|F8WHU7|F8WHU7\_MOUSE tr|E9PW15|E9PW15\_MOUSE sp|Q80U70|SUZ12\_MOUSE sp|Q61851-2|FGFR3\_MOUSE tr|Q61563|Q61563\_MOUSE tr|E9QNJ9|E9QNJ9\_MOUSE tr|F6TK14|F6TK14\_MOUSE tr|Q7TSI8|Q7TSI8\_MOUSE sp|Q61851|FGFR3\_MOUSE sp|Q8BIV7|S45A1\_MOUSE tr|F6WZB3|F6WZB3\_MOUSE tr|B0QZL3|B0QZL3\_MOUSE tr|Z4YKQ9|Z4YKQ9\_MOUSE sp|Q4QY64|ATAD5\_MOUSE sp|Q91ZP9|NECA2\_MOUSE tr|A0A075B6D7|A0A075B6D7\_MOUSE sp|P59644|PI5PA\_MOUSE tr|A2AKM6|A2AKM6\_MOUSE tr|F7ADQ2|F7ADQ2\_MOUSE tr|E9PVB8|E9PVB8\_MOUSE sp|Q8BY35|FGD2\_MOUSE tr|E9Q6H0|E9Q6H0\_MOUSE tr|A2AI52|A2AI52\_MOUSE tr|Q8BXH3|Q8BXH3\_MOUSE sp|Q61282|PGCA\_MOUSE tr|G3UXW8|G3UXW8\_MOUSE tr|B1AUX5|B1AUX5\_MOUSE tr|Q3TE27|Q3TE27\_MOUSE tr|B1AUY3|B1AUY3\_MOUSE tr|E9Q0P9|E9Q0P9\_MOUSE tr|E9Q3Y1|E9Q3Y1\_MOUSE tr|E9Q108|E9Q108\_MOUSE tr|E9PZQ9|E9PZQ9\_MOUSE tr|E9Q6X2|E9Q6X2\_MOUSE tr|E9PYY0|E9PYY0\_MOUSE tr|Q0GE24|Q0GE24\_MOUSE tr|E9Q4R2|E9Q4R2\_MOUSE sp|Q7TMA2|ZN503\_MOUSE tr|F8WIV2|F8WIV2\_MOUSE sp|Q6XP49|GLIS3\_MOUSE sp|Q60854|SPB6\_MOUSE tr|G3UVW5|G3UVW5\_MOUSE tr|K7E6F1|K7E6F1\_MOUSE tr|E9PZJ4|E9PZJ4\_MOUSE tr|F6XZS9|F6XZS9\_MOUSE sp|Q8BFW9|GTR12\_MOUSE sp|Q3UMB9|WASH7\_MOUSE tr|K7N712|K7N712\_MOUSE sp|Q99PT3|IN80B\_MOUSE sp|Q9DA73|CCD89\_MOUSE sp|Q9Z126|PLF4\_MOUSE sp|Q8BI06|CEMIP\_MOUSE sp|Q80U63|MFN2\_MOUSE tr|D6RGU3|D6RGU3\_MOUSE sp|Q91ZR5|CTSR1\_MOUSE tr|G5E8P2|G5E8P2\_MOUSE sp|P97433|ARG28\_MOUSE sp|O55196|ENAM\_MOUSE tr|G3X8T3|G3X8T3\_MOUSE sp|Q3V3Q4|PYDC3\_MOUSE sp|P24622-2|CRYAA\_MOUSE tr|D3YVM9|D3YVM9\_MOUSE sp|Q8BPI1|KTU\_MOUSE sp|Q8BPI1-2|KTU\_MOUSE tr|Q80W26|Q80W26\_MOUSE tr|E9QLR9|E9QLR9\_MOUSE sp|P97479|MYO7A\_MOUSE sp|P97479-2|MYO7A\_MOUSE tr|Q5MJ56|Q5MJ56\_MOUSE sp|O88689-3|PCDA4\_MOUSE sp|O88575|S620B\_MOUSE tr|S4R1L3|S4R1L3\_MOUSE tr|G5E8I3|G5E8I3\_MOUSE tr|A2RST7|A2RST7\_MOUSE sp|Q8C5H1|ANO4\_MOUSE tr|F6U2C2|F6U2C2\_MOUSE sp|P18469|HB2J\_MOUSE sp|P06342|HB2Q\_MOUSE tr|O78196|O78196\_MOUSE sp|P04231|HB23\_MOUSE sp|P01921|HB2D\_MOUSE sp|P18468|HB2I\_MOUSE sp|P01915|HB22\_MOUSE sp|Q6P5D3-3|DHX57\_MOUSE sp|P20040|HB24\_MOUSE tr|F8WIM6|F8WIM6\_MOUSE sp|Q80T03|MUC6\_MOUSE sp|Q80TM6-4|R3HD2\_MOUSE tr|E9Q9D6|E9Q9D6\_MOUSE tr|F6YZU5|F6YZU5\_MOUSE sp|Q8VHD8|HORN\_MOUSE tr|E9Q9I2|E9Q9I2\_MOUSE tr|E9Q9R9|E9Q9R9\_MOUSE tr|A2AQ51|A2AQ51\_MOUSE sp|P55014|S12A1\_MOUSE sp|P55014-4|S12A1\_MOUSE tr|A2AQ50|A2AQ50\_MOUSE sp|Q8CGF6|WDR47\_MOUSE tr|E9QP10|E9QP10\_MOUSE sp|Q9JM99-2|PRG4\_MOUSE sp|Q80TM9|NISCH\_MOUSE sp|Q80TM9-2|NISCH\_MOUSE sp|A2ALI5|AJAP1\_MOUSE sp|Q922D4-4|PP6R3\_MOUSE sp|Q922D4-3|PP6R3\_MOUSE tr|B7ZMZ7|B7ZMZ7\_MOUSE sp|B2RRE7|OTUD4\_MOUSE sp|Q3UTJ2-5|SRBS2\_MOUSE tr|E9PX79|E9PX79\_MOUSE tr|D3Z080|D3Z080\_MOUSE sp|Q6WKZ8-3|UBR2\_MOUSE sp|Q8CE72|CE042\_MOUSE tr|A2AUM0|A2AUM0\_MOUSE tr|F6WM66|F6WM66\_MOUSE tr|Q9JKY7|Q9JKY7\_MOUSE sp|Q8CAS9-3|PARP9\_MOUSE sp|Q640N1|AEBP1\_MOUSE tr|Q810Y7|Q810Y7\_MOUSE tr|B1AWL2|B1AWL2\_MOUSE sp|Q9CX00|IST1\_MOUSE sp|Q6PDH0|PHLB1\_MOUSE tr|D3Z4N0|D3Z4N0\_MOUSE tr|G3X9V8|G3X9V8\_MOUSE sp|Q6PDH0-2|PHLB1\_MOUSE tr|D3Z0X5|D3Z0X5\_MOUSE tr|E9PWB1|E9PWB1\_MOUSE sp|P10711-2|TCEA1\_MOUSE sp|P10711|TCEA1\_MOUSE tr|E9PYD5|E9PYD5\_MOUSE sp|A4Q9E5|TTLL3\_MOUSE tr|H7BX03|H7BX03\_MOUSE sp|Q920Q6|MSI2H\_MOUSE sp|Q9DBN9|DDX59\_MOUSE sp|Q920Q6-3|MSI2H\_MOUSE sp|Q80Y56|RBNS5\_MOUSE sp|Q920Q6-2|MSI2H\_MOUSE tr|A0A087WRB8|A0A087WRB8\_MOUSE tr|E9QL26|E9QL26\_MOUSE sp|Q9ER56|CAN12\_MOUSE tr|Q920Z2|Q920Z2\_MOUSE tr|E9PWV7|E9PWV7\_MOUSE sp|Q923K4|GTPB3\_MOUSE sp|A2AH22-5|AMRA1\_MOUSE tr|E9PZW8|E9PZW8\_MOUSE tr|B9EI38|B9EI38\_MOUSE tr|E9QKV6|E9QKV6\_MOUSE tr|A2AFI8|A2AFI8\_MOUSE sp|Q9QY06|MYO9B\_MOUSE sp|Q9QY06-2|MYO9B\_MOUSE sp|Q91ZI8|IOD3\_MOUSE tr|E9PWZ6|E9PWZ6\_MOUSE sp|Q9QY06-3|MYO9B\_MOUSE tr|Q7TQZ0|Q7TQZ0\_MOUSE tr|D3Z0K6|D3Z0K6\_MOUSE sp|Q5SUQ9-2|CTC1\_MOUSE sp|Q5SUQ9|CTC1\_MOUSE sp|Q5SUQ9-3|CTC1\_MOUSE tr|E0CXE7|E0CXE7\_MOUSE sp|Q6DFV5-3|HELZ\_MOUSE sp|Q6DFV5|HELZ\_MOUSE sp|Q9JLV5|CUL3\_MOUSE sp|Q9R0Q6|ARC1A\_MOUSE sp|P13597|ICAM1\_MOUSE sp|P13597-2|ICAM1\_MOUSE tr|E9Q4T8|E9Q4T8\_MOUSE tr|Q8C2W8|Q8C2W8\_MOUSE sp|O09010|LFNG\_MOUSE sp|P19182|IFRD1\_MOUSE sp|Q8CG47|SMC4\_MOUSE tr|D6RFB8|D6RFB8\_MOUSE sp|Q3UMY5-2|EMAL4\_MOUSE sp|Q3UMY5|EMAL4\_MOUSE sp|Q3UMY5-3|EMAL4\_MOUSE tr|F8WJ93|F8WJ93\_MOUSE tr|E9Q3R9|E9Q3R9\_MOUSE tr|E9Q444|E9Q444\_MOUSE tr|E9Q8Z1|E9Q8Z1\_MOUSE sp|Q8CJ19|MICA3\_MOUSE sp|Q8BQM8|EMAL5\_MOUSE tr|Q2LK61|Q2LK61\_MOUSE tr|Q2LKV8|Q2LKV8\_MOUSE sp|Q5Y5T3|ZDH23\_MOUSE tr|B7ZNY8|B7ZNY8\_MOUSE sp|Q9D1D6|CTHR1\_MOUSE tr|Q9JIC0|Q9JIC0\_MOUSE sp|Q8BVR6|RSPRY\_MOUSE sp|Q3U1V8|M3K9\_MOUSE sp|Q9D4B2|TTC25\_MOUSE sp|P41233|ABCA1\_MOUSE tr|E0CXG4|E0CXG4\_MOUSE sp|Q3UHK3|GREB1\_MOUSE sp|Q5DU14-2|MYO16\_MOUSE tr|F8WGF8|F8WGF8\_MOUSE sp|Q5DU14|MYO16\_MOUSE sp|Q5DU14-3|MYO16\_MOUSE sp|Q9Z2Q2|KNOP1\_MOUSE tr|D3YUE4|D3YUE4\_MOUSE sp|Q62273|S26A2\_MOUSE sp|P70227|ITPR3\_MOUSE sp|Q50L41|PA24F\_MOUSE sp|Q3TCX3|K0907\_MOUSE sp|Q3TCX3-2|K0907\_MOUSE sp|Q3TCX3-3|K0907\_MOUSE sp|P60882|MEGF8\_MOUSE tr|A2ALS3|A2ALS3\_MOUSE tr|E9PUJ8|E9PUJ8\_MOUSE tr|B2RXQ2|B2RXQ2\_MOUSE sp|A2AVJ5|PRR5L\_MOUSE sp|Q684R7-3|FREM1\_MOUSE sp|Q8C784|CP052\_MOUSE sp|Q8CB59-2|F161B\_MOUSE sp|Q8CB59|F161B\_MOUSE sp|E9Q9D5-1|RBL2A\_MOUSE sp|A2AP18|PLCH2\_MOUSE sp|A2AP18-4|PLCH2\_MOUSE tr|H3BK11|H3BK11\_MOUSE tr|F7AGW8|F7AGW8\_MOUSE tr|E0CXK2|E0CXK2\_MOUSE tr|E9Q8F4|E9Q8F4\_MOUSE tr|Q8BGK0|Q8BGK0\_MOUSE tr|Q8C9V5|Q8C9V5\_MOUSE tr|B1AWQ4|B1AWQ4\_MOUSE tr|B1AWQ1|B1AWQ1\_MOUSE sp|Q9ET47|ESPN\_MOUSE tr|B1AWQ3|B1AWQ3\_MOUSE sp|Q6PB44-2|PTN23\_MOUSE sp|Q6PB44|PTN23\_MOUSE tr|Q91XX9|Q91XX9\_MOUSE sp|Q80XZ4|I22R1\_MOUSE sp|Q8R2M2|TDIF2\_MOUSE sp|P70261|PALD\_MOUSE tr|E9Q8K6|E9Q8K6\_MOUSE sp|Q1W617|SHRM4\_MOUSE sp|Q1W617-2|SHRM4\_MOUSE sp|Q9QXN0-4|SHRM3\_MOUSE sp|Q9QXN0-3|SHRM3\_MOUSE sp|Q9CWU5|KHDC3\_MOUSE tr|G3UZ71|G3UZ71\_MOUSE sp|Q9CWU5-3|KHDC3\_MOUSE sp|Q9CWU5-2|KHDC3\_MOUSE sp|Q99MR3|S12A9\_MOUSE tr|G3UZ86|G3UZ86\_MOUSE sp|P48379-2|RFX2\_MOUSE tr|Q923Q8|Q923Q8\_MOUSE sp|Q5UKY4-7|MO2R3\_MOUSE sp|Q5UKY4-2|MO2R3\_MOUSE sp|Q8BG32|PSD11\_MOUSE sp|Q3UMR0-2|ANR27\_MOUSE sp|Q3UMR0-3|ANR27\_MOUSE sp|Q3UMR0|ANR27\_MOUSE sp|Q9R1Z8|VINEX\_MOUSE tr|G3UZW3|G3UZW3\_MOUSE sp|A2AKB9|DCA10\_MOUSE sp|A2AKB9-3|DCA10\_MOUSE sp|Q8C8T7|ELFN1\_MOUSE tr|Q8C1C2|Q8C1C2\_MOUSE sp|P39054-2|DYN2\_MOUSE tr|F8WIV5|F8WIV5\_MOUSE tr|Q3TCR7|Q3TCR7\_MOUSE tr|G3X9G4|G3X9G4\_MOUSE sp|P39054|DYN2\_MOUSE sp|Q9D5R3|CEP83\_MOUSE sp|P09581|CSF1R\_MOUSE sp|Q8CDI2|FBX43\_MOUSE sp|O54988-2|SLK\_MOUSE tr|G3X9B2|G3X9B2\_MOUSE sp|O54988|SLK\_MOUSE sp|Q99N50-5|SYTL2\_MOUSE tr|F6YZZ8|F6YZZ8\_MOUSE tr|B1AXJ6|B1AXJ6\_MOUSE sp|Q99N50-11|SYTL2\_MOUSE sp|Q99N50-8|SYTL2\_MOUSE sp|Q99N50-7|SYTL2\_MOUSE tr|B1AXJ4|B1AXJ4\_MOUSE tr|B1AXJ5|B1AXJ5\_MOUSE tr|E9PUC4|E9PUC4\_MOUSE sp|Q6NSW3-2|SPKAP\_MOUSE sp|Q8BV79-2|TRNK1\_MOUSE tr|G3UZC7|G3UZC7\_MOUSE tr|E9Q0M3|E9Q0M3\_MOUSE sp|Q8BLR7|RND1\_MOUSE sp|O89019-7|INVS\_MOUSE tr|E9Q0U7|E9Q0U7\_MOUSE sp|Q61699|HS105\_MOUSE sp|Q61699-2|HS105\_MOUSE sp|A2AN08-2|UBR4\_MOUSE tr|D6RIN1|D6RIN1\_MOUSE sp|P42703|LIFR\_MOUSE sp|Q3U1Y4-2|DEN4B\_MOUSE tr|E9QAB8|E9QAB8\_MOUSE sp|Q8K441|ABCA6\_MOUSE sp|Q3U1Y4|DEN4B\_MOUSE tr|A3KFM8|A3KFM8\_MOUSE tr|V9GXK6|V9GXK6\_MOUSE tr|G3X8T2|G3X8T2\_MOUSE sp|Q3U182|CRTC2\_MOUSE sp|Q0P678|ZCH18\_MOUSE tr|H3BIW0|H3BIW0\_MOUSE tr|E9Q0K5|E9Q0K5\_MOUSE tr|B1AVD1|B1AVD1\_MOUSE tr|B1AVD2|B1AVD2\_MOUSE sp|Q8R216|SIR4\_MOUSE sp|Q8BG36|DMRT2\_MOUSE tr|E9Q8C0|E9Q8C0\_MOUSE tr|D3Z6E8|D3Z6E8\_MOUSE sp|Q8BHE4|TM108\_MOUSE sp|Q9D0T7|GKN3\_MOUSE tr|A2AFP5|A2AFP5\_MOUSE sp|Q80U49|C170B\_MOUSE sp|Q3U2K0|F193B\_MOUSE sp|Q3U2K0-2|F193B\_MOUSE tr|E9QPZ3|E9QPZ3\_MOUSE tr|F6QFB4|F6QFB4\_MOUSE tr|F6RYL8|F6RYL8\_MOUSE tr|E9Q472|E9Q472\_MOUSE tr|F6RCU2|F6RCU2\_MOUSE sp|Q9R1V4|ADA11\_MOUSE tr|Q7TQG7|Q7TQG7\_MOUSE sp|Q9Z0U1|ZO2\_MOUSE tr|F7AWS1|F7AWS1\_MOUSE sp|Q80Z19|MUC2\_MOUSE sp|Q6PAL0|BEND3\_MOUSE tr|F7BQE4|F7BQE4\_MOUSE sp|Q62261|SPTB2\_MOUSE sp|E9Q634|MYO1E\_MOUSE tr|Q059V1|Q059V1\_MOUSE tr|F6U3S2|F6U3S2\_MOUSE sp|Q8BMQ3|BNC2\_MOUSE tr|H3BIU2|H3BIU2\_MOUSE sp|Q8BMQ3-2|BNC2\_MOUSE sp|A2AWL7-3|MGAP\_MOUSE sp|Q920R0|ALS2\_MOUSE tr|F8WHJ2|F8WHJ2\_MOUSE tr|Q6DFW5|Q6DFW5\_MOUSE tr|A2AEB7|A2AEB7\_MOUSE sp|Q99JT1|GATB\_MOUSE tr|A2AEB8|A2AEB8\_MOUSE tr|A2AEB5|A2AEB5\_MOUSE tr|A2AEB6|A2AEB6\_MOUSE tr|A2AEB3|A2AEB3\_MOUSE tr|A2AEB4|A2AEB4\_MOUSE tr|H3BJG4|H3BJG4\_MOUSE sp|Q9CXF4|TBC15\_MOUSE sp|Q5RJ54|ZSC26\_MOUSE tr|E9Q7M2|E9Q7M2\_MOUSE sp|Q8CJC5|NEUL3\_MOUSE tr|E9Q3N0|E9Q3N0\_MOUSE sp|Q9Z129|RECQ1\_MOUSE sp|Q9Z129-2|RECQ1\_MOUSE sp|Q61830|MRC1\_MOUSE sp|Q8BMB0-3|EMSY\_MOUSE sp|Q8BMB0|EMSY\_MOUSE tr|E9Q4S3|E9Q4S3\_MOUSE sp|Q61043|NIN\_MOUSE sp|Q61043-2|NIN\_MOUSE tr|G3X9M3|G3X9M3\_MOUSE tr|A2AH85|A2AH85\_MOUSE sp|O08810|U5S1\_MOUSE tr|G3UZ34|G3UZ34\_MOUSE sp|Q9JI70|MKKS\_MOUSE sp|Q9EST3-2|4ET\_MOUSE sp|P16675|PPGB\_MOUSE sp|Q6P4S8|INT1\_MOUSE sp|O55111|DSG2\_MOUSE sp|Q920R0-2|ALS2\_MOUSE tr|Q504P4|Q504P4\_MOUSE sp|P63017|HSP7C\_MOUSE sp|Q9DBA6|TYSD1\_MOUSE sp|Q6ZPY2-2|SMG5\_MOUSE sp|Q6IUP1|SOLH1\_MOUSE tr|E9Q616|E9Q616\_MOUSE sp|Q6PCM1|KDM3A\_MOUSE tr|E9Q743|E9Q743\_MOUSE tr|E9QM99|E9QM99\_MOUSE sp|Q8BZN6|DOC10\_MOUSE tr|A0A087WQ86|A0A087WQ86\_MOUSE tr|A0A087WRP5|A0A087WRP5\_MOUSE sp|P41183|BCL6\_MOUSE tr|Q9DAS5|Q9DAS5\_MOUSE tr|Q3UKP4|Q3UKP4\_MOUSE tr|Q3V2Q3|Q3V2Q3\_MOUSE sp|E9Q557|DESP\_MOUSE tr|F6R1B7|F6R1B7\_MOUSE tr|E9PZQ1|E9PZQ1\_MOUSE tr|F6WLY0|F6WLY0\_MOUSE tr|G3X9F7|G3X9F7\_MOUSE sp|P97927|LAMA4\_MOUSE sp|Q62137|JAK3\_MOUSE sp|Q62137-2|JAK3\_MOUSE sp|Q6PIX5|RHDF1\_MOUSE sp|Q8BYW1-2|RHG25\_MOUSE sp|Q8BYW1-3|RHG25\_MOUSE sp|Q8BYW1|RHG25\_MOUSE sp|Q61584-2|FXR1\_MOUSE tr|F7CKP4|F7CKP4\_MOUSE tr|F6R186|F6R186\_MOUSE Q2KJC7 sp|Q8BLB0|ZFP3\_MOUSE tr|B8JJZ9|B8JJZ9\_MOUSE sp|Q8BGB2|TTC7A\_MOUSE sp|Q3U1J1|TFPT\_MOUSE sp|Q9DA17|TSG13\_MOUSE sp|Q6J756|CAN11\_MOUSE sp|Q05421|CP2E1\_MOUSE sp|Q64287|IRF4\_MOUSE tr|E0CZ58|E0CZ58\_MOUSE sp|Q61818-2|RAI1\_MOUSE sp|Q61818|RAI1\_MOUSE tr|E9QQ18|E9QQ18\_MOUSE tr|E9PZ43|E9PZ43\_MOUSE sp|P27546-4|MAP4\_MOUSE tr|E9PWC0|E9PWC0\_MOUSE sp|Q7TS74|CKP2L\_MOUSE tr|B1AQZ2|B1AQZ2\_MOUSE tr|E9PUN2|E9PUN2\_MOUSE sp|O88705|HCN3\_MOUSE sp|Q9Z110|P5CS\_MOUSE sp|Q9Z110-2|P5CS\_MOUSE sp|O09012|PEX5\_MOUSE sp|Q9CQJ0|ACO15\_MOUSE tr|G3X9Z4|G3X9Z4\_MOUSE tr|E9Q9P4|E9Q9P4\_MOUSE sp|Q9JHB4|WDR31\_MOUSE sp|P28738|KIF5C\_MOUSE sp|Q9JHB4-2|WDR31\_MOUSE sp|Q8R418|DICER\_MOUSE tr|Q91XX0|Q91XX0\_MOUSE sp|Q5S003|SPG17\_MOUSE tr|A3KGI3|A3KGI3\_MOUSE tr|B2RUQ2|B2RUQ2\_MOUSE sp|Q70IV5|SYNEM\_MOUSE tr|E9Q236|E9Q236\_MOUSE tr|A2BDY3|A2BDY3\_MOUSE sp|G5E8K5-2|ANK3\_MOUSE sp|P53395|ODB2\_MOUSE tr|E9Q467|E9Q467\_MOUSE sp|Q61060|FOXD3\_MOUSE tr|B5THE2|B5THE2\_MOUSE sp|A3KGS3-2|RGPA2\_MOUSE tr|F7AI67|F7AI67\_MOUSE sp|Q8K3X4|I2BPL\_MOUSE sp|Q8BQQ1|ZDH14\_MOUSE tr|M0QW51|M0QW51\_MOUSE sp|O88196-3|TTC3\_MOUSE sp|P01869|IGH1M\_MOUSE sp|O88196-5|TTC3\_MOUSE sp|Q8BGQ2|CSRN2\_MOUSE sp|O88196|TTC3\_MOUSE tr|G5E8T2|G5E8T2\_MOUSE sp|O88196-2|TTC3\_MOUSE tr|K7N6U8|K7N6U8\_MOUSE sp|O88196-4|TTC3\_MOUSE sp|Q6NVD0|FREM2\_MOUSE tr|M0QWQ9|M0QWQ9\_MOUSE sp|O88196-6|TTC3\_MOUSE sp|Q61555|FBN2\_MOUSE sp|Q80X41-2|VRK1\_MOUSE tr|D3YYT0|D3YYT0\_MOUSE sp|Q80X41-3|VRK1\_MOUSE sp|Q80X41|VRK1\_MOUSE sp|P15116|CADH2\_MOUSE sp|Q80X41-5|VRK1\_MOUSE sp|Q8VID5|RECQ5\_MOUSE sp|O70372|TERT\_MOUSE sp|Q7TMF2|ERI1\_MOUSE sp|Q5SWP3|NACAD\_MOUSE tr|G3X9V7|G3X9V7\_MOUSE tr|E9Q3E9|E9Q3E9\_MOUSE sp|Q0VGY8-2|TANC1\_MOUSE sp|Q6A0A2-3|LAR4B\_MOUSE tr|G5E890|G5E890\_MOUSE sp|P48381|RFX3\_MOUSE sp|Q8C6K9-2|CO6A6\_MOUSE sp|Q91ZT9|ASB8\_MOUSE sp|Q8C547-2|HTR5B\_MOUSE tr|Q9D4I8|Q9D4I8\_MOUSE sp|Q8CJ12|GPR64\_MOUSE sp|Q8CJ12-3|GPR64\_MOUSE tr|Q14BH6|Q14BH6\_MOUSE tr|A2AHQ2|A2AHQ2\_MOUSE sp|Q8CJ12-5|GPR64\_MOUSE sp|Q8CJ12-4|GPR64\_MOUSE sp|Q8CJ12-2|GPR64\_MOUSE tr|E9PWG6|E9PWG6\_MOUSE sp|P26043|RADI\_MOUSE tr|A2AW86|A2AW86\_MOUSE sp|Q60767|LY75\_MOUSE tr|F7AMU5|F7AMU5\_MOUSE sp|Q8K1N2-2|PHLB2\_MOUSE sp|Q8K1N2|PHLB2\_MOUSE tr|D3Z069|D3Z069\_MOUSE tr|D3Z742|D3Z742\_MOUSE sp|Q8C525|M21D2\_MOUSE sp|Q9JL19|NCOA6\_MOUSE tr|B1ATV3|B1ATV3\_MOUSE tr|L7N2D1|L7N2D1\_MOUSE sp|Q9QZC1|TRPC3\_MOUSE tr|D3Z566|D3Z566\_MOUSE sp|Q8BT18|CN037\_MOUSE tr|K7N6Z2|K7N6Z2\_MOUSE tr|L7N269|L7N269\_MOUSE sp|Q80U78-2|PUM1\_MOUSE sp|Q80U78-3|PUM1\_MOUSE sp|Q80U78|PUM1\_MOUSE sp|Q6VGS5|DAPLE\_MOUSE sp|Q6VGS5-2|DAPLE\_MOUSE sp|Q3TQQ9-2|CA112\_MOUSE sp|Q3TQQ9|CA112\_MOUSE tr|B1AVN5|B1AVN5\_MOUSE sp|Q3TQQ9-3|CA112\_MOUSE sp|Q62383|SPT6H\_MOUSE sp|Q3U487|HECD3\_MOUSE sp|Q9QXK7|CPSF3\_MOUSE sp|Q9JKK8|ATR\_MOUSE sp|Q8K557|ONEC3\_MOUSE tr|E9QPK4|E9QPK4\_MOUSE sp|Q3UU96-2|MRCKA\_MOUSE tr|H7BX44|H7BX44\_MOUSE sp|Q810U4-3|NRCAM\_MOUSE sp|Q810U4-2|NRCAM\_MOUSE sp|Q810U4|NRCAM\_MOUSE sp|Q9WUB6|CLCKB\_MOUSE sp|Q9WUB6-2|CLCKB\_MOUSE tr|Q9EQ84|Q9EQ84\_MOUSE sp|Q4VC17|ATS18\_MOUSE sp|Q9D7V1|SH24A\_MOUSE sp|Q8VBT6|APOBR\_MOUSE sp|Q921D9|GRHL1\_MOUSE tr|Q3TBA3|Q3TBA3\_MOUSE sp|P21958|TAP1\_MOUSE sp|Q811S7-2|UBIP1\_MOUSE sp|Q811S7|UBIP1\_MOUSE sp|Q6KAS7-2|ZN521\_MOUSE sp|Q6KAS7|ZN521\_MOUSE sp|Q9CZK6|ANKS3\_MOUSE sp|Q7TSF0|DSG1C\_MOUSE sp|Q7TSY8|SGOL2\_MOUSE sp|Q3V0C1|ZMAT1\_MOUSE sp|Q9WTN3-3|SRBP1\_MOUSE sp|Q9WTN3|SRBP1\_MOUSE sp|Q5FWH7-2|S39AC\_MOUSE sp|Q9JM13|RABX5\_MOUSE tr|E9PZX4|E9PZX4\_MOUSE tr|G5E8Y4|G5E8Y4\_MOUSE sp|Q9D180|WDR65\_MOUSE tr|F6QGP8|F6QGP8\_MOUSE sp|P97496-2|SMRC1\_MOUSE tr|Q3UNN4|Q3UNN4\_MOUSE sp|P97496|SMRC1\_MOUSE sp|Q3UVV9|VWA3A\_MOUSE tr|E9Q0W9|E9Q0W9\_MOUSE sp|Q9Z2I4-2|ROBO3\_MOUSE tr|Q8BKL6|Q8BKL6\_MOUSE sp|Q61495|DSG1A\_MOUSE tr|E9QMD2|E9QMD2\_MOUSE sp|Q8BU91|S26A9\_MOUSE tr|E9QNY9|E9QNY9\_MOUSE sp|Q80TV8-2|CLAP1\_MOUSE tr|A0A087WRE8|A0A087WRE8\_MOUSE tr|E9PXU1|E9PXU1\_MOUSE sp|Q76LL6|FHOD3\_MOUSE sp|Q68FE8-3|Z280D\_MOUSE sp|Q76LL6-4|FHOD3\_MOUSE sp|Q76LL6-3|FHOD3\_MOUSE sp|Q76LL6-2|FHOD3\_MOUSE sp|Q9JKX8|UPK3A\_MOUSE sp|P50544|ACADV\_MOUSE tr|B1AR28|B1AR28\_MOUSE sp|Q9D6A1|MYO1H\_MOUSE sp|Q60934|GRIK1\_MOUSE tr|B7FAV1|B7FAV1\_MOUSE sp|P31648|SC6A1\_MOUSE sp|Q3UNH4|GRIN1\_MOUSE tr|B1AVB1|B1AVB1\_MOUSE tr|Q6NV99|Q6NV99\_MOUSE tr|E9Q5A6|E9Q5A6\_MOUSE sp|Q06180|PTN2\_MOUSE tr|D3YZ10|D3YZ10\_MOUSE tr|A0A087WRU2|A0A087WRU2\_MOUSE tr|D3Z6W2|D3Z6W2\_MOUSE sp|Q06180-2|PTN2\_MOUSE tr|D3Z525|D3Z525\_MOUSE sp|Q9QZ85|IIGP1\_MOUSE sp|Q3UWZ0|TRI75\_MOUSE tr|H3BJI8|H3BJI8\_MOUSE tr|Q8VFI8|Q8VFI8\_MOUSE sp|Q68FH0|PKP4\_MOUSE tr|F6WYQ5|F6WYQ5\_MOUSE sp|P70704|AT8A1\_MOUSE sp|Q7M721|TR120\_MOUSE tr|Q7TNB5|Q7TNB5\_MOUSE sp|Q99KW3|TARA\_MOUSE sp|Q99KW3-2|TARA\_MOUSE sp|Q99KW3-4|TARA\_MOUSE tr|Q810G6|Q810G6\_MOUSE sp|P07350|IFNA6\_MOUSE tr|Q810G5|Q810G5\_MOUSE sp|Q80SS5|IFNAC\_MOUSE tr|B9EJR8|B9EJR8\_MOUSE tr|D3Z5M7|D3Z5M7\_MOUSE tr|D3YZM4|D3YZM4\_MOUSE sp|O88689-2|PCDA4\_MOUSE sp|O88689|PCDA4\_MOUSE tr|E9PYU4|E9PYU4\_MOUSE tr|E9PYL1|E9PYL1\_MOUSE sp|A2A8L1|CHD5\_MOUSE tr|Q6PCM4|Q6PCM4\_MOUSE sp|P56655|CP238\_MOUSE sp|Q61464-5|ZN638\_MOUSE tr|E9QKZ7|E9QKZ7\_MOUSE sp|Q61464-2|ZN638\_MOUSE sp|P13808|B3A2\_MOUSE sp|P13808-3|B3A2\_MOUSE sp|Q8BVR6-3|RSPRY\_MOUSE sp|P13808-2|B3A2\_MOUSE sp|Q8BTZ7|GMPPB\_MOUSE tr|B7ZC54|B7ZC54\_MOUSE tr|B7ZC57|B7ZC57\_MOUSE tr|B7ZC55|B7ZC55\_MOUSE tr|Q91Y21|Q91Y21\_MOUSE tr|D3Z2A3|D3Z2A3\_MOUSE tr|Q6P1Y9|Q6P1Y9\_MOUSE tr|Q5PPR2|Q5PPR2\_MOUSE tr|D3YVY9|D3YVY9\_MOUSE sp|Q14AI0|DCC1\_MOUSE sp|Q8R3S6|EXOC1\_MOUSE tr|S4R236|S4R236\_MOUSE tr|S4R2F5|S4R2F5\_MOUSE sp|G5E8K5-4|ANK3\_MOUSE sp|G5E8K5-5|ANK3\_MOUSE tr|F6UTU1|F6UTU1\_MOUSE sp|P70704-3|AT8A1\_MOUSE sp|P70704-2|AT8A1\_MOUSE tr|F6U9Y2|F6U9Y2\_MOUSE tr|G3UXK8|G3UXK8\_MOUSE sp|P70700|RPA2\_MOUSE sp|D2EAC2-2|ZBED6\_MOUSE sp|D2EAC2|ZBED6\_MOUSE sp|Q63870|CO7A1\_MOUSE tr|E9PUP3|E9PUP3\_MOUSE tr|Q8BUG8|Q8BUG8\_MOUSE sp|Q499E4-2|DZI1L\_MOUSE tr|F7AC58|F7AC58\_MOUSE tr|Q0VF55|Q0VF55\_MOUSE sp|Q9QYB8|ADDB\_MOUSE sp|Q9QYB8-3|ADDB\_MOUSE sp|Q8CG03|PDE5A\_MOUSE sp|Q8BJ66|KAZD1\_MOUSE tr|D3Z224|D3Z224\_MOUSE sp|Q07279|NFE2\_MOUSE sp|Q6VVW5|ANPRB\_MOUSE sp|Q6VVW5-2|ANPRB\_MOUSE sp|Q9D4K7|CC105\_MOUSE sp|Q8C437-3|PEX5R\_MOUSE tr|F8SLQ3|F8SLQ3\_MOUSE sp|Q80V94|AP4E1\_MOUSE sp|Q920V1|B3GL1\_MOUSE tr|F6RUI8|F6RUI8\_MOUSE tr|V9GXN6|V9GXN6\_MOUSE sp|Q8R0S1|ATF7\_MOUSE sp|Q3UHD1|BAI1\_MOUSE tr|Q3US59|Q3US59\_MOUSE tr|G3X8Y9|G3X8Y9\_MOUSE tr|E9PVV8|E9PVV8\_MOUSE tr|F6WVU1|F6WVU1\_MOUSE tr|K3W4P7|K3W4P7\_MOUSE tr|J3QPS3|J3QPS3\_MOUSE tr|J3QNF4|J3QNF4\_MOUSE sp|P14142|GTR4\_MOUSE tr|J3QK17|J3QK17\_MOUSE tr|A2BDP0|A2BDP0\_MOUSE tr|K7N705|K7N705\_MOUSE tr|G3UYA8|G3UYA8\_MOUSE tr|A0A087WP42|A0A087WP42\_MOUSE tr|A0A087WQ95|A0A087WQ95\_MOUSE sp|Q8CHG7|RPGF2\_MOUSE tr|E9QNQ4|E9QNQ4\_MOUSE sp|Q8C456-2|FRITZ\_MOUSE sp|Q8C456|FRITZ\_MOUSE sp|Q8JZP9|GA2L1\_MOUSE sp|O54949|NLK\_MOUSE tr|G3UZ92|G3UZ92\_MOUSE tr|E9Q564|E9Q564\_MOUSE tr|Q78TU8|Q78TU8\_MOUSE tr|A9C450|A9C450\_MOUSE sp|Q4PZA2-2|ECE1\_MOUSE tr|Q3UD93|Q3UD93\_MOUSE tr|E9Q9W7|E9Q9W7\_MOUSE sp|Q8K442|ABC8A\_MOUSE sp|Q9JJ69-4|KCIP2\_MOUSE sp|Q9DBS4|ACOXL\_MOUSE sp|P97305|NFAC3\_MOUSE sp|P97305-3|NFAC3\_MOUSE tr|D3YZC8|D3YZC8\_MOUSE tr|M0QWF3|M0QWF3\_MOUSE sp|Q7TSI3|PP6R1\_MOUSE sp|Q63961|EGLN\_MOUSE tr|E9Q5I7|E9Q5I7\_MOUSE sp|Q62445|SP4\_MOUSE sp|Q7TSK2-3|SEZ6\_MOUSE sp|P42227-2|STAT3\_MOUSE sp|Q3USB7|PLCL1\_MOUSE tr|B1ARZ3|B1ARZ3\_MOUSE sp|P0C6A1|GTR7\_MOUSE tr|A2ASI9|A2ASI9\_MOUSE sp|Q8BY89|CTL2\_MOUSE sp|Q8BY89-2|CTL2\_MOUSE sp|P31001|DESM\_MOUSE tr|G3X8W1|G3X8W1\_MOUSE tr|F6RIN9|F6RIN9\_MOUSE sp|Q80U72-3|SCRIB\_MOUSE sp|Q80U72-4|SCRIB\_MOUSE sp|Q8C561-2|LMBD2\_MOUSE sp|Q5KU39|VPS41\_MOUSE sp|Q2VPA6|HELQ\_MOUSE sp|E9QAF0|SPT31\_MOUSE sp|Q8CHG3|GCC2\_MOUSE sp|P27577|ETS1\_MOUSE sp|P56481|GASR\_MOUSE tr|E9PWI6|E9PWI6\_MOUSE tr|B2RSU7|B2RSU7\_MOUSE sp|P59808|SASH1\_MOUSE tr|F8VQK5|F8VQK5\_MOUSE tr|A3KGR9|A3KGR9\_MOUSE sp|A2APU8|F205A\_MOUSE tr|D3YZF6|D3YZF6\_MOUSE sp|A2A259|PK2L1\_MOUSE sp|Q8BJS7|MAP10\_MOUSE tr|F6T4V9|F6T4V9\_MOUSE sp|Q9Z268|RASL1\_MOUSE sp|Q9WVL5|MORC1\_MOUSE sp|Q8CCJ9|P20L1\_MOUSE sp|Q8CCJ9-4|P20L1\_MOUSE tr|E9QKD1|E9QKD1\_MOUSE sp|Q3UHX0|NOL8\_MOUSE sp|Q692V3|GNN\_MOUSE sp|Q99MS7|EH1L1\_MOUSE tr|A0A075B646|A0A075B646\_MOUSE sp|Q7M6Y5-2|DEUP1\_MOUSE sp|Q7M6Y5|DEUP1\_MOUSE sp|Q8VHX6|FLNC\_MOUSE tr|D3Z576|D3Z576\_MOUSE sp|Q8VHX6-2|FLNC\_MOUSE tr|D3YW87|D3YW87\_MOUSE sp|Q3TBD2-3|HMHA1\_MOUSE sp|Q3TBD2-4|HMHA1\_MOUSE sp|P27600|GNA12\_MOUSE sp|Q7TQA1-3|IGSF1\_MOUSE sp|Q7TQA1-2|IGSF1\_MOUSE sp|Q3UY90-2|F198A\_MOUSE sp|Q3UY90|F198A\_MOUSE tr|E9PUN7|E9PUN7\_MOUSE sp|Q3UY90-3|F198A\_MOUSE sp|Q7TQA1|IGSF1\_MOUSE tr|F6SIC5|F6SIC5\_MOUSE tr|F7CDW2|F7CDW2\_MOUSE tr|F2Z3W0|F2Z3W0\_MOUSE tr|F2Z3X2|F2Z3X2\_MOUSE tr|F6X2K7|F6X2K7\_MOUSE tr|E9Q1M8|E9Q1M8\_MOUSE sp|Q811L6-2|MAST4\_MOUSE tr|E9Q4Q9|E9Q4Q9\_MOUSE sp|Q8K0L2|ENTP8\_MOUSE sp|Q8K0L2-2|ENTP8\_MOUSE sp|Q8K1G2|LBN\_MOUSE sp|O55225|OTOG\_MOUSE sp|P57776-3|EF1D\_MOUSE tr|G3UWC8|G3UWC8\_MOUSE sp|Q80TC6|ZSWM5\_MOUSE sp|Q61749|EI2BD\_MOUSE tr|D3Z780|D3Z780\_MOUSE tr|F8WGE3|F8WGE3\_MOUSE tr|Q5QNR3|Q5QNR3\_MOUSE tr|D3Z4K0|D3Z4K0\_MOUSE sp|P26323|FLI1\_MOUSE sp|Q8K389|CK5P2\_MOUSE sp|P97813|PLD2\_MOUSE tr|Q6NV49|Q6NV49\_MOUSE sp|Q9DC71|RT15\_MOUSE sp|Q9CQT7|DESI1\_MOUSE tr|Q8C0S5|Q8C0S5\_MOUSE sp|Q99KD5|UN45A\_MOUSE sp|Q9ERD6-2|RGPS2\_MOUSE sp|Q9JHI0|MMP19\_MOUSE sp|Q9ERD6|RGPS2\_MOUSE sp|O08523|TECTA\_MOUSE sp|O08523-2|TECTA\_MOUSE sp|Q8BXJ2-2|TREF1\_MOUSE sp|Q8BGT6|MILK1\_MOUSE tr|S4R199|S4R199\_MOUSE tr|E9QAS9|E9QAS9\_MOUSE tr|D3Z6U8|D3Z6U8\_MOUSE sp|P35922-3|FMR1\_MOUSE tr|E9QNF5|E9QNF5\_MOUSE sp|P35922-7|FMR1\_MOUSE tr|E9QAT0|E9QAT0\_MOUSE sp|A2AUQ7|GTA1L\_MOUSE sp|P35922|FMR1\_MOUSE sp|P35922-9|FMR1\_MOUSE tr|A2AMY5|A2AMY5\_MOUSE sp|Q91VX2|UBAP2\_MOUSE tr|E9QAS8|E9QAS8\_MOUSE tr|Q6AXB7|Q6AXB7\_MOUSE tr|F6WNY1|F6WNY1\_MOUSE tr|F6Q5A5|F6Q5A5\_MOUSE tr|A2RT18|A2RT18\_MOUSE tr|F6S5Z6|F6S5Z6\_MOUSE tr|A2AE21|A2AE21\_MOUSE sp|Q497L8|S22AG\_MOUSE sp|Q3UIZ8|MYLK3\_MOUSE sp|Q497L8-2|S22AG\_MOUSE sp|Q99MR1|PERQ1\_MOUSE sp|O08691|ARGI2\_MOUSE sp|Q80U56|AVL9\_MOUSE tr|G3UXW5|G3UXW5\_MOUSE sp|Q8C8U0-3|LIPB1\_MOUSE tr|E9PZF4|E9PZF4\_MOUSE sp|Q9ERC8|DSCAM\_MOUSE sp|Q80XB4-2|NRAP\_MOUSE sp|Q80XB4-4|NRAP\_MOUSE sp|Q80XB4|NRAP\_MOUSE tr|G3UYP9|G3UYP9\_MOUSE tr|D3Z5H5|D3Z5H5\_MOUSE tr|Q3V3N7|Q3V3N7\_MOUSE tr|E0CXB9|E0CXB9\_MOUSE sp|Q61301-3|CTNA2\_MOUSE sp|O70354|CAH11\_MOUSE sp|Q8CHV6|TAD2A\_MOUSE tr|B1AT25|B1AT25\_MOUSE sp|Q8C5R2|PRSR2\_MOUSE sp|Q6PDK8|DTX4\_MOUSE tr|E9PYZ7|E9PYZ7\_MOUSE sp|Q33DR3|DLP1\_MOUSE tr|G3UZ33|G3UZ33\_MOUSE tr|Q7TRC0|Q7TRC0\_MOUSE sp|Q8CC88-2|VWA8\_MOUSE sp|Q8BXJ2|TREF1\_MOUSE sp|Q8BXJ2-4|TREF1\_MOUSE tr|F8VQ94|F8VQ94\_MOUSE tr|D3Z3W9|D3Z3W9\_MOUSE tr|E9Q1I3|E9Q1I3\_MOUSE tr|V9GWS5|V9GWS5\_MOUSE sp|Q8BXA0|LRFN5\_MOUSE sp|Q925I1-2|ATAD3\_MOUSE sp|Q5DTV4|ZC12C\_MOUSE sp|Q925I1|ATAD3\_MOUSE sp|Q8BXA0-2|LRFN5\_MOUSE tr|H3BKI6|H3BKI6\_MOUSE sp|P16406|AMPE\_MOUSE tr|F7B9G4|F7B9G4\_MOUSE sp|Q5SS00|ZDBF2\_MOUSE tr|E9PX82|E9PX82\_MOUSE sp|Q8BU03|PWP2\_MOUSE sp|P84091|AP2M1\_MOUSE tr|Q3TWV4|Q3TWV4\_MOUSE sp|Q9QY84|ACL7A\_MOUSE tr|Q7TR79|Q7TR79\_MOUSE tr|G3UYK4|G3UYK4\_MOUSE tr|A2AKM8|A2AKM8\_MOUSE tr|G3UY80|G3UY80\_MOUSE tr|G3UXF6|G3UXF6\_MOUSE tr|A2AKM7|A2AKM7\_MOUSE sp|Q8BHB4|WDR3\_MOUSE tr|A2AKM5|A2AKM5\_MOUSE sp|Q02650|PAX5\_MOUSE tr|G3V008|G3V008\_MOUSE tr|F6ZRL3|F6ZRL3\_MOUSE tr|A2ARU6|A2ARU6\_MOUSE tr|E0CY10|E0CY10\_MOUSE tr|A0A087WSQ1|A0A087WSQ1\_MOUSE tr|L7N2E5|L7N2E5\_MOUSE tr|Q80Z08|Q80Z08\_MOUSE sp|Q9EQH3|VPS35\_MOUSE tr|F8VQE6|F8VQE6\_MOUSE tr|B7ZNL9|B7ZNL9\_MOUSE sp|Q63ZW7|INADL\_MOUSE sp|Q63ZW7-2|INADL\_MOUSE sp|A2AHL1|ANO3\_MOUSE sp|A2AHL1-2|ANO3\_MOUSE sp|Q8BU30|SYIC\_MOUSE sp|Q7TQK1|INT7\_MOUSE tr|Z4YL78|Z4YL78\_MOUSE sp|A2AGT5-2|CKAP5\_MOUSE sp|Q9CRD0-2|OCAD1\_MOUSE tr|E9PUP1|E9PUP1\_MOUSE sp|O09158|CP3AP\_MOUSE sp|Q9CRD0-3|OCAD1\_MOUSE sp|P61406|EST1A\_MOUSE tr|H3BJY3|H3BJY3\_MOUSE sp|Q91VY5|KDM4B\_MOUSE tr|Q3V118|Q3V118\_MOUSE sp|Q8CHC8|ADNP2\_MOUSE tr|A2AI57|A2AI57\_MOUSE sp|Q923J1|TRPM7\_MOUSE sp|Q9D1D4|TMEDA\_MOUSE tr|Q7M6X3|Q7M6X3\_MOUSE sp|Q9D1D4-2|TMEDA\_MOUSE tr|I7HPW1|I7HPW1\_MOUSE sp|Q8K370|ACD10\_MOUSE tr|E9Q3L2|E9Q3L2\_MOUSE sp|O35149|ZNT4\_MOUSE sp|Q5RJI5|BRSK1\_MOUSE tr|D3Z5P0|D3Z5P0\_MOUSE sp|Q8C052|MAP1S\_MOUSE tr|F6YXH5|F6YXH5\_MOUSE sp|Q6PZE0|MUC19\_MOUSE tr|A2BDW0|A2BDW0\_MOUSE tr|E9PUM6|E9PUM6\_MOUSE tr|D3Z356|D3Z356\_MOUSE tr|Q91Y20|Q91Y20\_MOUSE sp|P35991|BTK\_MOUSE tr|Q9CVV2|Q9CVV2\_MOUSE sp|Q9Z180|SETBP\_MOUSE sp|Q6NXK2|ZN532\_MOUSE tr|S4R1H5|S4R1H5\_MOUSE sp|Q6NXK2-2|ZN532\_MOUSE tr|A2AD32|A2AD32\_MOUSE sp|Q8VIM0|HAVR2\_MOUSE sp|Q3UIL6-2|PKHA7\_MOUSE sp|Q3UIL6-4|PKHA7\_MOUSE sp|Q6PGF3|MED16\_MOUSE sp|Q3UIL6-6|PKHA7\_MOUSE sp|Q3UIL6-5|PKHA7\_MOUSE sp|Q3UIL6-3|PKHA7\_MOUSE tr|G3UW74|G3UW74\_MOUSE tr|E9QP84|E9QP84\_MOUSE sp|Q3UIL6|PKHA7\_MOUSE sp|Q3UEB3-2|PUF60\_MOUSE sp|Q3UEB3-3|PUF60\_MOUSE sp|Q3UEB3|PUF60\_MOUSE sp|Q8C147|DOCK8\_MOUSE sp|Q8BJQ9|CGAT1\_MOUSE tr|L7N1W8|L7N1W8\_MOUSE tr|F7D4U1|F7D4U1\_MOUSE tr|J3QK40|J3QK40\_MOUSE sp|Q3TJ91|L2GL2\_MOUSE tr|J3QJU5|J3QJU5\_MOUSE sp|B8ZXI1|QTRD1\_MOUSE tr|L7N264|L7N264\_MOUSE sp|O70305-2|ATX2\_MOUSE sp|O70305|ATX2\_MOUSE sp|O70305-3|ATX2\_MOUSE tr|E9QM77|E9QM77\_MOUSE sp|Q3UWW6|GA2L3\_MOUSE sp|P97737|BMP3B\_MOUSE sp|D3YU81|RFX8\_MOUSE tr|D6RFT4|D6RFT4\_MOUSE Q3T052 sp|Q9D684-2|RIN2\_MOUSE sp|Q9D684|RIN2\_MOUSE tr|F8WGD2|F8WGD2\_MOUSE tr|D3Z337|D3Z337\_MOUSE sp|P36423|THAS\_MOUSE tr|E9Q7X8|E9Q7X8\_MOUSE sp|Q9WTW5|S22A3\_MOUSE tr|D3Z4F0|D3Z4F0\_MOUSE tr|D3Z1K9|D3Z1K9\_MOUSE tr|D3YVL2|D3YVL2\_MOUSE tr|Q5M6W0|Q5M6W0\_MOUSE sp|Q80TG1-2|KANL1\_MOUSE sp|Q5NCM1|S17A4\_MOUSE tr|A0A044PY53|A0A044PY53\_MOUSE sp|O08538|ANGP1\_MOUSE tr|Q3UED7|Q3UED7\_MOUSE sp|Q91YN0|CL004\_MOUSE sp|Q3THK3|T2FA\_MOUSE sp|Q3U5F4|YRDC\_MOUSE sp|Q6ZWR6-5|SYNE1\_MOUSE sp|Q9CWF6|BBS2\_MOUSE tr|F6R220|F6R220\_MOUSE sp|Q8BW49|TTC12\_MOUSE sp|Q7TPW1|NEXN\_MOUSE tr|E9QPQ1|E9QPQ1\_MOUSE tr|F6TRD7|F6TRD7\_MOUSE tr|B2RXW8|B2RXW8\_MOUSE sp|Q9JMB7|PIWL1\_MOUSE sp|Q9JMB7-2|PIWL1\_MOUSE tr|Q3UDF0|Q3UDF0\_MOUSE sp|P57757|CTNS\_MOUSE tr|A2AR26|A2AR26\_MOUSE sp|A8C756-2|THADA\_MOUSE tr|V9GXF0|V9GXF0\_MOUSE tr|V9GXP8|V9GXP8\_MOUSE tr|F8VPM7|F8VPM7\_MOUSE sp|Q99MI1-2|RB6I2\_MOUSE sp|Q99MI1|RB6I2\_MOUSE tr|V9GWT6|V9GWT6\_MOUSE tr|V9GXH3|V9GXH3\_MOUSE sp|Q9CQN7|RM41\_MOUSE sp|Q08274|DMWD\_MOUSE sp|Q9QUR6|PPCE\_MOUSE sp|Q64727|VINC\_MOUSE tr|E0CZ12|E0CZ12\_MOUSE tr|G3UXN3|G3UXN3\_MOUSE tr|G3UZP3|G3UZP3\_MOUSE tr|E9Q507|E9Q507\_MOUSE tr|K4DI71|K4DI71\_MOUSE sp|Q9DB00|GON4L\_MOUSE sp|Q60591|NFAC2\_MOUSE sp|Q60591-2|NFAC2\_MOUSE sp|P62814|VATB2\_MOUSE sp|Q80Y50-5|CMTA2\_MOUSE sp|Q80Y50-3|CMTA2\_MOUSE sp|Q80Y50-2|CMTA2\_MOUSE sp|Q80Y50-4|CMTA2\_MOUSE tr|B0QZH6|B0QZH6\_MOUSE tr|B0QZH4|B0QZH4\_MOUSE sp|Q80Y50|CMTA2\_MOUSE tr|E9PZA6|E9PZA6\_MOUSE sp|O89032-2|SPD2A\_MOUSE sp|O89032-3|SPD2A\_MOUSE sp|O89032|SPD2A\_MOUSE sp|Q7TPD1-3|FBX11\_MOUSE sp|Q7TPD1-2|FBX11\_MOUSE tr|F6T356|F6T356\_MOUSE sp|Q7TPD1|FBX11\_MOUSE tr|A0A087WPR9|A0A087WPR9\_MOUSE tr|D3YWB5|D3YWB5\_MOUSE sp|Q8C854|MYEF2\_MOUSE sp|Q7TN08|DACT2\_MOUSE sp|Q9Z0D9|CX3C1\_MOUSE sp|A0AUP1|CC112\_MOUSE tr|D3Z1J2|D3Z1J2\_MOUSE sp|Q09XV5|CHD8\_MOUSE tr|F7B2U5|F7B2U5\_MOUSE tr|D6RGR0|D6RGR0\_MOUSE sp|Q9ESK3|CAN10\_MOUSE sp|Q9R1M5-6|NALP5\_MOUSE sp|Q9R1M5-2|NALP5\_MOUSE sp|Q9R1M5-7|NALP5\_MOUSE sp|Q9R1M5-3|NALP5\_MOUSE sp|Q9R1M5-5|NALP5\_MOUSE sp|Q9R1M5-4|NALP5\_MOUSE tr|F6XYF6|F6XYF6\_MOUSE sp|Q9R1M5|NALP5\_MOUSE sp|Q9JJ57-2|KCIP1\_MOUSE sp|Q9JJ57-3|KCIP1\_MOUSE sp|Q9JJ57-4|KCIP1\_MOUSE sp|Q9JJ57|KCIP1\_MOUSE sp|Q9R0K7|AT2B2\_MOUSE tr|S4R1C4|S4R1C4\_MOUSE sp|Q6ZQH8|NU188\_MOUSE tr|F8WHB1|F8WHB1\_MOUSE sp|Q64HY3|SP9\_MOUSE sp|Q8K4E0|ALMS1\_MOUSE tr|A2ALB3|A2ALB3\_MOUSE sp|O08786|CCKAR\_MOUSE sp|Q8BXX2-2|ZBT49\_MOUSE tr|F8VQ45|F8VQ45\_MOUSE sp|Q7TQ65|TMC4\_MOUSE sp|Q7TQ65-3|TMC4\_MOUSE tr|D3YWB3|D3YWB3\_MOUSE sp|Q91XU3|PI42C\_MOUSE sp|Q3U3I9|ZN865\_MOUSE sp|Q6ZPJ0|TEX2\_MOUSE tr|A2AQN0|A2AQN0\_MOUSE tr|Q8C9D0|Q8C9D0\_MOUSE tr|E9PZ22|E9PZ22\_MOUSE sp|P97767-2|EYA1\_MOUSE sp|Q9EQF2|KELL\_MOUSE tr|G3UVV6|G3UVV6\_MOUSE sp|P97767|EYA1\_MOUSE sp|Q8VEM8|MPCP\_MOUSE sp|P56564|EAA1\_MOUSE tr|F7D418|F7D418\_MOUSE sp|Q6ZQ12|NINL\_MOUSE sp|Q6ZQ12-1|NINL\_MOUSE sp|Q6ZQ12-2|NINL\_MOUSE sp|Q7M725|TR107\_MOUSE sp|Q14AM7|CP072\_MOUSE tr|Q1WWJ5|Q1WWJ5\_MOUSE tr|Q9D2V6|Q9D2V6\_MOUSE sp|Q60825|NPT2A\_MOUSE sp|Q8BYH7|TBC17\_MOUSE tr|S4R1S7|S4R1S7\_MOUSE sp|Q9QYE6|GOGA5\_MOUSE tr|D3Z4M1|D3Z4M1\_MOUSE sp|Q9JM99-4|PRG4\_MOUSE sp|P04224|HA22\_MOUSE sp|P14439|HA23\_MOUSE sp|Q9CRB9|MIC19\_MOUSE tr|D3Z0L4|D3Z0L4\_MOUSE sp|Q62413|EPHA6\_MOUSE sp|Q0GGX2-2|ZN541\_MOUSE tr|Q9D9P1|Q9D9P1\_MOUSE sp|Q8VI47|MRP2\_MOUSE sp|P39098-2|MA1A2\_MOUSE sp|P39098|MA1A2\_MOUSE tr|D3Z6W3|D3Z6W3\_MOUSE sp|Q9CPN8|IF2B3\_MOUSE sp|Q8BRG8-2|TM209\_MOUSE tr|F8WGT2|F8WGT2\_MOUSE sp|Q91ZU9|NMD3B\_MOUSE sp|Q8BRG8-3|TM209\_MOUSE sp|Q8BRG8|TM209\_MOUSE tr|D3YZY3|D3YZY3\_MOUSE tr|D3Z130|D3Z130\_MOUSE tr|F6TUN6|F6TUN6\_MOUSE sp|P59111|KCNH8\_MOUSE sp|Q5SV80|MYO19\_MOUSE sp|Q8VDS7-1|CE57L\_MOUSE tr|G5E828|G5E828\_MOUSE tr|Q9DAV5|Q9DAV5\_MOUSE tr|S4R1D2|S4R1D2\_MOUSE tr|Q9D2U0|Q9D2U0\_MOUSE sp|O54782|MA2B2\_MOUSE sp|Q8CGW4|SOX30\_MOUSE tr|F7ARK3|F7ARK3\_MOUSE sp|Q60769|TNAP3\_MOUSE sp|Q9QX96|SALL2\_MOUSE sp|P29477|NOS2\_MOUSE tr|H3BKP5|H3BKP5\_MOUSE tr|D3Z6L3|D3Z6L3\_MOUSE tr|Q5NCB2|Q5NCB2\_MOUSE tr|O35192|O35192\_MOUSE tr|D3Z1K8|D3Z1K8\_MOUSE tr|G8JL83|G8JL83\_MOUSE tr|E9Q4R3|E9Q4R3\_MOUSE tr|F7BDN4|F7BDN4\_MOUSE sp|O88968|TCO2\_MOUSE tr|F7BGZ6|F7BGZ6\_MOUSE sp|Q8BQT2|FA69C\_MOUSE sp|Q9WV07|LOXE3\_MOUSE tr|E9PW39|E9PW39\_MOUSE sp|Q8R3J4|MTEF3\_MOUSE sp|Q6NZP2|GPBL1\_MOUSE P34955 sp|Q5GAM9|RNS11\_MOUSE sp|Q8C8U0|LIPB1\_MOUSE sp|A2A891|CMTA1\_MOUSE sp|A2A891-2|CMTA1\_MOUSE sp|A2A891-5|CMTA1\_MOUSE sp|Q9CXH7|SGOL1\_MOUSE tr|D3YVP5|D3YVP5\_MOUSE sp|Q80W21|GSTM7\_MOUSE tr|E9QAC8|E9QAC8\_MOUSE tr|D3YVP6|D3YVP6\_MOUSE tr|Q1HL35|Q1HL35\_MOUSE tr|E9Q1U8|E9Q1U8\_MOUSE sp|Q9JKX3|TFR2\_MOUSE sp|Q9JKX3-3|TFR2\_MOUSE tr|D3YY21|D3YY21\_MOUSE tr|D3YWJ0|D3YWJ0\_MOUSE sp|Q80WC3-3|TNC18\_MOUSE sp|Q99PL5|RRBP1\_MOUSE sp|Q8BHH1|AQP11\_MOUSE tr|A2AVJ7|A2AVJ7\_MOUSE tr|D3Z015|D3Z015\_MOUSE tr|E9Q7L6|E9Q7L6\_MOUSE sp|Q9EQT6|SYT13\_MOUSE sp|P28667|MRP\_MOUSE tr|F7CUU8|F7CUU8\_MOUSE sp|P14234|FGR\_MOUSE tr|H3BLN8|H3BLN8\_MOUSE tr|Q8C2Q7|Q8C2Q7\_MOUSE sp|O35737|HNRH1\_MOUSE tr|Z4YN28|Z4YN28\_MOUSE sp|A2AKB9-2|DCA10\_MOUSE tr|E9QLU9|E9QLU9\_MOUSE tr|E9QKY4|E9QKY4\_MOUSE sp|Q80UZ0|FGD5\_MOUSE tr|A7TU71|A7TU71\_MOUSE sp|A2ALU4|SHRM2\_MOUSE tr|A2AQT8|A2AQT8\_MOUSE tr|F8WID5|F8WID5\_MOUSE tr|B7ZNL3|B7ZNL3\_MOUSE tr|E9Q448|E9Q448\_MOUSE sp|Q80XA6|REPS2\_MOUSE sp|Q8BHB0|NOD1\_MOUSE tr|E9PZX1|E9PZX1\_MOUSE sp|Q9DCB4-9|ARP21\_MOUSE tr|E9Q4A0|E9Q4A0\_MOUSE sp|B9EHT4|CLIP3\_MOUSE sp|Q07113|MPRI\_MOUSE tr|G3UYF7|G3UYF7\_MOUSE tr|J3QQ27|J3QQ27\_MOUSE tr|Q3TJI8|Q3TJI8\_MOUSE tr|F2Z3U6|F2Z3U6\_MOUSE sp|P50172|DHI1\_MOUSE sp|Q91Z58|CF132\_MOUSE sp|Q6ZPG2|WDR90\_MOUSE tr|A2RS43|A2RS43\_MOUSE tr|H7BX49|H7BX49\_MOUSE tr|E9Q6T9|E9Q6T9\_MOUSE tr|E9Q7Q2|E9Q7Q2\_MOUSE tr|A2AJX0|A2AJX0\_MOUSE tr|D3Z5Y0|D3Z5Y0\_MOUSE sp|P15919|RAG1\_MOUSE sp|Q8K2W3|TXD11\_MOUSE tr|F7AYJ4|F7AYJ4\_MOUSE sp|Q9DAW9|CNN3\_MOUSE sp|Q5SF07|IF2B2\_MOUSE sp|Q3UPF5-2|ZCCHV\_MOUSE sp|Q3UPF5|ZCCHV\_MOUSE tr|D3Z5I1|D3Z5I1\_MOUSE tr|Q3U5D0|Q3U5D0\_MOUSE sp|Q80TL0|PPM1E\_MOUSE tr|E9QNZ5|E9QNZ5\_MOUSE sp|Q5XJV6|LMTK3\_MOUSE sp|Q71B07|D19L3\_MOUSE tr|A2AIX1|A2AIX1\_MOUSE tr|E9QAT4|E9QAT4\_MOUSE tr|Q497L9|Q497L9\_MOUSE sp|Q8K4R9|DLGP5\_MOUSE sp|Q8K4R9-3|DLGP5\_MOUSE sp|Q8K4R9-2|DLGP5\_MOUSE sp|Q6PAJ1|BCR\_MOUSE sp|Q8K2X1|AT10D\_MOUSE sp|Q8K2X1-2|AT10D\_MOUSE tr|E9Q5C9|E9Q5C9\_MOUSE sp|Q8CBH5|MFSD6\_MOUSE sp|D4PHA7-2|WSCD2\_MOUSE tr|G3UX50|G3UX50\_MOUSE sp|Q80TJ1-2|CAPS1\_MOUSE sp|P05201|AATC\_MOUSE sp|Q8VDU5|SNRK\_MOUSE tr|S4R268|S4R268\_MOUSE sp|Q99M28|RNPS1\_MOUSE sp|Q7TSG2|CTDP1\_MOUSE tr|Q91YH6|Q91YH6\_MOUSE sp|Q6PDI5|ECM29\_MOUSE tr|A2ALV7|A2ALV7\_MOUSE sp|Q6PDI5-2|ECM29\_MOUSE sp|P60229|EIF3E\_MOUSE sp|Q64338|PDE1C\_MOUSE tr|V9GXI9|V9GXI9\_MOUSE tr|Q8VGA9|Q8VGA9\_MOUSE sp|Q8BQM9-2|MD12L\_MOUSE sp|P46735|MYO1B\_MOUSE tr|Q8K2A4|Q8K2A4\_MOUSE tr|E9Q580|E9Q580\_MOUSE sp|P46735-2|MYO1B\_MOUSE sp|Q61549|EMR1\_MOUSE tr|Q7TQD7|Q7TQD7\_MOUSE tr|E9QNH6|E9QNH6\_MOUSE sp|Q8K2I2|CCHCR\_MOUSE tr|G3UWS7|G3UWS7\_MOUSE tr|A2AS44|A2AS44\_MOUSE tr|D3Z2C5|D3Z2C5\_MOUSE sp|P56818|BACE1\_MOUSE tr|E0CXB6|E0CXB6\_MOUSE tr|F6U6U5|F6U6U5\_MOUSE sp|Q60649|CLPB\_MOUSE tr|E9PY58|E9PY58\_MOUSE tr|E9PVK4|E9PVK4\_MOUSE sp|Q0HA38|TT21B\_MOUSE sp|Q8R4I7|NETO1\_MOUSE sp|O54827|AT10A\_MOUSE sp|P48281|VDR\_MOUSE sp|Q8VHK9|DHX36\_MOUSE tr|B0V2M9|B0V2M9\_MOUSE sp|Q8BHP2|NUTM1\_MOUSE sp|Q80SU7|GVIN1\_MOUSE sp|Q80U87|UBP8\_MOUSE sp|Q8R007|PVRL4\_MOUSE tr|F6W9D6|F6W9D6\_MOUSE sp|Q8R007-2|PVRL4\_MOUSE sp|Q99NH8-2|TREM2\_MOUSE sp|Q8BSP2-2|CNDH2\_MOUSE sp|Q3UMR5-2|MCU\_MOUSE tr|E9QAU9|E9QAU9\_MOUSE sp|Q3UMR5|MCU\_MOUSE sp|Q8BSP2|CNDH2\_MOUSE sp|Q8BSP2-3|CNDH2\_MOUSE tr|B2RXP1|B2RXP1\_MOUSE tr|E9Q892|E9Q892\_MOUSE sp|Q8BVU0|LRCH3\_MOUSE tr|B7ZWM8|B7ZWM8\_MOUSE sp|Q148W0|AT8B1\_MOUSE tr|E9PY48|E9PY48\_MOUSE sp|Q921F2|TADBP\_MOUSE tr|E9PY61|E9PY61\_MOUSE sp|Q8BHL6|FAP24\_MOUSE tr|Q8VFT2|Q8VFT2\_MOUSE sp|P59941|SIR6\_MOUSE sp|Q80Y19|RHGBA\_MOUSE tr|Q3UKP1|Q3UKP1\_MOUSE sp|Q6PAM1|TXLNA\_MOUSE tr|E9Q5M4|E9Q5M4\_MOUSE tr|E9Q711|E9Q711\_MOUSE tr|E9Q1T6|E9Q1T6\_MOUSE sp|Q9D4I2-3|MEII1\_MOUSE sp|Q9D4I2|MEII1\_MOUSE sp|Q2EY15|PRTG\_MOUSE tr|E9PVA2|E9PVA2\_MOUSE sp|B9EJV3|GRB1L\_MOUSE sp|P39061-2|COIA1\_MOUSE sp|Q5F2E7-2|NUFP2\_MOUSE sp|Q5F2E7|NUFP2\_MOUSE tr|D3YUW2|D3YUW2\_MOUSE sp|Q8R054|SRPX2\_MOUSE sp|Q8C7E7|STBD1\_MOUSE sp|Q9CRD2|EMC2\_MOUSE sp|Q8VHB5|CAH9\_MOUSE tr|E9PV80|E9PV80\_MOUSE sp|Q61026|NCOA2\_MOUSE sp|Q9D1H7|GET4\_MOUSE sp|Q8BYI6|PCAT2\_MOUSE sp|Q8BYI6-2|PCAT2\_MOUSE sp|Q9QXN0|SHRM3\_MOUSE sp|Q9QXN0-2|SHRM3\_MOUSE tr|E9PYG6|E9PYG6\_MOUSE sp|Q8VCZ8|RUSD1\_MOUSE sp|Q64455|PTPRJ\_MOUSE tr|A2AWF8|A2AWF8\_MOUSE tr|A2AWF9|A2AWF9\_MOUSE tr|E9Q4S7|E9Q4S7\_MOUSE tr|B1AW21|B1AW21\_MOUSE tr|F6YNQ1|F6YNQ1\_MOUSE sp|Q9Z2C5|MTM1\_MOUSE sp|P23818|GRIA1\_MOUSE sp|Q9R1M5-8|NALP5\_MOUSE sp|Q9WUU6|HNF4G\_MOUSE sp|Q8K214-2|SCMH1\_MOUSE sp|Q8K214|SCMH1\_MOUSE tr|E9QPS1|E9QPS1\_MOUSE sp|Q80TE7|LRRC7\_MOUSE sp|Q91W52-2|TMM19\_MOUSE tr|F6ZQA3|F6ZQA3\_MOUSE sp|Q99MB2|MTFR1\_MOUSE sp|Q8C5W3|TBCEL\_MOUSE sp|Q8C5W3-2|TBCEL\_MOUSE tr|H3BK01|H3BK01\_MOUSE sp|Q8R2K4|TAF6L\_MOUSE tr|K3W4L1|K3W4L1\_MOUSE sp|Q60875-3|ARHG2\_MOUSE sp|Q60875-2|ARHG2\_MOUSE sp|Q62240-2|KDM5D\_MOUSE sp|Q8JZN5|ACAD9\_MOUSE sp|Q62240|KDM5D\_MOUSE tr|A2AQM9|A2AQM9\_MOUSE sp|Q920C1|CRDL1\_MOUSE sp|Q920C1-2|CRDL1\_MOUSE tr|J3QPR1|J3QPR1\_MOUSE sp|P54254|ATX1\_MOUSE tr|Q5ND37|Q5ND37\_MOUSE tr|B1AVB5|B1AVB5\_MOUSE tr|B9EJ86|B9EJ86\_MOUSE tr|G3X9N6|G3X9N6\_MOUSE tr|B1AVB3|B1AVB3\_MOUSE tr|B1AVB2|B1AVB2\_MOUSE sp|Q7TSH2|KPBB\_MOUSE sp|Q91Z83|MYH7\_MOUSE tr|F6U8T2|F6U8T2\_MOUSE sp|Q80W31|ZN569\_MOUSE sp|Q8K0Q5|RHG18\_MOUSE tr|F6QD74|F6QD74\_MOUSE sp|Q60595|XL3A\_MOUSE sp|Q80U16|FA65B\_MOUSE sp|O08532-2|CA2D1\_MOUSE sp|O08532-5|CA2D1\_MOUSE sp|Q9DCB4-4|ARP21\_MOUSE sp|Q9DCB4-3|ARP21\_MOUSE tr|F7D425|F7D425\_MOUSE tr|E9Q1X8|E9Q1X8\_MOUSE sp|Q9DCB4|ARP21\_MOUSE sp|Q9DCB4-5|ARP21\_MOUSE sp|O08532|CA2D1\_MOUSE tr|S4R1G5|S4R1G5\_MOUSE sp|P59470|RPC2\_MOUSE sp|Q61809|LRRN1\_MOUSE tr|D6RG52|D6RG52\_MOUSE tr|F7AWK3|F7AWK3\_MOUSE tr|Q3ZB60|Q3ZB60\_MOUSE tr|A0A087WPR1|A0A087WPR1\_MOUSE sp|P20917-2|MAG\_MOUSE sp|P20917|MAG\_MOUSE sp|Q32Q92|ACOT6\_MOUSE sp|Q8BXQ3|LRTM1\_MOUSE sp|Q9JL70|FANCA\_MOUSE tr|F7CK17|F7CK17\_MOUSE sp|Q8BVP5|KC1G2\_MOUSE tr|Q99K78|Q99K78\_MOUSE tr|A2ASU7|A2ASU7\_MOUSE sp|P70315|WASP\_MOUSE tr|E9Q190|E9Q190\_MOUSE sp|Q91Z46|DUS7\_MOUSE sp|Q91Z46-2|DUS7\_MOUSE sp|Q80UE6|WNK4\_MOUSE tr|A2AQ41|A2AQ41\_MOUSE sp|Q80TY0|FNBP1\_MOUSE sp|Q80TY0-4|FNBP1\_MOUSE tr|F6R4N2|F6R4N2\_MOUSE tr|E9QAD0|E9QAD0\_MOUSE sp|Q99N32|KLOTB\_MOUSE sp|Q9EPW0-2|INP4A\_MOUSE sp|Q5FWI3|TMEM2\_MOUSE sp|Q03137|EPHA4\_MOUSE sp|Q03137-2|EPHA4\_MOUSE sp|Q9R092|H17B6\_MOUSE sp|Q61704|ITIH3\_MOUSE tr|E9PVS1|E9PVS1\_MOUSE tr|A0A087WQ16|A0A087WQ16\_MOUSE tr|E9QPY6|E9QPY6\_MOUSE sp|P70388-2|RAD50\_MOUSE sp|P70388|RAD50\_MOUSE tr|D3Z495|D3Z495\_MOUSE tr|D3YTL8|D3YTL8\_MOUSE sp|Q52KF3|SPIR1\_MOUSE sp|Q52KF3-2|SPIR1\_MOUSE sp|Q6VH22|IF172\_MOUSE tr|Q5SV02|Q5SV02\_MOUSE sp|Q52KF3-3|SPIR1\_MOUSE tr|L7N290|L7N290\_MOUSE sp|Q3USW5|FXRD2\_MOUSE tr|F8VQ52|F8VQ52\_MOUSE sp|Q01341|ADCY6\_MOUSE sp|Q8C0Q9|RPGF5\_MOUSE tr|J3QNF6|J3QNF6\_MOUSE sp|P20443|ARRS\_MOUSE sp|Q8VI63|MOB2\_MOUSE sp|Q9WVG5|LIPE\_MOUSE sp|P63166|SUMO1\_MOUSE sp|Q8VI63-2|MOB2\_MOUSE tr|A0A087WRQ4|A0A087WRQ4\_MOUSE tr|Q8C782|Q8C782\_MOUSE tr|Q8CC38|Q8CC38\_MOUSE tr|B1ATS5|B1ATS5\_MOUSE sp|Q64518|AT2A3\_MOUSE tr|O08643|O08643\_MOUSE sp|Q9Z0H4-6|CELF2\_MOUSE sp|Q9Z0H4-2|CELF2\_MOUSE sp|Q920Q2|REV1\_MOUSE sp|Q9WV80|SNX1\_MOUSE sp|Q9DA79|DPEP3\_MOUSE tr|F6UP77|F6UP77\_MOUSE sp|Q9D7Z6|CLCA1\_MOUSE sp|Q8BGA7|CG026\_MOUSE sp|Q9CQT1|MTNA\_MOUSE sp|Q6NZN1-2|PPRC1\_MOUSE sp|Q6NZN1|PPRC1\_MOUSE sp|Q6NZN1-5|PPRC1\_MOUSE tr|E9QPX1|E9QPX1\_MOUSE sp|P39061|COIA1\_MOUSE sp|P39061-1|COIA1\_MOUSE tr|A0A087WRT5|A0A087WRT5\_MOUSE sp|Q6NS59|F135A\_MOUSE tr|A0A087WP54|A0A087WP54\_MOUSE sp|P70453-2|PDE7A\_MOUSE tr|E9QNC6|E9QNC6\_MOUSE tr|G5E8Y2|G5E8Y2\_MOUSE sp|Q3UMB5|SMCR8\_MOUSE sp|Q3UMB5-2|SMCR8\_MOUSE sp|Q8C0S4|TT21A\_MOUSE tr|Q8CGT2|Q8CGT2\_MOUSE sp|Q9R016|BIR1E\_MOUSE tr|Q99NH4|Q99NH4\_MOUSE tr|Q8C833|Q8C833\_MOUSE sp|P70218|M4K1\_MOUSE tr|Q0VBD0|Q0VBD0\_MOUSE sp|Q9QZC2|PLXC1\_MOUSE tr|B9EHI3|B9EHI3\_MOUSE tr|A0A087WS26|A0A087WS26\_MOUSE sp|Q8VG04|OL478\_MOUSE tr|B1ARD6|B1ARD6\_MOUSE tr|Q14AF1|Q14AF1\_MOUSE sp|Q2TBE6|P4K2A\_MOUSE tr|D3YY41|D3YY41\_MOUSE sp|P42337|PK3CA\_MOUSE sp|Q9WUT3|KS6A2\_MOUSE tr|E9PYF5|E9PYF5\_MOUSE sp|P42859-2|HD\_MOUSE sp|Q9JMB8|CNTN6\_MOUSE sp|Q9JMB8-2|CNTN6\_MOUSE tr|E0CXE3|E0CXE3\_MOUSE sp|O35633|VIAAT\_MOUSE sp|O35633-2|VIAAT\_MOUSE sp|Q8K3W3|CASC3\_MOUSE sp|Q9JM99|PRG4\_MOUSE tr|F7CVJ5|F7CVJ5\_MOUSE tr|Q52KC3|Q52KC3\_MOUSE sp|P49718|MCM5\_MOUSE sp|Q7TMI3-2|UHRF2\_MOUSE sp|Q6NZN0|RBM26\_MOUSE sp|Q6NZN0-5|RBM26\_MOUSE sp|Q6NZN0-2|RBM26\_MOUSE sp|Q6NZN0-4|RBM26\_MOUSE sp|Q6NZN0-3|RBM26\_MOUSE tr|E9Q640|E9Q640\_MOUSE tr|E9PUF4|E9PUF4\_MOUSE sp|Q8BZH4|POGZ\_MOUSE tr|Q0VGT3|Q0VGT3\_MOUSE tr|E9PUE8|E9PUE8\_MOUSE sp|P70170-2|ABCC9\_MOUSE sp|P70170-3|ABCC9\_MOUSE sp|P70170|ABCC9\_MOUSE sp|Q8R2Z5|VWA1\_MOUSE tr|F8WHB9|F8WHB9\_MOUSE tr|E9Q993|E9Q993\_MOUSE sp|Q2VPD4|BMAL2\_MOUSE sp|Q68FD9|K1549\_MOUSE tr|D3YTS3|D3YTS3\_MOUSE tr|E9Q0I4|E9Q0I4\_MOUSE sp|Q8CFW1|ANO2\_MOUSE sp|Q9ES00|UBE4B\_MOUSE sp|Q80Z25|OFD1\_MOUSE tr|A2AEG2|A2AEG2\_MOUSE sp|A3KGK3-2|FR1L4\_MOUSE sp|A3KGK3|FR1L4\_MOUSE tr|Z4YL23|Z4YL23\_MOUSE sp|Q9CS00|CATIN\_MOUSE sp|Q9ESN3|TMM8A\_MOUSE sp|Q9ESN3-2|TMM8A\_MOUSE sp|Q91WU4-2|TMCO4\_MOUSE sp|Q91WU4|TMCO4\_MOUSE sp|Q6ZQ29|TAOK2\_MOUSE tr|Q3V360|Q3V360\_MOUSE sp|Q69Z38|PEAK1\_MOUSE sp|P57774|NPY\_MOUSE tr|F7AA26|F7AA26\_MOUSE tr|A2A483|A2A483\_MOUSE tr|E9Q8D1|E9Q8D1\_MOUSE tr|A2A484|A2A484\_MOUSE tr|Q6NXM6|Q6NXM6\_MOUSE tr|Z4YJE9|Z4YJE9\_MOUSE sp|B8ZXI1-2|QTRD1\_MOUSE tr|F6WNE9|F6WNE9\_MOUSE tr|E9QAF4|E9QAF4\_MOUSE tr|E9Q0Z3|E9Q0Z3\_MOUSE sp|Q8BFR1|ZCCHL\_MOUSE sp|Q91VM3-2|WIPI4\_MOUSE tr|F7CAT9|F7CAT9\_MOUSE tr|F6YHS5|F6YHS5\_MOUSE sp|Q91VM3|WIPI4\_MOUSE tr|F6Q8K5|F6Q8K5\_MOUSE tr|A2AEV2|A2AEV2\_MOUSE sp|Q62406-2|IRAK1\_MOUSE tr|D3YYC3|D3YYC3\_MOUSE tr|G5E8N3|G5E8N3\_MOUSE sp|Q80XE1|RIC8B\_MOUSE sp|Q2TPA8|HSDL2\_MOUSE sp|Q8BRH0-2|TMTC3\_MOUSE tr|Q8K1M3|Q8K1M3\_MOUSE sp|Q3TKY6|CWC27\_MOUSE sp|Q3U108-3|ARI5A\_MOUSE tr|D3Z7U1|D3Z7U1\_MOUSE sp|Q70IV5-2|SYNEM\_MOUSE tr|F7B6K4|F7B6K4\_MOUSE sp|Q62209|SYCP1\_MOUSE tr|G3X8R7|G3X8R7\_MOUSE sp|Q9CPT0|B2L14\_MOUSE tr|D3YZF2|D3YZF2\_MOUSE sp|A2A5R2|BIG2\_MOUSE sp|Q7TM95|TIGD3\_MOUSE sp|Q8VDD8|WASH1\_MOUSE sp|Q3U319|BRE1B\_MOUSE sp|Q3U2K0-3|F193B\_MOUSE tr|F6Q2Y9|F6Q2Y9\_MOUSE tr|H7BX26|H7BX26\_MOUSE sp|Q4VBD2|TAPT1\_MOUSE tr|Q9CZQ1|Q9CZQ1\_MOUSE sp|Q33DR2|DPS1\_MOUSE tr|E9Q8G5|E9Q8G5\_MOUSE tr|F7B4F0|F7B4F0\_MOUSE sp|Q8VCH8|UBXN4\_MOUSE sp|P04071|K1B16\_MOUSE sp|P07628|K1KB8\_MOUSE sp|Q61136|PRP4B\_MOUSE tr|F8WJC1|F8WJC1\_MOUSE tr|A2AJY5|A2AJY5\_MOUSE tr|B9EJX5|B9EJX5\_MOUSE tr|D3Z7K7|D3Z7K7\_MOUSE tr|D3Z2L9|D3Z2L9\_MOUSE sp|P97390|VPS45\_MOUSE sp|O54991|CNTP1\_MOUSE sp|Q9WTU3-2|SCN8A\_MOUSE tr|F7D6K4|F7D6K4\_MOUSE sp|Q8CCJ9-3|P20L1\_MOUSE sp|E9Q1U1-2|CC171\_MOUSE sp|E9Q1U1|CC171\_MOUSE sp|Q8K285|FCHO1\_MOUSE sp|Q7M6U3-2|TEX14\_MOUSE sp|Q7M6U3|TEX14\_MOUSE sp|B2RY83|HPSE2\_MOUSE sp|P22315|HEMH\_MOUSE tr|Q544X6|Q544X6\_MOUSE sp|P30415|NKTR\_MOUSE sp|Q9JJA9|GRASP\_MOUSE tr|B7XG49|B7XG49\_MOUSE sp|Q99PP2-2|ZN318\_MOUSE tr|B8JJA0|B8JJA0\_MOUSE sp|Q8BI84|MIA3\_MOUSE tr|E9QPJ5|E9QPJ5\_MOUSE sp|Q80U59|K0232\_MOUSE sp|A2A8U2-3|TM201\_MOUSE sp|A2A8U2|TM201\_MOUSE sp|E9PYY5|WDR78\_MOUSE sp|P09079|HXB5\_MOUSE sp|E9PYY5-2|WDR78\_MOUSE tr|I7HFV2|I7HFV2\_MOUSE tr|A0A087WQS5|A0A087WQS5\_MOUSE tr|I7HJI7|I7HJI7\_MOUSE sp|Q9CQR6|PPP6\_MOUSE sp|P56383|AT5G2\_MOUSE tr|Q8VGW6|Q8VGW6\_MOUSE sp|Q69ZB0-2|LRCC1\_MOUSE tr|D3YV76|D3YV76\_MOUSE tr|D3Z3C0|D3Z3C0\_MOUSE sp|P42208|SEPT2\_MOUSE sp|Q69ZB0|LRCC1\_MOUSE sp|P17563|SBP1\_MOUSE tr|D3YZU7|D3YZU7\_MOUSE tr|D3YYB1|D3YYB1\_MOUSE tr|G3UYY2|G3UYY2\_MOUSE tr|D3Z1S1|D3Z1S1\_MOUSE sp|Q63836|SBP2\_MOUSE tr|F6WYM0|F6WYM0\_MOUSE sp|P16882|GHR\_MOUSE sp|Q6P9K8-2|CSKI1\_MOUSE sp|Q6P9K8|CSKI1\_MOUSE tr|F8VQB1|F8VQB1\_MOUSE sp|Q80VP0-2|TCPR1\_MOUSE sp|Q80VP0|TCPR1\_MOUSE sp|Q8BMI0|FBX38\_MOUSE sp|Q69ZS7-3|HBS1L\_MOUSE sp|Q7TSH8|K0195\_MOUSE sp|Q8R2Q0|TRI29\_MOUSE sp|Q9Z206-2|ARHG8\_MOUSE tr|E9Q7C7|E9Q7C7\_MOUSE tr|E9Q5C2|E9Q5C2\_MOUSE tr|Q8C4F4|Q8C4F4\_MOUSE sp|Q60738|ZNT1\_MOUSE tr|E9QK53|E9QK53\_MOUSE tr|G3X9Y5|G3X9Y5\_MOUSE sp|Q9WUK7|OPN3\_MOUSE tr|Q8VGJ9|Q8VGJ9\_MOUSE sp|Q8C080|SNX16\_MOUSE tr|Q3V1K7|Q3V1K7\_MOUSE sp|Q8BXB6|SO2B1\_MOUSE tr|Q3UNZ2|Q3UNZ2\_MOUSE tr|Q8BZN7|Q8BZN7\_MOUSE sp|Q569Z6|TR150\_MOUSE sp|Q65Z40|WAPL\_MOUSE tr|B7ZP47|B7ZP47\_MOUSE tr|Q8VGY7|Q8VGY7\_MOUSE tr|Q68FG2|Q68FG2\_MOUSE sp|Q5SSH7-2|ZZEF1\_MOUSE sp|Q501J6|DDX17\_MOUSE tr|Q3U741|Q3U741\_MOUSE sp|Q5SSH7|ZZEF1\_MOUSE sp|Q8CEF1|FEM1C\_MOUSE sp|Q9R155|S26A4\_MOUSE sp|A2AJT4|PNISR\_MOUSE tr|Q8BHW4|Q8BHW4\_MOUSE sp|Q9DBY0|FOXP4\_MOUSE tr|E9PZR8|E9PZR8\_MOUSE tr|A2AJT5|A2AJT5\_MOUSE sp|Q7TT45|RRAGD\_MOUSE tr|B1AWT2|B1AWT2\_MOUSE tr|B1AWT4|B1AWT4\_MOUSE sp|Q99K70|RRAGC\_MOUSE tr|B1AWT3|B1AWT3\_MOUSE sp|Q8C131|HCAR1\_MOUSE sp|Q3UZV7-3|K132L\_MOUSE sp|Q3UZV7-2|K132L\_MOUSE sp|Q3UZV7|K132L\_MOUSE sp|Q8BRK9-2|MA2A2\_MOUSE sp|Q8CBW4|DC121\_MOUSE tr|D3Z5T8|D3Z5T8\_MOUSE tr|Q148R4|Q148R4\_MOUSE sp|Q8K4Z3|NNRE\_MOUSE sp|Q61739-2|ITA6\_MOUSE tr|Q5F258|Q5F258\_MOUSE sp|Q61739|ITA6\_MOUSE tr|Q3UR91|Q3UR91\_MOUSE tr|Q5R1H8|Q5R1H8\_MOUSE sp|Q80U58-3|PUM2\_MOUSE sp|Q80U58|PUM2\_MOUSE tr|Q3TQ29|Q3TQ29\_MOUSE sp|Q80U58-2|PUM2\_MOUSE sp|P46684|ZIC1\_MOUSE sp|P29755|AGTRB\_MOUSE tr|F6RGI3|F6RGI3\_MOUSE sp|Q8K3P1-5|P2RX2\_MOUSE sp|Q8K3P1-2|P2RX2\_MOUSE sp|Q8K3P1-3|P2RX2\_MOUSE sp|Q8K3P1|P2RX2\_MOUSE tr|D3YXU9|D3YXU9\_MOUSE tr|D3Z4E4|D3Z4E4\_MOUSE sp|Q8BWU3|F131A\_MOUSE tr|B1AWG4|B1AWG4\_MOUSE tr|E9Q214|E9Q214\_MOUSE tr|F6XCT0|F6XCT0\_MOUSE sp|Q62160|GDIR3\_MOUSE tr|Z4YMA7|Z4YMA7\_MOUSE sp|Q3UDP0|WDR41\_MOUSE tr|D3Z6W0|D3Z6W0\_MOUSE sp|Q61143|TRPC6\_MOUSE tr|Q9D5V1|Q9D5V1\_MOUSE tr|H3BL58|H3BL58\_MOUSE sp|P55014-2|S12A1\_MOUSE sp|Q7TSQ8|PDPR\_MOUSE tr|E9Q5N7|E9Q5N7\_MOUSE sp|Q3TDD9-2|PPR21\_MOUSE tr|D6RD07|D6RD07\_MOUSE tr|D3Z7I9|D3Z7I9\_MOUSE tr|D3YZ66|D3YZ66\_MOUSE tr|B1AW74|B1AW74\_MOUSE tr|B1AW75|B1AW75\_MOUSE sp|P14404|EVI1\_MOUSE tr|G3UZT5|G3UZT5\_MOUSE tr|E9Q6I3|E9Q6I3\_MOUSE sp|Q5XJY6|FANCB\_MOUSE tr|E9QMY5|E9QMY5\_MOUSE tr|Q3TEX6|Q3TEX6\_MOUSE sp|Q9ERD6-3|RGPS2\_MOUSE sp|Q9JMH9-6|MY18A\_MOUSE sp|Q8CIF6|SIDT2\_MOUSE P78385 sp|Q8CIF6-2|SIDT2\_MOUSE sp|Q9JMH9|MY18A\_MOUSE tr|E9QAX2|E9QAX2\_MOUSE tr|K3W4L0|K3W4L0\_MOUSE sp|Q9JMH9-4|MY18A\_MOUSE sp|Q99P69-2|NUF2\_MOUSE sp|Q9JMH9-1|MY18A\_MOUSE sp|Q80U57|RIMS3\_MOUSE sp|Q99P69|NUF2\_MOUSE tr|B2RRE2|B2RRE2\_MOUSE tr|F7BJK1|F7BJK1\_MOUSE tr|L7N241|L7N241\_MOUSE tr|Q8CFX3|Q8CFX3\_MOUSE sp|O35607|BMPR2\_MOUSE sp|Q8BKC8-3|PI4KB\_MOUSE sp|Q7TNK1|RFX4\_MOUSE sp|Q8VD65|PI3R4\_MOUSE sp|Q7TNK1-2|RFX4\_MOUSE sp|Q7TNK1-3|RFX4\_MOUSE sp|Q2VIS4|FILA2\_MOUSE tr|E0CXM2|E0CXM2\_MOUSE sp|Q91YI0|ARLY\_MOUSE sp|Q6P9N1|HYCCI\_MOUSE tr|F7D439|F7D439\_MOUSE tr|G3UYX2|G3UYX2\_MOUSE tr|Q3UR97|Q3UR97\_MOUSE sp|Q91W67|UBL7\_MOUSE sp|Q8R3B1|PLCD1\_MOUSE tr|G3UX38|G3UX38\_MOUSE sp|Q3V0F0|RIMB3\_MOUSE tr|E9PZY2|E9PZY2\_MOUSE sp|Q8BMD2|DZIP1\_MOUSE sp|Q8BMD2-3|DZIP1\_MOUSE tr|F6R6A4|F6R6A4\_MOUSE sp|Q8K1N4|SPAS2\_MOUSE sp|Q8BY98|SWAHD\_MOUSE tr|Q3TQ88|Q3TQ88\_MOUSE sp|P17679-2|GATA1\_MOUSE sp|A2AHJ4|BRWD3\_MOUSE sp|P17679|GATA1\_MOUSE tr|A8Y5P0|A8Y5P0\_MOUSE sp|Q64487-8|PTPRD\_MOUSE sp|Q64487-1|PTPRD\_MOUSE sp|Q64487-10|PTPRD\_MOUSE sp|Q64487-11|PTPRD\_MOUSE sp|Q64487|PTPRD\_MOUSE sp|Q64487-9|PTPRD\_MOUSE sp|Q64487-6|PTPRD\_MOUSE sp|Q64487-5|PTPRD\_MOUSE sp|Q64487-7|PTPRD\_MOUSE tr|G3UY75|G3UY75\_MOUSE tr|Q99N17|Q99N17\_MOUSE sp|Q9D8V0|HM13\_MOUSE sp|Q6P4T2|U520\_MOUSE sp|Q9D8V0-3|HM13\_MOUSE sp|Q9D8V0-4|HM13\_MOUSE tr|E9Q796|E9Q796\_MOUSE tr|B1AVB4|B1AVB4\_MOUSE sp|Q8R090|VMAT1\_MOUSE tr|E9Q468|E9Q468\_MOUSE sp|Q3UFD7|FFAR3\_MOUSE tr|G3X9E8|G3X9E8\_MOUSE tr|F6UK53|F6UK53\_MOUSE sp|P42703-2|LIFR\_MOUSE sp|A2AIV2-2|VIR\_MOUSE sp|Q9D2N9|VP33A\_MOUSE sp|P07743|BPIA2\_MOUSE sp|Q91W52|TMM19\_MOUSE tr|K4DI66|K4DI66\_MOUSE tr|H7BX94|H7BX94\_MOUSE sp|Q8R493|ACHB4\_MOUSE tr|Q5F2D9|Q5F2D9\_MOUSE tr|E9PW38|E9PW38\_MOUSE sp|Q91YI4-2|ARRB2\_MOUSE tr|B1AYY3|B1AYY3\_MOUSE sp|Q91YI4|ARRB2\_MOUSE tr|Q505N6|Q505N6\_MOUSE sp|P18653|KS6A1\_MOUSE sp|P97857|ATS1\_MOUSE sp|P05555-2|ITAM\_MOUSE tr|E9Q5K8|E9Q5K8\_MOUSE sp|P05555|ITAM\_MOUSE tr|E9QLJ3|E9QLJ3\_MOUSE tr|Q3U1U4|Q3U1U4\_MOUSE sp|Q6JPI3|MD13L\_MOUSE tr|G5E8F1|G5E8F1\_MOUSE tr|E9Q604|E9Q604\_MOUSE sp|Q91W98|S15A4\_MOUSE tr|A0A087WPC3|A0A087WPC3\_MOUSE sp|Q66JT0|WEE2\_MOUSE sp|Q5DRQ8|FCRLB\_MOUSE sp|Q9EQY0|ERN1\_MOUSE sp|Q8C9W3|ATS2\_MOUSE tr|Q792F9|Q792F9\_MOUSE sp|Q6P9Q4|FHOD1\_MOUSE tr|B1ARU7|B1ARU7\_MOUSE tr|B2RVY8|B2RVY8\_MOUSE sp|Q8R4F0|MCLN3\_MOUSE sp|P63318|KPCG\_MOUSE sp|Q8K243|TRI68\_MOUSE tr|G3X9I6|G3X9I6\_MOUSE sp|Q8CFG0|SULF2\_MOUSE sp|Q6R6I7|RXFP1\_MOUSE sp|Q7TNE1|SUCHY\_MOUSE sp|Q61627|GRID1\_MOUSE sp|Q5SSE9-2|ABCAD\_MOUSE sp|O70318|E41L2\_MOUSE sp|Q8CHP6|PHC3\_MOUSE tr|E9Q368|E9Q368\_MOUSE sp|O88291-3|ZN326\_MOUSE sp|Q9JKJ9|CP39A\_MOUSE tr|D6RG92|D6RG92\_MOUSE sp|O70472|TM131\_MOUSE sp|Q7TNF8-4|RIMB1\_MOUSE sp|Q7TNF8-2|RIMB1\_MOUSE sp|Q7TNF8|RIMB1\_MOUSE tr|Q5NCP6|Q5NCP6\_MOUSE sp|Q5UKY4-9|MO2R3\_MOUSE sp|Q5UKY4|MO2R3\_MOUSE sp|Q5UKY4-3|MO2R3\_MOUSE sp|Q5UKY4-8|MO2R3\_MOUSE sp|Q5UKY4-6|MO2R3\_MOUSE tr|Q0VF76|Q0VF76\_MOUSE sp|Q5UKY4-4|MO2R3\_MOUSE tr|E9Q7T2|E9Q7T2\_MOUSE tr|F6V8M6|F6V8M6\_MOUSE sp|Q8VCC9|SPON1\_MOUSE sp|Q9ESG6|P2Y14\_MOUSE sp|Q8CA95|PDE10\_MOUSE sp|Q8CA95-3|PDE10\_MOUSE sp|Q8CA95-2|PDE10\_MOUSE sp|Q9QUR8|SEM7A\_MOUSE sp|Q60929|MEF2A\_MOUSE tr|G3X987|G3X987\_MOUSE sp|D3YZF7|VS10L\_MOUSE tr|D3Z4D9|D3Z4D9\_MOUSE sp|Q61371|IFT88\_MOUSE tr|G3X9Z7|G3X9Z7\_MOUSE tr|E9QA35|E9QA35\_MOUSE tr|F8WH96|F8WH96\_MOUSE sp|Q9CPS6|HINT3\_MOUSE sp|Q9R0B6|LAMC3\_MOUSE sp|Q8C7U1|N4BP3\_MOUSE tr|A2ATM9|A2ATM9\_MOUSE tr|Q3TLM7|Q3TLM7\_MOUSE tr|Q8CGS6|Q8CGS6\_MOUSE tr|A2AAN2|A2AAN2\_MOUSE sp|Q8BMA6|SRP68\_MOUSE sp|Q61626|GRIK5\_MOUSE sp|Q9JJS6|BCDO1\_MOUSE tr|E9Q321|E9Q321\_MOUSE sp|P23116|EIF3A\_MOUSE tr|H3BLI8|H3BLI8\_MOUSE sp|Q64704-4|STX3\_MOUSE sp|Q64704-6|STX3\_MOUSE sp|Q6P2K3|CC067\_MOUSE sp|Q64704-2|STX3\_MOUSE tr|Q8R1B7|Q8R1B7\_MOUSE sp|Q64704-5|STX3\_MOUSE sp|Q64704|STX3\_MOUSE tr|G3UY45|G3UY45\_MOUSE sp|Q8CGB3|UACA\_MOUSE sp|Q8CGB3-3|UACA\_MOUSE sp|Q8CGB3-2|UACA\_MOUSE tr|Q8VES9|Q8VES9\_MOUSE tr|B1ASB3|B1ASB3\_MOUSE tr|E9PWM9|E9PWM9\_MOUSE tr|E9PVB9|E9PVB9\_MOUSE sp|Q9Z2I9|SUCB1\_MOUSE sp|Q8VEG6-2|CNO6L\_MOUSE sp|Q8VEG6|CNO6L\_MOUSE tr|F6VNP0|F6VNP0\_MOUSE sp|Q8BJM7-3|TYW1\_MOUSE sp|Q8BJM7|TYW1\_MOUSE tr|F6QKE4|F6QKE4\_MOUSE sp|Q3KNY0-4|IGFN1\_MOUSE sp|Q62469|ITA2\_MOUSE sp|Q8C7X2-2|EMC1\_MOUSE tr|Z4YJW0|Z4YJW0\_MOUSE sp|Q8C7X2|EMC1\_MOUSE sp|Q8BI06-3|CEMIP\_MOUSE sp|P16054|KPCE\_MOUSE tr|F6WV69|F6WV69\_MOUSE tr|F6RIQ1|F6RIQ1\_MOUSE sp|Q8BXA5|CLP1L\_MOUSE tr|G3UZ19|G3UZ19\_MOUSE sp|Q69Z98-2|BRSK2\_MOUSE tr|G3UXY1|G3UXY1\_MOUSE tr|G3UYN4|G3UYN4\_MOUSE tr|G3UXM5|G3UXM5\_MOUSE sp|Q69Z98-4|BRSK2\_MOUSE tr|G3UWV4|G3UWV4\_MOUSE sp|P70313|NOS3\_MOUSE sp|Q5DU28|PCX2\_MOUSE tr|D3Z2K6|D3Z2K6\_MOUSE sp|Q4JK59|TET2\_MOUSE sp|Q9JK24|P2R3C\_MOUSE sp|Q8C0T5-2|SI1L1\_MOUSE sp|Q8C0T5|SI1L1\_MOUSE tr|Q80W41|Q80W41\_MOUSE tr|K4DI67|K4DI67\_MOUSE sp|Q6ZQK0|CNDD3\_MOUSE sp|Q14C51|PTCD3\_MOUSE sp|Q7TT79|MCPH1\_MOUSE sp|Q9WVH6|ANGP4\_MOUSE sp|Q811W2|CP26B\_MOUSE tr|H3BKU4|H3BKU4\_MOUSE tr|D3Z7B5|D3Z7B5\_MOUSE sp|Q8JZZ7-2|LPHN2\_MOUSE sp|Q8BWY9|CIP2A\_MOUSE sp|P43027|GDF5\_MOUSE sp|O08811|ERCC2\_MOUSE tr|E9Q6K1|E9Q6K1\_MOUSE tr|F6YA33|F6YA33\_MOUSE tr|F6TMZ3|F6TMZ3\_MOUSE sp|Q9R229|BMP10\_MOUSE tr|Q8BGS5|Q8BGS5\_MOUSE tr|G3X8X0|G3X8X0\_MOUSE tr|J3QK52|J3QK52\_MOUSE sp|Q923Q2|STA13\_MOUSE tr|F8WIY7|F8WIY7\_MOUSE tr|Q3UKR0|Q3UKR0\_MOUSE sp|O35613|DAXX\_MOUSE tr|A2AIS5|A2AIS5\_MOUSE sp|Q8CIM8|INT4\_MOUSE sp|Q7TSK2-2|SEZ6\_MOUSE sp|Q7TSK2|SEZ6\_MOUSE sp|P35576|G6PC\_MOUSE tr|S4R1S3|S4R1S3\_MOUSE tr|Q3SXF8|Q3SXF8\_MOUSE sp|Q8CIQ6|MTR1B\_MOUSE sp|Q148V8|FA83H\_MOUSE tr|G3UXE3|G3UXE3\_MOUSE sp|Q9EQH7-2|NDST3\_MOUSE sp|Q9EQH7|NDST3\_MOUSE sp|P15392|CP2A4\_MOUSE sp|Q9R1S7|MRP6\_MOUSE tr|Q8C288|Q8C288\_MOUSE tr|Q8C4G3|Q8C4G3\_MOUSE sp|O70228|ATP9A\_MOUSE tr|G3X8R5|G3X8R5\_MOUSE tr|A2AQC3|A2AQC3\_MOUSE sp|Q80U72-2|SCRIB\_MOUSE sp|Q80U72|SCRIB\_MOUSE sp|Q9ERV7|PIDD1\_MOUSE sp|P49769-2|PSN1\_MOUSE sp|Q7TPC1|CDSN\_MOUSE sp|P49769|PSN1\_MOUSE sp|P97855|G3BP1\_MOUSE sp|Q8BH01|TMCO3\_MOUSE sp|Q8BH01-2|TMCO3\_MOUSE sp|E9Q3S4|M3K19\_MOUSE tr|E9PYI8|E9PYI8\_MOUSE sp|Q9JMA1|UBP14\_MOUSE sp|P11835|ITB2\_MOUSE tr|M0QWA7|M0QWA7\_MOUSE tr|Q542I8|Q542I8\_MOUSE tr|E9Q2T5|E9Q2T5\_MOUSE tr|E9QLU2|E9QLU2\_MOUSE sp|A7TZF3|SKIT4\_MOUSE sp|Q99MR8|MCCA\_MOUSE tr|A0A087WQV9|A0A087WQV9\_MOUSE sp|P48356-5|LEPR\_MOUSE sp|P48356-3|LEPR\_MOUSE sp|P48356-4|LEPR\_MOUSE sp|P48356|LEPR\_MOUSE sp|P48356-2|LEPR\_MOUSE tr|E9Q3G8|E9Q3G8\_MOUSE sp|Q6ZQF7-2|JADE2\_MOUSE sp|Q8BPM2-2|M4K5\_MOUSE sp|Q6ZQF7|JADE2\_MOUSE tr|A2AA59|A2AA59\_MOUSE sp|Q3U7R1|ESYT1\_MOUSE tr|E9Q046|E9Q046\_MOUSE sp|Q3TWN3-2|CNNM2\_MOUSE tr|F6X9B6|F6X9B6\_MOUSE sp|Q3TWN3|CNNM2\_MOUSE sp|Q8BQT2-2|FA69C\_MOUSE Q95M17 tr|Q9EQW4|Q9EQW4\_MOUSE sp|Q9CPY4|CDKA2\_MOUSE tr|G3UYI1|G3UYI1\_MOUSE tr|Q3UPZ0|Q3UPZ0\_MOUSE tr|A0A087WSS7|A0A087WSS7\_MOUSE tr|F6YHW2|F6YHW2\_MOUSE tr|G5E8J9|G5E8J9\_MOUSE sp|Q8CFE4|SCYL2\_MOUSE sp|Q8BPN8-3|DMXL2\_MOUSE sp|Q8CFE4-2|SCYL2\_MOUSE sp|Q80Z60|ECE2\_MOUSE sp|Q9QY96-2|CASR\_MOUSE sp|Q64487-3|PTPRD\_MOUSE sp|Q80Z60-2|ECE2\_MOUSE sp|O08640|KRA14\_MOUSE sp|Q9QY96|CASR\_MOUSE sp|Q9DCM7|NACC2\_MOUSE sp|Q60902-2|EP15R\_MOUSE sp|Q60902-3|EP15R\_MOUSE sp|Q60902-4|EP15R\_MOUSE sp|Q60902|EP15R\_MOUSE tr|K7N735|K7N735\_MOUSE tr|E9Q2A7|E9Q2A7\_MOUSE tr|J3QQ25|J3QQ25\_MOUSE sp|P23738|DCHS\_MOUSE tr|K7N711|K7N711\_MOUSE tr|E9Q2I4|E9Q2I4\_MOUSE sp|Q9JI57-7|GT2D1\_MOUSE sp|Q9JI57-6|GT2D1\_MOUSE sp|Q9JI57-3|GT2D1\_MOUSE sp|Q9JI57|GT2D1\_MOUSE sp|Q9JI57-2|GT2D1\_MOUSE sp|Q9JI57-8|GT2D1\_MOUSE sp|Q9JI57-9|GT2D1\_MOUSE sp|Q9JI57-5|GT2D1\_MOUSE tr|Q6PD35|Q6PD35\_MOUSE sp|Q9JI57-4|GT2D1\_MOUSE sp|Q9JI57-10|GT2D1\_MOUSE tr|B1ARD8|B1ARD8\_MOUSE tr|K3W4L7|K3W4L7\_MOUSE tr|D3Z2D7|D3Z2D7\_MOUSE sp|Q9DCG6|PBLD1\_MOUSE tr|Q9D4D3|Q9D4D3\_MOUSE sp|Q9CXN7|PBLD2\_MOUSE sp|O08842|GFRA2\_MOUSE sp|Q80TS8|SE1L3\_MOUSE tr|D3YWB4|D3YWB4\_MOUSE sp|Q3UKJ7-2|SMU1\_MOUSE sp|Q3UKJ7|SMU1\_MOUSE tr|D3Z2L2|D3Z2L2\_MOUSE tr|D3YYF1|D3YYF1\_MOUSE sp|Q8VHN7-4|GPR98\_MOUSE sp|Q0V8T7|CTP5C\_MOUSE tr|B2RQ71|B2RQ71\_MOUSE tr|E9PWR4|E9PWR4\_MOUSE sp|Q5SXY1-3|CYTSB\_MOUSE sp|Q5SXY1|CYTSB\_MOUSE tr|Q7TQU7|Q7TQU7\_MOUSE sp|P01904|HA21\_MOUSE sp|O35345|IMA7\_MOUSE tr|Q4FJZ2|Q4FJZ2\_MOUSE sp|Q5ISE2|Z36L3\_MOUSE tr|E9PX14|E9PX14\_MOUSE sp|Q80TH2-2|LAP2\_MOUSE sp|Q80TH2|LAP2\_MOUSE sp|P28359|HXD10\_MOUSE sp|Q61169-2|GATA6\_MOUSE tr|A0A087WQA5|A0A087WQA5\_MOUSE sp|Q6ZQ11|CHSS1\_MOUSE sp|Q62130|PTN14\_MOUSE tr|F6XWD4|F6XWD4\_MOUSE sp|Q3UMT1|PP12C\_MOUSE sp|Q8CHG5|AREL1\_MOUSE tr|E9PY98|E9PY98\_MOUSE tr|E9PZX5|E9PZX5\_MOUSE sp|Q80TS3-3|LPHN3\_MOUSE tr|D3YTW7|D3YTW7\_MOUSE tr|K3W4M8|K3W4M8\_MOUSE sp|Q9QXK9-2|SH22A\_MOUSE sp|Q80TS3-5|LPHN3\_MOUSE tr|D3YVT9|D3YVT9\_MOUSE sp|Q80TS3|LPHN3\_MOUSE tr|D3Z5M6|D3Z5M6\_MOUSE tr|D3Z4S7|D3Z4S7\_MOUSE tr|E0CYE2|E0CYE2\_MOUSE sp|Q8R3Q2|PP6R2\_MOUSE tr|G3X9K4|G3X9K4\_MOUSE tr|F6VMG1|F6VMG1\_MOUSE tr|B1AR42|B1AR42\_MOUSE tr|G3UWA2|G3UWA2\_MOUSE tr|Q6NSU2|Q6NSU2\_MOUSE tr|A0A087WQU7|A0A087WQU7\_MOUSE tr|B1AR44|B1AR44\_MOUSE tr|F6UP63|F6UP63\_MOUSE tr|D3YU33|D3YU33\_MOUSE sp|Q8CHK3|MBOA7\_MOUSE sp|Q4ZJM9|C1QL4\_MOUSE tr|B1AR39|B1AR39\_MOUSE sp|Q66X01|NAL9C\_MOUSE tr|E9PWB6|E9PWB6\_MOUSE sp|Q8BL06|UBP54\_MOUSE sp|P42669|PURA\_MOUSE sp|Q8BW72-2|KDM4A\_MOUSE tr|A2A8L9|A2A8L9\_MOUSE sp|Q8CAE9-3|PDXL2\_MOUSE sp|Q8CAE9|PDXL2\_MOUSE sp|Q8BW72|KDM4A\_MOUSE tr|G3X9D3|G3X9D3\_MOUSE tr|A2AGW7|A2AGW7\_MOUSE sp|Q9R0S2|MMP24\_MOUSE tr|D3YX51|D3YX51\_MOUSE tr|Q8BRF1|Q8BRF1\_MOUSE sp|P24610|PAX3\_MOUSE tr|F6QU68|F6QU68\_MOUSE sp|Q6PFX7|NYAP1\_MOUSE tr|E9PXJ0|E9PXJ0\_MOUSE sp|Q6PFX7-2|NYAP1\_MOUSE sp|Q9Z0H0|CDC7\_MOUSE tr|G3UVV7|G3UVV7\_MOUSE tr|E9Q8F1|E9Q8F1\_MOUSE sp|Q9Z0H0-2|CDC7\_MOUSE sp|Q3UXZ9|KDM5A\_MOUSE tr|Q66JY4|Q66JY4\_MOUSE tr|D3YVB9|D3YVB9\_MOUSE tr|F7BQW7|F7BQW7\_MOUSE tr|D3YVQ3|D3YVQ3\_MOUSE tr|E9Q7S0|E9Q7S0\_MOUSE tr|G3UZ87|G3UZ87\_MOUSE tr|G3UYM7|G3UYM7\_MOUSE tr|G3UY34|G3UY34\_MOUSE sp|Q8BL99-4|DOP1\_MOUSE sp|Q80TM9-6|NISCH\_MOUSE sp|O70585-2|DTNB\_MOUSE sp|Q80TM9-5|NISCH\_MOUSE tr|V9GX99|V9GX99\_MOUSE sp|Q80TM6-3|R3HD2\_MOUSE sp|O70585|DTNB\_MOUSE tr|Q8C8Z9|Q8C8Z9\_MOUSE tr|Q8K0N0|Q8K0N0\_MOUSE sp|Q3UYV9|NCBP1\_MOUSE sp|Q0KK55|VKIND\_MOUSE sp|Q8VCM8|NCLN\_MOUSE tr|D3YU17|D3YU17\_MOUSE sp|Q9DBC3|CMTR1\_MOUSE sp|Q922R5|P4R3B\_MOUSE sp|Q8BJW5|NOL11\_MOUSE tr|O55095|O55095\_MOUSE sp|Q8BJW5-2|NOL11\_MOUSE sp|A4Q9E5-3|TTLL3\_MOUSE sp|O70306|TBX15\_MOUSE sp|Q8R2N2|CIR1A\_MOUSE tr|E9QKZ2|E9QKZ2\_MOUSE sp|Q91YE6|IPO9\_MOUSE sp|Q8R0N6|HOT\_MOUSE tr|A0A087WRE7|A0A087WRE7\_MOUSE tr|Q9Z150|Q9Z150\_MOUSE tr|S4R1U7|S4R1U7\_MOUSE sp|Q80VA5-4|S3TC2\_MOUSE tr|Q3UH07|Q3UH07\_MOUSE sp|Q5DTW2|SMBT2\_MOUSE sp|Q5DTW2-2|SMBT2\_MOUSE tr|Q3UH63|Q3UH63\_MOUSE sp|Q6PDJ1-2|CAHD1\_MOUSE sp|Q6PDJ1|CAHD1\_MOUSE tr|Z4YK18|Z4YK18\_MOUSE sp|Q5SUV2|MYBPP\_MOUSE sp|Q8VI59-2|PCX3\_MOUSE sp|Q8VI59|PCX3\_MOUSE sp|Q9WVK8|CP46A\_MOUSE sp|Q6PH08|ERC2\_MOUSE tr|E9Q123|E9Q123\_MOUSE sp|Q6PH08-3|ERC2\_MOUSE sp|Q9EPU4|CPSF1\_MOUSE tr|E9Q770|E9Q770\_MOUSE tr|D3YTN4|D3YTN4\_MOUSE sp|Q8VCA6|T161A\_MOUSE sp|Q8R4U7|LUZP1\_MOUSE sp|Q7M6Y3-4|PICAL\_MOUSE sp|Q7M6Y3-2|PICAL\_MOUSE sp|Q7M6Y3-3|PICAL\_MOUSE tr|A2A974|A2A974\_MOUSE tr|D3YWS2|D3YWS2\_MOUSE tr|E9Q8T0|E9Q8T0\_MOUSE tr|E9Q9E7|E9Q9E7\_MOUSE tr|R4GML4|R4GML4\_MOUSE sp|Q8R1H8-2|BATF2\_MOUSE sp|Q8R1H8|BATF2\_MOUSE tr|M0QW59|M0QW59\_MOUSE sp|Q3UVC0|KSR2\_MOUSE tr|E9PXD1|E9PXD1\_MOUSE sp|P54320|ELN\_MOUSE sp|P97484|LIRB3\_MOUSE tr|E9Q1Z6|E9Q1Z6\_MOUSE tr|Q52KH6|Q52KH6\_MOUSE tr|G8JL51|G8JL51\_MOUSE sp|Q8K202-2|RPA49\_MOUSE sp|Q9D454|CX049\_MOUSE sp|Q8VHQ4|RB40C\_MOUSE tr|E9PXR4|E9PXR4\_MOUSE tr|E9Q016|E9Q016\_MOUSE sp|Q9WVF7|DPOE1\_MOUSE sp|Q8K202-3|RPA49\_MOUSE tr|E9Q708|E9Q708\_MOUSE tr|E9PX60|E9PX60\_MOUSE tr|E9Q7T0|E9Q7T0\_MOUSE tr|E9Q7E8|E9Q7E8\_MOUSE tr|E9PVU6|E9PVU6\_MOUSE tr|E9Q7E6|E9Q7E6\_MOUSE sp|P21803|FGFR2\_MOUSE tr|E9PX90|E9PX90\_MOUSE tr|E9PXV8|E9PXV8\_MOUSE tr|D3Z5M8|D3Z5M8\_MOUSE tr|E9Q5C3|E9Q5C3\_MOUSE tr|E9PX88|E9PX88\_MOUSE tr|E9PX67|E9PX67\_MOUSE tr|E9Q700|E9Q700\_MOUSE sp|P21803-2|FGFR2\_MOUSE sp|Q9DBN5-2|LONP2\_MOUSE sp|Q9DBN5|LONP2\_MOUSE sp|P47740|AL3A2\_MOUSE tr|B1ATI0|B1ATI0\_MOUSE tr|B1AV77|B1AV77\_MOUSE sp|Q0P678-2|ZCH18\_MOUSE sp|Q99KG3-2|RBM10\_MOUSE sp|Q99KG3-3|RBM10\_MOUSE tr|Q9D592|Q9D592\_MOUSE sp|Q99KG3|RBM10\_MOUSE tr|A2AQD5|A2AQD5\_MOUSE sp|Q922B9-3|SSFA2\_MOUSE sp|Q922B9|SSFA2\_MOUSE tr|G5E8J6|G5E8J6\_MOUSE tr|Q8C635|Q8C635\_MOUSE tr|A2AQD6|A2AQD6\_MOUSE tr|E9QKL6|E9QKL6\_MOUSE sp|P17426-2|AP2A1\_MOUSE tr|Q80YX0|Q80YX0\_MOUSE sp|P05214|TBA3\_MOUSE sp|P68373|TBA1C\_MOUSE sp|P68369|TBA1A\_MOUSE sp|P05213|TBA1B\_MOUSE tr|E9PYQ0|E9PYQ0\_MOUSE sp|Q9WVB3|TLE6\_MOUSE tr|D3Z5E1|D3Z5E1\_MOUSE tr|Q8K4I0|Q8K4I0\_MOUSE tr|H3BLH2|H3BLH2\_MOUSE tr|F6ZKZ3|F6ZKZ3\_MOUSE sp|Q9R112|SQRD\_MOUSE sp|Q61738-2|ITA7\_MOUSE sp|Q61738-3|ITA7\_MOUSE tr|F8WHJ9|F8WHJ9\_MOUSE sp|Q6P8J7|KCRS\_MOUSE tr|Q8R259|Q8R259\_MOUSE tr|A2AS54|A2AS54\_MOUSE sp|Q9CTN5|S6OS1\_MOUSE sp|Q8K2I9|FBX18\_MOUSE sp|Q9D709|TMM79\_MOUSE sp|Q9D2L6|GP115\_MOUSE sp|P21619|LMNB2\_MOUSE sp|Q6AW69|CGNL1\_MOUSE sp|Q6AW69-4|CGNL1\_MOUSE tr|B2RSU6|B2RSU6\_MOUSE sp|Q6AW69-5|CGNL1\_MOUSE sp|B2RUR8|OTU7B\_MOUSE sp|Q5U430-3|UBR3\_MOUSE tr|F6QC57|F6QC57\_MOUSE sp|Q5U430|UBR3\_MOUSE sp|Q91VM9-2|IPYR2\_MOUSE sp|Q3UR50|VW5B2\_MOUSE sp|Q3UYR4|ESPNL\_MOUSE tr|E0CZ01|E0CZ01\_MOUSE tr|H3BLK9|H3BLK9\_MOUSE sp|P98199|AT8B2\_MOUSE tr|D3YXQ5|D3YXQ5\_MOUSE tr|E9QAL4|E9QAL4\_MOUSE tr|Q91XX1|Q91XX1\_MOUSE sp|Q8BGW5|SOSB2\_MOUSE sp|Q6NXZ1|KRBA1\_MOUSE tr|D3Z4B8|D3Z4B8\_MOUSE sp|P08923-4|LTK\_MOUSE sp|P08923-2|LTK\_MOUSE sp|P08923|LTK\_MOUSE sp|P08923-3|LTK\_MOUSE sp|P0CL69|ZN703\_MOUSE tr|F6V2R5|F6V2R5\_MOUSE tr|Q6YJU1|Q6YJU1\_MOUSE tr|G3XA00|G3XA00\_MOUSE sp|Q68EF4|GRM4\_MOUSE sp|Q68EF4-2|GRM4\_MOUSE sp|Q9DBG3|AP2B1\_MOUSE sp|Q9DBG3-2|AP2B1\_MOUSE tr|H3BIY9|H3BIY9\_MOUSE tr|H3BKM0|H3BKM0\_MOUSE tr|Q8BHX6|Q8BHX6\_MOUSE sp|Q9JJR9|NRIP3\_MOUSE tr|D6RI55|D6RI55\_MOUSE sp|Q7TSJ2-3|MAP6\_MOUSE sp|Q3UE37|UBE2Z\_MOUSE tr|D3YWN4|D3YWN4\_MOUSE tr|Q8VGP4|Q8VGP4\_MOUSE tr|Q8BU59|Q8BU59\_MOUSE tr|F6TV37|F6TV37\_MOUSE tr|V9GX48|V9GX48\_MOUSE tr|E9QPF8|E9QPF8\_MOUSE tr|E9PZM7|E9PZM7\_MOUSE sp|Q9CQ62|DECR\_MOUSE sp|Q8R1F6-2|HID1\_MOUSE tr|H3BK65|H3BK65\_MOUSE sp|Q8R1F6|HID1\_MOUSE sp|Q9JL96|CATM\_MOUSE tr|D3YWW9|D3YWW9\_MOUSE tr|F6R5R5|F6R5R5\_MOUSE sp|Q9R0N3|SYT11\_MOUSE tr|S4R1M8|S4R1M8\_MOUSE sp|P58043|SESN2\_MOUSE tr|D6RCY9|D6RCY9\_MOUSE tr|A3KFV7|A3KFV7\_MOUSE sp|Q8BLX7|COGA1\_MOUSE sp|O35405|PLD3\_MOUSE sp|O88623-3|UBP2\_MOUSE sp|O88623|UBP2\_MOUSE tr|Q5SUF7|Q5SUF7\_MOUSE tr|Q5SUG0|Q5SUG0\_MOUSE tr|Q5SUF6|Q5SUF6\_MOUSE tr|F6QHF1|F6QHF1\_MOUSE tr|Q5SUG3|Q5SUG3\_MOUSE tr|Q5SUG4|Q5SUG4\_MOUSE sp|Q8CBB2|S15A5\_MOUSE tr|F8VPV8|F8VPV8\_MOUSE tr|Q3TJY1|Q3TJY1\_MOUSE sp|Q99L90|MCRS1\_MOUSE tr|B2RRD7|B2RRD7\_MOUSE tr|Q80XK2|Q80XK2\_MOUSE tr|E9PXP1|E9PXP1\_MOUSE tr|Q3U109|Q3U109\_MOUSE tr|F6ZJ27|F6ZJ27\_MOUSE sp|A7L9Z8|AT2C2\_MOUSE tr|A2A4A6|A2A4A6\_MOUSE sp|Q62077|PLCG1\_MOUSE tr|D6RIM5|D6RIM5\_MOUSE sp|Q8BXR5|NALCN\_MOUSE tr|E9QLE4|E9QLE4\_MOUSE tr|Z4YJK9|Z4YJK9\_MOUSE sp|Q8CHS7|DRS7C\_MOUSE sp|Q80TM6-2|R3HD2\_MOUSE tr|E9PX98|E9PX98\_MOUSE tr|E9Q013|E9Q013\_MOUSE tr|M0QWN7|M0QWN7\_MOUSE tr|E9PZ57|E9PZ57\_MOUSE tr|E9Q0F6|E9Q0F6\_MOUSE sp|Q6A009|LTN1\_MOUSE tr|Q8VG76|Q8VG76\_MOUSE tr|Q8BLK6|Q8BLK6\_MOUSE sp|P97438|KCNK2\_MOUSE sp|Q64HY3-2|SP9\_MOUSE sp|Q6QIY3-2|SCNAA\_MOUSE sp|Q6QIY3|SCNAA\_MOUSE tr|K3W4P8|K3W4P8\_MOUSE sp|A9Z1V5|VW5B1\_MOUSE tr|F8VQM0|F8VQM0\_MOUSE tr|E9QNX9|E9QNX9\_MOUSE sp|A2ABV5-2|MED14\_MOUSE tr|A2BDN7|A2BDN7\_MOUSE sp|Q60751|IGF1R\_MOUSE sp|Q91XT6|TAC2N\_MOUSE sp|P21440|MDR3\_MOUSE tr|D3YYI8|D3YYI8\_MOUSE sp|O09106|HDAC1\_MOUSE sp|Q61301|CTNA2\_MOUSE sp|Q60980|KLF3\_MOUSE sp|Q61301-2|CTNA2\_MOUSE tr|E9Q509|E9Q509\_MOUSE sp|Q8BRT1-5|CLAP2\_MOUSE tr|Q5SVP2|Q5SVP2\_MOUSE tr|Q9EQB5|Q9EQB5\_MOUSE tr|V9GXX2|V9GXX2\_MOUSE tr|V9GWW6|V9GWW6\_MOUSE sp|B1AY13-2|UBP24\_MOUSE sp|E9PZM4|CHD2\_MOUSE A2I7N1 tr|F7C9P2|F7C9P2\_MOUSE sp|Q80ZD3|S2611\_MOUSE sp|Q8BZA7|GPR26\_MOUSE tr|Q8VGZ3|Q8VGZ3\_MOUSE tr|Q8VGL1|Q8VGL1\_MOUSE sp|Q8VIM5|MYCD\_MOUSE sp|Q8CG65|SSPO\_MOUSE tr|E9QNG1|E9QNG1\_MOUSE sp|A2AL36-2|CNTRL\_MOUSE sp|Q60770|STXB3\_MOUSE sp|Q9Z0R6|ITSN2\_MOUSE sp|Q60770-2|STXB3\_MOUSE tr|G3UZD6|G3UZD6\_MOUSE sp|Q8BLN6|UNC80\_MOUSE sp|Q9D0N7|CAF1B\_MOUSE tr|Q3V0P9|Q3V0P9\_MOUSE sp|Q61468|MSLN\_MOUSE sp|Q69ZK5|KLH14\_MOUSE tr|D6RJ50|D6RJ50\_MOUSE sp|A2AAY5|SPD2B\_MOUSE sp|Q8K0H1-2|S47A1\_MOUSE sp|Q62406|IRAK1\_MOUSE tr|B1AUW9|B1AUW9\_MOUSE sp|Q8K0H1|S47A1\_MOUSE sp|Q8C167-2|PPCEL\_MOUSE tr|E9Q5N9|E9Q5N9\_MOUSE sp|Q8C167|PPCEL\_MOUSE sp|P06537|GCR\_MOUSE tr|Q8C393|Q8C393\_MOUSE tr|E9PUR6|E9PUR6\_MOUSE sp|Q9WV27|AT1A4\_MOUSE sp|P06537-3|GCR\_MOUSE sp|P13808-5|B3A2\_MOUSE sp|P06537-4|GCR\_MOUSE tr|E9PYV1|E9PYV1\_MOUSE sp|P06537-2|GCR\_MOUSE sp|P13808-4|B3A2\_MOUSE tr|D3YY34|D3YY34\_MOUSE sp|Q8C9H6|STRP2\_MOUSE tr|E9QPT4|E9QPT4\_MOUSE P12763 sp|Q8CHP6-2|PHC3\_MOUSE sp|Q3V038|TTC9A\_MOUSE tr|Q8C134|Q8C134\_MOUSE tr|A0A087WQU0|A0A087WQU0\_MOUSE sp|Q9ERD6-4|RGPS2\_MOUSE tr|E9Q3I3|E9Q3I3\_MOUSE tr|Q6NZH9|Q6NZH9\_MOUSE tr|J3QNG0|J3QNG0\_MOUSE sp|P16460|ASSY\_MOUSE sp|Q8VHR0|PCD18\_MOUSE sp|Q8BHY3|ANO1\_MOUSE sp|Q9WTP9|VAX2\_MOUSE sp|Q8BHY3-2|ANO1\_MOUSE tr|G8JL82|G8JL82\_MOUSE sp|Q8BYA0|TBCD\_MOUSE sp|Q80YR2-2|F16B2\_MOUSE tr|E9Q641|E9Q641\_MOUSE sp|Q80YR2|F16B2\_MOUSE sp|Q80XB4-3|NRAP\_MOUSE sp|Q7TPS5-3|C2CD5\_MOUSE tr|F6SXT0|F6SXT0\_MOUSE tr|E9Q026|E9Q026\_MOUSE sp|Q7TPS5|C2CD5\_MOUSE sp|Q9JMA7|CP341\_MOUSE sp|Q64459|CP3AB\_MOUSE sp|Q61789|LAMA3\_MOUSE sp|Q61789-2|LAMA3\_MOUSE sp|Q8C8V1-2|ZXDC\_MOUSE sp|Q6A4J8-3|UBP7\_MOUSE sp|Q6A4J8|UBP7\_MOUSE tr|E9Q4N0|E9Q4N0\_MOUSE sp|Q6A4J8-2|UBP7\_MOUSE tr|F8VPX1|F8VPX1\_MOUSE sp|Q8C8V1-3|ZXDC\_MOUSE tr|E9PXY8|E9PXY8\_MOUSE sp|Q8C8V1|ZXDC\_MOUSE tr|E9PWN8|E9PWN8\_MOUSE tr|D3Z0W0|D3Z0W0\_MOUSE sp|Q640P4|GL8D2\_MOUSE tr|E9QLV1|E9QLV1\_MOUSE tr|D3Z085|D3Z085\_MOUSE sp|Q3U108|ARI5A\_MOUSE sp|Q3U108-2|ARI5A\_MOUSE sp|Q2TB54|FCAMR\_MOUSE sp|Q2TB54-2|FCAMR\_MOUSE tr|E9Q0E2|E9Q0E2\_MOUSE sp|E9PV87|TALD3\_MOUSE sp|Q8K2A1|GULP1\_MOUSE sp|Q69Z28-2|ATS16\_MOUSE sp|Q8K2A1-2|GULP1\_MOUSE tr|E0CXC5|E0CXC5\_MOUSE tr|D6RGS8|D6RGS8\_MOUSE sp|Q69Z28|ATS16\_MOUSE sp|Q9QWG7-2|ST1B1\_MOUSE sp|Q9QWG7|ST1B1\_MOUSE tr|F7D3E1|F7D3E1\_MOUSE sp|P35546-2|RET\_MOUSE sp|P35546|RET\_MOUSE sp|Q9Z2C4|MTMR1\_MOUSE tr|I7HJQ9|I7HJQ9\_MOUSE sp|Q9QYY9|ADH4\_MOUSE tr|F6T9H9|F6T9H9\_MOUSE sp|P17981|NAR2A\_MOUSE sp|Q9D3H0-2|TMM80\_MOUSE sp|Q9D3H0|TMM80\_MOUSE sp|P63038|CH60\_MOUSE sp|Q8VCG4|CO8G\_MOUSE sp|Q6PFD9|NUP98\_MOUSE tr|Q9DAC2|Q9DAC2\_MOUSE tr|F7BZW0|F7BZW0\_MOUSE tr|F6TJX7|F6TJX7\_MOUSE tr|F2Z497|F2Z497\_MOUSE sp|Q3MI99|CCBE1\_MOUSE sp|B9EJA2-2|CTTB2\_MOUSE tr|E9QLH9|E9QLH9\_MOUSE sp|Q571I4|SG223\_MOUSE sp|Q9QXX4|CMC2\_MOUSE tr|H3BJZ7|H3BJZ7\_MOUSE tr|F6RAZ3|F6RAZ3\_MOUSE tr|V9GXP2|V9GXP2\_MOUSE sp|Q68FE8-2|Z280D\_MOUSE sp|Q07417|ACADS\_MOUSE sp|Q4KUS2|UN13A\_MOUSE sp|P59764|DOCK4\_MOUSE sp|Q3V140|ACRBP\_MOUSE sp|Q91YA2|KPSH1\_MOUSE sp|Q8R4T9-4|UT2\_MOUSE sp|Q3TTE0-4|ADAM5\_MOUSE tr|F6S1B5|F6S1B5\_MOUSE sp|O88974-4|SETB1\_MOUSE sp|Q921Q3|ALG1\_MOUSE sp|Q921Q3-2|ALG1\_MOUSE tr|J3QK70|J3QK70\_MOUSE tr|D3YYH9|D3YYH9\_MOUSE sp|O88974|SETB1\_MOUSE sp|Q5ND34-2|WDR81\_MOUSE sp|Q5ND34|WDR81\_MOUSE tr|K4DI77|K4DI77\_MOUSE sp|Q5ND34-3|WDR81\_MOUSE sp|Q9JI76|ADA21\_MOUSE sp|O54887|TSKS\_MOUSE tr|Q147V4|Q147V4\_MOUSE tr|G3UYN0|G3UYN0\_MOUSE sp|Q587J6|LITD1\_MOUSE tr|F6RMZ4|F6RMZ4\_MOUSE sp|P97369|NCF4\_MOUSE tr|A8XU21|A8XU21\_MOUSE sp|P53657|KPYR\_MOUSE tr|G3X925|G3X925\_MOUSE tr|Z4YJF5|Z4YJF5\_MOUSE sp|Q62234|MYOM1\_MOUSE sp|Q62234-2|MYOM1\_MOUSE sp|Q9JIF9|MYOTI\_MOUSE tr|F2Z470|F2Z470\_MOUSE sp|Q01237|HMDH\_MOUSE sp|Q9QY76|VAPB\_MOUSE tr|Q8BH80|Q8BH80\_MOUSE sp|Q8CDN9|LRRC9\_MOUSE tr|E9PX29|E9PX29\_MOUSE tr|Q91ZE6|Q91ZE6\_MOUSE sp|Q8C3K5|TMM72\_MOUSE sp|Q60604|ADSV\_MOUSE tr|G3X8Q5|G3X8Q5\_MOUSE sp|Q61147|CERU\_MOUSE tr|G3X9T8|G3X9T8\_MOUSE tr|E9PZD8|E9PZD8\_MOUSE sp|P08399|PHXR5\_MOUSE sp|Q7TM99|MOT9\_MOUSE tr|E9Q816|E9Q816\_MOUSE sp|Q6PDE7-2|GGT6\_MOUSE sp|Q8BHK1|NIPA1\_MOUSE sp|Q6PDE7|GGT6\_MOUSE tr|D3Z3P9|D3Z3P9\_MOUSE sp|A2A6H3|FBX47\_MOUSE sp|A2AQP0|MYH7B\_MOUSE sp|Q64438|ANG2\_MOUSE sp|P97368|MEIS3\_MOUSE sp|P97368-2|MEIS3\_MOUSE sp|Q8C2Q3-2|RBM14\_MOUSE tr|D6MZJ6|D6MZJ6\_MOUSE sp|Q6PE65|GPT2L\_MOUSE sp|P48379|RFX2\_MOUSE tr|E9Q8N6|E9Q8N6\_MOUSE sp|Q8CB49|TPRG1\_MOUSE sp|Q8R326-2|PSPC1\_MOUSE tr|Q9QZ19|Q9QZ19\_MOUSE sp|Q8R326|PSPC1\_MOUSE sp|O88845|AKA10\_MOUSE tr|A2A716|A2A716\_MOUSE sp|Q3V0B4|CC108\_MOUSE sp|O88451|RDH7\_MOUSE tr|F8VPL1|F8VPL1\_MOUSE sp|Q5RJF7-2|CA2D4\_MOUSE tr|A0A087WQH4|A0A087WQH4\_MOUSE sp|Q5RJF7|CA2D4\_MOUSE tr|D3YTQ4|D3YTQ4\_MOUSE sp|O70628|PDE9A\_MOUSE sp|Q3UPH7|ARH40\_MOUSE sp|Q3UPH7-2|ARH40\_MOUSE tr|S4R2U9|S4R2U9\_MOUSE sp|Q0VGT2|GLI2\_MOUSE sp|Q3UPH7-6|ARH40\_MOUSE tr|S4R189|S4R189\_MOUSE sp|Q3UPH7-3|ARH40\_MOUSE sp|Q91WS7|DEPD7\_MOUSE tr|Q6TA13|Q6TA13\_MOUSE sp|Q9WTV6|UBP18\_MOUSE tr|Q8CJC7|Q8CJC7\_MOUSE tr|E9Q8C9|E9Q8C9\_MOUSE sp|O70458|OSMR\_MOUSE sp|O70458-2|OSMR\_MOUSE tr|Q5SUF5|Q5SUF5\_MOUSE tr|E9PWL1|E9PWL1\_MOUSE tr|Q5SUG1|Q5SUG1\_MOUSE tr|Q5SUF8|Q5SUF8\_MOUSE tr|Q5SUF9|Q5SUF9\_MOUSE tr|Q5SUG2|Q5SUG2\_MOUSE tr|E9PWR7|E9PWR7\_MOUSE tr|F8WHT2|F8WHT2\_MOUSE tr|Q3TMK4|Q3TMK4\_MOUSE tr|E9Q9B1|E9Q9B1\_MOUSE sp|Q76I79|SSH1\_MOUSE sp|Q76I79-2|SSH1\_MOUSE sp|Q8BHJ6|SERC5\_MOUSE tr|B1AWI7|B1AWI7\_MOUSE sp|Q8BM55-2|TM214\_MOUSE sp|Q8BM55-3|TM214\_MOUSE tr|S4R295|S4R295\_MOUSE tr|E9PZP2|E9PZP2\_MOUSE sp|Q9QWH1|PHC2\_MOUSE tr|E9PUQ8|E9PUQ8\_MOUSE tr|B1AWN8|B1AWN8\_MOUSE tr|H9KV07|H9KV07\_MOUSE tr|Q8VFY3|Q8VFY3\_MOUSE sp|Q7TNH6|NPHP3\_MOUSE sp|Q4PZA2|ECE1\_MOUSE sp|Q4PZA2-4|ECE1\_MOUSE sp|Q4PZA2-3|ECE1\_MOUSE sp|Q80TS8-2|SE1L3\_MOUSE tr|B1AQ57|B1AQ57\_MOUSE sp|Q9JK91|MLH1\_MOUSE tr|D3YXH1|D3YXH1\_MOUSE sp|Q7TSN7|IGSF5\_MOUSE tr|E9PZW0|E9PZW0\_MOUSE tr|H3BKQ0|H3BKQ0\_MOUSE sp|Q6PGK7-2|CHSTA\_MOUSE sp|P11798-2|KCC2A\_MOUSE tr|F8VQ54|F8VQ54\_MOUSE sp|Q6B966|NAL14\_MOUSE tr|A2A9K7|A2A9K7\_MOUSE sp|Q60636-4|PRDM1\_MOUSE sp|Q8C015|PAK7\_MOUSE sp|Q5DU02|UBP22\_MOUSE tr|Q8BW84|Q8BW84\_MOUSE sp|Q9D0Z3-3|TMM53\_MOUSE sp|P58462|FOXP1\_MOUSE sp|P58462-4|FOXP1\_MOUSE tr|D3Z6Q4|D3Z6Q4\_MOUSE tr|D3Z6Q3|D3Z6Q3\_MOUSE tr|E9Q3M5|E9Q3M5\_MOUSE sp|P41230-3|KDM5C\_MOUSE tr|H3BLF2|H3BLF2\_MOUSE sp|P41230-2|KDM5C\_MOUSE tr|H3BL33|H3BL33\_MOUSE tr|H3BJM1|H3BJM1\_MOUSE tr|H3BJ24|H3BJ24\_MOUSE sp|P41230|KDM5C\_MOUSE sp|P58462-2|FOXP1\_MOUSE tr|E2S038|E2S038\_MOUSE sp|P58462-3|FOXP1\_MOUSE tr|F7AC41|F7AC41\_MOUSE tr|Q91VU7|Q91VU7\_MOUSE sp|Q8BWM0|PGES2\_MOUSE sp|Q8BGX4|OFCC1\_MOUSE sp|Q80U30|CL16A\_MOUSE tr|D3Z7I0|D3Z7I0\_MOUSE sp|Q0VGM9-2|RTEL1\_MOUSE sp|Q0VGM9-5|RTEL1\_MOUSE sp|Q0VGM9-4|RTEL1\_MOUSE sp|Q0VGM9-3|RTEL1\_MOUSE sp|Q0VGM9|RTEL1\_MOUSE tr|Z4YLW7|Z4YLW7\_MOUSE sp|Q9DBW0|CP4V2\_MOUSE tr|G3X8W7|G3X8W7\_MOUSE sp|Q8VC69|S22A6\_MOUSE sp|Q8BGZ4|CDC23\_MOUSE sp|Q921F4|HNRLL\_MOUSE tr|B2RT89|B2RT89\_MOUSE sp|P51829|ADCY7\_MOUSE sp|Q5VCS6-2|TDRD5\_MOUSE tr|J3QPB7|J3QPB7\_MOUSE sp|P23475|XRCC6\_MOUSE sp|Q3TJM4|CENPT\_MOUSE sp|Q5VCS6|TDRD5\_MOUSE sp|Q62245|SOS1\_MOUSE tr|B1AS46|B1AS46\_MOUSE sp|Q9CWL2|CASZ1\_MOUSE sp|Q8BUV8|GP107\_MOUSE tr|D3YW58|D3YW58\_MOUSE sp|O35484|AZIN1\_MOUSE sp|Q8CBD1|NRIP1\_MOUSE sp|Q61474-2|MSI1H\_MOUSE sp|Q99PV0|PRP8\_MOUSE sp|Q6NXJ0|WWC2\_MOUSE sp|P70270|RAD54\_MOUSE sp|Q8BMC0|LPAR6\_MOUSE tr|F6X844|F6X844\_MOUSE sp|Q9Z211|PX11A\_MOUSE sp|Q5DID3-2|UROL1\_MOUSE sp|O88282|BCL6B\_MOUSE sp|Q9D0L6|BAMBI\_MOUSE sp|Q32Q92-2|ACOT6\_MOUSE sp|Q8BWN8|ACOT4\_MOUSE sp|Q8BLF2-4|CDKL3\_MOUSE sp|P70232|NCHL1\_MOUSE tr|F7D5X7|F7D5X7\_MOUSE tr|Q8K350|Q8K350\_MOUSE sp|Q99L88|SNTB1\_MOUSE tr|D3Z103|D3Z103\_MOUSE sp|Q91ZP9-2|NECA2\_MOUSE sp|Q8BXR1|S7A14\_MOUSE sp|Q9EQH2|ERAP1\_MOUSE sp|Q8R3C1-2|CB042\_MOUSE tr|E9Q774|E9Q774\_MOUSE tr|E9Q777|E9Q777\_MOUSE sp|Q8R3C1-3|CB042\_MOUSE tr|E9QAD3|E9QAD3\_MOUSE sp|Q8R3C1|CB042\_MOUSE sp|Q02395|MTF2\_MOUSE sp|Q02395-2|MTF2\_MOUSE tr|A2A4H9|A2A4H9\_MOUSE sp|Q61576|FKB10\_MOUSE tr|E9PV03|E9PV03\_MOUSE tr|J3QNE8|J3QNE8\_MOUSE sp|Q8BUY8|GASP2\_MOUSE sp|Q5I012-3|S38AA\_MOUSE sp|Q5I012-2|S38AA\_MOUSE sp|Q5I012|S38AA\_MOUSE sp|Q5I012-4|S38AA\_MOUSE sp|Q3TTE0-2|ADAM5\_MOUSE sp|P60954-2|NOL4\_MOUSE tr|E9Q947|E9Q947\_MOUSE tr|F6WYJ5|F6WYJ5\_MOUSE sp|Q6NS69|AMER3\_MOUSE sp|Q80TF3|PCD19\_MOUSE tr|E9Q5E1|E9Q5E1\_MOUSE tr|A2AGW4|A2AGW4\_MOUSE sp|Q8C547-3|HTR5B\_MOUSE sp|P70280|VAMP7\_MOUSE sp|Q80W93-2|HYDIN\_MOUSE tr|Q9QUM5|Q9QUM5\_MOUSE sp|Q9WV93|HEY1\_MOUSE sp|Q692V3-3|GNN\_MOUSE sp|Q9WVM8|AADAT\_MOUSE sp|A8C756-3|THADA\_MOUSE tr|Z4YLN8|Z4YLN8\_MOUSE sp|Q9D3G5|STX11\_MOUSE tr|F7CD74|F7CD74\_MOUSE tr|Q3U5V8|Q3U5V8\_MOUSE sp|Q32MW3|ACO10\_MOUSE sp|Q8BFW4|TRI65\_MOUSE tr|G3UXL6|G3UXL6\_MOUSE tr|F7CZB5|F7CZB5\_MOUSE sp|Q3UIK4|MET14\_MOUSE tr|D3Z0R8|D3Z0R8\_MOUSE sp|O88416|GPR33\_MOUSE tr|J3QN68|J3QN68\_MOUSE sp|Q14AX6-2|CDK12\_MOUSE tr|Q9CYB4|Q9CYB4\_MOUSE sp|Q14AX6|CDK12\_MOUSE sp|Q61501|E2F1\_MOUSE sp|Q14AX6-3|CDK12\_MOUSE sp|Q9D328|TMM35\_MOUSE sp|D3Z4R1|HFM1\_MOUSE sp|Q8JZX4|SPF45\_MOUSE sp|Q8BH55|THNS1\_MOUSE sp|Q91V98|CD248\_MOUSE sp|Q8VCB2|MED25\_MOUSE tr|E9PUL2|E9PUL2\_MOUSE sp|Q8VCB2-3|MED25\_MOUSE sp|Q8CDN9-2|LRRC9\_MOUSE sp|Q8VCB2-2|MED25\_MOUSE sp|A2ABU4|MYOM3\_MOUSE sp|P68368|TBA4A\_MOUSE sp|P32240|PE2R4\_MOUSE sp|Q6DYE8|ENPP3\_MOUSE tr|G5E8P1|G5E8P1\_MOUSE tr|Z4YLZ2|Z4YLZ2\_MOUSE tr|F6U1Y8|F6U1Y8\_MOUSE tr|E9PZ26|E9PZ26\_MOUSE tr|B4YB48|B4YB48\_MOUSE sp|Q09163-3|DLK1\_MOUSE tr|Q925U3|Q925U3\_MOUSE tr|B4YB46|B4YB46\_MOUSE sp|Q09163-5|DLK1\_MOUSE sp|Q09163-2|DLK1\_MOUSE tr|E9PVN3|E9PVN3\_MOUSE sp|Q09163|DLK1\_MOUSE tr|J3QM82|J3QM82\_MOUSE sp|Q8BXA7|PHLP2\_MOUSE tr|Q96LX3|Q96LX3\_MOUSE tr|E9PVA5|E9PVA5\_MOUSE tr|Q3V3G0|Q3V3G0\_MOUSE tr|B7ZNH2|B7ZNH2\_MOUSE sp|P22933|GBRD\_MOUSE tr|Q91XW9|Q91XW9\_MOUSE sp|Q9D2G5-6|SYNJ2\_MOUSE sp|E9QMW4|CP096\_MOUSE sp|Q91Z96|BMP2K\_MOUSE sp|Q80UF9|OTOP3\_MOUSE sp|P97820|M4K4\_MOUSE tr|F8VQ32|F8VQ32\_MOUSE sp|Q80UF9-2|OTOP3\_MOUSE tr|D3YU32|D3YU32\_MOUSE tr|E9Q343|E9Q343\_MOUSE sp|Q62255-2|SALL3\_MOUSE tr|G5E876|G5E876\_MOUSE sp|Q62255|SALL3\_MOUSE sp|A2ATU0|DHTK1\_MOUSE sp|Q9QYB5-2|ADDG\_MOUSE sp|P21180|CO2\_MOUSE tr|B8JJN2|B8JJN2\_MOUSE sp|P21180-2|CO2\_MOUSE sp|Q9QYB5|ADDG\_MOUSE tr|Q80ZX0|Q80ZX0\_MOUSE tr|F6TYE6|F6TYE6\_MOUSE tr|E9Q0M7|E9Q0M7\_MOUSE sp|Q920Q8|NS1BP\_MOUSE sp|Q5SW25|P12L2\_MOUSE tr|Q8VEX7|Q8VEX7\_MOUSE tr|E9PYB3|E9PYB3\_MOUSE sp|A2AKX3-2|SETX\_MOUSE sp|A2AKX3|SETX\_MOUSE sp|Q8BVL3|SNX17\_MOUSE sp|Q5SQX6|CYFP2\_MOUSE sp|Q7TMA2-2|ZN503\_MOUSE sp|P09055|ITB1\_MOUSE sp|Q99KN1|ARRD1\_MOUSE sp|Q99KN1-2|ARRD1\_MOUSE tr|A2AIS9|A2AIS9\_MOUSE sp|Q99NC0|VGLL1\_MOUSE tr|F7BYG3|F7BYG3\_MOUSE sp|E9Q355-2|CTSG1\_MOUSE sp|E9Q355|CTSG1\_MOUSE tr|A2A8K5|A2A8K5\_MOUSE sp|Q148B6|SPERI\_MOUSE sp|Q3U0L2|AN33B\_MOUSE sp|Q3U0L2-2|AN33B\_MOUSE sp|Q8CB14|LAS2\_MOUSE sp|Q9D0E3|LYSM1\_MOUSE tr|Q14DT3|Q14DT3\_MOUSE sp|Q08509|EPS8\_MOUSE sp|Q3UHU5-3|MTCL1\_MOUSE sp|Q3UHU5|MTCL1\_MOUSE sp|Q91V83|TTI1\_MOUSE tr|G3UYU5|G3UYU5\_MOUSE tr|H3BJR0|H3BJR0\_MOUSE sp|Q8QZY2|GLCTK\_MOUSE sp|Q8BPM2|M4K5\_MOUSE sp|Q9Z1P8|ANGL4\_MOUSE tr|A2AE18|A2AE18\_MOUSE tr|A2AE19|A2AE19\_MOUSE sp|Q9D8N2|FA45A\_MOUSE sp|Q6P560|ZN182\_MOUSE tr|A2ATJ9|A2ATJ9\_MOUSE tr|E9Q166|E9Q166\_MOUSE tr|F8VPJ5|F8VPJ5\_MOUSE sp|Q58Y74|TRCG1\_MOUSE tr|E9QA91|E9QA91\_MOUSE sp|P59384|ATS15\_MOUSE sp|O35144|TERF2\_MOUSE tr|E9QM06|E9QM06\_MOUSE tr|D3YZ08|D3YZ08\_MOUSE sp|Q6PD19|CJ076\_MOUSE tr|A0A087WP47|A0A087WP47\_MOUSE tr|D3YYK9|D3YYK9\_MOUSE tr|E9Q156|E9Q156\_MOUSE sp|Q3U0S6|RAIN\_MOUSE sp|Q58A65|JIP4\_MOUSE sp|Q9DC50|OCTC\_MOUSE tr|L7N217|L7N217\_MOUSE sp|A2ADA5-2|PUSL1\_MOUSE tr|Q6NZD2|Q6NZD2\_MOUSE sp|Q8JZM7|CDC73\_MOUSE tr|F8WGX5|F8WGX5\_MOUSE tr|A3KG49|A3KG49\_MOUSE sp|Q6PNC0|DMXL1\_MOUSE sp|Q80TG1-3|KANL1\_MOUSE sp|Q9JM99-3|PRG4\_MOUSE sp|Q80TG1-4|KANL1\_MOUSE tr|Q8R2D9|Q8R2D9\_MOUSE sp|Q9DBE9|SPB1\_MOUSE sp|Q148W8|DUS27\_MOUSE tr|G3UY93|G3UY93\_MOUSE sp|Q9Z1Q9|SYVC\_MOUSE tr|F7CJT5|F7CJT5\_MOUSE sp|Q9Z0S3|CLD14\_MOUSE tr|Q2LKU9|Q2LKU9\_MOUSE tr|Q67EY4|Q67EY4\_MOUSE sp|Q91XE9|CR3L3\_MOUSE sp|Q8BWQ5|DCLK3\_MOUSE tr|C3S7Q5|C3S7Q5\_MOUSE sp|P97433-2|ARG28\_MOUSE tr|E9Q6F9|E9Q6F9\_MOUSE tr|J3QNV0|J3QNV0\_MOUSE sp|A2AWP8|ARGAL\_MOUSE tr|A2AWP6|A2AWP6\_MOUSE sp|A2AWP8-3|ARGAL\_MOUSE sp|A2AWP8-2|ARGAL\_MOUSE sp|A2AWP8-4|ARGAL\_MOUSE sp|Q99M74|KRT82\_MOUSE sp|P23819-3|GRIA2\_MOUSE sp|Q8K409|DPOLB\_MOUSE tr|D3YV80|D3YV80\_MOUSE sp|Q80TQ5-3|PKHM2\_MOUSE sp|Q80TQ5-2|PKHM2\_MOUSE tr|A2ADE0|A2ADE0\_MOUSE sp|Q80TQ5|PKHM2\_MOUSE tr|Z4YJW6|Z4YJW6\_MOUSE tr|A2A485|A2A485\_MOUSE tr|Q3UH28|Q3UH28\_MOUSE tr|Q3U1M7|Q3U1M7\_MOUSE tr|A2A482|A2A482\_MOUSE sp|Q99MV5-2|M10L1\_MOUSE sp|Q6PB70|ANO8\_MOUSE sp|Q8BGZ8|ZPLD1\_MOUSE tr|H3BJL2|H3BJL2\_MOUSE tr|A6PWD6|A6PWD6\_MOUSE sp|Q9CW46|RAVR1\_MOUSE tr|G3UVU7|G3UVU7\_MOUSE tr|Q8C8Y2|Q8C8Y2\_MOUSE tr|E9Q271|E9Q271\_MOUSE sp|P97329|KI20A\_MOUSE sp|Q9Z2H7|GIPC2\_MOUSE tr|Q99LF7|Q99LF7\_MOUSE sp|P31809|CEAM1\_MOUSE sp|P31809-3|CEAM1\_MOUSE tr|Q3LFS9|Q3LFS9\_MOUSE tr|Q925P3|Q925P3\_MOUSE sp|Q08093|CNN2\_MOUSE tr|A2AE45|A2AE45\_MOUSE sp|Q7M761|OVCH2\_MOUSE sp|Q8BMN3-2|ACHB3\_MOUSE sp|Q8BMN3|ACHB3\_MOUSE sp|Q80VA5-2|S3TC2\_MOUSE tr|F6YST4|F6YST4\_MOUSE sp|Q6P5H2|NEST\_MOUSE tr|F7DF70|F7DF70\_MOUSE sp|Q68FF6|GIT1\_MOUSE sp|Q6P5H2-2|NEST\_MOUSE tr|E9Q295|E9Q295\_MOUSE sp|Q8BXL9-6|IFFO1\_MOUSE sp|Q3TRJ4|K1C26\_MOUSE tr|E9PVA8|E9PVA8\_MOUSE sp|Q60953-2|PML\_MOUSE tr|Q9D2F8|Q9D2F8\_MOUSE tr|G3UXF0|G3UXF0\_MOUSE sp|Q60960|IMA5\_MOUSE sp|Q9JHJ5|5HT3B\_MOUSE tr|Q3UZ64|Q3UZ64\_MOUSE tr|E9PUG6|E9PUG6\_MOUSE sp|O35368-3|IFI3\_MOUSE sp|O35368|IFI3\_MOUSE sp|Q4VAB4-2|CNPY1\_MOUSE tr|D3YX36|D3YX36\_MOUSE tr|E9Q9W4|E9Q9W4\_MOUSE sp|Q3V089|RBM44\_MOUSE sp|A2A863|ITB4\_MOUSE sp|A2A863-2|ITB4\_MOUSE sp|Q7TPS0-3|KS6A6\_MOUSE sp|Q7TPS0-2|KS6A6\_MOUSE sp|Q7TPS0|KS6A6\_MOUSE sp|P21661|NEC2\_MOUSE sp|Q6PAM1-2|TXLNA\_MOUSE tr|Q8C116|Q8C116\_MOUSE tr|D3YZY0|D3YZY0\_MOUSE tr|Q7TRG9|Q7TRG9\_MOUSE sp|P21447|MDR1A\_MOUSE sp|Q6IRT3|PARI\_MOUSE tr|Q7TSG6|Q7TSG6\_MOUSE sp|Q9R0Y8|GBRG1\_MOUSE tr|D3Z5G7|D3Z5G7\_MOUSE tr|Q7TNL9|Q7TNL9\_MOUSE sp|Q3UMY5-4|EMAL4\_MOUSE tr|A2ASW0|A2ASW0\_MOUSE tr|K7N6V2|K7N6V2\_MOUSE Q6KB66-1 tr|E9Q6J8|E9Q6J8\_MOUSE sp|Q8BYZ7|ELMO3\_MOUSE tr|E9PVG7|E9PVG7\_MOUSE sp|Q9QXQ1|PDE7B\_MOUSE tr|D3Z797|D3Z797\_MOUSE sp|P39087-2|GRIK2\_MOUSE sp|P39087|GRIK2\_MOUSE sp|P52785|GUC2E\_MOUSE tr|B1AY50|B1AY50\_MOUSE tr|B1AY49|B1AY49\_MOUSE tr|E9PUU5|E9PUU5\_MOUSE sp|Q5SWD9-3|TSR1\_MOUSE sp|Q5SWD9|TSR1\_MOUSE sp|Q7TSF0-2|DSG1C\_MOUSE sp|Q9CXY1|TM175\_MOUSE tr|A2AEP6|A2AEP6\_MOUSE tr|B2KF92|B2KF92\_MOUSE sp|Q66X22|NAL9B\_MOUSE tr|Q80YS4|Q80YS4\_MOUSE tr|B1AWS8|B1AWS8\_MOUSE sp|Q02858|TIE2\_MOUSE sp|Q63ZW7-3|INADL\_MOUSE sp|Q9JK97|KCNQ4\_MOUSE sp|Q9JL16-2|ISG20\_MOUSE sp|Q9JL16|ISG20\_MOUSE tr|Q8C6P5|Q8C6P5\_MOUSE sp|Q9D529|WBP2L\_MOUSE tr|D6RFE3|D6RFE3\_MOUSE sp|Q6PDE8|TM180\_MOUSE sp|P41971|ELK3\_MOUSE tr|A2AED5|A2AED5\_MOUSE sp|Q8BGK6|YLAT2\_MOUSE sp|P61971|NTF2\_MOUSE sp|Q6PFX9|TNKS1\_MOUSE sp|Q6PFX9-2|TNKS1\_MOUSE tr|Q91Y17|Q91Y17\_MOUSE tr|F7CXY1|F7CXY1\_MOUSE sp|Q8BIG4|FBX28\_MOUSE sp|Q60636-5|PRDM1\_MOUSE sp|Q60636|PRDM1\_MOUSE tr|E9QNR7|E9QNR7\_MOUSE sp|Q60636-2|PRDM1\_MOUSE tr|Q8VIE6|Q8VIE6\_MOUSE sp|Q8BIY3|ZC12D\_MOUSE sp|Q9Z1M7|LARGE\_MOUSE ENSEMBL:ENSBTAP00000025008 sp|Q91WA6|SHRPN\_MOUSE sp|Q91WA6-2|SHRPN\_MOUSE tr|G3UYX4|G3UYX4\_MOUSE sp|Q8VHF2|CDHR5\_MOUSE tr|A0A087WQV8|A0A087WQV8\_MOUSE tr|Z4YLK3|Z4YLK3\_MOUSE tr|A0PJK7|A0PJK7\_MOUSE tr|E9PZ77|E9PZ77\_MOUSE sp|P57784|RU2A\_MOUSE tr|F7CPG6|F7CPG6\_MOUSE tr|E9PUW8|E9PUW8\_MOUSE sp|Q8K009|AL1L2\_MOUSE sp|Q8R4X3|RBM12\_MOUSE sp|Q9D211|C2AIL\_MOUSE sp|Q80XI6|M3K11\_MOUSE sp|Q06770|CBG\_MOUSE tr|E9Q9Q6|E9Q9Q6\_MOUSE tr|Q8VEX8|Q8VEX8\_MOUSE sp|Q66JX5|FR1OP\_MOUSE sp|P28481-4|CO2A1\_MOUSE sp|P28481-1|CO2A1\_MOUSE sp|Q9ERK9|P2RY6\_MOUSE sp|P28481-2|CO2A1\_MOUSE sp|P28481|CO2A1\_MOUSE sp|Q5RJG1|NOL10\_MOUSE sp|P28481-5|CO2A1\_MOUSE sp|P28481-7|CO2A1\_MOUSE sp|P28481-6|CO2A1\_MOUSE tr|D3Z5V3|D3Z5V3\_MOUSE tr|F6V9F1|F6V9F1\_MOUSE sp|Q7TN29|SMAP2\_MOUSE tr|F7AN16|F7AN16\_MOUSE sp|Q3UYH7|ARBK2\_MOUSE sp|Q8R0Y8|S2542\_MOUSE sp|Q8C761|WDR60\_MOUSE sp|Q8BLI0|ATL1\_MOUSE tr|E9PWW5|E9PWW5\_MOUSE sp|Q8CBB2-2|S15A5\_MOUSE sp|Q8K2J4|CCD14\_MOUSE sp|Q8K2J4-2|CCD14\_MOUSE tr|E9Q9T4|E9Q9T4\_MOUSE tr|F8VPL2|F8VPL2\_MOUSE sp|Q61194|P3C2A\_MOUSE sp|Q61194-2|P3C2A\_MOUSE sp|Q9QZS0|CO4A3\_MOUSE sp|Q8BW86-2|ARG33\_MOUSE sp|Q8BID8|FXL14\_MOUSE tr|G3XA21|G3XA21\_MOUSE tr|E0CZ22|E0CZ22\_MOUSE sp|Q8BX43|TR19L\_MOUSE tr|F8VPN4|F8VPN4\_MOUSE sp|Q925I7|PDGFD\_MOUSE sp|Q925I7-2|PDGFD\_MOUSE sp|Q61527-3|ERBB4\_MOUSE sp|Q61527-2|ERBB4\_MOUSE sp|Q61527|ERBB4\_MOUSE tr|E9QKD9|E9QKD9\_MOUSE tr|D6RJL7|D6RJL7\_MOUSE tr|G3X8R4|G3X8R4\_MOUSE tr|E9PWY2|E9PWY2\_MOUSE sp|Q9DBA9|TF2H1\_MOUSE sp|Q61464-7|ZN638\_MOUSE sp|P58500|M3KCL\_MOUSE sp|Q505G4|LDOCL\_MOUSE sp|P28828|PTPRM\_MOUSE tr|A6H6A5|A6H6A5\_MOUSE tr|E9QPW3|E9QPW3\_MOUSE sp|Q8BGK2|ARHL1\_MOUSE tr|Q99J77|Q99J77\_MOUSE sp|Q14BJ1|FA89A\_MOUSE sp|Q8VEF1-3|GRM1A\_MOUSE sp|Q8VEF1|GRM1A\_MOUSE tr|F8WJ74|F8WJ74\_MOUSE tr|A2AIX5|A2AIX5\_MOUSE sp|Q6NSQ9|G6PC3\_MOUSE tr|A2AKW1|A2AKW1\_MOUSE tr|A0A087WQK6|A0A087WQK6\_MOUSE sp|Q6PD21|SHB\_MOUSE tr|Q8BRB1|Q8BRB1\_MOUSE sp|P29319|EPHA3\_MOUSE tr|D3YXE5|D3YXE5\_MOUSE tr|G3UY50|G3UY50\_MOUSE tr|G3UXS8|G3UXS8\_MOUSE sp|Q6PD03|2A5A\_MOUSE sp|Q9R1R2|TRIM3\_MOUSE tr|Q3TDT0|Q3TDT0\_MOUSE sp|Q99KU6|CF089\_MOUSE tr|V9GXF2|V9GXF2\_MOUSE tr|Q6VYI5|Q6VYI5\_MOUSE tr|A0A087WSH7|A0A087WSH7\_MOUSE tr|A0A087WR97|A0A087WR97\_MOUSE tr|Q6VYI4|Q6VYI4\_MOUSE tr|A0A087WRZ5|A0A087WRZ5\_MOUSE sp|Q8C3K6|SC5A1\_MOUSE tr|Q9QXI6|Q9QXI6\_MOUSE tr|Q8R0B4|Q8R0B4\_MOUSE tr|Q8BLD4|Q8BLD4\_MOUSE sp|Q5SQM0|EMAL6\_MOUSE tr|V9GWX5|V9GWX5\_MOUSE sp|Q99MQ5|COPA1\_MOUSE sp|Q80TH2-1|LAP2\_MOUSE sp|P17156|HSP72\_MOUSE sp|Q3TZM9-2|ALG11\_MOUSE tr|A0A087WQR6|A0A087WQR6\_MOUSE sp|Q99N09|M4A6B\_MOUSE sp|Q64514|TPP2\_MOUSE tr|A0A075B5R2|A0A075B5R2\_MOUSE sp|Q3URQ0|TEX10\_MOUSE sp|Q9DAQ4-3|CB081\_MOUSE sp|Q9DAQ4|CB081\_MOUSE tr|F6R9F0|F6R9F0\_MOUSE sp|Q9CQ49|NCBP2\_MOUSE sp|P42209|SEPT1\_MOUSE tr|D3YVG1|D3YVG1\_MOUSE sp|Q3URY2-2|GEMC1\_MOUSE sp|Q3URY2-3|GEMC1\_MOUSE sp|O55208|FIGLA\_MOUSE tr|D3Z3V3|D3Z3V3\_MOUSE tr|F6SLK1|F6SLK1\_MOUSE sp|Q3URY2|GEMC1\_MOUSE sp|Q3B7Z2|OSBP1\_MOUSE tr|E1AZ71|E1AZ71\_MOUSE tr|D3Z6T0|D3Z6T0\_MOUSE tr|B2RRI2|B2RRI2\_MOUSE sp|Q689Z5|SBNO1\_MOUSE sp|Q689Z5-2|SBNO1\_MOUSE tr|Z4YKC4|Z4YKC4\_MOUSE tr|A2AMI2|A2AMI2\_MOUSE tr|Q9CQ68|Q9CQ68\_MOUSE sp|Q4KMM3-4|OXR1\_MOUSE sp|Q4KMM3|OXR1\_MOUSE sp|Q4KMM3-2|OXR1\_MOUSE sp|Q4KMM3-3|OXR1\_MOUSE sp|Q3TTY0-3|PLB1\_MOUSE sp|B2B9E1|TRIQK\_MOUSE sp|Q9JI78|NGLY1\_MOUSE sp|Q4VA53-3|PDS5B\_MOUSE sp|Q4VA53|PDS5B\_MOUSE tr|F8WHU5|F8WHU5\_MOUSE sp|Q61738-4|ITA7\_MOUSE tr|G3X9Q1|G3X9Q1\_MOUSE sp|Q61738|ITA7\_MOUSE sp|Q61738-5|ITA7\_MOUSE sp|Q61738-6|ITA7\_MOUSE sp|Q80U28-7|MADD\_MOUSE tr|A2AGQ4|A2AGQ4\_MOUSE sp|Q80U28-5|MADD\_MOUSE sp|F6SEU4|SYGP1\_MOUSE sp|Q80U28-2|MADD\_MOUSE tr|A2AGQ2|A2AGQ2\_MOUSE sp|Q80U28-14|MADD\_MOUSE sp|Q80U28-3|MADD\_MOUSE sp|Q80U28-4|MADD\_MOUSE sp|P40764|DLX2\_MOUSE tr|A2AGQ5|A2AGQ5\_MOUSE tr|A2AGQ9|A2AGQ9\_MOUSE sp|Q80U28-8|MADD\_MOUSE tr|A2AGQ7|A2AGQ7\_MOUSE tr|E9QN47|E9QN47\_MOUSE tr|A2AGQ8|A2AGQ8\_MOUSE sp|Q60I26-2|AL2CL\_MOUSE tr|J3QQ18|J3QQ18\_MOUSE sp|Q60I26|AL2CL\_MOUSE sp|Q80U28-11|MADD\_MOUSE sp|Q2EMV9|PAR14\_MOUSE tr|E9Q6B6|E9Q6B6\_MOUSE sp|Q9ESN9-5|JIP3\_MOUSE sp|Q9D513|MEIOB\_MOUSE sp|Q7TPM6|FSD1\_MOUSE sp|Q8C4P0|K1958\_MOUSE sp|Q7TPM6-2|FSD1\_MOUSE sp|Q03963|E2AK2\_MOUSE tr|Q8VI95|Q8VI95\_MOUSE tr|F2Z3U2|F2Z3U2\_MOUSE sp|Q920Q8-4|NS1BP\_MOUSE sp|Q920Q8-3|NS1BP\_MOUSE sp|Q99LS1|MMAD\_MOUSE tr|A2AQT7|A2AQT7\_MOUSE sp|P09027|HXD3\_MOUSE sp|Q91W86|VPS11\_MOUSE sp|Q8R088|GLP3L\_MOUSE sp|Q7TS63-2|ZFAT\_MOUSE tr|E0CX30|E0CX30\_MOUSE sp|Q8R088-2|GLP3L\_MOUSE tr|H3BJ07|H3BJ07\_MOUSE tr|H3BKI7|H3BKI7\_MOUSE sp|Q7TS63|ZFAT\_MOUSE tr|K3W4N3|K3W4N3\_MOUSE tr|H3BJ82|H3BJ82\_MOUSE tr|E0CY83|E0CY83\_MOUSE sp|Q8BL00|CDHR3\_MOUSE sp|Q8VEG6-3|CNO6L\_MOUSE tr|F6YUG5|F6YUG5\_MOUSE sp|Q924Z5|TRAM2\_MOUSE sp|P55066|NCAN\_MOUSE tr|E9Q8X5|E9Q8X5\_MOUSE sp|Q9QZQ0|NPAS3\_MOUSE tr|F8VQB2|F8VQB2\_MOUSE tr|Q3UWS5|Q3UWS5\_MOUSE tr|Q8BI38|Q8BI38\_MOUSE sp|Q8R4P4|TMC2\_MOUSE tr|D6RI27|D6RI27\_MOUSE tr|H3BJF4|H3BJF4\_MOUSE tr|Q6GTM0|Q6GTM0\_MOUSE sp|Q64112|IFIT2\_MOUSE sp|O35350|CAN1\_MOUSE tr|G5E899|G5E899\_MOUSE tr|Q3TN43|Q3TN43\_MOUSE sp|Q80XM3|KCNG4\_MOUSE sp|Q69ZK7-2|F214A\_MOUSE sp|Q69ZK7|F214A\_MOUSE sp|P0C7A3|MBOA4\_MOUSE sp|Q9QY40|PLXB3\_MOUSE sp|Q01063|PDE4D\_MOUSE sp|Q80X76|SPA3F\_MOUSE tr|Q8C048|Q8C048\_MOUSE tr|A2ALL9|A2ALL9\_MOUSE sp|Q80UM3|NAA15\_MOUSE tr|G3X8Y3|G3X8Y3\_MOUSE tr|F6Q3Y1|F6Q3Y1\_MOUSE tr|E9PWN3|E9PWN3\_MOUSE tr|E9PWN2|E9PWN2\_MOUSE sp|Q8BIJ6|SYIM\_MOUSE sp|Q8R2S9|ARP8\_MOUSE tr|E9Q559|E9Q559\_MOUSE sp|Q64518-3|AT2A3\_MOUSE sp|Q91ZT5|FGD4\_MOUSE tr|E9QQ33|E9QQ33\_MOUSE tr|E9PVE7|E9PVE7\_MOUSE sp|Q6XP49-2|GLIS3\_MOUSE sp|Q69ZI1|SH3R1\_MOUSE sp|Q69ZI1-2|SH3R1\_MOUSE sp|Q69ZI1-3|SH3R1\_MOUSE tr|F8WI95|F8WI95\_MOUSE tr|A3KGG1|A3KGG1\_MOUSE tr|A3KGG4|A3KGG4\_MOUSE sp|Q8CIP5-2|DISP2\_MOUSE tr|H3BLC2|H3BLC2\_MOUSE sp|Q3TES0|IQEC3\_MOUSE sp|Q99ME6-2|AT1B4\_MOUSE sp|Q99ME6|AT1B4\_MOUSE sp|Q3U6K5|SPAT6\_MOUSE sp|Q3U6K5-2|SPAT6\_MOUSE tr|A2AED8|A2AED8\_MOUSE sp|Q3UVK0|ERMP1\_MOUSE sp|P97474-2|PITX2\_MOUSE sp|P97474-4|PITX2\_MOUSE sp|P97474-3|PITX2\_MOUSE sp|Q8C963-3|CC159\_MOUSE sp|P97474|PITX2\_MOUSE sp|Q8K1C0-3|ANGE2\_MOUSE sp|Q8K1C0-2|ANGE2\_MOUSE sp|P28229|CXG1\_MOUSE sp|O55230|RA51D\_MOUSE sp|P97474-5|PITX2\_MOUSE sp|Q9JIK9|RT34\_MOUSE sp|Q8K1C0|ANGE2\_MOUSE tr|B1ARD4|B1ARD4\_MOUSE sp|B2RY04|DOCK5\_MOUSE sp|Q8VEA8-2|RAB7B\_MOUSE sp|Q8VEA8|RAB7B\_MOUSE tr|G5E8P5|G5E8P5\_MOUSE sp|P35583|FOXA2\_MOUSE sp|Q8K083|ZN536\_MOUSE sp|P27046|MA2A1\_MOUSE sp|Q6PH08-4|ERC2\_MOUSE tr|E9PX57|E9PX57\_MOUSE sp|Q3U0X8|TACT\_MOUSE tr|D3Z7R1|D3Z7R1\_MOUSE tr|Q7TRH7|Q7TRH7\_MOUSE sp|Q9Z0G7|ZBT22\_MOUSE sp|Q8CDG3|VCIP1\_MOUSE sp|Q91WL5|CP4CA\_MOUSE tr|E9Q6D3|E9Q6D3\_MOUSE sp|Q6GYP7-2|RGPA1\_MOUSE sp|P52963|E41LA\_MOUSE sp|Q6GYP7|RGPA1\_MOUSE sp|Q6GYP7-6|RGPA1\_MOUSE sp|Q6NXZ1-2|KRBA1\_MOUSE tr|Q8VF35|Q8VF35\_MOUSE sp|Q60754|MARCO\_MOUSE sp|Q7TSI1|PKHM1\_MOUSE tr|J3QM53|J3QM53\_MOUSE sp|Q8R2W9|PANK3\_MOUSE sp|Q8CFB4|GBP5\_MOUSE sp|Q3UFY8|MRRP1\_MOUSE sp|Q99LX5|MMTA2\_MOUSE sp|Q8BN58-3|RHG28\_MOUSE sp|Q69ZX6|MOR2A\_MOUSE sp|Q8R4T1|CYS1\_MOUSE sp|Q62010|OVGP1\_MOUSE sp|Q91Y63|S13A3\_MOUSE sp|Q925F4|RNF37\_MOUSE tr|A2A4U9|A2A4U9\_MOUSE sp|Q8VHI4|CARTF\_MOUSE tr|F7CR30|F7CR30\_MOUSE tr|D3YVY7|D3YVY7\_MOUSE tr|F6S4K9|F6S4K9\_MOUSE sp|Q8VEI3|HYAL3\_MOUSE tr|E9Q642|E9Q642\_MOUSE sp|Q8BN58-2|RHG28\_MOUSE sp|Q8BN58|RHG28\_MOUSE sp|P48725-2|PCNT\_MOUSE tr|H3BJI1|H3BJI1\_MOUSE tr|H3BK47|H3BK47\_MOUSE tr|Q7TRG2|Q7TRG2\_MOUSE tr|H3BJM2|H3BJM2\_MOUSE tr|E9Q2C4|E9Q2C4\_MOUSE sp|Q9DBN5-3|LONP2\_MOUSE tr|H3BLI4|H3BLI4\_MOUSE sp|Q63886|UD11\_MOUSE tr|H3BKJ3|H3BKJ3\_MOUSE tr|Q9D007|Q9D007\_MOUSE tr|D3YV00|D3YV00\_MOUSE sp|P98195|ATP9B\_MOUSE sp|Q8BY02|NKRF\_MOUSE sp|P98195-2|ATP9B\_MOUSE sp|Q7TT23|CT194\_MOUSE tr|E9QMA6|E9QMA6\_MOUSE tr|A2A7Q8|A2A7Q8\_MOUSE sp|E9Q6I0|V2116\_MOUSE tr|Q5SZT6|Q5SZT6\_MOUSE sp|P60335|PCBP1\_MOUSE tr|Q6P9N8|Q6P9N8\_MOUSE tr|B1AUW8|B1AUW8\_MOUSE sp|Q8C0P0|GWL\_MOUSE sp|Q80WV7|SRRM3\_MOUSE sp|P08003|PDIA4\_MOUSE sp|Q9WVQ1-3|MAGI2\_MOUSE tr|D3YZ29|D3YZ29\_MOUSE sp|P12388|PAI2\_MOUSE tr|F8VQ29|F8VQ29\_MOUSE tr|E9Q3A5|E9Q3A5\_MOUSE sp|Q8CH72|TRI32\_MOUSE sp|Q9CPV7-4|ZDHC6\_MOUSE sp|Q9CPV7-2|ZDHC6\_MOUSE sp|Q9CPV7|ZDHC6\_MOUSE sp|Q9CPV7-3|ZDHC6\_MOUSE tr|G3UWX1|G3UWX1\_MOUSE sp|Q99PT9|KIF19\_MOUSE sp|P35601-2|RFC1\_MOUSE tr|G3X8Z6|G3X8Z6\_MOUSE sp|Q80ZU7|BPIB3\_MOUSE sp|P35601|RFC1\_MOUSE tr|K7N747|K7N747\_MOUSE tr|F6S1X8|F6S1X8\_MOUSE tr|Q8VGW5|Q8VGW5\_MOUSE tr|D3YTV3|D3YTV3\_MOUSE tr|E9Q996|E9Q996\_MOUSE sp|P39053-3|DYN1\_MOUSE sp|B2RPV6|MMRN1\_MOUSE tr|Q8C2W3|Q8C2W3\_MOUSE sp|Q6TEK5|VKORL\_MOUSE sp|Q9D2G5-5|SYNJ2\_MOUSE sp|P56528|CD38\_MOUSE tr|H3BL17|H3BL17\_MOUSE tr|E9PWC1|E9PWC1\_MOUSE tr|B1AUW6|B1AUW6\_MOUSE tr|E9PYE1|E9PYE1\_MOUSE tr|A0A087WNU9|A0A087WNU9\_MOUSE tr|D3Z1P8|D3Z1P8\_MOUSE tr|Q8BR10|Q8BR10\_MOUSE sp|Q9CZJ0|MPPD2\_MOUSE sp|Q9DBB9|CPN2\_MOUSE tr|B1APT1|B1APT1\_MOUSE tr|A0A087WPE6|A0A087WPE6\_MOUSE tr|A0A087WP64|A0A087WP64\_MOUSE sp|Q2NKH9|GL6D1\_MOUSE tr|A0A087WNT3|A0A087WNT3\_MOUSE sp|Q91ZG2|MPPD1\_MOUSE tr|A0A087WR90|A0A087WR90\_MOUSE sp|P62484|ABI2\_MOUSE tr|A0A087WPP8|A0A087WPP8\_MOUSE tr|J3QN60|J3QN60\_MOUSE tr|A0A087WRS3|A0A087WRS3\_MOUSE tr|Q6AXD2|Q6AXD2\_MOUSE tr|Q8C646|Q8C646\_MOUSE sp|Q9JL19-2|NCOA6\_MOUSE tr|E9Q0C0|E9Q0C0\_MOUSE tr|Q6AXH6|Q6AXH6\_MOUSE tr|E9Q637|E9Q637\_MOUSE tr|Q8VFP4|Q8VFP4\_MOUSE sp|Q6EDY6-2|LR16A\_MOUSE tr|E9Q4H6|E9Q4H6\_MOUSE tr|G3X9T2|G3X9T2\_MOUSE sp|Q60668-3|HNRPD\_MOUSE tr|G3X9W0|G3X9W0\_MOUSE sp|P16092-4|FGFR1\_MOUSE sp|Q60668-2|HNRPD\_MOUSE sp|P16092-5|FGFR1\_MOUSE sp|P16092-6|FGFR1\_MOUSE tr|E9Q9B7|E9Q9B7\_MOUSE sp|Q60668|HNRPD\_MOUSE tr|F6ZV59|F6ZV59\_MOUSE tr|G5E8G0|G5E8G0\_MOUSE sp|P16092-3|FGFR1\_MOUSE sp|Q60668-4|HNRPD\_MOUSE sp|P16092|FGFR1\_MOUSE tr|J3QN85|J3QN85\_MOUSE sp|Q7TN98|CPEB4\_MOUSE sp|P16092-2|FGFR1\_MOUSE tr|F6SHF3|F6SHF3\_MOUSE sp|A2BFL2|ZY11A\_MOUSE tr|F8WHU3|F8WHU3\_MOUSE tr|A2AEW9|A2AEW9\_MOUSE tr|A2AEW8|A2AEW8\_MOUSE sp|Q8VD04|GRAP1\_MOUSE sp|P97467|AMD\_MOUSE sp|Q3U8K7-3|SV421\_MOUSE sp|Q3U8K7|SV421\_MOUSE sp|Q80UU2|RPP38\_MOUSE tr|F8VQA4|F8VQA4\_MOUSE sp|Q3U8K7-5|SV421\_MOUSE tr|A2AJG0|A2AJG0\_MOUSE tr|F7CUD4|F7CUD4\_MOUSE tr|A2AI87|A2AI87\_MOUSE tr|D3Z1X8|D3Z1X8\_MOUSE tr|Q3UZD0|Q3UZD0\_MOUSE sp|Q99P72|RTN4\_MOUSE tr|Q8BH78|Q8BH78\_MOUSE tr|A2AGC3|A2AGC3\_MOUSE tr|Q8BHF5|Q8BHF5\_MOUSE sp|Q99P72-1|RTN4\_MOUSE sp|Q99P72-3|RTN4\_MOUSE sp|Q640M6|GDPD5\_MOUSE sp|Q2NL51|GSK3A\_MOUSE tr|D3Z7E5|D3Z7E5\_MOUSE tr|G3UX88|G3UX88\_MOUSE tr|F7D5W0|F7D5W0\_MOUSE sp|Q91X43|SH319\_MOUSE tr|Q9QZ20|Q9QZ20\_MOUSE sp|Q8VDC0|SYLM\_MOUSE sp|Q9WTL4|INSRR\_MOUSE sp|Q61179|IRF9\_MOUSE tr|E9Q8M6|E9Q8M6\_MOUSE tr|E9PZJ2|E9PZJ2\_MOUSE sp|Q810B7|SLIK5\_MOUSE tr|D3YUX1|D3YUX1\_MOUSE sp|Q810B7-2|SLIK5\_MOUSE sp|Q6NZF1|ZC11A\_MOUSE tr|F8VQA1|F8VQA1\_MOUSE sp|P41241|CSK\_MOUSE sp|Q6PB75|PAPD7\_MOUSE sp|Q6PFQ7-2|RASL2\_MOUSE sp|Q6PFQ7|RASL2\_MOUSE sp|Q9R001|ATS5\_MOUSE sp|Q5DTI6-3|KAL1L\_MOUSE sp|Q5DTI6|KAL1L\_MOUSE sp|Q91XY4|PCDG4\_MOUSE tr|B2RXA8|B2RXA8\_MOUSE sp|Q9D2H5|TRI42\_MOUSE sp|P25785|TIMP2\_MOUSE tr|Q6PI17|Q6PI17\_MOUSE tr|Q8CBB7|Q8CBB7\_MOUSE tr|E9QMQ3|E9QMQ3\_MOUSE sp|P22892|AP1G1\_MOUSE sp|Q99KW3-1|TARA\_MOUSE sp|Q9QZK2|BCAR3\_MOUSE sp|Q8CJ78|ZN628\_MOUSE tr|H3BKW9|H3BKW9\_MOUSE tr|B8JJY3|B8JJY3\_MOUSE sp|Q6P5U7|NWD2\_MOUSE tr|V9GXL7|V9GXL7\_MOUSE sp|Q3UIZ8-2|MYLK3\_MOUSE sp|Q6F3F9|GP126\_MOUSE tr|D3Z630|D3Z630\_MOUSE sp|Q3TTA7|CBLB\_MOUSE tr|Z4YKL5|Z4YKL5\_MOUSE sp|Q5Y4Y6|GSDA3\_MOUSE sp|Q3TTA7-2|CBLB\_MOUSE tr|B9EKI5|B9EKI5\_MOUSE sp|Q91WG2-1|RABE2\_MOUSE sp|Q8JZV4|RBM41\_MOUSE tr|A2AG09|A2AG09\_MOUSE sp|P48441|IDUA\_MOUSE sp|Q8JZV4-2|RBM41\_MOUSE sp|P26952|IL3RA\_MOUSE sp|P26952-2|IL3RA\_MOUSE sp|Q3TC46|PATL1\_MOUSE sp|Q8R1U1|COG4\_MOUSE sp|P49429|HPPD\_MOUSE tr|G3UWH9|G3UWH9\_MOUSE tr|G3UWX2|G3UWX2\_MOUSE tr|Q8CDK1|Q8CDK1\_MOUSE tr|F7ALS6|F7ALS6\_MOUSE sp|Q8BQM9|MD12L\_MOUSE tr|M0QWZ1|M0QWZ1\_MOUSE sp|Q8R4E6|PURG\_MOUSE sp|Q8BLD6|ANR55\_MOUSE tr|E9Q0E9|E9Q0E9\_MOUSE sp|Q9Z1R4|CF047\_MOUSE sp|Q7TS74-2|CKP2L\_MOUSE tr|E0CYM9|E0CYM9\_MOUSE sp|Q33DR3-2|DLP1\_MOUSE tr|S4R1S0|S4R1S0\_MOUSE sp|Q8BML1|MICA2\_MOUSE sp|P25233|NECD\_MOUSE tr|D3Z5N5|D3Z5N5\_MOUSE sp|Q9Z1T6|FYV1\_MOUSE sp|Q9Z1T6-1|FYV1\_MOUSE tr|F6S7W6|F6S7W6\_MOUSE sp|Q6P4S6|SIK3\_MOUSE tr|E9PU87|E9PU87\_MOUSE sp|Q6P1C6|LRIG3\_MOUSE sp|O88393|TGBR3\_MOUSE sp|Q8R2G6|CCD80\_MOUSE tr|K7N6X4|K7N6X4\_MOUSE sp|Q99K82-9|SMOX\_MOUSE sp|Q99K82-2|SMOX\_MOUSE sp|Q99K82|SMOX\_MOUSE sp|Q6PCN7|HLTF\_MOUSE tr|F7AMQ8|F7AMQ8\_MOUSE tr|E0CYL9|E0CYL9\_MOUSE tr|F8WHQ8|F8WHQ8\_MOUSE tr|Q9CZ23|Q9CZ23\_MOUSE sp|Q80W32|IQCG\_MOUSE tr|A0A087WPW5|A0A087WPW5\_MOUSE tr|A0A087WS04|A0A087WS04\_MOUSE sp|Q61595|KTN1\_MOUSE sp|Q61595-14|KTN1\_MOUSE sp|Q91V95|PTH2R\_MOUSE sp|Q61595-6|KTN1\_MOUSE tr|A0A087WQD0|A0A087WQD0\_MOUSE sp|Q61595-10|KTN1\_MOUSE sp|Q61595-16|KTN1\_MOUSE sp|Q61595-11|KTN1\_MOUSE sp|Q61595-15|KTN1\_MOUSE tr|A0A087WRS1|A0A087WRS1\_MOUSE tr|A0A087WQI3|A0A087WQI3\_MOUSE tr|A0A087WS29|A0A087WS29\_MOUSE tr|E9Q3X0|E9Q3X0\_MOUSE tr|F8VQC7|F8VQC7\_MOUSE sp|Q61595-9|KTN1\_MOUSE sp|Q61595-4|KTN1\_MOUSE sp|Q61595-7|KTN1\_MOUSE tr|A0A087WP14|A0A087WP14\_MOUSE tr|A0A087WQG4|A0A087WQG4\_MOUSE tr|A0A087WP85|A0A087WP85\_MOUSE tr|A0A087WP48|A0A087WP48\_MOUSE sp|Q61595-2|KTN1\_MOUSE tr|A0A087WQF8|A0A087WQF8\_MOUSE tr|A0A087WQ25|A0A087WQ25\_MOUSE tr|A0A087WS23|A0A087WS23\_MOUSE sp|Q3TPX4|EXOC5\_MOUSE tr|A0A087WPX0|A0A087WPX0\_MOUSE sp|Q61595-5|KTN1\_MOUSE tr|A0A087WNW3|A0A087WNW3\_MOUSE tr|E9PZ92|E9PZ92\_MOUSE sp|Q61595-3|KTN1\_MOUSE sp|Q61595-13|KTN1\_MOUSE sp|Q9EQK5|MVP\_MOUSE tr|A0A087WQQ5|A0A087WQQ5\_MOUSE sp|Q61595-12|KTN1\_MOUSE sp|Q61595-8|KTN1\_MOUSE tr|F8WHB5|F8WHB5\_MOUSE sp|Q80YT7|MYOME\_MOUSE sp|Q69ZA1-2|CDK13\_MOUSE sp|A2AQ25|SKT\_MOUSE sp|Q91YK0-2|LRC49\_MOUSE tr|E9QM16|E9QM16\_MOUSE sp|P27641|XRCC5\_MOUSE sp|Q3UMF0-2|COBL1\_MOUSE sp|Q3UMF0|COBL1\_MOUSE sp|Q3UMF0-4|COBL1\_MOUSE tr|B1AZ14|B1AZ14\_MOUSE tr|E9QKE4|E9QKE4\_MOUSE sp|P70424|ERBB2\_MOUSE tr|B1AZ15|B1AZ15\_MOUSE sp|Q3UMF0-3|COBL1\_MOUSE tr|Q3TGS9|Q3TGS9\_MOUSE sp|Q60664|LRMP\_MOUSE tr|V9GXM1|V9GXM1\_MOUSE sp|Q9EPJ9|ARFG1\_MOUSE tr|G5E880|G5E880\_MOUSE sp|Q9EPJ9-2|ARFG1\_MOUSE tr|V9GWV1|V9GWV1\_MOUSE sp|Q925N4|CLD16\_MOUSE tr|Q3TQC7|Q3TQC7\_MOUSE sp|Q9WUZ9|ENTP5\_MOUSE sp|P58771-2|TPM1\_MOUSE tr|G5E8R0|G5E8R0\_MOUSE tr|E9Q452|E9Q452\_MOUSE sp|Q8C5V0|SPT32\_MOUSE tr|Q8BP43|Q8BP43\_MOUSE tr|D3YVR0|D3YVR0\_MOUSE tr|G5E8R1|G5E8R1\_MOUSE tr|Q8BSH3|Q8BSH3\_MOUSE sp|P58771|TPM1\_MOUSE tr|G5E8R2|G5E8R2\_MOUSE tr|E9Q454|E9Q454\_MOUSE tr|E9Q455|E9Q455\_MOUSE tr|Q8K0Z5|Q8K0Z5\_MOUSE tr|D3Z2H9|D3Z2H9\_MOUSE tr|E9Q450|E9Q450\_MOUSE tr|E9Q7Q3|E9Q7Q3\_MOUSE tr|D3Z6I8|D3Z6I8\_MOUSE tr|E9Q453|E9Q453\_MOUSE tr|E9Q456|E9Q456\_MOUSE sp|P21107-2|TPM3\_MOUSE sp|P21107|TPM3\_MOUSE tr|E9Q5J9|E9Q5J9\_MOUSE sp|Q8BGV8|MID51\_MOUSE tr|F6XQN1|F6XQN1\_MOUSE sp|Q9CXS4|CENPV\_MOUSE sp|Q9D1R2|KTI12\_MOUSE tr|A2AI62|A2AI62\_MOUSE sp|A2AP18-3|PLCH2\_MOUSE sp|Q9R0N0|GALK1\_MOUSE sp|A6H8H2|DEN4C\_MOUSE tr|E9Q449|E9Q449\_MOUSE sp|A6H8H2-2|DEN4C\_MOUSE sp|Q9CZ96-2|ZCRB1\_MOUSE sp|Q9CZ96|ZCRB1\_MOUSE tr|Q05BF2|Q05BF2\_MOUSE tr|E9QAH3|E9QAH3\_MOUSE tr|A2ACY8|A2ACY8\_MOUSE tr|E9Q963|E9Q963\_MOUSE sp|Q99MP8-2|BRAP\_MOUSE sp|Q99MP8|BRAP\_MOUSE sp|Q99MP8-3|BRAP\_MOUSE tr|Q3V0P4|Q3V0P4\_MOUSE tr|A2AKW4|A2AKW4\_MOUSE sp|Q9ESG2|ROP1\_MOUSE sp|P12804|FGL2\_MOUSE sp|P54276|MSH6\_MOUSE tr|D3Z1E7|D3Z1E7\_MOUSE tr|D3Z184|D3Z184\_MOUSE sp|P62915|TF2B\_MOUSE sp|P16283|B3A3\_MOUSE sp|Q61245-2|COBA1\_MOUSE sp|Q61245|COBA1\_MOUSE tr|B1AZR7|B1AZR7\_MOUSE tr|G3XA61|G3XA61\_MOUSE sp|P70414|NAC1\_MOUSE tr|G3X9J1|G3X9J1\_MOUSE tr|Q68FL0|Q68FL0\_MOUSE tr|A0JNT1|A0JNT1\_MOUSE sp|Q99104|MYO5A\_MOUSE tr|Q8BNV8|Q8BNV8\_MOUSE tr|G5E8Y0|G5E8Y0\_MOUSE tr|D3YZ62|D3YZ62\_MOUSE tr|D3Z4J3|D3Z4J3\_MOUSE sp|B2RXR6|ANR44\_MOUSE tr|E9QNQ2|E9QNQ2\_MOUSE tr|J3QK13|J3QK13\_MOUSE tr|Q8BJS9|Q8BJS9\_MOUSE tr|Q9JMA4|Q9JMA4\_MOUSE tr|G3X9D2|G3X9D2\_MOUSE tr|E9Q287|E9Q287\_MOUSE sp|Q3UB74|TBRG1\_MOUSE sp|E9QA28|CEA16\_MOUSE tr|E9Q382|E9Q382\_MOUSE sp|Q8CB19|PHTF2\_MOUSE sp|Q91YS8|KCC1A\_MOUSE tr|E0CZ42|E0CZ42\_MOUSE sp|Q6ZQ06|CE162\_MOUSE sp|Q6TXD4-2|DNMBP\_MOUSE tr|F6R114|F6R114\_MOUSE sp|Q922G0|S2536\_MOUSE tr|D6RH79|D6RH79\_MOUSE tr|E9Q9U8|E9Q9U8\_MOUSE tr|Q3UX37|Q3UX37\_MOUSE tr|A2ACT7|A2ACT7\_MOUSE tr|F7C583|F7C583\_MOUSE tr|F6S200|F6S200\_MOUSE tr|E9Q9X4|E9Q9X4\_MOUSE sp|Q3UDE2|TTL12\_MOUSE tr|F8VPQ2|F8VPQ2\_MOUSE tr|D3Z298|D3Z298\_MOUSE tr|D3Z1F6|D3Z1F6\_MOUSE sp|P18826|KPB1\_MOUSE tr|E9Q2J6|E9Q2J6\_MOUSE tr|A2AI89|A2AI89\_MOUSE sp|P18826-2|KPB1\_MOUSE tr|D3YVW4|D3YVW4\_MOUSE tr|D3YXV4|D3YXV4\_MOUSE tr|A2AI88|A2AI88\_MOUSE tr|E9Q2G8|E9Q2G8\_MOUSE tr|A2AI91|A2AI91\_MOUSE tr|E9Q8H5|E9Q8H5\_MOUSE sp|Q3UHH1-3|ZSWM8\_MOUSE sp|Q3UHH1|ZSWM8\_MOUSE sp|Q3UHH1-2|ZSWM8\_MOUSE sp|Q3V0Y1|SMEK3\_MOUSE tr|Q8VCD0|Q8VCD0\_MOUSE sp|P35918|VGFR2\_MOUSE sp|Q3U7U4|CA162\_MOUSE tr|F6V4L0|F6V4L0\_MOUSE sp|Q8BGQ6-2|EFC14\_MOUSE tr|Q6PCQ6|Q6PCQ6\_MOUSE sp|Q8BGQ6|EFC14\_MOUSE sp|Q9QY81|PO210\_MOUSE tr|E9PYI5|E9PYI5\_MOUSE tr|D3Z624|D3Z624\_MOUSE sp|A0JLX3|SMK4A\_MOUSE sp|P15331-2|PERI\_MOUSE sp|P15331-3|PERI\_MOUSE tr|G5E846|G5E846\_MOUSE tr|G3X981|G3X981\_MOUSE sp|P15331|PERI\_MOUSE tr|A0A087WQM4|A0A087WQM4\_MOUSE sp|Q8BGX3|LRTM2\_MOUSE sp|Q571E4|GALNS\_MOUSE tr|F6VCM8|F6VCM8\_MOUSE sp|Q99PV5|BHE41\_MOUSE sp|Q9JIT1|RNF32\_MOUSE tr|E9PZ86|E9PZ86\_MOUSE sp|Q9QXK9|SH22A\_MOUSE tr|E0CZ33|E0CZ33\_MOUSE tr|E9PWJ4|E9PWJ4\_MOUSE sp|Q8VD63|TSYL4\_MOUSE tr|F6URG7|F6URG7\_MOUSE tr|A0A087WPF7|A0A087WPF7\_MOUSE sp|Q9CWT6|DDX28\_MOUSE tr|D3YWY4|D3YWY4\_MOUSE tr|D3Z6T4|D3Z6T4\_MOUSE sp|Q925T6-3|GRIP1\_MOUSE tr|D3YZL8|D3YZL8\_MOUSE tr|A2A7B5|A2A7B5\_MOUSE tr|H7BX90|H7BX90\_MOUSE tr|H7BX09|H7BX09\_MOUSE sp|Q925T6-2|GRIP1\_MOUSE sp|Q925T6|GRIP1\_MOUSE tr|Q6GQT7|Q6GQT7\_MOUSE sp|Q9CU62|SMC1A\_MOUSE sp|Q6PCM1-2|KDM3A\_MOUSE sp|Q9EPL8|IPO7\_MOUSE sp|Q7TMB8|CYFP1\_MOUSE tr|A9C448|A9C448\_MOUSE sp|Q9D4D7|CC50C\_MOUSE sp|P07310|KCRM\_MOUSE tr|Q497Z0|Q497Z0\_MOUSE sp|Q80UK0|SESD1\_MOUSE tr|D6RFR4|D6RFR4\_MOUSE sp|Q8CIE2-3|ZMIZ2\_MOUSE tr|D6RE37|D6RE37\_MOUSE sp|Q8CIE2-2|ZMIZ2\_MOUSE tr|D6RCX3|D6RCX3\_MOUSE sp|Q925E0|SNTG2\_MOUSE sp|Q8CIE2|ZMIZ2\_MOUSE sp|P97481|EPAS1\_MOUSE tr|H9H9S3|H9H9S3\_MOUSE sp|Q9R0G7|ZEB2\_MOUSE tr|A2AMW3|A2AMW3\_MOUSE sp|Q99JY4|TRABD\_MOUSE tr|A0A075B5S3|A0A075B5S3\_MOUSE tr|G3X9N0|G3X9N0\_MOUSE tr|B7ZMQ5|B7ZMQ5\_MOUSE tr|Q4QQK7|Q4QQK7\_MOUSE tr|A2AW13|A2AW13\_MOUSE sp|Q5QD13|TAAR6\_MOUSE tr|Q7TRP1|Q7TRP1\_MOUSE tr|F6R2B3|F6R2B3\_MOUSE tr|Q8VF03|Q8VF03\_MOUSE sp|Q922R5-2|P4R3B\_MOUSE tr|Q9JK62|Q9JK62\_MOUSE tr|B8A5X0|B8A5X0\_MOUSE sp|Q8BYL4|SYYM\_MOUSE tr|E9PX65|E9PX65\_MOUSE sp|P82347|SGCD\_MOUSE sp|O88888|APBA3\_MOUSE sp|Q69Z66|MYSM1\_MOUSE sp|Q8C4X1-3|CENPX\_MOUSE sp|O09126|SEM4D\_MOUSE sp|Q62136|PTN21\_MOUSE tr|G5E8J4|G5E8J4\_MOUSE sp|Q99JY0|ECHB\_MOUSE sp|Q8BMB0-2|EMSY\_MOUSE sp|Q9JM73|SRF\_MOUSE tr|Q3TG86|Q3TG86\_MOUSE tr|G5E8H1|G5E8H1\_MOUSE tr|S4R290|S4R290\_MOUSE sp|P23819-4|GRIA2\_MOUSE sp|Q6ZPF3-4|TIAM2\_MOUSE sp|P23819|GRIA2\_MOUSE tr|H7BX33|H7BX33\_MOUSE sp|Q8R092|CA043\_MOUSE sp|Q80TG9|LRFN2\_MOUSE sp|Q9QUK6|TLR4\_MOUSE tr|Q8VFA2|Q8VFA2\_MOUSE tr|Q3UUV9|Q3UUV9\_MOUSE sp|E9Q7F2|RN169\_MOUSE sp|O35111|KCNK3\_MOUSE sp|Q8R0S2-2|IQEC1\_MOUSE tr|Q3U4X8|Q3U4X8\_MOUSE sp|Q8CIG0-2|DEP1A\_MOUSE sp|Q8CIG0|DEP1A\_MOUSE tr|D3Z6J1|D3Z6J1\_MOUSE sp|Q8R373|CLMP\_MOUSE sp|O55112|AFF2\_MOUSE tr|E9PWD5|E9PWD5\_MOUSE tr|E9Q5F4|E9Q5F4\_MOUSE sp|Q9JK88|SPI2\_MOUSE tr|E9Q606|E9Q606\_MOUSE sp|Q9CPV3|RM42\_MOUSE sp|Q8BX22-2|SALL4\_MOUSE sp|Q9CWE6|OOEP\_MOUSE sp|Q925J9-4|MED1\_MOUSE sp|Q925J9|MED1\_MOUSE sp|Q8C0V0|TLK1\_MOUSE sp|Q3U829|AP5Z1\_MOUSE sp|Q3UYG1|CC160\_MOUSE tr|Q80Y34|Q80Y34\_MOUSE tr|D3Z5I6|D3Z5I6\_MOUSE tr|A4GZ26|A4GZ26\_MOUSE sp|Q5DU25|IQEC2\_MOUSE tr|E9QAD8|E9QAD8\_MOUSE sp|Q8R370|USBP1\_MOUSE tr|F7C082|F7C082\_MOUSE sp|Q8CI75-2|DI3L2\_MOUSE sp|Q8BG21|FLOWR\_MOUSE sp|Q8CI75|DI3L2\_MOUSE tr|A6PWX1|A6PWX1\_MOUSE tr|G3X9R0|G3X9R0\_MOUSE tr|S4R1B9|S4R1B9\_MOUSE tr|A0A067XG50|A0A067XG50\_MOUSE sp|Q80UV9|TAF1\_MOUSE tr|D3YZK4|D3YZK4\_MOUSE sp|Q80UV9-3|TAF1\_MOUSE sp|Q8K299-2|SCAR5\_MOUSE sp|Q8K299-3|SCAR5\_MOUSE sp|Q8K299|SCAR5\_MOUSE sp|P20693-3|FCER2\_MOUSE sp|P20693-2|FCER2\_MOUSE sp|P20693|FCER2\_MOUSE tr|G5E836|G5E836\_MOUSE sp|Q924D0|RT4I1\_MOUSE sp|Q9D6X5|S52A3\_MOUSE sp|Q8R418-2|DICER\_MOUSE sp|Q9ESP1|SDF2L\_MOUSE tr|Q6XZL8|Q6XZL8\_MOUSE tr|F7CVL0|F7CVL0\_MOUSE sp|P23188|FURIN\_MOUSE tr|Q3UW98|Q3UW98\_MOUSE sp|Q80WR5|CA174\_MOUSE sp|Q8CH25-2|SLTM\_MOUSE sp|Q8CH25|SLTM\_MOUSE sp|P08122|CO4A2\_MOUSE tr|E9Q405|E9Q405\_MOUSE sp|Q9JMH9-7|MY18A\_MOUSE sp|Q9JMH9-5|MY18A\_MOUSE sp|Q9JMH9-2|MY18A\_MOUSE tr|E9QA74|E9QA74\_MOUSE sp|Q9JKX4|AATF\_MOUSE sp|Q9JKX4-3|AATF\_MOUSE tr|E0CYV9|E0CYV9\_MOUSE sp|Q01320|TOP2A\_MOUSE sp|Q9JLZ6|HIC2\_MOUSE sp|Q60817|NACA\_MOUSE sp|Q9JLZ6-2|HIC2\_MOUSE sp|Q9WVQ0|PMFBP\_MOUSE tr|Q91XZ4|Q91XZ4\_MOUSE sp|Q91WQ3|SYYC\_MOUSE tr|A2A7S7|A2A7S7\_MOUSE sp|Q9Z188-3|DYR1B\_MOUSE sp|Q9Z188|DYR1B\_MOUSE sp|Q9Z188-2|DYR1B\_MOUSE sp|Q9QZN1|FXL17\_MOUSE tr|H3BK37|H3BK37\_MOUSE sp|Q91VD9|NDUS1\_MOUSE tr|Q0VAV1|Q0VAV1\_MOUSE sp|Q80W88|HOMEZ\_MOUSE sp|Q80W88-2|HOMEZ\_MOUSE tr|E9PYA2|E9PYA2\_MOUSE tr|H3BL46|H3BL46\_MOUSE tr|B9EJW5|B9EJW5\_MOUSE sp|Q8K1J5|SDE2\_MOUSE tr|F6S4J6|F6S4J6\_MOUSE sp|Q9WTN3-4|SRBP1\_MOUSE sp|Q9WTN3-2|SRBP1\_MOUSE sp|Q64429|CP1B1\_MOUSE tr|G5E837|G5E837\_MOUSE sp|Q8VIH9|UR2R\_MOUSE sp|Q497N6|DDC8\_MOUSE tr|E9PYS0|E9PYS0\_MOUSE tr|Q8CBP6|Q8CBP6\_MOUSE sp|Q922Y0|DYRK3\_MOUSE sp|Q9JJX8|ST32B\_MOUSE sp|Q64467|G3PT\_MOUSE sp|Q8BH32|SUSD4\_MOUSE sp|Q8BH32-2|SUSD4\_MOUSE sp|O70481|UBR1\_MOUSE sp|O54834-3|RHG06\_MOUSE sp|O54834-4|RHG06\_MOUSE sp|O54834|RHG06\_MOUSE sp|Q570Y9-3|DPTOR\_MOUSE sp|Q570Y9|DPTOR\_MOUSE tr|B2ZRS5|B2ZRS5\_MOUSE sp|Q3V0L5|LRC43\_MOUSE sp|Q3V0L5-2|LRC43\_MOUSE sp|Q921S7|RM37\_MOUSE sp|Q571H0|NPA1P\_MOUSE tr|E9PU96|E9PU96\_MOUSE sp|Q9JKF1|IQGA1\_MOUSE tr|E9Q6A2|E9Q6A2\_MOUSE sp|Q9WUH7|SEM4G\_MOUSE tr|F6WMX7|F6WMX7\_MOUSE tr|F7BXQ1|F7BXQ1\_MOUSE tr|Q3TTW5|Q3TTW5\_MOUSE tr|E9Q6M7|E9Q6M7\_MOUSE sp|Q8BHI9|NIM1\_MOUSE sp|Q5DU28-4|PCX2\_MOUSE tr|F6X605|F6X605\_MOUSE sp|Q80YV4-2|PANK4\_MOUSE sp|Q5DU28-2|PCX2\_MOUSE sp|Q80YV4|PANK4\_MOUSE tr|G3UYA7|G3UYA7\_MOUSE sp|Q6P9P8|DEN2C\_MOUSE sp|Q9EQ32-3|BCAP\_MOUSE tr|E9Q4E4|E9Q4E4\_MOUSE ENSEMBL:ENSBTAP00000024146 tr|E0CXB2|E0CXB2\_MOUSE sp|Q6P9P8-2|DEN2C\_MOUSE sp|Q7TQK1-2|INT7\_MOUSE sp|Q9D7Y9|SLX4I\_MOUSE tr|Q8VET5|Q8VET5\_MOUSE sp|P47810|WEE1\_MOUSE tr|I7HLV5|I7HLV5\_MOUSE sp|Q99NE9|PBX4\_MOUSE tr|I7HPY1|I7HPY1\_MOUSE tr|D3Z318|D3Z318\_MOUSE sp|Q9D8T0|FAM3A\_MOUSE tr|I7HJS7|I7HJS7\_MOUSE tr|Q3SWS4|Q3SWS4\_MOUSE sp|Q9ESL8|FGF16\_MOUSE sp|Q63880-2|EST3A\_MOUSE sp|Q63880|EST3A\_MOUSE sp|Q3TMH2|SCRN3\_MOUSE tr|E9Q3H4|E9Q3H4\_MOUSE tr|A2AN91|A2AN91\_MOUSE sp|P40142|TKT\_MOUSE tr|V9GXW1|V9GXW1\_MOUSE sp|Q9DC22|DCAF6\_MOUSE sp|Q9QZR9-2|CO4A4\_MOUSE tr|F6UNU0|F6UNU0\_MOUSE sp|Q9QZR9|CO4A4\_MOUSE tr|F6U5G1|F6U5G1\_MOUSE ENSEMBL:ENSBTAP00000023055 sp|P0CB42|ALKB1\_MOUSE sp|Q9QWV9|CCNT1\_MOUSE sp|P97325|SIAT6\_MOUSE tr|A2AT63|A2AT63\_MOUSE sp|Q8BJL1|FBX30\_MOUSE sp|Q9Z0S2|LHX2\_MOUSE sp|Q9D1H0|RITA1\_MOUSE tr|V9GX98|V9GX98\_MOUSE tr|H3BKD9|H3BKD9\_MOUSE tr|D3Z230|D3Z230\_MOUSE sp|Q9JHP7|KDEL1\_MOUSE tr|D3YUL8|D3YUL8\_MOUSE sp|Q9EPW0|INP4A\_MOUSE tr|B1AWL4|B1AWL4\_MOUSE sp|Q9JHP7-2|KDEL1\_MOUSE tr|E9Q9A0|E9Q9A0\_MOUSE tr|B1AWL5|B1AWL5\_MOUSE tr|D3YTM1|D3YTM1\_MOUSE tr|D3YUD3|D3YUD3\_MOUSE sp|Q80VC6|TSAP1\_MOUSE sp|Q8BRF7-3|SCFD1\_MOUSE sp|Q9JLG8|CAN15\_MOUSE tr|A0A087WQ61|A0A087WQ61\_MOUSE sp|Q8BRF7|SCFD1\_MOUSE sp|G3XA57-2|RFIP2\_MOUSE sp|G3XA57|RFIP2\_MOUSE tr|G3UW57|G3UW57\_MOUSE sp|Q9Z1P5|CD320\_MOUSE tr|F6QRE9|F6QRE9\_MOUSE tr|F8WIN6|F8WIN6\_MOUSE sp|Q9Z0R4|ITSN1\_MOUSE tr|E9Q0N0|E9Q0N0\_MOUSE sp|Q99L04|DHRS1\_MOUSE sp|Q8BMA3|CNKR3\_MOUSE sp|O35696|SIA8B\_MOUSE tr|E9Q554|E9Q554\_MOUSE tr|F6RXI4|F6RXI4\_MOUSE sp|Q6I6G8-2|HECW2\_MOUSE tr|S4R1S6|S4R1S6\_MOUSE tr|A0A087WQZ2|A0A087WQZ2\_MOUSE sp|P01582|IL1A\_MOUSE tr|B2KFA9|B2KFA9\_MOUSE sp|Q3UHL1|CAMKV\_MOUSE sp|Q8C432|RN182\_MOUSE tr|B2KFA8|B2KFA8\_MOUSE tr|F6SLJ2|F6SLJ2\_MOUSE sp|Q99LH9|3BP5L\_MOUSE sp|Q9CUL5|IQCA1\_MOUSE tr|E9QMY8|E9QMY8\_MOUSE sp|Q8K114-2|INT9\_MOUSE sp|Q8C013|TPBGL\_MOUSE tr|A2ADJ4|A2ADJ4\_MOUSE tr|G3UZ07|G3UZ07\_MOUSE tr|G3UYG0|G3UYG0\_MOUSE sp|Q8C735-3|LIN9\_MOUSE sp|Q8C735|LIN9\_MOUSE sp|P07147|TYRP1\_MOUSE sp|Q8C735-2|LIN9\_MOUSE sp|Q8R0N6-2|HOT\_MOUSE tr|H9H9R8|H9H9R8\_MOUSE tr|H3BJT3|H3BJT3\_MOUSE sp|Q7TNF0|DOC2A\_MOUSE tr|D3YYV2|D3YYV2\_MOUSE sp|Q80TS8-3|SE1L3\_MOUSE tr|E9Q3S2|E9Q3S2\_MOUSE tr|F6RUV8|F6RUV8\_MOUSE sp|Q8C6D4|BEND5\_MOUSE sp|O88673|DGKA\_MOUSE sp|Q8VEB6|RNZ1\_MOUSE tr|Q91Z40|Q91Z40\_MOUSE sp|Q9QZ04|MAGL2\_MOUSE tr|B2RQV3|B2RQV3\_MOUSE sp|Q7TSI0|ZNF12\_MOUSE sp|Q8R1F9|RPP40\_MOUSE sp|Q9JJ11-2|TACC3\_MOUSE tr|E9QKE8|E9QKE8\_MOUSE tr|Q6NV52|Q6NV52\_MOUSE sp|Q7TSI0-2|ZNF12\_MOUSE sp|Q8VFB9|OL183\_MOUSE tr|Q99LH8|Q99LH8\_MOUSE sp|Q61575|FOXN1\_MOUSE sp|Q9JJ11|TACC3\_MOUSE sp|Q8C7W7-3|DCR1B\_MOUSE sp|Q8C7W7|DCR1B\_MOUSE tr|E9PX93|E9PX93\_MOUSE sp|Q60929-2|MEF2A\_MOUSE sp|Q8C119|NDNF\_MOUSE sp|Q60929-3|MEF2A\_MOUSE tr|E9PZY5|E9PZY5\_MOUSE tr|Q8C4N0|Q8C4N0\_MOUSE sp|Q9JJK5|HERP1\_MOUSE sp|Q8BND4|DX26B\_MOUSE sp|P22599|A1AT2\_MOUSE sp|Q9D5A0|SPESP\_MOUSE sp|Q3UR85-3|MRF\_MOUSE sp|Q3UR85|MRF\_MOUSE sp|Q505D1|ANR28\_MOUSE sp|G5E870|TRIPC\_MOUSE tr|A0A087WNZ7|A0A087WNZ7\_MOUSE tr|Q8VFM4|Q8VFM4\_MOUSE tr|Q3UP14|Q3UP14\_MOUSE sp|Q7TPS5-2|C2CD5\_MOUSE tr|Q8VFX6|Q8VFX6\_MOUSE sp|Q6P7F1-3|MPP4\_MOUSE tr|K3W4N9|K3W4N9\_MOUSE tr|D3Z0G8|D3Z0G8\_MOUSE tr|A0A087WQU9|A0A087WQU9\_MOUSE sp|Q6P7F1|MPP4\_MOUSE tr|E9Q0W7|E9Q0W7\_MOUSE sp|Q8CJ69|BMPER\_MOUSE tr|A2A838|A2A838\_MOUSE tr|F6XV41|F6XV41\_MOUSE tr|Q9R2A2|Q9R2A2\_MOUSE sp|O08785-2|CLOCK\_MOUSE sp|O08785|CLOCK\_MOUSE tr|A2A839|A2A839\_MOUSE tr|Q7TR76|Q7TR76\_MOUSE tr|F7BTY2|F7BTY2\_MOUSE sp|O89093|CCL20\_MOUSE tr|F8WHA7|F8WHA7\_MOUSE sp|O89093-2|CCL20\_MOUSE tr|A2ARZ0|A2ARZ0\_MOUSE tr|Q8C689|Q8C689\_MOUSE tr|E9Q5D6|E9Q5D6\_MOUSE sp|P69566|RANB9\_MOUSE sp|P69566-2|RANB9\_MOUSE tr|A2AG51|A2AG51\_MOUSE sp|A2AG50|MA7D2\_MOUSE tr|F6QKX8|F6QKX8\_MOUSE sp|A2AG50-2|MA7D2\_MOUSE sp|Q6VVW9|CP2R1\_MOUSE tr|H3BK48|H3BK48\_MOUSE tr|H3BK44|H3BK44\_MOUSE sp|Q8CI08-2|SLAI2\_MOUSE sp|Q8CI08|SLAI2\_MOUSE sp|Q80W00|PP1RA\_MOUSE sp|Q80W00-2|PP1RA\_MOUSE tr|Q922A3|Q922A3\_MOUSE sp|Q3UQ84|SYTM\_MOUSE tr|E9Q7C4|E9Q7C4\_MOUSE sp|Q8K1I7|WIPF1\_MOUSE sp|O35914|BNC1\_MOUSE tr|F8VPY0|F8VPY0\_MOUSE sp|Q8BRF7-2|SCFD1\_MOUSE tr|A2AK57|A2AK57\_MOUSE sp|P23780|BGAL\_MOUSE sp|Q60591-4|NFAC2\_MOUSE tr|J3QN61|J3QN61\_MOUSE sp|Q6Q899-4|DDX58\_MOUSE sp|Q8K330|SSH3\_MOUSE sp|Q9CXJ4|ABCB8\_MOUSE sp|Q8K330-2|SSH3\_MOUSE sp|Q6Q899|DDX58\_MOUSE sp|Q6Q899-3|DDX58\_MOUSE sp|Q80Z38-3|SHAN2\_MOUSE tr|A0A087WSM1|A0A087WSM1\_MOUSE sp|Q3TY65|ICA1L\_MOUSE sp|Q80ZA4-2|PKHL1\_MOUSE tr|B1AU74|B1AU74\_MOUSE sp|Q9CWP6|MSPD2\_MOUSE sp|Q9CWP6-3|MSPD2\_MOUSE sp|Q9CWP6-2|MSPD2\_MOUSE tr|A2A473|A2A473\_MOUSE tr|E9Q0B9|E9Q0B9\_MOUSE tr|A2A472|A2A472\_MOUSE sp|Q9EPK5-2|WWTR1\_MOUSE tr|Q3UMA3|Q3UMA3\_MOUSE sp|Q99LI8|HGS\_MOUSE tr|H3BKF8|H3BKF8\_MOUSE sp|A6H611|MIPEP\_MOUSE sp|P50220|NKX21\_MOUSE sp|Q91W97|HKDC1\_MOUSE sp|Q9CSU0-3|RPR1B\_MOUSE sp|Q9CSU0-2|RPR1B\_MOUSE sp|Q9CSU0|RPR1B\_MOUSE sp|Q7TMR0|PCP\_MOUSE sp|Q8BXL9-4|IFFO1\_MOUSE sp|Q6L8S8|B4GN3\_MOUSE sp|Q3URE9|LIGO2\_MOUSE sp|Q8R0Y6|AL1L1\_MOUSE tr|F7CZG3|F7CZG3\_MOUSE sp|Q6P5G3|MBTD1\_MOUSE tr|G3UZM6|G3UZM6\_MOUSE sp|Q8CG48|SMC2\_MOUSE sp|Q8BGF7-4|PAN2\_MOUSE sp|Q8BGF7-3|PAN2\_MOUSE sp|Q8BGF7|PAN2\_MOUSE sp|Q8BGF7-2|PAN2\_MOUSE tr|D3YTS4|D3YTS4\_MOUSE sp|Q9JHS3|LTOR2\_MOUSE sp|Q91YE7-2|RBM5\_MOUSE sp|Q91YE7|RBM5\_MOUSE tr|Q91XX4|Q91XX4\_MOUSE sp|Q08890|IDS\_MOUSE tr|E9Q7A8|E9Q7A8\_MOUSE sp|P51683|CCR2\_MOUSE sp|O88962|CP8B1\_MOUSE tr|F6XV02|F6XV02\_MOUSE tr|K7N6V1|K7N6V1\_MOUSE tr|G3X8T9|G3X8T9\_MOUSE sp|Q8C551|R51A1\_MOUSE tr|S4R2J8|S4R2J8\_MOUSE tr|S4R270|S4R270\_MOUSE tr|S4R1B8|S4R1B8\_MOUSE sp|D3Z6Q9|BIN2\_MOUSE tr|D3Z709|D3Z709\_MOUSE sp|Q9D415-2|DLGP1\_MOUSE sp|Q9D415|DLGP1\_MOUSE sp|Q9D415-4|DLGP1\_MOUSE sp|Q810I0|VP37D\_MOUSE tr|Q3UVU8|Q3UVU8\_MOUSE tr|E9QAR5|E9QAR5\_MOUSE tr|D3Z508|D3Z508\_MOUSE sp|Q8BHK0|PNMA2\_MOUSE sp|P70188|KIFA3\_MOUSE sp|P70188-2|KIFA3\_MOUSE tr|H3BJU6|H3BJU6\_MOUSE sp|Q9CZR3|TM40L\_MOUSE tr|D3YY29|D3YY29\_MOUSE sp|Q6PFE3|RA54B\_MOUSE sp|Q9JIA7|SPHK2\_MOUSE sp|Q66JT1|LRC8E\_MOUSE tr|E9QNP3|E9QNP3\_MOUSE sp|Q8CBF3-2|EPHB1\_MOUSE sp|Q7M720|T2R13\_MOUSE sp|Q8CBF3|EPHB1\_MOUSE sp|P52633|STAT6\_MOUSE sp|Q7TSK7|ATL2\_MOUSE tr|A2BG95|A2BG95\_MOUSE tr|Q52KB8|Q52KB8\_MOUSE sp|Q62233|SIX3\_MOUSE tr|F7D143|F7D143\_MOUSE tr|K7N5U4|K7N5U4\_MOUSE sp|O70348|DXO\_MOUSE tr|Q9D4E9|Q9D4E9\_MOUSE tr|G3UZ39|G3UZ39\_MOUSE tr|Q8CIE4|Q8CIE4\_MOUSE tr|G3UZV8|G3UZV8\_MOUSE sp|Q8C7K6|PCYXL\_MOUSE tr|Q3TET1|Q3TET1\_MOUSE sp|Q9QXK3-4|COPG2\_MOUSE tr|Q3UI47|Q3UI47\_MOUSE sp|P28741|KIF3A\_MOUSE sp|Q9QXK3|COPG2\_MOUSE sp|Q6P9R1|DDX51\_MOUSE sp|Q8K268|ABCF3\_MOUSE tr|B1AU25|B1AU25\_MOUSE sp|Q8K558|TRML1\_MOUSE tr|F8VPL5|F8VPL5\_MOUSE sp|Q9D024|CCD47\_MOUSE sp|Q8K558-2|TRML1\_MOUSE sp|Q9D024-2|CCD47\_MOUSE sp|P47713|PA24A\_MOUSE tr|Q9DBX5|Q9DBX5\_MOUSE sp|O70172|PI42A\_MOUSE tr|D3YWS8|D3YWS8\_MOUSE sp|Q8CBY3|LENG8\_MOUSE tr|D3YXV8|D3YXV8\_MOUSE tr|Q8VF52|Q8VF52\_MOUSE sp|Q6DFV8|VWDE\_MOUSE tr|B2RXC8|B2RXC8\_MOUSE sp|Q62191|RO52\_MOUSE sp|Q9EPC2|FGF23\_MOUSE sp|Q5SRX1|TM1L2\_MOUSE sp|Q5SRX1-2|TM1L2\_MOUSE tr|F6RQC1|F6RQC1\_MOUSE tr|Q5SXA4|Q5SXA4\_MOUSE tr|E9Q1P2|E9Q1P2\_MOUSE sp|Q8CCF0-3|PRP31\_MOUSE sp|P14432|HA1T\_MOUSE tr|F6QFD1|F6QFD1\_MOUSE tr|Q05A75|Q05A75\_MOUSE tr|F6TIX5|F6TIX5\_MOUSE sp|Q8CCF0-2|PRP31\_MOUSE sp|Q8CCF0|PRP31\_MOUSE sp|Q1LZI2|S35F3\_MOUSE sp|Q8R104-2|SIR3\_MOUSE sp|Q8R104|SIR3\_MOUSE sp|Q8BZN6-3|DOC10\_MOUSE sp|Q8BZN6-2|DOC10\_MOUSE tr|E9Q1R5|E9Q1R5\_MOUSE tr|Q62457|Q62457\_MOUSE tr|J3QN84|J3QN84\_MOUSE tr|J3QMI6|J3QMI6\_MOUSE tr|J3QN17|J3QN17\_MOUSE tr|J3QM72|J3QM72\_MOUSE tr|Q497R7|Q497R7\_MOUSE tr|J3QPK8|J3QPK8\_MOUSE tr|Q62461|Q62461\_MOUSE tr|J3KMI0|J3KMI0\_MOUSE tr|J3QM67|J3QM67\_MOUSE tr|Q5FWD5|Q5FWD5\_MOUSE tr|J3QPJ2|J3QPJ2\_MOUSE tr|A0A087WQ19|A0A087WQ19\_MOUSE tr|Q9D9T3|Q9D9T3\_MOUSE tr|J3QM57|J3QM57\_MOUSE tr|Q78EE2|Q78EE2\_MOUSE tr|J3QJZ2|J3QJZ2\_MOUSE tr|J3QMK7|J3QMK7\_MOUSE tr|J3QME2|J3QME2\_MOUSE tr|J3KMI7|J3KMI7\_MOUSE tr|J3KML6|J3KML6\_MOUSE tr|J3QK38|J3QK38\_MOUSE tr|Q149W4|Q149W4\_MOUSE tr|J3QPZ4|J3QPZ4\_MOUSE tr|Q5M8R5|Q5M8R5\_MOUSE sp|Q9Z0H4-11|CELF2\_MOUSE tr|S4R2U7|S4R2U7\_MOUSE tr|E9QA47|E9QA47\_MOUSE sp|Q9DCJ9|NPL\_MOUSE sp|Q3U3C9|GSE1\_MOUSE tr|F6VCI2|F6VCI2\_MOUSE sp|Q3U3C9-3|GSE1\_MOUSE sp|Q791T5-2|MTCH1\_MOUSE sp|Q791T5|MTCH1\_MOUSE sp|P83093|STIM2\_MOUSE sp|Q3U3C9-2|GSE1\_MOUSE tr|F6VP19|F6VP19\_MOUSE tr|I1E4X8|I1E4X8\_MOUSE sp|Q99N87|RT05\_MOUSE sp|Q9ESX4|NO40\_MOUSE sp|Q9ESX4-2|NO40\_MOUSE sp|O54774|AP3D1\_MOUSE sp|P59267|ZDHC2\_MOUSE sp|Q8CJ78-2|ZN628\_MOUSE sp|P31315|GSX1\_MOUSE tr|D3Z3U4|D3Z3U4\_MOUSE sp|Q6YCH2|TDPZ4\_MOUSE tr|Q8C2T6|Q8C2T6\_MOUSE sp|P33611|DPOA2\_MOUSE sp|P0C605|KGP1\_MOUSE tr|H9KUY5|H9KUY5\_MOUSE sp|P98063|BMP1\_MOUSE sp|Q3UR85-5|MRF\_MOUSE tr|J3QN14|J3QN14\_MOUSE sp|P42866-18|OPRM\_MOUSE tr|E9PVC1|E9PVC1\_MOUSE tr|Q5SVV3|Q5SVV3\_MOUSE sp|P03893|NU2M\_MOUSE tr|Q9MD59|Q9MD59\_MOUSE sp|Q3U2A8|SYVM\_MOUSE tr|Q9D451|Q9D451\_MOUSE sp|Q5XF89-2|AT133\_MOUSE sp|Q5XF89|AT133\_MOUSE tr|B7ZNA5|B7ZNA5\_MOUSE sp|Q3UES3|TNKS2\_MOUSE sp|Q8R3B7|BRD8\_MOUSE sp|Q80US4|ARP5\_MOUSE tr|V9GXE4|V9GXE4\_MOUSE sp|Q923E4|SIR1\_MOUSE tr|Q3UNI1|Q3UNI1\_MOUSE sp|Q80UK8|INT2\_MOUSE tr|A2ALV6|A2ALV6\_MOUSE tr|A2ALV8|A2ALV8\_MOUSE sp|Q8BI84-2|MIA3\_MOUSE tr|J3KMG3|J3KMG3\_MOUSE sp|O08746|MATN2\_MOUSE sp|O08746-2|MATN2\_MOUSE sp|Q64511|TOP2B\_MOUSE tr|Q8R2C2|Q8R2C2\_MOUSE sp|Q3THK7|GUAA\_MOUSE tr|G3UZZ6|G3UZZ6\_MOUSE tr|Q91WH7|Q91WH7\_MOUSE tr|G3UWP0|G3UWP0\_MOUSE sp|P48381-2|RFX3\_MOUSE tr|E9QNX7|E9QNX7\_MOUSE sp|Q9ERA6|TFP11\_MOUSE tr|A2AIV6|A2AIV6\_MOUSE sp|Q8BP86-3|SNPC4\_MOUSE sp|Q8BP86-2|SNPC4\_MOUSE sp|Q8BP86|SNPC4\_MOUSE sp|P60824|CIRBP\_MOUSE tr|D3YU80|D3YU80\_MOUSE tr|D6RDC2|D6RDC2\_MOUSE tr|K4DI65|K4DI65\_MOUSE tr|E9Q5B6|E9Q5B6\_MOUSE tr|B1AT10|B1AT10\_MOUSE sp|Q64464|CP3AD\_MOUSE sp|Q6NZQ0|CP093\_MOUSE tr|D3Z7X0|D3Z7X0\_MOUSE sp|Q6XUX1-2|DUSTY\_MOUSE tr|A0A087WP69|A0A087WP69\_MOUSE sp|Q924M7|MPI\_MOUSE tr|E9Q4Z9|E9Q4Z9\_MOUSE tr|Q91WD3|Q91WD3\_MOUSE tr|E9PZW6|E9PZW6\_MOUSE sp|Q6XUX1|DUSTY\_MOUSE tr|E9Q6V0|E9Q6V0\_MOUSE sp|Q6XUX1-3|DUSTY\_MOUSE sp|Q5PT54|NTCP5\_MOUSE sp|Q8BMG7|RBGPR\_MOUSE sp|Q6PIU9|YJ005\_MOUSE tr|F6ZCF0|F6ZCF0\_MOUSE sp|P50228|CXCL5\_MOUSE sp|Q68FG3|SPT2\_MOUSE P35900 sp|Q9CY28|GTPB8\_MOUSE sp|Q9CY28-2|GTPB8\_MOUSE tr|A6PW28|A6PW28\_MOUSE tr|H7BWZ4|H7BWZ4\_MOUSE tr|B0QZW1|B0QZW1\_MOUSE tr|G5E863|G5E863\_MOUSE sp|Q80XD9|CLC2E\_MOUSE tr|E9PXJ4|E9PXJ4\_MOUSE sp|Q501J6-2|DDX17\_MOUSE sp|Q9DBX3-2|SUSD2\_MOUSE sp|Q9DBX3|SUSD2\_MOUSE sp|Q8CIH5|PLCG2\_MOUSE sp|O54982|KCNU1\_MOUSE tr|G3X9P7|G3X9P7\_MOUSE tr|Q5NCU0|Q5NCU0\_MOUSE sp|Q64018|GLRA1\_MOUSE tr|Q3V117|Q3V117\_MOUSE sp|Q64018-2|GLRA1\_MOUSE sp|Q8CCS2|F155A\_MOUSE tr|Q8R277|Q8R277\_MOUSE sp|Q8BU51|BPIB6\_MOUSE sp|Q149F5|TMM71\_MOUSE sp|P86044|ANO9\_MOUSE sp|Q8VIC7|V1R45\_MOUSE sp|Q8C827|ZFP62\_MOUSE sp|Q8C827-3|ZFP62\_MOUSE tr|J3JS23|J3JS23\_MOUSE sp|Q8C827-2|ZFP62\_MOUSE sp|Q62318|TIF1B\_MOUSE tr|D6RFR0|D6RFR0\_MOUSE sp|Q9R0G8|NRK\_MOUSE sp|B2RQE8|RHG42\_MOUSE tr|B1B0C9|B1B0C9\_MOUSE sp|P15539|C11B2\_MOUSE tr|G3UWE4|G3UWE4\_MOUSE sp|Q9CR70|LAGE3\_MOUSE tr|J3JS94|J3JS94\_MOUSE sp|Q9QYY8|SPAST\_MOUSE sp|A2AQ25-2|SKT\_MOUSE sp|P68500|CNTN5\_MOUSE tr|E9PYK7|E9PYK7\_MOUSE sp|Q8R0W5|EST4A\_MOUSE sp|O54917|E2F6\_MOUSE tr|U3KLT0|U3KLT0\_MOUSE sp|Q9CQ48|NUDC2\_MOUSE tr|D3Z4F5|D3Z4F5\_MOUSE tr|Q8VFN4|Q8VFN4\_MOUSE tr|E9Q2X2|E9Q2X2\_MOUSE sp|O35864|CSN5\_MOUSE tr|F7BTZ2|F7BTZ2\_MOUSE tr|Q3UW66|Q3UW66\_MOUSE sp|Q99J99|THTM\_MOUSE sp|Q8K1N4-2|SPAS2\_MOUSE tr|A0A087WRB7|A0A087WRB7\_MOUSE tr|E9PX43|E9PX43\_MOUSE sp|A7XV07|SKIT8\_MOUSE sp|Q7TQG1|PKHA6\_MOUSE sp|O55071|CP2BJ\_MOUSE tr|F7CD45|F7CD45\_MOUSE tr|Q8BVC1|Q8BVC1\_MOUSE sp|Q80XK6|ATG2B\_MOUSE sp|P61622|ITA11\_MOUSE sp|Q9CPX7|RT16\_MOUSE sp|Q64437|ADH7\_MOUSE sp|Q8BZF8|PGM5\_MOUSE sp|Q8C0S1-2|DI3L1\_MOUSE tr|G3UWZ4|G3UWZ4\_MOUSE tr|E9Q4U5|E9Q4U5\_MOUSE sp|Q8C0S1|DI3L1\_MOUSE sp|Q91W90|TXND5\_MOUSE sp|Q91W69|EPN3\_MOUSE sp|Q9ES88|S13A2\_MOUSE tr|E9PXX7|E9PXX7\_MOUSE sp|Q9QXZ9-2|OPN4\_MOUSE sp|Q8VIM5-5|MYCD\_MOUSE sp|Q5I2A0|SPA3G\_MOUSE tr|F2Z405|F2Z405\_MOUSE tr|E9PWX7|E9PWX7\_MOUSE sp|A1A5B6|TBC25\_MOUSE sp|Q7TQA9|TR135\_MOUSE sp|Q8K3J1|NDUS8\_MOUSE tr|B9EII2|B9EII2\_MOUSE sp|Q8VDS7-2|CE57L\_MOUSE sp|Q9D9R7-3|DMRTC\_MOUSE sp|Q9D9R7-2|DMRTC\_MOUSE tr|Q7TNC9|Q7TNC9\_MOUSE tr|E9QAS7|E9QAS7\_MOUSE tr|D3YXH0|D3YXH0\_MOUSE sp|Q9D9R7|DMRTC\_MOUSE tr|Q5RKV8|Q5RKV8\_MOUSE tr|Q3TZT4|Q3TZT4\_MOUSE tr|B1AX33|B1AX33\_MOUSE tr|Q5XLJ1|Q5XLJ1\_MOUSE sp|Q00196-5|PO2F2\_MOUSE tr|G3UX44|G3UX44\_MOUSE sp|Q8CHW4|EI2BE\_MOUSE tr|V9GXE2|V9GXE2\_MOUSE sp|Q6NVF9|CPSF6\_MOUSE tr|H3BJW3|H3BJW3\_MOUSE tr|A0A087WP71|A0A087WP71\_MOUSE tr|H3BJ30|H3BJ30\_MOUSE tr|G3X977|G3X977\_MOUSE sp|Q61703|ITIH2\_MOUSE sp|Q66X19|NAL4E\_MOUSE sp|Q9Z0W3|NU160\_MOUSE sp|Q8BZL1-1|NEUR4\_MOUSE sp|Q9Z0W3-2|NU160\_MOUSE sp|Q8BZL1|NEUR4\_MOUSE tr|D3Z6P9|D3Z6P9\_MOUSE sp|Q6PE65-2|GPT2L\_MOUSE sp|Q6JHY2-5|SMGC\_MOUSE tr|Q7TQV3|Q7TQV3\_MOUSE tr|F6VRN1|F6VRN1\_MOUSE sp|A7TZF0-3|SKIT3\_MOUSE sp|Q80XH2|IMPG2\_MOUSE tr|E9QL13|E9QL13\_MOUSE sp|A2A5N8|LMBL1\_MOUSE sp|Q9ERE9|JIP2\_MOUSE sp|Q9DBT9|M2GD\_MOUSE sp|Q8C2Q3|RBM14\_MOUSE sp|Q80XH2-2|IMPG2\_MOUSE sp|Q3V1V3|ESF1\_MOUSE sp|Q8BYK5|PHAR3\_MOUSE sp|Q8C0M9|ASGL1\_MOUSE tr|E9PWQ6|E9PWQ6\_MOUSE sp|P48377|RFX1\_MOUSE sp|Q8BYK5-4|PHAR3\_MOUSE tr|E9Q260|E9Q260\_MOUSE sp|Q99MW1|STK31\_MOUSE tr|A2AHM4|A2AHM4\_MOUSE sp|Q8BYK5-2|PHAR3\_MOUSE sp|Q8BYK5-3|PHAR3\_MOUSE sp|Q62219-3|TGFI1\_MOUSE sp|Q80X44|ZBT24\_MOUSE sp|Q80X44-2|ZBT24\_MOUSE tr|E9QJU8|E9QJU8\_MOUSE sp|Q8CG79|ASPP2\_MOUSE tr|Q3V061|Q3V061\_MOUSE sp|Q6PGC1|DHX29\_MOUSE sp|P35374|AGTR2\_MOUSE sp|Q80XI7|VOME\_MOUSE tr|Q8C0K8|Q8C0K8\_MOUSE tr|D3YTX0|D3YTX0\_MOUSE tr|A0A087WQS2|A0A087WQS2\_MOUSE tr|B1AQJ3|B1AQJ3\_MOUSE sp|Q91YT0|NDUV1\_MOUSE sp|P09055-2|ITB1\_MOUSE tr|D3YUM1|D3YUM1\_MOUSE tr|D6RG60|D6RG60\_MOUSE tr|Q2NKI4|Q2NKI4\_MOUSE sp|Q8QZX2-2|HAUS3\_MOUSE sp|P19437|CD20\_MOUSE sp|Q8QZX2|HAUS3\_MOUSE sp|Q14BJ1-2|FA89A\_MOUSE sp|P10493|NID1\_MOUSE sp|Q08775-9|RUNX2\_MOUSE sp|Q08775-8|RUNX2\_MOUSE sp|Q08775-7|RUNX2\_MOUSE tr|E0CZC6|E0CZC6\_MOUSE tr|F6TRA6|F6TRA6\_MOUSE sp|A2RSQ0|DEN5B\_MOUSE tr|Q6P6L6|Q6P6L6\_MOUSE sp|O70220|FOXQ1\_MOUSE ENSEMBL:ENSBTAP00000016285 tr|Q8VG50|Q8VG50\_MOUSE sp|Q8BMA3-2|CNKR3\_MOUSE tr|A2APL5|A2APL5\_MOUSE sp|P43006-3|EAA2\_MOUSE sp|P43006-2|EAA2\_MOUSE sp|P43006|EAA2\_MOUSE tr|A2AQI7|A2AQI7\_MOUSE tr|G3X9L9|G3X9L9\_MOUSE tr|E9Q1A0|E9Q1A0\_MOUSE sp|Q8VIG0-2|ZCH14\_MOUSE sp|Q8VIG0|ZCH14\_MOUSE sp|A6X8Z5|RHG31\_MOUSE sp|Q8C0J2-4|A16L1\_MOUSE sp|Q8C0J2-2|A16L1\_MOUSE sp|Q8C0J2|A16L1\_MOUSE sp|Q8C0J2-3|A16L1\_MOUSE sp|Q8C0J2-5|A16L1\_MOUSE tr|A2AE27|A2AE27\_MOUSE sp|Q9DBT5|AMPD2\_MOUSE tr|Q3TLZ6|Q3TLZ6\_MOUSE sp|Q9D9Q6|CALR3\_MOUSE sp|Q91V80|APOF\_MOUSE tr|G3X940|G3X940\_MOUSE sp|Q8BZ21|KAT6A\_MOUSE tr|F6Z063|F6Z063\_MOUSE tr|G3UXK0|G3UXK0\_MOUSE tr|G3UZJ3|G3UZJ3\_MOUSE tr|E9PXR3|E9PXR3\_MOUSE tr|F6RFI0|F6RFI0\_MOUSE sp|P58006|SESN1\_MOUSE tr|K4DI59|K4DI59\_MOUSE sp|P97386-2|DNLI3\_MOUSE tr|B1AT03|B1AT03\_MOUSE sp|P97386|DNLI3\_MOUSE tr|Q80ZH7|Q80ZH7\_MOUSE tr|Q3UC82|Q3UC82\_MOUSE tr|F6V1P1|F6V1P1\_MOUSE sp|Q01065|PDE1B\_MOUSE sp|Q60670|SIK1\_MOUSE tr|B0R031|B0R031\_MOUSE sp|P56656|CP239\_MOUSE sp|Q3ULB5|PAK6\_MOUSE tr|G3XA18|G3XA18\_MOUSE tr|A2A545|A2A545\_MOUSE sp|Q8R3Z5-3|CACB1\_MOUSE sp|Q8R3Z5|CACB1\_MOUSE sp|P21956|MFGM\_MOUSE tr|A2A542|A2A542\_MOUSE sp|Q8R3Z5-2|CACB1\_MOUSE sp|Q8R3Z5-4|CACB1\_MOUSE tr|A2A543|A2A543\_MOUSE sp|Q9D7V9|NAAA\_MOUSE sp|P21956-2|MFGM\_MOUSE sp|Q64518-2|AT2A3\_MOUSE tr|B1ATS4|B1ATS4\_MOUSE sp|Q8VHJ7-2|PRGC2\_MOUSE sp|Q80TT8-2|CUL9\_MOUSE sp|Q8BM54|MYLIP\_MOUSE sp|Q9CZR8|EFTS\_MOUSE sp|Q8BM54-2|MYLIP\_MOUSE sp|Q61578|ADRO\_MOUSE sp|P26952-3|IL3RA\_MOUSE sp|P16056|MET\_MOUSE tr|F8VQL0|F8VQL0\_MOUSE tr|E9PXJ8|E9PXJ8\_MOUSE sp|Q64282|IFIT1\_MOUSE tr|Q8VFV0|Q8VFV0\_MOUSE sp|P15261|INGR1\_MOUSE sp|O35943|FRDA\_MOUSE sp|Q6PFX7-3|NYAP1\_MOUSE sp|Q91WI7|ITFG2\_MOUSE sp|P23578|ACRO\_MOUSE sp|Q91WI7-2|ITFG2\_MOUSE tr|E9QP99|E9QP99\_MOUSE tr|Q8CCA5|Q8CCA5\_MOUSE sp|Q6PAR0-3|KLD10\_MOUSE sp|Q6PAR0|KLD10\_MOUSE sp|Q6PAR0-2|KLD10\_MOUSE tr|E9PUA3|E9PUA3\_MOUSE tr|A0A087WQ39|A0A087WQ39\_MOUSE sp|Q8R0S2|IQEC1\_MOUSE sp|O08715-5|AKAP1\_MOUSE sp|O08715-2|AKAP1\_MOUSE sp|O08715-6|AKAP1\_MOUSE sp|O08715|AKAP1\_MOUSE tr|S4R1A8|S4R1A8\_MOUSE sp|O08715-4|AKAP1\_MOUSE sp|O08715-3|AKAP1\_MOUSE sp|P15089|CBPA3\_MOUSE sp|Q8C186|BPIFC\_MOUSE tr|Q3V1D7|Q3V1D7\_MOUSE tr|B7ZCG3|B7ZCG3\_MOUSE sp|Q794H2|NP1L3\_MOUSE sp|O08934|UNC4\_MOUSE sp|Q6ZPK0-6|PF21A\_MOUSE tr|F6W2Q5|F6W2Q5\_MOUSE tr|E9PV92|E9PV92\_MOUSE sp|Q6ZPK0-8|PF21A\_MOUSE sp|P42567|EPS15\_MOUSE sp|Q6ZPK0-3|PF21A\_MOUSE tr|Q5JC28|Q5JC28\_MOUSE sp|Q7M713|TR116\_MOUSE sp|P03911|NU4M\_MOUSE tr|Q7JCY6|Q7JCY6\_MOUSE sp|Q5PRE5|PRSR1\_MOUSE tr|G3X8Z1|G3X8Z1\_MOUSE sp|Q925N2|SFXN2\_MOUSE tr|A2ATP5|A2ATP5\_MOUSE tr|Q8C3A3|Q8C3A3\_MOUSE tr|B1AWH6|B1AWH6\_MOUSE tr|E9PYP0|E9PYP0\_MOUSE tr|E9Q7S6|E9Q7S6\_MOUSE tr|E9Q8J6|E9Q8J6\_MOUSE tr|E9PUL4|E9PUL4\_MOUSE sp|Q6GQT6|SCAP\_MOUSE sp|E9Q4S1|PDE8B\_MOUSE tr|A0A087WRD9|A0A087WRD9\_MOUSE sp|Q00899|TYY1\_MOUSE tr|D3YU55|D3YU55\_MOUSE tr|F6XC48|F6XC48\_MOUSE sp|Q8R151|ZNFX1\_MOUSE tr|A2A5R6|A2A5R6\_MOUSE tr|F8VPK8|F8VPK8\_MOUSE sp|Q9R1E0|FOXO1\_MOUSE sp|Q9Z0E1|M1AP\_MOUSE tr|F6SRP4|F6SRP4\_MOUSE tr|S4R238|S4R238\_MOUSE sp|Q8BRB7|KAT6B\_MOUSE sp|Q9CPT5|NOP16\_MOUSE tr|D3Z7M5|D3Z7M5\_MOUSE tr|Q3UID0|Q3UID0\_MOUSE sp|Q6PDG5|SMRC2\_MOUSE sp|Q6PDG5-2|SMRC2\_MOUSE sp|Q99NF2-5|NSMF\_MOUSE sp|Q99NF2-4|NSMF\_MOUSE sp|Q9ESJ0|XPO4\_MOUSE tr|F6TKU5|F6TKU5\_MOUSE tr|A2AJ92|A2AJ92\_MOUSE tr|A2AJ95|A2AJ95\_MOUSE sp|Q99NF2-3|NSMF\_MOUSE sp|Q99NF2|NSMF\_MOUSE tr|A2AJ96|A2AJ96\_MOUSE sp|Q99NF2-2|NSMF\_MOUSE sp|Q8C3Q5|SHSA7\_MOUSE sp|E9Q355-5|CTSG1\_MOUSE sp|Q8C3Q5-2|SHSA7\_MOUSE tr|F6VT60|F6VT60\_MOUSE tr|G3V000|G3V000\_MOUSE sp|Q8BFT9|SVOP\_MOUSE sp|O70374-2|MTG8R\_MOUSE sp|O70374|MTG8R\_MOUSE tr|Q3UGB2|Q3UGB2\_MOUSE sp|P21812|MCPT4\_MOUSE sp|Q5PR73|DIRA2\_MOUSE tr|F6TUM7|F6TUM7\_MOUSE tr|A2BI84|A2BI84\_MOUSE sp|Q01705-4|NOTC1\_MOUSE sp|Q6GQV7|EDRF1\_MOUSE sp|Q01705|NOTC1\_MOUSE tr|D3YZE7|D3YZE7\_MOUSE sp|Q01705-3|NOTC1\_MOUSE tr|E9Q9F9|E9Q9F9\_MOUSE tr|Q91WH2|Q91WH2\_MOUSE tr|F6VR19|F6VR19\_MOUSE sp|Q99LB2|DHRS4\_MOUSE sp|P17182|ENOA\_MOUSE tr|E9QAQ8|E9QAQ8\_MOUSE tr|Q8VDU4|Q8VDU4\_MOUSE tr|E9PXI0|E9PXI0\_MOUSE sp|P27870|VAV\_MOUSE sp|P63013-2|PRRX1\_MOUSE tr|E0CX99|E0CX99\_MOUSE sp|A6H603|NWD1\_MOUSE tr|A9C433|A9C433\_MOUSE sp|A6H603-4|NWD1\_MOUSE tr|F7DAC7|F7DAC7\_MOUSE sp|A6H603-2|NWD1\_MOUSE sp|A6H603-3|NWD1\_MOUSE tr|F6U6S4|F6U6S4\_MOUSE sp|Q9Z1D9|ZN394\_MOUSE tr|E0CYZ6|E0CYZ6\_MOUSE sp|Q8K2D0|F199X\_MOUSE sp|Q9CR84|AT5G1\_MOUSE sp|Q0P5W1-3|VPS8\_MOUSE sp|Q0P5W1|VPS8\_MOUSE tr|F8WI64|F8WI64\_MOUSE tr|F6ZLN8|F6ZLN8\_MOUSE tr|D3YUP0|D3YUP0\_MOUSE sp|Q8BG48|ST17B\_MOUSE sp|Q9D620|RFIP1\_MOUSE tr|H3BIX0|H3BIX0\_MOUSE tr|E9Q876|E9Q876\_MOUSE tr|L7N2B2|L7N2B2\_MOUSE sp|E9Q612|PTPRO\_MOUSE tr|E9Q4I1|E9Q4I1\_MOUSE sp|Q5RJH2|MCTP2\_MOUSE tr|G3V001|G3V001\_MOUSE sp|Q6PGG6|GNL3L\_MOUSE sp|Q8BUL5|KLHL7\_MOUSE tr|G8JL73|G8JL73\_MOUSE tr|D3YW60|D3YW60\_MOUSE sp|Q9D8U4|C1QT2\_MOUSE tr|G3UW75|G3UW75\_MOUSE sp|P60122|RUVB1\_MOUSE sp|Q99JZ0|SDCB2\_MOUSE tr|E9Q9T6|E9Q9T6\_MOUSE sp|Q6ZPL9|DDX55\_MOUSE tr|F7D0T8|F7D0T8\_MOUSE sp|Q9Z0U0|XPR1\_MOUSE tr|A2AAN9|A2AAN9\_MOUSE tr|A2AAP0|A2AAP0\_MOUSE sp|P80318|TCPG\_MOUSE tr|Q3U0I3|Q3U0I3\_MOUSE sp|Q3TEL6-2|RN157\_MOUSE sp|Q3TEL6|RN157\_MOUSE tr|D3Z2B4|D3Z2B4\_MOUSE sp|P55937-1|GOGA3\_MOUSE sp|B2RUP2|UN13D\_MOUSE sp|O35668|HAP1\_MOUSE sp|O35668-2|HAP1\_MOUSE sp|B2RUP2-2|UN13D\_MOUSE sp|P55937|GOGA3\_MOUSE tr|F2Z480|F2Z480\_MOUSE tr|Q8VGK8|Q8VGK8\_MOUSE tr|E9PV37|E9PV37\_MOUSE sp|Q9QYB2|DACH1\_MOUSE sp|Q9QYB2-2|DACH1\_MOUSE sp|Q9D2H2|KAD7\_MOUSE sp|Q9DA32-2|SUN5\_MOUSE sp|Q9DA32|SUN5\_MOUSE tr|F8WIC0|F8WIC0\_MOUSE tr|Q8VFX8|Q8VFX8\_MOUSE sp|P70407|CADH9\_MOUSE tr|F8WHU6|F8WHU6\_MOUSE tr|J3JS86|J3JS86\_MOUSE sp|Q01149|CO1A2\_MOUSE tr|A2ABQ5|A2ABQ5\_MOUSE sp|Q9D232-2|SPNS3\_MOUSE sp|Q5PR68|CE112\_MOUSE sp|Q7TMX5-2|SHQ1\_MOUSE sp|Q9D232|SPNS3\_MOUSE tr|S4R1I4|S4R1I4\_MOUSE tr|I7HLS4|I7HLS4\_MOUSE sp|Q7TMX5|SHQ1\_MOUSE sp|Q8R2R1|POMT1\_MOUSE tr|A2AVV6|A2AVV6\_MOUSE sp|Q91YE9|5NT1B\_MOUSE tr|A2ADZ2|A2ADZ2\_MOUSE tr|S4R2C1|S4R2C1\_MOUSE sp|Q64691|CAN3\_MOUSE sp|Q64691-2|CAN3\_MOUSE tr|A2AVV5|A2AVV5\_MOUSE tr|A2AVV7|A2AVV7\_MOUSE sp|Q91YE9-2|5NT1B\_MOUSE tr|S4R187|S4R187\_MOUSE tr|Q0VGP9|Q0VGP9\_MOUSE sp|Q9WVM6|TLL2\_MOUSE tr|E9Q5B9|E9Q5B9\_MOUSE tr|A2ALS7|A2ALS7\_MOUSE sp|P09103|PDIA1\_MOUSE sp|P97500-2|MYT1L\_MOUSE sp|P97500-4|MYT1L\_MOUSE tr|S4R183|S4R183\_MOUSE tr|G3V013|G3V013\_MOUSE tr|D3Z1P7|D3Z1P7\_MOUSE tr|G3UYZ0|G3UYZ0\_MOUSE tr|G3UXT8|G3UXT8\_MOUSE tr|E9QLD2|E9QLD2\_MOUSE sp|P97500|MYT1L\_MOUSE sp|Q6PCQ0-2|IQCE\_MOUSE sp|O54788|DFFB\_MOUSE sp|P59222|SREC2\_MOUSE sp|Q62418-3|DBNL\_MOUSE sp|Q62418|DBNL\_MOUSE sp|P43029|GDF7\_MOUSE sp|Q9Z0F6|RAD9A\_MOUSE sp|Q80VJ8|KASH5\_MOUSE sp|Q921Y2|IMP3\_MOUSE tr|Q8BM11|Q8BM11\_MOUSE sp|Q62418-2|DBNL\_MOUSE sp|P43029-2|GDF7\_MOUSE tr|Q6NXK9|Q6NXK9\_MOUSE sp|Q3UA37|QRIC1\_MOUSE sp|Q9D818-3|SAPC2\_MOUSE sp|Q9D818-2|SAPC2\_MOUSE tr|A2CG28|A2CG28\_MOUSE sp|Q9D818|SAPC2\_MOUSE sp|P31695|NOTC4\_MOUSE sp|Q91ZU6-8|DYST\_MOUSE tr|F6QZ02|F6QZ02\_MOUSE tr|E9Q550|E9Q550\_MOUSE sp|Q5SW45|MKS1\_MOUSE tr|G3X973|G3X973\_MOUSE sp|Q8R4Y4|STAB1\_MOUSE tr|F2Z423|F2Z423\_MOUSE sp|Q8BGC0|HTSF1\_MOUSE sp|Q9DBV3|DHX34\_MOUSE sp|Q9D3R5|F187A\_MOUSE sp|Q80U63-2|MFN2\_MOUSE tr|Q3TF37|Q3TF37\_MOUSE sp|O70468|MYPC3\_MOUSE tr|E9Q9T8|E9Q9T8\_MOUSE tr|Q3UIK0|Q3UIK0\_MOUSE sp|P49442|INPP\_MOUSE tr|E9Q8T4|E9Q8T4\_MOUSE sp|Q80Z10-2|ASTN2\_MOUSE sp|Q80Z10|ASTN2\_MOUSE sp|Q8BX51|SGCZ\_MOUSE tr|E0CXA1|E0CXA1\_MOUSE tr|F6UY44|F6UY44\_MOUSE tr|D3YZA9|D3YZA9\_MOUSE tr|D3YY31|D3YY31\_MOUSE sp|Q8CHT6|F169B\_MOUSE tr|Q9EQR4|Q9EQR4\_MOUSE sp|P58710|GGLO\_MOUSE sp|Q9Z351-12|KCNQ2\_MOUSE tr|D3YU59|D3YU59\_MOUSE sp|Q08642|PADI2\_MOUSE sp|Q9Z351-4|KCNQ2\_MOUSE sp|Q9CQ44|TM218\_MOUSE tr|E9Q3Y7|E9Q3Y7\_MOUSE sp|Q5PR69-2|K1211\_MOUSE sp|Q5PR69|K1211\_MOUSE tr|E9Q5L4|E9Q5L4\_MOUSE sp|Q9JME9|VTM2B\_MOUSE tr|Q5ICG5|Q5ICG5\_MOUSE sp|Q91WC3|ACSL6\_MOUSE sp|Q91WC3-2|ACSL6\_MOUSE tr|Q5F2C5|Q5F2C5\_MOUSE tr|Q8R1X1|Q8R1X1\_MOUSE tr|A0A087WQ75|A0A087WQ75\_MOUSE tr|A0A087WP73|A0A087WP73\_MOUSE sp|A2AM29|AF9\_MOUSE sp|Q9WUU9|GANP\_MOUSE sp|A2RT60|HTRA4\_MOUSE sp|Q8R316-2|HBP1\_MOUSE tr|E9Q1A8|E9Q1A8\_MOUSE sp|Q8R316|HBP1\_MOUSE tr|E9Q469|E9Q469\_MOUSE sp|Q3UUG6|TBC24\_MOUSE sp|Q5DID3-3|UROL1\_MOUSE sp|Q5DID3|UROL1\_MOUSE tr|D3Z7A9|D3Z7A9\_MOUSE tr|Q3UKV0|Q3UKV0\_MOUSE tr|F7BIN0|F7BIN0\_MOUSE sp|Q8R4T9|UT2\_MOUSE tr|G5E849|G5E849\_MOUSE sp|P61168-2|DRD2\_MOUSE sp|P61168|DRD2\_MOUSE sp|Q8R4T9-3|UT2\_MOUSE tr|E9PYQ4|E9PYQ4\_MOUSE sp|P35582|FOXA1\_MOUSE sp|Q8K2G4-2|BBS7\_MOUSE sp|Q99LI9|CLP1\_MOUSE tr|D6RDT5|D6RDT5\_MOUSE tr|B0QZI7|B0QZI7\_MOUSE tr|A2A697|A2A697\_MOUSE tr|B0QZI3|B0QZI3\_MOUSE sp|Q9D8K8|S2539\_MOUSE tr|B0QZI4|B0QZI4\_MOUSE sp|Q8K2G4|BBS7\_MOUSE ENSEMBL:ENSBTAP00000032840 tr|D6RE23|D6RE23\_MOUSE tr|B0QZI6|B0QZI6\_MOUSE tr|E9Q0R0|E9Q0R0\_MOUSE tr|B0QZI5|B0QZI5\_MOUSE tr|F7A4U8|F7A4U8\_MOUSE tr|A0A087WPL5|A0A087WPL5\_MOUSE tr|E9QNN1|E9QNN1\_MOUSE sp|O70133|DHX9\_MOUSE sp|O70133-2|DHX9\_MOUSE sp|Q91XF0|PNPO\_MOUSE tr|A2A6E7|A2A6E7\_MOUSE sp|Q0P5V2|SOBP\_MOUSE sp|P70232-2|NCHL1\_MOUSE tr|G3UYV5|G3UYV5\_MOUSE tr|Q3U6K9|Q3U6K9\_MOUSE tr|A0A087WQ46|A0A087WQ46\_MOUSE sp|Q99K85|SERC\_MOUSE sp|Q09163-6|DLK1\_MOUSE tr|Q9DAU5|Q9DAU5\_MOUSE sp|A2AM05-2|CNTLN\_MOUSE sp|Q61316|HSP74\_MOUSE sp|P35282|RAB21\_MOUSE sp|Q09163-4|DLK1\_MOUSE tr|Q3UQI0|Q3UQI0\_MOUSE tr|G3UWU8|G3UWU8\_MOUSE tr|Q3U2G2|Q3U2G2\_MOUSE sp|Q6P3Y5-2|Z280C\_MOUSE tr|Q7TQT8|Q7TQT8\_MOUSE sp|Q9JM99-5|PRG4\_MOUSE sp|Q3V0E1|CI131\_MOUSE sp|P06728|APOA4\_MOUSE sp|P63141|KCNA2\_MOUSE tr|F6S3V7|F6S3V7\_MOUSE tr|F7D043|F7D043\_MOUSE sp|A2AWP8-5|ARGAL\_MOUSE sp|Q9QZ05-3|E2AK4\_MOUSE tr|A2AUM2|A2AUM2\_MOUSE sp|Q9QZ05-2|E2AK4\_MOUSE tr|A2AUM1|A2AUM1\_MOUSE sp|Q9QZ05-4|E2AK4\_MOUSE tr|F6YZJ7|F6YZJ7\_MOUSE sp|A2AWL7-2|MGAP\_MOUSE sp|Q91ZQ5|RPE65\_MOUSE sp|Q9JIX8-4|ACINU\_MOUSE sp|Q9JIX8|ACINU\_MOUSE sp|O35551-3|RABE1\_MOUSE sp|O35551-5|RABE1\_MOUSE sp|O35551|RABE1\_MOUSE sp|O35551-2|RABE1\_MOUSE tr|J3QJV7|J3QJV7\_MOUSE sp|O35551-6|RABE1\_MOUSE sp|Q80UF4|SDCG8\_MOUSE tr|Q5NCH7|Q5NCH7\_MOUSE tr|H3BJY2|H3BJY2\_MOUSE tr|H3BL41|H3BL41\_MOUSE sp|Q91YP0|L2HDH\_MOUSE sp|Q9Z0H4-7|CELF2\_MOUSE sp|Q9Z0H4|CELF2\_MOUSE sp|Q9Z0H4-4|CELF2\_MOUSE sp|Q8BG54|SPTC3\_MOUSE sp|Q9Z0H4-5|CELF2\_MOUSE sp|Q61330|CNTN2\_MOUSE sp|Q9Z0H4-8|CELF2\_MOUSE sp|Q9Z0H4-9|CELF2\_MOUSE sp|Q9Z0H4-3|CELF2\_MOUSE tr|F8WIZ7|F8WIZ7\_MOUSE sp|Q8BXJ2-3|TREF1\_MOUSE tr|Q8VG95|Q8VG95\_MOUSE tr|D3YUI5|D3YUI5\_MOUSE sp|Q9CTN5-2|S6OS1\_MOUSE tr|D3YZ78|D3YZ78\_MOUSE tr|G3X9E9|G3X9E9\_MOUSE sp|Q99NH0|ANR17\_MOUSE tr|B1AV20|B1AV20\_MOUSE sp|O88573|AFF1\_MOUSE tr|A3KMF4|A3KMF4\_MOUSE tr|B1AVP1|B1AVP1\_MOUSE tr|E9Q921|E9Q921\_MOUSE sp|P12657|ACM1\_MOUSE sp|P49717|MCM4\_MOUSE tr|Q9D6L6|Q9D6L6\_MOUSE sp|P70698|PYRG1\_MOUSE tr|A0A087WPQ9|A0A087WPQ9\_MOUSE tr|R4GML3|R4GML3\_MOUSE tr|B0R041|B0R041\_MOUSE tr|B0R042|B0R042\_MOUSE sp|Q8BX90|FND3A\_MOUSE tr|F6TLV3|F6TLV3\_MOUSE tr|G3UYR9|G3UYR9\_MOUSE tr|G3UYG6|G3UYG6\_MOUSE tr|G3UWR2|G3UWR2\_MOUSE tr|E9PUA0|E9PUA0\_MOUSE tr|E9Q2M5|E9Q2M5\_MOUSE sp|Q05306|COAA1\_MOUSE tr|G3UZX7|G3UZX7\_MOUSE sp|Q6Y7W8|PERQ2\_MOUSE tr|Q8CB10|Q8CB10\_MOUSE sp|Q91Y44|BRDT\_MOUSE tr|D3YUW8|D3YUW8\_MOUSE sp|Q0VGT4-2|ZGRF1\_MOUSE tr|Q3UKN6|Q3UKN6\_MOUSE sp|P81117|NUCB2\_MOUSE tr|E9PV13|E9PV13\_MOUSE tr|F8WHK3|F8WHK3\_MOUSE tr|Q5SXC3|Q5SXC3\_MOUSE tr|E9PV32|E9PV32\_MOUSE tr|Q8VG26|Q8VG26\_MOUSE tr|Q5SXC4|Q5SXC4\_MOUSE sp|Q8CIP4|MARK4\_MOUSE sp|Q8CA95-4|PDE10\_MOUSE tr|A2AI76|A2AI76\_MOUSE tr|Q8CDD3|Q8CDD3\_MOUSE sp|Q9R1K7|TBD\_MOUSE tr|J3QPJ0|J3QPJ0\_MOUSE sp|Q8BVG4|DPP9\_MOUSE sp|Q8BVG4-2|DPP9\_MOUSE sp|D3YYM0|OVOL3\_MOUSE sp|P70390-2|SHOX2\_MOUSE sp|P70390|SHOX2\_MOUSE sp|Q61183-3|PAPOA\_MOUSE sp|Q8C6P8|ZFP57\_MOUSE tr|E9PZR3|E9PZR3\_MOUSE tr|Q62293|Q62293\_MOUSE sp|Q61183|PAPOA\_MOUSE sp|Q61183-4|PAPOA\_MOUSE sp|Q01063-2|PDE4D\_MOUSE tr|Q3V1Q3|Q3V1Q3\_MOUSE tr|B2KF57|B2KF57\_MOUSE sp|Q8BX05-3|GLPK5\_MOUSE tr|B1PSD9|B1PSD9\_MOUSE sp|Q80TL1|ADCY2\_MOUSE sp|Q8C6P8-2|ZFP57\_MOUSE tr|B2KF84|B2KF84\_MOUSE sp|Q8BX05-2|GLPK5\_MOUSE tr|E9PWC8|E9PWC8\_MOUSE tr|E9PVU7|E9PVU7\_MOUSE sp|Q8BX05|GLPK5\_MOUSE sp|A2AJ88-3|PLPL7\_MOUSE sp|Q76LS9|FA63A\_MOUSE sp|P61620|S61A1\_MOUSE tr|G3X9I7|G3X9I7\_MOUSE tr|E9QPB2|E9QPB2\_MOUSE sp|Q8CIT9|SBSN\_MOUSE tr|E9PVJ0|E9PVJ0\_MOUSE tr|F8VQ39|F8VQ39\_MOUSE sp|Q149C3|LIGO4\_MOUSE tr|Q3TWJ1|Q3TWJ1\_MOUSE tr|G5E8J0|G5E8J0\_MOUSE tr|Q8VCR0|Q8VCR0\_MOUSE tr|F6Q8A4|F6Q8A4\_MOUSE tr|G3UXP8|G3UXP8\_MOUSE tr|E9PWV3|E9PWV3\_MOUSE sp|O35516-2|NOTC2\_MOUSE sp|O35516|NOTC2\_MOUSE tr|F6RQA2|F6RQA2\_MOUSE sp|P28704-2|RXRB\_MOUSE sp|P28704|RXRB\_MOUSE tr|B1AXN3|B1AXN3\_MOUSE sp|Q8R3N6|THOC1\_MOUSE tr|A2AHG4|A2AHG4\_MOUSE tr|A2AHG2|A2AHG2\_MOUSE tr|E9QKW9|E9QKW9\_MOUSE sp|Q6ZPK0|PF21A\_MOUSE tr|Q80UI5|Q80UI5\_MOUSE sp|P26450|P85A\_MOUSE tr|A0A5C8|A0A5C8\_MOUSE sp|Q3U3D7|T131L\_MOUSE sp|Q3U3D7-2|T131L\_MOUSE sp|Q3U3D7-3|T131L\_MOUSE sp|Q8BM14|LIPK\_MOUSE tr|F8WJA1|F8WJA1\_MOUSE sp|P97808|FXYD5\_MOUSE sp|Q8BM14-2|LIPK\_MOUSE tr|E0CXN1|E0CXN1\_MOUSE sp|Q8CEI1|BOLA3\_MOUSE sp|Q8K3I4|MYRIP\_MOUSE tr|H3BLN5|H3BLN5\_MOUSE sp|Q8BVZ5|IL33\_MOUSE tr|F6YTG0|F6YTG0\_MOUSE tr|E9Q1H7|E9Q1H7\_MOUSE tr|F6VTN8|F6VTN8\_MOUSE sp|P33766|FPR1\_MOUSE tr|Q05CS0|Q05CS0\_MOUSE sp|Q8R502|LRC8C\_MOUSE tr|H3BLG3|H3BLG3\_MOUSE sp|Q6NY15-3|TSG10\_MOUSE sp|Q6NY15-2|TSG10\_MOUSE sp|Q6NY15|TSG10\_MOUSE sp|Q8CJG0|AGO2\_MOUSE tr|A0A087WS99|A0A087WS99\_MOUSE sp|Q3UVR3-2|TTBK2\_MOUSE tr|F6YT88|F6YT88\_MOUSE sp|Q91YM4-2|TBRG4\_MOUSE sp|Q91XU0|WRIP1\_MOUSE tr|E9PUT1|E9PUT1\_MOUSE sp|Q91XU0-2|WRIP1\_MOUSE sp|Q9DCJ7|AKIP\_MOUSE sp|Q91YM4|TBRG4\_MOUSE tr|O70517|O70517\_MOUSE tr|G3X9V9|G3X9V9\_MOUSE sp|Q9QZN1-2|FXL17\_MOUSE tr|D3Z5Q7|D3Z5Q7\_MOUSE sp|P61202-2|CSN2\_MOUSE sp|P61202|CSN2\_MOUSE tr|D3Z228|D3Z228\_MOUSE tr|L7MU96|L7MU96\_MOUSE tr|D3Z5B8|D3Z5B8\_MOUSE tr|A2ASJ1|A2ASJ1\_MOUSE tr|Q9EQ94|Q9EQ94\_MOUSE sp|P97355|SPSY\_MOUSE tr|A2AQE4|A2AQE4\_MOUSE tr|Q9EQB6|Q9EQB6\_MOUSE tr|F6Y0D0|F6Y0D0\_MOUSE sp|Q80WC9|ACSF4\_MOUSE sp|Q8BGB8|COQ4\_MOUSE sp|Q02496|MUC1\_MOUSE tr|F6VXK7|F6VXK7\_MOUSE tr|F7CGC9|F7CGC9\_MOUSE sp|Q8R0K4|CC137\_MOUSE sp|Q9R1X5|MRP5\_MOUSE tr|H3BLI5|H3BLI5\_MOUSE sp|Q7TSK3-3|PCDH8\_MOUSE tr|D3YUJ1|D3YUJ1\_MOUSE sp|Q3UDP0-2|WDR41\_MOUSE sp|A2AHG0-3|LZTS3\_MOUSE sp|A2AHG0|LZTS3\_MOUSE sp|A2AHG0-2|LZTS3\_MOUSE sp|Q91XS1|MTMR4\_MOUSE sp|Q91XS1-2|MTMR4\_MOUSE sp|P63046|ST4A1\_MOUSE tr|D3Z3X6|D3Z3X6\_MOUSE sp|P63046-2|ST4A1\_MOUSE tr|D3Z6H9|D3Z6H9\_MOUSE tr|D3Z6H7|D3Z6H7\_MOUSE sp|Q80TS3-2|LPHN3\_MOUSE sp|Q5HZG4-2|TAF3\_MOUSE sp|Q99J62|RFC4\_MOUSE tr|A0A087WQA8|A0A087WQA8\_MOUSE sp|Q3V080|ZN583\_MOUSE tr|Q7TPG0|Q7TPG0\_MOUSE tr|Q3TTN6|Q3TTN6\_MOUSE sp|Q8BHS8-4|SYBU\_MOUSE sp|Q80VF6|F181B\_MOUSE tr|A2AQF0|A2AQF0\_MOUSE tr|G3UXC3|G3UXC3\_MOUSE sp|P16951|ATF2\_MOUSE sp|Q9WVB0|RBPMS\_MOUSE tr|T1ECW4|T1ECW4\_MOUSE sp|Q9WVB0-2|RBPMS\_MOUSE tr|S4R1Y2|S4R1Y2\_MOUSE sp|O88852|TSYL1\_MOUSE sp|Q99JZ7|ERRFI\_MOUSE tr|L7N221|L7N221\_MOUSE tr|D6RIK6|D6RIK6\_MOUSE tr|Q8R1V9|Q8R1V9\_MOUSE tr|D3Z158|D3Z158\_MOUSE tr|Q8BML9|Q8BML9\_MOUSE sp|E9PV24|FIBA\_MOUSE P01966 sp|E9PV24-2|FIBA\_MOUSE tr|F6QGA9|F6QGA9\_MOUSE tr|A2CF73|A2CF73\_MOUSE tr|A2AFQ2|A2AFQ2\_MOUSE tr|Q99N15|Q99N15\_MOUSE sp|O08756|HCD2\_MOUSE sp|Q3UHX8|NKX63\_MOUSE tr|F6XPZ0|F6XPZ0\_MOUSE sp|C8YR32|LOXH1\_MOUSE tr|F8VQ31|F8VQ31\_MOUSE tr|A2AWQ2|A2AWQ2\_MOUSE tr|Q7TRC7|Q7TRC7\_MOUSE sp|Q8BK67|RCC2\_MOUSE sp|Q8VE96|S35F6\_MOUSE tr|J3KMP5|J3KMP5\_MOUSE tr|F2Z3X1|F2Z3X1\_MOUSE sp|Q9JJ04|B4GT4\_MOUSE sp|Q80VP1-2|EPN1\_MOUSE sp|Q80VP1|EPN1\_MOUSE sp|Q91YP2|NEUL\_MOUSE sp|Q9D856|S39A5\_MOUSE tr|Q9CQX6|Q9CQX6\_MOUSE tr|D3Z617|D3Z617\_MOUSE tr|Q8BLF6|Q8BLF6\_MOUSE tr|Q8BMG0|Q8BMG0\_MOUSE tr|Z4YKR8|Z4YKR8\_MOUSE sp|Q00196-7|PO2F2\_MOUSE sp|P06795|MDR1B\_MOUSE sp|Q64237|DOPO\_MOUSE sp|Q9JII2|PR5A1\_MOUSE tr|A0A087WQE8|A0A087WQE8\_MOUSE tr|F7CG42|F7CG42\_MOUSE sp|Q80WE4|KI20B\_MOUSE sp|Q6P5E6|GGA2\_MOUSE sp|Q80WE4-2|KI20B\_MOUSE sp|Q80WE4-4|KI20B\_MOUSE tr|E9Q0A6|E9Q0A6\_MOUSE sp|Q80WE4-3|KI20B\_MOUSE sp|Q6P5E6-2|GGA2\_MOUSE sp|Q9DAN9|CQ105\_MOUSE sp|Q8K2L8|TPC12\_MOUSE tr|Q4FZF9|Q4FZF9\_MOUSE tr|A2A8C4|A2A8C4\_MOUSE sp|Q14DK5|HIPL1\_MOUSE sp|Q8C190|VP9D1\_MOUSE sp|Q8C190-2|VP9D1\_MOUSE tr|Q3U280|Q3U280\_MOUSE tr|Q7TQX8|Q7TQX8\_MOUSE tr|H3BK41|H3BK41\_MOUSE sp|O35885|ASCL2\_MOUSE tr|E9Q6H8|E9Q6H8\_MOUSE sp|Q8BTS4|NUP54\_MOUSE tr|F6RUK9|F6RUK9\_MOUSE sp|Q64287-2|IRF4\_MOUSE tr|E9PUJ9|E9PUJ9\_MOUSE sp|A2AG50-3|MA7D2\_MOUSE sp|Q8VC98-2|PKHA4\_MOUSE tr|Q8C0A6|Q8C0A6\_MOUSE ENSEMBL:ENSBTAP00000001528 tr|D3YTK9|D3YTK9\_MOUSE tr|A2AQ99|A2AQ99\_MOUSE sp|Q07230|ZSCA2\_MOUSE tr|D3Z7F6|D3Z7F6\_MOUSE tr|A2AG11|A2AG11\_MOUSE tr|A2AG12|A2AG12\_MOUSE tr|A2AG10|A2AG10\_MOUSE tr|E9PWV0|E9PWV0\_MOUSE sp|Q6NS59-2|F135A\_MOUSE sp|P16879|FES\_MOUSE sp|P14733|LMNB1\_MOUSE tr|A0A087WQ08|A0A087WQ08\_MOUSE sp|Q9R0A1|CLCN2\_MOUSE tr|A9C437|A9C437\_MOUSE sp|O09043|NAPSA\_MOUSE sp|Q9Z1Z0-2|USO1\_MOUSE tr|F6WR45|F6WR45\_MOUSE sp|P56677|ST14\_MOUSE sp|Q9ES52-6|SHIP1\_MOUSE tr|Q8CB58|Q8CB58\_MOUSE tr|Q8BGJ5|Q8BGJ5\_MOUSE sp|Q9ES52-5|SHIP1\_MOUSE sp|Q9D8P4|RM17\_MOUSE sp|Q9D8P4-2|RM17\_MOUSE tr|H3BJT9|H3BJT9\_MOUSE tr|H3BIZ3|H3BIZ3\_MOUSE tr|F8VPY2|F8VPY2\_MOUSE sp|Q8C092|TAF5\_MOUSE sp|P97443-3|SMYD1\_MOUSE sp|Q920A7|AFG31\_MOUSE sp|Q8BL80|RHG22\_MOUSE sp|Q6S7F2|E2F7\_MOUSE tr|E9PZ06|E9PZ06\_MOUSE sp|Q8BL80-2|RHG22\_MOUSE sp|P40935|PNMT\_MOUSE tr|J3QP46|J3QP46\_MOUSE sp|A2A891-3|CMTA1\_MOUSE sp|A2A891-4|CMTA1\_MOUSE tr|Q8VET2|Q8VET2\_MOUSE sp|Q61088|FZD4\_MOUSE sp|Q3U1J4|DDB1\_MOUSE sp|Q3UHC0|TNR6C\_MOUSE tr|B7ZC94|B7ZC94\_MOUSE sp|Q6PFH3-2|DCA15\_MOUSE sp|Q6PFH3|DCA15\_MOUSE tr|D3YWX2|D3YWX2\_MOUSE sp|Q03526|ITK\_MOUSE tr|E9Q803|E9Q803\_MOUSE tr|Q5STT8|Q5STT8\_MOUSE sp|P15806-2|TFE2\_MOUSE sp|P15806|TFE2\_MOUSE tr|E9PVV1|E9PVV1\_MOUSE tr|F6T941|F6T941\_MOUSE tr|E9PWE5|E9PWE5\_MOUSE tr|E9PVV2|E9PVV2\_MOUSE tr|E9PWE3|E9PWE3\_MOUSE tr|E9PWE2|E9PWE2\_MOUSE tr|E9PWE4|E9PWE4\_MOUSE sp|Q14DL3|LRIQ3\_MOUSE tr|E9Q0X4|E9Q0X4\_MOUSE tr|D6RFS3|D6RFS3\_MOUSE tr|Q8K4L6|Q8K4L6\_MOUSE tr|D3YVG0|D3YVG0\_MOUSE sp|P03987|IGHG3\_MOUSE sp|O35375|NRP2\_MOUSE sp|O35375-2|NRP2\_MOUSE sp|Q8VCD7|KDM4C\_MOUSE sp|O35375-4|NRP2\_MOUSE sp|O35375-3|NRP2\_MOUSE tr|A2AP83|A2AP83\_MOUSE tr|G3X9L5|G3X9L5\_MOUSE sp|Q8BGD6|S38A9\_MOUSE sp|Q9Z0S5|CLD15\_MOUSE sp|Q6VNS1-3|NTRK3\_MOUSE tr|A0A087WRD6|A0A087WRD6\_MOUSE tr|F7DD14|F7DD14\_MOUSE tr|A2AM80|A2AM80\_MOUSE tr|Q3UGL1|Q3UGL1\_MOUSE sp|Q7TME2|SPAG5\_MOUSE tr|D3Z2M0|D3Z2M0\_MOUSE sp|Q60928|GGT1\_MOUSE sp|Q8C9B9-2|DIDO1\_MOUSE sp|Q9Z2Q2-3|KNOP1\_MOUSE sp|Q9Z2Q2-2|KNOP1\_MOUSE sp|Q91W29|COX42\_MOUSE tr|G3X9K7|G3X9K7\_MOUSE sp|A6H690-2|IQCAL\_MOUSE sp|P47963|RL13\_MOUSE tr|A2AF67|A2AF67\_MOUSE sp|Q60634-3|FLOT2\_MOUSE tr|O35700|O35700\_MOUSE sp|A2AF47|DOC11\_MOUSE tr|E9QNL8|E9QNL8\_MOUSE tr|D3YW95|D3YW95\_MOUSE tr|E9Q5M7|E9Q5M7\_MOUSE sp|P24063|ITAL\_MOUSE tr|D3Z627|D3Z627\_MOUSE tr|F6V513|F6V513\_MOUSE tr|B2RXQ9|B2RXQ9\_MOUSE sp|Q8R574|KPRB\_MOUSE sp|Q0P5W1-2|VPS8\_MOUSE sp|P23819-2|GRIA2\_MOUSE tr|E9QKC0|E9QKC0\_MOUSE tr|J3QNB0|J3QNB0\_MOUSE sp|Q9CQE8|CN166\_MOUSE sp|Q91XQ0-3|DYH8\_MOUSE sp|D3YYU8|OBSL1\_MOUSE sp|Q8CDV6|CCD63\_MOUSE tr|D3YXL1|D3YXL1\_MOUSE sp|Q8CDV6-2|CCD63\_MOUSE sp|Q3UHI0|CCSE2\_MOUSE sp|Q9CQA9|NTPCR\_MOUSE tr|E9Q9Q2|E9Q9Q2\_MOUSE tr|F7B2D1|F7B2D1\_MOUSE sp|Q3U1Y4-3|DEN4B\_MOUSE sp|Q3UTQ7|PRD10\_MOUSE tr|A0A087WRS6|A0A087WRS6\_MOUSE tr|E9Q622|E9Q622\_MOUSE tr|E9Q7U8|E9Q7U8\_MOUSE sp|Q3UZP0|MALD2\_MOUSE sp|Q3U1V6|UEVLD\_MOUSE sp|P04919-2|B3AT\_MOUSE sp|P04919|B3AT\_MOUSE sp|Q8C102|GALT5\_MOUSE sp|Q8BTN6|LENG9\_MOUSE sp|B0V2N1-6|PTPRS\_MOUSE sp|B0V2N1|PTPRS\_MOUSE sp|B0V2N1-2|PTPRS\_MOUSE sp|O08747|UNC5C\_MOUSE sp|O08747-2|UNC5C\_MOUSE tr|E9PVI4|E9PVI4\_MOUSE sp|Q8BFU3-5|RN214\_MOUSE sp|Q91WF3|ADCY4\_MOUSE sp|Q8BFU3-2|RN214\_MOUSE tr|D3YW76|D3YW76\_MOUSE sp|Q8BFU3-3|RN214\_MOUSE tr|F8WID1|F8WID1\_MOUSE sp|Q8BFU3-4|RN214\_MOUSE sp|Q8BFU3|RN214\_MOUSE sp|Q01887|RYK\_MOUSE tr|A0A087WPF9|A0A087WPF9\_MOUSE sp|Q9CQC6|BZW1\_MOUSE tr|F8VPT6|F8VPT6\_MOUSE sp|P97366|EVI5\_MOUSE sp|Q8K2P7|S38A1\_MOUSE tr|F8VQK7|F8VQK7\_MOUSE sp|P35979|RL12\_MOUSE tr|H3BLB0|H3BLB0\_MOUSE tr|M0QWU4|M0QWU4\_MOUSE sp|Q8K057-2|IFT80\_MOUSE tr|Q3T995|Q3T995\_MOUSE sp|Q8K057|IFT80\_MOUSE tr|D3Z157|D3Z157\_MOUSE tr|E9PUZ0|E9PUZ0\_MOUSE sp|P10820|PERF\_MOUSE sp|Q7TMI3-3|UHRF2\_MOUSE tr|D3YWI8|D3YWI8\_MOUSE tr|F7B673|F7B673\_MOUSE tr|F7D4S5|F7D4S5\_MOUSE sp|Q68FF7-2|SLAI1\_MOUSE sp|Q68FF7|SLAI1\_MOUSE sp|O55047-2|TLK2\_MOUSE tr|B1ASU9|B1ASU9\_MOUSE sp|O55047|TLK2\_MOUSE sp|Q8CBA2|SLFN5\_MOUSE tr|B7ZC85|B7ZC85\_MOUSE sp|O55047-3|TLK2\_MOUSE sp|O08532-3|CA2D1\_MOUSE sp|Q6NZK8-2|PTPC1\_MOUSE tr|F6UYF1|F6UYF1\_MOUSE tr|B8QI35|B8QI35\_MOUSE sp|O08532-4|CA2D1\_MOUSE sp|Q6NZK8-3|PTPC1\_MOUSE sp|P60469|LIPA3\_MOUSE sp|Q6NZK8|PTPC1\_MOUSE sp|P0C1P0|PIGY\_MOUSE sp|F8VQN3|GPR31\_MOUSE sp|E9Q5K9-2|YTDC1\_MOUSE sp|E9Q5K9-3|YTDC1\_MOUSE sp|Q9ERC3|SRTD3\_MOUSE tr|D6RG95|D6RG95\_MOUSE sp|E9Q5K9|YTDC1\_MOUSE tr|A0A087WNT5|A0A087WNT5\_MOUSE sp|P62748|HPCL1\_MOUSE sp|Q9JIY5|HTRA2\_MOUSE tr|E9PYT3|E9PYT3\_MOUSE sp|Q91YH5|ATLA3\_MOUSE sp|Q64127-2|TIF1A\_MOUSE sp|Q91YH5-2|ATLA3\_MOUSE sp|Q64127|TIF1A\_MOUSE tr|D3YX27|D3YX27\_MOUSE tr|D3YX28|D3YX28\_MOUSE sp|Q8BGE5-2|FANCM\_MOUSE tr|E9Q4N4|E9Q4N4\_MOUSE sp|Q8BY87|UBP47\_MOUSE tr|S4R1D4|S4R1D4\_MOUSE sp|Q8BY87-2|UBP47\_MOUSE sp|P58404-2|STRN4\_MOUSE sp|Q9JKA5|GPA33\_MOUSE tr|Q6PHQ9|Q6PHQ9\_MOUSE sp|P58404|STRN4\_MOUSE tr|A3KFU8|A3KFU8\_MOUSE tr|G3X9P1|G3X9P1\_MOUSE tr|Q3TQG9|Q3TQG9\_MOUSE sp|B1AS29|GRIK3\_MOUSE tr|E9Q7P1|E9Q7P1\_MOUSE sp|P0C090|RC3H2\_MOUSE sp|Q8CB27|OTU1\_MOUSE sp|A2AJQ3-2|D19L4\_MOUSE tr|A2AJQ1|A2AJQ1\_MOUSE sp|A2AJQ3|D19L4\_MOUSE sp|Q9DAA6|EXOS1\_MOUSE sp|Q9JK45|KCNQ5\_MOUSE tr|E9Q9F1|E9Q9F1\_MOUSE sp|Q8C2E4|PTCD1\_MOUSE tr|G3UYG5|G3UYG5\_MOUSE sp|Q9CR33|MANS1\_MOUSE tr|E9QAQ1|E9QAQ1\_MOUSE sp|Q6PD26|PIGS\_MOUSE tr|B8JJH0|B8JJH0\_MOUSE sp|Q80VH0|BANK1\_MOUSE sp|Q8CIA5-2|S35B4\_MOUSE sp|Q8CIA5|S35B4\_MOUSE sp|Q1RNF8|SAM11\_MOUSE sp|P28843|DPP4\_MOUSE sp|A0JP43|EFCB5\_MOUSE sp|A0JP43-2|EFCB5\_MOUSE sp|A0JP43-3|EFCB5\_MOUSE tr|F8VQI6|F8VQI6\_MOUSE sp|Q8BK06-2|FBX9\_MOUSE sp|Q8BK06|FBX9\_MOUSE sp|P55088-3|AQP4\_MOUSE sp|P55088|AQP4\_MOUSE sp|Q3V0T4|ITAD\_MOUSE sp|P55088-2|AQP4\_MOUSE tr|H3BKX8|H3BKX8\_MOUSE tr|E9PXZ7|E9PXZ7\_MOUSE tr|F2Z440|F2Z440\_MOUSE tr|A2BIE1|A2BIE1\_MOUSE sp|P55284|CADH5\_MOUSE tr|A0A087WR75|A0A087WR75\_MOUSE sp|Q7TT18|MCAF1\_MOUSE tr|L7N2D4|L7N2D4\_MOUSE sp|Q3TUL7-2|DCA17\_MOUSE sp|Q6P8H8|ALG8\_MOUSE sp|P29351|PTN6\_MOUSE sp|Q9CR35|CTRB1\_MOUSE tr|Z4YK64|Z4YK64\_MOUSE sp|P29351-3|PTN6\_MOUSE sp|Q3TUL7|DCA17\_MOUSE sp|P29351-2|PTN6\_MOUSE sp|P19426-2|NELFE\_MOUSE tr|E9Q2S0|E9Q2S0\_MOUSE sp|P19426|NELFE\_MOUSE sp|Q8VF66|OL469\_MOUSE sp|Q9ER74|SALL1\_MOUSE tr|Z4YJZ2|Z4YJZ2\_MOUSE tr|J3KMP6|J3KMP6\_MOUSE sp|Q8BP22|F92A1\_MOUSE tr|F6VZK2|F6VZK2\_MOUSE tr|Q6P5E3|Q6P5E3\_MOUSE tr|D6RJ18|D6RJ18\_MOUSE sp|Q80X80|C2C2L\_MOUSE sp|A6H5Y1|MPP9\_MOUSE sp|O88398|AVIL\_MOUSE tr|E9Q9D8|E9Q9D8\_MOUSE tr|F6SJS2|F6SJS2\_MOUSE tr|D6RJ73|D6RJ73\_MOUSE tr|D3Z618|D3Z618\_MOUSE sp|Q6P5D4|CP135\_MOUSE sp|Q69Z26|CNTN4\_MOUSE sp|Q9DBM0-2|ABCG8\_MOUSE tr|Q7TSR6|Q7TSR6\_MOUSE tr|E9Q0P2|E9Q0P2\_MOUSE sp|Q9DBM0|ABCG8\_MOUSE tr|F7C3A0|F7C3A0\_MOUSE tr|H3BIW6|H3BIW6\_MOUSE sp|A2AP18-2|PLCH2\_MOUSE sp|B2RX14|TUT4\_MOUSE tr|A2A8R7|A2A8R7\_MOUSE tr|D3Z0U7|D3Z0U7\_MOUSE tr|D3YYT4|D3YYT4\_MOUSE tr|A2CGA5|A2CGA5\_MOUSE sp|Q3UVY5|PCX4\_MOUSE sp|O08863|BIRC3\_MOUSE tr|E9QN69|E9QN69\_MOUSE sp|Q61070|EI24\_MOUSE tr|Q91VE4|Q91VE4\_MOUSE tr|E0CXC2|E0CXC2\_MOUSE ENSEMBL:ENSBTAP00000024466 tr|Q6GQT2|Q6GQT2\_MOUSE tr|F6ZGG4|F6ZGG4\_MOUSE sp|Q6ZQL4|WDR43\_MOUSE sp|Q8R086|SUOX\_MOUSE sp|Q6GQU6|LIGO3\_MOUSE sp|Q60636-3|PRDM1\_MOUSE sp|Q6P2B1|TNPO3\_MOUSE tr|A0A087WS76|A0A087WS76\_MOUSE sp|Q6P2B1-2|TNPO3\_MOUSE sp|Q2TJ95|RSPO3\_MOUSE tr|E9PV38|E9PV38\_MOUSE sp|Q80X50-2|UBP2L\_MOUSE sp|Q80X50-3|UBP2L\_MOUSE sp|Q80X50-5|UBP2L\_MOUSE sp|Q80X50-4|UBP2L\_MOUSE sp|Q80X50|UBP2L\_MOUSE sp|Q3U0J8|TBD2B\_MOUSE tr|Q9MD82|Q9MD82\_MOUSE sp|Q9QZW0-2|AT11C\_MOUSE sp|Q91ZJ0|MUS81\_MOUSE tr|Q9DC83|Q9DC83\_MOUSE sp|Q91ZU6-6|DYST\_MOUSE sp|Q8CB12|GSDC3\_MOUSE tr|E9QKK8|E9QKK8\_MOUSE sp|P03921|NU5M\_MOUSE sp|Q0VAV2|EXPH5\_MOUSE sp|P19070|CR2\_MOUSE sp|Q3TR54|GSDC4\_MOUSE sp|P97290|IC1\_MOUSE tr|Q9DAL4|Q9DAL4\_MOUSE sp|P70425|RIT2\_MOUSE tr|F6Q8D3|F6Q8D3\_MOUSE tr|F8WIN9|F8WIN9\_MOUSE sp|Q60604-2|ADSV\_MOUSE sp|Q9QZW0|AT11C\_MOUSE sp|P35821|PTN1\_MOUSE sp|Q62468|VILI\_MOUSE sp|Q99PE8|ABCG5\_MOUSE sp|Q2KHK6|GSDC2\_MOUSE sp|P12367|KAP2\_MOUSE tr|Q496Q8|Q496Q8\_MOUSE sp|P20615|HXB9\_MOUSE sp|Q9WVS8|MK07\_MOUSE sp|Q9WVS8-4|MK07\_MOUSE sp|Q9WVS8-5|MK07\_MOUSE tr|D3Z7X6|D3Z7X6\_MOUSE sp|Q69ZH9|RHG23\_MOUSE tr|B1AQY2|B1AQY2\_MOUSE sp|D3YZU1|SHAN1\_MOUSE tr|D3YZU5|D3YZU5\_MOUSE tr|D3YZU4|D3YZU4\_MOUSE sp|Q3TI53|SCHI1\_MOUSE sp|Q3TI53-7|SCHI1\_MOUSE sp|Q3T9Z9|ZUFSP\_MOUSE sp|Q3TI53-1|SCHI1\_MOUSE tr|S4R1J0|S4R1J0\_MOUSE sp|Q3TI53-6|SCHI1\_MOUSE sp|Q3TI53-3|SCHI1\_MOUSE sp|Q3T9Z9-3|ZUFSP\_MOUSE tr|F6WIM8|F6WIM8\_MOUSE sp|Q3T9Z9-2|ZUFSP\_MOUSE tr|G5E8X0|G5E8X0\_MOUSE tr|E9PZS9|E9PZS9\_MOUSE sp|Q3UV71|TMTC1\_MOUSE sp|O35099|M3K5\_MOUSE tr|V9GXA0|V9GXA0\_MOUSE sp|Q3UV71-2|TMTC1\_MOUSE tr|E9PWG9|E9PWG9\_MOUSE sp|Q8R332-2|NUPL1\_MOUSE tr|D3Z2U7|D3Z2U7\_MOUSE tr|F6QKL8|F6QKL8\_MOUSE sp|Q8R332-4|NUPL1\_MOUSE tr|D3Z773|D3Z773\_MOUSE sp|Q8R332|NUPL1\_MOUSE sp|P27699-3|CREM\_MOUSE tr|D3Z0R0|D3Z0R0\_MOUSE tr|D3Z011|D3Z011\_MOUSE tr|F6Q346|F6Q346\_MOUSE tr|Q9CQ87|Q9CQ87\_MOUSE tr|Q3TTH6|Q3TTH6\_MOUSE tr|E9PWV9|E9PWV9\_MOUSE sp|P27699-8|CREM\_MOUSE sp|P27699-2|CREM\_MOUSE tr|D3Z7M6|D3Z7M6\_MOUSE tr|E9PV35|E9PV35\_MOUSE sp|P27699-6|CREM\_MOUSE sp|Q8R332-3|NUPL1\_MOUSE tr|F6W4F5|F6W4F5\_MOUSE sp|P27699-7|CREM\_MOUSE tr|F6U9F5|F6U9F5\_MOUSE tr|D3YXV5|D3YXV5\_MOUSE sp|P27699-10|CREM\_MOUSE sp|P27699|CREM\_MOUSE tr|D3Z4K8|D3Z4K8\_MOUSE sp|P27699-5|CREM\_MOUSE sp|P27699-4|CREM\_MOUSE sp|P27699-9|CREM\_MOUSE tr|F6XRJ7|F6XRJ7\_MOUSE tr|F6RB97|F6RB97\_MOUSE sp|Q8CFL8|ZSWM3\_MOUSE sp|Q497V5-2|SRBD1\_MOUSE tr|S4R2S5|S4R2S5\_MOUSE tr|F8WGW3|F8WGW3\_MOUSE sp|Q497V5|SRBD1\_MOUSE tr|E9Q3Q7|E9Q3Q7\_MOUSE tr|Q80ZW1|Q80ZW1\_MOUSE tr|F6ZC60|F6ZC60\_MOUSE sp|P0DI97|NRX1B\_MOUSE sp|Q9CXL1|TM50A\_MOUSE sp|P19473|TUR8\_MOUSE sp|A2AB59|RHG27\_MOUSE tr|A2AKN5|A2AKN5\_MOUSE tr|E9PY34|E9PY34\_MOUSE sp|P10598|CASB\_MOUSE tr|A0A087WRV1|A0A087WRV1\_MOUSE tr|A0A075B641|A0A075B641\_MOUSE sp|Q62261-2|SPTB2\_MOUSE tr|Q8R283|Q8R283\_MOUSE tr|E9PVJ6|E9PVJ6\_MOUSE tr|A0A075B616|A0A075B616\_MOUSE sp|P24668|MPRD\_MOUSE sp|P58501|PAXB1\_MOUSE tr|E9PZ19|E9PZ19\_MOUSE tr|Q91X75|Q91X75\_MOUSE tr|E9Q5D4|E9Q5D4\_MOUSE sp|P20852|CP2A5\_MOUSE tr|D3Z169|D3Z169\_MOUSE sp|Q8R0X7|SGPL1\_MOUSE sp|P58682|TLR8\_MOUSE sp|Q80Z37|TOPRS\_MOUSE tr|D3Z271|D3Z271\_MOUSE tr|Q8BGM4|Q8BGM4\_MOUSE tr|Q8BY07|Q8BY07\_MOUSE sp|Q99MZ6|MYO7B\_MOUSE sp|P46425|GSTP2\_MOUSE tr|S4R278|S4R278\_MOUSE tr|D3Z634|D3Z634\_MOUSE tr|D3Z593|D3Z593\_MOUSE tr|E0CYL5|E0CYL5\_MOUSE tr|D3Z3G4|D3Z3G4\_MOUSE sp|Q80TS3-4|LPHN3\_MOUSE sp|Q75UR0|ANO5\_MOUSE sp|Q75UR0-3|ANO5\_MOUSE sp|Q99M96-2|ST7\_MOUSE tr|D3YUM7|D3YUM7\_MOUSE sp|Q99M96|ST7\_MOUSE sp|Q99M96-3|ST7\_MOUSE sp|Q99M96-5|ST7\_MOUSE sp|Q99M96-4|ST7\_MOUSE sp|Q99M96-7|ST7\_MOUSE sp|Q99M96-6|ST7\_MOUSE tr|H3BJC7|H3BJC7\_MOUSE sp|Q99M96-8|ST7\_MOUSE sp|Q99M96-9|ST7\_MOUSE P13646-1 sp|Q04592|PCSK5\_MOUSE sp|Q60780|GAS7\_MOUSE sp|Q9CZU4|ERAL1\_MOUSE sp|Q8R5F8|ES8L1\_MOUSE tr|Z4YM47|Z4YM47\_MOUSE sp|Q6PE87|CF165\_MOUSE sp|Q6PE87-2|CF165\_MOUSE sp|Q8BW88-4|PKHS1\_MOUSE sp|Q8BW88|PKHS1\_MOUSE sp|Q8BW88-3|PKHS1\_MOUSE sp|A1EGX6|FSCB\_MOUSE tr|E9QJV1|E9QJV1\_MOUSE sp|Q9D273-2|MMAB\_MOUSE sp|Q9D273|MMAB\_MOUSE sp|Q6A065-2|CE170\_MOUSE sp|Q9CPT4|CS010\_MOUSE Q3SZ57 sp|A1YIY0|FCRL6\_MOUSE sp|A1YIY0-2|FCRL6\_MOUSE sp|Q8VCF0|MAVS\_MOUSE tr|E9PUD3|E9PUD3\_MOUSE tr|Q9CY05|Q9CY05\_MOUSE tr|D3YXQ7|D3YXQ7\_MOUSE tr|H3BLG6|H3BLG6\_MOUSE sp|Q8VCY6|UTP6\_MOUSE tr|Z4YLV0|Z4YLV0\_MOUSE sp|O88291|ZN326\_MOUSE sp|Q8R480|NUP85\_MOUSE sp|Q8K0U3|TMM17\_MOUSE sp|Q99K82-8|SMOX\_MOUSE sp|Q9CWP4|LY6K\_MOUSE tr|Q3TLR8|Q3TLR8\_MOUSE sp|Q5DQR4-6|STB5L\_MOUSE tr|K7N686|K7N686\_MOUSE tr|F6RX08|F6RX08\_MOUSE tr|B2KGF7|B2KGF7\_MOUSE sp|Q91WQ5|TAF5L\_MOUSE sp|Q3TMX7-2|QSOX2\_MOUSE sp|Q3TMX7|QSOX2\_MOUSE sp|Q5QD16|TAAR3\_MOUSE tr|D3Z710|D3Z710\_MOUSE sp|Q6NSV7-2|F149B\_MOUSE sp|Q6NSV7-3|F149B\_MOUSE sp|Q6NSV7|F149B\_MOUSE tr|Q7TQS2|Q7TQS2\_MOUSE sp|Q76EC5|CHST9\_MOUSE tr|G5E8S7|G5E8S7\_MOUSE sp|Q61200|NXPH1\_MOUSE sp|Q8VDG3|PARN\_MOUSE tr|E9Q3I4|E9Q3I4\_MOUSE tr|E9Q2L0|E9Q2L0\_MOUSE tr|B2RUJ2|B2RUJ2\_MOUSE tr|B7ZNX6|B7ZNX6\_MOUSE sp|O08796|EF2K\_MOUSE tr|D3Z482|D3Z482\_MOUSE tr|A2APM4|A2APM4\_MOUSE sp|Q9D8G5|REG4\_MOUSE sp|Q8R4A3|DACT1\_MOUSE tr|D3Z5V0|D3Z5V0\_MOUSE sp|A2KF29|SMKTR\_MOUSE sp|P32507-2|PVRL2\_MOUSE sp|Q8C3B8|RFT1\_MOUSE sp|Q9DAZ2|PR2B1\_MOUSE sp|Q00897|A1AT4\_MOUSE tr|Q5SXK2|Q5SXK2\_MOUSE tr|D3YVX5|D3YVX5\_MOUSE sp|Q3UPC7|K0825\_MOUSE sp|Q8C262|IIGP5\_MOUSE tr|Q14BT6|Q14BT6\_MOUSE sp|Q61333|TNAP2\_MOUSE tr|Q7TRG4|Q7TRG4\_MOUSE tr|D3Z4L9|D3Z4L9\_MOUSE tr|F6UPJ1|F6UPJ1\_MOUSE sp|P42128|FOXK1\_MOUSE sp|Q3U0M1|TPPC9\_MOUSE sp|Q3U0M1-3|TPPC9\_MOUSE sp|Q3U0M1-2|TPPC9\_MOUSE tr|Q80YT3|Q80YT3\_MOUSE tr|A2ALW2|A2ALW2\_MOUSE tr|D3Z5B4|D3Z5B4\_MOUSE sp|P97449|AMPN\_MOUSE sp|Q9Z1B3-2|PLCB1\_MOUSE sp|Q9JJ59|ABCB9\_MOUSE sp|Q9JJ59-2|ABCB9\_MOUSE tr|D3YVH7|D3YVH7\_MOUSE sp|Q9Z1B3-3|PLCB1\_MOUSE sp|P50580|PA2G4\_MOUSE sp|Q9Z1B3|PLCB1\_MOUSE tr|Q8C6G3|Q8C6G3\_MOUSE sp|O88879-2|APAF\_MOUSE tr|G3XA09|G3XA09\_MOUSE sp|O88879|APAF\_MOUSE sp|P00683|RNAS1\_MOUSE sp|P57787|MOT4\_MOUSE sp|Q9EQR5|LIME1\_MOUSE tr|A2ABG4|A2ABG4\_MOUSE sp|P00342|LDHC\_MOUSE tr|F6QX71|F6QX71\_MOUSE tr|A2BDX0|A2BDX0\_MOUSE sp|Q8BTU7-2|RCCD1\_MOUSE sp|Q7TQ69|TMC3\_MOUSE tr|E9Q6Y0|E9Q6Y0\_MOUSE tr|E9Q133|E9Q133\_MOUSE tr|F8VQI4|F8VQI4\_MOUSE sp|Q8BTU7|RCCD1\_MOUSE tr|F6Q609|F6Q609\_MOUSE tr|Q3UN10|Q3UN10\_MOUSE sp|Q7TSK3|PCDH8\_MOUSE sp|Q5IBH6|SPDYB\_MOUSE sp|Q9EPB8|V1R54\_MOUSE sp|P56695|WFS1\_MOUSE sp|Q7TSK3-2|PCDH8\_MOUSE tr|A2AJ30|A2AJ30\_MOUSE sp|Q8K0Y7|CI142\_MOUSE sp|P25911-2|LYN\_MOUSE sp|Q6PHS9-5|CA2D2\_MOUSE sp|Q6PHS9-2|CA2D2\_MOUSE sp|P25911|LYN\_MOUSE tr|E9Q683|E9Q683\_MOUSE sp|Q6PHS9-4|CA2D2\_MOUSE sp|Q6PHS9-6|CA2D2\_MOUSE sp|Q6PHS9-3|CA2D2\_MOUSE sp|Q6PHS9|CA2D2\_MOUSE tr|F8WHM5|F8WHM5\_MOUSE sp|Q61543|GSLG1\_MOUSE tr|G3UYX0|G3UYX0\_MOUSE sp|Q8C1A3|MTRR\_MOUSE sp|Q7TQG0|ZBTB5\_MOUSE sp|Q9R1J0|NSDHL\_MOUSE sp|Q62240-4|KDM5D\_MOUSE sp|Q8BRB7-2|KAT6B\_MOUSE tr|F7AT68|F7AT68\_MOUSE sp|O70263-2|LNX1\_MOUSE tr|E9Q4N3|E9Q4N3\_MOUSE tr|E9Q6Q0|E9Q6Q0\_MOUSE sp|O70263|LNX1\_MOUSE sp|Q8CFA1|IRAK2\_MOUSE sp|Q8CFA1-2|IRAK2\_MOUSE sp|Q8CFA1-4|IRAK2\_MOUSE sp|Q80X44-3|ZBT24\_MOUSE tr|Q5RIN0|Q5RIN0\_MOUSE sp|Q9ES64|USH1C\_MOUSE tr|E9PYX1|E9PYX1\_MOUSE sp|P60191|RIMS4\_MOUSE sp|Q9ES64-2|USH1C\_MOUSE tr|D6RIM8|D6RIM8\_MOUSE tr|E9QMN1|E9QMN1\_MOUSE tr|B7ZCM9|B7ZCM9\_MOUSE sp|Q921K8|F115C\_MOUSE tr|Q8CDE5|Q8CDE5\_MOUSE tr|B7ZCN0|B7ZCN0\_MOUSE tr|B7ZCM8|B7ZCM8\_MOUSE sp|P0C871|PA24B\_MOUSE sp|Q3UR85-2|MRF\_MOUSE sp|A7TZF0-2|SKIT3\_MOUSE sp|A7TZF0|SKIT3\_MOUSE tr|A0A067XG49|A0A067XG49\_MOUSE tr|A0A087WSE2|A0A087WSE2\_MOUSE sp|Q8VHG2|AMOT\_MOUSE tr|F6QYS7|F6QYS7\_MOUSE tr|F7CN67|F7CN67\_MOUSE sp|Q8VCE9-3|PKHH3\_MOUSE tr|V9GXP3|V9GXP3\_MOUSE sp|Q6PD19-3|CJ076\_MOUSE tr|D3YY38|D3YY38\_MOUSE tr|D6RHS4|D6RHS4\_MOUSE tr|Q7TNQ7|Q7TNQ7\_MOUSE tr|D3Z5A0|D3Z5A0\_MOUSE tr|Q3UNV2|Q3UNV2\_MOUSE tr|G3X9S3|G3X9S3\_MOUSE sp|Q8VCE9|PKHH3\_MOUSE sp|Q8VCE9-4|PKHH3\_MOUSE tr|Q3U3L6|Q3U3L6\_MOUSE sp|Q8VCE9-2|PKHH3\_MOUSE tr|Q7TRJ0|Q7TRJ0\_MOUSE tr|D3Z599|D3Z599\_MOUSE sp|P34971|ADRB1\_MOUSE tr|H9KUZ0|H9KUZ0\_MOUSE sp|Q9Z2E1|MBD2\_MOUSE sp|Q7TPM9|MTMRA\_MOUSE sp|Q9JJ61|GLT16\_MOUSE tr|E9PY92|E9PY92\_MOUSE tr|E0CYI4|E0CYI4\_MOUSE sp|Q9WUB0|HOIL1\_MOUSE sp|Q8C8J0|STPG2\_MOUSE sp|Q9CZN8|GATA\_MOUSE tr|F8VQC9|F8VQC9\_MOUSE tr|Q5SXG9|Q5SXG9\_MOUSE sp|P20782|ACHE\_MOUSE sp|Q6PGN3-6|DCLK2\_MOUSE tr|A0A087WQF9|A0A087WQF9\_MOUSE tr|Q8VFS6|Q8VFS6\_MOUSE tr|A2A860|A2A860\_MOUSE sp|P97765|WBP2\_MOUSE tr|O08804|O08804\_MOUSE tr|Q8VF17|Q8VF17\_MOUSE tr|Q8VFS5|Q8VFS5\_MOUSE tr|Q8VF18|Q8VF18\_MOUSE tr|E9PXM3|E9PXM3\_MOUSE sp|Q02591|GSC\_MOUSE tr|J3QNK5|J3QNK5\_MOUSE sp|Q8BIH0|SP130\_MOUSE tr|E0CYP4|E0CYP4\_MOUSE sp|P24529|TY3H\_MOUSE sp|Q9WU60|ATRN\_MOUSE tr|F6TH61|F6TH61\_MOUSE tr|E9PUN6|E9PUN6\_MOUSE tr|D3YVL0|D3YVL0\_MOUSE sp|Q8CIK8|RFWD3\_MOUSE sp|Q9CW42|MARC1\_MOUSE sp|P23249|MOV10\_MOUSE tr|A0A087WPC2|A0A087WPC2\_MOUSE sp|Q9ESK9|RBCC1\_MOUSE tr|F7CC56|F7CC56\_MOUSE sp|Q8VC98|PKHA4\_MOUSE sp|Q14CH0|F171B\_MOUSE sp|Q14CH0-2|F171B\_MOUSE tr|E9QJX8|E9QJX8\_MOUSE tr|D3YW21|D3YW21\_MOUSE tr|D3Z3Y7|D3Z3Y7\_MOUSE sp|Q9D4H1|EXOC2\_MOUSE sp|Q8R3V6|CUED1\_MOUSE tr|W8DXL4|W8DXL4\_MOUSE sp|Q810B6|ANFY1\_MOUSE tr|J3QMG5|J3QMG5\_MOUSE sp|Q8BLI4|DSE\_MOUSE sp|Q8BMN4|LMLN\_MOUSE tr|D3Z601|D3Z601\_MOUSE sp|O89104|SYPL2\_MOUSE tr|G3UW83|G3UW83\_MOUSE sp|Q6NZL6|TONSL\_MOUSE tr|Q8VF34|Q8VF34\_MOUSE tr|G3X9F6|G3X9F6\_MOUSE sp|P29037|TBP\_MOUSE tr|E9Q1M6|E9Q1M6\_MOUSE tr|E9PUR0|E9PUR0\_MOUSE tr|G3UZ90|G3UZ90\_MOUSE tr|F6XYI9|F6XYI9\_MOUSE sp|Q3UHQ6|DOP2\_MOUSE sp|Q3UHQ6-2|DOP2\_MOUSE tr|G3UY23|G3UY23\_MOUSE sp|Q8VEB2|SAV1\_MOUSE tr|F6VRG5|F6VRG5\_MOUSE sp|Q8BW70|UBP38\_MOUSE sp|Q80TG1-5|KANL1\_MOUSE tr|E9Q3E2|E9Q3E2\_MOUSE sp|Q8CC35-2|SYNPO\_MOUSE sp|P97328|KHK\_MOUSE tr|E9Q1Q9|E9Q1Q9\_MOUSE tr|E9QL43|E9QL43\_MOUSE sp|Q8K0L9-2|ZBT20\_MOUSE sp|Q8CCG1|ZC21C\_MOUSE tr|V9GXG1|V9GXG1\_MOUSE sp|Q8K0L9|ZBT20\_MOUSE sp|P35917|VGFR3\_MOUSE tr|Q8VH16|Q8VH16\_MOUSE tr|E9Q6H6|E9Q6H6\_MOUSE sp|Q5SUV2-2|MYBPP\_MOUSE sp|Q6B8I0|DS13A\_MOUSE tr|V9GXG6|V9GXG6\_MOUSE tr|E9QA93|E9QA93\_MOUSE sp|P48999|LOX5\_MOUSE tr|Q8R2D8|Q8R2D8\_MOUSE sp|P15208|INSR\_MOUSE sp|Q04692|SMRCD\_MOUSE sp|Q66JQ7|CASC5\_MOUSE sp|Q04692-2|SMRCD\_MOUSE tr|F7CN51|F7CN51\_MOUSE tr|E9QKE5|E9QKE5\_MOUSE sp|Q9JLV6-2|PNKP\_MOUSE tr|A2A7P7|A2A7P7\_MOUSE sp|Q9JLV6|PNKP\_MOUSE tr|E9Q9A5|E9Q9A5\_MOUSE tr|G5E8N7|G5E8N7\_MOUSE sp|Q5DTN8|JKIP3\_MOUSE sp|Q9D6T0|NOSIP\_MOUSE sp|Q5F267|P5I13\_MOUSE sp|Q4VAC9-2|PKHG3\_MOUSE sp|Q5DTN8-2|JKIP3\_MOUSE tr|E9Q7K7|E9Q7K7\_MOUSE sp|Q8BVL9|JKIP1\_MOUSE sp|Q91YW3|DNJC3\_MOUSE sp|Q5DTN8-3|JKIP3\_MOUSE tr|G3UYZ9|G3UYZ9\_MOUSE sp|Q4VAC9|PKHG3\_MOUSE sp|Q9D051|ODPB\_MOUSE tr|D3Z4U5|D3Z4U5\_MOUSE sp|Q9D6T0-2|NOSIP\_MOUSE tr|A2AT40|A2AT40\_MOUSE sp|Q8BTI7|ANR52\_MOUSE sp|P59999|ARPC4\_MOUSE sp|Q8C0P0-2|GWL\_MOUSE sp|Q0KL01|UBX2B\_MOUSE tr|D3YZ98|D3YZ98\_MOUSE sp|Q6PF93|PK3C3\_MOUSE sp|Q6PF93-2|PK3C3\_MOUSE tr|E9QLS6|E9QLS6\_MOUSE sp|Q99LG4|TTC5\_MOUSE sp|Q6PD31|TRAK1\_MOUSE sp|Q80YC5|FA12\_MOUSE tr|B1AXP3|B1AXP3\_MOUSE tr|Q4TU83|Q4TU83\_MOUSE sp|Q8BXX2|ZBT49\_MOUSE tr|H7BX99|H7BX99\_MOUSE sp|Q99NF1|BCDO2\_MOUSE sp|Q5DTI8|ESYT3\_MOUSE tr|A2ANT6|A2ANT6\_MOUSE tr|F6R2G3|F6R2G3\_MOUSE sp|Q99K10|NLGN1\_MOUSE sp|Q8C7R4|UBA6\_MOUSE tr|D3Z061|D3Z061\_MOUSE tr|E9QK34|E9QK34\_MOUSE sp|Q8R2S8|CD177\_MOUSE sp|Q9D2J4|VSIG1\_MOUSE tr|F6PY46|F6PY46\_MOUSE sp|P23772|GATA3\_MOUSE sp|Q80XI4|PI42B\_MOUSE tr|F6UKP3|F6UKP3\_MOUSE tr|G3X8Q6|G3X8Q6\_MOUSE sp|O35643|AP1B1\_MOUSE sp|O08672|KIFC2\_MOUSE tr|Q5SVG5|Q5SVG5\_MOUSE tr|Q5SVG4|Q5SVG4\_MOUSE sp|Q6PD29|ZN513\_MOUSE tr|Q8C3U9|Q8C3U9\_MOUSE sp|Q9JLG8-2|CAN15\_MOUSE tr|H3BJ23|H3BJ23\_MOUSE sp|B7ZMP1|XPP3\_MOUSE tr|F7ALN1|F7ALN1\_MOUSE sp|Q6PD29-2|ZN513\_MOUSE tr|A0A075B659|A0A075B659\_MOUSE sp|Q61241|TSSK1\_MOUSE tr|G3UX35|G3UX35\_MOUSE tr|F7BCP8|F7BCP8\_MOUSE sp|Q8CC21-2|TTC19\_MOUSE sp|Q8C8N2|SCAI\_MOUSE sp|Q9ET54-5|PALLD\_MOUSE sp|Q9CZX2|CEP89\_MOUSE sp|Q9ET54-3|PALLD\_MOUSE sp|Q8CC21|TTC19\_MOUSE tr|E9Q9S2|E9Q9S2\_MOUSE sp|Q9D361|SNR48\_MOUSE sp|Q8CBC4|CNST\_MOUSE tr|G3V024|G3V024\_MOUSE sp|Q80WG5|LRC8A\_MOUSE tr|H7BWY6|H7BWY6\_MOUSE sp|Q3USS3|DRC1\_MOUSE tr|F6WKY8|F6WKY8\_MOUSE sp|Q68FF0|K1841\_MOUSE sp|Q68FF0-2|K1841\_MOUSE sp|Q9DBQ9-2|SWT1\_MOUSE tr|A2BHQ2|A2BHQ2\_MOUSE sp|P12960|CNTN1\_MOUSE sp|B2RXV4|FLVC1\_MOUSE sp|Q80TS3-6|LPHN3\_MOUSE tr|W4VSP8|W4VSP8\_MOUSE tr|S4R2N4|S4R2N4\_MOUSE sp|Q8C753|K0556\_MOUSE sp|Q8C753-3|K0556\_MOUSE sp|Q8C753-4|K0556\_MOUSE sp|Q8C753-2|K0556\_MOUSE tr|F6XQI1|F6XQI1\_MOUSE tr|G3UWZ3|G3UWZ3\_MOUSE sp|Q8VDZ4|ZDHC5\_MOUSE sp|O35660|GSTM6\_MOUSE sp|Q9CZ05-2|AT7L1\_MOUSE tr|Q8VEU6|Q8VEU6\_MOUSE tr|F6RPR7|F6RPR7\_MOUSE sp|Q9CZ05|AT7L1\_MOUSE sp|Q8CHR6|DPYD\_MOUSE sp|P34152-4|FAK1\_MOUSE sp|Q80UF7|TCAM1\_MOUSE sp|B2RXV4-2|FLVC1\_MOUSE sp|Q5U4D8|SC5A6\_MOUSE sp|Q60673|PTPRN\_MOUSE tr|A6MDD2|A6MDD2\_MOUSE tr|D3Z720|D3Z720\_MOUSE sp|Q8R0C0|RPC7L\_MOUSE tr|E9Q7U2|E9Q7U2\_MOUSE sp|Q3TTE0-3|ADAM5\_MOUSE sp|Q8CGU1|CACO1\_MOUSE tr|D3YXS1|D3YXS1\_MOUSE sp|Q61210-5|ARHG1\_MOUSE sp|Q61210-3|ARHG1\_MOUSE sp|Q61210-2|ARHG1\_MOUSE sp|Q61210-4|ARHG1\_MOUSE sp|Q61210|ARHG1\_MOUSE tr|E9PUF7|E9PUF7\_MOUSE tr|Q8VG94|Q8VG94\_MOUSE tr|F8VQD3|F8VQD3\_MOUSE tr|K7N662|K7N662\_MOUSE tr|F6ZN61|F6ZN61\_MOUSE sp|Q3U0V1|FUBP2\_MOUSE sp|Q7M711|TR113\_MOUSE sp|P97471|SMAD4\_MOUSE sp|Q3V384|LACE1\_MOUSE tr|G3X9F4|G3X9F4\_MOUSE sp|Q9WU78-3|PDC6I\_MOUSE tr|F8VQ07|F8VQ07\_MOUSE sp|Q8VD26|TM143\_MOUSE tr|J3QNK9|J3QNK9\_MOUSE sp|Q9WU78|PDC6I\_MOUSE sp|A2AKY4|Z804A\_MOUSE sp|Q8VD26-2|TM143\_MOUSE tr|D6RI80|D6RI80\_MOUSE sp|Q5SVT3-2|ETAA1\_MOUSE tr|Q7TQV1|Q7TQV1\_MOUSE sp|Q5SVT3-3|ETAA1\_MOUSE sp|Q5SVT3|ETAA1\_MOUSE sp|Q9Z2W9|GRIA3\_MOUSE tr|H3BJ52|H3BJ52\_MOUSE sp|P70290|EM55\_MOUSE sp|Q8CCE9-2|E4F1\_MOUSE tr|G3X9Z0|G3X9Z0\_MOUSE tr|A2AN84|A2AN84\_MOUSE sp|Q8R060|ZWILC\_MOUSE sp|Q8CCE9-3|E4F1\_MOUSE tr|Q6NV92|Q6NV92\_MOUSE tr|Q3URY5|Q3URY5\_MOUSE tr|H3BKB9|H3BKB9\_MOUSE tr|L7N456|L7N456\_MOUSE tr|Q8BX23|Q8BX23\_MOUSE sp|Q8CCE9-4|E4F1\_MOUSE tr|L7N458|L7N458\_MOUSE sp|Q9Z2W8|GRIA4\_MOUSE sp|Q8CCE9|E4F1\_MOUSE tr|B7ZCL8|B7ZCL8\_MOUSE sp|O55144|TLX3\_MOUSE sp|P59034|LRRC3\_MOUSE sp|Q0V8T8-2|CTP5B\_MOUSE sp|Q0V8T8|CTP5B\_MOUSE sp|Q9JIE3|OTOR\_MOUSE sp|Q9CPP7|LIPG\_MOUSE sp|Q8K0C1-2|IPO13\_MOUSE sp|Q8K0C1|IPO13\_MOUSE tr|Q3V3P1|Q3V3P1\_MOUSE sp|Q8BHN3-3|GANAB\_MOUSE sp|O70157|TOP3A\_MOUSE sp|Q8BHN3-2|GANAB\_MOUSE tr|Q5NCT0|Q5NCT0\_MOUSE tr|B0QZI1|B0QZI1\_MOUSE sp|Q8BHN3|GANAB\_MOUSE sp|Q9QWT9|KIFC1\_MOUSE tr|F6Z4B2|F6Z4B2\_MOUSE tr|Q5EBP8|Q5EBP8\_MOUSE sp|Q9QWT9-2|KIFC1\_MOUSE tr|A2AA66|A2AA66\_MOUSE tr|D3Z600|D3Z600\_MOUSE sp|O09012-2|PEX5\_MOUSE sp|Q6P1H6-2|ANKL2\_MOUSE tr|Q812F3|Q812F3\_MOUSE sp|Q6P1H6|ANKL2\_MOUSE tr|G8JL40|G8JL40\_MOUSE sp|Q811F1|ZBT41\_MOUSE sp|Q6P1H6-3|ANKL2\_MOUSE sp|A6H6A4-2|LRIQ4\_MOUSE sp|Q8BM85|TBCK\_MOUSE sp|P06339|HA15\_MOUSE tr|Q5U4C5|Q5U4C5\_MOUSE sp|Q8VI75|IPO4\_MOUSE sp|Q8BM85-2|TBCK\_MOUSE tr|E9PZA8|E9PZA8\_MOUSE sp|Q6ZQ03|FNBP4\_MOUSE sp|Q8VIM5-3|MYCD\_MOUSE sp|Q6ZQ03-3|FNBP4\_MOUSE sp|Q6ZQ03-2|FNBP4\_MOUSE sp|Q920P5-2|KAD5\_MOUSE sp|Q61771|KIF3B\_MOUSE sp|Q920P5|KAD5\_MOUSE tr|Q9R0U9|Q9R0U9\_MOUSE sp|P98200|AT8A2\_MOUSE tr|F6UFZ5|F6UFZ5\_MOUSE sp|Q6KAU7-2|PKHG2\_MOUSE sp|Q8R4G6|MGT5A\_MOUSE tr|F8VQ03|F8VQ03\_MOUSE tr|Q6P907|Q6P907\_MOUSE sp|Q9D3A9-5|TTYH1\_MOUSE tr|A2ALX7|A2ALX7\_MOUSE tr|G3UZI7|G3UZI7\_MOUSE sp|O54834-2|RHG06\_MOUSE tr|A2ALX6|A2ALX6\_MOUSE sp|P0C242|CCNO\_MOUSE tr|A2ABR0|A2ABR0\_MOUSE sp|Q9JJS0|SCUB2\_MOUSE sp|O88566|AXIN2\_MOUSE sp|Q7TN79|AKA7G\_MOUSE sp|Q9D8N0|EF1G\_MOUSE tr|A2AEP2|A2AEP2\_MOUSE tr|G3XA34|G3XA34\_MOUSE sp|Q810T2|CCNB3\_MOUSE sp|Q6PGE4|ZF316\_MOUSE sp|E9PVB3|CC175\_MOUSE sp|Q99K01|PDXD1\_MOUSE sp|Q99K01-2|PDXD1\_MOUSE sp|Q99K01-3|PDXD1\_MOUSE sp|Q99K01-4|PDXD1\_MOUSE tr|E9PZ44|E9PZ44\_MOUSE sp|Q7TMY7|IPO8\_MOUSE sp|Q7TMY7-2|IPO8\_MOUSE sp|P59270|B3GA2\_MOUSE tr|Q9EQ95|Q9EQ95\_MOUSE sp|P59270-2|B3GA2\_MOUSE tr|D3YY32|D3YY32\_MOUSE tr|Q9EQB8|Q9EQB8\_MOUSE tr|G3UW35|G3UW35\_MOUSE tr|E9QAQ5|E9QAQ5\_MOUSE sp|Q9WV60|GSK3B\_MOUSE sp|Q811Q9-2|PCY1B\_MOUSE tr|E9Q9Y3|E9Q9Y3\_MOUSE sp|Q8BV42|ZN786\_MOUSE sp|Q3UCQ1|FOXK2\_MOUSE sp|Q80TT8-4|CUL9\_MOUSE sp|Q3UCQ1-2|FOXK2\_MOUSE sp|P12382|PFKAL\_MOUSE sp|Q62266|SPR1A\_MOUSE sp|Q8BSN9|FAKD3\_MOUSE sp|Q8BSN9-2|FAKD3\_MOUSE tr|D3YVN5|D3YVN5\_MOUSE sp|Q8C407|YIPF4\_MOUSE sp|Q8C407-2|YIPF4\_MOUSE sp|Q3TY92|MBD6\_MOUSE sp|A2AWA9-2|RBGP1\_MOUSE tr|D3YVB6|D3YVB6\_MOUSE sp|A2AWA9|RBGP1\_MOUSE sp|P30545|ADA2B\_MOUSE sp|Q9JJZ2|TBA8\_MOUSE tr|Q925K6|Q925K6\_MOUSE sp|Q8BIZ6|SNIP1\_MOUSE tr|F8VQ23|F8VQ23\_MOUSE tr|F6VSK8|F6VSK8\_MOUSE sp|Q8BI79-2|CCD40\_MOUSE sp|Q8C0X8|SMKX\_MOUSE sp|Q8BM81|ADCL4\_MOUSE sp|Q9Z131-3|3BP5\_MOUSE tr|Q8VEU8|Q8VEU8\_MOUSE tr|D3Z1A5|D3Z1A5\_MOUSE sp|Q8CFZ4|GPC3\_MOUSE tr|H3BK87|H3BK87\_MOUSE sp|Q9D0R4|DDX56\_MOUSE sp|Q8BLY7|HPS6\_MOUSE sp|Q9QXH4|ITAX\_MOUSE tr|D3Z7A8|D3Z7A8\_MOUSE tr|B8JJH3|B8JJH3\_MOUSE tr|Q1WNP4|Q1WNP4\_MOUSE tr|Q3UU56|Q3UU56\_MOUSE sp|Q6NVG1|LPCT4\_MOUSE tr|E9QAL2|E9QAL2\_MOUSE sp|Q8BL74|TF3C2\_MOUSE tr|E0CXE2|E0CXE2\_MOUSE sp|Q8C7H1|MMAA\_MOUSE sp|Q9JIK5|DDX21\_MOUSE tr|B2RW38|B2RW38\_MOUSE tr|D3Z707|D3Z707\_MOUSE tr|D3Z5P2|D3Z5P2\_MOUSE sp|Q8BLH5|TSP50\_MOUSE sp|Q6PFX2-3|BEND6\_MOUSE tr|A2A652|A2A652\_MOUSE sp|Q00915|RET1\_MOUSE tr|F2Z493|F2Z493\_MOUSE sp|Q6PFX2|BEND6\_MOUSE sp|P37913|DNLI1\_MOUSE sp|Q7TSS2-2|UB2Q1\_MOUSE sp|Q7TSS2|UB2Q1\_MOUSE sp|Q9JHB2|OLF19\_MOUSE sp|F6VAN0|ATF6A\_MOUSE tr|A2AAU5|A2AAU5\_MOUSE sp|Q6DFV5-2|HELZ\_MOUSE tr|G3UZX8|G3UZX8\_MOUSE tr|A2AAU3|A2AAU3\_MOUSE sp|Q02257|PLAK\_MOUSE sp|Q7TQ65-2|TMC4\_MOUSE sp|P97411|ICA69\_MOUSE tr|D3Z118|D3Z118\_MOUSE sp|P97411-2|ICA69\_MOUSE tr|S4R217|S4R217\_MOUSE tr|D3Z376|D3Z376\_MOUSE sp|Q8BPX9|S15A3\_MOUSE tr|D3Z6H5|D3Z6H5\_MOUSE sp|Q8C167-3|PPCEL\_MOUSE sp|Q3UZ01|RBM40\_MOUSE sp|Q3UZ01-2|RBM40\_MOUSE sp|Q3UZD7|F71F1\_MOUSE sp|P97378|I12R2\_MOUSE sp|Q9CU24|THMS3\_MOUSE sp|Q640N1-2|AEBP1\_MOUSE sp|Q99MI1-4|RB6I2\_MOUSE sp|P17717|UDB17\_MOUSE sp|Q80X73|PELO\_MOUSE tr|Q8K169|Q8K169\_MOUSE sp|Q91VJ1|AIM2\_MOUSE tr|F8WHV4|F8WHV4\_MOUSE sp|Q0VF58|COJA1\_MOUSE tr|F6VMI1|F6VMI1\_MOUSE tr|Z4YK94|Z4YK94\_MOUSE tr|G3UZ13|G3UZ13\_MOUSE tr|F6QMB7|F6QMB7\_MOUSE tr|E9Q7B0|E9Q7B0\_MOUSE tr|Q8CCA6|Q8CCA6\_MOUSE sp|Q60715-2|P4HA1\_MOUSE sp|Q60715|P4HA1\_MOUSE tr|D3Z7H1|D3Z7H1\_MOUSE tr|D6RCK1|D6RCK1\_MOUSE tr|G3UWJ0|G3UWJ0\_MOUSE sp|Q08297|RAD51\_MOUSE tr|F6XVH0|F6XVH0\_MOUSE tr|A2AGE0|A2AGE0\_MOUSE tr|G3UXL4|G3UXL4\_MOUSE tr|Q5U4B1|Q5U4B1\_MOUSE tr|H3BKB7|H3BKB7\_MOUSE tr|F6RBX1|F6RBX1\_MOUSE sp|Q00196-2|PO2F2\_MOUSE tr|A3KGD2|A3KGD2\_MOUSE tr|Q5SXA5|Q5SXA5\_MOUSE sp|Q6DFV7|NCOA7\_MOUSE sp|Q61656|DDX5\_MOUSE tr|Q8BTS0|Q8BTS0\_MOUSE tr|S4R1I6|S4R1I6\_MOUSE sp|Q9WUN2|TBK1\_MOUSE tr|E9PWU7|E9PWU7\_MOUSE sp|O35231-2|KIFC3\_MOUSE sp|O35231|KIFC3\_MOUSE sp|Q61142|SPIN1\_MOUSE sp|Q9EQG3|SCEL\_MOUSE sp|Q61142-2|SPIN1\_MOUSE tr|Q3V2D1|Q3V2D1\_MOUSE sp|Q63994|CD33\_MOUSE tr|D3YUS5|D3YUS5\_MOUSE tr|E9PW37|E9PW37\_MOUSE tr|Q9CZ61|Q9CZ61\_MOUSE tr|E9PW09|E9PW09\_MOUSE sp|Q99MJ6|NHRF4\_MOUSE sp|Q8BH88|DEP1B\_MOUSE tr|A6X8Z9|A6X8Z9\_MOUSE sp|Q8K3R3-3|PLCD4\_MOUSE sp|O08585|CLCA\_MOUSE tr|Q6PFA2|Q6PFA2\_MOUSE tr|B1AWE1|B1AWE1\_MOUSE tr|F6YN80|F6YN80\_MOUSE tr|B1AWD8|B1AWD8\_MOUSE tr|B1AWE0|B1AWE0\_MOUSE tr|B1AWD9|B1AWD9\_MOUSE tr|D3YUF3|D3YUF3\_MOUSE sp|O35690|PHX2B\_MOUSE tr|E9QPM7|E9QPM7\_MOUSE tr|E9PY13|E9PY13\_MOUSE tr|E9PYI4|E9PYI4\_MOUSE tr|G3UXP9|G3UXP9\_MOUSE sp|Q62371|DDR2\_MOUSE tr|D3YUF4|D3YUF4\_MOUSE sp|Q64096|MCF2L\_MOUSE tr|E9PXE2|E9PXE2\_MOUSE tr|Q8C741|Q8C741\_MOUSE sp|O70582|LX12B\_MOUSE tr|G3UX72|G3UX72\_MOUSE tr|E9Q863|E9Q863\_MOUSE tr|E9PY12|E9PY12\_MOUSE tr|E9PXE1|E9PXE1\_MOUSE tr|D3Z4E7|D3Z4E7\_MOUSE tr|Q99N75|Q99N75\_MOUSE sp|Q9R0H0-2|ACOX1\_MOUSE tr|E9QKB6|E9QKB6\_MOUSE tr|F7B335|F7B335\_MOUSE tr|D3YV10|D3YV10\_MOUSE sp|Q9R0H0|ACOX1\_MOUSE tr|A2AKI0|A2AKI0\_MOUSE tr|D3Z3H4|D3Z3H4\_MOUSE sp|P0C1Q2|PDE11\_MOUSE sp|Q6AW69-3|CGNL1\_MOUSE sp|E9Q6B2|CC85C\_MOUSE sp|Q33DR3-3|DLP1\_MOUSE sp|Q8BGH2|SAM50\_MOUSE sp|Q8BGC9|CREG2\_MOUSE tr|K7N6B6|K7N6B6\_MOUSE sp|Q9WTK5|NFKB2\_MOUSE sp|Q6RHW0|K1C9\_MOUSE sp|Q91VH2|SNX9\_MOUSE sp|Q9DCD2|SYF1\_MOUSE tr|G3XA53|G3XA53\_MOUSE tr|G3X8V6|G3X8V6\_MOUSE sp|Q63912|OMGP\_MOUSE sp|Q3UMW7|MAPK3\_MOUSE sp|Q8K4J2|COE4\_MOUSE sp|Q00196-3|PO2F2\_MOUSE sp|Q8K4J2-4|COE4\_MOUSE sp|Q00196|PO2F2\_MOUSE sp|Q60780-2|GAS7\_MOUSE sp|Q00196-4|PO2F2\_MOUSE tr|Q3U432|Q3U432\_MOUSE tr|B1ATI9|B1ATI9\_MOUSE sp|Q8K4J2-2|COE4\_MOUSE tr|F6VR53|F6VR53\_MOUSE tr|A2BI80|A2BI80\_MOUSE sp|Q99PU5|ACBG1\_MOUSE sp|Q00196-6|PO2F2\_MOUSE tr|Q8VFW5|Q8VFW5\_MOUSE tr|D3Z4E0|D3Z4E0\_MOUSE sp|O09046-2|OXLA\_MOUSE sp|O09046|OXLA\_MOUSE sp|Q8R2K1-4|FUCM\_MOUSE tr|Q8R0S9|Q8R0S9\_MOUSE sp|Q00612|G6PD1\_MOUSE tr|Q6PCP3|Q6PCP3\_MOUSE tr|A3KG36|A3KG36\_MOUSE sp|Q8R0K9|E2F4\_MOUSE sp|Q9EPK5|WWTR1\_MOUSE tr|E9Q3T8|E9Q3T8\_MOUSE sp|Q9Z0R6-2|ITSN2\_MOUSE tr|E9PZ41|E9PZ41\_MOUSE sp|P61315|G3ST3\_MOUSE P07224 sp|Q99PL5-12|RRBP1\_MOUSE sp|Q99PL5-11|RRBP1\_MOUSE sp|Q9DCT8|CRIP2\_MOUSE sp|Q99PL5-5|RRBP1\_MOUSE sp|Q99PL5-7|RRBP1\_MOUSE sp|Q9QY15|DDX25\_MOUSE tr|B7ZMW1|B7ZMW1\_MOUSE sp|Q99PL5-3|RRBP1\_MOUSE sp|Q99PL5-10|RRBP1\_MOUSE sp|Q0VET5|LMTD2\_MOUSE sp|Q9QY15-2|DDX25\_MOUSE tr|J3QNJ4|J3QNJ4\_MOUSE tr|H9H9T3|H9H9T3\_MOUSE sp|Q99PL5-6|RRBP1\_MOUSE sp|Q99PL5-2|RRBP1\_MOUSE sp|Q99PL5-9|RRBP1\_MOUSE sp|Q99PL5-8|RRBP1\_MOUSE sp|Q99PL5-4|RRBP1\_MOUSE sp|Q61885|MOG\_MOUSE tr|D3YXH8|D3YXH8\_MOUSE sp|Q3UV74|ITB2L\_MOUSE tr|F6U4F1|F6U4F1\_MOUSE sp|Q3UV74-3|ITB2L\_MOUSE sp|Q80YR7|CLSPN\_MOUSE sp|Q5U462|CDCP1\_MOUSE sp|Q3UV74-2|ITB2L\_MOUSE sp|Q61001|LAMA5\_MOUSE sp|Q80SX8-3|PIF1\_MOUSE sp|P97377-2|CDK2\_MOUSE tr|E9Q7C1|E9Q7C1\_MOUSE tr|F7C033|F7C033\_MOUSE tr|Q7TMC8|Q7TMC8\_MOUSE tr|G3X8S4|G3X8S4\_MOUSE sp|Q924H2|MED15\_MOUSE sp|P97377|CDK2\_MOUSE sp|A2AKK5|ACNT1\_MOUSE tr|Q7TRM0|Q7TRM0\_MOUSE sp|A2AKK5-2|ACNT1\_MOUSE tr|G3UYQ4|G3UYQ4\_MOUSE tr|F6T9T6|F6T9T6\_MOUSE tr|Q8VG49|Q8VG49\_MOUSE tr|E9PWU0|E9PWU0\_MOUSE tr|F8WJ31|F8WJ31\_MOUSE tr|A2AVP4|A2AVP4\_MOUSE tr|S4R2S6|S4R2S6\_MOUSE sp|Q9JHH2|TSN32\_MOUSE tr|E9Q149|E9Q149\_MOUSE sp|O70362|PHLD\_MOUSE sp|Q8CG85|MAMC2\_MOUSE tr|D3YZB3|D3YZB3\_MOUSE sp|A2AKQ8|T30A2\_MOUSE tr|Q8VHE1|Q8VHE1\_MOUSE sp|P48758|CBR1\_MOUSE sp|P10925|ZFY1\_MOUSE tr|Q149D4|Q149D4\_MOUSE sp|Q80W68|KIRR1\_MOUSE sp|Q32MD9|CDON\_MOUSE tr|A2AVC0|A2AVC0\_MOUSE tr|B1ARY8|B1ARY8\_MOUSE sp|Q8C7V3|UTP15\_MOUSE tr|B1AU42|B1AU42\_MOUSE tr|B1AU43|B1AU43\_MOUSE sp|Q8BLF2-7|CDKL3\_MOUSE sp|Q8BLF2-8|CDKL3\_MOUSE tr|E9PW51|E9PW51\_MOUSE tr|E9Q4Q6|E9Q4Q6\_MOUSE tr|F2Z3Z6|F2Z3Z6\_MOUSE sp|P12961|7B2\_MOUSE sp|Q8BLF2|CDKL3\_MOUSE tr|F7A487|F7A487\_MOUSE sp|Q66PY1-2|SCUB3\_MOUSE sp|Q925U4|EDEM1\_MOUSE sp|Q8BLF2-6|CDKL3\_MOUSE sp|Q8BLF2-2|CDKL3\_MOUSE sp|Q8BLF2-5|CDKL3\_MOUSE tr|A0A087WS92|A0A087WS92\_MOUSE sp|Q66PY1|SCUB3\_MOUSE tr|D3Z2R0|D3Z2R0\_MOUSE sp|Q8CJ19-4|MICA3\_MOUSE sp|P51432|PLCB3\_MOUSE tr|A2A7T3|A2A7T3\_MOUSE sp|Q80TZ9|RERE\_MOUSE sp|Q62052|P\_MOUSE sp|P10637-3|TAU\_MOUSE sp|Q9R0H2-3|MUCEN\_MOUSE sp|Q9R0H2|MUCEN\_MOUSE tr|G3X9F5|G3X9F5\_MOUSE sp|Q62381|TLL1\_MOUSE sp|Q9CQS9-2|HAUS2\_MOUSE sp|Q5SXA9|KIBRA\_MOUSE sp|Q9CQS9|HAUS2\_MOUSE sp|O35698|RBY1A\_MOUSE sp|Q60990|RBY1B\_MOUSE tr|D3Z700|D3Z700\_MOUSE tr|L7N1Z9|L7N1Z9\_MOUSE tr|B2RWR6|B2RWR6\_MOUSE tr|J3QK27|J3QK27\_MOUSE tr|A0A087WR82|A0A087WR82\_MOUSE tr|A0A087WPY7|A0A087WPY7\_MOUSE tr|G5E825|G5E825\_MOUSE sp|Q8CGE9|RGS12\_MOUSE tr|D3Z0G6|D3Z0G6\_MOUSE tr|E9Q652|E9Q652\_MOUSE tr|Q3TTW9|Q3TTW9\_MOUSE sp|A2AJN7|S4A11\_MOUSE tr|D3Z0G5|D3Z0G5\_MOUSE tr|D3Z0G7|D3Z0G7\_MOUSE tr|E9Q1K7|E9Q1K7\_MOUSE sp|Q9JIA1|LGI1\_MOUSE tr|E9Q945|E9Q945\_MOUSE sp|Q8K4P8|HECW1\_MOUSE tr|E0CZ26|E0CZ26\_MOUSE tr|Q0PD08|Q0PD08\_MOUSE sp|Q8K4P8-2|HECW1\_MOUSE sp|Q8C0I1|ADAS\_MOUSE tr|H3BKN2|H3BKN2\_MOUSE tr|E9PZC2|E9PZC2\_MOUSE tr|F7D6F6|F7D6F6\_MOUSE tr|A2AL50|A2AL50\_MOUSE sp|P05532-2|KIT\_MOUSE sp|P05532|KIT\_MOUSE tr|E9Q8T2|E9Q8T2\_MOUSE tr|E9Q6A1|E9Q6A1\_MOUSE tr|D3Z1E3|D3Z1E3\_MOUSE tr|Q99L59|Q99L59\_MOUSE tr|S4R186|S4R186\_MOUSE tr|F6QP00|F6QP00\_MOUSE sp|Q9EPW9|TLR6\_MOUSE tr|Q3UV88|Q3UV88\_MOUSE tr|D3Z4L5|D3Z4L5\_MOUSE tr|D3Z5P8|D3Z5P8\_MOUSE tr|Q62029|Q62029\_MOUSE sp|Q810H5|GALP\_MOUSE tr|D3Z6Y5|D3Z6Y5\_MOUSE sp|Q8K1B8|URP2\_MOUSE tr|F8VQ95|F8VQ95\_MOUSE sp|Q6Y685|TACC1\_MOUSE sp|P16301|LCAT\_MOUSE tr|F7DCB1|F7DCB1\_MOUSE sp|Q8CE96|TRM6\_MOUSE sp|Q9JIS5|SV2A\_MOUSE tr|D3Z622|D3Z622\_MOUSE tr|A0A087WPT8|A0A087WPT8\_MOUSE tr|G5E8Y6|G5E8Y6\_MOUSE sp|Q922H4|GMPPA\_MOUSE sp|Q99MS7-2|EH1L1\_MOUSE tr|Q5SU71|Q5SU71\_MOUSE sp|Q01776|GNRHR\_MOUSE sp|P27773|PDIA3\_MOUSE sp|Q8K296|MTMR3\_MOUSE tr|B1ATD5|B1ATD5\_MOUSE sp|Q9JLM9|GRB14\_MOUSE tr|M0QW74|M0QW74\_MOUSE tr|R4GML8|R4GML8\_MOUSE sp|Q8K296-2|MTMR3\_MOUSE tr|B1ATD4|B1ATD4\_MOUSE sp|Q9CQF0|RM11\_MOUSE tr|Q8BS01|Q8BS01\_MOUSE sp|Q60677|ITAE\_MOUSE sp|P56657|CP240\_MOUSE tr|E9PXC3|E9PXC3\_MOUSE tr|E9PUD1|E9PUD1\_MOUSE tr|Z4YMB8|Z4YMB8\_MOUSE sp|Q99LE3|LYSM3\_MOUSE tr|A0A087WQB7|A0A087WQB7\_MOUSE sp|Q58A65-6|JIP4\_MOUSE sp|Q58A65-2|JIP4\_MOUSE sp|Q58A65-3|JIP4\_MOUSE sp|Q58A65-4|JIP4\_MOUSE tr|E9Q7C8|E9Q7C8\_MOUSE sp|Q8C1D8|IWS1\_MOUSE tr|E0CYU2|E0CYU2\_MOUSE sp|Q58A65-5|JIP4\_MOUSE tr|F8WH31|F8WH31\_MOUSE tr|Q569X9|Q569X9\_MOUSE sp|P42866-4|OPRM\_MOUSE sp|Q7TSG1-2|CE120\_MOUSE sp|Q7TSG1|CE120\_MOUSE sp|P42866-2|OPRM\_MOUSE sp|P42866-13|OPRM\_MOUSE sp|P42866-14|OPRM\_MOUSE sp|P42866-5|OPRM\_MOUSE sp|P42866-19|OPRM\_MOUSE sp|P42866-6|OPRM\_MOUSE tr|E9Q2X5|E9Q2X5\_MOUSE sp|P42866-10|OPRM\_MOUSE sp|P42866-16|OPRM\_MOUSE sp|P42866|OPRM\_MOUSE sp|Q8BUI3|LRWD1\_MOUSE sp|P42866-7|OPRM\_MOUSE sp|P42866-9|OPRM\_MOUSE sp|P42866-15|OPRM\_MOUSE sp|P42866-12|OPRM\_MOUSE sp|P42866-11|OPRM\_MOUSE sp|P42866-8|OPRM\_MOUSE sp|P42866-3|OPRM\_MOUSE sp|P42866-17|OPRM\_MOUSE tr|E9Q7D4|E9Q7D4\_MOUSE tr|E9Q7D3|E9Q7D3\_MOUSE sp|E9Q6Z5|AUNIP\_MOUSE tr|F6RND9|F6RND9\_MOUSE tr|G3X9G7|G3X9G7\_MOUSE tr|Q8BJ45|Q8BJ45\_MOUSE tr|A2AVB0|A2AVB0\_MOUSE sp|Q9JIP4|PANX1\_MOUSE tr|F6YNX9|F6YNX9\_MOUSE tr|G5E8Z6|G5E8Z6\_MOUSE sp|Q9Z1F6|LECT1\_MOUSE tr|Q3UEB4|Q3UEB4\_MOUSE tr|D3YV77|D3YV77\_MOUSE sp|Q9R008|KIME\_MOUSE sp|Q5HZI1|MTUS1\_MOUSE sp|Q9R099|TBL2\_MOUSE sp|Q8CDC7|ZBTB9\_MOUSE sp|Q8VCA6-3|T161A\_MOUSE sp|Q8VCA6-2|T161A\_MOUSE sp|Q9EQ47|V1R44\_MOUSE tr|F6UNW2|F6UNW2\_MOUSE sp|Q3UXL4|KIZ\_MOUSE tr|Q76M72|Q76M72\_MOUSE tr|Q76M71|Q76M71\_MOUSE sp|Q99K82-7|SMOX\_MOUSE sp|Q61967|ZFP90\_MOUSE sp|Q99K82-6|SMOX\_MOUSE sp|Q3ULD5|MCCB\_MOUSE sp|Q69ZL1|FGD6\_MOUSE sp|O88286-3|WIZ\_MOUSE tr|A2AER4|A2AER4\_MOUSE sp|Q8CGN4|BCOR\_MOUSE sp|B2RQC2|UBP42\_MOUSE tr|A2AQH1|A2AQH1\_MOUSE tr|Q61635|Q61635\_MOUSE tr|Q7JCZ3|Q7JCZ3\_MOUSE sp|Q8CGN4-4|BCOR\_MOUSE sp|Q8CGN4-3|BCOR\_MOUSE sp|P00158|CYB\_MOUSE sp|Q8CGN4-2|BCOR\_MOUSE sp|Q9R0M8|S35A2\_MOUSE tr|E9Q3N1|E9Q3N1\_MOUSE sp|Q09143|CTR1\_MOUSE sp|Q8K3G9|DP13B\_MOUSE sp|Q61753|SERA\_MOUSE tr|L7N1Z7|L7N1Z7\_MOUSE tr|L7N2E9|L7N2E9\_MOUSE tr|H3BL20|H3BL20\_MOUSE sp|Q9CW03|SMC3\_MOUSE tr|H7BXB6|H7BXB6\_MOUSE sp|Q80U30-4|CL16A\_MOUSE sp|Q80U30-3|CL16A\_MOUSE sp|Q80U30-2|CL16A\_MOUSE sp|Q8BVG5|GLT14\_MOUSE sp|Q8R2S1|RPKL1\_MOUSE tr|Z4YLG3|Z4YLG3\_MOUSE tr|G5E908|G5E908\_MOUSE tr|G3UWB7|G3UWB7\_MOUSE sp|O88898-3|P63\_MOUSE tr|S4R181|S4R181\_MOUSE sp|O88898|P63\_MOUSE sp|Q9D517|PLCC\_MOUSE sp|Q9CZ69|CKLF6\_MOUSE sp|Q3UHD9-2|AGAP2\_MOUSE sp|Q3UHD9|AGAP2\_MOUSE tr|G3UVW9|G3UVW9\_MOUSE sp|Q8VCB1|NDC1\_MOUSE sp|P61982|1433G\_MOUSE tr|S4R1N2|S4R1N2\_MOUSE tr|G3UZL7|G3UZL7\_MOUSE tr|E9QM19|E9QM19\_MOUSE sp|Q9D5Z5|MSS51\_MOUSE sp|Q8R1J1-2|TM6S2\_MOUSE sp|Q9JIC3|DCR1A\_MOUSE sp|Q8R1J1|TM6S2\_MOUSE tr|Q80ZM5|Q80ZM5\_MOUSE sp|Q00422|GABPA\_MOUSE sp|Q8C4U2|TM145\_MOUSE sp|Q6P9Q6-2|FKB15\_MOUSE sp|Q6P9Q6|FKB15\_MOUSE tr|Q5EC55|Q5EC55\_MOUSE sp|Q00560|IL6RB\_MOUSE tr|Q0P521|Q0P521\_MOUSE sp|Q3UHA3-2|SPTCS\_MOUSE sp|P50396|GDIA\_MOUSE sp|P43136|NR2F6\_MOUSE sp|Q9R0C8-3|VAV3\_MOUSE sp|Q9R0C8|VAV3\_MOUSE tr|G3UY81|G3UY81\_MOUSE tr|E9PZ15|E9PZ15\_MOUSE sp|Q80Z71|TENN\_MOUSE tr|E9Q686|E9Q686\_MOUSE tr|E9Q1F6|E9Q1F6\_MOUSE tr|G5E8N5|G5E8N5\_MOUSE sp|P06151|LDHA\_MOUSE sp|Q3UL36-2|ARGL1\_MOUSE tr|E9Q994|E9Q994\_MOUSE tr|D3YZQ9|D3YZQ9\_MOUSE tr|D3Z736|D3Z736\_MOUSE sp|Q3UL36|ARGL1\_MOUSE tr|D3YXQ2|D3YXQ2\_MOUSE sp|Q8R081|HNRPL\_MOUSE tr|G5E924|G5E924\_MOUSE sp|Q60954-2|MEIS1\_MOUSE tr|S4R2H6|S4R2H6\_MOUSE sp|Q91YI1|ATG13\_MOUSE sp|P08551|NFL\_MOUSE tr|S4R2F3|S4R2F3\_MOUSE tr|Q3UVI3|Q3UVI3\_MOUSE sp|O88898-4|P63\_MOUSE sp|O88898-2|P63\_MOUSE sp|P86176|TIGIT\_MOUSE sp|Q8R3B7-2|BRD8\_MOUSE sp|Q3UHX0-2|NOL8\_MOUSE sp|Q8BSL4|HS3S5\_MOUSE sp|Q6GQX6|ANKS6\_MOUSE tr|D3Z6R8|D3Z6R8\_MOUSE tr|Q641K9|Q641K9\_MOUSE tr|F2Z3W4|F2Z3W4\_MOUSE tr|A2ANT9|A2ANT9\_MOUSE sp|Q62179|SEM4B\_MOUSE sp|Q3V140-2|ACRBP\_MOUSE sp|P11627|L1CAM\_MOUSE tr|E9Q8W4|E9Q8W4\_MOUSE tr|A2AFL5|A2AFL5\_MOUSE tr|Q8VFW2|Q8VFW2\_MOUSE tr|F6WND1|F6WND1\_MOUSE tr|E9Q174|E9Q174\_MOUSE tr|E9PVU0|E9PVU0\_MOUSE tr|E9Q3L1|E9Q3L1\_MOUSE tr|V9GXM4|V9GXM4\_MOUSE tr|E9Q175|E9Q175\_MOUSE tr|V9GX76|V9GX76\_MOUSE tr|K7N608|K7N608\_MOUSE sp|Q8BYI9-2|TENR\_MOUSE sp|Q8BYI9|TENR\_MOUSE tr|E9QAG4|E9QAG4\_MOUSE tr|D3Z2G6|D3Z2G6\_MOUSE tr|E9Q4Z6|E9Q4Z6\_MOUSE tr|E9Q2C3|E9Q2C3\_MOUSE sp|Q8C008|DZAN1\_MOUSE sp|Q61702|ITIH1\_MOUSE tr|F8WJ05|F8WJ05\_MOUSE sp|Q9ERE8|MESD1\_MOUSE tr|F6YIN5|F6YIN5\_MOUSE tr|F6VJC5|F6VJC5\_MOUSE sp|O35185|BHE40\_MOUSE sp|Q8K310|MATR3\_MOUSE tr|L7N1X2|L7N1X2\_MOUSE sp|Q62132|PTPRR\_MOUSE tr|A0A087WPQ7|A0A087WPQ7\_MOUSE tr|A0A087WQE1|A0A087WQE1\_MOUSE sp|Q9ERZ6|FIGN\_MOUSE sp|P18155|MTDC\_MOUSE sp|Q6EAL8|IL31\_MOUSE sp|Q91ZV3|DCBD2\_MOUSE tr|E0CX25|E0CX25\_MOUSE tr|D3YZT2|D3YZT2\_MOUSE tr|Q8BVD2|Q8BVD2\_MOUSE sp|P61460-2|DEPD5\_MOUSE sp|P61460|DEPD5\_MOUSE sp|Q8R311|CTGE5\_MOUSE sp|Q8BW75|AOFB\_MOUSE sp|Q9CQS4|S2546\_MOUSE sp|Q5SV77-3|GGNB2\_MOUSE sp|Q5SV77-2|GGNB2\_MOUSE sp|Q8VCV9|NAGTA\_MOUSE sp|Q6A0A9|F120A\_MOUSE tr|D3Z0Q9|D3Z0Q9\_MOUSE sp|Q8BKI2-2|TNR6B\_MOUSE sp|Q9ESW4-2|AGK\_MOUSE sp|Q9ESW4|AGK\_MOUSE sp|Q922Z0|OXDD\_MOUSE sp|Q8BX02-2|KANK2\_MOUSE sp|Q60I26-3|AL2CL\_MOUSE sp|Q9D799|FMT\_MOUSE tr|A2AUF7|A2AUF7\_MOUSE tr|A0A075B5X2|A0A075B5X2\_MOUSE sp|Q8BX02|KANK2\_MOUSE tr|E9PY38|E9PY38\_MOUSE sp|Q3UZY0-5|SFI1\_MOUSE sp|Q99M31|HSP7E\_MOUSE tr|Q5NC01|Q5NC01\_MOUSE sp|Q99M31-2|HSP7E\_MOUSE tr|A8Y5K4|A8Y5K4\_MOUSE sp|Q5HZI1-2|MTUS1\_MOUSE sp|Q5HZI1-3|MTUS1\_MOUSE sp|Q6P1E1-2|ZMIZ1\_MOUSE sp|Q6P1E1|ZMIZ1\_MOUSE sp|Q9JLV2-2|TP4AP\_MOUSE sp|Q6R5N8|TLR13\_MOUSE sp|Q8C7R7-3|RFX6\_MOUSE sp|P97784|CRY1\_MOUSE sp|Q8C7R7-2|RFX6\_MOUSE sp|Q8C7R7|RFX6\_MOUSE sp|Q9QY39|PDZD4\_MOUSE sp|Q8BI29|SARG\_MOUSE tr|Q8VFF1|Q8VFF1\_MOUSE tr|A2AFG3|A2AFG3\_MOUSE sp|Q8BMS1|ECHA\_MOUSE tr|A2A7Q5|A2A7Q5\_MOUSE sp|B2RR83|YTDC2\_MOUSE sp|Q3V1T4-2|P3H1\_MOUSE tr|B0R106|B0R106\_MOUSE tr|A6PW84|A6PW84\_MOUSE tr|A2AER3|A2AER3\_MOUSE sp|Q3V1T4-3|P3H1\_MOUSE sp|Q3UST5|CP089\_MOUSE tr|F6QH15|F6QH15\_MOUSE sp|Q3V1T4|P3H1\_MOUSE sp|Q99LL3|CHSTC\_MOUSE sp|Q61412|VSX2\_MOUSE sp|Q05AH6-2|CK084\_MOUSE sp|Q05AH6|CK084\_MOUSE tr|L7N2D0|L7N2D0\_MOUSE tr|E9Q9C5|E9Q9C5\_MOUSE tr|F7AB82|F7AB82\_MOUSE sp|O54916|REPS1\_MOUSE sp|P10852|4F2\_MOUSE sp|P63082|VATL\_MOUSE sp|O54916-4|REPS1\_MOUSE tr|Q8R5L1|Q8R5L1\_MOUSE sp|Q3UTJ2-3|SRBS2\_MOUSE sp|Q3UTJ2-4|SRBS2\_MOUSE sp|P10852-2|4F2\_MOUSE tr|F8WJ67|F8WJ67\_MOUSE sp|Q9WUB7|CLCKA\_MOUSE sp|Q0GA42|CNNM1\_MOUSE sp|Q922M5|CDA7L\_MOUSE tr|H3BJV7|H3BJV7\_MOUSE tr|Q8BY78|Q8BY78\_MOUSE sp|Q9Z191|EYA4\_MOUSE sp|O88974-3|SETB1\_MOUSE tr|A2A5J6|A2A5J6\_MOUSE sp|O35615|FOG1\_MOUSE sp|Q9DBD5|PELP1\_MOUSE sp|Q14B62|PTHD1\_MOUSE tr|D3YZ97|D3YZ97\_MOUSE sp|Q14B62-2|PTHD1\_MOUSE tr|F8VQ11|F8VQ11\_MOUSE sp|P0DJF2|PT117\_MOUSE tr|D3YUB7|D3YUB7\_MOUSE tr|A0A087WPN2|A0A087WPN2\_MOUSE sp|Q3UH53-2|SDK1\_MOUSE sp|Q9D565|WDR64\_MOUSE sp|Q9D565-2|WDR64\_MOUSE tr|L7N215|L7N215\_MOUSE tr|E9QM22|E9QM22\_MOUSE sp|Q3UH53|SDK1\_MOUSE tr|Q8R2D0|Q8R2D0\_MOUSE tr|D3Z2V4|D3Z2V4\_MOUSE sp|O35664|INAR2\_MOUSE sp|Q149B8|PERM1\_MOUSE sp|O08792|COE2\_MOUSE tr|A2AJ38|A2AJ38\_MOUSE tr|B1AS97|B1AS97\_MOUSE sp|Q8BJ63-2|AP5M1\_MOUSE sp|Q5XJY5|COPD\_MOUSE sp|P33215|NEDD1\_MOUSE sp|Q6R5P0|TLR11\_MOUSE tr|V9GWT0|V9GWT0\_MOUSE sp|Q8BTC1|CL073\_MOUSE tr|V9GXG7|V9GXG7\_MOUSE tr|Q8C897|Q8C897\_MOUSE tr|Q9D9G4|Q9D9G4\_MOUSE sp|Q9CXZ1|NDUS4\_MOUSE tr|Q8BSL0|Q8BSL0\_MOUSE tr|D3Z716|D3Z716\_MOUSE sp|Q64332|SYN2\_MOUSE sp|Q64332-2|SYN2\_MOUSE sp|Q91X05|PODO\_MOUSE sp|Q80T85|DCAF5\_MOUSE sp|Q8CI43|MYL6B\_MOUSE sp|Q8JZK9|HMCS1\_MOUSE sp|Q8CI96-3|CLIP4\_MOUSE tr|A0A087WRT4|A0A087WRT4\_MOUSE tr|A0A087WR52|A0A087WR52\_MOUSE sp|Q8K2K6|AGFG1\_MOUSE sp|Q8K2K6-1|AGFG1\_MOUSE tr|A0A087WSR7|A0A087WSR7\_MOUSE tr|A0A087WNV1|A0A087WNV1\_MOUSE sp|Q8K2K6-2|AGFG1\_MOUSE tr|Q3UZG4|Q3UZG4\_MOUSE sp|P31230|AIMP1\_MOUSE sp|Q8BWY7-3|S39AB\_MOUSE sp|Q3V300|KIF22\_MOUSE sp|Q8BWY7|S39AB\_MOUSE sp|Q8BWY7-2|S39AB\_MOUSE sp|A2AX52|CO6A4\_MOUSE tr|F6V9M5|F6V9M5\_MOUSE tr|G3UZZ3|G3UZZ3\_MOUSE tr|Q3T9X3|Q3T9X3\_MOUSE sp|Q3UHJ0|AAK1\_MOUSE sp|Q3UHJ0-2|AAK1\_MOUSE sp|Q8BJS4|SUN2\_MOUSE tr|A8Y5G9|A8Y5G9\_MOUSE sp|Q8BI79-3|CCD40\_MOUSE sp|Q8BJS4-3|SUN2\_MOUSE tr|E0CY39|E0CY39\_MOUSE tr|E0CY32|E0CY32\_MOUSE sp|Q8BJS4-2|SUN2\_MOUSE sp|Q8QZX5|LSM10\_MOUSE tr|H3BJ55|H3BJ55\_MOUSE sp|Q8C8V1-4|ZXDC\_MOUSE sp|P32299-2|BKRB2\_MOUSE sp|Q9CZ91|SRFB1\_MOUSE sp|Q8R2K1-3|FUCM\_MOUSE sp|Q9QZQ1|AFAD\_MOUSE sp|Q9QZQ1-2|AFAD\_MOUSE tr|E9PYX7|E9PYX7\_MOUSE tr|E9Q852|E9Q852\_MOUSE tr|E9Q9C3|E9Q9C3\_MOUSE sp|Q91VY5-2|KDM4B\_MOUSE sp|Q6GQT9|NOMO1\_MOUSE tr|A0A087WQG8|A0A087WQG8\_MOUSE sp|Q99MT2|MSH4\_MOUSE tr|E9QLE2|E9QLE2\_MOUSE tr|F6UTR6|F6UTR6\_MOUSE tr|E9QL25|E9QL25\_MOUSE sp|Q8BIK4-2|DOCK9\_MOUSE tr|D3YVU4|D3YVU4\_MOUSE tr|D3YVU7|D3YVU7\_MOUSE sp|Q8BIK4|DOCK9\_MOUSE sp|Q6P1G2|KDM2B\_MOUSE tr|D6RFP8|D6RFP8\_MOUSE tr|E9QMR2|E9QMR2\_MOUSE tr|F8VPN7|F8VPN7\_MOUSE tr|G3UYU7|G3UYU7\_MOUSE sp|Q9D0V8|CINP\_MOUSE tr|A6H6S0|A6H6S0\_MOUSE sp|Q5SUR0|PUR4\_MOUSE tr|F8WJA0|F8WJA0\_MOUSE sp|Q9ESV0|DDX24\_MOUSE tr|K7N728|K7N728\_MOUSE tr|E9Q5D8|E9Q5D8\_MOUSE tr|E9Q5C7|E9Q5C7\_MOUSE tr|F6W043|F6W043\_MOUSE tr|E9Q9B0|E9Q9B0\_MOUSE sp|O35071|KIF1C\_MOUSE sp|Q8JZP3|KLHL2\_MOUSE sp|Q8K0D5|EFGM\_MOUSE tr|F6QJK4|F6QJK4\_MOUSE tr|D3YUR1|D3YUR1\_MOUSE sp|P51175|PPOX\_MOUSE tr|E9QKG6|E9QKG6\_MOUSE sp|Q9ERF3|WDR61\_MOUSE tr|D6RDC7|D6RDC7\_MOUSE sp|Q8K3F2|MMP21\_MOUSE sp|Q91V14|S12A5\_MOUSE tr|D6RJ72|D6RJ72\_MOUSE sp|Q91V14-2|S12A5\_MOUSE tr|G5E8W1|G5E8W1\_MOUSE tr|Q8BVQ0|Q8BVQ0\_MOUSE sp|A1Y9I9|TOMT\_MOUSE tr|Q8BI57|Q8BI57\_MOUSE tr|Q3UHS1|Q3UHS1\_MOUSE sp|Q9EPR5|SORC2\_MOUSE tr|E9QPX3|E9QPX3\_MOUSE tr|G3X9X5|G3X9X5\_MOUSE tr|A2AQE9|A2AQE9\_MOUSE tr|V9GXX5|V9GXX5\_MOUSE sp|P16951-2|ATF2\_MOUSE sp|Q69ZJ7|RIC1\_MOUSE tr|F6S7U1|F6S7U1\_MOUSE sp|Q7M761-2|OVCH2\_MOUSE tr|E9QPA1|E9QPA1\_MOUSE sp|Q61324|ARNT2\_MOUSE sp|Q9DBY0-2|FOXP4\_MOUSE sp|Q9DBY0-3|FOXP4\_MOUSE tr|D3Z726|D3Z726\_MOUSE sp|Q3TTE0|ADAM5\_MOUSE tr|E0CY15|E0CY15\_MOUSE tr|D3Z5K9|D3Z5K9\_MOUSE sp|Q80Z38-2|SHAN2\_MOUSE sp|Q80Z38|SHAN2\_MOUSE tr|B1AWC9|B1AWC9\_MOUSE sp|Q8C437-4|PEX5R\_MOUSE sp|Q8C437|PEX5R\_MOUSE tr|F8SLP9|F8SLP9\_MOUSE sp|P01634|KV5A2\_MOUSE tr|D3YYH0|D3YYH0\_MOUSE sp|P70298|CUX2\_MOUSE sp|Q9JIY2-3|HAKAI\_MOUSE sp|P05622|PGFRB\_MOUSE sp|Q9JIY2|HAKAI\_MOUSE tr|A0A087WPK1|A0A087WPK1\_MOUSE tr|F6UMH9|F6UMH9\_MOUSE tr|A0A087WSE1|A0A087WSE1\_MOUSE sp|Q8VDI7|UBAC1\_MOUSE sp|Q9JIY2-2|HAKAI\_MOUSE tr|E9QPE2|E9QPE2\_MOUSE sp|Q9JIY2-4|HAKAI\_MOUSE tr|E9QN12|E9QN12\_MOUSE tr|E9QM14|E9QM14\_MOUSE sp|O54826|AF10\_MOUSE sp|Q3TWF6|WDR70\_MOUSE sp|Q91VC7|PP14A\_MOUSE tr|A2AS70|A2AS70\_MOUSE tr|E9Q4J3|E9Q4J3\_MOUSE tr|A0A087WSM9|A0A087WSM9\_MOUSE tr|G3X934|G3X934\_MOUSE tr|Q7TRY2|Q7TRY2\_MOUSE sp|Q91YL7-2|PG2IP\_MOUSE sp|Q91YL7|PG2IP\_MOUSE sp|Q9JJF2|SPAG4\_MOUSE sp|Q923T9-3|KCC2G\_MOUSE sp|Q923T9-2|KCC2G\_MOUSE sp|Q923T9|KCC2G\_MOUSE tr|B1AQG3|B1AQG3\_MOUSE tr|J3KMQ6|J3KMQ6\_MOUSE tr|E9Q7E9|E9Q7E9\_MOUSE sp|Q3V3Q7|PACS2\_MOUSE sp|Q9CYI0|NJMU\_MOUSE sp|Q99JY9|ARP3\_MOUSE tr|E9Q3X4|E9Q3X4\_MOUSE sp|Q8C0T9|ADCYA\_MOUSE sp|P59438|HPS5\_MOUSE sp|O89109|KCNN4\_MOUSE sp|Q8C561-3|LMBD2\_MOUSE sp|Q8K2A8|ALG3\_MOUSE sp|P54818|GALC\_MOUSE sp|Q5SV06|SPT22\_MOUSE Q3MHH8 tr|B0QZP0|B0QZP0\_MOUSE sp|A2AC93|DNAI2\_MOUSE sp|Q8BN78|KAISO\_MOUSE tr|A2A3Z2|A2A3Z2\_MOUSE sp|A2AC93-2|DNAI2\_MOUSE tr|R4GML5|R4GML5\_MOUSE sp|Q8CHS2|DJC22\_MOUSE tr|A0A087WSM0|A0A087WSM0\_MOUSE tr|A2A6U3|A2A6U3\_MOUSE sp|Q9Z176|P2R3D\_MOUSE tr|E9Q7A5|E9Q7A5\_MOUSE tr|E9Q8R5|E9Q8R5\_MOUSE sp|Q80UG5-3|SEPT9\_MOUSE tr|A0A087WS63|A0A087WS63\_MOUSE sp|Q80UG5|SEPT9\_MOUSE tr|E9PXW0|E9PXW0\_MOUSE tr|A2AK24|A2AK24\_MOUSE tr|Q8CF60|Q8CF60\_MOUSE sp|Q9EQZ7-2|RIMS2\_MOUSE tr|F7CZC4|F7CZC4\_MOUSE tr|D3YZD1|D3YZD1\_MOUSE tr|D3YYU4|D3YYU4\_MOUSE sp|Q8C4X2|KC1G3\_MOUSE tr|Q8C266|Q8C266\_MOUSE sp|O70273-3|EHF\_MOUSE tr|E9Q9F7|E9Q9F7\_MOUSE tr|A2AJ26|A2AJ26\_MOUSE tr|F6Q7Q7|F6Q7Q7\_MOUSE sp|P41234|ABCA2\_MOUSE tr|E9PUA5|E9PUA5\_MOUSE sp|P86045|TM207\_MOUSE tr|G3UXN2|G3UXN2\_MOUSE sp|Q3U0M1-4|TPPC9\_MOUSE sp|Q8R1G2|CMBL\_MOUSE sp|O88427-2|CAC1H\_MOUSE sp|O88427|CAC1H\_MOUSE sp|Q8C4X1|CENPX\_MOUSE sp|Q3TDN0|DISP1\_MOUSE sp|Q8C4X1-2|CENPX\_MOUSE tr|E9Q6P9|E9Q6P9\_MOUSE tr|A2AC07|A2AC07\_MOUSE sp|Q6ZQ29-2|TAOK2\_MOUSE sp|Q64321|ZBT7B\_MOUSE sp|Q3TZX8-3|NOL9\_MOUSE sp|Q9WU81-2|SPX2\_MOUSE sp|Q9WU81|SPX2\_MOUSE tr|B1AXP0|B1AXP0\_MOUSE sp|Q3TZX8|NOL9\_MOUSE sp|Q3TZX8-2|NOL9\_MOUSE tr|B1AXN8|B1AXN8\_MOUSE sp|Q3TTI8|YL021\_MOUSE tr|B1AXN9|B1AXN9\_MOUSE tr|F6UG16|F6UG16\_MOUSE sp|Q3TTI8-2|YL021\_MOUSE tr|B1AXN5|B1AXN5\_MOUSE sp|P18654|KS6A3\_MOUSE sp|Q91Z49-2|UIF\_MOUSE sp|A2ARP1|VIP1\_MOUSE sp|Q91Z49-3|UIF\_MOUSE sp|A2ARP1-2|VIP1\_MOUSE sp|Q91Z49|UIF\_MOUSE sp|A2ARP1-3|VIP1\_MOUSE sp|P55850|DSC3\_MOUSE sp|Q8R0J1|PKHG6\_MOUSE sp|Q3ZT31|SNX25\_MOUSE tr|Q8BLB5|Q8BLB5\_MOUSE sp|P55850-2|DSC3\_MOUSE tr|E9Q1K0|E9Q1K0\_MOUSE tr|G5E8S6|G5E8S6\_MOUSE tr|E9Q941|E9Q941\_MOUSE tr|F6RSC2|F6RSC2\_MOUSE tr|Q8CAE5|Q8CAE5\_MOUSE sp|Q8BGZ4-2|CDC23\_MOUSE tr|D3Z4M4|D3Z4M4\_MOUSE sp|Q8C310-3|ROBO4\_MOUSE sp|Q8BZN2|KCNV1\_MOUSE sp|Q3UR70|TGFA1\_MOUSE sp|Q8BHL3|TB10B\_MOUSE sp|Q8CEC6|PPWD1\_MOUSE sp|P02716|ACHD\_MOUSE sp|O09110|MP2K3\_MOUSE sp|O09110-2|MP2K3\_MOUSE sp|Q7TT37|ELP1\_MOUSE sp|Q5U431|GPR39\_MOUSE sp|O88856|TPST2\_MOUSE sp|Q059U7|INTU\_MOUSE tr|Q3TQN1|Q3TQN1\_MOUSE sp|Q8R124|KLH36\_MOUSE tr|Q3TR11|Q3TR11\_MOUSE sp|Q99JN2-2|KLH22\_MOUSE sp|Q6PAN7|PRR18\_MOUSE tr|D6RDF3|D6RDF3\_MOUSE tr|D3YV11|D3YV11\_MOUSE sp|Q9EQG9-2|C43BP\_MOUSE sp|Q9EQG9|C43BP\_MOUSE tr|Q8CEC2|Q8CEC2\_MOUSE sp|Q8BWT1|THIM\_MOUSE sp|O88492-2|PLIN4\_MOUSE sp|Q66X01-2|NAL9C\_MOUSE tr|E9PUI4|E9PUI4\_MOUSE sp|Q8VDP3|MICA1\_MOUSE tr|H3BJS0|H3BJS0\_MOUSE sp|Q8VDP3-3|MICA1\_MOUSE sp|Q80V62|FACD2\_MOUSE tr|E9Q6L9|E9Q6L9\_MOUSE sp|P50283|CD7\_MOUSE sp|Q5QNQ6|OSBP2\_MOUSE tr|Q6PDB7|Q6PDB7\_MOUSE sp|A2A7Z8|ADCL3\_MOUSE tr|F6U6L3|F6U6L3\_MOUSE tr|H3BKU0|H3BKU0\_MOUSE sp|Q8C0N2|GPAT3\_MOUSE sp|Q80YQ2|MED23\_MOUSE tr|D3YUE3|D3YUE3\_MOUSE tr|F8WJB0|F8WJB0\_MOUSE sp|Q8CCJ9-2|P20L1\_MOUSE tr|E9QNV2|E9QNV2\_MOUSE sp|Q3TWI9|CSCL2\_MOUSE sp|Q80YQ2-2|MED23\_MOUSE sp|Q9JK37|MYOZ1\_MOUSE sp|Q6P1G2-3|KDM2B\_MOUSE sp|Q8BI72|CARF\_MOUSE tr|A9C471|A9C471\_MOUSE sp|P10649|GSTM1\_MOUSE tr|F6WHQ7|F6WHQ7\_MOUSE sp|Q9QUK4|BIR1B\_MOUSE tr|Q5SU48|Q5SU48\_MOUSE tr|H9H9R6|H9H9R6\_MOUSE sp|Q7TN98-5|CPEB4\_MOUSE sp|Q7TN98-2|CPEB4\_MOUSE tr|Q99PL7|Q99PL7\_MOUSE sp|P13516|ACOD1\_MOUSE sp|P13011|ACOD2\_MOUSE tr|Q3KQM4|Q3KQM4\_MOUSE sp|Q9CPX5-4|HRSL5\_MOUSE tr|G3UVU1|G3UVU1\_MOUSE sp|Q9CPX5|HRSL5\_MOUSE tr|E9PVX5|E9PVX5\_MOUSE tr|Q52KG3|Q52KG3\_MOUSE tr|F6S0H0|F6S0H0\_MOUSE sp|Q04888|SOX10\_MOUSE tr|A0A087WQD6|A0A087WQD6\_MOUSE tr|A0A087WSU2|A0A087WSU2\_MOUSE sp|Q80V42|CBPM\_MOUSE sp|O35082|KLOT\_MOUSE tr|B2KF89|B2KF89\_MOUSE tr|D3Z2K3|D3Z2K3\_MOUSE sp|Q920A7-2|AFG31\_MOUSE sp|Q61481-2|PDE1A\_MOUSE sp|Q61481|PDE1A\_MOUSE tr|Q5I7S8|Q5I7S8\_MOUSE tr|Q8BRR9|Q8BRR9\_MOUSE tr|F6X0V1|F6X0V1\_MOUSE tr|Q9JLL9|Q9JLL9\_MOUSE sp|P53351|PLK2\_MOUSE tr|Q9WVA7|Q9WVA7\_MOUSE tr|A0A087WR56|A0A087WR56\_MOUSE sp|Q3U564|DCP1B\_MOUSE sp|Q3U564-2|DCP1B\_MOUSE tr|B9EIX0|B9EIX0\_MOUSE sp|Q8VE97|SRSF4\_MOUSE tr|A2A837|A2A837\_MOUSE tr|Q542V3|Q542V3\_MOUSE sp|Q6AZB0-4|BOC\_MOUSE tr|D3Z763|D3Z763\_MOUSE sp|Q8R0W1|CIPC\_MOUSE sp|Q6AZB0-3|BOC\_MOUSE sp|Q8R0W1-2|CIPC\_MOUSE tr|Z4YJJ0|Z4YJJ0\_MOUSE sp|Q6AZB0|BOC\_MOUSE sp|Q9JKL5|CHP3\_MOUSE tr|D0EM46|D0EM46\_MOUSE sp|P58281-2|OPA1\_MOUSE sp|Q9CXG9|PHF19\_MOUSE sp|P58281|OPA1\_MOUSE sp|Q02053|UBA1\_MOUSE tr|F6YZ61|F6YZ61\_MOUSE tr|D3YU50|D3YU50\_MOUSE tr|F6RQD1|F6RQD1\_MOUSE sp|P43404|ZAP70\_MOUSE sp|P80314|TCPB\_MOUSE tr|Q6P6L5|Q6P6L5\_MOUSE sp|Q6P1E7|PRIPO\_MOUSE sp|Q04207|TF65\_MOUSE sp|Q04207-2|TF65\_MOUSE tr|G5E8C2|G5E8C2\_MOUSE sp|Q811T9-3|DISC1\_MOUSE sp|Q811T9|DISC1\_MOUSE sp|Q91XD6|VPS36\_MOUSE sp|Q811T9-2|DISC1\_MOUSE sp|P55097|CATK\_MOUSE sp|Q8K4B2|IRAK3\_MOUSE tr|A2CGA3|A2CGA3\_MOUSE sp|P0CAX8|TGAP1\_MOUSE tr|E9Q440|E9Q440\_MOUSE sp|Q7TSP5|VTCN1\_MOUSE sp|Q5ND28|SREC\_MOUSE tr|B7ZC28|B7ZC28\_MOUSE sp|Q6P9S7|GLT10\_MOUSE tr|H3BL19|H3BL19\_MOUSE tr|H3BKH2|H3BKH2\_MOUSE tr|H3BJI6|H3BJI6\_MOUSE tr|H3BKL6|H3BKL6\_MOUSE tr|E9PY90|E9PY90\_MOUSE tr|D3Z3H0|D3Z3H0\_MOUSE sp|P15306|TRBM\_MOUSE tr|Q8R359|Q8R359\_MOUSE sp|Q8VE52|OGRL1\_MOUSE sp|A2ASQ1-3|AGRIN\_MOUSE tr|D3Z5P5|D3Z5P5\_MOUSE sp|P40694|SMBP2\_MOUSE tr|E9Q2K2|E9Q2K2\_MOUSE tr|M0QWP1|M0QWP1\_MOUSE sp|A2ASQ1|AGRIN\_MOUSE tr|Q9Z1M2|Q9Z1M2\_MOUSE tr|Z4YK85|Z4YK85\_MOUSE sp|A2ASQ1-2|AGRIN\_MOUSE sp|Q8BLR2|CPNE4\_MOUSE tr|E9Q572|E9Q572\_MOUSE tr|D6RDD8|D6RDD8\_MOUSE tr|D3Z210|D3Z210\_MOUSE sp|B2RPU2|PLHD1\_MOUSE sp|Q8BWL5-5|RBMS3\_MOUSE sp|Q8BWL5-2|RBMS3\_MOUSE sp|P63075|FGF17\_MOUSE tr|G3UZJ9|G3UZJ9\_MOUSE sp|Q8BWL5-3|RBMS3\_MOUSE sp|Q8BWL5-6|RBMS3\_MOUSE sp|Q8BWL5|RBMS3\_MOUSE sp|Q8BWL5-4|RBMS3\_MOUSE tr|F7CUP3|F7CUP3\_MOUSE tr|K7N652|K7N652\_MOUSE tr|E9PW61|E9PW61\_MOUSE tr|B2KFA6|B2KFA6\_MOUSE tr|A2AWA7|A2AWA7\_MOUSE sp|Q8BHN5|RBM45\_MOUSE tr|B2KFA7|B2KFA7\_MOUSE sp|A2AWA9-3|RBGP1\_MOUSE tr|Q9DAG7|Q9DAG7\_MOUSE tr|Q9DAR2|Q9DAR2\_MOUSE sp|Q3UZ18|ICE2\_MOUSE sp|P21183|IL5RA\_MOUSE sp|P01725|LV1C\_MOUSE tr|G3UYF3|G3UYF3\_MOUSE sp|Q8K1L2|SPIN4\_MOUSE sp|Q6IRU5-2|CLCB\_MOUSE sp|Q99LJ2|ABTB1\_MOUSE sp|Q6IRU5-3|CLCB\_MOUSE sp|Q6IRU5|CLCB\_MOUSE sp|Q8BVA5-2|CB043\_MOUSE sp|Q8BVA5|CB043\_MOUSE tr|H3BLF9|H3BLF9\_MOUSE tr|D3Z7F5|D3Z7F5\_MOUSE tr|H3BLJ1|H3BLJ1\_MOUSE tr|H3BIX8|H3BIX8\_MOUSE sp|Q66X03|NAL9A\_MOUSE tr|A2AC95|A2AC95\_MOUSE tr|H3BJ59|H3BJ59\_MOUSE tr|H3BL15|H3BL15\_MOUSE tr|E9QKM4|E9QKM4\_MOUSE sp|Q66X03-2|NAL9A\_MOUSE sp|P61963|DCAF7\_MOUSE sp|Q80VY9|DHX33\_MOUSE tr|A3QK59|A3QK59\_MOUSE tr|K3W4M7|K3W4M7\_MOUSE tr|A2AIX0|A2AIX0\_MOUSE sp|Q9JII1|INP5E\_MOUSE sp|P15379-4|CD44\_MOUSE sp|P15379|CD44\_MOUSE tr|A2APM2|A2APM2\_MOUSE sp|P15379-12|CD44\_MOUSE sp|P15379-11|CD44\_MOUSE tr|E9QKM8|E9QKM8\_MOUSE sp|P15379-9|CD44\_MOUSE sp|P15379-7|CD44\_MOUSE sp|P15379-8|CD44\_MOUSE sp|P15379-13|CD44\_MOUSE tr|A2APM1|A2APM1\_MOUSE sp|Q5ND04|HSF5\_MOUSE sp|A2AVZ9|S43A3\_MOUSE sp|Q3V0C1-2|ZMAT1\_MOUSE tr|G5E8E4|G5E8E4\_MOUSE tr|Z4YL92|Z4YL92\_MOUSE tr|E9Q0M0|E9Q0M0\_MOUSE tr|E9Q1L6|E9Q1L6\_MOUSE tr|E9Q929|E9Q929\_MOUSE sp|Q9Z127|LAT1\_MOUSE tr|D3YZC7|D3YZC7\_MOUSE sp|Q9JLN6-3|ADA28\_MOUSE sp|Q9JLN6|ADA28\_MOUSE tr|D6RGM3|D6RGM3\_MOUSE sp|P31650|S6A11\_MOUSE sp|Q9JLN6-2|ADA28\_MOUSE sp|Q75NR7-2|RECQ4\_MOUSE sp|Q9D738|ASB12\_MOUSE tr|E9Q329|E9Q329\_MOUSE tr|Q5SW50|Q5SW50\_MOUSE tr|Q5SW49|Q5SW49\_MOUSE sp|Q8BI22|CE128\_MOUSE sp|Q75NR7|RECQ4\_MOUSE sp|Q8C811|S35E2\_MOUSE tr|Q6RKD8|Q6RKD8\_MOUSE tr|G3XA51|G3XA51\_MOUSE tr|F6Q1R4|F6Q1R4\_MOUSE sp|Q9D7W5|MED8\_MOUSE sp|Q8BTT6|DIEXF\_MOUSE tr|F6XNW8|F6XNW8\_MOUSE sp|Q6ZQ18|EFR3B\_MOUSE sp|Q6ZQ18-2|EFR3B\_MOUSE tr|Q9Z116|Q9Z116\_MOUSE sp|O70251|EF1B\_MOUSE sp|Q99246|CAC1D\_MOUSE sp|Q99246-3|CAC1D\_MOUSE sp|Q99246-4|CAC1D\_MOUSE tr|Q6PF96|Q6PF96\_MOUSE sp|E9Q5F9|SETD2\_MOUSE sp|Q8CEE6|PASK\_MOUSE tr|Q3UYD7|Q3UYD7\_MOUSE sp|Q9QUS6|TRIM1\_MOUSE sp|E9Q5F9-2|SETD2\_MOUSE tr|B1AVF4|B1AVF4\_MOUSE tr|B1AVF5|B1AVF5\_MOUSE sp|Q921G7|ETFD\_MOUSE tr|F6VTG4|F6VTG4\_MOUSE tr|D3YXB7|D3YXB7\_MOUSE sp|Q9CZV8-4|FXL20\_MOUSE tr|D3YZN4|D3YZN4\_MOUSE sp|Q3ULF4|SPG7\_MOUSE tr|D3Z1Z1|D3Z1Z1\_MOUSE sp|Q9CZV8|FXL20\_MOUSE sp|Q9CZV8-3|FXL20\_MOUSE tr|Q9WU32|Q9WU32\_MOUSE tr|F6W695|F6W695\_MOUSE sp|Q3UD82|PARP8\_MOUSE tr|F8WIK2|F8WIK2\_MOUSE sp|A2ADA5|PUSL1\_MOUSE tr|Q8BK32|Q8BK32\_MOUSE tr|A2APA8|A2APA8\_MOUSE tr|B1ATB3|B1ATB3\_MOUSE tr|E0CYM8|E0CYM8\_MOUSE sp|P97797-2|SHPS1\_MOUSE sp|P26262|KLKB1\_MOUSE tr|E0CYX5|E0CYX5\_MOUSE sp|P97797|SHPS1\_MOUSE sp|Q8C0L9|GPCP1\_MOUSE tr|Q6P6I8|Q6P6I8\_MOUSE sp|Q7TN60|TMC6\_MOUSE sp|Q7TN60-3|TMC6\_MOUSE sp|Q8BMG1|ATRIP\_MOUSE tr|I6L9G2|I6L9G2\_MOUSE tr|E9Q6L5|E9Q6L5\_MOUSE sp|Q91WT9|CBS\_MOUSE sp|Q9EP97|SENP3\_MOUSE sp|Q8BKH7-2|SIN1\_MOUSE sp|Q8BKH7|SIN1\_MOUSE tr|A2AR15|A2AR15\_MOUSE sp|Q8BKH7-3|SIN1\_MOUSE tr|A2AR12|A2AR12\_MOUSE sp|Q8VDZ4-2|ZDHC5\_MOUSE sp|Q02780-2|NFIA\_MOUSE sp|Q5BKP2|UBP13\_MOUSE tr|F6ZRC5|F6ZRC5\_MOUSE tr|F6WT10|F6WT10\_MOUSE tr|J3QSN2|J3QSN2\_MOUSE tr|E9PY55|E9PY55\_MOUSE sp|Q7TSJ6|LATS2\_MOUSE tr|F8VQL9|F8VQL9\_MOUSE sp|Q9WU42|NCOR2\_MOUSE tr|G3UXG3|G3UXG3\_MOUSE tr|G3UY78|G3UY78\_MOUSE tr|E9Q701|E9Q701\_MOUSE sp|Q9WTW4|TFEC\_MOUSE tr|Q8BV14|Q8BV14\_MOUSE sp|Q920R6|VPP4\_MOUSE sp|P11088|FILA\_MOUSE sp|Q7TN60-2|TMC6\_MOUSE tr|A2AUR7|A2AUR7\_MOUSE sp|Q62172|RBP1\_MOUSE tr|Q9D031|Q9D031\_MOUSE sp|Q01730|RSU1\_MOUSE tr|B7ZWN4|B7ZWN4\_MOUSE tr|A2AGR1|A2AGR1\_MOUSE tr|Q3UN63|Q3UN63\_MOUSE sp|Q80U28-9|MADD\_MOUSE tr|D3YW10|D3YW10\_MOUSE tr|A2AGR0|A2AGR0\_MOUSE sp|Q80U28-6|MADD\_MOUSE sp|Q80U28-10|MADD\_MOUSE sp|Q80U28-15|MADD\_MOUSE sp|Q80U28|MADD\_MOUSE sp|Q91W82|UB2E2\_MOUSE tr|Q3UJB0|Q3UJB0\_MOUSE tr|A6PWP8|A6PWP8\_MOUSE sp|Q60696|PMEL\_MOUSE tr|A2AGQ3|A2AGQ3\_MOUSE sp|Q80U28-13|MADD\_MOUSE tr|D3YXD1|D3YXD1\_MOUSE tr|A2AGQ6|A2AGQ6\_MOUSE tr|Q1WLP7|Q1WLP7\_MOUSE sp|Q80U28-12|MADD\_MOUSE tr|Q9CZB2|Q9CZB2\_MOUSE tr|D3YWN5|D3YWN5\_MOUSE tr|F6XDR3|F6XDR3\_MOUSE sp|Q8C3F2|F120C\_MOUSE tr|Q7TRI8|Q7TRI8\_MOUSE sp|Q9D5U9-2|MICLK\_MOUSE sp|Q9D5U9|MICLK\_MOUSE tr|G3UZJ6|G3UZJ6\_MOUSE sp|P35980|RL18\_MOUSE tr|F8WJD6|F8WJD6\_MOUSE sp|P48967|MPIP3\_MOUSE sp|Q69Z36|MEX3B\_MOUSE sp|Q6P9K8-3|CSKI1\_MOUSE tr|Q5SVI3|Q5SVI3\_MOUSE sp|P28652|KCC2B\_MOUSE tr|Q5SVJ0|Q5SVJ0\_MOUSE sp|Q9QZB6-2|NR4A3\_MOUSE sp|Q9QZB6|NR4A3\_MOUSE tr|F6R7A2|F6R7A2\_MOUSE sp|Q5NCX5-2|NEUL4\_MOUSE sp|Q5SVR0|TBC9B\_MOUSE tr|D3YY51|D3YY51\_MOUSE tr|E9QAG2|E9QAG2\_MOUSE tr|H3BK72|H3BK72\_MOUSE sp|Q9D4J7-2|PHF6\_MOUSE tr|Q6P8Q6|Q6P8Q6\_MOUSE sp|Q5SVR0-2|TBC9B\_MOUSE sp|Q9D4J7|PHF6\_MOUSE sp|Q5NCX5|NEUL4\_MOUSE tr|H3BJZ3|H3BJZ3\_MOUSE sp|Q6P4S6-2|SIK3\_MOUSE tr|F6WKU5|F6WKU5\_MOUSE tr|F6U8X4|F6U8X4\_MOUSE sp|Q8K2V6|IPO11\_MOUSE sp|Q8C5V5|WDR27\_MOUSE sp|Q8K2V6-2|IPO11\_MOUSE sp|Q8BRG6|KLH24\_MOUSE sp|P58391|KCNN3\_MOUSE sp|Q8K1J6|TRNT1\_MOUSE tr|B7ZCF1|B7ZCF1\_MOUSE sp|Q8K1J6-2|TRNT1\_MOUSE sp|P15388|KCNC1\_MOUSE tr|D6REU3|D6REU3\_MOUSE tr|Q497J0|Q497J0\_MOUSE sp|Q812G0-2|MGT4A\_MOUSE tr|A0A087WPH7|A0A087WPH7\_MOUSE tr|L7N257|L7N257\_MOUSE tr|E9PVV3|E9PVV3\_MOUSE tr|Q7TRG0|Q7TRG0\_MOUSE tr|E9Q759|E9Q759\_MOUSE sp|Q91ZT5-2|FGD4\_MOUSE tr|F8WGX0|F8WGX0\_MOUSE tr|V9GXK0|V9GXK0\_MOUSE tr|B1AYB5|B1AYB5\_MOUSE sp|Q9QXY7|XK\_MOUSE sp|Q9WV92-8|E41L3\_MOUSE tr|F2Z3W9|F2Z3W9\_MOUSE tr|H3BKK7|H3BKK7\_MOUSE tr|E0CYX1|E0CYX1\_MOUSE sp|Q8BUR3|FOXJ3\_MOUSE sp|Q8R115|TMM82\_MOUSE sp|P43135|COT2\_MOUSE tr|Q3TAY5|Q3TAY5\_MOUSE sp|Q60632|COT1\_MOUSE tr|D3YYP4|D3YYP4\_MOUSE tr|F7CII1|F7CII1\_MOUSE tr|Q32NY6|Q32NY6\_MOUSE sp|Q8BUR3-2|FOXJ3\_MOUSE tr|E0CYS8|E0CYS8\_MOUSE tr|B8JJI9|B8JJI9\_MOUSE tr|Q5SXE4|Q5SXE4\_MOUSE sp|Q61097|KSR1\_MOUSE sp|Q61097-2|KSR1\_MOUSE sp|Q9DB73|NB5R1\_MOUSE sp|Q9DB73-2|NB5R1\_MOUSE tr|G3UZG6|G3UZG6\_MOUSE sp|Q9CZU3|SK2L2\_MOUSE sp|Q3UN70|MRFL\_MOUSE sp|Q8BIR2-2|ASTE1\_MOUSE tr|V9GXG0|V9GXG0\_MOUSE sp|Q8BIR2|ASTE1\_MOUSE sp|Q9D650|FA84A\_MOUSE sp|Q8R3P0|ACY2\_MOUSE tr|F6VSA8|F6VSA8\_MOUSE sp|Q8BI55|DYRK4\_MOUSE tr|B9EHG2|B9EHG2\_MOUSE sp|Q80U16-2|FA65B\_MOUSE tr|H3BL10|H3BL10\_MOUSE sp|Q80U16-5|FA65B\_MOUSE sp|A6X935-2|ITIH4\_MOUSE tr|E9Q5L2|E9Q5L2\_MOUSE sp|A6X935|ITIH4\_MOUSE sp|A2APT9|KLD7A\_MOUSE tr|E9PVD2|E9PVD2\_MOUSE sp|P57785|LFTY2\_MOUSE sp|Q91YI1-2|ATG13\_MOUSE sp|Q8VHD6|GTR10\_MOUSE sp|Q7TPV4|MBB1A\_MOUSE tr|D3YU22|D3YU22\_MOUSE tr|E9Q8W9|E9Q8W9\_MOUSE sp|Q6PGE7|SC6A7\_MOUSE tr|F8WGG3|F8WGG3\_MOUSE sp|Q91W36|UBP3\_MOUSE tr|B0QZL5|B0QZL5\_MOUSE sp|Q8JZZ7|LPHN2\_MOUSE sp|P63280|UBC9\_MOUSE tr|Q8C493|Q8C493\_MOUSE tr|E9PUA6|E9PUA6\_MOUSE tr|Q80X81|Q80X81\_MOUSE tr|D3YZ64|D3YZ64\_MOUSE sp|Q8CAY6|THIC\_MOUSE tr|F2Z459|F2Z459\_MOUSE sp|Q7TQF7|AMPH\_MOUSE tr|Q810S7|Q810S7\_MOUSE sp|Q8BG79|C19L2\_MOUSE sp|Q80UN1|KCTD9\_MOUSE tr|G3XA25|G3XA25\_MOUSE sp|Q8K4J2-3|COE4\_MOUSE sp|Q8BVY0|RL1D1\_MOUSE sp|Q8CBH5-2|MFSD6\_MOUSE sp|Q8BG79-2|C19L2\_MOUSE sp|Q8R4E6-2|PURG\_MOUSE tr|D3Z183|D3Z183\_MOUSE tr|Q8BQ38|Q8BQ38\_MOUSE sp|P51480|CD2A1\_MOUSE tr|E9Q9K8|E9Q9K8\_MOUSE tr|Q8BKP2|Q8BKP2\_MOUSE tr|Q8C0S0|Q8C0S0\_MOUSE sp|Q9D3J9|CX021\_MOUSE tr|A2BHR0|A2BHR0\_MOUSE sp|Q9QXJ1-2|APBB1\_MOUSE sp|Q9QXJ1|APBB1\_MOUSE tr|Q8R293|Q8R293\_MOUSE sp|Q9QUM4-2|SLAF1\_MOUSE sp|Q9QUM4|SLAF1\_MOUSE sp|Q9WUD1|CHIP\_MOUSE sp|P41136|ID2\_MOUSE tr|E9Q004|E9Q004\_MOUSE sp|Q3UJK4|GTPB2\_MOUSE tr|E9QA45|E9QA45\_MOUSE tr|Q4FE56|Q4FE56\_MOUSE sp|P70398|USP9X\_MOUSE sp|Q76LS9-2|FA63A\_MOUSE sp|Q8BM96|GP128\_MOUSE tr|Q8VFV3|Q8VFV3\_MOUSE sp|Q8CBE3|WDR37\_MOUSE sp|Q6DIA2|EX3L4\_MOUSE sp|Q8BXA1|GOLI4\_MOUSE tr|D3YVW2|D3YVW2\_MOUSE tr|B9EHL3|B9EHL3\_MOUSE sp|Q8CCE9-5|E4F1\_MOUSE sp|Q9JIG4-2|PPR3F\_MOUSE sp|Q9JIG4|PPR3F\_MOUSE tr|G8JL81|G8JL81\_MOUSE tr|S4R170|S4R170\_MOUSE tr|Q8VGS0|Q8VGS0\_MOUSE tr|Q7TR70|Q7TR70\_MOUSE tr|Q7TR67|Q7TR67\_MOUSE tr|A2BDZ3|A2BDZ3\_MOUSE tr|Q7TR47|Q7TR47\_MOUSE tr|Q7TR63|Q7TR63\_MOUSE sp|Q8C9X6-2|EPC1\_MOUSE tr|D5MNX2|D5MNX2\_MOUSE sp|Q8C9X6|EPC1\_MOUSE tr|D3YWW1|D3YWW1\_MOUSE tr|Q8VC81|Q8VC81\_MOUSE sp|Q99P27|PG12B\_MOUSE sp|Q2TV84-2|TRPM1\_MOUSE tr|E9QAE3|E9QAE3\_MOUSE sp|Q2TV84|TRPM1\_MOUSE tr|F6UMA3|F6UMA3\_MOUSE tr|B1ARR7|B1ARR7\_MOUSE sp|Q9QZ49|UBXN8\_MOUSE sp|Q9JIT0|LMBR1\_MOUSE sp|Q9JIT0-3|LMBR1\_MOUSE tr|Q3TTF2|Q3TTF2\_MOUSE tr|J3QM78|J3QM78\_MOUSE tr|Q6P3D8|Q6P3D8\_MOUSE tr|Q9D481|Q9D481\_MOUSE tr|Q3TWZ5|Q3TWZ5\_MOUSE tr|A2A7A7|A2A7A7\_MOUSE sp|Q8CFX1|G6PE\_MOUSE sp|Q9DBA8|HUTI\_MOUSE sp|Q80ZN5|CST13\_MOUSE sp|Q32M21-2|GSDA2\_MOUSE sp|Q32M21|GSDA2\_MOUSE tr|G3UYE5|G3UYE5\_MOUSE sp|Q8R4Y8|RTTN\_MOUSE sp|Q9D1H7-2|GET4\_MOUSE sp|Q640R3|HECAM\_MOUSE tr|Q9QXC6|Q9QXC6\_MOUSE tr|Q3TPU9|Q3TPU9\_MOUSE sp|P70371|TERF1\_MOUSE sp|P68181-4|KAPCB\_MOUSE sp|Q80ZU0|ARL5A\_MOUSE tr|J3QJW3|J3QJW3\_MOUSE tr|S4R169|S4R169\_MOUSE tr|K4DI76|K4DI76\_MOUSE sp|Q80TJ1-4|CAPS1\_MOUSE sp|Q80TJ1|CAPS1\_MOUSE sp|P97441|ZNT3\_MOUSE sp|P59235|NUP43\_MOUSE sp|Q91ZA3|PCCA\_MOUSE sp|P30678|GNA15\_MOUSE sp|Q8R1S9|S38A4\_MOUSE tr|A2ATP6|A2ATP6\_MOUSE sp|Q8VE19|MIO\_MOUSE sp|Q8C854-2|MYEF2\_MOUSE sp|Q8C854-1|MYEF2\_MOUSE tr|F6XJA1|F6XJA1\_MOUSE tr|A0A087WPR8|A0A087WPR8\_MOUSE sp|Q80XS6|SMAG2\_MOUSE sp|Q9WUU8-3|TNIP1\_MOUSE tr|D3Z2W0|D3Z2W0\_MOUSE sp|Q61614|EDNRA\_MOUSE tr|E9QM75|E9QM75\_MOUSE sp|Q9WVJ9|FBLN4\_MOUSE sp|Q6V3W6|CC135\_MOUSE sp|Q8BJW6-2|EIF2A\_MOUSE tr|D3YZZ6|D3YZZ6\_MOUSE sp|Q8BJW6|EIF2A\_MOUSE tr|E9Q3N9|E9Q3N9\_MOUSE tr|Q3TGG2|Q3TGG2\_MOUSE tr|G5E8D6|G5E8D6\_MOUSE tr|E9Q5Q0|E9Q5Q0\_MOUSE sp|Q7TQH0-3|ATX2L\_MOUSE sp|Q7TQH0|ATX2L\_MOUSE sp|Q9WUU8-2|TNIP1\_MOUSE sp|Q7TQH0-2|ATX2L\_MOUSE sp|Q9WUU8|TNIP1\_MOUSE sp|Q6PCX9|TRI37\_MOUSE tr|E0CYT3|E0CYT3\_MOUSE sp|Q3TLP5|ECHD2\_MOUSE sp|Q7M6Y2|SOX11\_MOUSE tr|Q8K5B9|Q8K5B9\_MOUSE tr|E0CXC8|E0CXC8\_MOUSE sp|Q9WU39|SCNNG\_MOUSE sp|Q3TLP5-2|ECHD2\_MOUSE sp|Q06831|SOX4\_MOUSE tr|Q8VET8|Q8VET8\_MOUSE sp|Q8BYI6-3|PCAT2\_MOUSE tr|Q3UWK8|Q3UWK8\_MOUSE sp|P48754|BRCA1\_MOUSE sp|Q9D0K1|PEX13\_MOUSE tr|Q3V2K7|Q3V2K7\_MOUSE tr|F6WSY2|F6WSY2\_MOUSE sp|Q923B6|STEA4\_MOUSE tr|D3Z3N9|D3Z3N9\_MOUSE sp|Q8BM65-4|NYAP2\_MOUSE sp|Q921W8|SCT1A\_MOUSE sp|Q8BM65-3|NYAP2\_MOUSE sp|Q8BM65|NYAP2\_MOUSE tr|A2ABP9|A2ABP9\_MOUSE sp|P28705|RXRG\_MOUSE tr|Q8VFT4|Q8VFT4\_MOUSE tr|E9Q9V9|E9Q9V9\_MOUSE sp|Q8BID6|ZBT46\_MOUSE tr|E9PUE2|E9PUE2\_MOUSE tr|D6RJJ7|D6RJJ7\_MOUSE tr|A2AUL9|A2AUL9\_MOUSE sp|Q9QZL6|UBP21\_MOUSE sp|Q62392|PHLA1\_MOUSE tr|F6QZ47|F6QZ47\_MOUSE tr|E9PV12|E9PV12\_MOUSE tr|G3X914|G3X914\_MOUSE sp|Q9DA97|SEP14\_MOUSE tr|S4R2P6|S4R2P6\_MOUSE tr|E9PY28|E9PY28\_MOUSE tr|E9PUS2|E9PUS2\_MOUSE tr|D6RHK5|D6RHK5\_MOUSE tr|B1AVK5|B1AVK5\_MOUSE sp|O88986|KBL\_MOUSE tr|E9PWY6|E9PWY6\_MOUSE sp|Q925I7-3|PDGFD\_MOUSE sp|P70701|WN10A\_MOUSE sp|Q9CZ00|DBND1\_MOUSE tr|G3XA29|G3XA29\_MOUSE tr|G3XA16|G3XA16\_MOUSE sp|Q9CQE1|NPS3B\_MOUSE sp|Q9JLK4|CABP2\_MOUSE tr|E0CXZ2|E0CXZ2\_MOUSE sp|Q9JLK4-2|CABP2\_MOUSE tr|G5E8W3|G5E8W3\_MOUSE tr|Q8K5C8|Q8K5C8\_MOUSE tr|E9Q9P8|E9Q9P8\_MOUSE tr|Q8C373|Q8C373\_MOUSE tr|E9QLA5|E9QLA5\_MOUSE sp|Q8C804|SPICE\_MOUSE sp|Q8BMJ2|SYLC\_MOUSE tr|Q3UGX8|Q3UGX8\_MOUSE sp|Q61324-2|ARNT2\_MOUSE tr|D3YZ02|D3YZ02\_MOUSE sp|Q8R4D1-2|SL9A8\_MOUSE tr|A2A464|A2A464\_MOUSE sp|Q8R4D1|SL9A8\_MOUSE sp|P98197|AT11A\_MOUSE sp|Q80YA8|CRUM2\_MOUSE tr|E9Q3G7|E9Q3G7\_MOUSE sp|O35239|PTN9\_MOUSE sp|O54916-3|REPS1\_MOUSE tr|F6R6F1|F6R6F1\_MOUSE tr|D3Z2E3|D3Z2E3\_MOUSE sp|Q8BVN0|CC122\_MOUSE sp|Q9QZ11|EXO1\_MOUSE tr|E9Q632|E9Q632\_MOUSE tr|E0CXC7|E0CXC7\_MOUSE sp|Q9QXT0|CNPY2\_MOUSE tr|F6V619|F6V619\_MOUSE tr|F6YDG9|F6YDG9\_MOUSE tr|F7ATU7|F7ATU7\_MOUSE tr|G3UWT0|G3UWT0\_MOUSE sp|Q5RKR3|ISLR2\_MOUSE tr|E9Q9N7|E9Q9N7\_MOUSE tr|A2AJX4|A2AJX4\_MOUSE tr|G3UYK2|G3UYK2\_MOUSE sp|Q761V0-2|SC6A5\_MOUSE sp|Q761V0|SC6A5\_MOUSE tr|B2RQX9|B2RQX9\_MOUSE sp|P51827-2|AFF3\_MOUSE sp|P51827|AFF3\_MOUSE tr|F6TU54|F6TU54\_MOUSE tr|D3YYI6|D3YYI6\_MOUSE tr|D6RHK8|D6RHK8\_MOUSE tr|D3YUR3|D3YUR3\_MOUSE tr|F8WJA7|F8WJA7\_MOUSE sp|Q5UAK0-4|MIER1\_MOUSE sp|Q5UAK0|MIER1\_MOUSE sp|Q5UAK0-1|MIER1\_MOUSE sp|P11930|NUD19\_MOUSE sp|Q5UAK0-5|MIER1\_MOUSE sp|Q5UAK0-2|MIER1\_MOUSE tr|Q3KNI6|Q3KNI6\_MOUSE sp|Q60597-3|ODO1\_MOUSE sp|Q60597|ODO1\_MOUSE tr|Z4YJV4|Z4YJV4\_MOUSE sp|Q60597-2|ODO1\_MOUSE sp|Q60597-4|ODO1\_MOUSE tr|Q6NXL1|Q6NXL1\_MOUSE sp|Q8CCX5|KT222\_MOUSE sp|Q8CCX5-2|KT222\_MOUSE sp|Q62507|COCH\_MOUSE sp|P33215-2|NEDD1\_MOUSE tr|A0A087WRT7|A0A087WRT7\_MOUSE tr|F6V339|F6V339\_MOUSE tr|F7BQ65|F7BQ65\_MOUSE tr|Q9CZ19|Q9CZ19\_MOUSE tr|D6RFU4|D6RFU4\_MOUSE sp|P09541|MYL4\_MOUSE tr|A2A6Q8|A2A6Q8\_MOUSE sp|Q9QXM0|ABHD2\_MOUSE tr|D6REI6|D6REI6\_MOUSE tr|I7HJS2|I7HJS2\_MOUSE sp|Q762D5|S35D2\_MOUSE tr|H3BJJ8|H3BJJ8\_MOUSE sp|Q762D5-2|S35D2\_MOUSE tr|B7FAU2|B7FAU2\_MOUSE tr|Q91WF0|Q91WF0\_MOUSE tr|J3JS92|J3JS92\_MOUSE sp|Q3U4H6|HEXDC\_MOUSE sp|D3YX43|VSI10\_MOUSE sp|Q3U2V3|NUD18\_MOUSE tr|E9PVW4|E9PVW4\_MOUSE sp|Q9DBM2|ECHP\_MOUSE tr|D3Z536|D3Z536\_MOUSE tr|F7CBI4|F7CBI4\_MOUSE sp|Q5NCR9|NSRP1\_MOUSE tr|Q8C1R9|Q8C1R9\_MOUSE sp|Q9D7Z3-2|NOL7\_MOUSE sp|P0CG14|CTF8A\_MOUSE tr|Q8BJT9|Q8BJT9\_MOUSE sp|Q9D7Z3|NOL7\_MOUSE sp|Q3UUG6-2|TBC24\_MOUSE sp|Q8CDM8|F16B1\_MOUSE sp|Q80VI1|TRI56\_MOUSE tr|Q8VGK3|Q8VGK3\_MOUSE sp|Q6NZL0|SOGA3\_MOUSE tr|D3Z6B9|D3Z6B9\_MOUSE tr|F6UMF8|F6UMF8\_MOUSE tr|E9QAI4|E9QAI4\_MOUSE sp|Q920B0|FRM4B\_MOUSE sp|Q9EQH7-3|NDST3\_MOUSE tr|E9QME5|E9QME5\_MOUSE tr|E9QP19|E9QP19\_MOUSE sp|Q6QR59|F115E\_MOUSE sp|Q9ER99|RSCA1\_MOUSE sp|Q8C754|VPS52\_MOUSE sp|Q99PP7-2|TRI33\_MOUSE sp|Q99PP7|TRI33\_MOUSE tr|G3UY33|G3UY33\_MOUSE sp|Q8C754-2|VPS52\_MOUSE sp|Q9JM05|PIAS4\_MOUSE tr|D6REF7|D6REF7\_MOUSE tr|E9PY31|E9PY31\_MOUSE sp|Q9Z1G4|VPP1\_MOUSE sp|Q9Z1G4-2|VPP1\_MOUSE sp|Q9WV92-3|E41L3\_MOUSE sp|Q9WV92-6|E41L3\_MOUSE sp|Q9WV92-5|E41L3\_MOUSE sp|Q9WV92|E41L3\_MOUSE sp|Q9WV92-7|E41L3\_MOUSE sp|Q9Z1G4-3|VPP1\_MOUSE tr|K3W4T3|K3W4T3\_MOUSE sp|Q9WV92-4|E41L3\_MOUSE sp|Q9WV92-2|E41L3\_MOUSE sp|Q80XI3-4|IF4G3\_MOUSE sp|Q80XI3-2|IF4G3\_MOUSE sp|Q80XI3-3|IF4G3\_MOUSE sp|Q80XI3|IF4G3\_MOUSE sp|Q80WQ2|VAC14\_MOUSE sp|Q6P205|XL3B\_MOUSE sp|Q61806|XL3C\_MOUSE sp|Q8K201|KCT2\_MOUSE sp|P62046|LRCH1\_MOUSE tr|B1B0S0|B1B0S0\_MOUSE sp|Q6X6Z7|TEKT3\_MOUSE tr|A2AW41|A2AW41\_MOUSE tr|Q8VHM5|Q8VHM5\_MOUSE sp|P39038|CADH4\_MOUSE tr|B0V2N5|B0V2N5\_MOUSE sp|Q9JHI4-3|S13A1\_MOUSE sp|Q9ES57|MO2R1\_MOUSE tr|A0A087WR66|A0A087WR66\_MOUSE sp|Q8BTP3|MO2R5\_MOUSE sp|Q6XJV4|MO2R4\_MOUSE sp|Q9JHI4|S13A1\_MOUSE tr|E9PZ81|E9PZ81\_MOUSE tr|M0QWY6|M0QWY6\_MOUSE tr|H3BJX3|H3BJX3\_MOUSE tr|D6RGA8|D6RGA8\_MOUSE sp|Q9D413-2|SH2D6\_MOUSE sp|Q9D413|SH2D6\_MOUSE tr|F6VYQ6|F6VYQ6\_MOUSE sp|Q6P4U0|THS7B\_MOUSE tr|D3Z007|D3Z007\_MOUSE sp|Q62521|ZIC3\_MOUSE sp|Q62521-2|ZIC3\_MOUSE sp|Q9JKN6|NOVA1\_MOUSE tr|B1AS06|B1AS06\_MOUSE sp|Q6PFD5|DLGP3\_MOUSE sp|Q8BVQ5|PPME1\_MOUSE sp|O35126|ATN1\_MOUSE sp|P35505|FAAA\_MOUSE sp|P41593|PTH1R\_MOUSE sp|Q99KU6-2|CF089\_MOUSE sp|Q5I043|UBP28\_MOUSE sp|Q5I043-2|UBP28\_MOUSE tr|F2Z3Y2|F2Z3Y2\_MOUSE sp|P58544|BTBD1\_MOUSE sp|Q91XE0|GLYAT\_MOUSE sp|Q8K031|STAR8\_MOUSE tr|D3YWF6|D3YWF6\_MOUSE sp|Q7TQI3|OTUB1\_MOUSE tr|F6TK47|F6TK47\_MOUSE tr|A2A4I9|A2A4I9\_MOUSE sp|Q812C9|AOC2\_MOUSE sp|Q8VEH8|ERLEC\_MOUSE sp|Q6DID3|SCAF8\_MOUSE sp|A2A8L5|PTPRF\_MOUSE sp|Q9D495|SYCE1\_MOUSE sp|Q6P1D7|SLX4\_MOUSE sp|P70275|SEM3E\_MOUSE sp|Q9D0W5|PPIL1\_MOUSE sp|O35144-2|TERF2\_MOUSE sp|Q810B9|SLIK3\_MOUSE tr|Q8VG16|Q8VG16\_MOUSE sp|B2RXC1|TPC11\_MOUSE sp|Q14AT2-2|TEX11\_MOUSE sp|Q9D5V6|SYAP1\_MOUSE sp|Q9QYF9|NDRG3\_MOUSE tr|Q8VCV2|Q8VCV2\_MOUSE sp|Q91VN1|ZNF24\_MOUSE tr|E9Q6W8|E9Q6W8\_MOUSE tr|E9QAI0|E9QAI0\_MOUSE sp|Q7TQE7|K0895\_MOUSE tr|E9QA71|E9QA71\_MOUSE sp|A0JLY1|CC173\_MOUSE tr|Q8VGN8|Q8VGN8\_MOUSE tr|Q8VFX7|Q8VFX7\_MOUSE sp|Q9EQW7|KI13A\_MOUSE tr|F8VQ75|F8VQ75\_MOUSE tr|Q8VGE5|Q8VGE5\_MOUSE tr|D3YWH1|D3YWH1\_MOUSE tr|A0A087WS74|A0A087WS74\_MOUSE tr|A0A087WRP4|A0A087WRP4\_MOUSE tr|H3BJV1|H3BJV1\_MOUSE sp|P70452|STX4\_MOUSE tr|A0A087WQX8|A0A087WQX8\_MOUSE tr|A2ASU6|A2ASU6\_MOUSE tr|A0A087WSC6|A0A087WSC6\_MOUSE tr|A0A087WNY6|A0A087WNY6\_MOUSE sp|Q9DBQ9|SWT1\_MOUSE sp|Q8C863-2|ITCH\_MOUSE sp|Q9DBQ9-3|SWT1\_MOUSE tr|F7B8F0|F7B8F0\_MOUSE sp|Q8C863|ITCH\_MOUSE sp|Q99PU7|BAP1\_MOUSE sp|Q9DAQ4-2|CB081\_MOUSE tr|G3UXK2|G3UXK2\_MOUSE tr|E0CZH2|E0CZH2\_MOUSE tr|B2RT14|B2RT14\_MOUSE sp|O35344|IMA4\_MOUSE tr|D3Z5I9|D3Z5I9\_MOUSE sp|Q9CXK9|RBM33\_MOUSE sp|Q91ZZ3|SYUB\_MOUSE tr|D6RGB6|D6RGB6\_MOUSE sp|Q6PGG2|GMIP\_MOUSE sp|Q91Z96-2|BMP2K\_MOUSE sp|Q6PGG2-2|GMIP\_MOUSE tr|F7BJW2|F7BJW2\_MOUSE sp|P11438|LAMP1\_MOUSE tr|D3YU97|D3YU97\_MOUSE sp|O09061|PSB1\_MOUSE tr|Q8VFU2|Q8VFU2\_MOUSE sp|Q2LA85|TRML2\_MOUSE tr|E9PUT0|E9PUT0\_MOUSE tr|Q7TRX4|Q7TRX4\_MOUSE tr|E9QNT8|E9QNT8\_MOUSE sp|Q02357-2|ANK1\_MOUSE sp|Q02357|ANK1\_MOUSE sp|Q02357-4|ANK1\_MOUSE tr|B7ZW98|B7ZW98\_MOUSE tr|D3Z5M4|D3Z5M4\_MOUSE tr|G8JL84|G8JL84\_MOUSE sp|Q99N95|RM03\_MOUSE tr|Q0VGY9|Q0VGY9\_MOUSE tr|G5E8J2|G5E8J2\_MOUSE tr|D3YTV8|D3YTV8\_MOUSE tr|D3Z456|D3Z456\_MOUSE sp|Q02357-5|ANK1\_MOUSE sp|Q9WVC6-2|SGK1\_MOUSE sp|Q02357-3|ANK1\_MOUSE sp|Q02357-6|ANK1\_MOUSE sp|Q9D4H2|GCC1\_MOUSE sp|Q61152|PTN18\_MOUSE tr|A0A087WPR5|A0A087WPR5\_MOUSE tr|A2ALV3|A2ALV3\_MOUSE sp|Q5RI75|RASEF\_MOUSE sp|Q5RI75-2|RASEF\_MOUSE sp|Q91XQ5|CHSTF\_MOUSE tr|B1AS48|B1AS48\_MOUSE sp|Q4FZD7|PLK5\_MOUSE sp|Q2EG98-7|PK1L3\_MOUSE tr|G3X967|G3X967\_MOUSE sp|Q2EG98-5|PK1L3\_MOUSE sp|P48302|EDNRB\_MOUSE sp|P31324|KAP3\_MOUSE sp|P41163|ETV2\_MOUSE tr|B2RXC6|B2RXC6\_MOUSE tr|G3UXR2|G3UXR2\_MOUSE sp|P97321|SEPR\_MOUSE sp|Q8BLK9-3|KS6C1\_MOUSE tr|H3BJ67|H3BJ67\_MOUSE tr|H3BL92|H3BL92\_MOUSE tr|H3BKQ3|H3BKQ3\_MOUSE tr|H3BJ57|H3BJ57\_MOUSE sp|Q9DBL1|ACDSB\_MOUSE tr|E9Q5L3|E9Q5L3\_MOUSE tr|H3BJX1|H3BJX1\_MOUSE sp|Q6KAU7-3|PKHG2\_MOUSE tr|E9Q499|E9Q499\_MOUSE tr|D3Z5N8|D3Z5N8\_MOUSE sp|Q6KAU7|PKHG2\_MOUSE tr|G5E8T4|G5E8T4\_MOUSE tr|G3UYW9|G3UYW9\_MOUSE sp|P39053-2|DYN1\_MOUSE tr|Z4YKA3|Z4YKA3\_MOUSE sp|P39053-4|DYN1\_MOUSE tr|L7N210|L7N210\_MOUSE sp|P39053|DYN1\_MOUSE sp|Q3TEA8|HP1B3\_MOUSE tr|Z4YKB8|Z4YKB8\_MOUSE sp|Q3TEA8-2|HP1B3\_MOUSE sp|P39053-5|DYN1\_MOUSE sp|P39053-6|DYN1\_MOUSE sp|Q3TEA8-3|HP1B3\_MOUSE tr|D6RHM0|D6RHM0\_MOUSE sp|Q9R0A0|PEX14\_MOUSE sp|Q66JZ4|TCAIM\_MOUSE tr|G3X983|G3X983\_MOUSE sp|P35639|DDIT3\_MOUSE tr|A2AMI7|A2AMI7\_MOUSE sp|Q7TSV9|ENKD1\_MOUSE tr|D6RH82|D6RH82\_MOUSE tr|Q91YS4|Q91YS4\_MOUSE sp|Q6P6L0|FIL1L\_MOUSE sp|G5E8K5-6|ANK3\_MOUSE tr|Q9EPG6|Q9EPG6\_MOUSE sp|Q8BMD7-2|MORC4\_MOUSE tr|Q5NBZ7|Q5NBZ7\_MOUSE tr|D3YXZ3|D3YXZ3\_MOUSE sp|Q8BMD7|MORC4\_MOUSE sp|O88448|KLC2\_MOUSE tr|E0CYH7|E0CYH7\_MOUSE tr|E9Q725|E9Q725\_MOUSE sp|Q60889|OLF5\_MOUSE tr|E9QQ35|E9QQ35\_MOUSE sp|O70167|P3C2G\_MOUSE sp|Q9QUQ5-2|TRPC4\_MOUSE sp|Q9QUQ5|TRPC4\_MOUSE tr|E9Q1S6|E9Q1S6\_MOUSE tr|B1ARC0|B1ARC0\_MOUSE tr|F6T4M4|F6T4M4\_MOUSE tr|B1ARB9|B1ARB9\_MOUSE sp|Q9R123|NAT6\_MOUSE tr|E9QNE9|E9QNE9\_MOUSE sp|Q6NZN1-3|PPRC1\_MOUSE sp|Q99JP0|M4K3\_MOUSE tr|B7ZCE0|B7ZCE0\_MOUSE sp|O35711-2|LIPB2\_MOUSE sp|Q9QX11-2|CYH1\_MOUSE sp|O35711-4|LIPB2\_MOUSE tr|F7DD52|F7DD52\_MOUSE sp|Q9QX11|CYH1\_MOUSE tr|Q3TZ02|Q3TZ02\_MOUSE sp|Q920A9-2|FCRLA\_MOUSE sp|Q9R1Q9|VAS1\_MOUSE sp|Q920A9|FCRLA\_MOUSE tr|Q3TKX1|Q3TKX1\_MOUSE tr|A2AF28|A2AF28\_MOUSE sp|Q91W43|GCSP\_MOUSE tr|E9PZF7|E9PZF7\_MOUSE sp|Q9Z321|TOP3B\_MOUSE tr|F6SDD7|F6SDD7\_MOUSE tr|Q8VEZ7|Q8VEZ7\_MOUSE sp|Q91YM2|RHG35\_MOUSE tr|Q9D5S6|Q9D5S6\_MOUSE tr|E0CYW4|E0CYW4\_MOUSE sp|P70224|GIMA1\_MOUSE tr|Q3TFI6|Q3TFI6\_MOUSE sp|Q5SSL4|ABR\_MOUSE sp|Q5SSL4-2|ABR\_MOUSE tr|E9PUE7|E9PUE7\_MOUSE tr|D3YYN7|D3YYN7\_MOUSE sp|Q8BMJ8|SP8\_MOUSE sp|Q8VEF1-2|GRM1A\_MOUSE sp|Q6PIE5|AT1A2\_MOUSE sp|Q9D2P8|MOBP\_MOUSE sp|Q3UW53|NIBAN\_MOUSE sp|Q6RUT8|CC154\_MOUSE sp|Q80ZQ3|CH074\_MOUSE tr|Q9D1F9|Q9D1F9\_MOUSE sp|Q8K4E0-2|ALMS1\_MOUSE tr|Q7TR24|Q7TR24\_MOUSE tr|F7DEJ0|F7DEJ0\_MOUSE sp|Q9D4V4-2|TAF1D\_MOUSE tr|Q8VGE8|Q8VGE8\_MOUSE sp|P59913|PCMD1\_MOUSE sp|Q9D4V4|TAF1D\_MOUSE tr|S4R1L6|S4R1L6\_MOUSE sp|Q9D4V4-3|TAF1D\_MOUSE sp|Q80UK7|SAS6\_MOUSE tr|F6TBA6|F6TBA6\_MOUSE tr|A0A087WQR0|A0A087WQR0\_MOUSE tr|A0A087WP77|A0A087WP77\_MOUSE tr|A0A087WR47|A0A087WR47\_MOUSE sp|A2RSY6-2|TRM1L\_MOUSE tr|E9QAX5|E9QAX5\_MOUSE sp|A2RSY6|TRM1L\_MOUSE tr|E0CXD4|E0CXD4\_MOUSE tr|V9GWV3|V9GWV3\_MOUSE sp|Q91WN4|KMO\_MOUSE sp|Q91WN4-2|KMO\_MOUSE sp|Q8VFD0|OL486\_MOUSE sp|Q9DBF7|CWC25\_MOUSE tr|Q7TRU9|Q7TRU9\_MOUSE sp|Q05A80|CAPR2\_MOUSE sp|Q9JKB3|YBOX3\_MOUSE sp|Q9DBF7-2|CWC25\_MOUSE sp|Q05A80-2|CAPR2\_MOUSE sp|Q9JKB3-2|YBOX3\_MOUSE sp|P03888|NU1M\_MOUSE sp|P15501|SPBP\_MOUSE tr|S4R244|S4R244\_MOUSE tr|E9Q6X8|E9Q6X8\_MOUSE tr|E0CYL2|E0CYL2\_MOUSE sp|Q9EQ16|CXCR6\_MOUSE tr|I1E4X1|I1E4X1\_MOUSE sp|Q61979|NNAT\_MOUSE sp|P26618|PGFRA\_MOUSE sp|Q8CIW6-3|S26A6\_MOUSE tr|B1AVY2|B1AVY2\_MOUSE sp|Q8CIW6-2|S26A6\_MOUSE sp|P26049|GBRA3\_MOUSE sp|Q8CIW6|S26A6\_MOUSE tr|G8JL74|G8JL74\_MOUSE sp|Q3UZ45|MCIN\_MOUSE sp|Q9WVE8|PACN2\_MOUSE sp|O35134|RPA1\_MOUSE tr|G3UXA6|G3UXA6\_MOUSE tr|B1AT82|B1AT82\_MOUSE sp|Q9D3R6|KATL2\_MOUSE sp|Q9D3R6-3|KATL2\_MOUSE sp|Q9D0M1|KPRA\_MOUSE tr|D3Z4J2|D3Z4J2\_MOUSE sp|Q9WV56|GDF2\_MOUSE sp|Q7TPD2|F185A\_MOUSE sp|Q80UJ7|RB3GP\_MOUSE tr|G3X999|G3X999\_MOUSE sp|Q99MV5-3|M10L1\_MOUSE sp|Q8BH53-3|CG063\_MOUSE tr|Q9D3I0|Q9D3I0\_MOUSE tr|G3UYS5|G3UYS5\_MOUSE sp|Q8BH53-4|CG063\_MOUSE sp|Q8BH53|CG063\_MOUSE sp|Q9WV69|DEMA\_MOUSE sp|P62881|GBB5\_MOUSE sp|P62881-2|GBB5\_MOUSE tr|Q32NY8|Q32NY8\_MOUSE sp|Q8K1R3|PNPT1\_MOUSE sp|Q8K1R3-2|PNPT1\_MOUSE sp|Q9WV69-3|DEMA\_MOUSE sp|O70571|PDK4\_MOUSE sp|Q1H9T6-2|ZBT48\_MOUSE sp|Q9ET37|SC5A4\_MOUSE sp|Q60736|ZP3R\_MOUSE tr|Q8BRN2|Q8BRN2\_MOUSE sp|Q5BKS4|ERI2\_MOUSE sp|Q8BFR1-2|ZCCHL\_MOUSE tr|Q0VGU5|Q0VGU5\_MOUSE sp|Q3UIW5-2|RNF10\_MOUSE sp|A2AP18-5|PLCH2\_MOUSE tr|F7BE84|F7BE84\_MOUSE tr|A2A7A1|A2A7A1\_MOUSE sp|Q8K3F6|KCNQ3\_MOUSE sp|Q3TTP0|SHP1L\_MOUSE sp|A2AAJ9-3|OBSCN\_MOUSE sp|O35723|DNJB3\_MOUSE tr|E9Q0N2|E9Q0N2\_MOUSE tr|Q5RL26|Q5RL26\_MOUSE tr|G3XA59|G3XA59\_MOUSE tr|Q9DC47|Q9DC47\_MOUSE tr|A0A087WSP1|A0A087WSP1\_MOUSE tr|E9PUK8|E9PUK8\_MOUSE sp|O35495-3|CDK14\_MOUSE sp|Q9D995|CNTD1\_MOUSE sp|Q7TT28|REXO1\_MOUSE sp|O35495|CDK14\_MOUSE sp|Q91ZQ1|PDE6C\_MOUSE sp|Q8BFZ3|ACTBL\_MOUSE sp|Q91ZQ1-2|PDE6C\_MOUSE sp|Q7TMY4|THOC7\_MOUSE tr|Q3UQ19|Q3UQ19\_MOUSE sp|Q7TMY4-2|THOC7\_MOUSE sp|P70310|NTR2\_MOUSE tr|E9Q8I7|E9Q8I7\_MOUSE sp|Q810U5-2|CCD50\_MOUSE sp|Q810U5|CCD50\_MOUSE tr|E9PZN0|E9PZN0\_MOUSE tr|F6UK66|F6UK66\_MOUSE sp|Q810U5-3|CCD50\_MOUSE tr|E9Q3K1|E9Q3K1\_MOUSE tr|D3Z1X9|D3Z1X9\_MOUSE sp|P24270|CATA\_MOUSE sp|Q8BYR5-5|CAPS2\_MOUSE sp|Q5Y5T1|ZDH20\_MOUSE tr|E0CX56|E0CX56\_MOUSE sp|Q5Y5T1-2|ZDH20\_MOUSE sp|Q7TNR9|ARHG4\_MOUSE sp|Q69ZN7-2|MYOF\_MOUSE tr|E9PX19|E9PX19\_MOUSE sp|Q8VCB3|GYS2\_MOUSE tr|B7ZC71|B7ZC71\_MOUSE sp|Q3TLR7|DTL\_MOUSE tr|B0R093|B0R093\_MOUSE sp|P56399|UBP5\_MOUSE tr|E9Q025|E9Q025\_MOUSE tr|Q3U4W8|Q3U4W8\_MOUSE sp|Q3TLR7-2|DTL\_MOUSE tr|G3X931|G3X931\_MOUSE sp|Q9EPN1-2|NBEA\_MOUSE tr|J3KMM3|J3KMM3\_MOUSE sp|Q99P25|TXIP1\_MOUSE tr|G3UXR0|G3UXR0\_MOUSE tr|B1ATA9|B1ATA9\_MOUSE tr|Q9EQ41|Q9EQ41\_MOUSE sp|Q8CH18|CCAR1\_MOUSE sp|Q8BYN5|FSD1L\_MOUSE tr|F8WGT4|F8WGT4\_MOUSE sp|Q8CH18-3|CCAR1\_MOUSE tr|E0CXZ1|E0CXZ1\_MOUSE sp|Q5U901|CBPA6\_MOUSE tr|E9Q2R3|E9Q2R3\_MOUSE tr|Q8R4Y1|Q8R4Y1\_MOUSE tr|D3Z6M5|D3Z6M5\_MOUSE tr|J3QM95|J3QM95\_MOUSE sp|Q0VBM2|FA83B\_MOUSE sp|Q8BYN5-2|FSD1L\_MOUSE tr|Q9EQN9|Q9EQN9\_MOUSE sp|O88623-2|UBP2\_MOUSE sp|Q8BRM6-2|MEI4L\_MOUSE tr|A0A087WP18|A0A087WP18\_MOUSE sp|Q99MV7-3|RNF17\_MOUSE sp|O88623-4|UBP2\_MOUSE sp|Q8BRM6|MEI4L\_MOUSE sp|Q9R1T2|SAE1\_MOUSE sp|Q9R1T2-2|SAE1\_MOUSE sp|Q9Z0L0|TPBG\_MOUSE sp|O35465-2|FKBP8\_MOUSE sp|O35465|FKBP8\_MOUSE tr|O54909|O54909\_MOUSE sp|Q9D4V6|TO20L\_MOUSE sp|P58022-2|LOXL2\_MOUSE sp|Q80ZD8-2|AMGO1\_MOUSE sp|Q8VBV3|EXOS2\_MOUSE sp|P58022|LOXL2\_MOUSE tr|D3Z124|D3Z124\_MOUSE sp|Q61092|LAMC2\_MOUSE sp|P28352|APEX1\_MOUSE tr|F6QA74|F6QA74\_MOUSE P15636 sp|O09100|GATA2\_MOUSE sp|Q08091|CNN1\_MOUSE sp|Q08091-2|CNN1\_MOUSE sp|O54983|CRYM\_MOUSE sp|Q9QVP9|FAK2\_MOUSE tr|Q3TKD0|Q3TKD0\_MOUSE tr|Q3UDE9|Q3UDE9\_MOUSE tr|E9Q2A6|E9Q2A6\_MOUSE tr|E9Q529|E9Q529\_MOUSE tr|H3BLC5|H3BLC5\_MOUSE sp|Q8BFY9-2|TNPO1\_MOUSE sp|Q8BFY9|TNPO1\_MOUSE tr|H3BKE6|H3BKE6\_MOUSE tr|F6TQS9|F6TQS9\_MOUSE tr|Q8BLU0|Q8BLU0\_MOUSE tr|V9GXT1|V9GXT1\_MOUSE tr|F6TQN1|F6TQN1\_MOUSE sp|B2RXC5|ZN382\_MOUSE tr|Q7TRA0|Q7TRA0\_MOUSE P20930 tr|S4R1V5|S4R1V5\_MOUSE sp|Q61003-2|CD6\_MOUSE sp|Q7TQI8|TSYL2\_MOUSE sp|Q61003|CD6\_MOUSE tr|S4R184|S4R184\_MOUSE sp|Q61003-3|CD6\_MOUSE sp|P19221|THRB\_MOUSE tr|A0A075B5U6|A0A075B5U6\_MOUSE sp|Q6IEE6|T132E\_MOUSE tr|B2RXM5|B2RXM5\_MOUSE tr|H3BK79|H3BK79\_MOUSE sp|Q02819|NUCB1\_MOUSE sp|Q52KR3-2|PRUN2\_MOUSE tr|H3BJ74|H3BJ74\_MOUSE tr|Q3TTI9|Q3TTI9\_MOUSE tr|Q8VBU5|Q8VBU5\_MOUSE tr|B0V3P4|B0V3P4\_MOUSE tr|Q6IQY6|Q6IQY6\_MOUSE tr|B1AWD0|B1AWD0\_MOUSE tr|B0V3P3|B0V3P3\_MOUSE sp|Q9WV76|AP4B1\_MOUSE tr|F6U8U1|F6U8U1\_MOUSE tr|B1AWC8|B1AWC8\_MOUSE tr|G5E884|G5E884\_MOUSE tr|Q8BYG7|Q8BYG7\_MOUSE tr|E0CYF2|E0CYF2\_MOUSE tr|E9PWE0|E9PWE0\_MOUSE sp|Q99J87|DHX58\_MOUSE sp|P23506|PIMT\_MOUSE tr|E0CYV0|E0CYV0\_MOUSE tr|F7D432|F7D432\_MOUSE sp|P23506-2|PIMT\_MOUSE tr|Q66JV6|Q66JV6\_MOUSE sp|Q9D6Z0|ALKB7\_MOUSE sp|Q9D6Z0-2|ALKB7\_MOUSE sp|Q80V31|CE104\_MOUSE tr|V9GXW2|V9GXW2\_MOUSE tr|Q7TSQ9|Q7TSQ9\_MOUSE tr|Q3TQX1|Q3TQX1\_MOUSE tr|D3YZQ3|D3YZQ3\_MOUSE tr|D3YUS6|D3YUS6\_MOUSE sp|Q8K2A7|INT10\_MOUSE tr|Q9CZI4|Q9CZI4\_MOUSE sp|Q9D8U7|DTWD1\_MOUSE sp|Q7TS72|IP3KC\_MOUSE sp|E9Q5G3|KIF23\_MOUSE sp|P28700|RXRA\_MOUSE sp|Q8C145|S39A6\_MOUSE tr|D3Z7N4|D3Z7N4\_MOUSE tr|F8VQ79|F8VQ79\_MOUSE sp|Q8C5S7|REC8\_MOUSE tr|A0A087WSU6|A0A087WSU6\_MOUSE sp|Q9JMI7|TX101\_MOUSE tr|F6TAR7|F6TAR7\_MOUSE sp|Q6PCL9|PAPOG\_MOUSE tr|Q9WUD0|Q9WUD0\_MOUSE tr|S4R1Q3|S4R1Q3\_MOUSE sp|Q8CIL4|CA131\_MOUSE tr|G3X8Q9|G3X8Q9\_MOUSE tr|Q8BY05|Q8BY05\_MOUSE tr|D3YTP3|D3YTP3\_MOUSE tr|E9Q1A5|E9Q1A5\_MOUSE tr|E9QAR4|E9QAR4\_MOUSE tr|E9QAR6|E9QAR6\_MOUSE sp|P70259|GP143\_MOUSE tr|E9QAR1|E9QAR1\_MOUSE tr|D3YX49|D3YX49\_MOUSE tr|W4VSP2|W4VSP2\_MOUSE sp|Q61321-2|SIX4\_MOUSE tr|Q8R2A5|Q8R2A5\_MOUSE tr|B2RU69|B2RU69\_MOUSE sp|Q61321|SIX4\_MOUSE sp|P70412|CUZD1\_MOUSE tr|H3BL91|H3BL91\_MOUSE tr|Q6NV66|Q6NV66\_MOUSE sp|Q8CF66|LTOR4\_MOUSE tr|A0A087WP13|A0A087WP13\_MOUSE tr|O08797|O08797\_MOUSE sp|Q64285|CEL\_MOUSE sp|Q6PCQ0-3|IQCE\_MOUSE sp|P34152|FAK1\_MOUSE sp|P34152-3|FAK1\_MOUSE sp|P34152-2|FAK1\_MOUSE sp|Q8BNY6|NCS1\_MOUSE tr|D3Z3K9|D3Z3K9\_MOUSE sp|Q9QYM8|CENPH\_MOUSE tr|D3YYQ9|D3YYQ9\_MOUSE tr|D3Z344|D3Z344\_MOUSE tr|Q91VM8|Q91VM8\_MOUSE tr|A2AQJ8|A2AQJ8\_MOUSE sp|Q8BVW0|GANC\_MOUSE tr|E9Q7L1|E9Q7L1\_MOUSE tr|Q8C725|Q8C725\_MOUSE tr|Q9CQ73|Q9CQ73\_MOUSE sp|Q9JIX9|FASTK\_MOUSE sp|Q8QZY2-2|GLCTK\_MOUSE tr|E9QA06|E9QA06\_MOUSE tr|D3Z4M5|D3Z4M5\_MOUSE sp|Q9JJA2|COG8\_MOUSE sp|Q3UUF8|AN34B\_MOUSE sp|P24860|CCNB1\_MOUSE tr|B8JK65|B8JK65\_MOUSE tr|G3UY65|G3UY65\_MOUSE ENSEMBL:ENSBTAP00000006074 tr|Q68EM3|Q68EM3\_MOUSE tr|B7ZD29|B7ZD29\_MOUSE tr|F2Z3X3|F2Z3X3\_MOUSE sp|Q9QXW9|LAT2\_MOUSE sp|Q6GYP7-3|RGPA1\_MOUSE sp|Q6GYP7-5|RGPA1\_MOUSE sp|Q711T7|NADE\_MOUSE tr|F6TQW2|F6TQW2\_MOUSE sp|Q91VA0|ACSM1\_MOUSE tr|D3Z106|D3Z106\_MOUSE sp|Q91VA0-2|ACSM1\_MOUSE tr|E9Q396|E9Q396\_MOUSE tr|G3V023|G3V023\_MOUSE sp|O35181|NRG3\_MOUSE sp|Q9Z2D6|MECP2\_MOUSE sp|Q9Z2D6-2|MECP2\_MOUSE tr|E9PX53|E9PX53\_MOUSE tr|S4R1J9|S4R1J9\_MOUSE sp|A3KGV1-4|ODFP2\_MOUSE sp|A3KGV1-3|ODFP2\_MOUSE tr|E9QPR5|E9QPR5\_MOUSE sp|Q9D0I8|MRT4\_MOUSE sp|Q8K2V1|PP4R1\_MOUSE sp|A3KGV1-2|ODFP2\_MOUSE sp|P18911-2|RARG\_MOUSE sp|O54734|OST48\_MOUSE sp|A3KGV1|ODFP2\_MOUSE tr|E9Q7T7|E9Q7T7\_MOUSE sp|Q8K2V1-2|PP4R1\_MOUSE sp|O35226-3|PSMD4\_MOUSE sp|O35226-2|PSMD4\_MOUSE sp|O35226|PSMD4\_MOUSE sp|P59383|LRRN4\_MOUSE sp|Q9D9R0|RN125\_MOUSE sp|O55225-2|OTOG\_MOUSE sp|Q8K071|TM221\_MOUSE sp|Q9Z248-2|AEBP2\_MOUSE sp|Q9QY93|DCTP1\_MOUSE sp|Q9Z248|AEBP2\_MOUSE sp|Q3UUV5|SKAP1\_MOUSE sp|Q3UUV5-2|SKAP1\_MOUSE sp|Q8C0V9-2|FRMD6\_MOUSE Q5D862 sp|Q3UUV5-4|SKAP1\_MOUSE sp|Q8C0V9|FRMD6\_MOUSE sp|Q3UUV5-3|SKAP1\_MOUSE tr|E9Q613|E9Q613\_MOUSE sp|Q8BPP1|MB212\_MOUSE sp|Q9D5V2|KLH10\_MOUSE sp|Q9WTM3|SEM6C\_MOUSE sp|Q923A2|SPDLY\_MOUSE sp|Q8BVP1|TRIML\_MOUSE tr|E9Q7P6|E9Q7P6\_MOUSE sp|Q05860|FMN1\_MOUSE sp|Q05860-3|FMN1\_MOUSE sp|Q05860-2|FMN1\_MOUSE sp|Q8CHW1|THAP1\_MOUSE sp|Q8R183|TIMD2\_MOUSE sp|P97318-2|DAB1\_MOUSE tr|G5E8Q7|G5E8Q7\_MOUSE sp|P97318|DAB1\_MOUSE tr|A8C1R4|A8C1R4\_MOUSE sp|A6H687|SAC31\_MOUSE sp|P97318-8|DAB1\_MOUSE tr|B9EI20|B9EI20\_MOUSE tr|E9QL24|E9QL24\_MOUSE sp|Q8CIZ9-3|NOX1\_MOUSE sp|O08584|KLF6\_MOUSE sp|Q8CIZ9-2|NOX1\_MOUSE sp|A3KFU9-3|PTHD2\_MOUSE tr|G3X8S2|G3X8S2\_MOUSE tr|Q8BPQ2|Q8BPQ2\_MOUSE tr|E9Q6Y6|E9Q6Y6\_MOUSE sp|A3KFU9|PTHD2\_MOUSE sp|A3KFU9-2|PTHD2\_MOUSE tr|E0CYB0|E0CYB0\_MOUSE sp|Q8CIZ9|NOX1\_MOUSE sp|P97435|ENTK\_MOUSE tr|H3BLA4|H3BLA4\_MOUSE tr|D3Z511|D3Z511\_MOUSE sp|Q91WM6|EVA1A\_MOUSE sp|Q9Z1P5-2|CD320\_MOUSE tr|F6XXU0|F6XXU0\_MOUSE sp|Q8VHB5-2|CAH9\_MOUSE sp|Q8CIQ7|DOCK3\_MOUSE sp|Q3TCT4|ENTP7\_MOUSE sp|Q61923|KCNA6\_MOUSE sp|Q3TCT4-2|ENTP7\_MOUSE tr|F8VPQ1|F8VPQ1\_MOUSE tr|A0A087WNU6|A0A087WNU6\_MOUSE tr|A0A087WPK3|A0A087WPK3\_MOUSE sp|Q400C8|AMZ2\_MOUSE tr|E9Q9T1|E9Q9T1\_MOUSE tr|B1AT60|B1AT60\_MOUSE Q28085 sp|Q3UZ39-2|LRRF1\_MOUSE sp|Q400C8-2|AMZ2\_MOUSE tr|B1AT59|B1AT59\_MOUSE tr|G5E8E1|G5E8E1\_MOUSE tr|D3YZB2|D3YZB2\_MOUSE sp|Q9D2G5-2|SYNJ2\_MOUSE sp|Q8K2J0|PLCD3\_MOUSE tr|S4R2D5|S4R2D5\_MOUSE sp|Q3UEN2|FA35A\_MOUSE sp|Q9D906|ATG7\_MOUSE tr|V9GX38|V9GX38\_MOUSE sp|Q8R242|DIAC\_MOUSE sp|Q61847|MEP1B\_MOUSE tr|D3Z0R4|D3Z0R4\_MOUSE tr|D3Z0R3|D3Z0R3\_MOUSE sp|Q61847-2|MEP1B\_MOUSE sp|P61021|RAB5B\_MOUSE sp|Q8BGS1|E41L5\_MOUSE sp|Q8BGS1-3|E41L5\_MOUSE tr|D6RFR7|D6RFR7\_MOUSE tr|B0R071|B0R071\_MOUSE sp|Q8C5T8|CC113\_MOUSE sp|P31245|HXA2\_MOUSE tr|D3Z340|D3Z340\_MOUSE tr|H3BL43|H3BL43\_MOUSE sp|P58058|NADK\_MOUSE tr|B0R070|B0R070\_MOUSE sp|Q9JLT2|TREA\_MOUSE tr|F6XSD0|F6XSD0\_MOUSE tr|W4VSP9|W4VSP9\_MOUSE sp|Q9QXK2|RAD18\_MOUSE tr|Q6LC96|Q6LC96\_MOUSE tr|D3Z734|D3Z734\_MOUSE sp|Q7M6Y3|PICAL\_MOUSE sp|Q7M6Y3-5|PICAL\_MOUSE tr|B2RVE2|B2RVE2\_MOUSE sp|P70388-3|RAD50\_MOUSE sp|Q7TSY6|CELF4\_MOUSE sp|P32300|OPRD\_MOUSE tr|E9PYP7|E9PYP7\_MOUSE sp|P33534|OPRK\_MOUSE tr|D3Z733|D3Z733\_MOUSE sp|Q9QXG2|RAE1\_MOUSE tr|E9Q392|E9Q392\_MOUSE sp|Q7TSY6-6|CELF4\_MOUSE sp|Q7M6Y3-6|PICAL\_MOUSE sp|Q8VCD5|MED17\_MOUSE sp|O88200|CLC11\_MOUSE tr|F6YJY2|F6YJY2\_MOUSE sp|Q9ERZ6-2|FIGN\_MOUSE sp|O88665|BRD7\_MOUSE tr|F7A8L1|F7A8L1\_MOUSE sp|P01736|TVB3\_MOUSE sp|O70209|PDLI3\_MOUSE sp|Q9R0S3|MMP17\_MOUSE sp|Q9JHJ3|NCUG1\_MOUSE sp|Q2UY11|COSA1\_MOUSE sp|Q2UY11-2|COSA1\_MOUSE tr|F6YKN8|F6YKN8\_MOUSE sp|P01868|IGHG1\_MOUSE tr|Q8R2B4|Q8R2B4\_MOUSE tr|B2RT27|B2RT27\_MOUSE tr|G3UX39|G3UX39\_MOUSE sp|P19157|GSTP1\_MOUSE sp|Q9CQT2|RBM7\_MOUSE tr|A0A075B5Y9|A0A075B5Y9\_MOUSE tr|E0CYE3|E0CYE3\_MOUSE sp|Q5XF90-4|AT134\_MOUSE sp|Q6ZPT1|KLHL9\_MOUSE sp|Q9CYZ8|SSBP2\_MOUSE sp|P15307|REL\_MOUSE tr|Q3U0V6|Q3U0V6\_MOUSE sp|Q9CYZ8-2|SSBP2\_MOUSE tr|E9Q6D6|E9Q6D6\_MOUSE tr|A4QPD3|A4QPD3\_MOUSE sp|Q8CFI7|RPB2\_MOUSE sp|Q3UPL0|SC31A\_MOUSE sp|Q3UPL0-2|SC31A\_MOUSE tr|E9PU88|E9PU88\_MOUSE tr|D3YXG2|D3YXG2\_MOUSE sp|O09198|MAL\_MOUSE sp|O09198-2|MAL\_MOUSE sp|Q69Z89-3|RADIL\_MOUSE sp|Q69Z89|RADIL\_MOUSE sp|Q69Z89-5|RADIL\_MOUSE sp|Q69Z89-2|RADIL\_MOUSE sp|Q3TCN2|PLBL2\_MOUSE sp|Q9JJ28|FLII\_MOUSE tr|A2ASX1|A2ASX1\_MOUSE tr|D3YWJ9|D3YWJ9\_MOUSE sp|O88396|GRPE2\_MOUSE sp|Q3UZ39|LRRF1\_MOUSE tr|D3Z4J5|D3Z4J5\_MOUSE sp|Q8BJY1|PSMD5\_MOUSE sp|Q5SPL2|PHF12\_MOUSE sp|Q8CH02|SUGP1\_MOUSE sp|Q62240-3|KDM5D\_MOUSE sp|Q5SPL2-2|PHF12\_MOUSE sp|Q8C3X8|LMF2\_MOUSE tr|E9Q144|E9Q144\_MOUSE sp|Q8R2R3|AAGAB\_MOUSE sp|Q9CRG1|TM7S3\_MOUSE tr|Q9D2T6|Q9D2T6\_MOUSE tr|Q3TP97|Q3TP97\_MOUSE tr|B9EJA3|B9EJA3\_MOUSE tr|E9PVW8|E9PVW8\_MOUSE tr|E9PVR6|E9PVR6\_MOUSE sp|Q91WF7|FIG4\_MOUSE tr|G3UWK6|G3UWK6\_MOUSE tr|E9Q7I7|E9Q7I7\_MOUSE sp|Q64487-12|PTPRD\_MOUSE tr|G3UX60|G3UX60\_MOUSE sp|P01872-2|IGHM\_MOUSE tr|G3UYU6|G3UYU6\_MOUSE tr|E9PU92|E9PU92\_MOUSE tr|G5E897|G5E897\_MOUSE tr|F6V5I7|F6V5I7\_MOUSE sp|D3Z5L6|S18B1\_MOUSE sp|Q8BUG2|CNDP1\_MOUSE sp|O35680|RT12\_MOUSE sp|Q8K1A6|C2D1A\_MOUSE sp|Q91V12-3|BACH\_MOUSE tr|A0A087WQ85|A0A087WQ85\_MOUSE sp|Q9CWR0|ARHGP\_MOUSE sp|Q91V12-2|BACH\_MOUSE tr|E9PX94|E9PX94\_MOUSE tr|E9PYH2|E9PYH2\_MOUSE sp|Q9CWR0-2|ARHGP\_MOUSE tr|B1ASZ3|B1ASZ3\_MOUSE tr|A0A087WRI3|A0A087WRI3\_MOUSE tr|F6SH61|F6SH61\_MOUSE sp|Q91V12-4|BACH\_MOUSE sp|Q91V12|BACH\_MOUSE tr|B2RWG1|B2RWG1\_MOUSE sp|Q9Z319|CORIN\_MOUSE tr|D3Z614|D3Z614\_MOUSE tr|F6YNC4|F6YNC4\_MOUSE tr|F6VLT8|F6VLT8\_MOUSE tr|Q78FW7|Q78FW7\_MOUSE sp|Q60585|ZFP30\_MOUSE sp|Q9JHW9|AL1A3\_MOUSE tr|E9Q5I0|E9Q5I0\_MOUSE sp|Q8BZH0|S39AD\_MOUSE sp|Q8BZH0-2|S39AD\_MOUSE sp|Q9QZS8|SH2D3\_MOUSE tr|E9PW23|E9PW23\_MOUSE sp|Q3UPR7|TM102\_MOUSE sp|Q8K0W3|RN208\_MOUSE sp|Q61009|SCRB1\_MOUSE tr|B2RUS7|B2RUS7\_MOUSE sp|Q61666|HIRA\_MOUSE sp|Q61666-2|HIRA\_MOUSE sp|Q8C7B8|ZSWM4\_MOUSE sp|Q61666-3|HIRA\_MOUSE tr|D3YWJ1|D3YWJ1\_MOUSE tr|Q3V2F3|Q3V2F3\_MOUSE sp|Q9WVB4|SLIT3\_MOUSE sp|O35903|CCL25\_MOUSE tr|L7MU65|L7MU65\_MOUSE tr|E9Q9J3|E9Q9J3\_MOUSE tr|D3YX91|D3YX91\_MOUSE tr|A8Y5N1|A8Y5N1\_MOUSE sp|Q60793|KLF4\_MOUSE tr|K9J7D1|K9J7D1\_MOUSE sp|Q99P21|MUTYH\_MOUSE tr|E9Q8M1|E9Q8M1\_MOUSE tr|S4R1X7|S4R1X7\_MOUSE tr|E9PVL3|E9PVL3\_MOUSE tr|E9PZT5|E9PZT5\_MOUSE sp|Q91ZD6|EAF2\_MOUSE tr|D3Z7I2|D3Z7I2\_MOUSE sp|Q9D0B5|TSTD3\_MOUSE sp|Q05910|ADAM8\_MOUSE tr|Q3U7G2|Q3U7G2\_MOUSE tr|B2RW07|B2RW07\_MOUSE tr|K4DI60|K4DI60\_MOUSE tr|S4R1U4|S4R1U4\_MOUSE sp|Q9CR75|TNR12\_MOUSE sp|Q504N7|IFIXL\_MOUSE tr|Q8VFP2|Q8VFP2\_MOUSE sp|Q9DAD6|PROF3\_MOUSE sp|Q91ZZ5|RXFP2\_MOUSE tr|J3KMQ5|J3KMQ5\_MOUSE tr|J3QPB4|J3QPB4\_MOUSE sp|Q9EP71|RAI14\_MOUSE tr|D3Z3D2|D3Z3D2\_MOUSE tr|E9Q0U9|E9Q0U9\_MOUSE tr|A2A9Z2|A2A9Z2\_MOUSE tr|A2A9Z1|A2A9Z1\_MOUSE sp|P70217|HXD13\_MOUSE sp|O08919|NUMBL\_MOUSE tr|F6S185|F6S185\_MOUSE tr|B1AT13|B1AT13\_MOUSE sp|Q8CDK2|CBPC2\_MOUSE sp|Q8K3R3-2|PLCD4\_MOUSE sp|Q8K3R3|PLCD4\_MOUSE sp|Q8CDK2-2|CBPC2\_MOUSE sp|Q8CDK2-3|CBPC2\_MOUSE sp|Q8CDK2-6|CBPC2\_MOUSE sp|Q6P8I4|PCNP\_MOUSE sp|Q8CDK2-4|CBPC2\_MOUSE tr|D6RGL8|D6RGL8\_MOUSE sp|Q8CDK2-5|CBPC2\_MOUSE tr|Q3TM89|Q3TM89\_MOUSE sp|Q8K3R3-4|PLCD4\_MOUSE tr|D6RGN2|D6RGN2\_MOUSE tr|D6RDQ3|D6RDQ3\_MOUSE sp|Q9WU42-2|NCOR2\_MOUSE sp|Q99MS8|TPGS1\_MOUSE tr|E9Q9V3|E9Q9V3\_MOUSE sp|Q9DBG1|CP27A\_MOUSE tr|F8VPJ9|F8VPJ9\_MOUSE tr|E0CYW0|E0CYW0\_MOUSE sp|Q7TNT2|FACR2\_MOUSE sp|P10923|OSTP\_MOUSE tr|D3Z4N2|D3Z4N2\_MOUSE tr|B2RR19|B2RR19\_MOUSE sp|Q7TNT2-2|FACR2\_MOUSE tr|E9Q6R9|E9Q6R9\_MOUSE tr|D3Z513|D3Z513\_MOUSE sp|O88310|ITL1A\_MOUSE tr|F8WIP8|F8WIP8\_MOUSE sp|Q8C0D5|ETUD1\_MOUSE P31096 sp|P16382-3|IL4RA\_MOUSE tr|A2A5P3|A2A5P3\_MOUSE sp|P48972|MYBB\_MOUSE sp|P16382-2|IL4RA\_MOUSE sp|P16382|IL4RA\_MOUSE sp|P97309|MESP1\_MOUSE sp|Q9JJY3|NSMA2\_MOUSE tr|D3YWV4|D3YWV4\_MOUSE sp|P70669|PHEX\_MOUSE sp|Q8BH57-3|WDR48\_MOUSE sp|Q8BH57|WDR48\_MOUSE sp|Q8BH57-2|WDR48\_MOUSE tr|A2AC80|A2AC80\_MOUSE sp|P70396|DLX5\_MOUSE sp|P08042-2|ZFP1\_MOUSE tr|Q3UI68|Q3UI68\_MOUSE sp|P70396-2|DLX5\_MOUSE sp|Q9R002|IFI2\_MOUSE sp|P70396-3|DLX5\_MOUSE sp|P08042|ZFP1\_MOUSE sp|Q3V0K9|PLSI\_MOUSE sp|Q8JZP2|SYN3\_MOUSE sp|Q8C0T7|MFSD9\_MOUSE sp|O88842-2|FGD3\_MOUSE sp|O54824-2|IL16\_MOUSE tr|F7CXJ2|F7CXJ2\_MOUSE tr|Q3TNB8|Q3TNB8\_MOUSE sp|O88842|FGD3\_MOUSE sp|Q61016|GBG7\_MOUSE tr|G3X9Q2|G3X9Q2\_MOUSE tr|Z4YL99|Z4YL99\_MOUSE sp|Q61592|GAS6\_MOUSE sp|Q99NA9|PCGF6\_MOUSE tr|E9Q4C0|E9Q4C0\_MOUSE sp|Q924C6|LOXL4\_MOUSE sp|Q6P7F1-4|MPP4\_MOUSE tr|E9Q333|E9Q333\_MOUSE sp|Q9QXZ9|OPN4\_MOUSE sp|Q9Z0X1|AIFM1\_MOUSE tr|E9Q600|E9Q600\_MOUSE sp|Q8BGY7|F210A\_MOUSE sp|Q6PAL8|DEN5A\_MOUSE sp|Q61140|BCAR1\_MOUSE tr|E9Q0V3|E9Q0V3\_MOUSE tr|G3UYK9|G3UYK9\_MOUSE sp|P23950|TISB\_MOUSE tr|Q7TS33|Q7TS33\_MOUSE sp|Q9QXX0|JAG1\_MOUSE sp|Q78IK2|USMG5\_MOUSE sp|Q4VA55|MUML1\_MOUSE sp|Q9JIE6|TSLP\_MOUSE sp|Q9QZ29|IGB1B\_MOUSE tr|H3BKN4|H3BKN4\_MOUSE sp|Q8VFD2|OL490\_MOUSE tr|F7AHX3|F7AHX3\_MOUSE tr|F6V527|F6V527\_MOUSE tr|A2ARY0|A2ARY0\_MOUSE tr|Q9QZ22|Q9QZ22\_MOUSE sp|P59729|RIN3\_MOUSE sp|Q64331|MYO6\_MOUSE sp|Q9Z1Z2|STRAP\_MOUSE sp|Q8BVM4|AZIN2\_MOUSE sp|Q60749|KHDR1\_MOUSE sp|Q9WU38|SCNNB\_MOUSE tr|Q3UDN6|Q3UDN6\_MOUSE tr|D3YUA8|D3YUA8\_MOUSE sp|Q9DBG6|RPN2\_MOUSE tr|D3YUA5|D3YUA5\_MOUSE sp|Q9CRB2|NHP2\_MOUSE sp|Q8VBV4|FBXW7\_MOUSE tr|A2ACG7|A2ACG7\_MOUSE sp|Q8K3K8|OPTN\_MOUSE sp|Q8BIK6|TMPS7\_MOUSE sp|P32043|HXC5\_MOUSE tr|D6RG81|D6RG81\_MOUSE sp|P10854|H2B1M\_MOUSE sp|P08505|IL6\_MOUSE sp|A4FUQ5|FPRS4\_MOUSE tr|E9Q517|E9Q517\_MOUSE sp|Q8K363|DDX18\_MOUSE tr|E9Q3V0|E9Q3V0\_MOUSE sp|P28571|SC6A9\_MOUSE sp|P28571-1|SC6A9\_MOUSE sp|P28571-2|SC6A9\_MOUSE sp|Q9Z2I8|SUCB2\_MOUSE sp|Q924K8-2|MTA3\_MOUSE tr|E9Q794|E9Q794\_MOUSE tr|Q3U3A7|Q3U3A7\_MOUSE tr|Q3UII8|Q3UII8\_MOUSE sp|Q924K8|MTA3\_MOUSE sp|Q91YX0|THMS2\_MOUSE sp|Q91ZX6-2|SENP2\_MOUSE sp|Q91ZX6|SENP2\_MOUSE sp|Q2EMV9-2|PAR14\_MOUSE sp|Q91ZX6-3|SENP2\_MOUSE tr|E9Q8M8|E9Q8M8\_MOUSE tr|Q8VFP8|Q8VFP8\_MOUSE sp|Q8C4S8|DEN2A\_MOUSE sp|Q8VII8|MCR\_MOUSE tr|A3KN90|A3KN90\_MOUSE sp|Q6VNS1-2|NTRK3\_MOUSE tr|D3Z473|D3Z473\_MOUSE sp|Q62443|NPTX1\_MOUSE tr|D3Z2D2|D3Z2D2\_MOUSE tr|H7BX21|H7BX21\_MOUSE tr|D6RIL1|D6RIL1\_MOUSE sp|Q6VNS1|NTRK3\_MOUSE sp|Q6P902|TXND2\_MOUSE sp|Q6H1V1|BEST3\_MOUSE tr|B8JJG3|B8JJG3\_MOUSE sp|E9PVD1|CCD62\_MOUSE sp|A2ACP1-2|TT39A\_MOUSE sp|A2ACP1|TT39A\_MOUSE tr|Q50E62|Q50E62\_MOUSE sp|Q8BHP7|OLM2A\_MOUSE tr|E9Q0Q0|E9Q0Q0\_MOUSE tr|A2CEQ8|A2CEQ8\_MOUSE tr|A2CEQ6|A2CEQ6\_MOUSE sp|D3YYU8-2|OBSL1\_MOUSE sp|Q8CGZ9|PR7B1\_MOUSE tr|E9Q3V7|E9Q3V7\_MOUSE sp|P58466|CTDS1\_MOUSE sp|P83503|NYX\_MOUSE sp|Q9D565-3|WDR64\_MOUSE sp|Q8K2D3|EDC3\_MOUSE sp|Q8R314|S35F5\_MOUSE sp|Q9ERR8|ZN319\_MOUSE sp|Q9DCB4-6|ARP21\_MOUSE tr|L7N2E3|L7N2E3\_MOUSE tr|K7N743|K7N743\_MOUSE tr|M0QWL4|M0QWL4\_MOUSE tr|L7N2D2|L7N2D2\_MOUSE sp|P37040|NCPR\_MOUSE sp|Q9DCB4-2|ARP21\_MOUSE tr|K7N693|K7N693\_MOUSE tr|E9PYQ5|E9PYQ5\_MOUSE sp|Q9DCB4-7|ARP21\_MOUSE tr|E0CZ08|E0CZ08\_MOUSE tr|E0CYS3|E0CYS3\_MOUSE tr|E0CXW0|E0CXW0\_MOUSE tr|M0QWI0|M0QWI0\_MOUSE tr|K7N5W1|K7N5W1\_MOUSE sp|Q9DCB4-8|ARP21\_MOUSE tr|Q5PPQ4|Q5PPQ4\_MOUSE tr|L7N2A3|L7N2A3\_MOUSE tr|E0CY19|E0CY19\_MOUSE tr|L7N2E2|L7N2E2\_MOUSE tr|K7N6G6|K7N6G6\_MOUSE sp|Q9DCB4-10|ARP21\_MOUSE tr|E0CYU3|E0CYU3\_MOUSE tr|Q8VFG4|Q8VFG4\_MOUSE sp|Q5DTJ9|MYPN\_MOUSE sp|Q3UP87|ELNE\_MOUSE sp|Q2TL60|ZN667\_MOUSE sp|Q62018-3|CTR9\_MOUSE sp|Q62018|CTR9\_MOUSE sp|Q9Z1S8|GAB2\_MOUSE sp|Q91WU2-2|S22A7\_MOUSE sp|Q91WU2|S22A7\_MOUSE tr|Q3ZB57|Q3ZB57\_MOUSE sp|Q9DBR7-2|MYPT1\_MOUSE tr|A2AP29|A2AP29\_MOUSE sp|Q9DBR7|MYPT1\_MOUSE tr|Q3TZY4|Q3TZY4\_MOUSE sp|Q80W04|TMCC2\_MOUSE sp|P02772|FETA\_MOUSE sp|A6H8H5|KCNB2\_MOUSE tr|D3Z589|D3Z589\_MOUSE tr|J3QNZ1|J3QNZ1\_MOUSE tr|Q80VZ7|Q80VZ7\_MOUSE sp|Q3UH68-2|LIMC1\_MOUSE tr|A4UUI2|A4UUI2\_MOUSE sp|Q99JG7|TNIP2\_MOUSE tr|A0A075B5L2|A0A075B5L2\_MOUSE sp|Q3UH68|LIMC1\_MOUSE sp|Q61850|FOXC2\_MOUSE sp|Q3UVL4|VPS51\_MOUSE tr|H3BKC6|H3BKC6\_MOUSE tr|Q3UXL1|Q3UXL1\_MOUSE tr|G3XA14|G3XA14\_MOUSE sp|A9JSM3|TM238\_MOUSE tr|Q32ME0|Q32ME0\_MOUSE sp|Q9D1Q6|ERP44\_MOUSE tr|F7AGT6|F7AGT6\_MOUSE tr|B1AR82|B1AR82\_MOUSE tr|M0QW64|M0QW64\_MOUSE tr|Q5SWZ5|Q5SWZ5\_MOUSE tr|A0A087WR85|A0A087WR85\_MOUSE tr|F6W3K2|F6W3K2\_MOUSE sp|Q8QZR1|ATTY\_MOUSE sp|Q9WUB3|PYGM\_MOUSE tr|E9PUM3|E9PUM3\_MOUSE tr|E0CYA7|E0CYA7\_MOUSE tr|D3Z307|D3Z307\_MOUSE tr|C0KTP6|C0KTP6\_MOUSE sp|Q9DCH4|EIF3F\_MOUSE tr|Q8VFG3|Q8VFG3\_MOUSE tr|F6YRW4|F6YRW4\_MOUSE sp|P59997|KDM2A\_MOUSE sp|O88502|PDE8A\_MOUSE sp|Q63934|PO4F2\_MOUSE sp|P09041|PGK2\_MOUSE sp|P49722|PSA2\_MOUSE tr|F8WIS9|F8WIS9\_MOUSE sp|Q9JHZ2|ANKH\_MOUSE tr|F6S054|F6S054\_MOUSE sp|Q3U481|MFS12\_MOUSE tr|E0CXE0|E0CXE0\_MOUSE sp|Q9JHN8|STK19\_MOUSE tr|E0CXS4|E0CXS4\_MOUSE sp|Q9CQE5|RGS10\_MOUSE tr|G3XA20|G3XA20\_MOUSE tr|Q3YAA9|Q3YAA9\_MOUSE sp|Q3UWA6-2|GUC2C\_MOUSE sp|Q9QXT8-3|CSEN\_MOUSE sp|Q3UWA6|GUC2C\_MOUSE tr|A2AHT3|A2AHT3\_MOUSE sp|Q9QXT8|CSEN\_MOUSE sp|Q8CFE6|S38A2\_MOUSE sp|Q8BP71-2|RFOX2\_MOUSE sp|Q9D964|GATM\_MOUSE sp|Q8BP71|RFOX2\_MOUSE sp|Q99J56|DERL1\_MOUSE tr|E9QMJ1|E9QMJ1\_MOUSE tr|E9QN63|E9QN63\_MOUSE sp|Q9QWY8-3|ASAP1\_MOUSE sp|Q9QWY8|ASAP1\_MOUSE sp|Q9QWY8-4|ASAP1\_MOUSE sp|Q8BUK6|HOOK3\_MOUSE tr|V9GXX3|V9GXX3\_MOUSE sp|Q8K1S7|KREM2\_MOUSE sp|Q8BXR9-2|OSBL6\_MOUSE sp|Q8BXR9|OSBL6\_MOUSE sp|P26954|IL3B2\_MOUSE sp|Q5STT6|FA71B\_MOUSE sp|Q8BNV1|TRM2A\_MOUSE tr|Q3UYU2|Q3UYU2\_MOUSE tr|J3QNS6|J3QNS6\_MOUSE tr|A2ATW2|A2ATW2\_MOUSE tr|A0A087WPX2|A0A087WPX2\_MOUSE tr|Q5SYH9|Q5SYH9\_MOUSE tr|E9Q4H5|E9Q4H5\_MOUSE tr|E9PUQ7|E9PUQ7\_MOUSE tr|F6ZHN3|F6ZHN3\_MOUSE sp|Q61066|NR0B1\_MOUSE sp|Q00175|PRGR\_MOUSE tr|B1AVU6|B1AVU6\_MOUSE sp|Q9WUZ5|TNNI1\_MOUSE tr|F6QC77|F6QC77\_MOUSE sp|Q6ZQ89-3|MARH6\_MOUSE sp|Q5XKN4|JAGN1\_MOUSE sp|Q6ZQ89|MARH6\_MOUSE sp|Q6PDS3-3|SARM1\_MOUSE sp|Q3UM18|LSG1\_MOUSE tr|Q5M8S1|Q5M8S1\_MOUSE sp|Q6PDS3-2|SARM1\_MOUSE sp|Q6VMN6|PRLHR\_MOUSE sp|Q91YP3|DEOC\_MOUSE sp|Q3UM18-2|LSG1\_MOUSE sp|Q6PDS3|SARM1\_MOUSE tr|F2Z436|F2Z436\_MOUSE sp|Q6P8X6|UBP50\_MOUSE sp|Q9EPL0|XYLT2\_MOUSE sp|Q8K3E5|AHI1\_MOUSE tr|E9QP54|E9QP54\_MOUSE tr|D6RCG3|D6RCG3\_MOUSE sp|P97946|VEGFD\_MOUSE sp|Q61221|HIF1A\_MOUSE sp|Q8K1L0|CREB5\_MOUSE sp|Q8K3E5-2|AHI1\_MOUSE sp|Q6P8X6-2|UBP50\_MOUSE sp|Q61221-2|HIF1A\_MOUSE tr|Q8BGU0|Q8BGU0\_MOUSE tr|Q6UKZ0|Q6UKZ0\_MOUSE tr|Q7TRE0|Q7TRE0\_MOUSE sp|Q9Z1J2|NEK4\_MOUSE sp|P31254|UBA1Y\_MOUSE sp|Q9Z1J2-2|NEK4\_MOUSE sp|Q3UX10|TBAL3\_MOUSE tr|Q6GTE9|Q6GTE9\_MOUSE tr|Q8BVT3|Q8BVT3\_MOUSE tr|G3UY94|G3UY94\_MOUSE tr|A2A799|A2A799\_MOUSE sp|P47750|TSHR\_MOUSE tr|E9Q1A6|E9Q1A6\_MOUSE sp|Q5EE38|ACD\_MOUSE sp|Q5EE38-2|ACD\_MOUSE tr|S4R2U3|S4R2U3\_MOUSE tr|S4R211|S4R211\_MOUSE tr|Q3URI6|Q3URI6\_MOUSE sp|P01680|KV4A1\_MOUSE sp|Q6P5D3-4|DHX57\_MOUSE tr|E9Q2H8|E9Q2H8\_MOUSE tr|G5E8T9|G5E8T9\_MOUSE tr|D3YUX8|D3YUX8\_MOUSE sp|Q99KB8-2|GLO2\_MOUSE tr|Q8VFA8|Q8VFA8\_MOUSE tr|D3YWI0|D3YWI0\_MOUSE sp|Q99KB8|GLO2\_MOUSE sp|Q64280|LFTY1\_MOUSE tr|F6T5T2|F6T5T2\_MOUSE tr|F8WHL1|F8WHL1\_MOUSE sp|Q8BHB3|LAX1\_MOUSE sp|Q5IRJ6-2|ZNT9\_MOUSE sp|Q5IRJ6|ZNT9\_MOUSE sp|P97347|RPTN\_MOUSE tr|E9Q624|E9Q624\_MOUSE tr|E9QA22|E9QA22\_MOUSE tr|Q497W1|Q497W1\_MOUSE sp|Q9DAA6-2|EXOS1\_MOUSE sp|Q8K120-2|NFAC4\_MOUSE tr|F6VMP2|F6VMP2\_MOUSE sp|Q8K120|NFAC4\_MOUSE sp|Q9CZ42|NNRD\_MOUSE sp|Q921P9|TCAL1\_MOUSE sp|Q9QWV4|MLF1\_MOUSE sp|Q9CZ42-3|NNRD\_MOUSE sp|Q9CZ42-2|NNRD\_MOUSE tr|Q8JZS2|Q8JZS2\_MOUSE tr|J3QMM7|J3QMM7\_MOUSE sp|Q8BH01-3|TMCO3\_MOUSE tr|K3W4M4|K3W4M4\_MOUSE sp|Q71RI9|KAT3\_MOUSE tr|Q8VFX1|Q8VFX1\_MOUSE sp|Q71RI9-2|KAT3\_MOUSE tr|D3Z4I2|D3Z4I2\_MOUSE sp|Q80X85|RT07\_MOUSE tr|Q3UZ35|Q3UZ35\_MOUSE sp|Q8QZX0|SBK1\_MOUSE tr|B9EIX3|B9EIX3\_MOUSE sp|Q8BZQ7|ANC2\_MOUSE tr|G5E874|G5E874\_MOUSE sp|O70166|STMN3\_MOUSE sp|Q8BGI4|FA13A\_MOUSE tr|Q9DAX0|Q9DAX0\_MOUSE sp|P51437|CRAMP\_MOUSE sp|Q9JHH2-2|TSN32\_MOUSE sp|Q9JHH2-5|TSN32\_MOUSE sp|Q9JHH2-3|TSN32\_MOUSE sp|Q3TA38|T120B\_MOUSE tr|D3Z0S2|D3Z0S2\_MOUSE sp|Q3TA38-2|T120B\_MOUSE tr|A0A075B5U0|A0A075B5U0\_MOUSE sp|Q9WVS7-2|MP2K5\_MOUSE tr|A0A087WS07|A0A087WS07\_MOUSE sp|Q9JHH2-4|TSN32\_MOUSE sp|Q9WVS7|MP2K5\_MOUSE sp|Q8CCT7|NSUN3\_MOUSE sp|Q9JHH2-6|TSN32\_MOUSE sp|Q9D1F5|CE045\_MOUSE tr|J3QMP7|J3QMP7\_MOUSE sp|P30935|SSR3\_MOUSE sp|P57680-2|EVC\_MOUSE tr|E9Q2E6|E9Q2E6\_MOUSE sp|P57680|EVC\_MOUSE tr|A2AK85|A2AK85\_MOUSE sp|Q3UHH1-5|ZSWM8\_MOUSE sp|Q8BZ47|ZN609\_MOUSE tr|E9Q1Q1|E9Q1Q1\_MOUSE sp|Q60592|MAST2\_MOUSE tr|B1AST7|B1AST7\_MOUSE tr|B1AST8|B1AST8\_MOUSE tr|E9QLW6|E9QLW6\_MOUSE sp|Q9CQK3|RDM1\_MOUSE sp|Q9R1C6|DGKE\_MOUSE tr|F6RR30|F6RR30\_MOUSE sp|O55143-2|AT2A2\_MOUSE tr|F6WYC8|F6WYC8\_MOUSE sp|O55143|AT2A2\_MOUSE tr|J3KMM5|J3KMM5\_MOUSE sp|Q80T32|GP133\_MOUSE sp|Q9QYS1|WNT16\_MOUSE sp|O88667|RAD\_MOUSE sp|Q8C7E9|CSTFT\_MOUSE sp|Q8C5L3-3|CNOT2\_MOUSE sp|Q66X22-2|NAL9B\_MOUSE tr|Q6PGA2|Q6PGA2\_MOUSE sp|Q91YT2-2|RN185\_MOUSE tr|F8WJ50|F8WJ50\_MOUSE sp|Q8BTY2-2|S4A7\_MOUSE sp|Q8BTY2|S4A7\_MOUSE tr|Q9CUK2|Q9CUK2\_MOUSE tr|E9PV98|E9PV98\_MOUSE sp|Q9ESN9|JIP3\_MOUSE sp|Q8R088-3|GLP3L\_MOUSE sp|Q9ESN9-6|JIP3\_MOUSE tr|E9Q987|E9Q987\_MOUSE sp|Q9ESN9-4|JIP3\_MOUSE sp|Q62318-2|TIF1B\_MOUSE tr|H3BJ12|H3BJ12\_MOUSE sp|Q9CYB0|TRI13\_MOUSE tr|K3W4Q3|K3W4Q3\_MOUSE tr|E9Q6E0|E9Q6E0\_MOUSE tr|K3W4S4|K3W4S4\_MOUSE tr|J3QNR6|J3QNR6\_MOUSE sp|Q9ESN9-2|JIP3\_MOUSE sp|Q9CQ45|NENF\_MOUSE sp|Q9ESN9-3|JIP3\_MOUSE sp|Q3ULW6-3|CCD33\_MOUSE sp|Q6ZQ38|CAND1\_MOUSE P17697 sp|Q05AA6|DRP2\_MOUSE tr|A2A5F7|A2A5F7\_MOUSE sp|Q3ULW6-4|CCD33\_MOUSE sp|Q91YU6|LZTS2\_MOUSE tr|B1AV35|B1AV35\_MOUSE tr|Q3TMP1|Q3TMP1\_MOUSE tr|F6UIS1|F6UIS1\_MOUSE tr|J3QNJ8|J3QNJ8\_MOUSE sp|P15702|LEUK\_MOUSE sp|Q8QZR0|TR1L1\_MOUSE sp|P70206|PLXA1\_MOUSE tr|V9GXE9|V9GXE9\_MOUSE tr|V9GX74|V9GX74\_MOUSE tr|V9GXA5|V9GXA5\_MOUSE sp|Q9D9W1|PIFO\_MOUSE tr|D3YY91|D3YY91\_MOUSE tr|F6TBG5|F6TBG5\_MOUSE sp|Q925H1|TRPS1\_MOUSE sp|Q9D9W1-2|PIFO\_MOUSE sp|Q9QY01|ULK2\_MOUSE tr|G3UW90|G3UW90\_MOUSE tr|Q80V18|Q80V18\_MOUSE sp|Q8CIV8|TBCE\_MOUSE tr|G5E8Q6|G5E8Q6\_MOUSE tr|E0CZ81|E0CZ81\_MOUSE tr|G3UY57|G3UY57\_MOUSE sp|Q8BZJ7-2|DCNL2\_MOUSE tr|Q8R096|Q8R096\_MOUSE sp|Q8BZJ7|DCNL2\_MOUSE tr|B1AUG0|B1AUG0\_MOUSE sp|Q01405|SC23A\_MOUSE sp|Q64143|P55G\_MOUSE tr|E9Q1S3|E9Q1S3\_MOUSE sp|Q8BH79-2|ANO10\_MOUSE sp|Q8BH79|ANO10\_MOUSE sp|Q91V27|MELPH\_MOUSE sp|Q8BH79-4|ANO10\_MOUSE sp|Q69ZB0-3|LRCC1\_MOUSE sp|Q9CQ35|GRPL2\_MOUSE tr|V9GX53|V9GX53\_MOUSE sp|Q9CQ35-2|GRPL2\_MOUSE tr|Q3V1N5|Q3V1N5\_MOUSE sp|A0JNT9|BICR1\_MOUSE tr|M0QWK9|M0QWK9\_MOUSE sp|Q9EQW6|OLIG2\_MOUSE tr|F6R5X6|F6R5X6\_MOUSE tr|Q8VGU6|Q8VGU6\_MOUSE tr|Q8C8I3|Q8C8I3\_MOUSE sp|O70230|ZN143\_MOUSE sp|O70230-2|ZN143\_MOUSE tr|D3Z722|D3Z722\_MOUSE sp|Q8BMF4|ODP2\_MOUSE tr|F6V6T4|F6V6T4\_MOUSE tr|A2AGL8|A2AGL8\_MOUSE sp|Q8BVN7|S2541\_MOUSE sp|Q8BVN7-2|S2541\_MOUSE sp|Q9D304|RN128\_MOUSE sp|Q9D300-2|RGF1C\_MOUSE tr|E9Q5B4|E9Q5B4\_MOUSE tr|A2ARU5|A2ARU5\_MOUSE tr|E9Q973|E9Q973\_MOUSE sp|Q8R368|DYXC1\_MOUSE sp|Q8VCW4|UN93B\_MOUSE tr|Q3UU93|Q3UU93\_MOUSE sp|Q8K3F7|TDH\_MOUSE tr|E9PYK0|E9PYK0\_MOUSE sp|Q9JI10|STK3\_MOUSE sp|P80560|PTPR2\_MOUSE tr|E9Q8P6|E9Q8P6\_MOUSE sp|Q921D9-2|GRHL1\_MOUSE sp|Q9JI10-2|STK3\_MOUSE tr|E9QMF0|E9QMF0\_MOUSE tr|Q05AC5|Q05AC5\_MOUSE sp|O70138|MMP8\_MOUSE sp|P40223|CSF3R\_MOUSE sp|Q9D1R1|T126B\_MOUSE sp|Q922D8|C1TC\_MOUSE tr|Q6P8H4|Q6P8H4\_MOUSE sp|Q9WUT7|CCR9\_MOUSE sp|Q3TZA2|CDKL4\_MOUSE sp|P62500-3|T22D1\_MOUSE tr|E9QLZ1|E9QLZ1\_MOUSE tr|F6Z1P8|F6Z1P8\_MOUSE sp|Q7TQA1-5|IGSF1\_MOUSE tr|D3Z0V7|D3Z0V7\_MOUSE sp|Q9WTJ4|FIZ1\_MOUSE sp|P62500|T22D1\_MOUSE tr|Q6IMH7|Q6IMH7\_MOUSE tr|G3UY12|G3UY12\_MOUSE sp|Q9JKE2|TREM1\_MOUSE tr|Q3UTY5|Q3UTY5\_MOUSE sp|P50992|ATP4B\_MOUSE tr|Q3UWJ5|Q3UWJ5\_MOUSE tr|Q8CDM3|Q8CDM3\_MOUSE sp|Q7TN84|SYT14\_MOUSE sp|Q9R0X5|RPGR\_MOUSE tr|A2ADP5|A2ADP5\_MOUSE sp|Q9R0X5-3|RPGR\_MOUSE sp|Q9R0X5-2|RPGR\_MOUSE tr|A2ADP2|A2ADP2\_MOUSE tr|A0A067XG46|A0A067XG46\_MOUSE sp|Q9R0X5-4|RPGR\_MOUSE sp|P61161|ARP2\_MOUSE tr|D6RFD9|D6RFD9\_MOUSE tr|Q8VDU3|Q8VDU3\_MOUSE tr|Q8C7I4|Q8C7I4\_MOUSE tr|A0A087WRM8|A0A087WRM8\_MOUSE sp|A7RDN6|RNLS\_MOUSE tr|Q8R435|Q8R435\_MOUSE sp|Q8BJ37|TYDP1\_MOUSE sp|P52189|KCNJ4\_MOUSE tr|F6WBP9|F6WBP9\_MOUSE tr|B8JJC1|B8JJC1\_MOUSE sp|Q8VC42|MIC1\_MOUSE tr|E9Q079|E9Q079\_MOUSE sp|Q9DBI2|BBS10\_MOUSE sp|Q9QXP0|RHCG\_MOUSE tr|D3Z6V7|D3Z6V7\_MOUSE tr|Q08EC9|Q08EC9\_MOUSE sp|Q64514-2|TPP2\_MOUSE sp|P55065|PLTP\_MOUSE sp|Q9D0S9|HINT2\_MOUSE tr|A2A5K2|A2A5K2\_MOUSE sp|Q9JM55|TOB2\_MOUSE sp|Q3UKC1|TAXB1\_MOUSE sp|Q3UNX5-2|ACSM3\_MOUSE sp|Q3UNX5|ACSM3\_MOUSE sp|P05531|XLR\_MOUSE sp|P35436|NMDE1\_MOUSE tr|Q9D6C3|Q9D6C3\_MOUSE tr|Q8BS74|Q8BS74\_MOUSE sp|Q9DC60|UBIA1\_MOUSE tr|F6R2M8|F6R2M8\_MOUSE sp|Q99MB3-2|TM2D1\_MOUSE sp|Q99MB3|TM2D1\_MOUSE sp|Q8VBY2|KKCC1\_MOUSE tr|B1AUD3|B1AUD3\_MOUSE tr|E9PX23|E9PX23\_MOUSE sp|P35487|ODPAT\_MOUSE tr|D3Z1L1|D3Z1L1\_MOUSE tr|F8WHY8|F8WHY8\_MOUSE sp|Q8K4B0|MTA1\_MOUSE sp|P28236|CXA8\_MOUSE tr|F2Z4B7|F2Z4B7\_MOUSE sp|Q922I5|FOXI1\_MOUSE sp|Q8CIG3|KDM1B\_MOUSE tr|F6WVB0|F6WVB0\_MOUSE sp|O70230-3|ZN143\_MOUSE tr|G5E867|G5E867\_MOUSE tr|Q8K596|Q8K596\_MOUSE tr|A2A821|A2A821\_MOUSE tr|Q8BIS1|Q8BIS1\_MOUSE sp|P21614|VTDB\_MOUSE sp|Q9DBR3|ARMC8\_MOUSE sp|Q8CHP0|ZC3H3\_MOUSE tr|D3YV89|D3YV89\_MOUSE sp|Q9DBR3-2|ARMC8\_MOUSE tr|Q91XZ8|Q91XZ8\_MOUSE tr|D3Z6I1|D3Z6I1\_MOUSE tr|D3Z081|D3Z081\_MOUSE sp|Q14C37|LEMD1\_MOUSE tr|G3X920|G3X920\_MOUSE sp|Q60680-3|IKKA\_MOUSE tr|E9Q605|E9Q605\_MOUSE sp|Q60680-2|IKKA\_MOUSE tr|E9QNL4|E9QNL4\_MOUSE sp|Q60680|IKKA\_MOUSE tr|Q9DAT7|Q9DAT7\_MOUSE sp|Q9R0N4|SYT10\_MOUSE tr|Q9DAY4|Q9DAY4\_MOUSE tr|D3Z6E3|D3Z6E3\_MOUSE sp|Q8C079|STRP1\_MOUSE sp|Q8C079-4|STRP1\_MOUSE sp|Q8C079-3|STRP1\_MOUSE tr|D3YXR4|D3YXR4\_MOUSE sp|Q9Z2U2-2|ZN292\_MOUSE sp|Q9D071-3|MMS19\_MOUSE sp|Q9Z2U2|ZN292\_MOUSE tr|D3Z7G1|D3Z7G1\_MOUSE tr|F7C9N6|F7C9N6\_MOUSE sp|Q9D071|MMS19\_MOUSE sp|O88455|DHCR7\_MOUSE tr|B8JJZ4|B8JJZ4\_MOUSE tr|F6S534|F6S534\_MOUSE sp|Q8BFR2|FSTL5\_MOUSE sp|Q60700|M3K12\_MOUSE sp|Q08376|ZBT14\_MOUSE sp|Q61074|PPM1G\_MOUSE sp|Q9Z0P4|PALM\_MOUSE sp|Q8BJ42-2|DLGP2\_MOUSE sp|Q8BJ42|DLGP2\_MOUSE tr|Q0VF59|Q0VF59\_MOUSE sp|Q61036-2|PAK3\_MOUSE sp|Q8CAI1-2|CC142\_MOUSE sp|Q61036|PAK3\_MOUSE sp|Q8CAI1|CC142\_MOUSE sp|P29387|GBB4\_MOUSE sp|Q05BQ1-2|TUTLA\_MOUSE sp|P14211|CALR\_MOUSE sp|Q8BG21-3|FLOWR\_MOUSE tr|F7CPS9|F7CPS9\_MOUSE sp|Q8BG21-2|FLOWR\_MOUSE tr|D3YZZ2|D3YZZ2\_MOUSE tr|E9Q3A7|E9Q3A7\_MOUSE tr|Q5SPA9|Q5SPA9\_MOUSE tr|E9PXW3|E9PXW3\_MOUSE tr|A0A087WQX0|A0A087WQX0\_MOUSE tr|F6Y3V0|F6Y3V0\_MOUSE sp|Q64487-2|PTPRD\_MOUSE sp|Q9CQ04|OGFD2\_MOUSE sp|Q5DU56-3|NLRC3\_MOUSE sp|Q5DU56-4|NLRC3\_MOUSE sp|Q5DU56|NLRC3\_MOUSE sp|Q9ES28-2|ARHG7\_MOUSE sp|Q99LH1|NOG2\_MOUSE sp|Q9WTQ5-2|AKA12\_MOUSE sp|Q62463|V1AR\_MOUSE sp|Q9ES28-5|ARHG7\_MOUSE sp|Q9ES28|ARHG7\_MOUSE tr|J3QQ49|J3QQ49\_MOUSE sp|Q9WTQ5|AKA12\_MOUSE sp|P59268|ZDHC9\_MOUSE sp|Q5SDA5|GUC2F\_MOUSE sp|Q5DU56-5|NLRC3\_MOUSE tr|A2CEE8|A2CEE8\_MOUSE tr|E9Q2I1|E9Q2I1\_MOUSE sp|Q8CD15|MINA\_MOUSE sp|Q8R1X6|SPG20\_MOUSE tr|Q8K4W8|Q8K4W8\_MOUSE sp|Q6PD05|ZN821\_MOUSE sp|Q80TE4-2|SI1L2\_MOUSE sp|Q80TE4|SI1L2\_MOUSE sp|Q8R1X6-2|SPG20\_MOUSE sp|Q60953|PML\_MOUSE sp|Q8BZX4-2|SREK1\_MOUSE sp|Q7TSG3|FBX5\_MOUSE sp|P70121|ZHX1\_MOUSE sp|Q68FE7|T151B\_MOUSE sp|Q8BI55-2|DYRK4\_MOUSE tr|H3BK31|H3BK31\_MOUSE tr|A0A087WSG8|A0A087WSG8\_MOUSE sp|Q7TPQ3-2|SHPRH\_MOUSE sp|P16110|LEG3\_MOUSE sp|Q7TQI7|ABTB2\_MOUSE sp|Q7TPQ3|SHPRH\_MOUSE tr|Q91Z28|Q91Z28\_MOUSE sp|Q7TPQ3-5|SHPRH\_MOUSE sp|Q7TNM2|TRI46\_MOUSE sp|Q99MK8|ARBK1\_MOUSE tr|Q8C253|Q8C253\_MOUSE tr|Q7TS64|Q7TS64\_MOUSE tr|D3YXA6|D3YXA6\_MOUSE tr|Q0ZNK3|Q0ZNK3\_MOUSE sp|P25322|CCND1\_MOUSE tr|B1ARA9|B1ARA9\_MOUSE sp|Q08481-4|PECA1\_MOUSE sp|Q08481-2|PECA1\_MOUSE sp|A2A5Z6|SMUF2\_MOUSE tr|B1ARB3|B1ARB3\_MOUSE sp|Q8BGT8|PHIPL\_MOUSE sp|Q08481-3|PECA1\_MOUSE tr|D3YTQ5|D3YTQ5\_MOUSE sp|Q08481|PECA1\_MOUSE tr|F7D3N3|F7D3N3\_MOUSE sp|Q80XL6|ACD11\_MOUSE sp|A2A5Z6-2|SMUF2\_MOUSE sp|P16546|SPTN1\_MOUSE tr|A3KGU5|A3KGU5\_MOUSE sp|Q91W92|BORG5\_MOUSE tr|E9Q447|E9Q447\_MOUSE tr|F6PYU5|F6PYU5\_MOUSE tr|D3Z5L4|D3Z5L4\_MOUSE sp|P16546-2|SPTN1\_MOUSE tr|A3KGU7|A3KGU7\_MOUSE tr|E9Q0G1|E9Q0G1\_MOUSE sp|Q8CGY8-2|OGT1\_MOUSE sp|Q8CH36|S36A4\_MOUSE sp|Q9CQJ6|DENR\_MOUSE tr|F6XRP9|F6XRP9\_MOUSE sp|Q8CGY8|OGT1\_MOUSE tr|F6ZZ61|F6ZZ61\_MOUSE Q03247 tr|D3YYZ0|D3YYZ0\_MOUSE tr|A2APZ1|A2APZ1\_MOUSE tr|F6Z1E7|F6Z1E7\_MOUSE tr|E9Q1T3|E9Q1T3\_MOUSE tr|E9PX30|E9PX30\_MOUSE tr|E9Q3F3|E9Q3F3\_MOUSE sp|Q05769|PGH2\_MOUSE sp|Q61599|GDIR2\_MOUSE tr|E9Q2T8|E9Q2T8\_MOUSE sp|P36371|TAP2\_MOUSE sp|Q02085|SNAI1\_MOUSE tr|A6H692|A6H692\_MOUSE sp|Q9WU66|SFRP5\_MOUSE sp|A7TZE6-3|SKIT1\_MOUSE tr|A0A087WS19|A0A087WS19\_MOUSE sp|Q91ZV4|MOGT1\_MOUSE sp|Q8CBX0|CSC1\_MOUSE tr|D3Z4F6|D3Z4F6\_MOUSE tr|F6ZDC5|F6ZDC5\_MOUSE tr|D3YVJ1|D3YVJ1\_MOUSE sp|Q5U4E0|LHPL4\_MOUSE sp|Q7TS48|OL180\_MOUSE tr|E9Q305|E9Q305\_MOUSE tr|E9PXF8|E9PXF8\_MOUSE tr|F8VQ90|F8VQ90\_MOUSE tr|E9Q0D4|E9Q0D4\_MOUSE sp|Q8VHS2|CRUM1\_MOUSE sp|Q9D9H8|CB069\_MOUSE tr|Q6P4T1|Q6P4T1\_MOUSE tr|B2RXZ2|B2RXZ2\_MOUSE sp|Q8BKG3|PTK7\_MOUSE tr|E9QAS1|E9QAS1\_MOUSE sp|Q8VHS2-3|CRUM1\_MOUSE tr|B7ZC62|B7ZC62\_MOUSE sp|Q9WVD5|ORNT1\_MOUSE sp|Q8BJ05-2|ZC3HE\_MOUSE sp|Q8CIV3-2|LIPH\_MOUSE sp|Q9D5T7|HORM1\_MOUSE sp|Q8BIV3|RNBP6\_MOUSE tr|M0QWU0|M0QWU0\_MOUSE sp|Q8CIV3-3|LIPH\_MOUSE sp|Q8CIV3|LIPH\_MOUSE sp|Q6NXI8|CXXC4\_MOUSE sp|Q9D5T7-2|HORM1\_MOUSE tr|F6T9C3|F6T9C3\_MOUSE sp|O55106|STRN\_MOUSE tr|F6Z700|F6Z700\_MOUSE tr|F8WH41|F8WH41\_MOUSE tr|Q6NZR5|Q6NZR5\_MOUSE sp|Q80U16-6|FA65B\_MOUSE sp|Q62148|AL1A2\_MOUSE sp|Q8CHY6|P66A\_MOUSE tr|E9QMN5|E9QMN5\_MOUSE tr|F7CIM2|F7CIM2\_MOUSE tr|Q8VFF7|Q8VFF7\_MOUSE tr|Q3TAA8|Q3TAA8\_MOUSE tr|Q8K356|Q8K356\_MOUSE sp|Q923W9|ADA33\_MOUSE sp|Q8K358|PIGU\_MOUSE sp|Q923W9-2|ADA33\_MOUSE sp|Q2TBA3|MALT1\_MOUSE tr|Q7TR57|Q7TR57\_MOUSE sp|Q8K382|DEN1A\_MOUSE sp|Q2TBA3-2|MALT1\_MOUSE tr|Q8VHE2|Q8VHE2\_MOUSE sp|Q9D4A5-2|CP086\_MOUSE tr|G3UWQ0|G3UWQ0\_MOUSE sp|Q9D4A5|CP086\_MOUSE tr|G3UZ29|G3UZ29\_MOUSE sp|P58463-2|FOXP2\_MOUSE sp|P58463|FOXP2\_MOUSE tr|D3Z142|D3Z142\_MOUSE sp|Q8VFM9|OLF24\_MOUSE tr|F8WIZ9|F8WIZ9\_MOUSE sp|Q3U1J1-2|TFPT\_MOUSE tr|O89006|O89006\_MOUSE sp|P10751|ZFP11\_MOUSE tr|E9Q4X4|E9Q4X4\_MOUSE tr|E0CZC0|E0CZC0\_MOUSE sp|Q8BR07-3|BICD1\_MOUSE sp|Q8BR07|BICD1\_MOUSE tr|B2KG46|B2KG46\_MOUSE sp|Q8BR07-4|BICD1\_MOUSE tr|G3UXK5|G3UXK5\_MOUSE tr|Q6ZWQ7|Q6ZWQ7\_MOUSE sp|Q3TQB2|FXRD1\_MOUSE sp|Q8C3S2-3|TNG6\_MOUSE sp|Q8C3S2-2|TNG6\_MOUSE sp|Q8C3S2|TNG6\_MOUSE sp|Q99JR1|SFXN1\_MOUSE sp|A2AH22-3|AMRA1\_MOUSE tr|F6S6E4|F6S6E4\_MOUSE tr|G5E8R5|G5E8R5\_MOUSE tr|D3YUD9|D3YUD9\_MOUSE sp|A2AH22-4|AMRA1\_MOUSE sp|Q91YK0|LRC49\_MOUSE sp|Q8C160|MSLNL\_MOUSE sp|P14719|ILRL1\_MOUSE tr|F7CCK8|F7CCK8\_MOUSE tr|A1L3P4|A1L3P4\_MOUSE tr|B0QZV3|B0QZV3\_MOUSE tr|F8VPQ4|F8VPQ4\_MOUSE sp|Q3V1H1|CKAP2\_MOUSE sp|Q812A2|SRGP3\_MOUSE tr|E9QN14|E9QN14\_MOUSE sp|Q9ESX2|SP6\_MOUSE sp|Q9WVM1|RGAP1\_MOUSE sp|Q9R0M4|PODXL\_MOUSE tr|F6WMR9|F6WMR9\_MOUSE sp|Q8VI84|NOC3L\_MOUSE tr|Q8VFQ7|Q8VFQ7\_MOUSE sp|P43120|HHEX\_MOUSE tr|A0A087WPT7|A0A087WPT7\_MOUSE tr|E9PUC3|E9PUC3\_MOUSE sp|P21275|BMP4\_MOUSE tr|G3UXH1|G3UXH1\_MOUSE sp|Q69ZX8|ABLM3\_MOUSE tr|B1AQD9|B1AQD9\_MOUSE sp|Q9Z2R6|U119A\_MOUSE sp|Q6P549|SHIP2\_MOUSE sp|Q9D518|F227B\_MOUSE tr|F2Z3W3|F2Z3W3\_MOUSE sp|Q6PIX5-2|RHDF1\_MOUSE tr|A2AQX6|A2AQX6\_MOUSE sp|Q8BMF8|GLDN\_MOUSE sp|Q91YQ1|RAB7L\_MOUSE tr|Q6IQX8|Q6IQX8\_MOUSE sp|Q9D5D8|CDYL2\_MOUSE sp|E9Q649|GCNT4\_MOUSE tr|F6Q739|F6Q739\_MOUSE tr|A2ATR8|A2ATR8\_MOUSE tr|G3UXA9|G3UXA9\_MOUSE tr|A0A087WPL4|A0A087WPL4\_MOUSE tr|Q9D3U9|Q9D3U9\_MOUSE tr|H3BJR8|H3BJR8\_MOUSE tr|D3Z0J5|D3Z0J5\_MOUSE sp|Q9QYC0-2|ADDA\_MOUSE tr|B1AVN8|B1AVN8\_MOUSE tr|F8WGR0|F8WGR0\_MOUSE tr|F8WHZ9|F8WHZ9\_MOUSE tr|E9Q1K3|E9Q1K3\_MOUSE sp|Q9QUI1|FA89B\_MOUSE tr|E9QAC6|E9QAC6\_MOUSE tr|D3Z2T4|D3Z2T4\_MOUSE tr|F6ZW42|F6ZW42\_MOUSE tr|V9GXR5|V9GXR5\_MOUSE tr|G3UZE8|G3UZE8\_MOUSE sp|P32115-2|PAX4\_MOUSE sp|P32115-3|PAX4\_MOUSE sp|Q99LG0|UBP16\_MOUSE sp|P32115|PAX4\_MOUSE tr|B7ZNC8|B7ZNC8\_MOUSE sp|Q3TTY0-2|PLB1\_MOUSE sp|Q8C031|LRC4C\_MOUSE sp|E9PZJ8-2|ASCC3\_MOUSE sp|Q9D2Q2|TRM44\_MOUSE sp|O35343|IMA3\_MOUSE tr|D3Z5E6|D3Z5E6\_MOUSE sp|Q61387|COX7R\_MOUSE tr|E9PZS8|E9PZS8\_MOUSE tr|D3Z518|D3Z518\_MOUSE sp|Q8C633|CABS1\_MOUSE sp|D3YVF0|AKAP5\_MOUSE sp|Q91VZ6|SMAP1\_MOUSE tr|Q80YW9|Q80YW9\_MOUSE tr|H3BIV5|H3BIV5\_MOUSE tr|D3YVX4|D3YVX4\_MOUSE sp|Q9EST8-2|IKBZ\_MOUSE sp|Q9EST8|IKBZ\_MOUSE sp|Q9EST8-3|IKBZ\_MOUSE sp|O08705|NTCP\_MOUSE tr|F6T4B0|F6T4B0\_MOUSE sp|Q8JZU2|TXTP\_MOUSE sp|O09113|OTP\_MOUSE sp|Q6VVW5-3|ANPRB\_MOUSE tr|D3YYJ6|D3YYJ6\_MOUSE sp|Q80YA9|CNKR2\_MOUSE sp|Q80YA9-2|CNKR2\_MOUSE sp|Q99K41|EMIL1\_MOUSE sp|P49070|CAMLG\_MOUSE sp|P23242|CXA1\_MOUSE tr|D6RH37|D6RH37\_MOUSE sp|Q810F8|TBX10\_MOUSE tr|A0JNU1|A0JNU1\_MOUSE tr|Q8BWG6|Q8BWG6\_MOUSE tr|E9Q4Q3|E9Q4Q3\_MOUSE sp|P70268-2|PKN1\_MOUSE sp|P70268|PKN1\_MOUSE tr|A2AK33|A2AK33\_MOUSE sp|Q03385|GNDS\_MOUSE tr|Q6QX36|Q6QX36\_MOUSE tr|Q3TWC1|Q3TWC1\_MOUSE sp|P63056-2|NOE3\_MOUSE tr|D6RJI6|D6RJI6\_MOUSE tr|D3YTM3|D3YTM3\_MOUSE sp|P63056|NOE3\_MOUSE sp|P54265-4|DMPK\_MOUSE sp|Q0VFX2|CI117\_MOUSE tr|L7N477|L7N477\_MOUSE tr|H3BKL4|H3BKL4\_MOUSE tr|Q9QXN7|Q9QXN7\_MOUSE sp|P09066|HME2\_MOUSE sp|Q91WD7|KI18A\_MOUSE tr|S4R1C6|S4R1C6\_MOUSE sp|Q8BYR5-2|CAPS2\_MOUSE tr|E9PVF7|E9PVF7\_MOUSE sp|Q8BYR5-4|CAPS2\_MOUSE tr|E9Q5C0|E9Q5C0\_MOUSE sp|Q8BYR5-3|CAPS2\_MOUSE tr|E9Q835|E9Q835\_MOUSE sp|Q8BYR5-6|CAPS2\_MOUSE sp|Q8BYR5|CAPS2\_MOUSE tr|F6QMQ3|F6QMQ3\_MOUSE sp|Q9CY62-2|RN181\_MOUSE tr|A0A087WPE1|A0A087WPE1\_MOUSE sp|Q9CY62|RN181\_MOUSE sp|O35658|C1QBP\_MOUSE sp|Q64299|NOV\_MOUSE sp|Q80YU0|ABHGB\_MOUSE tr|F6UDS6|F6UDS6\_MOUSE sp|Q6A044|F1891\_MOUSE sp|Q99ME2|WDR6\_MOUSE sp|Q8BMG7-2|RBGPR\_MOUSE sp|O88843|CRADD\_MOUSE sp|Q9DC26|S46A3\_MOUSE sp|Q504M8|RAB26\_MOUSE sp|Q91Y47|FA11\_MOUSE tr|F7AUV3|F7AUV3\_MOUSE tr|E0CZ46|E0CZ46\_MOUSE sp|Q925P2-2|CEAM2\_MOUSE sp|Q5DTL9-2|S4A10\_MOUSE sp|Q3UHB1|NT5D3\_MOUSE tr|F7C041|F7C041\_MOUSE tr|H3BIY7|H3BIY7\_MOUSE sp|Q8C3F7|KLH30\_MOUSE sp|Q5DTL9|S4A10\_MOUSE sp|Q925P2|CEAM2\_MOUSE sp|P97497|SHBG\_MOUSE tr|B1AWV9|B1AWV9\_MOUSE sp|Q91VB4|HPS3\_MOUSE tr|Q8VET4|Q8VET4\_MOUSE sp|P01027|CO3\_MOUSE sp|Q9JIF7|COPB\_MOUSE sp|Q8BML1-2|MICA2\_MOUSE tr|F6TLJ4|F6TLJ4\_MOUSE tr|F6WSV0|F6WSV0\_MOUSE sp|Q8BML1-3|MICA2\_MOUSE tr|F6YJ89|F6YJ89\_MOUSE sp|Q9CR81|TEX12\_MOUSE sp|Q921I1|TRFE\_MOUSE tr|F7CJN9|F7CJN9\_MOUSE sp|Q6P6P7|PARP6\_MOUSE sp|Q8BZ25|ANKK1\_MOUSE tr|E9Q035|E9Q035\_MOUSE tr|G5E856|G5E856\_MOUSE tr|Q9D0Z8|Q9D0Z8\_MOUSE sp|Q6P9S1|ATMIN\_MOUSE tr|F7BAE9|F7BAE9\_MOUSE sp|A2ANX9|ZN711\_MOUSE tr|E9PUP8|E9PUP8\_MOUSE tr|F8WGY3|F8WGY3\_MOUSE sp|Q6RI63|F120B\_MOUSE sp|Q62452|UD19\_MOUSE tr|Q91Y82|Q91Y82\_MOUSE sp|Q64446|ATP7B\_MOUSE sp|Q6RI63-2|F120B\_MOUSE sp|Q8BP31|RN122\_MOUSE tr|F6Z9X8|F6Z9X8\_MOUSE sp|Q8CHT1-2|NGEF\_MOUSE sp|O70361|PER3\_MOUSE sp|Q8QZW7|GBRP\_MOUSE sp|P23906|IRF2\_MOUSE sp|P52955|LBX1\_MOUSE tr|D3YWA9|D3YWA9\_MOUSE sp|Q9EQ28|DPOD3\_MOUSE sp|Q8C0V1|TERB1\_MOUSE sp|C4P6S0|SHTAP\_MOUSE sp|Q8C0V1-2|TERB1\_MOUSE tr|Q8BH76|Q8BH76\_MOUSE sp|Q8BP47|SYNC\_MOUSE Q2KJ62 sp|Q3UHC7-2|DAB2P\_MOUSE sp|Q3UHC7-3|DAB2P\_MOUSE sp|Q3UHC7-4|DAB2P\_MOUSE tr|F6RK07|F6RK07\_MOUSE tr|A2AUX5|A2AUX5\_MOUSE tr|Q9D5F6|Q9D5F6\_MOUSE tr|F6QQP6|F6QQP6\_MOUSE sp|Q3UHC7|DAB2P\_MOUSE tr|A2AUX3|A2AUX3\_MOUSE sp|Q8CAE2-2|HENMT\_MOUSE sp|P30275|KCRU\_MOUSE tr|Q8R1A8|Q8R1A8\_MOUSE sp|Q8C1Z8|TM10A\_MOUSE sp|Q8CAE2|HENMT\_MOUSE sp|P22272|IL6RA\_MOUSE sp|Q6P7W2|SHKB1\_MOUSE sp|Q6P7W2-2|SHKB1\_MOUSE sp|Q8C176|TAF2\_MOUSE sp|P51660|DHB4\_MOUSE tr|S4R2R1|S4R2R1\_MOUSE sp|O08650|HYAS3\_MOUSE sp|Q8BLE7|VGLU2\_MOUSE tr|A0A075B5R3|A0A075B5R3\_MOUSE sp|O88327|CTNL1\_MOUSE sp|P26361-3|CFTR\_MOUSE tr|Q7TSV0|Q7TSV0\_MOUSE tr|F6U9G7|F6U9G7\_MOUSE tr|Q69Z88|Q69Z88\_MOUSE sp|P26361-2|CFTR\_MOUSE sp|O88643|PAK1\_MOUSE sp|Q9CYC5|DSN1\_MOUSE tr|A2ATT9|A2ATT9\_MOUSE sp|Q9JLR1|S61A2\_MOUSE tr|Q3LR78|Q3LR78\_MOUSE sp|Q64441|CP24A\_MOUSE tr|F6Z595|F6Z595\_MOUSE sp|Q924C1-2|XPO5\_MOUSE sp|Q6PGN1|ERFE\_MOUSE sp|P16388|KCNA1\_MOUSE sp|P31809-2|CEAM1\_MOUSE sp|O09009|RFNG\_MOUSE tr|A2ATG3|A2ATG3\_MOUSE tr|Q5ND45|Q5ND45\_MOUSE sp|Q60750|EPHA1\_MOUSE sp|Q78IQ7|S39A4\_MOUSE tr|F6XXE6|F6XXE6\_MOUSE sp|Q78IQ7-2|S39A4\_MOUSE tr|G3UW97|G3UW97\_MOUSE sp|Q922M7|ASHWN\_MOUSE tr|Q8N7N6|Q8N7N6\_MOUSE sp|Q91WW5|MRGA1\_MOUSE tr|H3BKY4|H3BKY4\_MOUSE tr|F6TL54|F6TL54\_MOUSE tr|G3X972|G3X972\_MOUSE tr|Q4KL31|Q4KL31\_MOUSE tr|Q62056|Q62056\_MOUSE tr|B2RSG7|B2RSG7\_MOUSE tr|F7A0H7|F7A0H7\_MOUSE sp|Q8VHK5|MLC1\_MOUSE sp|Q8CFZ5|S22AC\_MOUSE sp|P41731|CD63\_MOUSE tr|E9QP87|E9QP87\_MOUSE tr|S4R2S1|S4R2S1\_MOUSE tr|F6XSA1|F6XSA1\_MOUSE tr|S4R2F4|S4R2F4\_MOUSE tr|E9Q9F5|E9Q9F5\_MOUSE sp|Q8VIM5-2|MYCD\_MOUSE sp|Q8VIM5-4|MYCD\_MOUSE tr|E9Q1G8|E9Q1G8\_MOUSE sp|O55131|SEPT7\_MOUSE sp|P16297|IL2RB\_MOUSE sp|Q8VEM9|KLDC3\_MOUSE sp|Q3U827|RN180\_MOUSE sp|Q3U827-3|RN180\_MOUSE sp|Q3U827-2|RN180\_MOUSE sp|Q3U827-4|RN180\_MOUSE tr|E9Q360|E9Q360\_MOUSE sp|Q91ZV8|GP124\_MOUSE tr|Q8BFT1|Q8BFT1\_MOUSE sp|Q9ER75|IRX6\_MOUSE tr|J3QMG7|J3QMG7\_MOUSE sp|Q9EPM5|SYNCI\_MOUSE tr|B1AQX9|B1AQX9\_MOUSE tr|B1AQX7|B1AQX7\_MOUSE tr|B1AQX6|B1AQX6\_MOUSE sp|Q8C3I8|HGH1\_MOUSE sp|Q9EPM5-3|SYNCI\_MOUSE sp|Q9JIS8|S12A4\_MOUSE tr|F8WIJ0|F8WIJ0\_MOUSE tr|G3UY21|G3UY21\_MOUSE tr|G3UXV6|G3UXV6\_MOUSE tr|E9Q824|E9Q824\_MOUSE tr|F6QKA8|F6QKA8\_MOUSE sp|Q5QD07|TAA8A\_MOUSE tr|G3UYX7|G3UYX7\_MOUSE tr|G3X909|G3X909\_MOUSE tr|E9Q3K3|E9Q3K3\_MOUSE sp|Q8BXK8|AGAP1\_MOUSE tr|G3UZ88|G3UZ88\_MOUSE sp|Q9R1B9|SLIT2\_MOUSE tr|A0A087WRF2|A0A087WRF2\_MOUSE tr|A2ABX0|A2ABX0\_MOUSE sp|Q99K82-3|SMOX\_MOUSE tr|E9Q549|E9Q549\_MOUSE sp|P97772|GRM1\_MOUSE tr|A2ANQ4|A2ANQ4\_MOUSE sp|Q99K82-4|SMOX\_MOUSE tr|Q8VGM9|Q8VGM9\_MOUSE tr|V9GXE5|V9GXE5\_MOUSE tr|D3Z0H9|D3Z0H9\_MOUSE tr|D3YWL2|D3YWL2\_MOUSE sp|Q6PFX8|RIMKA\_MOUSE sp|E9Q735|UBE4A\_MOUSE sp|Q9CZD5|IF3M\_MOUSE tr|E9Q1L2|E9Q1L2\_MOUSE sp|Q9JLN9-2|MTOR\_MOUSE tr|Q6PFE1|Q6PFE1\_MOUSE tr|E9QA66|E9QA66\_MOUSE sp|P15092|IFI4\_MOUSE tr|B1AUN3|B1AUN3\_MOUSE tr|E9Q1F5|E9Q1F5\_MOUSE sp|P15092-2|IFI4\_MOUSE tr|B1AUN2|B1AUN2\_MOUSE sp|Q6ZQA6|IGSF3\_MOUSE sp|Q3V050|S47A2\_MOUSE tr|Z4YMV2|Z4YMV2\_MOUSE sp|Q9EPK7-2|XPO7\_MOUSE sp|Q9R0C0-2|BL1S6\_MOUSE sp|Q9EPK7|XPO7\_MOUSE sp|Q9R0C0|BL1S6\_MOUSE tr|E9PUW7|E9PUW7\_MOUSE tr|Q8BJL9|Q8BJL9\_MOUSE tr|A0A087WQ01|A0A087WQ01\_MOUSE sp|P97412-2|LYST\_MOUSE sp|Q9DAM7|TM263\_MOUSE tr|D3Z3Q0|D3Z3Q0\_MOUSE tr|G3UX96|G3UX96\_MOUSE tr|G3UZ85|G3UZ85\_MOUSE tr|G3UXI3|G3UXI3\_MOUSE tr|G3UXH9|G3UXH9\_MOUSE tr|G3UYN2|G3UYN2\_MOUSE tr|Q6UAH0|Q6UAH0\_MOUSE tr|G3UXP7|G3UXP7\_MOUSE sp|O54942|CLD5\_MOUSE sp|Q99KP6-2|PRP19\_MOUSE sp|Q99KP6|PRP19\_MOUSE tr|F6X8H7|F6X8H7\_MOUSE sp|Q99KP6-3|PRP19\_MOUSE sp|B0V2N1-3|PTPRS\_MOUSE sp|B0V2N1-4|PTPRS\_MOUSE sp|P48168|GLRB\_MOUSE tr|G3UZF4|G3UZF4\_MOUSE tr|E9Q3E3|E9Q3E3\_MOUSE tr|E9QAF1|E9QAF1\_MOUSE tr|Q7TRH4|Q7TRH4\_MOUSE tr|D3YYY8|D3YYY8\_MOUSE sp|Q8R3J5|CHAC1\_MOUSE sp|P55096|ABCD3\_MOUSE sp|Q60967|PAPS1\_MOUSE tr|M0QWR8|M0QWR8\_MOUSE tr|L7N251|L7N251\_MOUSE tr|B1AVP0|B1AVP0\_MOUSE sp|Q9WUP1|RAMP3\_MOUSE tr|E9Q9N3|E9Q9N3\_MOUSE tr|B2RQT2|B2RQT2\_MOUSE sp|Q99K51|PLST\_MOUSE tr|L7N293|L7N293\_MOUSE sp|Q640L5-2|CCD18\_MOUSE tr|Q0VDY1|Q0VDY1\_MOUSE tr|B1AX58|B1AX58\_MOUSE tr|E9PUW2|E9PUW2\_MOUSE sp|Q9D2K4-2|IQCH\_MOUSE sp|Q9D2K4|IQCH\_MOUSE tr|B7ZNG8|B7ZNG8\_MOUSE tr|B2RUB3|B2RUB3\_MOUSE tr|F6XVP7|F6XVP7\_MOUSE tr|B8JJN0|B8JJN0\_MOUSE sp|Q9JHE3|ASAH2\_MOUSE sp|Q7TQP3|GP119\_MOUSE sp|O08750|NFIL3\_MOUSE tr|B8JJM9|B8JJM9\_MOUSE sp|Q8K2C6|SIR5\_MOUSE tr|Q3V1F1|Q3V1F1\_MOUSE tr|E9QB04|E9QB04\_MOUSE tr|E9PW68|E9PW68\_MOUSE tr|H3BKN0|H3BKN0\_MOUSE tr|B1AWQ0|B1AWQ0\_MOUSE sp|Q9ET47-5|ESPN\_MOUSE tr|E9PY08|E9PY08\_MOUSE sp|Q9ET47-7|ESPN\_MOUSE sp|Q3V3V9|LR16C\_MOUSE sp|Q9CZW2|CENPN\_MOUSE sp|Q99NG0|ARIP4\_MOUSE tr|B1AWP8|B1AWP8\_MOUSE tr|E9QKL0|E9QKL0\_MOUSE sp|Q9ET47-3|ESPN\_MOUSE sp|Q9ET47-2|ESPN\_MOUSE tr|B1AWP9|B1AWP9\_MOUSE sp|Q9ET47-4|ESPN\_MOUSE sp|Q9ET47-6|ESPN\_MOUSE tr|B1AWP7|B1AWP7\_MOUSE tr|D3Z6E7|D3Z6E7\_MOUSE tr|F8WHA0|F8WHA0\_MOUSE sp|Q5SSM3|RHG44\_MOUSE sp|Q9D2G5|SYNJ2\_MOUSE sp|Q8C8Y5|SAMD7\_MOUSE tr|F8WHD8|F8WHD8\_MOUSE tr|F6RUL9|F6RUL9\_MOUSE tr|A0A087WNQ8|A0A087WNQ8\_MOUSE sp|Q6PAV2-2|HERC4\_MOUSE sp|Q6PAV2|HERC4\_MOUSE tr|Q3V490|Q3V490\_MOUSE tr|Q8VFK4|Q8VFK4\_MOUSE tr|Q7TRW1|Q7TRW1\_MOUSE sp|Q9WVC8|S26A3\_MOUSE sp|Q9Z2V4|PCKGC\_MOUSE tr|A0A087WSB9|A0A087WSB9\_MOUSE tr|M0QWE6|M0QWE6\_MOUSE sp|Q9Z1P7|KANK3\_MOUSE sp|P49182|HEP2\_MOUSE tr|Q3UT66|Q3UT66\_MOUSE tr|G3UXN4|G3UXN4\_MOUSE sp|Q61180|SCNNA\_MOUSE tr|H3BKC4|H3BKC4\_MOUSE tr|H3BJC3|H3BJC3\_MOUSE tr|Q3USG4|Q3USG4\_MOUSE tr|H3BLI2|H3BLI2\_MOUSE tr|Q8BIV1|Q8BIV1\_MOUSE sp|O08848|RO60\_MOUSE tr|F6TM12|F6TM12\_MOUSE tr|Q6GQV2|Q6GQV2\_MOUSE tr|E9QAT3|E9QAT3\_MOUSE sp|Q8CE33-2|KLH11\_MOUSE sp|Q8CE33|KLH11\_MOUSE sp|Q80VM3|TTC29\_MOUSE tr|E9QLU4|E9QLU4\_MOUSE tr|G3UYM5|G3UYM5\_MOUSE sp|P57722|PCBP3\_MOUSE sp|Q9QYS2|GRM3\_MOUSE sp|P57722-2|PCBP3\_MOUSE tr|B2RY58|B2RY58\_MOUSE sp|O70507|HCN4\_MOUSE sp|Q8VCF5|TSN10\_MOUSE tr|Q3UGA5|Q3UGA5\_MOUSE sp|Q9CXV1|DHSD\_MOUSE tr|F7DBB3|F7DBB3\_MOUSE sp|P97298|PEDF\_MOUSE tr|D6RFQ2|D6RFQ2\_MOUSE sp|Q9QZK2-2|BCAR3\_MOUSE tr|Q9QWU6|Q9QWU6\_MOUSE sp|Q9CQ79|TXND9\_MOUSE tr|A2AP84|A2AP84\_MOUSE tr|G5E8M7|G5E8M7\_MOUSE tr|Q8C239|Q8C239\_MOUSE tr|A6XA72|A6XA72\_MOUSE tr|A0A087WQJ4|A0A087WQJ4\_MOUSE sp|Q01815-3|CAC1C\_MOUSE sp|Q01815-2|CAC1C\_MOUSE tr|A0A087WPP1|A0A087WPP1\_MOUSE tr|A0A087WRM3|A0A087WRM3\_MOUSE tr|A0A087WS67|A0A087WS67\_MOUSE tr|A0A087WPZ8|A0A087WPZ8\_MOUSE tr|A0A087WS40|A0A087WS40\_MOUSE tr|F7C376|F7C376\_MOUSE tr|C7TQ59|C7TQ59\_MOUSE tr|F8WJL1|F8WJL1\_MOUSE tr|A0A087WSE7|A0A087WSE7\_MOUSE tr|A0A087WQT9|A0A087WQT9\_MOUSE tr|A0A087WRZ6|A0A087WRZ6\_MOUSE sp|Q01815|CAC1C\_MOUSE tr|C7TQ57|C7TQ57\_MOUSE tr|Q0PCR6|Q0PCR6\_MOUSE tr|A0A087WR02|A0A087WR02\_MOUSE tr|Q91VN7|Q91VN7\_MOUSE tr|B8JJX9|B8JJX9\_MOUSE sp|Q60948|MAD4\_MOUSE tr|B8JJY0|B8JJY0\_MOUSE tr|Q91W94|Q91W94\_MOUSE sp|Q9JLT4-3|TRXR2\_MOUSE tr|Q3UZ22|Q3UZ22\_MOUSE tr|A0A075B617|A0A075B617\_MOUSE sp|Q8C120|SH3R3\_MOUSE sp|Q924N4|S12A6\_MOUSE sp|Q8C120-2|SH3R3\_MOUSE tr|Q6P6P5|Q6P6P5\_MOUSE sp|Q924N4-2|S12A6\_MOUSE tr|A2AGJ9|A2AGJ9\_MOUSE tr|Q3V0N8|Q3V0N8\_MOUSE tr|A0A075B653|A0A075B653\_MOUSE tr|F7DAY5|F7DAY5\_MOUSE sp|Q6KAU8|A16L2\_MOUSE tr|E9Q0E7|E9Q0E7\_MOUSE tr|D3Z653|D3Z653\_MOUSE tr|F6YB25|F6YB25\_MOUSE sp|Q9CYD3|CRTAP\_MOUSE sp|Q6KAU8-2|A16L2\_MOUSE tr|D6RH56|D6RH56\_MOUSE sp|Q8N7N5|DCAF8\_MOUSE tr|Q8C2R4|Q8C2R4\_MOUSE sp|Q91X86|TMM98\_MOUSE tr|D3YZT4|D3YZT4\_MOUSE sp|Q9DAP0|LRC46\_MOUSE sp|P23949|TISD\_MOUSE sp|Q9EPT5|SO2A1\_MOUSE tr|E9QKT1|E9QKT1\_MOUSE sp|O08759|UBE3A\_MOUSE tr|A0A087WNT2|A0A087WNT2\_MOUSE tr|Q80T54|Q80T54\_MOUSE sp|Q9DB34|CHM2A\_MOUSE sp|Q8R4H7|NAGS\_MOUSE tr|Q99JA5|Q99JA5\_MOUSE sp|Q80YE7-2|DAPK1\_MOUSE sp|Q80YE7|DAPK1\_MOUSE sp|Q3U3C9-4|GSE1\_MOUSE tr|A0A087WPY4|A0A087WPY4\_MOUSE sp|Q8R4H7-2|NAGS\_MOUSE tr|Q8BSH2|Q8BSH2\_MOUSE sp|Q80X89|UD2A1\_MOUSE tr|S4R2U5|S4R2U5\_MOUSE sp|Q9D7H3|RTCA\_MOUSE sp|Q8R0F3|SUMF1\_MOUSE tr|D6RD00|D6RD00\_MOUSE tr|E9Q9U6|E9Q9U6\_MOUSE sp|O08644-3|EPHB6\_MOUSE tr|D6REI7|D6REI7\_MOUSE tr|D3Z4Q1|D3Z4Q1\_MOUSE sp|O08644-2|EPHB6\_MOUSE sp|Q9Z1L3|DEDD\_MOUSE sp|Q9JKC7|AP4M1\_MOUSE tr|E9Q0Z7|E9Q0Z7\_MOUSE tr|E9Q416|E9Q416\_MOUSE tr|D3Z0F7|D3Z0F7\_MOUSE tr|V9GWW3|V9GWW3\_MOUSE tr|E9Q265|E9Q265\_MOUSE tr|D3Z0I1|D3Z0I1\_MOUSE sp|P02830|HXA7\_MOUSE tr|Q91VA7|Q91VA7\_MOUSE sp|Q8JZM2|PR15L\_MOUSE sp|Q9D3S3|SNX29\_MOUSE tr|E9Q709|E9Q709\_MOUSE sp|Q3URY6|ARMC2\_MOUSE sp|P97326|CADH6\_MOUSE sp|Q91ZU0|ASB7\_MOUSE sp|P30875|SSR2\_MOUSE tr|Q7TRA1|Q7TRA1\_MOUSE sp|P30875-2|SSR2\_MOUSE tr|Q7TRA2|Q7TRA2\_MOUSE tr|L7N2A0|L7N2A0\_MOUSE sp|P03995|GFAP\_MOUSE sp|P03995-2|GFAP\_MOUSE sp|Q9Z248-5|AEBP2\_MOUSE tr|Z4YLE4|Z4YLE4\_MOUSE sp|Q8CGT6|PIWL4\_MOUSE sp|Q8CGT6-3|PIWL4\_MOUSE sp|Q8CGT6-2|PIWL4\_MOUSE tr|D3Z4E6|D3Z4E6\_MOUSE sp|Q3ZK22|VEZA\_MOUSE sp|Q9CPP6|NDUA5\_MOUSE tr|D3YTQ8|D3YTQ8\_MOUSE sp|Q3ZK22-4|VEZA\_MOUSE sp|Q3ZK22-3|VEZA\_MOUSE tr|Q9EQA2|Q9EQA2\_MOUSE sp|Q6DFV6|FN3C1\_MOUSE sp|Q8CDI6-2|CD158\_MOUSE tr|J3QMS6|J3QMS6\_MOUSE sp|Q8C0Q9-3|RPGF5\_MOUSE sp|Q9CWL2-2|CASZ1\_MOUSE sp|Q9QYI7|DNJB8\_MOUSE sp|Q9D968|HCFC2\_MOUSE tr|F6T088|F6T088\_MOUSE sp|Q7TN29-2|SMAP2\_MOUSE sp|Q3V1G4|OLM2B\_MOUSE tr|B7ZBV4|B7ZBV4\_MOUSE tr|B7ZBV8|B7ZBV8\_MOUSE sp|Q9Z351|KCNQ2\_MOUSE sp|Q9Z351-3|KCNQ2\_MOUSE tr|F6U250|F6U250\_MOUSE tr|F6S419|F6S419\_MOUSE sp|Q9D6Z1|NOP56\_MOUSE tr|Q6PFF0|Q6PFF0\_MOUSE tr|E0CXL1|E0CXL1\_MOUSE tr|F6V095|F6V095\_MOUSE tr|E9QP73|E9QP73\_MOUSE sp|Q8CEF9|T132C\_MOUSE tr|Q7TSH6|Q7TSH6\_MOUSE tr|F7CHQ7|F7CHQ7\_MOUSE sp|Q8CE93|FA26D\_MOUSE tr|A2APD7|A2APD7\_MOUSE sp|Q9DA75|NLS1\_MOUSE sp|Q80XH4|WSCD1\_MOUSE tr|E9Q2E5|E9Q2E5\_MOUSE tr|Q6P8S1|Q6P8S1\_MOUSE sp|Q8BZX4|SREK1\_MOUSE tr|H3BK39|H3BK39\_MOUSE tr|A2AL79|A2AL79\_MOUSE sp|Q8VCC8|RPGF3\_MOUSE tr|Q3V0M1|Q3V0M1\_MOUSE sp|Q91Y74|SIA4C\_MOUSE tr|B7ZWJ3|B7ZWJ3\_MOUSE tr|Q3UU43|Q3UU43\_MOUSE tr|F6WLR1|F6WLR1\_MOUSE sp|Q9JHP7-3|KDEL1\_MOUSE sp|Q8VCC8-2|RPGF3\_MOUSE tr|F7CTY9|F7CTY9\_MOUSE tr|G5E8I2|G5E8I2\_MOUSE tr|D3Z7H2|D3Z7H2\_MOUSE sp|Q3TPJ7-2|MIDN\_MOUSE sp|Q3TPJ7|MIDN\_MOUSE sp|Q8C0X2-2|SL9B1\_MOUSE sp|Q3TPJ7-3|MIDN\_MOUSE sp|Q8C0N0|SMKZ\_MOUSE sp|Q8C0X2|SL9B1\_MOUSE tr|Q32P14|Q32P14\_MOUSE tr|F6VKC7|F6VKC7\_MOUSE tr|L7MUC9|L7MUC9\_MOUSE sp|Q9Z0E3-6|AIRE\_MOUSE sp|Q9Z0E3-5|AIRE\_MOUSE sp|P97321-3|SEPR\_MOUSE sp|Q9Z0E3-4|AIRE\_MOUSE sp|Q9Z0E3-7|AIRE\_MOUSE sp|Q61292|LAMB2\_MOUSE tr|G3UYG3|G3UYG3\_MOUSE sp|Q9Z0E3-2|AIRE\_MOUSE sp|Q9Z0E3|AIRE\_MOUSE sp|Q9Z0E3-3|AIRE\_MOUSE sp|Q9Z0E3-8|AIRE\_MOUSE sp|P97321-2|SEPR\_MOUSE sp|Q76KJ5|RPA34\_MOUSE tr|E9Q9B2|E9Q9B2\_MOUSE Q99PS0 tr|D3Z6D5|D3Z6D5\_MOUSE sp|Q99PS0|K1C23\_MOUSE sp|Q80ZI6|LRSM1\_MOUSE sp|Q8CDE2|CALI\_MOUSE sp|Q6TYB5|FEZ2\_MOUSE tr|F8VQG7|F8VQG7\_MOUSE sp|Q3UKU1|ELL2\_MOUSE tr|J3QPK0|J3QPK0\_MOUSE sp|Q9CZJ1|UTP11\_MOUSE tr|Q3V0A7|Q3V0A7\_MOUSE tr|Q8BUY2|Q8BUY2\_MOUSE sp|Q9CSP9|TTC14\_MOUSE sp|Q9CSP9-4|TTC14\_MOUSE tr|J3QPI0|J3QPI0\_MOUSE tr|H3BJ21|H3BJ21\_MOUSE sp|Q9QY14|FOXE3\_MOUSE sp|Q9JHL1-2|NHRF2\_MOUSE tr|D3Z3P4|D3Z3P4\_MOUSE tr|Q8BI58|Q8BI58\_MOUSE tr|J3QMY9|J3QMY9\_MOUSE tr|S4R266|S4R266\_MOUSE sp|P48962|ADT1\_MOUSE sp|O54928|SOCS5\_MOUSE tr|G3UY13|G3UY13\_MOUSE sp|Q9CY52|THG1\_MOUSE tr|Q9CQT0|Q9CQT0\_MOUSE sp|Q6WBX7|RAD9B\_MOUSE tr|D3Z3W7|D3Z3W7\_MOUSE sp|Q6P2K6|P4R3A\_MOUSE tr|E9QLK8|E9QLK8\_MOUSE tr|Q7TRR2|Q7TRR2\_MOUSE sp|Q810U2|TEDM1\_MOUSE tr|E9QAV0|E9QAV0\_MOUSE tr|E9Q481|E9Q481\_MOUSE tr|L7N224|L7N224\_MOUSE tr|D3Z420|D3Z420\_MOUSE sp|Q810S1|MCUB\_MOUSE sp|Q8CFV2|F149A\_MOUSE sp|P01865|GCAM\_MOUSE sp|P16882-2|GHR\_MOUSE sp|Q3UFY0|RRP36\_MOUSE tr|F6YWH6|F6YWH6\_MOUSE sp|Q08AU7|MADL2\_MOUSE tr|F6VRJ8|F6VRJ8\_MOUSE tr|E9QN41|E9QN41\_MOUSE sp|Q8C9H6-3|STRP2\_MOUSE sp|Q3TUA9|SG196\_MOUSE sp|Q05860-5|FMN1\_MOUSE sp|D3Z6S9|ERIP6\_MOUSE sp|Q68ED7|CRTC1\_MOUSE tr|F6RUA3|F6RUA3\_MOUSE sp|Q8C9H6-4|STRP2\_MOUSE tr|H3BJ70|H3BJ70\_MOUSE sp|Q8BT60|CPNE3\_MOUSE sp|Q8CJG1|AGO1\_MOUSE sp|Q91VD1-2|LEG12\_MOUSE sp|Q91VD1|LEG12\_MOUSE sp|Q9ER10|BSSP4\_MOUSE tr|Q7TML0|Q7TML0\_MOUSE sp|P68181-2|KAPCB\_MOUSE sp|Q61598-2|GDIB\_MOUSE sp|Q9QZD8|DIC\_MOUSE sp|Q8CHP8|PGP\_MOUSE tr|A0A087WRP2|A0A087WRP2\_MOUSE sp|Q8CIS0|CAR11\_MOUSE sp|P68181|KAPCB\_MOUSE sp|Q8CIS0-2|CAR11\_MOUSE tr|B1AYM9|B1AYM9\_MOUSE sp|P68181-3|KAPCB\_MOUSE sp|Q61598|GDIB\_MOUSE tr|E9Q5H3|E9Q5H3\_MOUSE tr|E9PVG9|E9PVG9\_MOUSE sp|O54799|NMBR\_MOUSE tr|L7MTY4|L7MTY4\_MOUSE tr|E9PY17|E9PY17\_MOUSE sp|Q9Z248-3|AEBP2\_MOUSE sp|Q9Z248-4|AEBP2\_MOUSE sp|Q8BJ71-2|NUP93\_MOUSE sp|Q8BJ71|NUP93\_MOUSE tr|K3W4L8|K3W4L8\_MOUSE tr|D3Z1C6|D3Z1C6\_MOUSE sp|P58069|RASA2\_MOUSE tr|Z4YJG5|Z4YJG5\_MOUSE tr|A2AM57|A2AM57\_MOUSE sp|Q9D1B8|DCD2C\_MOUSE sp|O89019-4|INVS\_MOUSE tr|F8WGQ1|F8WGQ1\_MOUSE sp|O89019|INVS\_MOUSE sp|P58390|KCNN2\_MOUSE sp|Q9D1B8-2|DCD2C\_MOUSE tr|B7ZCN3|B7ZCN3\_MOUSE sp|P01724|LV1B\_MOUSE sp|P01723|LV1A\_MOUSE sp|P01726|LV1D\_MOUSE tr|Q9EP55|Q9EP55\_MOUSE tr|Q9CQ76|Q9CQ76\_MOUSE tr|A0A075B663|A0A075B663\_MOUSE sp|P70288|HDAC2\_MOUSE tr|F7A0E9|F7A0E9\_MOUSE sp|Q8CFT2-2|SET1B\_MOUSE sp|Q9CQI3|GMFB\_MOUSE tr|D3YY93|D3YY93\_MOUSE sp|Q8CFT2|SET1B\_MOUSE sp|P07759|SPA3K\_MOUSE sp|P35689|ERCC5\_MOUSE sp|Q8JZR6-2|S4A8\_MOUSE sp|Q3TQF0|FBX31\_MOUSE tr|J3QQ46|J3QQ46\_MOUSE sp|Q8JZR6|S4A8\_MOUSE tr|D3YVY0|D3YVY0\_MOUSE sp|Q6PDM4|CCD36\_MOUSE tr|E9QM61|E9QM61\_MOUSE sp|O09174|AMACR\_MOUSE sp|Q99PJ1-8|PCD15\_MOUSE sp|Q99PJ1|PCD15\_MOUSE sp|Q99PJ1-4|PCD15\_MOUSE tr|E9Q159|E9Q159\_MOUSE sp|Q6ZQ73|CAND2\_MOUSE sp|Q99PJ1-9|PCD15\_MOUSE sp|P80315|TCPD\_MOUSE tr|G5E839|G5E839\_MOUSE sp|Q99PJ1-2|PCD15\_MOUSE sp|Q99PJ1-6|PCD15\_MOUSE sp|Q99PJ1-7|PCD15\_MOUSE sp|Q99PJ1-5|PCD15\_MOUSE sp|Q91WA1|TIPIN\_MOUSE sp|Q8R121-2|ZPI\_MOUSE tr|A5H0M4|A5H0M4\_MOUSE sp|Q8R121|ZPI\_MOUSE tr|F6X7B3|F6X7B3\_MOUSE sp|Q9Z1Z0-3|USO1\_MOUSE sp|Q80TA6|MTMRC\_MOUSE sp|Q80US8|MAD3\_MOUSE sp|Q80TA6-2|MTMRC\_MOUSE tr|D3Z234|D3Z234\_MOUSE sp|Q8BH49|SESQ1\_MOUSE tr|H3BJI3|H3BJI3\_MOUSE tr|F6RWQ6|F6RWQ6\_MOUSE sp|Q8CBC4-3|CNST\_MOUSE sp|Q9DBS4-2|ACOXL\_MOUSE sp|Q60766|IRGM1\_MOUSE sp|Q60766-2|IRGM1\_MOUSE tr|E9PXH9|E9PXH9\_MOUSE sp|Q8BGW4|DC122\_MOUSE sp|Q8CI51|PDLI5\_MOUSE tr|S4R220|S4R220\_MOUSE tr|A0A087WPM6|A0A087WPM6\_MOUSE tr|Q3UT37|Q3UT37\_MOUSE sp|Q8CI51-2|PDLI5\_MOUSE sp|O08677|KNG1\_MOUSE tr|D3YTY9|D3YTY9\_MOUSE sp|O08677-3|KNG1\_MOUSE sp|Q8BH48|UBAP1\_MOUSE sp|Q8BH48-2|UBAP1\_MOUSE sp|P20662|ZFY2\_MOUSE tr|H3BJF7|H3BJF7\_MOUSE tr|Q9CWD9|Q9CWD9\_MOUSE sp|P33434|MMP2\_MOUSE tr|F6V243|F6V243\_MOUSE sp|P33434-2|MMP2\_MOUSE sp|Q5NBU5|FBX39\_MOUSE tr|G3UYP4|G3UYP4\_MOUSE sp|Q3U8K7-4|SV421\_MOUSE tr|J9JIB2|J9JIB2\_MOUSE sp|Q3U8K7-2|SV421\_MOUSE tr|D6RG71|D6RG71\_MOUSE tr|H3BLE5|H3BLE5\_MOUSE sp|Q9DB30|PHKG2\_MOUSE sp|P34152-9|FAK1\_MOUSE tr|A0A087WP46|A0A087WP46\_MOUSE sp|Q5SXJ3|FANCJ\_MOUSE tr|F6RWW8|F6RWW8\_MOUSE sp|Q9Z1Q5|CLIC1\_MOUSE Q05443 sp|Q5FW53|MBPHL\_MOUSE sp|Q8CDK3|IQUB\_MOUSE sp|Q8BGS2-2|BOLA2\_MOUSE sp|Q8BGS2|BOLA2\_MOUSE sp|A3KGB4|TBC8B\_MOUSE tr|S4R213|S4R213\_MOUSE sp|Q9JLY7|DUS14\_MOUSE tr|Q8VG65|Q8VG65\_MOUSE tr|G3X8X3|G3X8X3\_MOUSE sp|A2A995|CA168\_MOUSE tr|G3UXZ6|G3UXZ6\_MOUSE tr|G3UY95|G3UY95\_MOUSE tr|E9PZ08|E9PZ08\_MOUSE sp|Q8BSM7|LAT3\_MOUSE sp|Q8CCK0|H2AW\_MOUSE sp|Q80VW5-6|WHRN\_MOUSE tr|A2ATS4|A2ATS4\_MOUSE sp|P54615|OSTR\_MOUSE sp|Q9ESX5|DKC1\_MOUSE sp|Q8BK72|RT27\_MOUSE tr|F6UIK0|F6UIK0\_MOUSE tr|H3BKQ7|H3BKQ7\_MOUSE sp|P86547|OSTC2\_MOUSE tr|Q3UXW4|Q3UXW4\_MOUSE sp|Q8BGM5|BEST2\_MOUSE tr|H3BJD0|H3BJD0\_MOUSE tr|H3BJA6|H3BJA6\_MOUSE sp|P86546|OSTCN\_MOUSE tr|Q7TN74|Q7TN74\_MOUSE sp|Q99LL5|PWP1\_MOUSE tr|F8WJ82|F8WJ82\_MOUSE tr|H3BJD6|H3BJD6\_MOUSE tr|H3BL28|H3BL28\_MOUSE sp|Q62264|THRSP\_MOUSE tr|E9Q9U2|E9Q9U2\_MOUSE sp|Q8BZ97|PRDM8\_MOUSE sp|Q3USZ2|UBFL1\_MOUSE tr|B2RU90|B2RU90\_MOUSE sp|P35413|GPR3\_MOUSE tr|E9QNS0|E9QNS0\_MOUSE sp|Q91WS2|NALP6\_MOUSE tr|F6TC98|F6TC98\_MOUSE sp|Q91WS2-2|NALP6\_MOUSE tr|F8UKQ6|F8UKQ6\_MOUSE tr|E9Q1U2|E9Q1U2\_MOUSE sp|Q91YE8|SYNP2\_MOUSE sp|Q8VC97|BUP1\_MOUSE tr|D3YVV9|D3YVV9\_MOUSE tr|D3YWJ3|D3YWJ3\_MOUSE sp|Q7TNV0|DEK\_MOUSE sp|P25444|RS2\_MOUSE sp|Q14B80|KCNC2\_MOUSE tr|E9Q4P1|E9Q4P1\_MOUSE tr|Q6PGC9|Q6PGC9\_MOUSE sp|Q80Y55|BSDC1\_MOUSE tr|A2AVM0|A2AVM0\_MOUSE tr|E9Q8Y1|E9Q8Y1\_MOUSE tr|D3YVJ6|D3YVJ6\_MOUSE tr|D6RFA5|D6RFA5\_MOUSE tr|D3YVC1|D3YVC1\_MOUSE tr|H3BJK3|H3BJK3\_MOUSE tr|D6REH0|D6REH0\_MOUSE tr|D6RDB7|D6RDB7\_MOUSE tr|D3Z768|D3Z768\_MOUSE sp|P0C6A2|MAMD1\_MOUSE tr|F8WHF9|F8WHF9\_MOUSE tr|A6X922|A6X922\_MOUSE tr|F6X9C5|F6X9C5\_MOUSE sp|Q8BZP8|NPSR1\_MOUSE sp|P42228|STAT4\_MOUSE tr|D3Z2Y1|D3Z2Y1\_MOUSE tr|Q3V157|Q3V157\_MOUSE sp|Q8R2X8|GO45\_MOUSE sp|Q08351|TPOR\_MOUSE sp|Q8R2X8-2|GO45\_MOUSE tr|L7N465|L7N465\_MOUSE sp|Q61114|BPIB1\_MOUSE sp|Q7TPQ3-3|SHPRH\_MOUSE sp|Q9D9H3|MB3L1\_MOUSE tr|F7CHR4|F7CHR4\_MOUSE tr|B2GVN8|B2GVN8\_MOUSE tr|A6PWW6|A6PWW6\_MOUSE sp|Q9WUK4|RFC2\_MOUSE sp|Q8VGK5|OLF50\_MOUSE tr|B2FDE2|B2FDE2\_MOUSE tr|G5E912|G5E912\_MOUSE tr|F6R3T1|F6R3T1\_MOUSE tr|H3BL14|H3BL14\_MOUSE tr|H3BKL2|H3BKL2\_MOUSE sp|Q3SXC5|ARL14\_MOUSE sp|Q8JZN7|MIRO2\_MOUSE sp|Q9R0N8|SYT6\_MOUSE sp|P63085|MK01\_MOUSE sp|Q6ZPS2|CRNS1\_MOUSE sp|Q6ZPS2-2|CRNS1\_MOUSE sp|Q9R0N8-2|SYT6\_MOUSE tr|A2AQ92|A2AQ92\_MOUSE tr|Q3UY13|Q3UY13\_MOUSE tr|F6VEI7|F6VEI7\_MOUSE sp|Q80SU4|IFNAD\_MOUSE tr|Q8C8S6|Q8C8S6\_MOUSE tr|B1AYH5|B1AYH5\_MOUSE tr|E9PX09|E9PX09\_MOUSE tr|Q8CIU5|Q8CIU5\_MOUSE tr|Q8CIU6|Q8CIU6\_MOUSE tr|I7HPV7|I7HPV7\_MOUSE sp|Q61839|ANFC\_MOUSE sp|Q9EQR3-2|KCNN1\_MOUSE tr|Q8CIU3|Q8CIU3\_MOUSE tr|Q9EQY5|Q9EQY5\_MOUSE sp|Q3UUQ7|PGAP1\_MOUSE tr|A2AK51|A2AK51\_MOUSE tr|Q6XBG2|Q6XBG2\_MOUSE tr|E9PWH4|E9PWH4\_MOUSE sp|A7XUZ6|SKIT6\_MOUSE tr|J3QMS2|J3QMS2\_MOUSE sp|P38532-2|HSF1\_MOUSE sp|P38532|HSF1\_MOUSE sp|A7XUZ6-2|SKIT6\_MOUSE sp|Q3ULZ2|FHDC1\_MOUSE sp|Q3UVD5|LGR6\_MOUSE tr|D3Z6S4|D3Z6S4\_MOUSE tr|B1AWD1|B1AWD1\_MOUSE tr|Q9QZU3|Q9QZU3\_MOUSE tr|A2AJQ2|A2AJQ2\_MOUSE sp|O08742|GPV\_MOUSE sp|Q8K045|PKN3\_MOUSE tr|Q8VFX3|Q8VFX3\_MOUSE sp|O88508|DNM3A\_MOUSE sp|Q8BG16|S6A15\_MOUSE tr|D3Z748|D3Z748\_MOUSE sp|O88508-2|DNM3A\_MOUSE sp|Q64695|EPCR\_MOUSE sp|D3YUB6|BTBD8\_MOUSE tr|Q8R568|Q8R568\_MOUSE sp|A2A6Q5-2|CDC27\_MOUSE sp|A2A6Q5-3|CDC27\_MOUSE sp|P27106|MIS\_MOUSE sp|Q64519|SDC3\_MOUSE sp|Q8R507|FKTN\_MOUSE tr|D6RCG1|D6RCG1\_MOUSE tr|F6UXV2|F6UXV2\_MOUSE sp|Q64737-2|PUR2\_MOUSE sp|P56404|AQP8\_MOUSE sp|Q3TTY0-4|PLB1\_MOUSE tr|Q8VF80|Q8VF80\_MOUSE tr|B0R058|B0R058\_MOUSE sp|Q8BH75|RNF41\_MOUSE sp|Q8C0L9-2|GPCP1\_MOUSE tr|A3KFV6|A3KFV6\_MOUSE tr|Q14DT9|Q14DT9\_MOUSE sp|Q921T2|TOIP1\_MOUSE sp|P58406|HRH3\_MOUSE tr|K7N749|K7N749\_MOUSE tr|E9Q522|E9Q522\_MOUSE tr|Z4YLG9|Z4YLG9\_MOUSE sp|Q921T2-2|TOIP1\_MOUSE sp|Q921T2-3|TOIP1\_MOUSE sp|Q9ER88-2|RT29\_MOUSE sp|Q9ER88|RT29\_MOUSE tr|E9Q540|E9Q540\_MOUSE tr|G3UWZ6|G3UWZ6\_MOUSE tr|G3UWM7|G3UWM7\_MOUSE tr|E9Q5S3|E9Q5S3\_MOUSE tr|D3YYC9|D3YYC9\_MOUSE tr|F6YC07|F6YC07\_MOUSE tr|G3X9M0|G3X9M0\_MOUSE tr|E9Q7T5|E9Q7T5\_MOUSE tr|E9Q292|E9Q292\_MOUSE tr|D3Z5Q8|D3Z5Q8\_MOUSE sp|Q6ZPG2-2|WDR90\_MOUSE sp|Q64302|T4S1\_MOUSE tr|F8VPL4|F8VPL4\_MOUSE sp|Q7M717|TR102\_MOUSE sp|Q8VHI5-2|VITRN\_MOUSE sp|Q8C2K5-2|RASL3\_MOUSE tr|D3Z6Z7|D3Z6Z7\_MOUSE sp|Q8C2K5|RASL3\_MOUSE sp|Q9DBI0-3|TMPS6\_MOUSE sp|Q9DBI0|TMPS6\_MOUSE sp|P26618-2|PGFRA\_MOUSE sp|Q9DBI0-2|TMPS6\_MOUSE sp|Q3UPI1|F198B\_MOUSE tr|B1ATZ1|B1ATZ1\_MOUSE tr|Q9D4E6|Q9D4E6\_MOUSE tr|K7N741|K7N741\_MOUSE tr|B1ATZ0|B1ATZ0\_MOUSE tr|B1AXB9|B1AXB9\_MOUSE tr|B5B2R5|B5B2R5\_MOUSE sp|Q9D321|S35A4\_MOUSE tr|A2AQC8|A2AQC8\_MOUSE sp|Q66JX5-2|FR1OP\_MOUSE sp|B9EKI3|TMF1\_MOUSE tr|D6RJK7|D6RJK7\_MOUSE sp|Q9D6W8|CQ059\_MOUSE sp|Q8BL06-3|UBP54\_MOUSE tr|A0A087WPG6|A0A087WPG6\_MOUSE tr|A2AI78|A2AI78\_MOUSE tr|Q6XPS7|Q6XPS7\_MOUSE tr|A0A075B5T4|A0A075B5T4\_MOUSE sp|Q7M718|TR124\_MOUSE tr|J3QPW4|J3QPW4\_MOUSE sp|Q0VBD2|MCM10\_MOUSE tr|D3YWX9|D3YWX9\_MOUSE tr|D3YU13|D3YU13\_MOUSE sp|Q9D5U8-2|CNBD2\_MOUSE sp|Q9D5U8|CNBD2\_MOUSE sp|Q8CII9-2|CRLF2\_MOUSE sp|Q8CII9-3|CRLF2\_MOUSE sp|Q8BFR5-2|EFTU\_MOUSE sp|Q8BFR5|EFTU\_MOUSE tr|D3YVN7|D3YVN7\_MOUSE sp|Q8CII9|CRLF2\_MOUSE sp|Q8CDZ2|MT21E\_MOUSE sp|P11404|FABPH\_MOUSE sp|Q0VF94|NKPD1\_MOUSE tr|H7BX48|H7BX48\_MOUSE tr|E9Q6Z7|E9Q6Z7\_MOUSE tr|Q7TR87|Q7TR87\_MOUSE sp|Q8BGV4|TTI2\_MOUSE sp|Q3TLI0|TPC10\_MOUSE sp|P70277|SIA7B\_MOUSE tr|F8VQF9|F8VQF9\_MOUSE tr|Q8K1G9|Q8K1G9\_MOUSE tr|A2AIJ1|A2AIJ1\_MOUSE sp|Q8BWR2-2|PITH1\_MOUSE tr|E0CY79|E0CY79\_MOUSE tr|A2AIJ2|A2AIJ2\_MOUSE tr|A2ACQ1|A2ACQ1\_MOUSE tr|A2AIJ3|A2AIJ3\_MOUSE tr|B1AV57|B1AV57\_MOUSE sp|Q8K0Z7|TACO1\_MOUSE tr|K7N649|K7N649\_MOUSE tr|B1AV58|B1AV58\_MOUSE sp|P40237|CD82\_MOUSE tr|A2ACP9|A2ACP9\_MOUSE sp|Q8BWR2|PITH1\_MOUSE sp|Q8BUH8|SENP7\_MOUSE tr|E9Q1L5|E9Q1L5\_MOUSE tr|A2AQC9|A2AQC9\_MOUSE sp|Q9D0P5|PYGO1\_MOUSE sp|Q9D1I2|BINCA\_MOUSE tr|A6PW46|A6PW46\_MOUSE tr|Q8C8Y3|Q8C8Y3\_MOUSE sp|Q99K74-3|MED24\_MOUSE sp|Q99P31|HPBP1\_MOUSE tr|E9Q2U5|E9Q2U5\_MOUSE tr|Q8VFQ3|Q8VFQ3\_MOUSE tr|L7N2A4|L7N2A4\_MOUSE sp|P68404|KPCB\_MOUSE sp|P68404-2|KPCB\_MOUSE sp|Q60997-2|DMBT1\_MOUSE sp|Q60997|DMBT1\_MOUSE sp|Q60997-3|DMBT1\_MOUSE tr|E9QPG8|E9QPG8\_MOUSE sp|Q60997-4|DMBT1\_MOUSE sp|Q3V132|ADT4\_MOUSE tr|Q91WA8|Q91WA8\_MOUSE sp|Q8K1X1|WDR11\_MOUSE tr|B7ZNY3|B7ZNY3\_MOUSE tr|G5E8J3|G5E8J3\_MOUSE tr|Q8BIC7|Q8BIC7\_MOUSE tr|Q497V9|Q497V9\_MOUSE sp|Q60857|SC6A4\_MOUSE tr|E9PZW7|E9PZW7\_MOUSE sp|Q3USH1|F196A\_MOUSE sp|Q91X88|PMGT1\_MOUSE sp|Q9DAS4|PR8A8\_MOUSE tr|S4R265|S4R265\_MOUSE tr|Q5NC63|Q5NC63\_MOUSE sp|Q8BMD8|SCMC1\_MOUSE sp|A2RTL5|RSRC2\_MOUSE sp|Q91X88-3|PMGT1\_MOUSE sp|Q91X88-2|PMGT1\_MOUSE sp|Q02242|PDCD1\_MOUSE sp|Q9DAS4-2|PR8A8\_MOUSE tr|S4R2L4|S4R2L4\_MOUSE tr|S4R1M5|S4R1M5\_MOUSE tr|E9QAW0|E9QAW0\_MOUSE sp|O54956|DPOE2\_MOUSE sp|F7BWT7-2|TSN15\_MOUSE sp|F7BWT7|TSN15\_MOUSE tr|E9Q281|E9Q281\_MOUSE sp|Q3U9N9-2|MOT10\_MOUSE sp|Q3U9N9|MOT10\_MOUSE sp|Q6P9R4|ARHGI\_MOUSE sp|Q9D483|RPC3\_MOUSE tr|Q8CDC9|Q8CDC9\_MOUSE sp|Q923W1|TGS1\_MOUSE tr|J3QP10|J3QP10\_MOUSE sp|Q6P9R4-2|ARHGI\_MOUSE sp|Q9JIG7|CCD22\_MOUSE sp|P60766|CDC42\_MOUSE sp|P70278|STRA8\_MOUSE tr|F2Z435|F2Z435\_MOUSE tr|A9C445|A9C445\_MOUSE sp|Q9WVC6-3|SGK1\_MOUSE sp|Q8C0Z1|ITFG3\_MOUSE sp|Q3U410|KLH21\_MOUSE sp|P62071|RRAS2\_MOUSE sp|Q7TML3|S35F2\_MOUSE sp|Q61112|CAB45\_MOUSE tr|E0CXD0|E0CXD0\_MOUSE tr|D3Z2D9|D3Z2D9\_MOUSE tr|Q8C7D3|Q8C7D3\_MOUSE sp|P97334|NKX23\_MOUSE sp|P59511|ATS20\_MOUSE sp|Q8R361|RFIP5\_MOUSE sp|Q7TSH4|CP110\_MOUSE tr|G3UWP9|G3UWP9\_MOUSE sp|Q9R157|ADA18\_MOUSE sp|Q80XJ2|RN168\_MOUSE tr|Q8R3X6|Q8R3X6\_MOUSE sp|Q9R087|GPC6\_MOUSE tr|E9PYW4|E9PYW4\_MOUSE tr|E0CXS1|E0CXS1\_MOUSE sp|Q9R1X4-6|TIM\_MOUSE sp|Q61288|ACVL1\_MOUSE sp|Q9R1X4-2|TIM\_MOUSE sp|Q99J79|DDB2\_MOUSE sp|Q9R1X4-3|TIM\_MOUSE sp|Q9R1X4|TIM\_MOUSE sp|Q8VC16|LRC14\_MOUSE tr|K7N684|K7N684\_MOUSE sp|Q8BQZ8|KCNS3\_MOUSE tr|L7MU98|L7MU98\_MOUSE sp|Q8BGE5-3|FANCM\_MOUSE sp|Q8K2P6|RFESD\_MOUSE tr|A0A087WR69|A0A087WR69\_MOUSE sp|Q9WVL6|EXTL3\_MOUSE tr|H3BL83|H3BL83\_MOUSE sp|Q3UHU5-2|MTCL1\_MOUSE tr|B8JJI7|B8JJI7\_MOUSE sp|P51885|LUM\_MOUSE tr|Q6P1H4|Q6P1H4\_MOUSE sp|Q80WJ1|GGN\_MOUSE tr|G5E8M4|G5E8M4\_MOUSE sp|A2AHC3-2|CAMP1\_MOUSE tr|D3YY79|D3YY79\_MOUSE tr|D3YWW4|D3YWW4\_MOUSE tr|D3Z4Q6|D3Z4Q6\_MOUSE tr|D3YVX1|D3YVX1\_MOUSE tr|D3Z2C6|D3Z2C6\_MOUSE tr|D3YYH6|D3YYH6\_MOUSE tr|D3YVY4|D3YVY4\_MOUSE tr|D3YWW2|D3YWW2\_MOUSE tr|D3Z266|D3Z266\_MOUSE tr|O70518|O70518\_MOUSE tr|A2AHC4|A2AHC4\_MOUSE sp|P26369|U2AF2\_MOUSE tr|D3YXV6|D3YXV6\_MOUSE sp|P84102|SERF2\_MOUSE sp|Q80W49-3|CRBG3\_MOUSE sp|P53762|ARNT\_MOUSE tr|B1ATU5|B1ATU5\_MOUSE sp|Q8C2P3|DUS1L\_MOUSE sp|Q80TB8|VAT1L\_MOUSE tr|Q3ULM2|Q3ULM2\_MOUSE tr|E9QLT6|E9QLT6\_MOUSE tr|D3Z434|D3Z434\_MOUSE sp|P53762-2|ARNT\_MOUSE sp|Q80W49|CRBG3\_MOUSE sp|Q80W49-2|CRBG3\_MOUSE tr|Q8VGP3|Q8VGP3\_MOUSE tr|Q7TQT2|Q7TQT2\_MOUSE sp|A2AWP0|BIRC7\_MOUSE sp|Q6DFV1|CNDG2\_MOUSE tr|Q3UAM9|Q3UAM9\_MOUSE sp|O70423-2|AOC3\_MOUSE tr|G3X8Z7|G3X8Z7\_MOUSE sp|O35253|SMAD7\_MOUSE sp|Q7TQA4|TR140\_MOUSE tr|D3YXW7|D3YXW7\_MOUSE tr|G3UX42|G3UX42\_MOUSE tr|F7BY36|F7BY36\_MOUSE sp|O35253-2|SMAD7\_MOUSE sp|O70423|AOC3\_MOUSE tr|G3UXH8|G3UXH8\_MOUSE sp|P03940|CP21A\_MOUSE sp|Q9CZV5|ST65G\_MOUSE sp|Q8BIQ3|ZNF2\_MOUSE sp|Q9D0M3|CY1\_MOUSE tr|D3Z6Y8|D3Z6Y8\_MOUSE sp|O54941|SMCE1\_MOUSE sp|P19096|FAS\_MOUSE sp|P47857-2|PFKAM\_MOUSE sp|P47857-3|PFKAM\_MOUSE sp|Q7TNS7|ASIC4\_MOUSE sp|Q3UX62|CC114\_MOUSE sp|Q8C078-4|KKCC2\_MOUSE tr|D3YYS9|D3YYS9\_MOUSE sp|P47857|PFKAM\_MOUSE tr|D3Z6V5|D3Z6V5\_MOUSE tr|H3BKX1|H3BKX1\_MOUSE tr|F6XQ35|F6XQ35\_MOUSE tr|Q8VCS9|Q8VCS9\_MOUSE sp|Q8BGF8|S35D3\_MOUSE sp|P46938-2|YAP1\_MOUSE sp|P46938|YAP1\_MOUSE tr|G3UYV4|G3UYV4\_MOUSE tr|G3UY62|G3UY62\_MOUSE tr|G3UYA6|G3UYA6\_MOUSE tr|D3Z362|D3Z362\_MOUSE sp|P83917|CBX1\_MOUSE tr|A2BGJ5|A2BGJ5\_MOUSE tr|Q925T4|Q925T4\_MOUSE tr|Q7TPM0|Q7TPM0\_MOUSE tr|D3YU28|D3YU28\_MOUSE tr|F6YR19|F6YR19\_MOUSE sp|P30730|LSHR\_MOUSE tr|Q9CYJ8|Q9CYJ8\_MOUSE sp|Q8BZK4|S35F4\_MOUSE sp|Q8BZK4-2|S35F4\_MOUSE sp|Q99N03|M4A10\_MOUSE sp|P0C027|NUD10\_MOUSE tr|F7CW61|F7CW61\_MOUSE sp|Q9D7B1|DUS2L\_MOUSE tr|A0A087WRZ0|A0A087WRZ0\_MOUSE sp|P0C028|NUD11\_MOUSE sp|Q99N03-2|M4A10\_MOUSE sp|Q6PCQ0|IQCE\_MOUSE sp|Q8CJ67-2|STAU2\_MOUSE sp|Q8CJ67|STAU2\_MOUSE tr|E9QPS5|E9QPS5\_MOUSE sp|Q8K4G1-3|LTBP4\_MOUSE tr|B2RX70|B2RX70\_MOUSE sp|Q9WVL0|MAAI\_MOUSE tr|F6T4L3|F6T4L3\_MOUSE tr|E9Q7L8|E9Q7L8\_MOUSE sp|Q8K4G1|LTBP4\_MOUSE sp|P20033-2|PDGFA\_MOUSE tr|V9GX57|V9GX57\_MOUSE tr|Q99L56|Q99L56\_MOUSE sp|P17439|GLCM\_MOUSE tr|G5E891|G5E891\_MOUSE tr|Q3UT49|Q3UT49\_MOUSE tr|V9GXF6|V9GXF6\_MOUSE sp|P20033|PDGFA\_MOUSE sp|Q6PFD6|KI18B\_MOUSE tr|E9PV07|E9PV07\_MOUSE tr|D3Z1J5|D3Z1J5\_MOUSE sp|Q80VF6-2|F181B\_MOUSE sp|Q9EPU5|TNR21\_MOUSE sp|Q9EPR2-2|PG12A\_MOUSE sp|Q9EPR2|PG12A\_MOUSE tr|E9PZ95|E9PZ95\_MOUSE tr|E9PVI7|E9PVI7\_MOUSE tr|E9Q2P3|E9Q2P3\_MOUSE tr|E9PWX4|E9PWX4\_MOUSE tr|S4R2N6|S4R2N6\_MOUSE sp|Q9CZ04-2|CSN7A\_MOUSE tr|Q3TJB1|Q3TJB1\_MOUSE sp|B9EKE5|CATIP\_MOUSE sp|P50586|TUB\_MOUSE sp|Q8CGI1|F193A\_MOUSE sp|B9EKE5-2|CATIP\_MOUSE sp|P50586-2|TUB\_MOUSE sp|Q3UU35|OVOS\_MOUSE sp|Q9ES28-6|ARHG7\_MOUSE sp|Q9ES28-4|ARHG7\_MOUSE sp|Q9ES28-7|ARHG7\_MOUSE sp|Q3UGM2|NEK10\_MOUSE tr|J3JSA1|J3JSA1\_MOUSE sp|Q91W20|LRC26\_MOUSE sp|Q9DB25|ALG5\_MOUSE tr|G5E8B9|G5E8B9\_MOUSE sp|Q91ZE5|EMR4\_MOUSE sp|P21129|P3\_MOUSE tr|K3W4R8|K3W4R8\_MOUSE tr|E9QMK6|E9QMK6\_MOUSE sp|P17710|HXK1\_MOUSE sp|Q99K46|UBP11\_MOUSE tr|G3X8V5|G3X8V5\_MOUSE sp|P17710-4|HXK1\_MOUSE sp|Q8K2Y0|RN219\_MOUSE sp|P17710-2|HXK1\_MOUSE tr|G3UVV4|G3UVV4\_MOUSE sp|Q8K0H5|TAF10\_MOUSE sp|B9EKX1|PTHD4\_MOUSE sp|P17710-3|HXK1\_MOUSE sp|Q60987|FOXG1\_MOUSE sp|Q9ERD8|PARVG\_MOUSE tr|Q91Y06|Q91Y06\_MOUSE tr|E9PYG5|E9PYG5\_MOUSE sp|Q8BL66|EEA1\_MOUSE tr|Q921N8|Q921N8\_MOUSE tr|B1AU89|B1AU89\_MOUSE sp|Q810B8|SLIK4\_MOUSE sp|Q4VA53-2|PDS5B\_MOUSE tr|Q8VET1|Q8VET1\_MOUSE sp|P51945|CCNG1\_MOUSE sp|Q9QXK8|NMU\_MOUSE sp|Q8VHK8-2|TM11D\_MOUSE tr|G3UX03|G3UX03\_MOUSE tr|A2AEV5|A2AEV5\_MOUSE tr|Q9D350|Q9D350\_MOUSE tr|F6TVX7|F6TVX7\_MOUSE sp|Q6P5B0|RRP12\_MOUSE sp|O35316|SC6A6\_MOUSE sp|Q9EQI8|RM46\_MOUSE sp|Q8VHK8|TM11D\_MOUSE sp|Q921V7|CTL3\_MOUSE sp|Q5RJI4|PKDCC\_MOUSE sp|Q6IMB1|RSLBA\_MOUSE tr|P70305|P70305\_MOUSE sp|Q8R422|CD109\_MOUSE sp|Q99KC8|VMA5A\_MOUSE sp|O70324|MOT8\_MOUSE tr|D3Z2F0|D3Z2F0\_MOUSE sp|Q1EHW4|SAP25\_MOUSE tr|Q7TR25|Q7TR25\_MOUSE sp|A2AGH6-3|MED12\_MOUSE sp|A2ABV5-4|MED14\_MOUSE sp|A2ABV5-3|MED14\_MOUSE sp|Q9JKT3|TA2R4\_MOUSE sp|Q8R1F0|L10K\_MOUSE sp|Q00342|FLT3\_MOUSE sp|P10166|MYCL\_MOUSE sp|Q8BR93-3|HARB1\_MOUSE sp|Q60778|IKBB\_MOUSE sp|Q8C1A5|THOP1\_MOUSE sp|Q3U7R1-2|ESYT1\_MOUSE sp|Q8K3P5-2|CNOT6\_MOUSE sp|Q8K064|F174B\_MOUSE sp|Q8K3P5|CNOT6\_MOUSE sp|Q9JJD0|THA11\_MOUSE sp|Q8BX13|UBE3D\_MOUSE tr|V9GX00|V9GX00\_MOUSE tr|Q14AD7|Q14AD7\_MOUSE tr|E9PXX0|E9PXX0\_MOUSE tr|A6PW97|A6PW97\_MOUSE tr|F2Z401|F2Z401\_MOUSE sp|Q8BH06-3|I17RE\_MOUSE sp|Q3UVL4-2|VPS51\_MOUSE sp|Q8BH06-2|I17RE\_MOUSE sp|Q8BYR2|LATS1\_MOUSE sp|Q99LI5|ZN281\_MOUSE sp|Q8K070|SAM14\_MOUSE tr|F2Z489|F2Z489\_MOUSE tr|H3BJJ5|H3BJJ5\_MOUSE tr|H3BKB1|H3BKB1\_MOUSE tr|S4R1Y5|S4R1Y5\_MOUSE sp|Q7TN33|CELF6\_MOUSE tr|D3YU11|D3YU11\_MOUSE sp|Q7TN33-2|CELF6\_MOUSE tr|Q3V0K6|Q3V0K6\_MOUSE sp|Q8BQU7|TMM74\_MOUSE sp|Q9JJ80|RPF2\_MOUSE tr|H3BJY0|H3BJY0\_MOUSE tr|G3X926|G3X926\_MOUSE tr|G3X9Z2|G3X9Z2\_MOUSE tr|Q9D3R8|Q9D3R8\_MOUSE tr|F6SPK0|F6SPK0\_MOUSE sp|Q61085|RHPN1\_MOUSE tr|E9Q7Q7|E9Q7Q7\_MOUSE tr|B2RV89|B2RV89\_MOUSE sp|Q9JJG9|NOA1\_MOUSE sp|Q01721|GAS1\_MOUSE tr|Q5GAN1|Q5GAN1\_MOUSE sp|Q9QY23-2|PKP3\_MOUSE sp|Q9QY23|PKP3\_MOUSE tr|Q3URQ4|Q3URQ4\_MOUSE sp|Q9JKF7|RM39\_MOUSE sp|Q8C850|SCAR3\_MOUSE tr|E9QAH2|E9QAH2\_MOUSE tr|B7ZCM1|B7ZCM1\_MOUSE tr|E9PZ07|E9PZ07\_MOUSE tr|G3X970|G3X970\_MOUSE tr|E9PVX4|E9PVX4\_MOUSE tr|V9GXL1|V9GXL1\_MOUSE sp|P34968|5HT2C\_MOUSE sp|P23150|HA2J\_MOUSE tr|E9Q2Y4|E9Q2Y4\_MOUSE sp|Q7TN98-4|CPEB4\_MOUSE tr|A3KGW0|A3KGW0\_MOUSE sp|A3KGV1-6|ODFP2\_MOUSE sp|Q7TN98-3|CPEB4\_MOUSE sp|A3KGV1-7|ODFP2\_MOUSE tr|V9GXZ0|V9GXZ0\_MOUSE sp|A3KGV1-5|ODFP2\_MOUSE tr|A3KGV9|A3KGV9\_MOUSE sp|Q99J38|T30A1\_MOUSE sp|Q61646|HPT\_MOUSE sp|Q6NXW6|RAD17\_MOUSE sp|Q7TSQ1-2|CL18A\_MOUSE tr|A0A087WPI3|A0A087WPI3\_MOUSE sp|P97438-2|KCNK2\_MOUSE tr|H3BKQ2|H3BKQ2\_MOUSE sp|P24549|AL1A1\_MOUSE sp|Q9CQI4|CK074\_MOUSE sp|Q9D8M4|RL7L\_MOUSE tr|A0A087WQD7|A0A087WQD7\_MOUSE tr|A0A087WRY6|A0A087WRY6\_MOUSE tr|Q80XE3|Q80XE3\_MOUSE sp|Q925S4|IL24\_MOUSE tr|A0A087WQW6|A0A087WQW6\_MOUSE sp|Q8BG89-2|ZN365\_MOUSE tr|I7HPX7|I7HPX7\_MOUSE tr|D6RIQ2|D6RIQ2\_MOUSE sp|O88844|IDHC\_MOUSE tr|A0A087WRS9|A0A087WRS9\_MOUSE tr|D3YVY3|D3YVY3\_MOUSE sp|Q6PAQ9|TMG3\_MOUSE sp|Q9JHU3|CDK20\_MOUSE sp|Q8BG89|ZN365\_MOUSE tr|A0A087WRM4|A0A087WRM4\_MOUSE tr|A0A087WPT4|A0A087WPT4\_MOUSE tr|Q99N18|Q99N18\_MOUSE tr|Q8VCA4|Q8VCA4\_MOUSE tr|I7HFT9|I7HFT9\_MOUSE tr|F6RTS1|F6RTS1\_MOUSE sp|Q07133|H1T\_MOUSE sp|Q8BX80|ENASE\_MOUSE tr|F6ZTU1|F6ZTU1\_MOUSE tr|F7CXI4|F7CXI4\_MOUSE sp|Q61624|ZN148\_MOUSE sp|Q9R1L5|MAST1\_MOUSE sp|Q3TP92|CNEP1\_MOUSE tr|A0A075B5L0|A0A075B5L0\_MOUSE sp|O88796|RPP30\_MOUSE sp|Q8BLY3|LRFN3\_MOUSE sp|Q9JJE4|PAQR4\_MOUSE tr|D3Z6Z3|D3Z6Z3\_MOUSE tr|F6TN13|F6TN13\_MOUSE tr|F6WBW4|F6WBW4\_MOUSE tr|E9PYQ6|E9PYQ6\_MOUSE sp|P24815|3BHS1\_MOUSE sp|P18419|SVS4\_MOUSE tr|F7DDW0|F7DDW0\_MOUSE tr|D3Z119|D3Z119\_MOUSE tr|Q9CUJ5|Q9CUJ5\_MOUSE sp|Q8CDP0-3|CBPC3\_MOUSE tr|Q3U422|Q3U422\_MOUSE sp|Q8CDP0-2|CBPC3\_MOUSE sp|Q8CDP0|CBPC3\_MOUSE tr|D3Z3L4|D3Z3L4\_MOUSE sp|Q99MU3-5|DSRAD\_MOUSE tr|F6UNQ3|F6UNQ3\_MOUSE sp|Q99MU3-2|DSRAD\_MOUSE sp|P19091|ANDR\_MOUSE sp|Q99MU3|DSRAD\_MOUSE tr|D3YYJ4|D3YYJ4\_MOUSE sp|P59438-3|HPS5\_MOUSE sp|Q9CZ49|KLH35\_MOUSE tr|H7BX06|H7BX06\_MOUSE sp|Q8K2T1|NMRL1\_MOUSE sp|Q8K2T1-2|NMRL1\_MOUSE tr|D3YU12|D3YU12\_MOUSE sp|Q9D8V0-2|HM13\_MOUSE tr|D3Z1N2|D3Z1N2\_MOUSE sp|Q3UIW5|RNF10\_MOUSE tr|Q6PGJ8|Q6PGJ8\_MOUSE sp|O08677-2|KNG1\_MOUSE tr|F6YU83|F6YU83\_MOUSE sp|P52734|FGD1\_MOUSE sp|Q80UU9|PGRC2\_MOUSE sp|Q9DBC0|SELO\_MOUSE sp|Q924X2|CPT1B\_MOUSE sp|Q8BHA3|DTD2\_MOUSE sp|Q8BHA3-2|DTD2\_MOUSE tr|Q8BMV6|Q8BMV6\_MOUSE sp|Q8VCZ3|THAP7\_MOUSE tr|C3VD30|C3VD30\_MOUSE tr|A2A550|A2A550\_MOUSE tr|Q8C799|Q8C799\_MOUSE sp|Q8K212|PACS1\_MOUSE sp|O70622|RTN2\_MOUSE sp|P97499|TEP1\_MOUSE tr|Q7TS04|Q7TS04\_MOUSE sp|O70622-2|RTN2\_MOUSE tr|E0CYW7|E0CYW7\_MOUSE sp|Q8VHJ7|PRGC2\_MOUSE sp|P18572|BASI\_MOUSE sp|Q6ZQF0|TOPB1\_MOUSE tr|E9PVC2|E9PVC2\_MOUSE sp|P45448|NR5A2\_MOUSE sp|O88746-2|TOM1\_MOUSE tr|Q8VCL4|Q8VCL4\_MOUSE sp|Q9CZW2-3|CENPN\_MOUSE sp|Q8BKC5-2|IPO5\_MOUSE tr|A2RTS9|A2RTS9\_MOUSE sp|Q9CQF9|PCYOX\_MOUSE tr|D3Z275|D3Z275\_MOUSE tr|F6ZDJ1|F6ZDJ1\_MOUSE sp|Q7TQA5|T2R39\_MOUSE tr|Q3ULK5|Q3ULK5\_MOUSE sp|Q8BKC5|IPO5\_MOUSE tr|J3QMJ1|J3QMJ1\_MOUSE sp|Q6XQH0|G3ST2\_MOUSE sp|Q8BWY3|ERF1\_MOUSE tr|D3YW25|D3YW25\_MOUSE sp|Q6PEM6|GRAM3\_MOUSE tr|G3UX37|G3UX37\_MOUSE tr|Q7TNE6|Q7TNE6\_MOUSE tr|Q9D2W5|Q9D2W5\_MOUSE sp|Q3TIR1|TPC13\_MOUSE tr|J3QNW4|J3QNW4\_MOUSE tr|H3BK45|H3BK45\_MOUSE tr|F8WHN2|F8WHN2\_MOUSE sp|Q3TIR1-3|TPC13\_MOUSE sp|Q3TIR1-2|TPC13\_MOUSE tr|E9Q9P2|E9Q9P2\_MOUSE sp|Q8VBT9-3|ASPC1\_MOUSE tr|E9PUM5|E9PUM5\_MOUSE tr|A2ABZ8|A2ABZ8\_MOUSE tr|A2AC05|A2AC05\_MOUSE sp|Q8VBT9-2|ASPC1\_MOUSE sp|Q8VBT9|ASPC1\_MOUSE tr|E9Q8B6|E9Q8B6\_MOUSE sp|Q8BFW7-4|LPP\_MOUSE tr|A2AC02|A2AC02\_MOUSE tr|F6Z9H5|F6Z9H5\_MOUSE tr|F7CRQ2|F7CRQ2\_MOUSE tr|E9Q8B5|E9Q8B5\_MOUSE tr|A2AC03|A2AC03\_MOUSE sp|P54987|IRG1\_MOUSE sp|Q3U1U7|AHRR\_MOUSE tr|D3Z7M3|D3Z7M3\_MOUSE tr|E9Q4H1|E9Q4H1\_MOUSE sp|Q3U1U7-2|AHRR\_MOUSE sp|P48318|DCE1\_MOUSE sp|Q3U285|LCORL\_MOUSE sp|Q3U285-2|LCORL\_MOUSE tr|A0A075B686|A0A075B686\_MOUSE sp|Q9D7Y9-3|SLX4I\_MOUSE tr|A0A075B687|A0A075B687\_MOUSE sp|Q64311|NTAN1\_MOUSE tr|A2AHF0|A2AHF0\_MOUSE tr|F6SS03|F6SS03\_MOUSE tr|D3YZ99|D3YZ99\_MOUSE sp|Q9D7Y9-2|SLX4I\_MOUSE sp|E9QMW4-2|CP096\_MOUSE tr|H3BLN9|H3BLN9\_MOUSE tr|A2A412|A2A412\_MOUSE sp|P63015|PAX6\_MOUSE sp|P63015-2|PAX6\_MOUSE sp|P63015-3|PAX6\_MOUSE tr|H3BL23|H3BL23\_MOUSE tr|Q8VFZ6|Q8VFZ6\_MOUSE tr|A2AMV1|A2AMV1\_MOUSE tr|H3BKX9|H3BKX9\_MOUSE sp|Q6GQX2|NCK5L\_MOUSE tr|Q7TRU4|Q7TRU4\_MOUSE tr|Q8VEW1|Q8VEW1\_MOUSE tr|H3BJB5|H3BJB5\_MOUSE tr|E9PXF3|E9PXF3\_MOUSE sp|Q68G58|APEX2\_MOUSE tr|A2AFM3|A2AFM3\_MOUSE tr|A2AFM4|A2AFM4\_MOUSE sp|Q68G58-2|APEX2\_MOUSE sp|O70146|TESK1\_MOUSE sp|Q569E4|MENT\_MOUSE sp|Q9EPQ1|TLR1\_MOUSE tr|D3YUZ7|D3YUZ7\_MOUSE tr|Q91Y01|Q91Y01\_MOUSE tr|A2AWL3|A2AWL3\_MOUSE tr|Q7TRD6|Q7TRD6\_MOUSE sp|A2RSY6-3|TRM1L\_MOUSE sp|P62325|BTG1\_MOUSE tr|V9GX28|V9GX28\_MOUSE tr|A0A087WQ23|A0A087WQ23\_MOUSE tr|E9PWI8|E9PWI8\_MOUSE sp|Q99N99|S5A2\_MOUSE sp|Q9CZ82|MED18\_MOUSE tr|F8VQL6|F8VQL6\_MOUSE tr|F7BJB9|F7BJB9\_MOUSE sp|Q921B4|NRIF2\_MOUSE sp|Q9CUL5-2|IQCA1\_MOUSE tr|G5E869|G5E869\_MOUSE tr|Q3V1C1|Q3V1C1\_MOUSE sp|P97431|IRF6\_MOUSE sp|Q9D0Z3|TMM53\_MOUSE tr|G3UZX5|G3UZX5\_MOUSE sp|P15533-2|TR30A\_MOUSE sp|P15533|TR30A\_MOUSE sp|Q9D0Z3-2|TMM53\_MOUSE sp|Q50L43|PA24D\_MOUSE sp|Q9D1Z2|CK091\_MOUSE sp|Q9D8Y7|TP8L2\_MOUSE sp|Q45KJ6-2|LN28B\_MOUSE tr|E9QAP8|E9QAP8\_MOUSE sp|Q0P557-3|MIEAP\_MOUSE sp|Q45KJ6|LN28B\_MOUSE tr|J3QPR5|J3QPR5\_MOUSE sp|Q45KJ6-4|LN28B\_MOUSE sp|Q0P557|MIEAP\_MOUSE sp|Q9DCD5|TJAP1\_MOUSE sp|B1AQL3|TM235\_MOUSE sp|Q8K0F1|TBC23\_MOUSE sp|Q0P557-2|MIEAP\_MOUSE sp|Q9CQX5|CLDN1\_MOUSE tr|D3Z5G0|D3Z5G0\_MOUSE tr|A0A087WPX3|A0A087WPX3\_MOUSE sp|O88512|AP1G2\_MOUSE sp|Q810U3|NFASC\_MOUSE tr|Q7TRB8|Q7TRB8\_MOUSE tr|A2ASK1|A2ASK1\_MOUSE tr|Q9EQ99|Q9EQ99\_MOUSE tr|Q7TRB7|Q7TRB7\_MOUSE sp|Q7TS68|NSUN6\_MOUSE tr|Q9EQ98|Q9EQ98\_MOUSE tr|Q9EQA0|Q9EQA0\_MOUSE sp|Q7TQP4|RL3R2\_MOUSE sp|Q6A078-2|CE290\_MOUSE sp|Q5RJH6-3|SMG7\_MOUSE sp|Q5RJH6|SMG7\_MOUSE sp|Q5RJH6-2|SMG7\_MOUSE tr|Z4YJK8|Z4YJK8\_MOUSE sp|Q61183-2|PAPOA\_MOUSE tr|E9QAY4|E9QAY4\_MOUSE tr|V9GXT8|V9GXT8\_MOUSE sp|A4Q9F3|TTL10\_MOUSE sp|A4Q9F3-2|TTL10\_MOUSE tr|D3YU68|D3YU68\_MOUSE tr|B1ASM0|B1ASM0\_MOUSE sp|Q3TDQ1|STT3B\_MOUSE sp|Q2PFD7-4|PSD3\_MOUSE tr|S4R264|S4R264\_MOUSE sp|Q8BZ64|TECT1\_MOUSE tr|D6RDQ2|D6RDQ2\_MOUSE tr|S4R1Q9|S4R1Q9\_MOUSE tr|Q6P8Y2|Q6P8Y2\_MOUSE sp|Q99P87|RETN\_MOUSE sp|P97494|GSH1\_MOUSE sp|Q570Y9-2|DPTOR\_MOUSE sp|Q04859-2|MAK\_MOUSE tr|F8VQ01|F8VQ01\_MOUSE sp|Q69ZB8|ZCHC2\_MOUSE tr|F6SJC5|F6SJC5\_MOUSE tr|H3BJ48|H3BJ48\_MOUSE sp|Q4KWH5-5|PLCH1\_MOUSE sp|P11798|KCC2A\_MOUSE sp|Q69ZB8-2|ZCHC2\_MOUSE tr|H3BJM4|H3BJM4\_MOUSE sp|O70283|WNT2B\_MOUSE tr|Q8VGK6|Q8VGK6\_MOUSE sp|Q91VV4-2|DEN2D\_MOUSE sp|P23607|ZFA\_MOUSE sp|Q91VV4|DEN2D\_MOUSE tr|A2A653|A2A653\_MOUSE tr|A0A087WRZ9|A0A087WRZ9\_MOUSE tr|S4R219|S4R219\_MOUSE sp|Q8R3N2|MFS6L\_MOUSE tr|G3UXN1|G3UXN1\_MOUSE tr|D3YUE2|D3YUE2\_MOUSE sp|Q61398|PCOC1\_MOUSE Q2HJF0 tr|Q9JI81|Q9JI81\_MOUSE tr|D3Z7D5|D3Z7D5\_MOUSE sp|Q91VL9|ZBTB1\_MOUSE sp|Q02788|CO6A2\_MOUSE tr|D3YY85|D3YY85\_MOUSE tr|D3YY86|D3YY86\_MOUSE sp|Q8R5C8|ZMY11\_MOUSE tr|Q8BJZ0|Q8BJZ0\_MOUSE tr|D3YXX1|D3YXX1\_MOUSE tr|D3Z6K9|D3Z6K9\_MOUSE tr|D3YYD0|D3YYD0\_MOUSE sp|Q3UZZ4|OLFM4\_MOUSE tr|L7N2C1|L7N2C1\_MOUSE tr|A8VI09|A8VI09\_MOUSE tr|M0QWJ8|M0QWJ8\_MOUSE tr|L7N208|L7N208\_MOUSE sp|Q8BJA2|S41A1\_MOUSE sp|O08791|COE3\_MOUSE tr|L7N452|L7N452\_MOUSE tr|F6SP87|F6SP87\_MOUSE tr|D3Z2W4|D3Z2W4\_MOUSE tr|A2ART2|A2ART2\_MOUSE tr|Q08BU3|Q08BU3\_MOUSE sp|O55074|AKA7A\_MOUSE P02676 sp|Q5SV77-4|GGNB2\_MOUSE tr|H7BX83|H7BX83\_MOUSE sp|Q5SV77|GGNB2\_MOUSE tr|D3YUG3|D3YUG3\_MOUSE sp|Q8C0C4|CCSE1\_MOUSE sp|Q9CZX8|RS19\_MOUSE tr|D3YUT3|D3YUT3\_MOUSE tr|J3QK23|J3QK23\_MOUSE tr|D3Z5R8|D3Z5R8\_MOUSE tr|B2RQS1|B2RQS1\_MOUSE sp|Q9ERG2|STRN3\_MOUSE sp|P30561|AHR\_MOUSE tr|Q3U5D9|Q3U5D9\_MOUSE sp|Q8K368|FANCI\_MOUSE tr|D3YX57|D3YX57\_MOUSE tr|Q8K076|Q8K076\_MOUSE tr|F6SH36|F6SH36\_MOUSE tr|G3UYV1|G3UYV1\_MOUSE tr|Q5SS55|Q5SS55\_MOUSE sp|Q3UHD2|GFOD1\_MOUSE tr|Q7TRN0|Q7TRN0\_MOUSE sp|Q3TL44|NLRX1\_MOUSE sp|Q9WUH5|TRI10\_MOUSE sp|Q3UM29|COG7\_MOUSE tr|E9Q4K2|E9Q4K2\_MOUSE tr|Q60607|Q60607\_MOUSE sp|P36895|BMR1A\_MOUSE sp|Q64310|SURF4\_MOUSE tr|A0A087WQ26|A0A087WQ26\_MOUSE tr|D3Z4V0|D3Z4V0\_MOUSE tr|D3Z6J2|D3Z6J2\_MOUSE tr|D3YWR3|D3YWR3\_MOUSE tr|D3YU23|D3YU23\_MOUSE tr|E0CYD2|E0CYD2\_MOUSE tr|D3YWB1|D3YWB1\_MOUSE tr|Q8BI23|Q8BI23\_MOUSE sp|P58801|RIPK2\_MOUSE sp|P22366|MYD88\_MOUSE tr|S4R299|S4R299\_MOUSE tr|B2RR24|B2RR24\_MOUSE tr|A6X8J1|A6X8J1\_MOUSE tr|J3QNE4|J3QNE4\_MOUSE tr|Q9D515|Q9D515\_MOUSE tr|E9Q3G2|E9Q3G2\_MOUSE tr|A2A6N9|A2A6N9\_MOUSE sp|Q3UHD6-2|SNX27\_MOUSE tr|E9PZT7|E9PZT7\_MOUSE sp|Q80W69-2|CL071\_MOUSE sp|Q8K1S2|UNC5D\_MOUSE tr|E9PZN4|E9PZN4\_MOUSE tr|H3BK33|H3BK33\_MOUSE tr|F6QNN0|F6QNN0\_MOUSE sp|Q3B7Z2-2|OSBP1\_MOUSE sp|Q80W69|CL071\_MOUSE sp|Q8VEC4|CAHM2\_MOUSE sp|Q3UHD6|SNX27\_MOUSE sp|Q64444|CAH4\_MOUSE tr|Q497K6|Q497K6\_MOUSE tr|E9QAQ2|E9QAQ2\_MOUSE tr|D3Z2E8|D3Z2E8\_MOUSE tr|E9Q9S7|E9Q9S7\_MOUSE tr|E9Q283|E9Q283\_MOUSE sp|Q8QZR7|PDK1L\_MOUSE sp|Q8CDF7|EXD1\_MOUSE tr|A2A9L3|A2A9L3\_MOUSE sp|Q9D3P8|PLRKT\_MOUSE tr|B1B1A4|B1B1A4\_MOUSE sp|Q9D8I2|P2Y13\_MOUSE sp|Q9ESF4|LFG1\_MOUSE sp|O08970|TUFT1\_MOUSE tr|Q3TB04|Q3TB04\_MOUSE sp|Q8BGQ1-2|SPE39\_MOUSE sp|Q80ZS3|RT26\_MOUSE sp|Q5F297|S35G3\_MOUSE sp|Q8BIE6-2|FRM4A\_MOUSE sp|O35423|SPYA\_MOUSE sp|Q8BIE6|FRM4A\_MOUSE sp|Q3TUL7-3|DCA17\_MOUSE tr|E9Q6S2|E9Q6S2\_MOUSE tr|Q8C5U0|Q8C5U0\_MOUSE sp|O35423-2|SPYA\_MOUSE sp|Q9QUT0|RHAG\_MOUSE tr|H3BIZ7|H3BIZ7\_MOUSE tr|H3BKV0|H3BKV0\_MOUSE sp|Q8VDY9|CAAP1\_MOUSE sp|Q8BGQ1|SPE39\_MOUSE tr|B2RQF0|B2RQF0\_MOUSE sp|Q05722|CO9A1\_MOUSE tr|A2AV96|A2AV96\_MOUSE tr|G3X995|G3X995\_MOUSE P06868 tr|B1ASX6|B1ASX6\_MOUSE sp|O35936|ALOX8\_MOUSE tr|E9QLW0|E9QLW0\_MOUSE sp|Q8CHT3|INT5\_MOUSE sp|Q91X56|S1PR5\_MOUSE sp|Q32NZ6-2|TMC5\_MOUSE sp|Q9R0H2-4|MUCEN\_MOUSE sp|Q9R0H2-2|MUCEN\_MOUSE tr|A2AWK4|A2AWK4\_MOUSE tr|F6RST1|F6RST1\_MOUSE sp|Q8CI03-2|FWCH1\_MOUSE sp|Q8CI03|FWCH1\_MOUSE sp|Q8CI03-3|FWCH1\_MOUSE tr|D6RG78|D6RG78\_MOUSE sp|Q5JCT0|GCNT3\_MOUSE tr|D3Z3T8|D3Z3T8\_MOUSE tr|Q7TS07|Q7TS07\_MOUSE sp|Q8CDP0-4|CBPC3\_MOUSE sp|Q8JZS6|N42L2\_MOUSE sp|Q3TYD4|ARSG\_MOUSE sp|Q64378|FKBP5\_MOUSE sp|O35593|PSDE\_MOUSE tr|H3BK58|H3BK58\_MOUSE tr|B8JJC2|B8JJC2\_MOUSE sp|Q9R1S8|CAN7\_MOUSE tr|F2Z3X0|F2Z3X0\_MOUSE tr|H3BJA9|H3BJA9\_MOUSE tr|F6Z4J2|F6Z4J2\_MOUSE tr|F7DD37|F7DD37\_MOUSE tr|Q8VG46|Q8VG46\_MOUSE tr|E9PUE6|E9PUE6\_MOUSE tr|G3X9W4|G3X9W4\_MOUSE sp|Q99K24|S39A3\_MOUSE sp|Q61572|FOXC1\_MOUSE sp|Q99ML4|FA69B\_MOUSE sp|Q9JI11|STK4\_MOUSE tr|J3QMB8|J3QMB8\_MOUSE sp|A2AED3|FNDC7\_MOUSE tr|Q3ZAR9|Q3ZAR9\_MOUSE sp|P49117|NR2C2\_MOUSE sp|Q3UMW7-2|MAPK3\_MOUSE sp|Q3UMW7-3|MAPK3\_MOUSE tr|H3BKZ0|H3BKZ0\_MOUSE tr|D3YU53|D3YU53\_MOUSE sp|A2AED3-4|FNDC7\_MOUSE tr|Q7TR71|Q7TR71\_MOUSE tr|Q8BM41|Q8BM41\_MOUSE tr|F6YIR2|F6YIR2\_MOUSE tr|J3QNA8|J3QNA8\_MOUSE tr|D3YUK2|D3YUK2\_MOUSE tr|G5E872|G5E872\_MOUSE sp|Q6NS65|GPR17\_MOUSE sp|Q8BZ98-2|DYN3\_MOUSE tr|E9QLL2|E9QLL2\_MOUSE sp|Q8BZ98|DYN3\_MOUSE tr|F8VQE2|F8VQE2\_MOUSE sp|Q9QXL1|KI21B\_MOUSE tr|E9Q0A4|E9Q0A4\_MOUSE sp|P10078-2|ZFP28\_MOUSE tr|Q8C9T1|Q8C9T1\_MOUSE tr|E9PZ00|E9PZ00\_MOUSE sp|P41155|TNFC\_MOUSE tr|Q8BFQ1|Q8BFQ1\_MOUSE sp|Q9WTR5|CAD13\_MOUSE sp|P10078|ZFP28\_MOUSE tr|G3V002|G3V002\_MOUSE sp|Q61207|SAP\_MOUSE tr|Q3UTB2|Q3UTB2\_MOUSE sp|Q9Z0L4|BMP15\_MOUSE tr|J3QPG5|J3QPG5\_MOUSE sp|Q91YD9|WASL\_MOUSE tr|K3W4L3|K3W4L3\_MOUSE tr|E9PUN1|E9PUN1\_MOUSE tr|A2AI92|A2AI92\_MOUSE tr|B1AX35|B1AX35\_MOUSE tr|Q91UZ1|Q91UZ1\_MOUSE sp|Q8C1Z7|BBS4\_MOUSE sp|P37238-2|PPARG\_MOUSE sp|Q8BHC9|FUT11\_MOUSE sp|Q3UPL0-3|SC31A\_MOUSE sp|P37238|PPARG\_MOUSE sp|Q9CWU9|NUP37\_MOUSE tr|D3Z0B9|D3Z0B9\_MOUSE tr|H3BKR4|H3BKR4\_MOUSE tr|F6W207|F6W207\_MOUSE tr|F8WI30|F8WI30\_MOUSE sp|Q9JMD1|SMBT1\_MOUSE sp|Q9QZS2|RNF4\_MOUSE sp|P35922-10|FMR1\_MOUSE tr|J3QPB5|J3QPB5\_MOUSE tr|H3BKS9|H3BKS9\_MOUSE sp|Q6A051-3|ATRN1\_MOUSE tr|V9GX62|V9GX62\_MOUSE sp|P35922-4|FMR1\_MOUSE sp|Q6A051|ATRN1\_MOUSE tr|A2A723|A2A723\_MOUSE tr|S4R2C9|S4R2C9\_MOUSE sp|Q5FW52|MLIP\_MOUSE tr|F7D3B8|F7D3B8\_MOUSE sp|Q5SXY1-2|CYTSB\_MOUSE tr|F6RSR4|F6RSR4\_MOUSE sp|Q9D162-3|CC167\_MOUSE P01044-1 sp|Q9Z0Y7|IRS4\_MOUSE P01045-1 sp|P97466|NOGG\_MOUSE tr|I6L890|I6L890\_MOUSE Q6NTB9 tr|G3UWM2|G3UWM2\_MOUSE tr|D3YV60|D3YV60\_MOUSE sp|Q6IE25|CASPG\_MOUSE sp|Q9CQ01|RNT2\_MOUSE sp|Q6Y5D8|RHG10\_MOUSE sp|O08540|MSMB\_MOUSE O76009 sp|Q6Y5D8-2|RHG10\_MOUSE tr|E9PY84|E9PY84\_MOUSE sp|Q7TNH6-2|NPHP3\_MOUSE sp|Q9CZH8|CCD77\_MOUSE tr|F6R7D9|F6R7D9\_MOUSE tr|D3YXA4|D3YXA4\_MOUSE sp|Q6Y5D8-4|RHG10\_MOUSE sp|Q3V209|TMUB2\_MOUSE sp|Q61985-3|NF2L1\_MOUSE tr|A2A6D2|A2A6D2\_MOUSE sp|Q9WTN0|GGPPS\_MOUSE tr|E9Q361|E9Q361\_MOUSE tr|I7HLR7|I7HLR7\_MOUSE sp|Q61985|NF2L1\_MOUSE sp|Q811D2|ANR26\_MOUSE sp|Q8CII0|ZBT8B\_MOUSE tr|F6ZLH4|F6ZLH4\_MOUSE tr|Q7TRG7|Q7TRG7\_MOUSE sp|Q920P3|BRNP1\_MOUSE sp|Q9Z2G6-2|SE1L1\_MOUSE tr|J3QJX3|J3QJX3\_MOUSE sp|Q9Z2G6|SE1L1\_MOUSE tr|D3Z6T8|D3Z6T8\_MOUSE sp|O08717|INHBE\_MOUSE tr|A2AVT5|A2AVT5\_MOUSE sp|Q9DCA7|CALY\_MOUSE tr|E9QA54|E9QA54\_MOUSE sp|Q9EP78|CHST7\_MOUSE sp|Q6S5J6-2|KRIT1\_MOUSE sp|Q6S5J6|KRIT1\_MOUSE sp|Q9JI48|PLAC8\_MOUSE tr|F8WIG5|F8WIG5\_MOUSE tr|B1AVP5|B1AVP5\_MOUSE sp|Q8BHE1|GEMI8\_MOUSE sp|Q6S5J6-3|KRIT1\_MOUSE sp|Q8C6D4-2|BEND5\_MOUSE sp|Q80TB7-2|ZSWM6\_MOUSE tr|F6Q2G7|F6Q2G7\_MOUSE sp|Q9Z207|DIAP3\_MOUSE sp|A1L3C1|F71E1\_MOUSE sp|Q80TB7|ZSWM6\_MOUSE tr|Q4PLS0|Q4PLS0\_MOUSE sp|Q8K4L0|DDX54\_MOUSE tr|B9EJL3|B9EJL3\_MOUSE tr|B1ATA0|B1ATA0\_MOUSE tr|A0A087WP80|A0A087WP80\_MOUSE sp|Q8BM92|CADH7\_MOUSE sp|P70120|HES5\_MOUSE sp|Q9CXB8|ALPK1\_MOUSE tr|E9QNX4|E9QNX4\_MOUSE tr|E9Q8M9|E9Q8M9\_MOUSE sp|Q60695|RGL1\_MOUSE tr|E9Q8N0|E9Q8N0\_MOUSE tr|E9PWT2|E9PWT2\_MOUSE tr|Q810Y9|Q810Y9\_MOUSE sp|O88967|YMEL1\_MOUSE sp|Q8K349|GIMA6\_MOUSE tr|Q9CY80|Q9CY80\_MOUSE sp|Q3USJ8|FCSD2\_MOUSE sp|Q3USJ8-3|FCSD2\_MOUSE sp|Q3USJ8-2|FCSD2\_MOUSE tr|A2APZ7|A2APZ7\_MOUSE sp|Q06348|PRRX2\_MOUSE tr|A2A7F4|A2A7F4\_MOUSE sp|P36898|BMR1B\_MOUSE tr|E9PVI0|E9PVI0\_MOUSE sp|P51125-4|ICAL\_MOUSE sp|Q8VE08|FBX33\_MOUSE sp|Q9D7K2|TEN1L\_MOUSE sp|Q9DBJ6|JOS1\_MOUSE sp|P51125-5|ICAL\_MOUSE tr|E9Q532|E9Q532\_MOUSE sp|Q8CB14-4|LAS2\_MOUSE tr|Q5SU72|Q5SU72\_MOUSE sp|Q61510|TRI25\_MOUSE tr|Q3TN15|Q3TN15\_MOUSE sp|Q9ES74|NEK7\_MOUSE sp|E9Q3T6|PRD14\_MOUSE sp|Q80UU1-2|ANKZ1\_MOUSE sp|Q922H7|RSLBB\_MOUSE tr|Q9ER67|Q9ER67\_MOUSE sp|Q80UW2|FBX2\_MOUSE sp|Q924A0-2|TF7L2\_MOUSE sp|Q3UYV8|DAAF3\_MOUSE sp|Q924A0-8|TF7L2\_MOUSE tr|E9Q990|E9Q990\_MOUSE tr|D3Z2K5|D3Z2K5\_MOUSE sp|Q62225|CISH\_MOUSE sp|P45377|ALD2\_MOUSE sp|Q9JJN4|SCO2A\_MOUSE sp|Q9ESL0|SCO2B\_MOUSE sp|Q91WE1|SNX15\_MOUSE tr|D3Z479|D3Z479\_MOUSE sp|Q9DBH0|WWP2\_MOUSE sp|Q62170|SELPL\_MOUSE tr|A1L1B4|A1L1B4\_MOUSE sp|Q6TXD4|DNMBP\_MOUSE sp|Q75UR0-2|ANO5\_MOUSE tr|D3YZQ6|D3YZQ6\_MOUSE tr|D3Z4X4|D3Z4X4\_MOUSE sp|Q8K1I3|SPP24\_MOUSE sp|Q60988|STIL\_MOUSE tr|Q3TA56|Q3TA56\_MOUSE tr|A2AM65|A2AM65\_MOUSE tr|A2AD39|A2AD39\_MOUSE tr|Q8C0C3|Q8C0C3\_MOUSE tr|F6ZRX7|F6ZRX7\_MOUSE sp|Q9WVG6|CARM1\_MOUSE sp|P52875|TM165\_MOUSE tr|G3UYE0|G3UYE0\_MOUSE sp|Q9D1H8|RM53\_MOUSE tr|D3YUP1|D3YUP1\_MOUSE tr|V9GXN8|V9GXN8\_MOUSE sp|Q66JW3|TOX\_MOUSE sp|Q61609|S20A1\_MOUSE sp|Q9WVG6-2|CARM1\_MOUSE sp|Q66JW3-2|TOX\_MOUSE tr|Q3U1Q3|Q3U1Q3\_MOUSE tr|Q3T9A5|Q3T9A5\_MOUSE sp|Q69ZR9-2|F208A\_MOUSE tr|E9QK04|E9QK04\_MOUSE sp|Q64131-2|RUNX3\_MOUSE tr|E9QAT9|E9QAT9\_MOUSE sp|P97798-4|NEO1\_MOUSE sp|Q9DC70|NDUS7\_MOUSE sp|Q8BQ86|CHST8\_MOUSE sp|Q9ERP3|TRI54\_MOUSE sp|P97798-2|NEO1\_MOUSE sp|P97798-5|NEO1\_MOUSE sp|Q99MA9|NKX61\_MOUSE sp|Q69ZR9|F208A\_MOUSE sp|P97798|NEO1\_MOUSE sp|Q8BZM1|GLMN\_MOUSE sp|Q64131|RUNX3\_MOUSE sp|P97798-3|NEO1\_MOUSE tr|D3YUM9|D3YUM9\_MOUSE sp|A6H6A9-4|RBG1L\_MOUSE sp|Q9QWK4|CD5L\_MOUSE sp|Q9CR41-2|HYPK\_MOUSE sp|Q9CR41|HYPK\_MOUSE tr|B9EKS2|B9EKS2\_MOUSE tr|B7ZCF3|B7ZCF3\_MOUSE tr|Q80Y74|Q80Y74\_MOUSE tr|Z4YML9|Z4YML9\_MOUSE sp|Q9CQZ6|NDUB3\_MOUSE sp|Q8BKU8|TM87B\_MOUSE sp|Q8BKU8-2|TM87B\_MOUSE sp|Q8BG34|UBX10\_MOUSE sp|Q8BKU8-3|TM87B\_MOUSE tr|D3YVC9|D3YVC9\_MOUSE tr|F7BHW1|F7BHW1\_MOUSE tr|D3Z1Y8|D3Z1Y8\_MOUSE tr|Q1LZI5|Q1LZI5\_MOUSE tr|A0A087WR44|A0A087WR44\_MOUSE sp|Q8CGW9|DMRTD\_MOUSE tr|B8JKF6|B8JKF6\_MOUSE sp|Q91WB2|PPAC3\_MOUSE tr|F7CWV4|F7CWV4\_MOUSE sp|Q9D3R3|CEP72\_MOUSE sp|Q9R1K5|FZR\_MOUSE tr|M0QWW4|M0QWW4\_MOUSE sp|Q921R7|S35A5\_MOUSE sp|Q9D805|CAN9\_MOUSE sp|Q99ME7|TBX19\_MOUSE sp|O35256|PR4A1\_MOUSE sp|Q91WL0|ES8L3\_MOUSE tr|Q7TR95|Q7TR95\_MOUSE sp|O88597|BECN1\_MOUSE sp|Q8VCG3|WDR74\_MOUSE tr|A2AFG8|A2AFG8\_MOUSE tr|A0A087WQW4|A0A087WQW4\_MOUSE tr|F7C090|F7C090\_MOUSE tr|E9Q698|E9Q698\_MOUSE tr|A2A888|A2A888\_MOUSE tr|A2AFG7|A2AFG7\_MOUSE sp|P20334|TNR9\_MOUSE tr|Q6PGJ3|Q6PGJ3\_MOUSE tr|A0A087WRX8|A0A087WRX8\_MOUSE sp|Q9ERI6|RDH14\_MOUSE sp|Q9JIY0|PKHO1\_MOUSE tr|F6VV25|F6VV25\_MOUSE tr|Q7TR53|Q7TR53\_MOUSE sp|Q9JJC8|NIPA2\_MOUSE tr|S4R193|S4R193\_MOUSE sp|Q08761|PROS\_MOUSE sp|Q80TL4|K1045\_MOUSE tr|S4R202|S4R202\_MOUSE sp|Q9DCL9|PUR6\_MOUSE sp|Q9CYK2-2|QPCT\_MOUSE sp|Q9CYK2|QPCT\_MOUSE sp|Q9Z140|CPNE6\_MOUSE sp|Q8BNJ6|NETO2\_MOUSE sp|Q8BNJ6-2|NETO2\_MOUSE sp|Q8BKE9|IFT74\_MOUSE tr|Q3UYN2|Q3UYN2\_MOUSE tr|Q8VGB8|Q8VGB8\_MOUSE tr|D3Z2N7|D3Z2N7\_MOUSE sp|Q61488|DHH\_MOUSE tr|I7HJS5|I7HJS5\_MOUSE sp|Q78T81|F102A\_MOUSE tr|D6RFS6|D6RFS6\_MOUSE sp|Q91YY2|B4GT3\_MOUSE sp|Q00896|A1AT3\_MOUSE sp|Q9ERH8|S28A3\_MOUSE sp|P07758|A1AT1\_MOUSE tr|Q6PFB2|Q6PFB2\_MOUSE tr|F7BUB8|F7BUB8\_MOUSE tr|Q3UT76|Q3UT76\_MOUSE tr|Q7TRH5|Q7TRH5\_MOUSE sp|Q8CFK2|TF3B\_MOUSE sp|Q8VE37|RCC1\_MOUSE sp|Q32M07|KAD8\_MOUSE sp|Q62351|TFR1\_MOUSE sp|Q8CHK4-3|KAT5\_MOUSE sp|Q8CHK4-2|KAT5\_MOUSE sp|Q63ZW7-4|INADL\_MOUSE sp|Q61146|OCLN\_MOUSE sp|Q8CHK4|KAT5\_MOUSE sp|P83861|QRFPR\_MOUSE sp|Q3U3E2|F117B\_MOUSE sp|Q9JIX8-2|ACINU\_MOUSE tr|E9Q3A0|E9Q3A0\_MOUSE sp|Q3URY6-2|ARMC2\_MOUSE tr|F7CPS1|F7CPS1\_MOUSE tr|Q52KR6|Q52KR6\_MOUSE tr|F6RJ39|F6RJ39\_MOUSE tr|B8JJ91|B8JJ91\_MOUSE tr|F6Q8C0|F6Q8C0\_MOUSE sp|Q9JIX8-3|ACINU\_MOUSE sp|Q80WG7|TRI36\_MOUSE tr|B8JJ92|B8JJ92\_MOUSE tr|Q9DBN8|Q9DBN8\_MOUSE sp|Q3UC65|RSRP1\_MOUSE sp|Q6WQJ1|DGLA\_MOUSE sp|P30306|MPIP2\_MOUSE sp|A2AJA9-2|CI172\_MOUSE tr|Q8C084|Q8C084\_MOUSE sp|A2AJA9|CI172\_MOUSE tr|A2ADB2|A2ADB2\_MOUSE tr|G3UW50|G3UW50\_MOUSE sp|B1AT66|MOT7\_MOUSE sp|Q99JW5|EPCAM\_MOUSE sp|P26955|IL3RB\_MOUSE tr|O35204|O35204\_MOUSE sp|Q8BMJ7|CGRF1\_MOUSE sp|O08789|MNT\_MOUSE sp|P11103|PARP1\_MOUSE sp|Q62219-4|TGFI1\_MOUSE sp|Q8R4Y8-3|RTTN\_MOUSE tr|F6S5E4|F6S5E4\_MOUSE sp|Q62219-10|TGFI1\_MOUSE sp|Q62219|TGFI1\_MOUSE sp|Q62219-2|TGFI1\_MOUSE sp|Q62219-7|TGFI1\_MOUSE tr|F7AIF3|F7AIF3\_MOUSE tr|E9Q1D5|E9Q1D5\_MOUSE sp|Q62219-5|TGFI1\_MOUSE sp|Q62219-9|TGFI1\_MOUSE tr|E9PYQ1|E9PYQ1\_MOUSE sp|Q9QZR5-3|HIPK2\_MOUSE sp|Q91X49|MALL\_MOUSE sp|Q8R2Q6|TECT3\_MOUSE sp|Q8R2Q6-2|TECT3\_MOUSE tr|F6U238|F6U238\_MOUSE sp|Q9QUR7|PIN1\_MOUSE tr|S4R2E2|S4R2E2\_MOUSE sp|Q99N69|LPXN\_MOUSE sp|Q8K348|ACV1C\_MOUSE tr|Q3V348|Q3V348\_MOUSE sp|Q9QWT9-3|KIFC1\_MOUSE sp|Q8CIX8|LGSN\_MOUSE tr|A2AJR5|A2AJR5\_MOUSE sp|Q9D415-5|DLGP1\_MOUSE tr|D3Z6F4|D3Z6F4\_MOUSE tr|A2AJR6|A2AJR6\_MOUSE sp|Q9WVL2|STAT2\_MOUSE sp|Q9D415-3|DLGP1\_MOUSE tr|G3X9P9|G3X9P9\_MOUSE sp|Q8R092-2|CA043\_MOUSE sp|Q8R092-1|CA043\_MOUSE tr|L7N212|L7N212\_MOUSE tr|A2ANU5|A2ANU5\_MOUSE sp|P0DM40|FR1L5\_MOUSE sp|P60843|IF4A1\_MOUSE sp|Q8VEC3|GP110\_MOUSE sp|Q8BZW4-2|ZN120\_MOUSE sp|Q8BKE6|CP20A\_MOUSE sp|O35601-2|FYB\_MOUSE tr|L7N1Y5|L7N1Y5\_MOUSE sp|Q91WJ0|FRS3\_MOUSE tr|A2RTP5|A2RTP5\_MOUSE sp|P21729|GRPR\_MOUSE sp|O35601|FYB\_MOUSE sp|Q9Z1W8|AT12A\_MOUSE tr|L7N1Y1|L7N1Y1\_MOUSE sp|Q8CFI0|NED4L\_MOUSE sp|Q8CFI0-3|NED4L\_MOUSE tr|G3X9H8|G3X9H8\_MOUSE sp|Q8CFI0-2|NED4L\_MOUSE tr|E9PXB7|E9PXB7\_MOUSE sp|Q8K297|GT251\_MOUSE sp|Q6ZQ82|RHG26\_MOUSE sp|Q504P2|CL12A\_MOUSE tr|D3YXD8|D3YXD8\_MOUSE tr|F6TQP9|F6TQP9\_MOUSE tr|F6XBS7|F6XBS7\_MOUSE tr|F6Q7M1|F6Q7M1\_MOUSE sp|Q8R550-6|SH3K1\_MOUSE tr|F6XTB7|F6XTB7\_MOUSE tr|E9QAQ3|E9QAQ3\_MOUSE sp|Q8R066|C1QT4\_MOUSE tr|E9Q5G2|E9Q5G2\_MOUSE sp|Q8C5N3|CWC22\_MOUSE tr|E9PY43|E9PY43\_MOUSE tr|Q8BQR7|Q8BQR7\_MOUSE sp|Q9D2I5-5|ARMC9\_MOUSE sp|P43117|PF2R\_MOUSE sp|Q9D2I5-3|ARMC9\_MOUSE sp|Q9D2I5|ARMC9\_MOUSE sp|Q8BGA9|OXA1L\_MOUSE tr|D3Z3B6|D3Z3B6\_MOUSE tr|Q8VFP3|Q8VFP3\_MOUSE sp|P21784|RAG2\_MOUSE sp|Q9DAK8-2|LRC51\_MOUSE tr|G5E8I1|G5E8I1\_MOUSE sp|Q9DAK8|LRC51\_MOUSE tr|Q8VFW4|Q8VFW4\_MOUSE tr|D3Z410|D3Z410\_MOUSE sp|Q99JN2|KLH22\_MOUSE sp|P97817|CDR2\_MOUSE sp|Q9WV34-2|MPP2\_MOUSE sp|Q9WV34|MPP2\_MOUSE tr|D3YVA6|D3YVA6\_MOUSE sp|O55123|MMP10\_MOUSE tr|H9KV05|H9KV05\_MOUSE tr|Q8C1E5|Q8C1E5\_MOUSE tr|D3YVB2|D3YVB2\_MOUSE tr|E9PZG0|E9PZG0\_MOUSE tr|Q5SWK8|Q5SWK8\_MOUSE tr|D3Z6T7|D3Z6T7\_MOUSE tr|Q59IW6|Q59IW6\_MOUSE tr|Q4ZGD8|Q4ZGD8\_MOUSE tr|Q5SWK4|Q5SWK4\_MOUSE sp|P10287|CADH3\_MOUSE tr|A2A9P2|A2A9P2\_MOUSE tr|E9PUV2|E9PUV2\_MOUSE tr|E9PVR4|E9PVR4\_MOUSE sp|Q07802|COE1\_MOUSE tr|Q99MW6|Q99MW6\_MOUSE sp|P08508|FCGR3\_MOUSE sp|Q07802-2|COE1\_MOUSE sp|Q8VE01|DUS18\_MOUSE tr|Q8R499|Q8R499\_MOUSE tr|Q059P4|Q059P4\_MOUSE sp|Q9CS72|FLIP1\_MOUSE sp|Q3URV1|BROMI\_MOUSE sp|Q99L88-2|SNTB1\_MOUSE sp|Q3URV1-2|BROMI\_MOUSE tr|E9Q598|E9Q598\_MOUSE sp|P01745|HVM01\_MOUSE tr|A0A075B684|A0A075B684\_MOUSE tr|A0A075B5R4|A0A075B5R4\_MOUSE tr|A0A075B5W9|A0A075B5W9\_MOUSE sp|Q3TCN2-2|PLBL2\_MOUSE tr|A0A075B5W5|A0A075B5W5\_MOUSE tr|A0A075B5U8|A0A075B5U8\_MOUSE sp|P97459|NPAS1\_MOUSE tr|A0A075B5V9|A0A075B5V9\_MOUSE tr|F6T652|F6T652\_MOUSE P17690 tr|A2AKU3|A2AKU3\_MOUSE tr|A0A075B5U2|A0A075B5U2\_MOUSE sp|Q8K183|PDXK\_MOUSE sp|Q8C0Y0-3|PP4R4\_MOUSE tr|Q05BZ2|Q05BZ2\_MOUSE sp|Q61597|CRGC\_MOUSE sp|P29594|CASP2\_MOUSE tr|D3Z7G7|D3Z7G7\_MOUSE sp|Q6P6P7-2|PARP6\_MOUSE tr|E9PYY9|E9PYY9\_MOUSE tr|F6UEA3|F6UEA3\_MOUSE tr|Q3V2A7|Q3V2A7\_MOUSE tr|A3RLD5|A3RLD5\_MOUSE tr|E9Q861|E9Q861\_MOUSE sp|P04344|CRGB\_MOUSE tr|F7CCJ3|F7CCJ3\_MOUSE tr|D3Z7B0|D3Z7B0\_MOUSE sp|Q3V096-3|ANR42\_MOUSE sp|Q8BXL9|IFFO1\_MOUSE tr|D6RH90|D6RH90\_MOUSE sp|P28231|CXB3\_MOUSE sp|O35691|PININ\_MOUSE tr|Q3TUQ5|Q3TUQ5\_MOUSE sp|Q3V096-2|ANR42\_MOUSE sp|Q3V096|ANR42\_MOUSE tr|A2ATE3|A2ATE3\_MOUSE tr|Q3UWV0|Q3UWV0\_MOUSE tr|S4R1K3|S4R1K3\_MOUSE sp|A2A9A2|DMTA2\_MOUSE tr|A2AE32|A2AE32\_MOUSE sp|Q8C5N3-2|CWC22\_MOUSE tr|F6T4X0|F6T4X0\_MOUSE tr|A2AK44|A2AK44\_MOUSE sp|P20612|GNAT1\_MOUSE sp|Q9DBE0|CSAD\_MOUSE tr|F7C528|F7C528\_MOUSE tr|B1AYU4|B1AYU4\_MOUSE tr|F7A7F2|F7A7F2\_MOUSE tr|Q8VGZ6|Q8VGZ6\_MOUSE tr|E9Q614|E9Q614\_MOUSE sp|Q4VA45|CK095\_MOUSE tr|F6QPU5|F6QPU5\_MOUSE tr|B1AYU7|B1AYU7\_MOUSE tr|A2AK42|A2AK42\_MOUSE sp|P50149|GNAT2\_MOUSE sp|Q8VCC6|CCM2L\_MOUSE tr|B1AR17|B1AR17\_MOUSE sp|Q810A7|DDX42\_MOUSE tr|Q7TR20|Q7TR20\_MOUSE tr|D3YXK9|D3YXK9\_MOUSE tr|D6RFU2|D6RFU2\_MOUSE tr|G3UY26|G3UY26\_MOUSE sp|D3Z1Q2|MRAP2\_MOUSE sp|Q99LR1|ABD12\_MOUSE sp|P0C191|GPVI\_MOUSE tr|E9PZV8|E9PZV8\_MOUSE tr|B7ZNC5|B7ZNC5\_MOUSE tr|Q3UIX4|Q3UIX4\_MOUSE tr|E9Q6E5|E9Q6E5\_MOUSE tr|E9Q255|E9Q255\_MOUSE tr|A2AV95|A2AV95\_MOUSE sp|Q9EQ45|V1R46\_MOUSE tr|Q8BV04|Q8BV04\_MOUSE tr|Q9D8U9|Q9D8U9\_MOUSE sp|A3KGF7|PLCB2\_MOUSE sp|Q3UTQ8-2|CDKL5\_MOUSE tr|E9PYI3|E9PYI3\_MOUSE tr|Q8VFK5|Q8VFK5\_MOUSE sp|Q811B3|ATS12\_MOUSE tr|B1ASY6|B1ASY6\_MOUSE tr|B2FDE5|B2FDE5\_MOUSE sp|P23881|TCEA3\_MOUSE sp|Q922G2|FA76A\_MOUSE sp|Q8R3F9|STPAP\_MOUSE sp|Q8VHP6|CDHR1\_MOUSE tr|F8WH78|F8WH78\_MOUSE tr|Q3UR42|Q3UR42\_MOUSE sp|Q8BG95|MYPT2\_MOUSE sp|Q920Q4-2|VPS16\_MOUSE tr|Q920G5|Q920G5\_MOUSE sp|Q61345|FOXD1\_MOUSE tr|A6H644|A6H644\_MOUSE sp|Q920Q4|VPS16\_MOUSE tr|G3X8X7|G3X8X7\_MOUSE tr|D3YZC1|D3YZC1\_MOUSE sp|Q99MN9|PCCB\_MOUSE sp|Q8CCH7|FOG2\_MOUSE sp|A2A5E6|Z385C\_MOUSE sp|A2A5E6-2|Z385C\_MOUSE tr|A2AVT0|A2AVT0\_MOUSE tr|L7MU57|L7MU57\_MOUSE tr|D3YXZ0|D3YXZ0\_MOUSE tr|F2Z462|F2Z462\_MOUSE sp|Q6RUT8-3|CC154\_MOUSE sp|P97306|STAC\_MOUSE tr|S4R1F0|S4R1F0\_MOUSE sp|Q9DCC3|CC107\_MOUSE sp|Q9DCC3-2|CC107\_MOUSE sp|Q2TA57|ASPH1\_MOUSE tr|E9QAZ9|E9QAZ9\_MOUSE tr|E9PZJ7|E9PZJ7\_MOUSE tr|E9PUE1|E9PUE1\_MOUSE tr|L7N279|L7N279\_MOUSE tr|K7N789|K7N789\_MOUSE sp|Q8BSI6|R3HC1\_MOUSE tr|E9Q9Q5|E9Q9Q5\_MOUSE tr|E9Q2J5|E9Q2J5\_MOUSE tr|A0A087WR28|A0A087WR28\_MOUSE tr|E9PWW0|E9PWW0\_MOUSE tr|E9Q1N6|E9Q1N6\_MOUSE tr|E9PZK8|E9PZK8\_MOUSE tr|Q8R2C7|Q8R2C7\_MOUSE sp|Q66GT5|PTPM1\_MOUSE tr|Q9D9A9|Q9D9A9\_MOUSE sp|Q61382|TRAF4\_MOUSE tr|B7ZC19|B7ZC19\_MOUSE tr|B2KF50|B2KF50\_MOUSE tr|A2AHK0|A2AHK0\_MOUSE sp|Q8BXQ2|PIGT\_MOUSE tr|Q6PGF5|Q6PGF5\_MOUSE sp|Q8BI17|SH2D7\_MOUSE tr|D3Z0R7|D3Z0R7\_MOUSE tr|E9Q5L6|E9Q5L6\_MOUSE sp|Q9D2H9|DAAF1\_MOUSE sp|P63158|HMGB1\_MOUSE tr|D3Z7Q6|D3Z7Q6\_MOUSE sp|Q9JKA3|TR103\_MOUSE tr|E9QN68|E9QN68\_MOUSE sp|Q8C310|ROBO4\_MOUSE sp|Q9D0R2|SYTC\_MOUSE sp|Q8C310-2|ROBO4\_MOUSE tr|E9PUJ1|E9PUJ1\_MOUSE tr|A0A087WPL3|A0A087WPL3\_MOUSE tr|G3V019|G3V019\_MOUSE tr|G3UXD0|G3UXD0\_MOUSE tr|A0A087WQA4|A0A087WQA4\_MOUSE sp|Q80WC9-2|ACSF4\_MOUSE tr|E9PUI6|E9PUI6\_MOUSE sp|O55135|IF6\_MOUSE sp|Q80WC9-3|ACSF4\_MOUSE sp|Q78ZA7|NP1L4\_MOUSE sp|Q9ERB5|SO1C1\_MOUSE tr|Q8R2A7|Q8R2A7\_MOUSE tr|A0A075B683|A0A075B683\_MOUSE sp|Q6ZQ58|LARP1\_MOUSE tr|F7D291|F7D291\_MOUSE sp|Q91VA3|CAN8\_MOUSE tr|D3Z6W9|D3Z6W9\_MOUSE tr|Z4YJT3|Z4YJT3\_MOUSE tr|D3YX99|D3YX99\_MOUSE sp|Q9CQH3|NDUB5\_MOUSE sp|Q571G4|LIN54\_MOUSE tr|F6Y6V5|F6Y6V5\_MOUSE sp|Q3UWA4|TRI40\_MOUSE sp|Q9Z2Z9|GFPT2\_MOUSE sp|Q8BJI4|PO6F2\_MOUSE sp|Q9CX83|ARMX1\_MOUSE tr|F6XRI9|F6XRI9\_MOUSE sp|Q8VCA5|TMPS4\_MOUSE tr|Q9D104|Q9D104\_MOUSE tr|D3Z4T9|D3Z4T9\_MOUSE sp|Q9R020|ZRAB2\_MOUSE tr|F8WJ94|F8WJ94\_MOUSE tr|D3Z4U0|D3Z4U0\_MOUSE sp|Q60654-3|KLRA7\_MOUSE sp|Q8K0Z9|GP153\_MOUSE sp|P17751|TPIS\_MOUSE tr|E9PUD0|E9PUD0\_MOUSE sp|Q60654-2|KLRA7\_MOUSE sp|Q60654|KLRA7\_MOUSE sp|Q9R020-2|ZRAB2\_MOUSE sp|Q5RJI4-2|PKDCC\_MOUSE sp|Q920F6|SMC1B\_MOUSE sp|A2RSQ1|GLBL3\_MOUSE tr|F6ZBY9|F6ZBY9\_MOUSE sp|A2RSQ1-2|GLBL3\_MOUSE tr|F8WIH9|F8WIH9\_MOUSE sp|Q9JMJ2|FBXW4\_MOUSE sp|Q62441|TLE4\_MOUSE tr|Q5NCB5|Q5NCB5\_MOUSE sp|Q5RKZ7|MOCS1\_MOUSE sp|Q8BGY6|B3GN5\_MOUSE sp|Q9JKS4-6|LDB3\_MOUSE sp|Q3UFK8|FRMD8\_MOUSE sp|Q9JKS4-3|LDB3\_MOUSE sp|Q9JKS4-4|LDB3\_MOUSE sp|Q9JKS4-5|LDB3\_MOUSE sp|Q6PIX9-2|CQ080\_MOUSE sp|Q6PIX9|CQ080\_MOUSE sp|P58929|GMEB2\_MOUSE sp|P06802-2|ENPP1\_MOUSE sp|P06802|ENPP1\_MOUSE tr|G3X9S2|G3X9S2\_MOUSE sp|P58681|TLR7\_MOUSE sp|D3Z5L9|WISP3\_MOUSE tr|Q78Y72|Q78Y72\_MOUSE sp|P97503|NKX32\_MOUSE sp|Q9D5Y0|CG031\_MOUSE sp|Q9CZR2|NALD2\_MOUSE tr|G3UWC2|G3UWC2\_MOUSE sp|Q3U285-3|LCORL\_MOUSE tr|F8WHM3|F8WHM3\_MOUSE sp|Q3U285-6|LCORL\_MOUSE tr|D3YWB7|D3YWB7\_MOUSE sp|P16283-3|B3A3\_MOUSE sp|P16283-2|B3A3\_MOUSE tr|E9PXY2|E9PXY2\_MOUSE sp|P58459|ATS10\_MOUSE sp|Q9DBD0|ICA\_MOUSE tr|Q6NZQ2|Q6NZQ2\_MOUSE tr|Q80ZX9|Q80ZX9\_MOUSE tr|D3YYN4|D3YYN4\_MOUSE tr|G3UYE8|G3UYE8\_MOUSE tr|F6RLJ7|F6RLJ7\_MOUSE sp|P14206|RSSA\_MOUSE sp|Q80X91|F110D\_MOUSE tr|Q7TS32|Q7TS32\_MOUSE sp|Q60795|NF2L2\_MOUSE sp|A2A8U2-2|TM201\_MOUSE sp|Q69ZS8|KAZRN\_MOUSE tr|G5E8Q8|G5E8Q8\_MOUSE sp|Q3U492-2|KCP\_MOUSE sp|Q8BRC6-1|MAAT1\_MOUSE sp|A4Q9E4|TTLL2\_MOUSE tr|A2AJ14|A2AJ14\_MOUSE tr|E9QNB3|E9QNB3\_MOUSE sp|Q8JZN3|KCJ14\_MOUSE tr|S4R2Q0|S4R2Q0\_MOUSE sp|Q3U492|KCP\_MOUSE tr|Q3UJQ9|Q3UJQ9\_MOUSE tr|E9PZS1|E9PZS1\_MOUSE sp|Q99LH4|ZN672\_MOUSE tr|J3QNM7|J3QNM7\_MOUSE tr|E0CYL0|E0CYL0\_MOUSE tr|Q3UX49|Q3UX49\_MOUSE sp|Q8BRC6|MAAT1\_MOUSE sp|Q9D0K2|SCOT1\_MOUSE sp|Q7TMK6|HOOK2\_MOUSE sp|Q8BTM8-2|FLNA\_MOUSE sp|Q8BTU1-2|CFA20\_MOUSE sp|Q91ZW2|OFUT1\_MOUSE sp|Q8BTU1|CFA20\_MOUSE sp|Q99LB7|SARDH\_MOUSE sp|P46061|RAGP1\_MOUSE sp|Q99J93|IFM2\_MOUSE sp|A7XV14|SKI11\_MOUSE tr|A6PWC2|A6PWC2\_MOUSE sp|Q6VNB8-2|WDFY3\_MOUSE sp|Q99KK9|SYHM\_MOUSE Q3SZV7 tr|F6TQ19|F6TQ19\_MOUSE tr|F7D926|F7D926\_MOUSE tr|F8WH72|F8WH72\_MOUSE sp|P70178|SIX5\_MOUSE sp|P53612|PGTB2\_MOUSE sp|Q766D5|B4GN4\_MOUSE tr|H3BJB7|H3BJB7\_MOUSE tr|Q3TVF4|Q3TVF4\_MOUSE sp|P49962|SRP09\_MOUSE tr|D3YZX8|D3YZX8\_MOUSE tr|F2Z488|F2Z488\_MOUSE sp|Q64389|CD52\_MOUSE sp|Q62481|VPS72\_MOUSE tr|Q8BRT3|Q8BRT3\_MOUSE tr|H3BJ29|H3BJ29\_MOUSE sp|Q9R069|BCAM\_MOUSE sp|Q8R4B8-3|NALP3\_MOUSE tr|A0A087WSI8|A0A087WSI8\_MOUSE sp|Q8R4B8|NALP3\_MOUSE sp|Q9D0V8-2|CINP\_MOUSE sp|Q9D0V8-3|CINP\_MOUSE tr|F7C3I9|F7C3I9\_MOUSE tr|E9PYD6|E9PYD6\_MOUSE sp|Q8R4B8-2|NALP3\_MOUSE sp|Q9WVQ1-4|MAGI2\_MOUSE tr|D3YU51|D3YU51\_MOUSE sp|P81270-3|ERG\_MOUSE sp|Q99MH5|NDK5\_MOUSE sp|Q9WVQ1-2|MAGI2\_MOUSE sp|P81270-4|ERG\_MOUSE tr|I1E4Y2|I1E4Y2\_MOUSE tr|G5E8F2|G5E8F2\_MOUSE sp|P81270|ERG\_MOUSE tr|V9GXS1|V9GXS1\_MOUSE tr|E9PY05|E9PY05\_MOUSE tr|A0A087WS86|A0A087WS86\_MOUSE sp|Q9WVQ1|MAGI2\_MOUSE sp|P81270-2|ERG\_MOUSE sp|Q99MH5-2|NDK5\_MOUSE sp|Q8VCQ3|NRBF2\_MOUSE tr|Q6P8P8|Q6P8P8\_MOUSE sp|Q99J78|DEFI8\_MOUSE tr|G3X9U5|G3X9U5\_MOUSE tr|Q3TF45|Q3TF45\_MOUSE sp|P51791|CLCN3\_MOUSE sp|O35304|VACHT\_MOUSE sp|Q58NB6|DHRS9\_MOUSE tr|Q3TYJ1|Q3TYJ1\_MOUSE sp|Q8VHI5|VITRN\_MOUSE sp|Q62018-2|CTR9\_MOUSE tr|F6X687|F6X687\_MOUSE tr|B1AX30|B1AX30\_MOUSE tr|A3KN63|A3KN63\_MOUSE tr|L7N291|L7N291\_MOUSE tr|L7N284|L7N284\_MOUSE tr|L7N2A2|L7N2A2\_MOUSE tr|F7D1B3|F7D1B3\_MOUSE tr|L7MUE8|L7MUE8\_MOUSE tr|F6XVS9|F6XVS9\_MOUSE tr|F6SCS1|F6SCS1\_MOUSE tr|E9PX73|E9PX73\_MOUSE tr|J3QP00|J3QP00\_MOUSE sp|Q01965|LY9\_MOUSE tr|F7A7Y6|F7A7Y6\_MOUSE tr|J3QNG9|J3QNG9\_MOUSE tr|J3QMX3|J3QMX3\_MOUSE tr|L7N200|L7N200\_MOUSE sp|Q3TZ65|OD3L2\_MOUSE tr|J3QNA6|J3QNA6\_MOUSE tr|L7N294|L7N294\_MOUSE tr|S4R2G4|S4R2G4\_MOUSE tr|E9PV93|E9PV93\_MOUSE tr|W4VSP1|W4VSP1\_MOUSE tr|J3QNE1|J3QNE1\_MOUSE tr|F6R3J9|F6R3J9\_MOUSE sp|Q6Q473|CLCA4\_MOUSE sp|Q6V5K9|ZN474\_MOUSE sp|Q99KN9|EPN4\_MOUSE sp|Q9DBU6|RSRC1\_MOUSE tr|E9Q6P8|E9Q6P8\_MOUSE tr|Q9DA18|Q9DA18\_MOUSE tr|E9Q4T9|E9Q4T9\_MOUSE tr|Q5SUH7|Q5SUH7\_MOUSE tr|Q5SUH6|Q5SUH6\_MOUSE tr|Q8BR75|Q8BR75\_MOUSE tr|D3YXG1|D3YXG1\_MOUSE tr|Q5FWB9|Q5FWB9\_MOUSE tr|G5E883|G5E883\_MOUSE tr|F7AEX1|F7AEX1\_MOUSE tr|G3UZ61|G3UZ61\_MOUSE tr|F6QY34|F6QY34\_MOUSE sp|O54689|CCR6\_MOUSE sp|Q9JLQ0|CD2AP\_MOUSE sp|Q60819|I15RA\_MOUSE sp|Q9EPR4|S23A2\_MOUSE tr|F7CAK3|F7CAK3\_MOUSE sp|Q9QUL3|PA2GE\_MOUSE sp|Q8VEL0|MSPD1\_MOUSE sp|Q8VEL0-2|MSPD1\_MOUSE tr|F7AYH6|F7AYH6\_MOUSE sp|Q91YL3|UCKL1\_MOUSE sp|P48776|T23O\_MOUSE tr|E9PYK5|E9PYK5\_MOUSE sp|Q9D3A8-2|CAPON\_MOUSE sp|Q9WUU7|CATZ\_MOUSE tr|D3YTT2|D3YTT2\_MOUSE sp|Q9D3A8|CAPON\_MOUSE sp|Q60641-2|NR1H4\_MOUSE sp|Q60641|NR1H4\_MOUSE tr|E0CYH9|E0CYH9\_MOUSE tr|Q3TRB6|Q3TRB6\_MOUSE sp|Q8BUM9-2|UBP43\_MOUSE tr|Q9D8C9|Q9D8C9\_MOUSE tr|A2CG25|A2CG25\_MOUSE tr|A6X8K9|A6X8K9\_MOUSE sp|Q8BMD2-2|DZIP1\_MOUSE sp|Q3UX61|NAA11\_MOUSE sp|Q8BUM9|UBP43\_MOUSE sp|Q9QUP5|HPLN1\_MOUSE tr|A6PWR8|A6PWR8\_MOUSE tr|Q9D2Z1|Q9D2Z1\_MOUSE tr|Q6S9I0|Q6S9I0\_MOUSE sp|Q9EQF5|DPYS\_MOUSE sp|P13864|DNMT1\_MOUSE sp|Q3UFT3|GAREM\_MOUSE sp|Q91WE6-5|CDKAL\_MOUSE sp|Q8R2Z5-2|VWA1\_MOUSE sp|Q9R014|CATJ\_MOUSE tr|E9PY33|E9PY33\_MOUSE tr|F8WHZ0|F8WHZ0\_MOUSE sp|Q04735|CDK16\_MOUSE sp|Q8C5P5|NT5D1\_MOUSE sp|Q04735-2|CDK16\_MOUSE sp|Q8BGA3|LRRT2\_MOUSE sp|Q8C5P5-2|NT5D1\_MOUSE sp|Q8C5P5-3|NT5D1\_MOUSE sp|Q8JZW4|CPNE5\_MOUSE tr|E9QN01|E9QN01\_MOUSE sp|Q5H8B9|FREM3\_MOUSE tr|S4R2G9|S4R2G9\_MOUSE tr|A2A5J8|A2A5J8\_MOUSE tr|Q3KNZ4|Q3KNZ4\_MOUSE sp|O88721|V2R\_MOUSE sp|P21300|ALD1\_MOUSE tr|G5E895|G5E895\_MOUSE sp|Q497K5|ARRD5\_MOUSE tr|D3Z494|D3Z494\_MOUSE sp|Q810C0|SLIK2\_MOUSE tr|G3UXR6|G3UXR6\_MOUSE tr|D3YYD1|D3YYD1\_MOUSE tr|G3X9X1|G3X9X1\_MOUSE tr|D3Z039|D3Z039\_MOUSE sp|Q6PFG8|OLIG3\_MOUSE sp|P56726|SMO\_MOUSE sp|Q9CUL5-3|IQCA1\_MOUSE sp|Q9QUJ7-2|ACSL4\_MOUSE tr|E9Q3H6|E9Q3H6\_MOUSE sp|Q9QUJ7|ACSL4\_MOUSE sp|P97313-2|PRKDC\_MOUSE tr|Q3UZ87|Q3UZ87\_MOUSE sp|Q9QUM0|ITA2B\_MOUSE sp|Q9JJ89|CCD86\_MOUSE sp|Q8R3W7|AGR3\_MOUSE sp|Q9JIS7|CAC1F\_MOUSE sp|Q9CWK8|SNX2\_MOUSE sp|P34056|AP2A\_MOUSE tr|Q8BPN4|Q8BPN4\_MOUSE sp|Q62186|SSRD\_MOUSE sp|P34056-3|AP2A\_MOUSE sp|A2ALU4-2|SHRM2\_MOUSE tr|G3UWS6|G3UWS6\_MOUSE sp|P34056-4|AP2A\_MOUSE sp|P34056-2|AP2A\_MOUSE tr|Q9D8L3|Q9D8L3\_MOUSE sp|Q8BJ05|ZC3HE\_MOUSE sp|Q8BJ05-3|ZC3HE\_MOUSE sp|Q9DCG2-2|CD302\_MOUSE sp|Q01338|ADA2A\_MOUSE tr|A2CG07|A2CG07\_MOUSE tr|A0A087WRL4|A0A087WRL4\_MOUSE tr|H3BKJ7|H3BKJ7\_MOUSE sp|Q9DCG2|CD302\_MOUSE sp|Q6NXH8|MET25\_MOUSE tr|H3BJE3|H3BJE3\_MOUSE tr|Q3URE6|Q3URE6\_MOUSE sp|Q8K1E0-2|STX5\_MOUSE tr|H3BJ02|H3BJ02\_MOUSE tr|Q7TRM9|Q7TRM9\_MOUSE sp|Q8K1E0|STX5\_MOUSE sp|Q7TMB8-2|CYFP1\_MOUSE sp|Q9JJV0|CRBA4\_MOUSE tr|F7BP06|F7BP06\_MOUSE sp|B2RY50|ARMC4\_MOUSE tr|E9QAS6|E9QAS6\_MOUSE tr|E9Q6P5|E9Q6P5\_MOUSE sp|Q8CJC5-2|NEUL3\_MOUSE sp|Q61475|DAF1\_MOUSE tr|E0CZ76|E0CZ76\_MOUSE tr|B1ATQ3|B1ATQ3\_MOUSE sp|Q8BGD5|CPT1C\_MOUSE sp|Q3UPE3-2|RMI2\_MOUSE sp|P70274|SEPP1\_MOUSE tr|D6RFT9|D6RFT9\_MOUSE tr|E0CZC5|E0CZC5\_MOUSE sp|Q9DB98|LENG1\_MOUSE tr|G3UYW2|G3UYW2\_MOUSE sp|P14152|MDHC\_MOUSE tr|E0CX80|E0CX80\_MOUSE sp|Q3TH73|TTYH2\_MOUSE sp|Q3TH73-2|TTYH2\_MOUSE sp|P25085|IL1RA\_MOUSE tr|A2AIU1|A2AIU1\_MOUSE sp|Q9QYB8-2|ADDB\_MOUSE tr|A0A087WPL8|A0A087WPL8\_MOUSE sp|Q8C0V7|SMKW\_MOUSE sp|P25085-2|IL1RA\_MOUSE tr|Q91Y03|Q91Y03\_MOUSE tr|Q3TBV5|Q3TBV5\_MOUSE sp|Q91WB7|UBTD1\_MOUSE sp|Q9EQP2|EHD4\_MOUSE tr|Q8VGL2|Q8VGL2\_MOUSE tr|D3Z6Z5|D3Z6Z5\_MOUSE sp|P58044|IDI1\_MOUSE tr|G3XA48|G3XA48\_MOUSE tr|H3BLF8|H3BLF8\_MOUSE sp|Q9D4P7|GSTT4\_MOUSE tr|J3QMA4|J3QMA4\_MOUSE sp|Q80U95|UBE3C\_MOUSE sp|Q3U0D9-3|HACE1\_MOUSE sp|Q3U0D9-4|HACE1\_MOUSE sp|Q3U0D9|HACE1\_MOUSE sp|Q9Z100|CPXM1\_MOUSE tr|E9PXI1|E9PXI1\_MOUSE tr|Q2Q5T5|Q2Q5T5\_MOUSE tr|A0A075B658|A0A075B658\_MOUSE sp|Q8CEG0|PANX3\_MOUSE tr|B1ARV8|B1ARV8\_MOUSE tr|G3UYL6|G3UYL6\_MOUSE sp|Q91UZ5|IMPA2\_MOUSE tr|A2A9P6|A2A9P6\_MOUSE sp|P11087|CO1A1\_MOUSE sp|Q99KE8|ZFP64\_MOUSE sp|P24788|CD11B\_MOUSE tr|A2AQR3|A2AQR3\_MOUSE sp|P24788-2|CD11B\_MOUSE sp|Q50H33-2|KCTD8\_MOUSE sp|A2AKG8|FOCAD\_MOUSE sp|Q50H33|KCTD8\_MOUSE tr|E9PYJ6|E9PYJ6\_MOUSE tr|G5E8T3|G5E8T3\_MOUSE tr|E9Q8N4|E9Q8N4\_MOUSE sp|Q61107|GBP4\_MOUSE tr|D3YUH5|D3YUH5\_MOUSE sp|Q91WA3|HDA11\_MOUSE tr|Q3V0U9|Q3V0U9\_MOUSE tr|F6QZR7|F6QZR7\_MOUSE tr|D6RHP8|D6RHP8\_MOUSE tr|G3UWQ4|G3UWQ4\_MOUSE tr|Q9EQA5|Q9EQA5\_MOUSE sp|Q8VDG5-2|PPCS\_MOUSE sp|Q6P9Y7|ZFP82\_MOUSE tr|S4R1G9|S4R1G9\_MOUSE tr|A0A075B5P5|A0A075B5P5\_MOUSE tr|A0A087WQF4|A0A087WQF4\_MOUSE sp|Q8VDG5|PPCS\_MOUSE tr|E9Q7E0|E9Q7E0\_MOUSE sp|Q6P9Y7-2|ZFP82\_MOUSE sp|Q8K2Y0-2|RN219\_MOUSE tr|B1AYG6|B1AYG6\_MOUSE sp|Q8K2Y0-3|RN219\_MOUSE tr|B1AYG7|B1AYG7\_MOUSE sp|Q9DBV4|MXRA8\_MOUSE sp|Q8C6J9|NAL4B\_MOUSE sp|Q9Z0T9|ITB6\_MOUSE tr|A8Y5E7|A8Y5E7\_MOUSE sp|Q9QXN5|MIOX\_MOUSE sp|Q8VE11|MTMR6\_MOUSE tr|A2AE89|A2AE89\_MOUSE sp|Q9CR57|RL14\_MOUSE tr|A2AV05|A2AV05\_MOUSE tr|D3Z013|D3Z013\_MOUSE sp|Q99NH7|S26A5\_MOUSE tr|H3BJG2|H3BJG2\_MOUSE tr|Q32MT6|Q32MT6\_MOUSE sp|P25918|CD19\_MOUSE tr|Q810H1|Q810H1\_MOUSE sp|Q924A0-4|TF7L2\_MOUSE sp|Q5SV42|ILEUC\_MOUSE tr|D3YTU3|D3YTU3\_MOUSE tr|E9Q0W2|E9Q0W2\_MOUSE sp|Q9QXZ6|SO1A1\_MOUSE sp|Q9EP96|SO1A4\_MOUSE tr|Q08EK6|Q08EK6\_MOUSE sp|Q99J94|SO1A6\_MOUSE tr|E9QQ90|E9QQ90\_MOUSE tr|E9QQ89|E9QQ89\_MOUSE sp|Q8BKW4-2|ZCHC4\_MOUSE tr|Q9DBN1|Q9DBN1\_MOUSE tr|Q3US60|Q3US60\_MOUSE tr|A0A087WQG5|A0A087WQG5\_MOUSE tr|E9Q2P6|E9Q2P6\_MOUSE sp|Q91VF2|HNMT\_MOUSE tr|B2RPU8|B2RPU8\_MOUSE sp|Q4KL78|KHD1C\_MOUSE tr|S4R246|S4R246\_MOUSE sp|Q3UWR2|KHD1A\_MOUSE sp|Q9QZM8|FBX17\_MOUSE tr|F8VPZ5|F8VPZ5\_MOUSE tr|S4R2C6|S4R2C6\_MOUSE sp|P11609|CD1D1\_MOUSE sp|P11610|CD1D2\_MOUSE sp|Q922E6|FAKD2\_MOUSE tr|S4R2A8|S4R2A8\_MOUSE sp|Q8K1T4-2|LY65B\_MOUSE sp|Q8K1T4|LY65B\_MOUSE sp|Q924H9|DQX1\_MOUSE tr|J3QP71|J3QP71\_MOUSE tr|B1AT36|B1AT36\_MOUSE sp|Q8CB96-2|RASF4\_MOUSE tr|Q3TRH2|Q3TRH2\_MOUSE sp|Q8C1M2|ZN428\_MOUSE tr|A2A521|A2A521\_MOUSE sp|Q8K3V1|SPAT4\_MOUSE sp|Q8CB96|RASF4\_MOUSE tr|H3BLD9|H3BLD9\_MOUSE sp|Q8CB96-3|RASF4\_MOUSE tr|H3BL05|H3BL05\_MOUSE sp|Q9D8W5|PSD12\_MOUSE tr|Q8CFZ0|Q8CFZ0\_MOUSE tr|G3UWL6|G3UWL6\_MOUSE tr|G3UYP0|G3UYP0\_MOUSE tr|G3UWJ1|G3UWJ1\_MOUSE tr|F6W8M5|F6W8M5\_MOUSE tr|F6V6F0|F6V6F0\_MOUSE sp|A2AA28|MET23\_MOUSE sp|Q9Z1L2|SNG4\_MOUSE tr|F6QXT4|F6QXT4\_MOUSE tr|B8JK50|B8JK50\_MOUSE tr|G3X9M2|G3X9M2\_MOUSE sp|Q8VBW5-3|BBX\_MOUSE sp|Q8BYH0-2|DRGX\_MOUSE sp|Q8VBW5-4|BBX\_MOUSE sp|Q8VBW5|BBX\_MOUSE sp|P97372|PSME2\_MOUSE sp|Q8BYH0|DRGX\_MOUSE tr|B0QZP3|B0QZP3\_MOUSE sp|Q04899|CDK18\_MOUSE sp|O88632-2|SEM3F\_MOUSE tr|D6RJ20|D6RJ20\_MOUSE sp|O88632|SEM3F\_MOUSE sp|Q9R049-2|AMFR\_MOUSE sp|Q9R049|AMFR\_MOUSE sp|Q8CHD8|RFIP3\_MOUSE tr|Q8K1K3|Q8K1K3\_MOUSE sp|Q9D903|EBP2\_MOUSE sp|Q9CTH6|FCF1\_MOUSE tr|A0A087WNS2|A0A087WNS2\_MOUSE sp|Q06138|CAB39\_MOUSE sp|Q8BG41|PSB11\_MOUSE sp|Q8CHD8-2|RFIP3\_MOUSE tr|E9Q186|E9Q186\_MOUSE tr|A2ACZ1|A2ACZ1\_MOUSE sp|Q9QXG9|TINF2\_MOUSE sp|Q8CHD8-3|RFIP3\_MOUSE tr|Q3U4P5|Q3U4P5\_MOUSE tr|Q9D3U4|Q9D3U4\_MOUSE tr|F6W459|F6W459\_MOUSE sp|Q61144-2|PSN2\_MOUSE sp|Q9WU01|KHDR2\_MOUSE sp|Q61144|PSN2\_MOUSE tr|D3Z0X1|D3Z0X1\_MOUSE sp|Q9CZB9|TM128\_MOUSE tr|B1AWN4|B1AWN4\_MOUSE sp|Q9R1C8|5HT6R\_MOUSE sp|Q9EPK6|SIL1\_MOUSE tr|E9Q623|E9Q623\_MOUSE tr|A2AEW1|A2AEW1\_MOUSE sp|Q9D404|OXSM\_MOUSE sp|Q6TL19|GUC2G\_MOUSE sp|P42
[truncated: 498,671 more chars]
