# Supplementary material for: Phospho-proteomic analyses of B-Raf protein complexes reveal new regulatory principles
Source: Oncotarget. 2016 Mar 28;7(18):26628–52. doi: 10.18632/oncotarget.8427 (PMC5042004; doi:10.18632/oncotarget.8427)
Supplement: Supplementary file 11 [file oncotarget-07-26628-s011.pdf]

2way ANOVA<br>Multiple comparisons						
1	Within each row, compare columns (simple effects within rows)					
2						
3	Number of families	2				
4	Number of comparisons per family	15				
5	Alpha	0.05				
6						
7	Uncorrected Fisher's LSD	Mean Diff.	95% CI of diff.	Significant?	Summary	Individual P Value
8						
9	NS					
10	V vs. WT	-0.7411	-1.869 to 0.3869	No	ns	0.1857
11	V vs. T401	-2.059	-3.187 to -0.9311	Yes	**	0.0011
12	V vs. S419	-0.7830	-1.911 to 0.3450	No	ns	0.1631
13	V vs. D594A	-1.173	-2.301 to -0.04448	Yes	*	0.0424
14	V vs. D594A T401	-0.7819	-1.910 to 0.3461	No	ns	0.1637
15	WT vs. T401	-1.318	-2.446 to -0.1900	Yes	*	0.0243
16	WT vs. S419	-0.04190	-1.170 to 1.086	No	ns	0.9390
17	WT vs. D594A	-0.4314	-1.559 to 0.6966	No	ns	0.4344
18	WT vs. D594A T401	-0.04079	-1.169 to 1.087	No	ns	0.9406
19	T401 vs. S419	1.276	0.1481 to 2.404	Yes	*	0.0286
20	T401 vs. D594A	0.8866	-0.2414 to 2.015	No	ns	0.1167
21	T401 vs. D594A T401	1.277	0.1492 to 2.405	Yes	*	0.0284
22	S419 vs. D594A	-0.3895	-1.518 to 0.7385	No	ns	0.4797
23	S419 vs. D594A T401	0.001103	-1.127 to 1.129	No	ns	0.9984
24	D594A vs. D594A T401	0.3906	-0.7374 to 1.519	No	ns	0.4784
25						
26	NF1					
27	V vs. WT	-1.373	-2.501 to -0.2448	Yes	*	0.0195
28	V vs. T401	-2.007	-3.135 to -0.8791	Yes	**	0.0014
29	V vs. S419	-1.274	-2.402 to -0.1455	Yes	*	0.0288
30	V vs. D594A	-2.599	-3.727 to -1.471	Yes	***	0.0001
31	V vs. D594A T401	-2.496	-3.624 to -1.368	Yes	***	0.0002
32	WT vs. T401	-0.6343	-1.762 to 0.4938	No	ns	0.2546
33	WT vs. S419	0.09932	-1.029 to 1.227	No	ns	0.8561
34	WT vs. D594A	-1.226	-2.354 to -0.09768	Yes	*	0.0347
35	WT vs. D594A T401	-1.123	-2.251 to 0.004999	No	ns	0.0509
36	T401 vs. S419	0.7336	-0.3944 to 1.862	No	ns	0.1900
37	T401 vs. D594A	-0.5914	-1.719 to 0.5366	No	ns	0.2871
38	T401 vs. D594A T401	-0.4888	-1.617 to 0.6393	No	ns	0.3769
39	S419 vs. D594A	-1.325	-2.453 to -0.1970	Yes	*	0.0236
40	S419 vs. D594A T401	-1.222	-2.350 to -0.09432	Yes	*	0.0351
41	D594A vs. D594A T401	0.1027	-1.025 to 1.231	No	ns	0.8513
42						

2way ANOVA
Multiple comparisons

43									
44	Test details	Mean 1	Mean 2	Mean Diff.	SE of diff.	N1	N2	t	DF
45									
46	NS								
47	V vs. WT	0.2589	1.000	-0.7411	0.5408	3	3	1.370	20
48	V vs. T401	0.2589	2.318	-2.059	0.5408	3	3	3.808	20
49	V vs. S419	0.2589	1.042	-0.7830	0.5408	3	3	1.448	20
50	V vs. D594A	0.2589	1.431	-1.173	0.5408	3	3	2.168	20
51	V vs. D594A T401	0.2589	1.041	-0.7819	0.5408	3	3	1.446	20
52	WT vs. T401	1.000	2.318	-1.318	0.5408	3	3	2.437	20
53	WT vs. S419	1.000	1.042	-0.04190	0.5408	3	3	0.07748	20
54	WT vs. D594A	1.000	1.431	-0.4314	0.5408	3	3	0.7978	20
55	WT vs. D594A T401	1.000	1.041	-0.04079	0.5408	3	3	0.07544	20
56	T401 vs. S419	2.318	1.042	1.276	0.5408	3	3	2.360	20
57	T401 vs. D594A	2.318	1.431	0.8866	0.5408	3	3	1.640	20
58	T401 vs. D594A T401	2.318	1.041	1.277	0.5408	3	3	2.362	20
59	S419 vs. D594A	1.042	1.431	-0.3895	0.5408	3	3	0.7203	20
60	S419 vs. D594A T401	1.042	1.041	0.001103	0.5408	3	3	0.002039	20
61	D594A vs. D594A T401	1.431	1.041	0.3906	0.5408	3	3	0.7223	20
62									
63	NF1								
64	V vs. WT	0.4942	1.867	-1.373	0.5408	3	3	2.539	20
65	V vs. T401	0.4942	2.501	-2.007	0.5408	3	3	3.712	20
66	V vs. S419	0.4942	1.768	-1.274	0.5408	3	3	2.355	20
67	V vs. D594A	0.4942	3.093	-2.599	0.5408	3	3	4.805	20
68	V vs. D594A T401	0.4942	2.990	-2.496	0.5408	3	3	4.615	20
69	WT vs. T401	1.867	2.501	-0.6343	0.5408	3	3	1.173	20
70	WT vs. S419	1.867	1.768	0.09932	0.5408	3	3	0.1837	20
71	WT vs. D594A	1.867	3.093	-1.226	0.5408	3	3	2.267	20
72	WT vs. D594A T401	1.867	2.990	-1.123	0.5408	3	3	2.077	20
73	T401 vs. S419	2.501	1.768	0.7336	0.5408	3	3	1.357	20
74	T401 vs. D594A	2.501	3.093	-0.5914	0.5408	3	3	1.094	20
75	T401 vs. D594A T401	2.501	2.990	-0.4888	0.5408	3	3	0.9038	20
76	S419 vs. D594A	1.768	3.093	-1.325	0.5408	3	3	2.450	20
77	S419 vs. D594A T401	1.768	2.990	-1.222	0.5408	3	3	2.260	20
78	D594A vs. D594A T401	3.093	2.990	0.1027	0.5408	3	3	0.1899	20

2way ANOVA<br>Multiple comparisons									
1	Compare each cell mean with the other cell mean in that column.								
2									
3	Number of families	1							
4	Number of comparisons per family	6							
5	Alpha	0.05							
6									
7	Uncorrected Fisher's LSD	Mean Diff.	95% CI of diff.	Significant?	Summary	Individual P Value			
8									
9	NS - NF1								
10	V	-0.2353	-1.797 to 1.326	No	ns	0.7585			
11	WT	-0.8671	-2.429 to 0.6947	No	ns	0.2632			
12	T401	-0.1833	-1.745 to 1.378	No	ns	0.8107			
13	S419	-0.7258	-2.288 to 0.8360	No	ns	0.3470			
14	D594A	-1.661	-3.223 to -0.09957	Yes	*	0.0380			
15	D594A T401	-1.949	-3.511 to -0.3875	Yes	*	0.0166			
16									
17									
18	Test details	Mean 1	Mean 2	Mean Diff.	SE of diff.	N1	N2	t	DF
19									
20	NS - NF1								
21	V	0.2589	0.4942	-0.2353	0.7567	3	3	0.3110	24
22	WT	1.000	1.867	-0.8671	0.7567	3	3	1.146	24
23	T401	2.318	2.501	-0.1833	0.7567	3	3	0.2422	24
24	S419	1.042	1.768	-0.7258	0.7567	3	3	0.9592	24
25	D594A	1.431	3.093	-1.661	0.7567	3	3	2.195	24
26	D594A T401	1.041	2.990	-1.949	0.7567	3	3	2.576	24
